# Fibrous Pressure Sensor with Unique Resistance Increase under Partial Compression: Coaxial Wet‐Spun TiO_2_/Graphene/Thermoplastic Polyurethane Multi‐Wall Multifunctional Fiber

**DOI:** 10.1002/adma.202509631

**Published:** 2025-07-16

**Authors:** Ziwei Chen, Dandan Xie, Kanae Kojima, Chunxia Gao, Jian Shi, Jian Xing, Hideaki Morikawa, Chunhong Zhu

**Affiliations:** ^1^ Graduate School of Medicine Science and Technology Shinshu University Tokida Ueda Nagano 386–8567 Japan; ^2^ Institute for Fiber Engineering and Science (IFES) Shinshu University 3‐15‐1 Tokida Ueda Nagano 386–8567 Japan; ^3^ School of Chemistry and Chemical Engineering Yangzhou University Yangzhou 225002 China; ^4^ Faculty of Textile Science and Technology Shinshu University 3‐15‐1 Tokida Ueda Nagano 386–8567 Japan; ^5^ School of Textile and Garment Anhui Polytechnic University Wuhu 241000 China

**Keywords:** coaxial wet‐spinning, multi‐wall structure, pressure‐sensitive fiber sensor, sensing mechanism, smart sensing array

## Abstract

Fiber‐shaped resistive pressure‐sensitive sensors are rare. Although fibers are widely used in strain sensors owing to their structural advantages, developing fiber‐shaped resistive pressure sensors is challenging. This challenge arises because the fiber acts as a series circuit along its axis, requiring increased resistance in the compressed region for a significant signal. Coaxial wet‐spinning is used to create a multi‐walled fiber. The outer layer, made of thermoplastic polyurethane (TPU)/TiO_2_ slurry, ensured a smooth surface, while the inner core, containing 4% graphene nanoplatelets (GNPs), is extruded at 20 mL h^−1^. Unlike conventional resistance‐increasing pressure sensors, TGTMW fiber shows rising resistance under compression due to microcracks in its multi‐wall structure. Its pressure sensitivity is evaluated using various 3D‐printed indenters and showcasing its excellent performance. This sensor has found promising applications in remote motion detection, press or slide differentiation through wavelet transforms applied to high‐speed sensing data, and real‐time signal acquisition from a multi‐channel sensing array. Additionally, intuitive visualization software is developed for the sensing array application and implemented a CNN‐based machine learning algorithm for data analysis. The system achieved a recognition accuracy of ≈99.6% for 12 different compression modes. This work is believed to propose a new mechanism and design for fiber‐based pressure sensors.

## Graphical abstract

TGTMW fibers are designed to show a sharp resistance increase under pressure, as the multi‐wall structure in the transition region between compressed and uncompressed areas bends and forms microcracks. This design breaks the physical sensitivity limit of conventional fiber‐shaped pressure sensors, which usually show resistance reduction under pressure and are constrained by their series structure under partial compression.

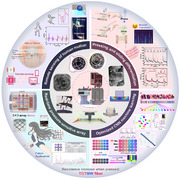

## 1 Introduction

Resistive pressure‐sensitive sensors in the form of single fibers are relatively rare in current relative research,^[^ ^1^, ^2^, ^3^, ^4^, ^5^, ^6^, ^7^ ^]^ despite the abundance of excellent work on aerogel‐based,^[^ ^8^, ^9^, ^10^ ^]^ film‐based,^[^ ^11^, ^12^, ^13^, ^14^ ^]^ and 3D printing‐based^[^ ^15^, ^16^, ^17^ ^]^ pressure sensors. The structural design of flexible sensors plays a crucial role in their performance. In the case of flexible strain sensors,^[^ ^18^, ^19^ ^]^ fiber‐based structures^[^ ^20^, ^21^, ^22^ ^]^ have already been widely adopted due to their advantages, such as small volume, low cost, user‐friendliness, and high adaptability. These advantages remain highly relevant for pressure‐sensitive sensors and may be even more critical, particularly in applications requiring a small size. The introduction of fiber‐based structures can significantly improve traditional pressure‐sensitive arrays,^[^ ^10^, ^23^, ^24^, ^25^ ^]^ tactile receptors,^[^ ^26^, ^27^ ^]^ and so on, which often suffer from excessive size and complex designs. Furthermore, compared to the complex production methods required for fabricating thin films or 3D aerogels through freeze‐drying,^[^ ^28^, ^29^ ^]^ fiber‐shaped sensors can be continuously manufactured via wet spinning^[^ ^30^, ^31^ ^]^ and other fiber‐forming techniques.^[^ ^20^ ^]^ This makes them highly competitive in terms of cost for industrial‐scale production and commercialization.

One of the key challenges in developing fiber‐based pressure‐sensitive sensors is ensuring that they exhibit an open‐circuit behavior—meaning that their resistance increases rather than decreases under applied pressure. This characteristic is essential because a fiber can be regarded as a series circuit along its central axis. If only a section of the fiber is compressed, a clear and effective sensing response can only be achieved if the resistance of that compressed segment increases. The reason lies in the additive nature of resistance in a series circuit: if the resistance of a segment decreases under pressure, the total fiber resistance can only reduce up to the sum of the remaining uncompressed segments. However, if the resistance of a segment increases under pressure, it can potentially rise several times, leading to a more significant change in the total resistance of the fiber. This property is crucial for achieving a highly responsive and efficient sensing mechanism.

Common research on pressure‐sensitive sensors often employs nanomaterials such as graphenes,^[^ ^28^, ^32^ ^]^ MXenes,^[^ ^17^, ^33^, ^34^, ^35^ ^]^ or CNTs^[^ ^36^, ^37^, ^38^ ^]^ as conductive phases. These materials are typically integrated into films or aerogels through various matrix compositions and fabrication techniques. Despite differences in structure, their sensing mechanism remains similar: when pressure is applied to the matrix, the distance between conductive materials decreases, resulting in a significant reduction in overall resistance.^[^ ^39^, ^40^ ^]^ Under the same voltage, the current increases significantly, even by several times, due to the drastic reduction in resistance, which gives these research findings a high gauge factor (GF). For fiber‐based pressure‐sensitive sensors requiring a substantial increase in resistance under compression, traditional design strategies, while valuable, may not be groundbreaking. For example, in a recent study by Ma et al. published in 2025,^[^ ^31^ ^]^ multifunctional fibers were fabricated via wet spinning. However, their pressure‐sensitive response required compression over a large portion of the fiber to induce a significant increase in current, thereby limiting their practical applications. Furthermore, the vast majority of papers with the keyword “fiber” in their titles actually refer to pressure‐sensitive sensors made of fibers rather than pressure‐sensitive sensors composed of a single fiber.^[^ ^28^, ^32^, ^41^ ^]^ If a fiber could exhibit a pronounced response signal when compressed at any small localized section, it would unlock unique and highly significant applications, leading to groundbreaking advancements in real‐world scenarios.

Departing from conventional flexible pressure sensor designs, we developed a fiber with a multi‐walled internal architecture. Upon compression, a localized portion of the fiber undergoes deformation, during which the multi‐walled structure bends and develops microcracks. This deformation disrupts the conductive network along the fiber's central axis, leading to a significant increase in overall electrical resistance. To realize this unique design, we employed coaxial wet‐spinning.^[^ ^22^, ^30^ ^]^ For the outer shell, thermoplastic polyurethane (TPU) with titanium dioxide (TiO_2_) was extruded at a slow rate (4 mL h^−1^). When this layer contacted the coagulation bath (pure water), it rapidly solidified, forming a smooth surface. Meanwhile, the core layer was composed of a slurry containing 10 wt.% TPU and 4 wt.% graphene nanoplatelets (GNPs), extruded at a much higher rate of 20 mL h^−1^. Under this composition, due to the high surface area and significant loading of GNPs, TPU undergoes phase separation and precipitates onto the GNPs surfaces during solvent exchange. As a result, fibers with special multi‐walled structures in cross‐section are formed by this special spinning method. Given its material composition—TiO_2_, GNPs, and TPU—and its unique multi‐wall structure, we have named this fiber TGTMW. To investigate its sensing mechanism, we utilized 3D‐printed indenters of various shapes to test the response of TGTMW fibers under different conditions. We further demonstrated three key applications of the TGTMW fiber: Remote human motion sensing via Bluetooth I), distinguishing press/slide interactions II) by using three parallel TGTMW fibers and applying wavelet transform‐based high‐speed data processing, real‐time pressure‐sensitive arrays III) by arranging three TGTMW fibers along both horizontal and vertical axes, combined with software and a CNN‐based machine learning algorithm. Notably, the latter two applications are challenging to achieve with other sensor architectures and are rarely explored in existing studies. Therefore, the TGTMW fiber, with its innovative design, distinctive structure, and versatile applications, holds immense potential for advancing flexible sensors and next‐generation smart devices.

## 2 Results and Discussion

**Figure 1:**
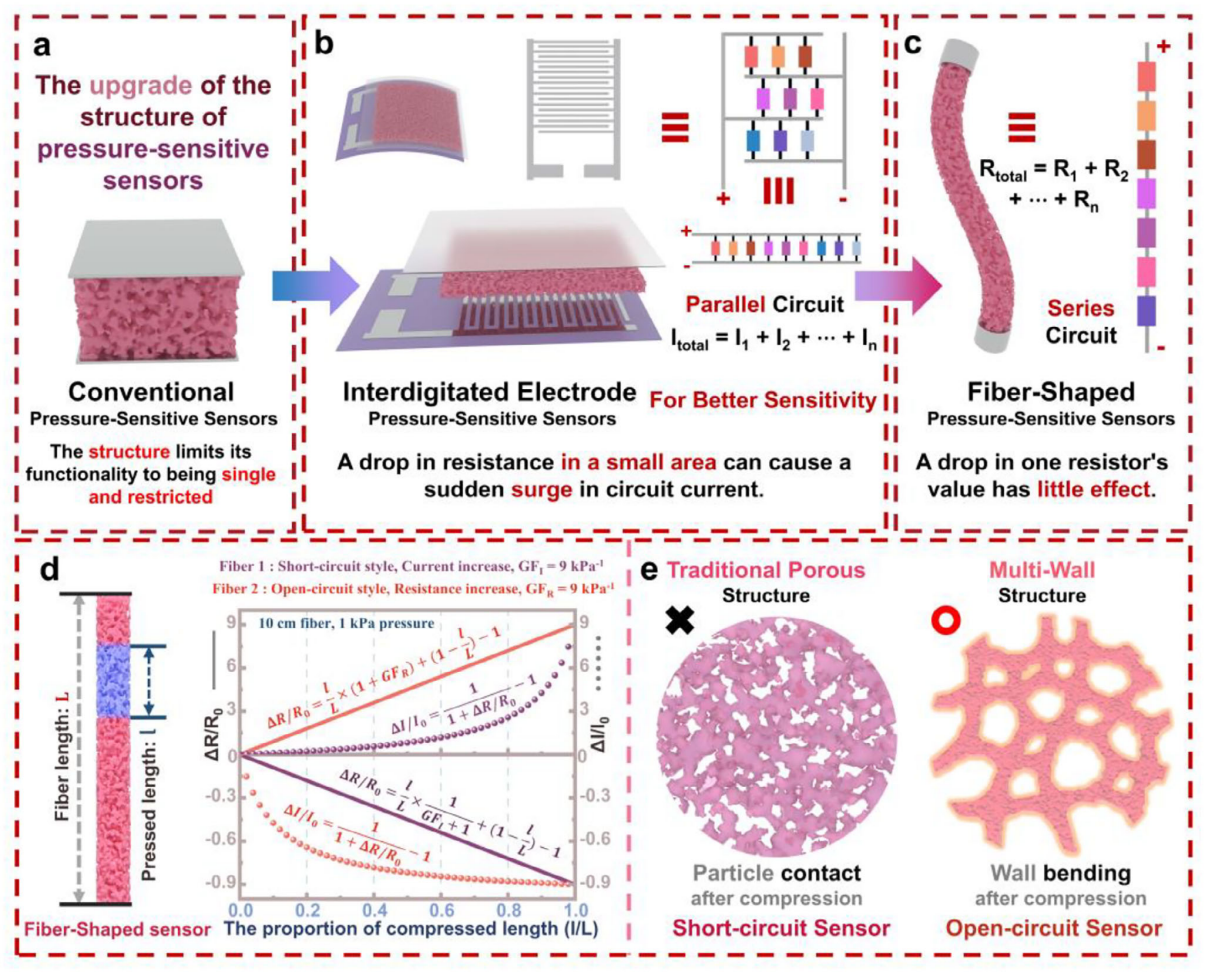
The impact of structure on the function and design of resistive pressure sensors: a) traditional pressure‐sensitive sensor, b) film‐based pressure‐sensitive sensor using interdigitated electrodes, c) fiber‐based pressure‐sensitive sensor, d) the importance of an open‐circuit structure for fiber‐based series pressure‐sensitive sensors, e) the influence of internal cross‐sectional structure on fiber sensing mechanisms.

The structure of resistive pressure sensors significantly affects their functionality and design, as illustrated in **Figure** **1**. Figure 1a depicts a traditional pressure‐sensitive sensor like aerogels,^[^ ^8^, ^9^, ^10^, ^28^, ^29^ ^]^ where a pressure‐sensitive material is sandwiched between two electrodes. When under pressure, the resistance between the electrodes decreases, enabling sensing functionality. However, conventional pressure sensors suffer from drawbacks such as large volume and insufficient flexibility. To overcome these limitations, researchers have developed more flexible and highly sensitive film‐like pressure sensors by integrating interdigitated electrodes with pressure‐sensitive materials,^[^ ^42^, ^43^ ^]^ as shown in Figure 1b. Essentially, this design follows a parallel circuit configuration. When any small region of the film experiences pressure, its resistance decreases, and due to the principles of parallel circuits, the overall resistance of the film drops significantly (resulting in a substantial increase in current at a constant voltage). This design not only ensures a more reasonable electrode distribution but also greatly enhances sensor sensitivity. Less commonly studied fiber‐based pressure‐sensitive sensors are illustrated in Figure 1c. Unlike film‐like pressure sensors, which follow a parallel configuration, fiber sensors operate in a series configuration along the fiber's circular axis. In this structure, a localized decrease in resistance has little effect on the overall resistance of the fiber. A noticeable sensing signal appears only when a specific segment of the fiber exhibits a significant increase in resistance.

This principle is further explained through calculations, as illustrated in Figure 1d. Suppose two fibers, ***fiber 1*** and ***fiber 2***, are composed of materials with similar properties. ***Fiber 1*** follows a short‐circuit mechanism, where applying a pressure of 1 kPa reduces its resistance to 10% of its original value (increasing the current to ten times its original value at constant voltage), resulting in a Gauge Factor for I (**GF_I_**) of 9 kPa^−1^. On the other hand, ***fiber 2*** follows an open‐circuit mechanism, where the same 1 kPa pressure increases its resistance to ten times its original value (reducing the current to 10% of its initial value), yielding a Gauge Factor for R (**GF_R_**) of 9 kPa^−1^. If both fibers were used to fabricate a traditional pressure sensor subjected to uniform pressure, they would theoretically exhibit the same Gauge Factor of 9 kPa^−1^. However, when structured as a fiber‐based sensor with only 10% of its length experiencing the 1 kPa pressure, the resulting signals differ significantly. For the short‐circuit ***fiber 1***, its total resistance decreases by ≈9%, leading to a current increase of ≈9.89% under a constant voltage. In contrast, for the open‐circuit ***fiber2***, its total resistance increases by 90%, causing a 47.37% decrease in current. This results in signal strength differences by several orders of magnitude, which has a profound impact on practical applications. Therefore, as shown in Figure 1e, conventional porous short‐circuit structures fail to meet the requirements of fiber‐based pressure‐sensitive sensors. Instead, a multi‐walled open‐circuit structure is necessary to ensure that deformation under pressure leads to an increase in resistance, thereby enabling the fabrication of high‐performance fiber‐based pressure sensors.

**Figure 2:**
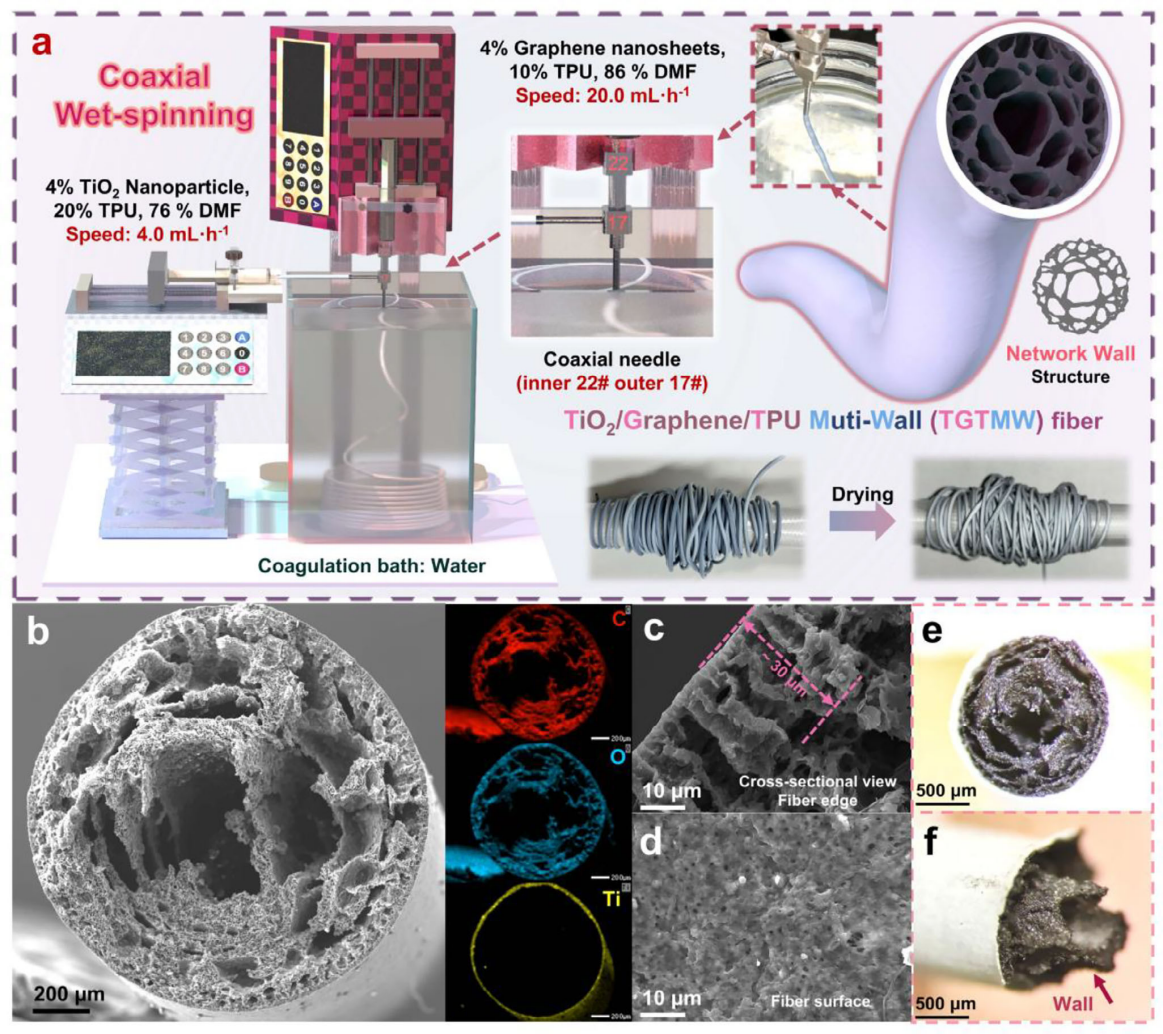
Preparation and characterization of TGTMW fibers: a) Schematic diagram of the wet‐spinning setup. b) Cross‐sectional characterization of TGTMW fibers. c, d) Shell structure characterization of TGTMW fibers. e, f) Optical microscopy characterization of TGTMW fibers.

To address existing challenges in the field of fiber‐based pressure‐sensitive sensors, we designed TGTMW fibers with a multi‐walled cross‐sectional structure. **Figure** **2** illustrates the preparation process and characterization of TGTMW fibers. As shown in Figure 2a, we constructed the fiber preparation setup using two syringe pumps for wet‐spinning. The titanium dioxide (TiO_2_) in the shell layer serves two primary functions: first, it increases the density of the spinning dope, enabling the fibers to settle more effectively into the coagulation bath; second, it enhances the viscosity of the dope and accelerates solvent exchange with water, ensuring quick fiber formation upon contact with water, thereby preventing shrinkage during solvent exchange. The core layer contains thermoplastic polyurethane (TPU) in a concentration half that of the shell layer but is loaded with a significant amount of high‐surface‐area graphene nanoplatelets (GNPs) (see Figure S1, Supporting Information for the SEM image of GNPs). We attempted wet spinning using core spinning dopes with various GNPs contents. As summarized in Table S1 and Figures S2–S4 (Supporting Information). Taking into account both the electrical conductivity of the fibers and the feasibility of the wet spinning process, we ultimately selected a GNPs content of 4% for the core spinning dope. The composition of the core layer allows for rapid flow and extrusion at high velocity, which helps to expand the fiber diameter to provide sufficient internal space for the formation of the multi‐walled structure. During the spinning process, the fibers appear light blue and turn white upon drying, and they can be continuously produced in large quantities.

Figure 2b presents a SEM image of the TGTMW fiber cross‐section after liquid nitrogen fracture, revealing the TPU/GNPs core forming an approximately multi‐walled structure with a diameter of ≈1.4 mm. The EDS mapping further confirms the fiber's structural features, with the outer shell composed of TiO_2_‐containing TPU and the inner core exhibiting a multi‐walled TPU/GNPs composite. Figure 2c,d provide structural observations of the fiber shell, showing a relatively smooth and uniform shell from both lateral and axial perspectives, with a thickness of ≈30 µm—significantly smaller than the overall fiber diameter. The well‐balanced structure, characterized by a “thin shell and thick core”, ensures sufficient protection while minimizing the negative impact of the shell layer on the pressure‐sensing performance of the internal multi‐walled structure. Additional SEM images related to the fiber shell are provided in Figure S5 (Supporting Information). Figure 2e,f displays optical microscope images of the fiber cross‐section after brittle fracture, observed from axial and lateral views. The axial view distinctly highlights the multi‐walled structure surrounding the core, while the lateral view captures the fracture of a specific wall layer during the brittle fracture process, further emphasizing the characteristic multi‐walled architecture.

**Figure 3:**
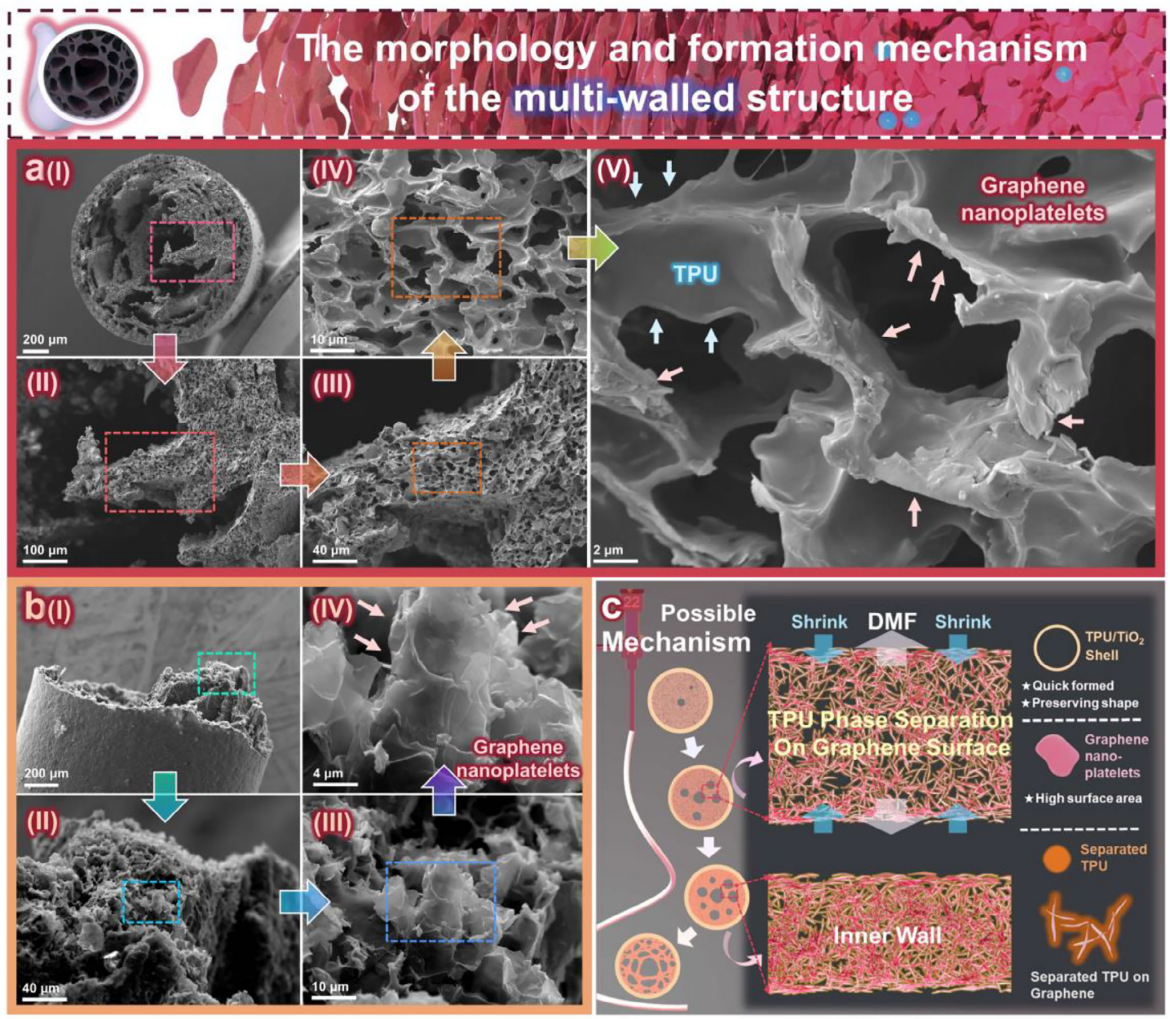
Characterization and formation mechanism analysis of the multi‐wall structure in TGTMW fiber: a) Axial cross‐section SEM characterization, b) lateral SEM characterization at the fracture, c) formation mechanism of the multi‐wall structure in TGTMW fiber.

To further analyze the morphology of the multi‐wall structure inside the TGTMW fiber, a detailed investigation is presented in **Figure** **3**. Figure 3a provides an axial cross‐sectional characterization of a liquid nitrogen‐fractured TGTMW fiber. Figure 3a‐I shows the cross‐section of a multi‐wall TGTMW fiber, and by progressively magnifying the selected area, Figure 3a‐II reveals that the wall thickness in the multi‐wall structure is ≈100 µm. Further magnification in Figure 3a‐III, IV reveals that the walls of the multi‐wall structure actually contain axially oriented pores. In Figure 3a‐V, the detailed structure of these axial pores is displayed: graphene nanoplatelets (GNPs) are attached to the inner walls of the axial pores. Along the direction indicated by the light red arrows, raised sheet‐like structures characteristic of GNPs can be observed. Additionally, some thermoplastic polyurethane (TPU) is attached to the surface of the GNPs, as indicated by the light blue arrows. The sticky morphology observed is characteristic of TPU. Therefore, it is inferred that the microscopic structure of the walls inside TGTMW fiber consists of TPU adhering to axially oriented GNPs, forming a honeycomb‐like structure.

To further verify this hypothesis, we examined the lateral fracture of the TGTMW fiber in Figure 3b. Figure 3b‐I clearly shows the inner walls of the multi‐wall fiber after brittle fracture. Figure 3b‐II reveals that the fracture surface is not entirely smooth. Upon further magnification, a significant amount of GNPs distributed along the fiber axis can be observed on the fracture surface, as shown in Figure 3b‐III. Figure 3b‐IV clearly displays axially aligned graphene nanoplatelets at a fracture site, providing additional evidence that the microstructure of the internal walls in TGTMW fiber likely exhibits a honeycomb‐like arrangement along the fiber axis.

Additionally, we have included more relevant SEM images in the supporting materials. In Figure S6 (Supporting Information), we clearly present the structural features of the inner wall edges and capture the structure of GNPs, along with some TPU microspheres closely adhering to the inner fiber walls (these microspheres can also be observed in the lower‐left corner of Figure 3a‐III. To better observe the inner wall surface of the fiber, the fiber was immersed in liquid nitrogen and then crushed (Figure S7, Supporting Information), and its inner wall was examined using SEM, as shown in Figures S8 and S9 (Supporting Information). The SEM images intuitively reveal GNPs on the inner walls, along with a small number of TPU microspheres, demonstrating that GNPs adhere closely to the inner walls, suggesting an affinity between GNPs and TPU. Additionally, since the flattened fiber outer skin is easier to focus on, Figure S10 (Supporting Information) provides an SEM image of the fiber's outer skin, demonstrating its smoothness.

Based on Figure 3a,b and Figures S6–S10 (Supporting Information), the following characteristics of the multi‐wall structure in TGTMW fiber can be summarized: I) TPU and GNPs exhibit good affinity, and GNPs are closely attached to the inner walls of the TGTMW fiber. II) The inner walls of the fiber exhibit anisotropic structures, with GNPs displaying a certain degree of orientation along the direction of growth on the inner wall. III) A small number of TPU microspheres of varying sizes exist within the fiber interior. Based on these characteristics and the structural features of the multi‐wall TGTMW fiber, a possible formation mechanism is proposed (as shown in Figure 3c). During the wet spinning process, the shell layer consists of high‐concentration TPU slurry containing TiO_2_, which immediately solidifies upon contact with the coagulation bath (water). Meanwhile, water molecules penetrate the fiber interior through micro‐pores in the formed outer layer (Figure S5, Supporting Information), while DMF in the slurry diffuses into the coagulation bath. As solvent exchange progresses, the water content in the TPU/GNPs/DMF slurry inside the fiber reaches a critical level, leading to phase separation and TPU precipitation. Since the GNPs used are non‐functionalized, their surfaces primarily consist of sp^2^‐hybridized carbon atoms, lacking polar groups, making them hydrophobic. TPU, being an amphiphilic polymer, has hydrophobic segments that preferentially aggregate or adsorb onto the GNPs surfaces, promoting TPU precipitation at these interfaces and forming a semi‐solid phase. The remaining liquid phase primarily consists of DMF/H_2_O along with trace amounts of unprecipitated TPU. The liquid phase continuously undergoes solvent exchange within the semi‐solid components, where TPU precipitates on the surface of GNPs but has not yet fully solidified. This process leads to the loss of DMF from the semi‐solid components, resulting in gradual shrinkage and solidification. It progressively thins the semi‐solid phase, forming wall‐like TPU/GNPs structures, ultimately giving rise to the multi‐wall structure inside the fiber. Since the liquid phase still contains DMF and trace amounts of TPU that have not yet fully precipitated, during the subsequent soaking and cleaning process to completely remove DMF, the TPU—due to its extremely low concentration—precipitates in the form of tiny microspheres and adheres to the multi‐wall structure.

**Figure 4:**
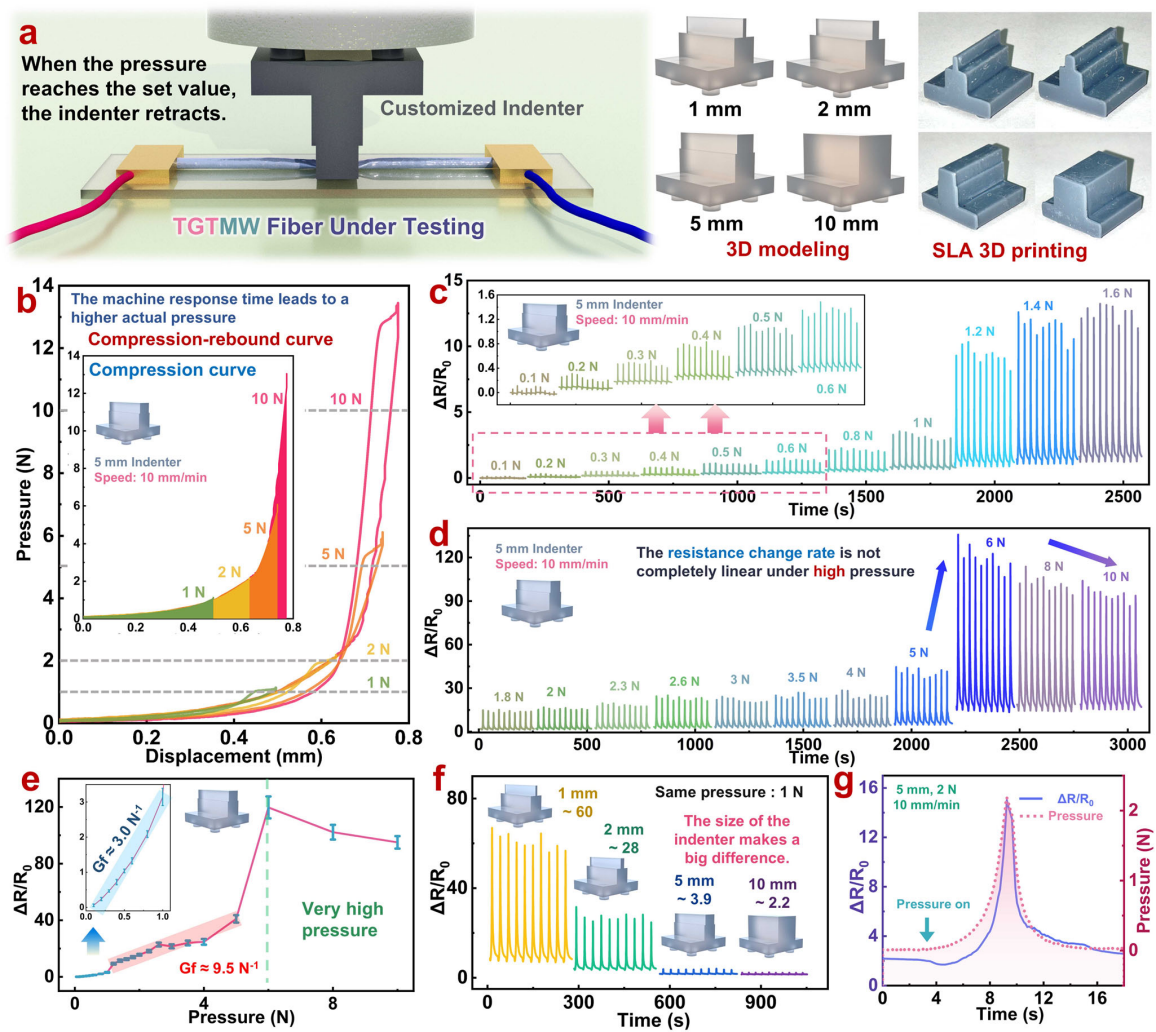
Piezoresistive sensing performance of TGTMW fibers a) Piezoresistive sensing test setup; b) Compression‐recovery curve using a 5 mm indenter; c, d) Electrical resistance response of the fiber under different pressure levels applied with a 5 mm indenter; e) Relationship between fiber resistance change and applied pressure; f) Compression signals of the fiber under a 1 N force applied with indenters of different sizes; g) Resistance response waveform of the fiber under compression.

In **Figure** **4**, we evaluated the compression sensing performance of TGTMW fibers. Notably, due to the elongated structure of fiber‐based compression sensors, only a localized region of the fiber is typically compressed during practical use. This localized region compression characteristic grants fiber‐based pressure sensors a significantly broader range of applications compared to traditional flexible piezoresistive sensors. However, this also necessitates a unique testing methodology to accurately assess their sensing performance. As illustrated in Figure 4a, indenters of different sizes (1, 2, 5, and 10 mm in width) were designed and 3D‐printed using a photocuring printer. These indenters were then affixed to a benchtop tensile‐compression testing machine, which applied a controlled downward compression onto the TGTMW fiber connected to an electrometer at both ends. The machine retracted once the sensor detected the predefined force, and the design schematics of indenters and photographs of the compression testing apparatus are shown in Figure S11 (Supporting Information). The compression speed was set at 10 mm min^−1^, primarily to account for the sensor's response time during force detection and retraction. Given that the fiber is slender, with a diameter of only 1.4 mm, a higher compression speed would result in excessive displacement of the testing machine within the sensor's response time, significantly increasing the actual pressure applied to the fiber.

To simulate fingertip‐like compression, we first conducted tests using a 5 mm indenter. As shown in Figure 4b, the pressure exerted on the fiber increases exponentially with the strain. When the displacement reaches ≈0.75 mm, the applied pressure reaches 10 N. However, due to the response time of the testing instrument, even a 0.01 mm displacement during retraction can substantially increase the actual pressure on the fiber. Therefore, in Figure 4, the actual pressure applied to the fiber is slightly higher than the programmed pressure (within 20% for pressures below 5 N and within 40% for 5–10 N). The values in the figure represent the programmed pressure settings.

As depicted in Figure 4c, the TGTMW fiber can detect a minimum pressure of 0.1 N, equivalent to a light fingertip touch. As the applied pressure increases, the electrical resistance response of the fiber also increases. At 1 N, the resistance reaches ≈2.5 times its initial value. Additionally, Figure 4d shows that the resistance signal continues to increase with rising pressure. Notably, at 5–6 N, there is a sudden resistance jump—from a 40‐fold increase at 5 N to a 110‐fold increase at 6 N. Beyond this point, the rate of resistance changes to decrease slightly under higher pressures. Figure 4e summarizes the effect of different pressure levels on the fiber's resistance change when using a 5 mm indenter. The Gauge Factor (GF) of the sensor is 3.0 N^−1^ in the 0.1–1 N range and 9.5 N^−1^ in the 1–5 N range, respectively. When the applied force exceeds 6 N, the resistance change rate slightly declines but remains around 100 times the initial resistance. A similar phenomenon was observed when using a 10 mm indenter under pressures above 12 N (Figure S12, Supporting Information), and the reason for this phenomenon may be related to the unique mechanism of TGTMW, which differs from other pressure‐sensitive sensors. Relevant details are provided in Figures S13 and S14 (Supporting Information).

To further investigate the effect of indenter size, we tested fibers under 1 N compression using indenters of various sizes (Figure 4f). The results show that indenter size significantly affects the fiber's compression signal. Smaller indenters produce a larger resistance change under the same applied force. In other words, at a constant pressure, a smaller contact area leads to a greater resistance variation in the fiber. Figure 4g presents the resistance response waveform of the fiber under compression. The resistance initially decreases slightly upon pressure application but then increases sharply, rather than exhibiting a perfectly linear correlation with pressure. This behavior may be attributed to the complex deformation process of the multi‐walled structure under compression. Additionally, we conducted a 3200‐cycle durability test using a 5 mm indenter and 0.8 N force (equivalent to the force applied when touching a touchscreen or keyboard). As shown in Figure S15 (Supporting Information), the TGTMW fibrous pressure‐sensitive sensor maintained a high signal‐to‐noise ratio throughout 3,200 test cycles, though signal intensity exhibited gradual attenuation as cycling progressed. This attenuation stems from the viscoelastic properties of the TPU material and has minimal impact on subsequent applications. Furthermore, it can be addressed in future work through algorithmic compensation and reinforcement of the polymer matrix.

In summary, TGTMW multi‐walled fibers exhibit a strong signal response under partial compression. Even a light fingertip touch, such as tapping a keyboard, can increase their resistance several times, demonstrating exceptional sensitivity. The 0.1–5 N detection range is sufficient for most everyday applications. However, compared to traditional foam/aerogel/film‐based bulk pressure sensors, TGTMW fibers show some non‐linearity in their signal peaks. This discrepancy arises from fundamental differences in sensing mechanisms and the vast disparity in conductive network volume between fiber‐based and bulk sensors. For comparison, the tested TGTMW fiber measures ≈4 cm in length and 1.4 mm in diameter, with a conductive network volume of only 0.0615 cm^3^. In contrast, a conventional conductive foam/aerogel/hydrogel sensor of 2 cm diameter and thickness has a conductive network volume of ≈6.28 cm^3^—≈100 times larger than that of the fiber sensor. The volume of TGTMW multi‐walled fibers is only ≈1% of that of traditional pressure‐sensitive sensors. Despite their small size, they can generate sufficiently large signals, and their structural advantages enable a wide range of applications, making them highly promising.

**Figure 5:**
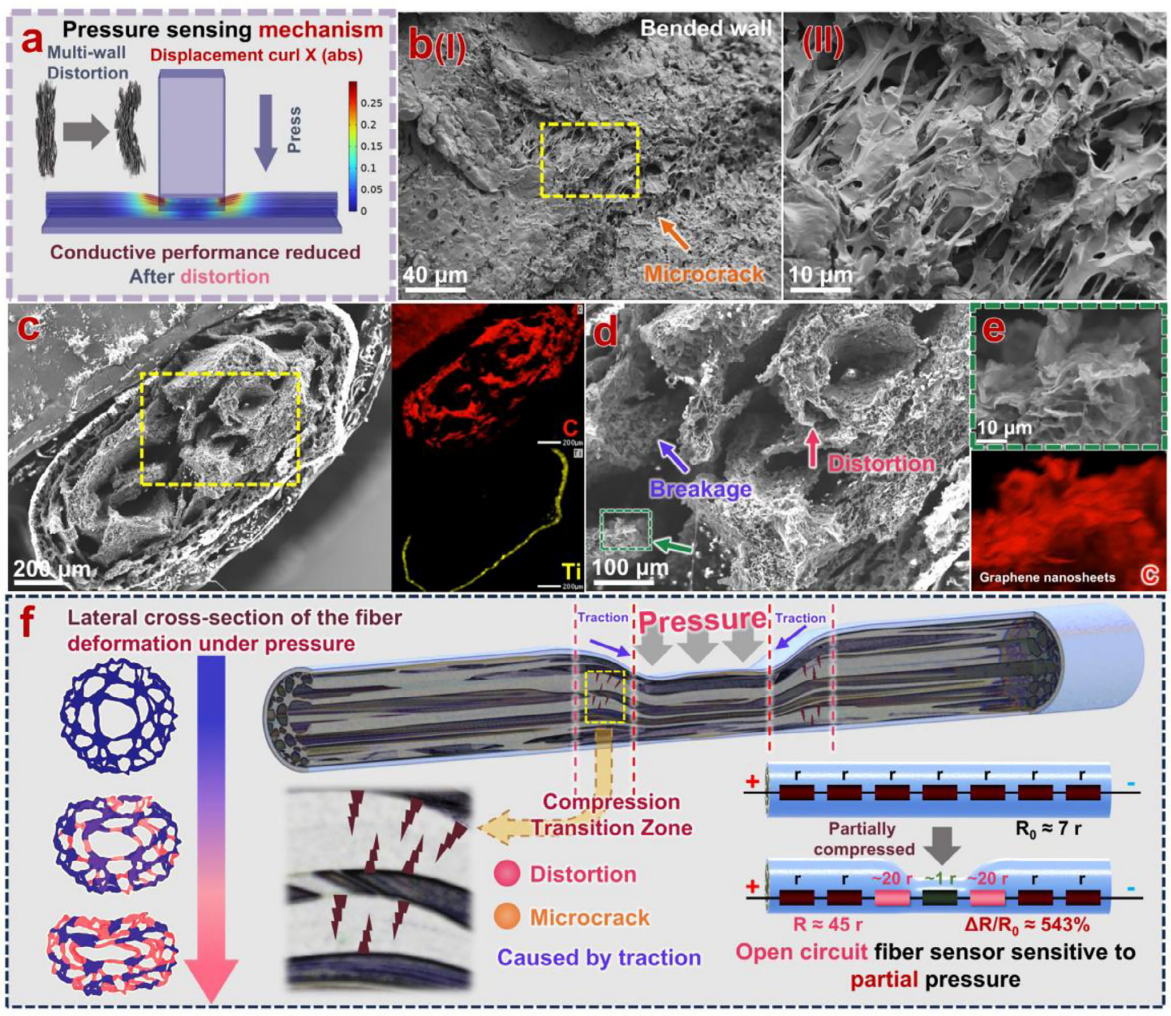
Analysis of the pressure‐sensing mechanism of TGTMW multi‐wall fibers: a) Finite element simulation showing the bending deformation of the wall structure under compression; b) FESEM image of microcracks formed in the bent multi‐wall structure. c–e) SEM images of compressed TGTMW fibers; f) Schematic illustration of the compression‐sensing mechanism in TGTMW multi‐wall fibers.

Based on the pressure‐sensing performance presented in Figure 4 and the structural characteristics of TGTMW fibers, we summarized the pressure‐sensing mechanism of TGTMW fibers in **Figure** **5**. As shown in Figure 5a, we conducted finite element analysis to evaluate the absolute value of the displacement curl in the axial direction within the multi‐walled fiber under compression, which represents the degree of bending of the inner walls. Detailed results are presented in Figure S16 (Supporting Information), illustrating that the inner walls in the transition region of the fiber experience significant bending when subjected to pressure. As explained from multiple perspectives in Figure 3 and Figures S6,S8 and S9 (Supporting Information), the inner wall structure of TGTMW fibers consists of graphene nanoplatelets (GNPs) partially aligned along the fiber axis, creating anisotropy within the fiber. Under normal conditions, GNPs stack with relatively large contact areas, enabling efficient electron transport. However, when the inner wall structure bends, the stacking alignment between GNPs is partially disrupted, significantly reducing electron transport capability. This bending and deformation of the structure results in increased electrical resistance. In addition, microcracks generated in the inner walls after bending—also observable in Figure 5b and Figure S17 (Supporting Information)—further hinder electron transport, leading to a sharp increase in resistance in the transition region after the fiber is compressed. To intuitively demonstrate the bending and deformation of the internal walls during compression, we captured the behavior of TGTMW fibers under compression using microscopy in Movie S1 (Supporting Information). Selected frames from one compression‐release cycle are presented in Figure S18 (Supporting Information). To further examine the microscopic structure under compression, the TGTMW fibers were fixed under applied pressure and observed via SEM (Figure 5c). It was clearly observed that internal walls exhibited deformation after compression. Under higher magnification, shown in Figure 5d, portions of the internal wall structures not only experienced distortion but also displayed breakage in some regions. In these damaged regions, GNPs were visible (Figure 5e).

Based on the analysis above, we illustrate the mechanism behind the increase in electrical resistance of partially compressed TGTMW fibers in Figure 5f. When a specific portion of a fiber is compressed, deformation occurs locally, causing the cylindrical fiber to flatten and internal walls to distort. In the transitional regions between compressed (flattened) and uncompressed (cylindrical) portions, the axial wall structures within the fibers are pulled and experience significant distortion and microcracks. Since the electrodes are connected at the two ends of the fiber and current travels axially, this axial distortion greatly increases electrical resistance, making this phenomenon the primary cause for the high sensitivity of TGTMW fibers. This mechanism also explains why the electrical response varies dramatically when indenters of different sizes compress the fiber under identical pressure. A smaller indenter, under the same load, induces a greater axial bending and distortion of the internal wall structures. Additionally, this mechanism clarifies why resistance signals unexpectedly decrease when pressures exceed 6 N using a 5‐mm indenter. Under excessive pressure, fiber deformation reaches its limit, and axial deformation of the internal walls also saturates. In this scenario, the GNPs structures become tightly compacted, which can instead decrease local resistance.

Furthermore, the resistance changes in regions directly under compression are relatively complex; walls‐oriented perpendicular to the applied force may see reduced resistance due to compaction, whereas walls undergoing bending deformation or breakage experience increased resistance. This complexity may contribute to some nonlinearities observed in the pressure‐response signal of TGTMW fibers. Nevertheless, this does not significantly affect the overall sensing performance. The dramatic increase in resistance at transitional regions between compressed and non‐compressed areas, due to axial distortion, remains the dominant reason TGTMW fibers exhibit excellent sensitivity for detecting localized pressure. Finally, the logical connections among the characterizations, mechanisms, and observed phenomena of the TGTMW fiber are summarized in Figure S19 (Supporting Information) and explained in detail.

**Figure 6:**
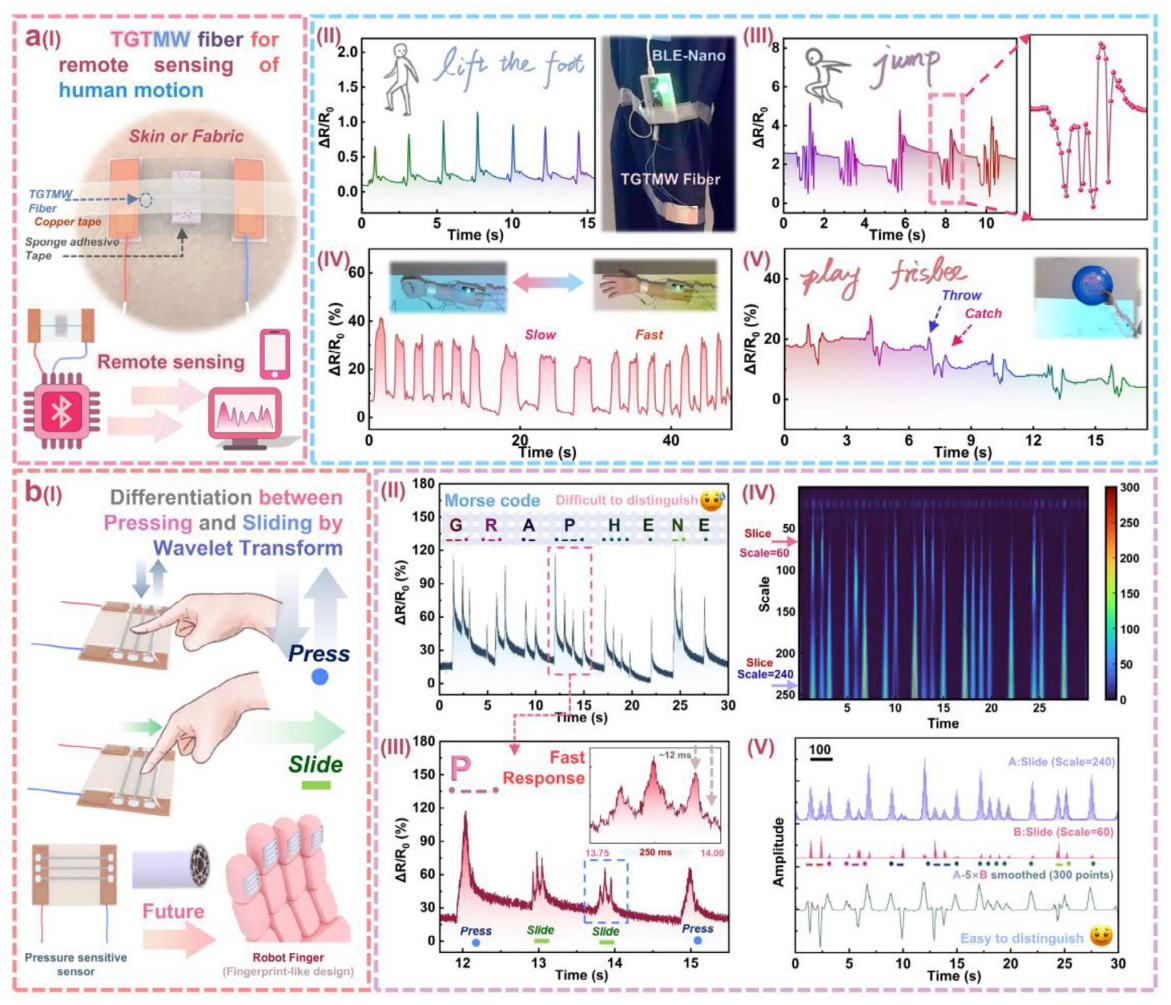
TGTMW multi‐walled fiber application demonstration: a) Remote human motion sensing based on Bluetooth, b) press/slide differentiation application achieved by connecting three TGTMW fibers in parallel and processing high‐speed data with wavelet transform.

In **Figure** **6**, we demonstrate some specific applications of TGTMW fibers. Due to their unique fiber‐like structural advantages, TGTMW fibers not only perform well in traditional pressure‐sensitive sensor applications but also enable unique applications that traditional pressure‐sensitive sensors cannot achieve. As shown in Figure 6a‐I, TGTMW fibers can be assembled into a sensor that can be attached to the human body surface (the corresponding photograph and structural schematic diagram are shown in Figure S20, Supporting Information). A BLE‐Nano microcontroller, powered by a power bank, transmits signals via Bluetooth to a computer for remote sensing. We connected the computer to a projector, and through a Graphical User Interface (GUI) window (as shown in Figure S21, Supporting Information), intuitive real‐time remote motion detection was achieved. In Figure 6a‐II, III, we attached the sensor to the pants. When the wearer moves, the force generated by leg movements is transmitted to the sensor, which applies pressure on the TGTMW fibers through foam tape, thereby achieving high‐precision sensing. We tested two types of movements: foot lifting and jumping (the relevant test process video is provided in Movie S2, Supporting Information). It can be observed that whether it is foot lifting or jumping, the sensor made from TGTMW fibers captures clear, specific, and highly reproducible signals with excellent differentiation. This demonstrates that sensors made from TGTMW fibers possess high sensitivity, capturing detailed movements and not only detecting actions but also distinguishing between different movements due to their unique response characteristics.

Additionally, the TGTMW fiber‐based sensor can be fixed on the wrist, where wrist muscle movements exert different pressures on the sensor, enabling motion detection. As shown in Figure 6a‐IV, when clenching a fist, the muscles apply pressure on the sensor, resulting in a significant change in resistance detected by the sensor. When opening and closing the fist at different speeds, the sensor made from TGTMW fibers can accurately and in real‐time detect the state of hand movements, indicating its potential applications in hand motion detection. In Figure 6a‐V, we tested the detection of complex movements (playing frisbee). This activity involves complex hand muscle movements, and since the position where the frisbee lands varies, the hand needs to move in different ways. The sensor made from TGTMW fibers is able to accurately capture the complex signals corresponding to hand muscle movements in real‐time during the “playing frisbee” process. For example, in the figure, two peaks observed around 7.5 s correspond to the relatively standard throwing and catching of the frisbee. Occasionally, due to the tester's skill level, the throwing/catching posture may not be particularly elegant or standard, resulting in a signal that does not exhibit a standard double peak. This demonstrates the application of TGTMW fiber‐based sensors in complex motion evaluation. The complete test video of the two experiments is provided in Movie S3 (Supporting Information).

Furthermore, TGTMW multi‐walled fibers enable unique applications that traditional pressure‐sensitive sensors cannot achieve. For instance, by connecting three TGTMW fibers in parallel, the assembled pressure‐sensitive sensor (The photo and schematic diagram are shown in Figure S22, Supporting Information) can not only detect traditional pressure signals but also capture more detailed information. As shown in Figure 6b‐I, it enables differentiation between finger press and slide actions. The principle behind this is that for pressing, all three fibers experience pressure simultaneously, whereas for sliding, the three fibers experience pressure sequentially. If sliding occurs at a speed similar to that of using a touch screen, the pressure on each fiber may be spaced ≈0.02 s apart, which appears as a multiple peak pattern with a separation of around 20 ms in the image. Therefore, the press and slide exhibit different peak shapes. While it may be difficult to distinguish these peak shapes in second‐scale images, subsequent data processing techniques such as wavelet transform can be used to visualize and differentiate press/slide actions. This function requires the sensor to have a slender structure and a fast response time, which is difficult to achieve with non‐fiber‐shaped pressure‐sensitive sensors.

We used an Arduino Uno microcontroller to collect data (The code and methodology are shown in Figure S23, Supporting Information). The collected data was processed using continuous wavelet transform (Morlet wavelet), enabling automatic differentiation of press/slide actions after subsequent processing. We demonstrated a simple application using Morse code. Traditional pressure‐sensitive sensors typically use long press/short press or strong press/weak press to input Morse code, which is time‐consuming or prone to errors. However, using TGTMW fiber‐based pressure‐sensitive sensors, “press” can represent “·” and “slide” can represent “−”, allowing for high‐speed and accurate data collection. As shown in Figure 6b‐II, taking the Morse code for “graphene” as an example, it is difficult to distinguish between “·” and “−” in the 30‐s scale image. However, by zooming into a specific region of the image, as shown in Figure 6b‐III, within the 3.8‐s interval from 11.7 to 15.5 s, individual peaks (press) and triple peaks (slide) can be clearly identified. Furthermore, in the 250‐ms magnified section from 13.75 to 14.00 s, the triple peak characteristic representing a slide can be observed. The shortest duration of the individual rising/falling stages is 12 ms, indicating that the TGTMW sensor has an extremely fast response time of less than 12 ms, far superior to other types of pressure‐sensitive sensors. However, manually or visually distinguishing between single peaks (press) and triple peaks (slide) is inefficient. Therefore, the continuous wavelet transform (CWT) can be used for automatic and efficient differentiation.

Figure 6b‐IV presents a continuous wavelet transform scalogram of high‐speed sampled data over 30 s, where the differences between press/slide peaks can be clearly observed. Due to the presence of triple peaks, the slide exhibits relatively strong signals in the higher frequency region (Scale = 60), whereas press peaks do not have this characteristic. Furthermore, as shown in Figure 6b‐V, we extracted intensity curves A and B by cutting across the wavelet scalogram at Scale = 240 and Scale = 60, respectively. The press peaks exhibit relatively strong signals on curve A (Scale = 240, low‐frequency region), while slide peaks exhibit stronger signals on curve B (Scale = 60, high‐frequency region). By applying the formula, A−5 × B and performing a 300‐point sliding smoothing, we obtained a characteristic curve representing the peak patterns. If the value at a specific time point is less than 0, it indicates a slide signal detected by the TGTMW fiber‐based sensor. If it is greater than 0, it indicates a press signal detected by the sensor, thus enabling the differentiation of external stimuli. Additionally, in the supporting materials, we further tested additional external stimuli such as fast slide, slow slide, press, and fast tap. The corresponding wavelet transforms process (Figure S24, Supporting Information) and analyses (Figure S25, Supporting Information) further expanded its functionality. In addition, as shown in Figure S26 (Supporting Information), comprehensive biocompatibility evaluation, including live/dead fluorescence staining and quantitative CCK‐8 analysis over 5 days, consistently demonstrates that the TGTMW fiber exhibits excellent biocompatibility with no significant cytotoxic effects on L929 fibroblast cells.^[^ ^44^ ^]^ This feature can be utilized in artificial tactile applications, as merely sensing pressure is far from sufficient for electronic skin.

In conclusion, in Figure 6, we illustrate two application examples demonstrating that TGTMW fibers not only exhibit excellent performance in traditional pressure‐sensitive sensor applications but also leverage their structural advantages to achieve unique functionalities, showcasing significant application potential.

**Figure 7:**
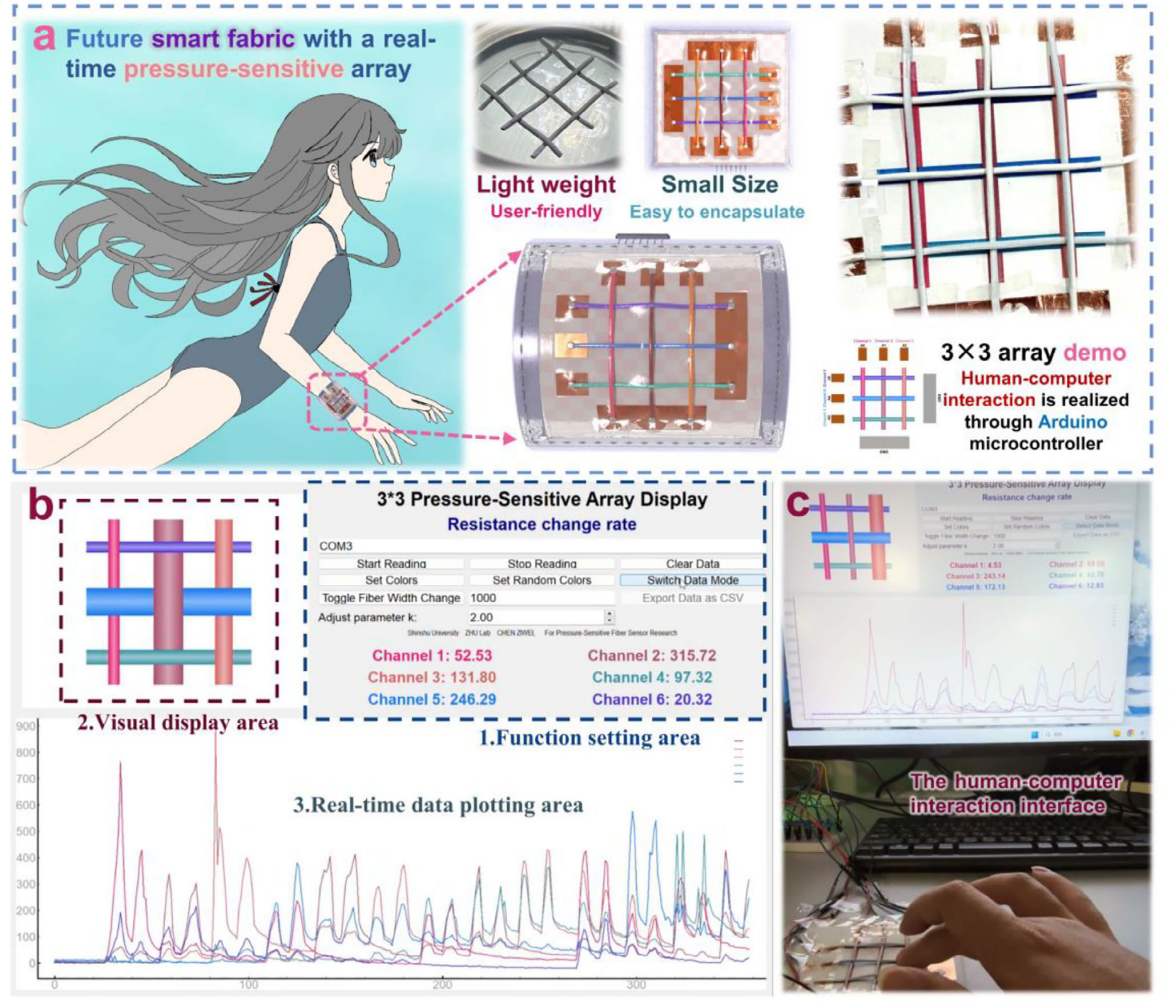
Demonstration of the real‐time pressure‐sensitive array application enabled by TGTMW fibers. a) Potential application scenarios and advantages, b) 3 × 3 array testing interface, c) 3 × 3 array application test photograph.

**Figure** **7** presents another application of TGTMW fibers, where a pressure‐sensitive signal monitoring system for human‐machine interaction is achieved through a multi‐channel TGTMW fiber array, resembling a woven structure. While numerous studies have explored pressure‐sensitive arrays using traditional pressure sensors, these arrays often suffer from large volume and complex wiring, significantly limiting their application scenarios. As illustrated in Figure 7a, TGTMW fibers hold great potential for future human‐machine interaction devices in specialized environments. On one hand, TGTMW fibers are lightweight, ensuring no burden on the human body. More importantly, their fiber structure offers compact size and shape adaptability, making them easy to encapsulate for additional functionalities such as waterproofing. In the future, multiple TGTMW fibers can be assembled into an array to develop an intelligent wristband for deep‐sea applications. By pressing different positions on the wristband, users can send various signals, such as “All Good,” “Task Completed,” or “SOS.”

Due to experimental constraints, we have currently fabricated only a 3 × 3 array for preliminary testing, with the sample preparation process shown in Figure S27 (Supporting Information). As depicted in Figure 7b, to visually demonstrate the application of the 3 × 3 pressure‐sensitive array fabricated from TGTMW fibers, we developed a simple program that connects the array to an Arduino Uno microcontroller. The left window of the interface visually displays the pressure distribution on the fiber array. In the displayed instance, the center point is pressed, and the bottom section of the interface shows the resistance variation of six fibers, providing data for signal analysis and further processing. The corresponding test photograph is shown in Figure 7c, where the real‐time display of the pressing position on the computer screen precisely matches the actual pressing location. The demonstration video, available as Movie S4 (Supporting Information) (including recorded footage and screen recording), further highlights the unique properties and advantages of TGTMW fibers in pressure‐sensitive array applications.

**Figure 8:**
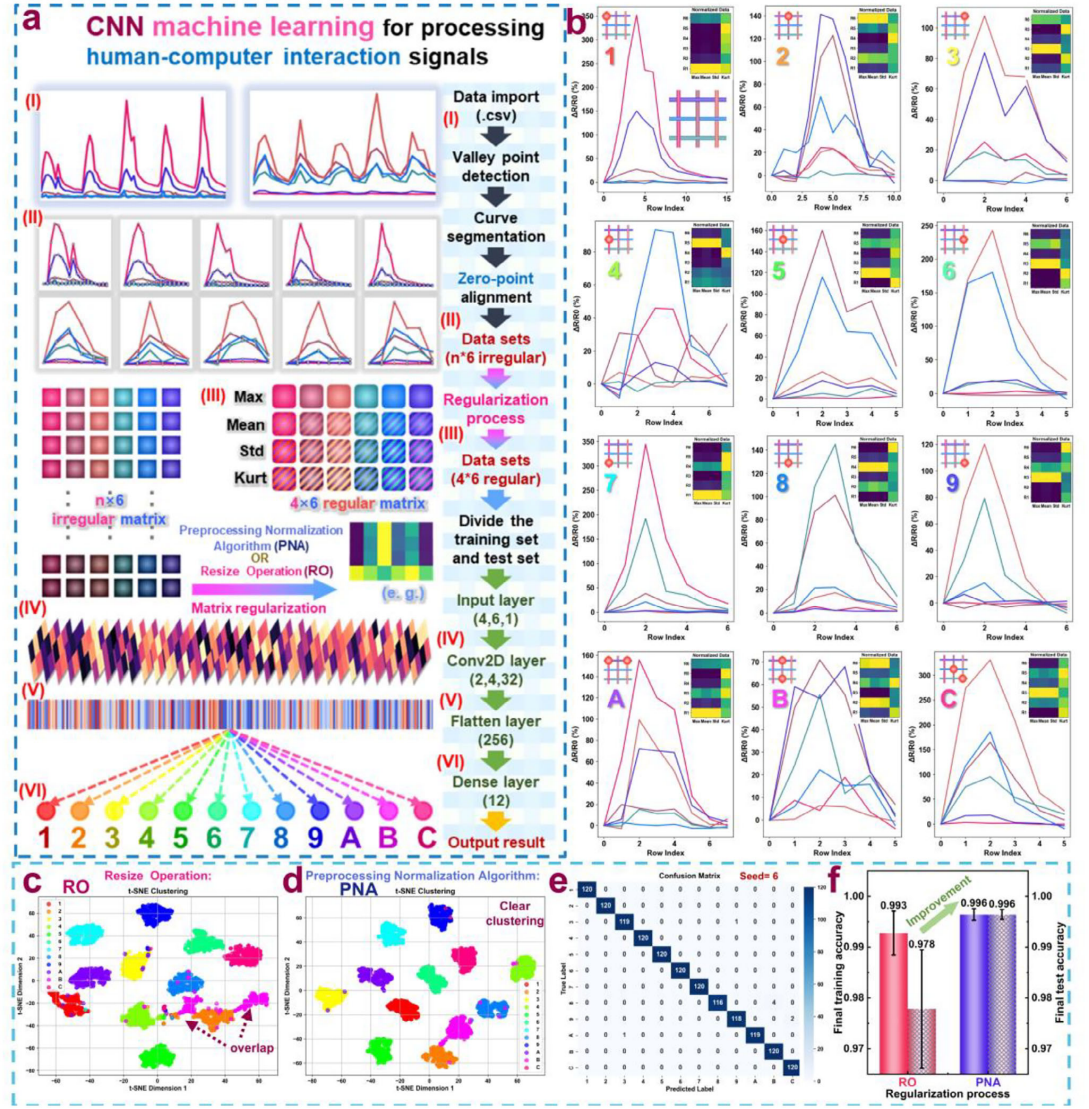
The use of convolutional neural networks (CNN) machine learning to achieve signal differentiation in a 3 × 3 pressure‐sensitive sensing array fabricated with TGTMW fibers; a) Illustration of the training algorithm, b) display of the six‐channel data from the 3 × 3 pressure‐sensitive array and the matrix processed by the Preprocessing Normalization Algorithm (PNA) c, d) t‐SNE dimensionality reduction plots for different data preprocessing methods (PNA and RO), e) confusion matrix after data preprocessing using PNA, f) performance differences in training under the two data preprocessing algorithms.

As shown in Figure 7, we used six TGTMW fibers to fabricate a 3 × 3 pressure‐sensitive array capable of real‐time monitoring of compressions at different positions. We utilized relevant programs to display applications through images and videos. To further enhance this application and enable functionalities such as automatic alarm activation/deactivation, headlamp activation, and camera activation upon touching a specific position on the 3 × 3 pressure‐sensitive array in extreme conditions, the information from the array should be automatically classified and recognized. We adopted convolutional neural networks (CNN) machine learning as the classification method, which is demonstrated in **Figure** **8**.

As illustrated in Figure 8a, we present the method of processing the six‐channel electrical signals from the 3 × 3 pressure‐sensitive array made of TGTMW fibers and ultimately recognizing them as actual human‐machine interaction information through CNN machine learning. First, the collected resistance variation rate signals, as shown in Figure S28 (Supporting Information), form a continuous six‐channel signal. Due to the inherent elasticity of TPU, there is some baseline drift in the signal. Therefore, the first step is to automatically segment the continuous signal (I) and shift each channel's signal along the Y‐axis so that its first point is zero (II). This operation avoids the influence of baseline drift on signal differentiation. After this step, the corresponding data for each pressure‐sensitive action becomes an n × 6 matrix, where n represents the duration of the compression action in time steps. For example, if the action lasts 400 ms, n = 8. Since the duration of each pressure‐sensitive action varies, the corresponding data matrices are irregular and require subsequent normalization before machine learning. Considering computational power and energy consumption in practical applications, we chose a 4 × 6 matrix, which was used for CNN machine learning. The small matrix size allows for rapid computation using only a single convolutional layer. In the process of converting the n × 6 matrix into a 4 × 6 matrix (III), we designed a method where each row's values correspond to the maximum value, mean value, standard deviation, and kurtosis of the signal in that channel over time. After normalization, we obtained a 4 × 6 matrix, named the Preprocessing Normalization Algorithm (PNA). As a comparison, we simply selected rows 2 to 5 of the original matrix (excluding the first row, which is all zeros due to baseline correction) and named this method the Resize Operation (RO). The specific code and explanation are provided in Figure S29 (Supporting Information). After passing through only one convolutional layer (IV), the matrix was flattened (V) and finally classified into 12 different categories by a fully connected layer. The model structure is shown in Figure S30 (Supporting Information). Through this series of processes, we successfully converted the six‐channel electrical signals from TGTMW fibers into recognized pressing patterns.

For the 12 different compression modes, we collected 240 compression signals per mode, totaling 2 880 compression signals for CNN machine learning. We split them into a 50% test set and a 50% training set. As shown in Figure 8b, a portion of the test data for machine learning is displayed. The line graph represents the segmented and baseline‐corrected data, and the transposed PNA‐processed matrix is placed in the upper‐right corner of the graph for better visualization. Additionally, the complete dataset was uploaded as a 96‐page PDF as supporting material. We fixed random seeds 0–9 and conducted 10 CNN machine learning tests and t‐SNE dimensionality reductions using the 2 880 4 × 6 matrices processed by the PNA and RO algorithms. The corresponding t‐SNE plots and confusion matrices of the test results are shown in Figure S31 (Supporting Information). Taking seed = 6 for example, as shown in Figure 8c,d, some data points under the RO algorithm overlap, while the PNA algorithm results in better separation, demonstrating that our PNA algorithm effectively distinguishes the signals from the pressure‐sensitive array made of TGTMW fibers. Even in the worst training result (seed = 6), as shown in Figure 8e, the classification performance remains excellent. The training curves are presented in Figure S32 (Supporting Information).

Furthermore, as shown in Figure 8f, the results of 10 training tests indicate that, after preprocessing with the PNA algorithm, the 6‐TGTMW fiber‐based pressure‐sensitive array achieved an average recognition accuracy of 99.6% for 12 different compression modes using only a 4 × 6 small matrix, highlighting its potential in human‐machine interaction. Additionally, we conducted further CNN machine learning analysis to address another potential issue in practical applications of TGTMW fiber arrays: false triggering that may occur when pressing the fiber segments located between the target node and its adjacent nodes. Using similar principles, we performed CNN machine learning as illustrated in Figure S33 (Supporting Information). The results demonstrate that the TGTMW‐designed array, combined with the machine learning methodology established in Figure 8, achieved ≈96% identification accuracy for this type of error. The corresponding data is provided as supporting documentation in PDF format. These findings indicate that the fiber design and CNN machine learning algorithm developed in this work not only accurately identify compression states but also exhibit excellent resolution for distinguishing false triggering events. Consequently, the system demonstrates strong compatibility and significant application value for practical implementation.

## 3 Conclusion

This work takes the relatively limited research on fiber‐based pressure‐sensitive sensors as a starting point. By analyzing the characteristics and differences between fiber‐based structures and common film/aerogel pressure‐sensitive sensor structures, we were inspired to design and fabricate TGTMW fibers with a unique multi‐wall structure through controlled wet spinning. SEM analysis from different angles and scales revealed that the inner walls of the fibers exhibit a certain degree of orientation (anisotropy) of GNPs, and we proposed a formation mechanism for TGTMW fibers. In pressure‐sensitive performance tests using a desktop tensile‐compression machine, TGTMW, due to its significantly smaller volume—only a little fraction of traditional pressure‐sensitive sensors—exhibited some limitations in peak linearity. However, it demonstrated sufficient sensitivity and applicability (suitable for partial area compression of different sizes). Based on its performance and structure, we inferred the mechanism of resistance increase under compression. The main reason is that the inner wall of the transition region between the compressed and uncompressed areas undergoes distortion, leading to a deterioration in the electrical conductivity of the internally oriented graphene. In practical applications, TGTMW far outperforms existing pressure‐sensitive sensors. In common use cases, such as remote human motion monitoring, TGTMW exhibited accurate, real‐time, and stable performance. Furthermore, thanks to its unique fiber structure, three fibers can be assembled in parallel to form a pressure‐sensitive sensor capable of distinguishing between different stimuli, such as pressing and sliding. Finally, we designed a 3 × 3 pressure‐sensitive array and demonstrated its performance and functionality through programming, CNN‐based machine learning. This highlights its potential as a human‐machine interaction device in extreme environments in the future.

Overall, in this study, we explored a novel structure and working mechanism to develop a pressure‐sensitive sensor with an innovative configuration. Through a comparison with other resistive‐type pressure sensors^[^ ^16^, ^31^, ^45^, ^46^, ^47^, ^48^, ^49^ ^]^ (Table S2, Supporting Information), the TGTMW fiber‐based pressure sensor demonstrated several distinctive features, including a rare fiber‐shaped structure, a unique resistance‐increasing mechanism, and a partial compression response capability, making it suitable for specialized applications. This work represents a pioneering advancement in the field of fiber‐shaped pressure‐sensitive sensors.

## 4 Experimental Section

### Materials

Thermoplastic polyurethane (TPU) was provided by Covestro AG, Germany (product code 385SX). N,N‐Dimethylformamide (DMF) was sourced from Fujifilm Wako Pure Chemical Corporation, Japan. Graphene nanoplatelets (GNPs) were supplied by Strem Chemicals, USA, with specifications of 6–8 nm in thickness and ≈5 µm in width. Nano‐TiO_2_, used as an additive for the shell structure and having a primary particle size of 21 nm, was provided by Sigma–Aldrich, USA. All chemicals were of analytical grade and were used as received. Deionized water was utilized in all experiments.

### Slurry Configuration—Core Part

Add 8.6 g of DMF into a thoroughly clean and dry container, then add 0.4 g of graphene nanoplatelets (GNPs). To ensure better dispersion of the GNPs, stir the mixture and subject it to ultrasonic dispersion for 1 h. Then, add 1 g of TPU particles and heat while stirring at 90 °C for 10–20 h in a dry environment until the TPU was completely dissolved; follow with another ultrasonic treatment for 0.5 h and stirring for 0.5 h, repeating the cycle three times to ensure complete dispersion of the GNPs.

### Slurry Configuration—Shell Part

Add 7.6 g of DMF into a thoroughly clean and dry container, then add 0.4 g of nano‐TiO_2_. To disperse the nano‐TiO_2_, stir the mixture and subject it to ultrasonic dispersion for 1 h. Then, add 2 g of TPU particles and heat while stirring at 90 °C for 10–20 h in a dry environment until the TPU was completely dissolved and obtain a white slurry. Follow with an additional ultrasonic dispersion for 0.5 h to further ensure complete dispersion.

### Wet‐Spinning Process

To achieve precise control over the dimensions of the core–shell structure, two syringe pumps with 2 cm diameter syringes were used to independently control the spinning speeds of the core and shell layers, both of which were connected to a coaxial needle for wet spinning. The coaxial needle employed has an outer diameter corresponding to 17 gauge and an inner diameter corresponding to 22 gauge. Environmentally friendly, pure water was chosen as the coagulation bath. The horizontally positioned syringe pump was set at a slower speed of 4.0 mL h^−1^ for the shell, while the vertically positioned syringe pump was set at a higher speed (20.0 mL h^−1^) for the rapid extrusion of the GNPs‐containing core slurry. Since the phase‐separated TPU requires a certain amount of time to fully solidify, the fibers should be left to stand for more than 4 h after spinning before being retrieved, and then soaked in deionized water for over 12 h to completely remove the DMF. Finally, the fibers were naturally air‐dried at room temperature, yielding the TiO_2_/GNPs/TPU Multi‐Wall (TGTMW) fibers.

### Characterizations and Measurements

The surface morphology and structural features were observed using Field Emission Scanning Electron Microscopy (FESEM, JSM‐IT800SHL, JEOL, Japan). EDS analysis data were also obtained using the JEOL EDS system integrated with the FESEM. The optical microscopy of the fibers was conducted using a high‐speed microscope (VW‐9000, Keyence, Japan). For testing the compression sensing performance of the fibers under different compression conditions, various sizes of indenters were designed and fabricated using Nova3D whale3 light‐curing 3D printer and engineering UV‐curable resin, and then connected to a benchtop tensile compression testing machine (MCT‐2150, A&D, Japan). Both ends of TGTMW fibers were connected with an electrometer (Keithley‐6514, Keithley Instruments, USA) for resistance signal measurement. For remote sensing application tests, a Bluetooth integrated BLE‐Nano microcontroller was used, whereas all other application tests employed an Arduino Uno microcontroller. This study involved only the author's self‐experimentation with minimal risk and did not involve the collection of private or sensitive data. The research protocol was reviewed by the Faculty of Textile Science and Technology, Shinshu University, and was determined to be exempt from formal ethics approval due to its low‐risk nature and self‐experimental design. Written informed consent was obtained from the participant prior to conducting the experiments. All experimental procedures were conducted in accordance with institutional guidelines and ethical standards for self‐experimentation research.

## Conflict of Interest

The authors declare no conflict of interest.

## Supporting information



Supporting Information

Supplemental Movie 1

Supplemental Movie 2

Supplemental Movie 3

Supplemental Movie 4

Supporting Information

Supporting Information

## Data Availability

The data that support the findings of this study are available from the corresponding author upon reasonable request.
