# Supplementary material for: Fibrous Pressure Sensor with Unique Resistance Increase under Partial Compression: Coaxial Wet‐Spun TiO2/Graphene/Thermoplastic Polyurethane Multi‐Wall Multifunctional Fiber
Source: Adv Mater. 2025 Jul 16;37(40):2509631. doi: 10.1002/adma.202509631 (PMC12510290; doi:10.1002/adma.202509631)
Supplement: Supplementary file 1 — Supporting Information [file ADMA-37-2509631-s005.docx]

**Supporting information**

**Fibrous Pressure Sensor with Unique Resistance Increase under Partial Compression: Coaxial Wet-Spun TiO_2_/Graphene/Thermoplastic Polyurethane Multi-Wall Multifunctional Fiber**

Ziwei Chen^a,b^, Dandan Xie^a,b^, Kanae Kojima^b^, Chunxia Gao^c^, Jian Shi^a,b,d^, Jian Xing^e^, Hideaki Morikawa^a,d^, Chunhong Zhu ^a,b,d,*^

^a^ Graduate School of Medicine, Science and Technology, Shinshu University, Tokida, Ueda, Nagano 386-8567, Japan

^b^ Institute for Fiber Engineering and Science (IFES), Shinshu University, 3-15-1 Tokida, Ueda, Nagano 386-8567, Japan

^c^ School of Chemistry and Chemical Engineering, Yangzhou University, Yangzhou 225002, China

^d^ Faculty of Textile Science and Technology, Shinshu University, 3-15-1 Tokida, Ueda, Nagano 386-8567, Japan

^e^ School of Textile and Garment, Anhui Polytechnic University, Wuhu, 241000, China

* Corresponding author at: Institute for Fiber Engineering and Science (IFES), Shinshu University, Tokida, Ueda, Nagano 386-8567, Japan.

E-mail address: zhu@shinshu-u.ac.jp (C. Zhu).


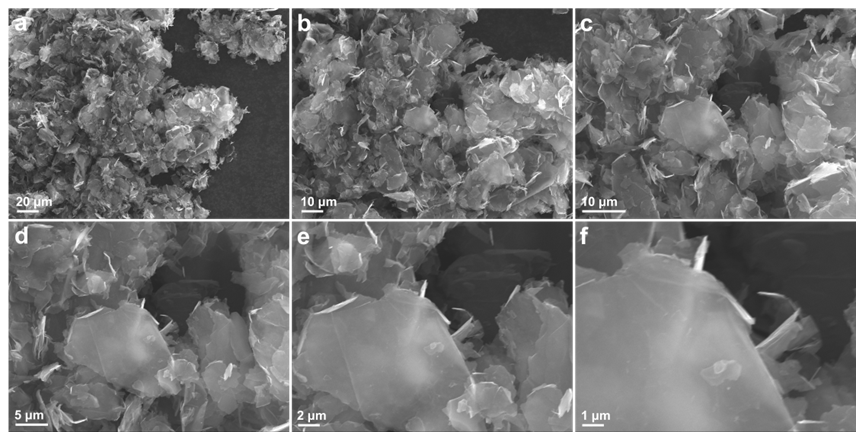


**Figure S1**. SEM Images of graphene nanoplatelets (GNPs)

As shown in **Fig.S1**, the graphene nanoplatelets (GNPs) used in this study have a width of approximately 5 μm and an extremely thin thickness. The material exhibits minimal surface impurities and possesses a large specific surface area.

**Table S1.** Effect of different GNPs contents in core spinning dope on spinning results.

| No. | Core | Shell | Coagulation Bath | Result |
| --- | --- | --- | --- | --- |
| 1 | **2% GNPs**  10% TPU | 4% TiO_2_  20% TPU | Water | Due to fiber flotation, the spinning failed, as shown in **Fig.S2**. |
| 2 | **2% GNPs**  10% TPU | 4% TiO_2_  20% TPU | 90% Water + 10% EtOH | A very fluffy multi-walled structure was obtained, which is non-conductive, as shown in **Fig.S3**. |
| 3 | **3% GNPs**  10% TPU | 4% TiO_2_  20% TPU | Water | A fluffy multi-walled structure, which is non-conductive, as shown in **Fig.S4**. |
| 4 | **4% GNPs**  10% TPU | 4% TiO_2_  20% TPU | Water | TGTMW fibers exhibit excellent performance, with structures shown in the **Fig.3** and **Figs.S5-6** |
| 5 | **5% GNPs**  10% TPU | 4% TiO_2_  20% TPU | Water | Spinning failed due to an unsuitable slurry formulation, which caused needle clogging and prevented spinning. |

As shown in **Table S1**, we investigated the effect of various GNPs contents on the internal multi-walled structure of TGTMW fibers. When the GNPs content is low (e.g., 2%), the solvent used—DMF—has a lower density than water, and the relatively low concentrations of TPU and dispersed GNPs are insufficient to increase the overall density of the spinning dope. As a result, during wet-spinning, the fibers tend to float in the coagulation bath, leading to spinning failure and contamination of the bath, as shown in **Fig.S2**. To address this, we added ethanol to the coagulation bath to reduce its density, which allowed the successful fabrication of multi-walled fibers with 2% GNPs (FESEM images were shown in **Fig.S3**). However, these fibers remained non-conductive due to the low GNPs content. When the GNPs content was increased to 3%, spinning was successful using water as the coagulation bath, but the resulting fibers still exhibited extremely poor conductivity (beyond the measurement range of a multimeter), FESEM images were shown in **Fig.S4**. At 4% GNP content, the TGTMW fibers demonstrated excellent structure and sensing performance; the fibers investigated throughout this study were prepared at this concentration. Further increasing the GNPs content led to excessive viscosity, frequently clogging the core side of the coaxial needle and resulting in spinning failure. Therefore, a GNPs concentration of 4% was chosen as the optimal value in this work.


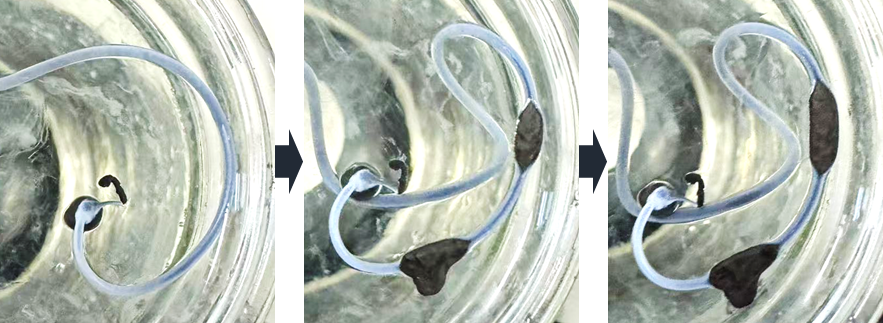


**Figure S2.** Phenomenon observed during coaxial wet spinning when GNP content is 2% and water is used as the coagulation bath.

As shown in **Fig.S2** when the GNPs content is 2%, the density of the spinning dope is lower than that of water, causing the extruded fibers to float on the surface of the coagulation bath. While floating, the shell layer of the coaxial fiber is not yet fully solidified. Internal stress from the core can lead to rupture of the fiber, resulting in leakage of the TPU/GNPs core material. This ultimately causes spinning failure and contaminates the coagulation bath. Therefore, wet spinning under these conditions (2% GNPs and water as coagulation bath) is not feasible.


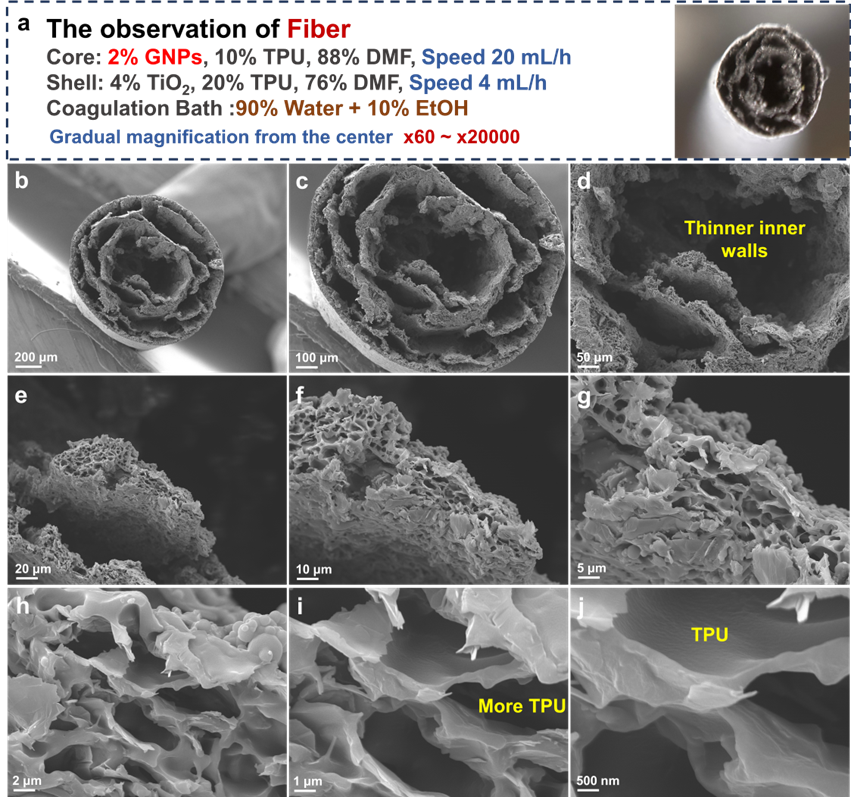


**Figure S3.** FESEM observations of fibers prepared with 2% GNPs content: (a) Macroscopic image;
(b–j) Magnified views at different magnifications (from 60× to 20,000×).

As shown in **Fig.S3**, when the GNPs content is only 2%, and wet spinning is achieved by reducing the density of the coagulation bath, the resulting fibers still exhibit a multi-walled structure. However, due to the insufficient GNPs content, no conductive pathways are formed within the fiber, rendering it non-conductive and lacking sensing capability. Compared to the multi-walled fibers discussed in this study, although both exhibit multi-walled architectures, the inner walls of the 2% GNPs fiber are thinner and more irregular in shape. This is attributed to the lower concentration of GNPs within the inner walls. Furthermore, microscopic observations reveal a higher presence of TPU in the inner walls—features that differ significantly from those of the TGTMW fibers containing 4% GNPs as described in the main text.


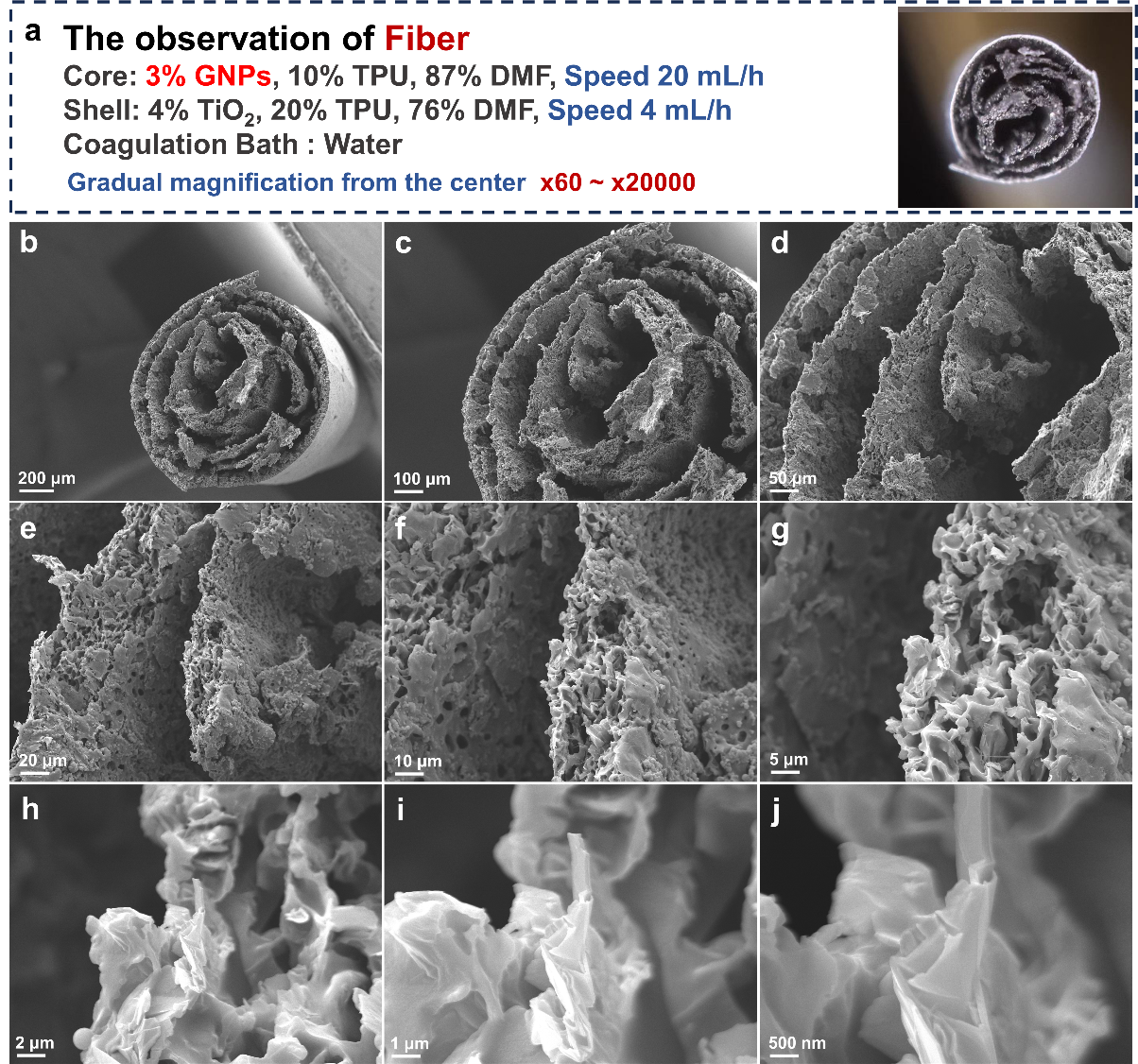


**Figure S4.** FESEM observations of fibers prepared with 3% GNPs content: (a) Macroscopic image;
(b–j) Magnified views at different magnifications (from 60× to 20,000×).

As shown in **Fig.S4**, when the GNPs content is 3%, the resulting multi-walled fiber structure lies between those of the fibers with 2% and 4% GNPs. The TPU content remains relatively high, and due to the still insufficient content of GNPs, the fiber does not exhibit electrical conductivity or sensing performance.


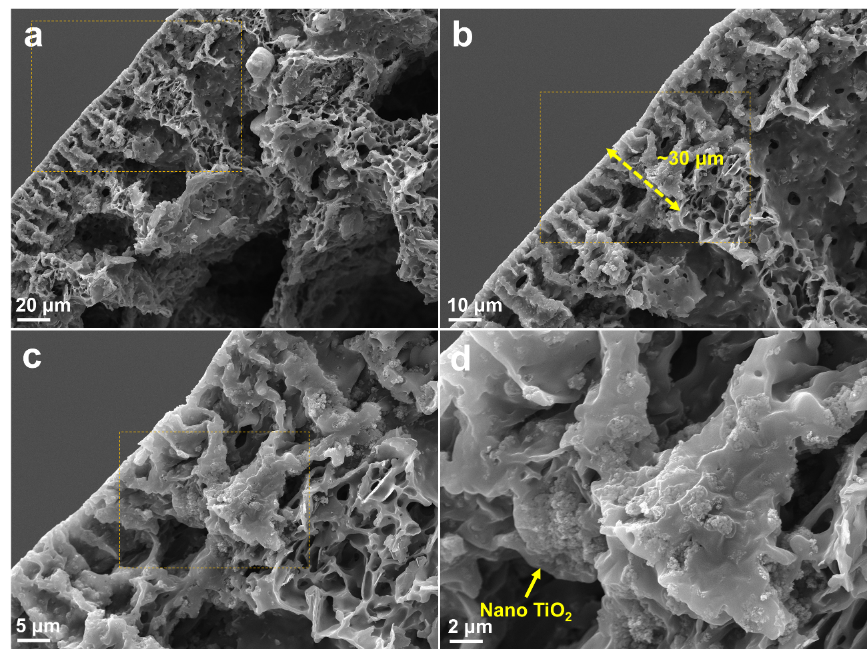


**Figure S5**. Additional SEM images of the fiber shell at different magnifications: (a) 500×, (b) 1000×, (c) 2000×, and (d) 5000×

As shown in **Fig.S5**, these SEM images provide further insights into the fiber surface at different magnifications. In **Fig.S5(a–b)**, the fiber surface exhibits a high degree of smoothness, with uniformly sized pores distributed within the approximately 30 μm-thick outer layer. These pores are likely formed during the solvent exchange process. In **Fig.S5(c–d)**, the distribution of nano-TiO_2_ within the fibers can be observed. Although some degree of nanoparticle aggregation is present, the agglomerates remain uniformly dispersed within the fiber shell, with diameters of less than 1 μm.


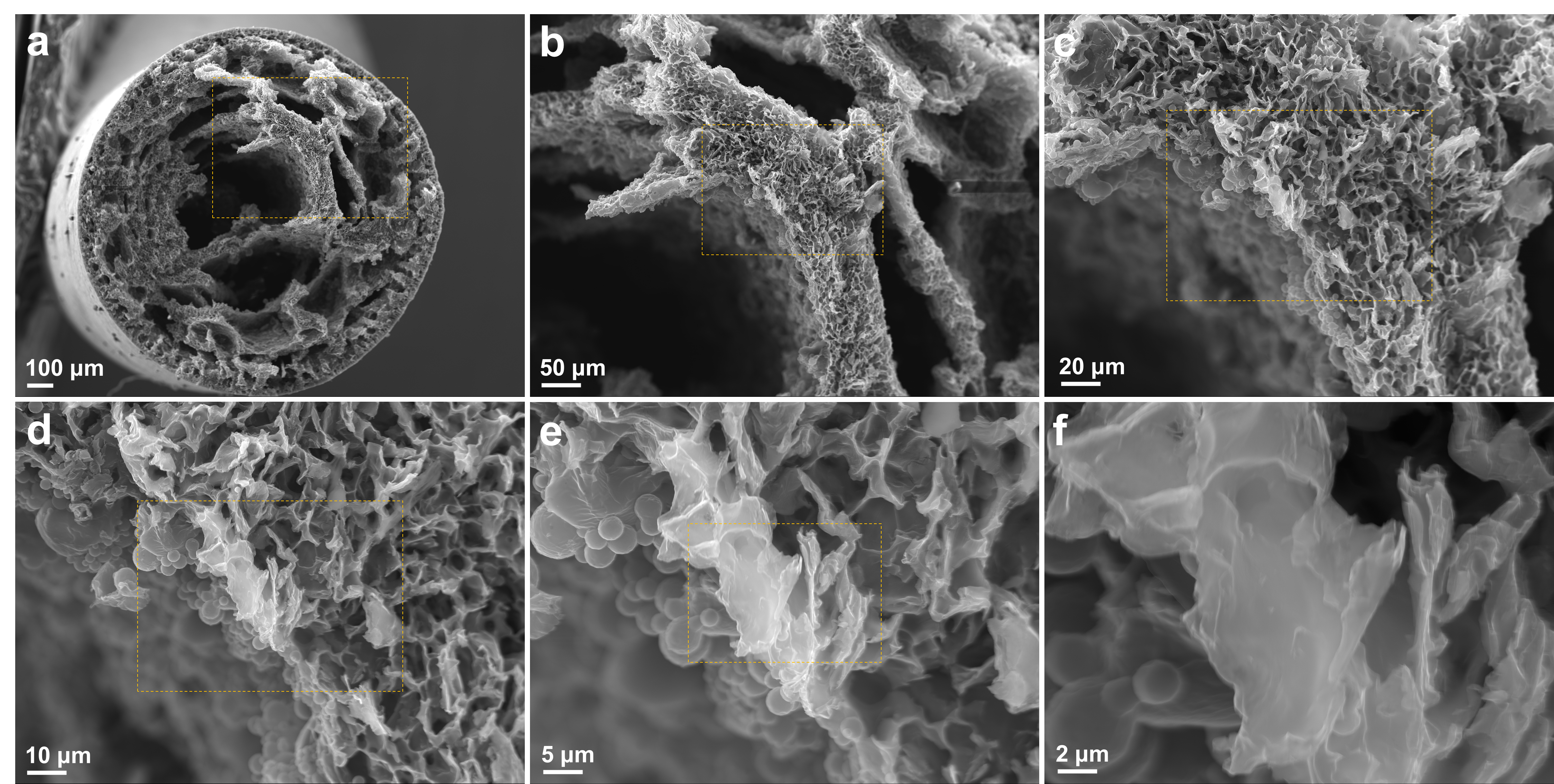


**Figure S6**. Additional cross-sectional images of TGTMW fibers

As shown in **Fig.S6**, these are additional cross-sectional images of TGTMW fibers. **Figs.S6(a–c)** illustrate a gradual magnification process from a complete fiber, revealing the internal structural features of the multi-walled TGTMW fibers.

In **Figs.S6(d–e)**, some TPU microspheres appear at the edges of the wall-like structures. Based on the mechanism proposed in **Fig.3(c)**, these microspheres may have precipitated during the final DMF removal process, as a small amount of TPU remained in the DMF/H_2_O phase after fiber formation. Additionally, graphene nanoplatelets can be observed at the edges. In **Figs.S6(e–f)**, the GNPs structure is clearly visible. These images provide supplementary insights into the structural features at the inner wall edges of the multi-walled fiber.


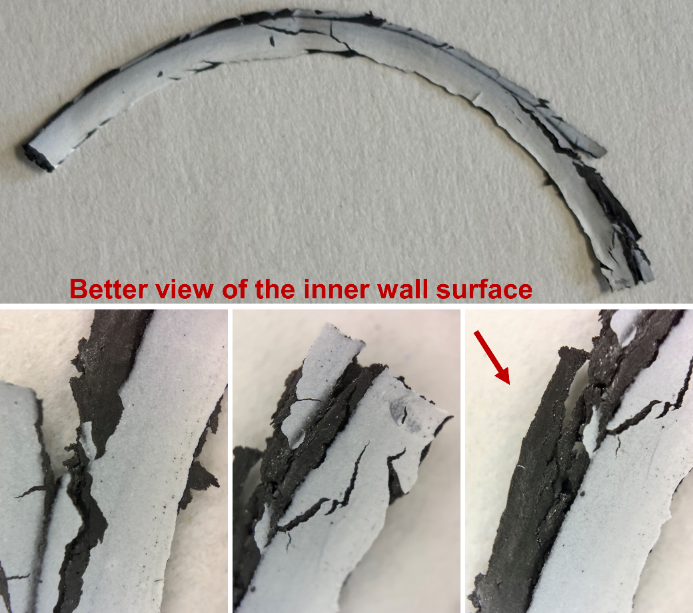


**Figure S7**. Crushing TGTMW fibers in liquid nitrogen for better observation of the inner wall surface

As shown in **Fig.S7**, to better observe the inner wall surface of TGTMW fibers, we cooled the fibers in liquid nitrogen and immediately crushed them using a heavy object. The principle behind this method is that liquid nitrogen lowers the TPU temperature below its glass transition temperature, making it brittle. When subjected to compression by a heavy object, the internal structure crushes, exposing the TPU/GNPs inner wall structure for clearer observation.


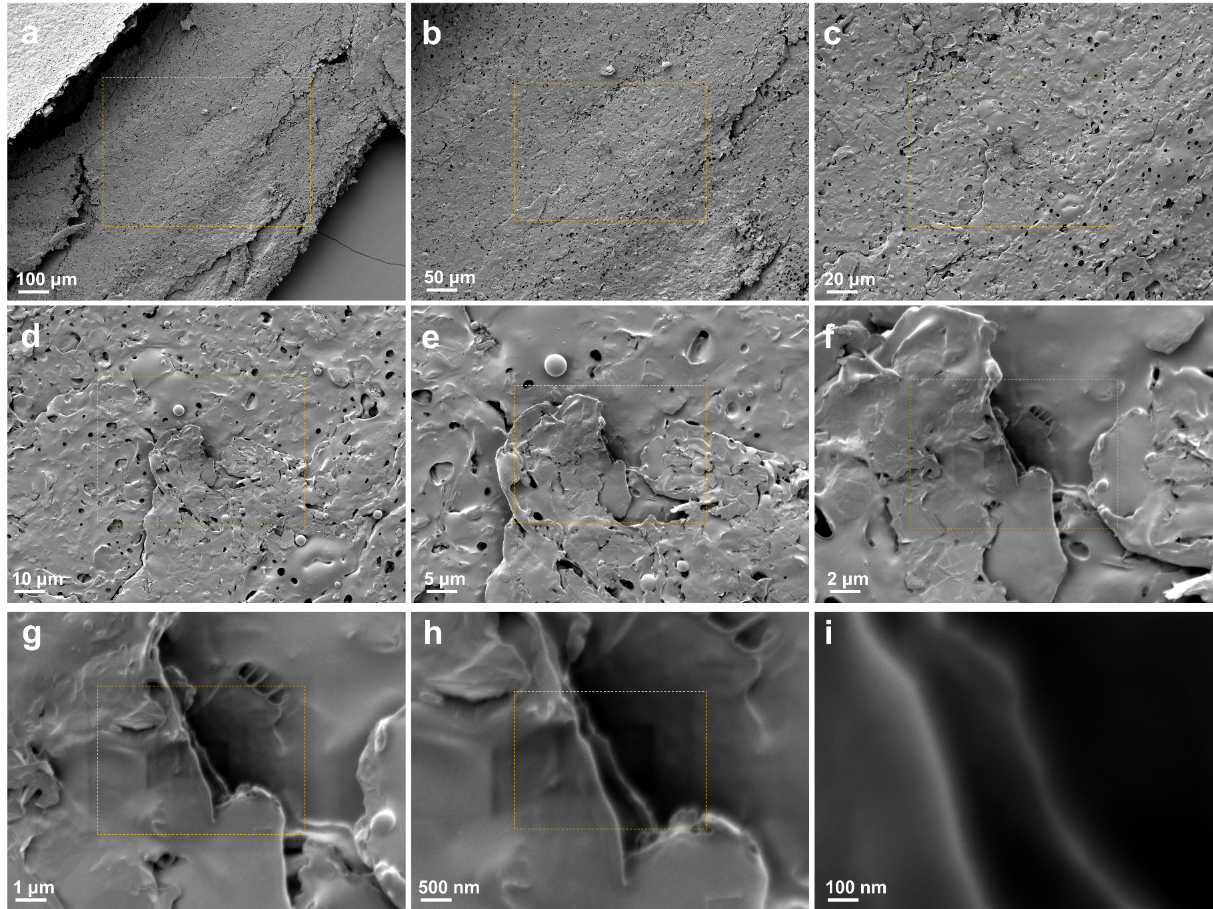


**Figure S8.** SEM images of the inner wall of TGTMW fibers after crushing.

As shown in **Fig.S8**, these are SEM images of the crushed inner walls of TGTMW fibers. From **Fig.S8(a)** to **Fig.S8(i)**, the magnification gradually increases from 100× to 100,000×, progressively zooming in from the central region.

In the low-magnification SEM images **(Fig.S8(a–c))**, the lateral view of the inner wall edges appears relatively smooth. This surface structure differs significantly from the cross-sectional structures shown in **Fig.3(a)** and **Fig.S6**, suggesting that the fiber's inner wall is oriented along the fiber axis. This provides evidence of anisotropy within the fiber's inner wall. In the medium-magnification SEM images **(Fig.S8(d–f))**, sheet-like graphene nanoplatelets (GNPs) can be seen closely adhering to the inner wall. This observation indicates a certain degree of affinity between TPU and GNPs, as well as a degree of orientation of GNPs along the fiber axis. Furthermore, in the high-magnification SEM images (**Fig.S8(g–i)**), a detailed observation of GNPs is possible. The graphene thickness is measured at approximately 5–10 nm, which aligns with the description provided in the sample specifications.


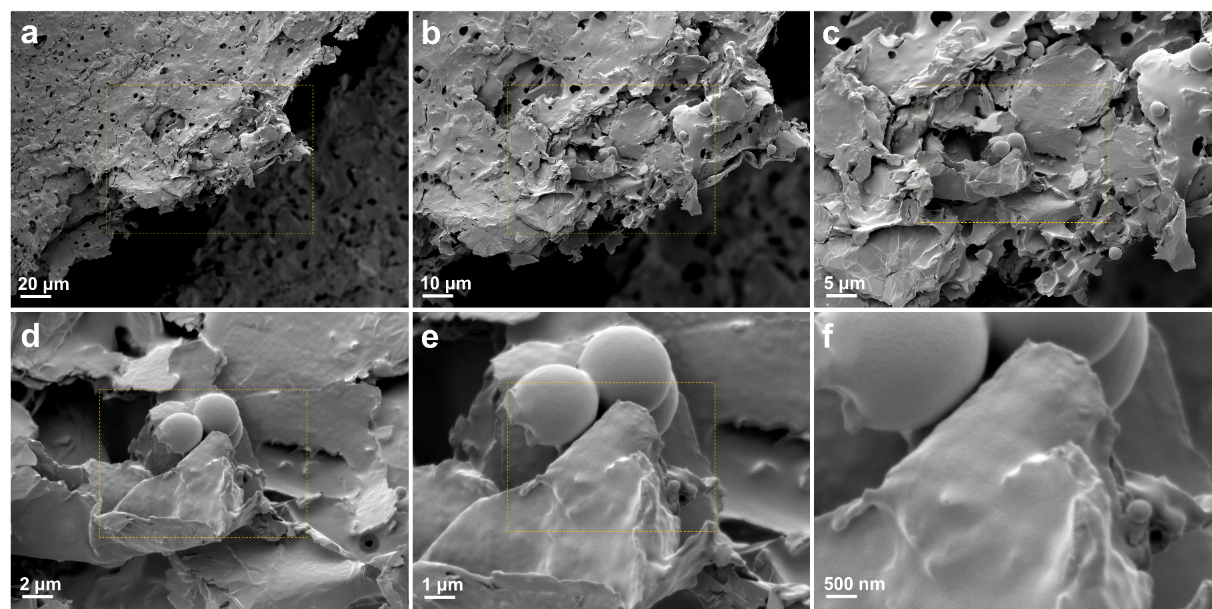


**Figure S9**. SEM images of the inner wall of TGTMW fibers after crushing (fracture region).

As shown in **Fig.S9**, these are SEM images of the fractured region of the inner wall of TGTMW fibers after crushing. From **Fig.S9(a)** to **Fig.S9(f)**, the magnification gradually increases from 500× to 20,000×, progressively zooming in from the central area. In **Fig.S9**, layered graphene nanoplatelet (GNPs) stacking can be observed, providing supplementary structural information on TGTMW fibers.


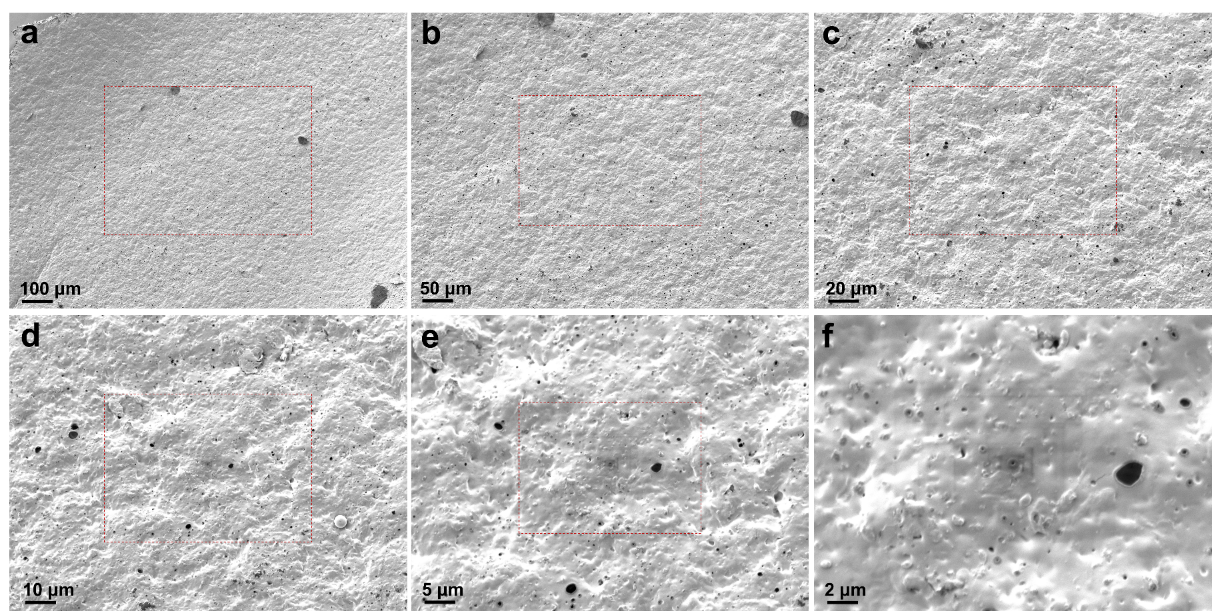


**Figure S10.** SEM images of the TiO_2_/TPU shell structure of TGTMW fibers.

As shown in **Fig.S10**, these are SEM images of the TiO_2_/TPU structure on the fiber surface. When the fibers were crushed using the method described in **Fig.S7**, the fiber surface formed a nearly flat plane instead of maintaining its original cylindrical shape. This flattening made it easier to focus and observe under SEM.

From **Fig.S10**, it can be seen that the fiber surface is relatively smooth, without noticeable shrinkage or irregular undulations. This is primarily due to the composition of the shell-layer slurry. The addition of TiO_2_ increased the slurry’s viscosity and influenced the solvent exchange rate with water, allowing the shell layer to rapidly solidify upon contact with the coagulation bath.


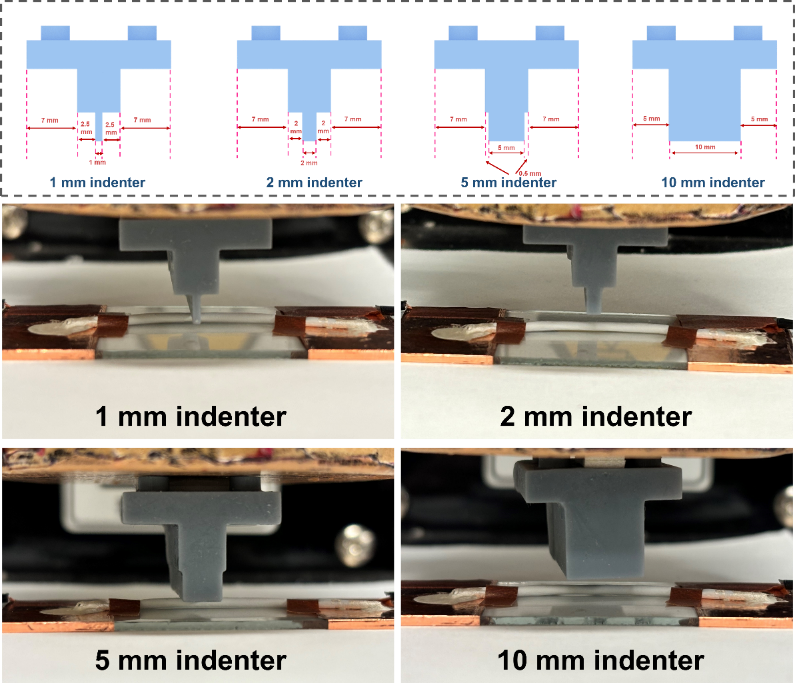


**Figure S11.** The design schematics of indenters and photographs of the compression testing apparatus.


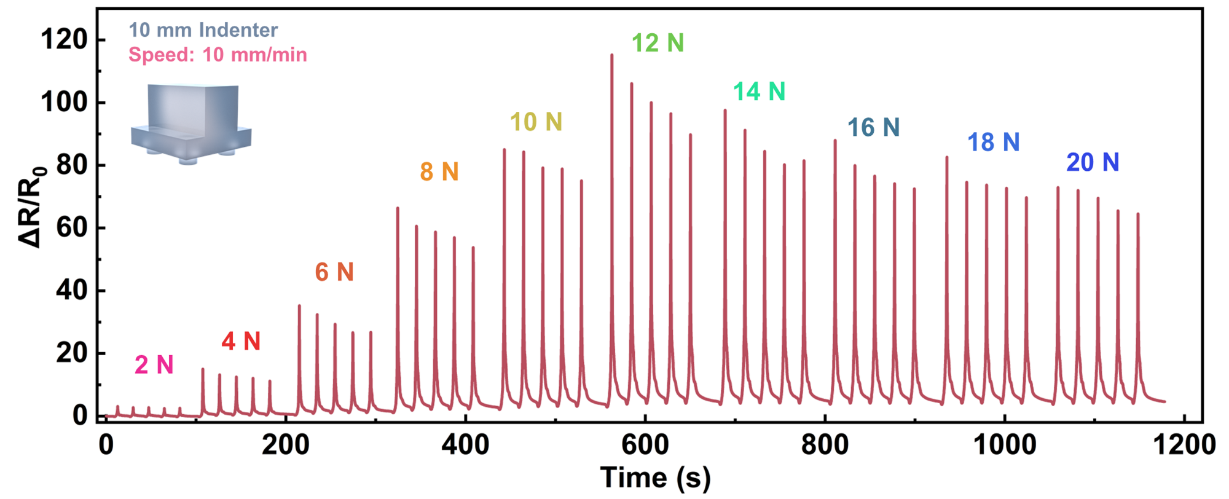


**Figure S12**. Resistance change behavior of a 4 cm-long TGTMW fiber under compression using a 10 mm-diameter indenter.

As shown in **Fig.S12**, when the TGTMW fiber was compressed using a 10 mm indenter, the resistance change rate started to decrease as the applied force exceeded approximately 12 N. This trend is consistent with the results shown in **Fig.4(d)**, where a 5 mm indenter was used and a similar decrease was observed at higher pressures. This phenomenon may be attributed to the fiber being fully flattened under large force (as illustrated in **Fig.S13** under high-pressure conditions), leading to a limit in the bending of the compressed transition region. Moreover, the decrease in resistance may result from the internal graphene nanosheets becoming more compact under pressure, thus reducing electrical resistance.


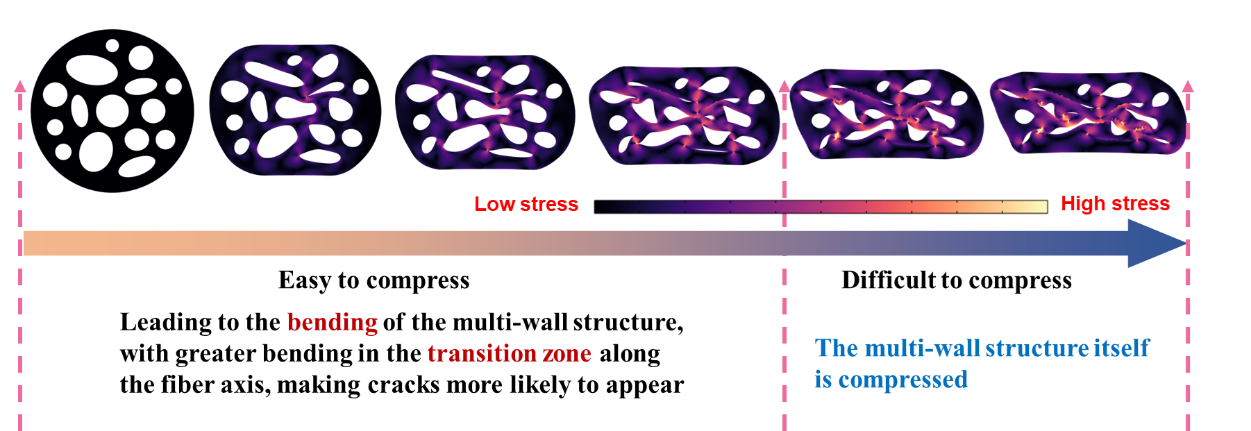


**Figure S13**. Compression response mechanism of TGTMW fiber multi-wall structures under different pressure conditions.

As shown in **Fig.S13**, we developed a simple qualitative model to simulate the TGTMW fiber multi-wall structure behavior. Under low pressure, the fiber response primarily involves bending deformation of multi-wall structures, causing microcracks along certain inner walls in the fiber axial direction (as shown in **Fig.S17**). This obstructs conductive pathways along the circuit direction, ultimately resulting in significant resistance increase. When multi-wall structure deformation reaches its limit—essentially when the structures are compressed together—continued compression affects the inner walls. At this point, further bending along the fiber axis increases minimally, and compression of the TPU/GNPs composite in the inner walls may actually reduce interlayer contact distances between GNPs (Under high pressure, the stacking of graphene can be clearly observed by FESEM, as shown in **Fig.S14**), potentially causing resistance changes in certain segments.


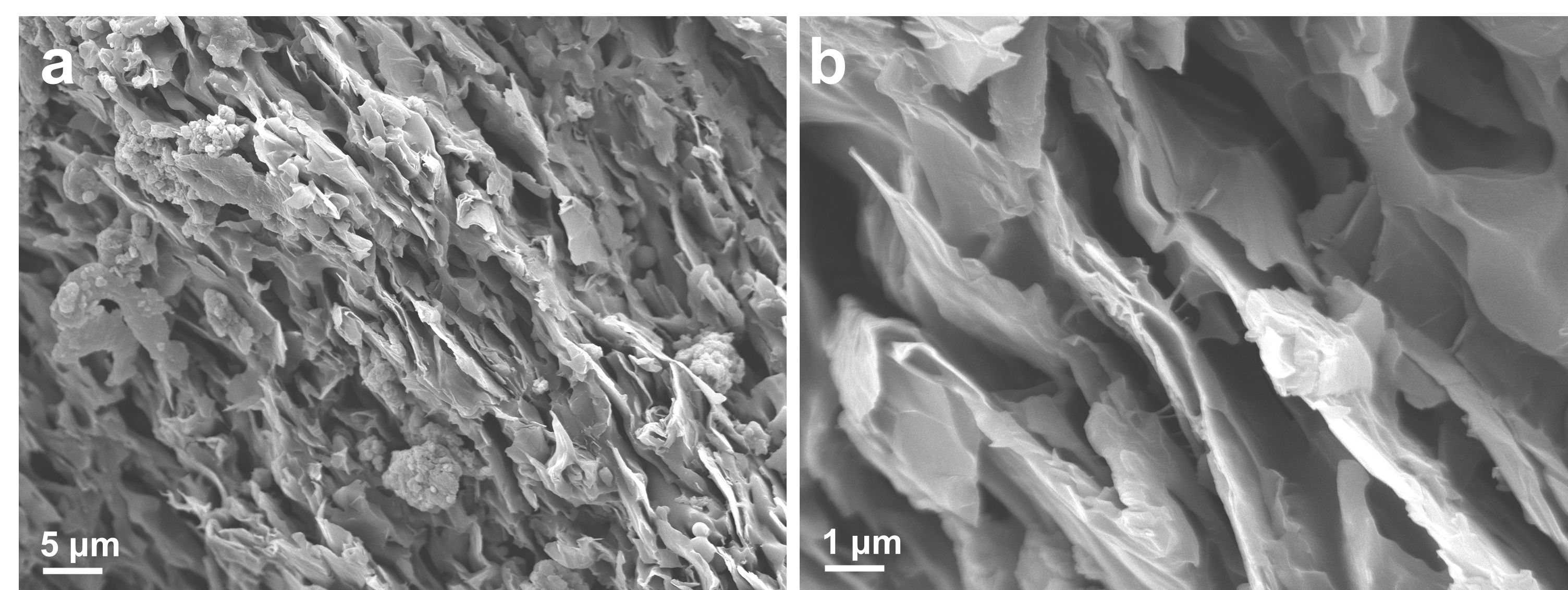


**Figure S14**. FESEM images of the inner wall changes of TGTMW fibers after being subjected to high pressure

After being subjected to high pressure, the conductive network of GNPs inside the TGTMW fiber becomes more compact, and the shortened distance between the GNPs leads to a reduction in resistance to some extent. Due to the excellent elasticity of TGTMW fibers, even under strong compression with a 5 mm diameter indenter—such as a force of 20 N—the fibers gradually rebound during the SEM imaging process. To fix this phenomenon for better electron microscope observation, we simulated an extreme compression condition (over 100 N force maintained for 5 minutes). Under these conditions, the compressed state of the inner wall can be clearly observed. As shown in **Fig.S14**, the conductive network between graphene sheets becomes significantly denser.


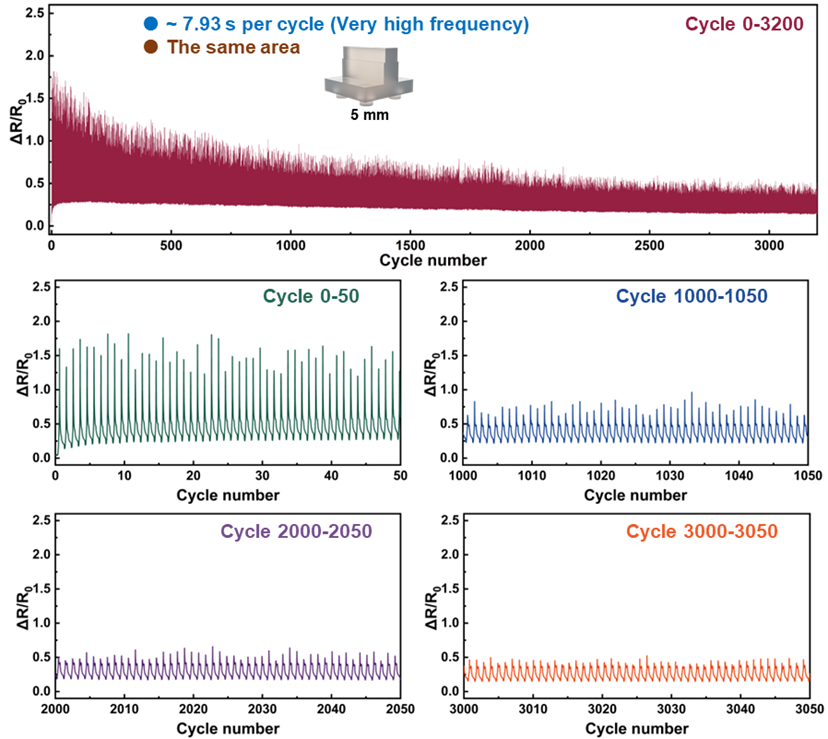


**Figure S15**. 3200 Cycles test on the same area of TGTMW fibers using a 5 mm indenter with 0.8 N force

As shown in **Fig.S15**, the TGTMW fibers were subjected to 3200 compression cycles using a 5 mm indenter under a pressure of 0.8 N. Although the signal gradually diminishes during cycling, the sensor continues to function properly and maintains stable peak shapes with high signal-to-noise ratio, demonstrating its ability to operate stably over long-term use. Furthermore, due to the fibrous structure of the TGTMW fibers, electrical disconnection does not occur after extended cycling. Each small segment can act as an independent sensing unit, significantly extending the sensor's lifetime beyond 3200 cycles. While the signal intensity declines with repeated use, the signal itself does not become erroneous or noisy, and this attenuation can potentially be compensated for using algorithms in future applications.


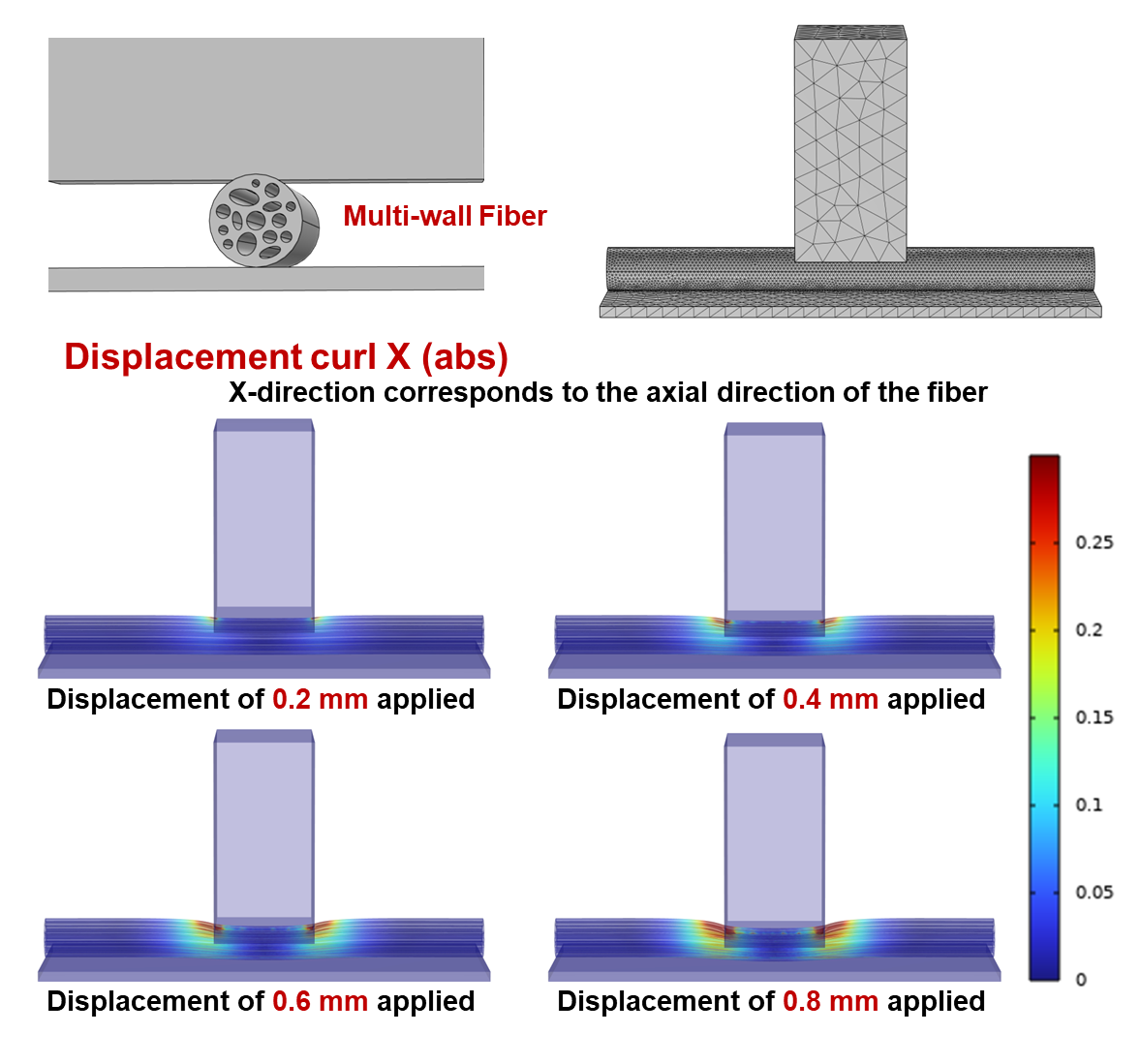


**Figure S16.** Finite element simulation results of a multi-walled structured fiber under axial compression using COMSOL Multiphysics.

As shown in **Fig.S16**, we conducted a three-dimensional finite element simulation using COMSOL Multiphysics to investigate the mechanical behavior of a multi-walled structured fiber under compressive loading. The model simulates a rigid indenter gradually applying axial displacement from the top of the fiber, inducing both global deformation and localized structural response. The primary aim is to understand how the internal multi-wall structure responds to compression and its potential impact on the macroscopic properties of the fiber.

To accurately capture the local mechanical behavior arising from the complex geometry, a finely meshed model was employed. An unstructured tetrahedral mesh was used, consisting of 378,845 tetrahedral elements, 85,189 nodes, 100,850 triangular surface elements, 15,345 edge elements, and 146 vertex elements, ensuring good mesh quality and numerical stability throughout the simulation.

Under various compressive displacement conditions, we extracted the distribution of the Displacement Curl X (absolute value), which represents the absolute value of the curl of the displacement vector field in the axial (X) direction. This parameter effectively characterizes local rotational and shear deformation within the fiber induced by compression, serving as a key indicator of potential torsion and micro-crack formation in the multi-walled structure.

Simulation results reveal that as the indenter is progressively driven into the fiber, a distinct transition zone forms between the compressed and uncompressed regions. Within this zone, the axial displacement curl exhibits significant amplification, indicating a pronounced tendency for local bending and torsional deformation. For the GNPs/TPU multi-walled structure in this study, elevated displacement curl values in this region suggest that the internal walls are more susceptible to deformation-induced micro-cracking. Such localized structural responses can disrupt the conductive pathways in the fiber, thereby contributing to an overall increase in electrical resistance. These simulation results provide a support for the conclusion presented in **Fig.5f** of the main text, highlighting the critical role of the transition region in the compression-induced increase in fiber resistance.


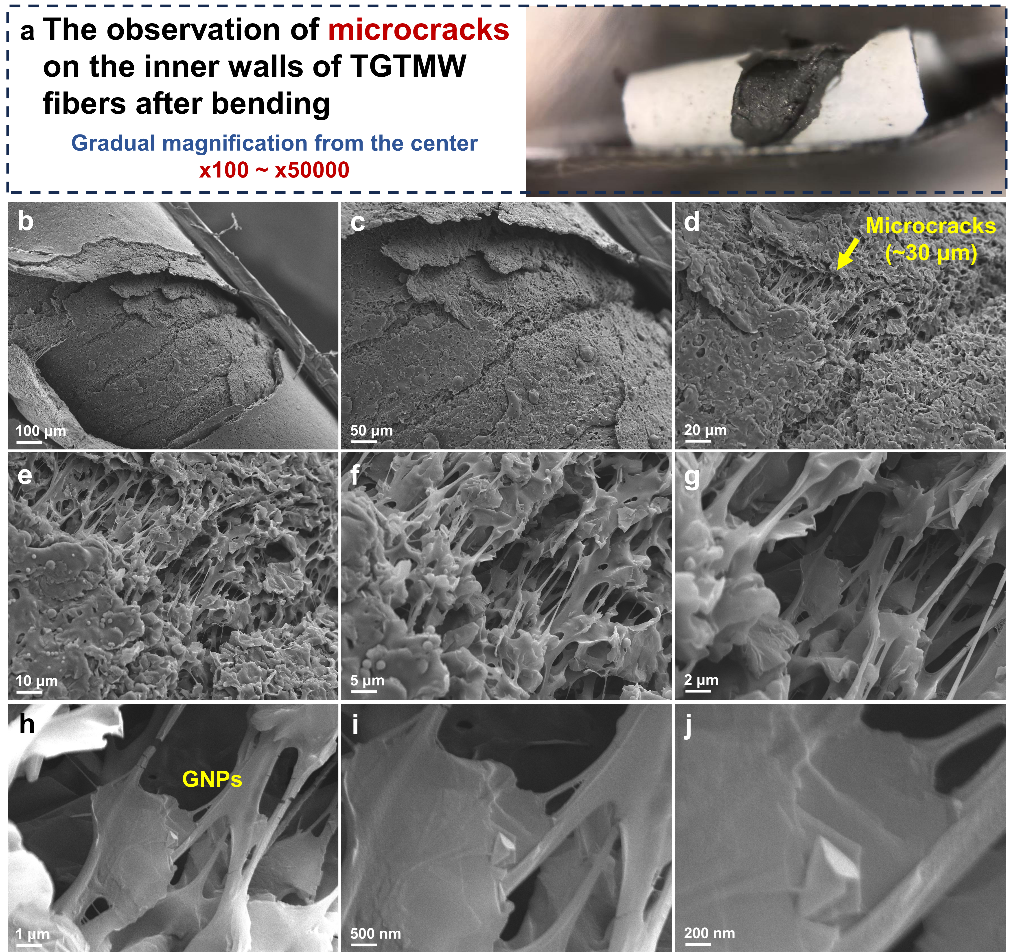


**Figure S17**. FESEM observation of microcracks on the internal multi-walled structure of TGTMW fibers after bending. (a) Macroscopic image; (b–j) show magnified views at different scales (100× to 50000×).

As shown in **Fig.S17**, the fiber was first cooled in liquid nitrogen and then crushed to expose its internal multi-walled structure. This structure was subsequently bent to simulate the deformation it might experience under compressive stress. FESEM imaging was then used to observe the surface morphology. Clear evidence of microcracks can be seen on the inner walls of the bent multi-wall structure, with typical crack widths around 30 μm. These cracks disrupt the conductive pathways between adjacent GNPs, leading to a significant local increase in electrical resistance. As a result, the overall resistance of the fiber increases, contributing to its high strain sensitivity.


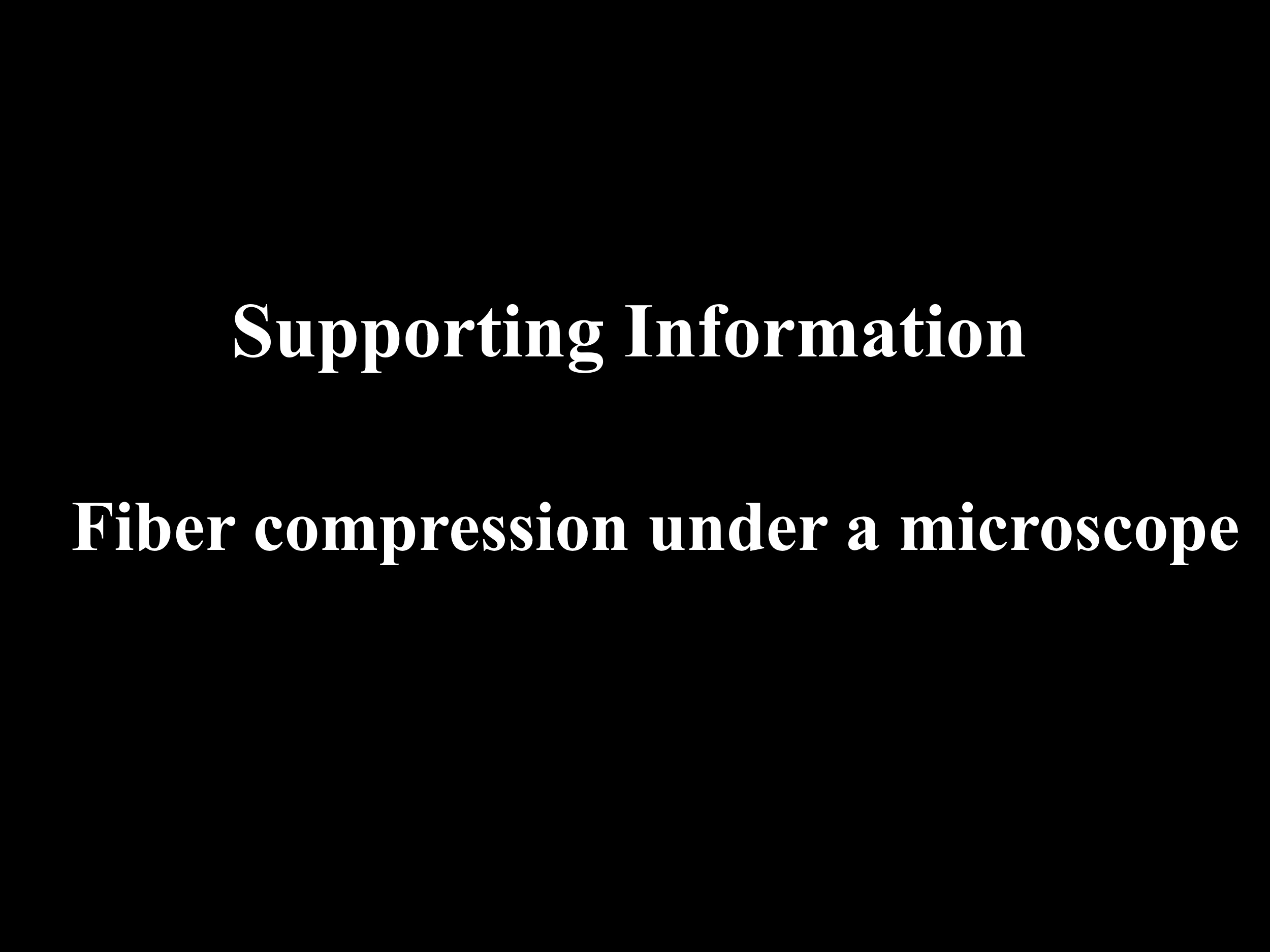


**Movie S1.** Microscopic observation of TGTMW fiber deformation under compression

As shown in **Movie.S1**, we used microscopy to capture the real-time behavior of TGTMW fibers under compression, vividly demonstrating the bending and deformation of their internal walls.


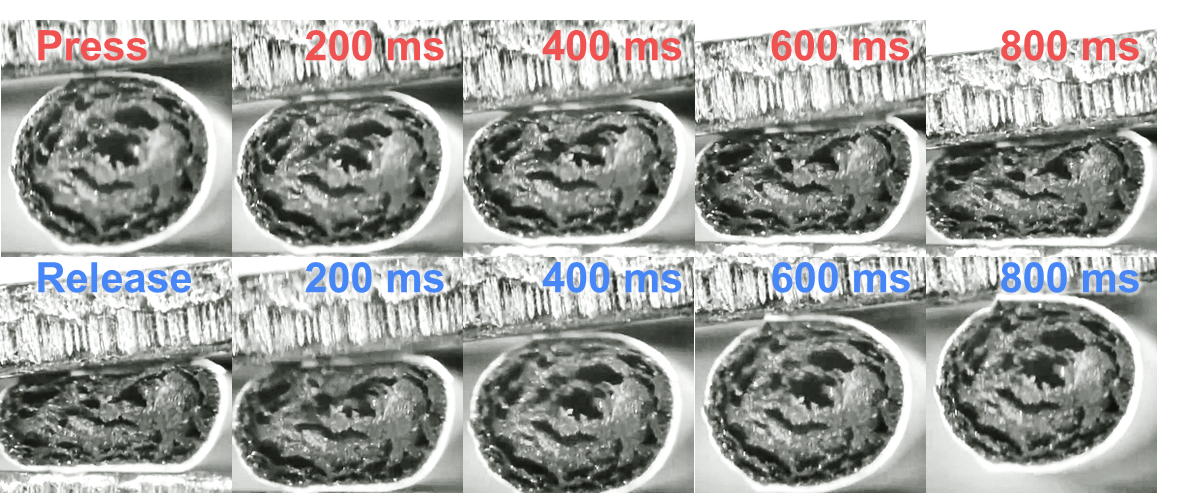


**Figure S18**. Microscopic images of TGTMW fibers during compression


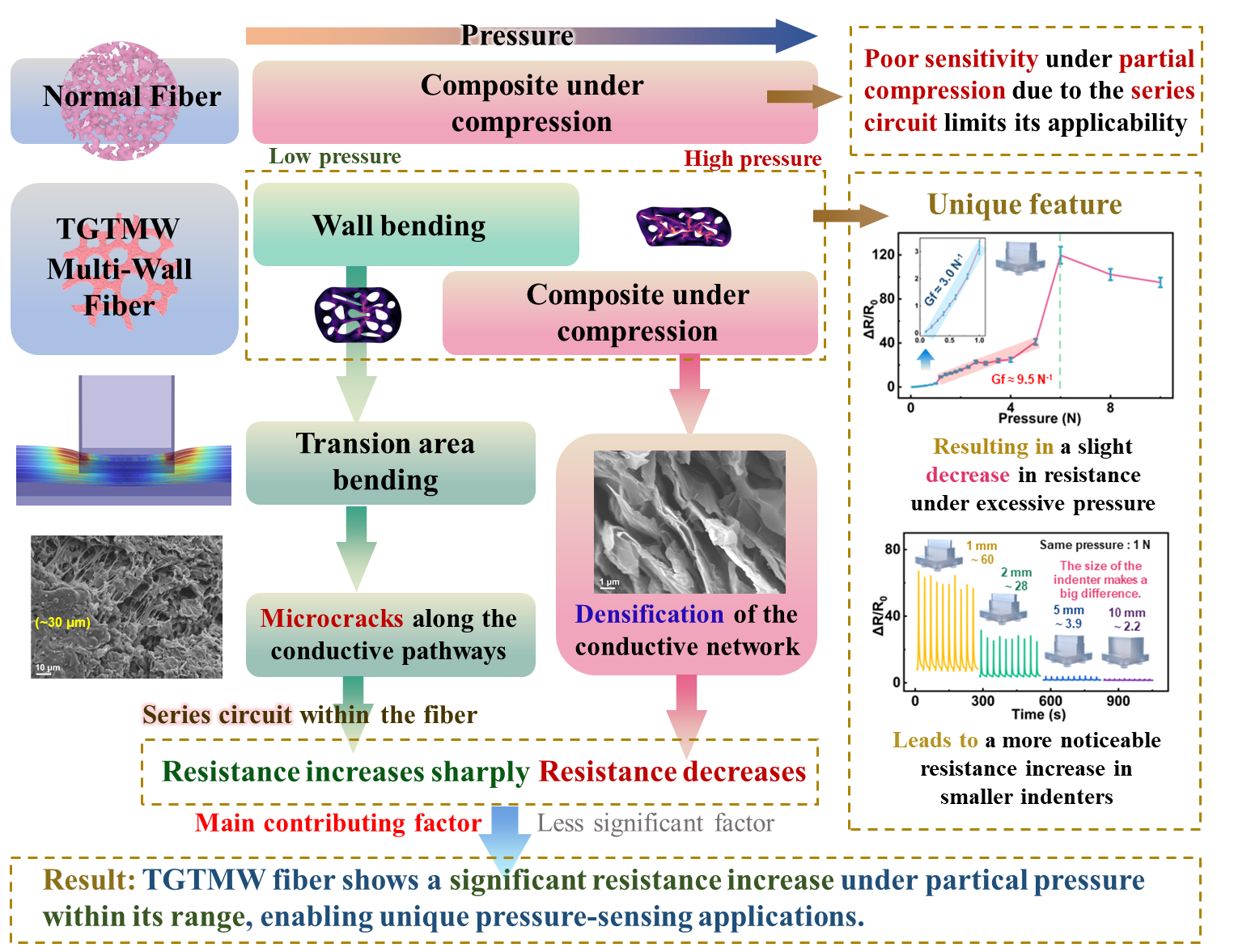


**Figure S19.** Logical diagram of the sensing mechanism of the TGTMW fiber.

As shown in **Fig.S19**, we systematically analyzed the pressure response mechanism of the TGTMW fiber. Traditional pressure-sensitive fibers typically undergo a single-stage response during compression: the internal conductive composite material is compacted, leading to a slight decrease in resistance. However, due to the overall series-connection structure of the fiber, this localized resistance reduction has a limited effect on the total resistance, resulting in relatively low sensitivity.

In contrast, the TGTMW multi-walled fiber we designed exhibits a distinct two-stage response under compression. In the initial stage, the fiber walls bend along the direction of the applied force. Only when a sufficiently large force is applied do the walls reach their bending limit and begin to experience compressive deformation. During the bending stage, a pronounced transition zone forms between compressed and uncompressed regions of the fiber, where the axial curvature is the greatest. Given that the internal GNPs/TPU composite material is partially oriented along the inner walls of the fiber, microcracks tend to form under axial bending. Since electrical current flows along the fiber’s axial direction, these microcracks significantly impede charge transport, resulting in a sharp increase in resistance within the transition region and, consequently, a pronounced increase in the total fiber resistance.

When the applied pressure exceeds approximately 12 N per centimeter (e.g., using a 10 mm-diameter indenter), the fiber becomes nearly fully flattened, and the multi-walled structure reaches its bending limit. At this point, the generation of new microcracks in the transition region becomes limited. Instead, compression is primarily exerted on the inner walls of the fiber, densifying the conductive network and causing a slight decrease in resistance. Due to the series resistance structure of the fiber, the resistance increase in the transition region dominates the response initially. The subsequent resistance decrease only becomes noticeable once the resistance in the transition region begins to saturate. This explains the observed plateau or even slight decline in resistance change rate under higher pressure conditions.

Moreover, repeated high-pressure compression at the same location may lead to relaxation of the TPU matrix, redistributing internal stress and reducing the curvature and number of microcracks in the transition region. This structural evolution may contribute both to the reduced resistance change rate under high pressure and to the gradual signal attenuation observed during long-term cyclic testing.

It is also worth noting that the size of the indenter significantly affects the sensing response. Under the same applied force, a smaller indenter induces a more concentrated deformation, resulting in greater bending in the transition region and thus a larger signal output.

In summary, the unique compression response mechanism of the TGTMW fiber—characterized by microcrack-induced resistance increase during wall bending and resistance decrease from conductive network densification under high pressure—endows the fiber with high sensitivity and result in nonlinear signal characteristics. This mechanism not only enhances its potential for applications in smart textiles and flexible electronics but also provides theoretical guidance and structural design strategies for the development of next-generation pressure-sensitive fibers.


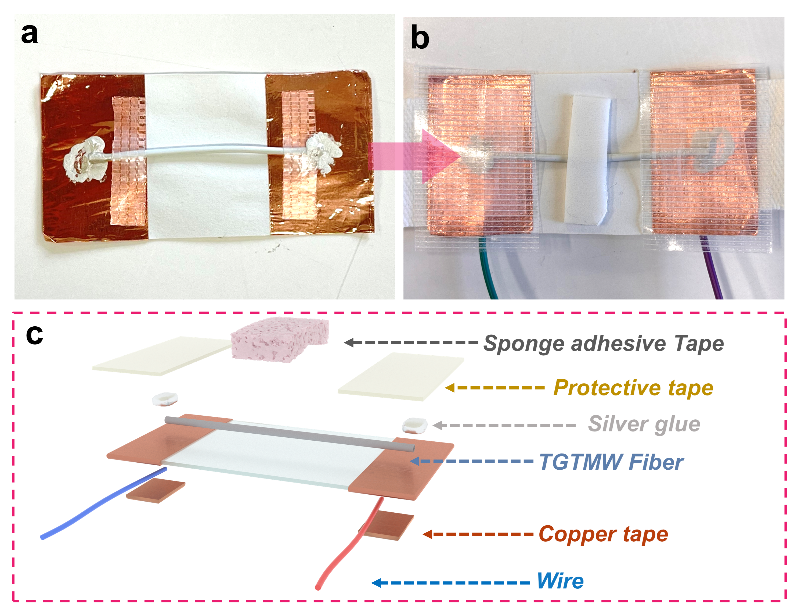


**Figure S20**. Demonstration of the sensor for human motion detection fabricated using TGTMW fibers. (a-b) Photographs, (c) Schematic diagram of the structure.

In **Fig.S20(a-b)**, photographs of the sensor for human motion detection fabricated using TGTMW fiber are shown. As illustrated, the entire sensor utilizes a TGTMW fiber approximately 4 cm in length. A piece of sponge adhesive is attached to the center of the fiber, which serves two purposes: first, it enables adhesion to fabric/skin, enhancing the accuracy of signal detection; second, the sponge adhesive applies partial pressure to the fiber, causing distortion in the inner wall structure of the TGTMW fiber. This distortion alters the resistance, thereby enabling the sensing function.

**Figure S20(c)** presents a schematic diagram of the sensor structure. A piece of paper or plastic sheet is used as the base material, with two copper tapes folded and attached to both ends of the base material. Conductive silver adhesive is applied on one side of the base material to connect the TGTMW fiber, and a sponge adhesive is affixed to the center of the fiber. On the other side of the base material, wires are connected as electrodes. The electrodes are positioned on the opposite side of the base material to prevent traction effects that could interfere with sensor operation, thereby ensuring greater accuracy of the sensing signals.


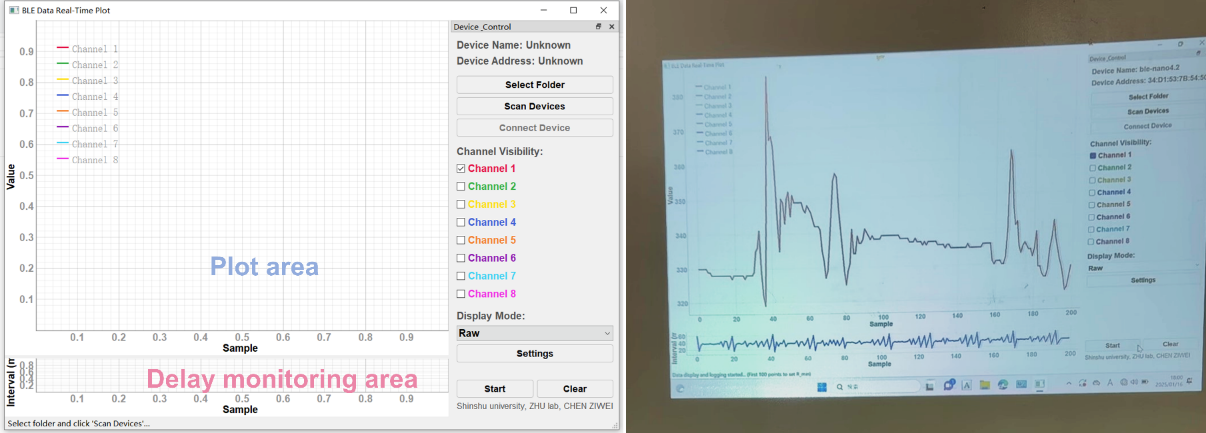


**Figure S21**. Implementation of a real-time Bluetooth Graphical User Interface (GUI) window for Real-time plotting of Bluetooth sensor signals.

As shown in **Fig.S21**, we utilized Python's Bleak library and PyQt5 to receive Bluetooth Low Energy (BLE) signals, perform real-time plotting, and save the data. For better visualization, we connected the computer to a projector and recorded a video using a smartphone. The recorded video (**Movie.S2-3**) provides an intuitive demonstration of human motion, including simple leg/arm movements and more complex activities such as playing frisbee, which can be detected in real time with extremely low latency.


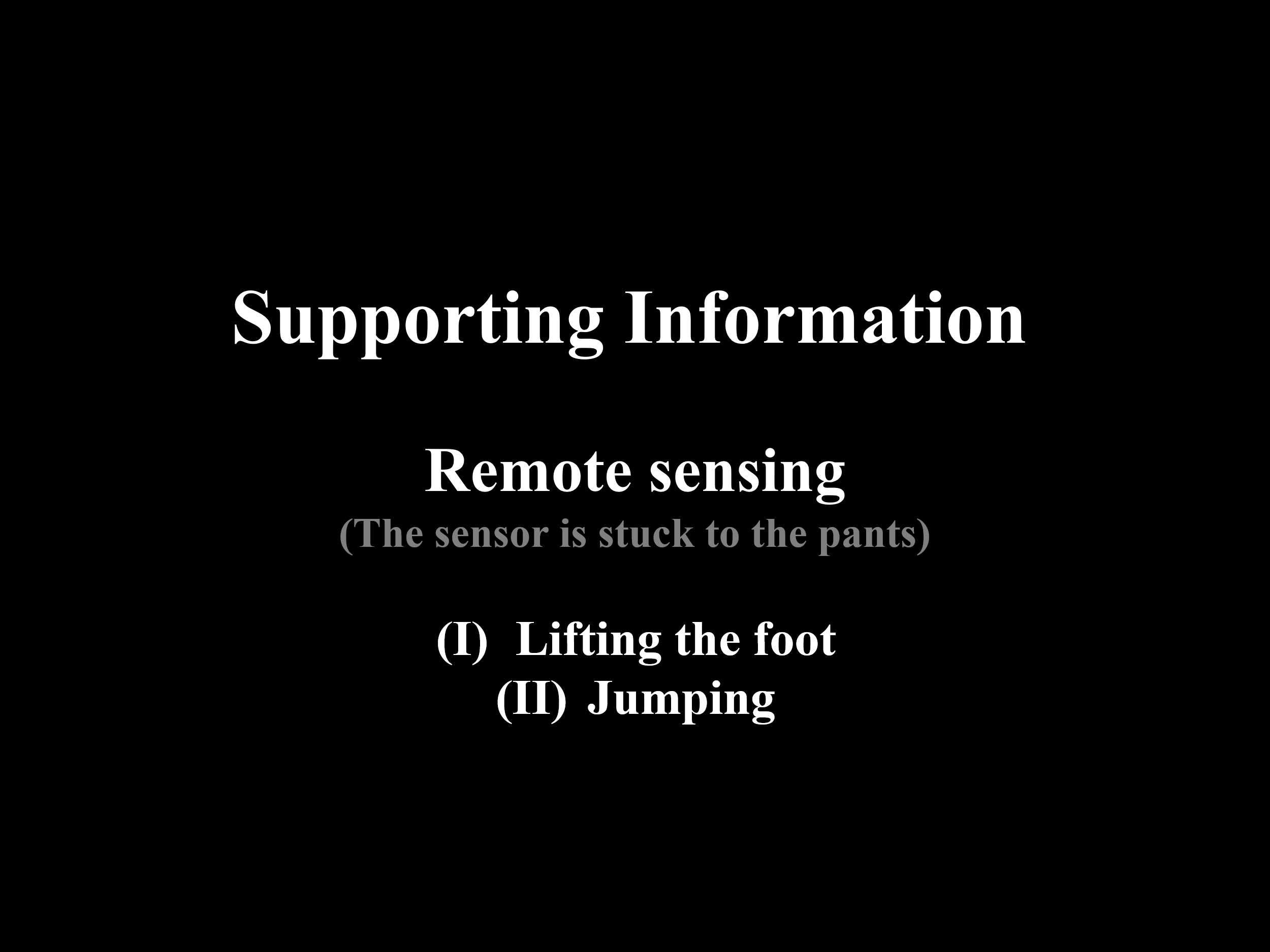


**Movie S2**. Video of real-time monitoring of both foot lifting and jumping movements using sensors prepared with TGTMW fibers


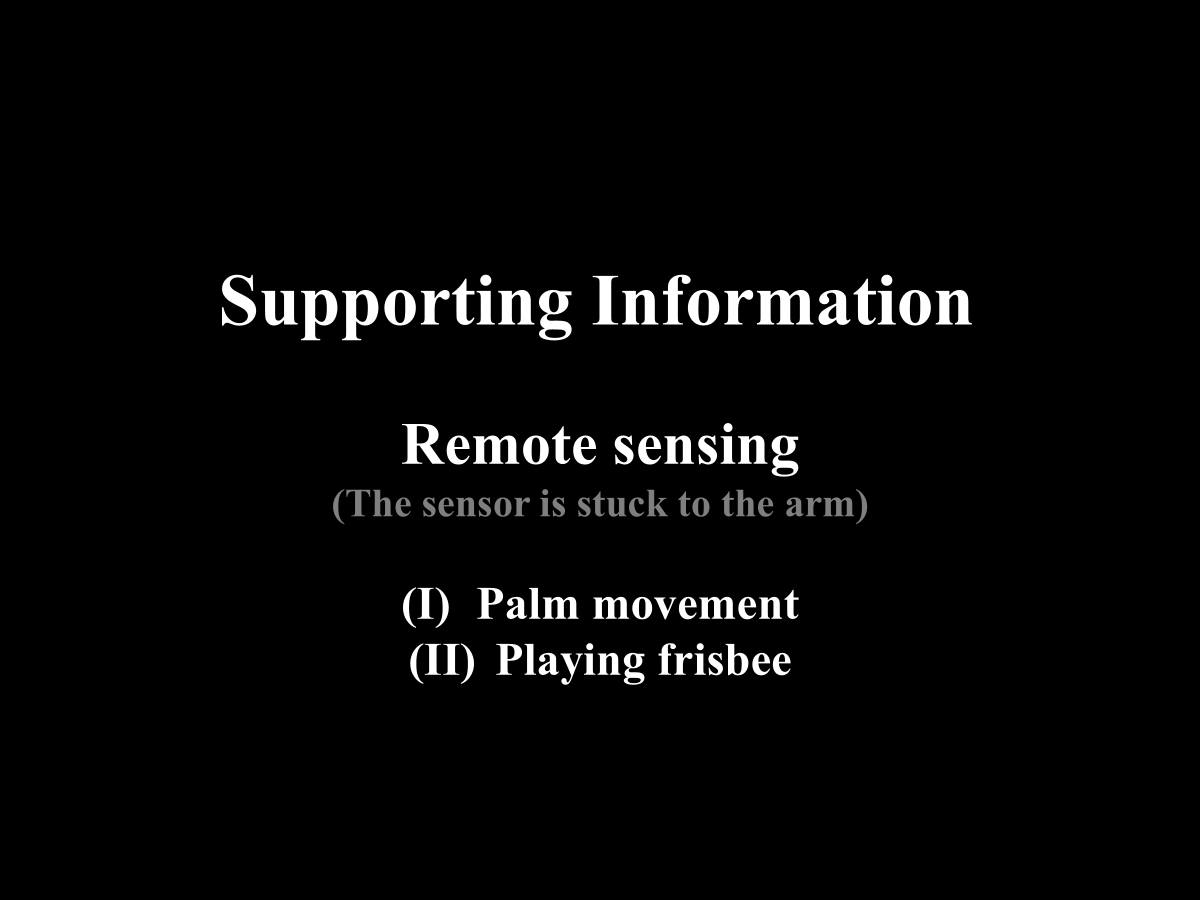


**Movie S3**. Video of real-time monitoring of both foot palm movement and playing frisbee movements using sensors prepared with TGTMW fibers


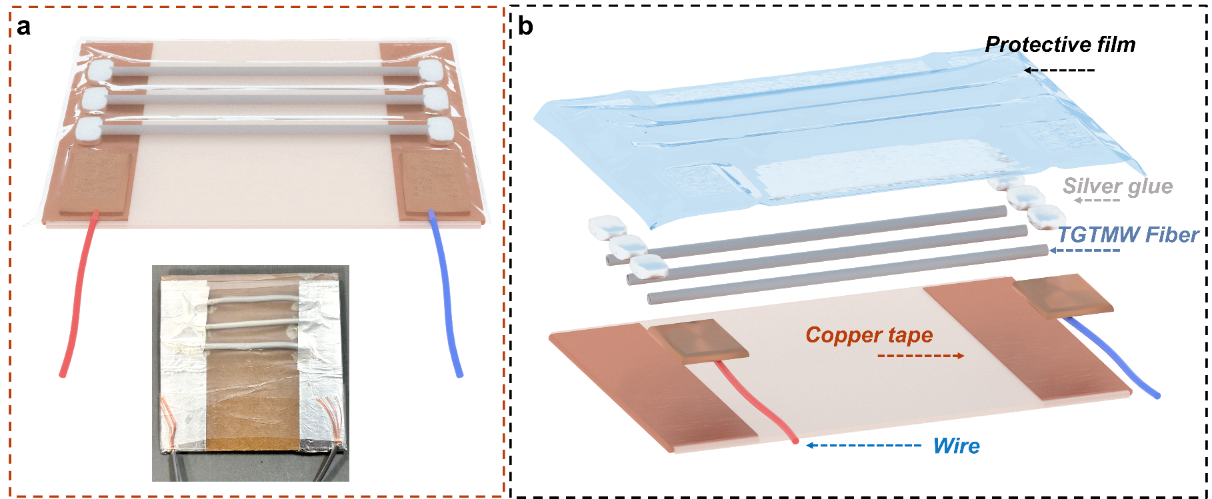


**Figure S22**. The photo and schematic diagram of a specialized pressure-sensitive sensor assembled by arranging three TGTMW fibers in parallel.

As shown in **Fig.S22(a)**, the sensor is constructed by aligning three TGTMW fibers in parallel. When disassembled, as illustrated in **Fig.S22(b)**, the sensor consists of several components. From bottom to top, these include a base material with conductive copper/aluminum tape attached, serving as the electrode; conductive wires functioning as electrodes; three TGTMW fibers, both ends of which are coated with conductive silver paste and fixed onto the base material (an additional layer of conductive tape can be applied for extra fixation); and finally, a thin protective film covering the assembly. The primary purpose of this film is to prevent excessive force or long fingernails from pulling and damaging the TGTMW fibers when sliding fingers across the sensor. Additionally, it provides appropriate friction between the sensor and the fingers, reducing the risk of damage and thereby extending the sensor’s lifespan.


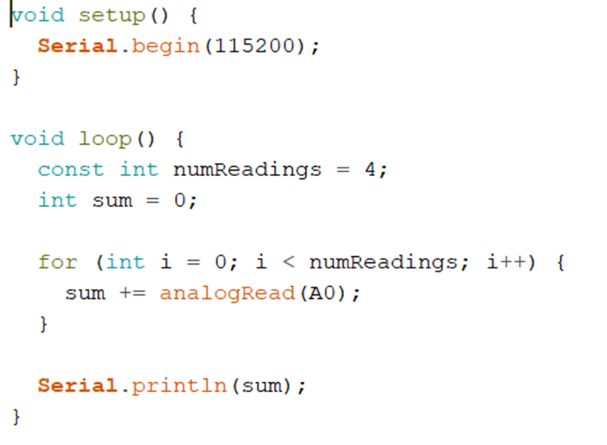


**Figure S23**. Code for acquiring high-speed sensor signals using an Arduino Uno microcontroller

To balance errors while ensuring a high sampling rate, as shown in **Fig.S23**, we chose to use the built-in ADC of the Arduino Uno to read signals 4 times and sum the outputs. The resistance is then calculated using the principle of series voltage division, which allows us to calculate the rate of resistance change. Considering the microcontroller's memory, clock speed, and the time required for serial output, the actual sampling rate typically reaches around 1600–1800 cps, which is sufficient for our application needs. After transmitting the data to a computer via the serial port, the computer records the timestamps and stores the data for further analysis.


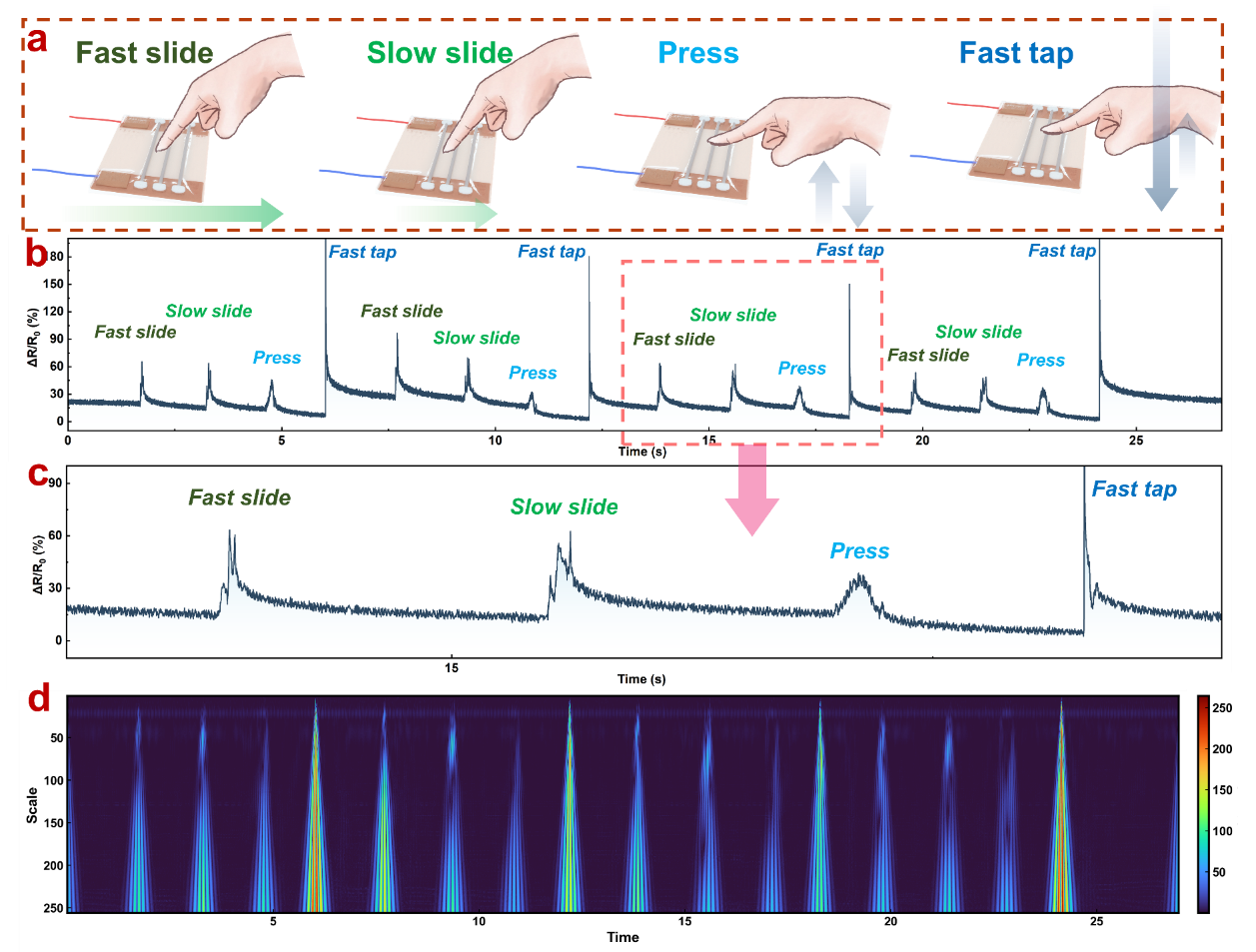


**Figure S24**. Testing with more external stimuli such as fast slide, slow slide, press, and fast tap, and wavelet transform analysis

In **Fig.S24**, we present the wavelet transform analysis of a specially designed pressure-sensitive sensor, fabricated using three TGTMW multi-walled fibers, in response to various external stimuli for differentiation.

**Figure S24(a)** illustrates the four different types of external stimuli:

**(I) Fast slide:** Simulating a quick finger swipe across the screen when skipping unwanted content while browsing a smartphone.

**(II) Slow slide:** Mimicking a slower swiping motion when flipping through text while reading.

**(III) Press:** Representing the typical pressing motion when interacting with a touchscreen.

**(IV) Fast tap:** Simulating rapid tapping on the screen in moments of excitement.

These actions were performed continuously four times, and the corresponding signals are shown in **Fig.S24(b)**. A zoomed-in view in **Fig.S24(c)** highlights the distinct characteristics of these signal peaks. The collected data was then processed using the Morlet wavelet for continuous wavelet transformation, generating the wavelet scalogram displayed in **(Fig.S24d)**, which serve as the basis for further analysis (see **Fig.S25**).


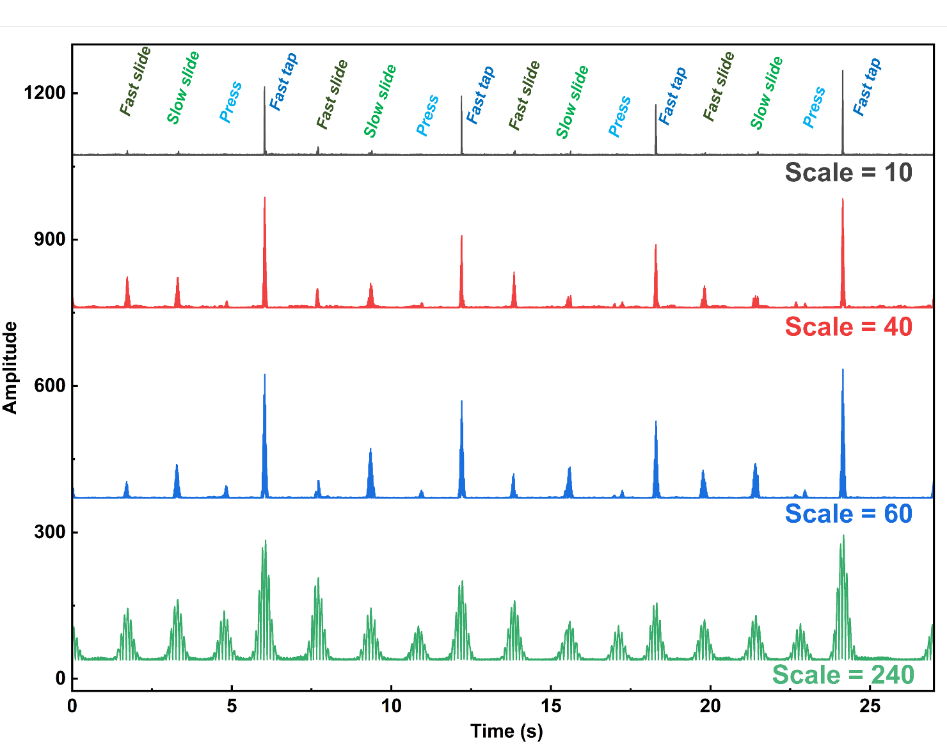


**Figure S25**. Analysis of wavelet transform results for various external stimuli using slices from the scalogram

As shown in **Fig.S25**, we applied slicing to the wavelet scalogram from **Fig.S24**. Compared to the application in the main text, which differentiates between press and slide actions, distinguishing among four different types of stimuli is more complex. Therefore, we extracted slices at Scale = 10, 40, 60, and 240, corresponding to ultrahigh-frequency, high-frequency (I), high-frequency (II), and low-frequency signals, respectively. The detailed analysis is as follows:

**Scale = 10 (Ultrahigh Frequency):** This scale captures rapid changes in electrical signals, reflecting the instantaneous response of the pressure-sensitive sensor made from three TGTMW fibers. This corresponds to the **fast tap** motion performed in an excited state. The slice at Scale = 10 shows a strong signal, allowing us to effectively distinguish **fast tap** from other stimuli.

**Scale = 40, 60 (High Frequency):** Unlike the analysis in the main text, which differentiates press from slide using only **Scale = 60** (representing the triple peaks caused by fiber sliding), distinguishing between fast slide and slow slide requires more detailed frequency information. Here, we use two slices:

For fast slide, the **Scale = 40** slice exhibits a slightly higher peak at the corresponding time.

For slow slide, the **Scale = 60** slice exhibits a slightly higher peak at the corresponding time.

By excluding **fast tap** (which shows strong signals at **Scale = 10**) and **press** (which has very weak peak intensity), this method allows for the differentiation of **fast slide** and **slow slide**.

**Scale = 240 (Low Frequency):** This scale is used to verify whether the three TGTMW fiber-based pressure-sensitive sensor is being stimulated at a given moment. If a signal peak appears at Scale = 240 while no significant peaks are observed at higher frequencies, the action can be identified as a slow **press**.


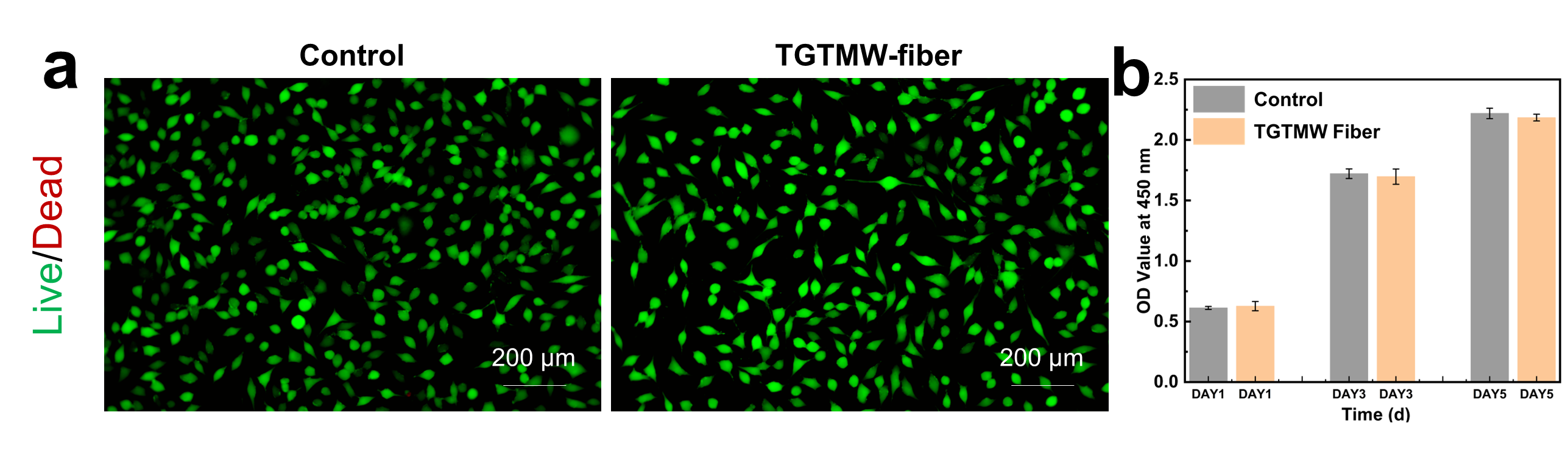


**Figure S26**. Biocompatibility tests of TGTMW fiber: (a) live/dead staining of L929 cells, (b) CCK-8 cell viability assay

As shown in **Fig.S26**, the biocompatibility of TGTMW fiber was evaluated with mouse fibroblast L929 cells (OriCell, China). To prepare the extract medium, sterilized samples were incubated in DMEM medium supplemented with 10% fetal bovine serum (FBS) at 37°C for 24 h at a concentration of 50 mg/mL. For live/dead staining, L929 cells were seeded in 48-well plates at a density of 2 × 10^3^ cells/well and incubated for 24 h at 37 °C. The cells were then treated with the TGTMW fiber extract medium, while untreated cells served as controls. After 24 h of incubation, cell viability was assessed using calcein-AM/propidium iodide (PI) staining for 15 min. Fluorescence microscopy (CKX53, Olympus, Japan) was used to capture images, with live and dead cells visualized as green (calcein-AM) and red (PI), respectively. Quantitative analysis was conducted using the CCK-8 assay on days 1, 3, and 5. Cells were seeded in 96-well plates at a density of 3 × 10^2^ cells/well and treated with the extract medium under the same conditions. At each time point, 10 µL of CCK-8 reagent was added to each well, followed by 4 h of incubation at 37 °C. Absorbance was measured at 450 nm using a microplate reader (CYTATION 5, BioTek).

Both live/dead staining and CCK-8 assays demonstrated the excellent biocompatibility of the TGTMW fibers (**Fig.S26(a**)). Fluorescence imaging revealed minimal red fluorescence across all groups, indicating negligible cytotoxicity. Consistently, CCK-8 analysis showed no significant difference in cell viability between the treated and control groups across all timepoints (days 1, 3, and 5), further supporting the absence of cytotoxic effects. These results demonstrate that TGTMW fiber are non-cytotoxic and hold strong potential for flexible sensors and smart textiles.


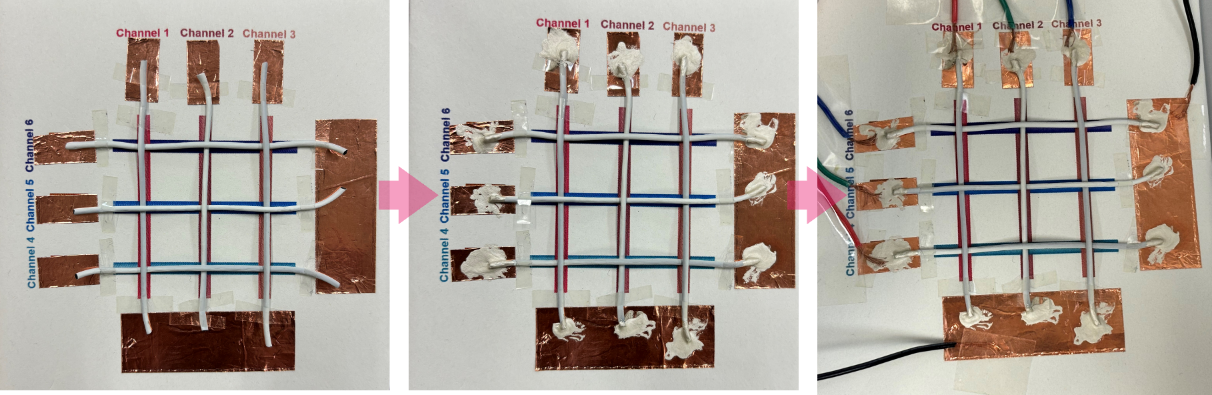


**Figure S27**. Fabrication process of a 3×3 pressure-sensitive array using TGTMW fibers.

As shown in **Fig.S27**, conductive copper tape was attached to the substrate material (for better visualization, we selected paper printed with an array structure). The TGTMW fibers were fixed onto the substrate in a woven-like structure, coated with conductive silver paste, and dried. Finally, wires were connected to the copper tape, and the array was integrated with an Arduino Uno microcontroller through a series voltage division method to achieve real-time pressure-sensitive signal sensing.


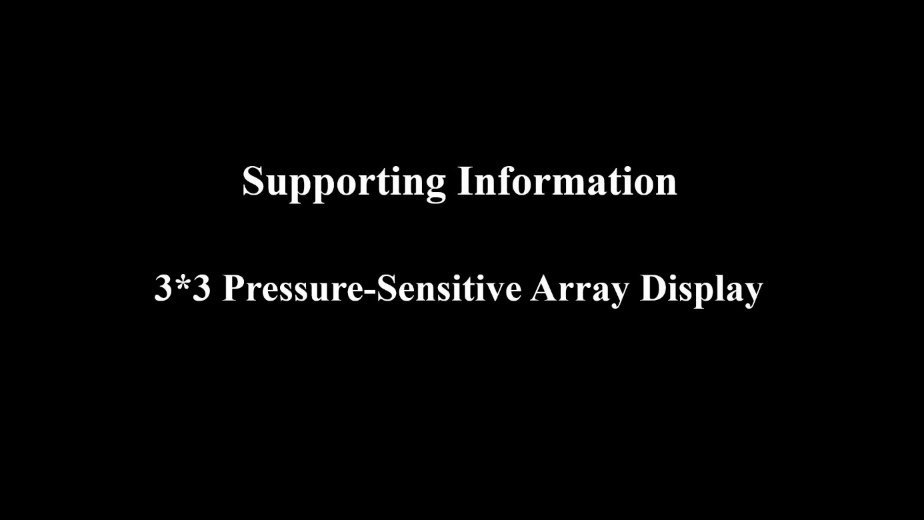


**Movie S4**. Test video of the 3×3 pressure-sensitive sensor array prepared by TGTMW fibers


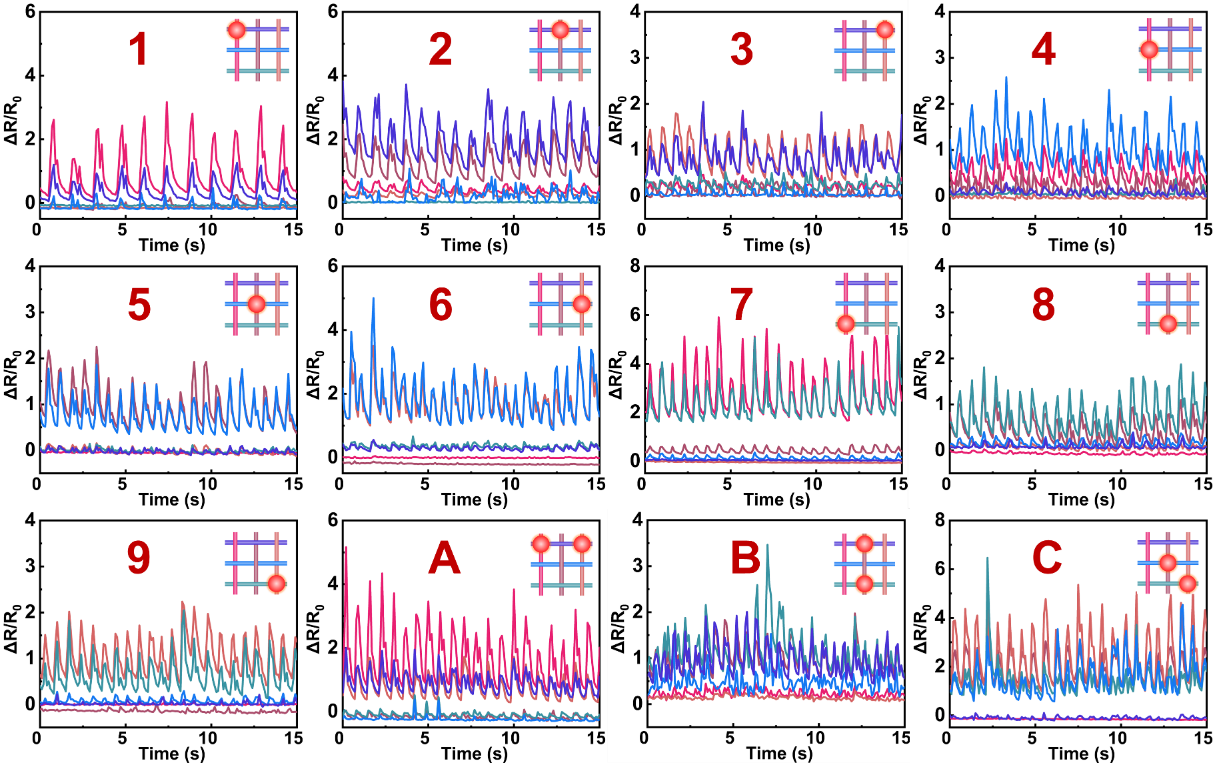


**Figure S28**. Six-Channel Resistance Change Rate Signals Collected in a Computer

As shown in **Fig.S28**, this data is obtained from six channels (A0–A5) of an Arduino Uno connected to a computer via analog input interfaces. Using the principle of series voltage division, the signals are converted into resistance change rates, resulting in continuous electrical signal curves. Due to the viscoelasticity of the TPU, baseline drift occurs. In the subsequent processing, it is necessary to segment these continuous electrical signal curves so that each peak corresponds to a single action. Additionally, the six signals should be shifted along the Y-axis to align their starting points at zero, thereby leveling the baseline.


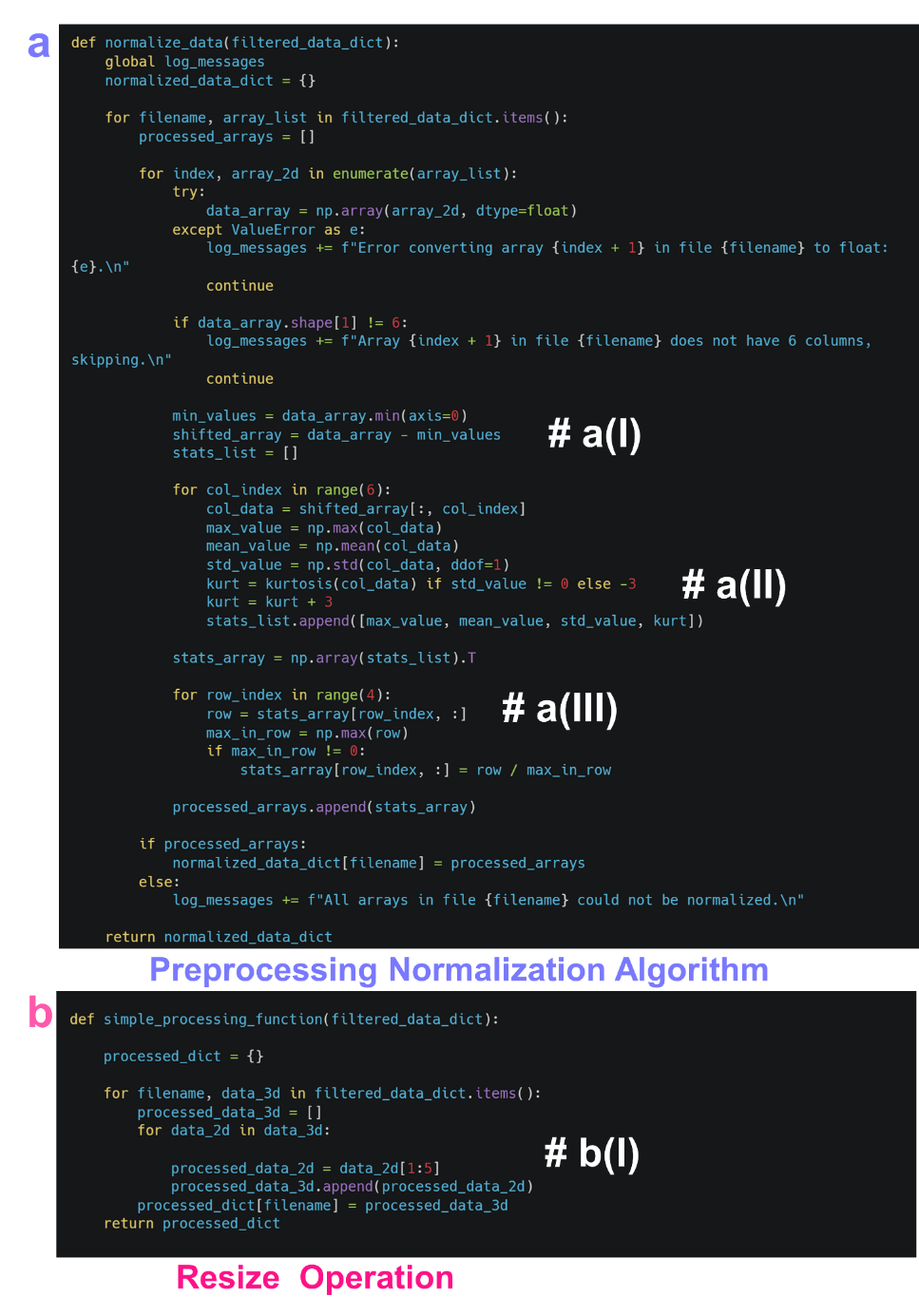


**Figure S29.** Explanation of the Preprocessing Normalization Algorithm (PNA) and Resize Operation (RO) algorithm

**Figure S29** illustrates the functions used in these two algorithms, both of which take a three-dimensional array dictionary as input and output. **Fig.S29(a)** corresponds to the Preprocessing Normalization Algorithm (PNA), while **Fig.S29(b)** represents the simple Resize Operation (RO).

The purpose of both functions is to transform an n×6 matrix, corresponding to a specific compression operation, into a standardized 4×6 matrix for CNN-based machine learning. Notably, in **a(I)**, an additional data shift is applied to ensure that the minimum value is 0, preventing negative values in the matrix. Although the separated data has already undergone a baseline shift process (as shown in **Fig.8(a-b)**, where all six signals start from 0), in some cases, due to resistance reduction, negative values may still appear. To avoid this, we perform another shift to ensure all values are non-negative, guaranteeing that the maximum value, mean, and standard deviation remain positive.

In **a(II)**, a constant value of +3 is added to the kurtosis to keep it non-negative as well. In **a(III)**, each element in the array is divided by its maximum value to ensure that the resulting 4×6 matrix does not exceed 1. These steps are implemented to ensure that all elements in the matrix remain within the range of 0-1.

For the simple Resize Operation (RO), the method directly extracts rows 1, 2, 3, and 4 of the original matrix. Since Python's NumPy indexing starts from 0, this actually corresponds to extracting rows 2, 3, 4, and 5 of the original data. The first row is not used because, due to baseline shifting, its values are all zero and thus meaningless. To ensure a fair comparison between the two algorithms, only rows 2-5 are selected.

Both algorithms effectively convert an irregular n×6 matrix into a standardized 4×6 matrix suitable for further processing.


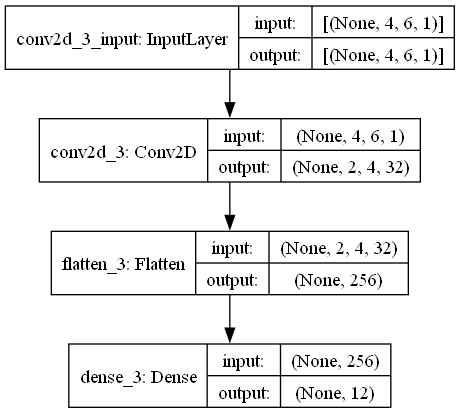


**Figure S30.** The CNN model architecture used for processing data from the 3×3 pressure-sensitive array made of classified TGTMW fibers.

As shown in **Fig.S30**, this CNN model processes 4×6 single-channel images (obtained by transforming the original n×6 matrix using the method shown in **Fig.S30**). First, a convolutional layer extracts 32 feature maps. Then, a flattening layer converts these feature maps into a one-dimensional vector. Finally, a dense layer outputs predictions for 12 categories, making it suitable for classification tasks.


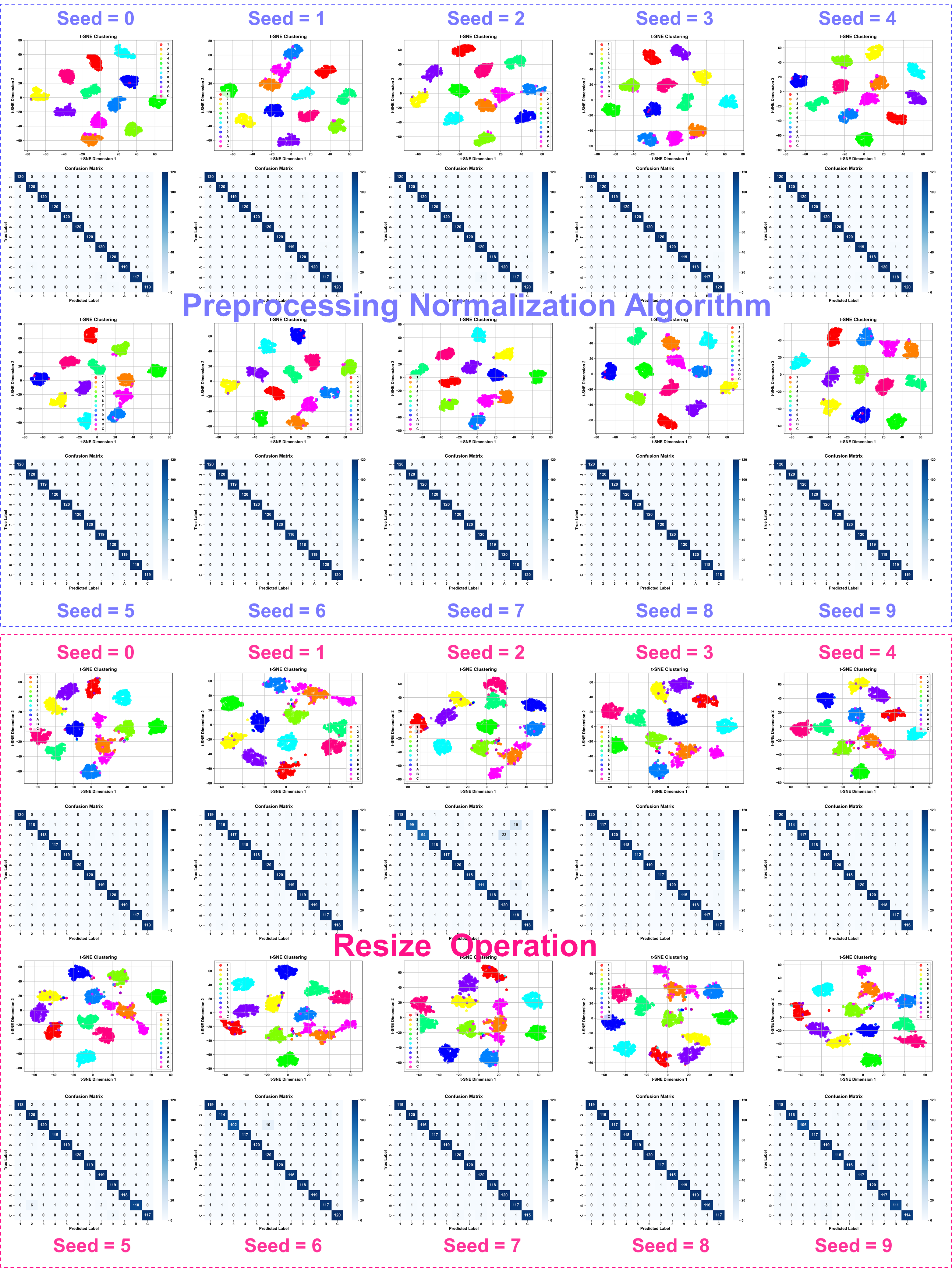


**Figure S31.** Results of 10 CNN machine learning tests and t-SNE dimensionality reduction for both PNA and RO algorithms with fixed random seeds.

As shown in **Fig.S31**, after processing the six pressure-sensitive signal channels of TGTMW fibers using the Preprocessing Normalization Algorithm (PNA), 12 different compression signals can be well distinguished. Compared to directly resizing the matrix, this approach offers certain advantages, demonstrating the high classification accuracy of the pressure sensor array prepared using TGTMW fibers.


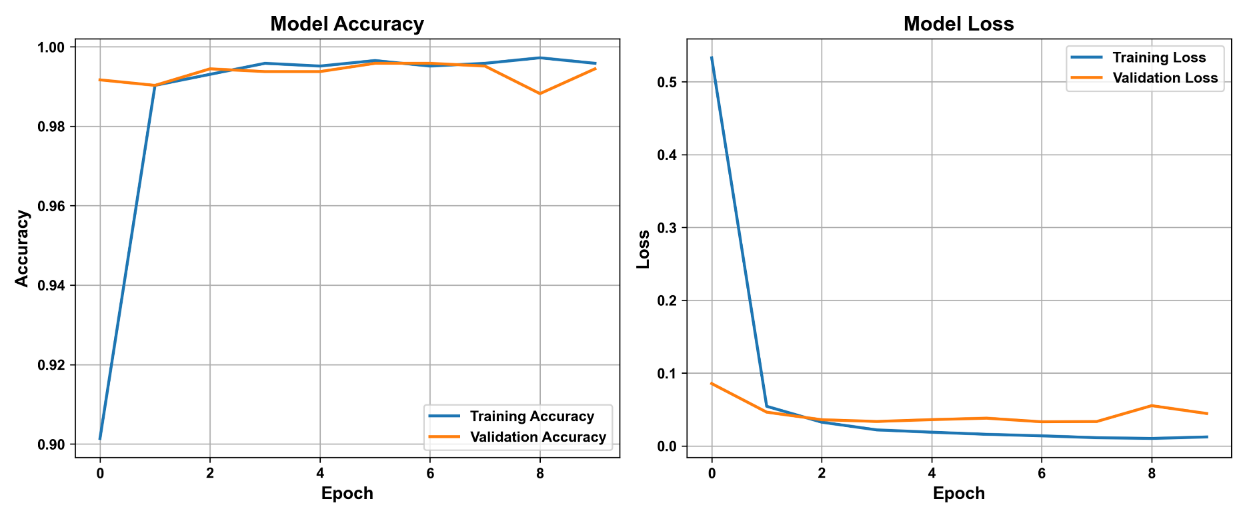


**Figure S32.** Learning curve of CNN machine learning using the PNA algorithm on the TGTMW 3×3 pressure-sensitive array with seed = 6.


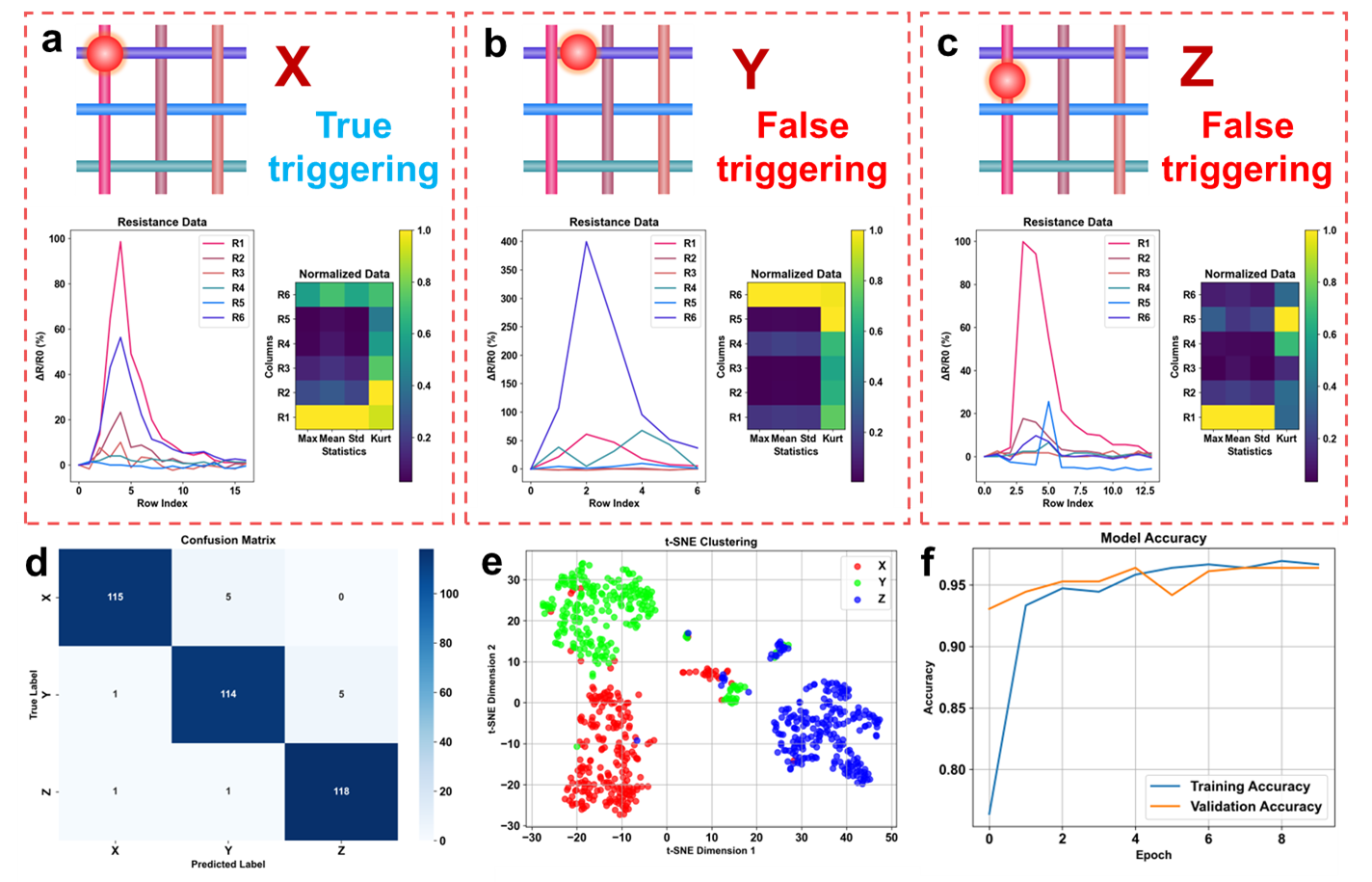


**Figure S33.** CNN-based classification results for distinguishing true and false triggering events in the TGTMW fiber array: (a) True activation on a single node (labeled as X); (b) False triggering caused by compression between horizontally adjacent nodes (labeled as Y); (c) False triggering caused by compression between vertically adjacent nodes (labeled as Z); (d) Confusion matrix showing classification performance among the three types of events; (e) t-SNE visualization of sample clustering based on CNN feature embeddings; (f) Training curve of the CNN model demonstrating convergence and learning efficiency.

As illustrated in **Fig.S33**, we employed three distinct triggering scenarios for analysis: one example of normal activation (**Fig.S33(a)**) and two types of false triggering occurring at the intermediate regions between horizontally adjacent nodes (**Fig.S33(b**)) and vertically adjacent nodes (**Fig.S33(c)**), respectively. For each scenario, we collected 240 sample datasets and conducted CNN machine learning tests using the same methodology and PNA algorithm described in **Fig.8(a)**.

The test results demonstrate that, as shown in **Fig.S33(d)**, the machine learning classification of the TGTMW pressure-sensitive array sensor achieved an identification accuracy exceeding 96% for distinguishing between normal node activation and false triggering caused by compression between the target node and its horizontally/vertically adjacent nodes. This indicates that the TGTMW fiber-based pressure-sensitive array possesses high resolution capability for discriminating between true and false triggering events, thereby enhancing signal transmission accuracy in practical applications. Furthermore, as depicted in **Fig.S33(e)**, the t-SNE plot reveals good sample separation between correct triggering and false triggering events. However, due to some inherent variability in human testing procedures, occasional misclassification may still occur in rare instances. **Fig.S33(f)** presents the CNN model's learning curve, which demonstrates high training efficiency and stable convergence.

In summary, the CNN machine learning approach enables identification of false triggering phenomena in TGTMW fiber arrays with remarkably high accuracy, establishing significant application value for this sensing technology.

**Table S2.** Comparison between this work and other related studies.

| Ref. | Type | Mechanism | Preparation | Condition of partial compression | | Detection pressure capability |
| --- | --- | --- | --- | --- | --- | --- |
| 【1】 | Aerogel | Resistance  decrease | Mxene + Freeze-drying | Not mentioned | 0.2 Pa - 1 kPa  Gf = 0.04 - 0.37 kPa^-1^ | |
| 【2】 | Film | Resistance  decrease | Mxene + Spraying + silk-screen printing | Not mentioned | 0.2 Pa – 141 kPa  Gf = 6.95 - 2602 kPa^-1^ | |
| 【3】 | Film | Resistance  decrease | 3D print + CVD | Not mentioned | 50 Pa – 39.2 kPa  High sensitivity | |
| 【4】 | Aerogel | Resistance  decrease | MXene/GO + Hydrothermal + Solvent exchange | Not mentioned | 0.4 Pa – 200 kPa  High sensitivity | |
| 【5】 | Aerogel | Resistance  decrease | GO/PUF + hot pressing + freeze casting | Not mentioned | 0.4 Pa – 12.6 MPa  High sensitivity | |
| 【6】 | Film | Resistance  decrease | PDMS + Thermal Evaporation + SAM | Not mentioned | 0.5 Pa – 25 kPa  High sensitivity | |
| 【7】 | Fiber | Resistance  decrease | MXene + CNF + Coaxial wet-spinning | Not mentioned | 3 Pa – 233.33 kPa | |
| This  Work | **Fiber** | Resistance  **Increase** by microcracks | GNPs + Coaxial wet-spinning | **Feasible** | 0.1 – 6 N by 5 mm indenter | |

**Table S2** presents a comparison between the TGTMW pressure-sensitive fiber developed in this study and other reported pressure sensors. The TGTMW-based sensor demonstrates several unique features:

**Type:** The sensor adopts a fibrous structure, which is relatively rare compared to the mainstream aerogel and film-based pressure sensors. The fiber-based design offers distinct advantages in terms of compact size, adaptability, and ease of integration, especially for wearable applications.

**Mechanism:** The working mechanism involves the formation of microcracks along the fiber axis under compression, leading to a significant increase in resistance. This fundamentally differs from the conventional mechanism where compression leads to increased contact area and decreased resistance due to denser conductive pathways. This novel mechanism offers particular advantages in series circuit configurations.

**Preparation:** While materials such as MXene and GO, as well as fabrication techniques like freeze-drying and CVD, are commonly used in pressure sensor development, our approach utilizes cost-effective graphene nanoplatelets (GNPs) and enables continuous fabrication through coaxial wet spinning. This method is both scalable and economical.

**Condition of Partial Compression:** To our knowledge, previous studies have not addressed this aspect. In our work, we specifically demonstrate that only a small segment of the TGTMW fiber needs to be compressed to generate a distinct pressure response. This has been validated using various pressing indenters. The results indicate that compressing any part of the fiber with a fingertip yields a clear signal. This unique feature arises from our sensor’s mechanism of increased resistance upon compression. In contrast, resistance-decreasing fiber sensors typically exhibit a lower sensitivity when only partially compressed, as the resistance change is smaller than the compression ratio (because of the principle of series circuit)—our design overcomes this limitation.

**Sensitivity:** When simulating fingertip contact using a 5 mm-diameter pressing indenter, the TGTMW fiber exhibits a detection range of 0.1–6 N, which covers most practical scenarios, particularly in smart textiles (e.g., light touch, pressing, sliding).

In summary, the distinctive working mechanism of the TGTMW fiber-based sensor enables functionalities beyond those of conventional flexible pressure sensors. The structural and functional design presented in this work could open up new possibilities for the development of next-generation fiber-based pressure sensors.

**Reference:**

[1] J. Ren, X. Huang, R. Han, G. Chen, Q. Li, Z. Zhou, Avian Bone‐Inspired Super Fatigue Resistant MXene‐Based Aerogels with Human‐Like Tactile Perception for Multilevel Information Encryption Assisted by Machine Learning, Advanced Functional Materials 34(39) (2024) e2403091.

[2] Z. Yu, C. Deng, J. Sun, X. Zhang, Y. Liu, C. Liu, F. Seidi, J. Han, Q. Yong, H. Xiao, Cellulosic Nonwovens Incorporated with Fully Utilized MXene Precursor as Smart Pressure Sensor and Multi‐Protection Materials, Advanced Functional Mater ials 34(38) (2024) e2402707.

[3] J. Baek, Y. Shan, M. Mylvaganan, Y. Zhang, X. Yang, F. Qin, K. Zhao, H.W. Song, H. Mao, S. Lee, Mold-Free Manufacturing of Highly Sensitive and Fast-Response Pressure Sensors Through High-Resolution 3D Printing and Conformal Oxidative Chemical Vapor Deposition Polymers, Advanced Materials 35(41) (2023) e2304070.

[4] W. Zhu, Y. Zhuang, J. Weng, Q. Huang, G. Lai, L. Li, M. Chen, K. Xia, Z. Lu, M. Wu, Z. Zou, Evolution of Naturally Dried MXene-Based Composite Aerogels with Flash Joule Annealing for Large-Scale Production of Highly Sensitive Customized Sensors, Advanced Materials 36(33) (2024) e2407138.

[5] X. Zhang, Z. Hu, Q. Sun, X. Liang, P. Gu, J. Huang, G. Zu, Bioinspired Gradient Stretchable Aerogels for Ultrabroad-Range-Response Pressure-Sensitive Wearable Electronics and High-Efficient Separators, Angewandte Chemie International Edition 62(1) (2023) e202213952.

[6] S.W. Kim, J.H. Lee, H.J. Ko, S. Lee, G.Y. Bae, D. Kim, G. Lee, S.G. Lee, K. Cho, Mechanically Robust and Linearly Sensitive Soft Piezoresistive Pressure Sensor for a Wearable Human-Robot Interaction System, ACS Nano 18(4) (2024) 3151-3160.

[7] D. Ma, H. Fang, J. Sun, T. Jiang, A fiber-shaped sensor constructed by coaxial wet-spinning for dual-mode sensing, Journal of Materials Chemistry A 13(8) (2025) 5870-5881.
