# Supplementary material for: Fibrous Pressure Sensor with Unique Resistance Increase under Partial Compression: Coaxial Wet‐Spun TiO2/Graphene/Thermoplastic Polyurethane Multi‐Wall Multifunctional Fiber
Source: Adv Mater. 2025 Jul 16;37(40):2509631. doi: 10.1002/adma.202509631 (PMC12510290; doi:10.1002/adma.202509631)

ADVANCED MATERIALS

Supporting Information

for *Adv. Mater.*, DOI 10.1002/adma.202509631

Fibrous Pressure Sensor with Unique Resistance Increase under Partial Compression: Coaxial Wet-Spun TiO_2/Graphene/Thermoplastic Polyurethane Multi-Wall Multifunctional Fiber

*Ziwei Chen, Dandan Xie, Kanae Kojima, Chunxia Gao, Jian Shi, Jian Xing, Hideaki Morikawa and Chunhong Zhu**

Pressure-sensitive data of type 1

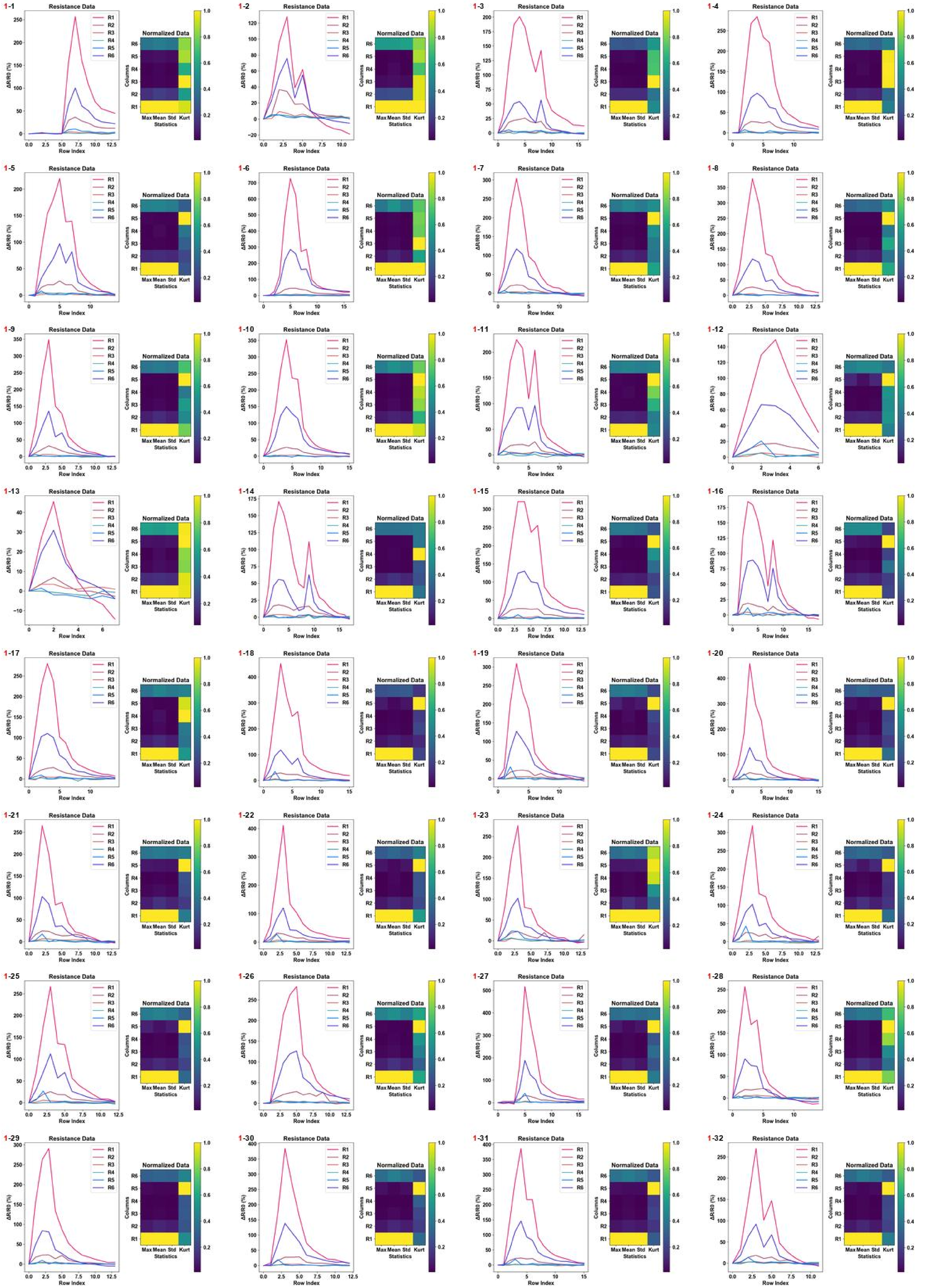

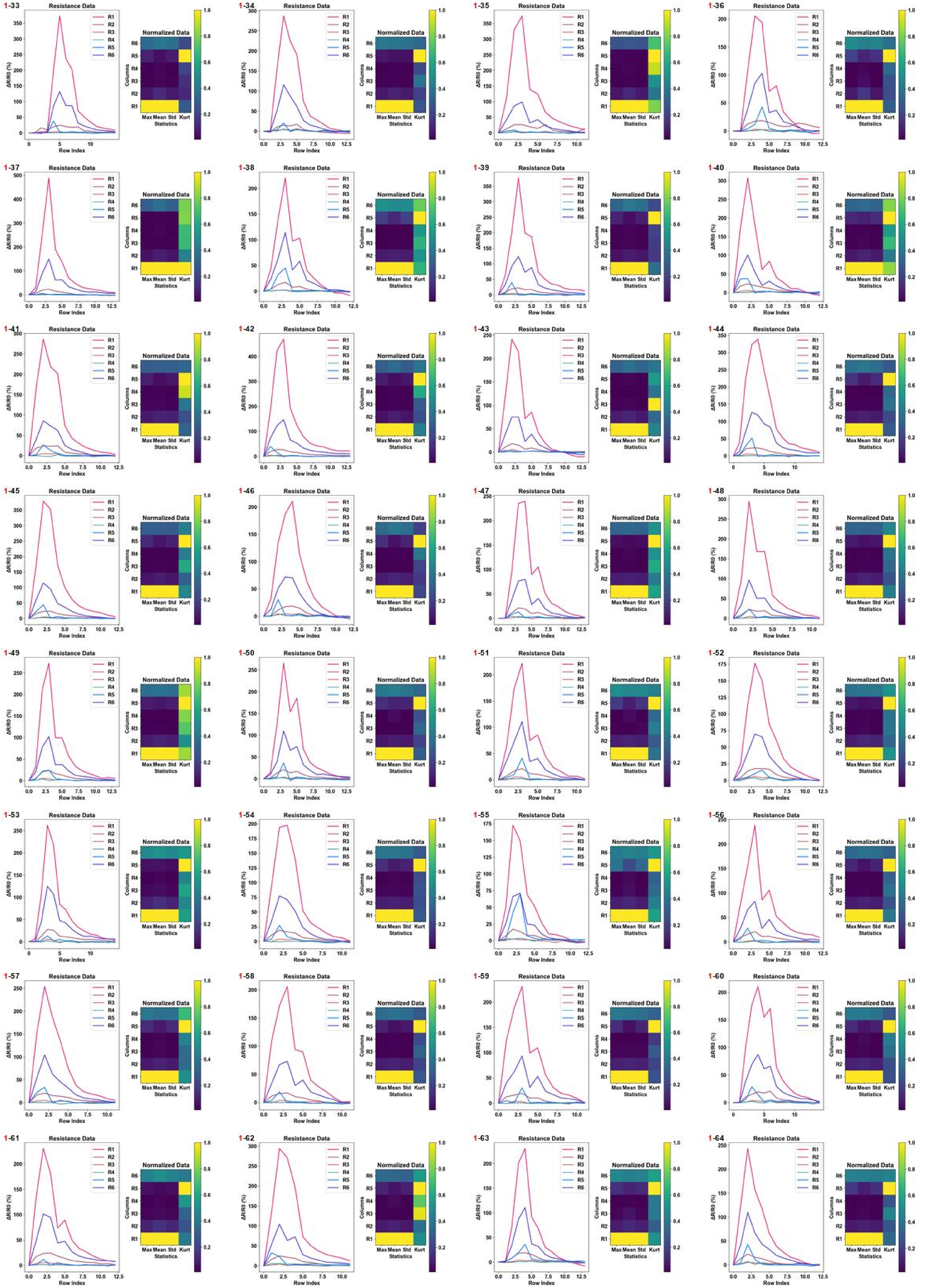
1-33
1-34
1-35
1-36
1-37
1-38
1-39
1-40
1-41
1-42
1-43
1-44
1-45
1-46
1-47
1-48
1-49
1-50
1-51
1-52
1-53
1-54
1-55
1-56
1-57
1-58
1-59
1-60
1-61
1-62
1-63
1-64
Resistance Data
ΔR/R0 (%)
Row Index
R1
R2
R3
R4
R5
R6
Normalized Data
Columns
Max Mean Std Kurt
Statistics

1-65
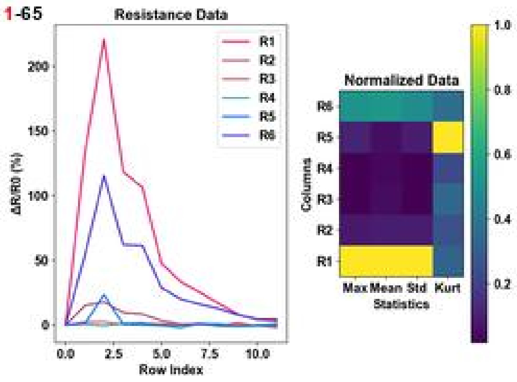

1-66
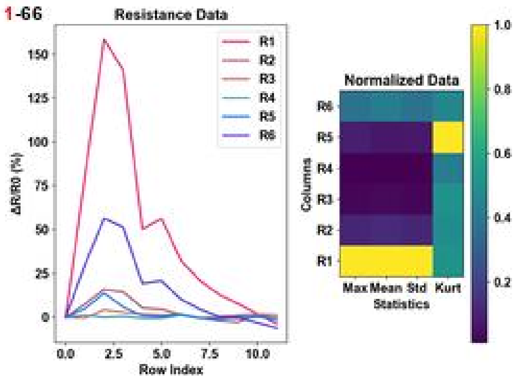

1-67
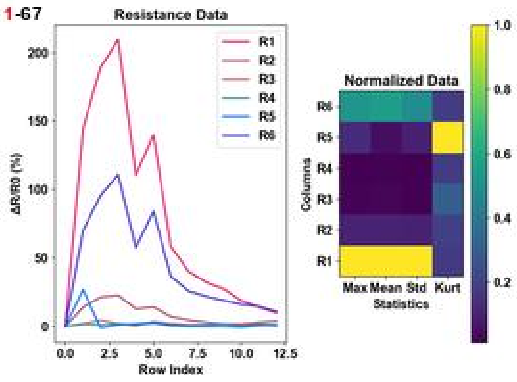

1-68
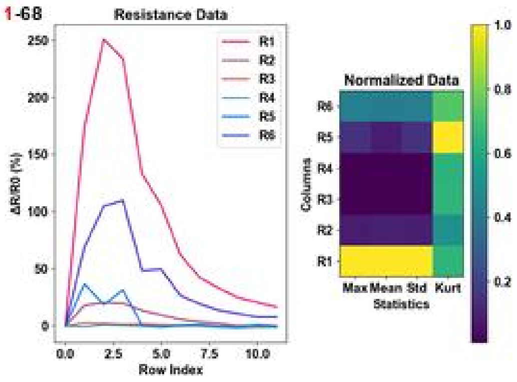

1-69
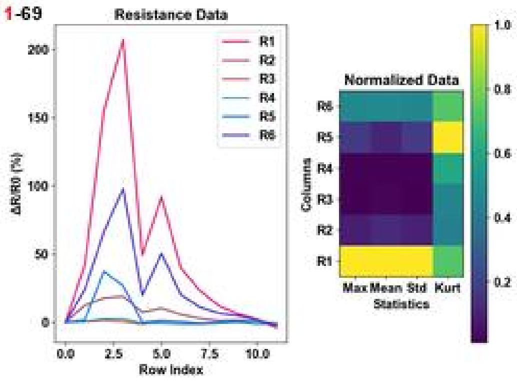

1-70
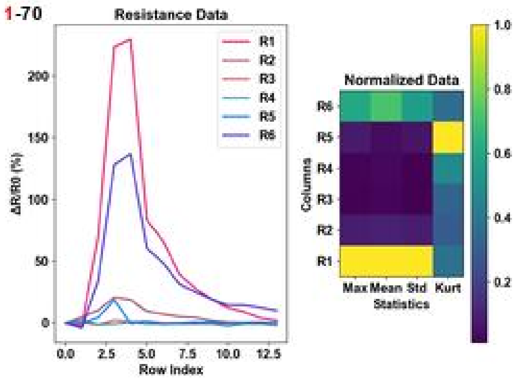

1-71
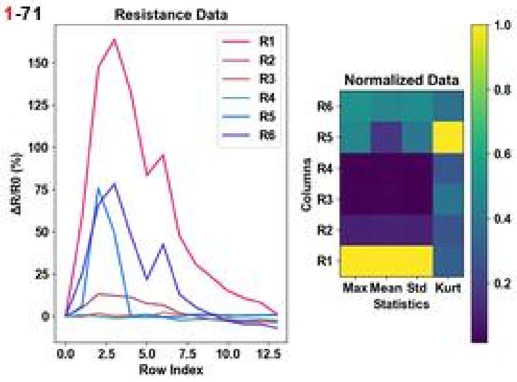

1-72
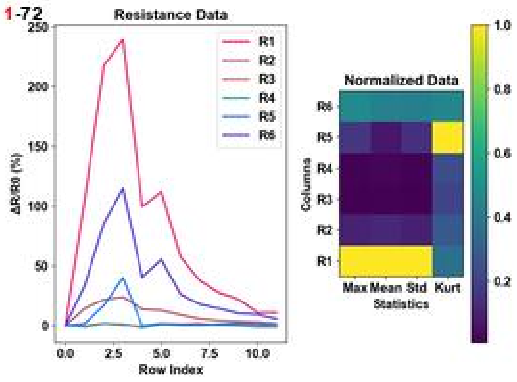

1-73
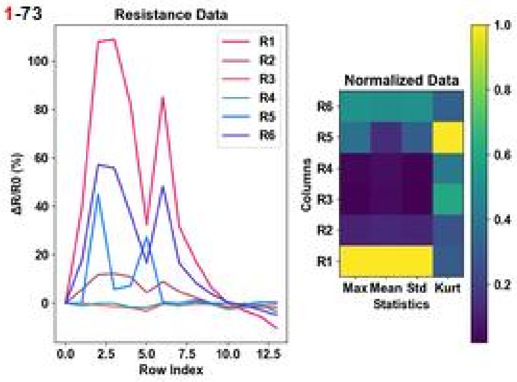

1-74
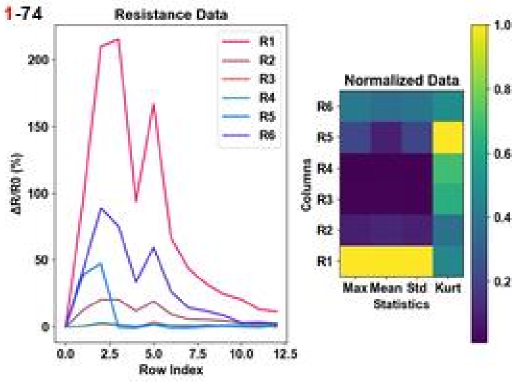

1-75
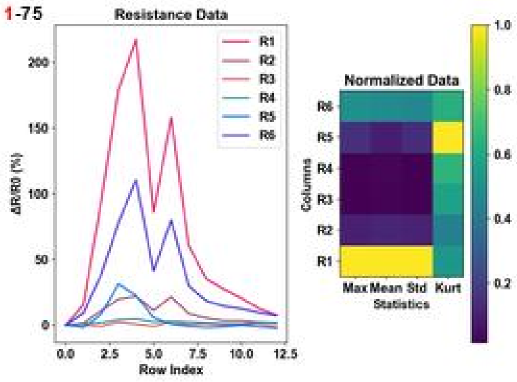

1-76
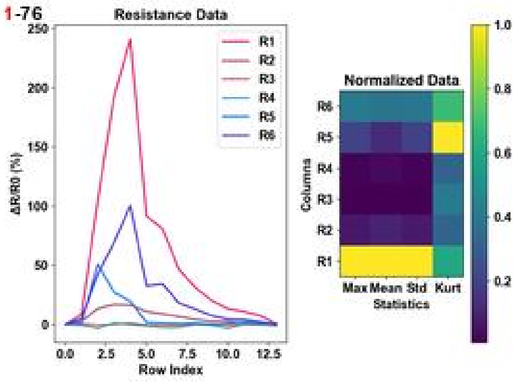

1-77
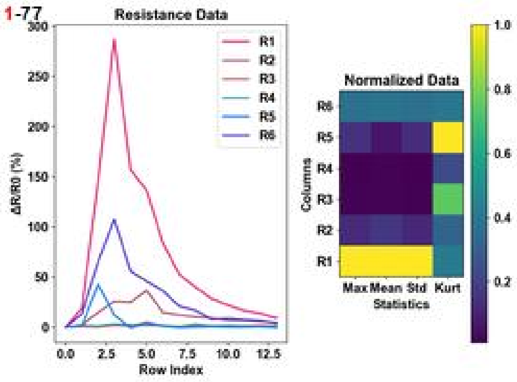

1-78
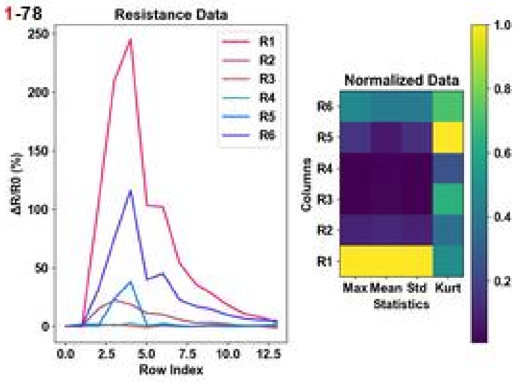

1-79
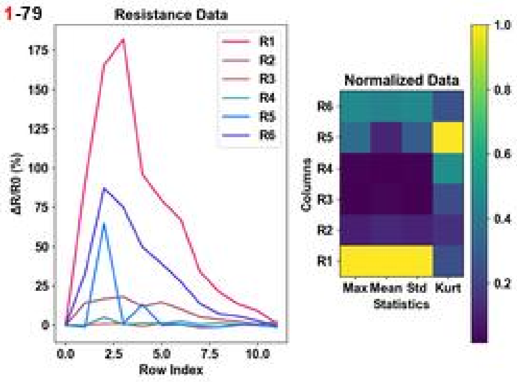

1-80
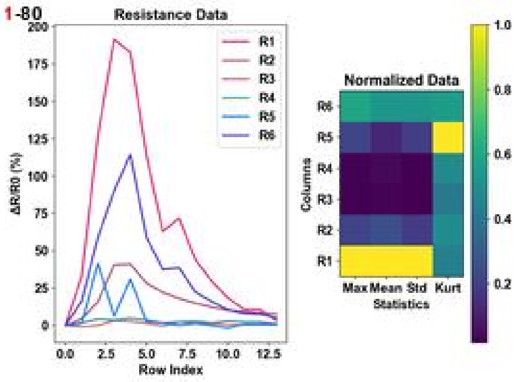

1-81
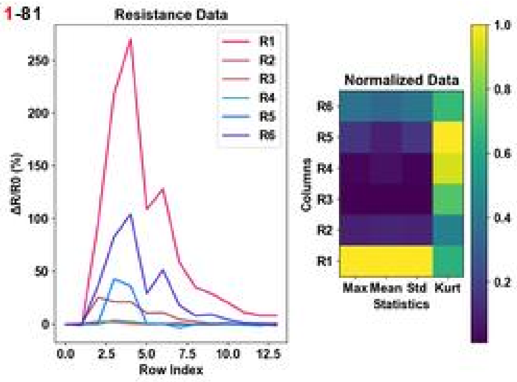

1-82
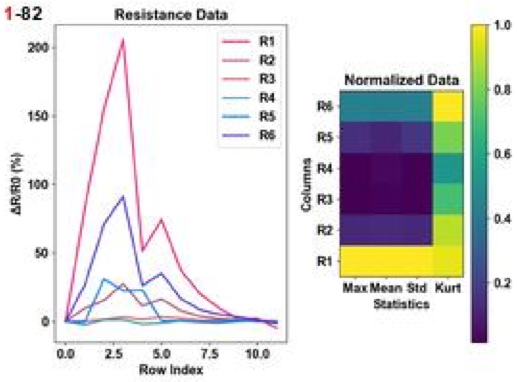

1-83
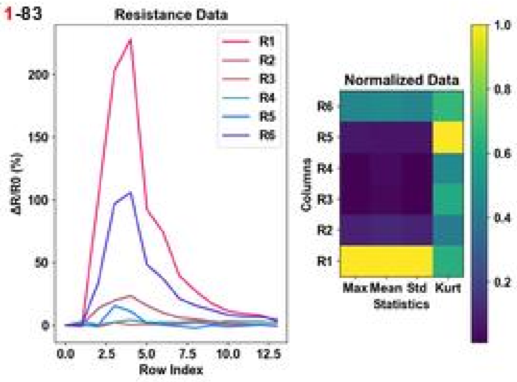

1-84
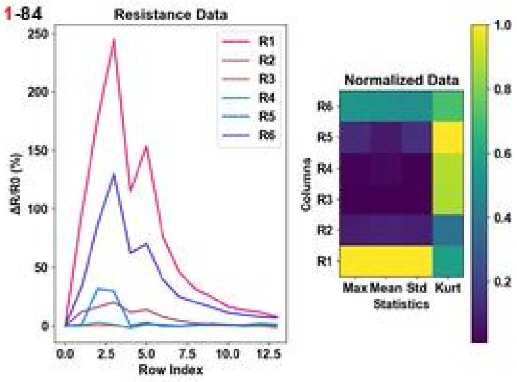

1-85
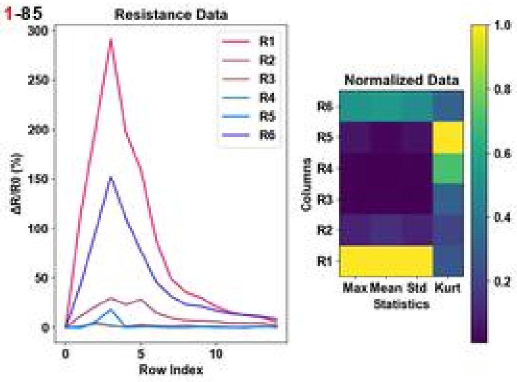

1-86
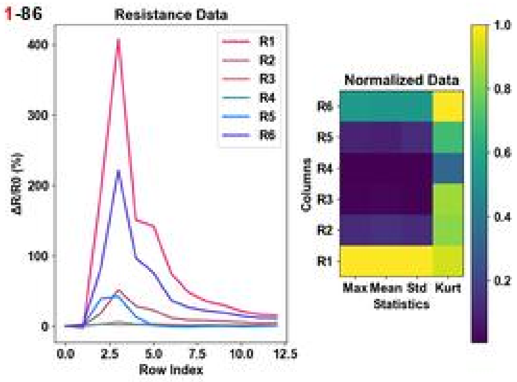

1-87
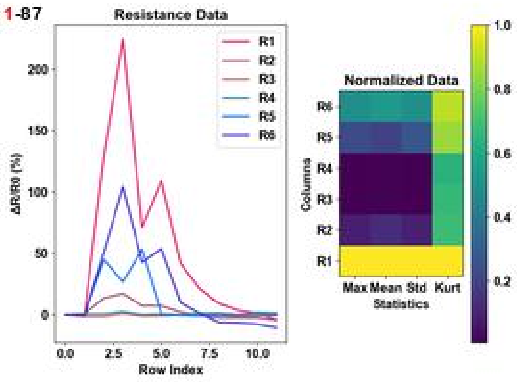

1-88
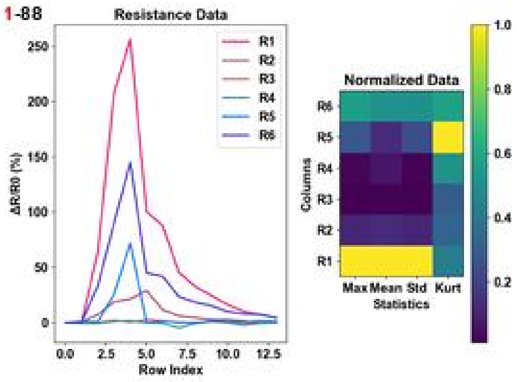

1-89
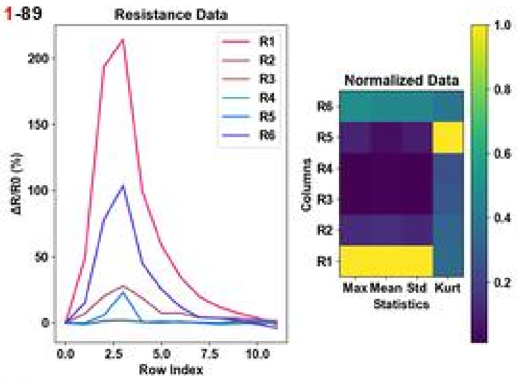

1-90
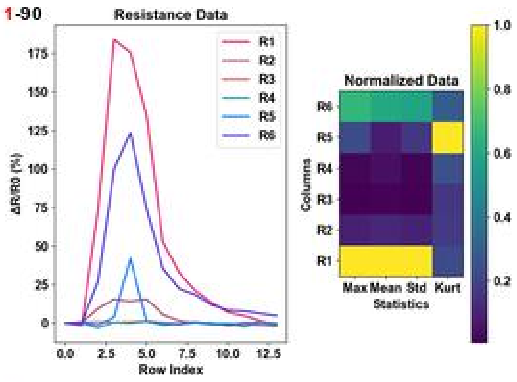

1-91
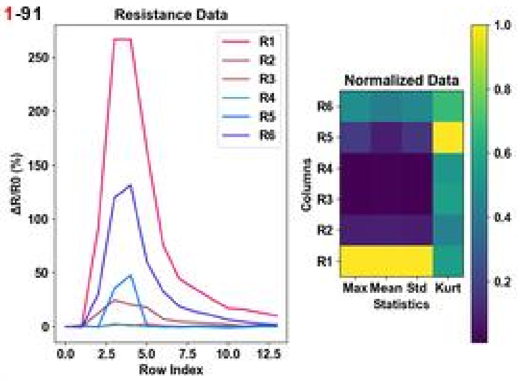

1-92
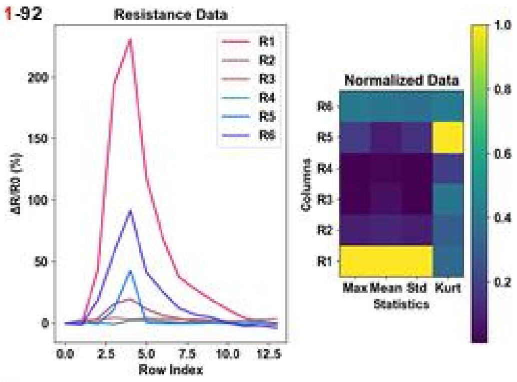

1-93
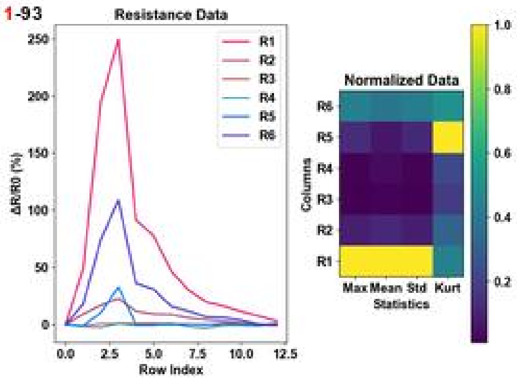

1-94
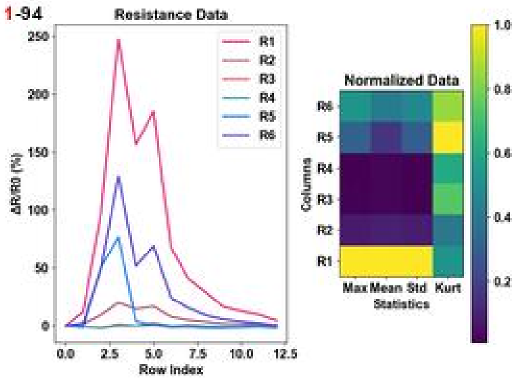

1-95
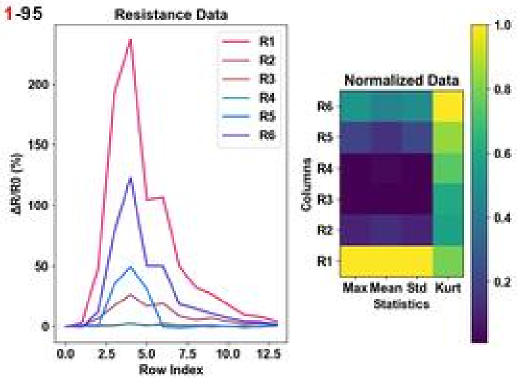

1-96
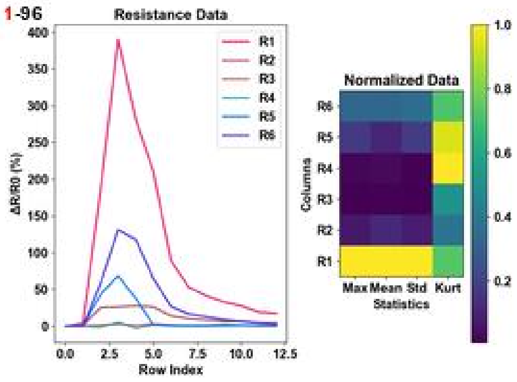

Pressure-sensitive data of type 1

1-97
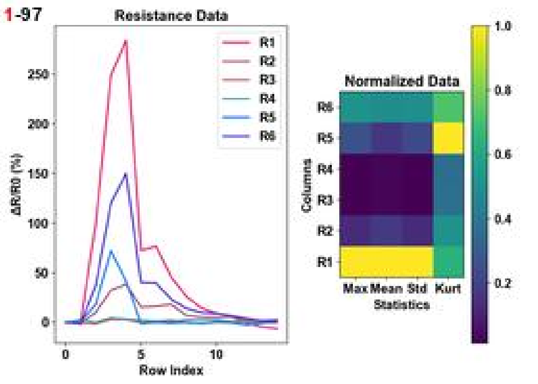

1-98
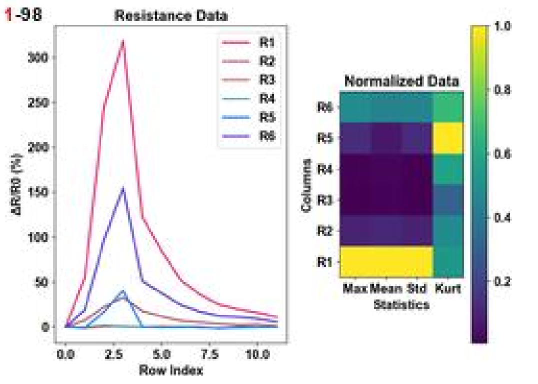

1-99
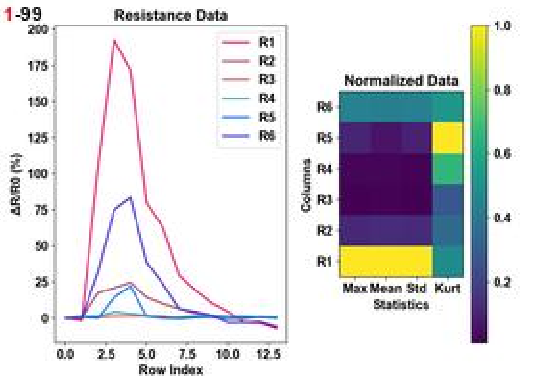

1-100
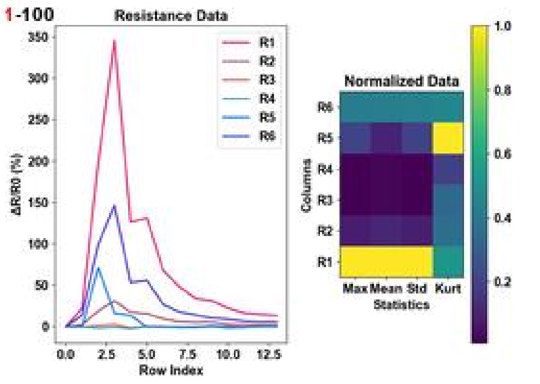

1-101
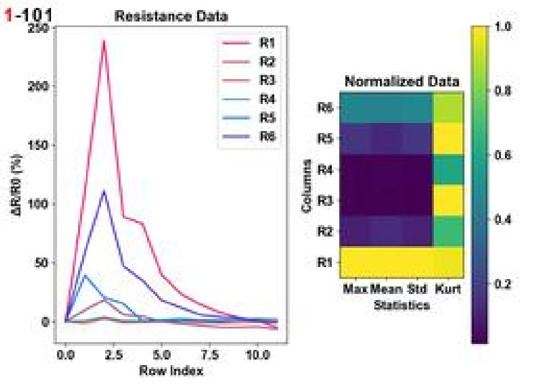

1-102
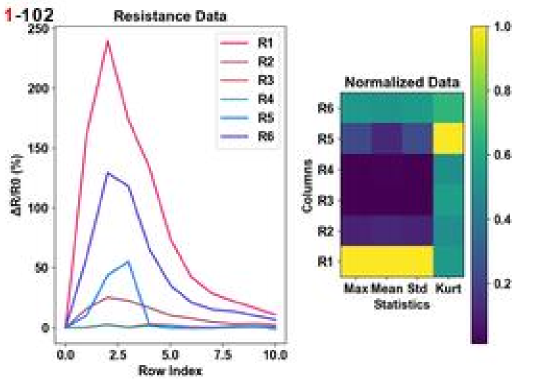

1-103
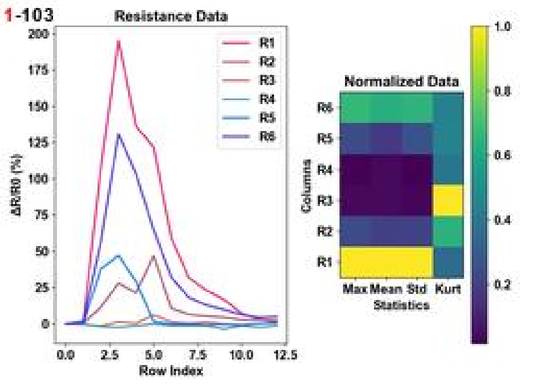

1-104
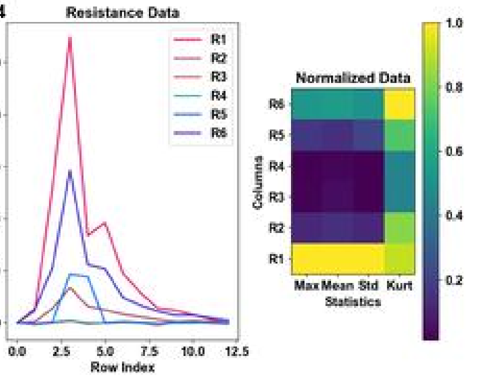

1-105
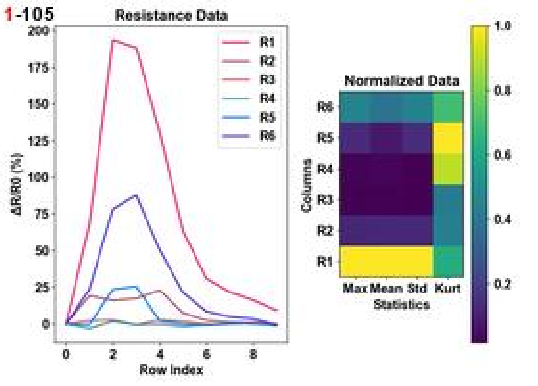

1-106
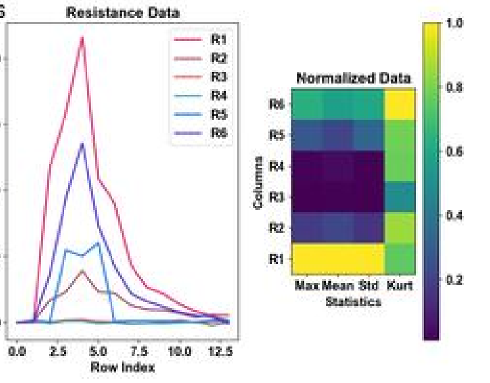

1-107
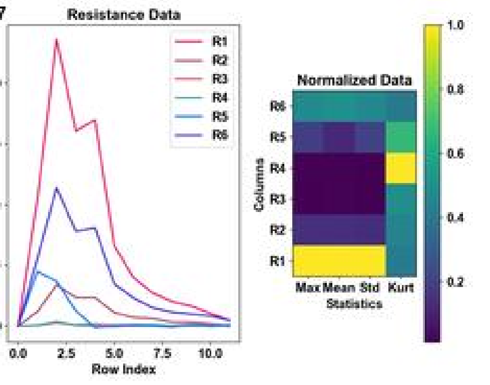

1-108
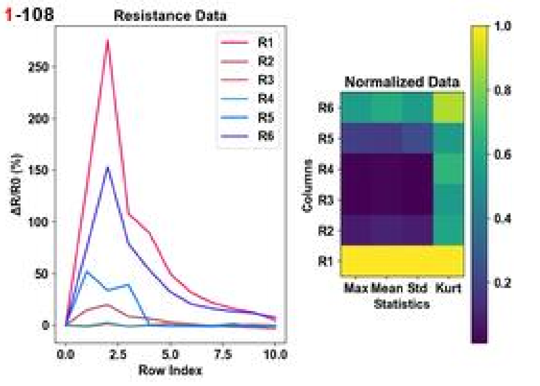

1-109
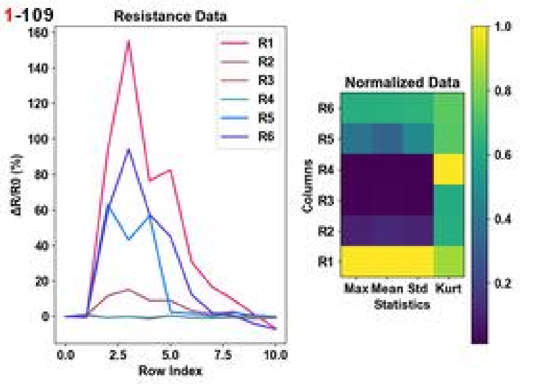

1-110
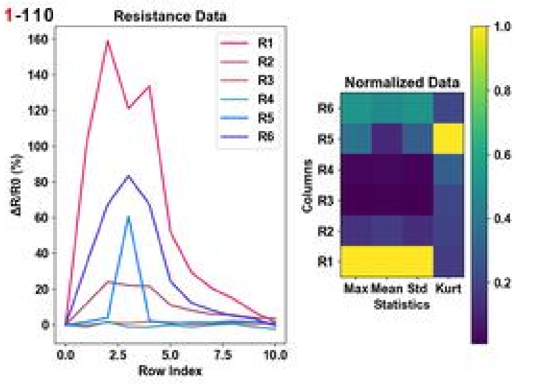

1-111
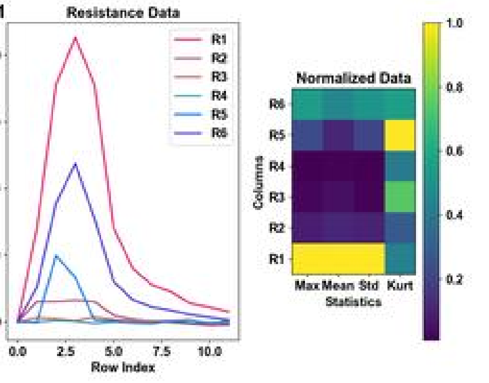

1-112
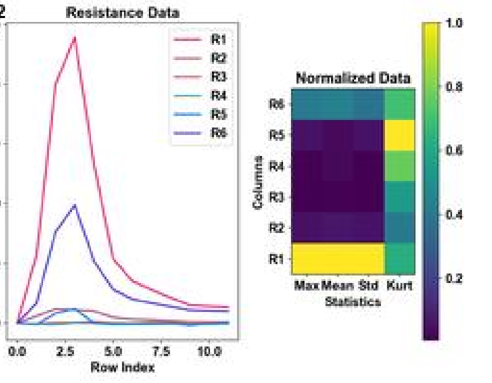

1-113
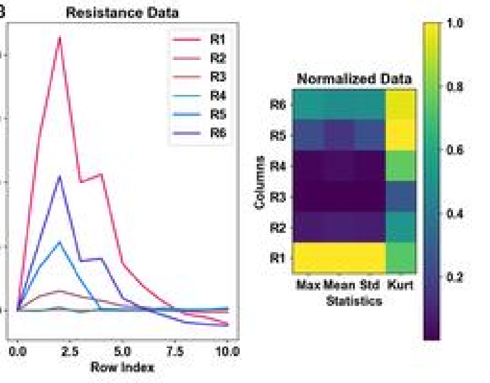

1-114
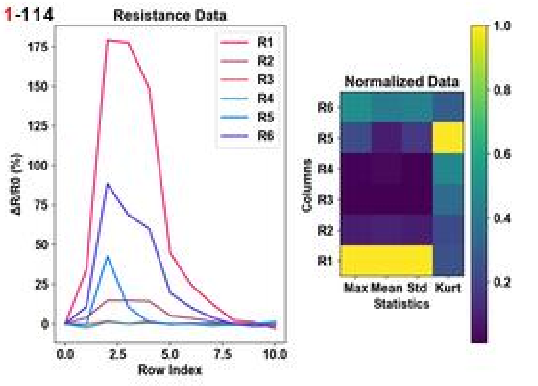

1-115
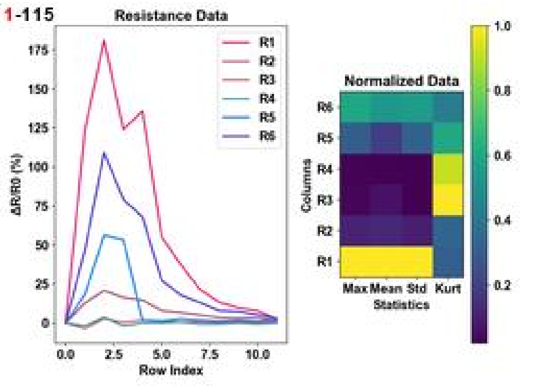

1-116
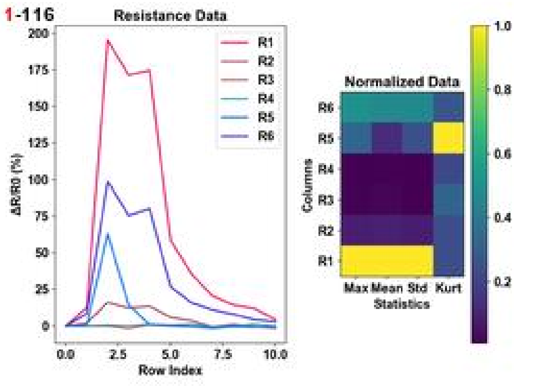

1-117
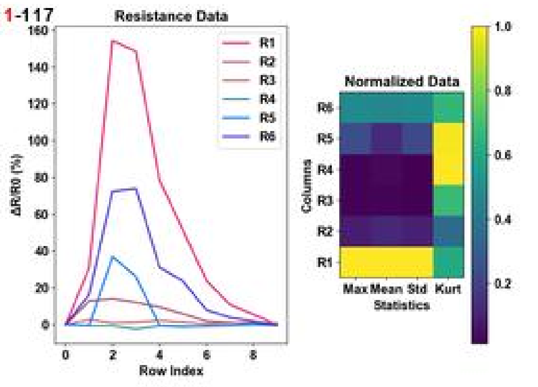

1-118
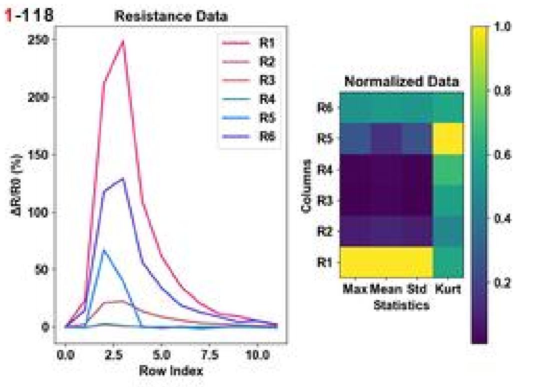

1-119
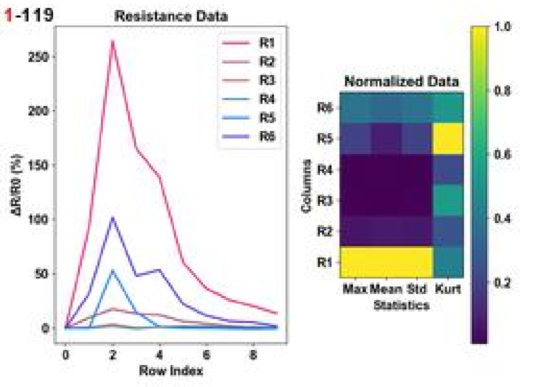

1-120
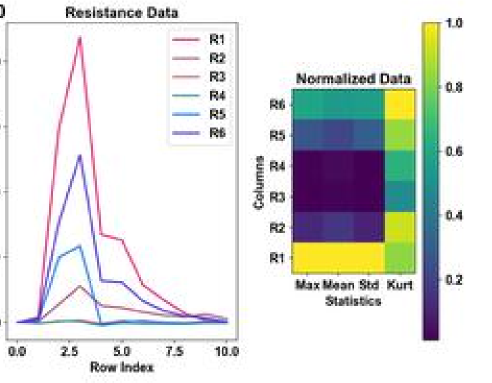

1-121
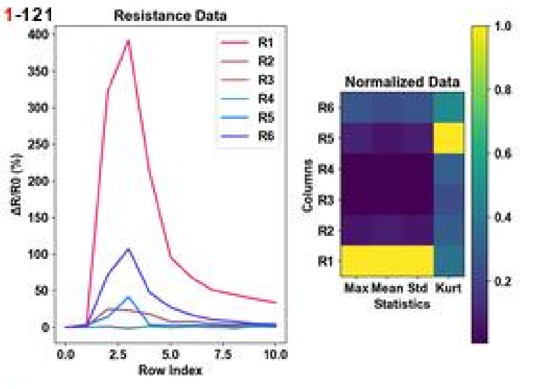

1-122
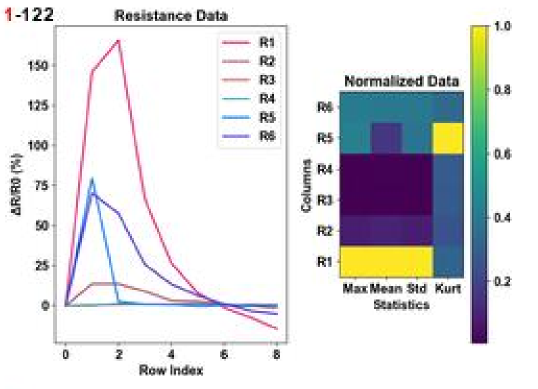

1-123
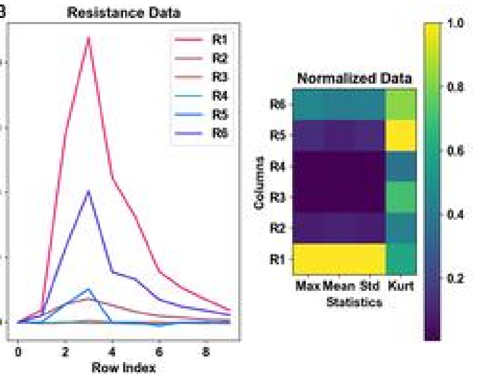

1-124
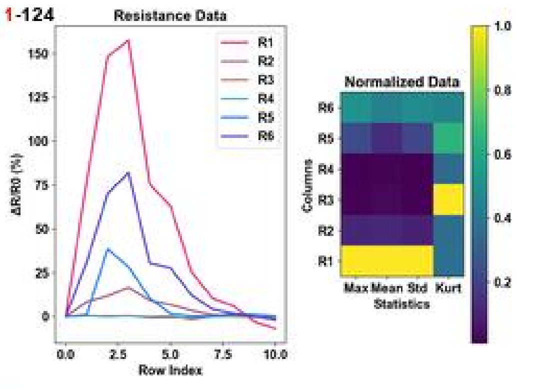

1-125
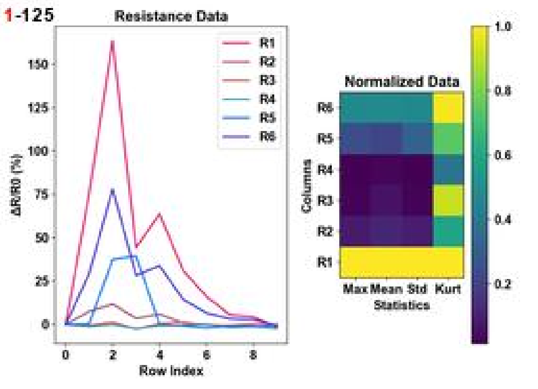

1-126
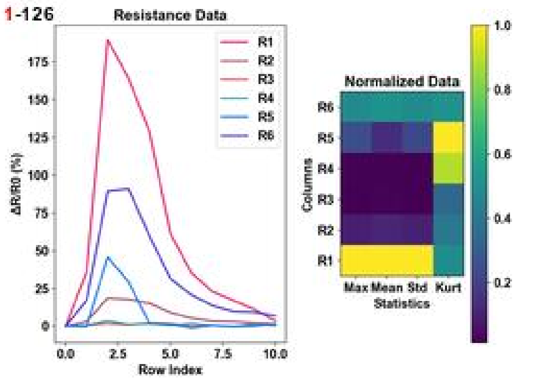

1-127
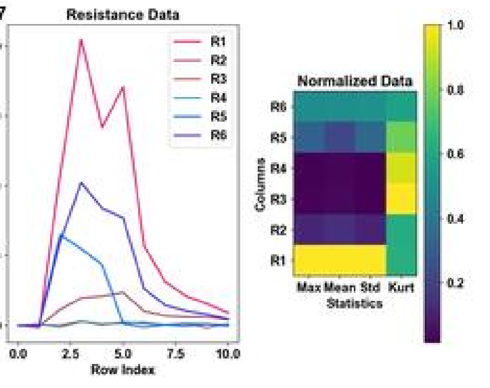

1-128
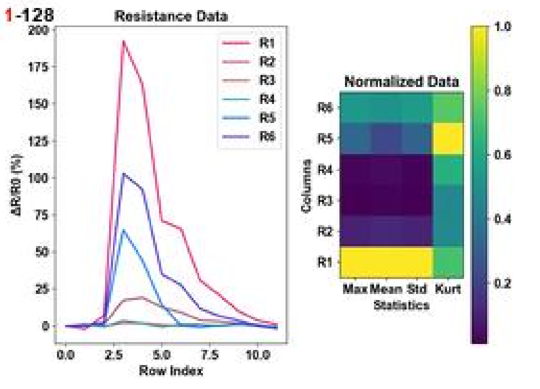

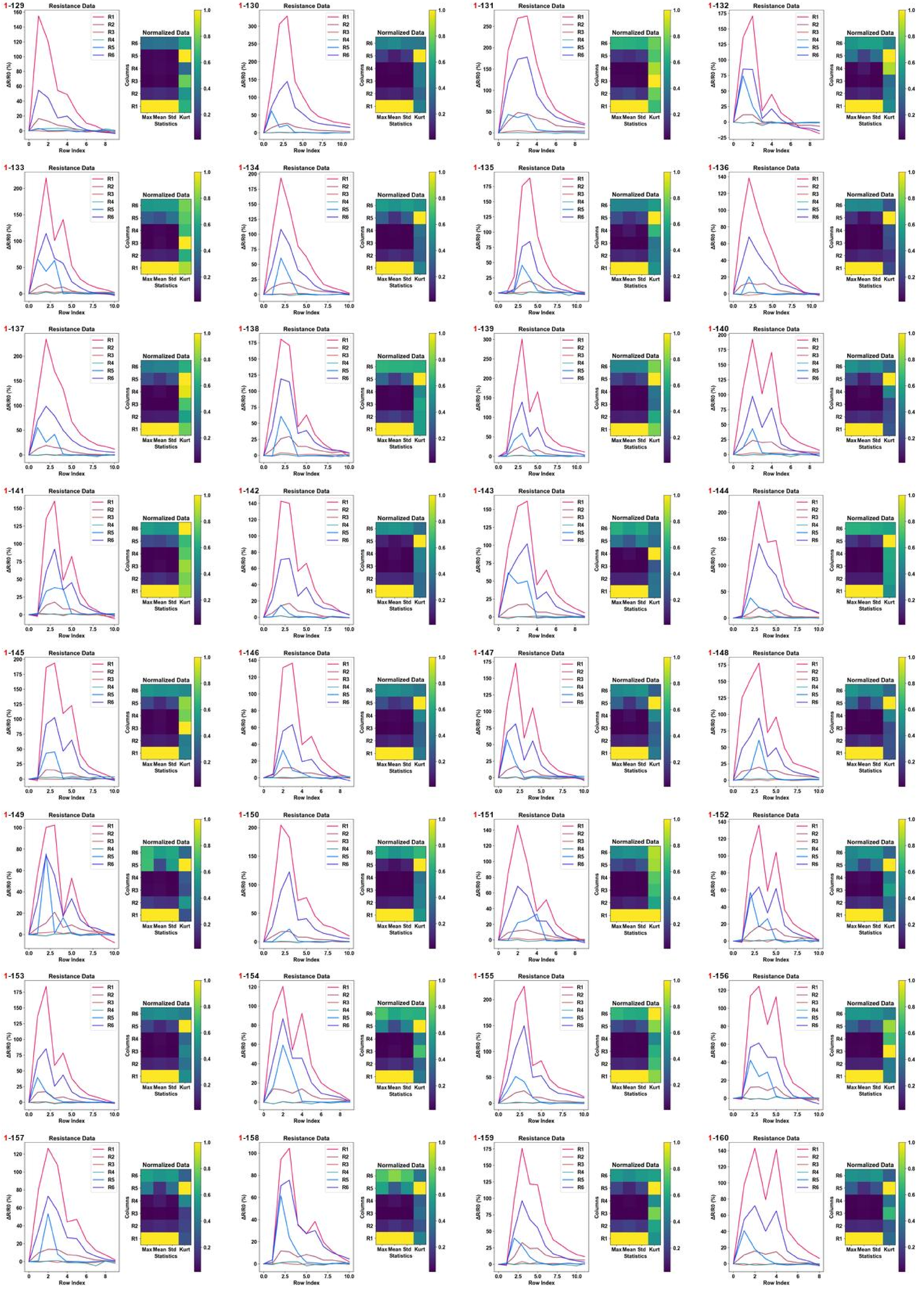
1-129
1-130
1-131
1-132
1-133
1-134
1-135
1-136
1-137
1-138
1-139
1-140
1-141
1-142
1-143
1-144
1-145
1-146
1-147
1-148
1-149
1-150
1-151
1-152
1-153
1-154
1-155
1-156
1-157
1-158
1-159
1-160
Resistance Data
ΔR/R0 (%)
Row Index
R1
R2
R3
R4
R5
R6
Normalized Data
Columns
Statistics
Max
Mean
Std
Kurt

1-161
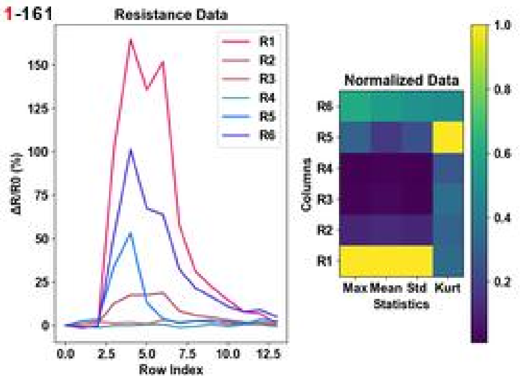

1-162
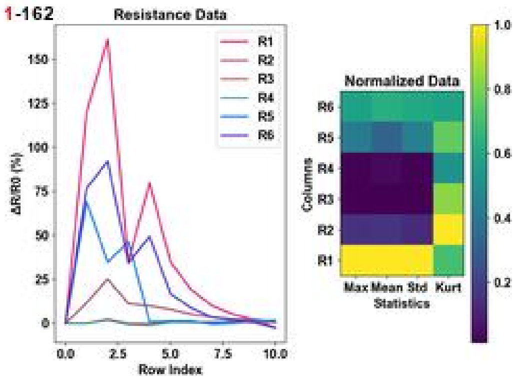

1-163
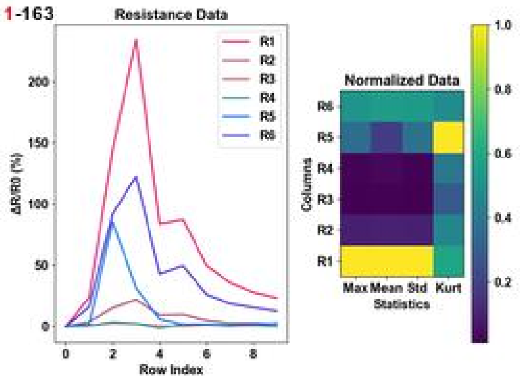

1-164
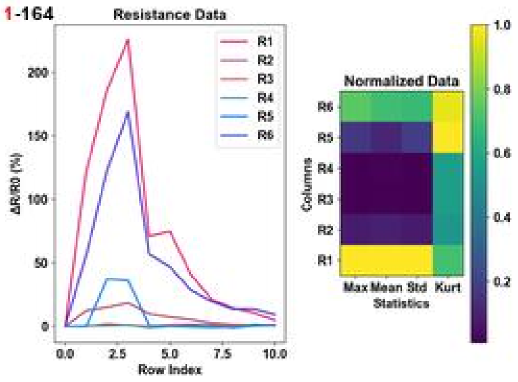

1-165
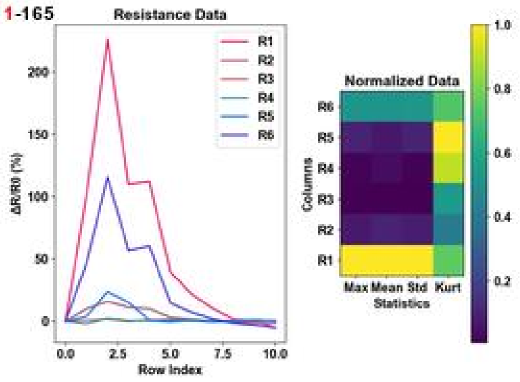

1-166
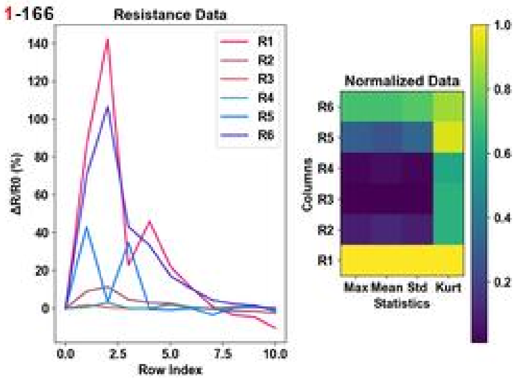

1-167
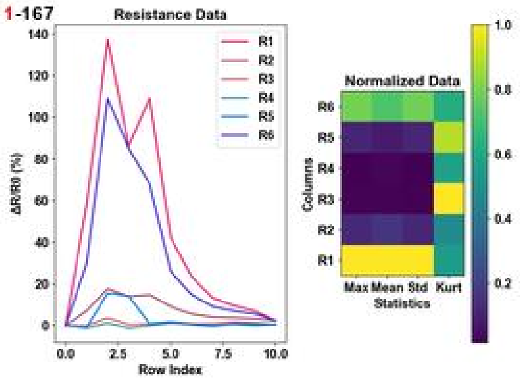

1-168
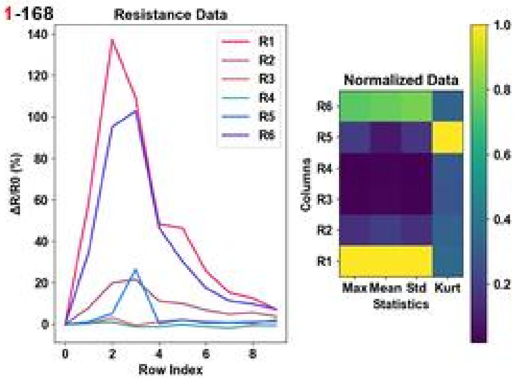

1-169
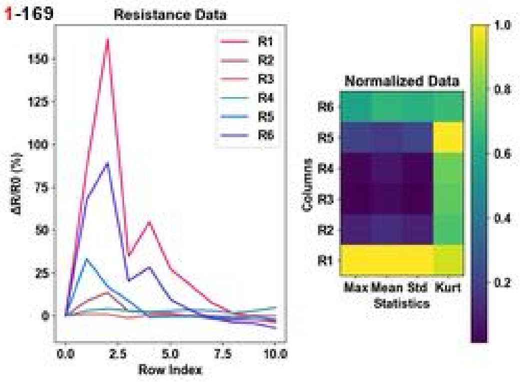

1-170
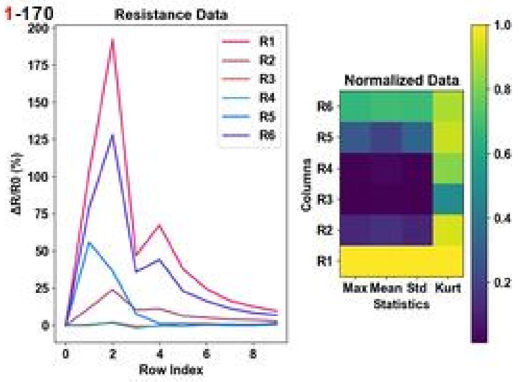

1-171
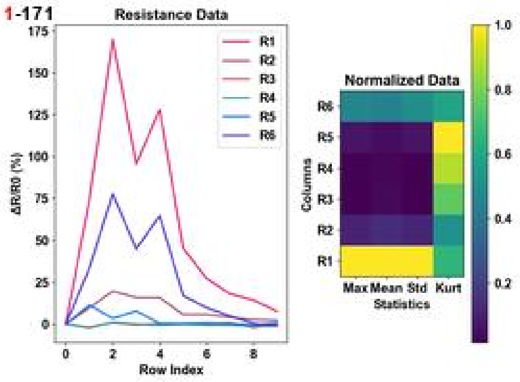

1-172
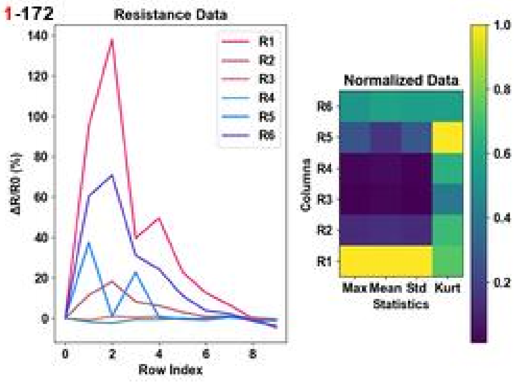

1-173
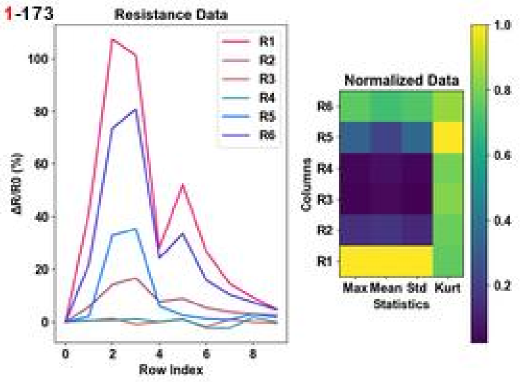

1-174
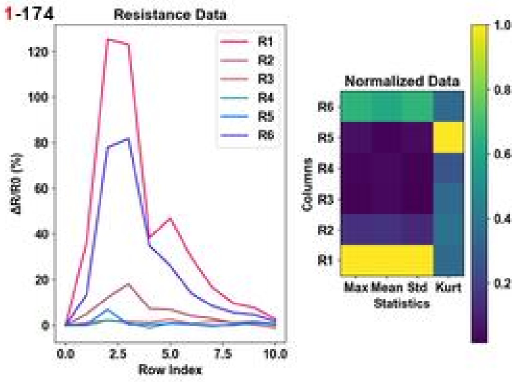

1-175
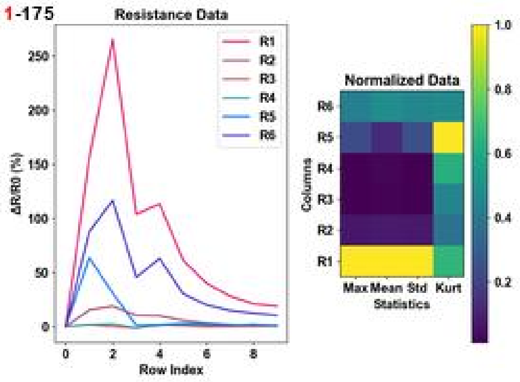

1-176
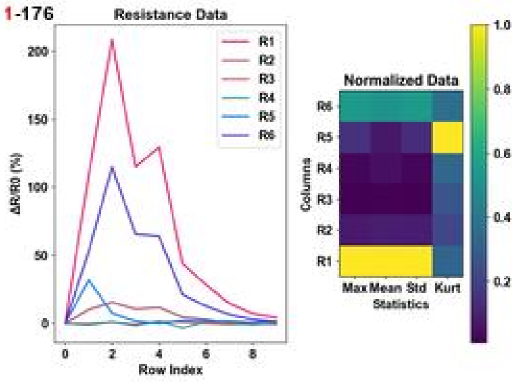

1-177
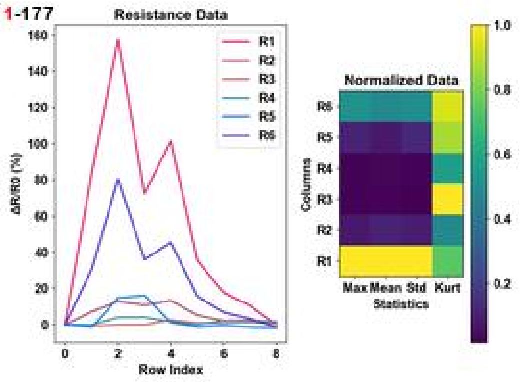

1-178
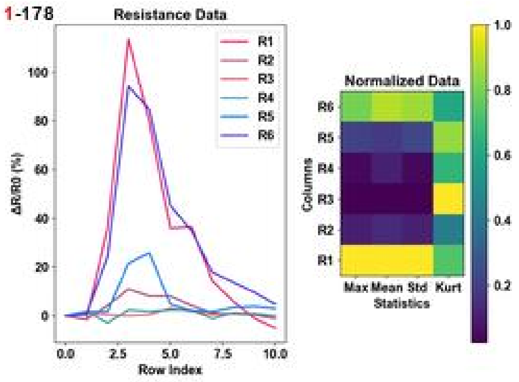

1-179
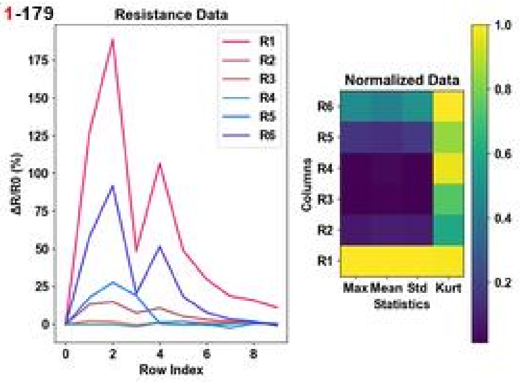

1-180
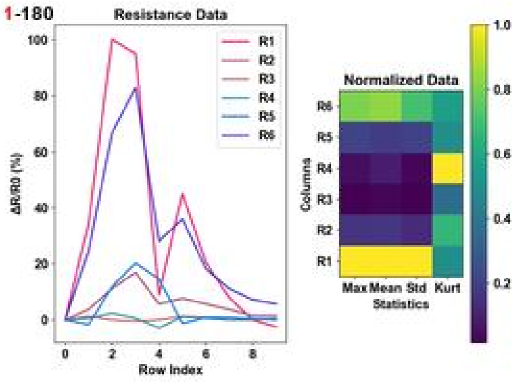

1-181
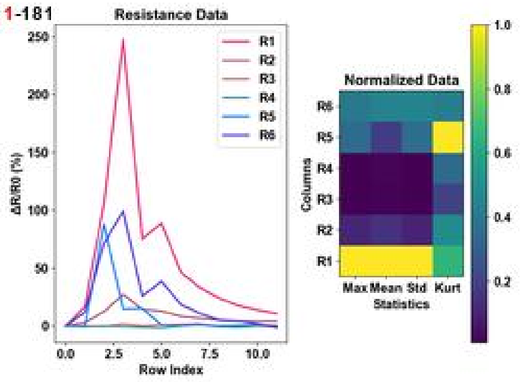

1-182
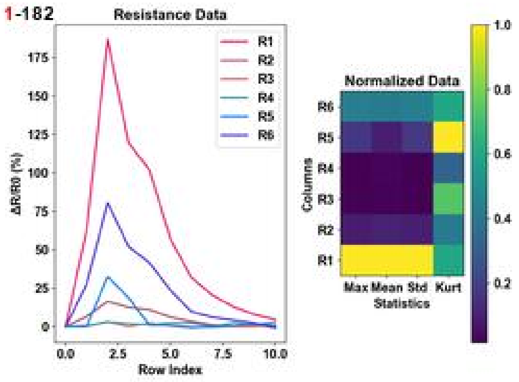

1-183
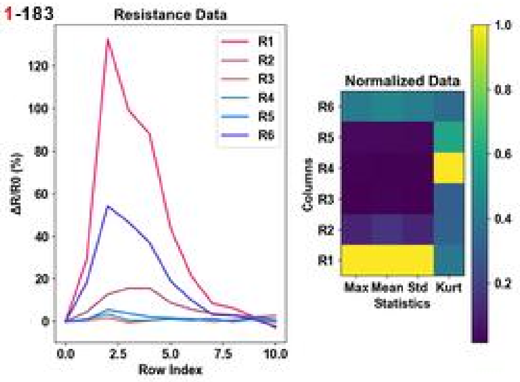

1-184
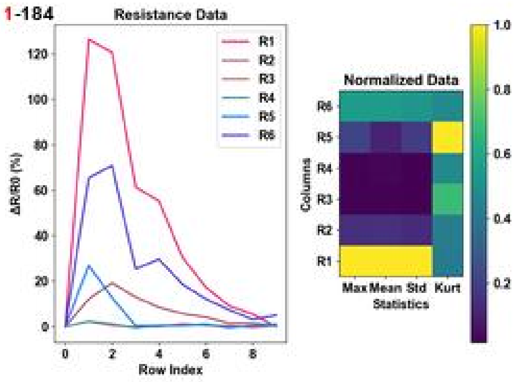

1-185
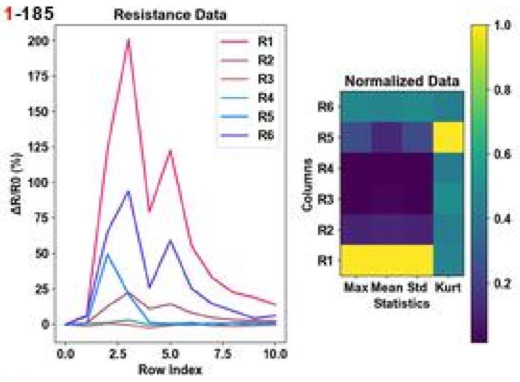

1-186
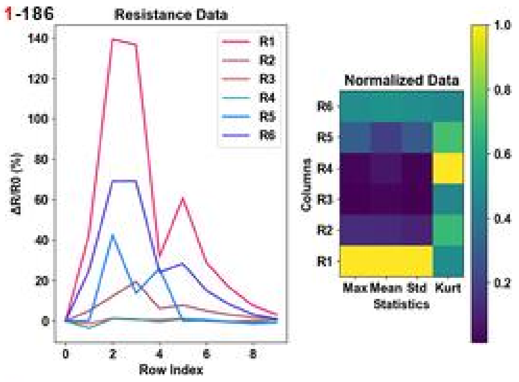

1-187
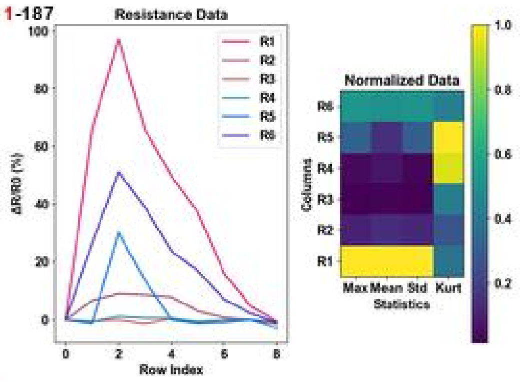

1-188
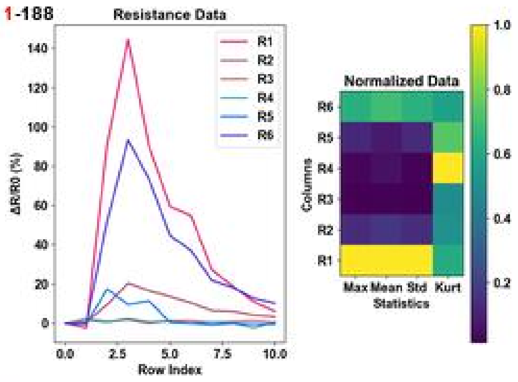

1-189
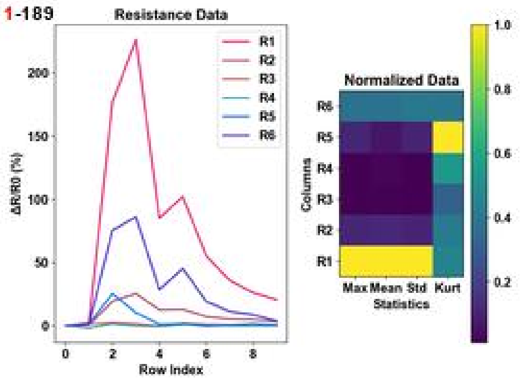

1-190
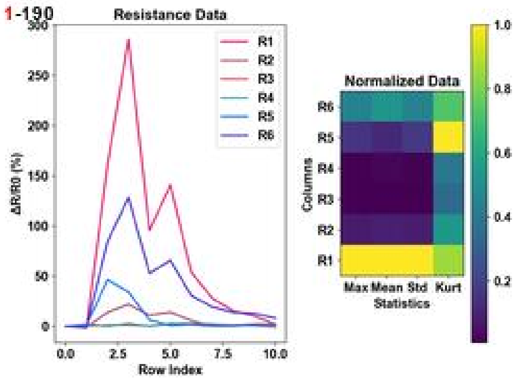

1-191
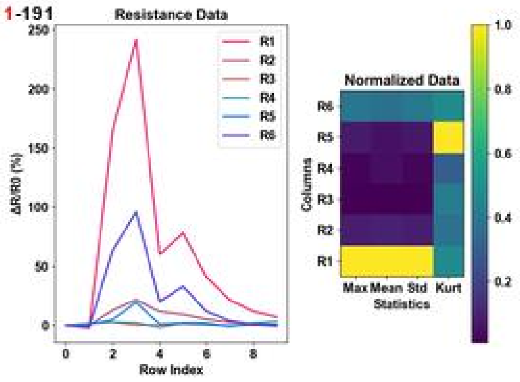

1-192
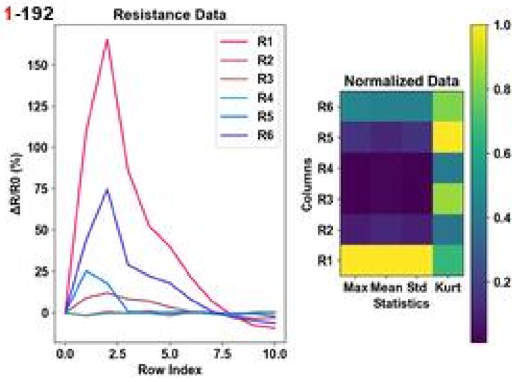

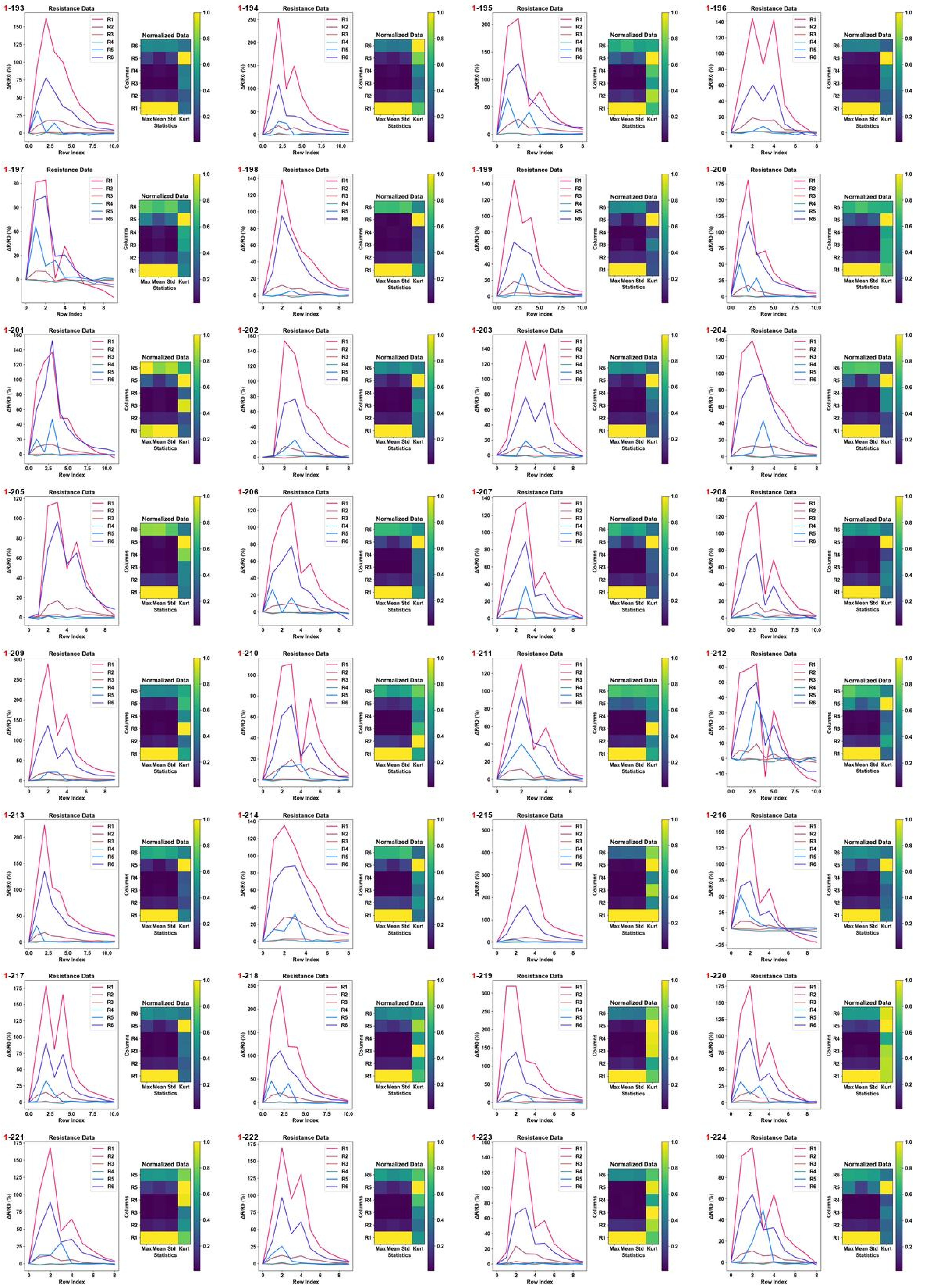
1-193
1-194
1-195
1-196
1-197
1-198
1-199
1-200
1-201
1-202
1-203
1-204
1-205
1-206
1-207
1-208
1-209
1-210
1-211
1-212
1-213
1-214
1-215
1-216
1-217
1-218
1-219
1-220
1-221
1-222
1-223
1-224
Resistance Data
ΔR/R0 (%)
Row Index
R1
R2
R3
R4
R5
R6
Normalized Data
Columns
Statistics
Max
Mean
Std
Kurt

1-225
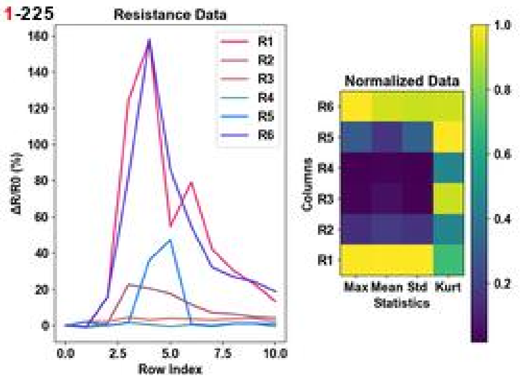

1-226
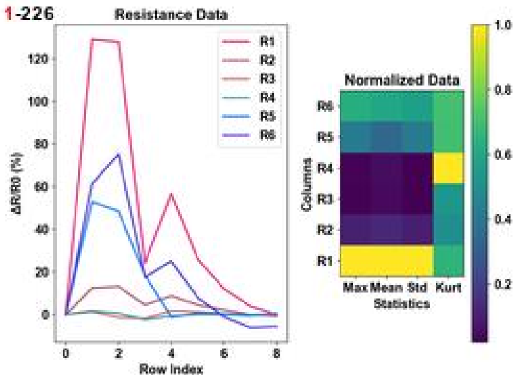

1-227
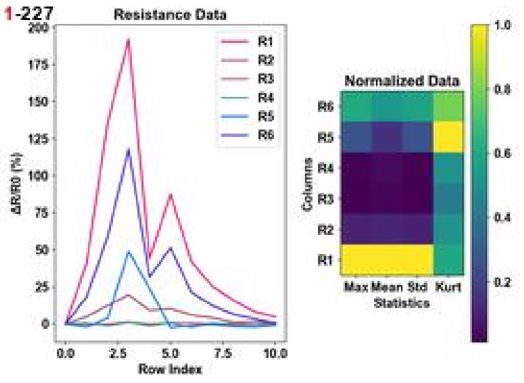

1-228
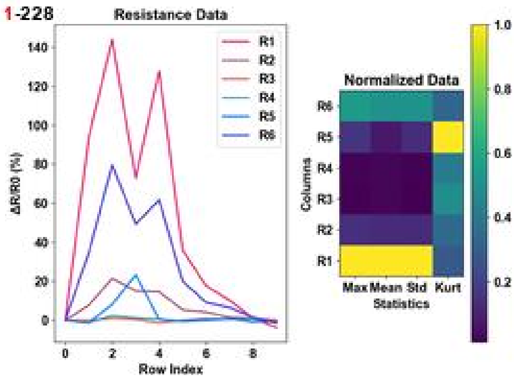

1-229
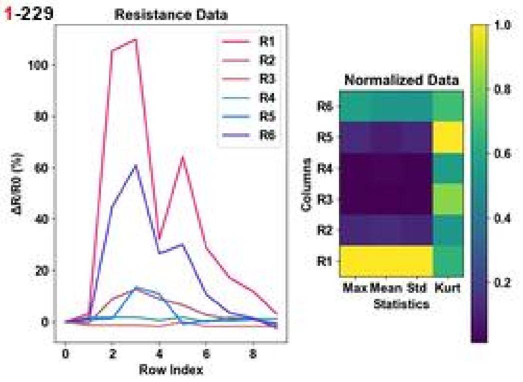

1-230
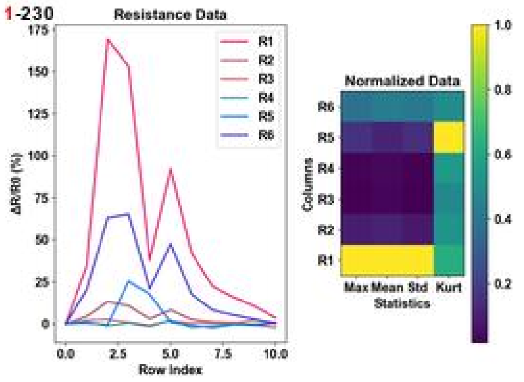

1-231
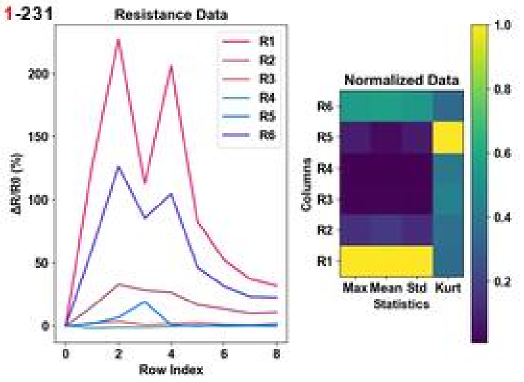

1-232
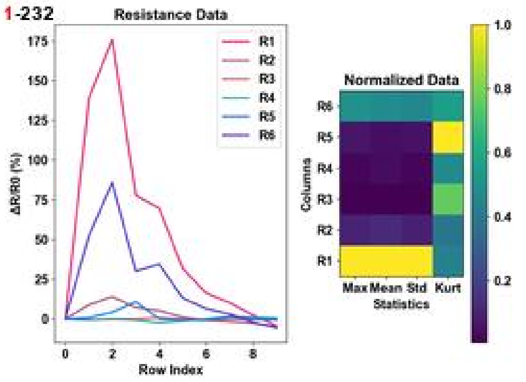

1-233
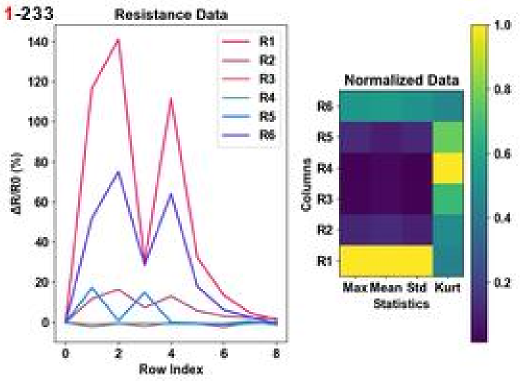

1-234
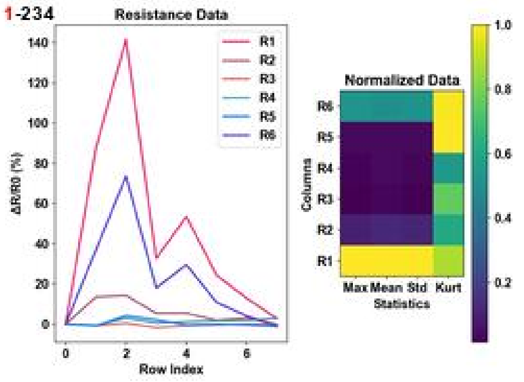

1-235
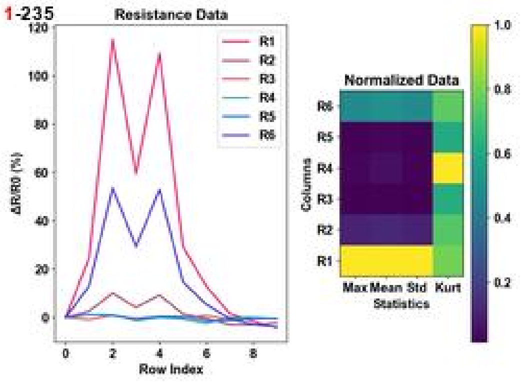

1-236
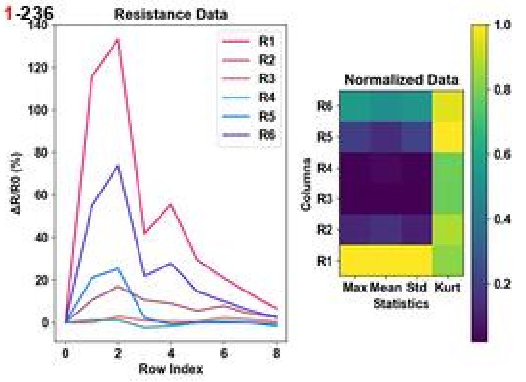

1-237
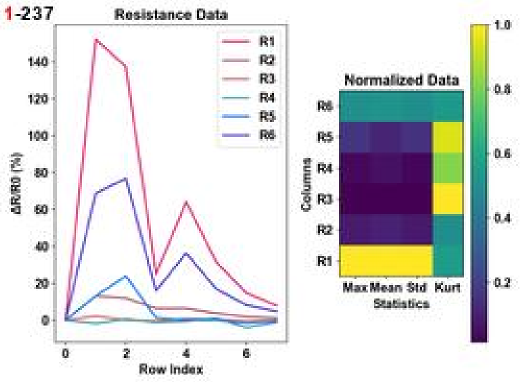

1-238
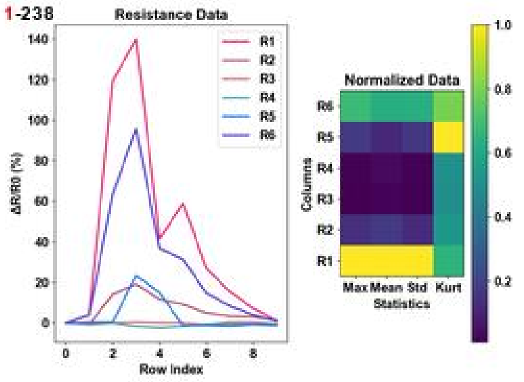

1-239
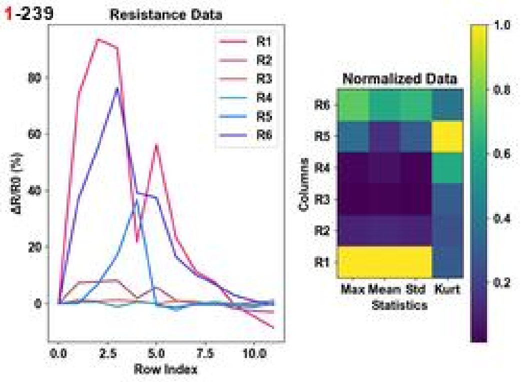

1-240
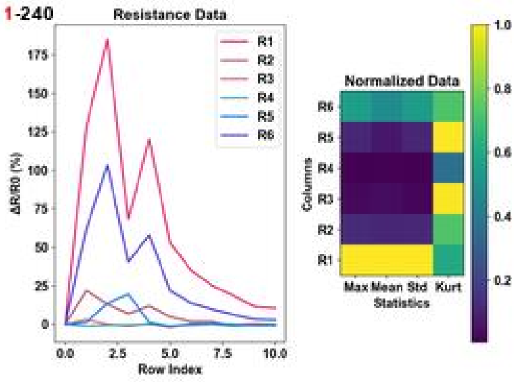

Pressure-sensitive data of type 2

2-1
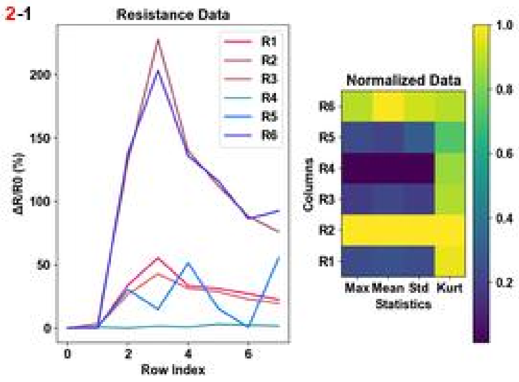

2-2
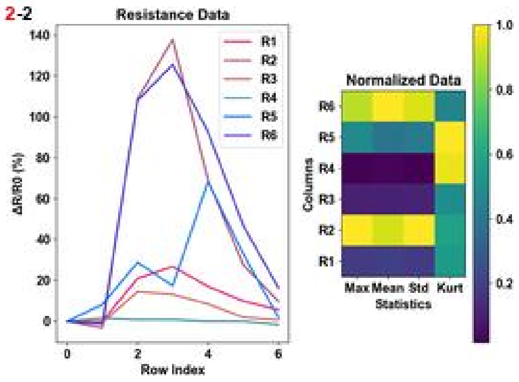

2-3
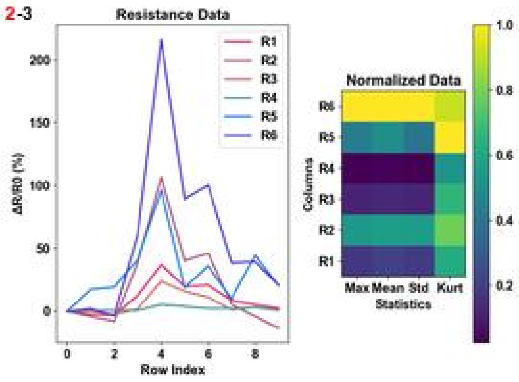

2-4
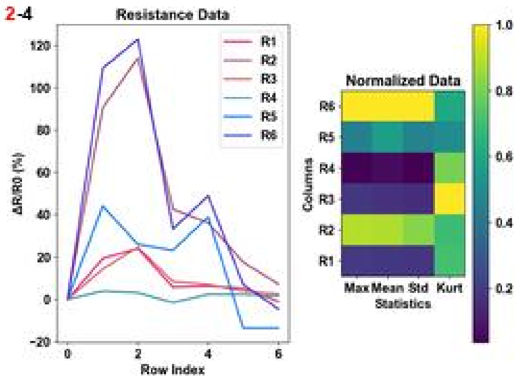

2-5
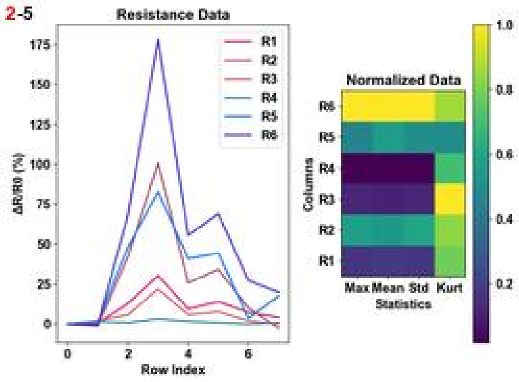

2-6
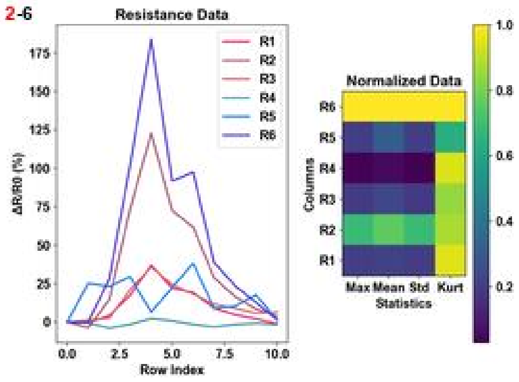

2-7
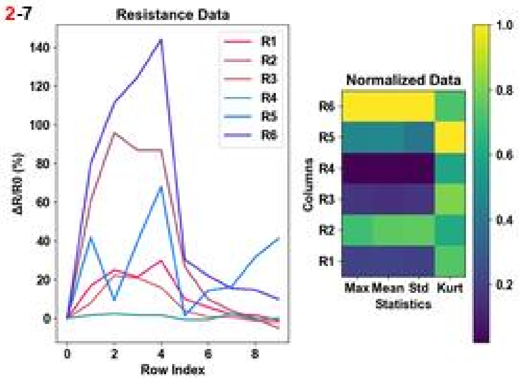

2-8
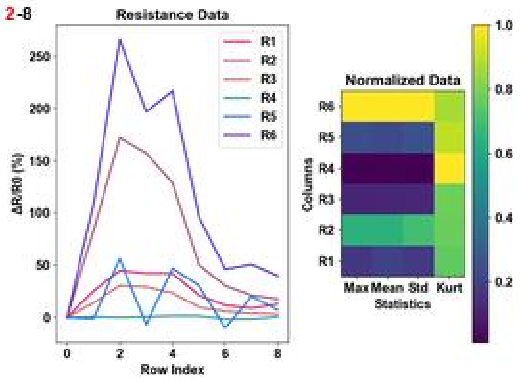

2-9
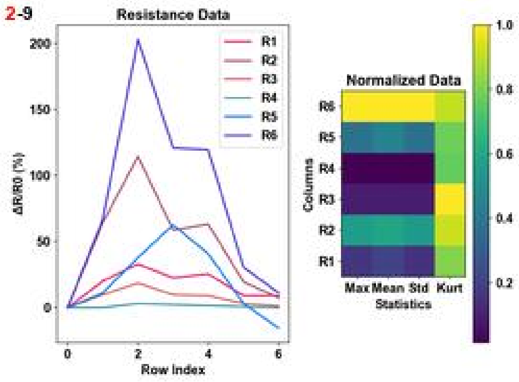

2-10
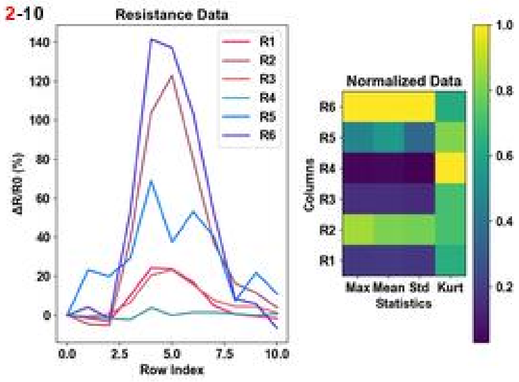

2-11
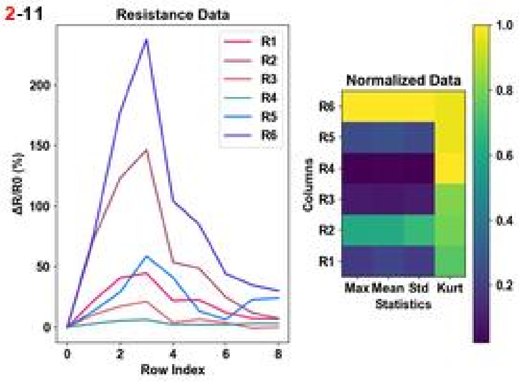

2-12
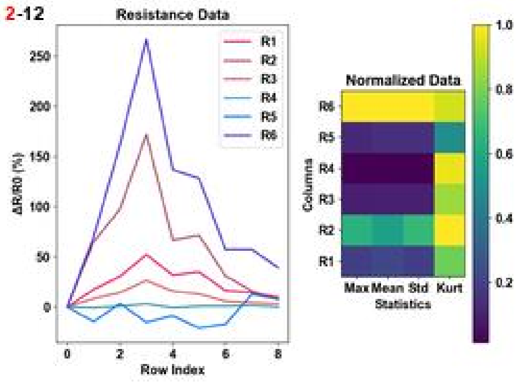

2-13
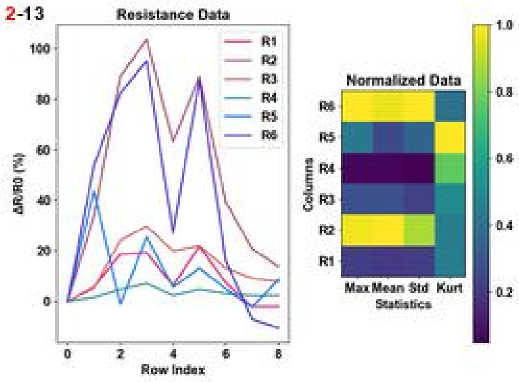

2-14
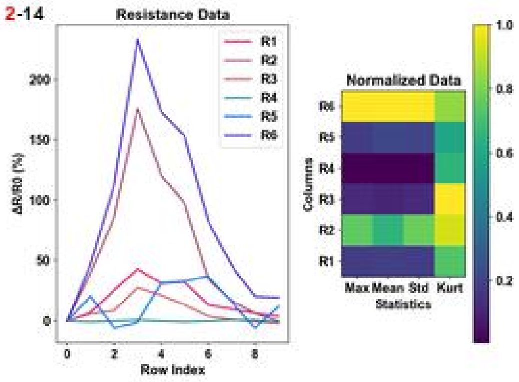

2-15
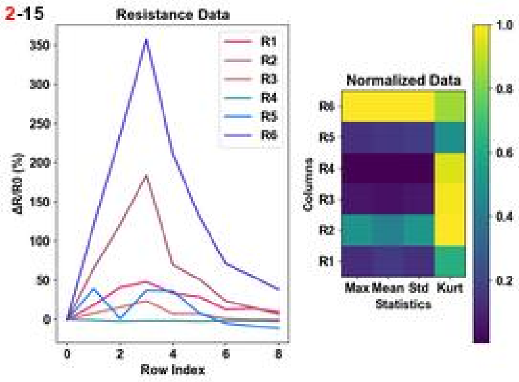

2-16
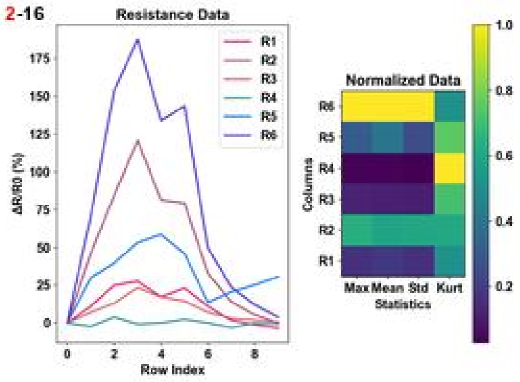

2-17
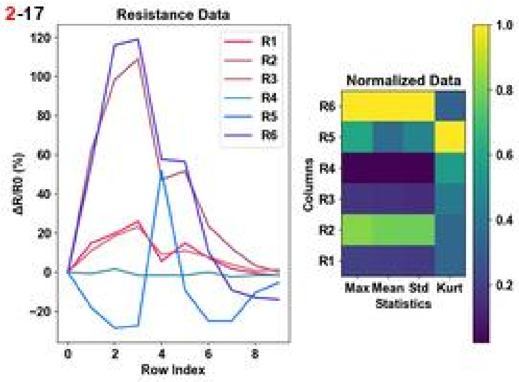

2-18
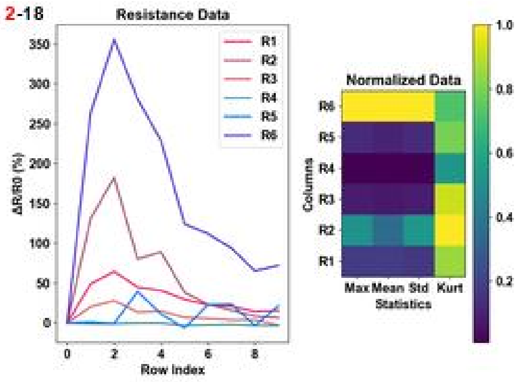

2-19
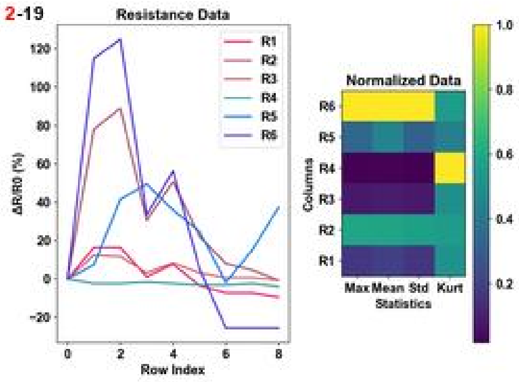

2-20
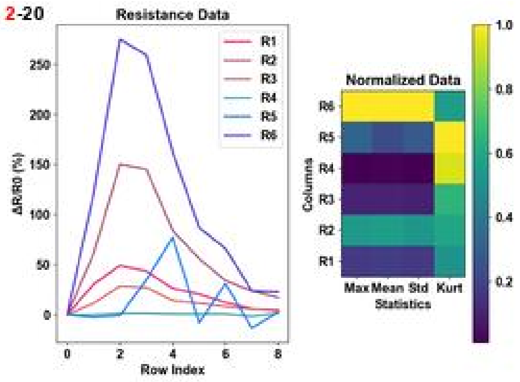

2-21
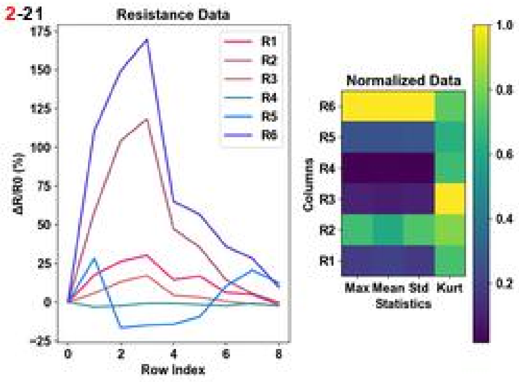

2-22
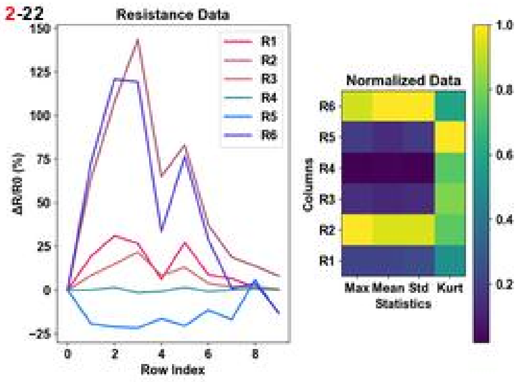

2-23
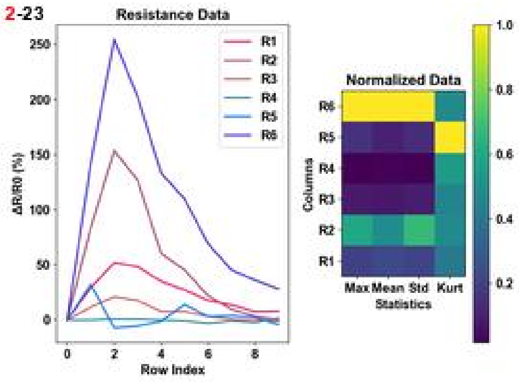

2-24
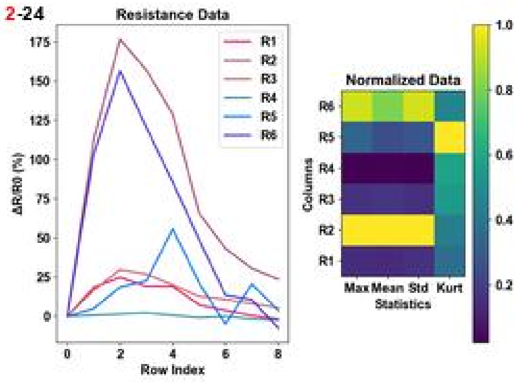

2-25
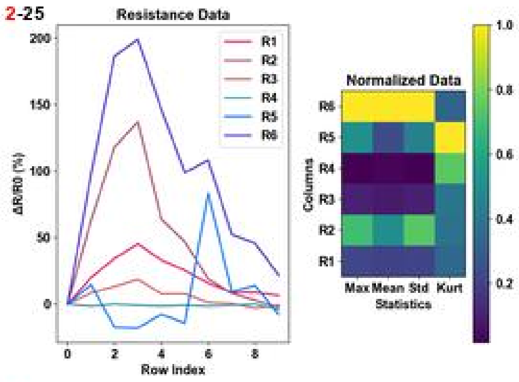

2-26
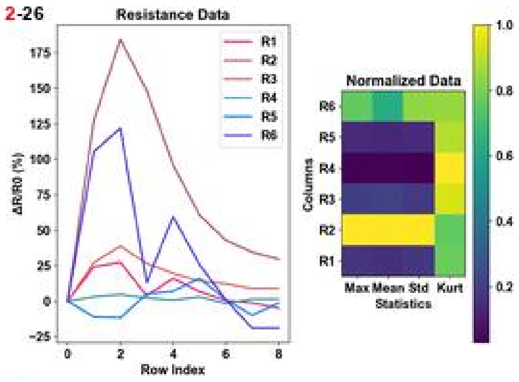

2-27
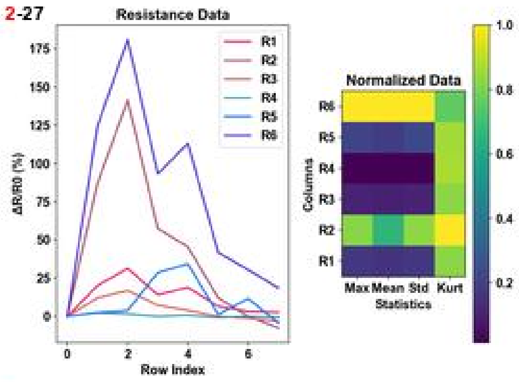

2-28
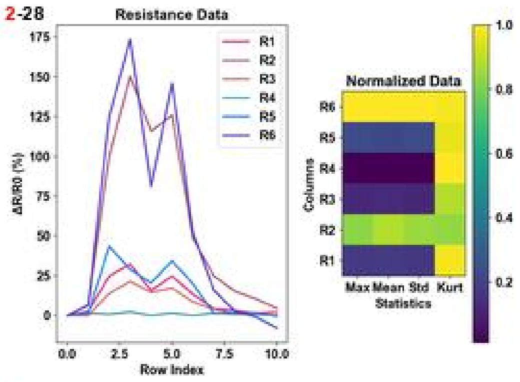

2-29
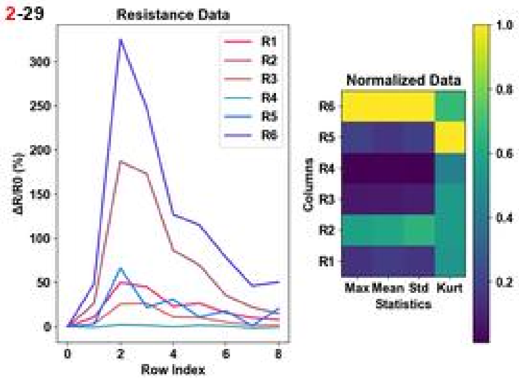

2-30
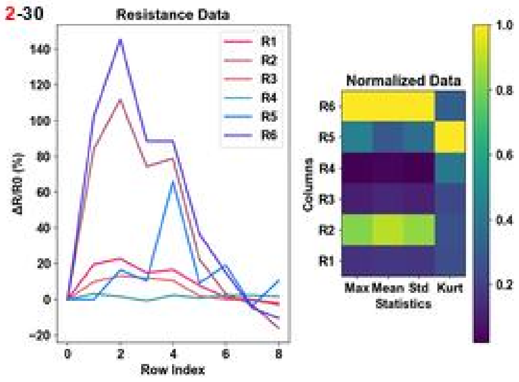

2-31
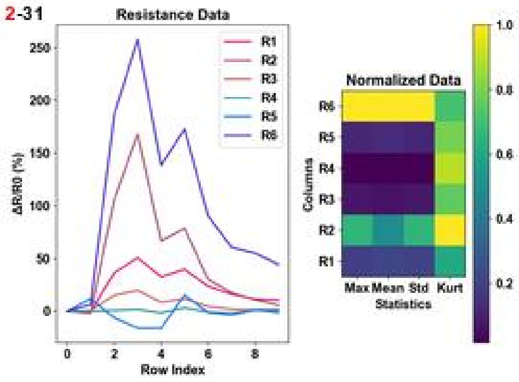

2-32
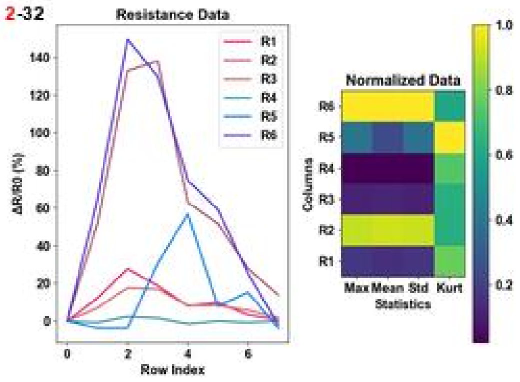

2-33
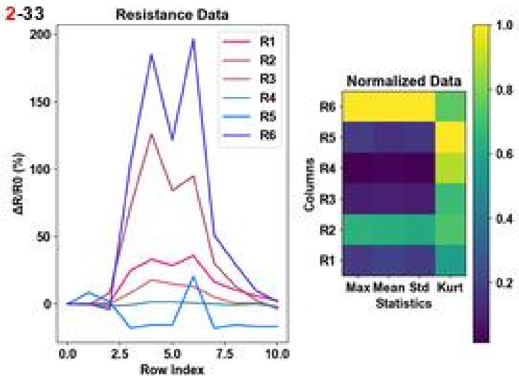

2-34
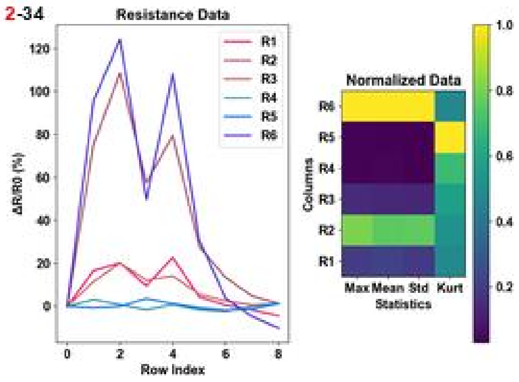

2-35
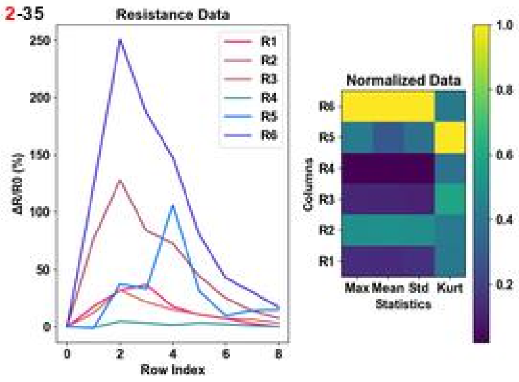

2-36
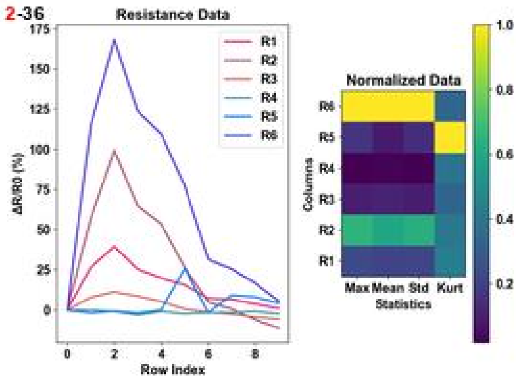

2-37
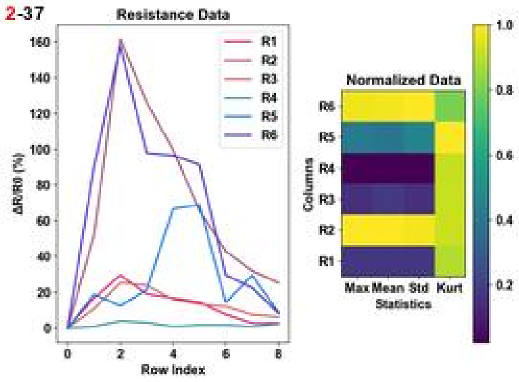

2-38
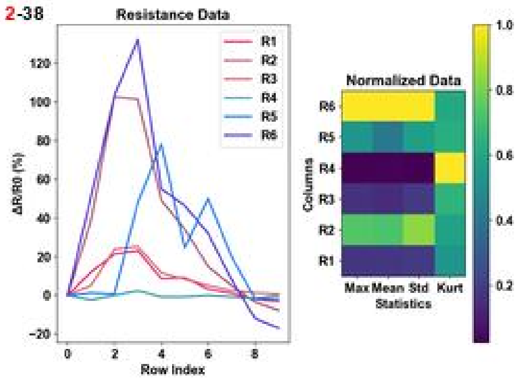

2-39
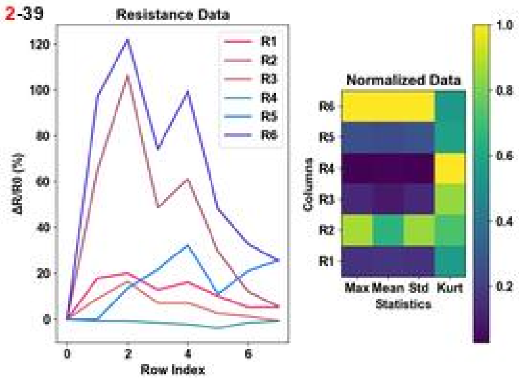

2-40
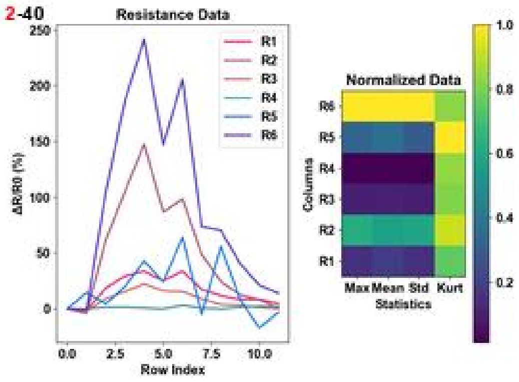

2-41
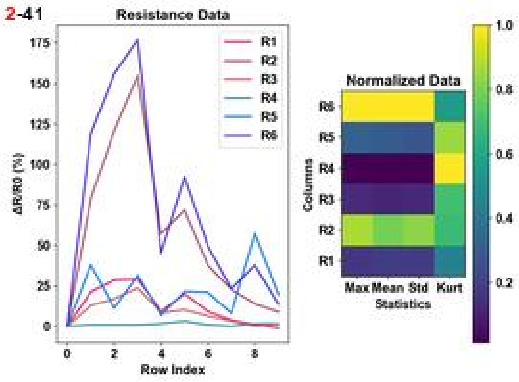

2-42
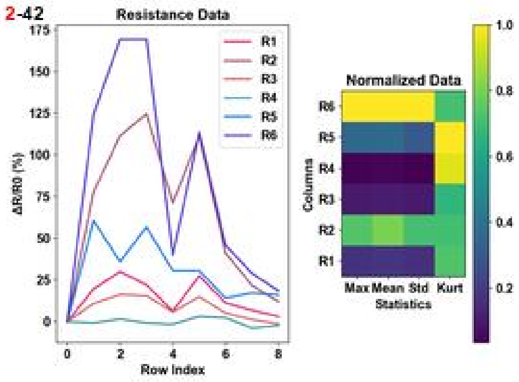

2-43
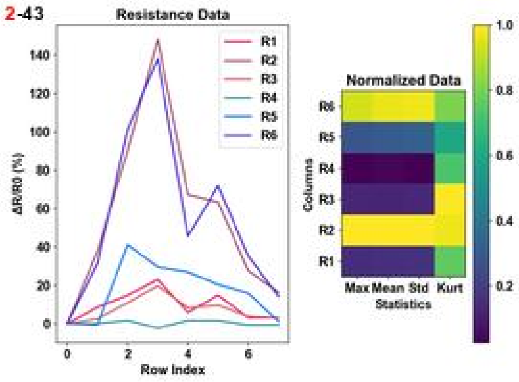

2-44
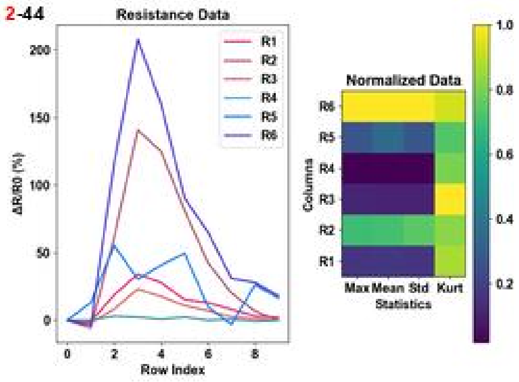

2-45
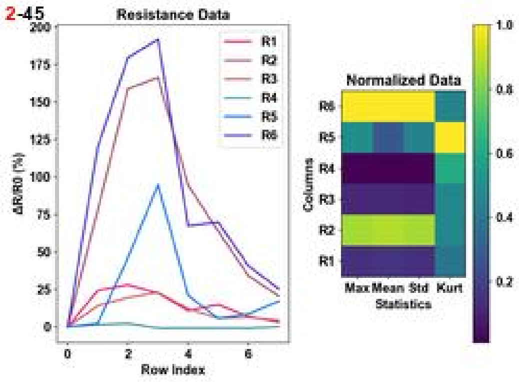

2-46
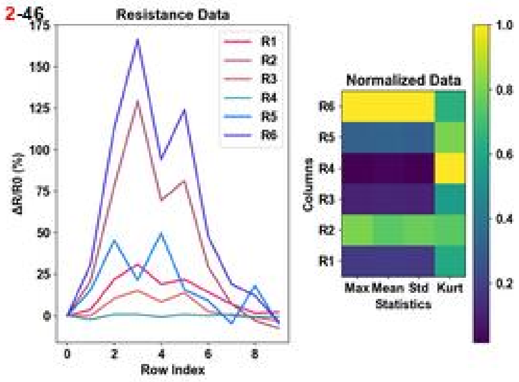

2-47
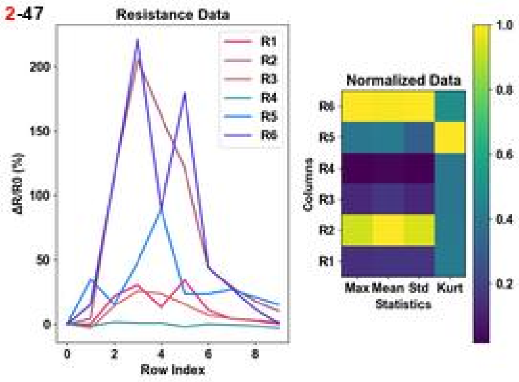

2-48
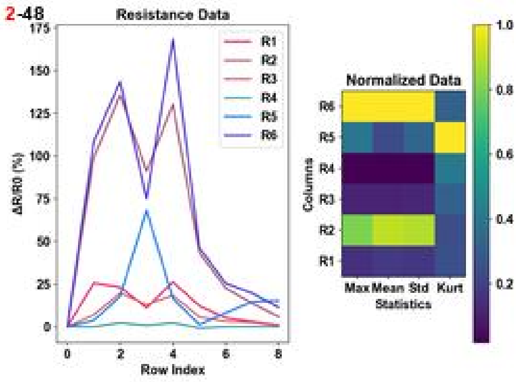

2-49
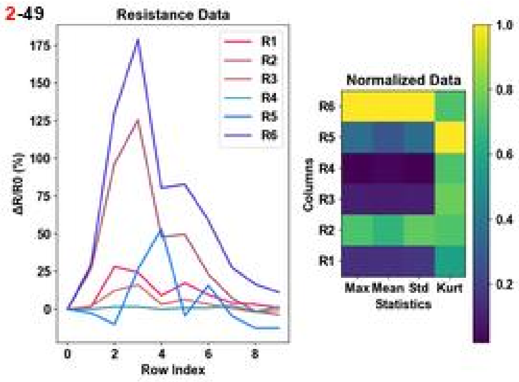

2-50
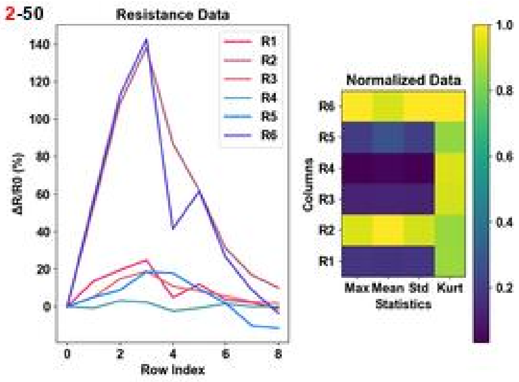

2-51
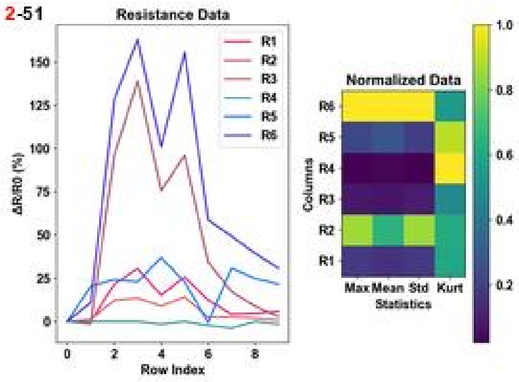

2-52
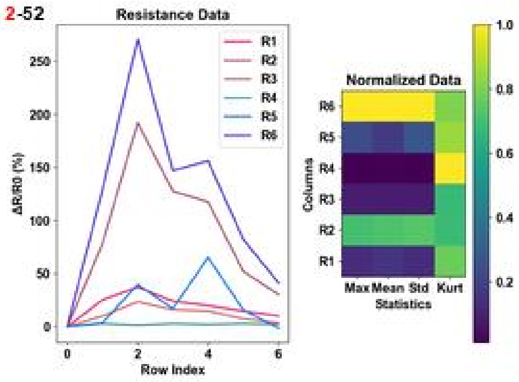

2-53
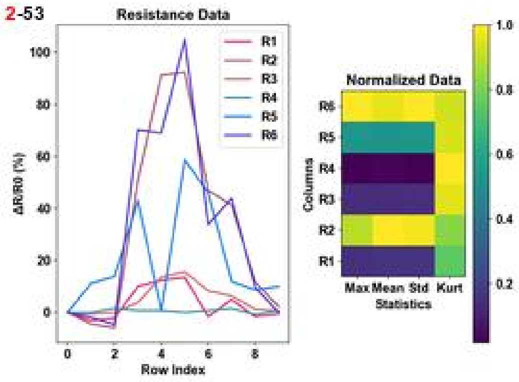

2-54
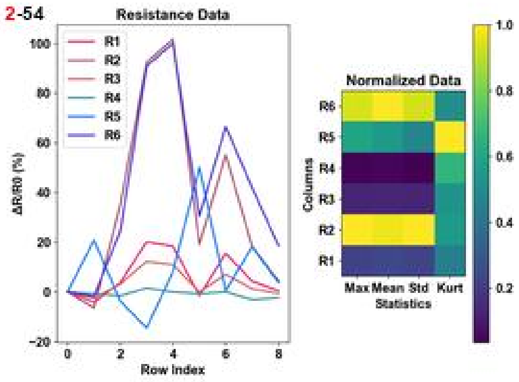

2-55
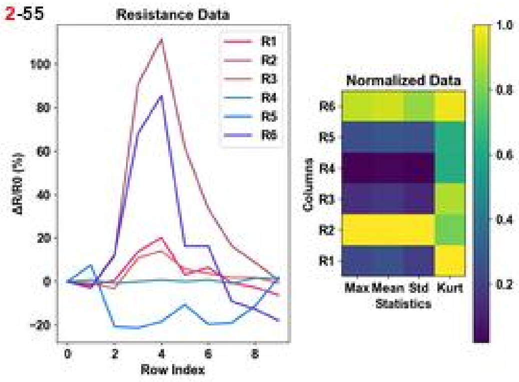

2-56
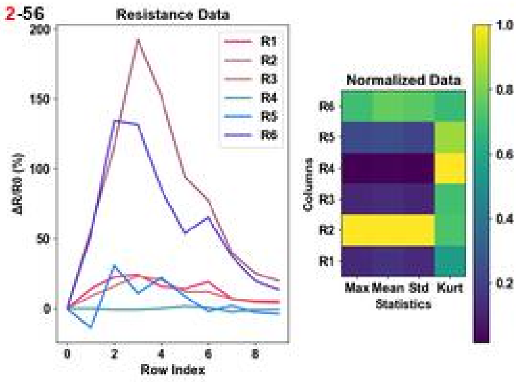

2-57
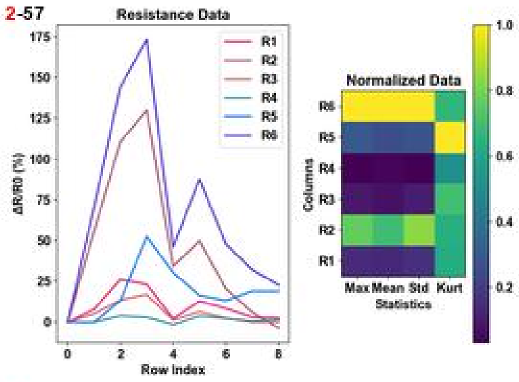

2-58
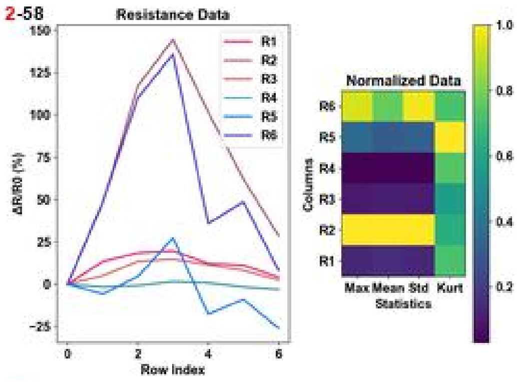

2-59
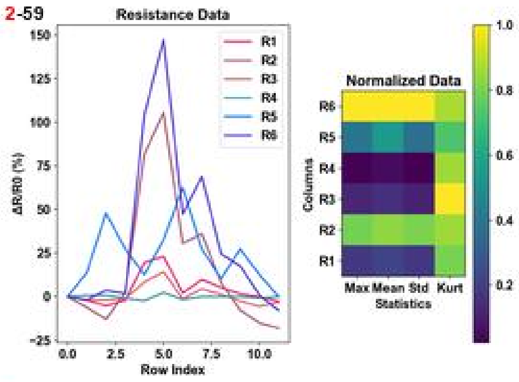

2-60
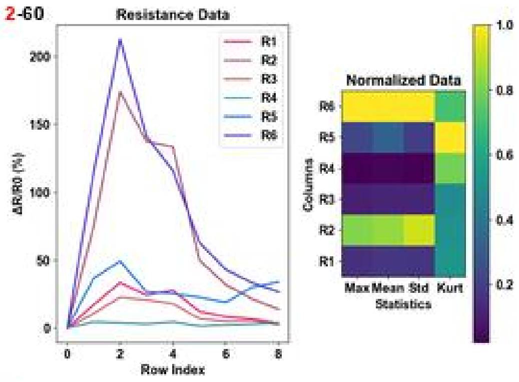

2-61
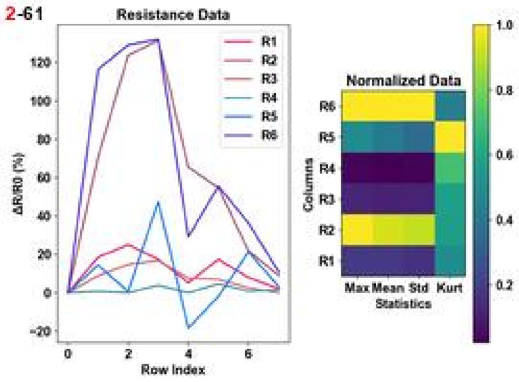

2-62
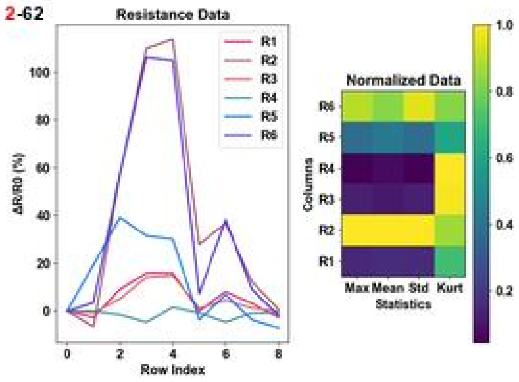

2-63
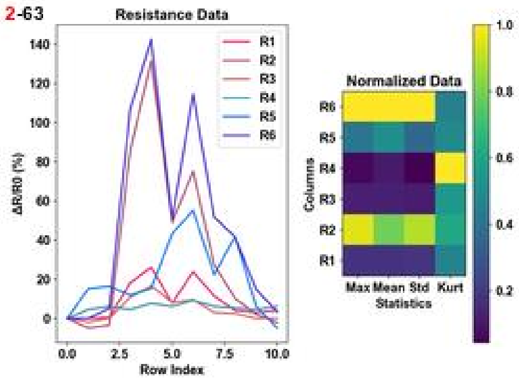

2-64
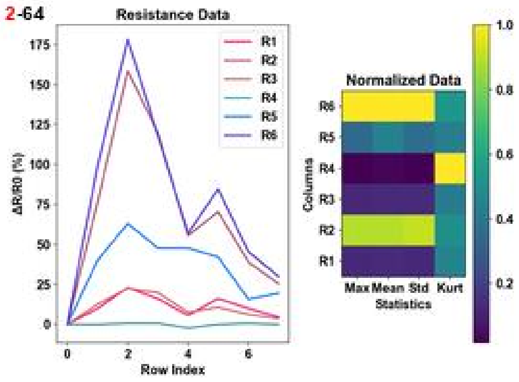

Pressure-sensitive data of type 2

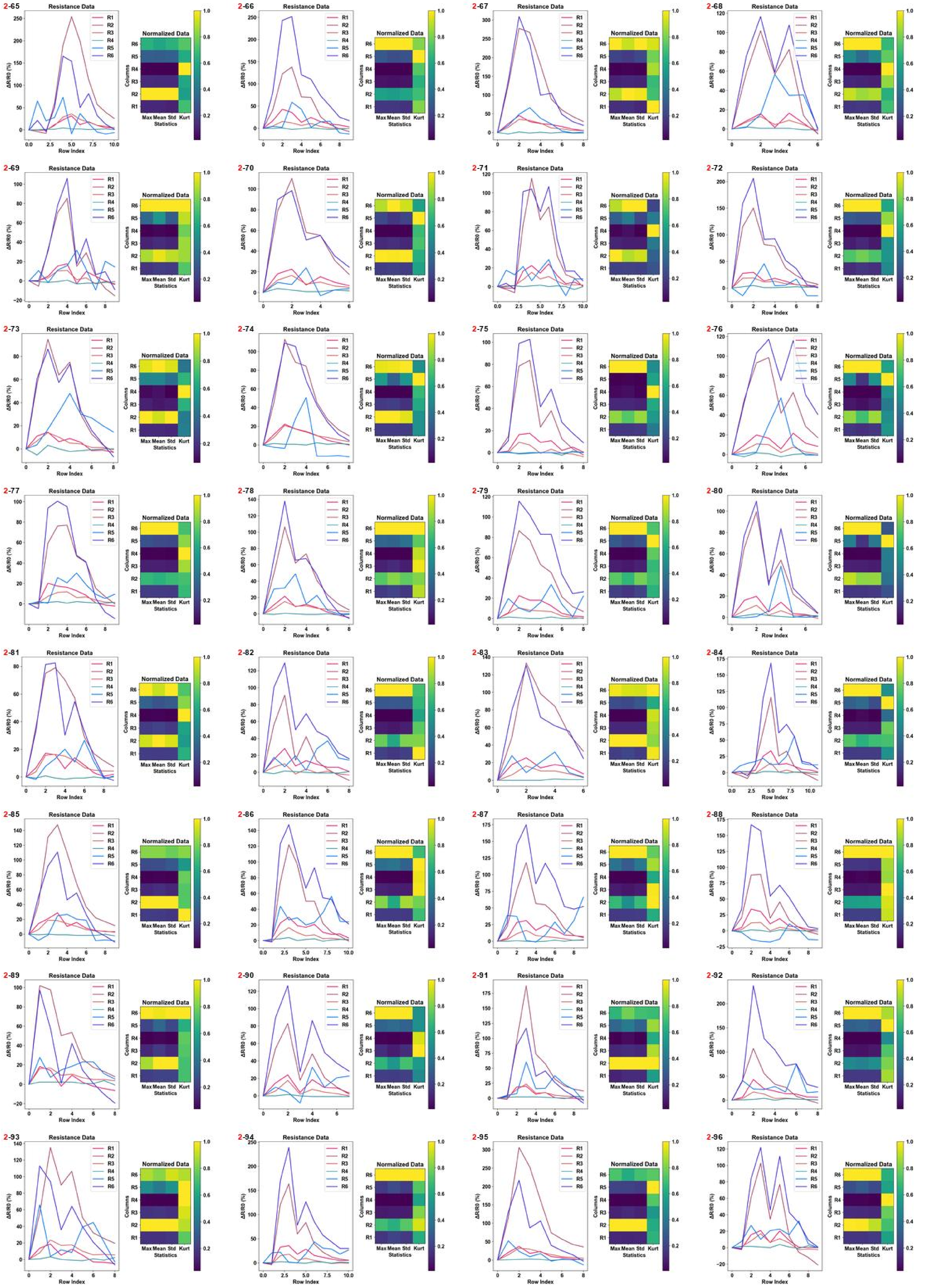

2-97
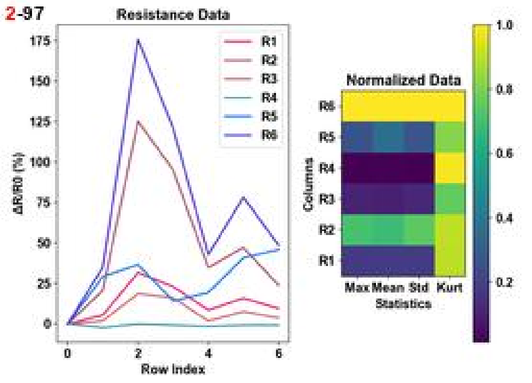

2-98
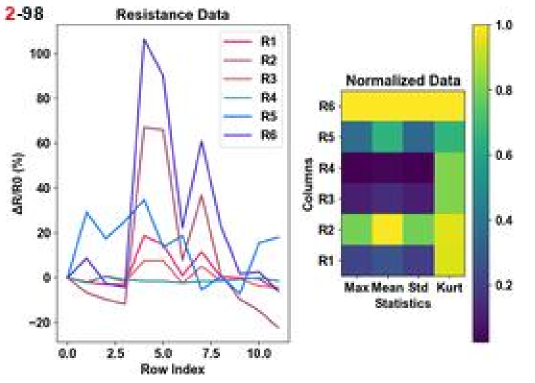

2-99
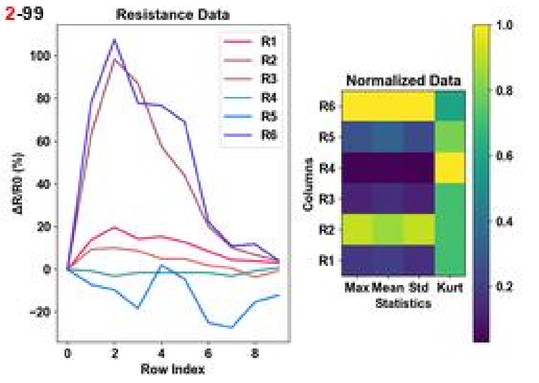

2-100
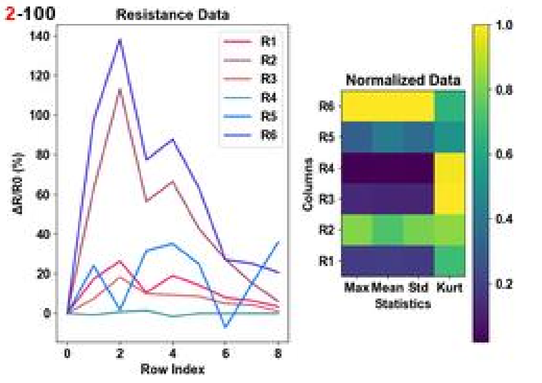

2-101
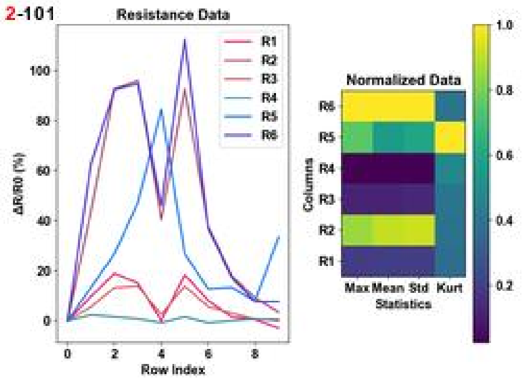

2-102
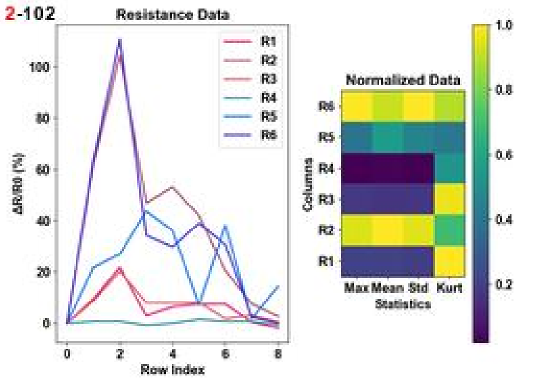

2-103
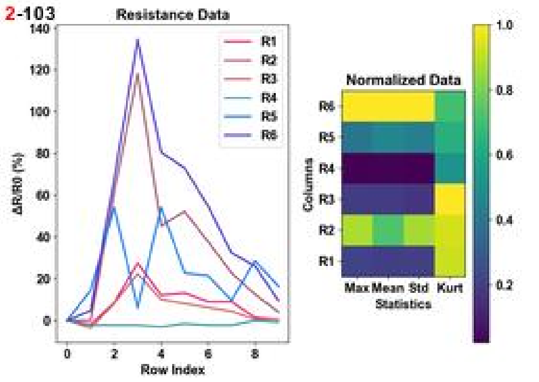

2-104
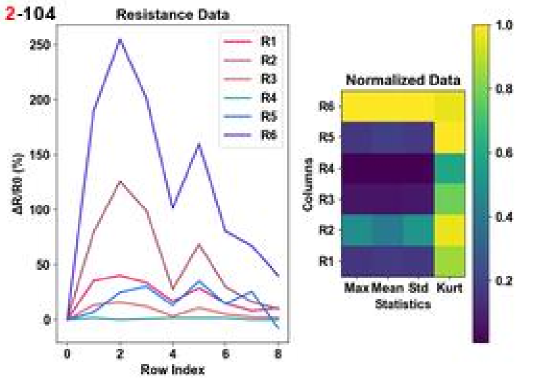

2-105
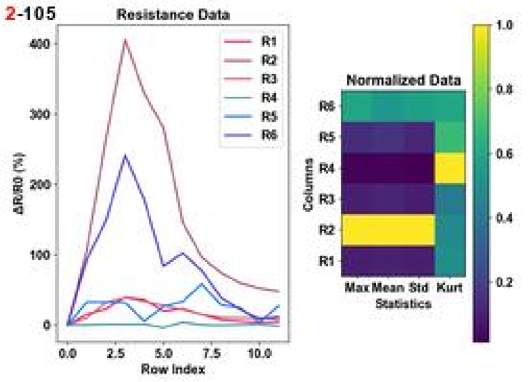

2-106
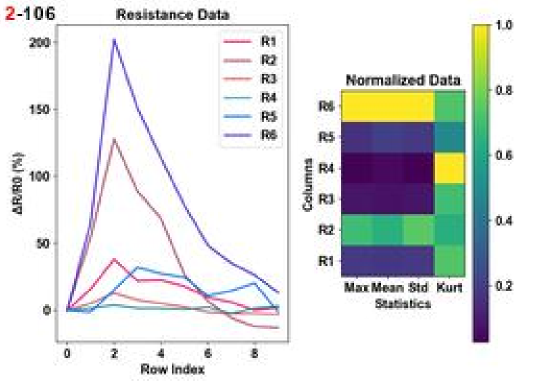

2-107
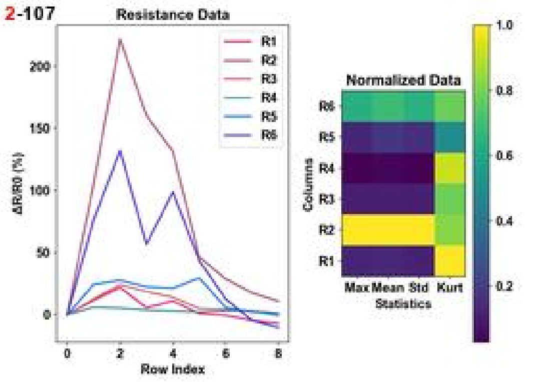

2-108
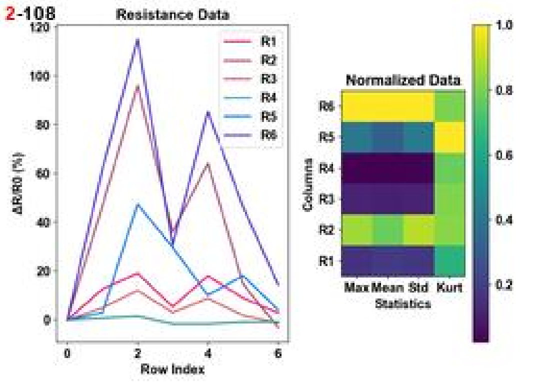

2-109
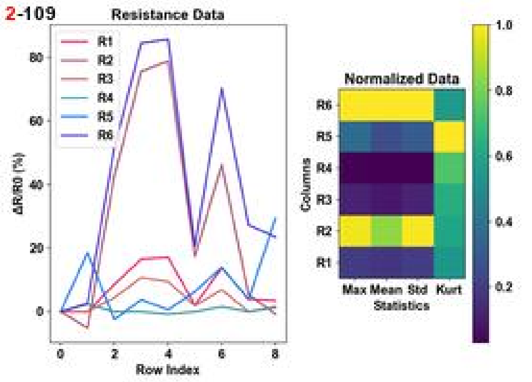

2-110
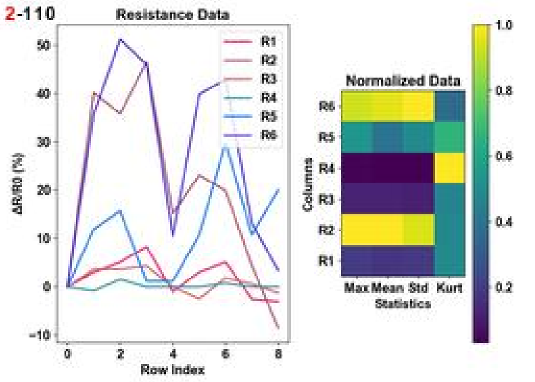

2-111
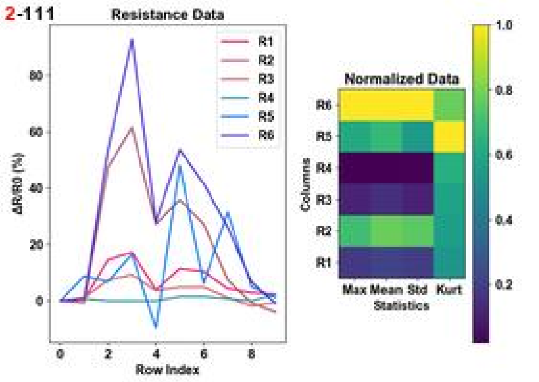

2-112
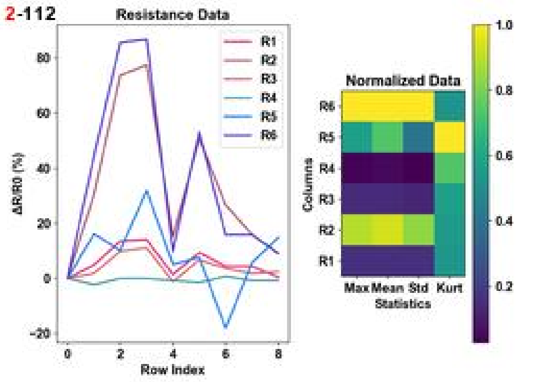

2-113
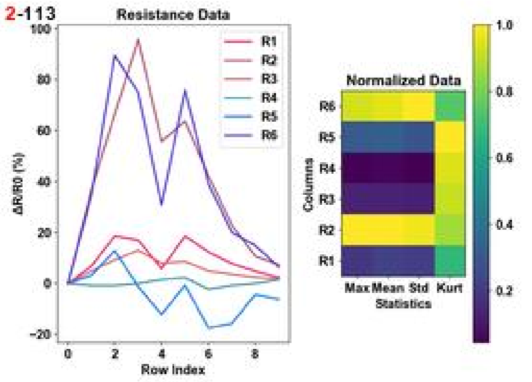

2-114
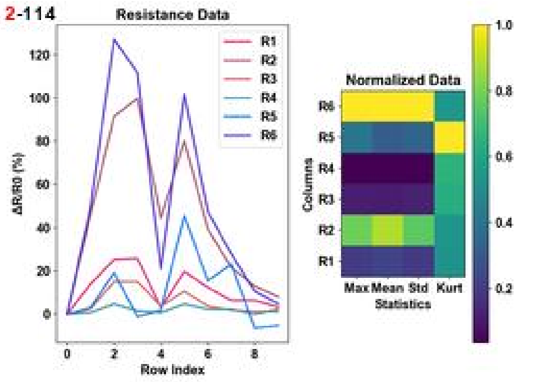

2-115
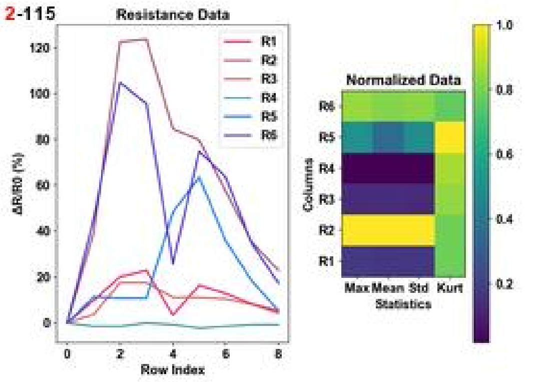

2-116
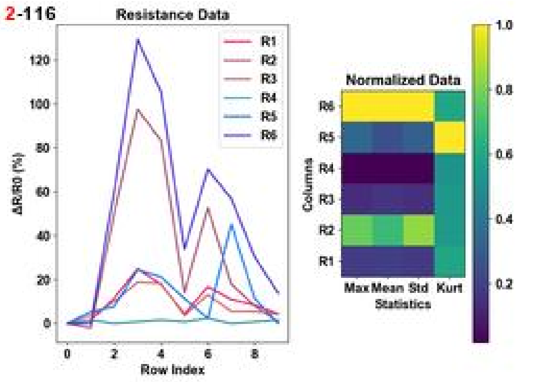

2-117
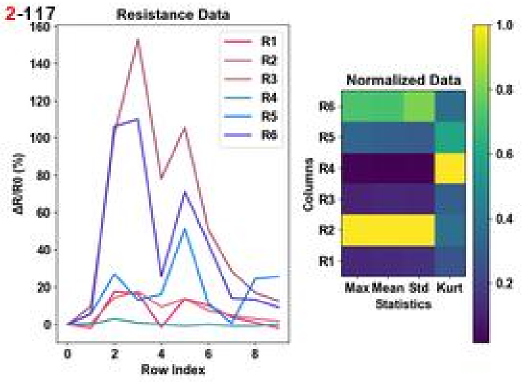

2-118
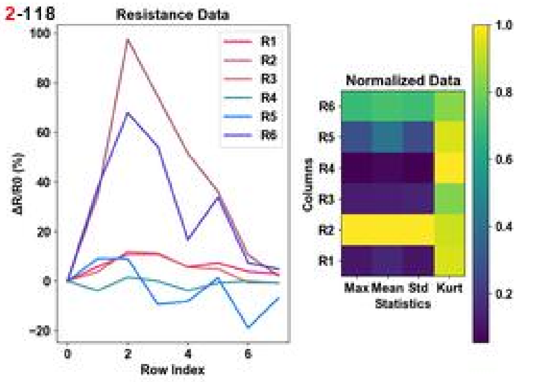

2-119
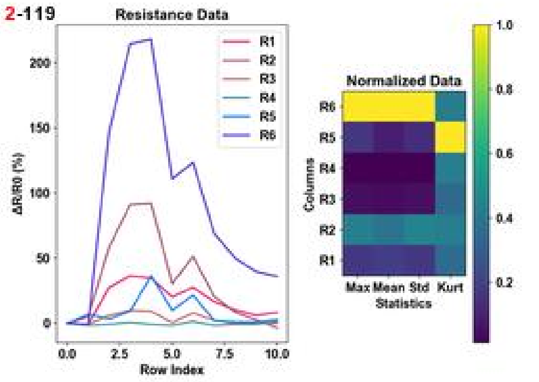

2-120
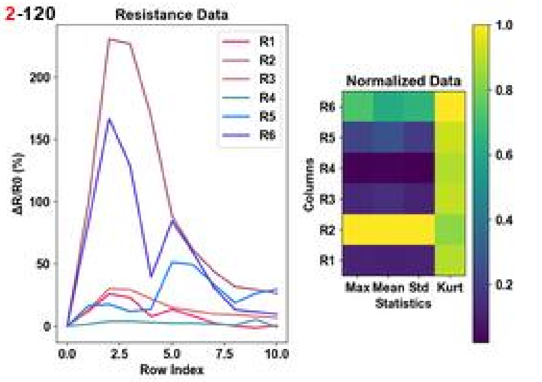

2-121
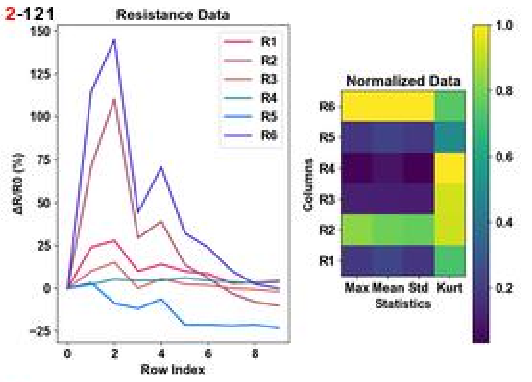

2-122
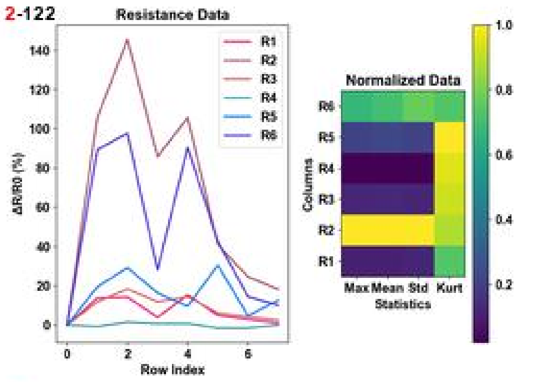

2-123
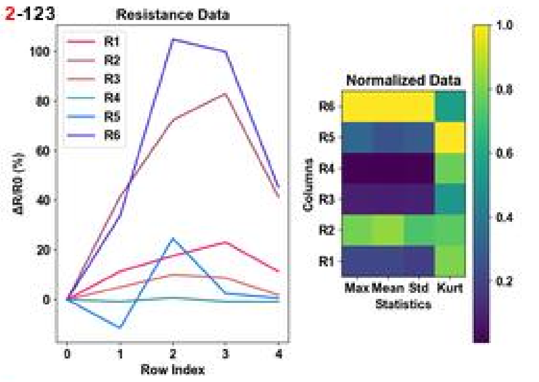

2-124
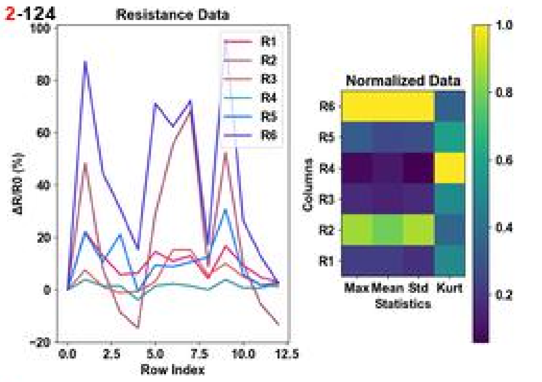

2-125
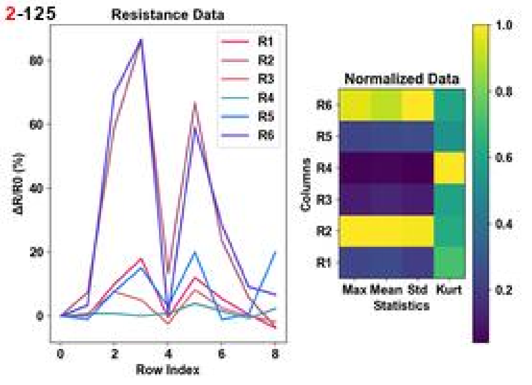

2-126
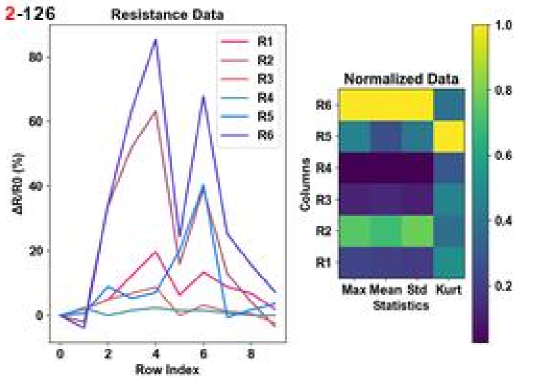

2-127
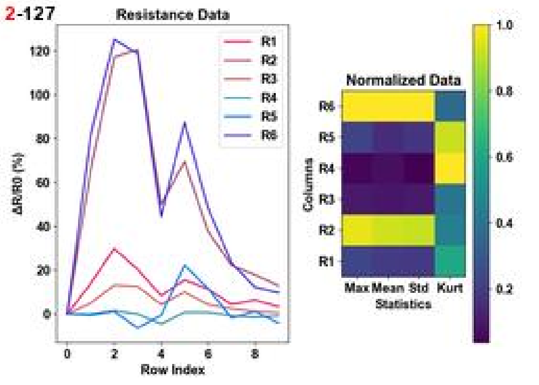

2-128
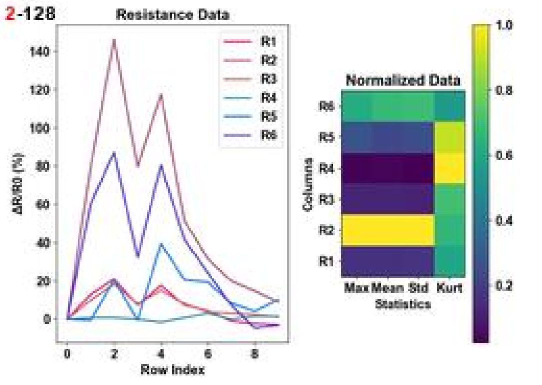

2-129
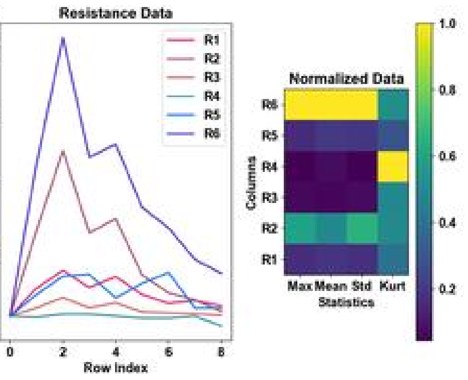

2-130
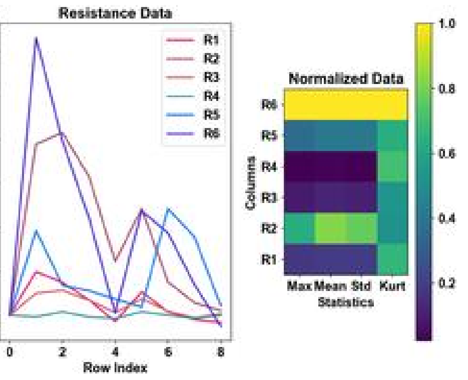

2-131
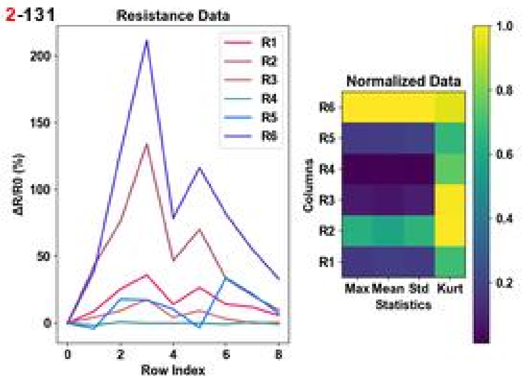

2-132
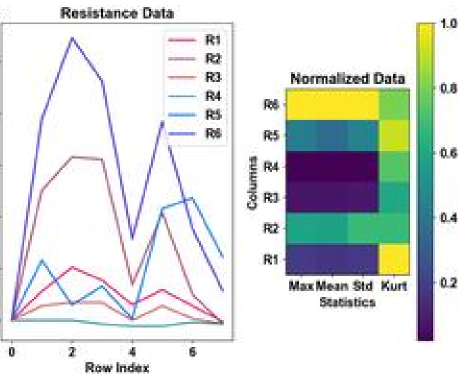

2-133
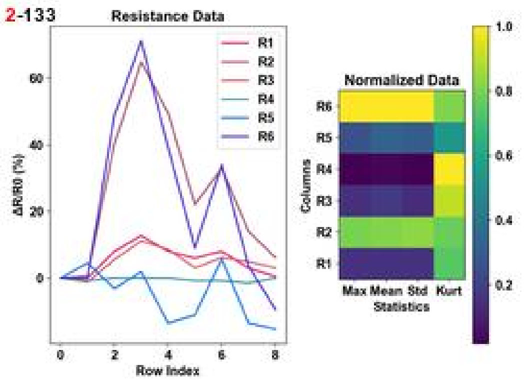

2-134
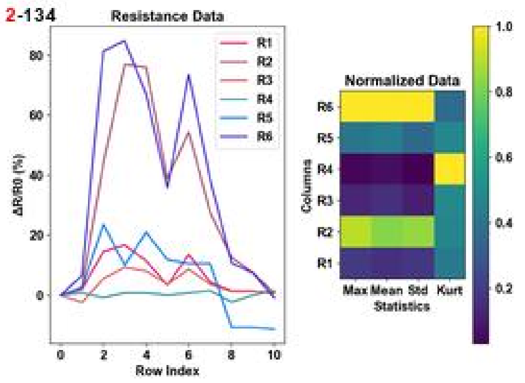

2-135
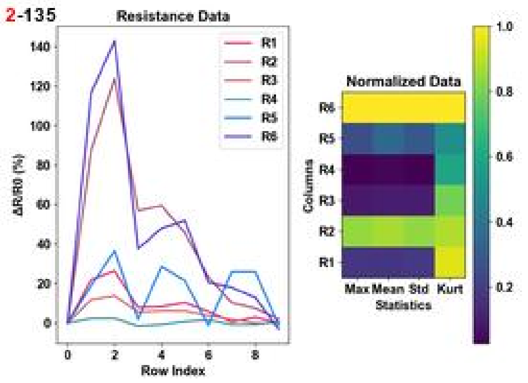

2-136
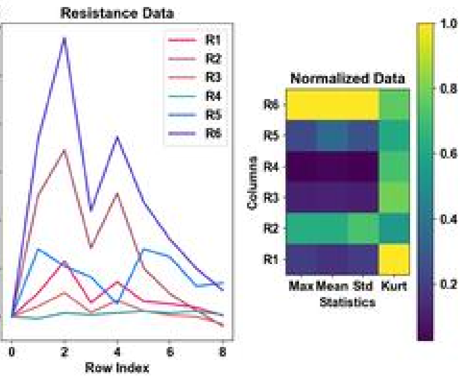

2-137
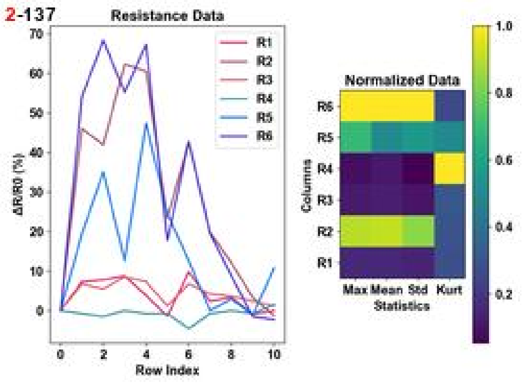

2-138
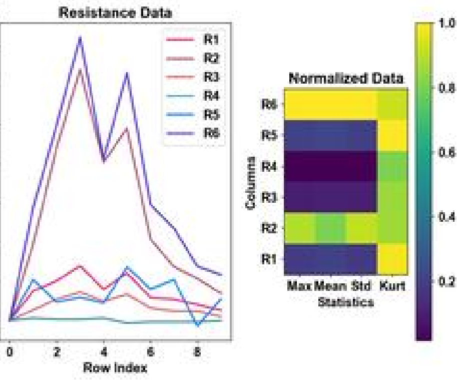

2-139
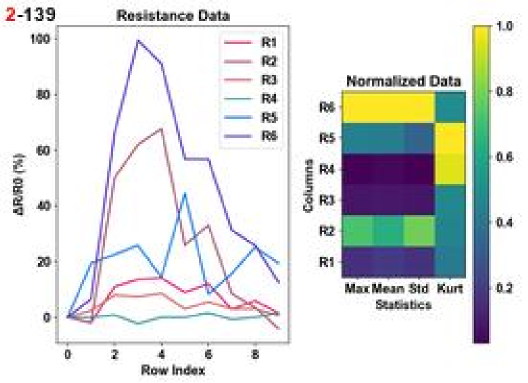

2-140
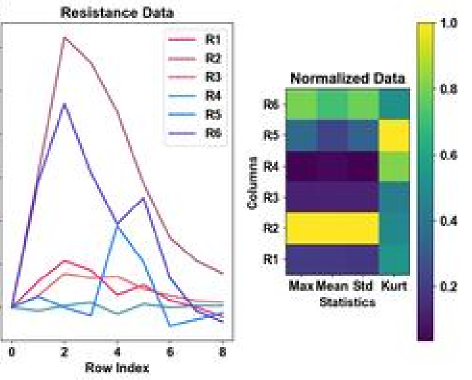

2-141
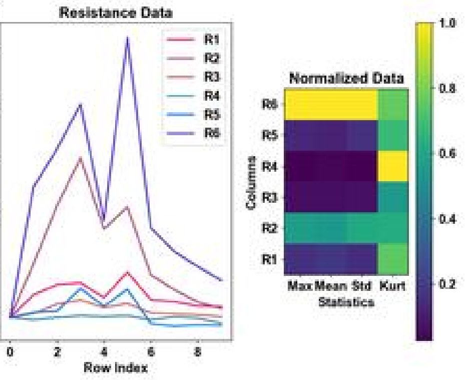

2-142
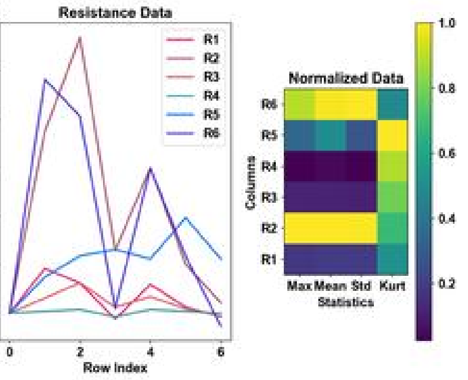

2-143
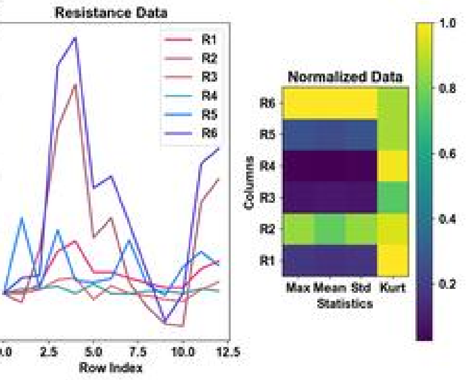

2-144
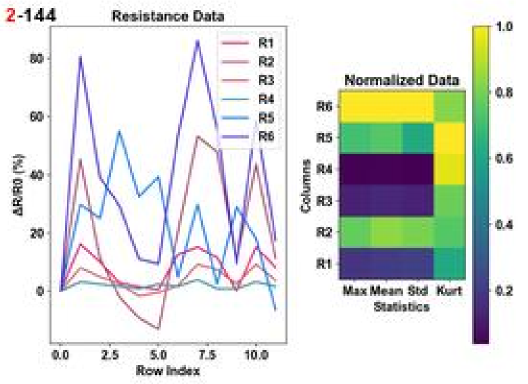

2-145
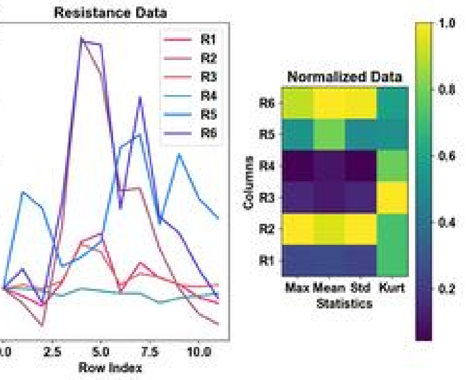

2-146
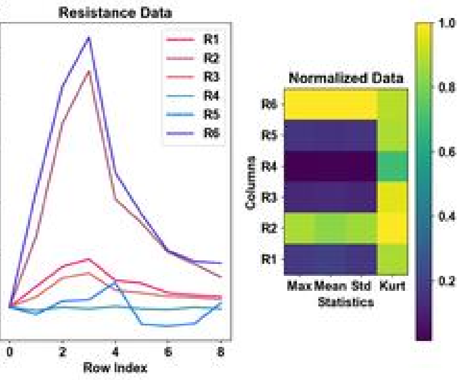

2-147
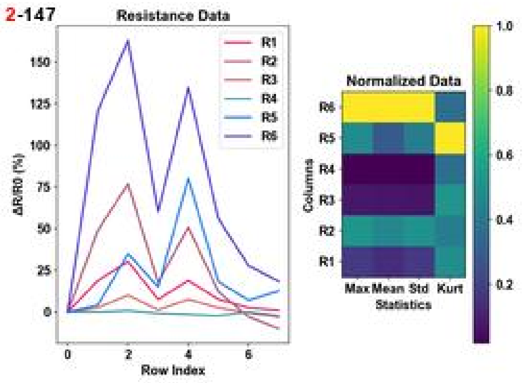

2-148
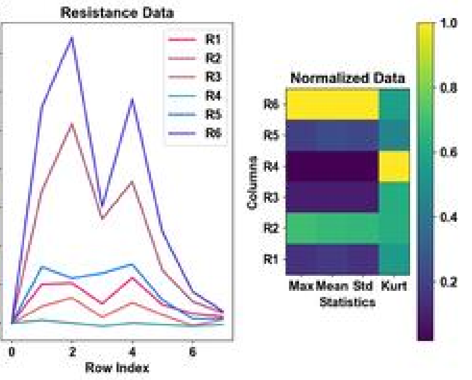

2-149
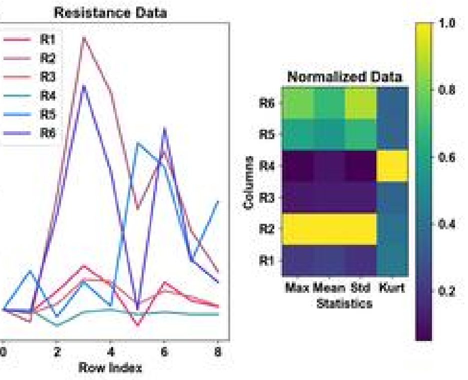

2-150
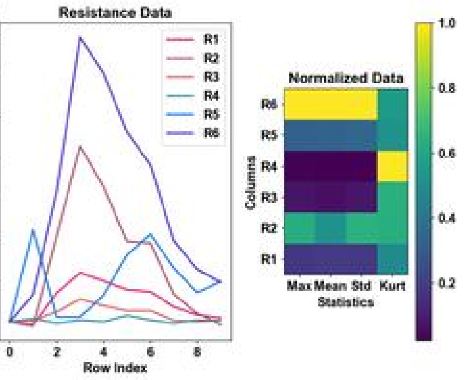

2-151
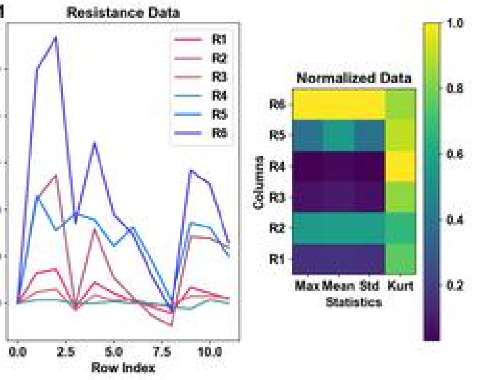

2-152
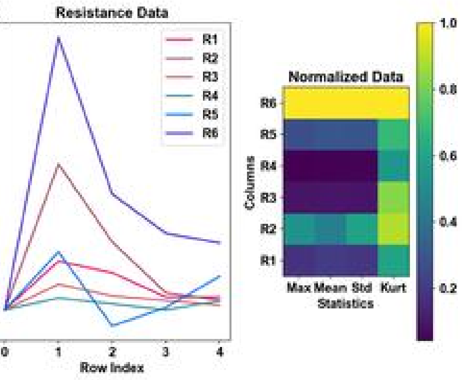

2-153
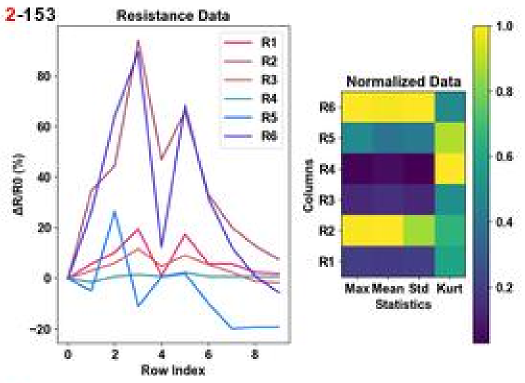

2-154
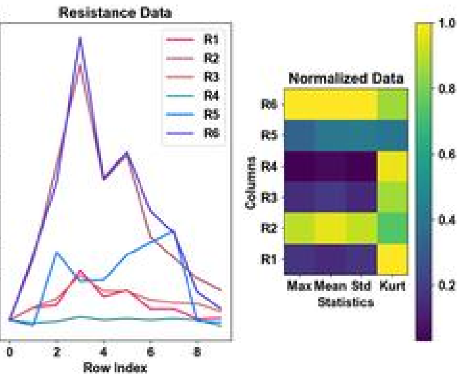

2-155
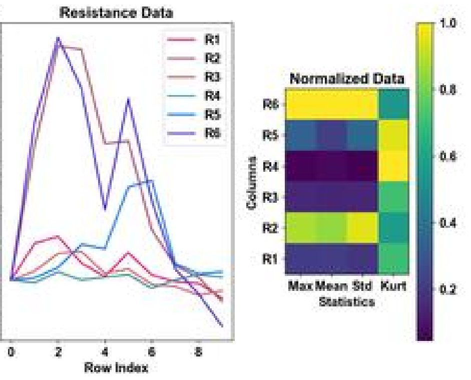

2-156
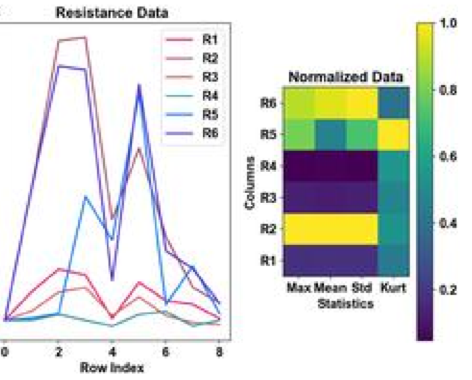

2-157
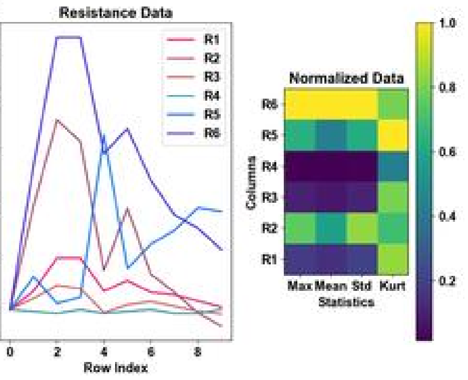

2-158
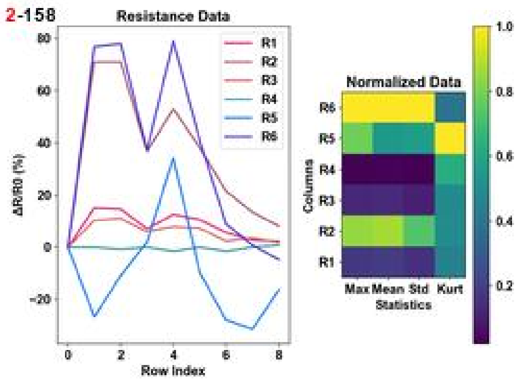

2-159
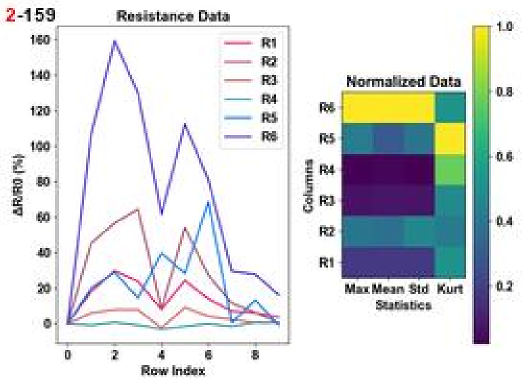

2-160
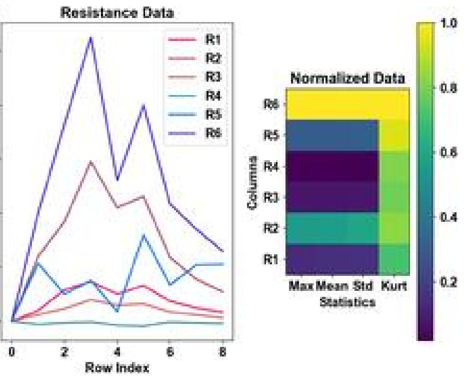

2-161

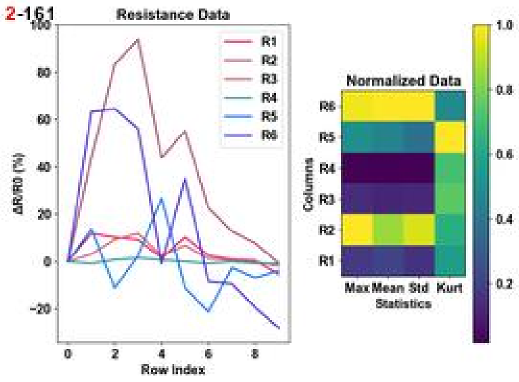

2-162

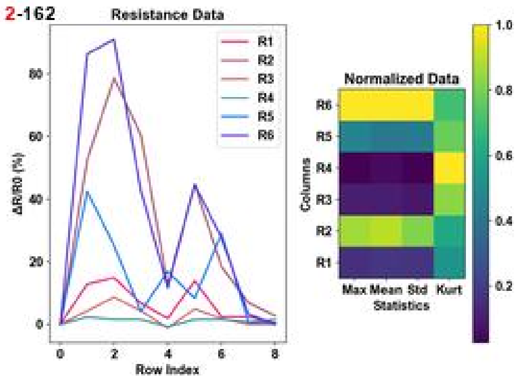

2-163

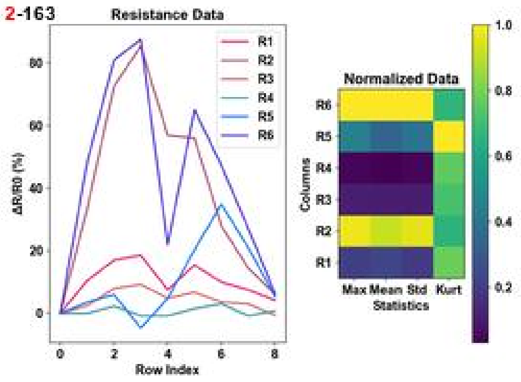

2-164

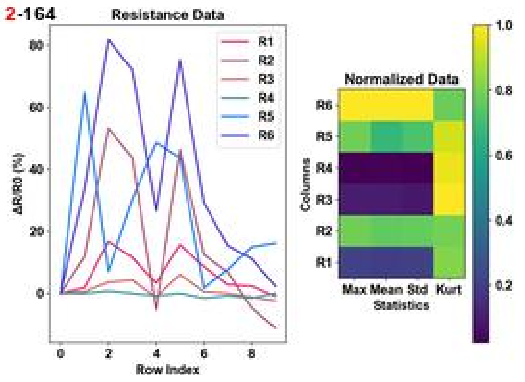

2-165

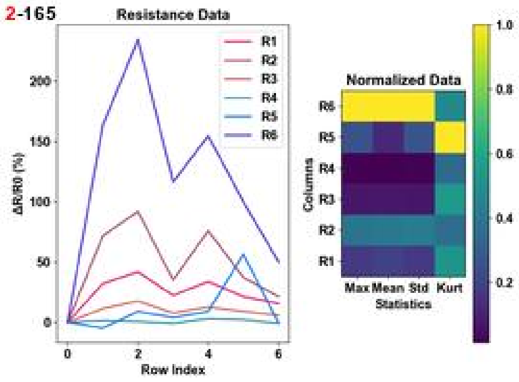

2-166

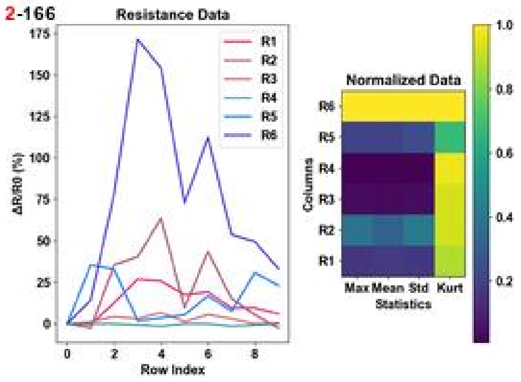

2-167

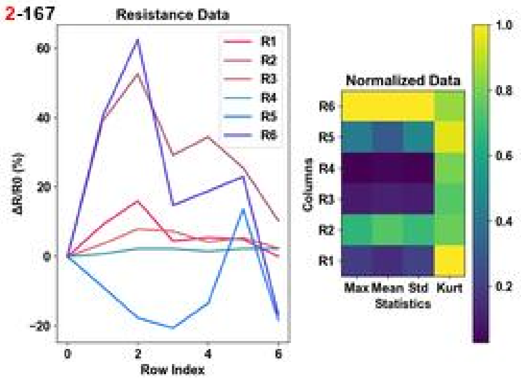

2-168

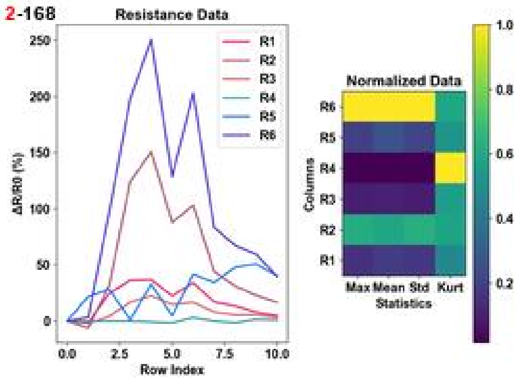

2-169

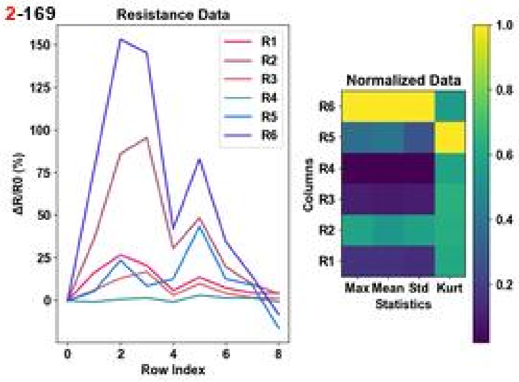

2-170

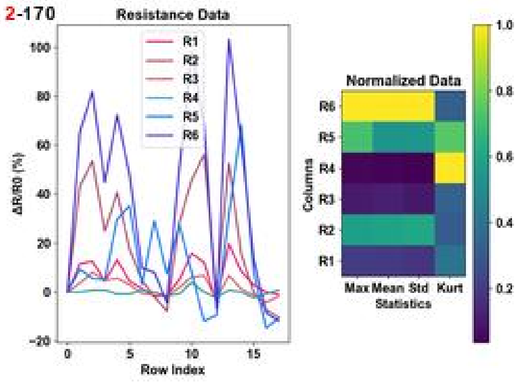

2-171

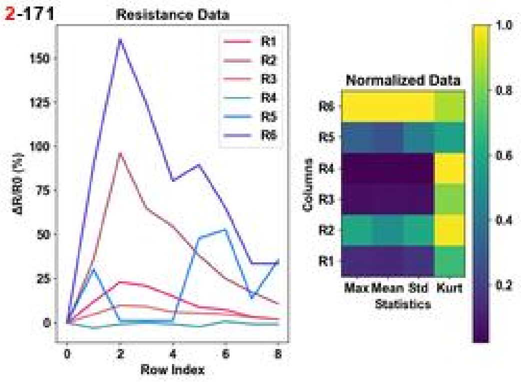

2-172

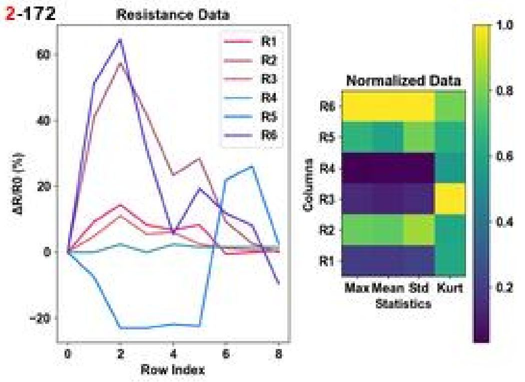

2-173

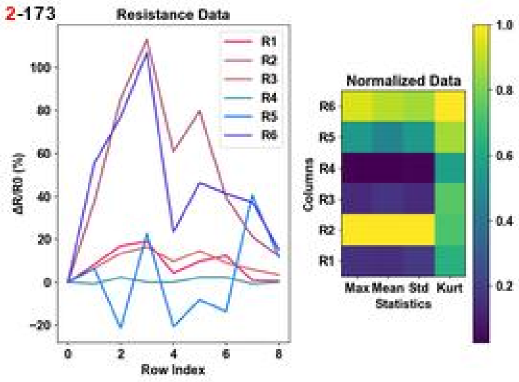

2-174

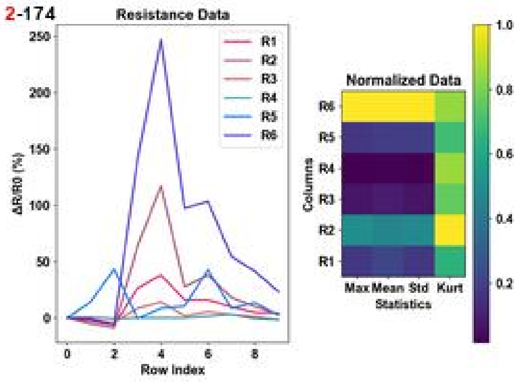

2-175

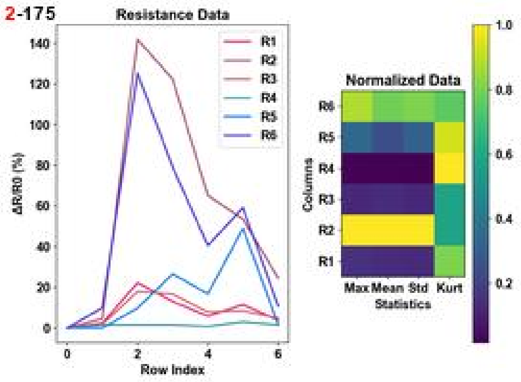

2-176

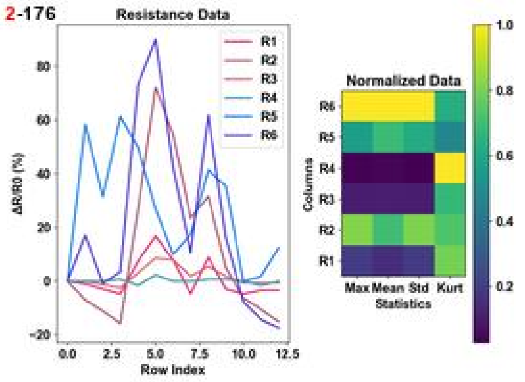

2-177

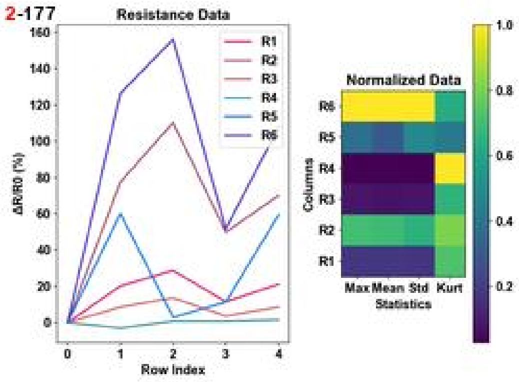

2-178

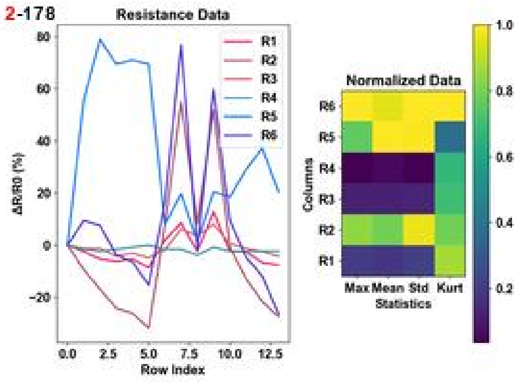

2-179

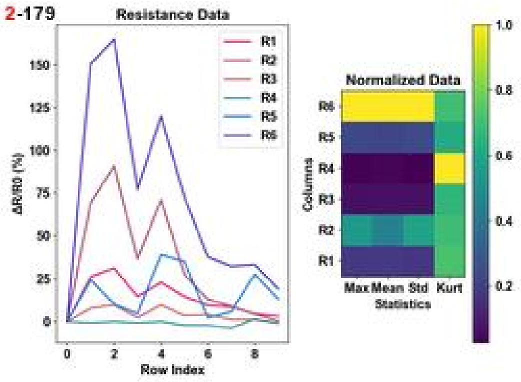

2-180

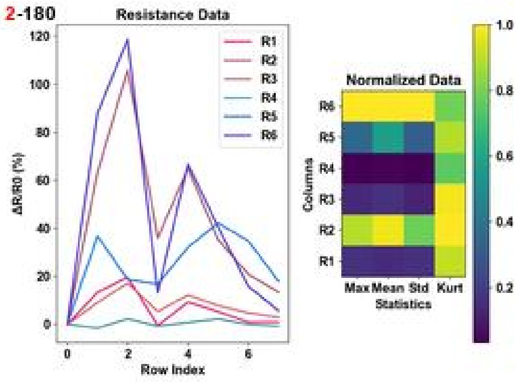

2-181

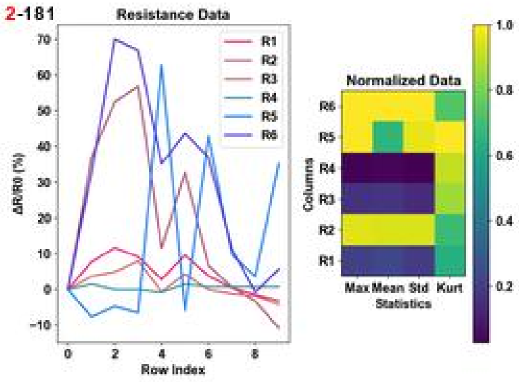

2-182

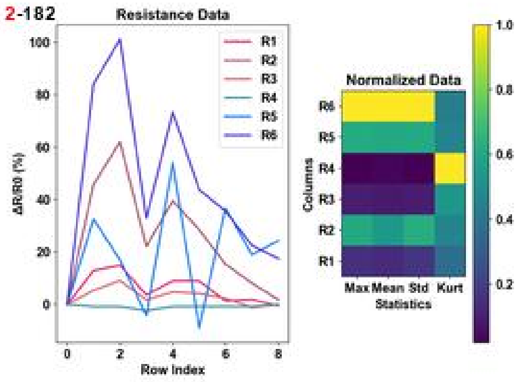

2-183

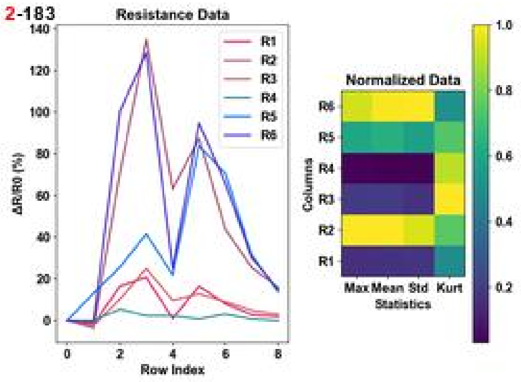

2-184

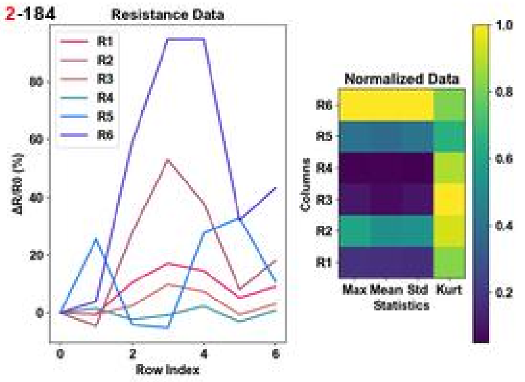

2-185

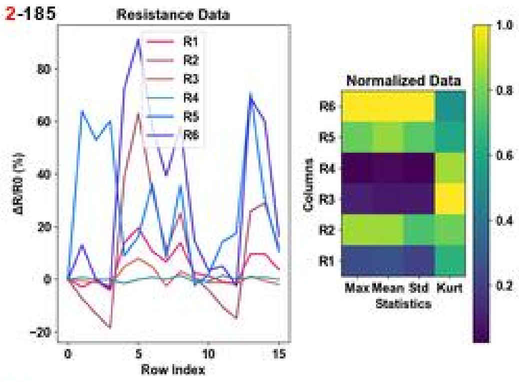

2-186

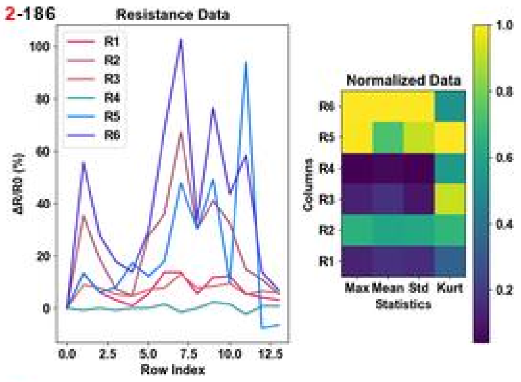

2-187

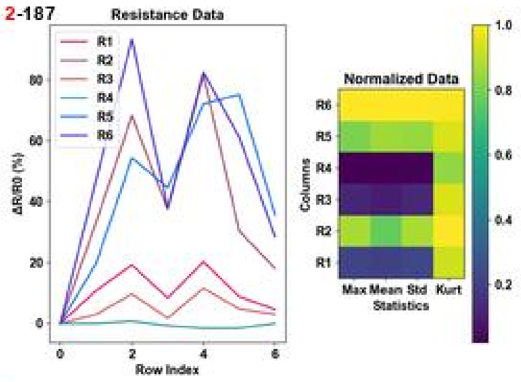

2-188

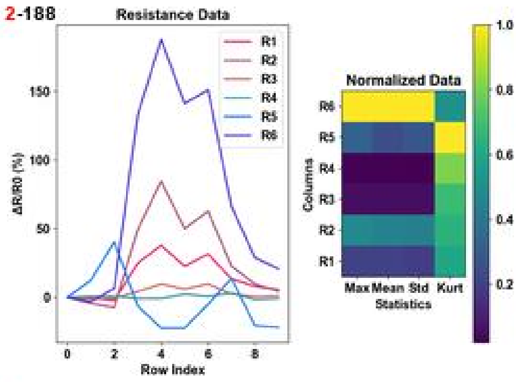

2-189

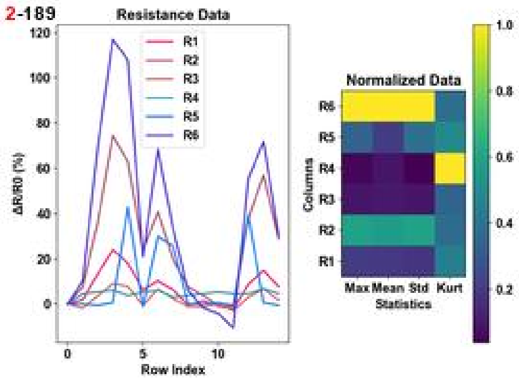

2-190

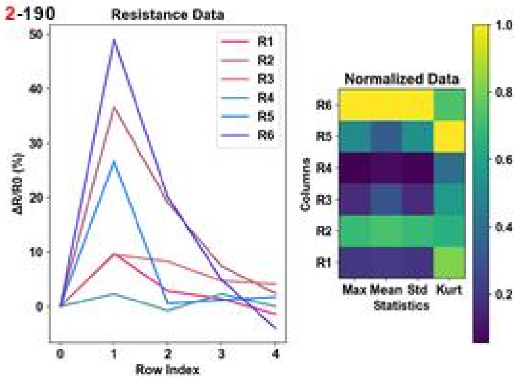

2-191

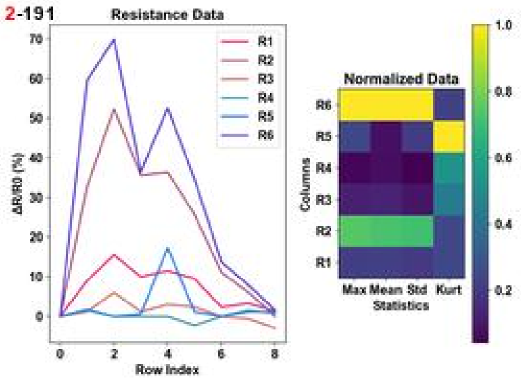

2-192

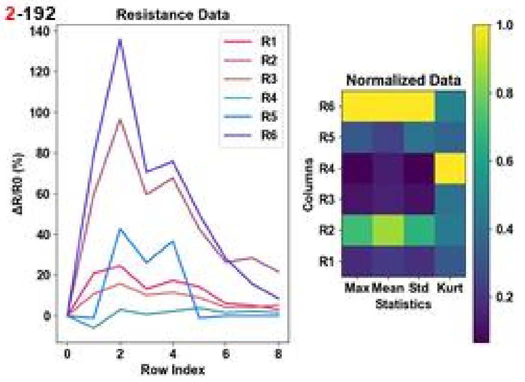

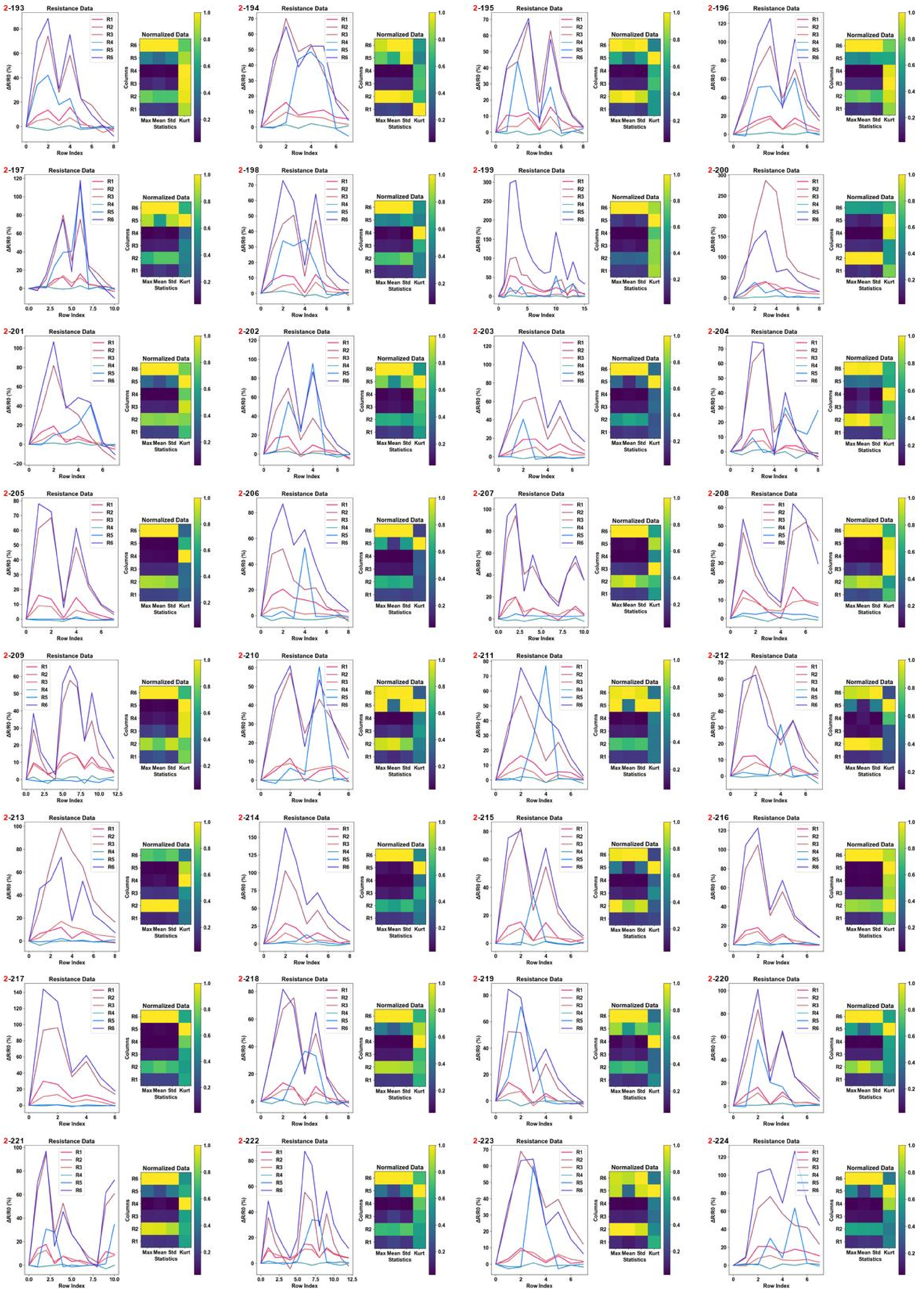
2-193
2-194
2-195
2-196
2-197
2-198
2-199
2-200
2-201
2-202
2-203
2-204
2-205
2-206
2-207
2-208
2-209
2-210
2-211
2-212
2-213
2-214
2-215
2-216
2-217
2-218
2-219
2-220
2-221
2-222
2-223
2-224
Resistance Data
Normalized Data
ΔR/R0 (%)
Row Index
Columns
Statistics
Max Mean Std Kurt
R1 R2 R3 R4 R5 R6

2-225

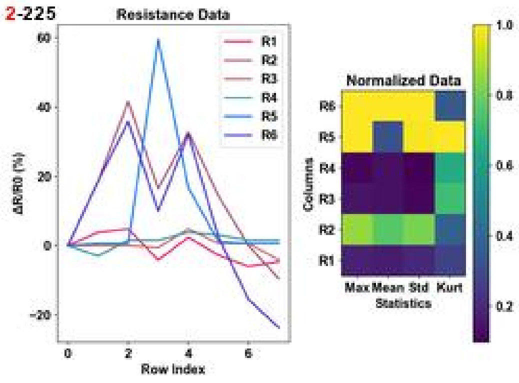

2-226

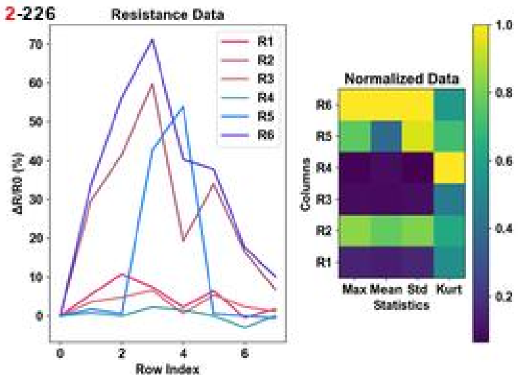

2-227

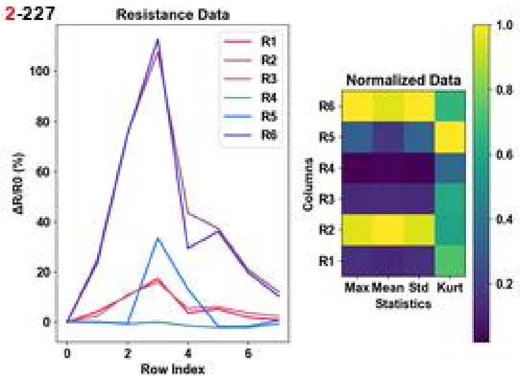

2-228

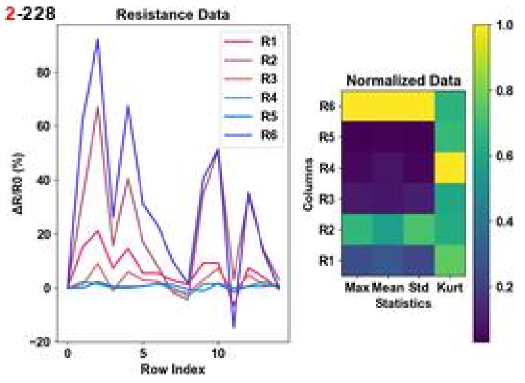

2-229

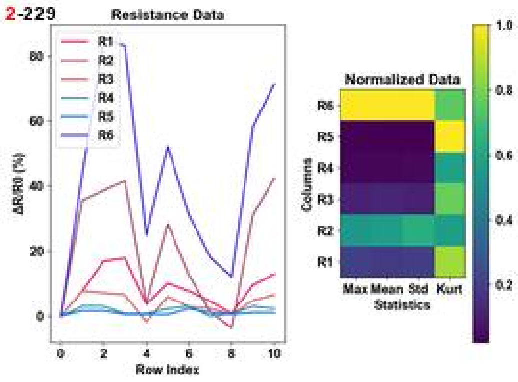

2-230

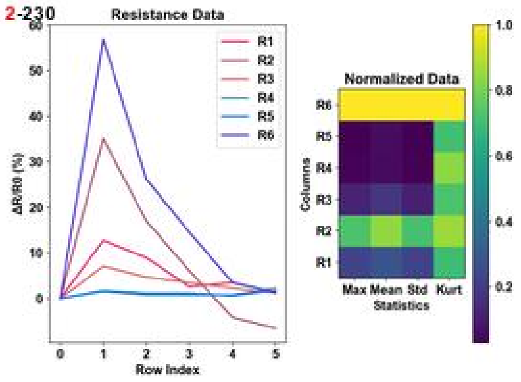

2-231

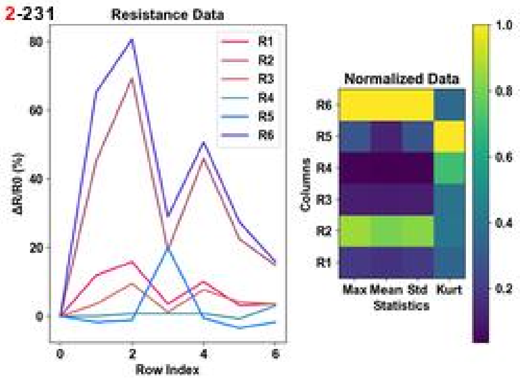

2-232

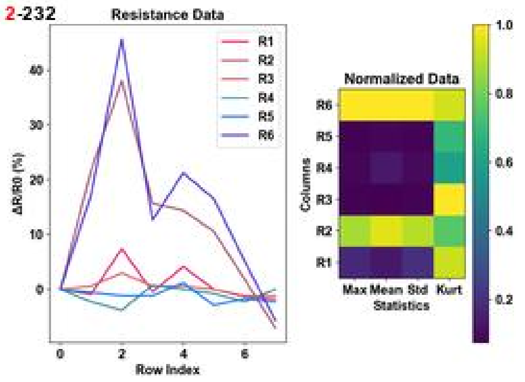

2-233

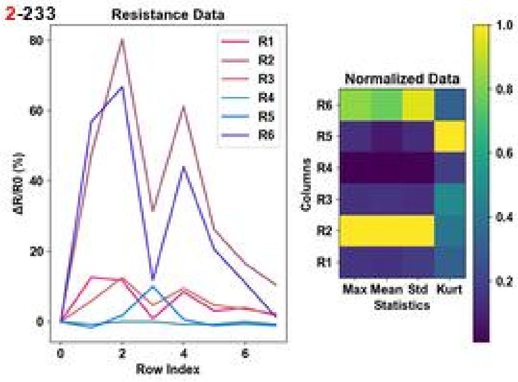

2-234

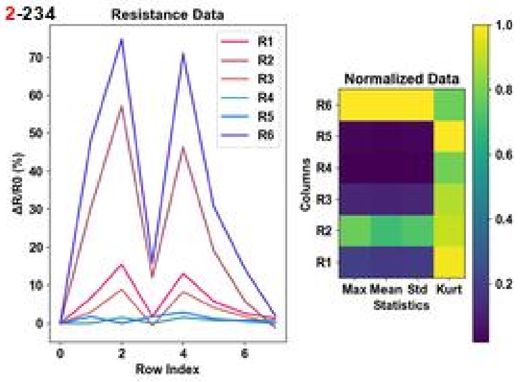

2-235

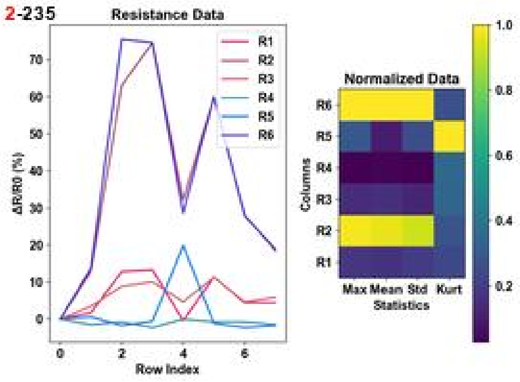

2-236

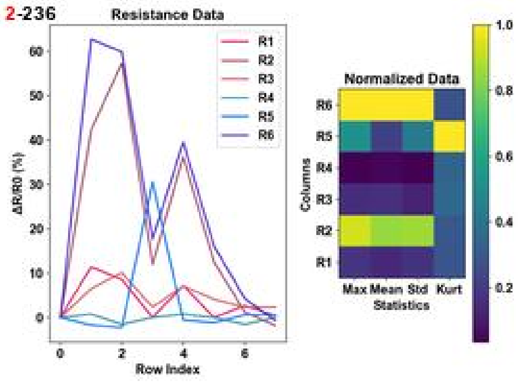

2-237

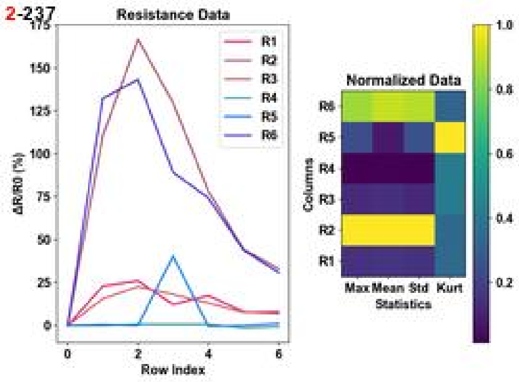

2-238

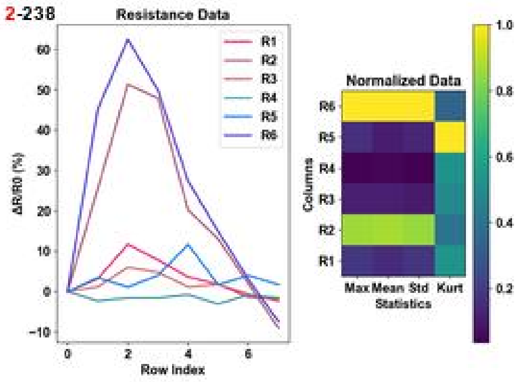

2-239

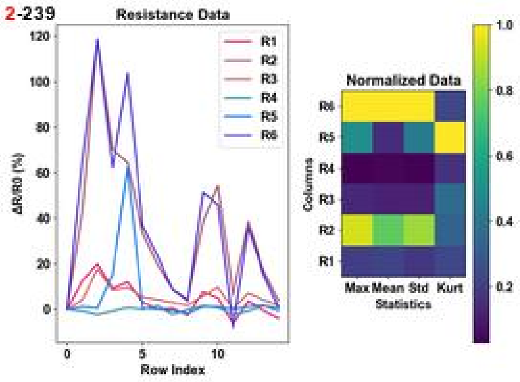

2-240

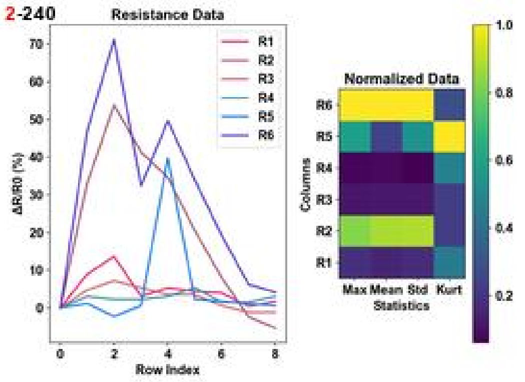

Pressure-sensitive data of type 3

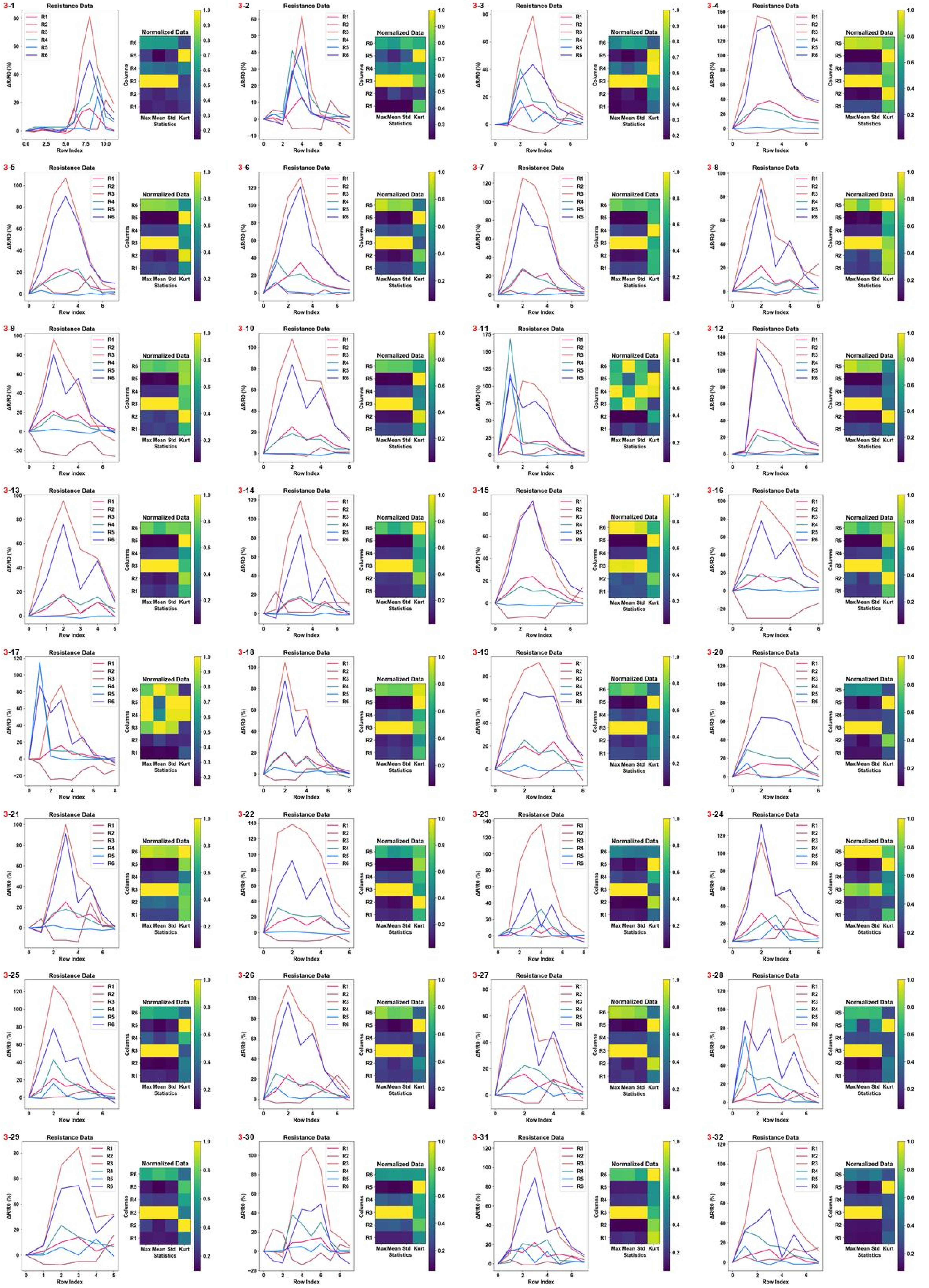

3-33
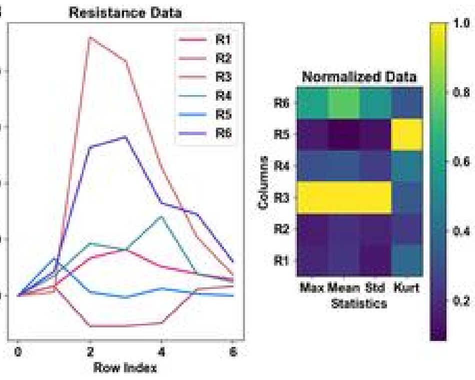

3-34
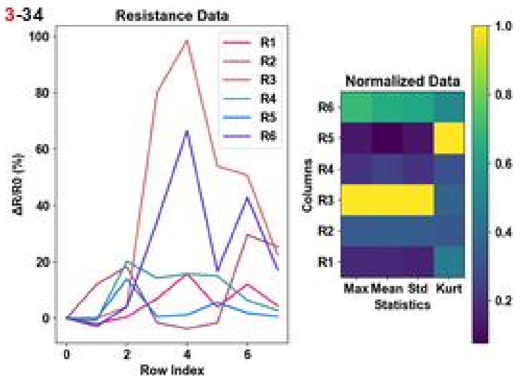

3-35
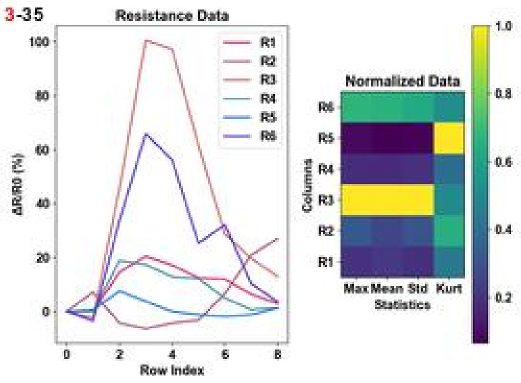

3-36
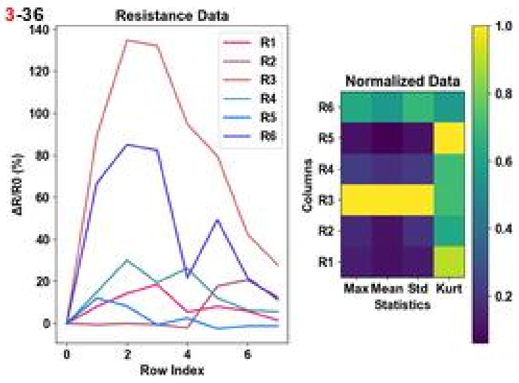

3-37
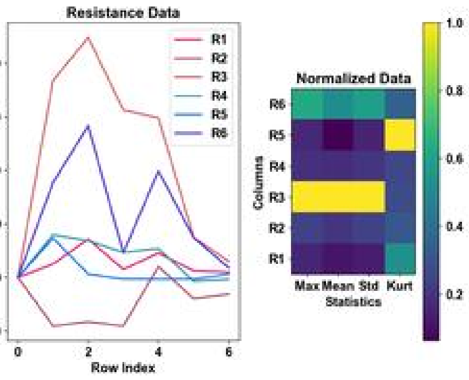

3-38
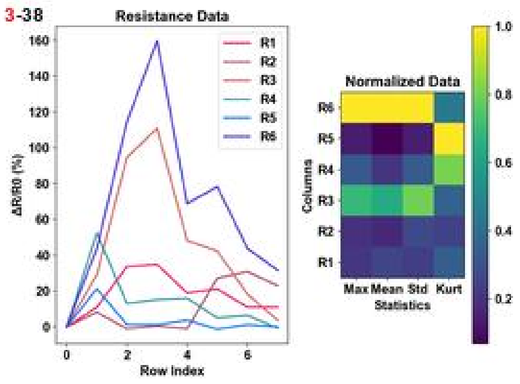

3-39
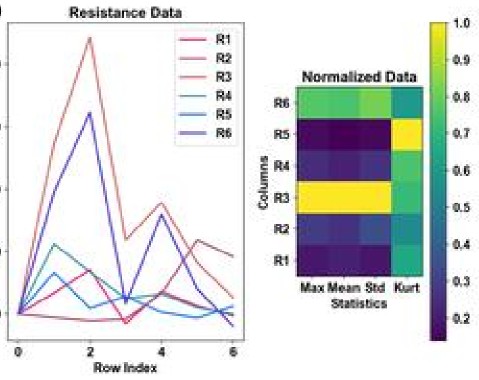

3-40
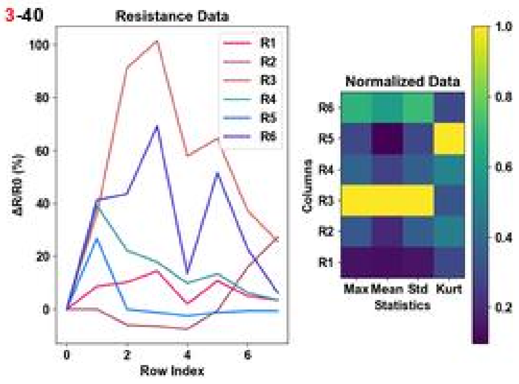

3-41
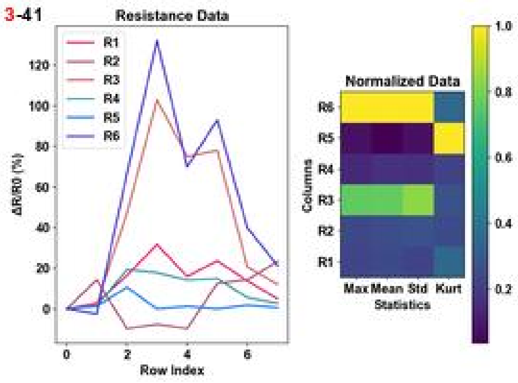

3-42
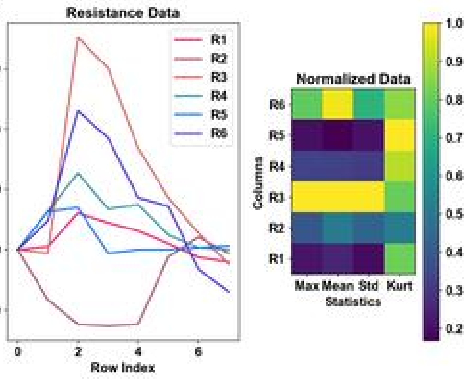

3-43
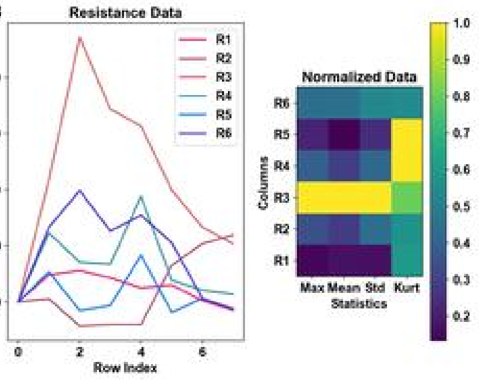

3-44
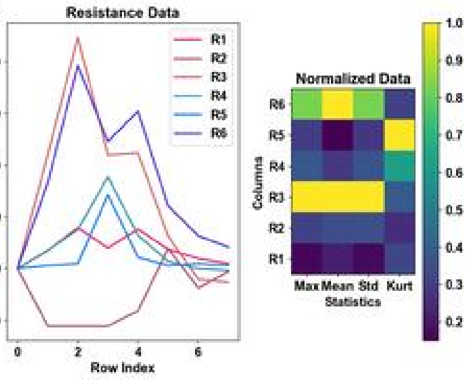

3-45
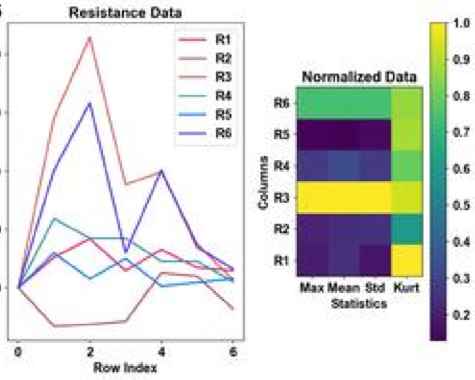

3-46
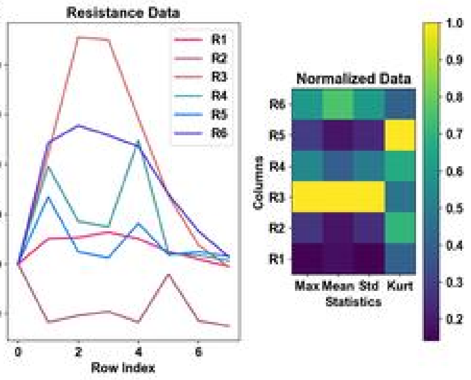

3-47
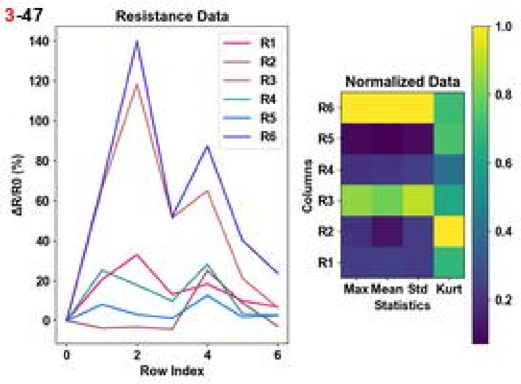

3-48
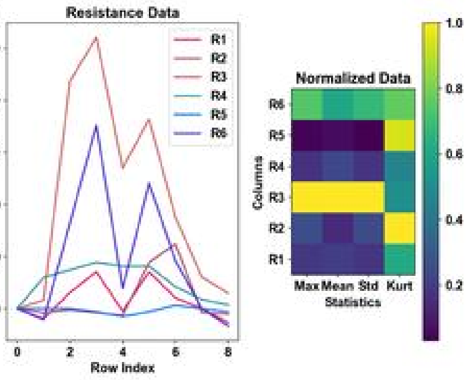

3-49
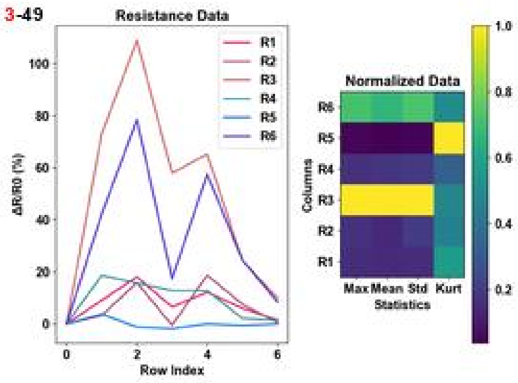

3-50
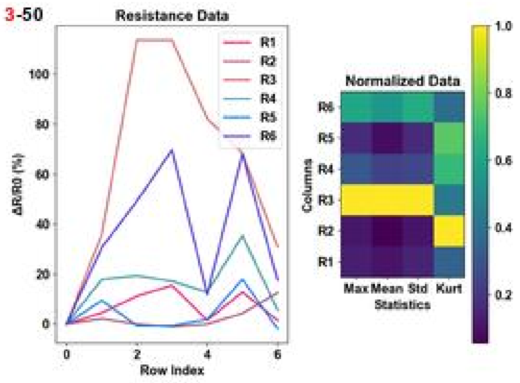

3-51
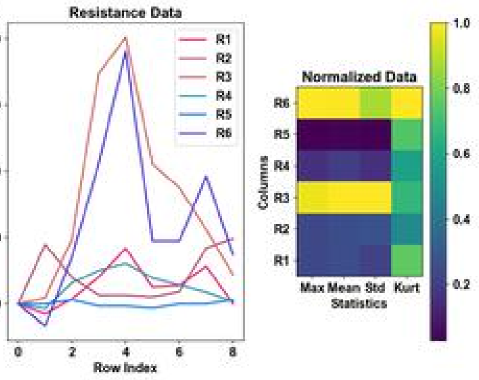

3-52
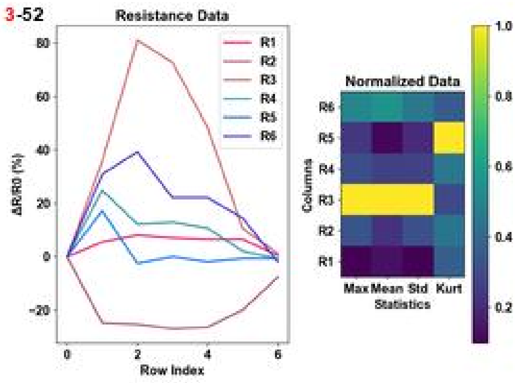

3-53
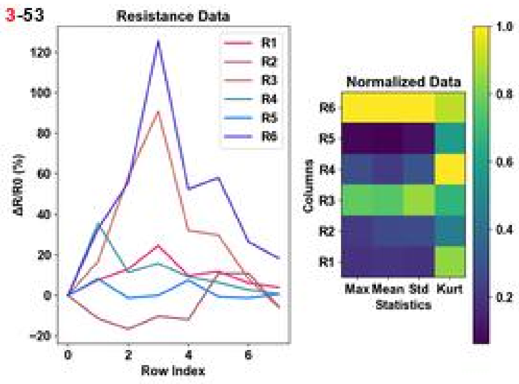

3-54
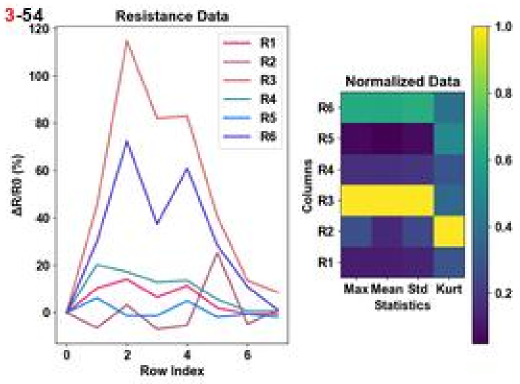

3-55
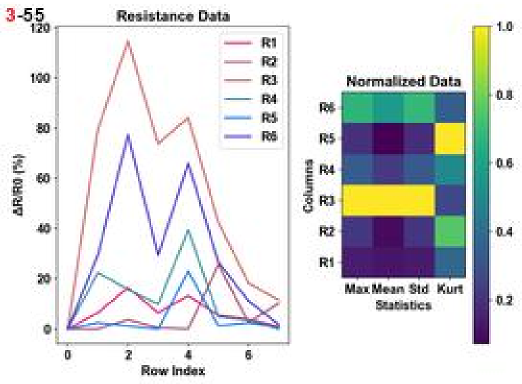

3-56
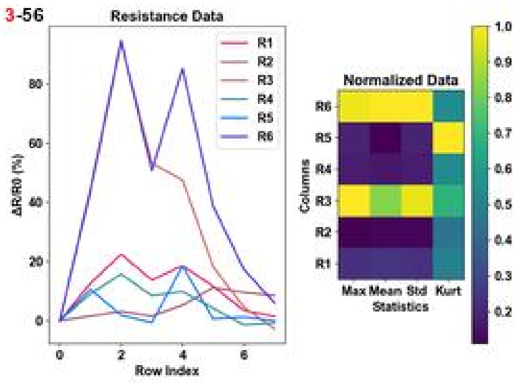

3-57
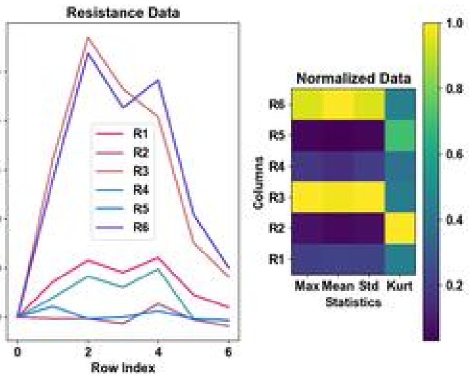

3-58
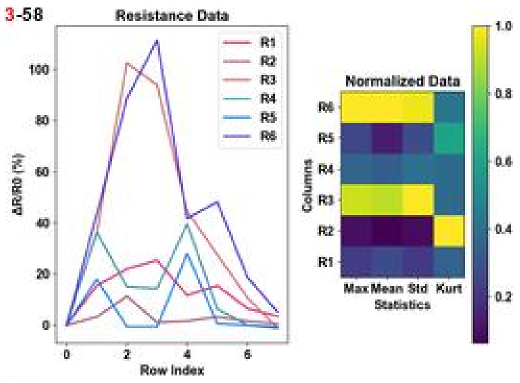

3-59
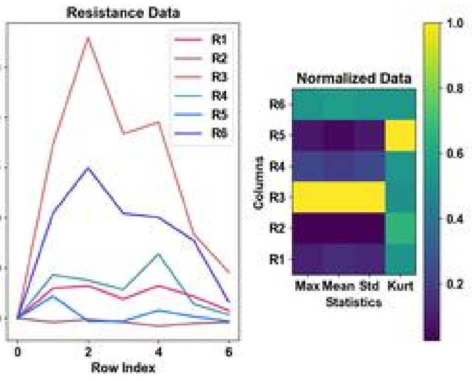

3-60
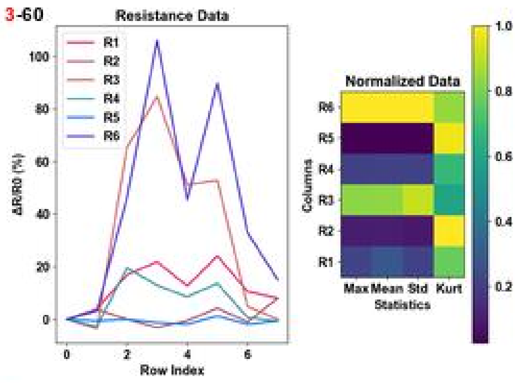

3-61
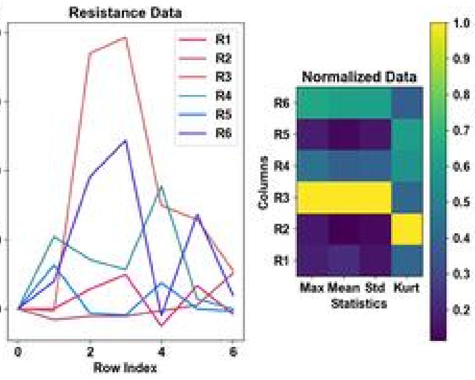

3-62
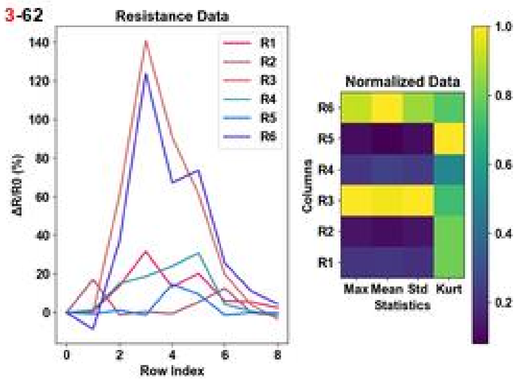

3-63
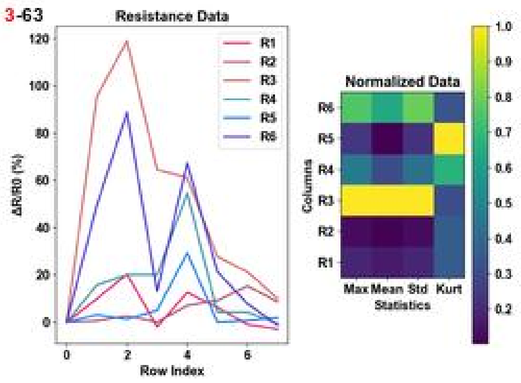

3-64
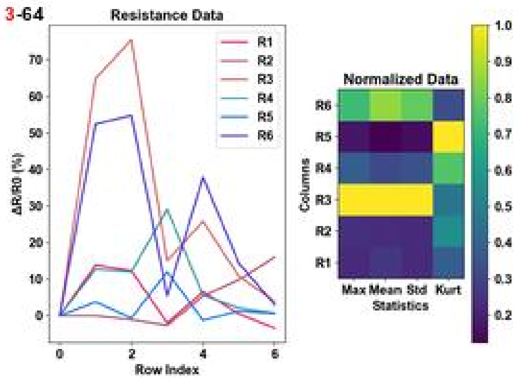

3-65
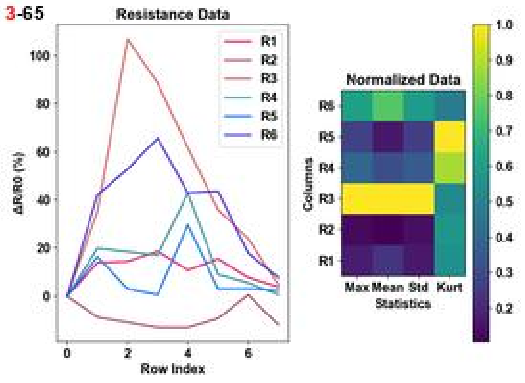

3-66
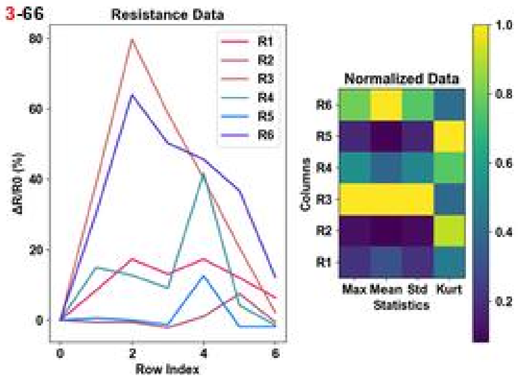

3-67
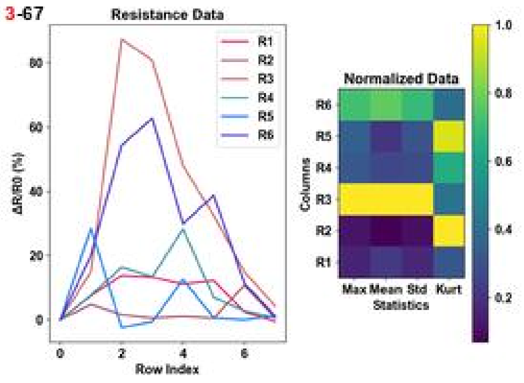

3-68
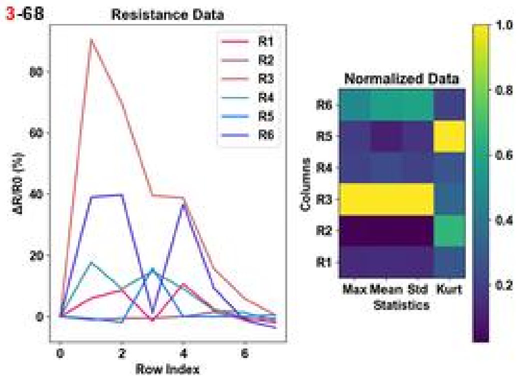

3-69
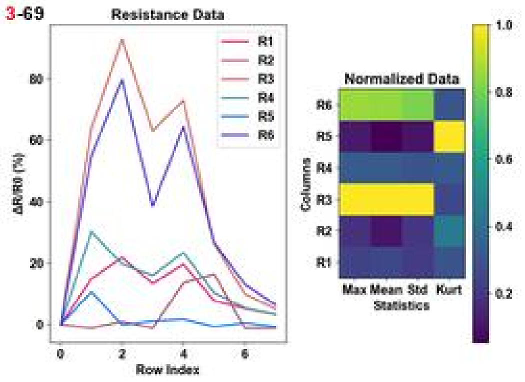

3-70
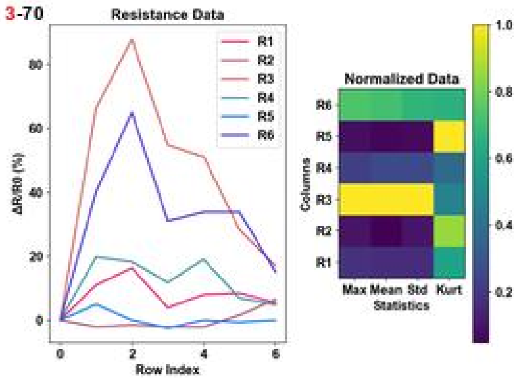

3-71
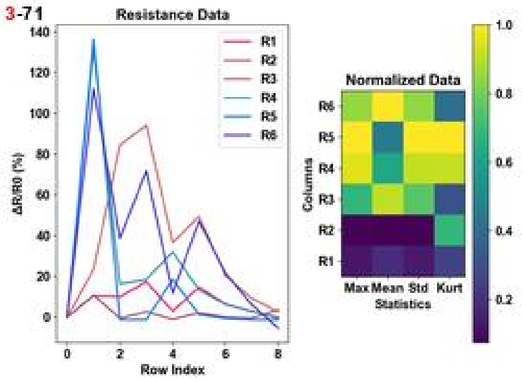

3-72
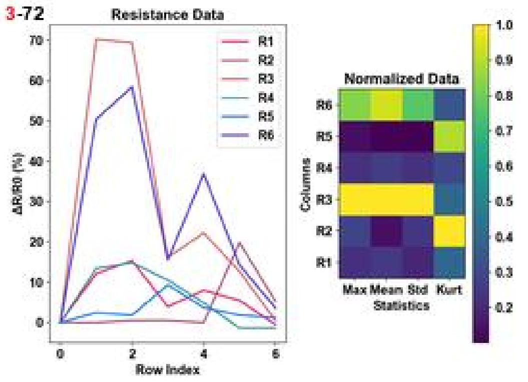

3-73
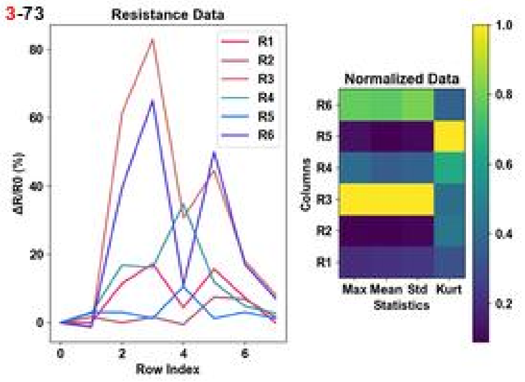

3-74
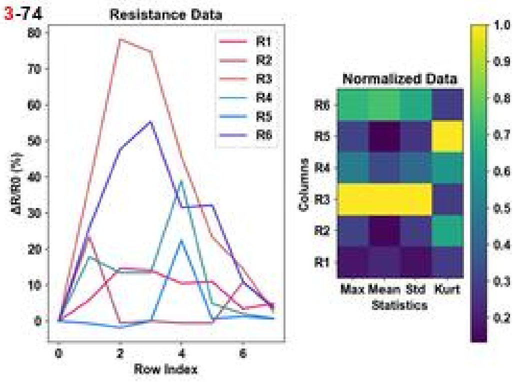

3-75
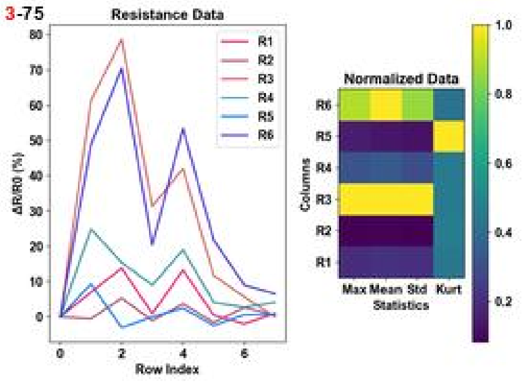

3-76
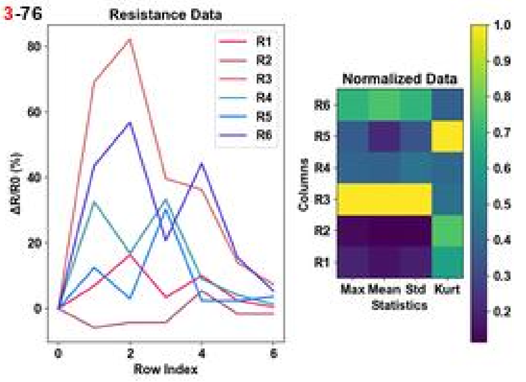

3-77
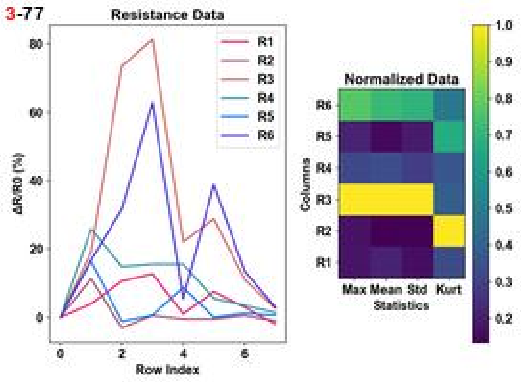

3-78
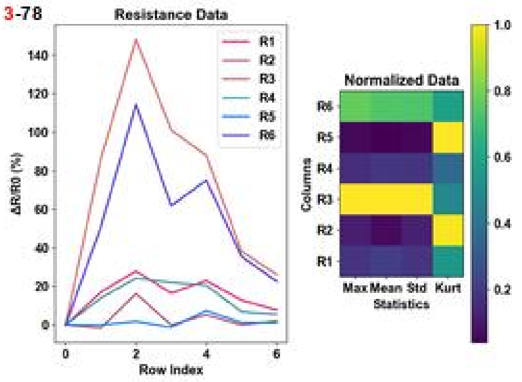

3-79
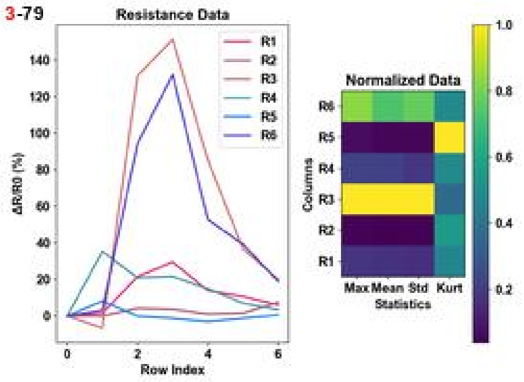

3-80
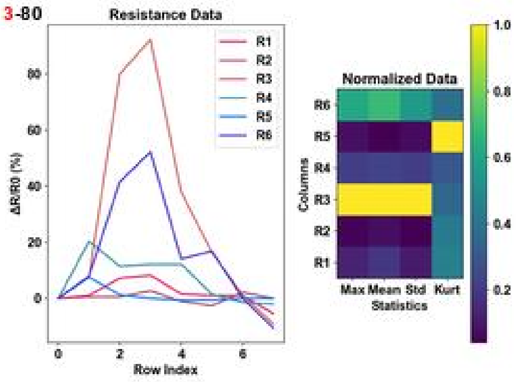

3-81
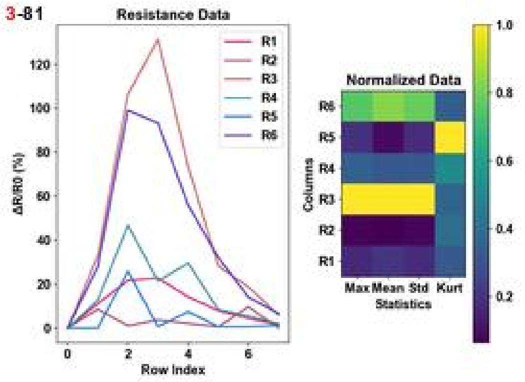

3-82
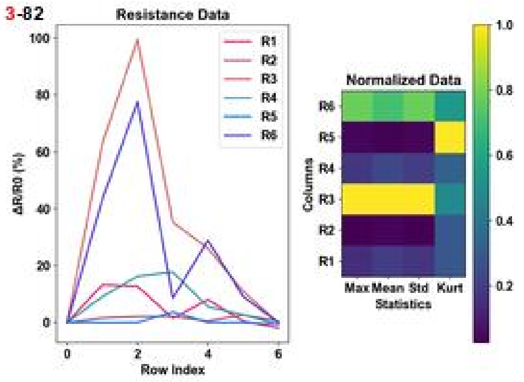

3-83
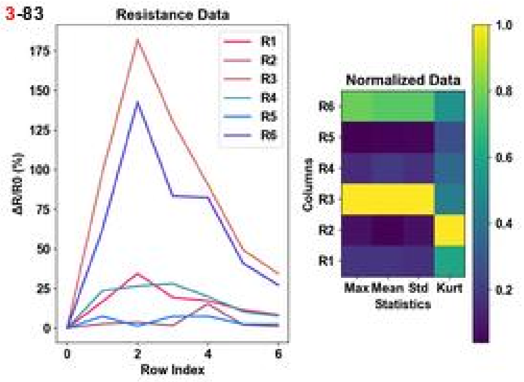

3-84
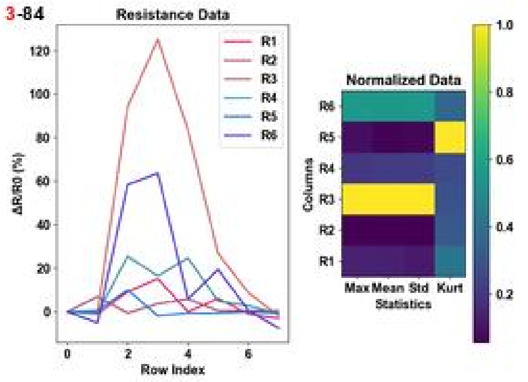

3-85
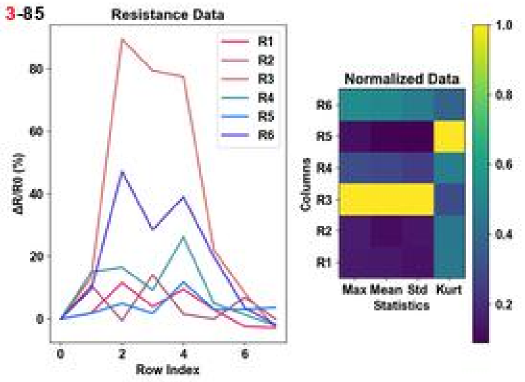

3-86
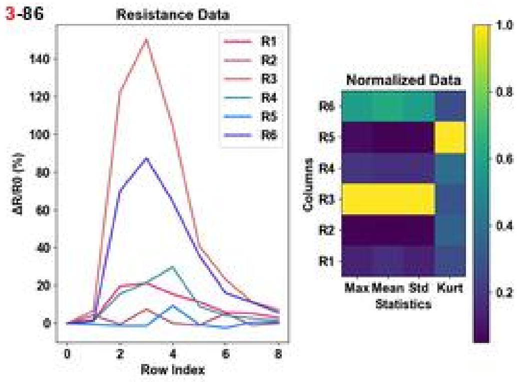

3-87
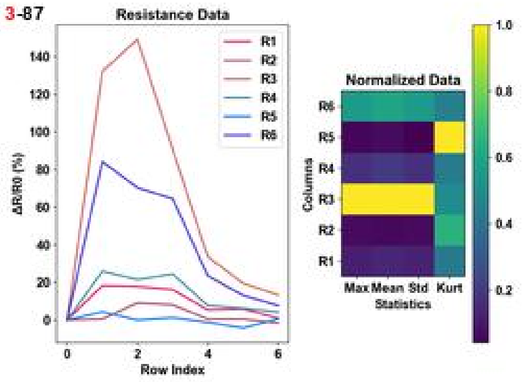

3-88
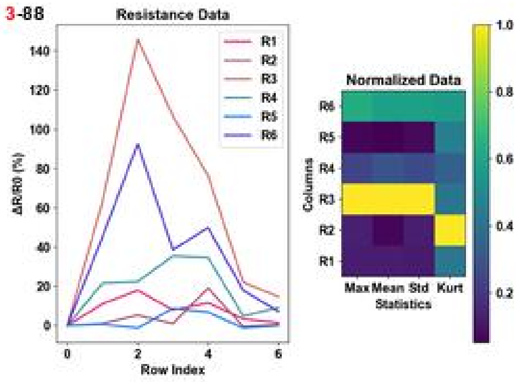

3-89
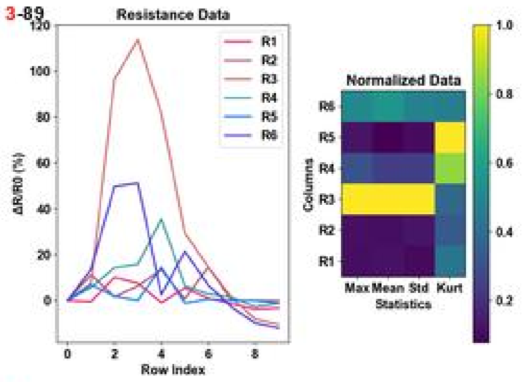

3-90
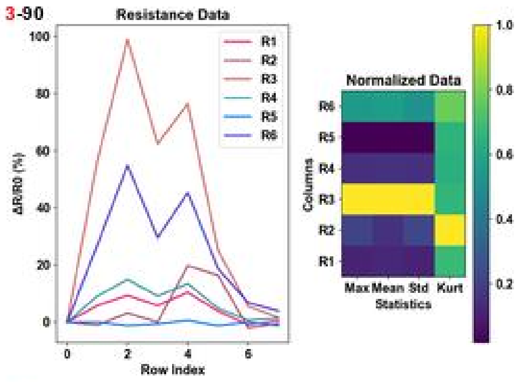

3-91
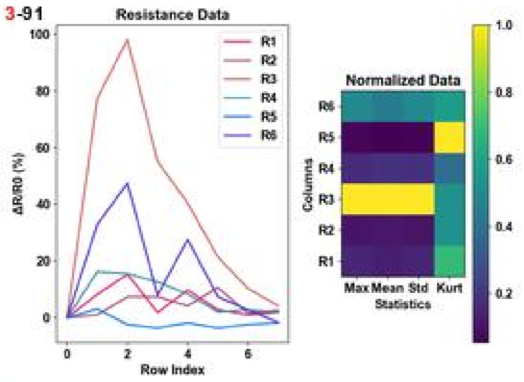

3-92
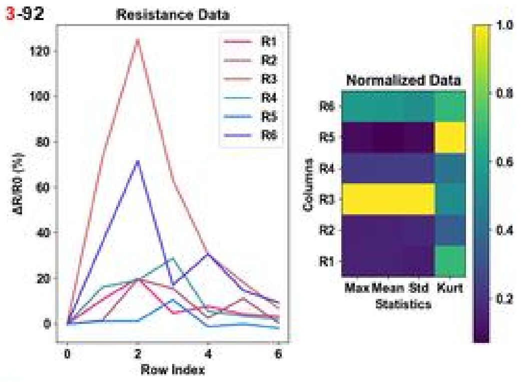

3-93
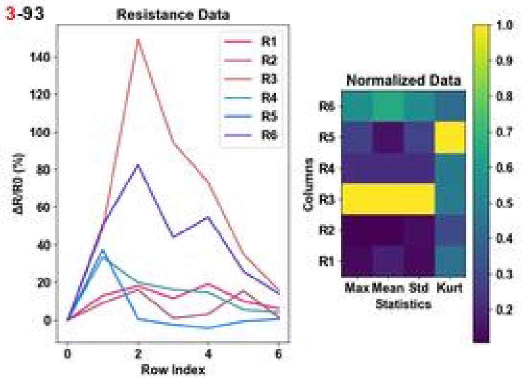

3-94
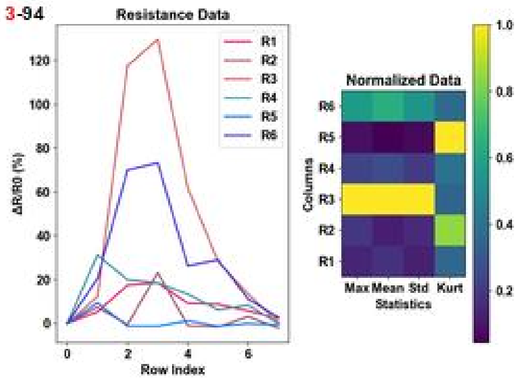

3-95
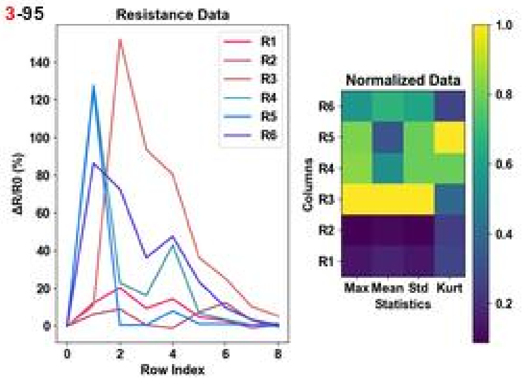

3-96
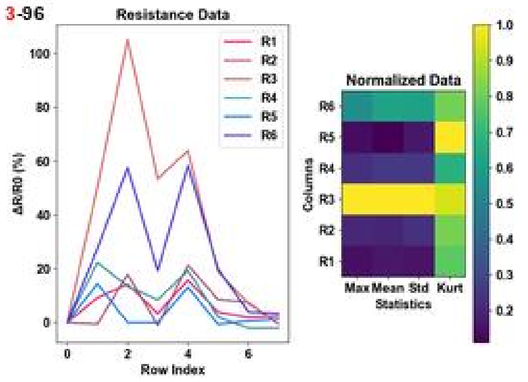

3-97
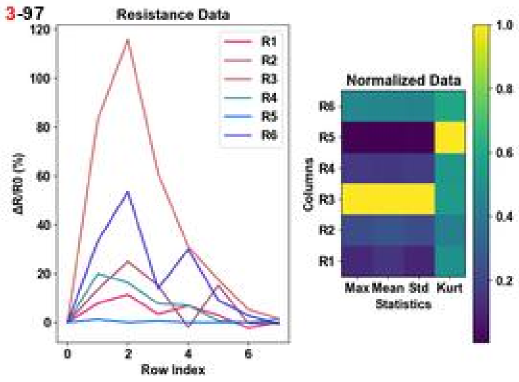

3-98
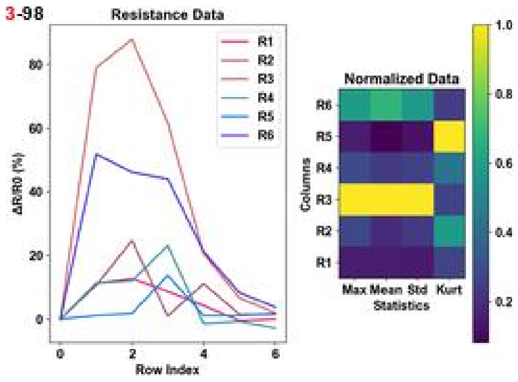

3-99
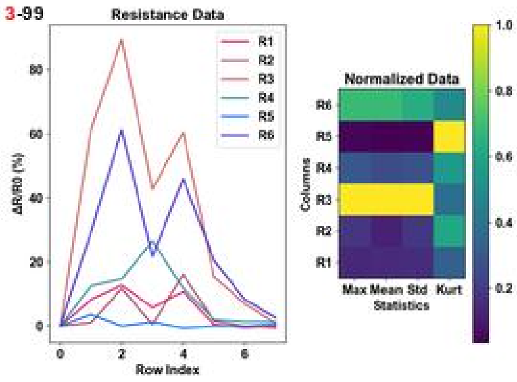

3-100
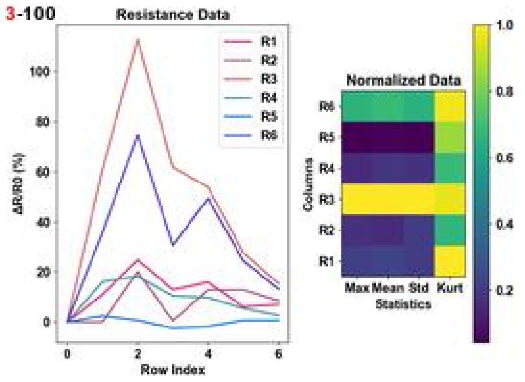

3-101
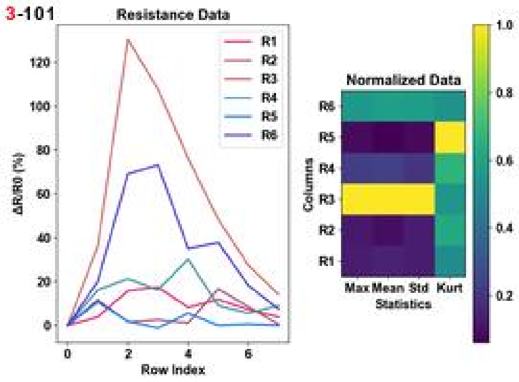

3-102
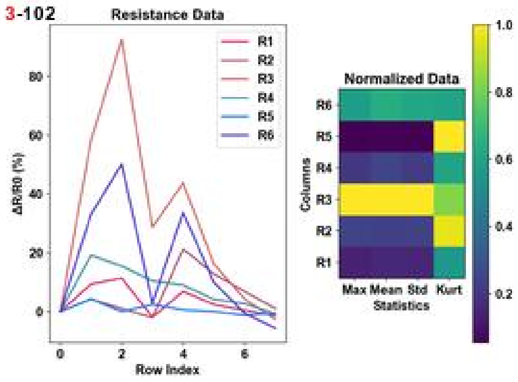

3-103
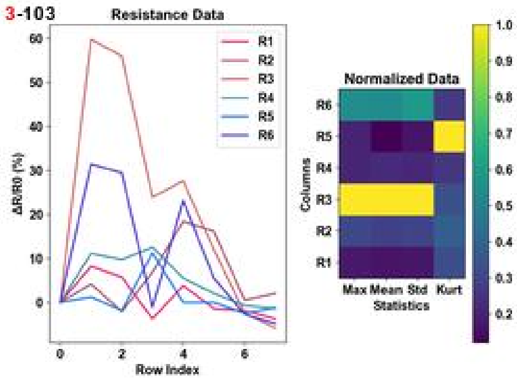

3-104
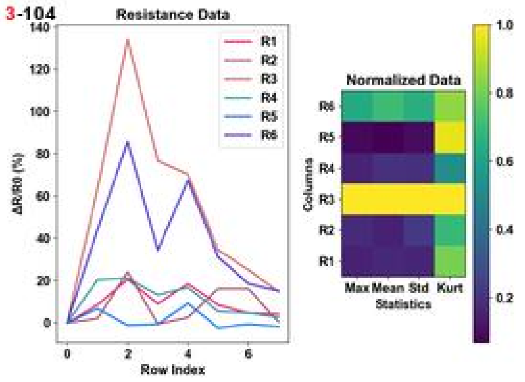

3-105
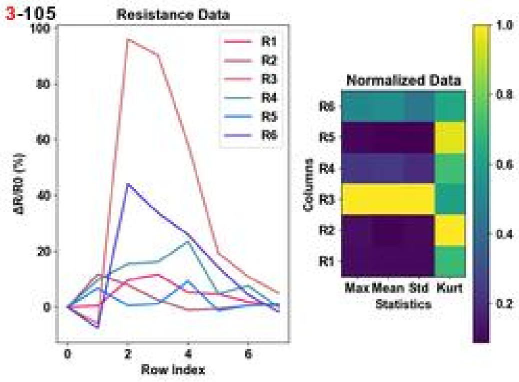

3-106
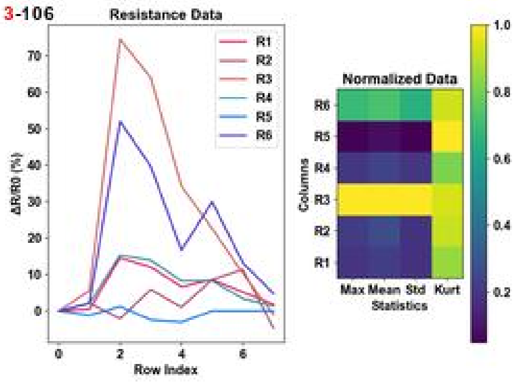

3-107
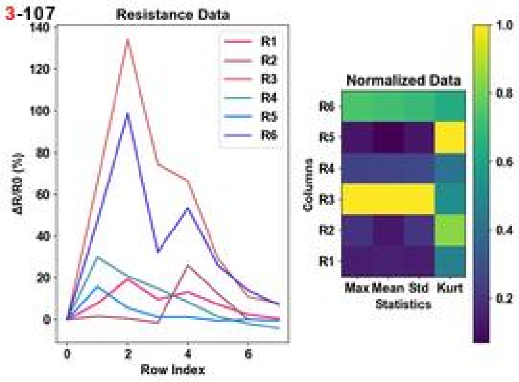

3-108
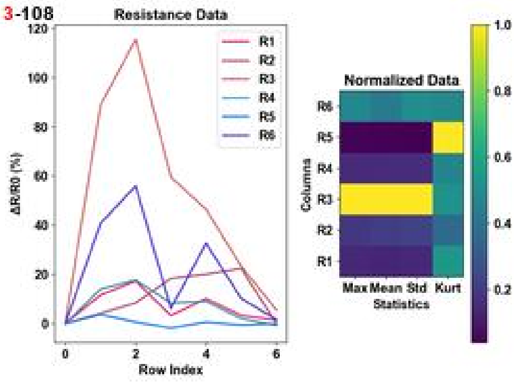

3-109
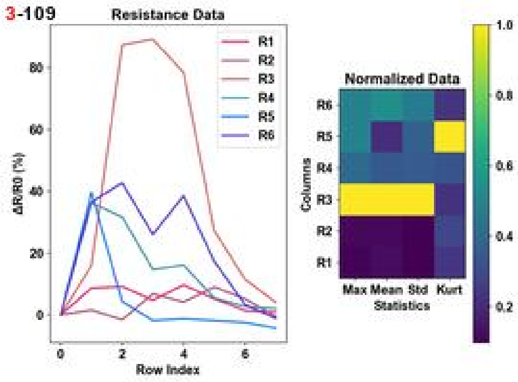

3-110
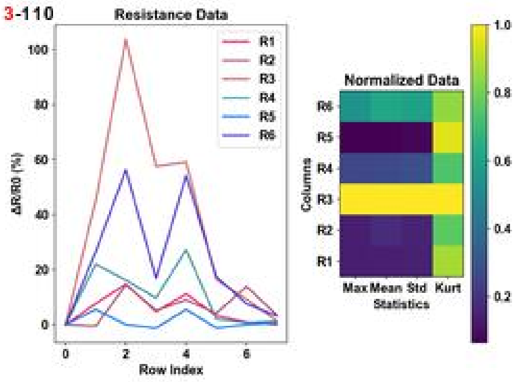

3-111
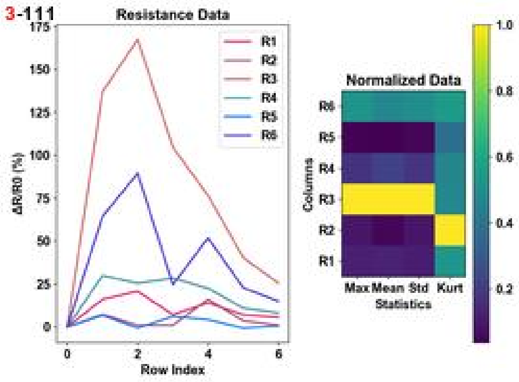

3-112
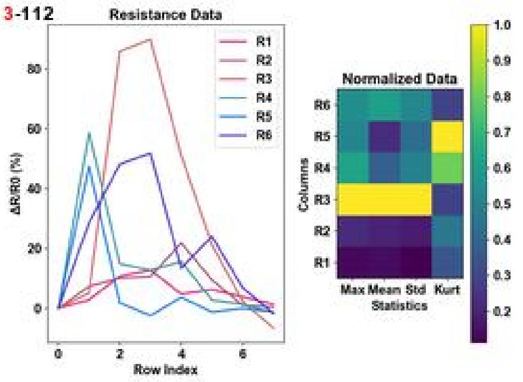

3-113
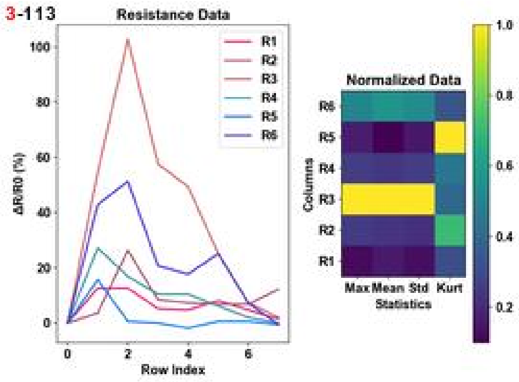

3-114
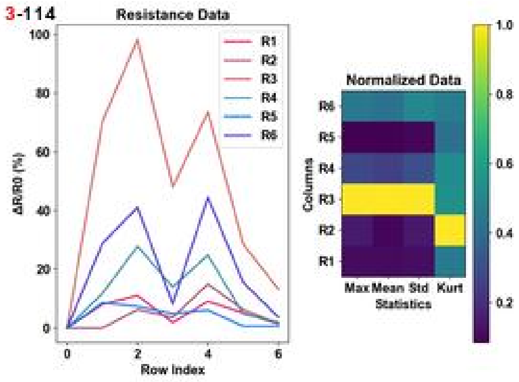

3-115
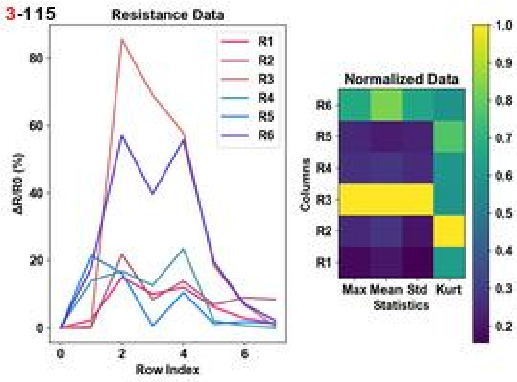

3-116
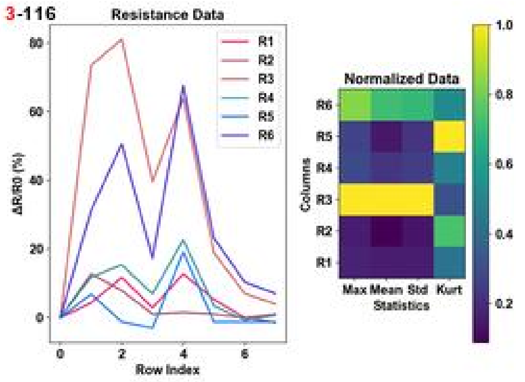

3-117
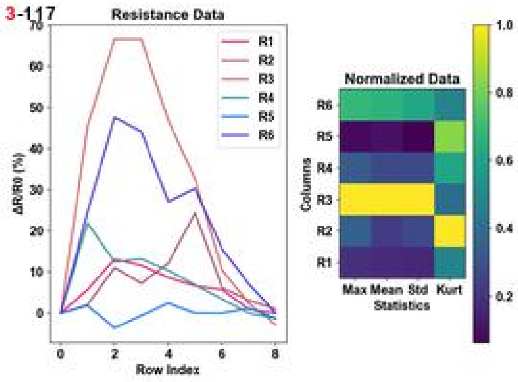

3-118
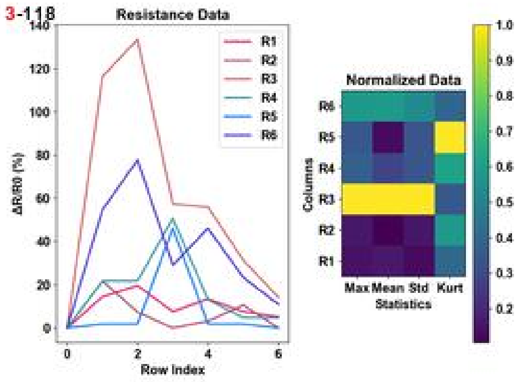

3-119
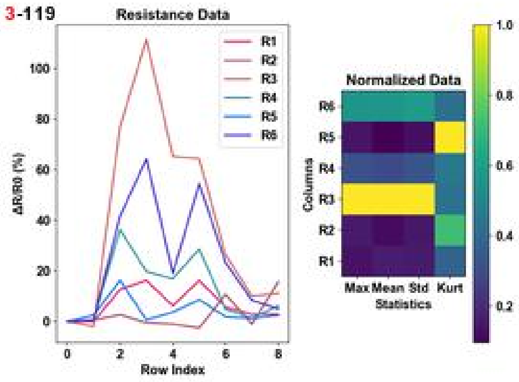

3-120
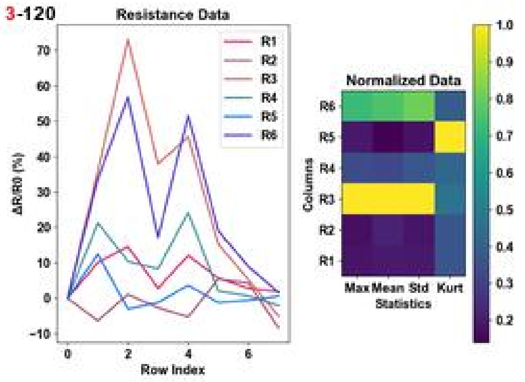

3-121
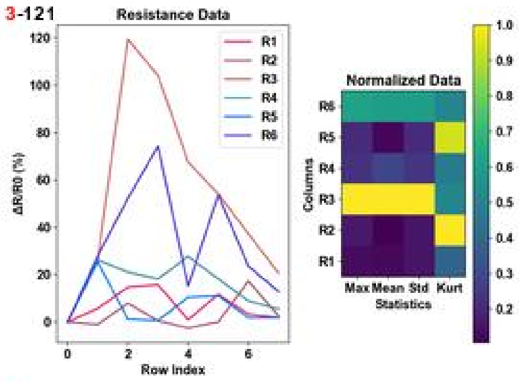

3-122
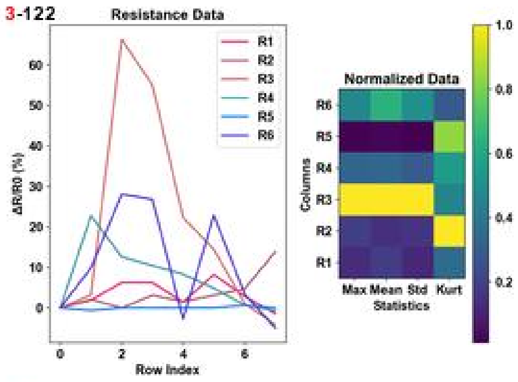

3-123
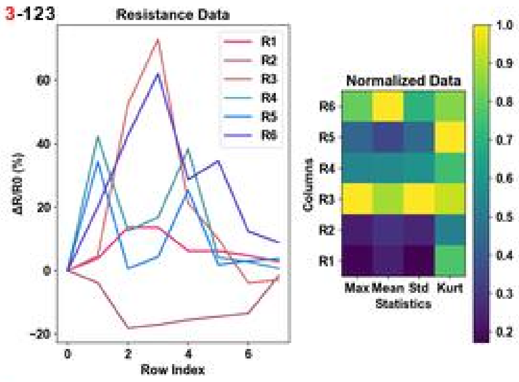

3-124
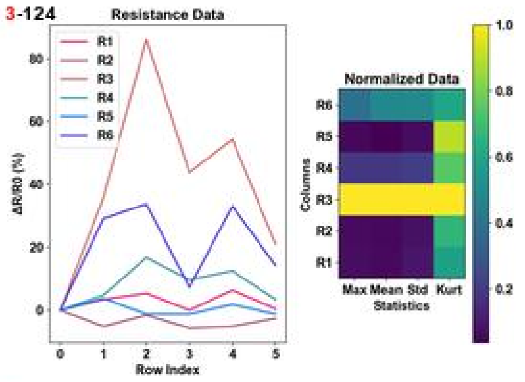

3-125
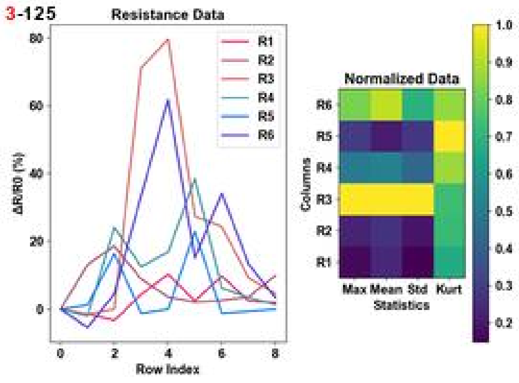

3-126
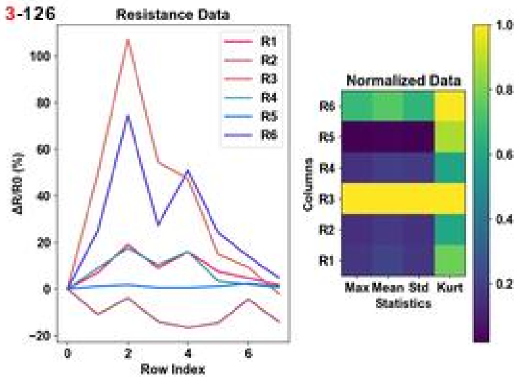

3-127
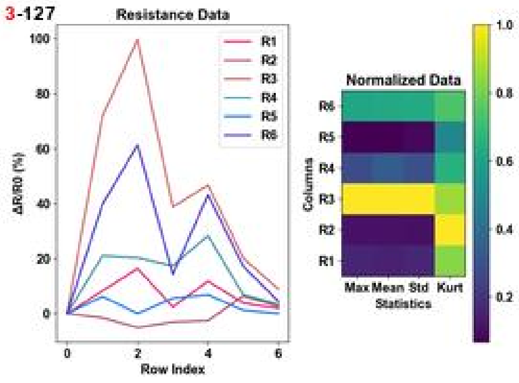

3-128
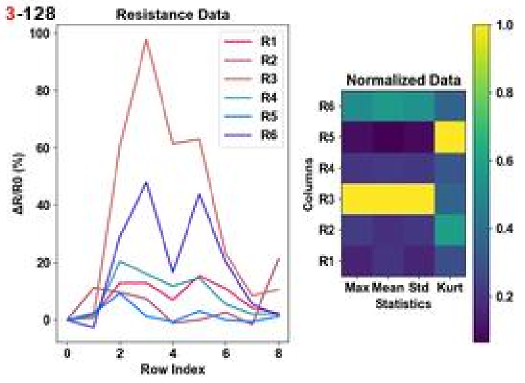

3-129
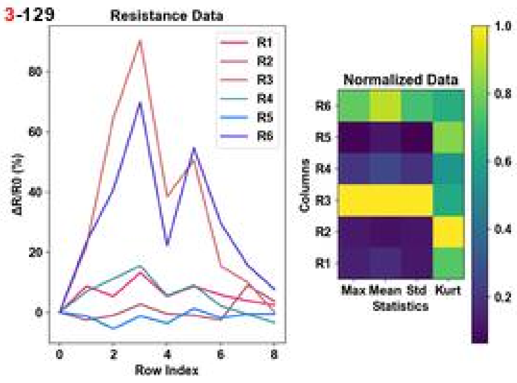
3-130
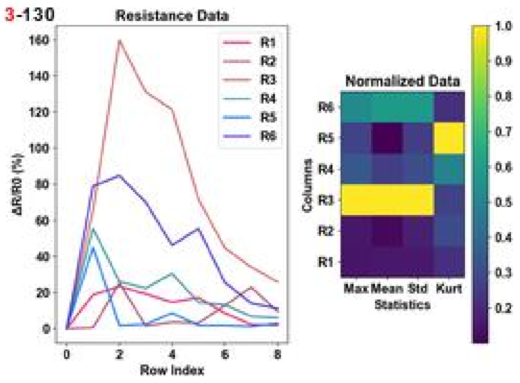
3-131
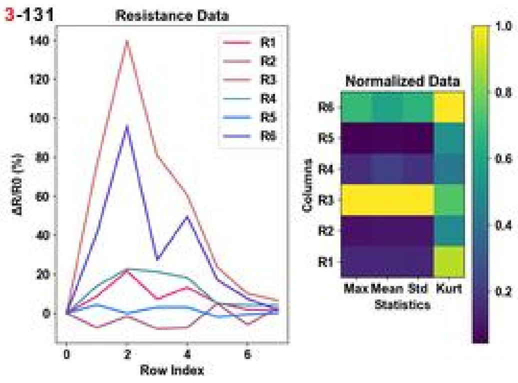
3-132
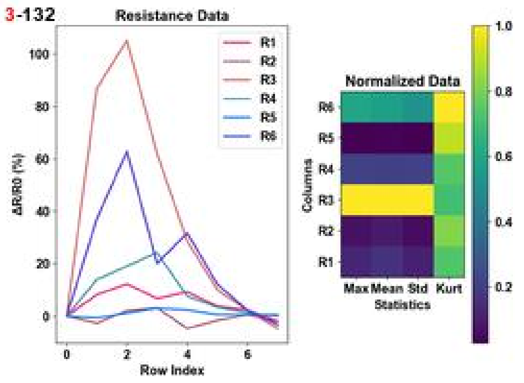
3-133
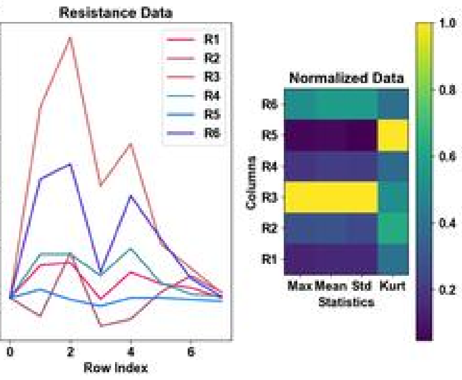
3-134
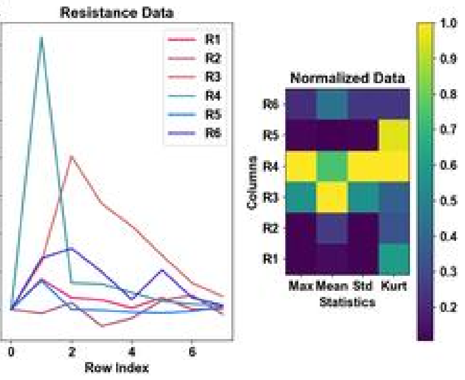
3-135
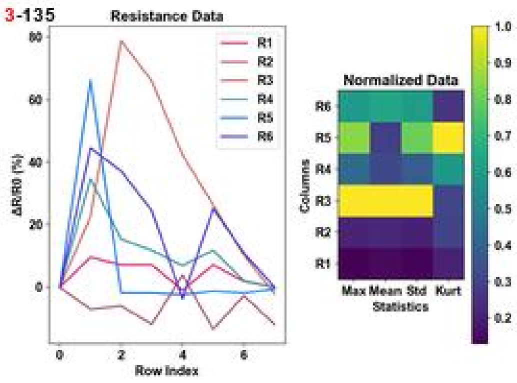
3-136
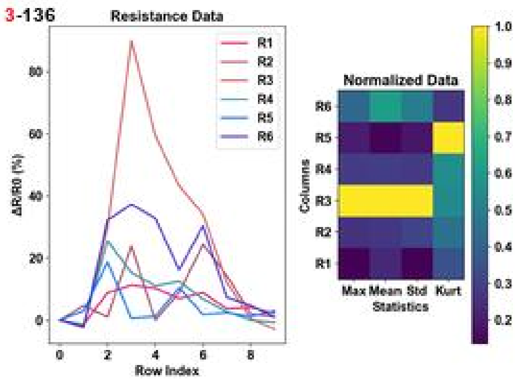
3-137
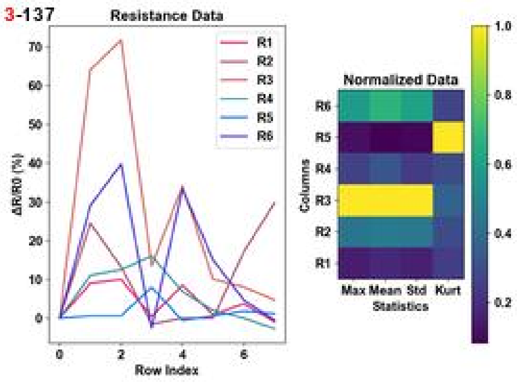
3-138
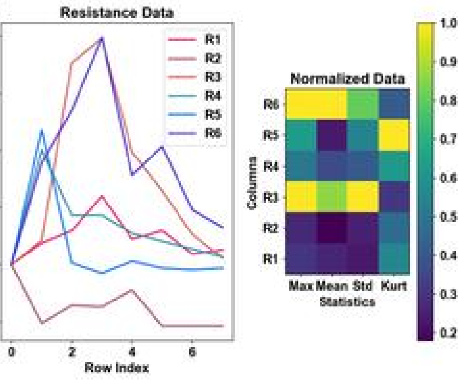
3-139
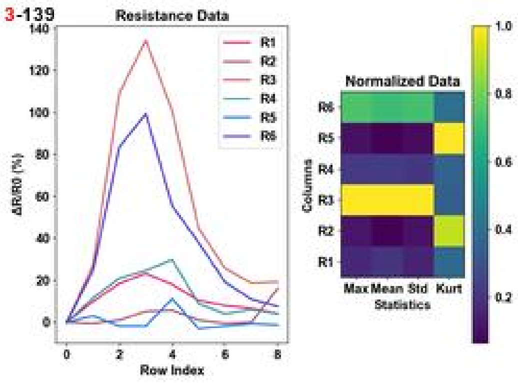
3-140
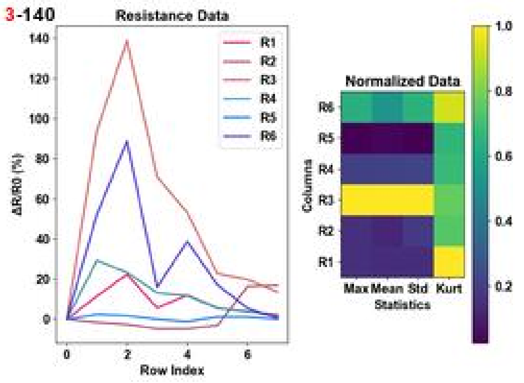
3-141
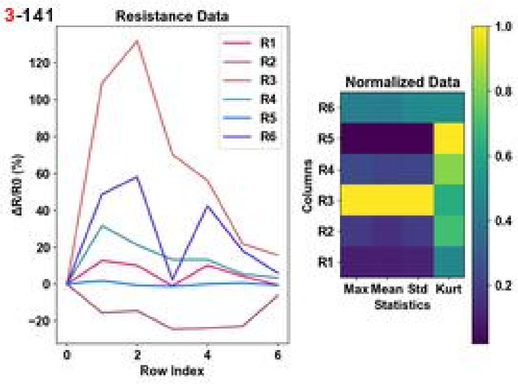
3-142
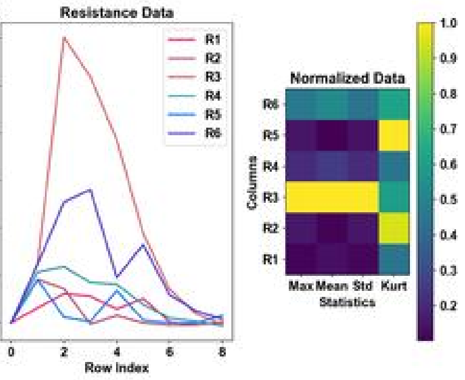
3-143
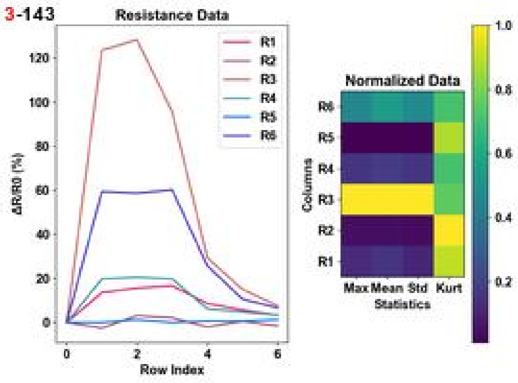
3-144
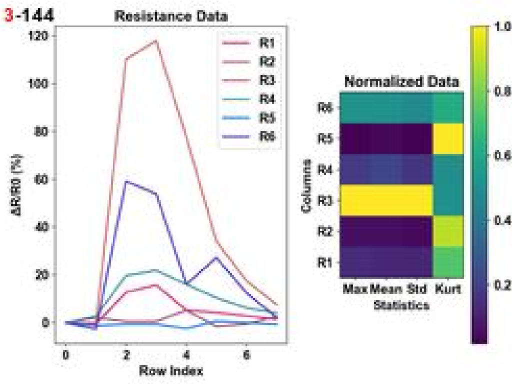
3-145
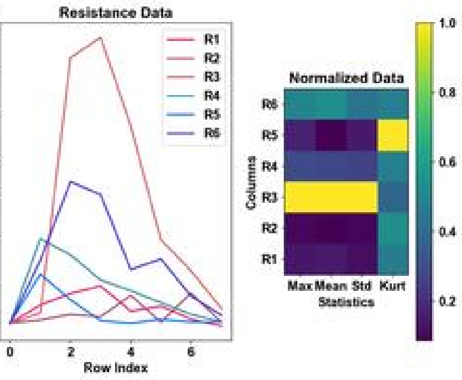
3-146
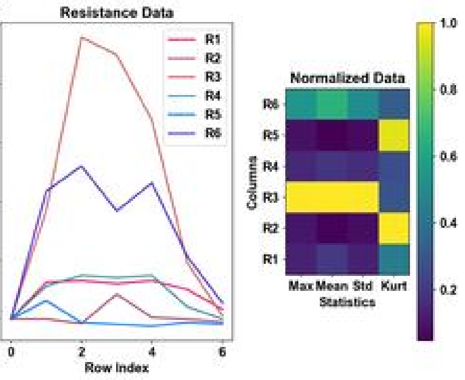
3-147
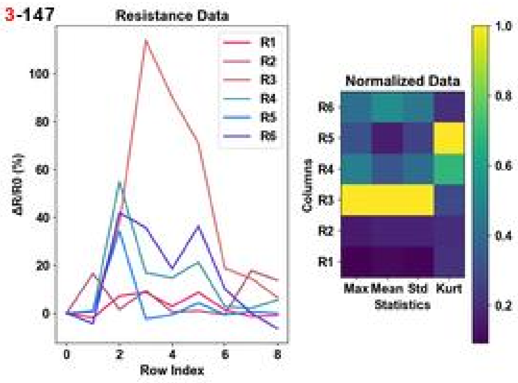
3-148
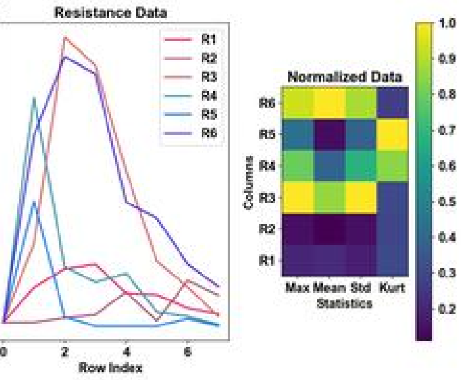
3-149
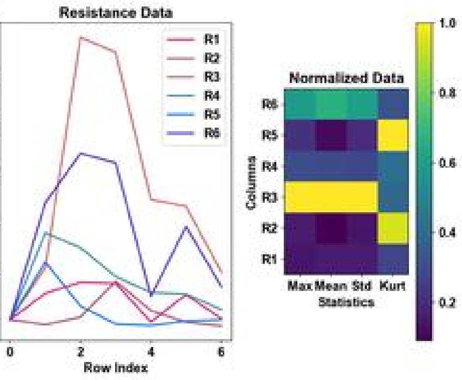
3-150
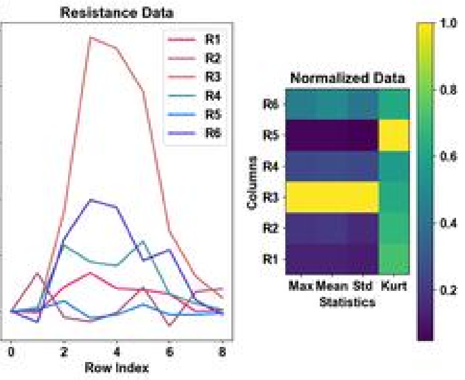
3-151
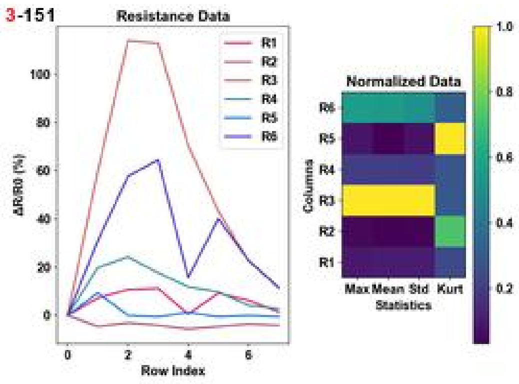
3-152
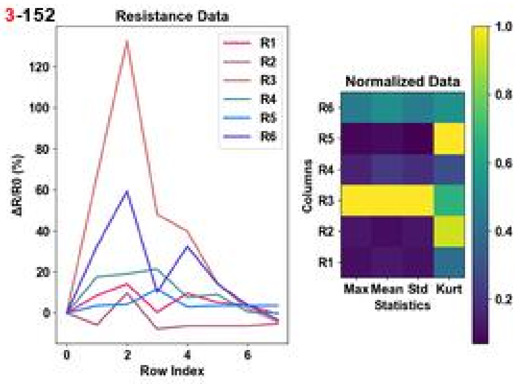
3-153
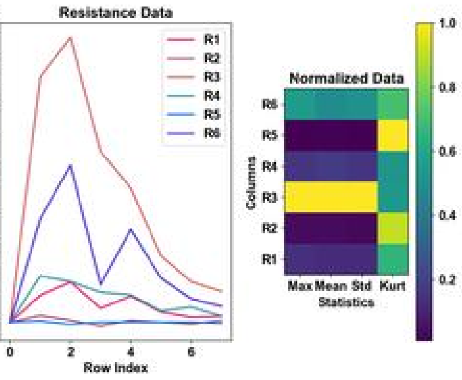
3-154
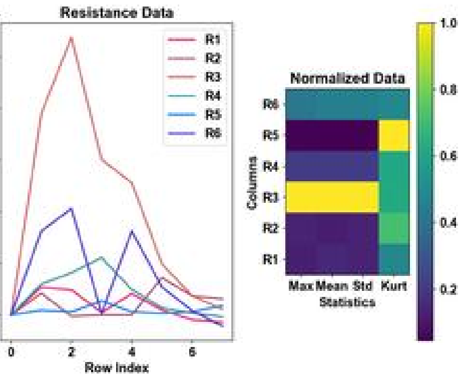
3-155
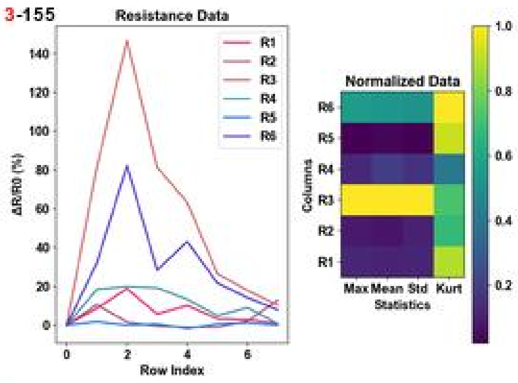
3-156
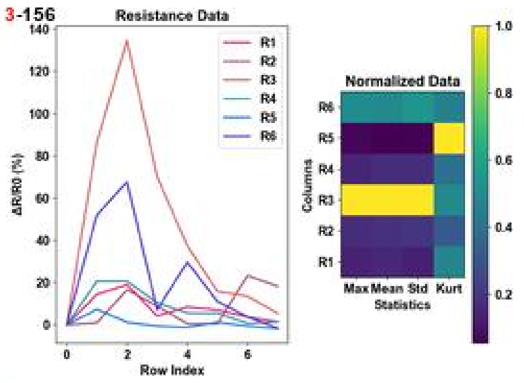
3-157
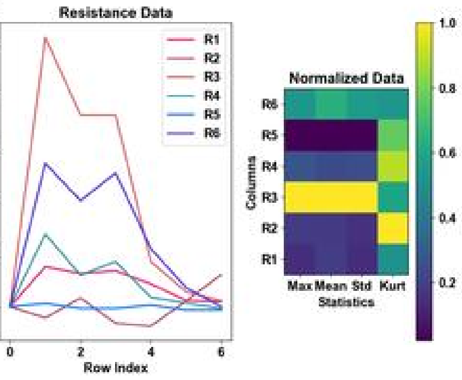
3-158
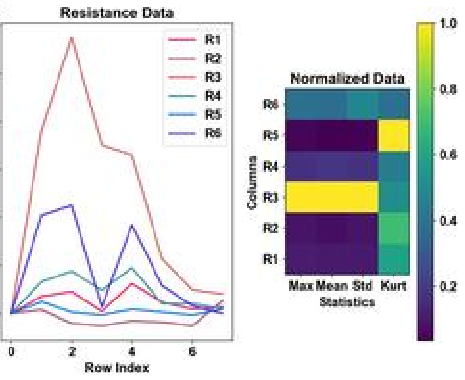
3-159
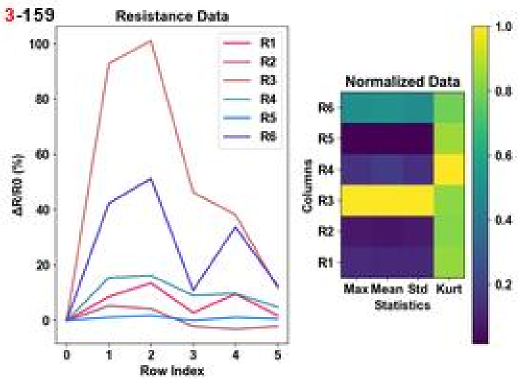
3-160
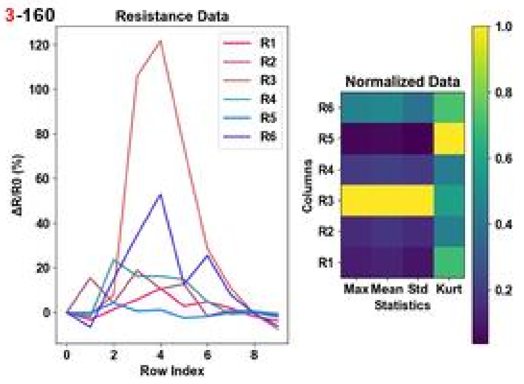

3-161
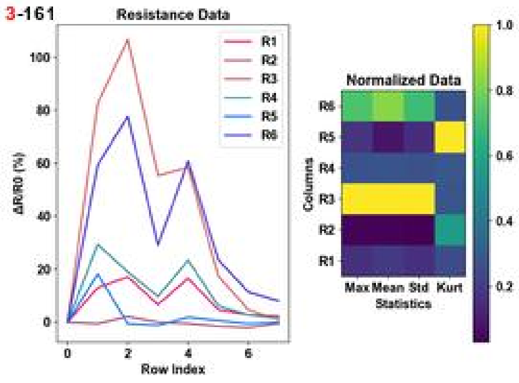

3-162
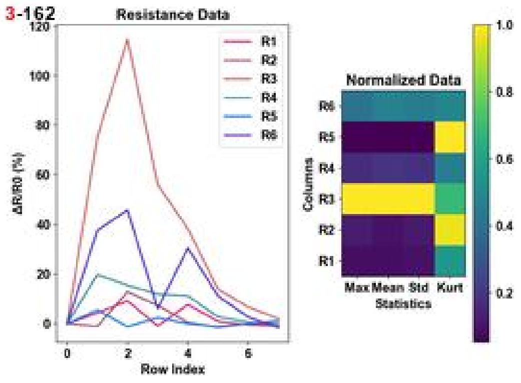

3-163
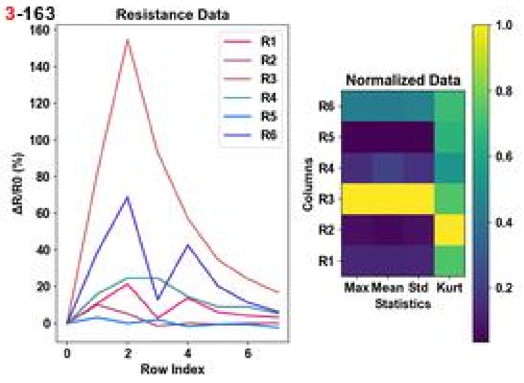

3-164
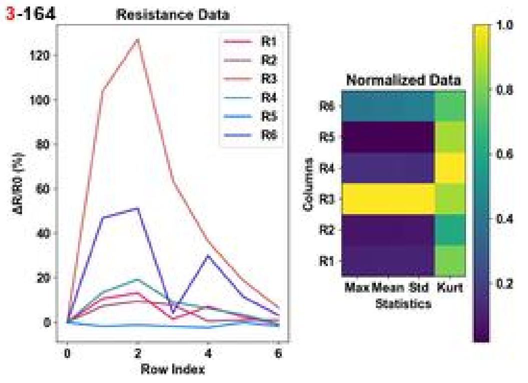

3-165
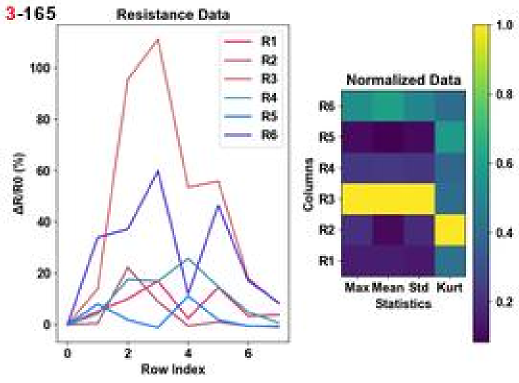

3-166
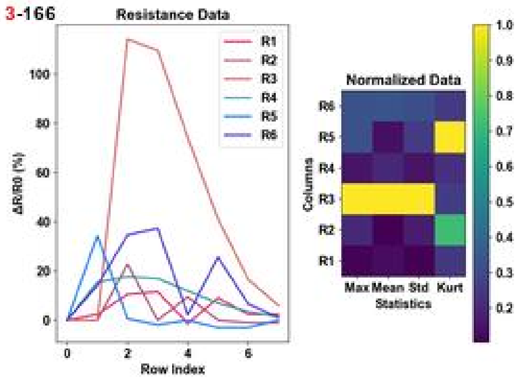

3-167
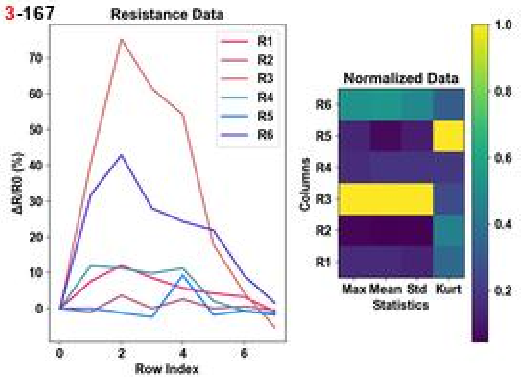

3-168
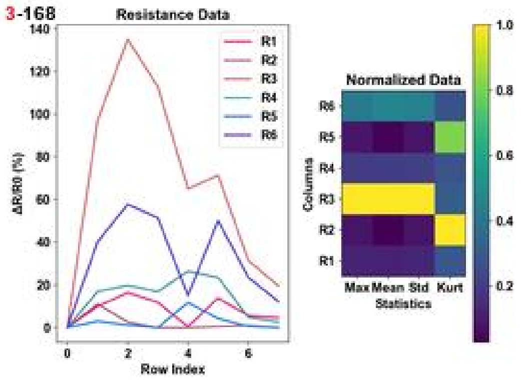

3-169
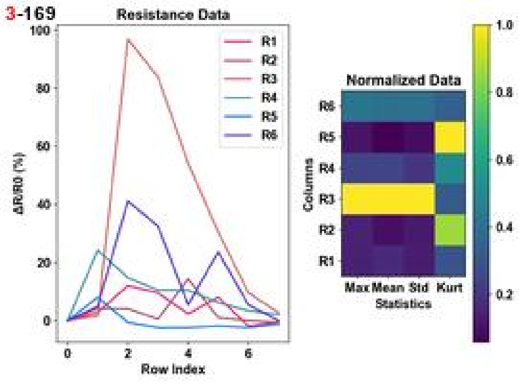

3-170
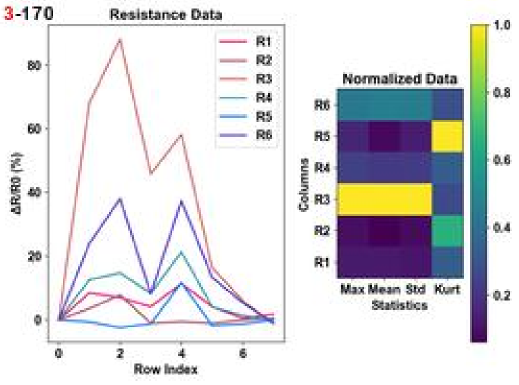

3-171
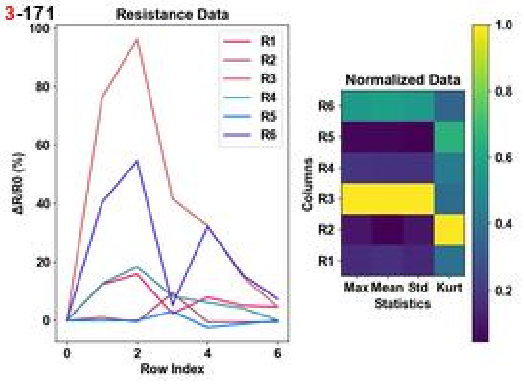

3-172
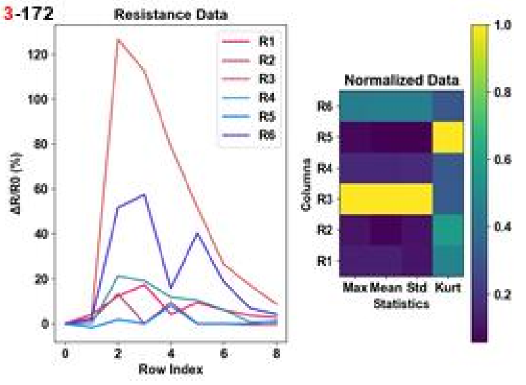

3-173
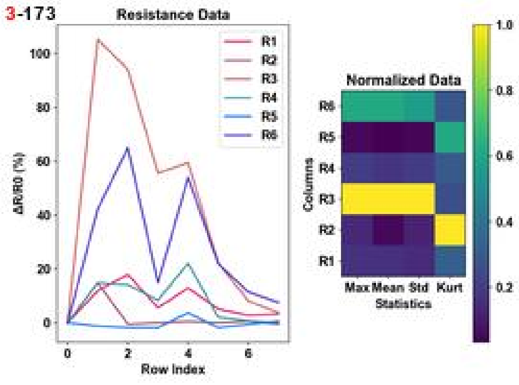

3-174
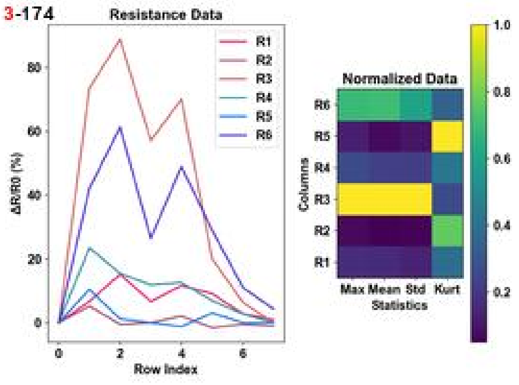

3-175
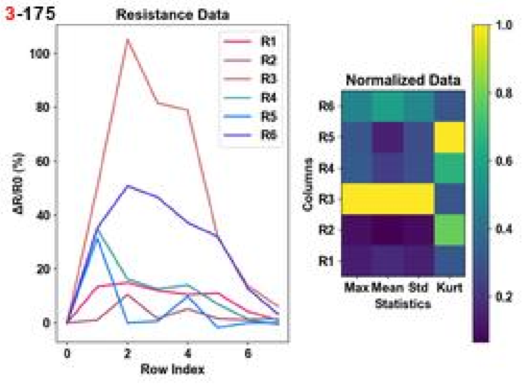

3-176
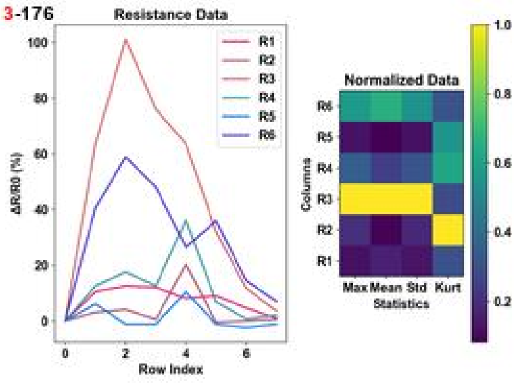

3-177
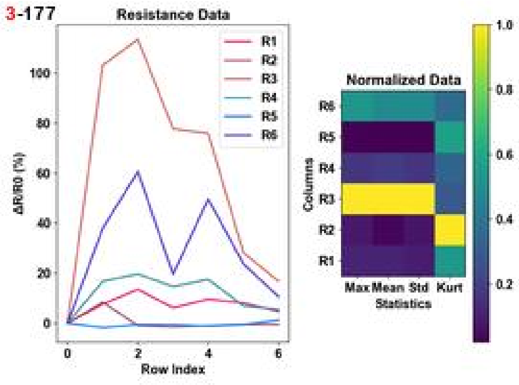

3-178
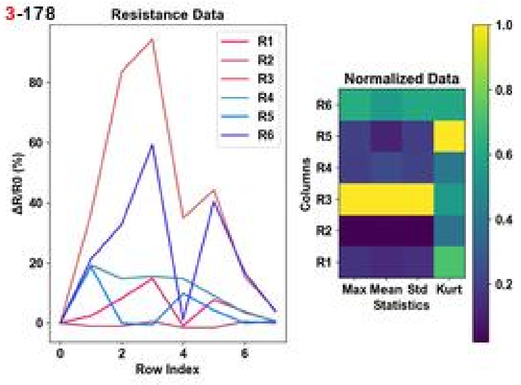

3-179
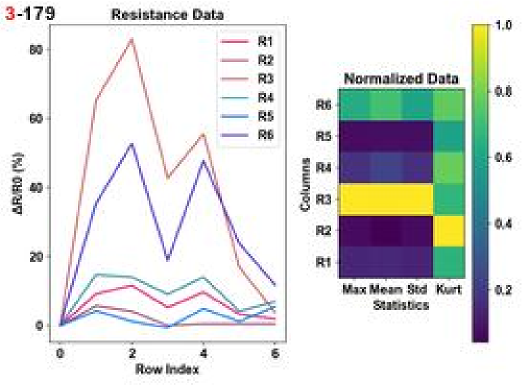

3-180
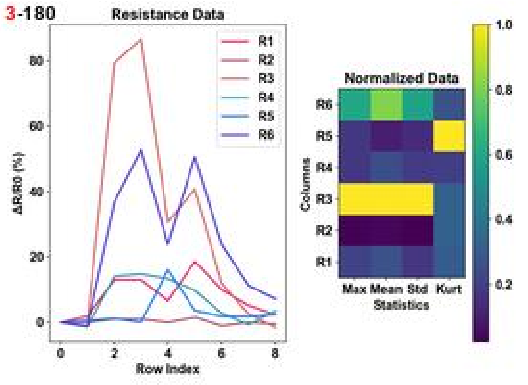

3-181
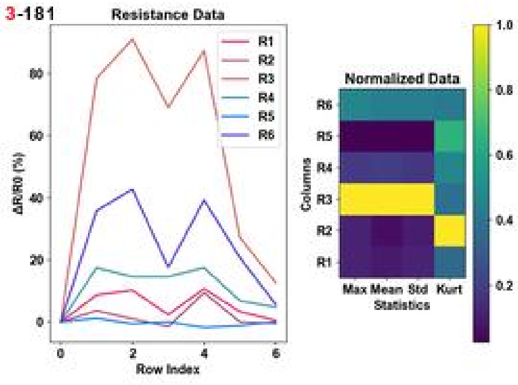

3-182
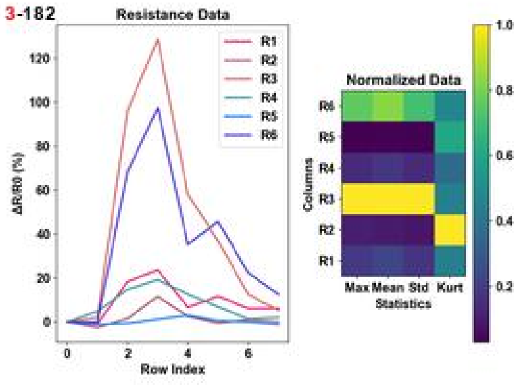

3-183
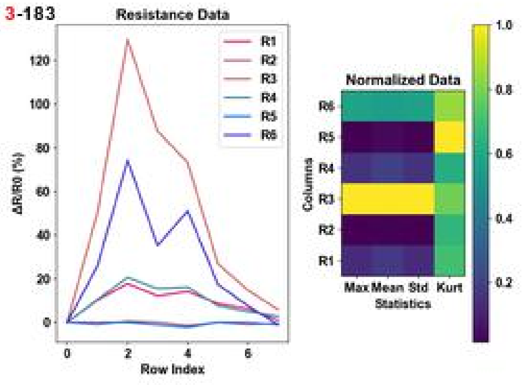

3-184
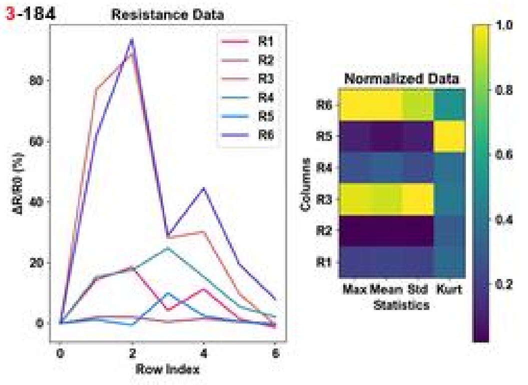

3-185
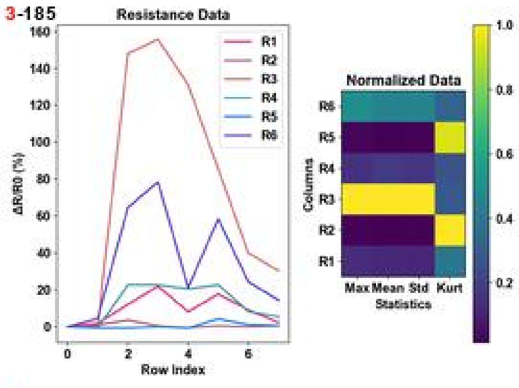

3-186
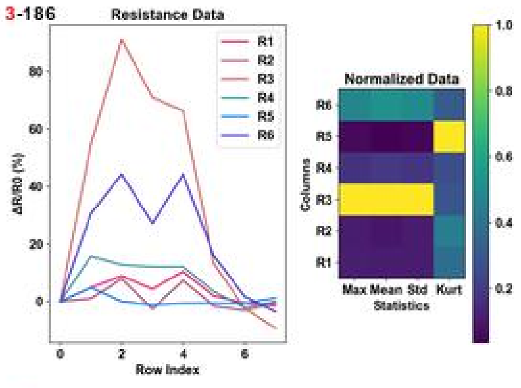

3-187
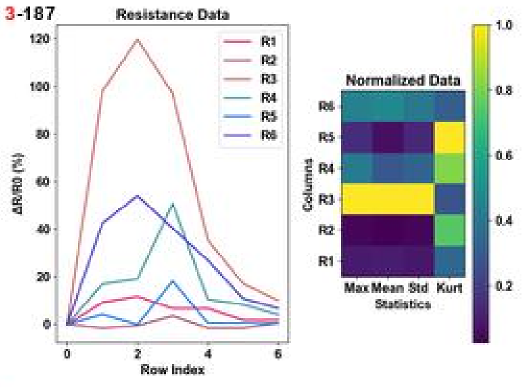

3-188
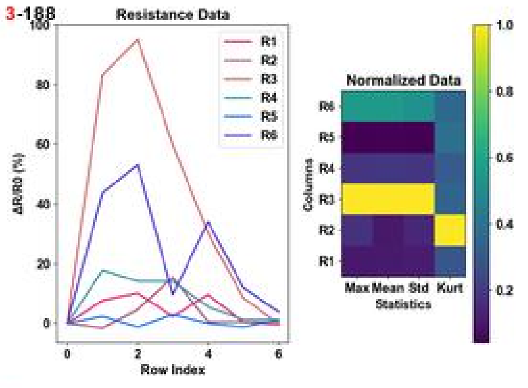

3-189
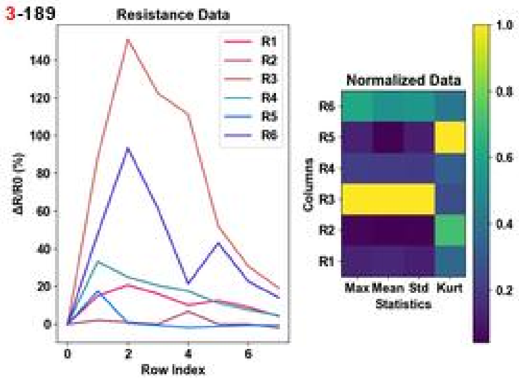

3-190
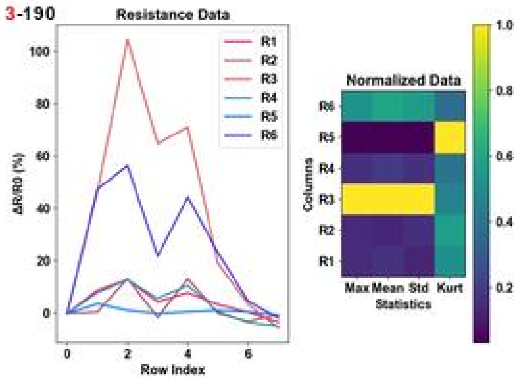

3-191
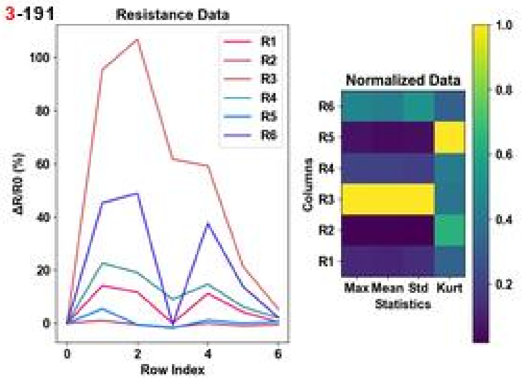

3-192
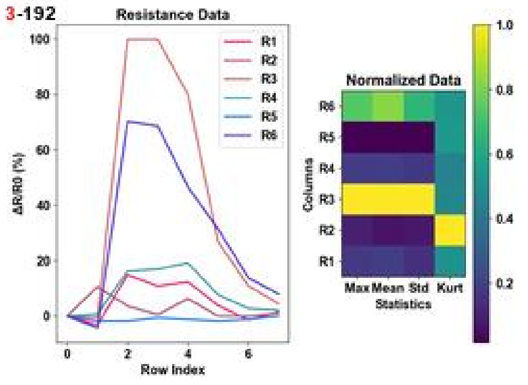

3-193

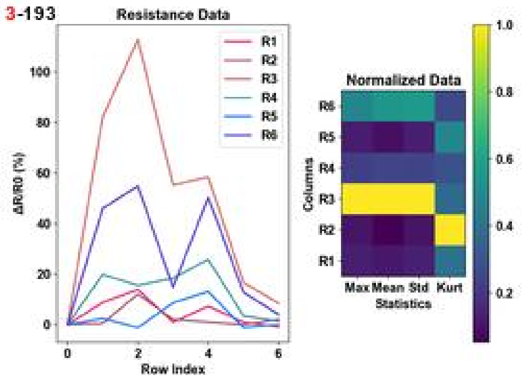

3-194

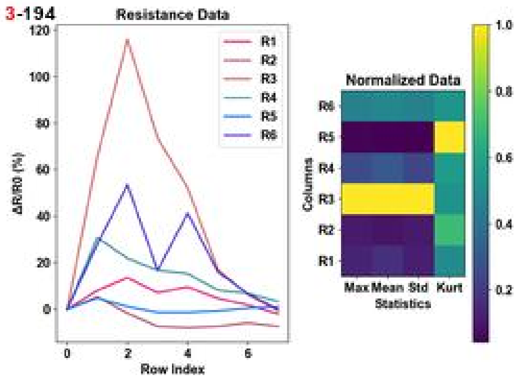

3-195

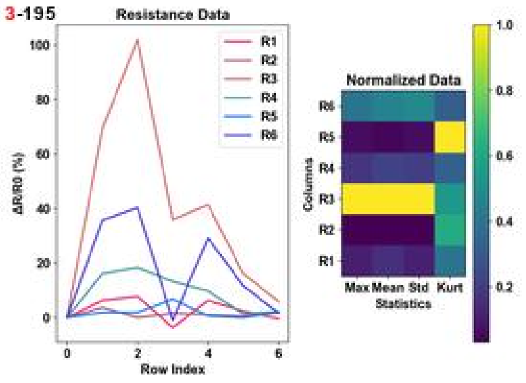

3-196

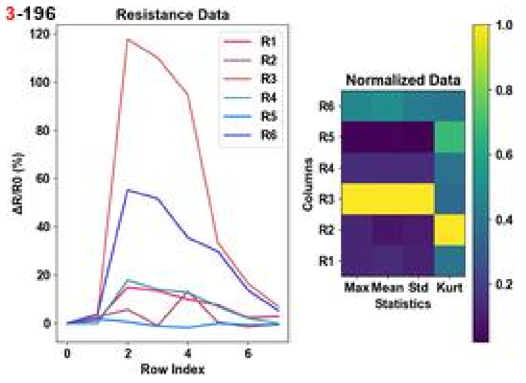

3-197

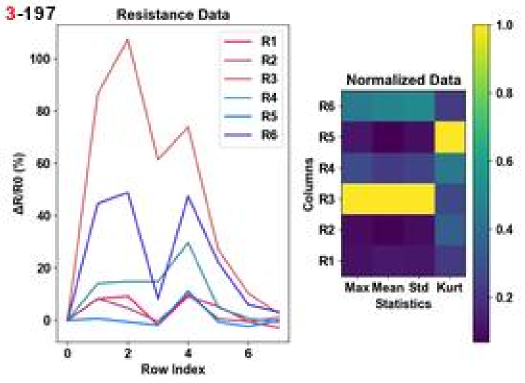

3-198

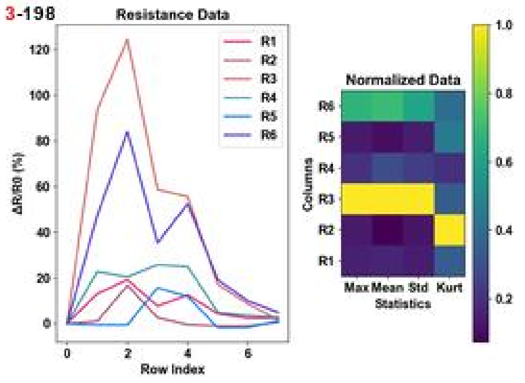

3-199

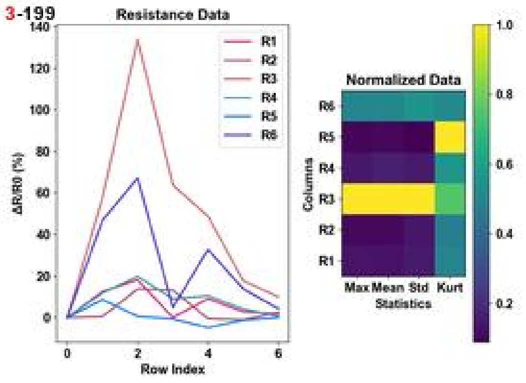

3-200

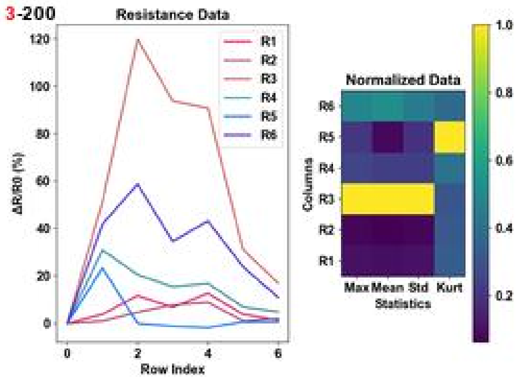

3-201

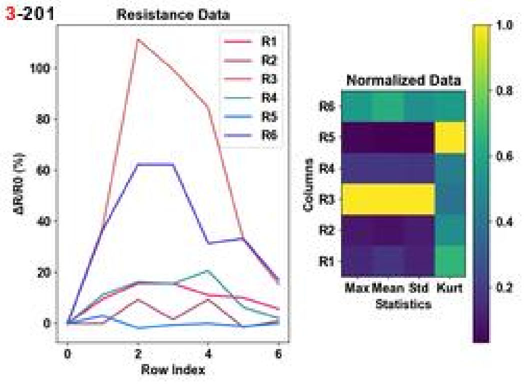

3-202

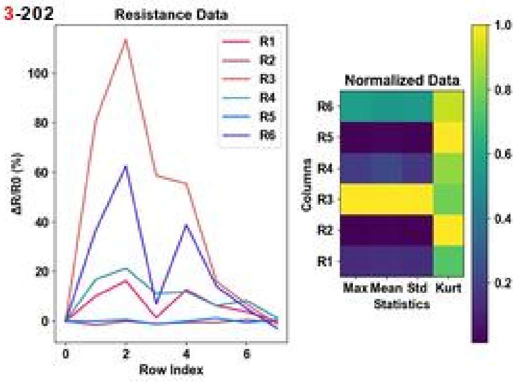

3-203

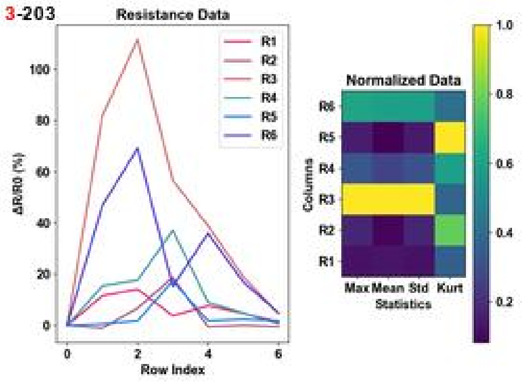

3-204

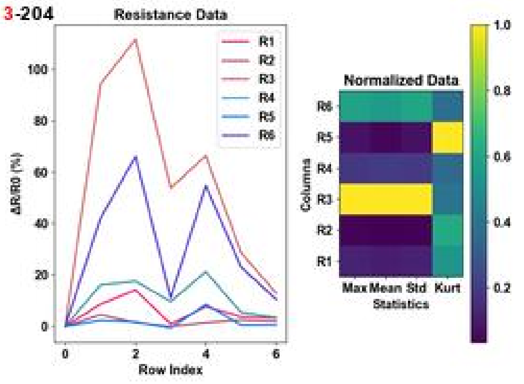

3-205

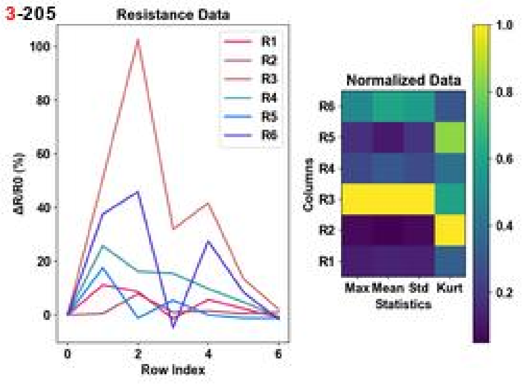

3-206

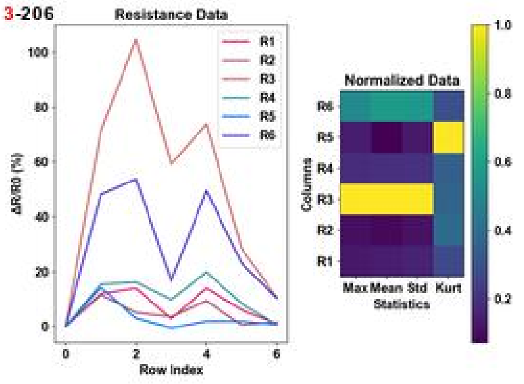

3-207

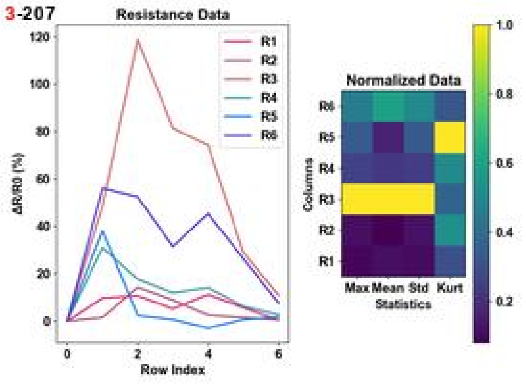

3-208

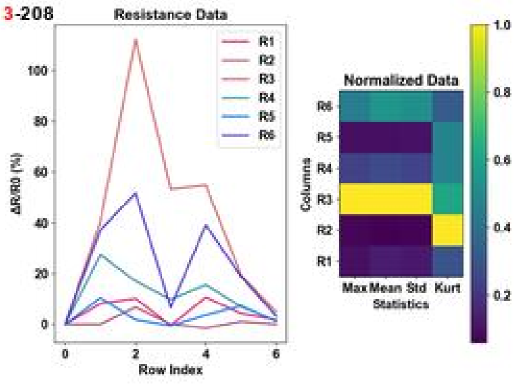

3-209

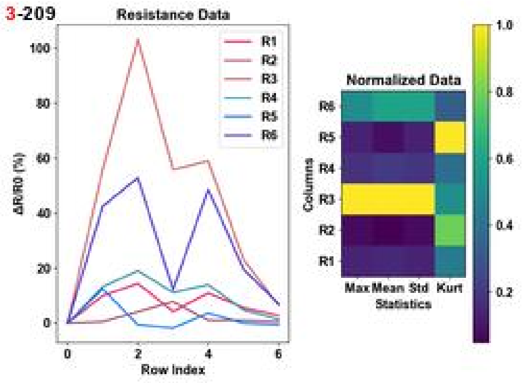

3-210

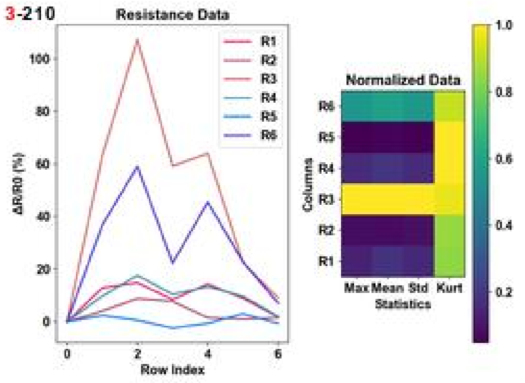

3-211

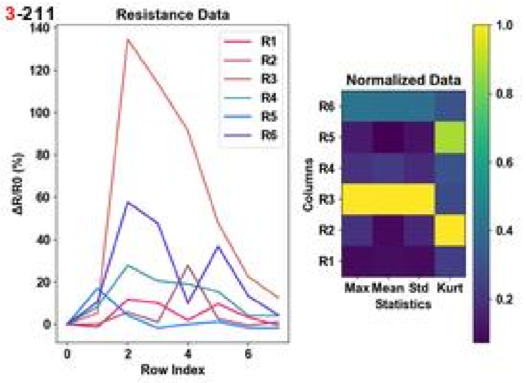

3-212

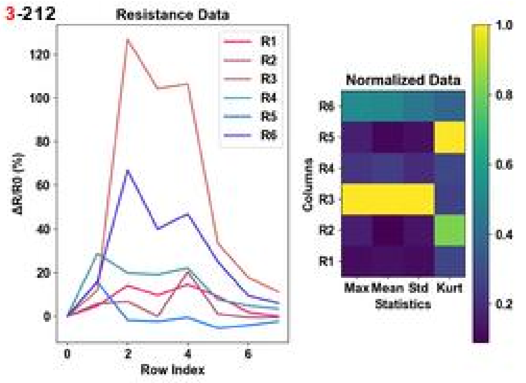

3-213

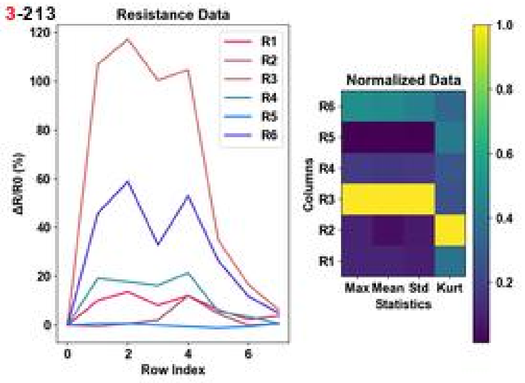

3-214

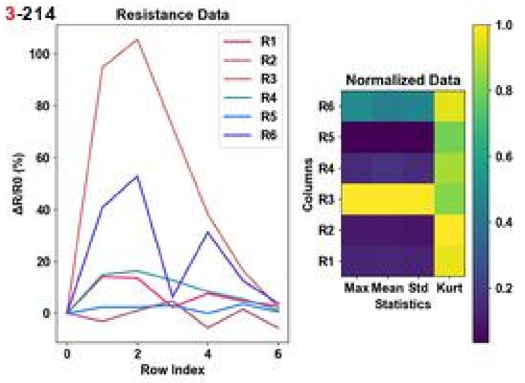

3-215

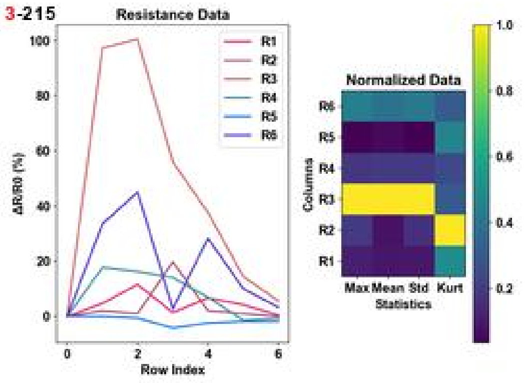

3-216

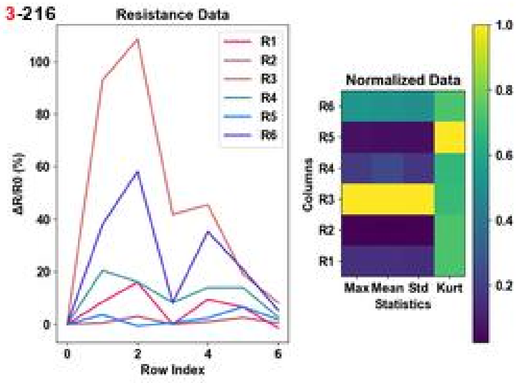

3-217

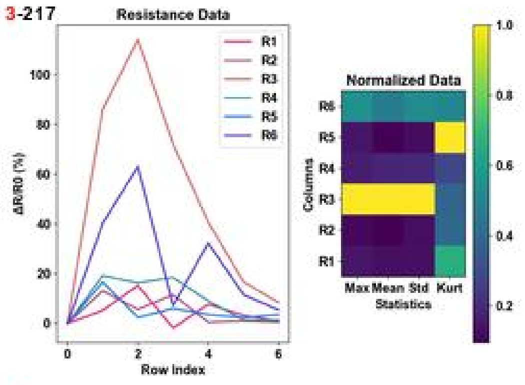

3-218

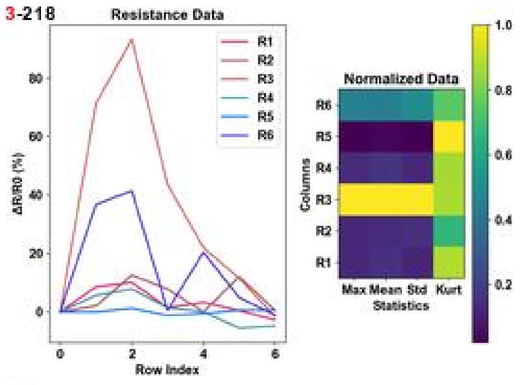

3-219

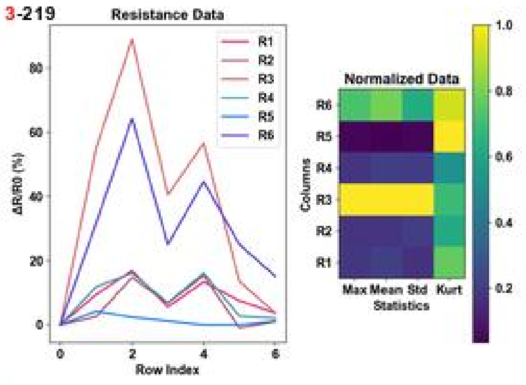

3-220

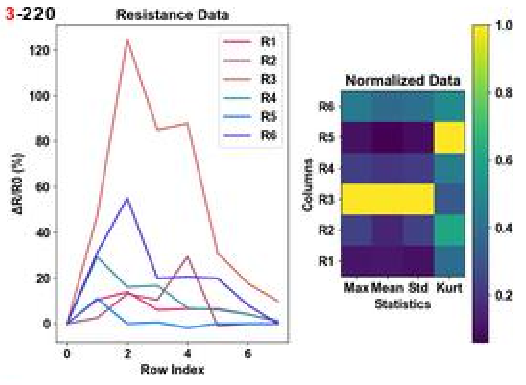

3-221

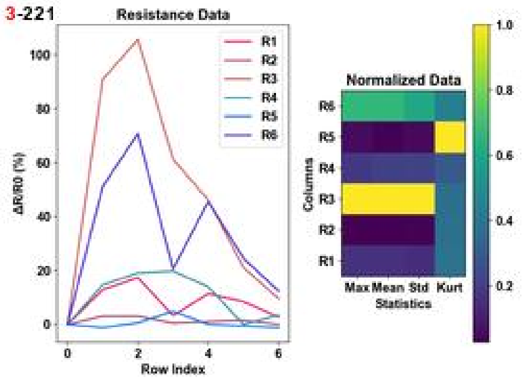

3-222

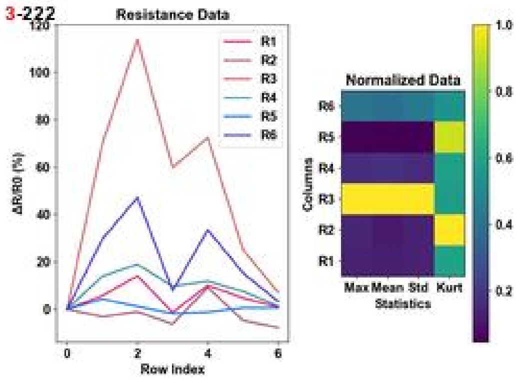

3-223

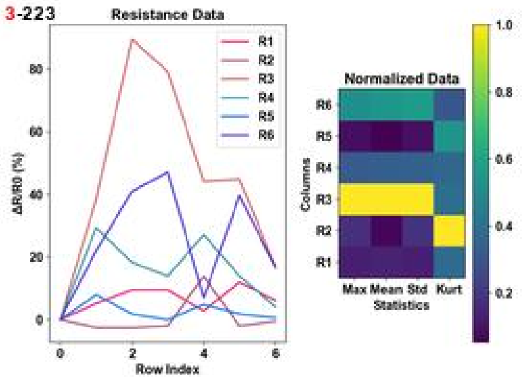

3-224

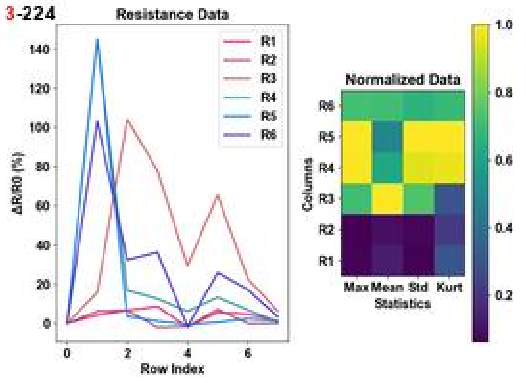

3-225

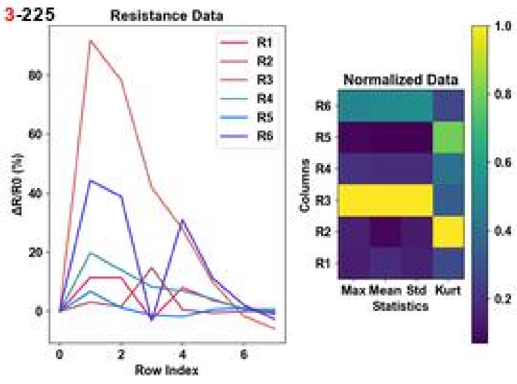

3-226

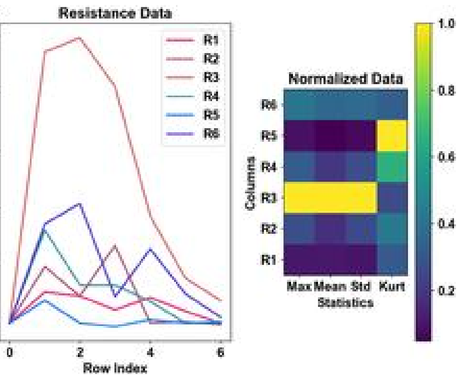

3-227

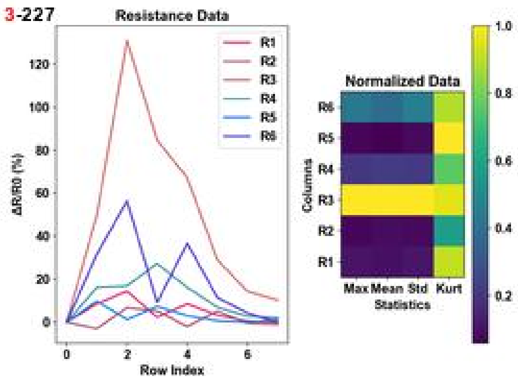

3-228

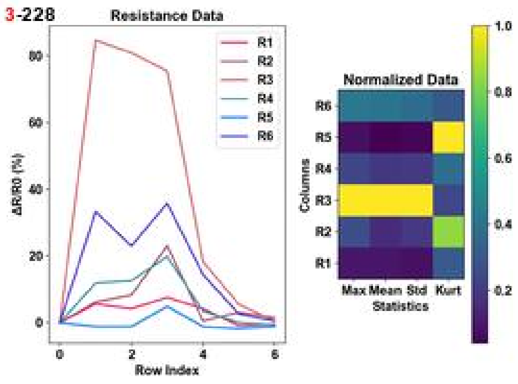

3-229

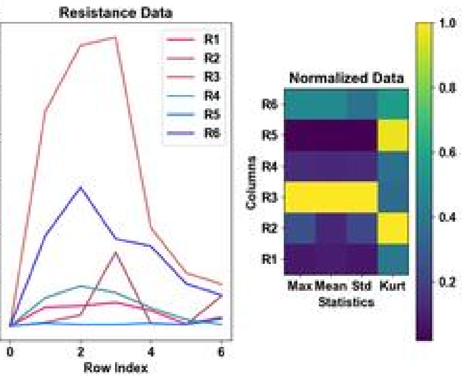

3-230

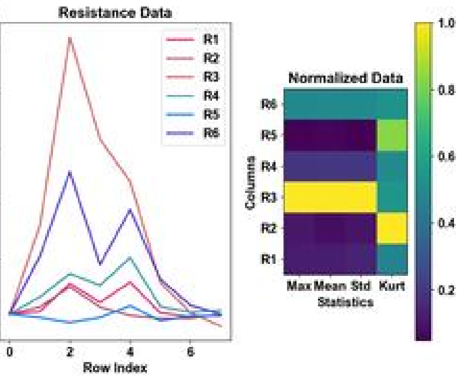

3-231

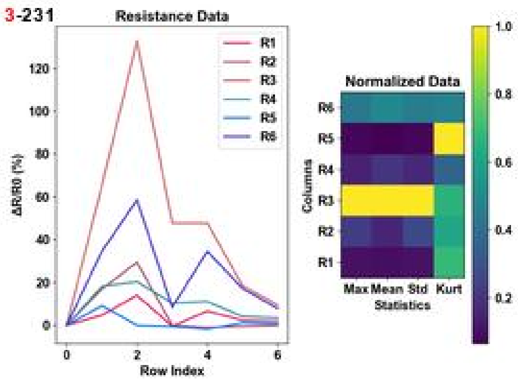

3-232

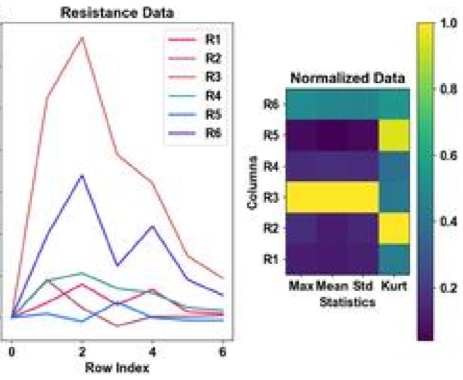

3-233

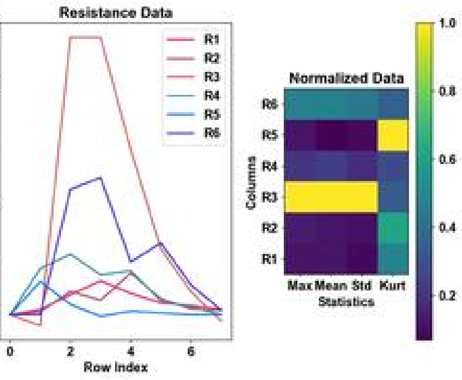

3-234

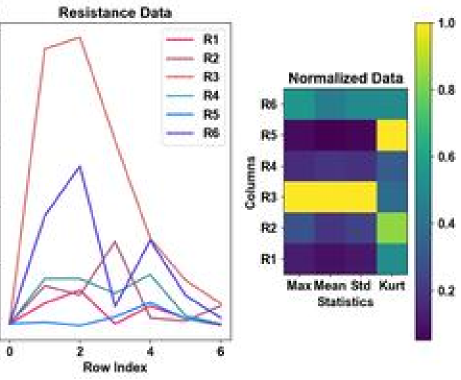

3-235

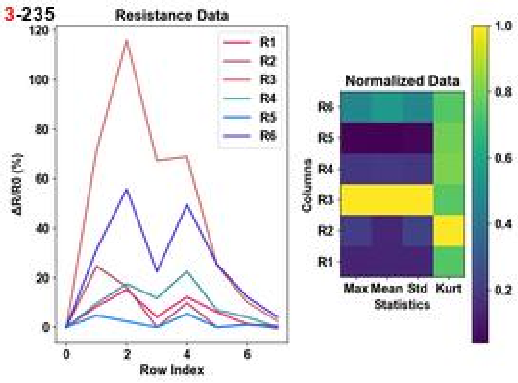

3-236

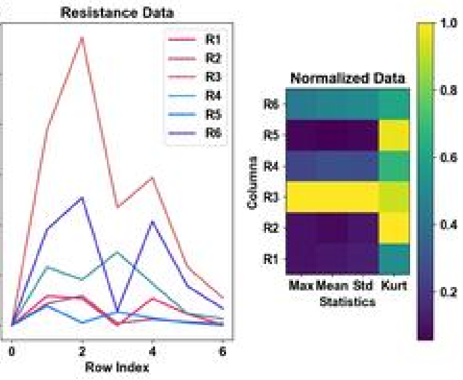

3-237

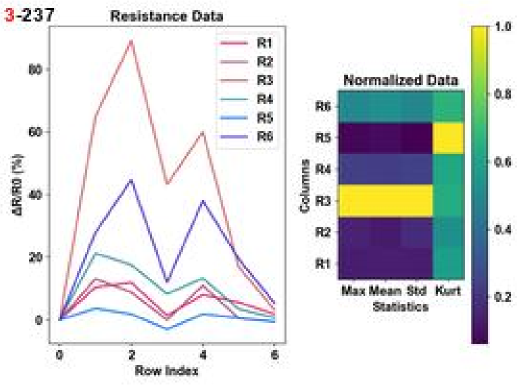

3-238

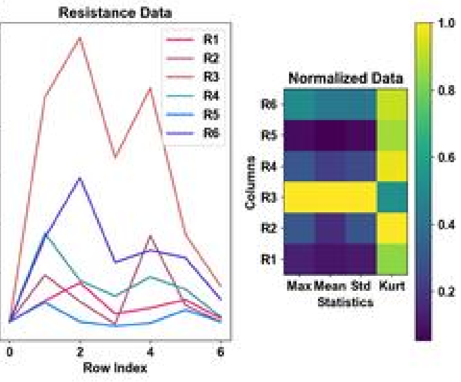

3-239

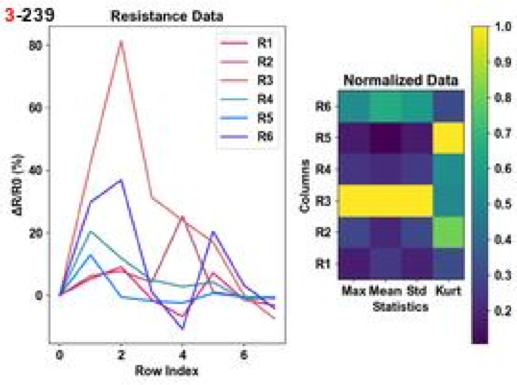

3-240

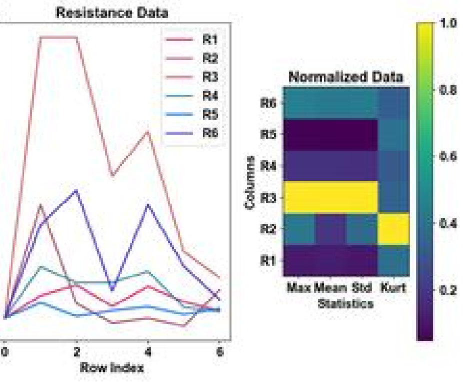

Pressure-sensitive data of type 4

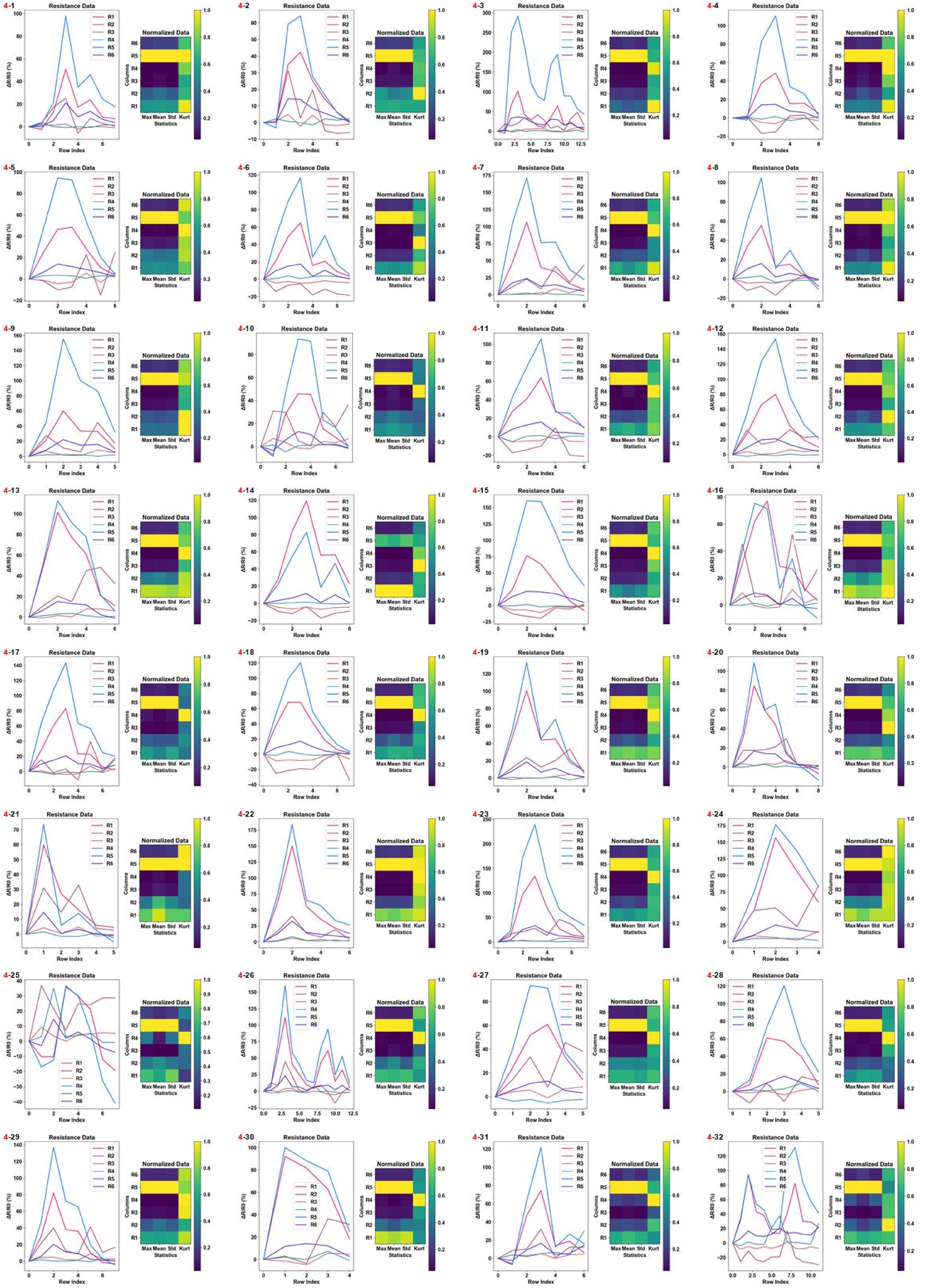

4-33
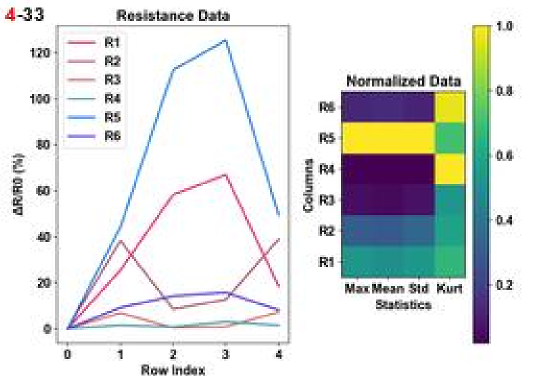

4-34
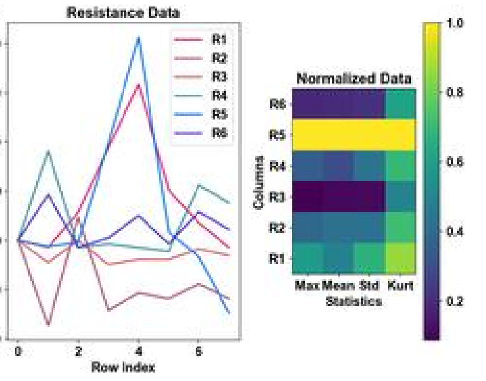

4-35
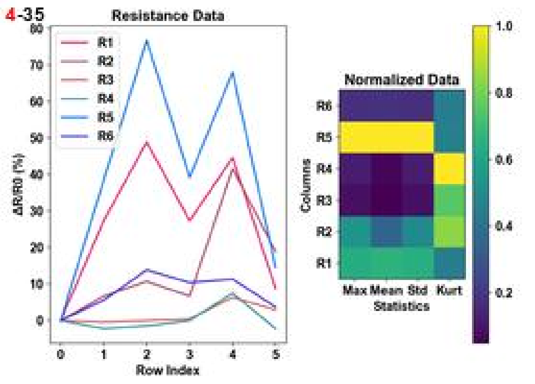

4-36
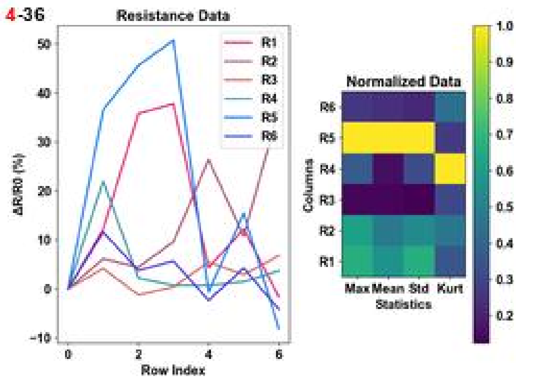

4-37
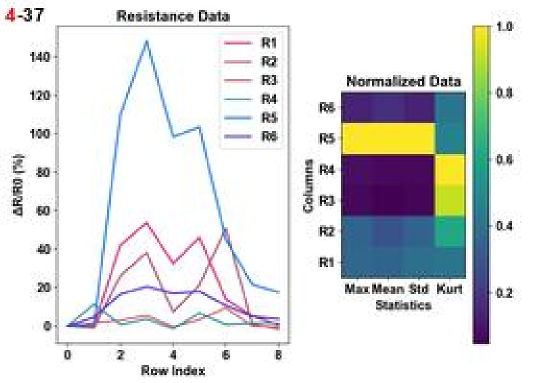

4-38
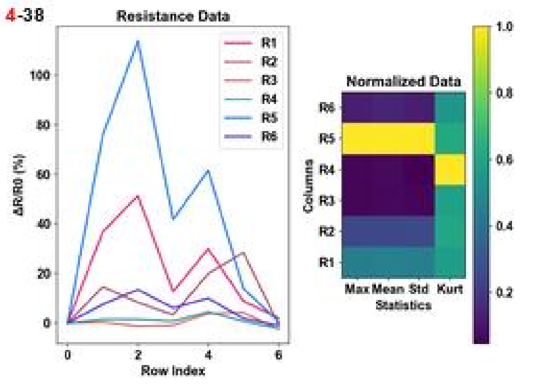

4-39
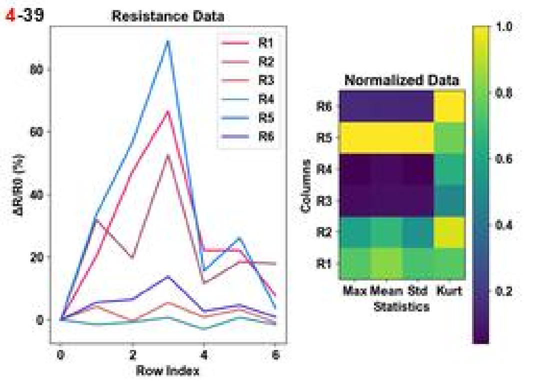

4-40
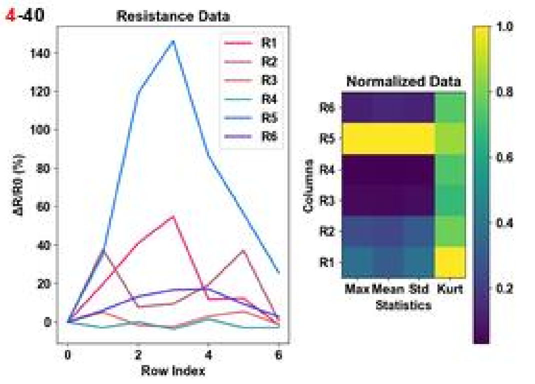

4-41
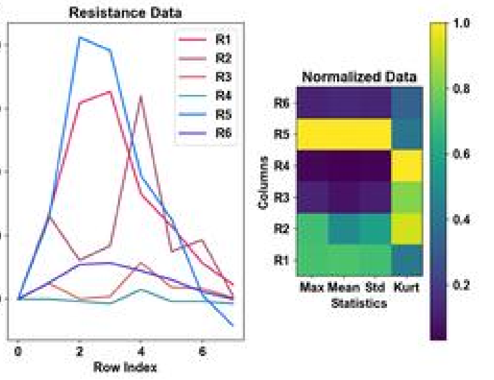

4-42
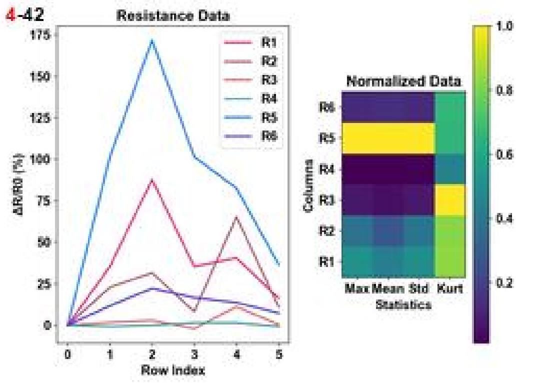

4-43
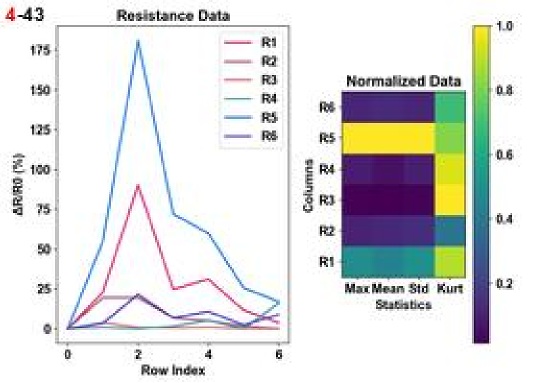

4-44
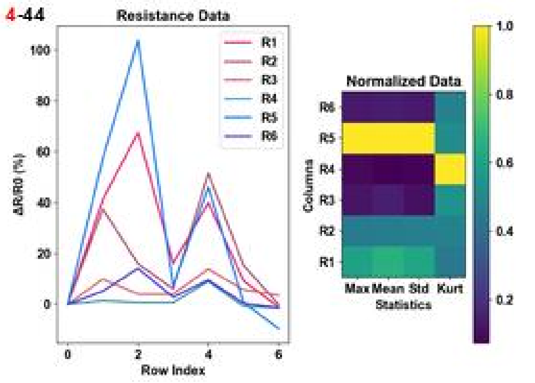

4-45
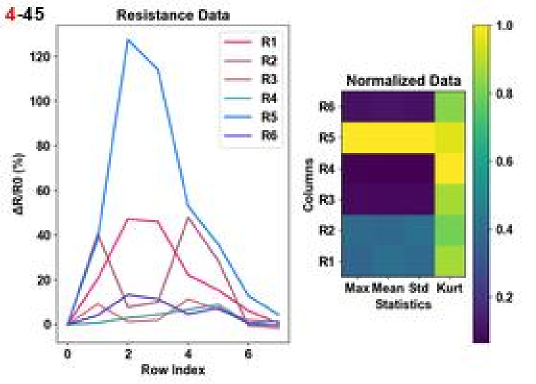

4-46
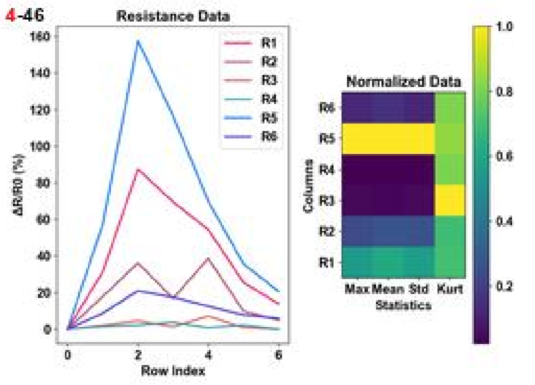

4-47
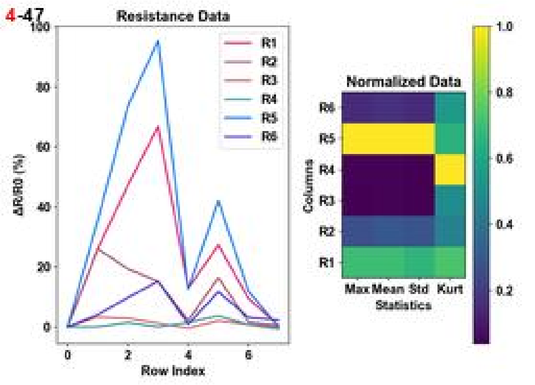

4-48
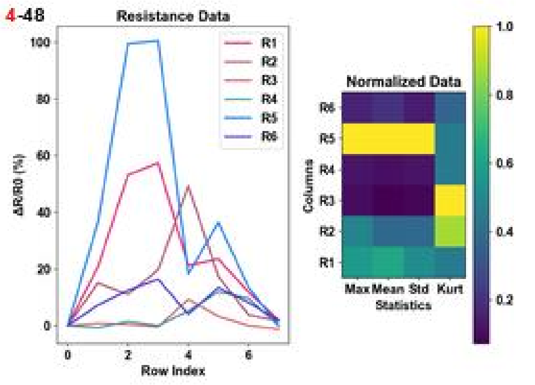

4-49
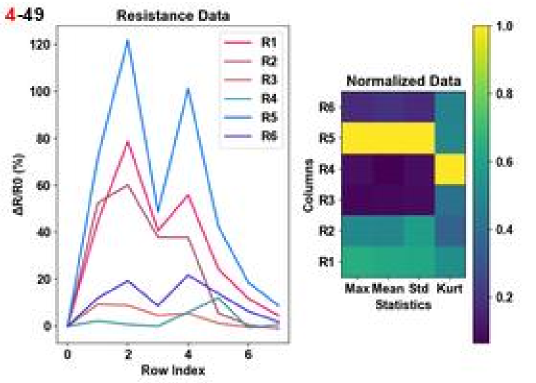

4-50
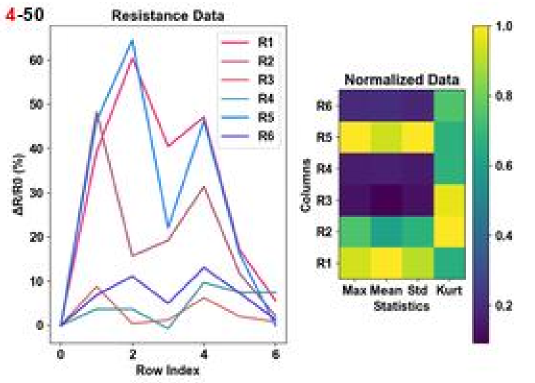

4-51
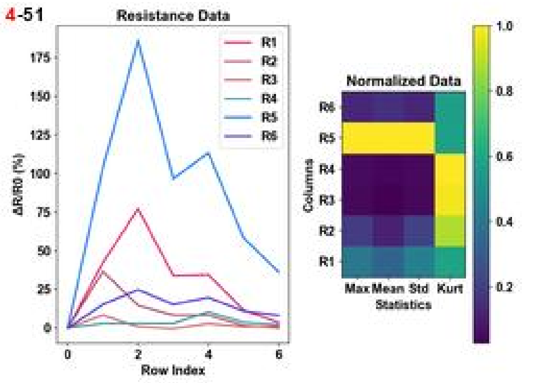

4-52
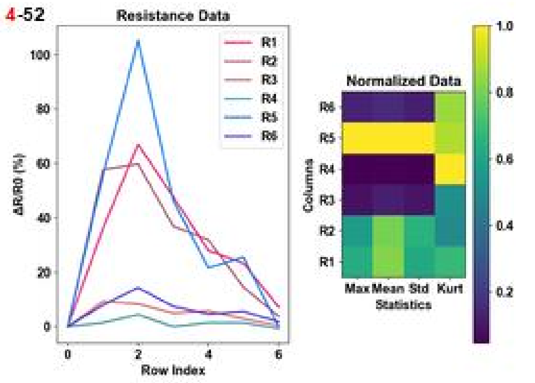

4-53
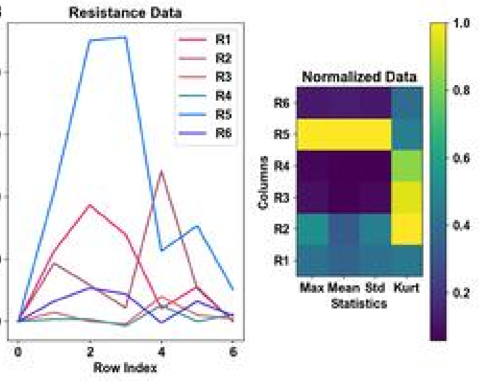

4-54
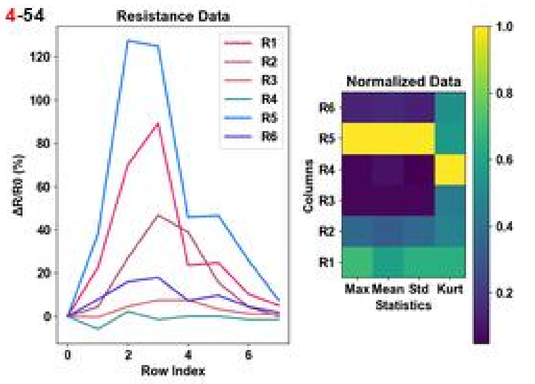

4-55
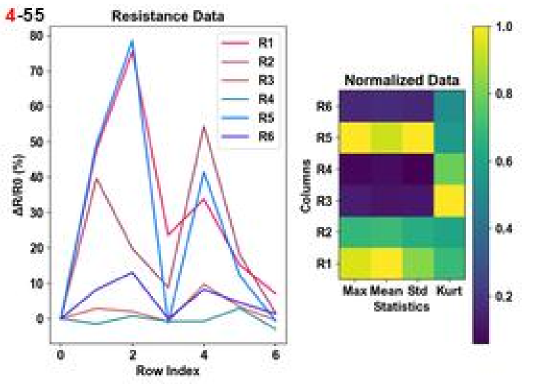

4-56
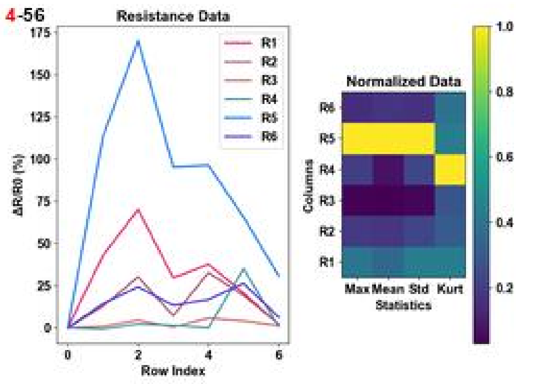

4-57
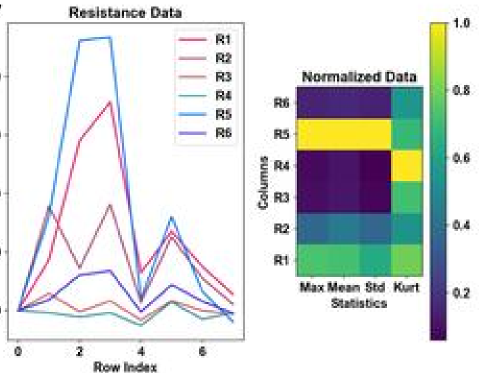

4-58
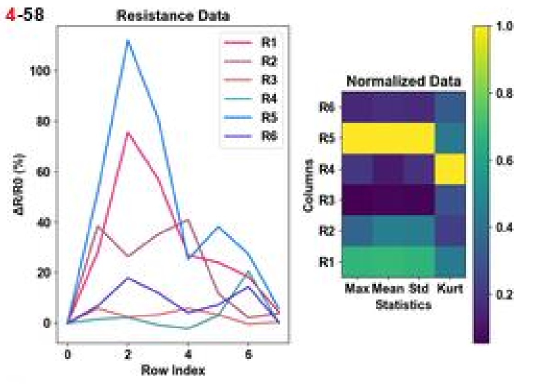

4-59
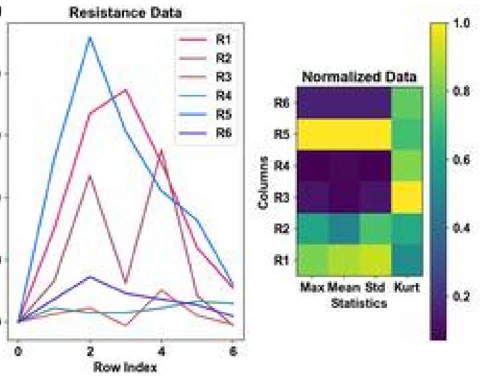

4-60
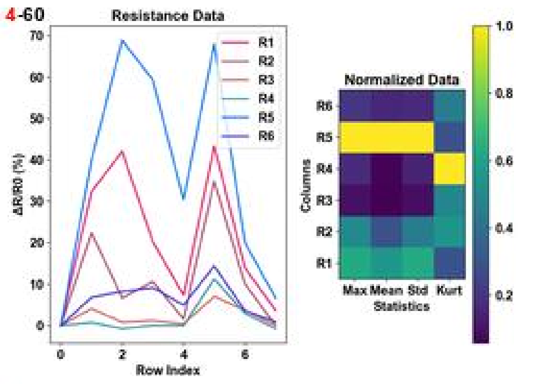

4-61
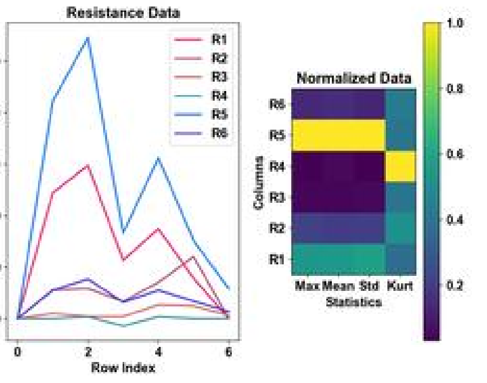

4-62
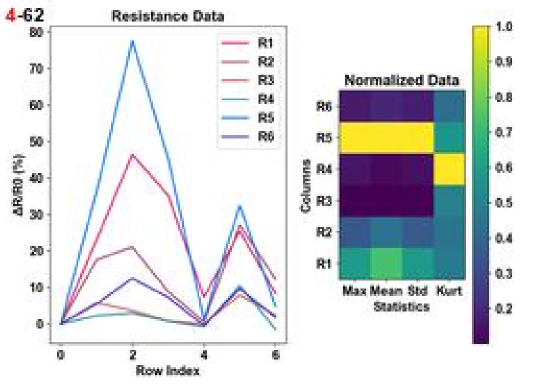

4-63
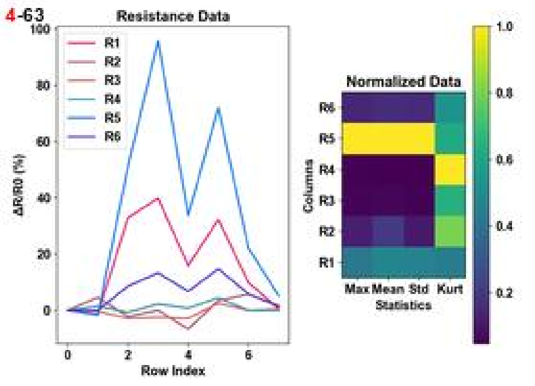

4-64
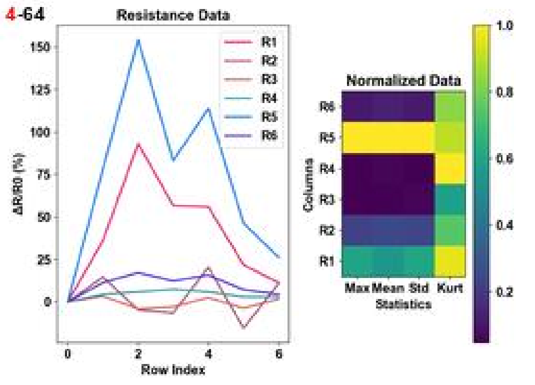

Pressure-sensitive data of type 4

4-65

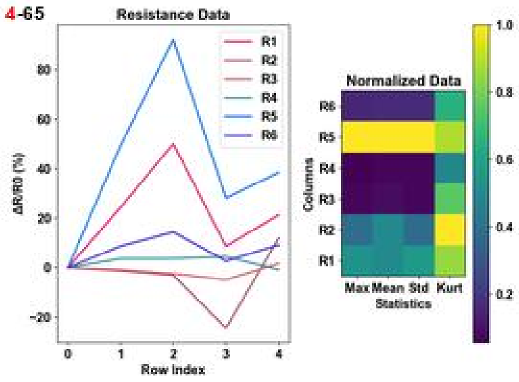

4-66

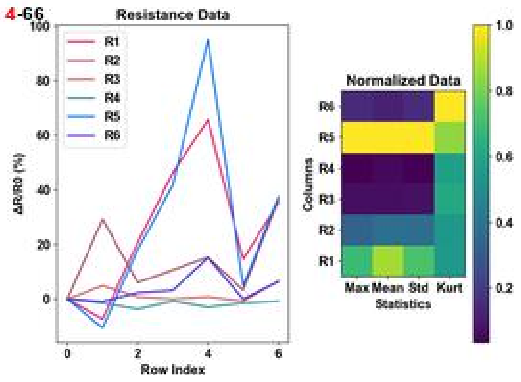

4-67

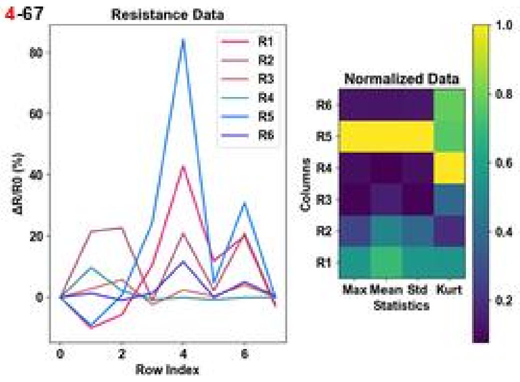

4-68

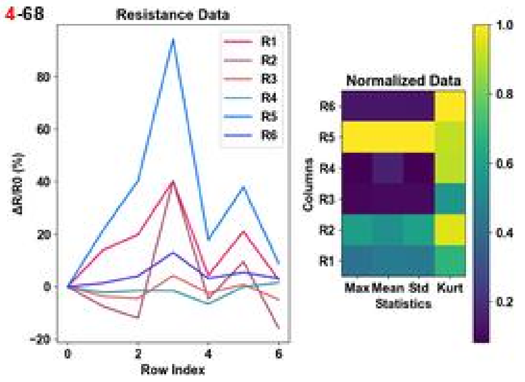

4-69

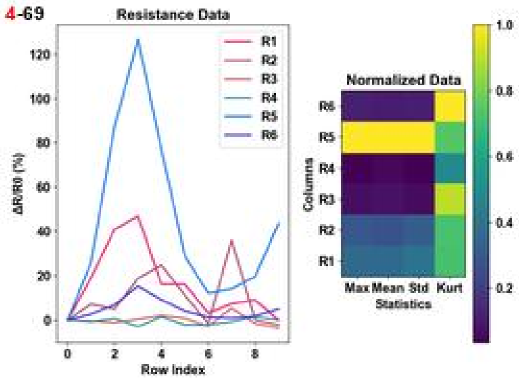

4-70

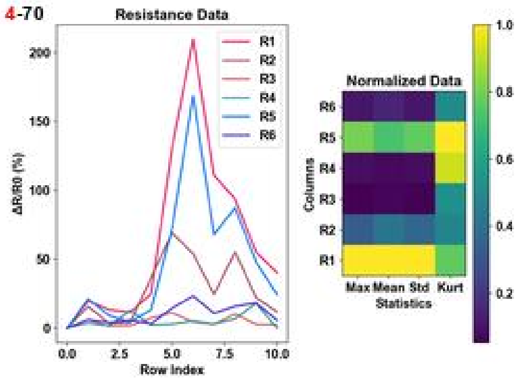

4-71

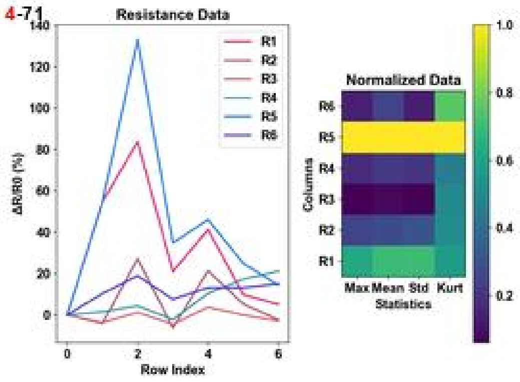

4-72

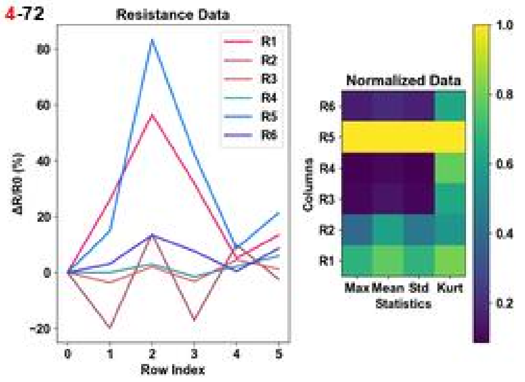

4-73

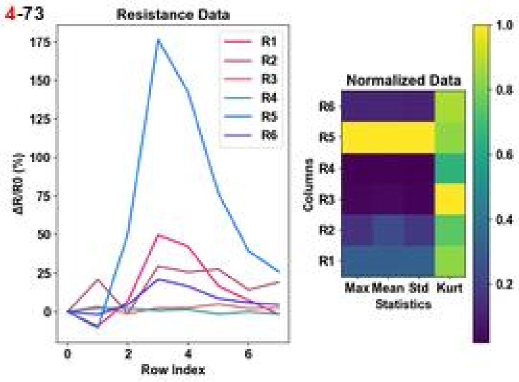

4-74

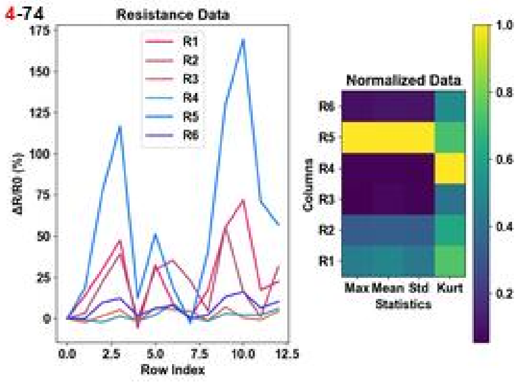

4-75

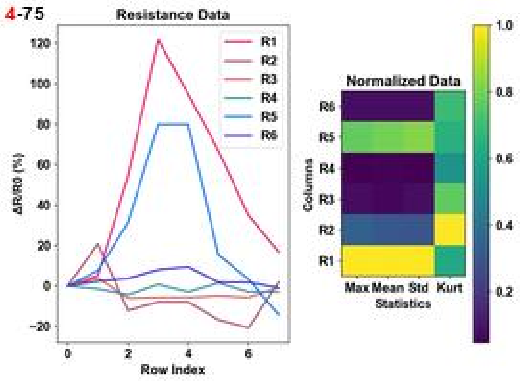

4-76

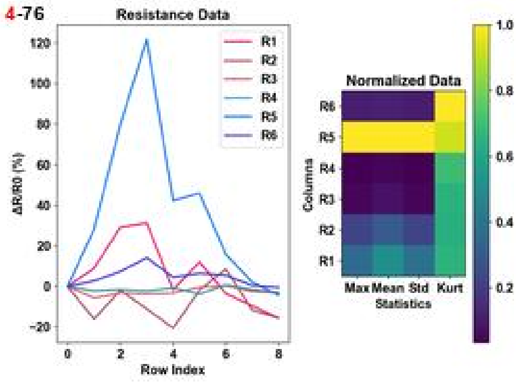

4-77

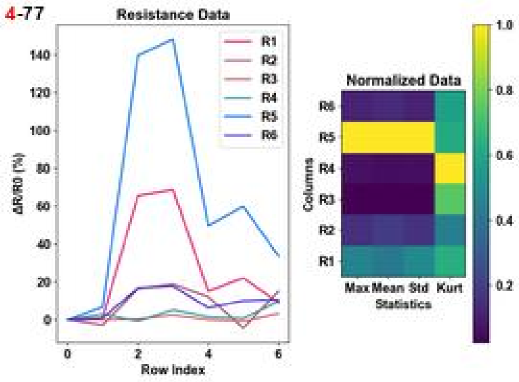

4-78

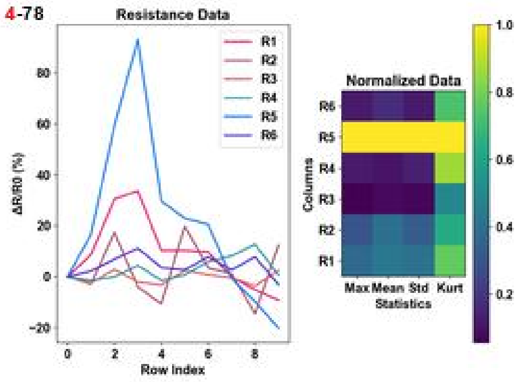

4-79

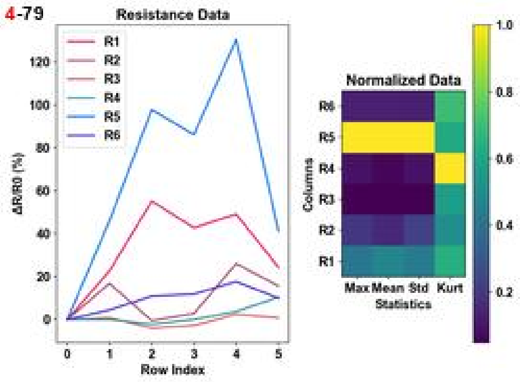

4-80

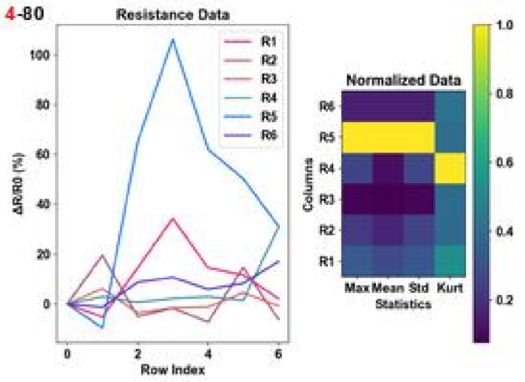

4-81

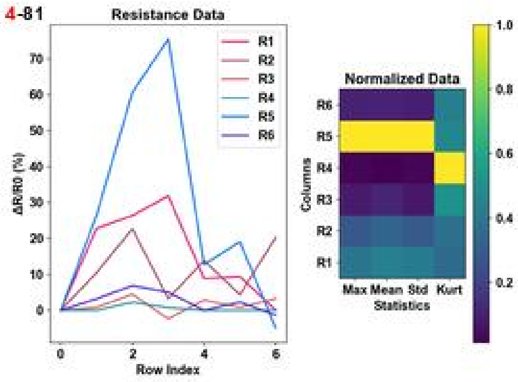

4-82

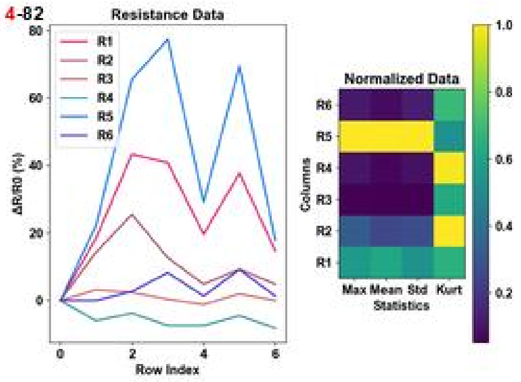

4-83

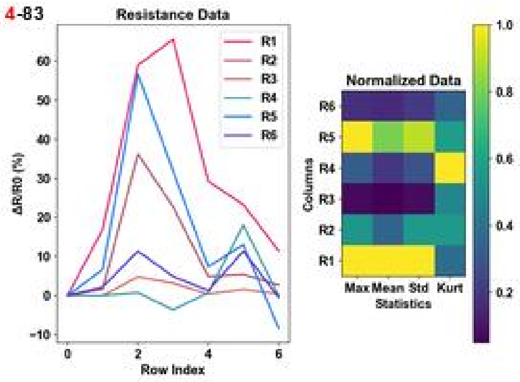

4-84

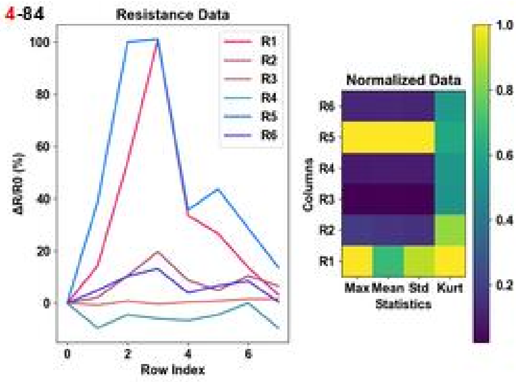

4-85

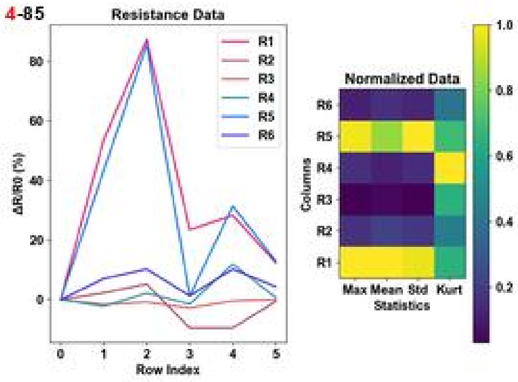

4-86

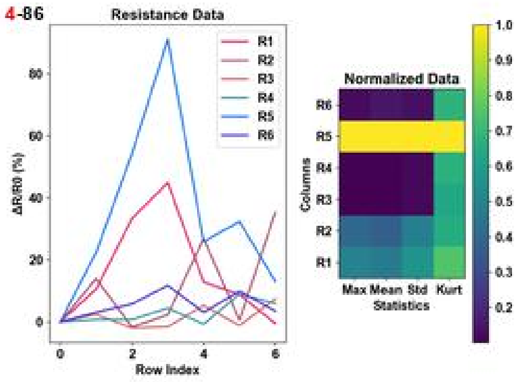

4-87

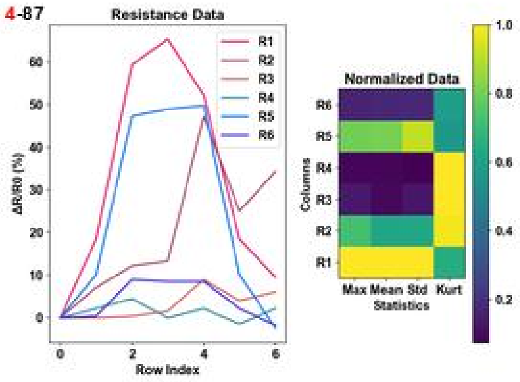

4-88

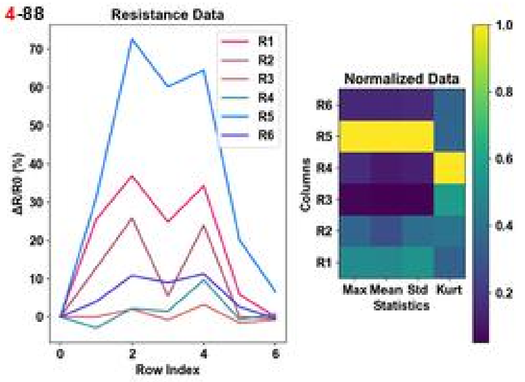

4-89

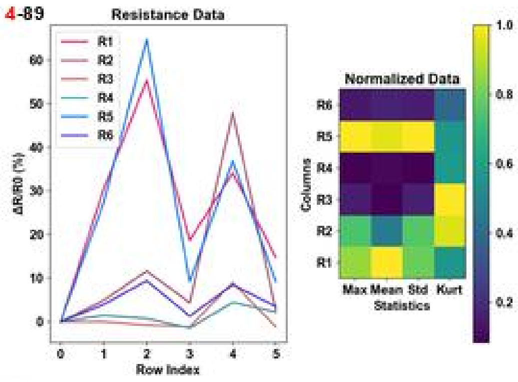

4-90

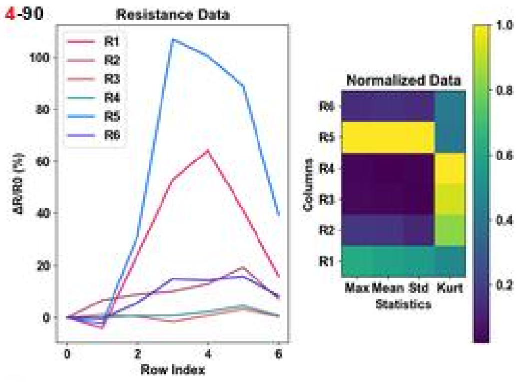

4-91

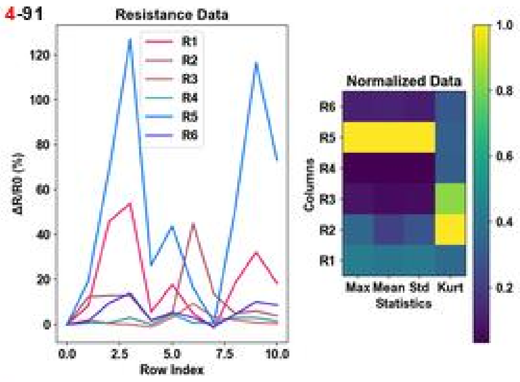

4-92

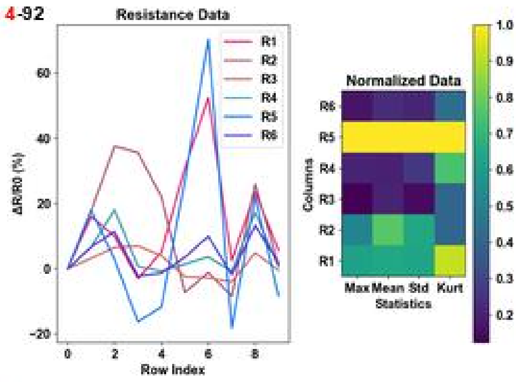

4-93

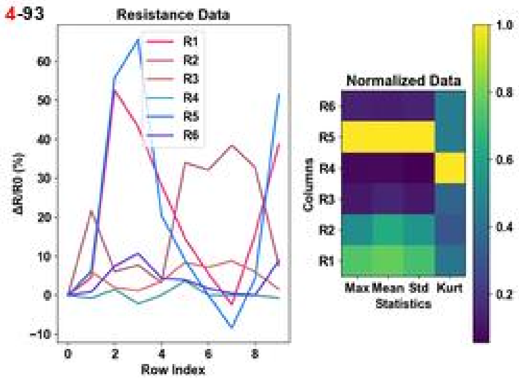

4-94

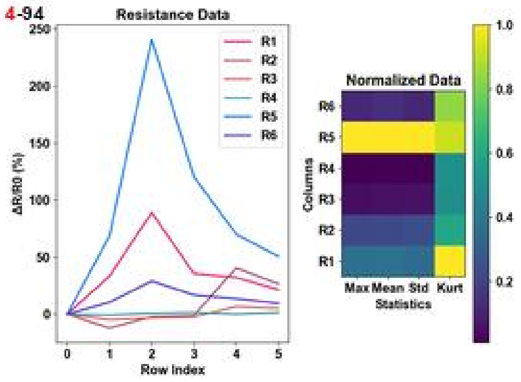

4-95

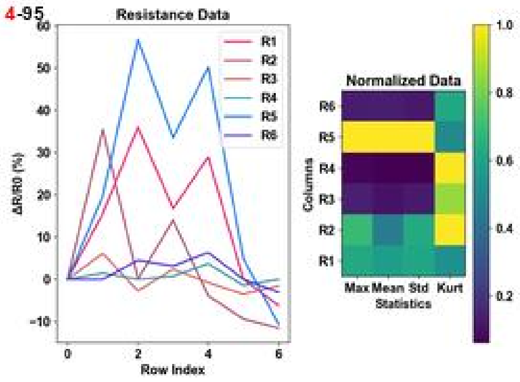

4-96

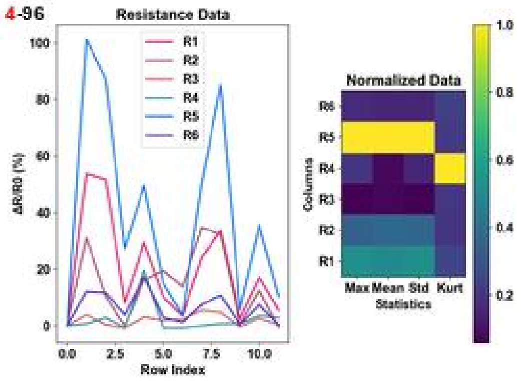

4-97
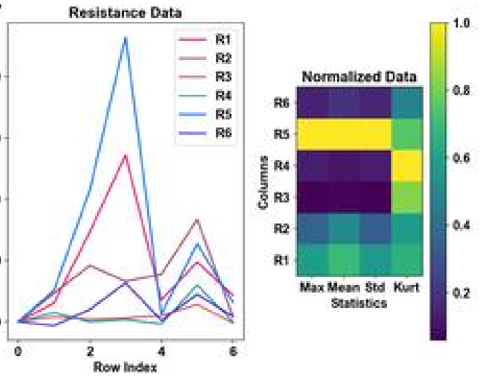

4-98
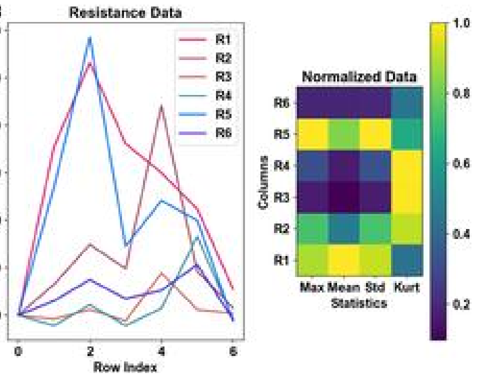

4-99
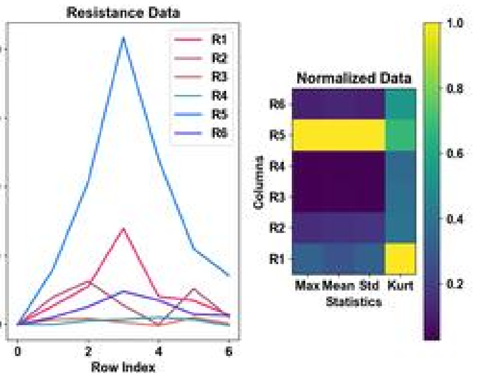

4-100
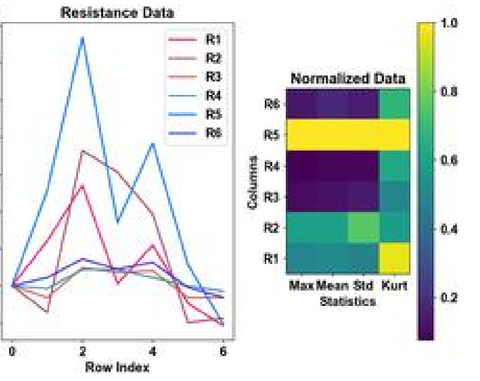

4-101
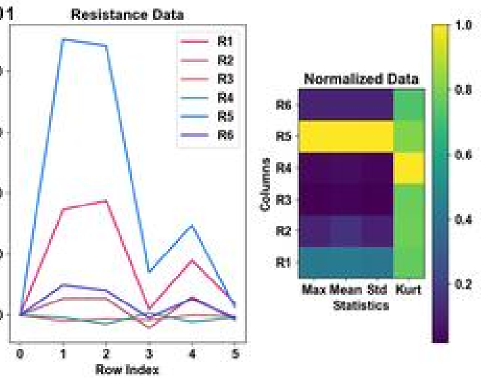

4-102
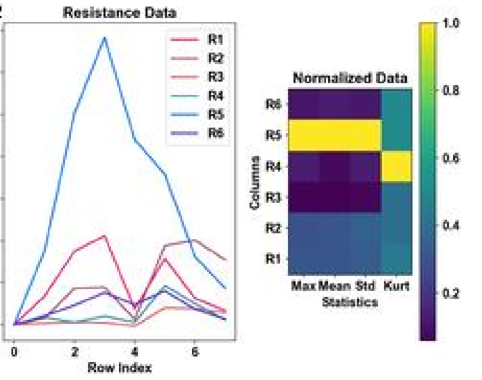

4-103
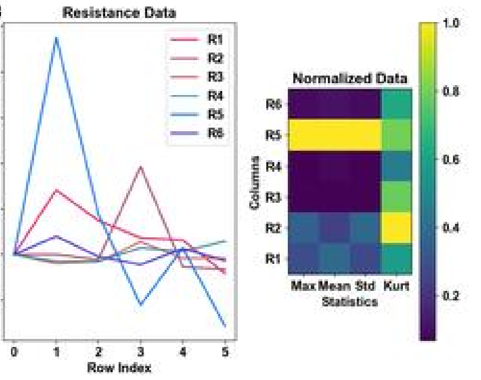

4-104
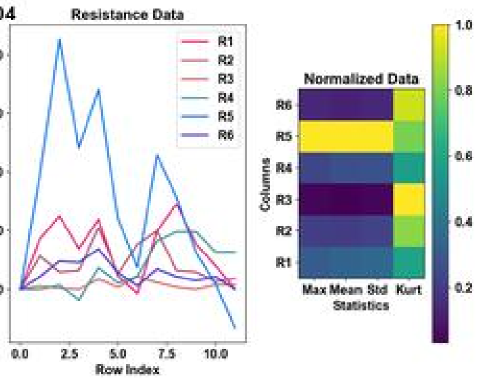

4-105
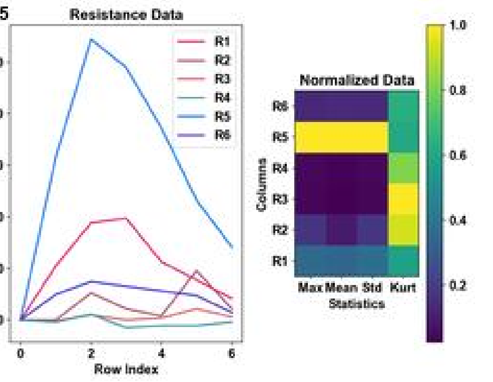

4-106
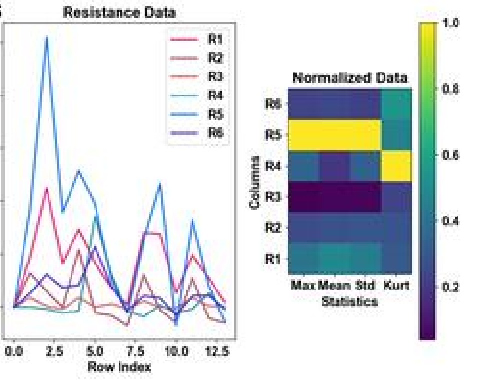

4-107
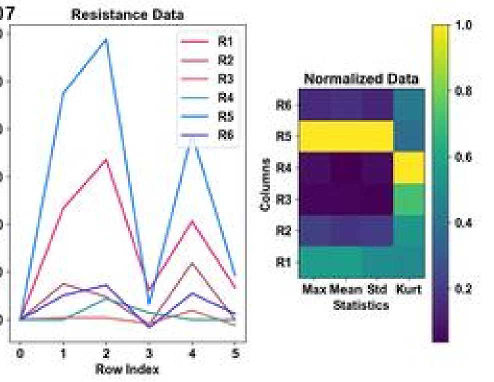

4-108
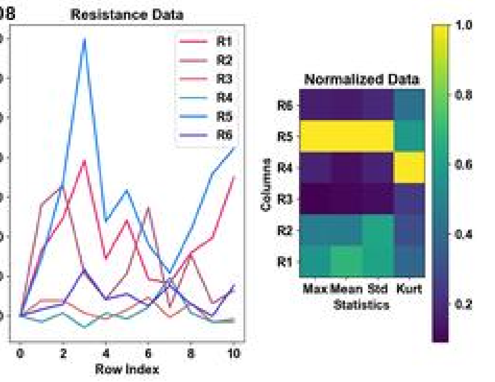

4-109
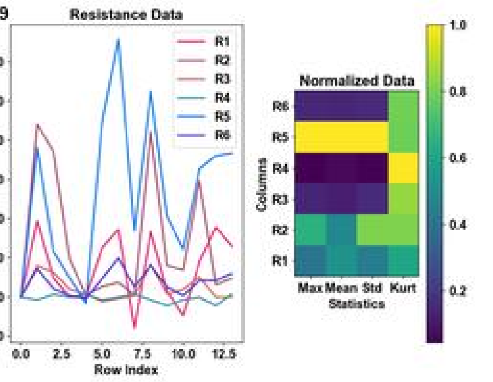

4-110
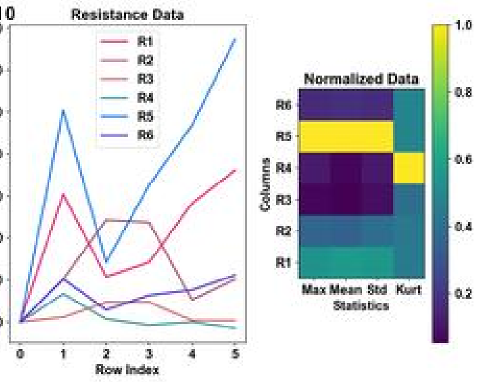

4-111
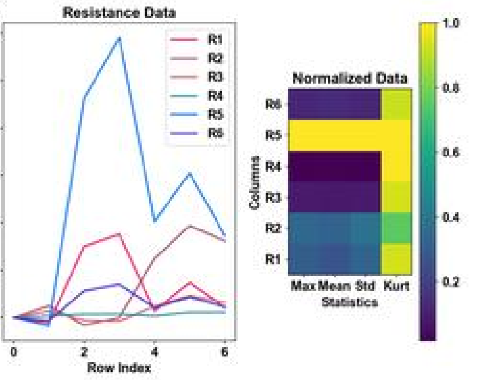

4-112
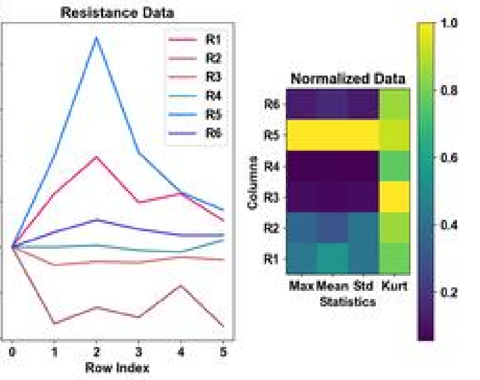

4-113
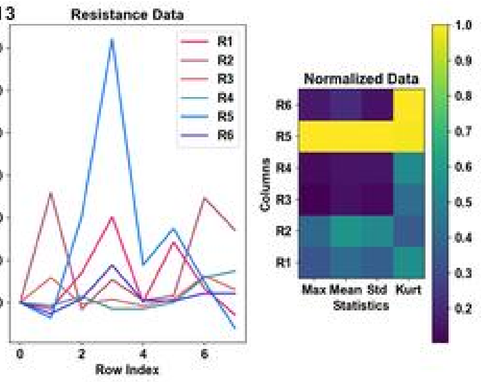

4-114
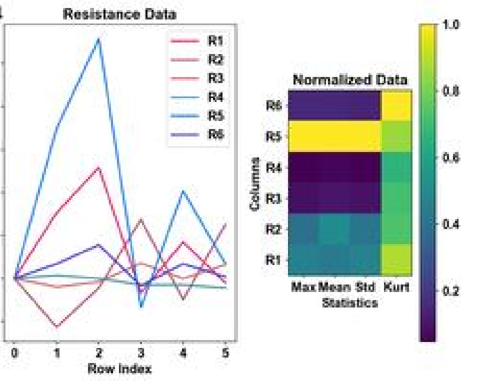

4-115
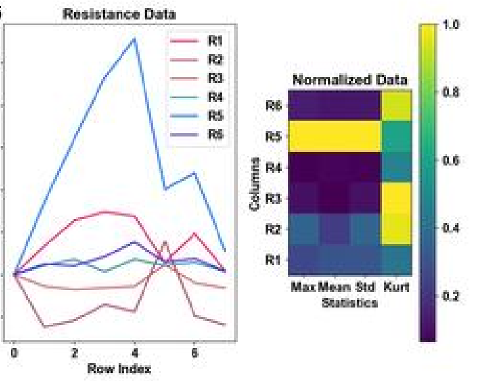

4-116
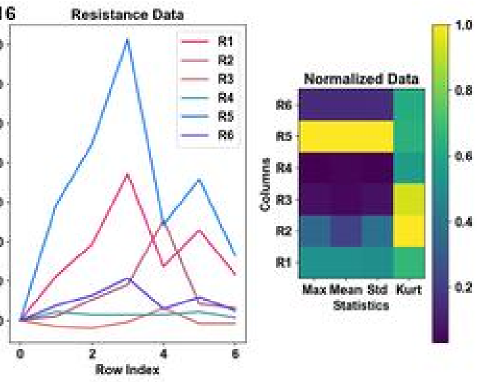

4-117
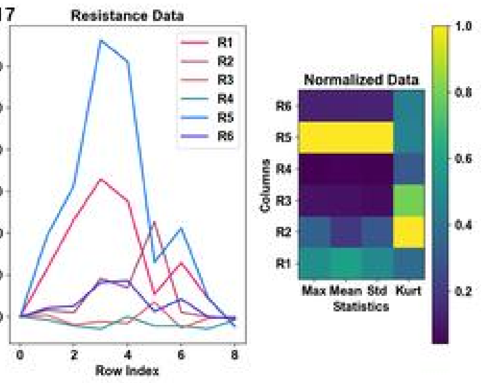

4-118
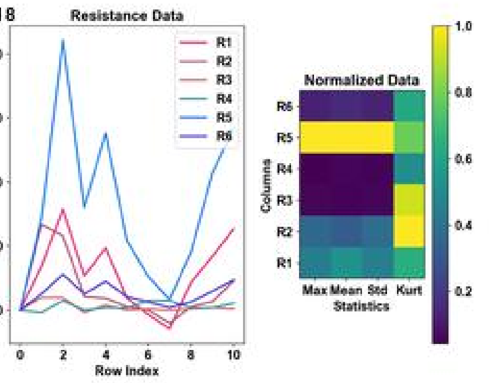

4-119
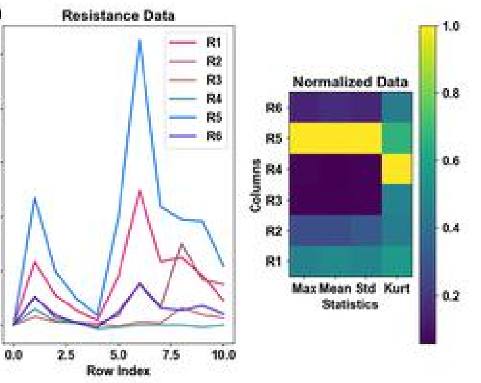

4-120
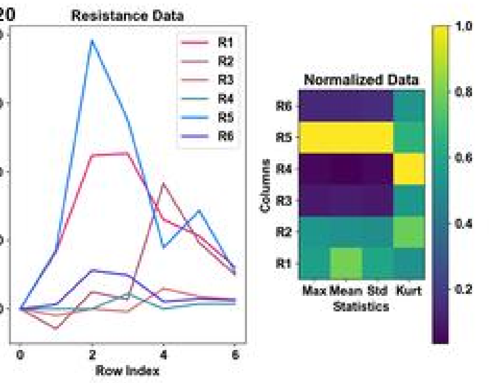

4-121
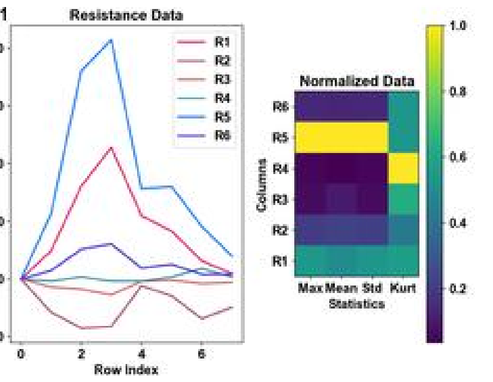

4-122
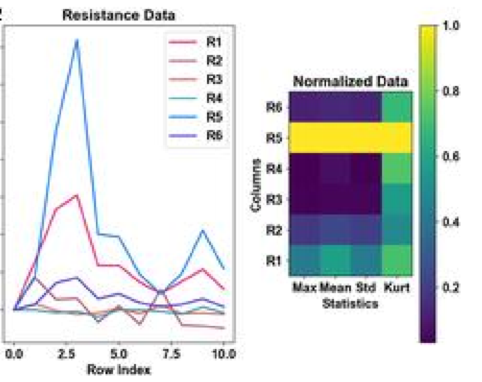

4-123
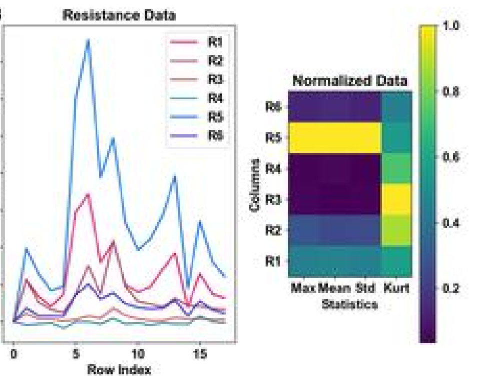

4-124
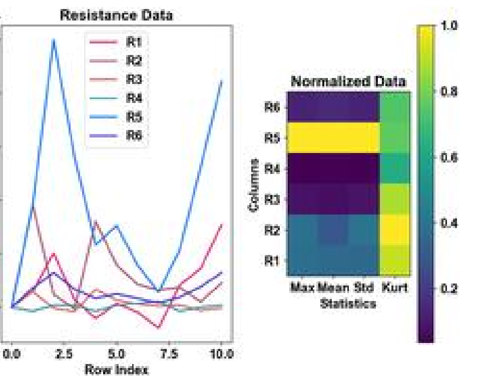

4-125
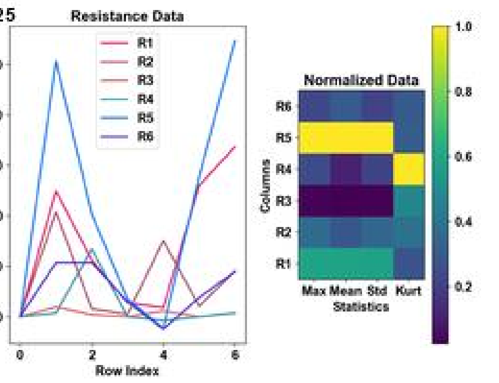

4-126
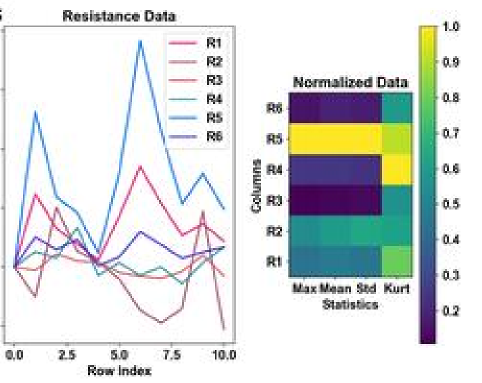

4-127
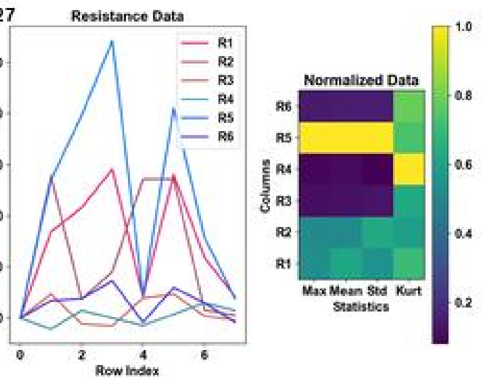

4-128
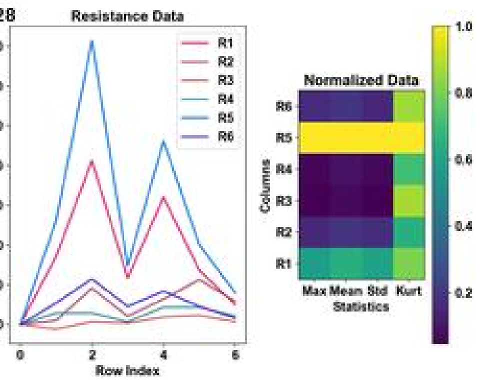

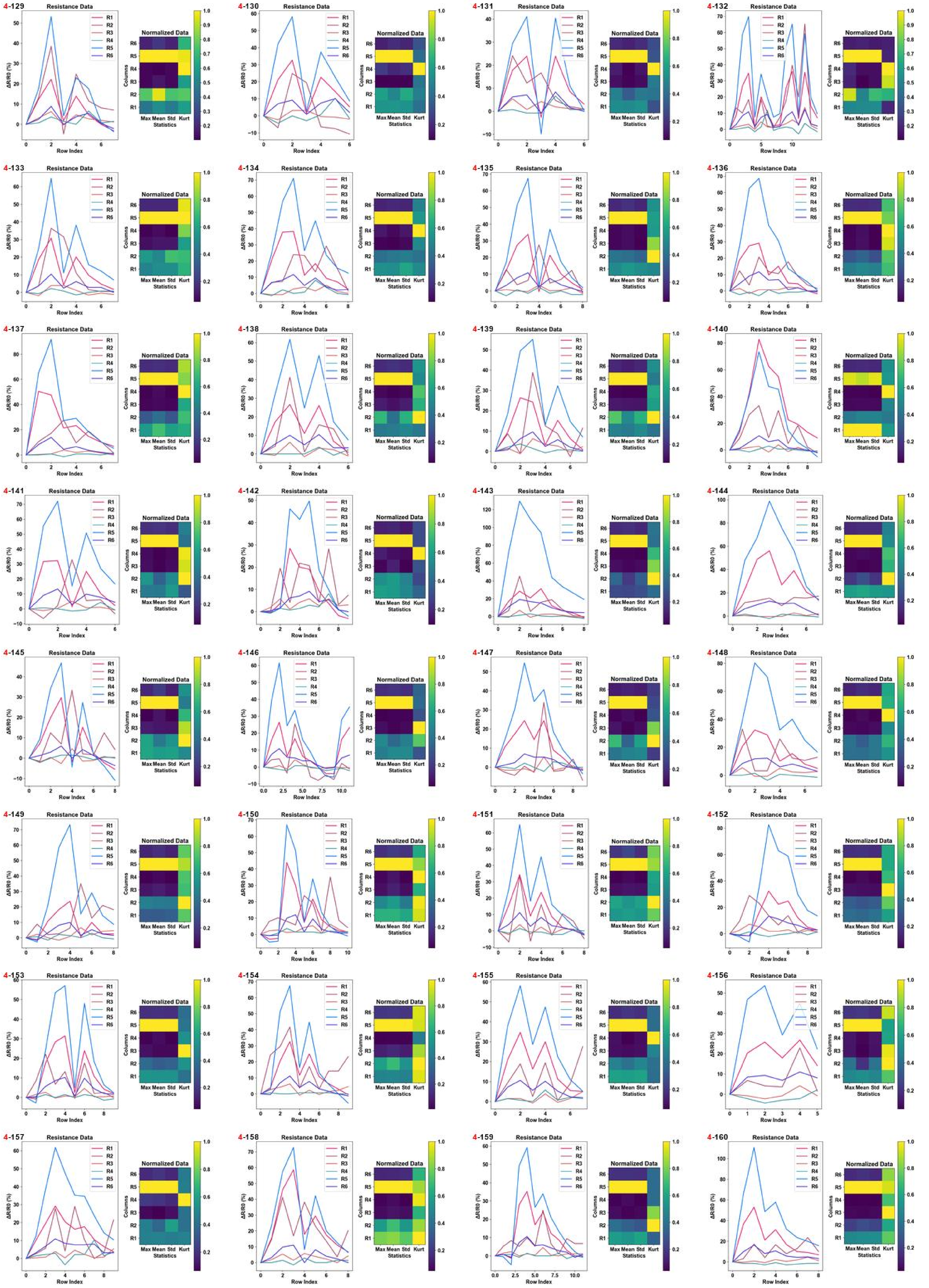
4-129
4-130
4-131
4-132
4-133
4-134
4-135
4-136
4-137
4-138
4-139
4-140
4-141
4-142
4-143
4-144
4-145
4-146
4-147
4-148
4-149
4-150
4-151
4-152
4-153
4-154
4-155
4-156
4-157
4-158
4-159
4-160
Resistance Data
Normalized Data
ΔR/R0 (%)
Row Index
Columns
Statistics
Max Mean Std Kurt
R1
R2
R3
R4
R5
R6

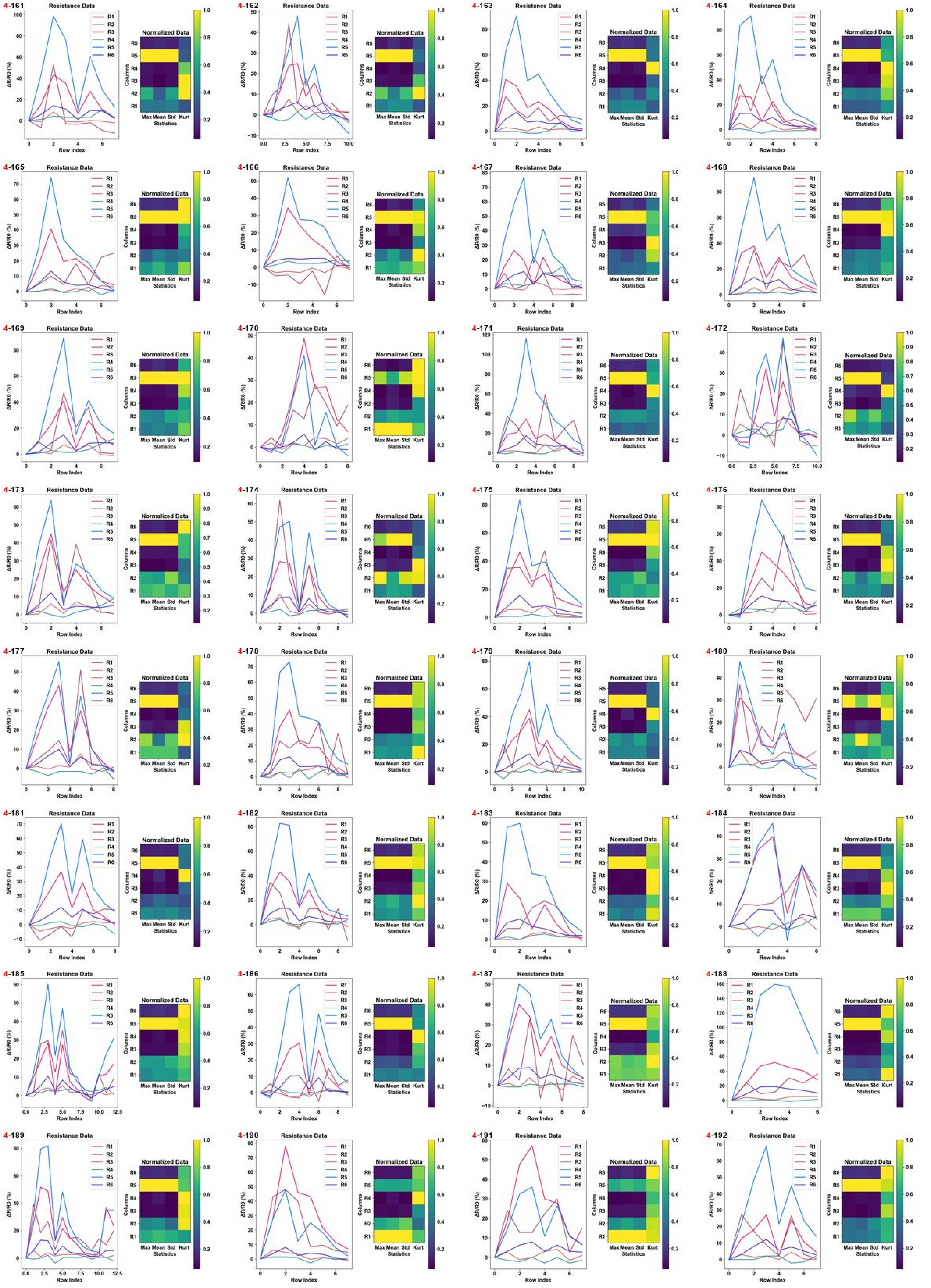
4-161
4-162
4-163
4-164
4-165
4-166
4-167
4-168
4-169
4-170
4-171
4-172
4-173
4-174
4-175
4-176
4-177
4-178
4-179
4-180
4-181
4-182
4-183
4-184
4-185
4-186
4-187
4-188
4-189
4-190
4-191
4-192
Resistance Data
Normalized Data
ΔR/R0 (%)
Row Index
Columns
Statistics
Max Mean Std Kurt
R1
R2
R3
R4
R5
R6

Pressure-sensitive data of type 4

4-193
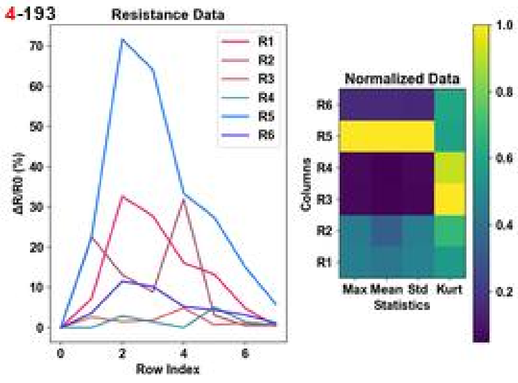

4-194
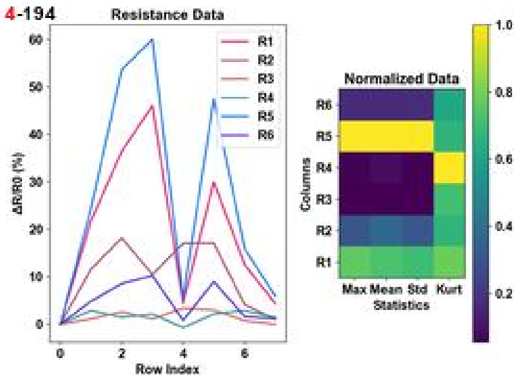

4-195
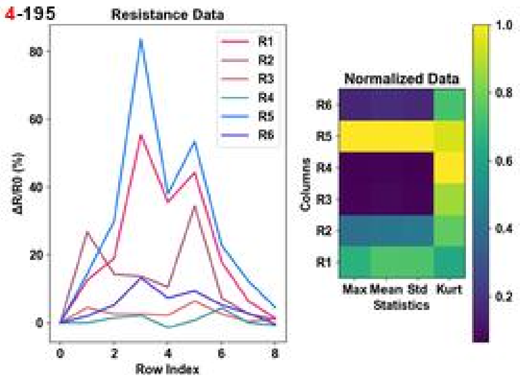

4-196
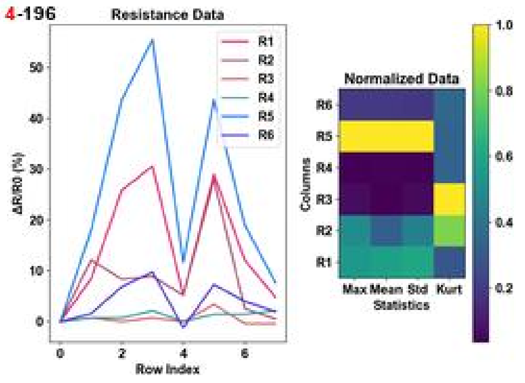

4-197
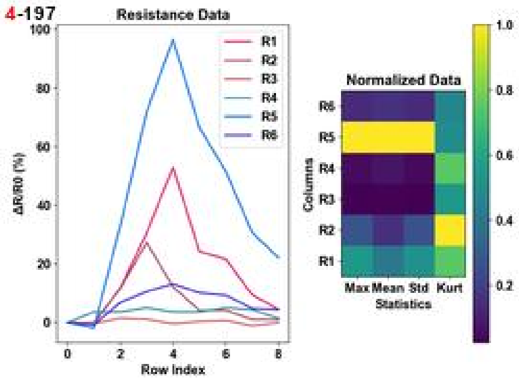

4-198
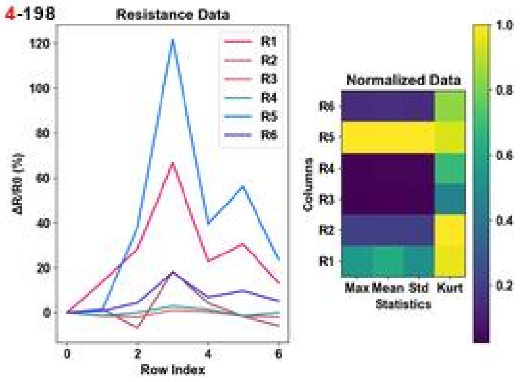

4-199
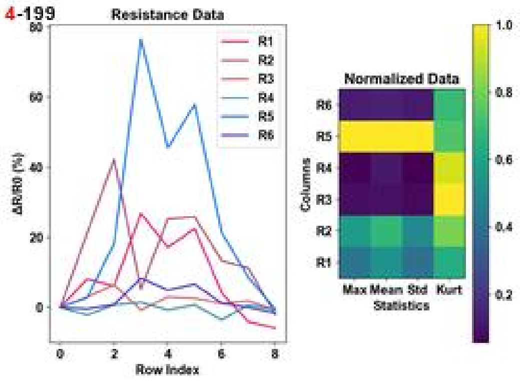

4-200
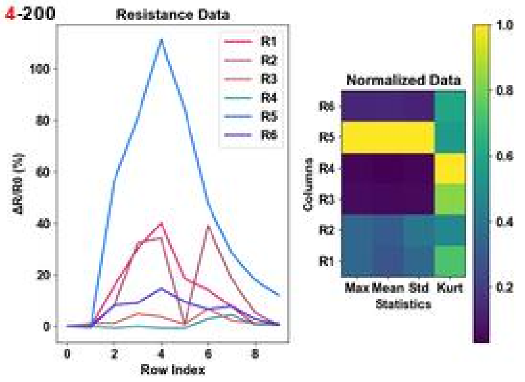

4-201
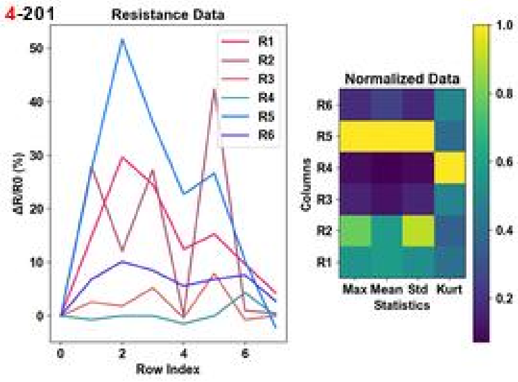

4-202
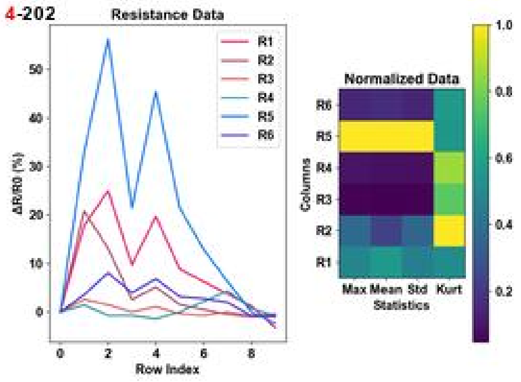

4-203
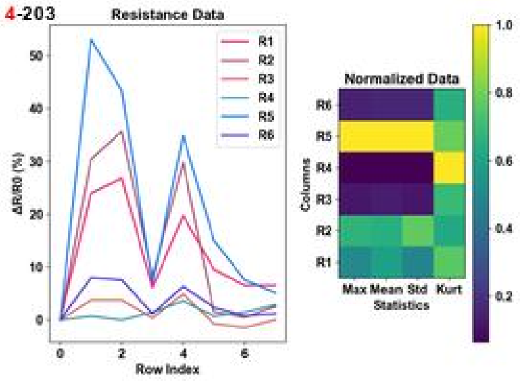

4-204
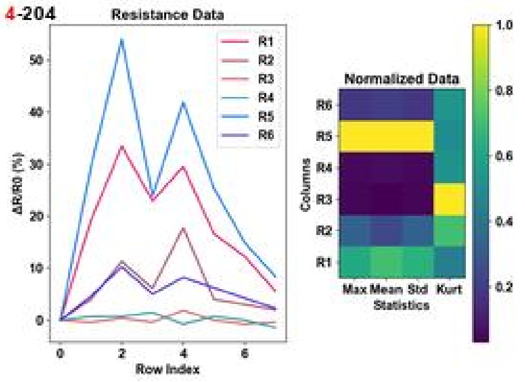

4-205
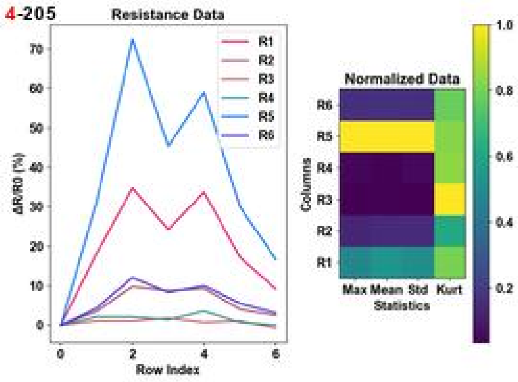

4-206
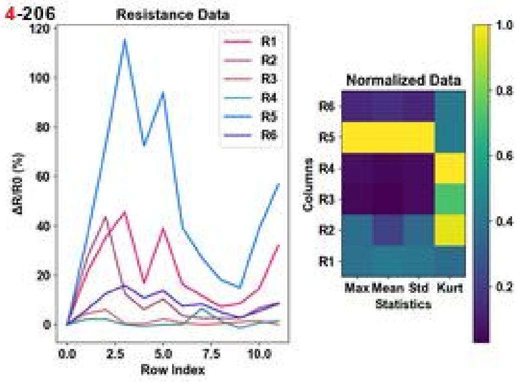

4-207
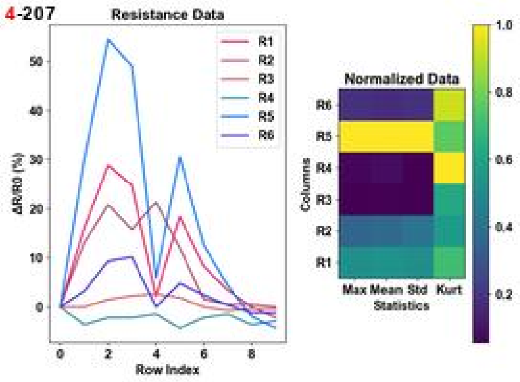

4-208
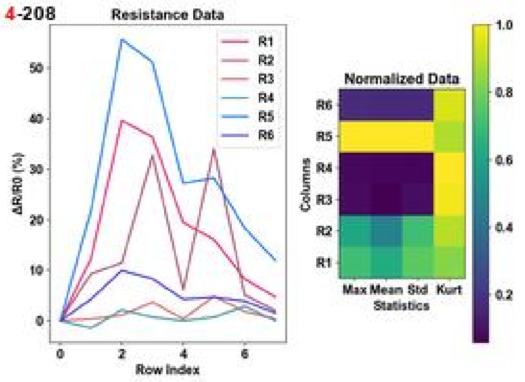

4-209
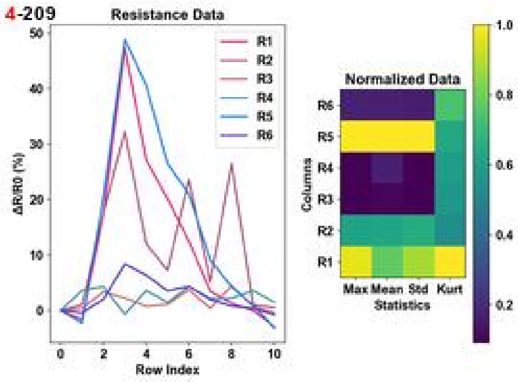

4-210
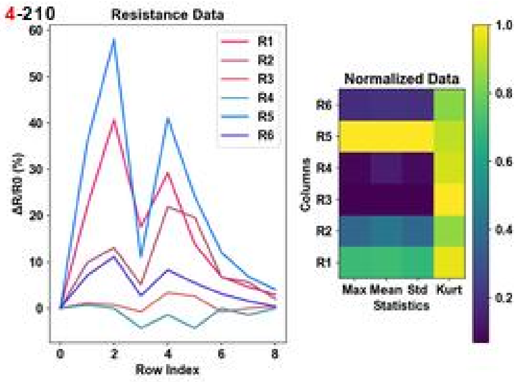

4-211
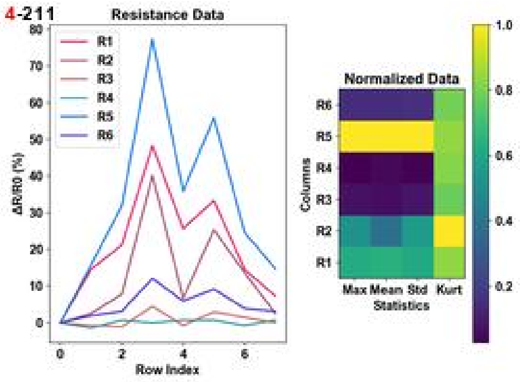

4-212
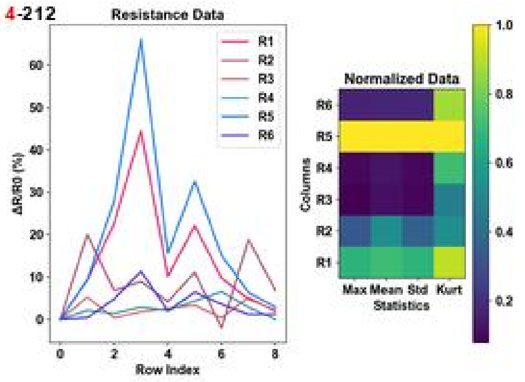

4-213
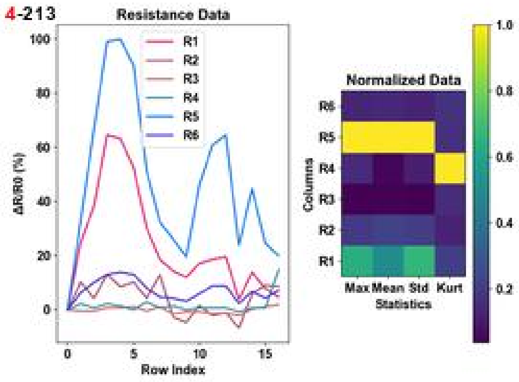

4-214
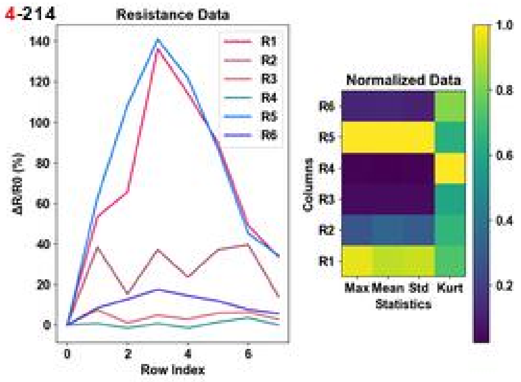

4-215
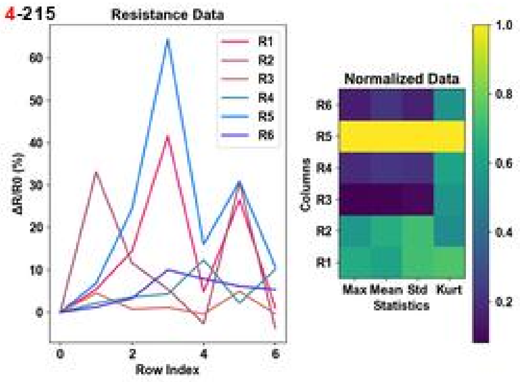

4-216
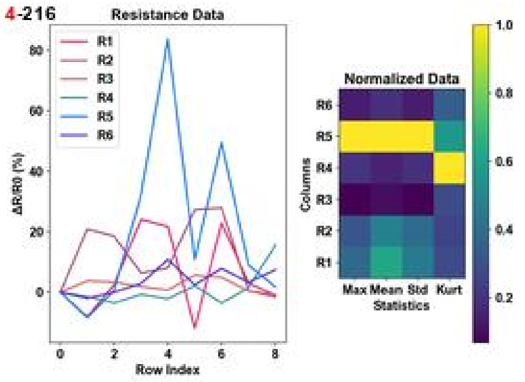

4-217
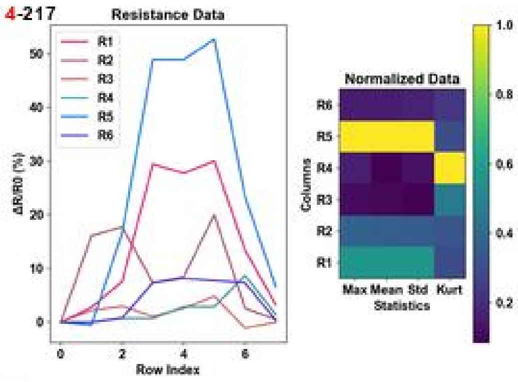

4-218
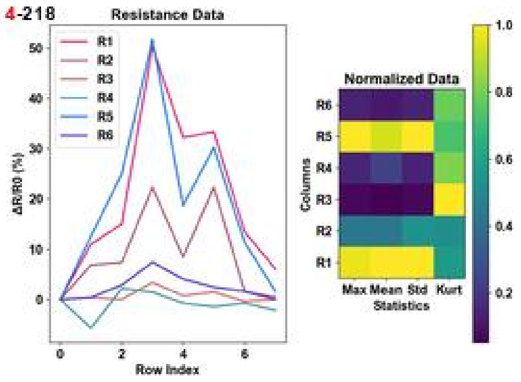

4-219
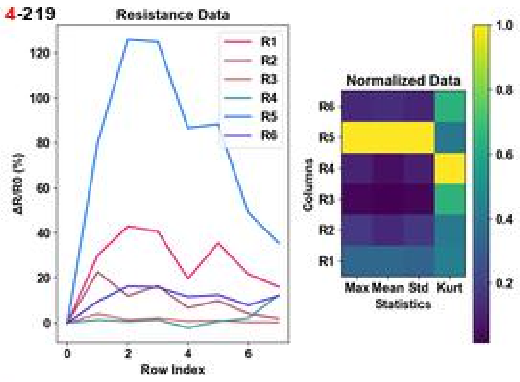

4-220
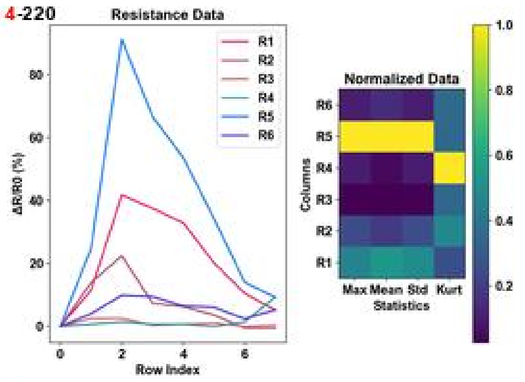

4-221
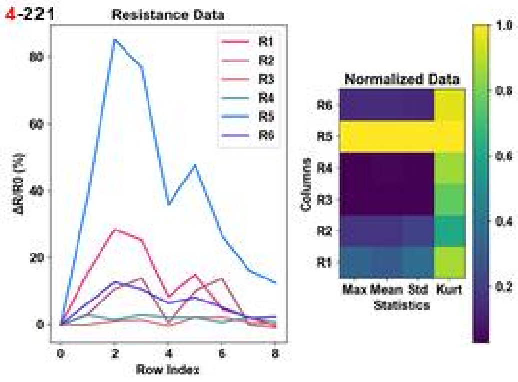

4-222
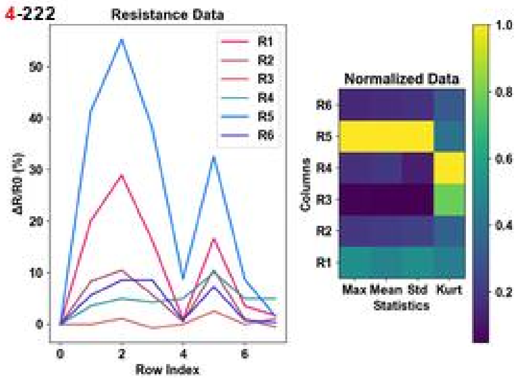

4-223
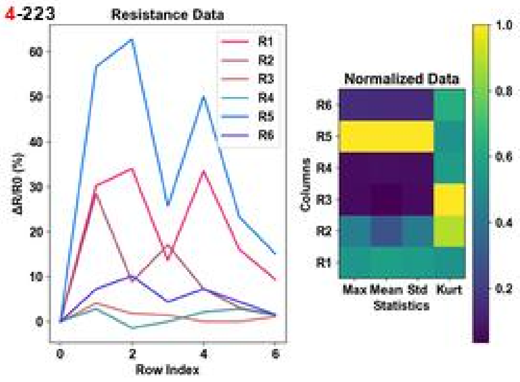

4-224
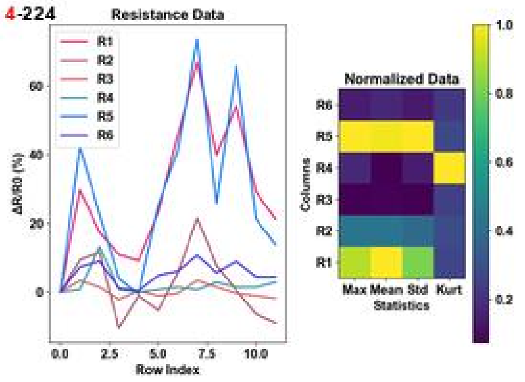

4-225

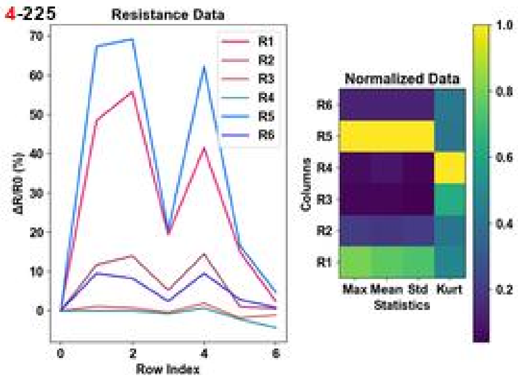

4-226

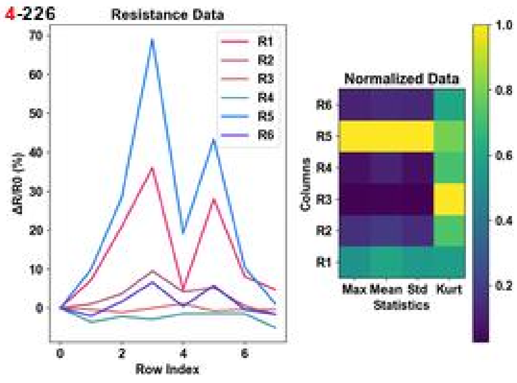

4-227

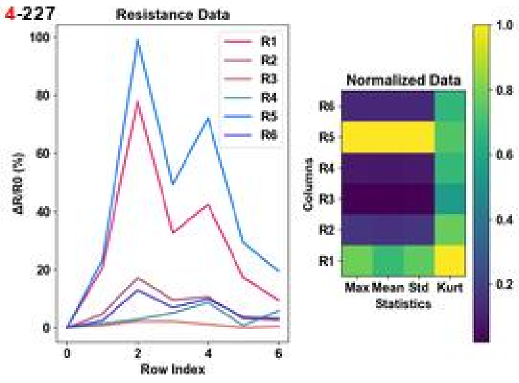

4-228

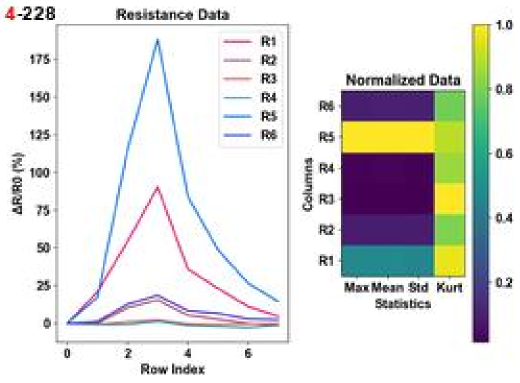

4-229

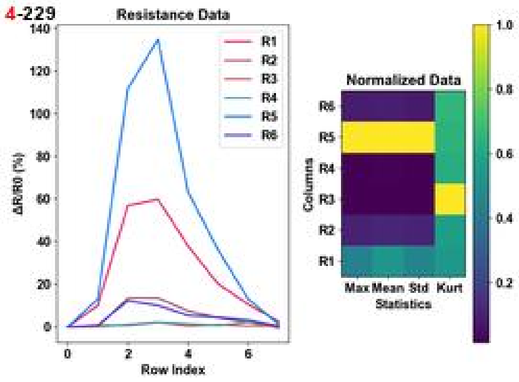

4-230

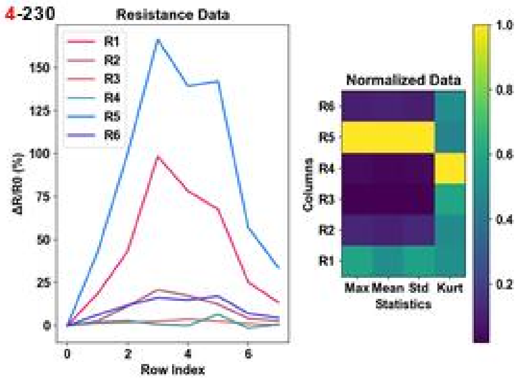

4-231

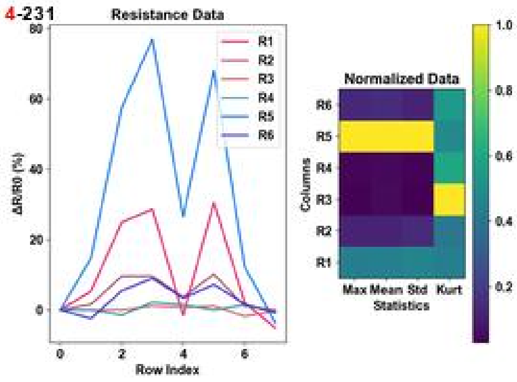

4-232

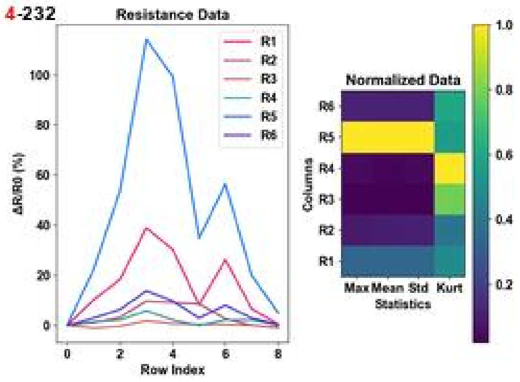

4-233

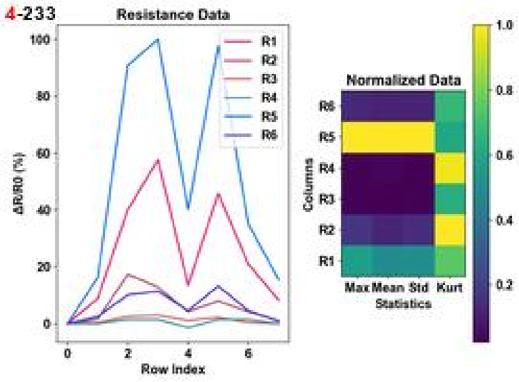

4-234

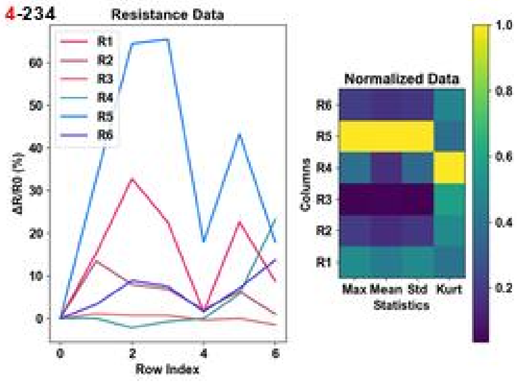

4-235

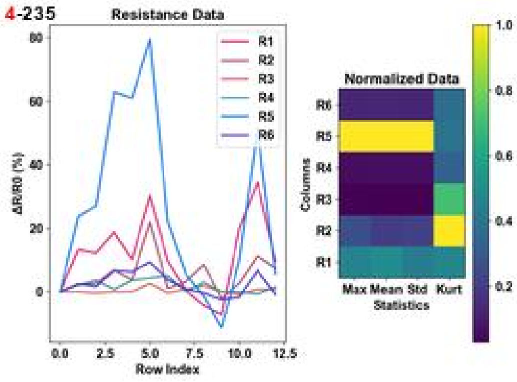

4-236

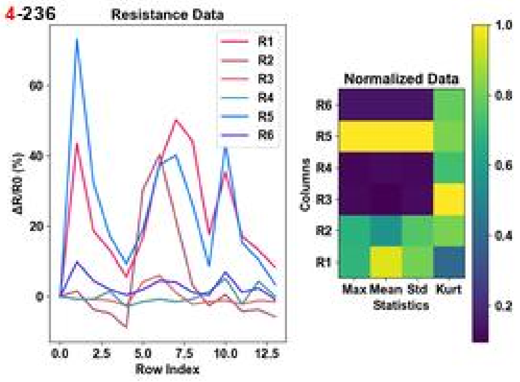

4-237

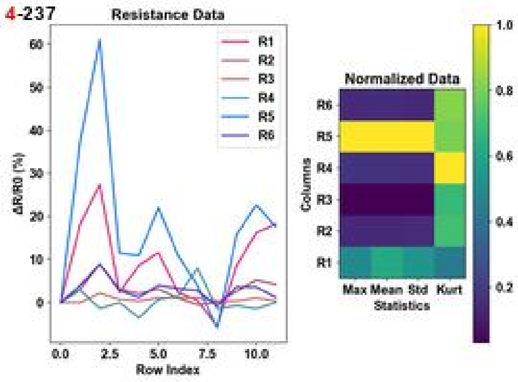

4-238

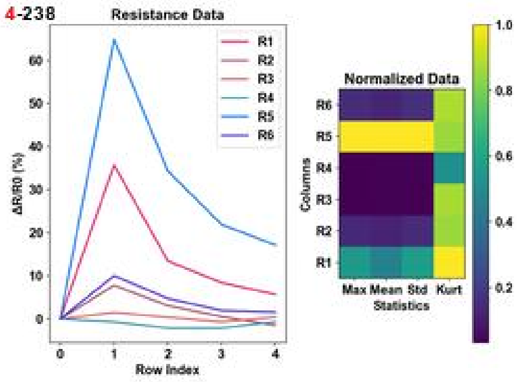

4-239

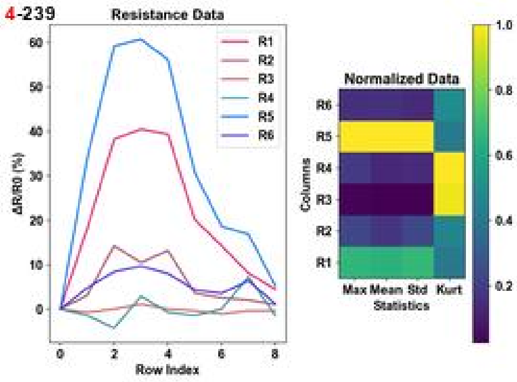

4-240

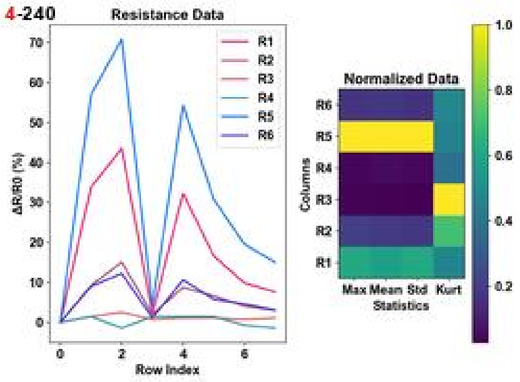

Pressure-sensitive data of type 5

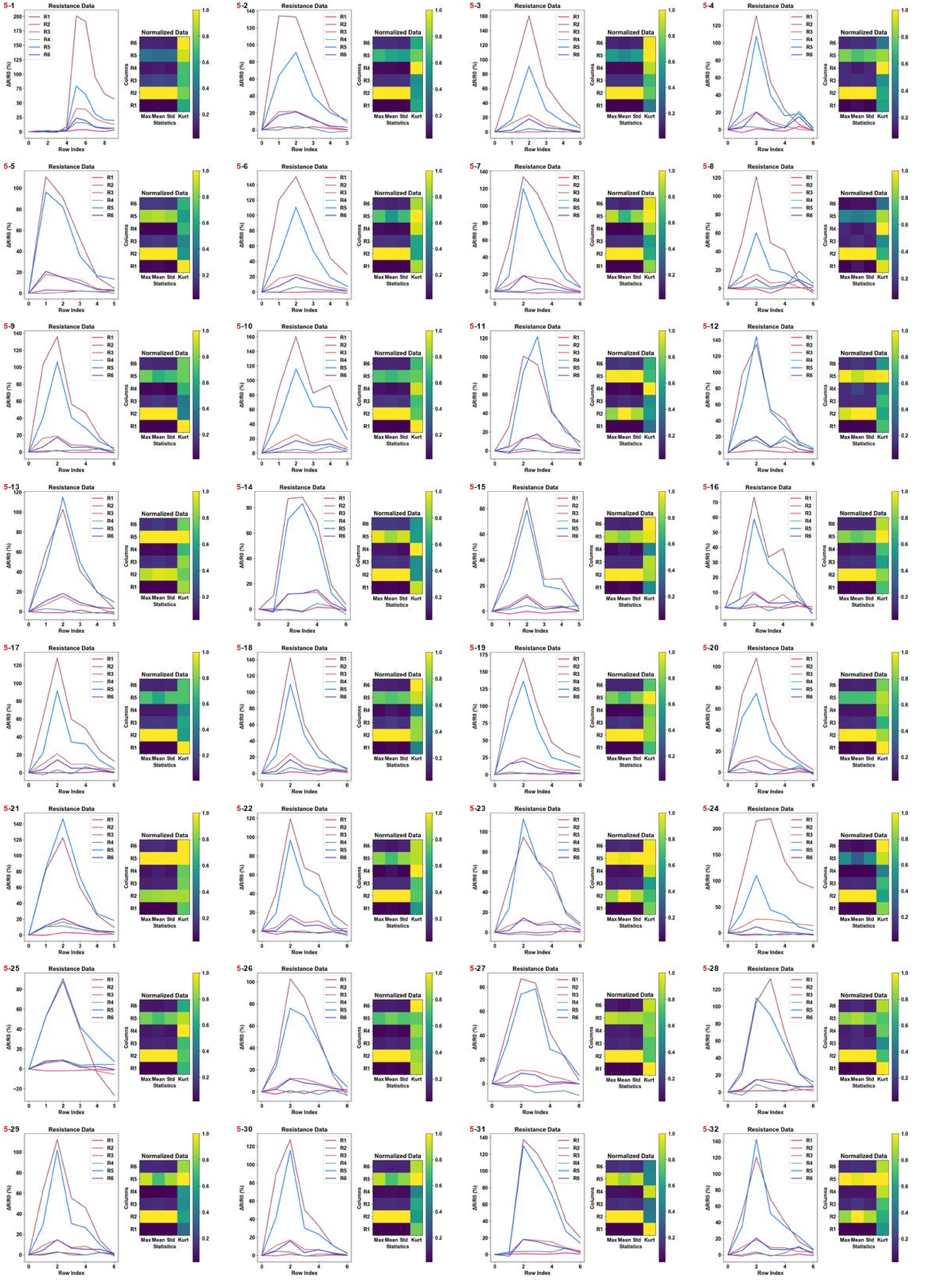

Pressure-sensitive data of type 5

5-33
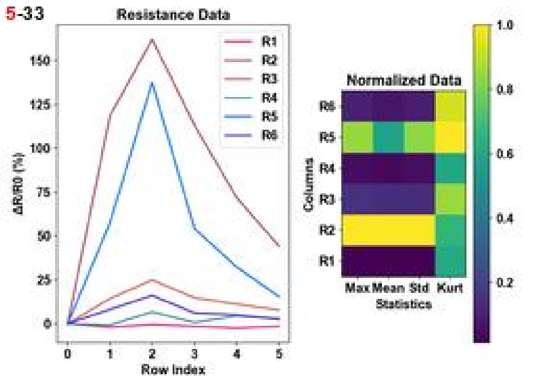

5-34
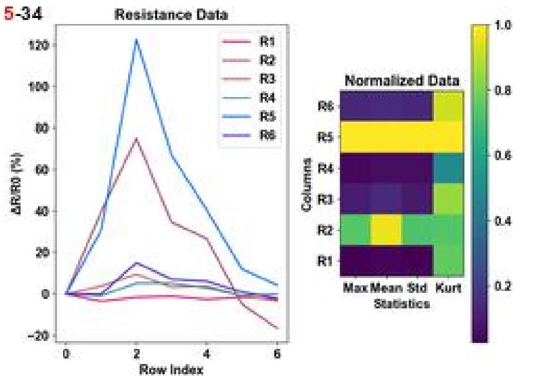

5-35
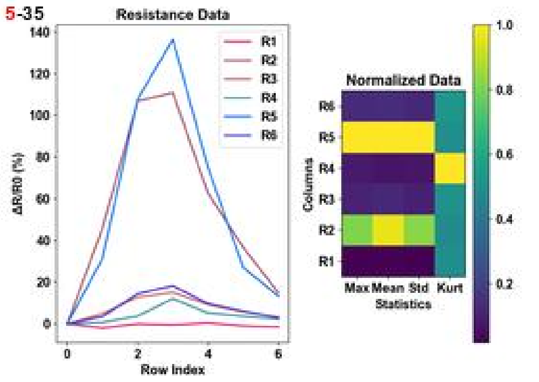

5-36
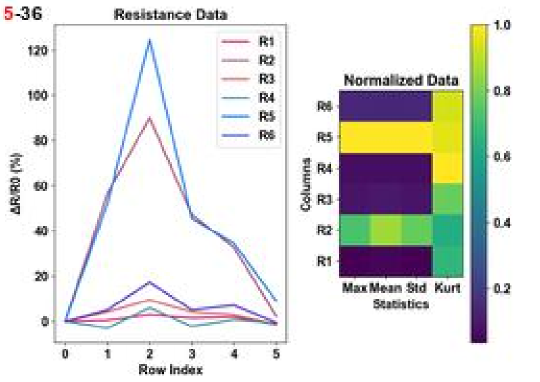

5-37
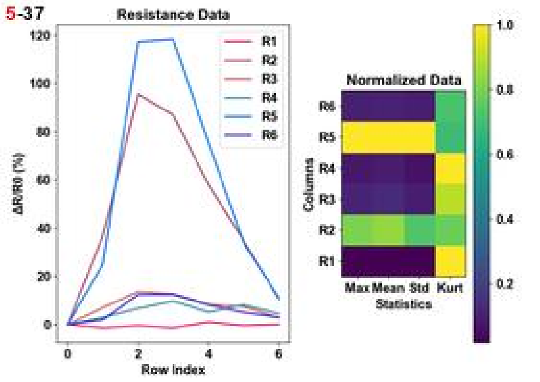

5-38
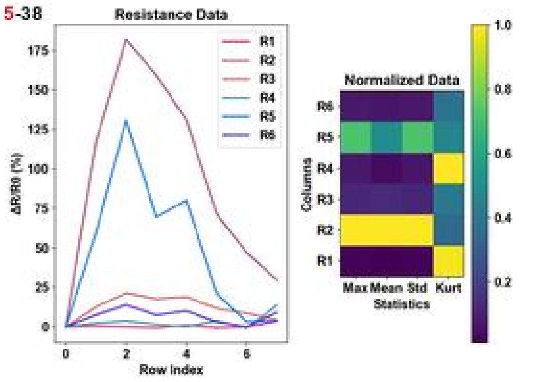

5-39
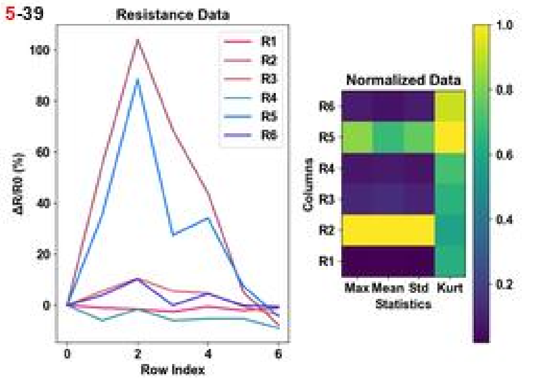

5-40
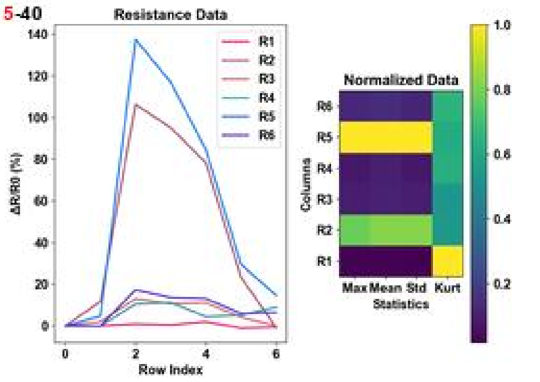

5-41
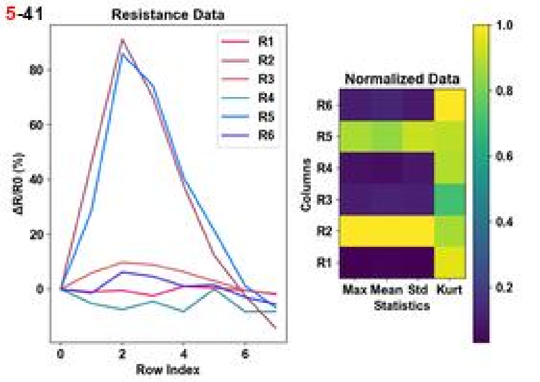

5-42
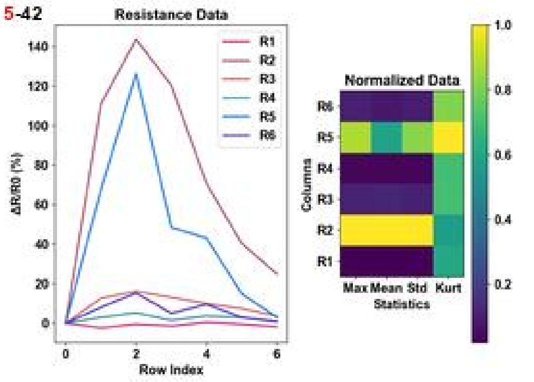

5-43
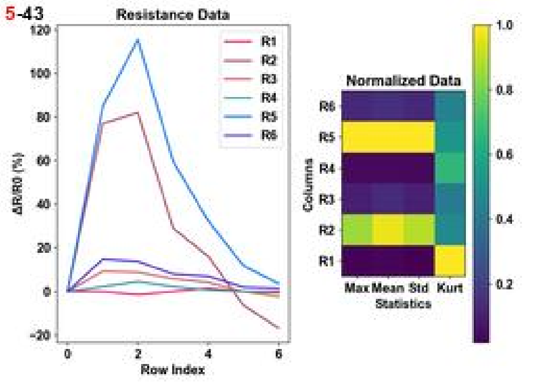

5-44
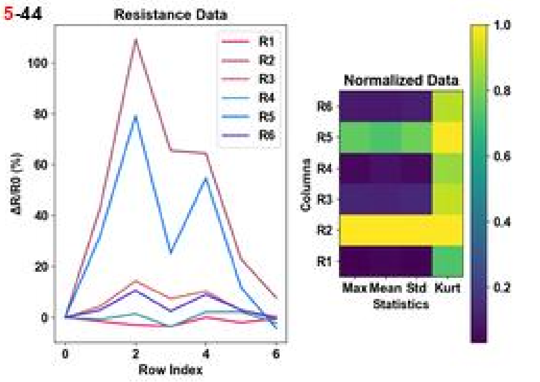

5-45
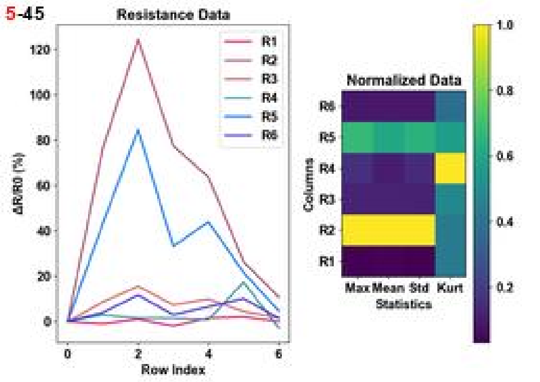

5-46
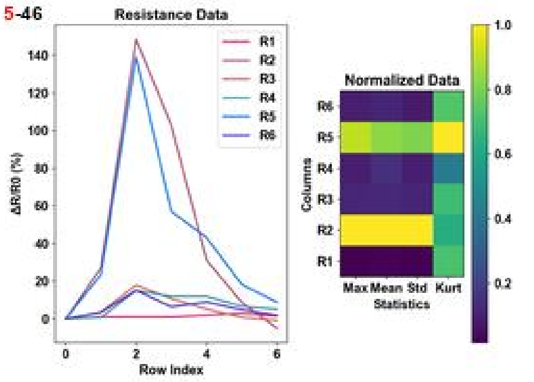

5-47
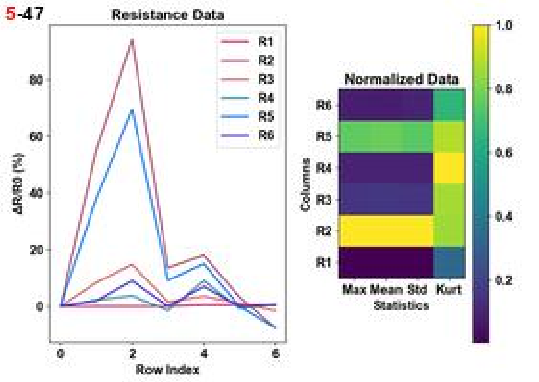

5-48
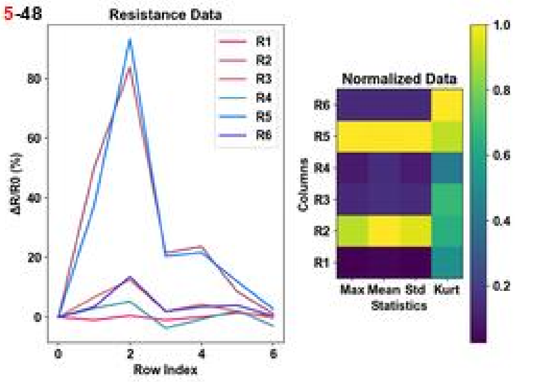

5-49
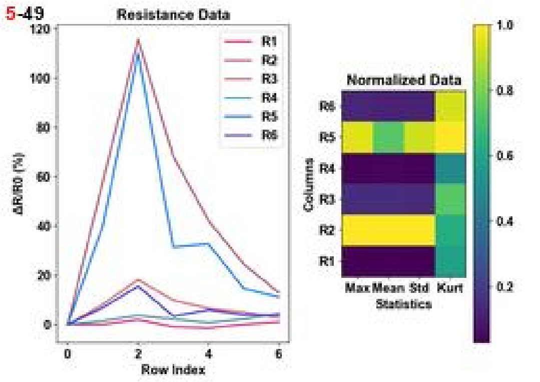

5-50
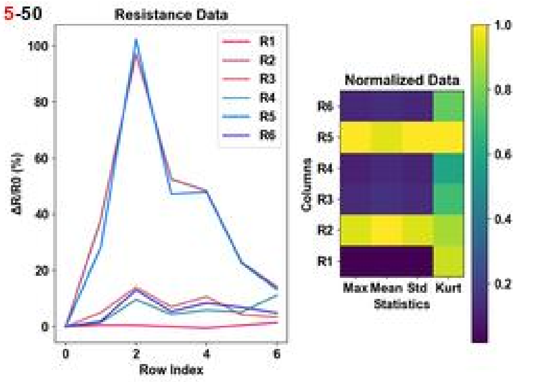

5-51
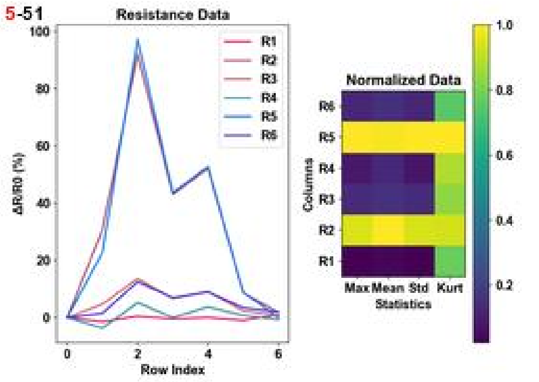

5-52
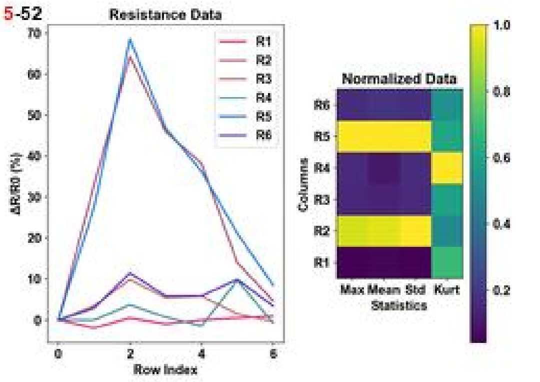

5-53
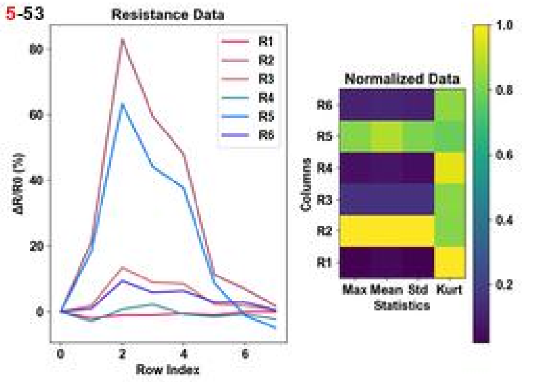

5-54
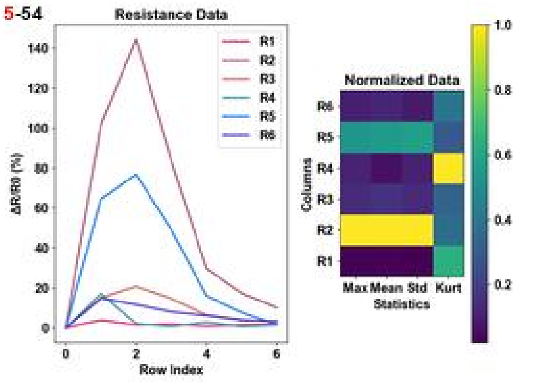

5-55
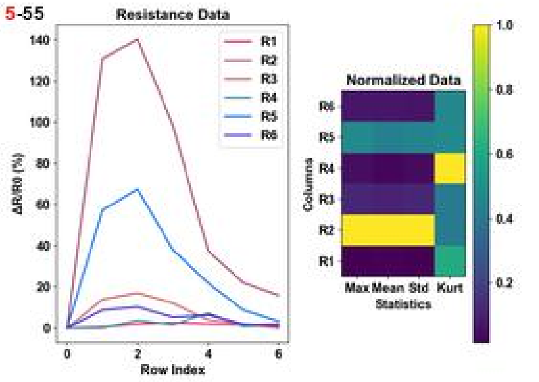

5-56
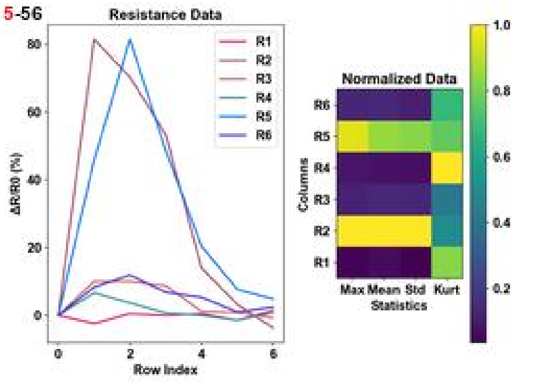

5-57
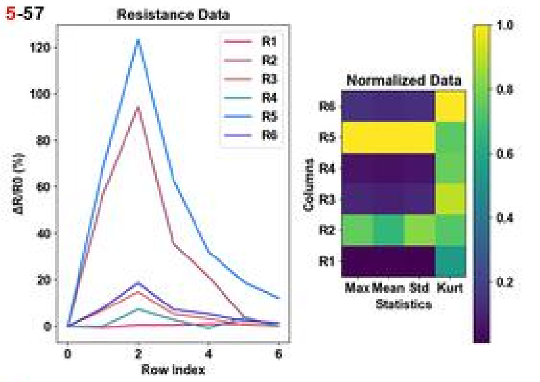

5-58
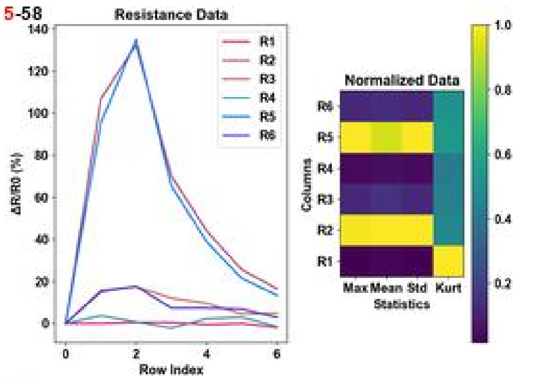

5-59
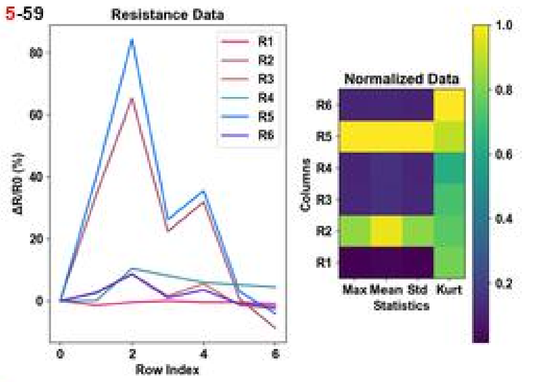

5-60
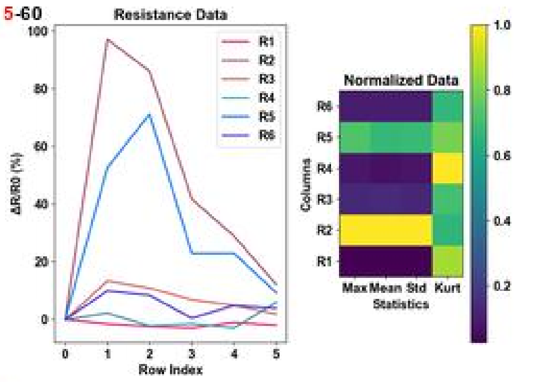

5-61
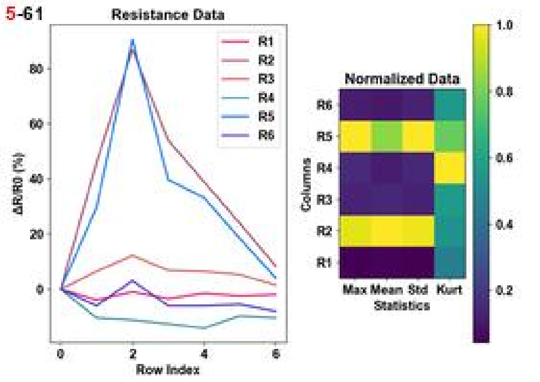

5-62
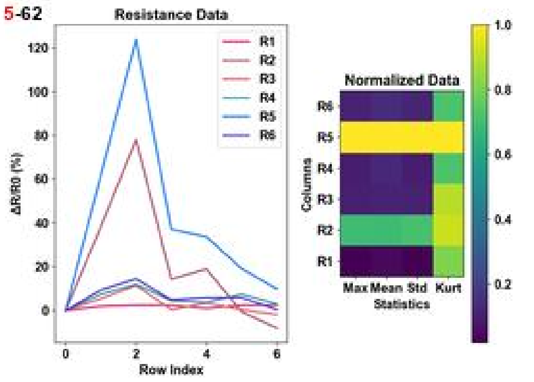

5-63
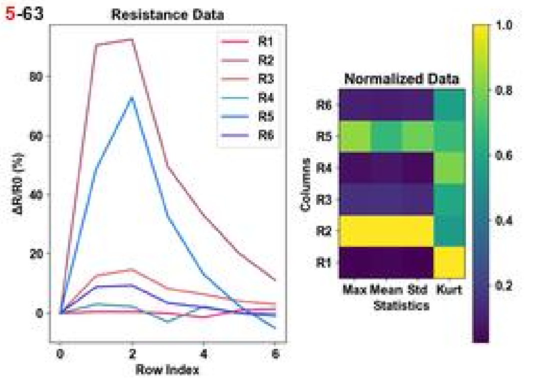

5-64
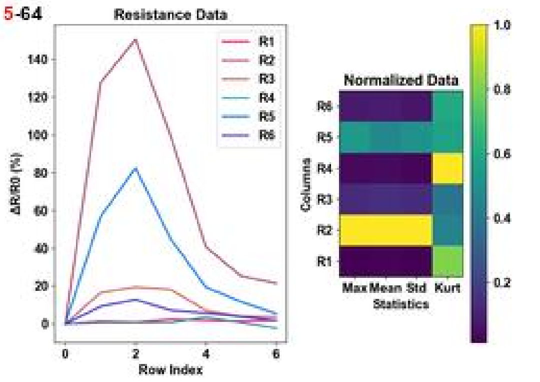

Pressure-sensitive data of type 5

5-65
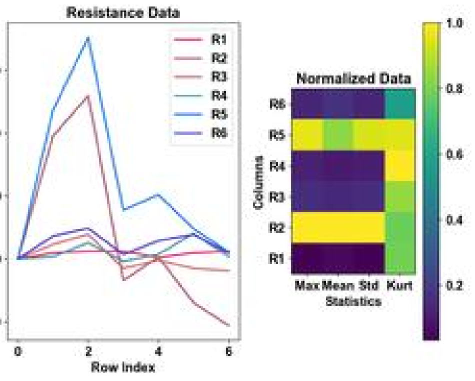

5-66
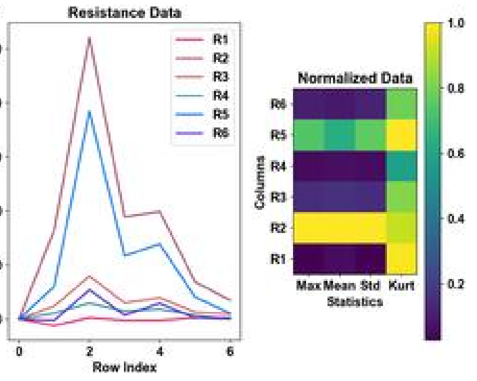

5-67
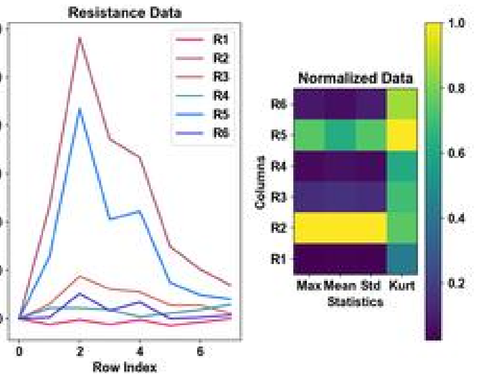

5-68
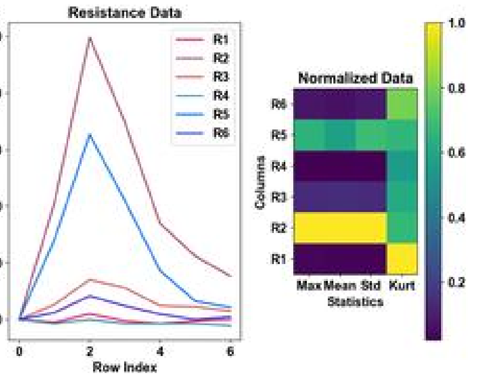

5-69
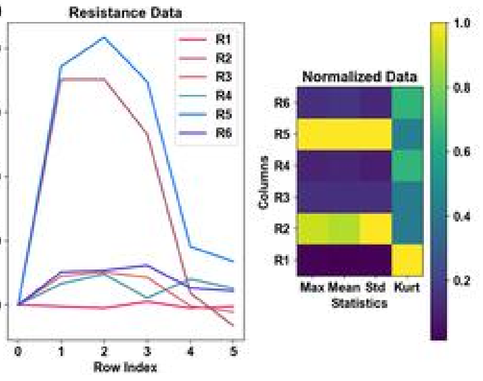

5-70
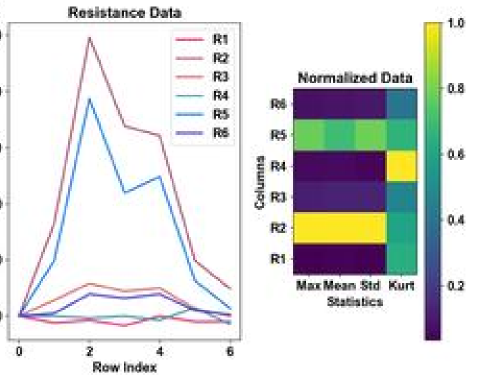

5-71
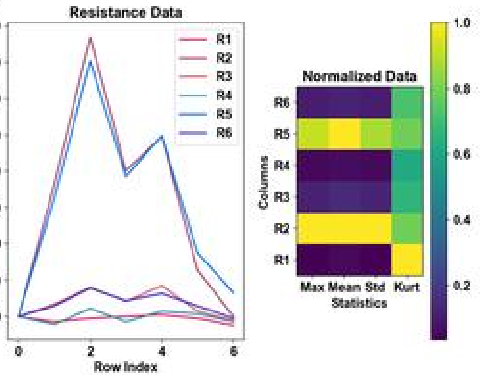

5-72
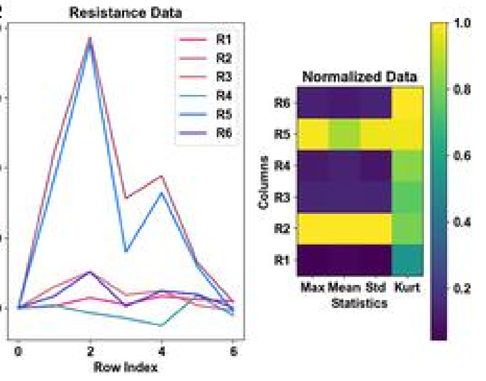

5-73
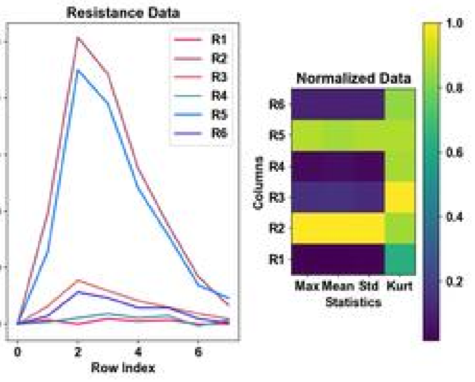

5-74
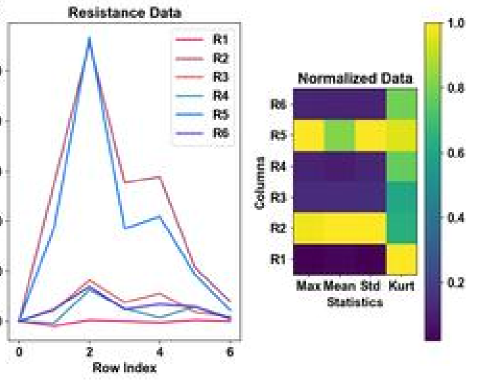

5-75
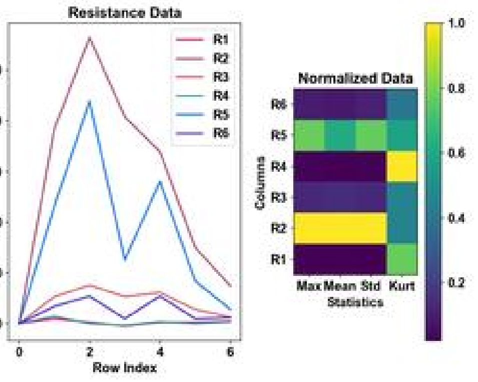

5-76
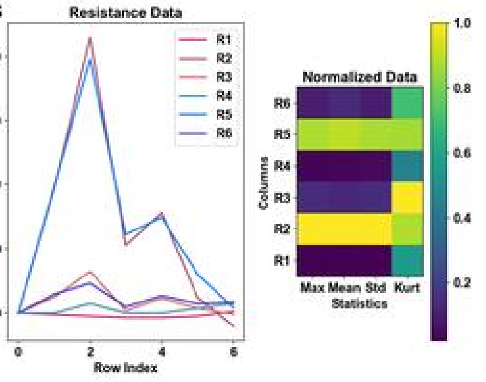

5-77
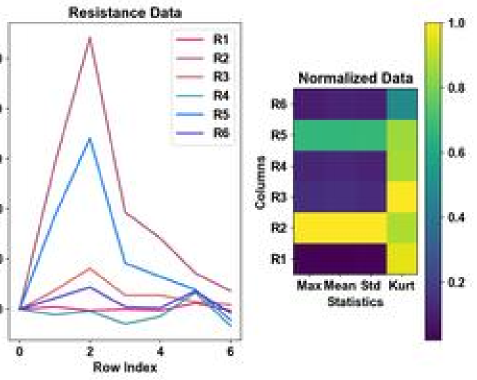

5-78
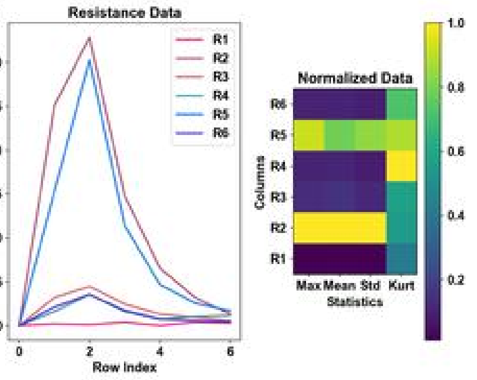

5-79
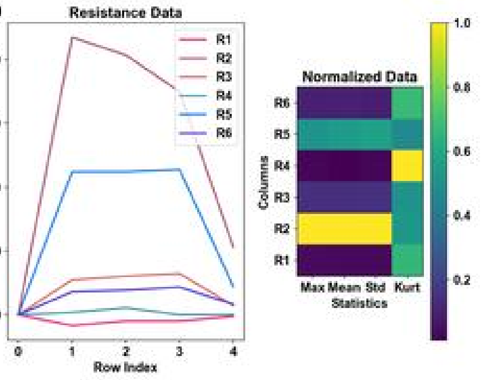

5-80
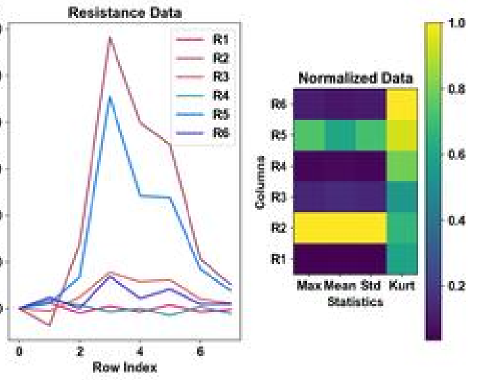

5-81
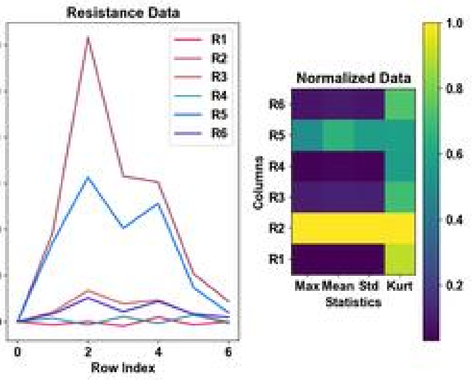

5-82
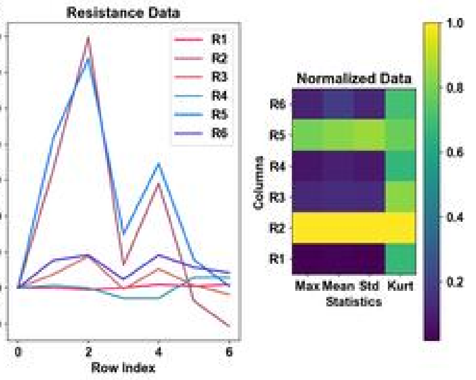

5-83
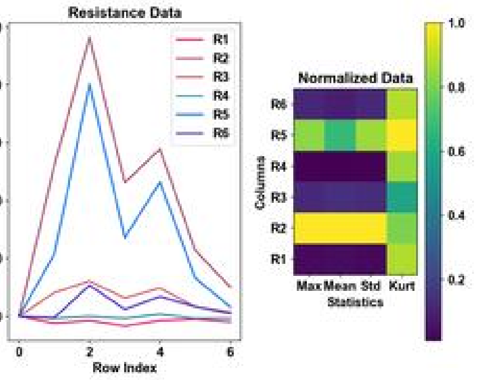

5-84
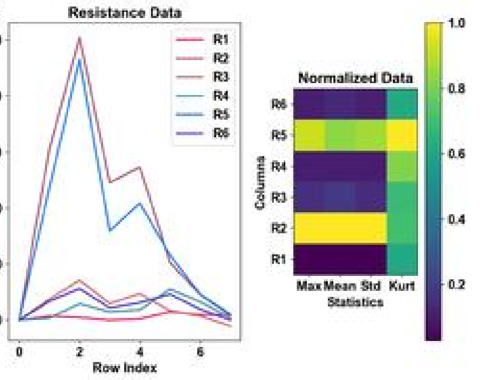

5-85
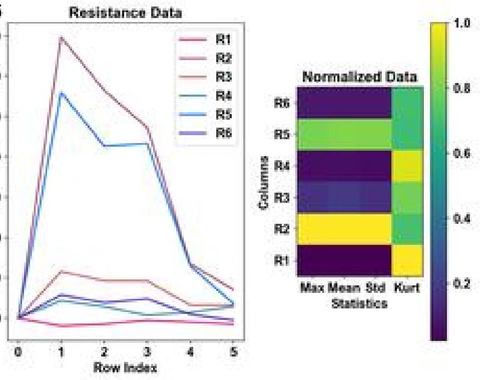

5-86
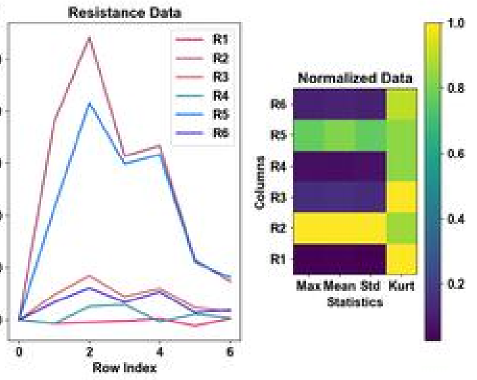

5-87
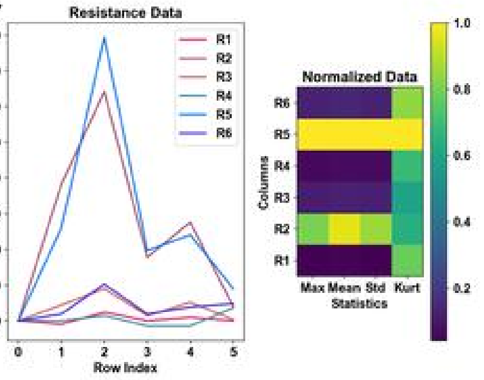

5-88
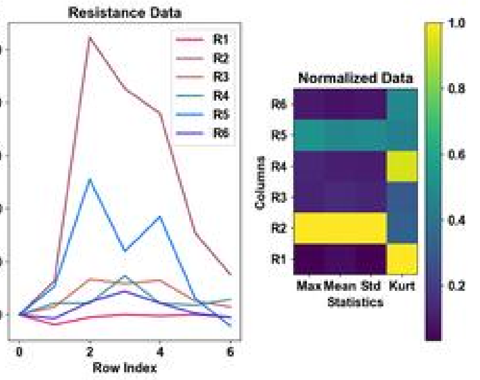

5-89
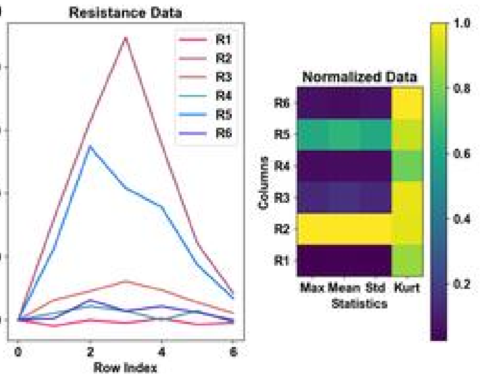

5-90
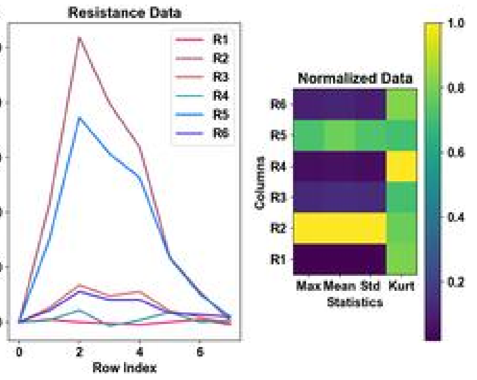

5-91
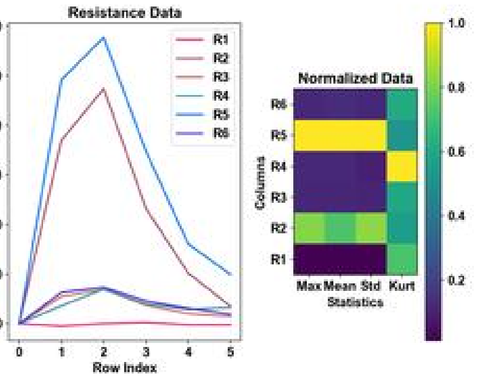

5-92
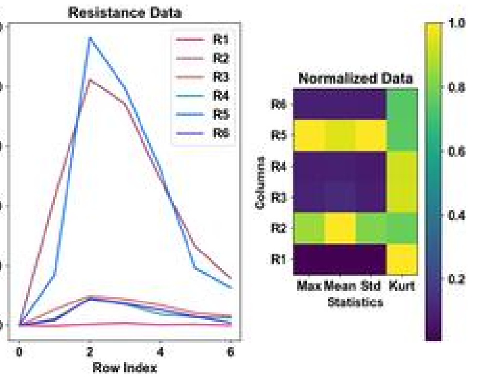

5-93
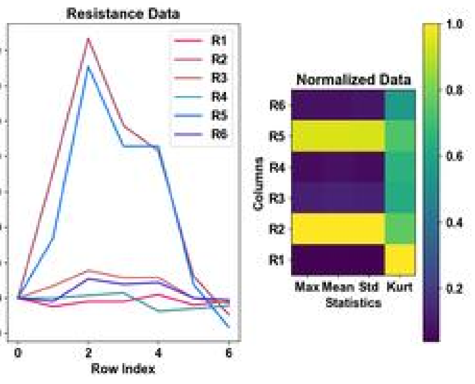

5-94
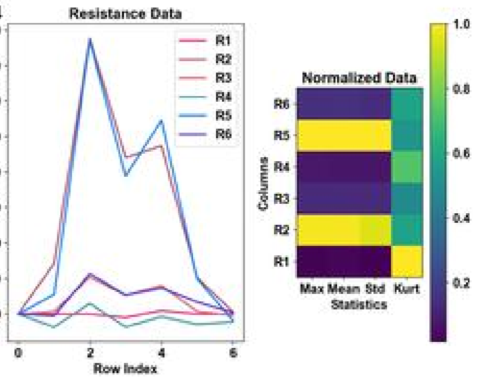

5-95
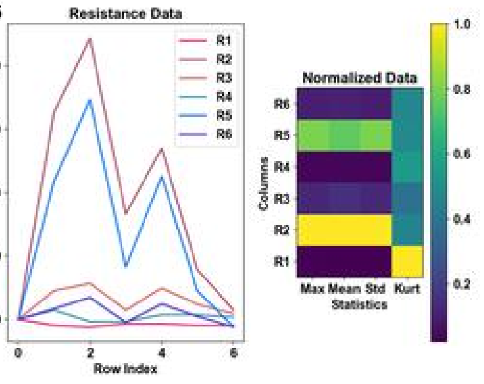

5-96
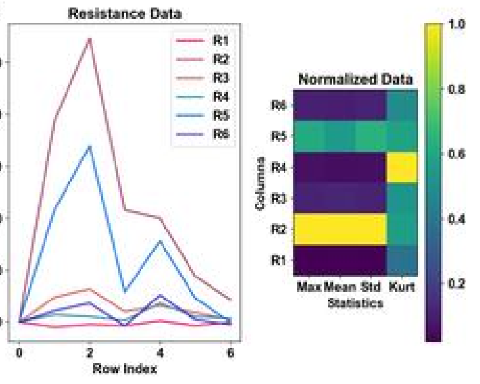

Pressure-sensitive data of type 5

5-97
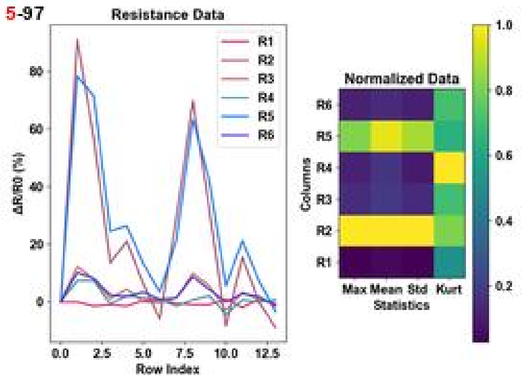
5-98
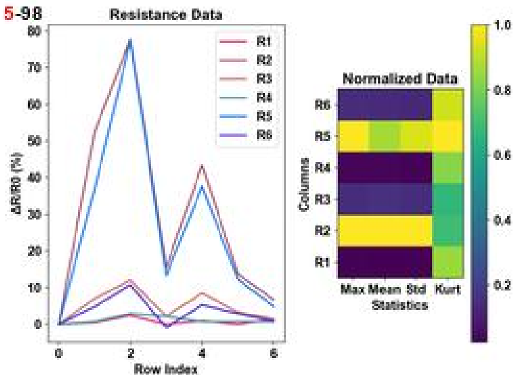
5-99
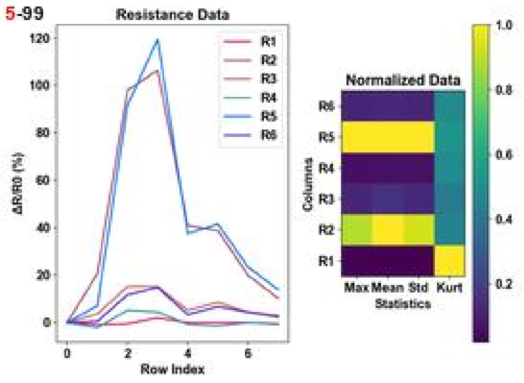
5-100
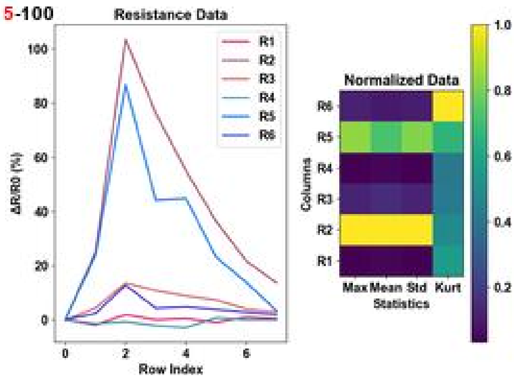
5-101
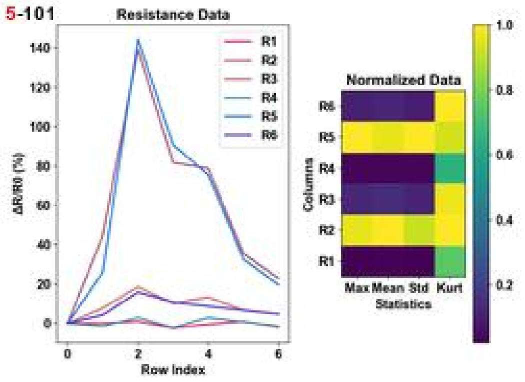
5-102
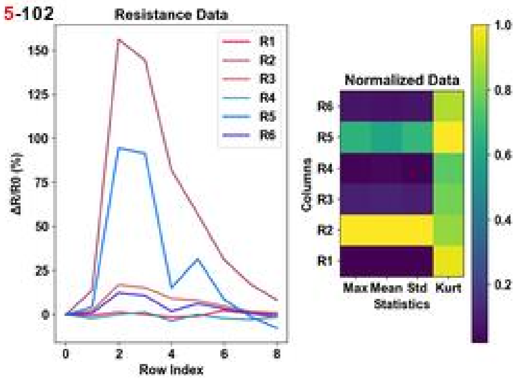
5-103
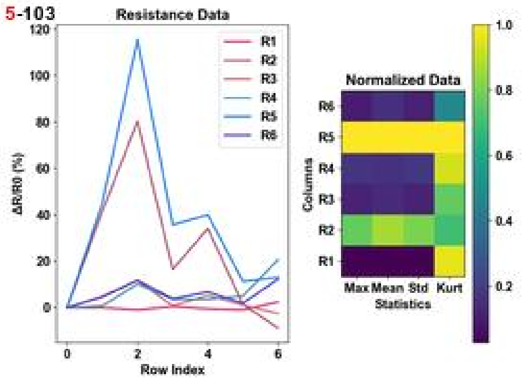
5-104
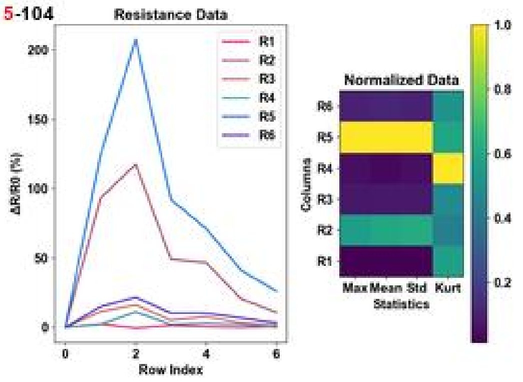
5-105
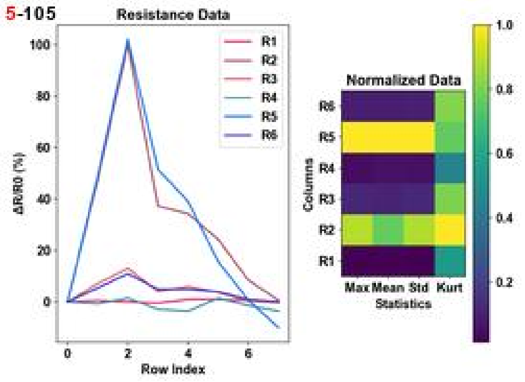
5-106
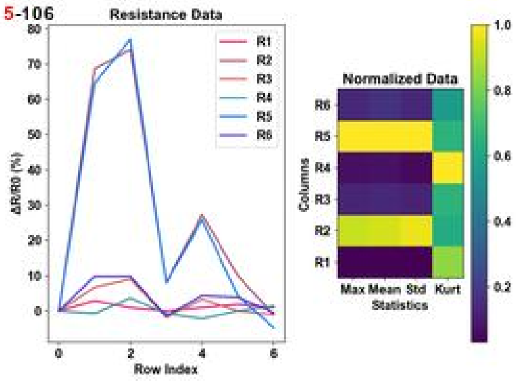
5-107
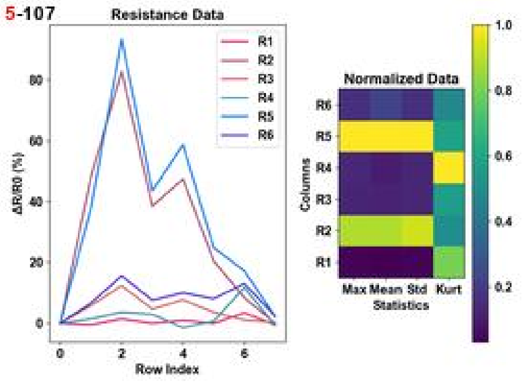
5-108
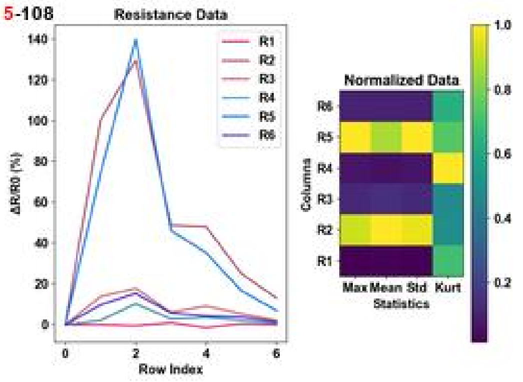
5-109
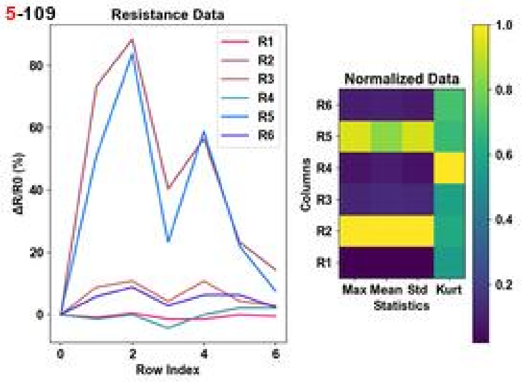
5-110
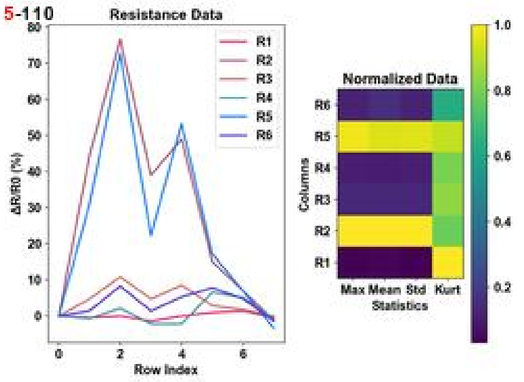
5-111
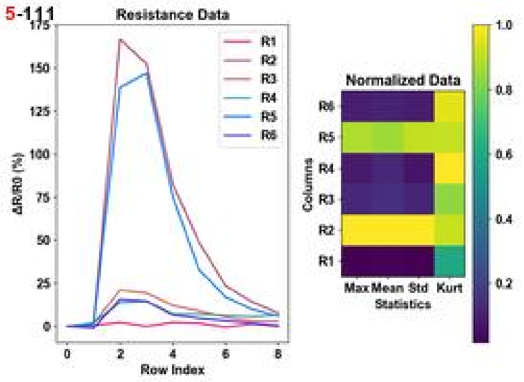
5-112
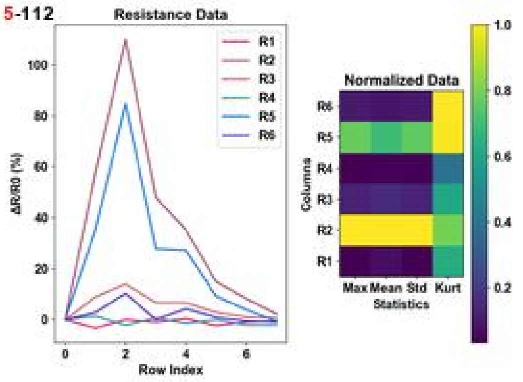
5-113
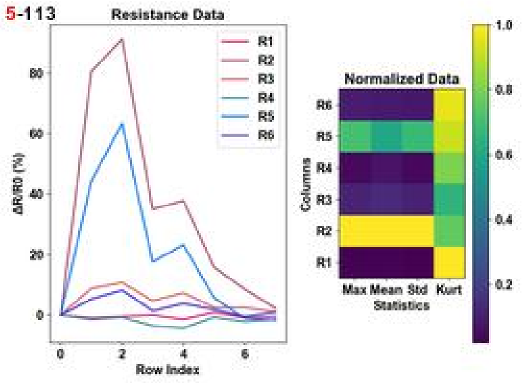
5-114
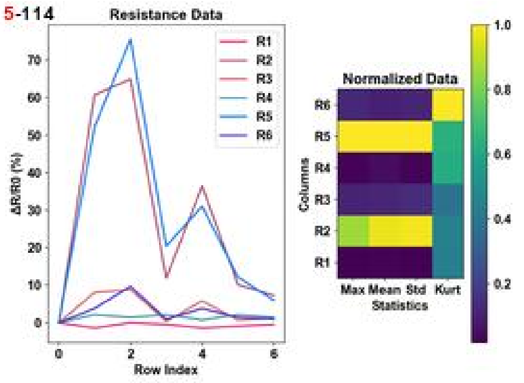
5-115
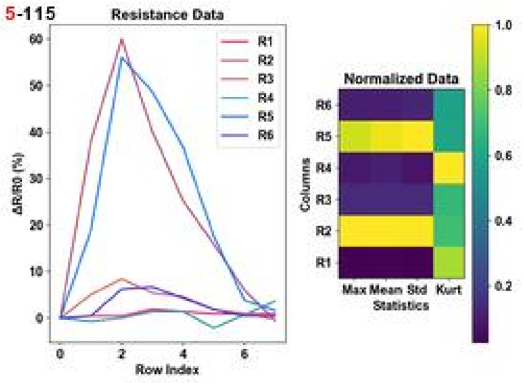
5-116
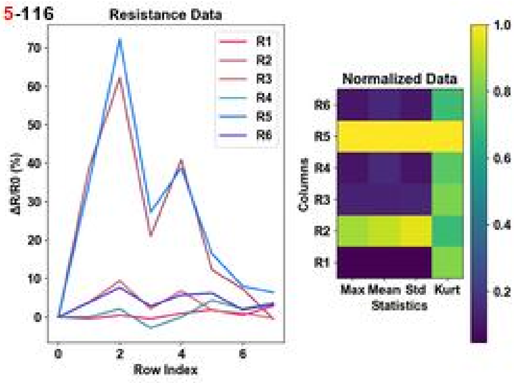
5-117
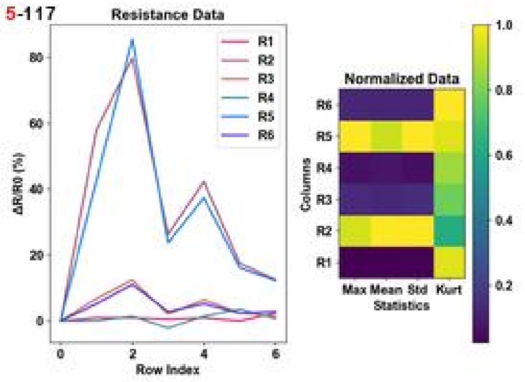
5-118
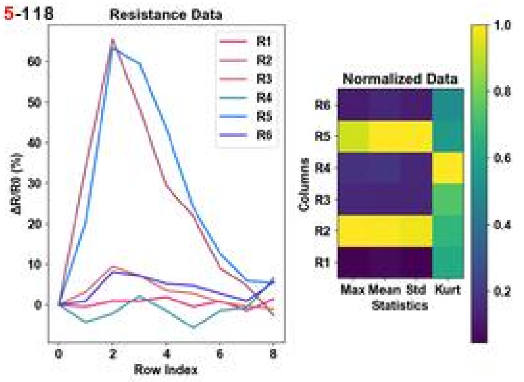
5-119
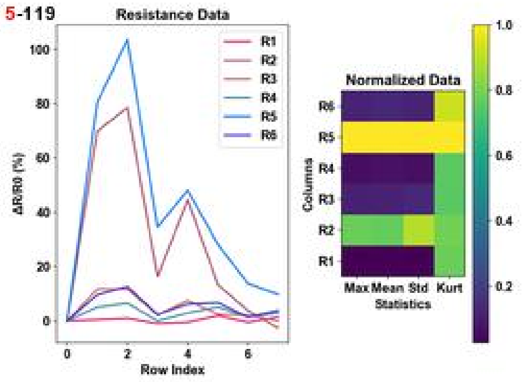
5-120
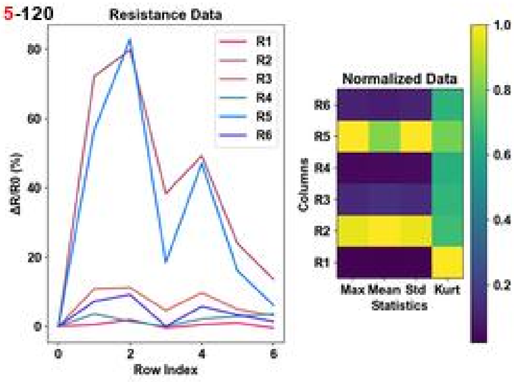
5-121
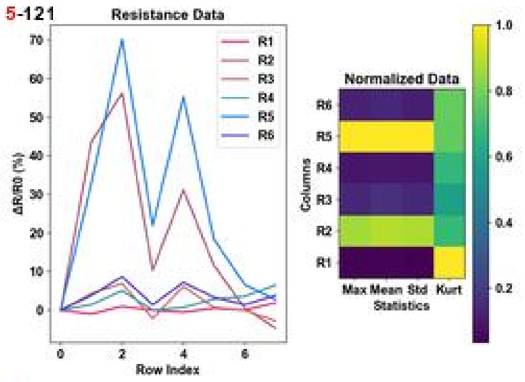
5-122
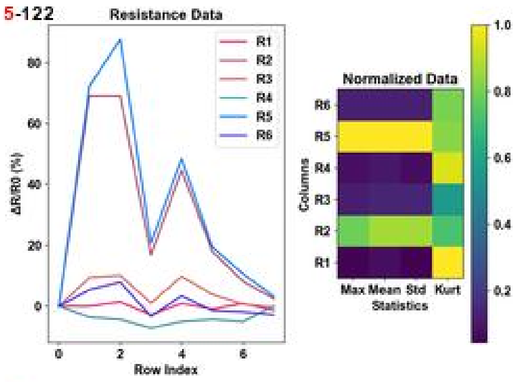
5-123
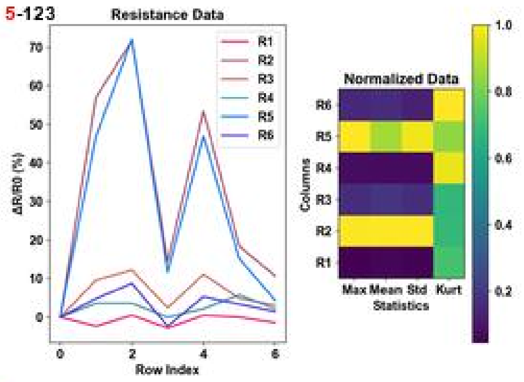
5-124
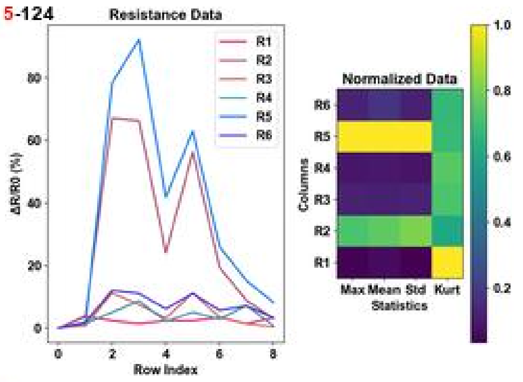
5-125
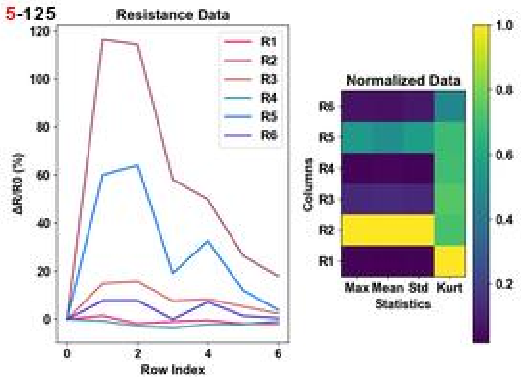
5-126
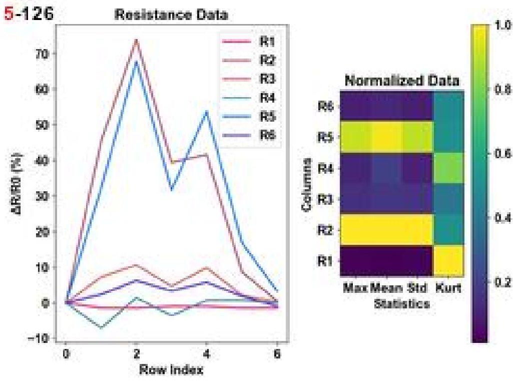
5-127
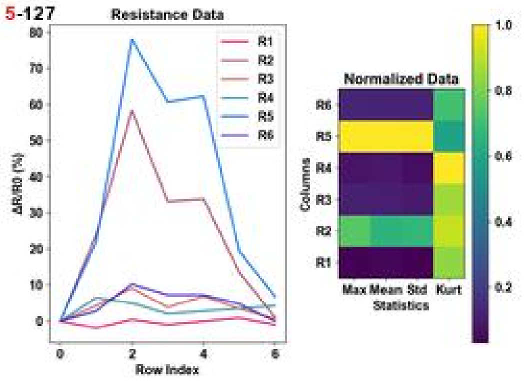
5-128
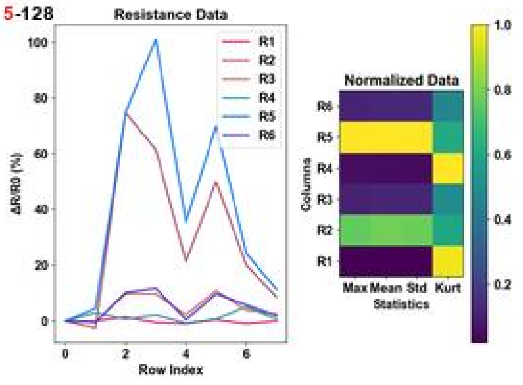

5-129
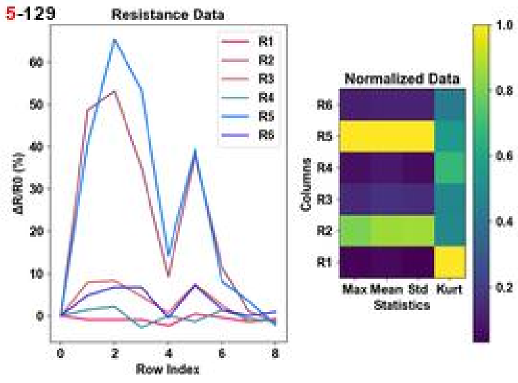

5-130
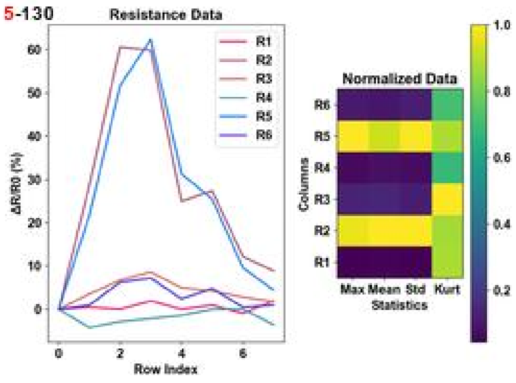

5-131
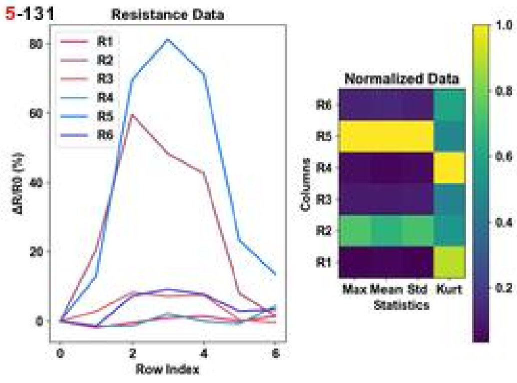

5-132
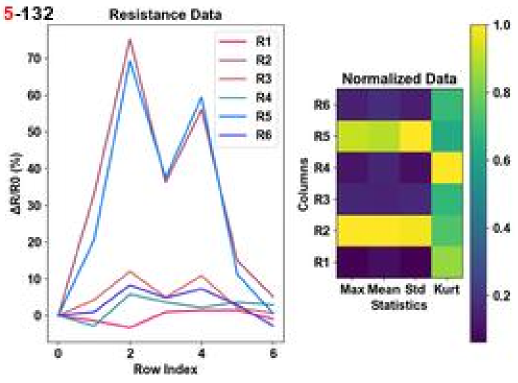

5-133
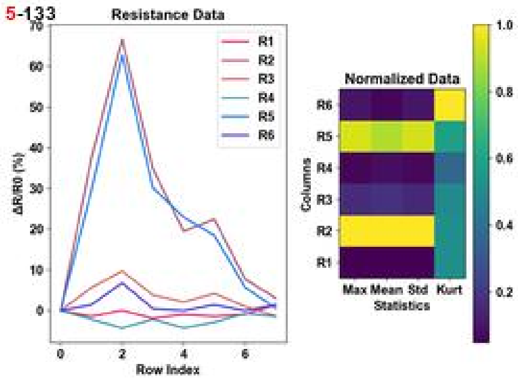

5-134
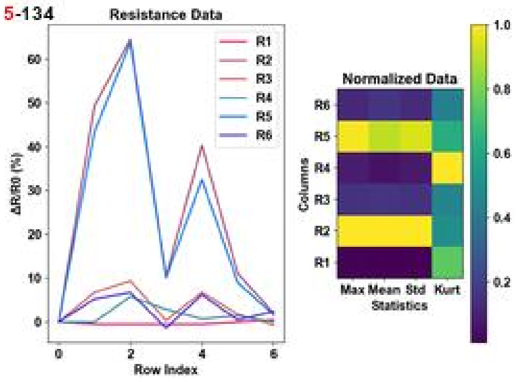

5-135
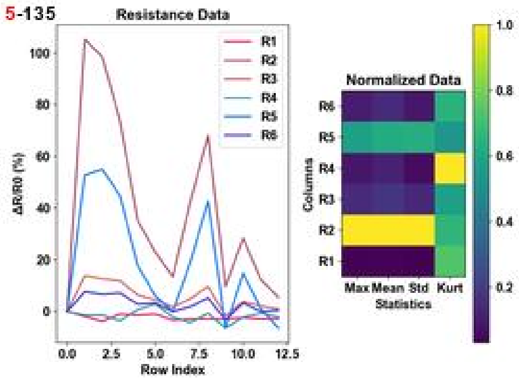

5-136
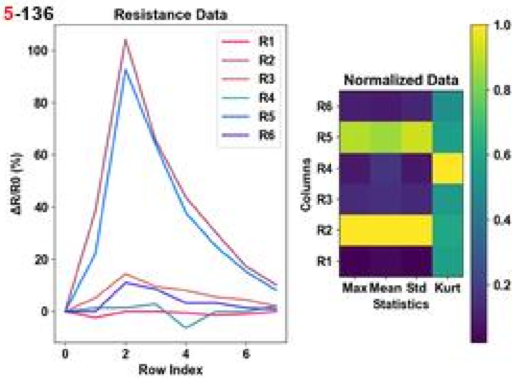

5-137
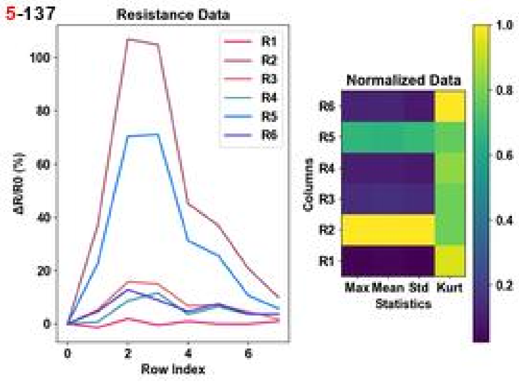

5-138
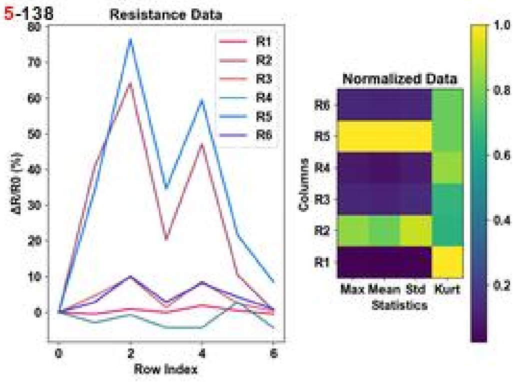

5-139
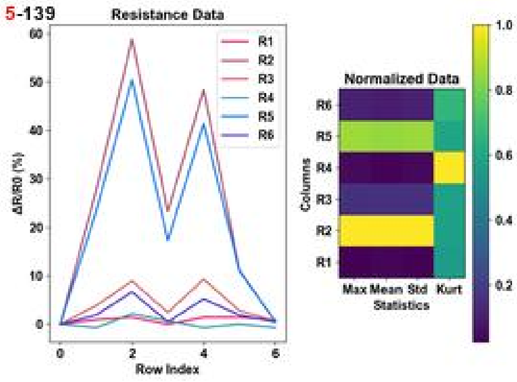

5-140
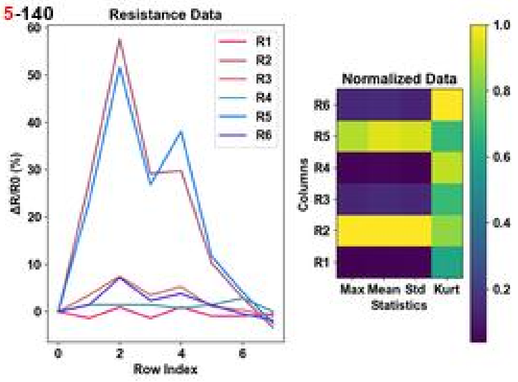

5-141
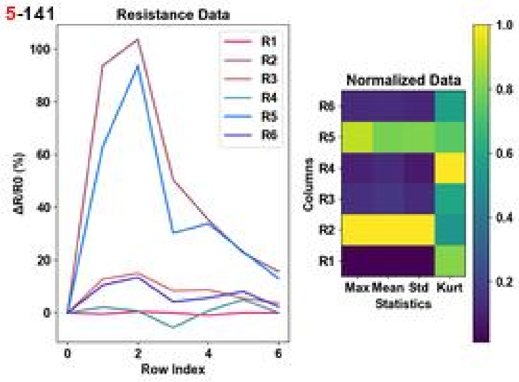

5-142
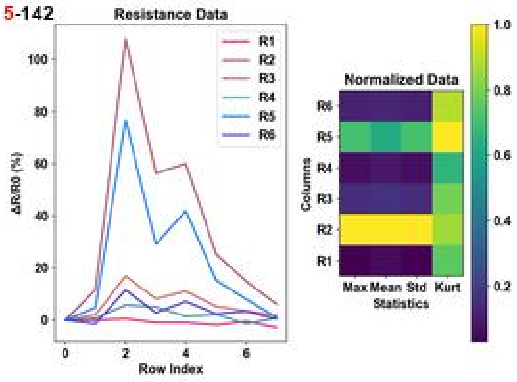

5-143
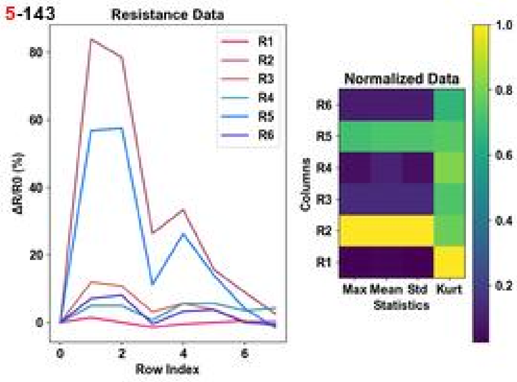

5-144
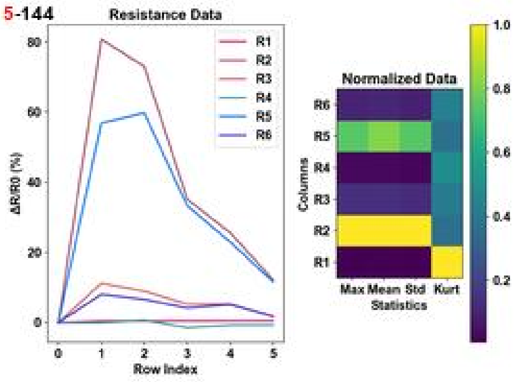

5-145
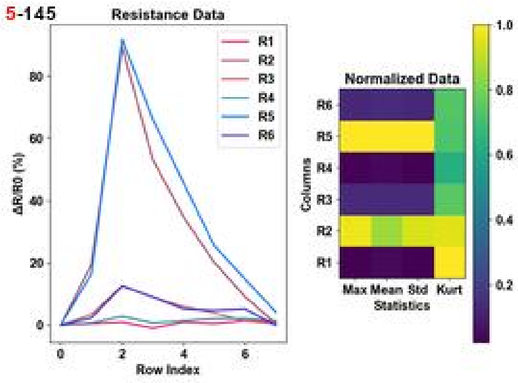

5-146
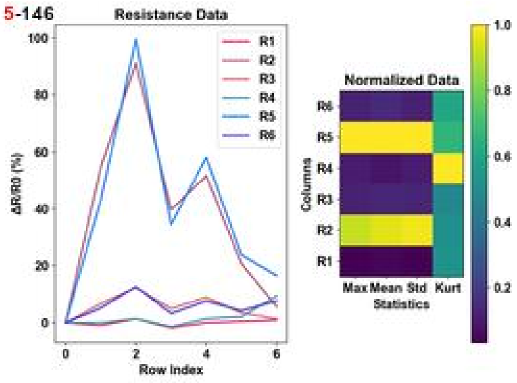

5-147
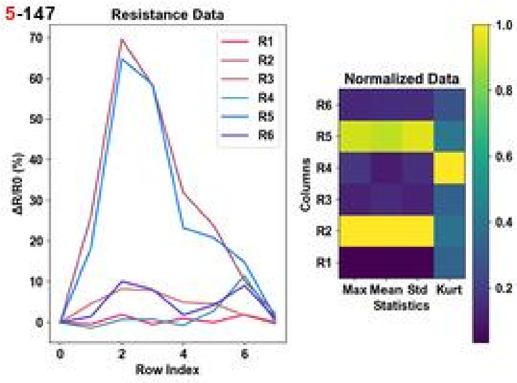

5-148
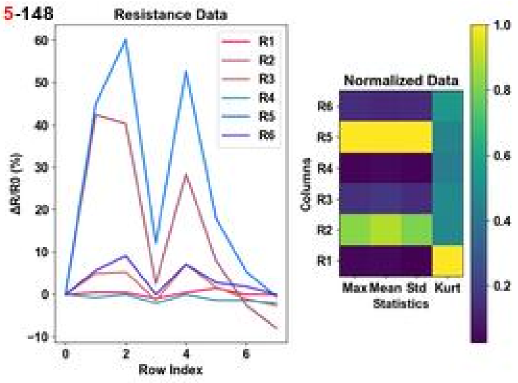

5-149
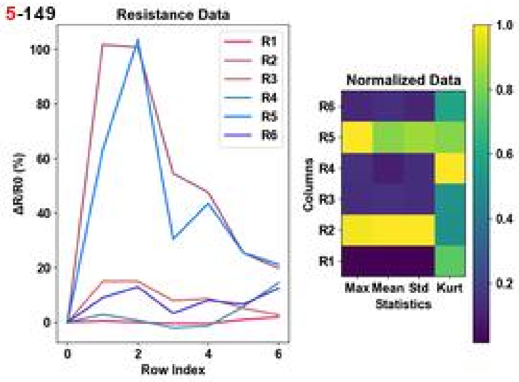

5-150
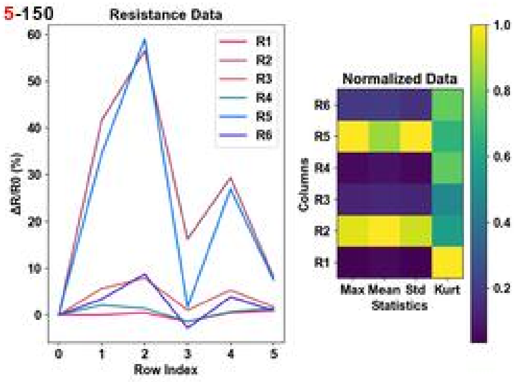

5-151
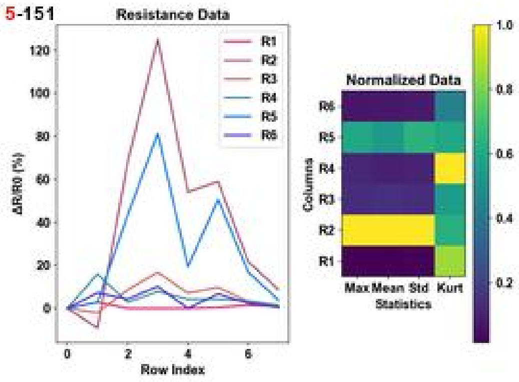

5-152
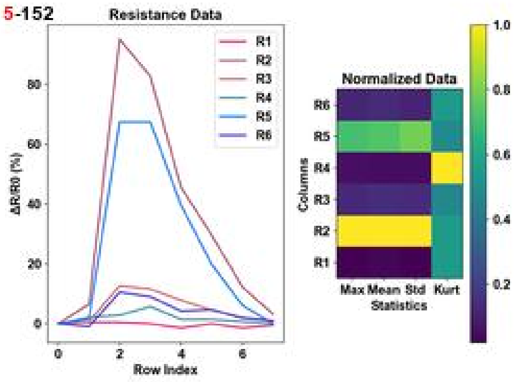

5-153
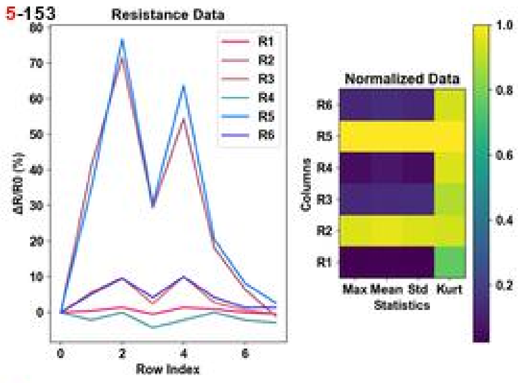

5-154
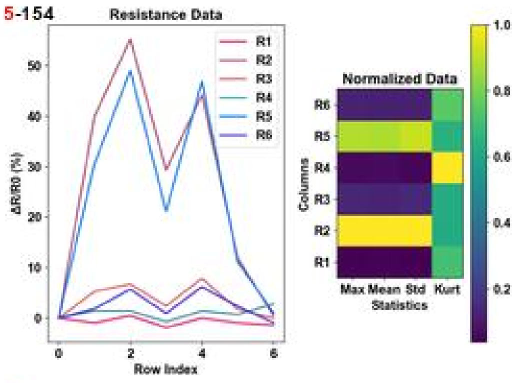

5-155
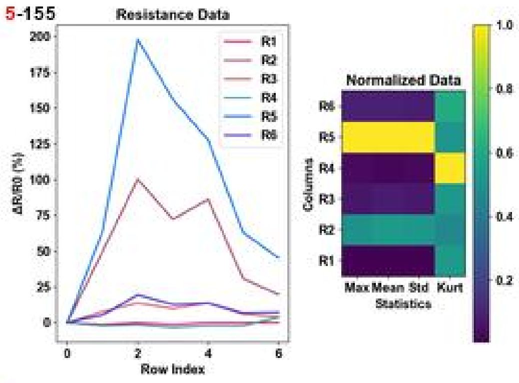

5-156
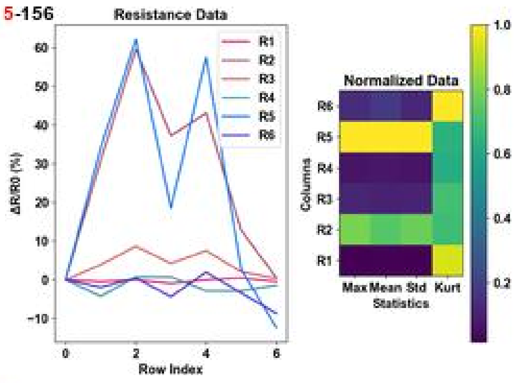

5-157
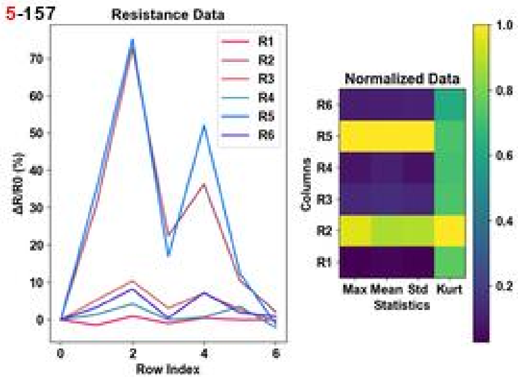

5-158
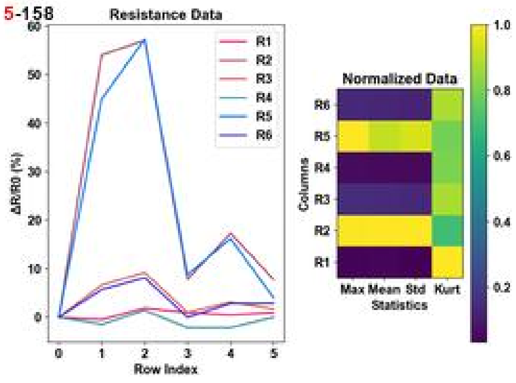

5-159
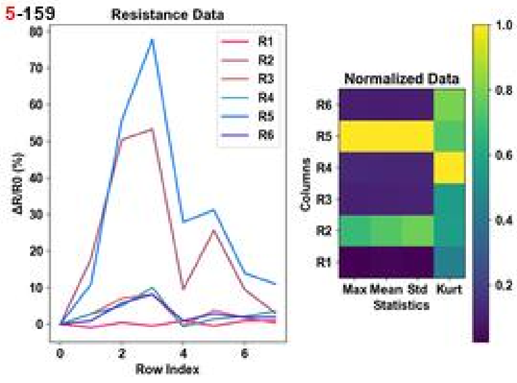

5-160
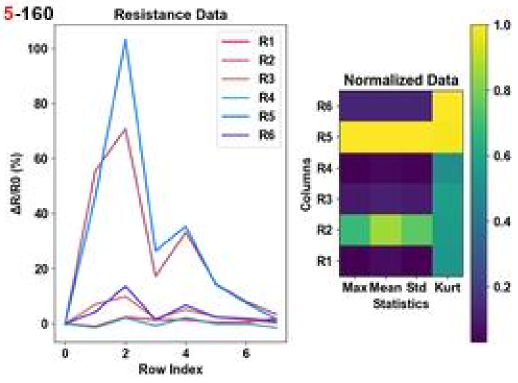

5-161
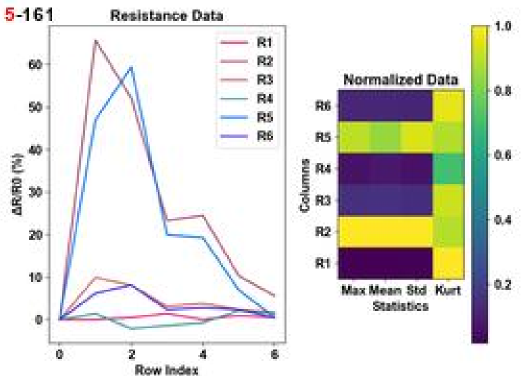

5-162
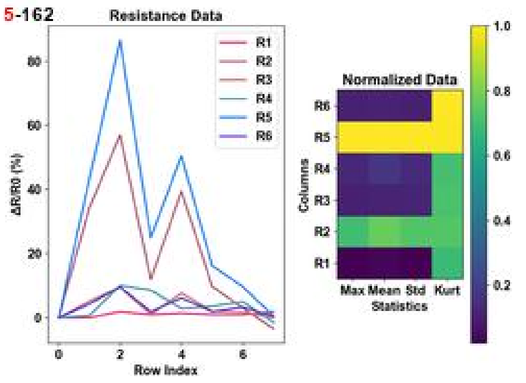

5-163
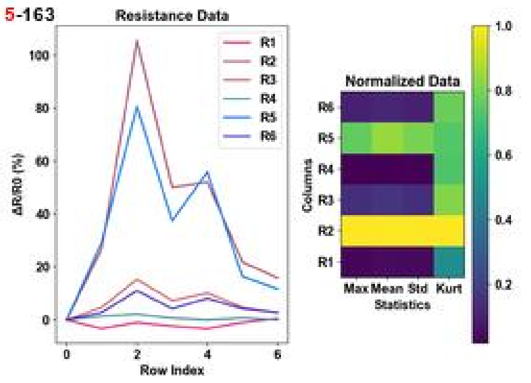

5-164
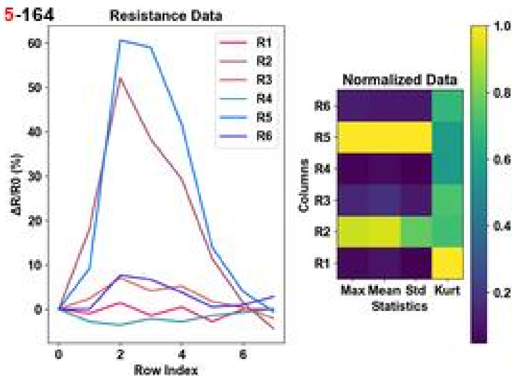

5-165
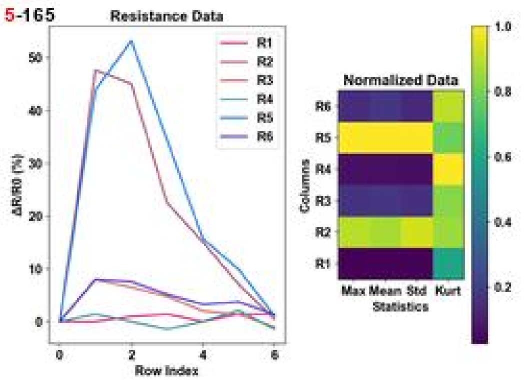

5-166
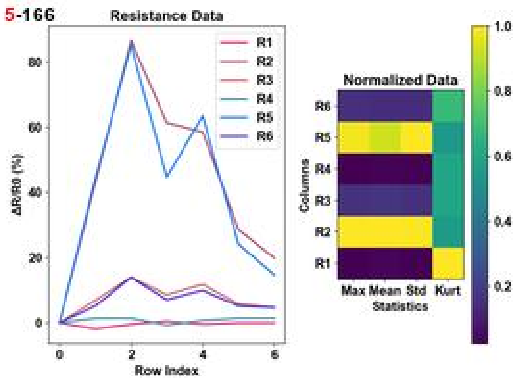

5-167
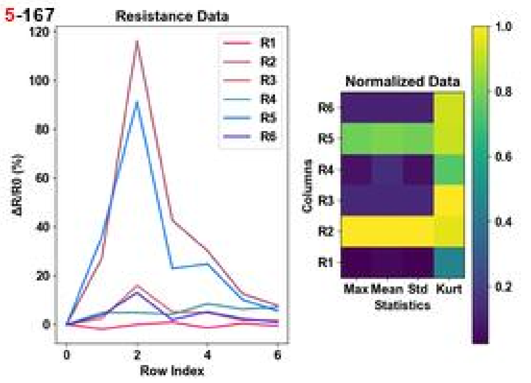

5-168
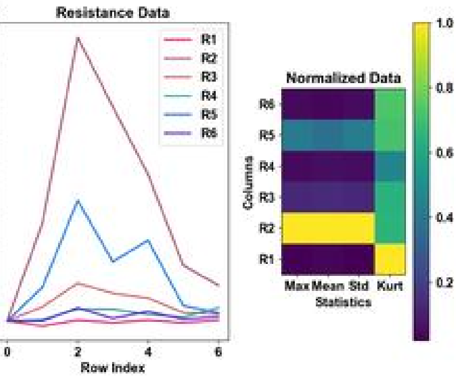

5-169
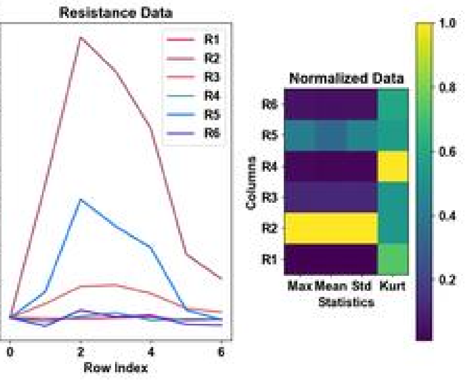

5-170
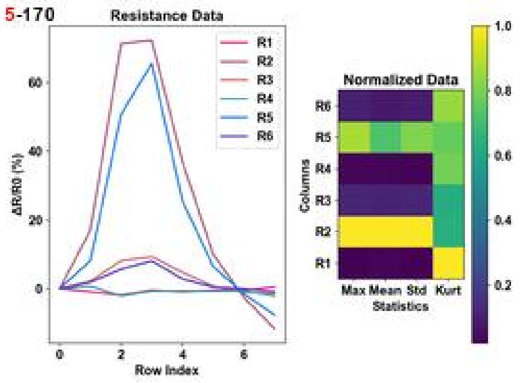

5-171
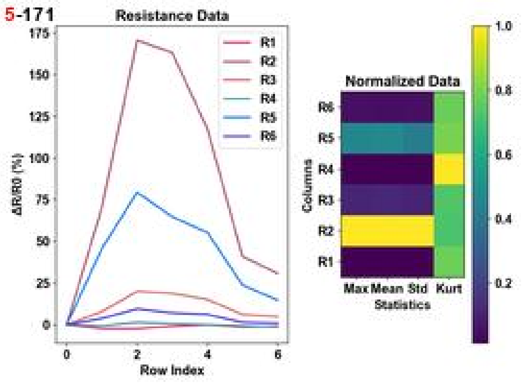

5-172
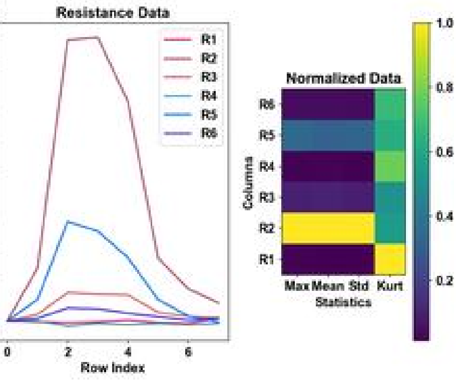

5-173
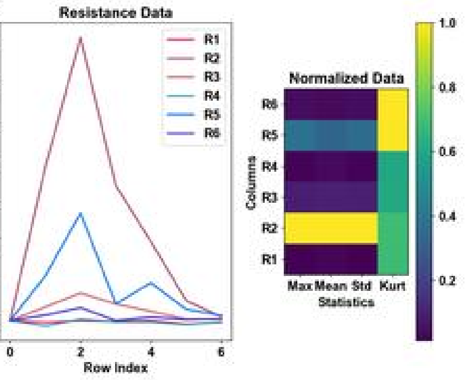

5-174
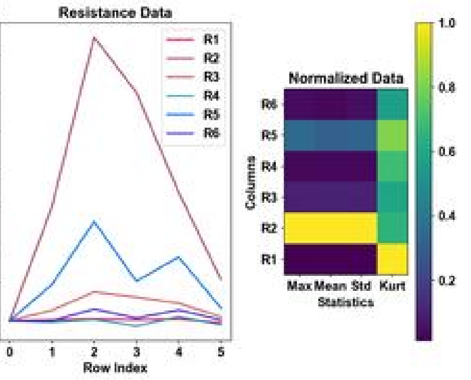

5-175
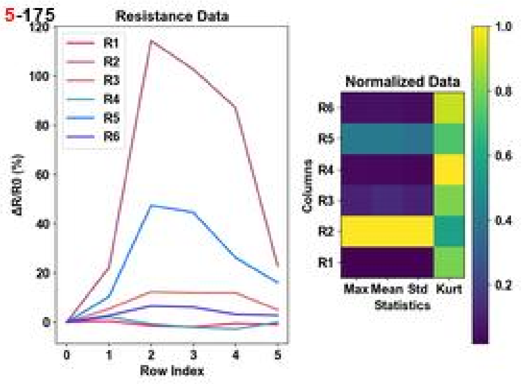

5-176
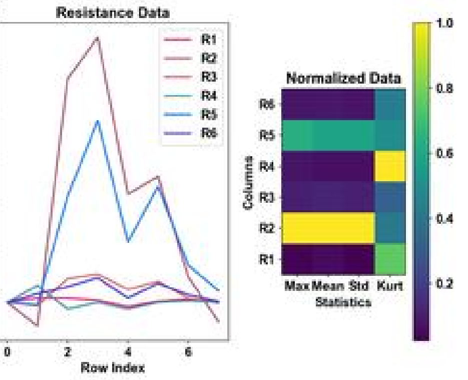

5-177
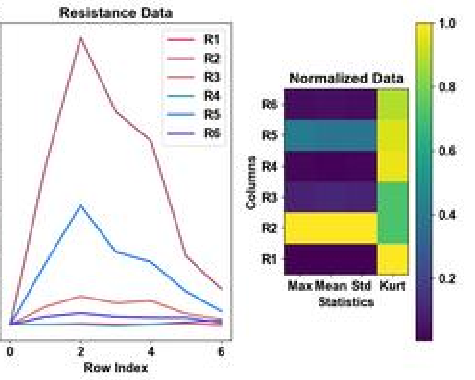

5-178
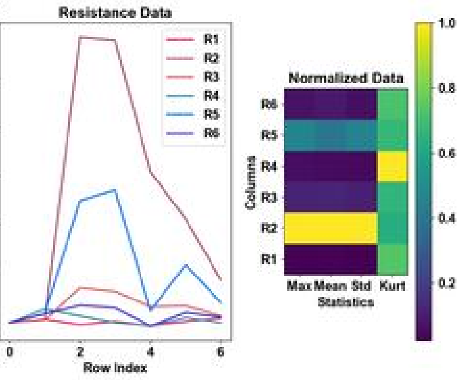

5-179
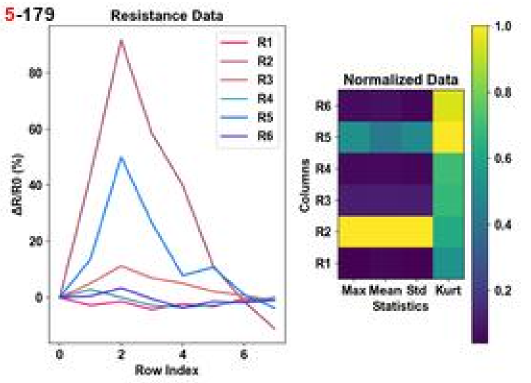

5-180
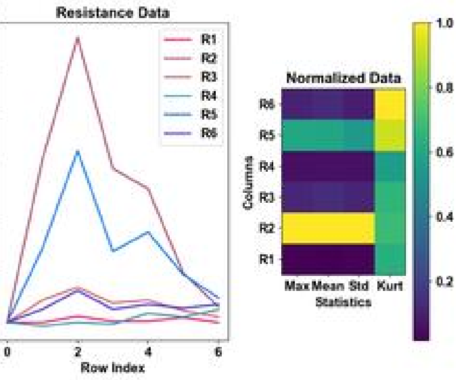

5-181
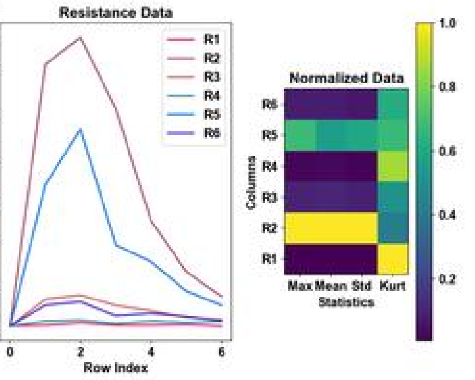

5-182
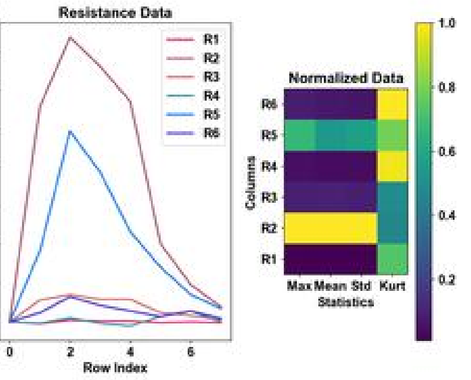

5-183
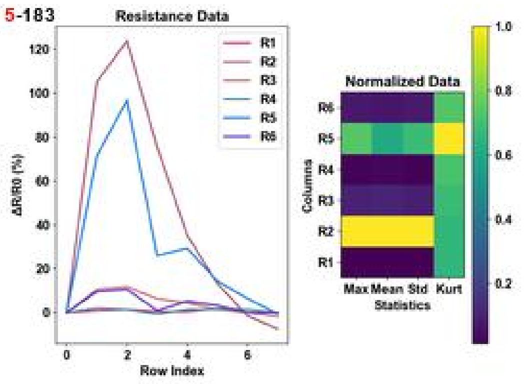

5-184
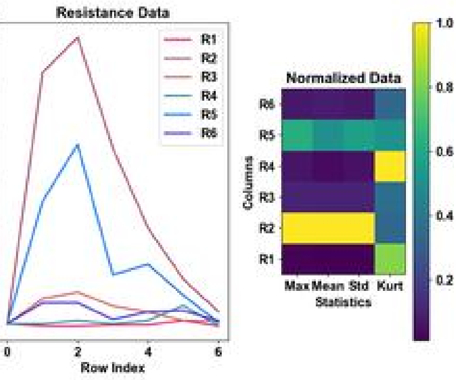

5-185
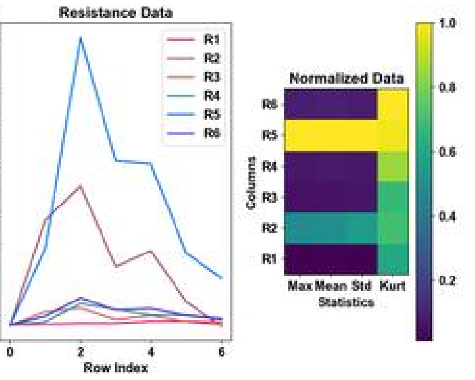

5-186
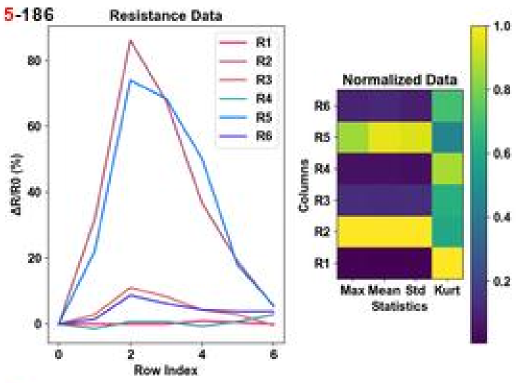

5-187
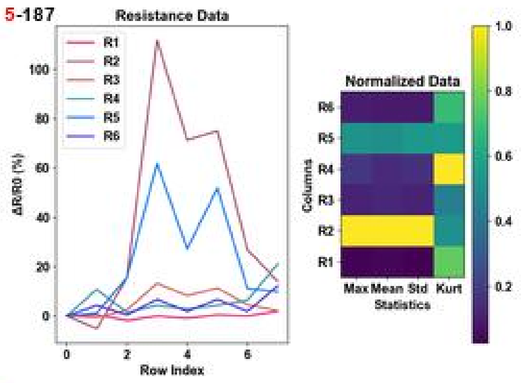

5-188
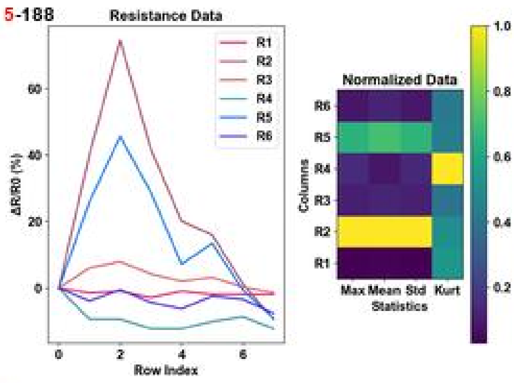

5-189
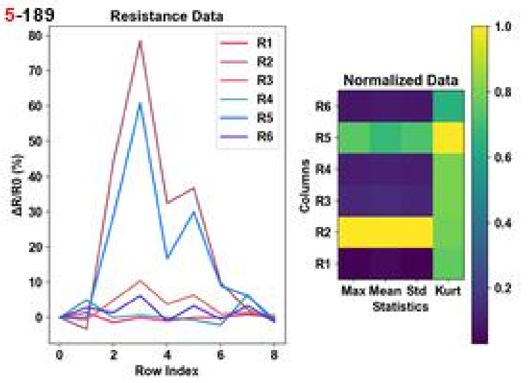

5-190
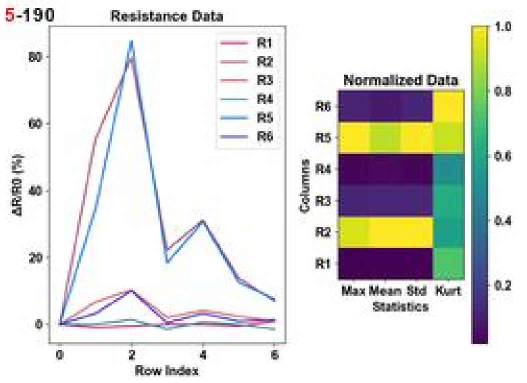

5-191
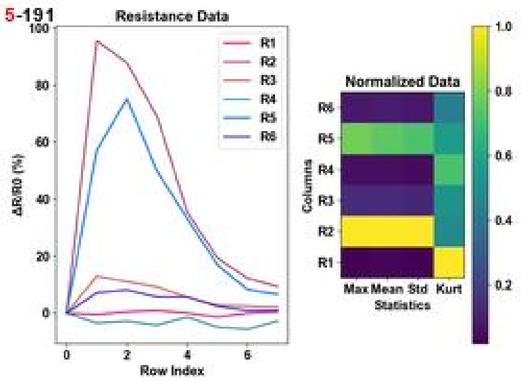

5-192
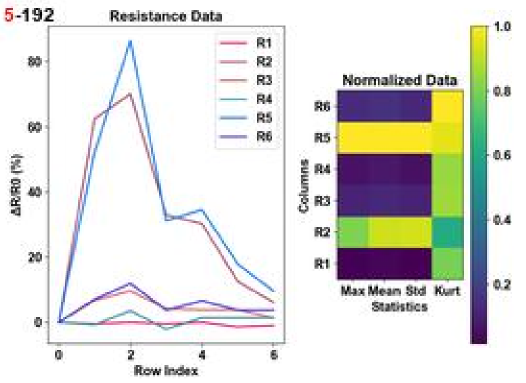

Pressure-sensitive data of type 5

5-193
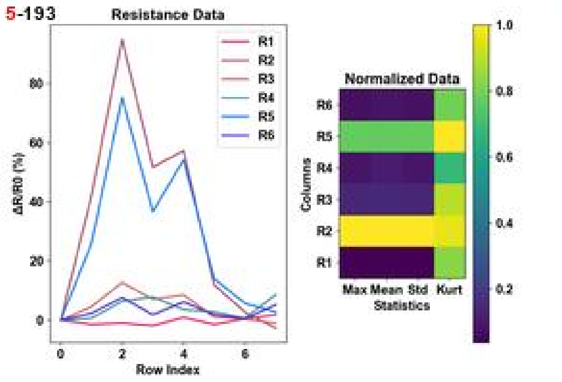

5-194
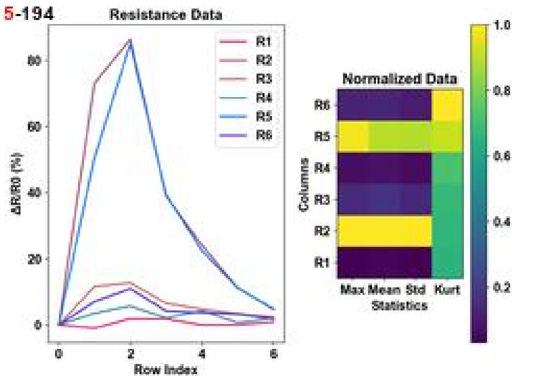

5-195
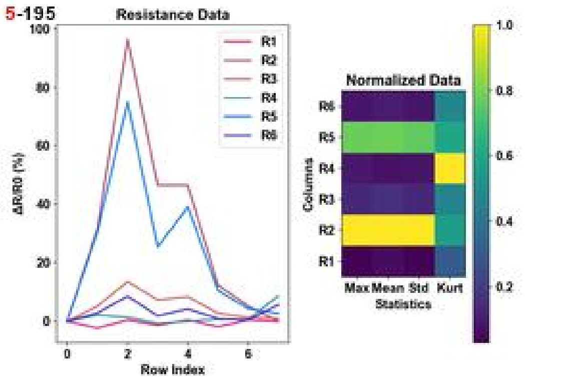

5-196
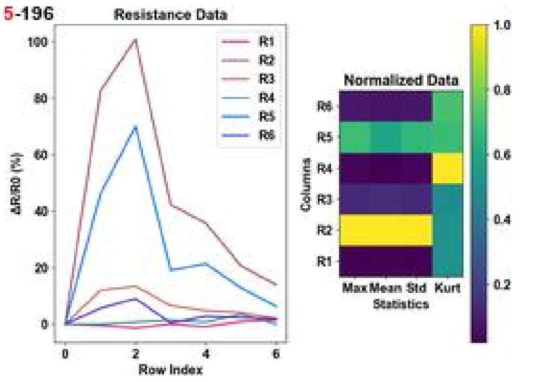

5-197
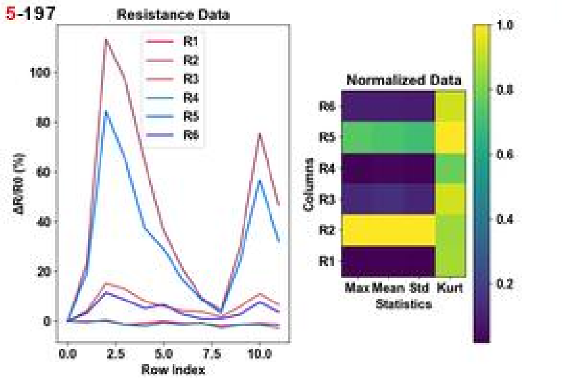

5-198
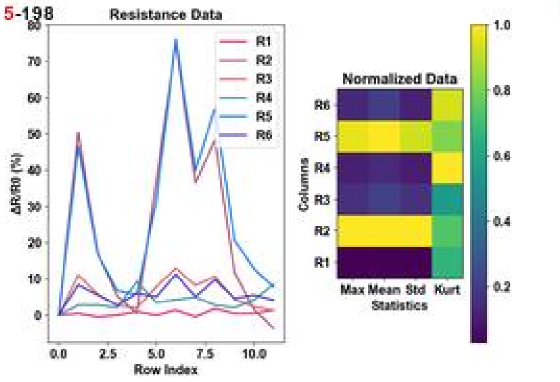

5-199
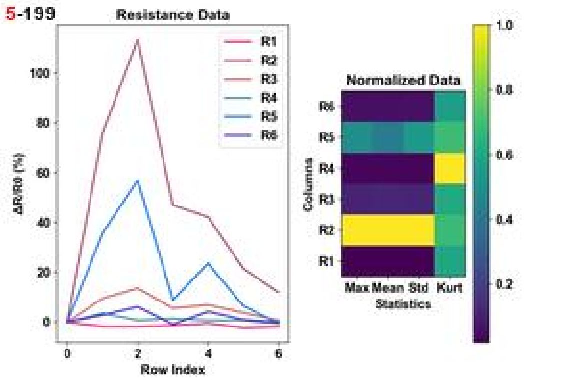

5-200
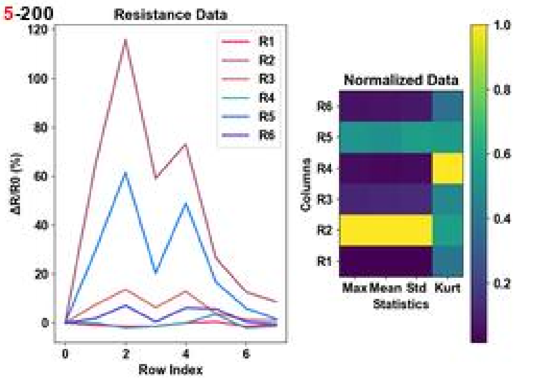

5-201
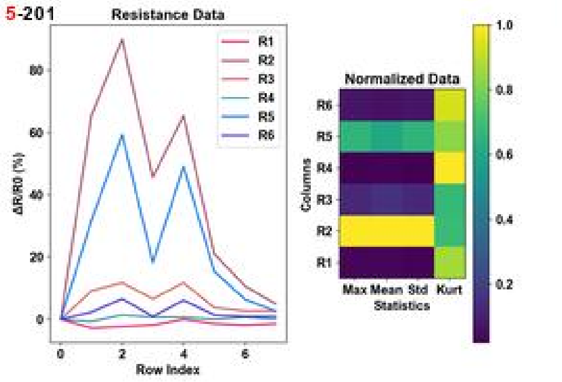

5-202
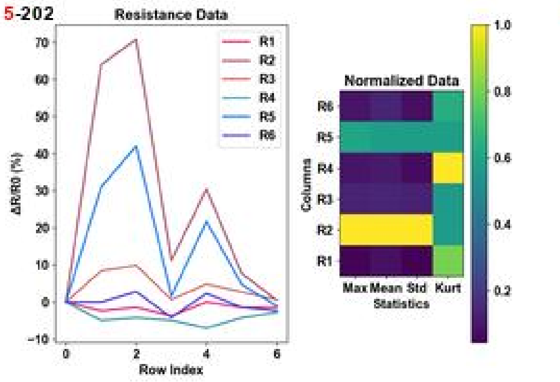

5-203
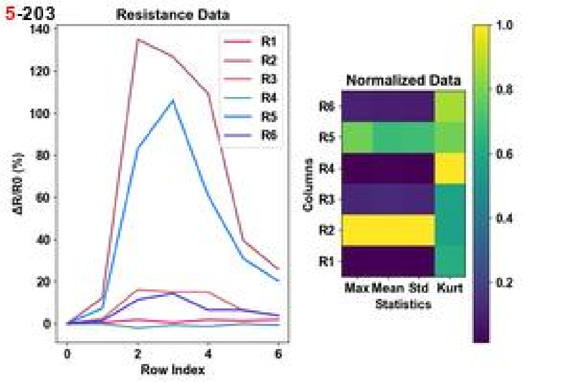

5-204
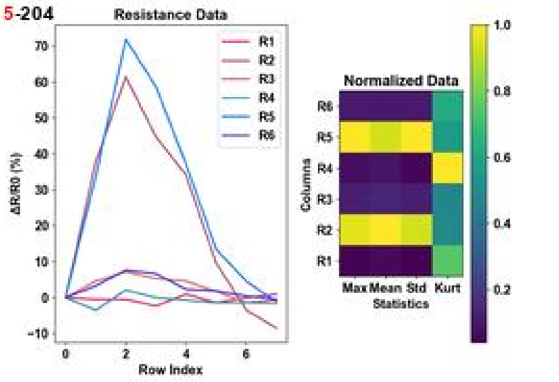

5-205
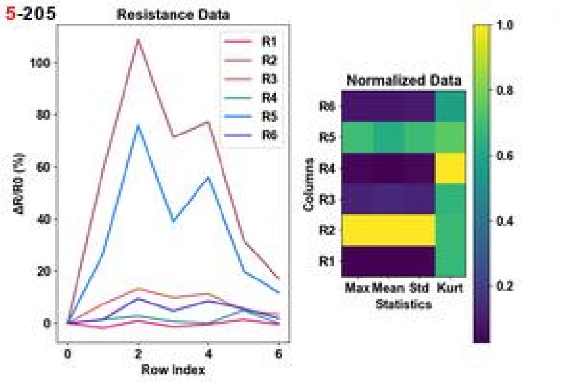

5-206
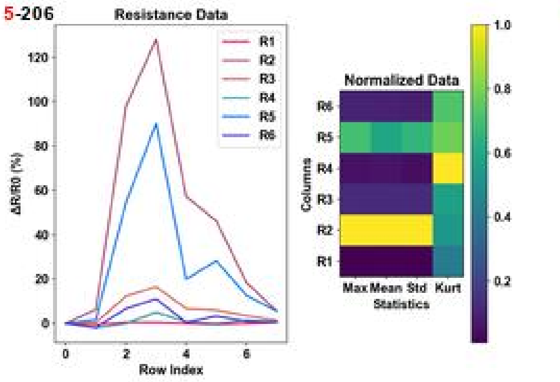

5-207
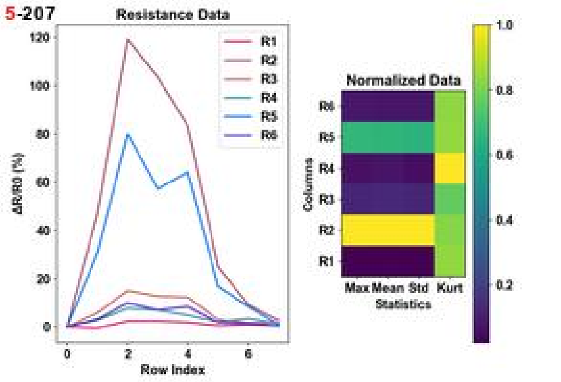

5-208
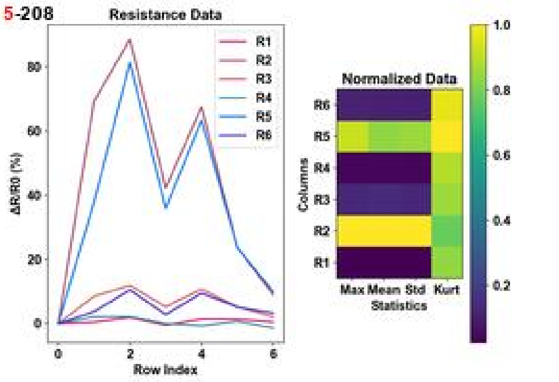

5-209
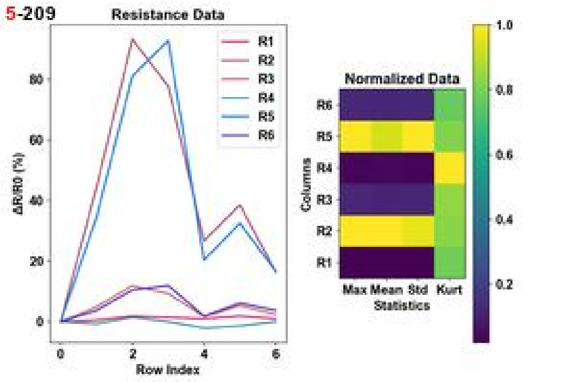

5-210
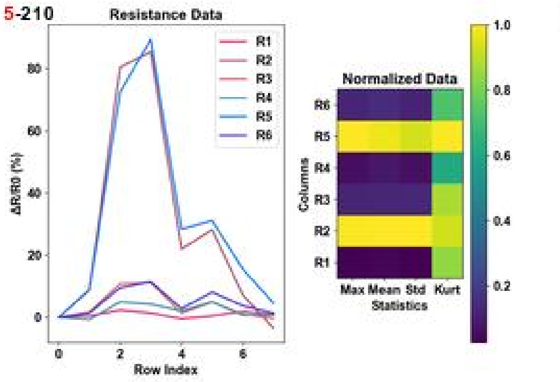

5-211
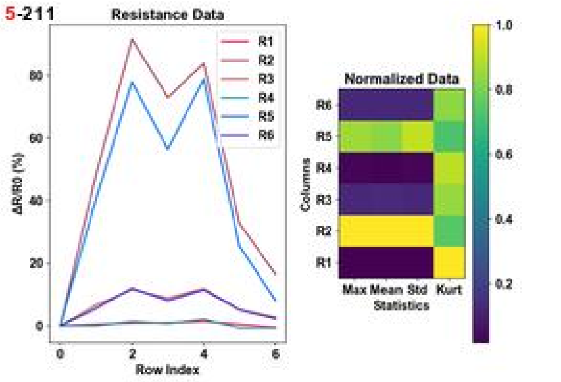

5-212
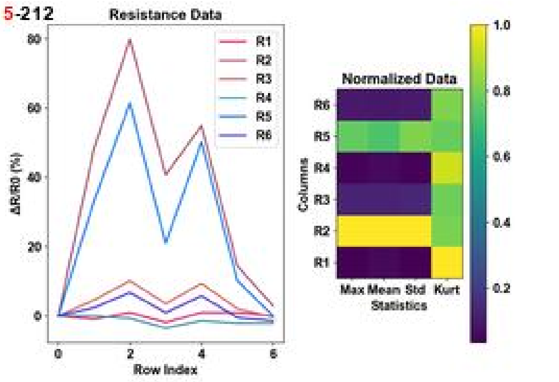

5-213
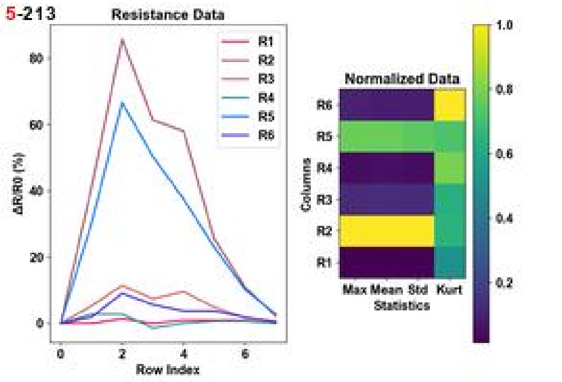

5-214
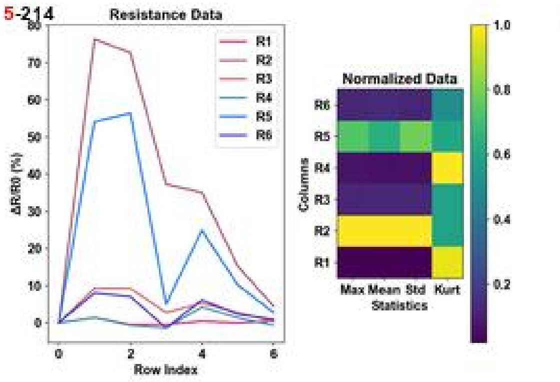

5-215
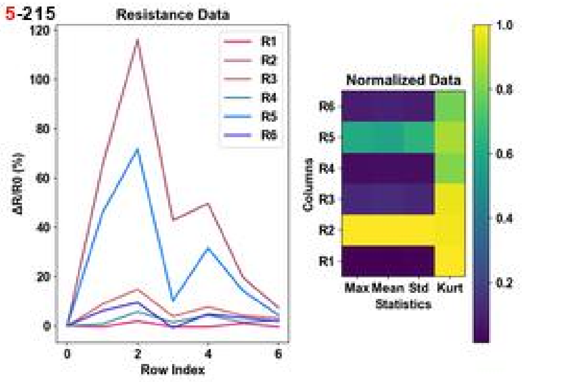

5-216
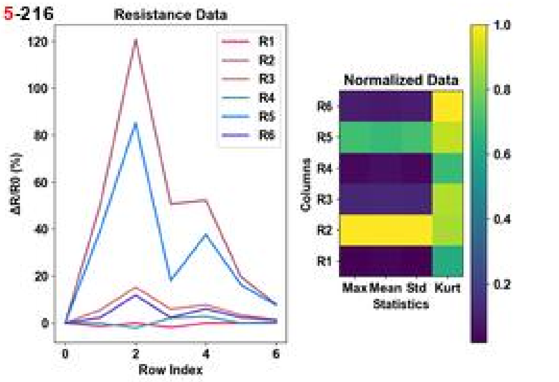

5-217
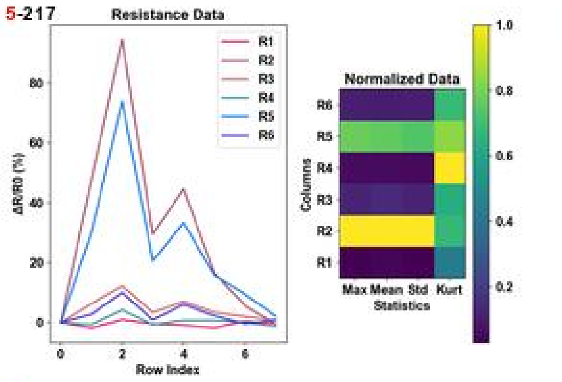

5-218
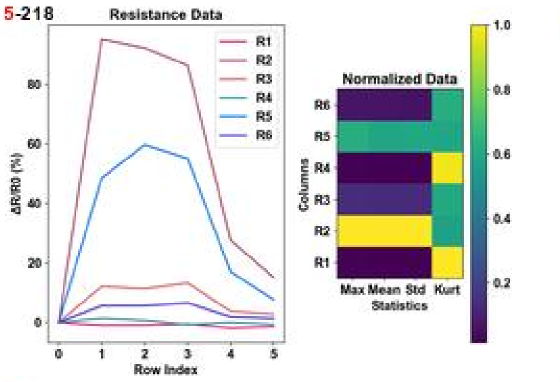

5-219
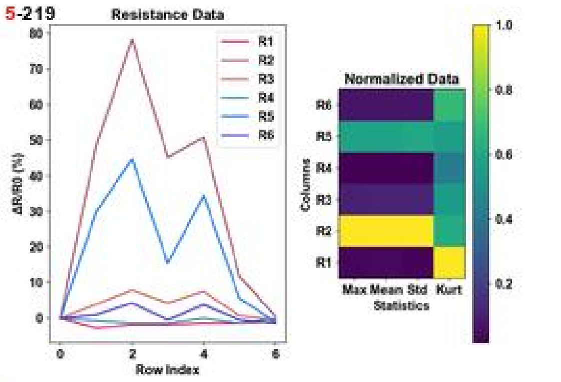

5-220
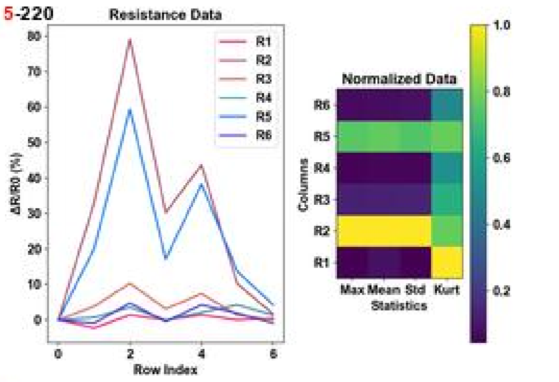

5-221
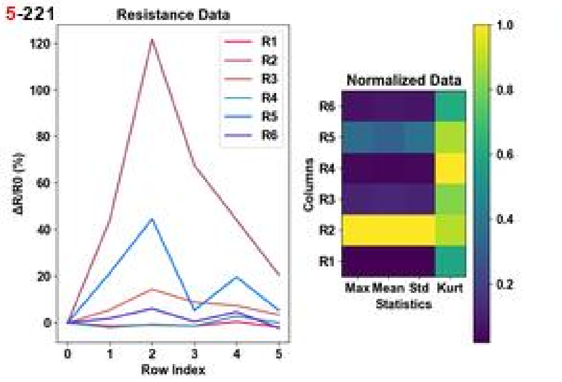

5-222
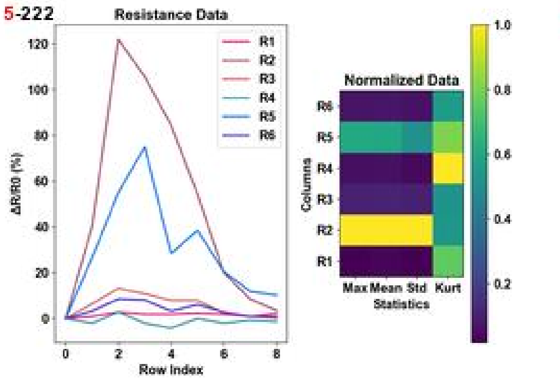

5-223
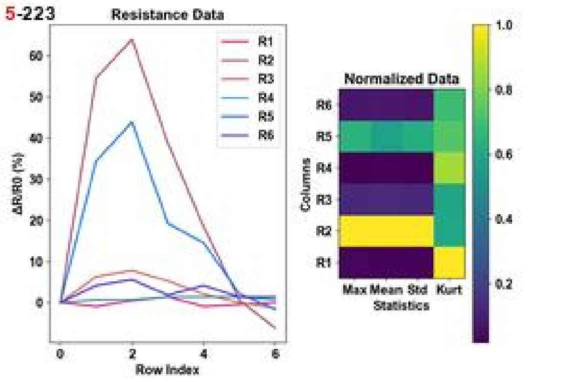

5-224
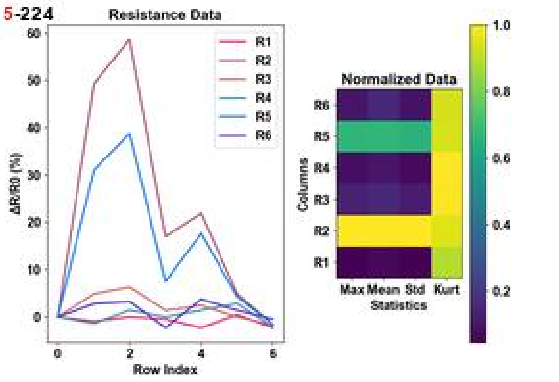

5-225
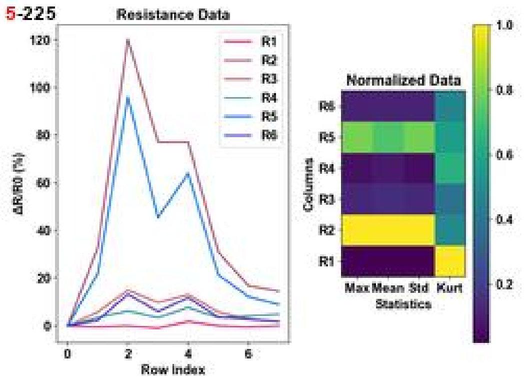

5-226
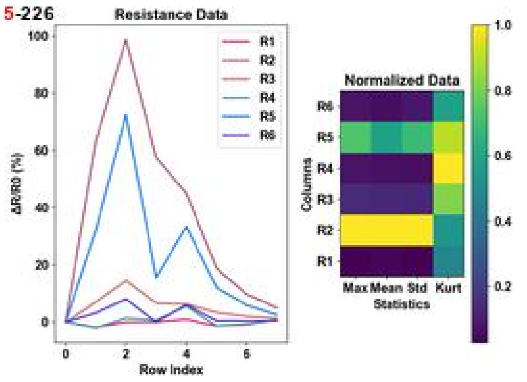

5-227
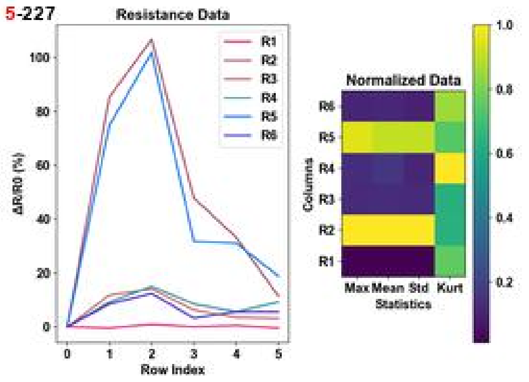

5-228
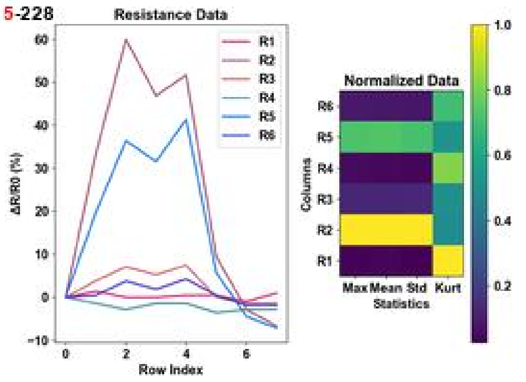

5-229
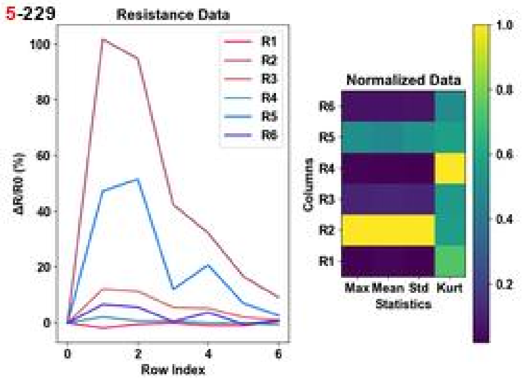

5-230
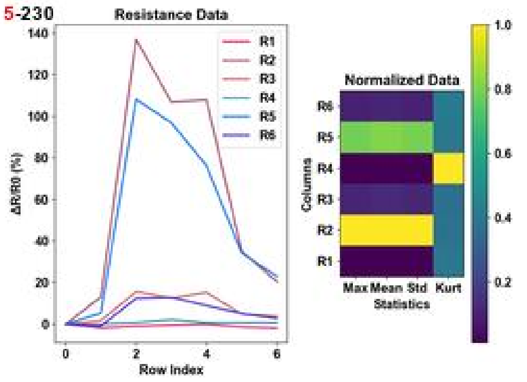

5-231
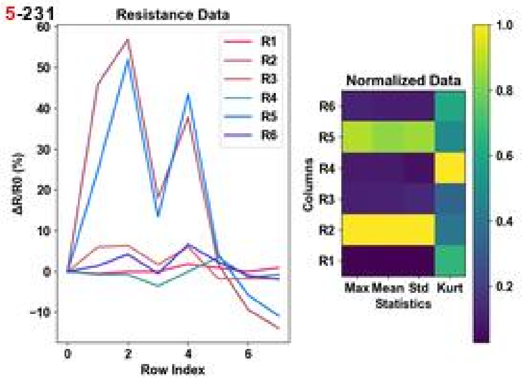

5-232
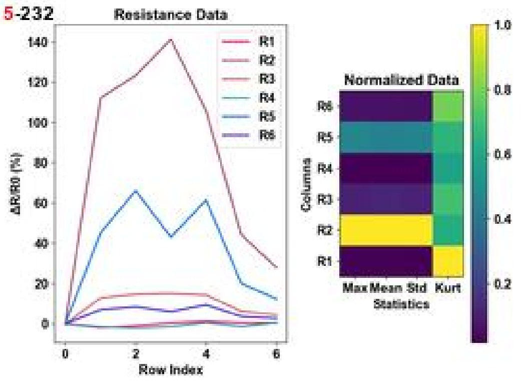

5-233
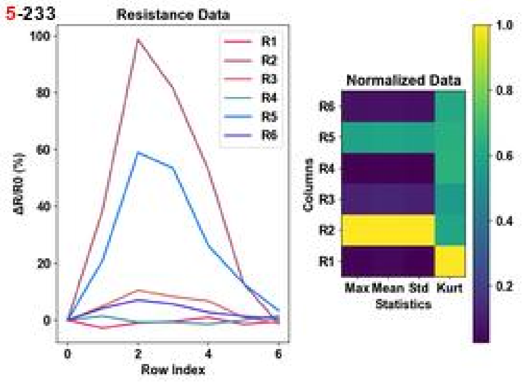

5-234
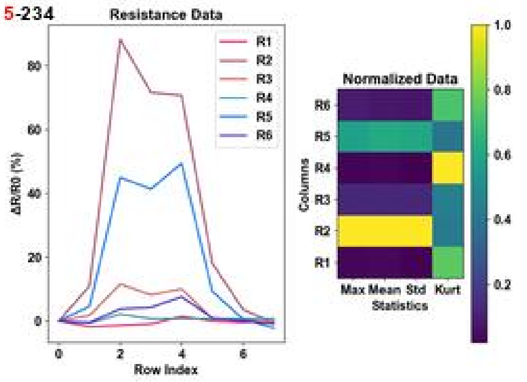

5-235
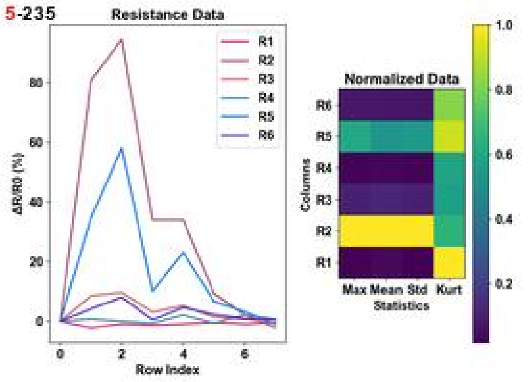

5-236
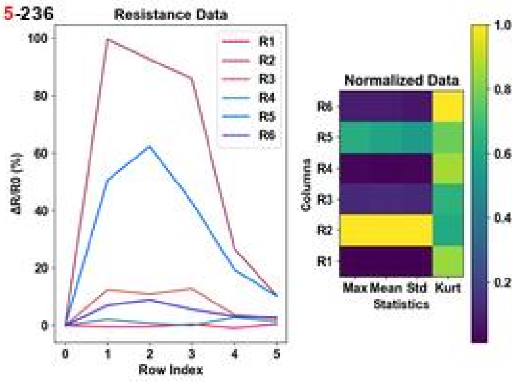

5-237
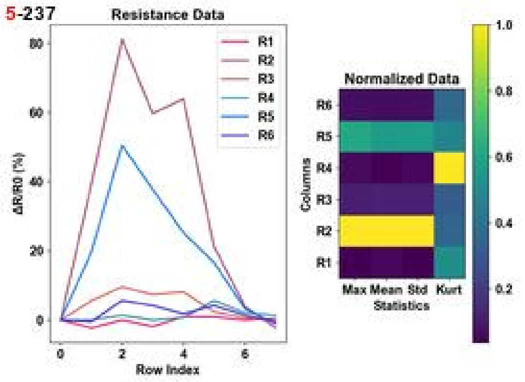

5-238
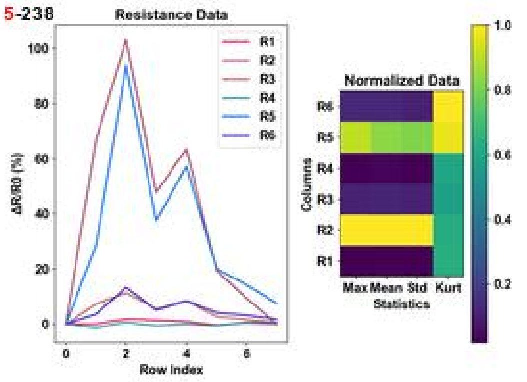

5-239
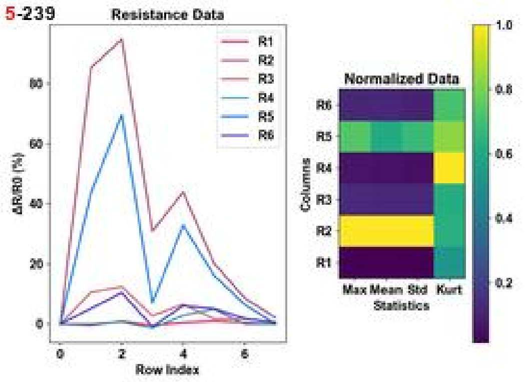

5-240
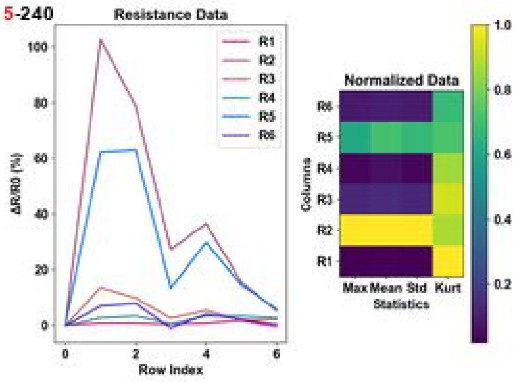

Pressure-sensitive data of type 6

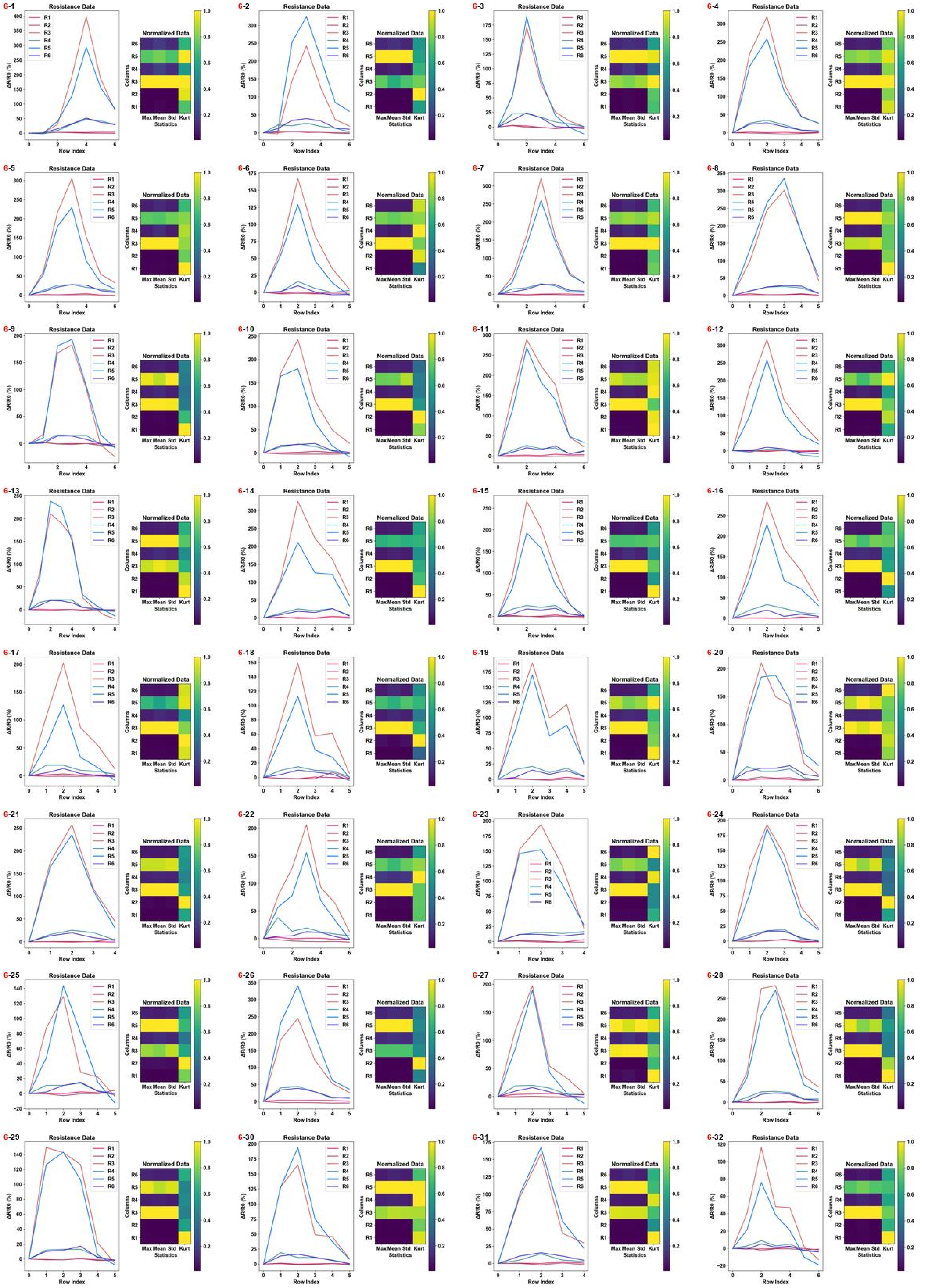

6-33
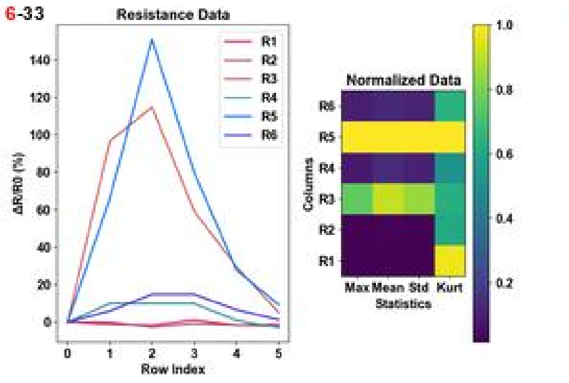

6-34
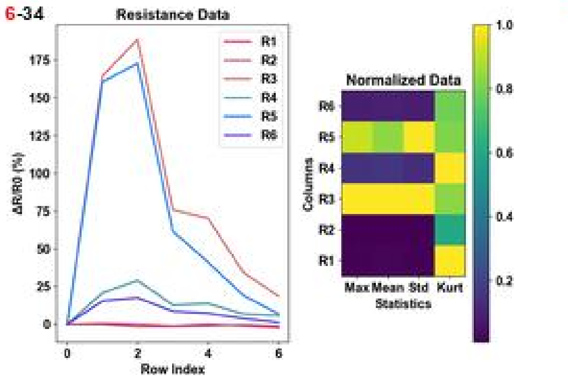

6-35
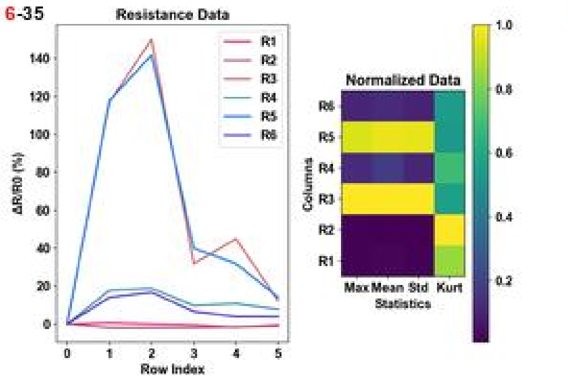

6-36
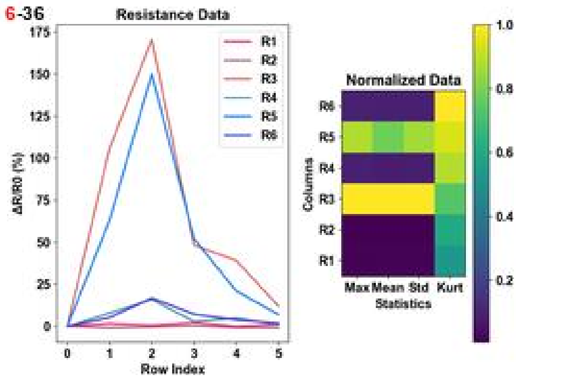

6-37
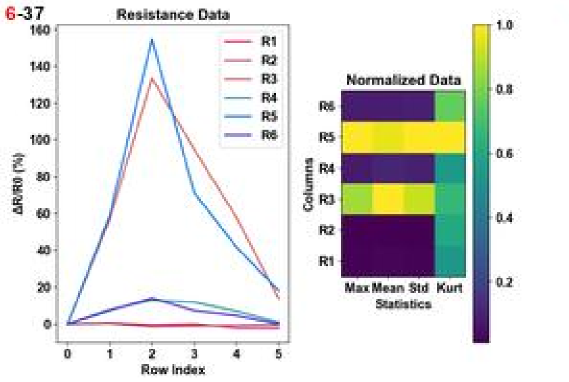

6-38
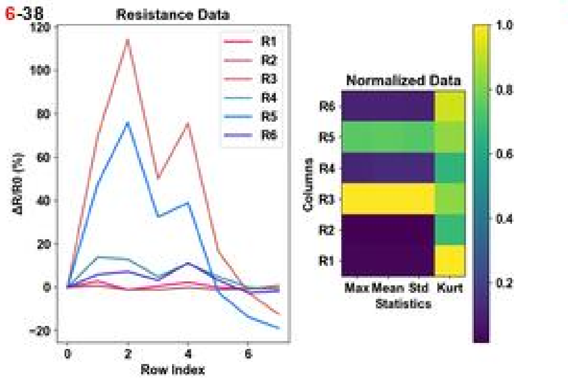

6-39
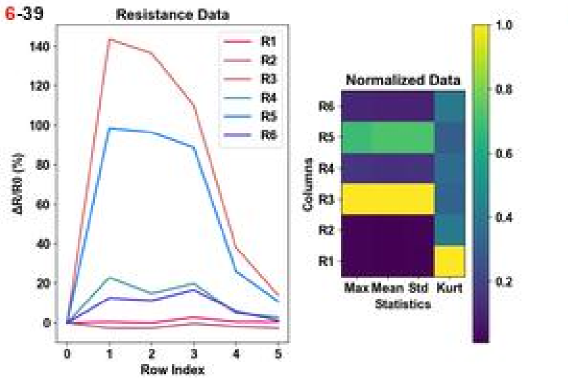

6-40
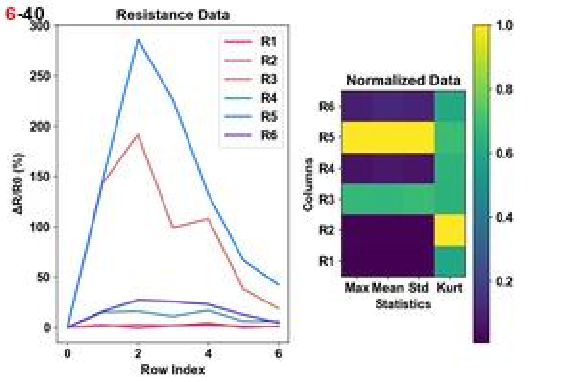

6-41
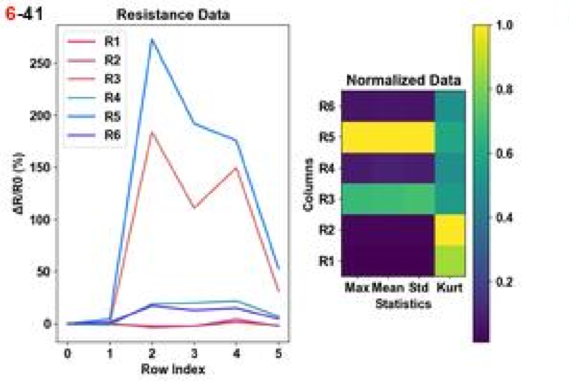

6-42
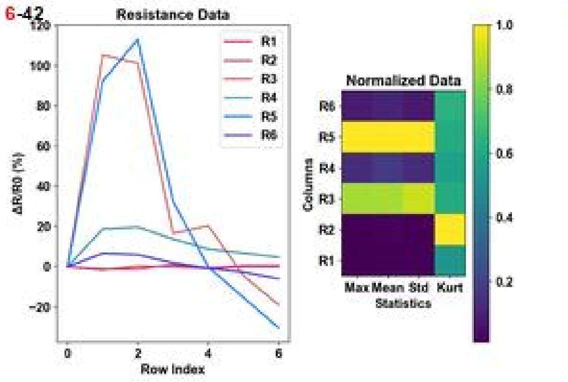

6-43
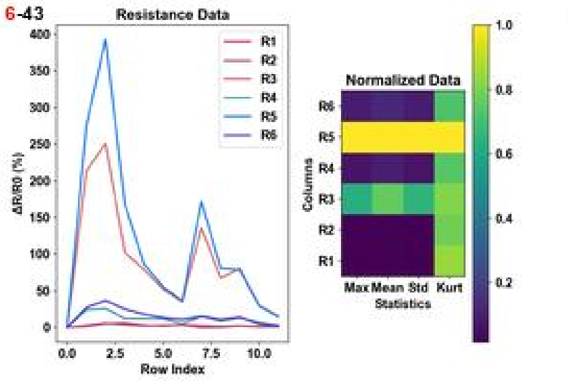

6-44
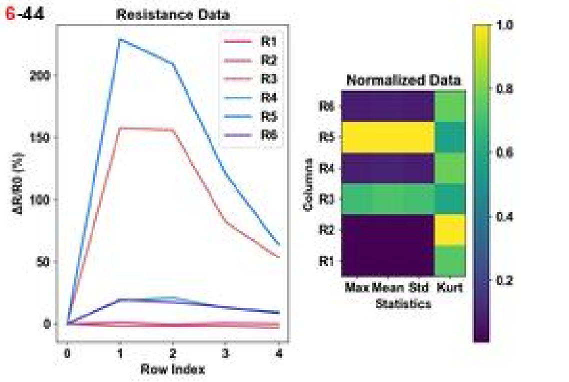

6-45
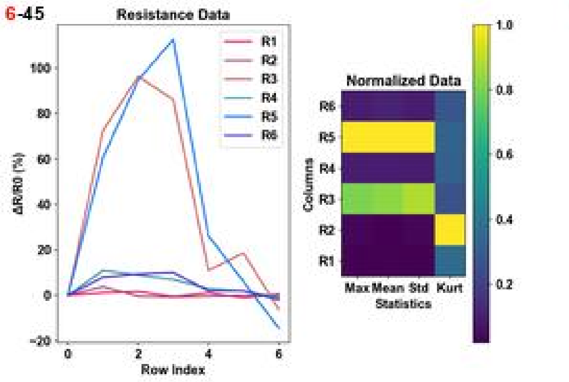

6-46
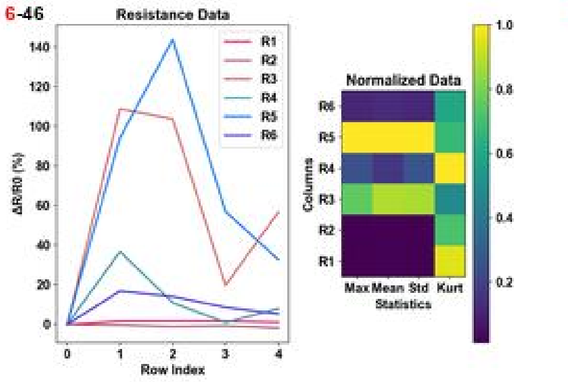

6-47
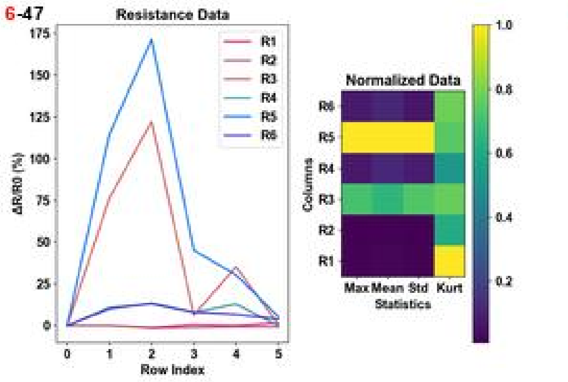

6-48
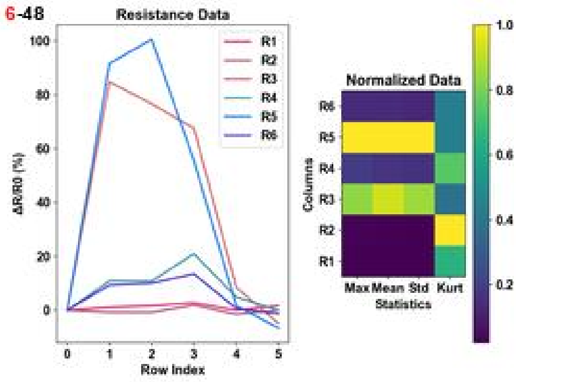

6-49
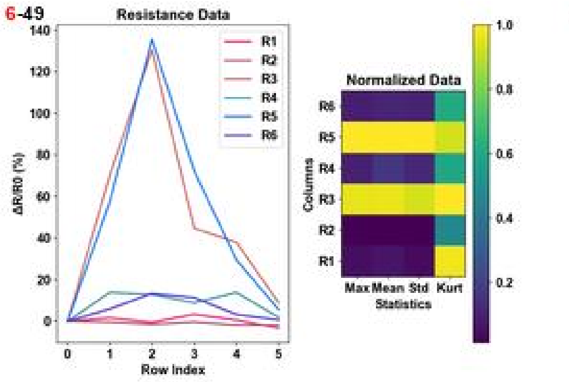

6-50
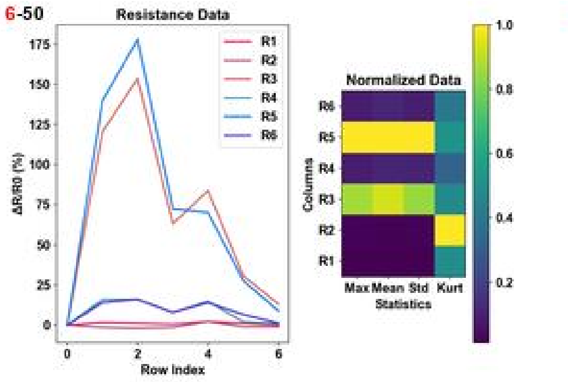

6-51
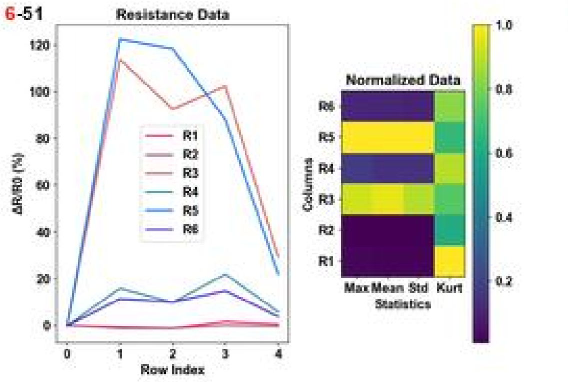

6-52
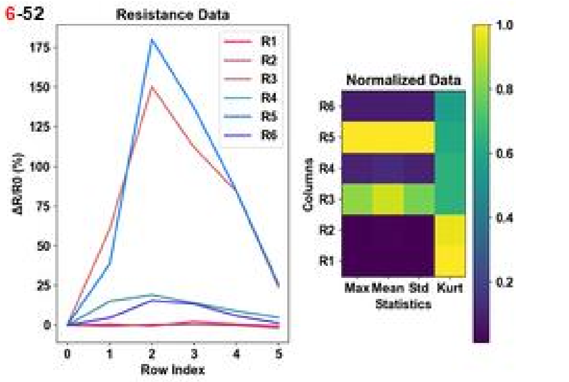

6-53
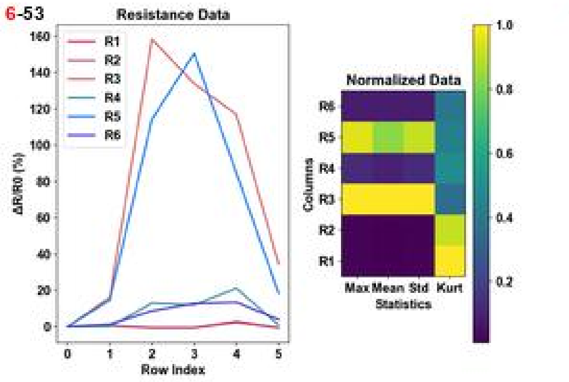

6-54
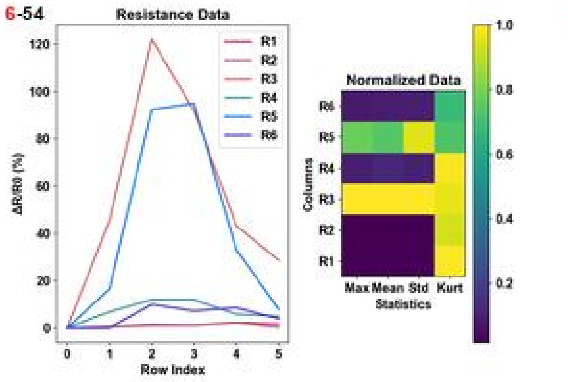

6-55
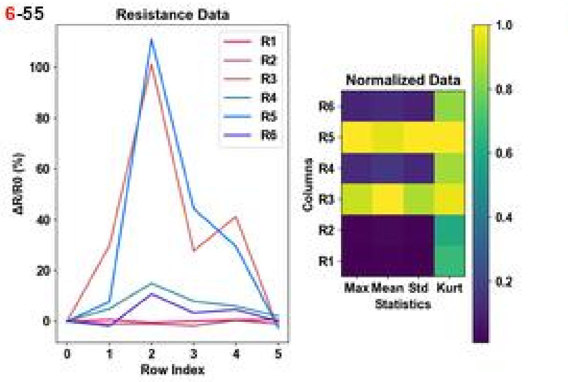

6-56
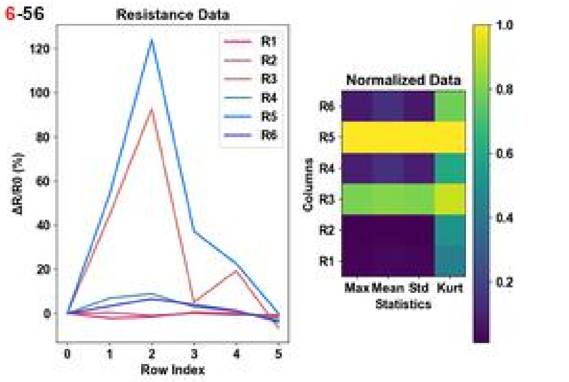

6-57
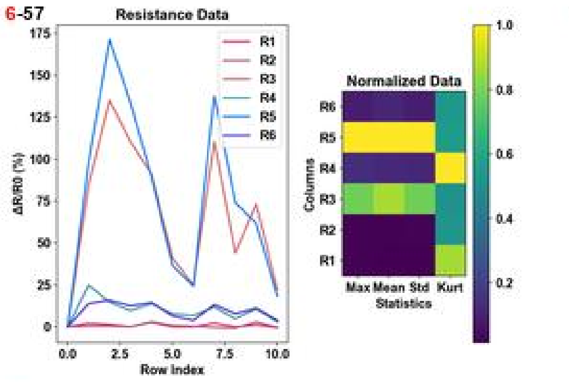

6-58
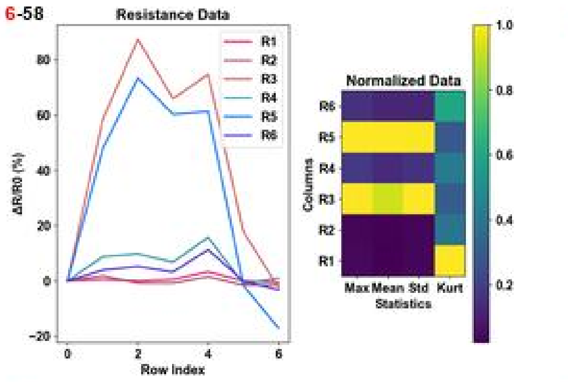

6-59
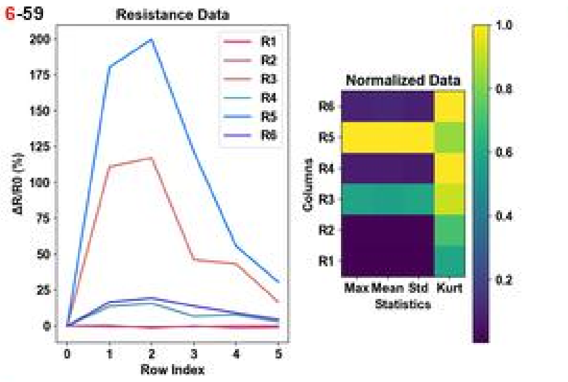

6-60
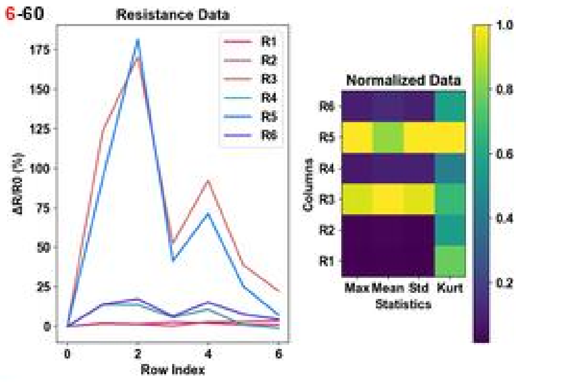

6-61
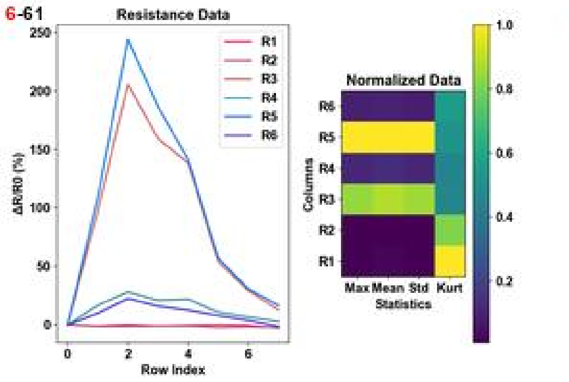

6-62
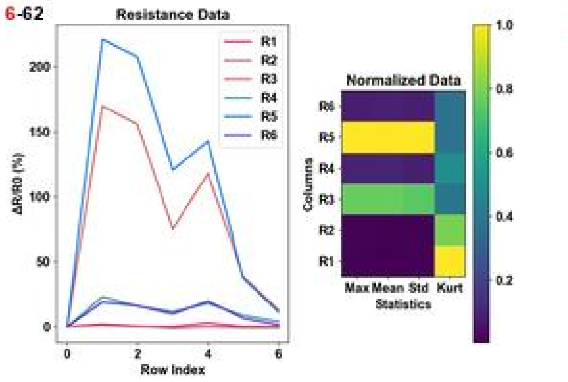

6-63
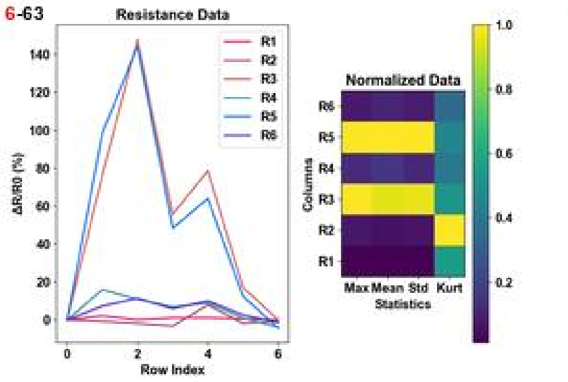

6-64
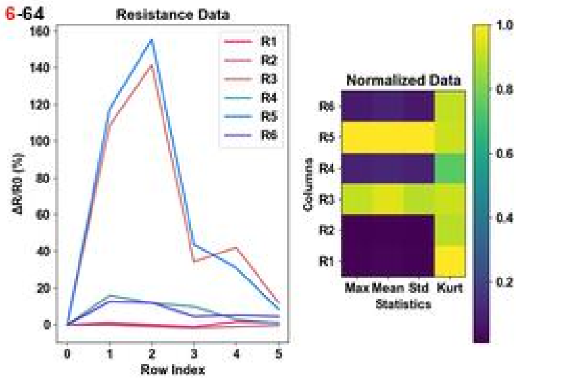

6-65
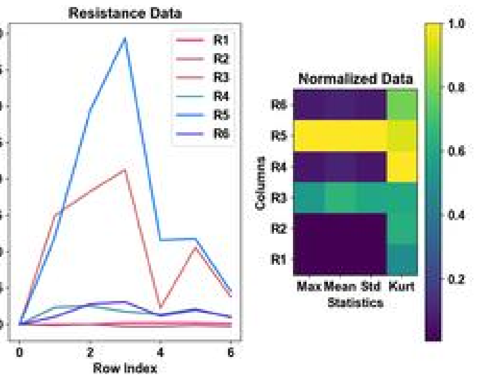

6-66
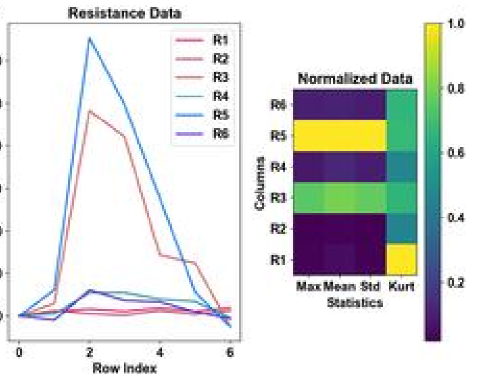

6-67
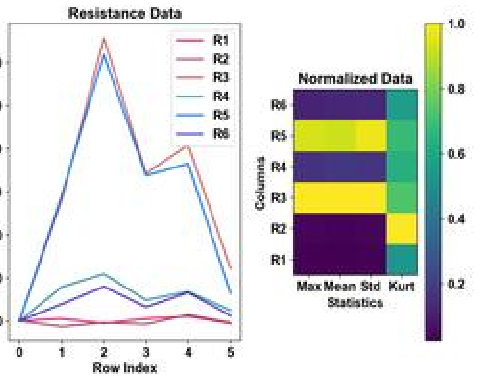

6-68
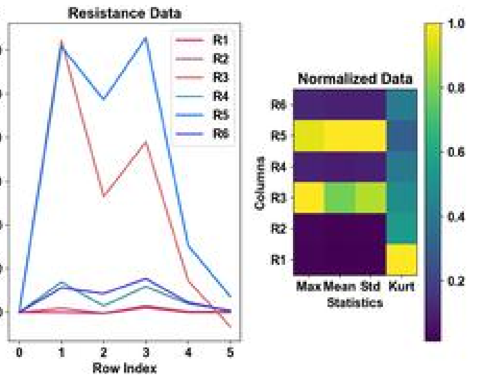

6-69
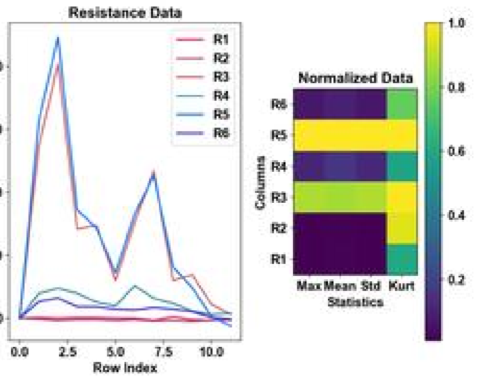

6-70
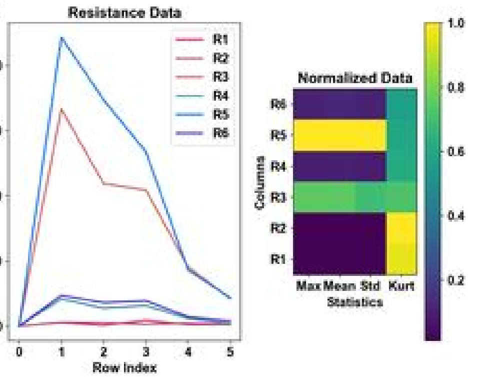

6-71
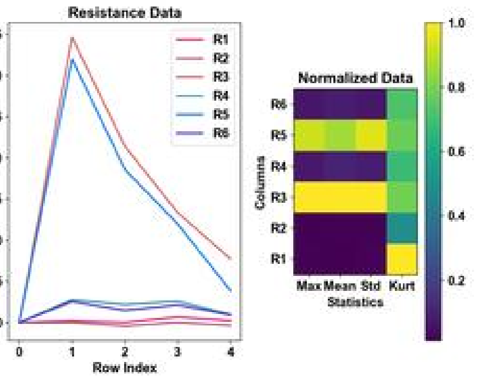

6-72
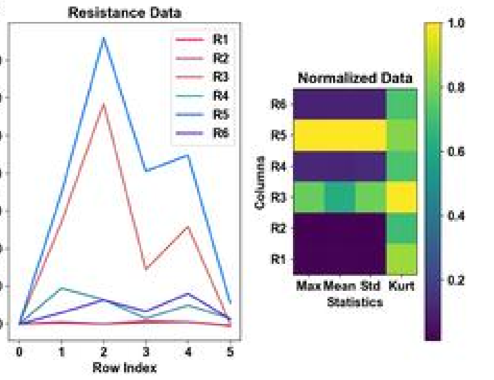

6-73
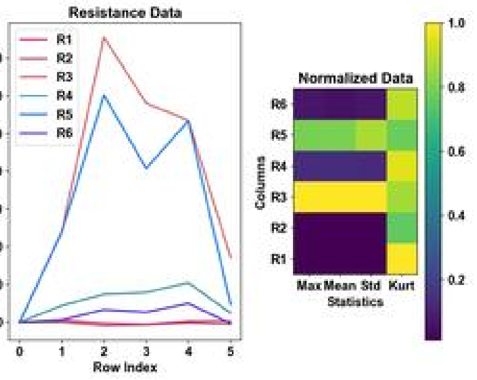

6-74
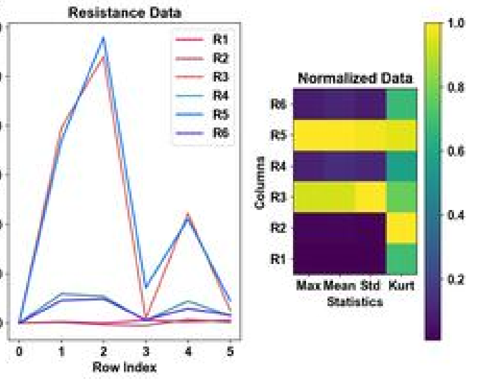

6-75
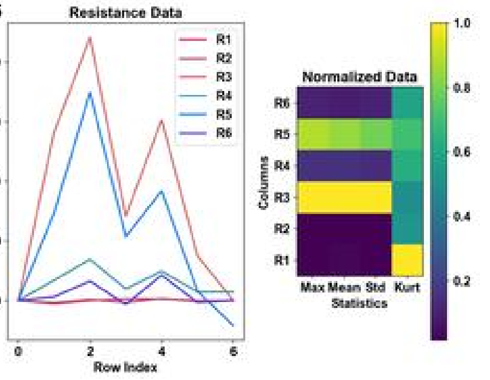

6-76
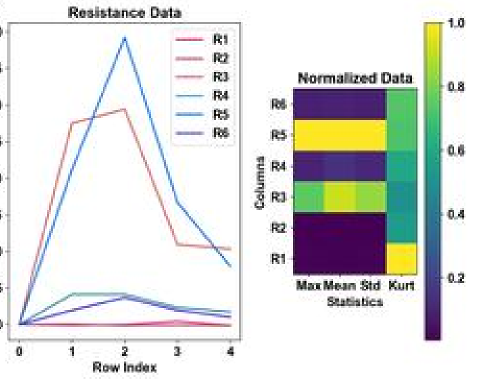

6-77
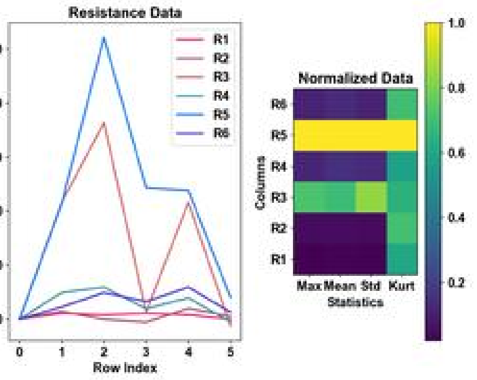

6-78
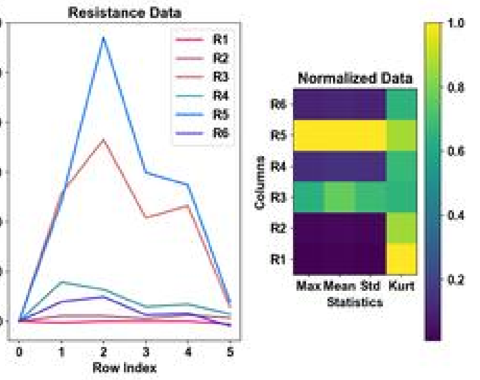

6-79
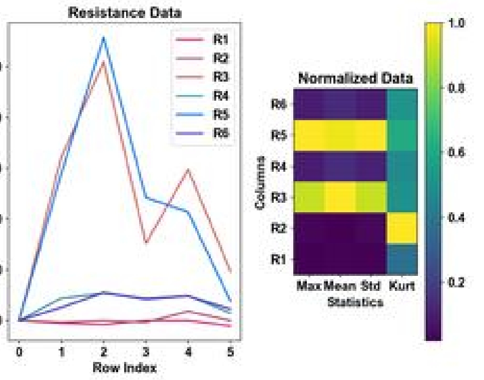

6-80
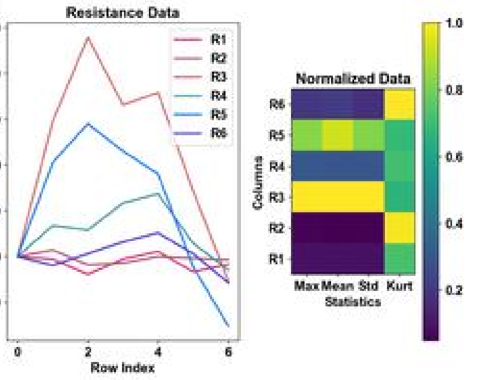

6-81
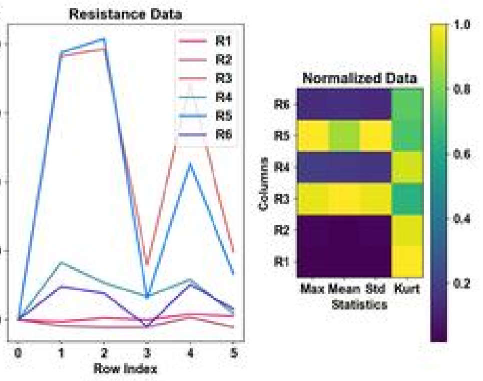

6-82
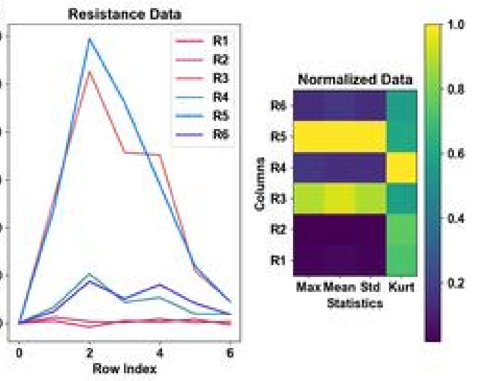

6-83
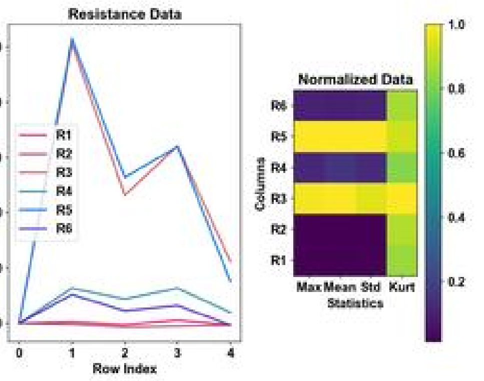

6-84
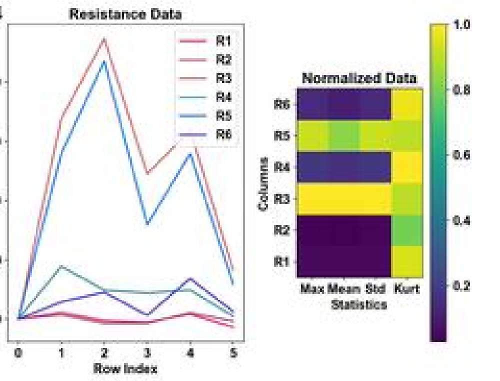

6-85
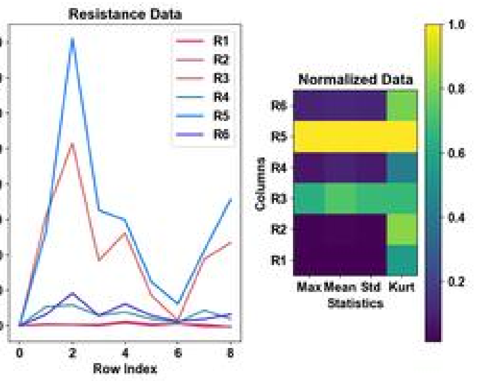

6-86
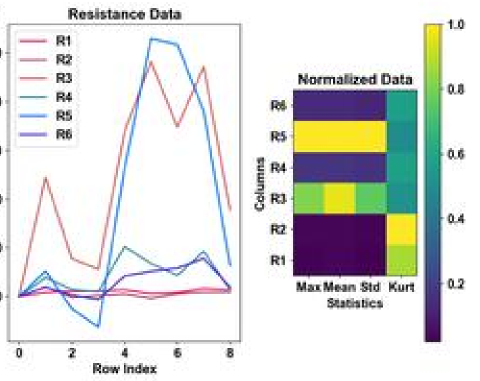

6-87
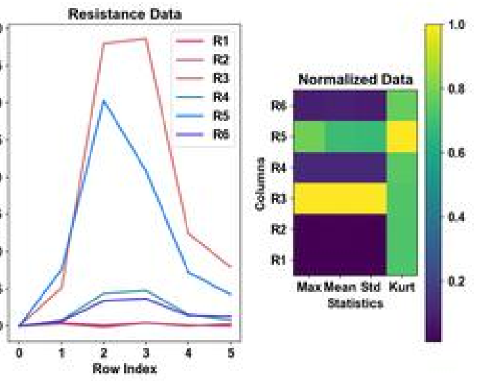

6-88
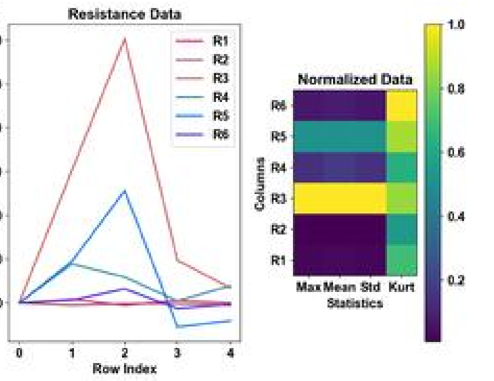

6-89
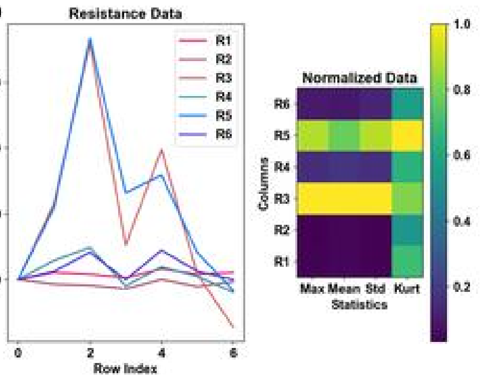

6-90
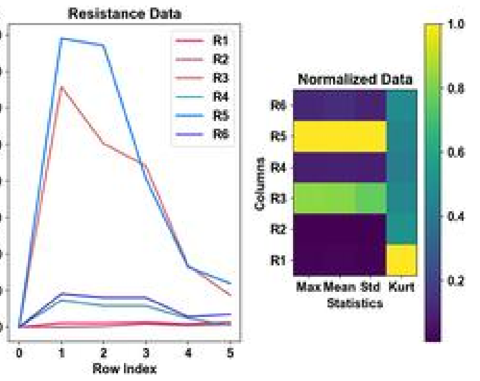

6-91
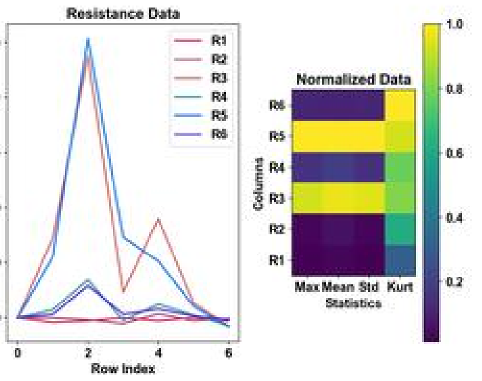

6-92
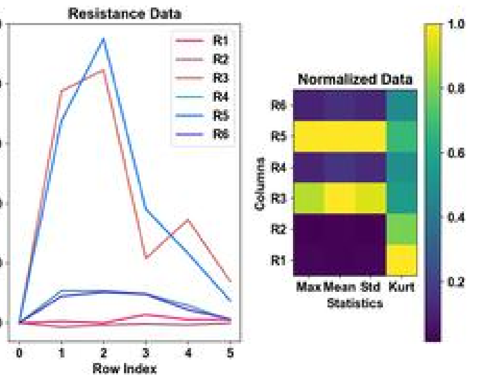

6-93
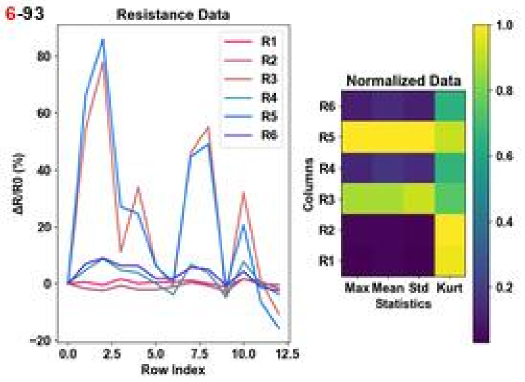

6-94
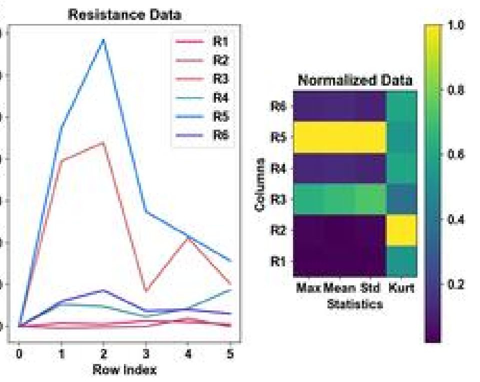

6-95
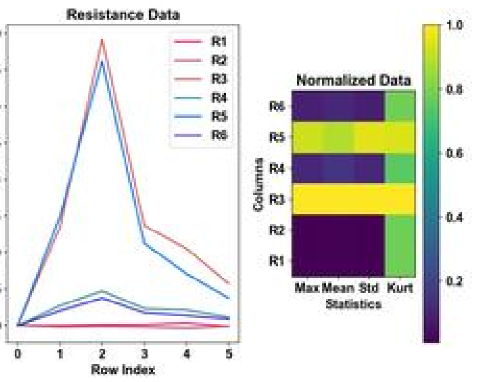

6-96
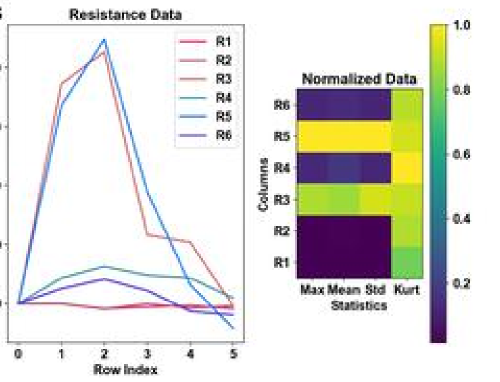

6-97
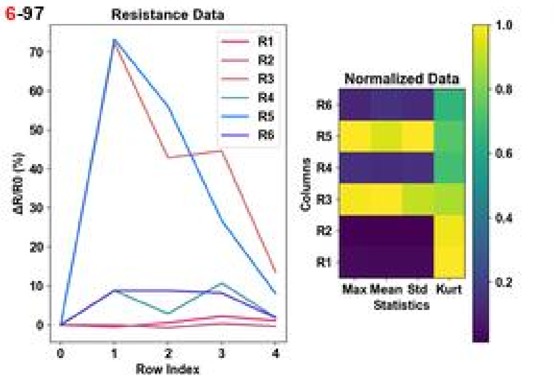

6-98
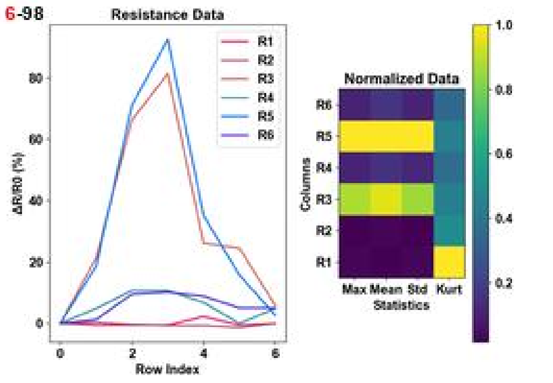

6-99
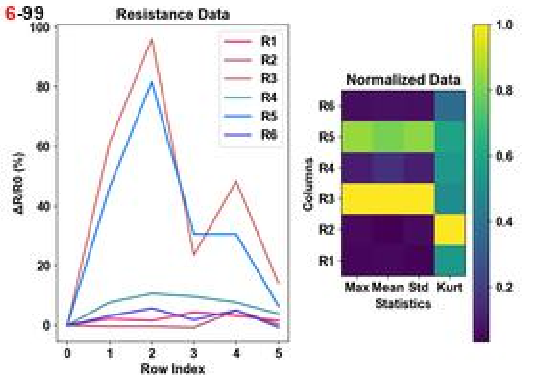

6-100
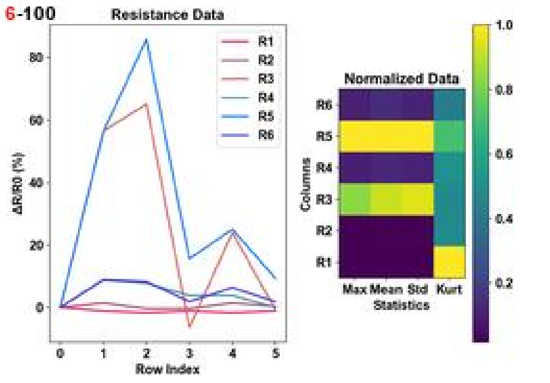

6-101
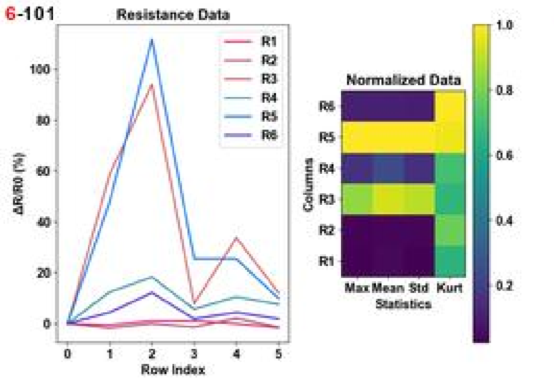

6-102
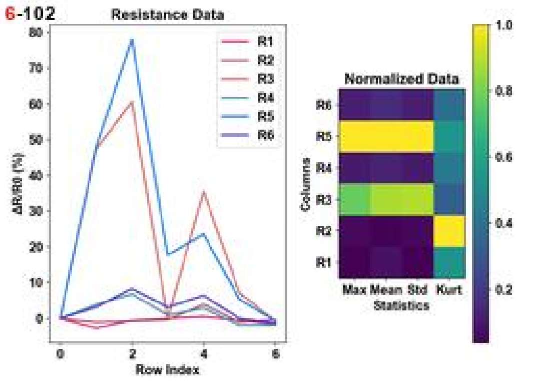

6-103
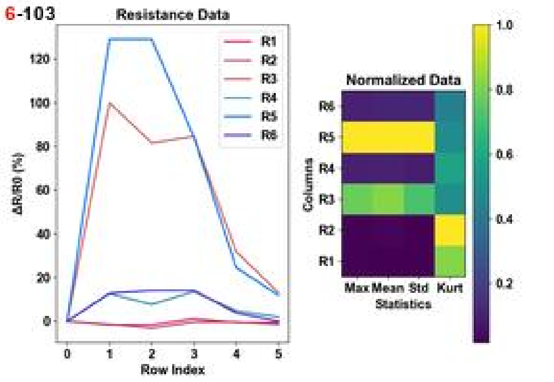

6-104
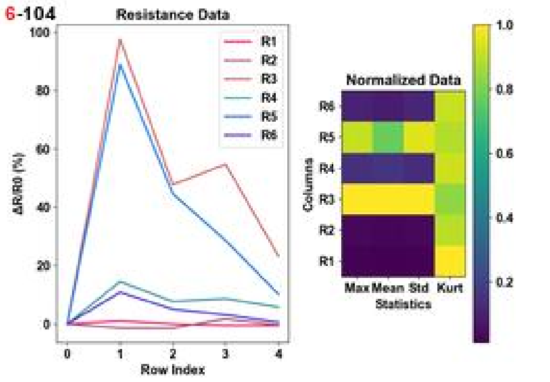

6-105
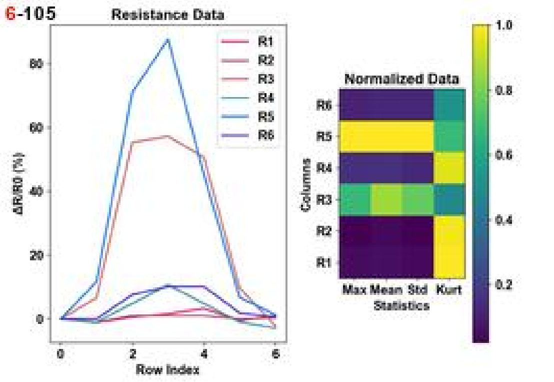

6-106
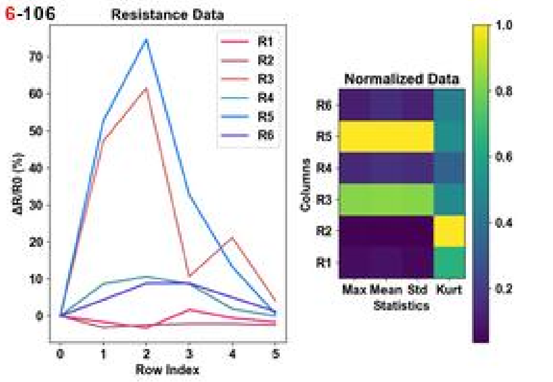

6-107
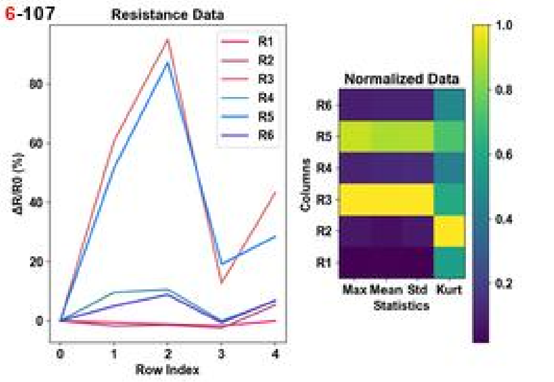

6-108
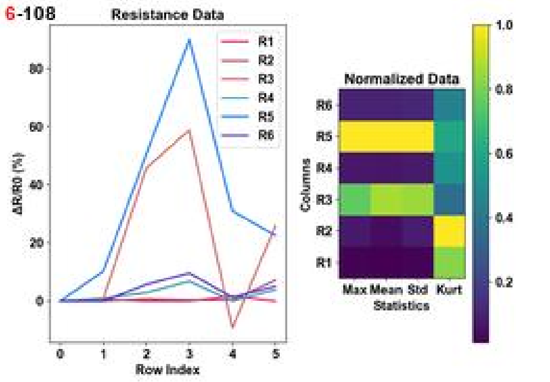

6-109
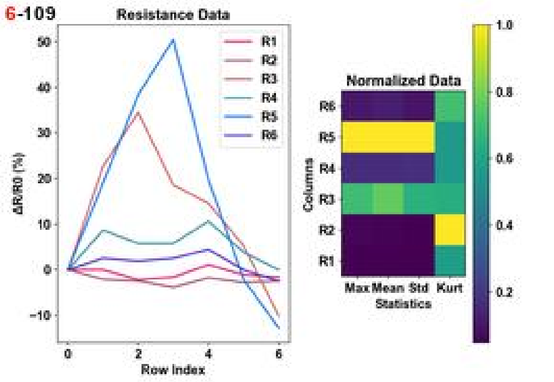

6-110
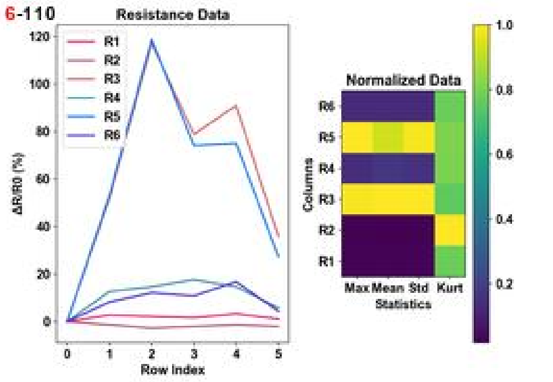

6-111
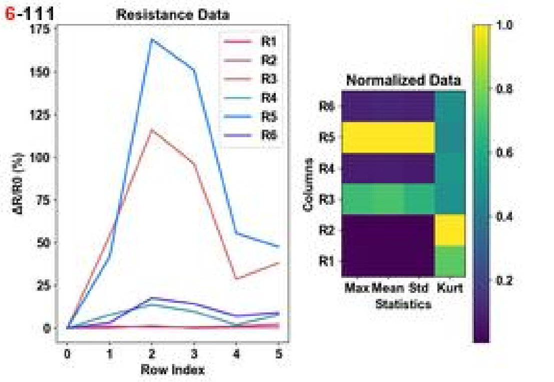

6-112
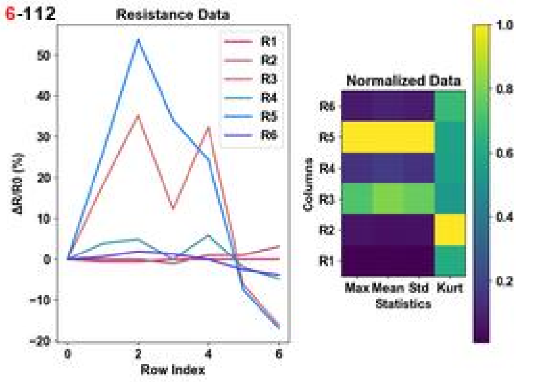

6-113
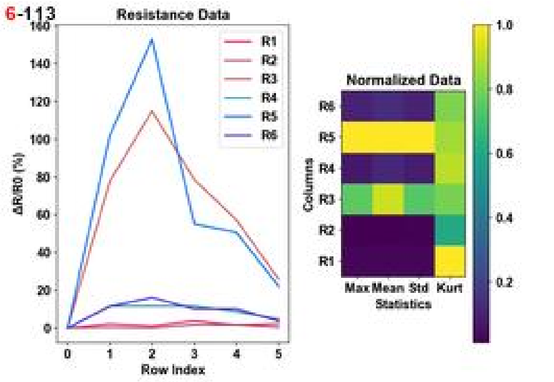

6-114
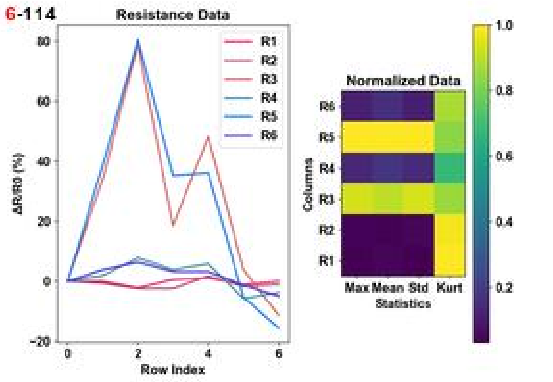

6-115
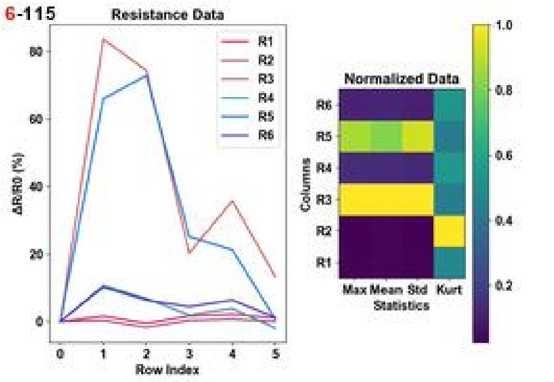

6-116
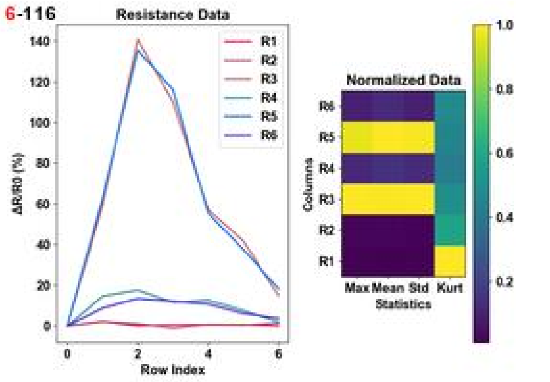

6-117
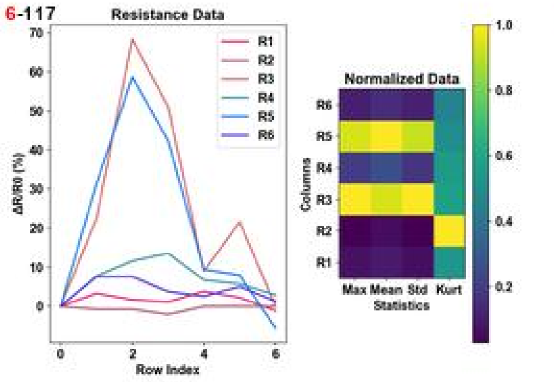

6-118
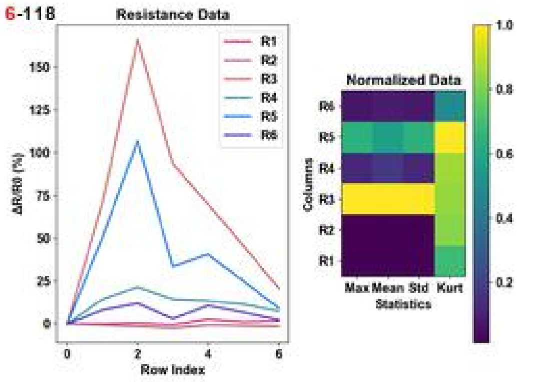

6-119
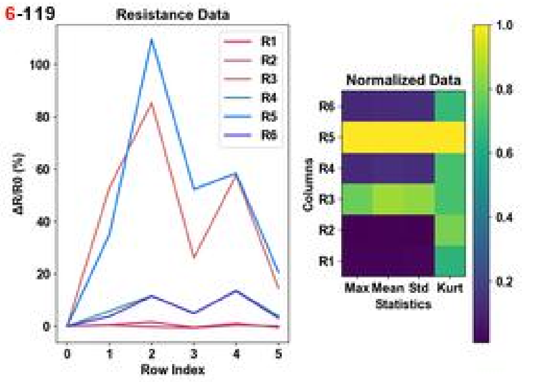

6-120
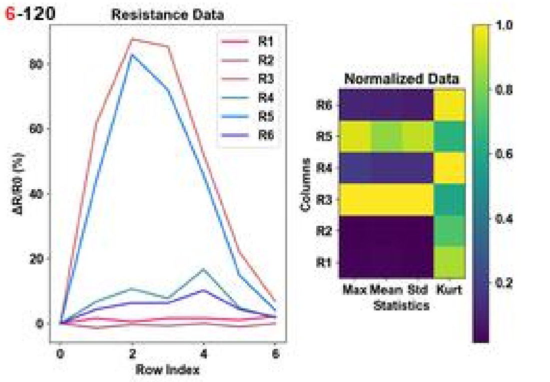

6-121
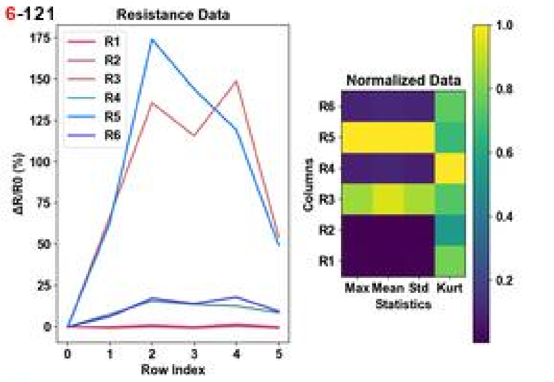

6-122
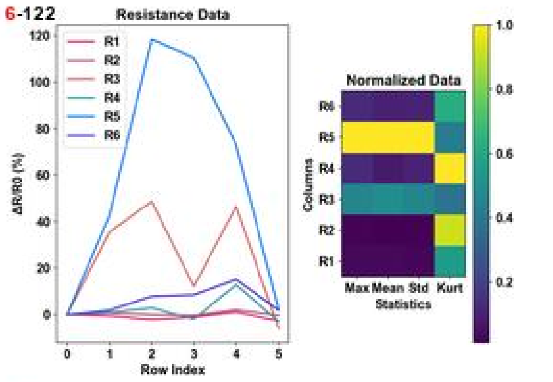

6-123
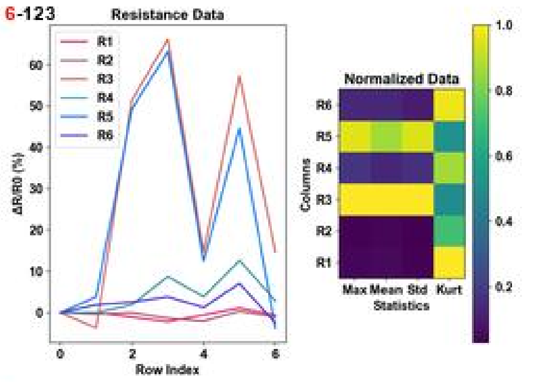

6-124
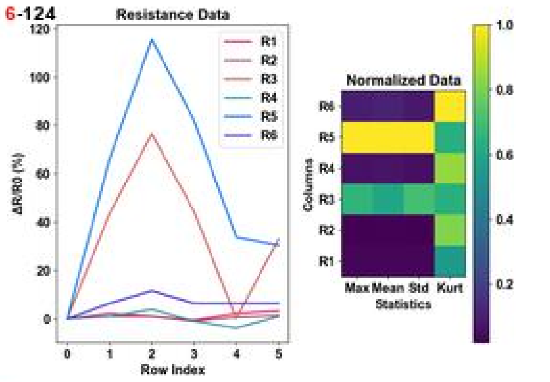

6-125
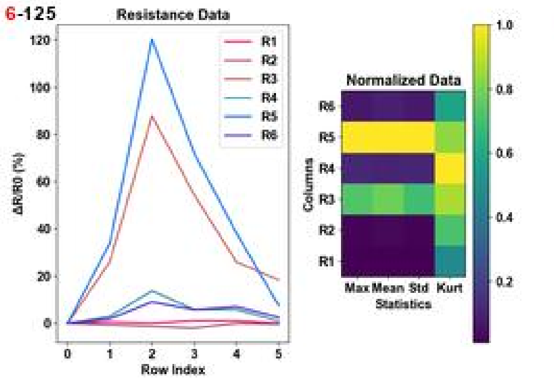

6-126
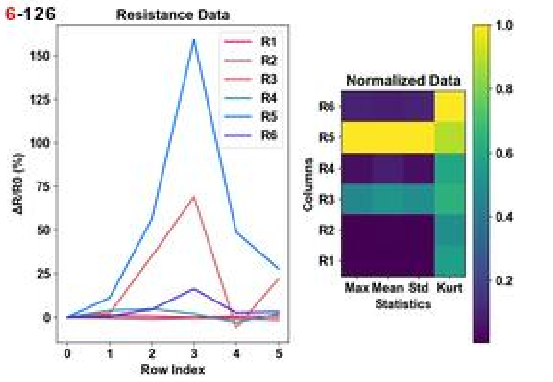

6-127
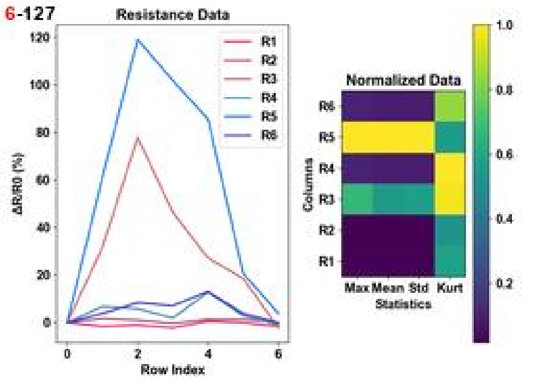

6-128
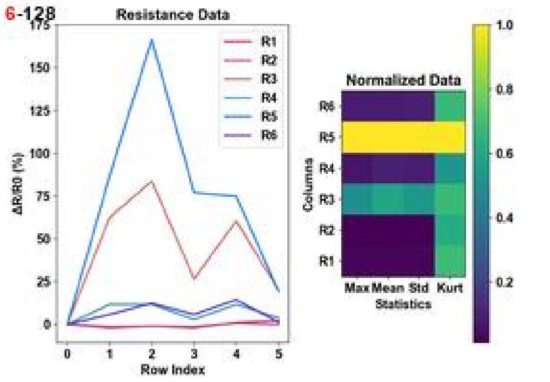

6-129
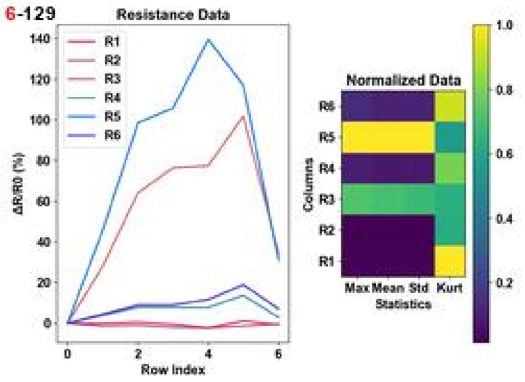

6-130
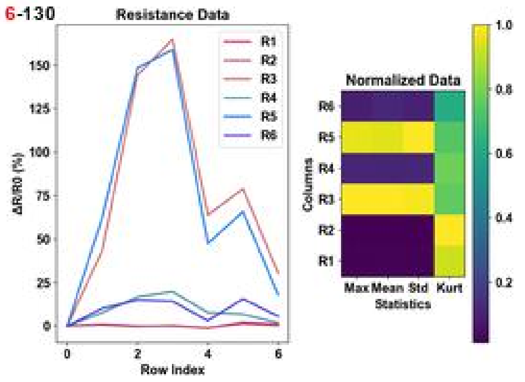

6-131
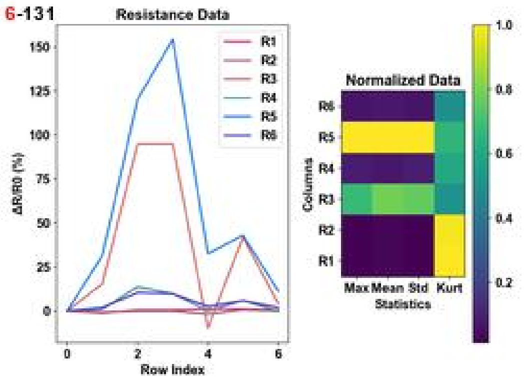

6-132
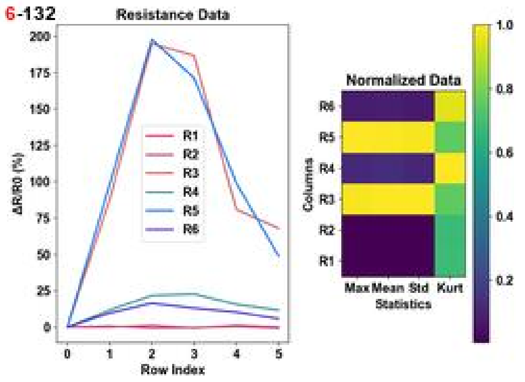

6-133
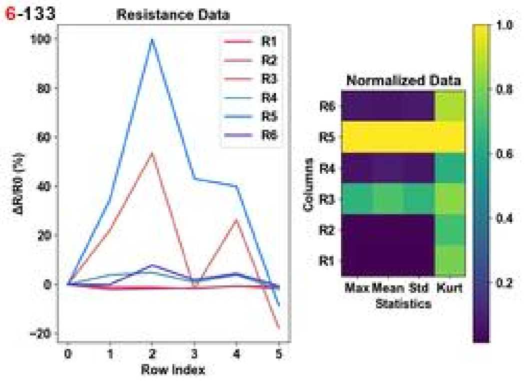

6-134
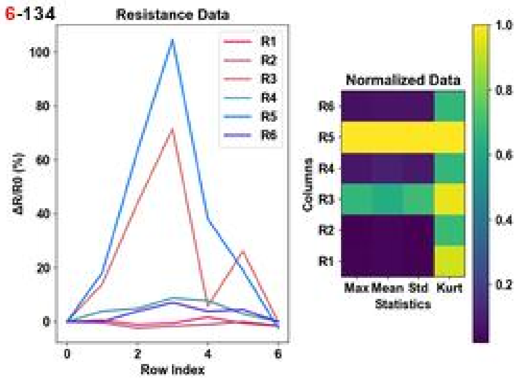

6-135
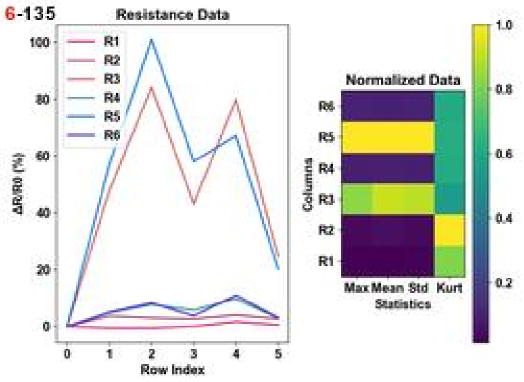

6-136
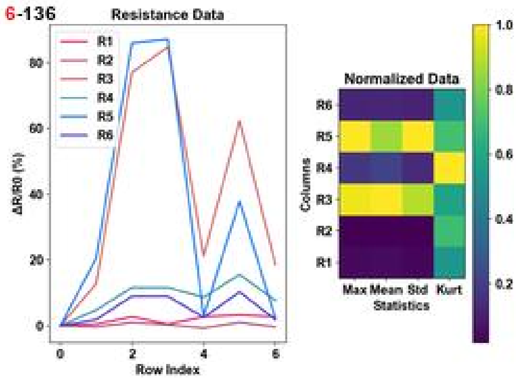

6-137
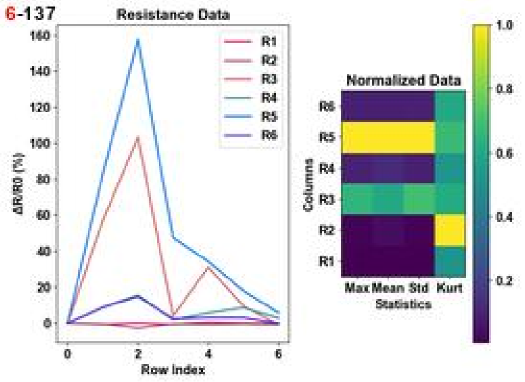

6-138
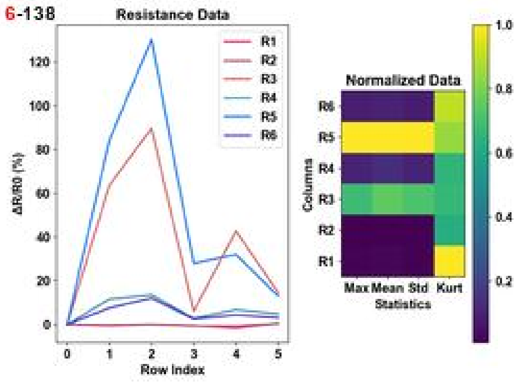

6-139
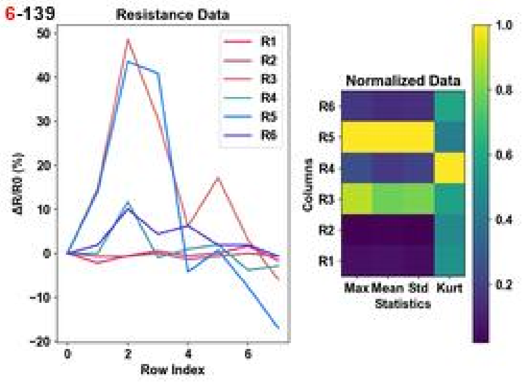

6-140
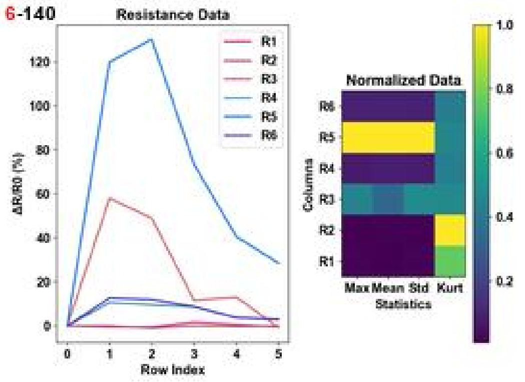

6-141
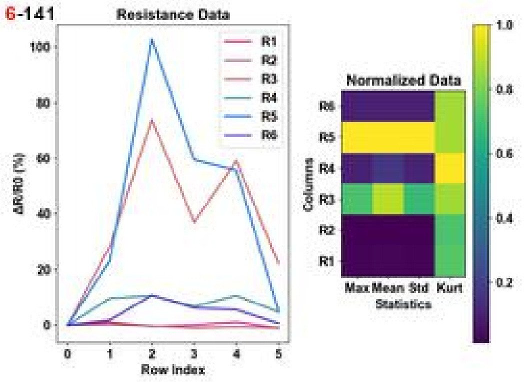

6-142
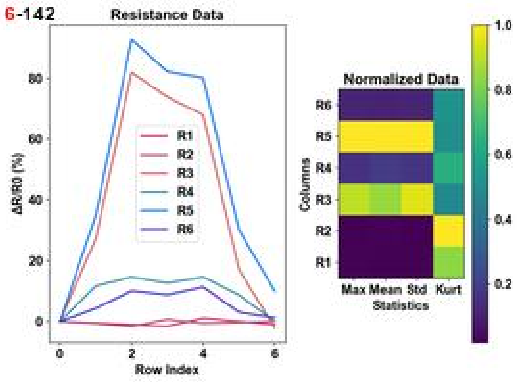

6-143
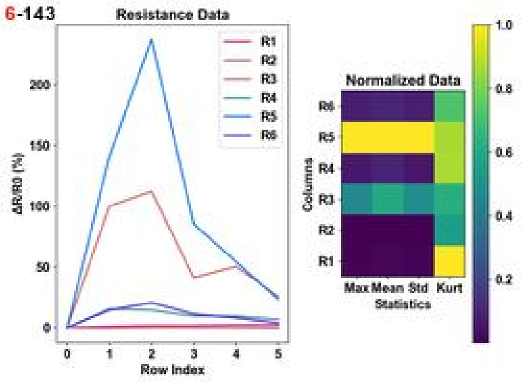

6-144
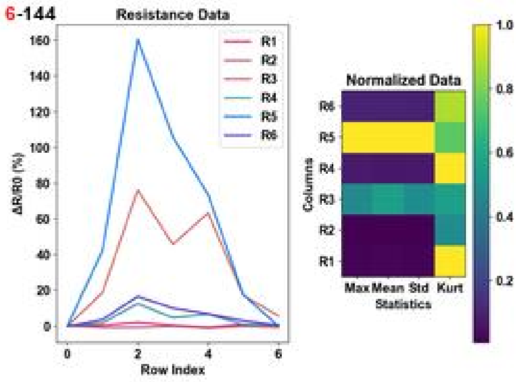

6-145
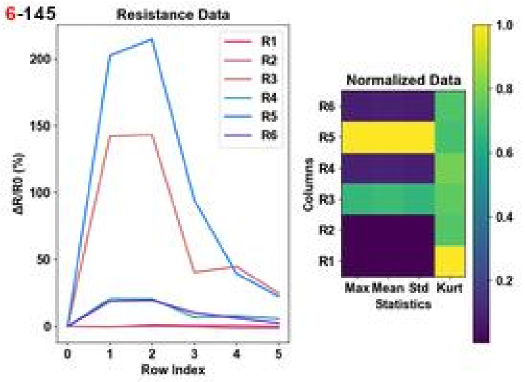

6-146
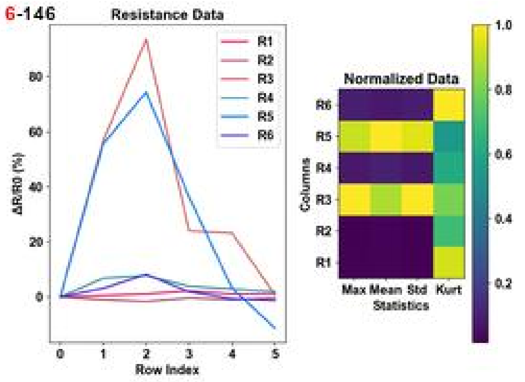

6-147
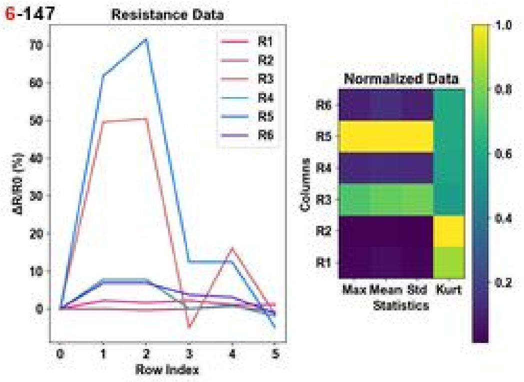

6-148
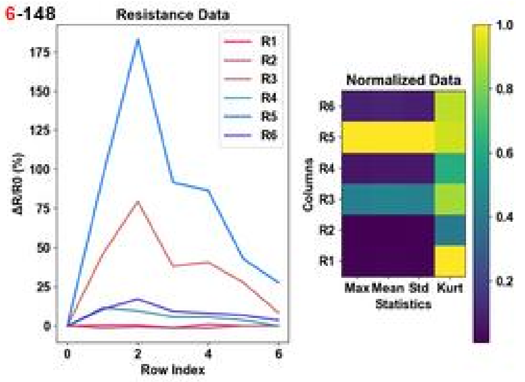

6-149
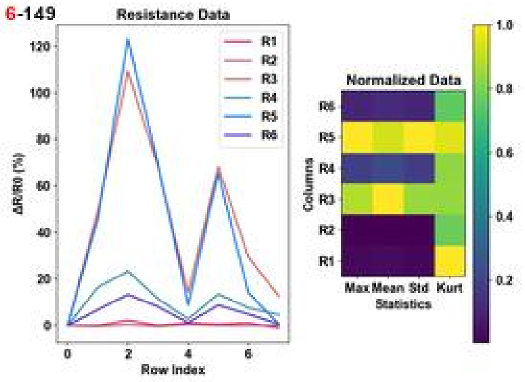

6-150
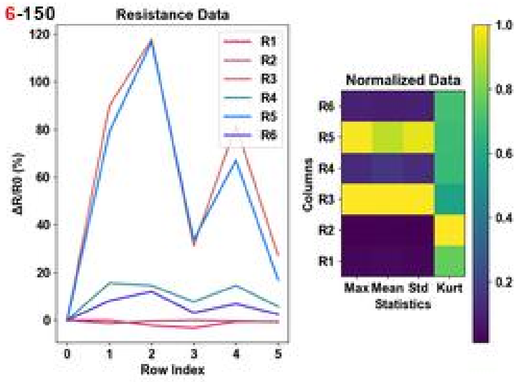

6-151
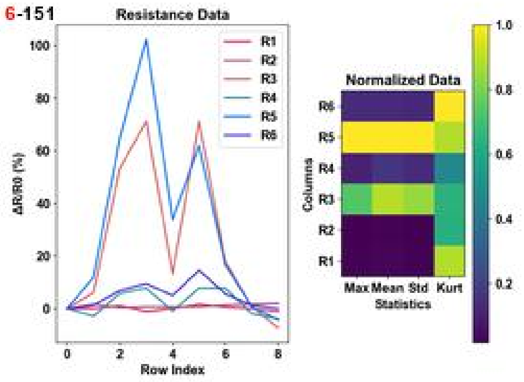

6-152
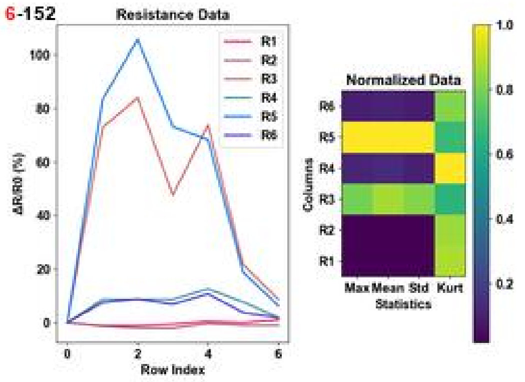

6-153
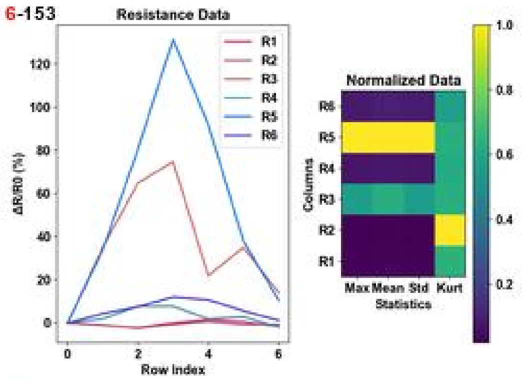

6-154
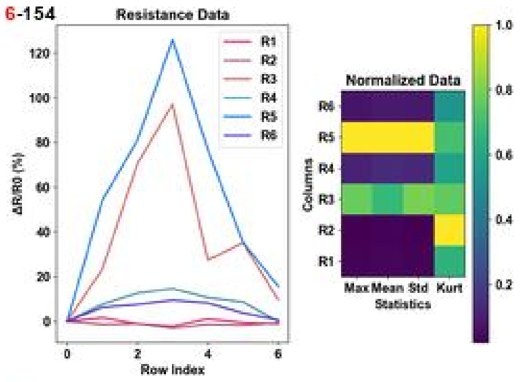

6-155
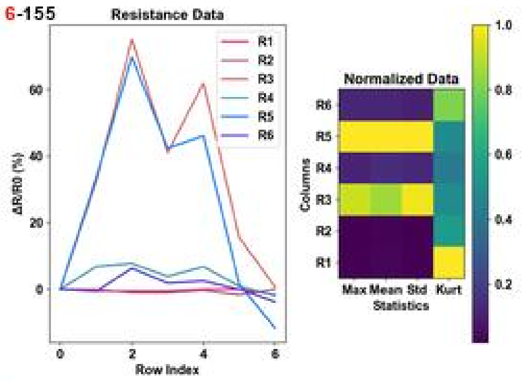

6-156
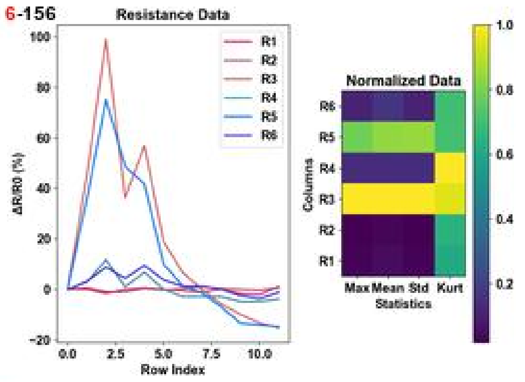

6-157
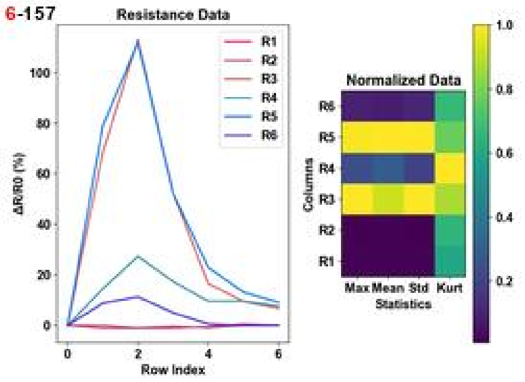

6-158
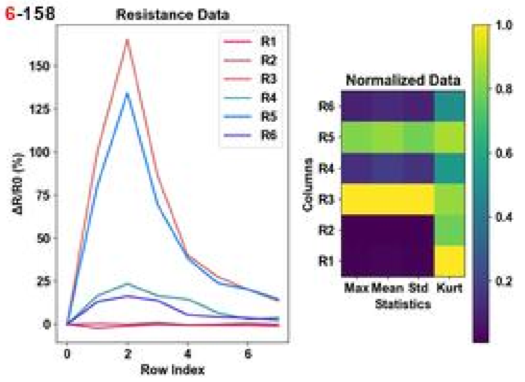

6-159
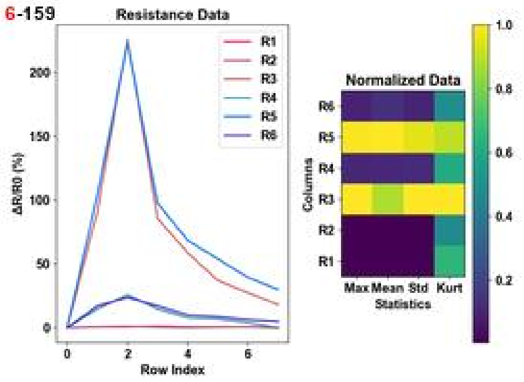

6-160
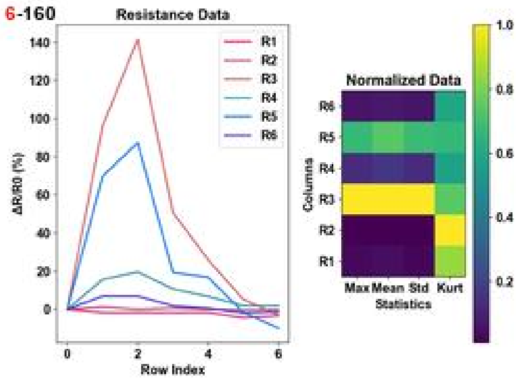

6-161
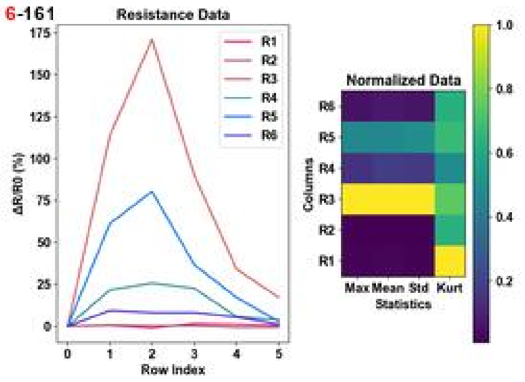

6-162
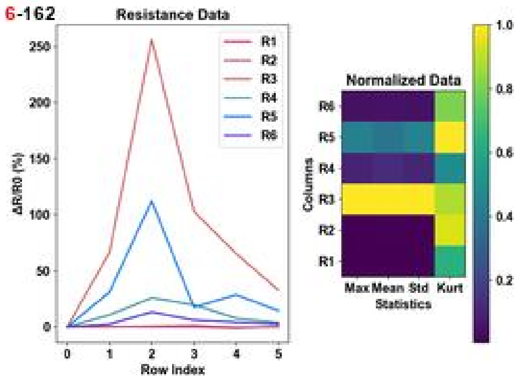

6-163
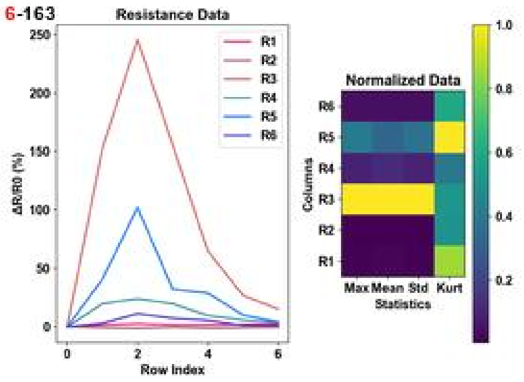

6-164
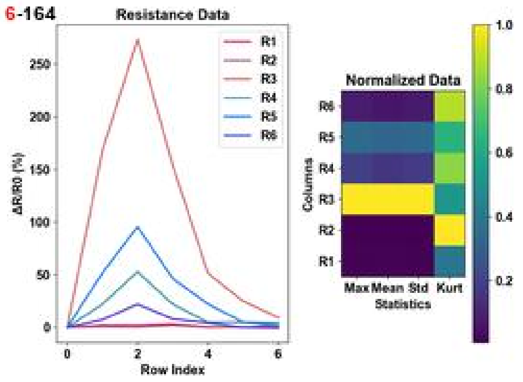

6-165
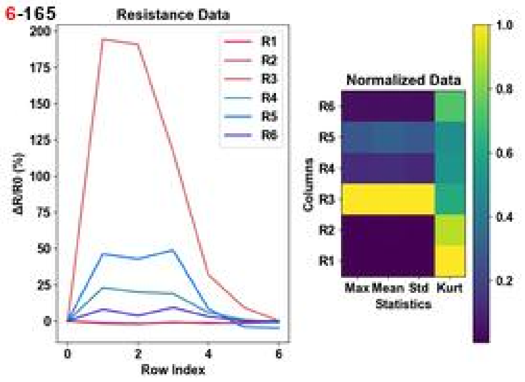

6-166
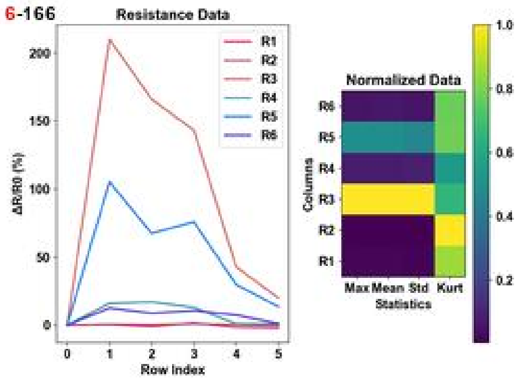

6-167
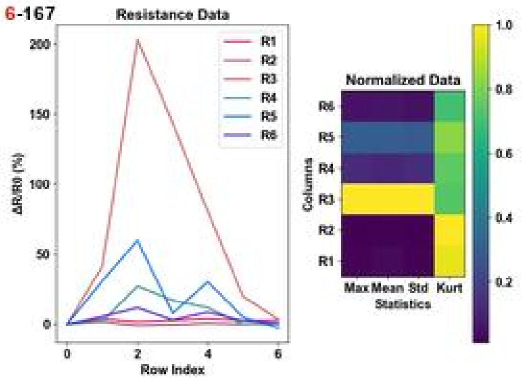

6-168
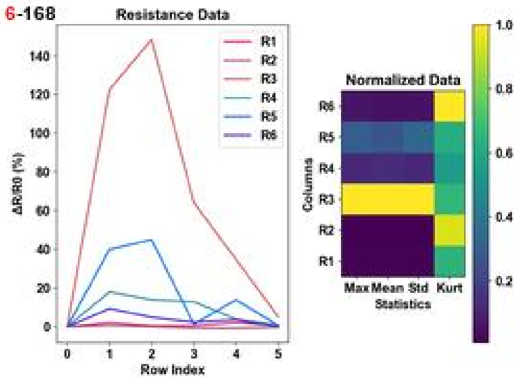

6-169
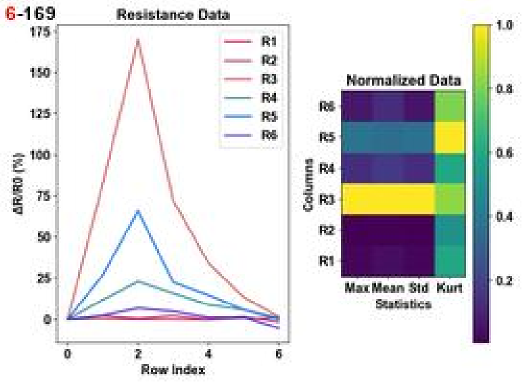

6-170
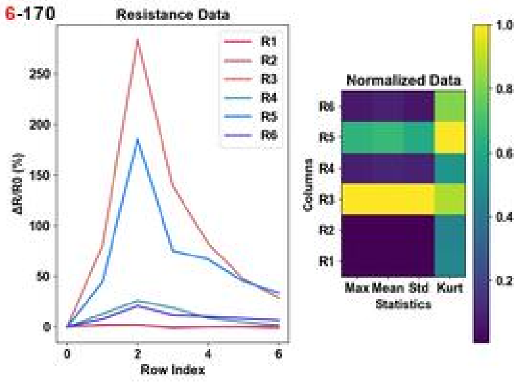

6-171
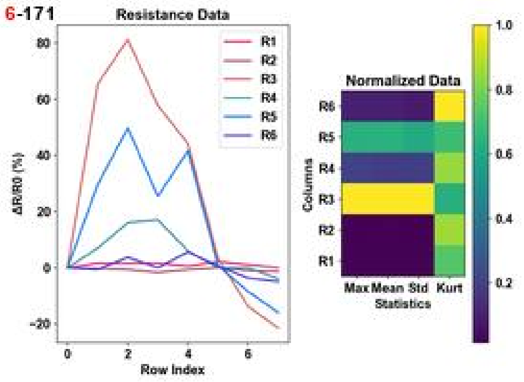

6-172
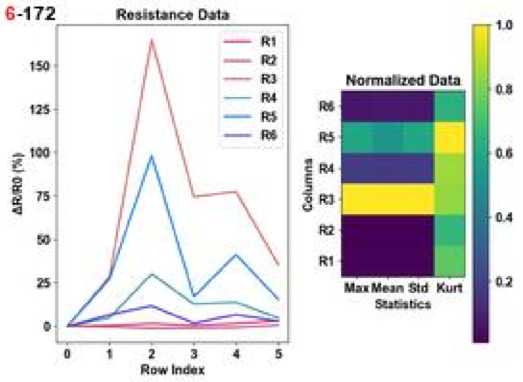

6-173
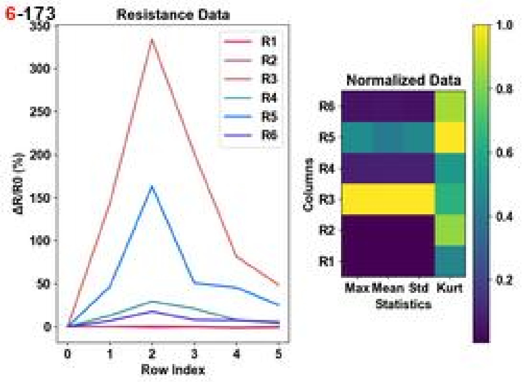

6-174
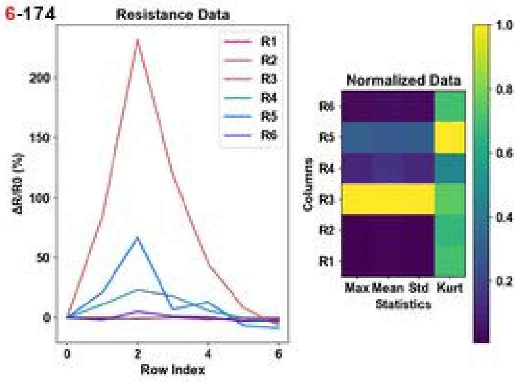

6-175
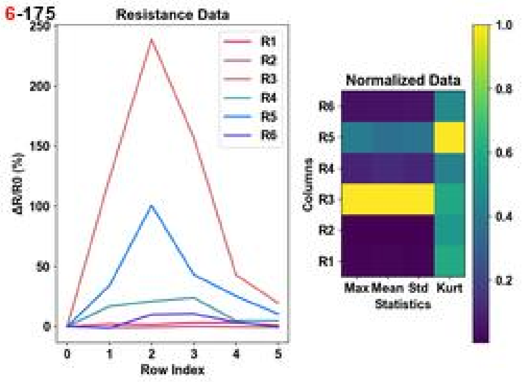

6-176
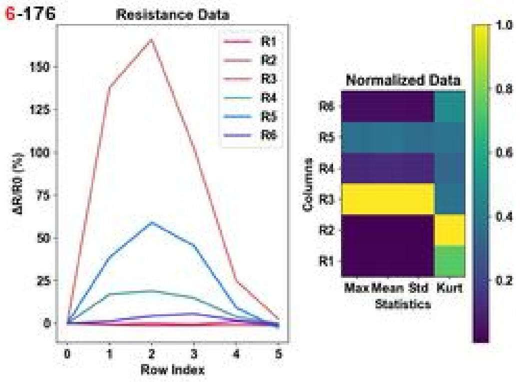

6-177
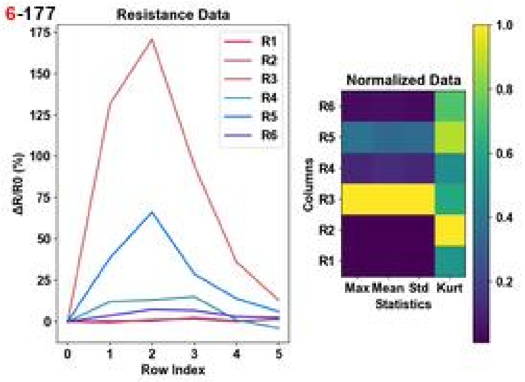

6-178
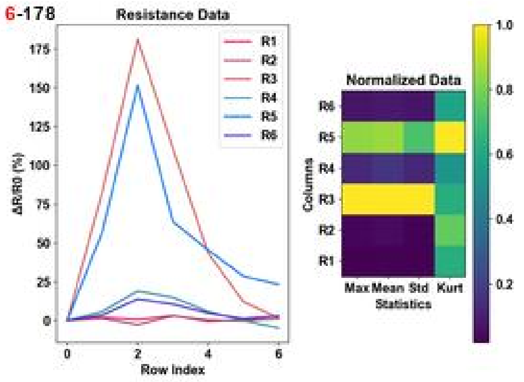

6-179
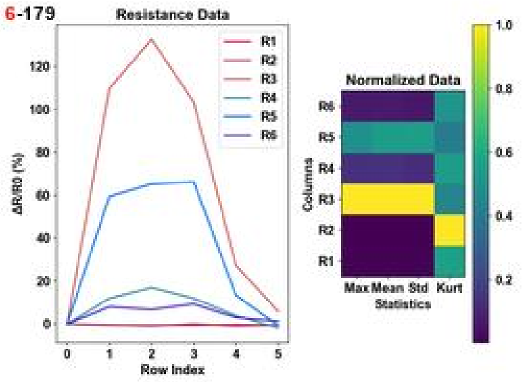

6-180
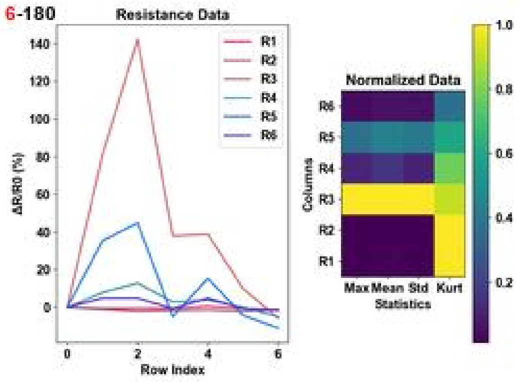

6-181
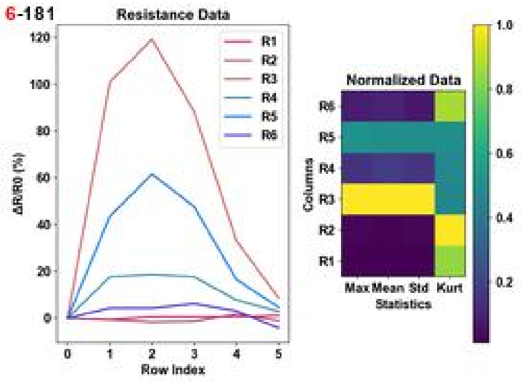

6-182
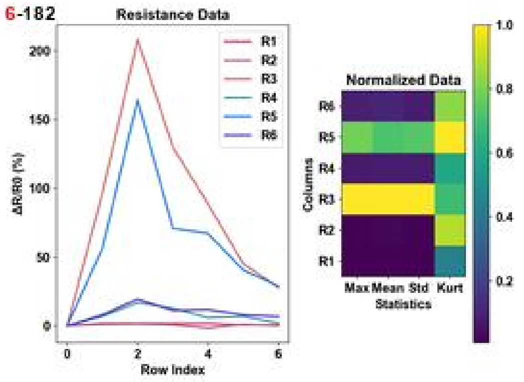

6-183
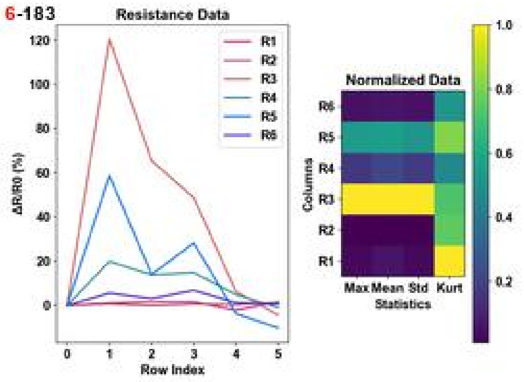

6-184
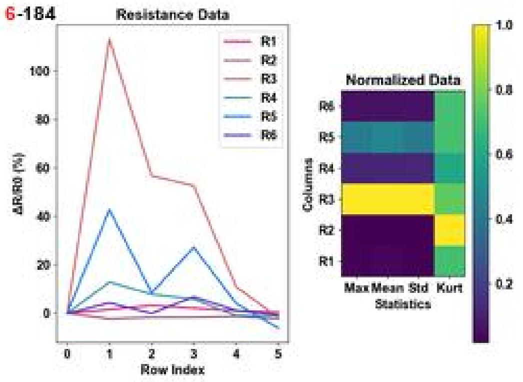

6-185
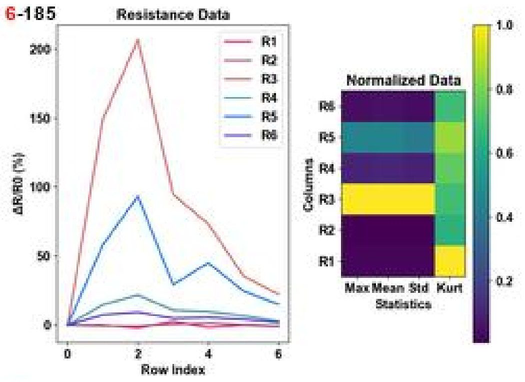

6-186
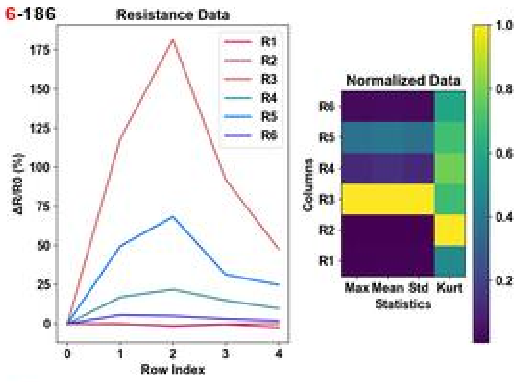

6-187
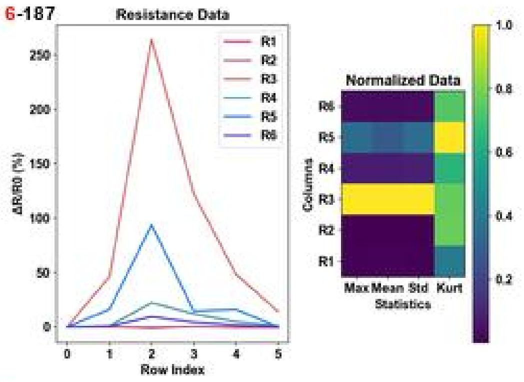

6-188
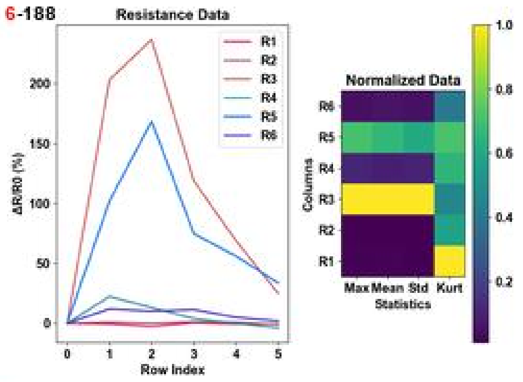

6-189
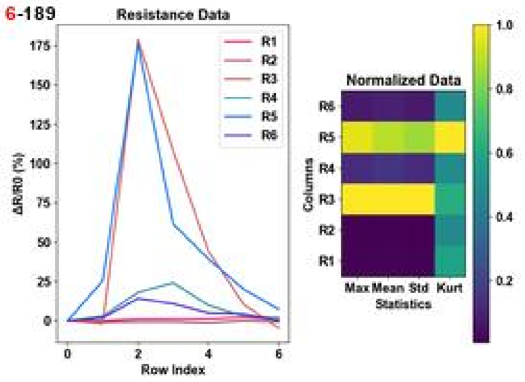

6-190
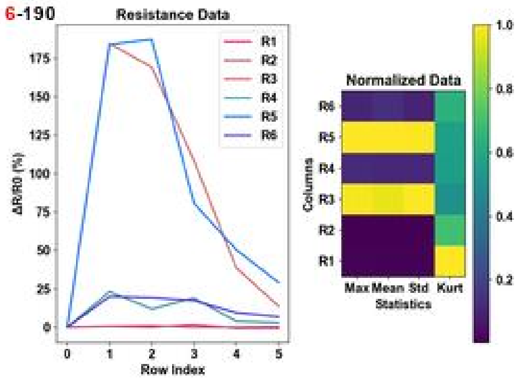

6-191
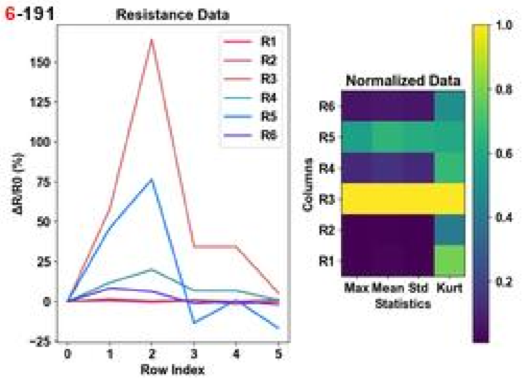

6-192
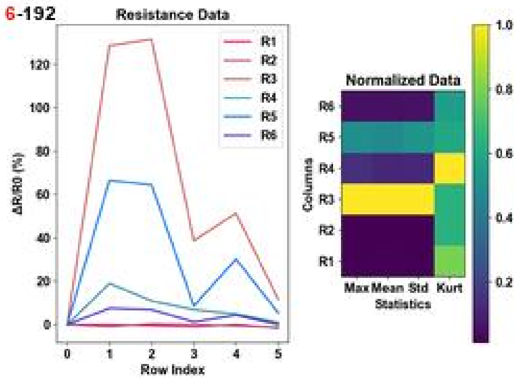

6-193
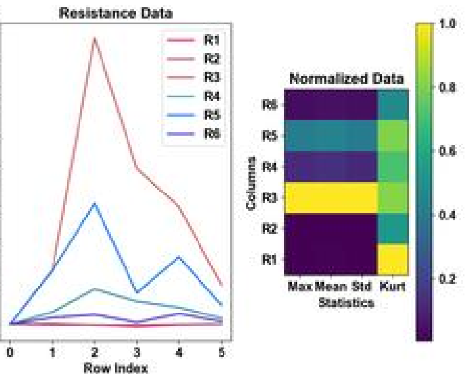

6-194
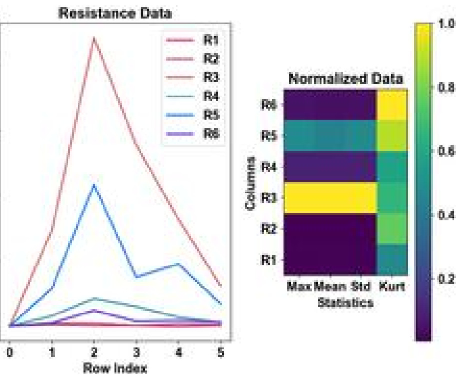

6-195
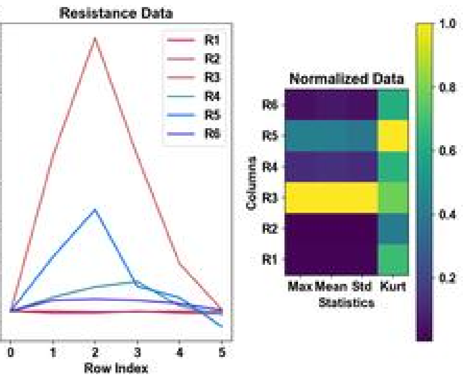

6-196
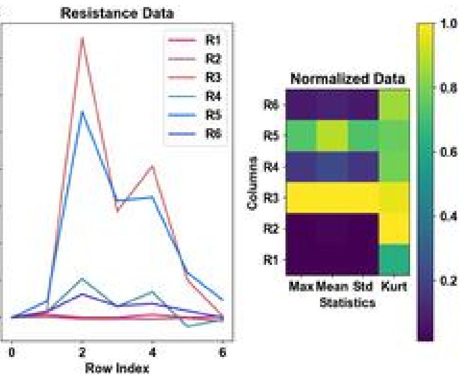

6-197
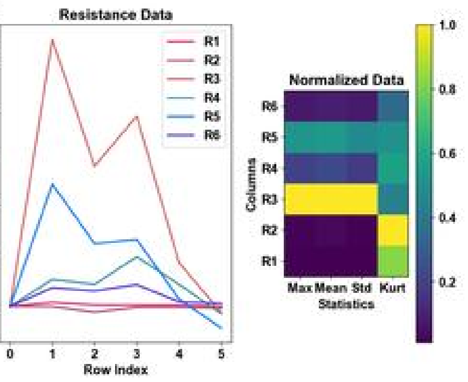

6-198
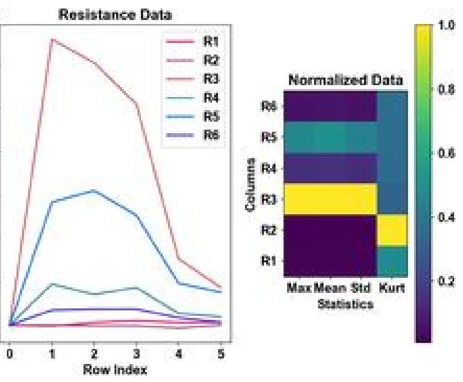

6-199
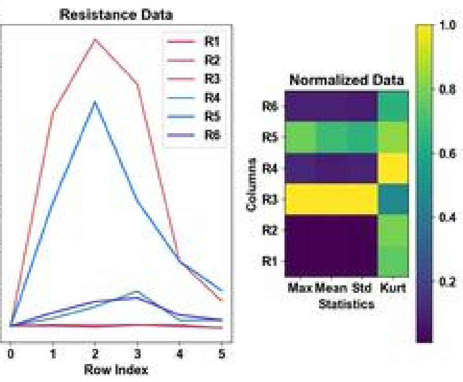

6-200
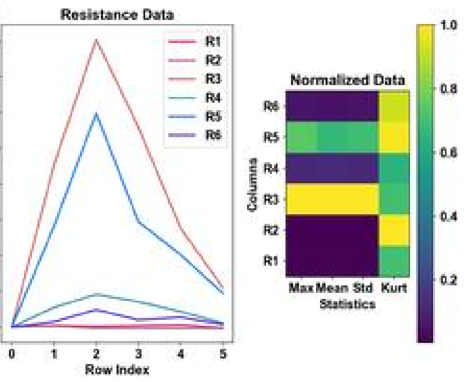

6-201
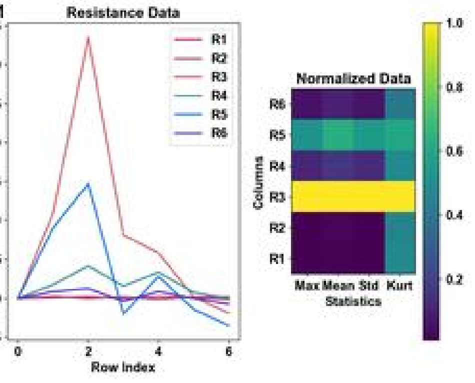

6-202
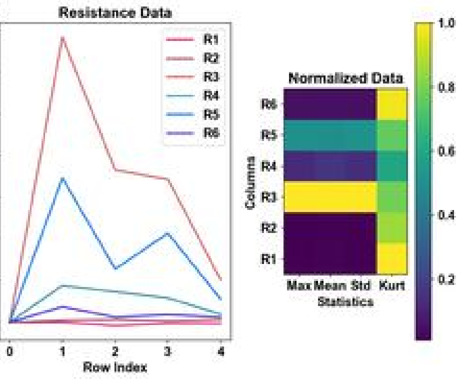

6-203
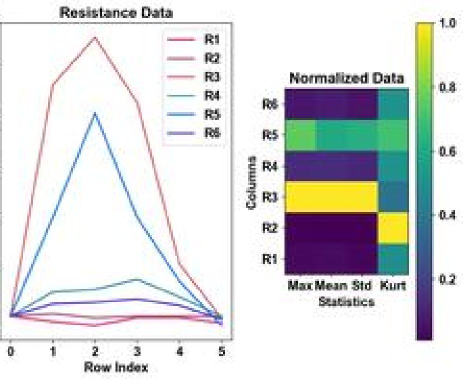

6-204
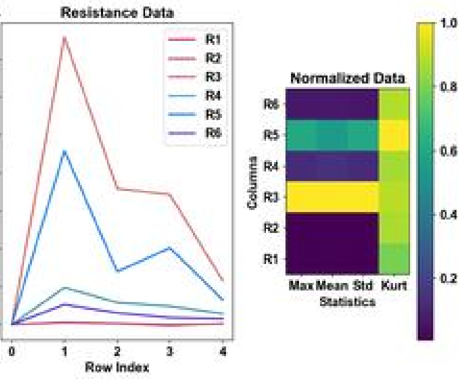

6-205
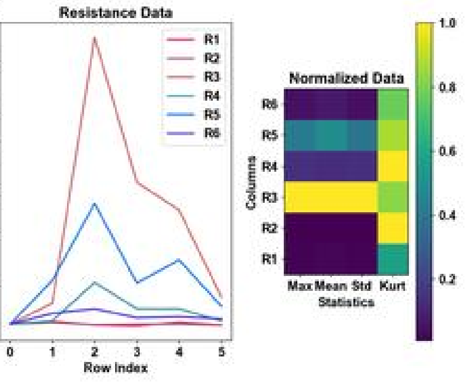

6-206
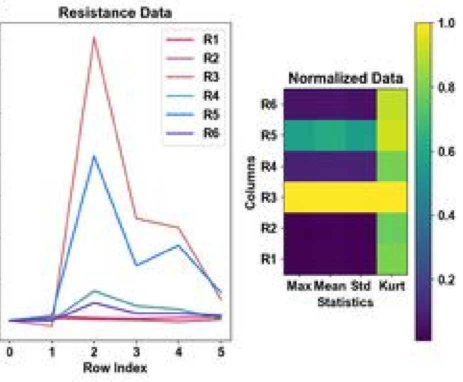

6-207
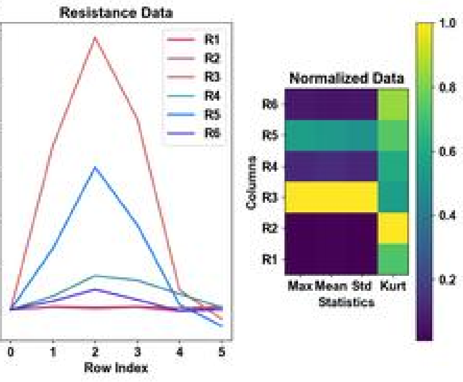

6-208
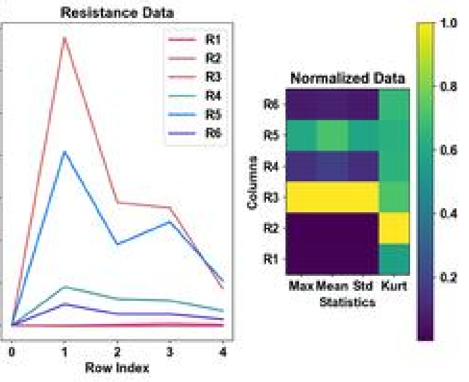

6-209
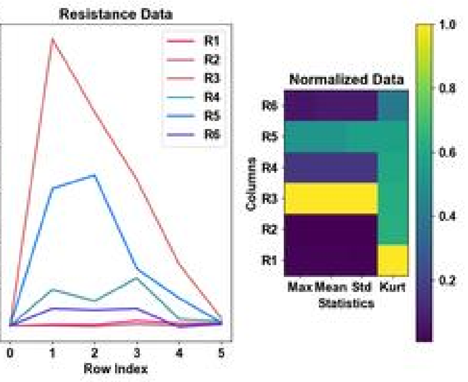

6-210
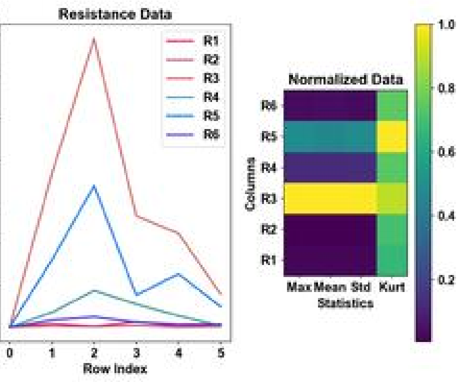

6-211
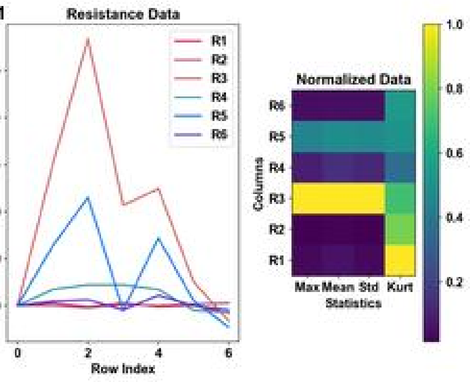

6-212
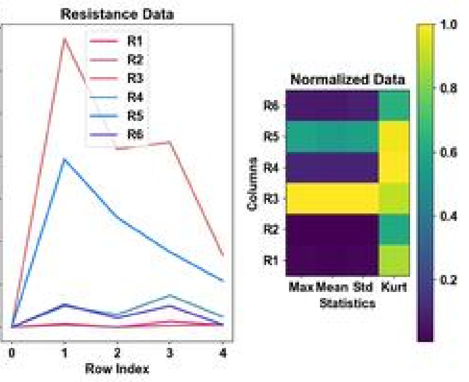

6-213
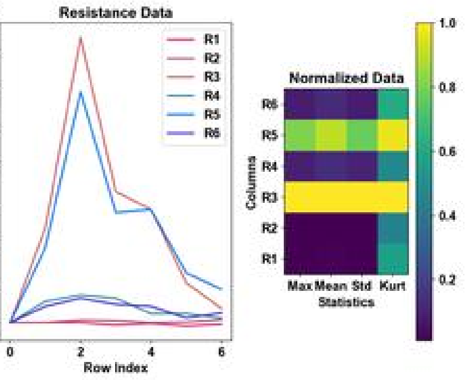

6-214
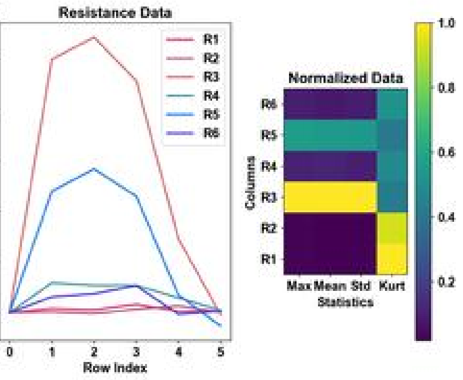

6-215
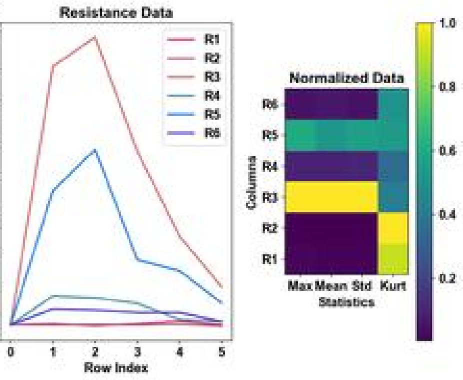

6-216
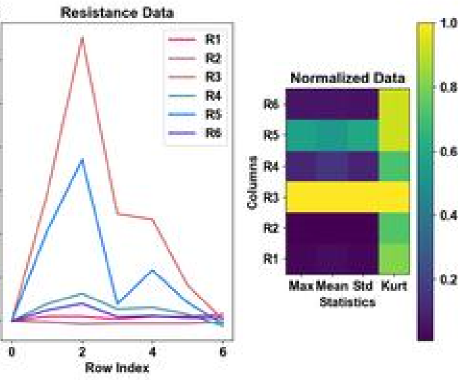

6-217
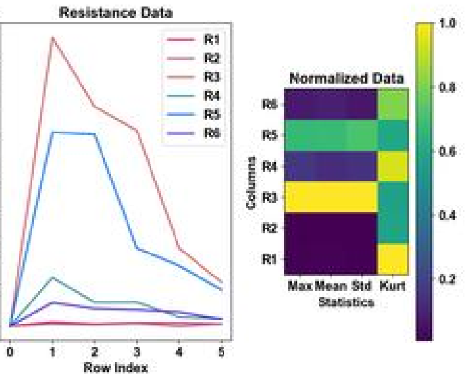

6-218
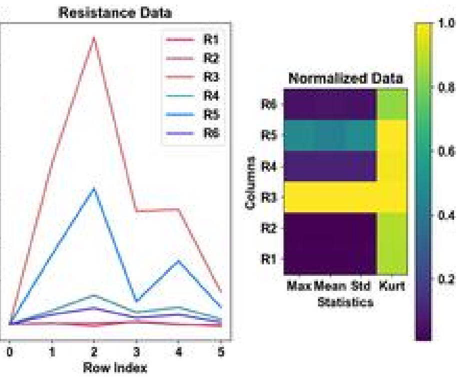

6-219
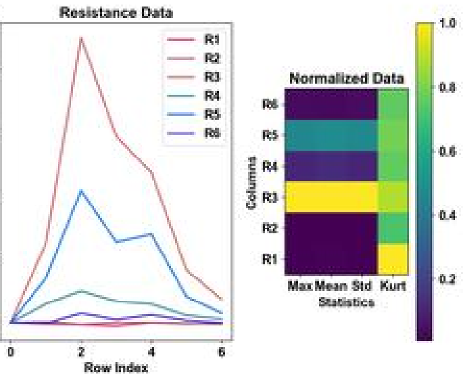

6-220
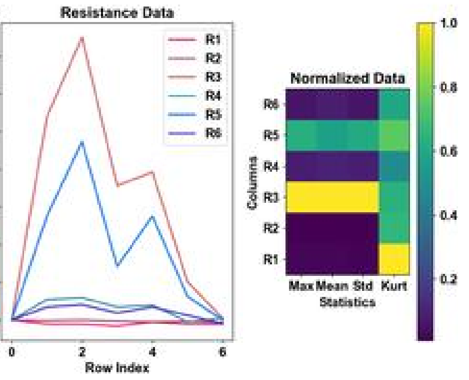

6-221
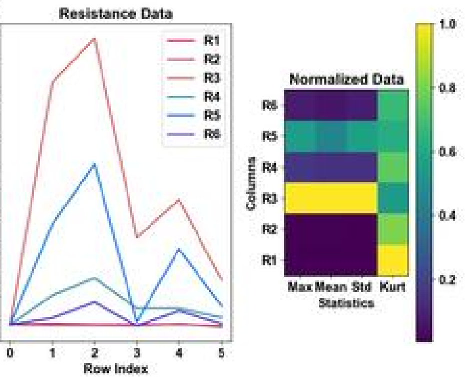

6-222
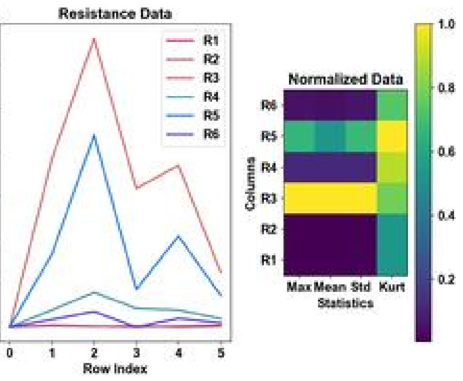

6-223
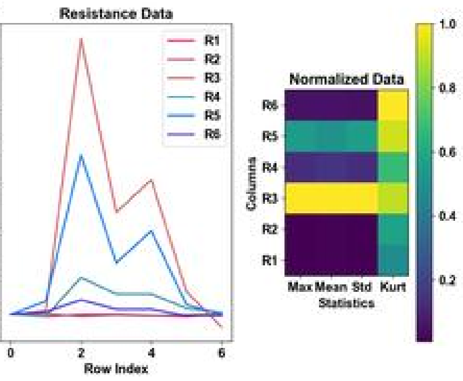

6-224
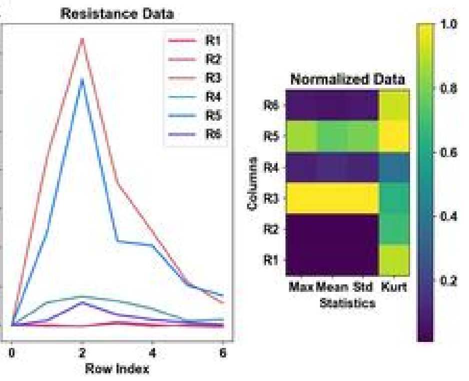

6-225
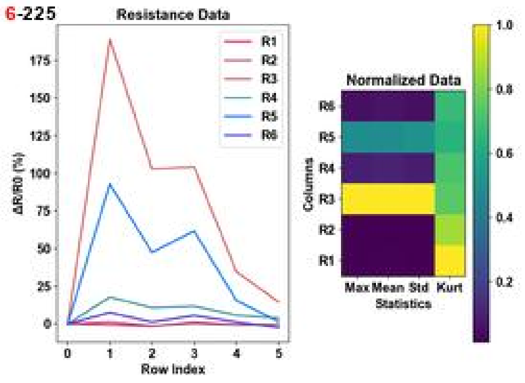

6-226
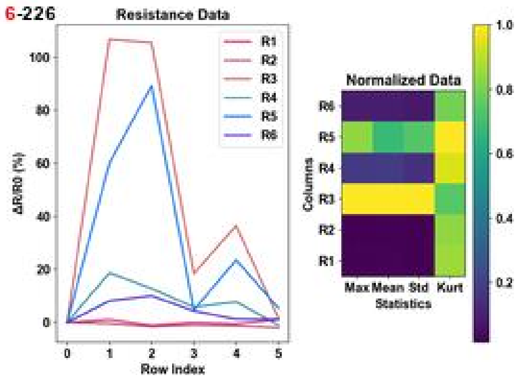

6-227
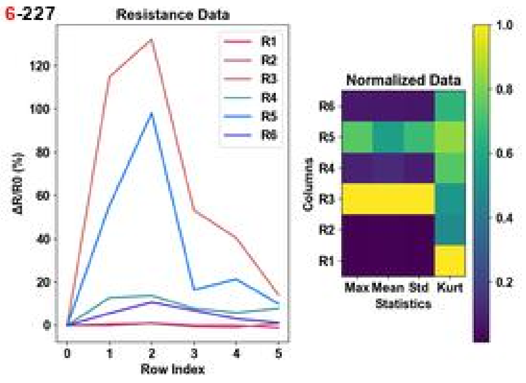

6-228
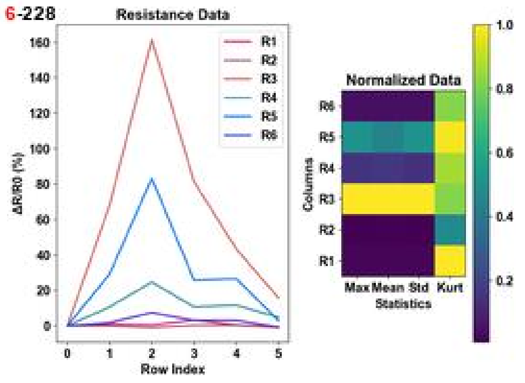

6-229
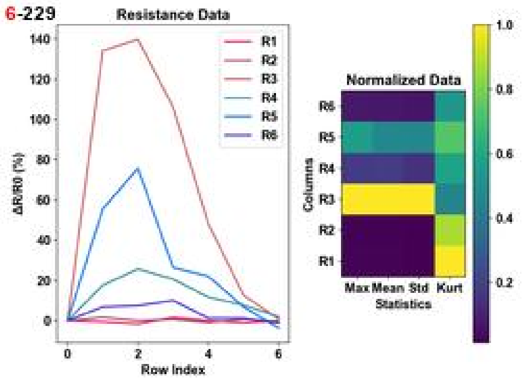

6-230
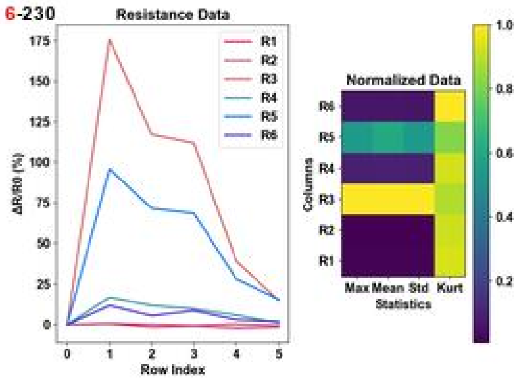

6-231
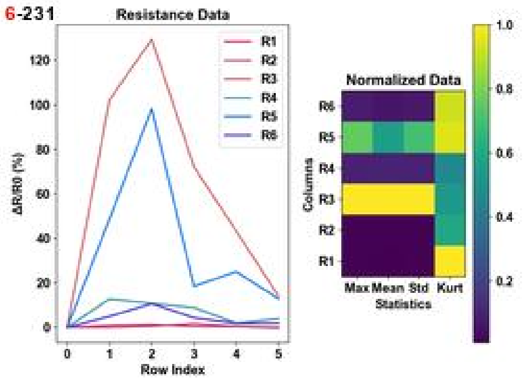

6-232
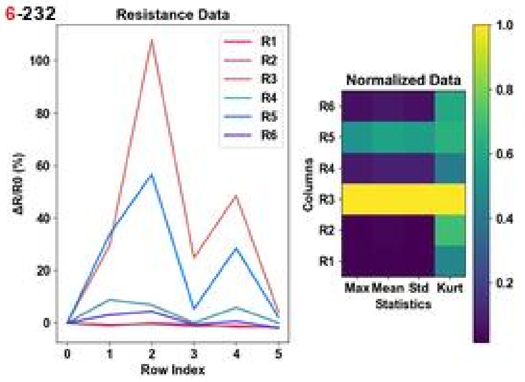

6-233
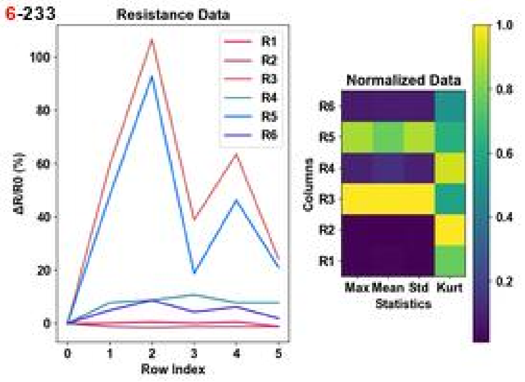

6-234
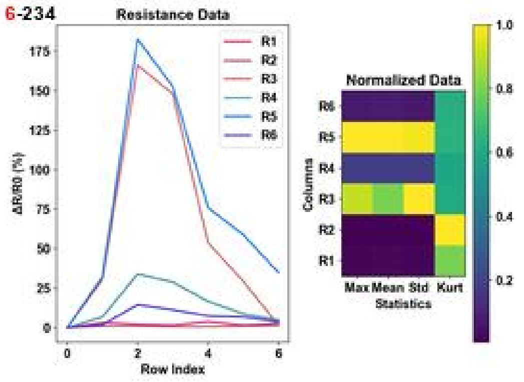

6-235
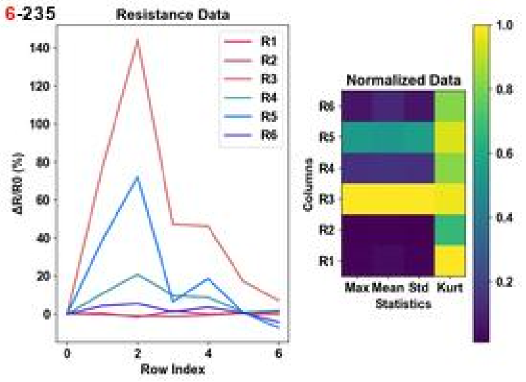

6-236
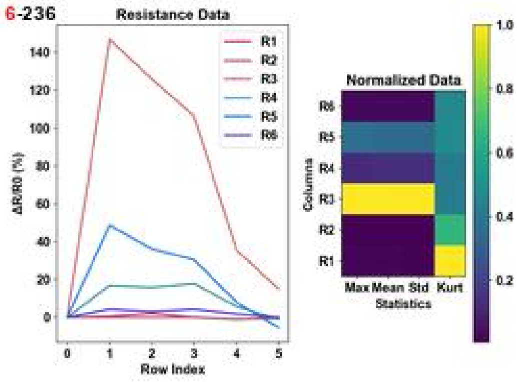

6-237
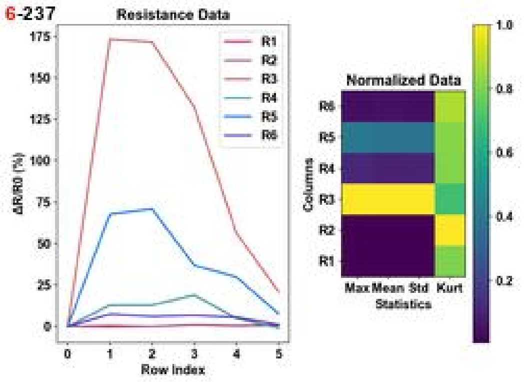

6-238
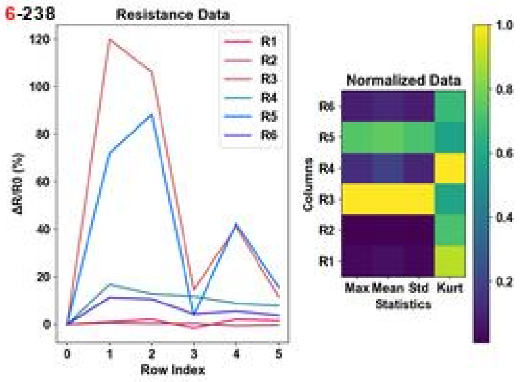

6-239
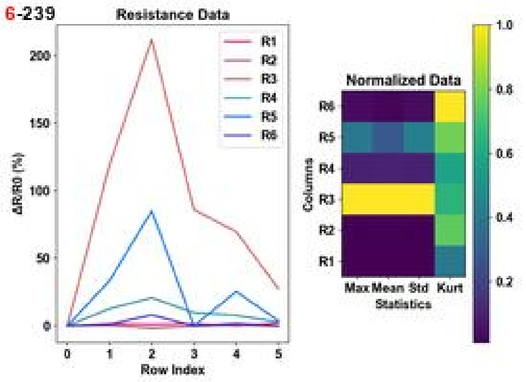

6-240
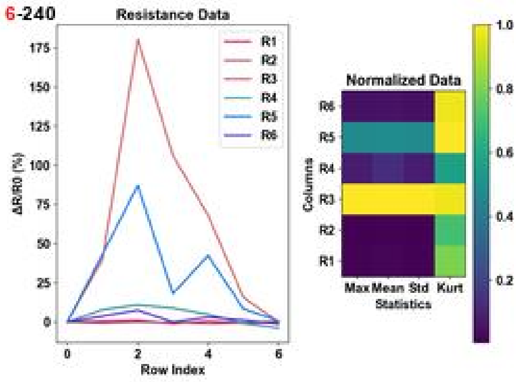

Pressure-sensitive data of type 7

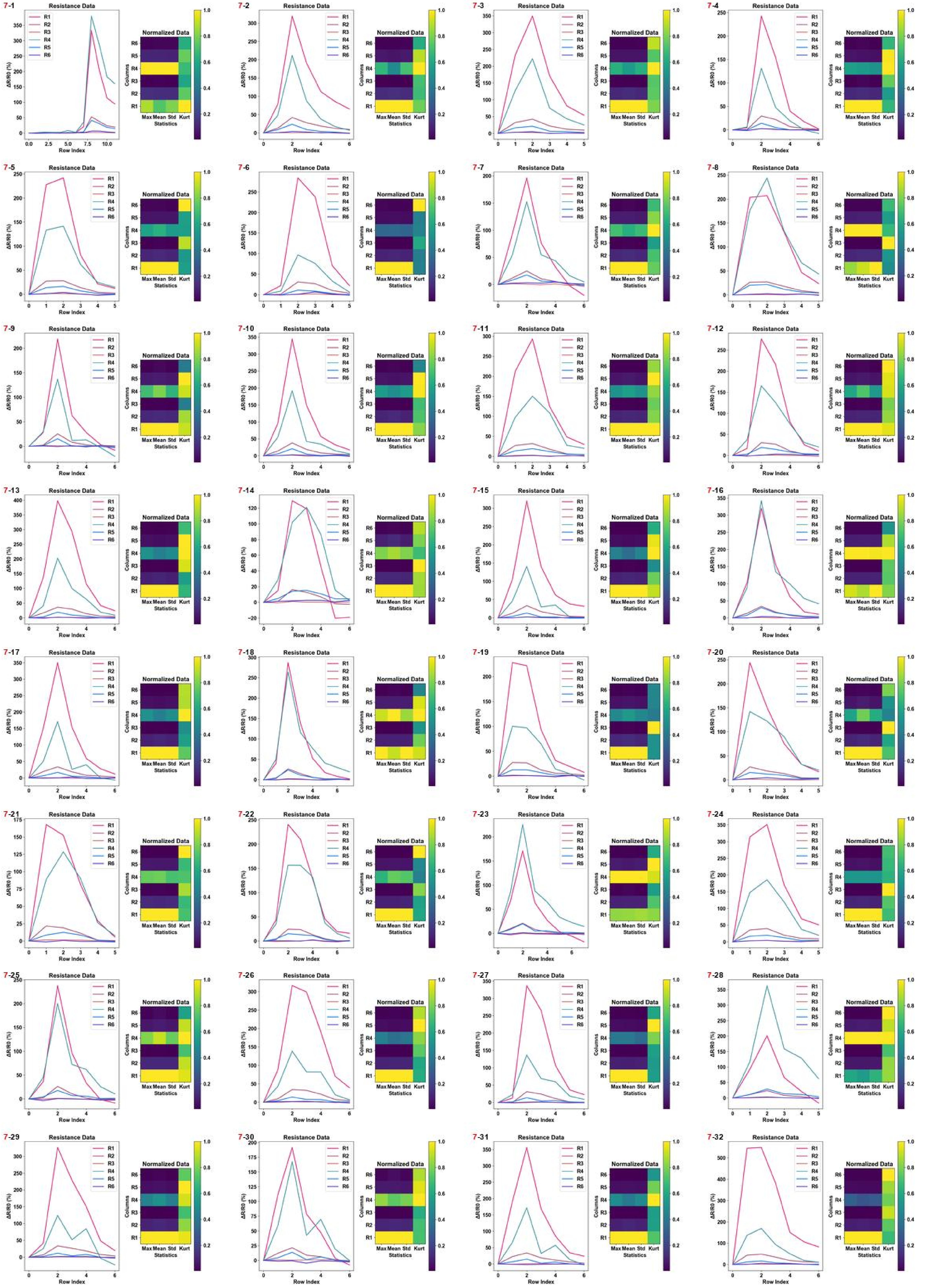

7-33
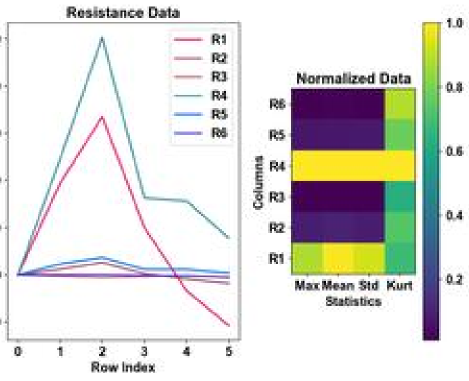

7-34
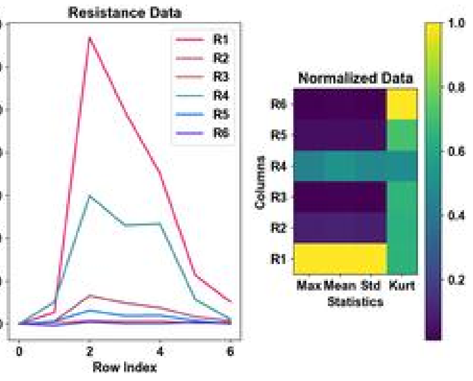

7-35
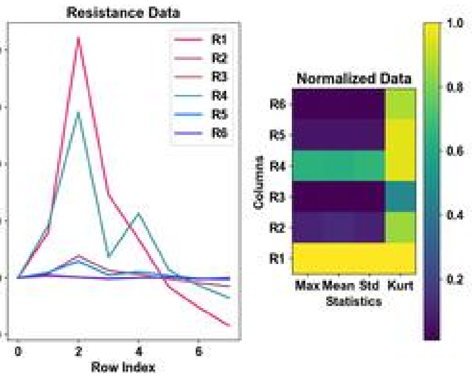

7-36
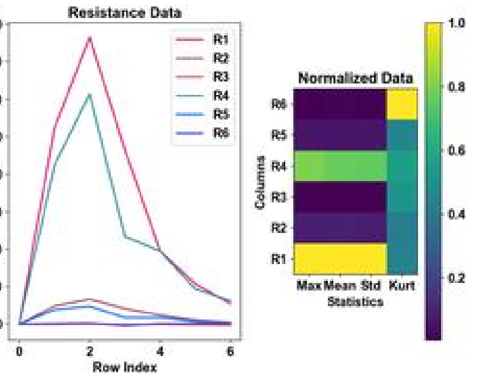

7-37
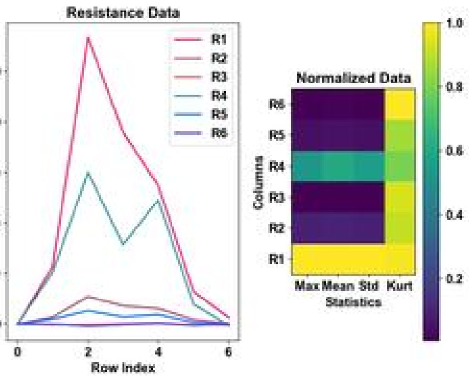

7-38
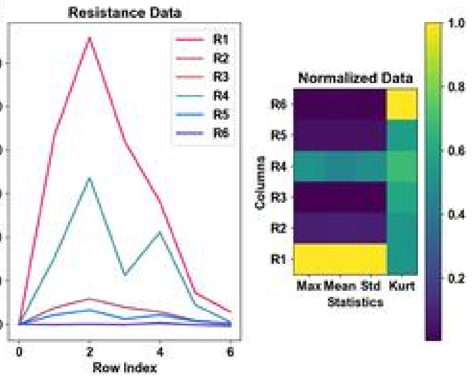

7-39
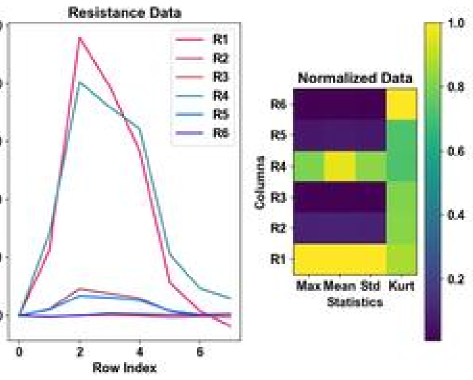

7-40
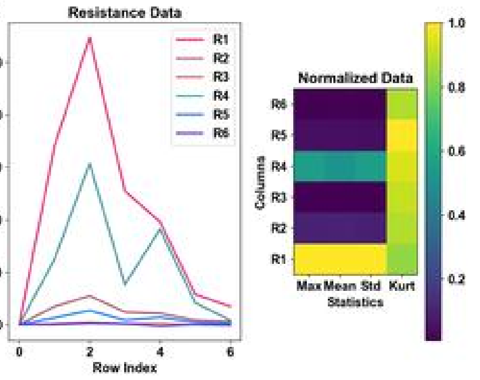

7-41
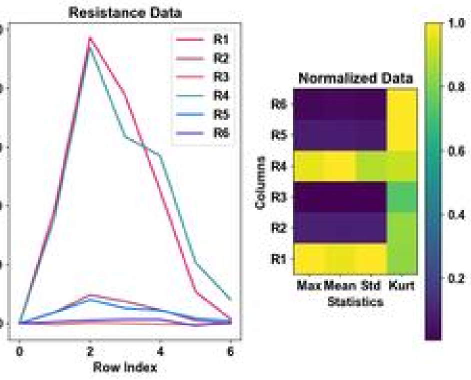

7-42
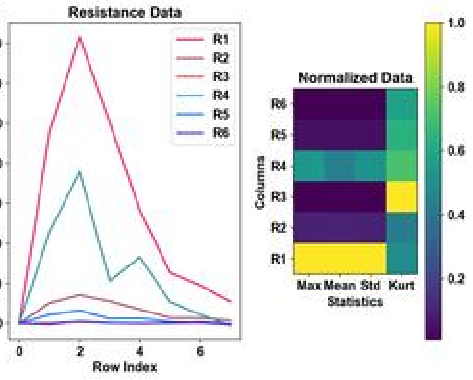

7-43
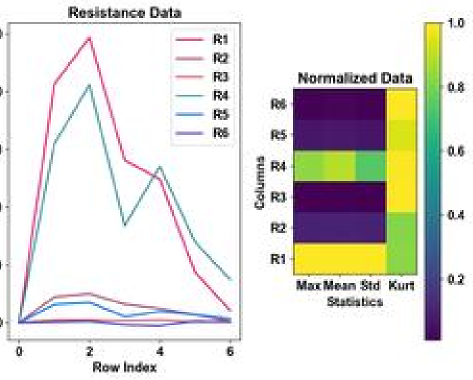

7-44
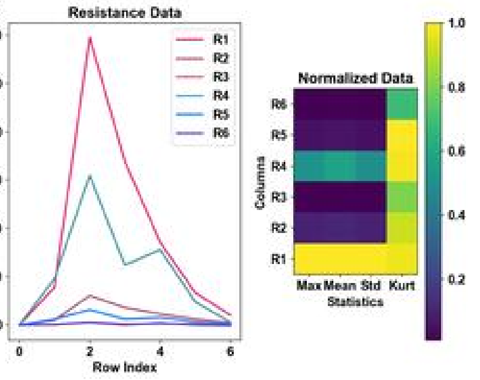

7-45
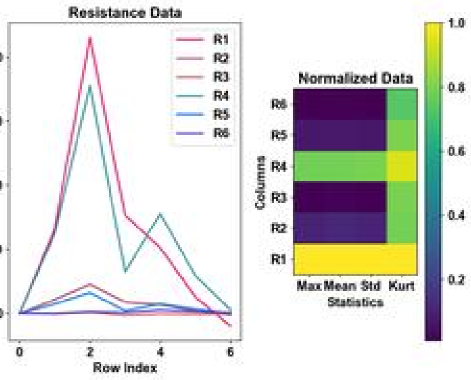

7-46
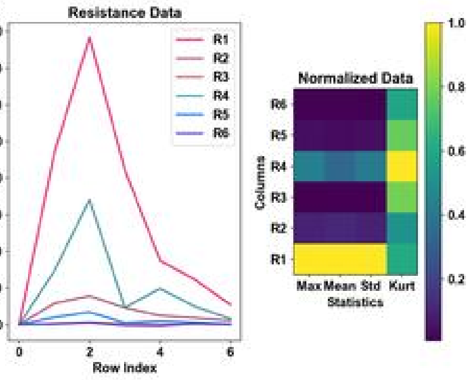

7-47
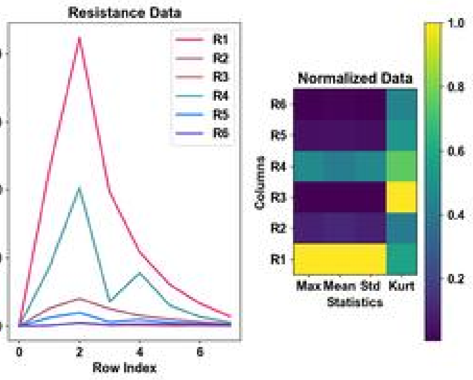

7-48
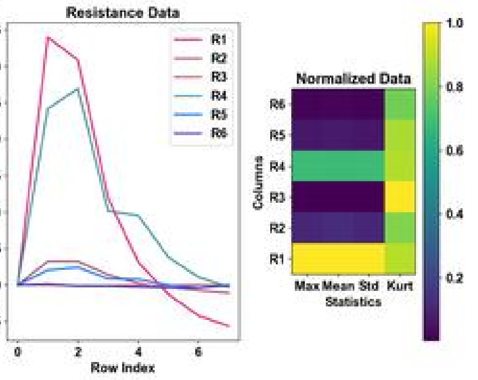

7-49
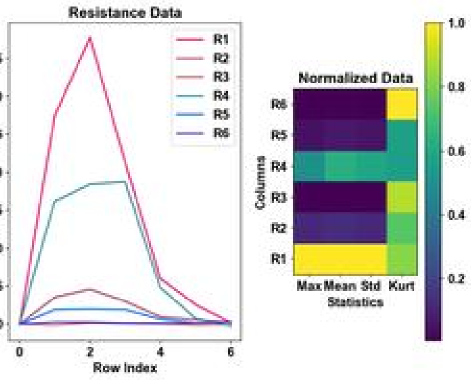

7-50
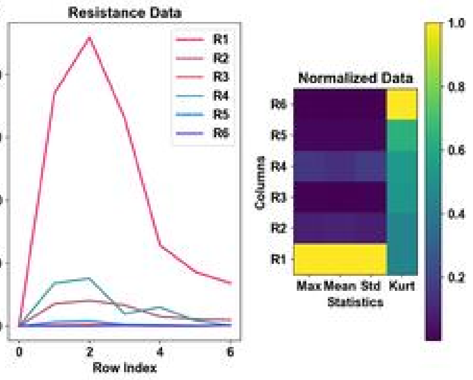

7-51
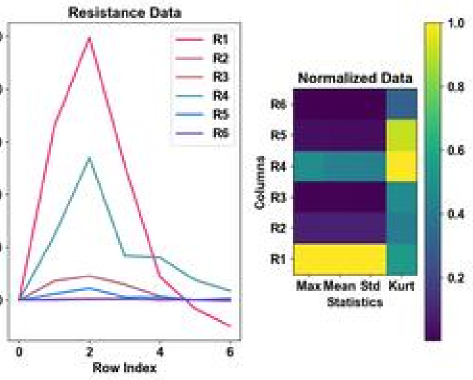

7-52
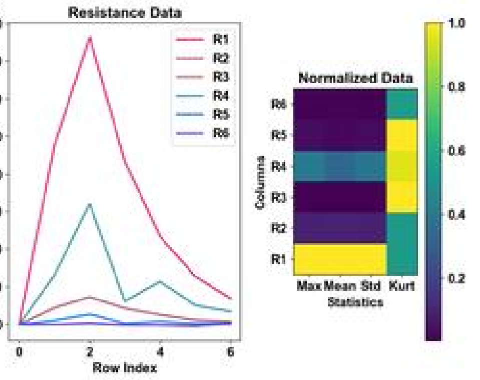

7-53
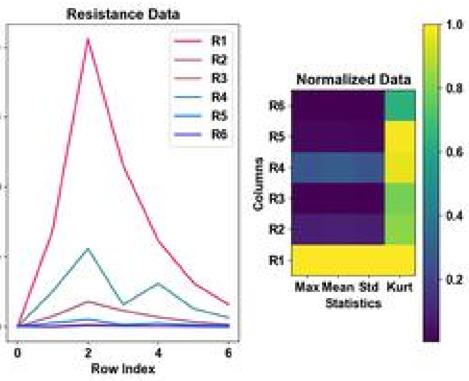

7-54
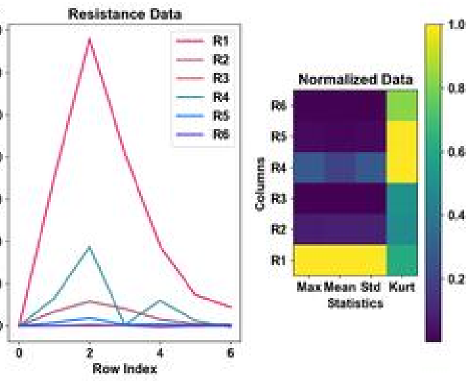

7-55
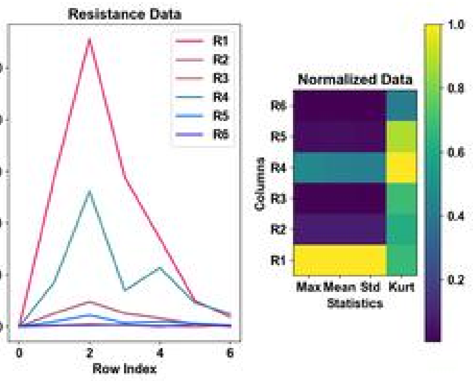

7-56
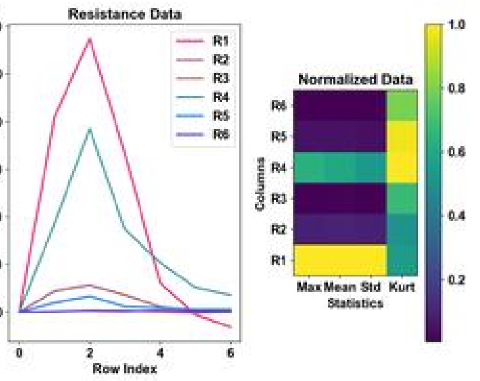

7-57
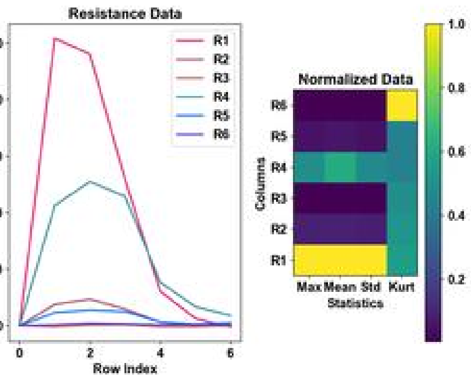

7-58
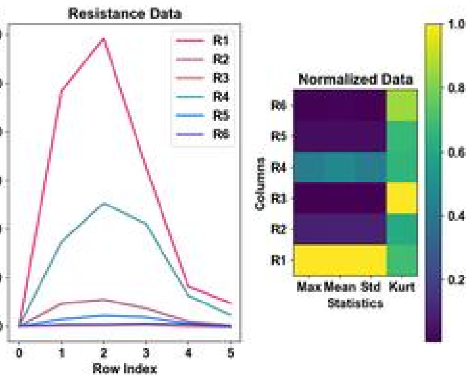

7-59
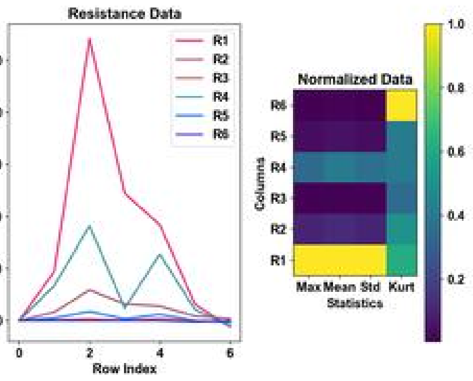

7-60
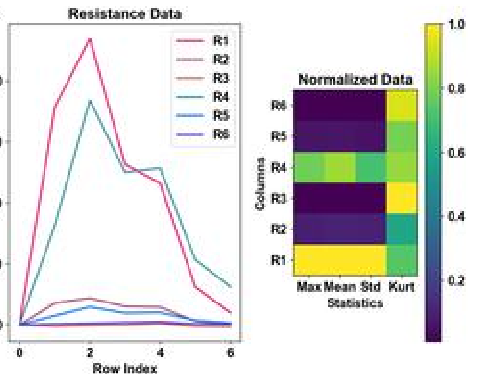

7-61
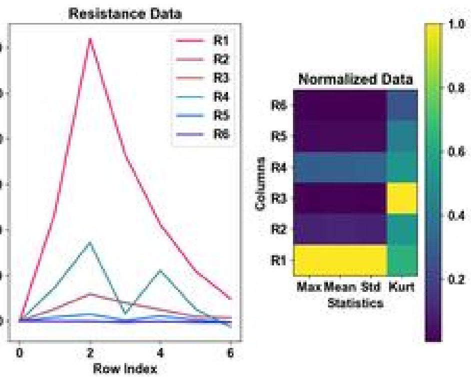

7-62
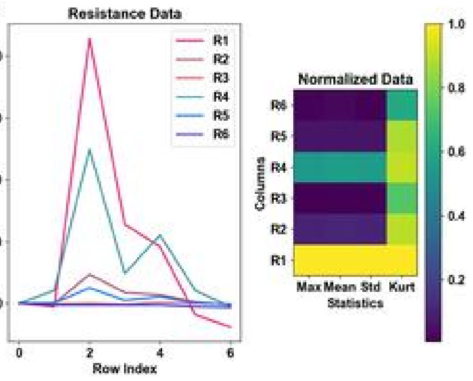

7-63
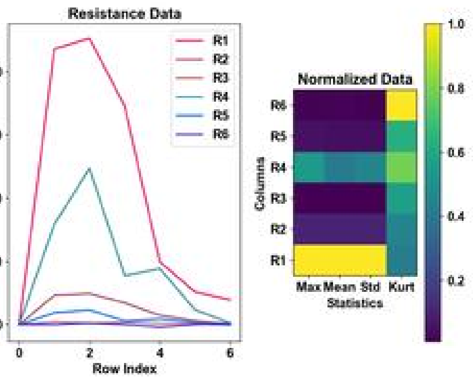

7-64
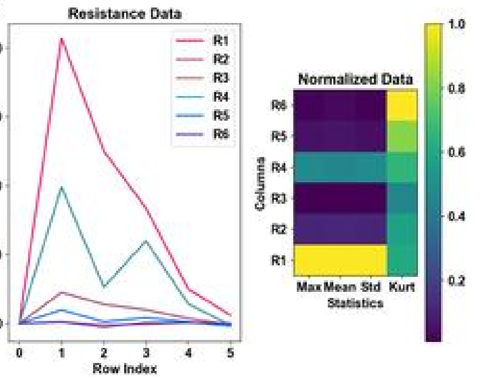

Pressure-sensitive data of type 7

7-65
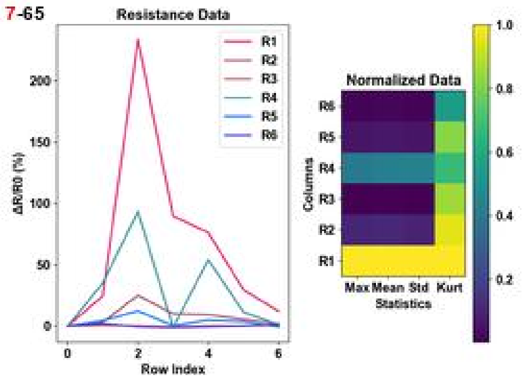

7-66
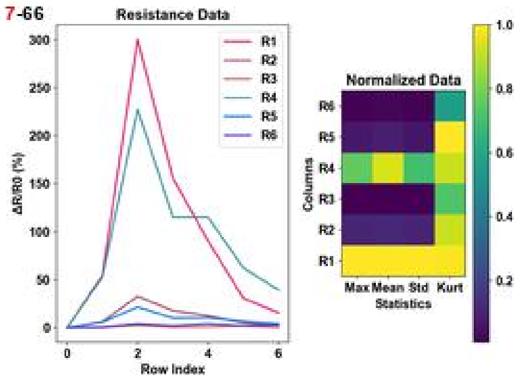

7-67
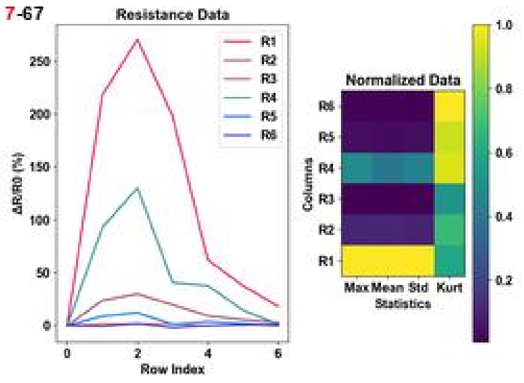

7-68
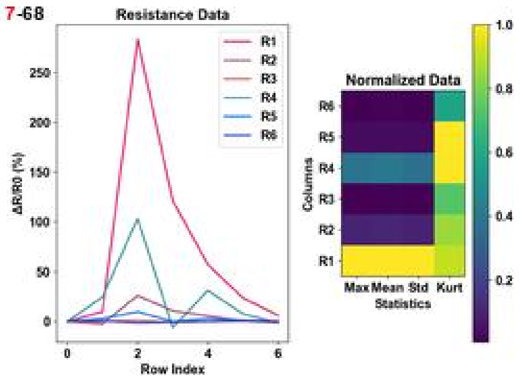

7-69
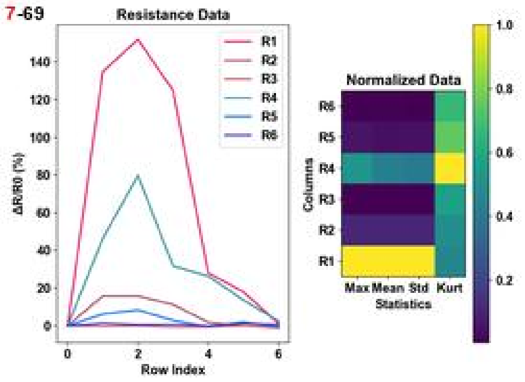

7-70
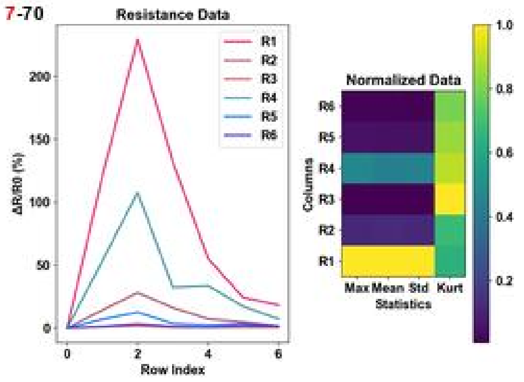

7-71
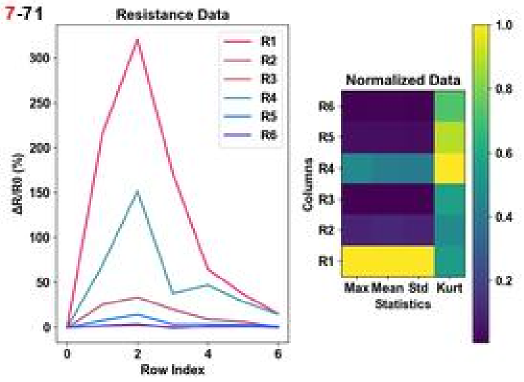

7-72
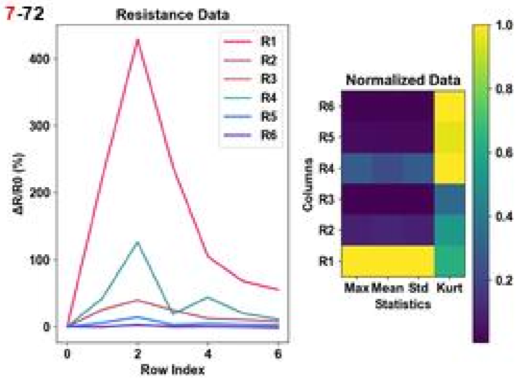

7-73
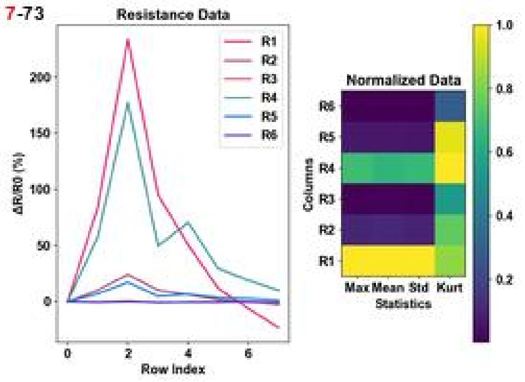

7-74
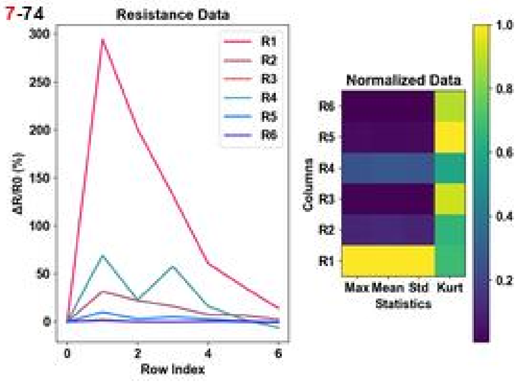

7-75
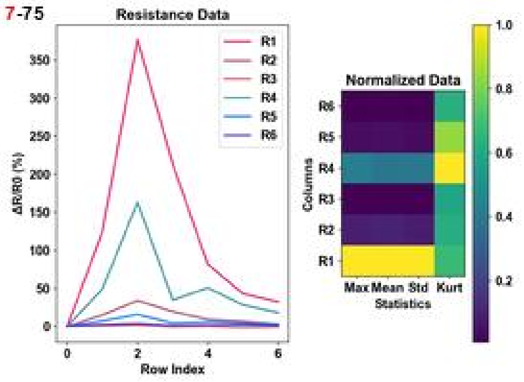

7-76
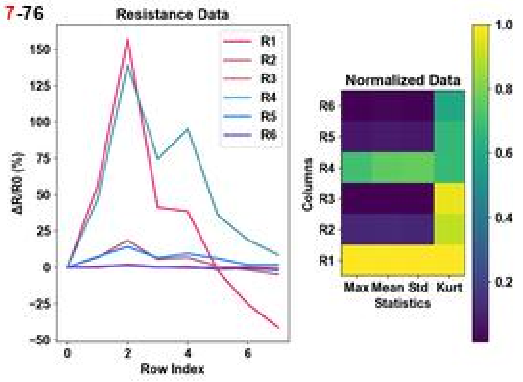

7-77
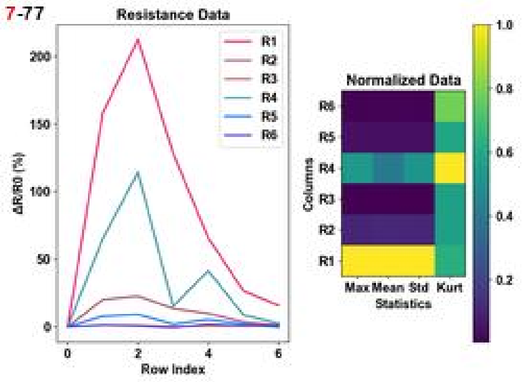

7-78
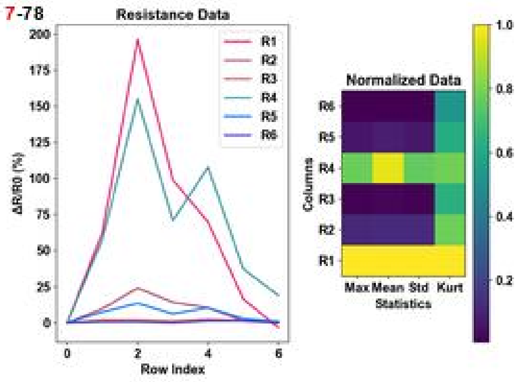

7-79
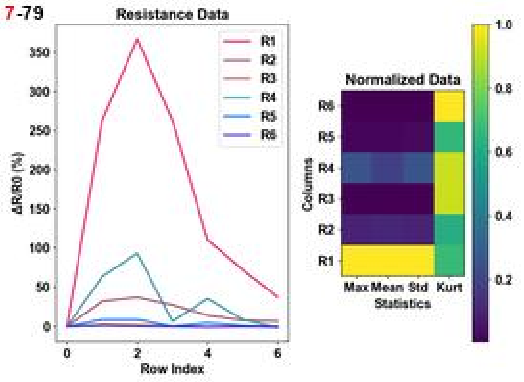

7-80
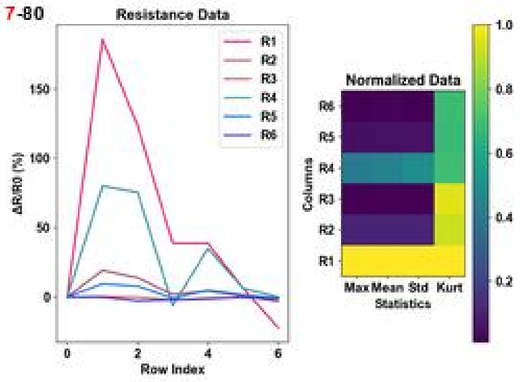

7-81
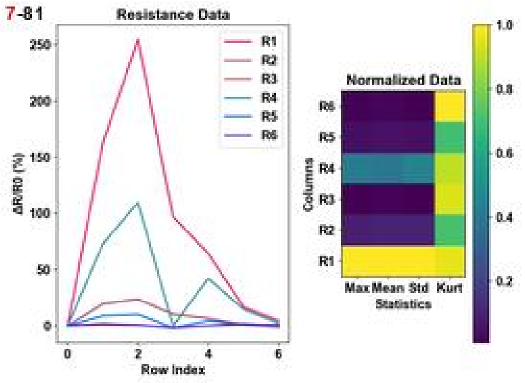

7-82
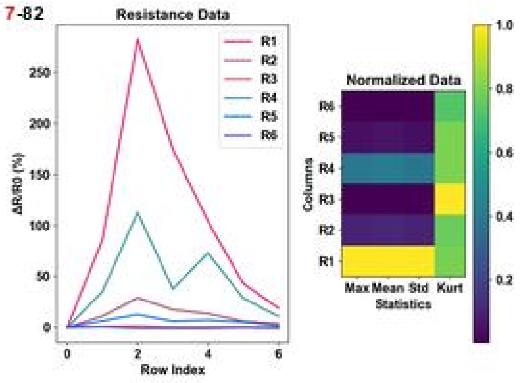

7-83
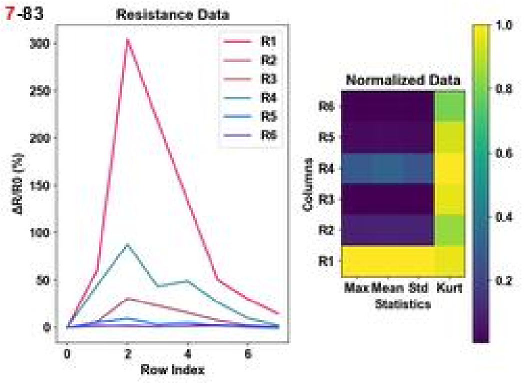

7-84
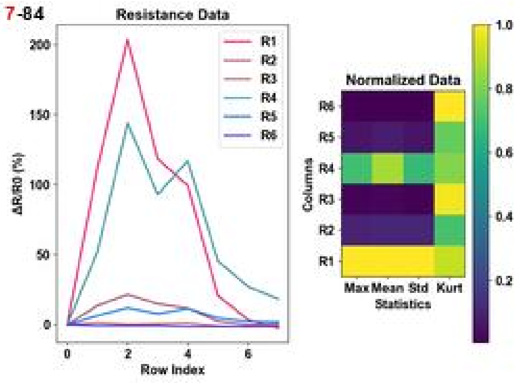

7-85
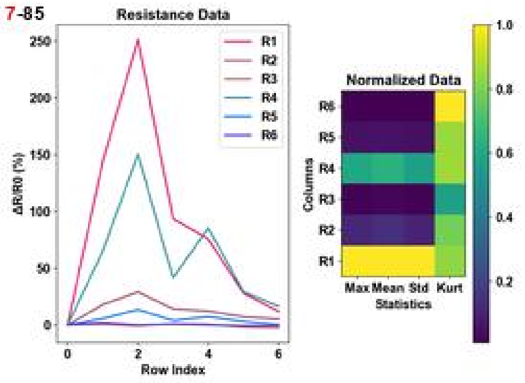

7-86
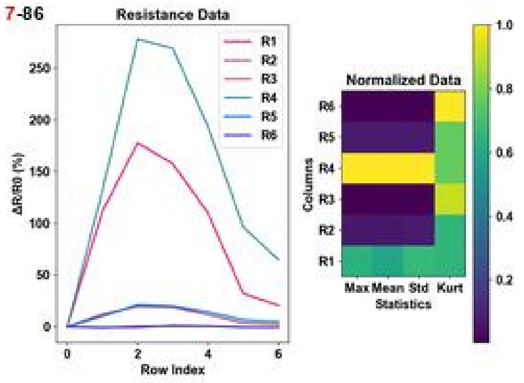

7-87
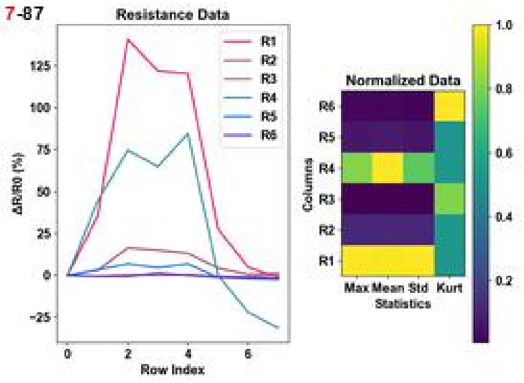

7-88
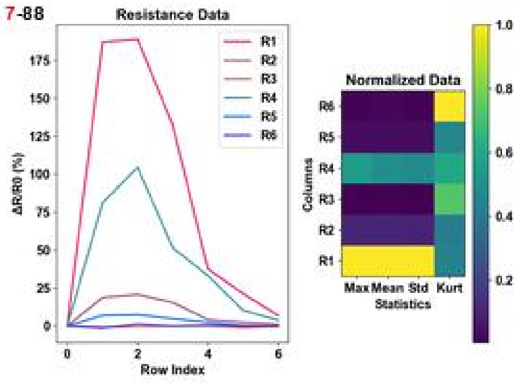

7-89
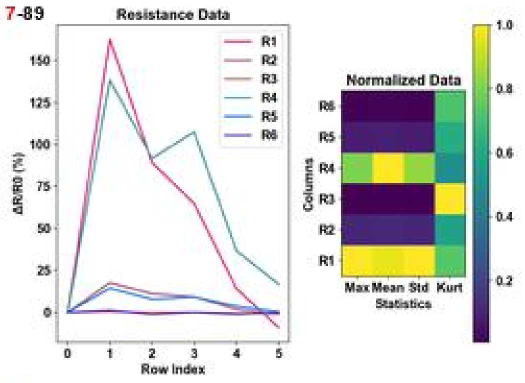

7-90
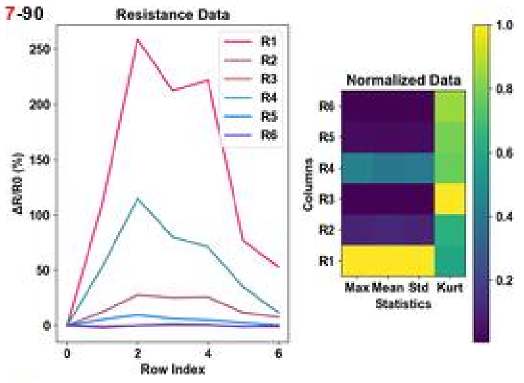

7-91
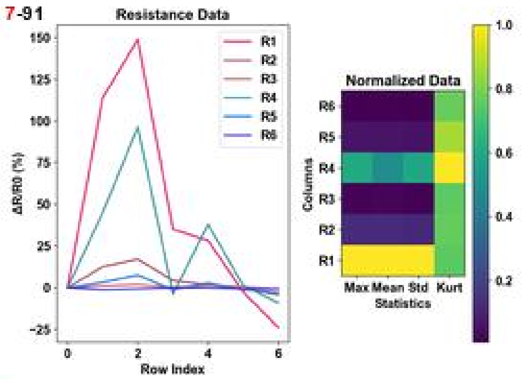

7-92
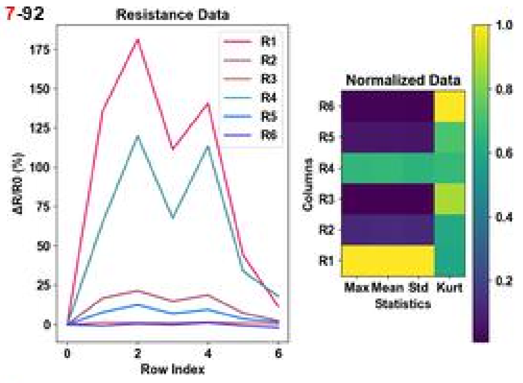

7-93
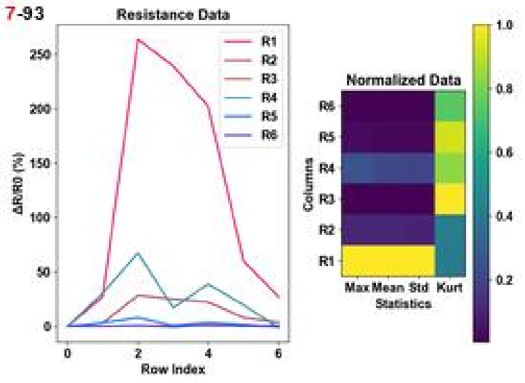

7-94
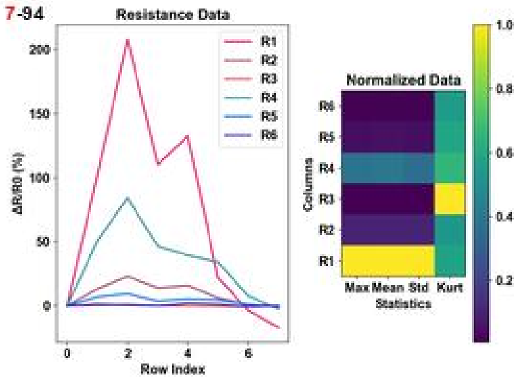

7-95
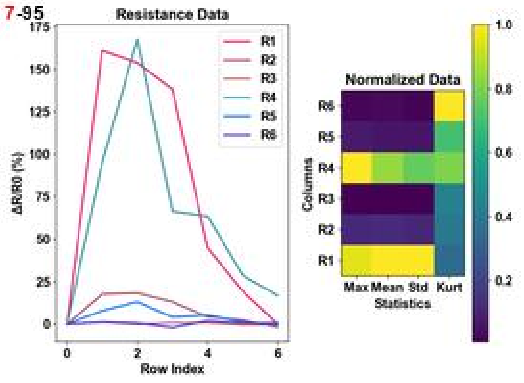

7-96
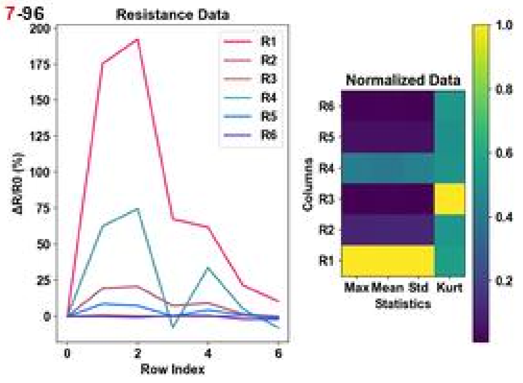

7-97
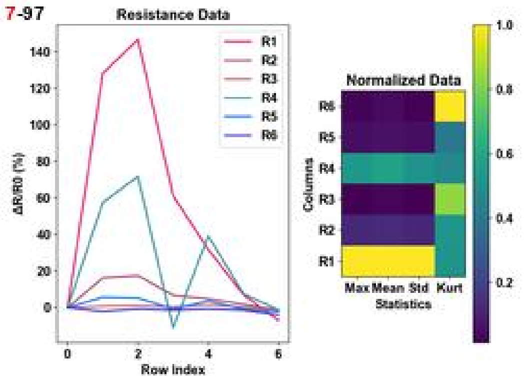

7-98
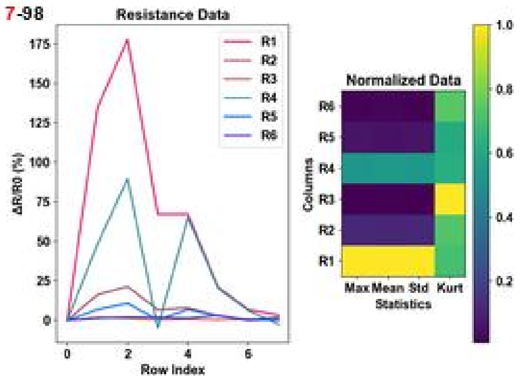

7-99
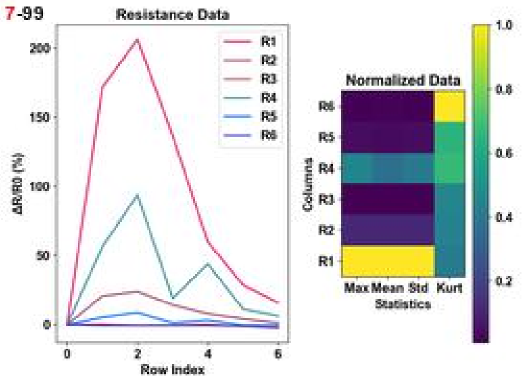

7-100
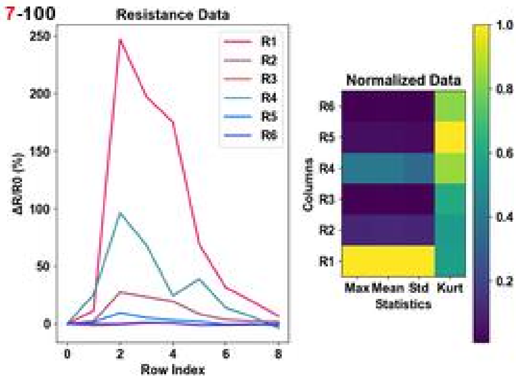

7-101
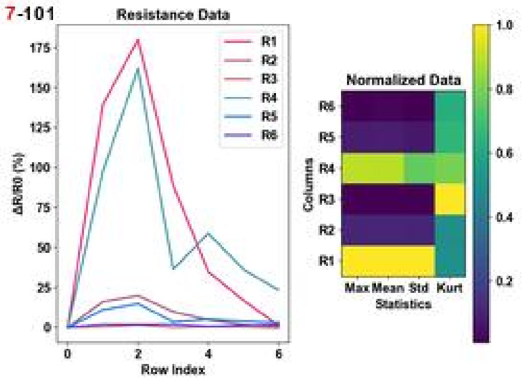

7-102
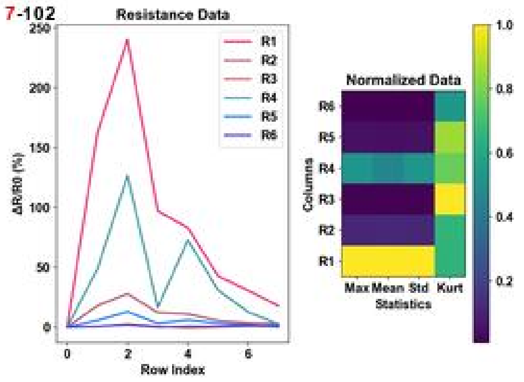

7-103
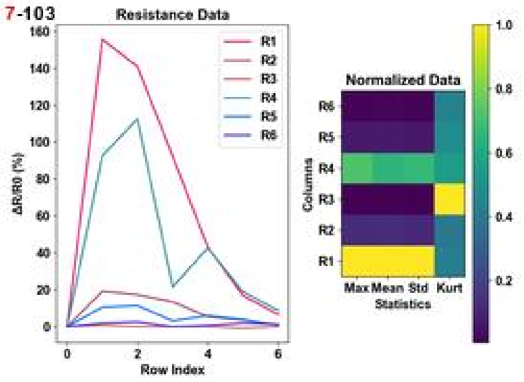

7-104
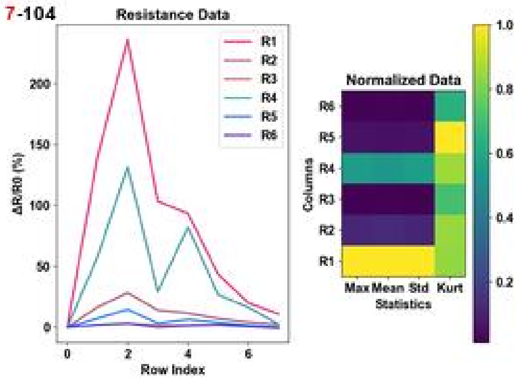

7-105
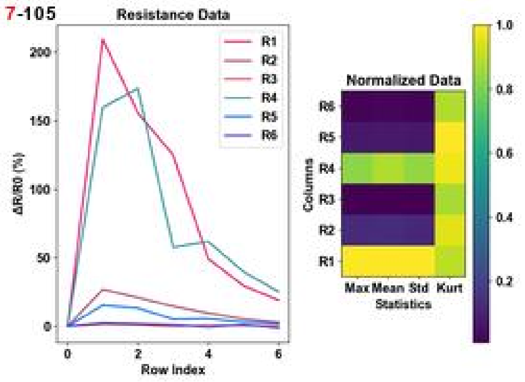

7-106
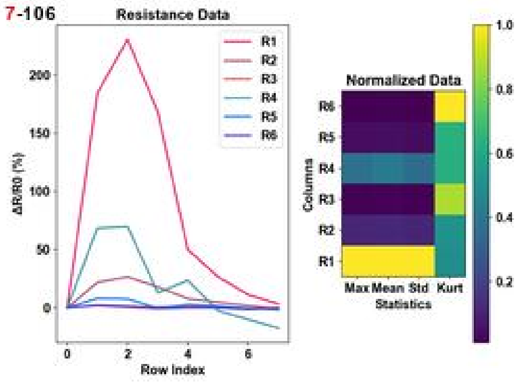

7-107
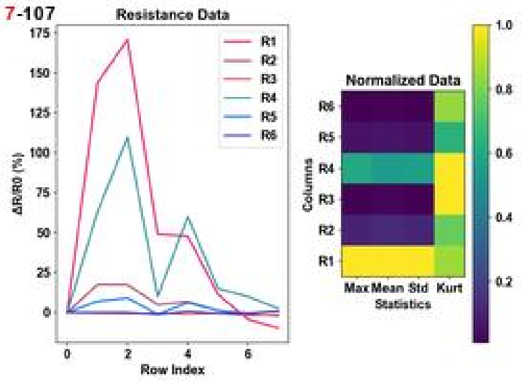

7-108
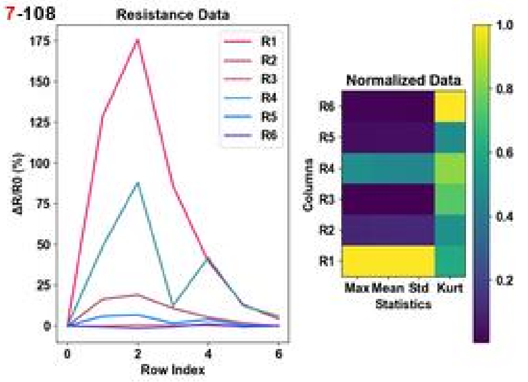

7-109
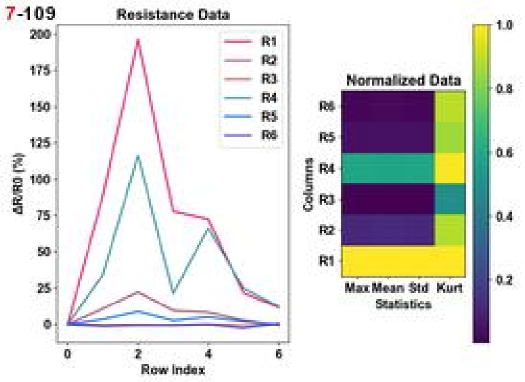

7-110
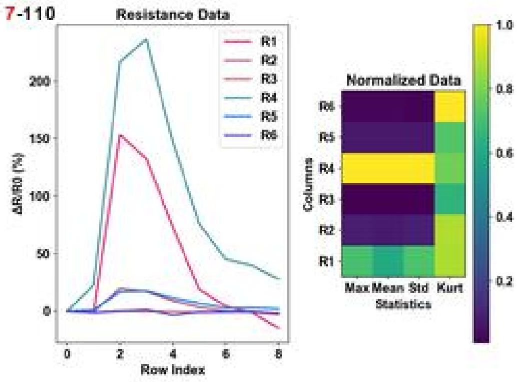

7-111
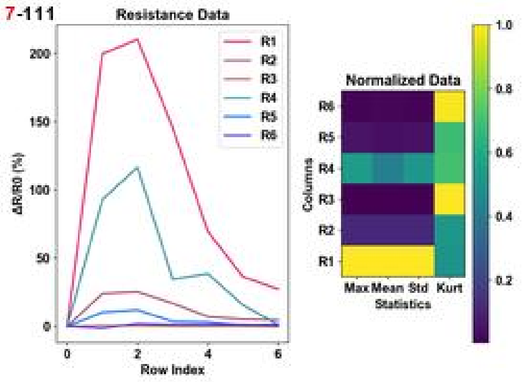

7-112
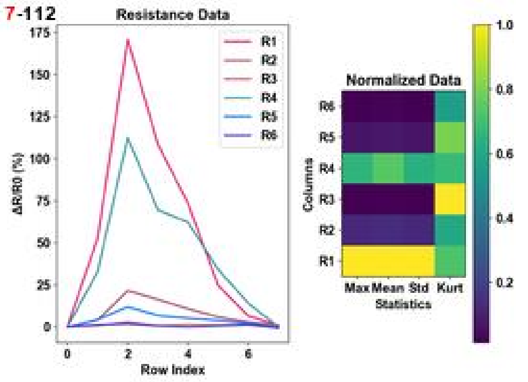

7-113
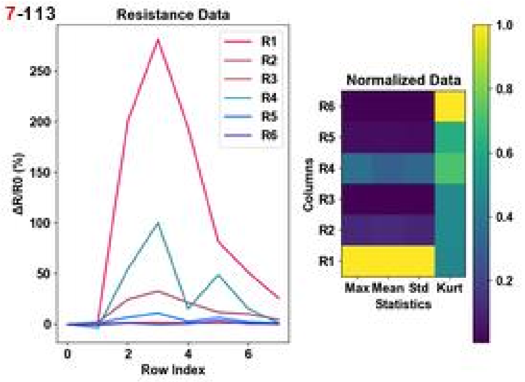

7-114
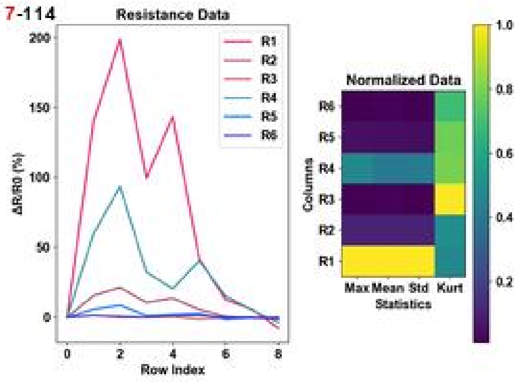

7-115
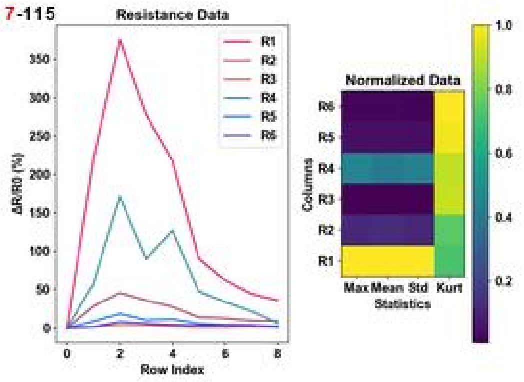

7-116
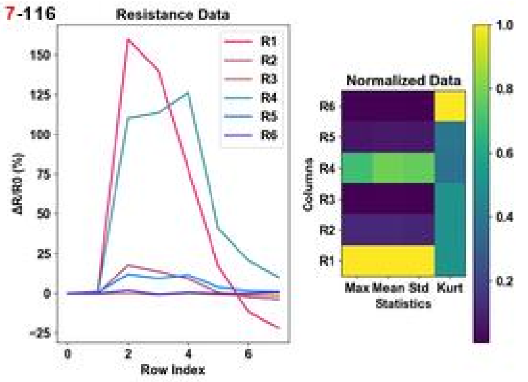

7-117
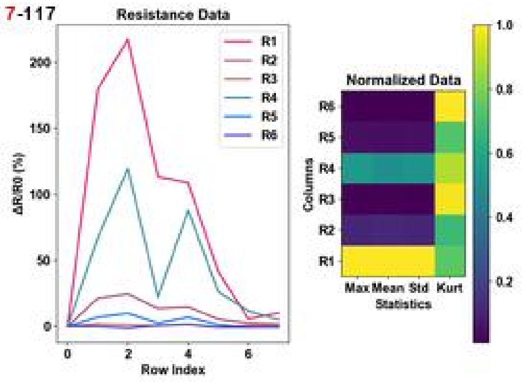

7-118
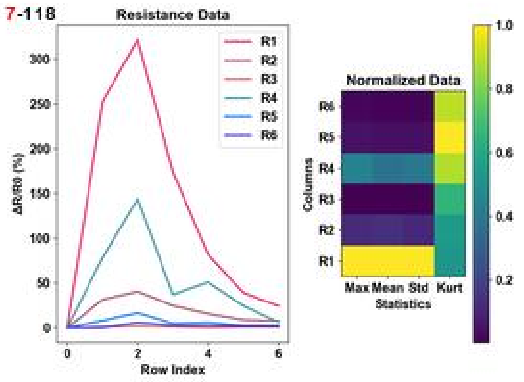

7-119
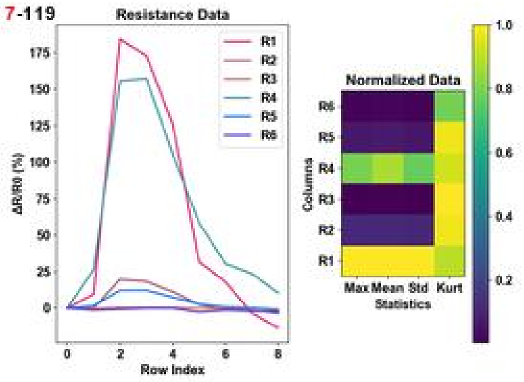

7-120
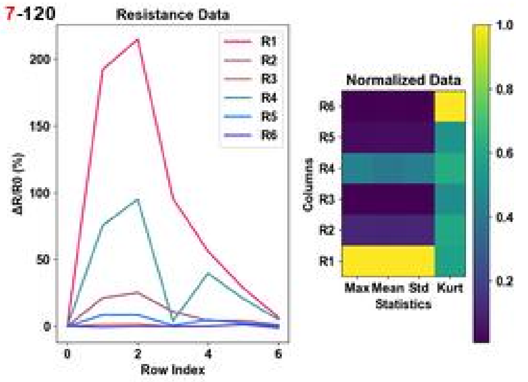

7-121
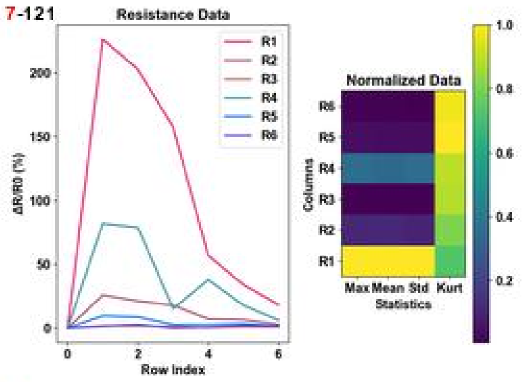

7-122
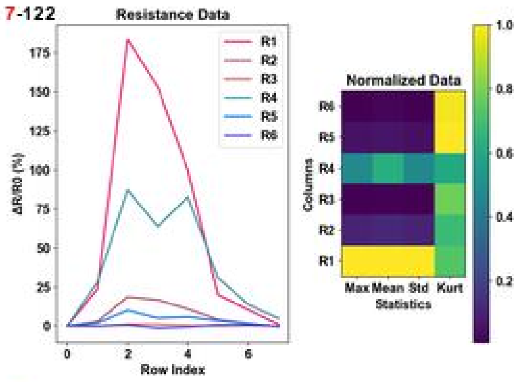

7-123
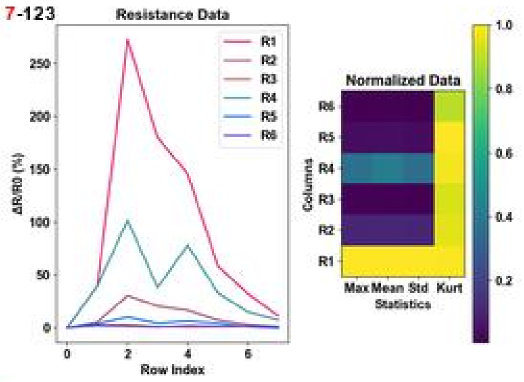

7-124
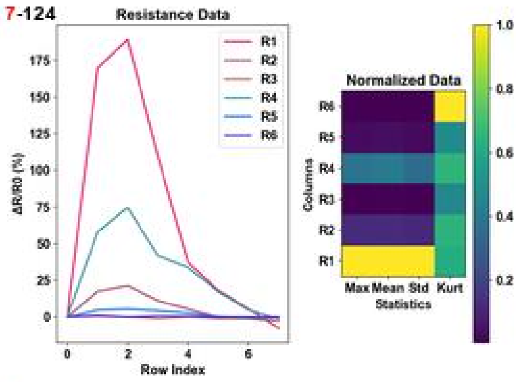

7-125
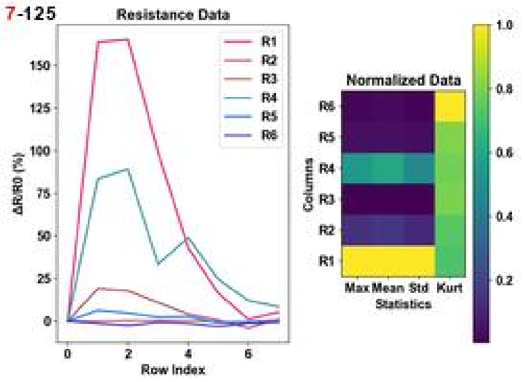

7-126
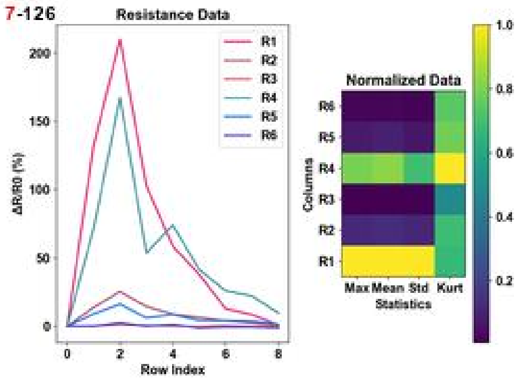

7-127
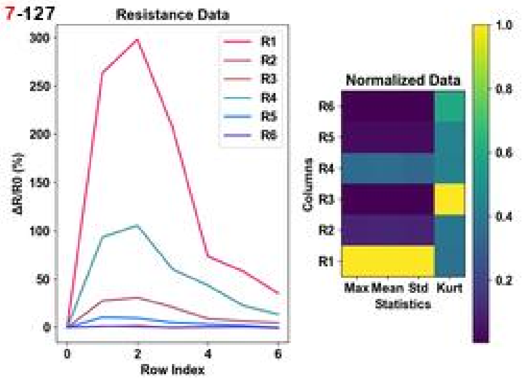

7-128
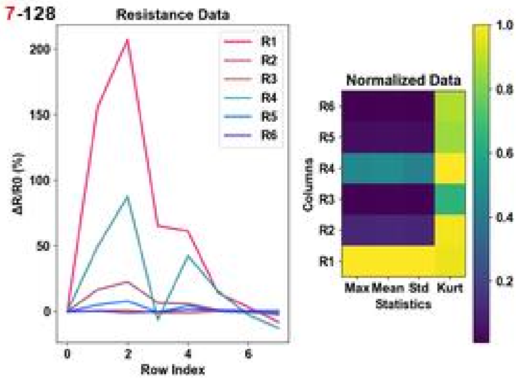

7-129
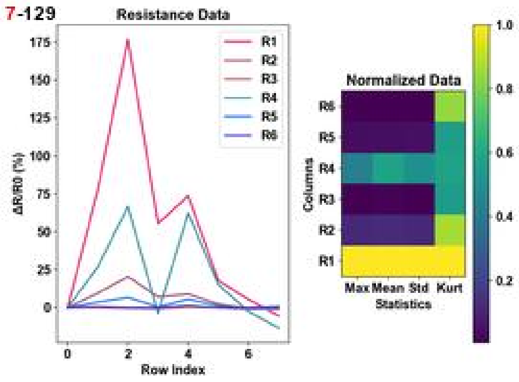

7-130
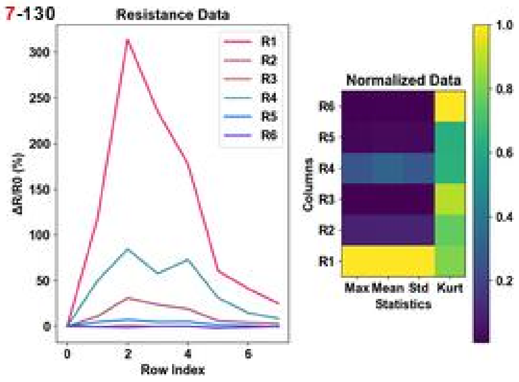

7-131
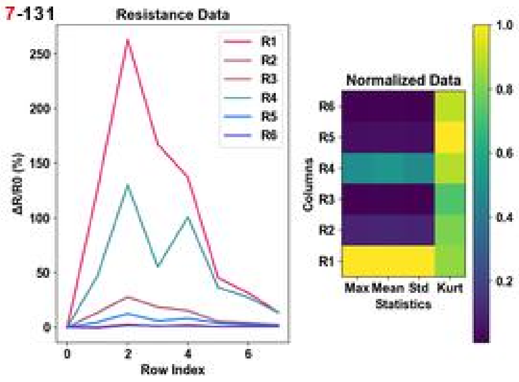

7-132
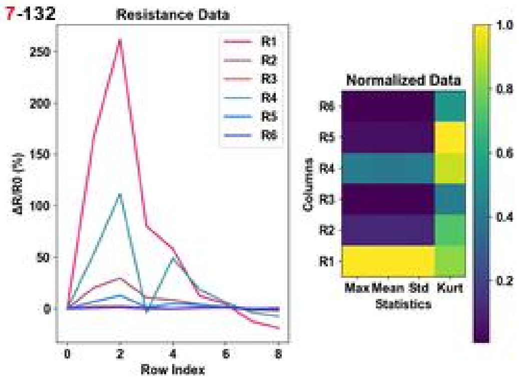

7-133
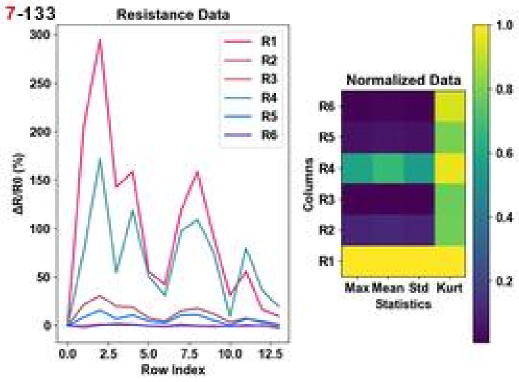

7-134
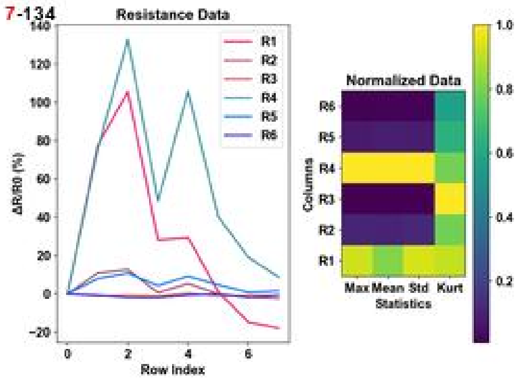

7-135
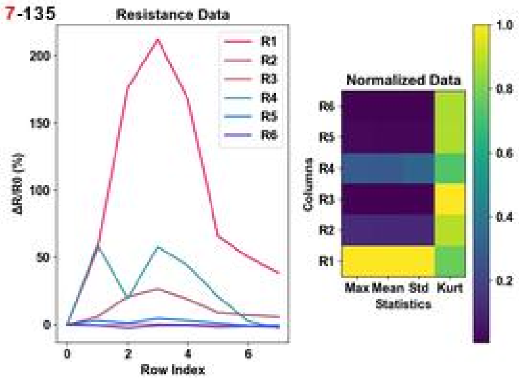

7-136
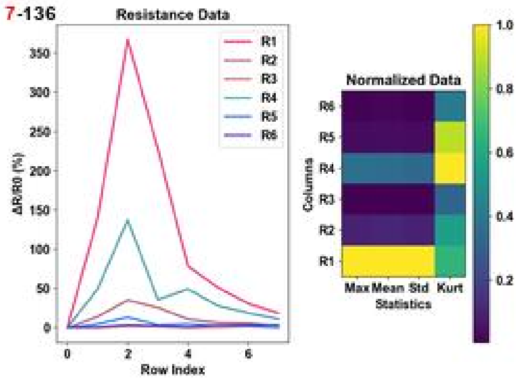

7-137
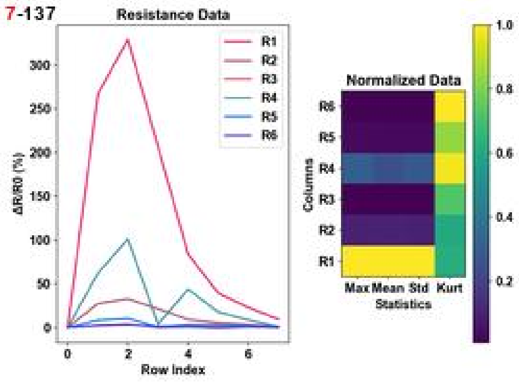

7-138
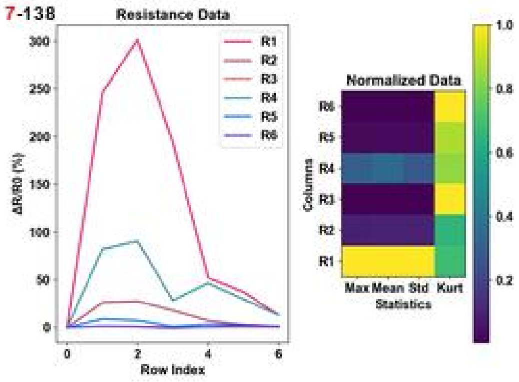

7-139
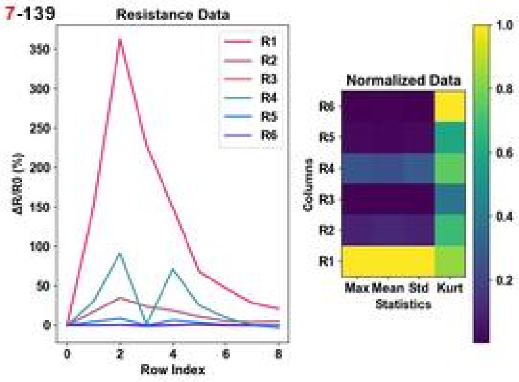

7-140
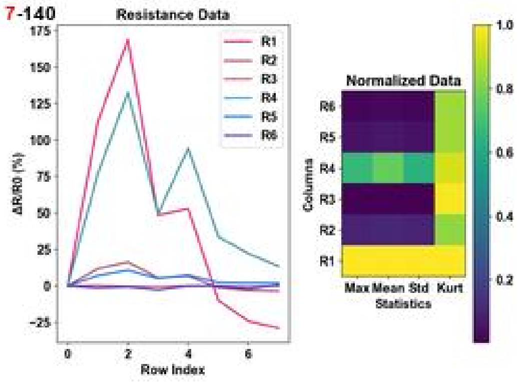

7-141
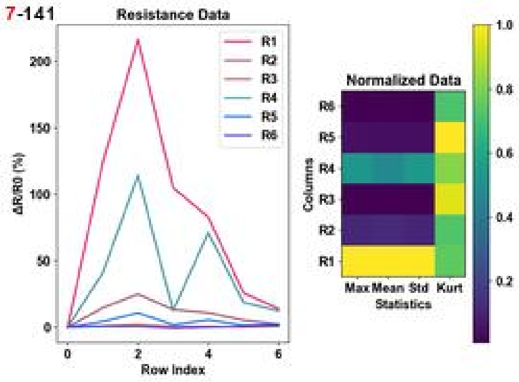

7-142
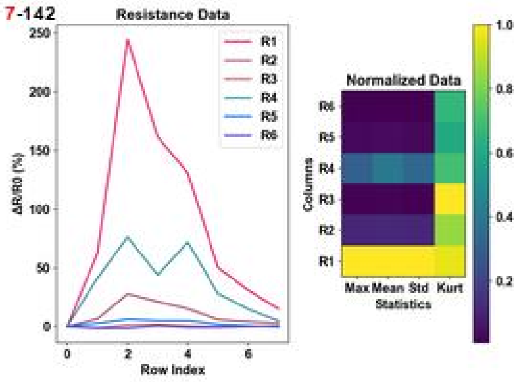

7-143
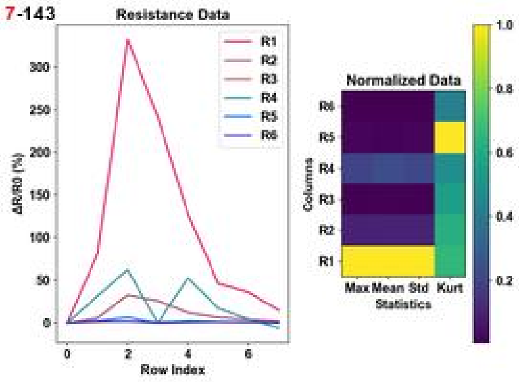

7-144
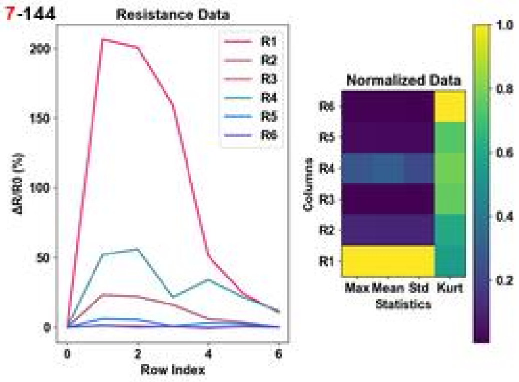

7-145
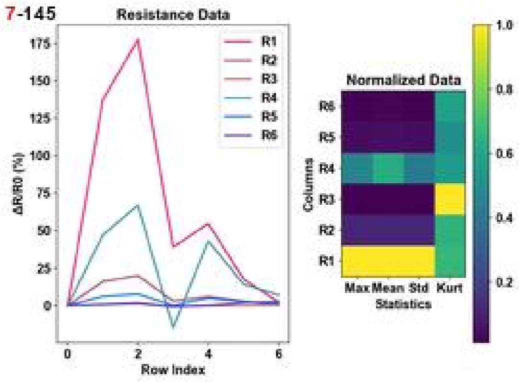

7-146
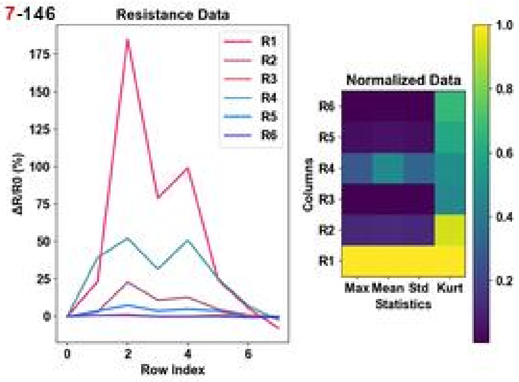

7-147
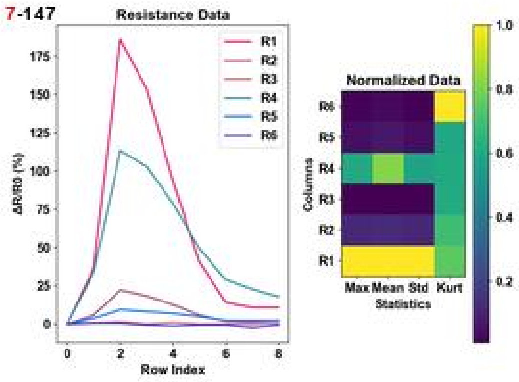

7-148
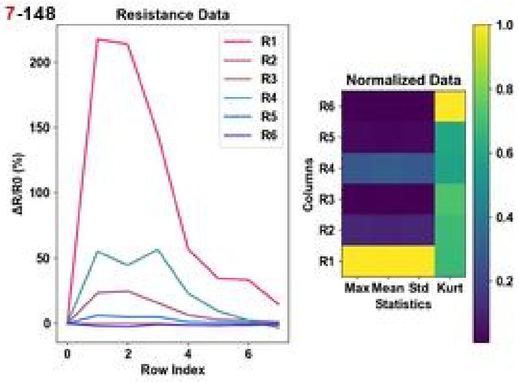

7-149
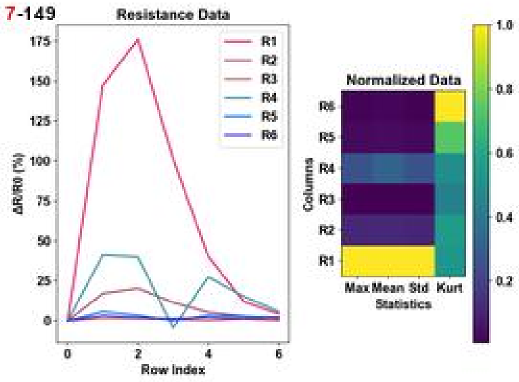

7-150
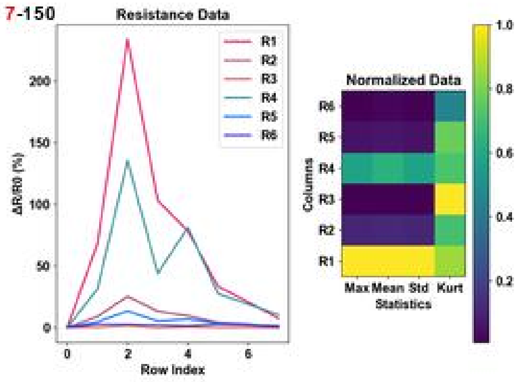

7-151
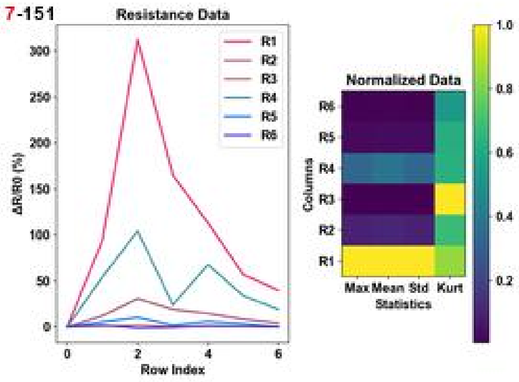

7-152
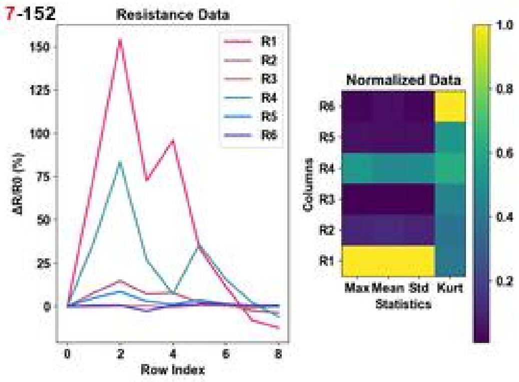

7-153
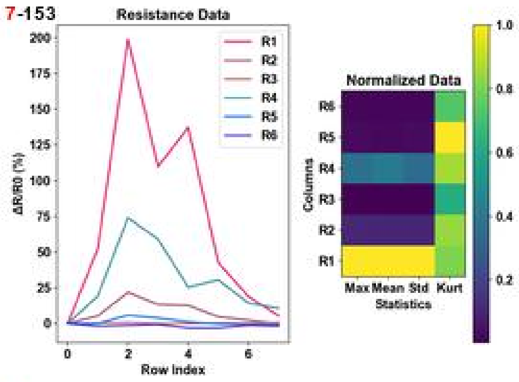

7-154
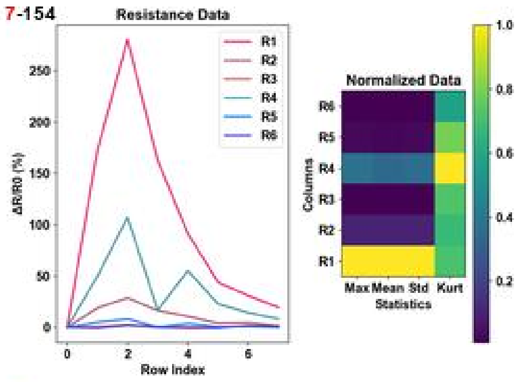

7-155
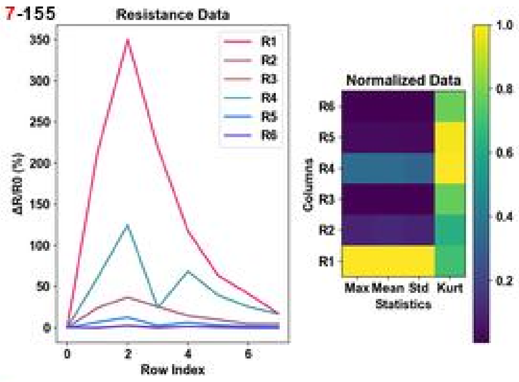

7-156
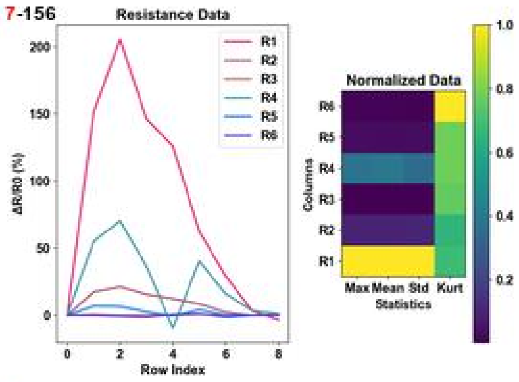

7-157
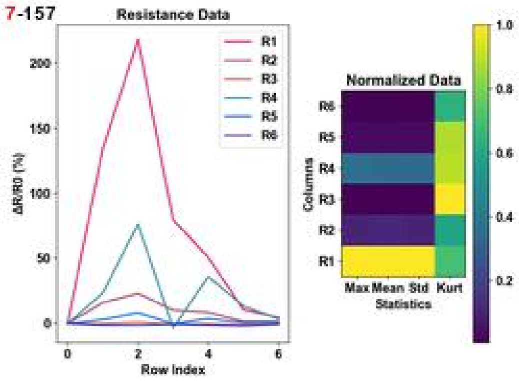

7-158
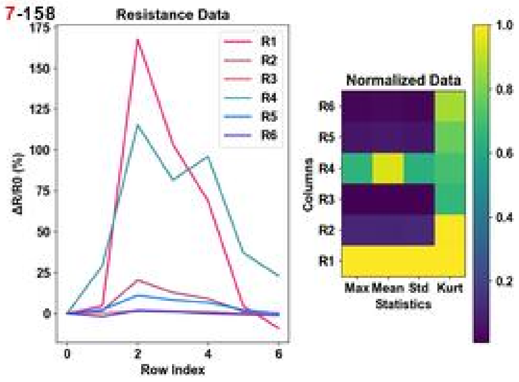

7-159
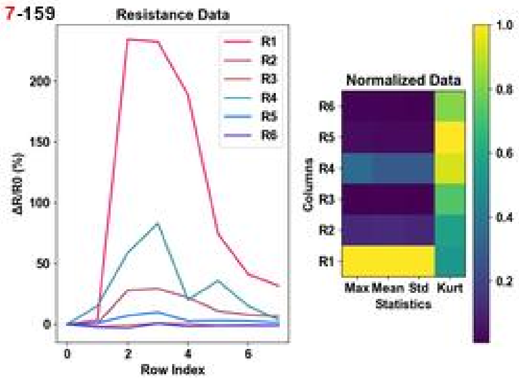

7-160
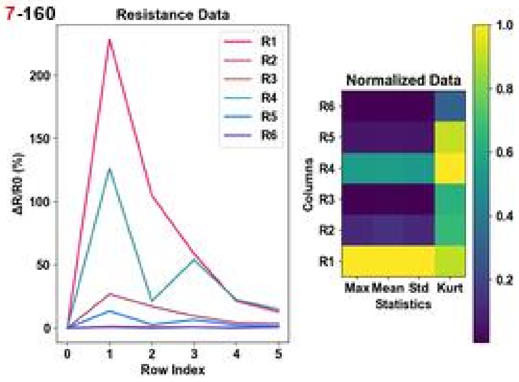

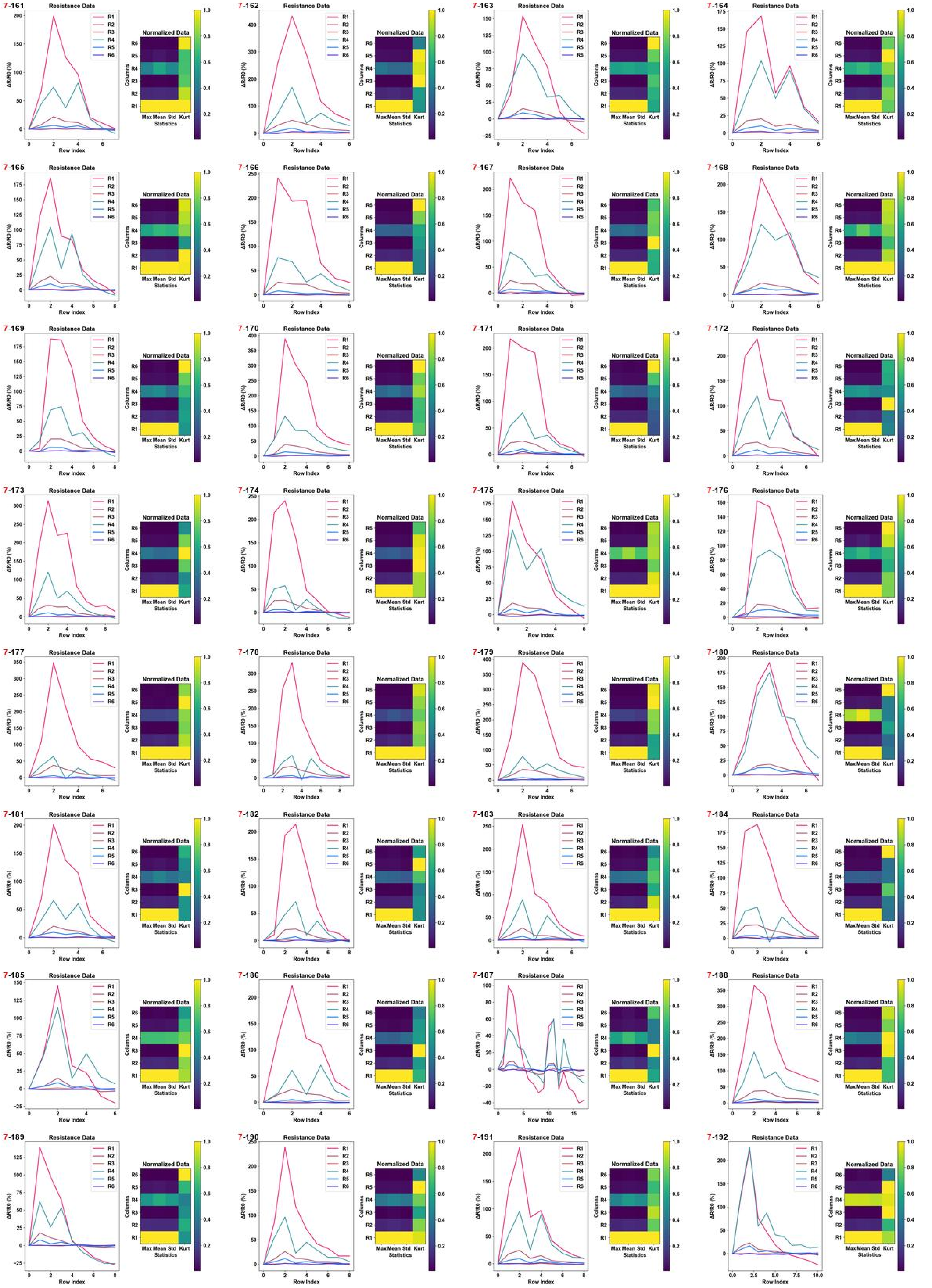
7-161
7-162
7-163
7-164
7-165
7-166
7-167
7-168
7-169
7-170
7-171
7-172
7-173
7-174
7-175
7-176
7-177
7-178
7-179
7-180
7-181
7-182
7-183
7-184
7-185
7-186
7-187
7-188
7-189
7-190
7-191
7-192
Resistance Data
Normalized Data
ΔR/R0 (%)
Row Index
Columns
Statistics
Max Mean Std Kurt
R1
R2
R3
R4
R5
R6

Pressure-sensitive data of type 7

7-193
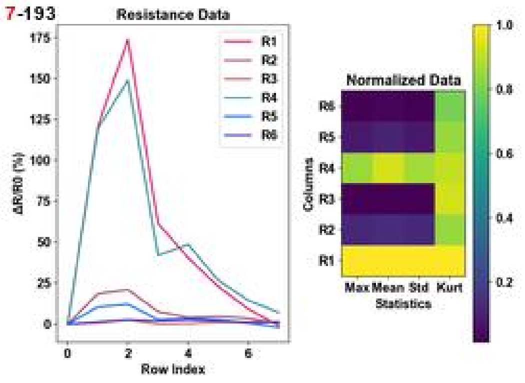

7-194
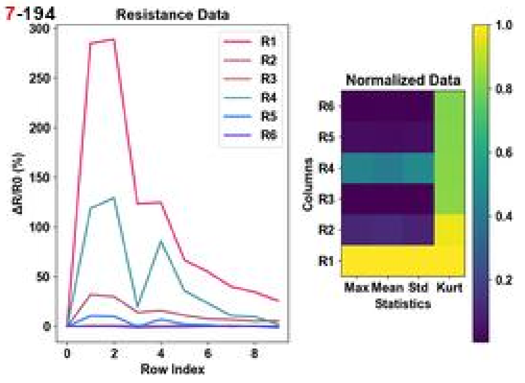

7-195
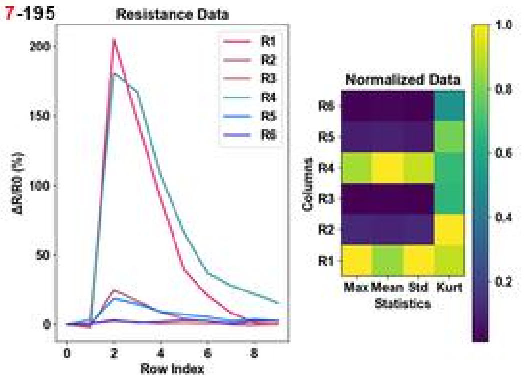

7-196
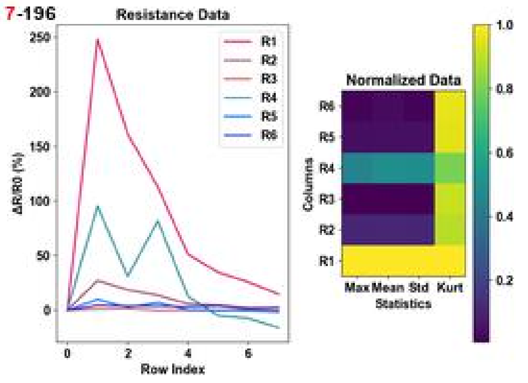

7-197
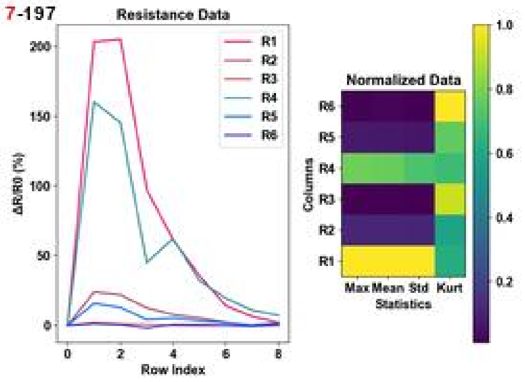

7-198
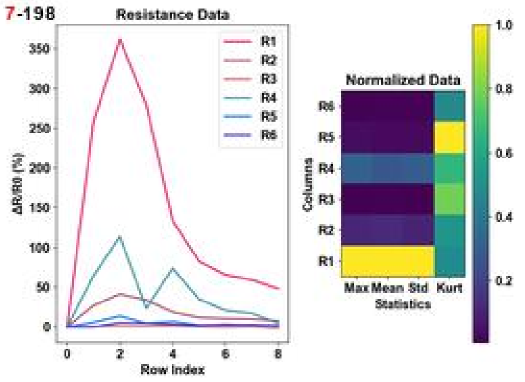

7-199
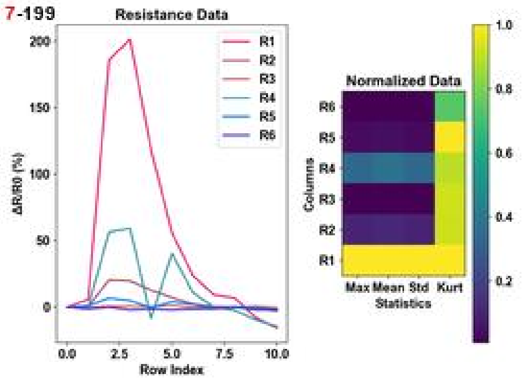

7-200
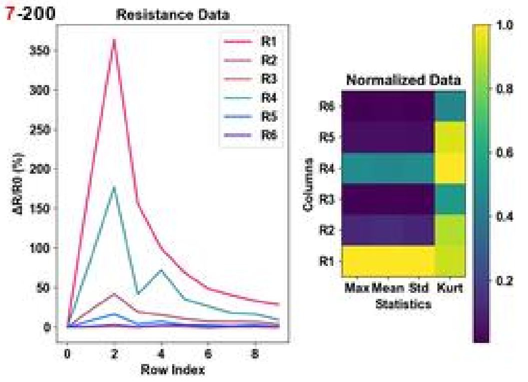

7-201
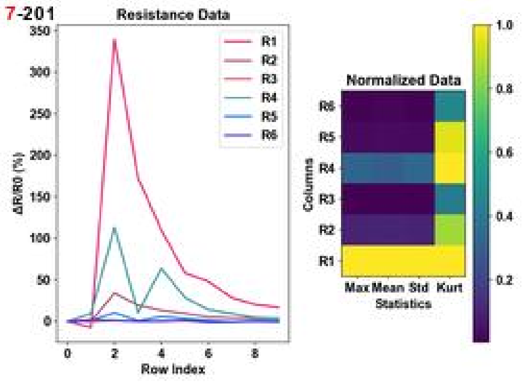

7-202
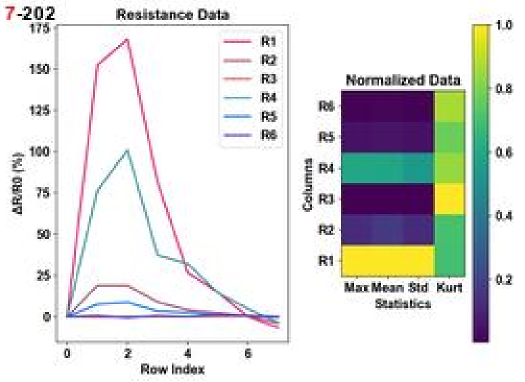

7-203
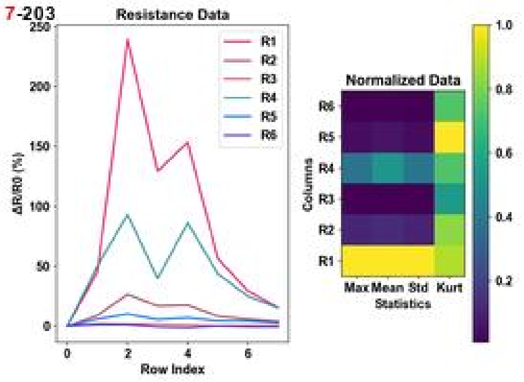

7-204
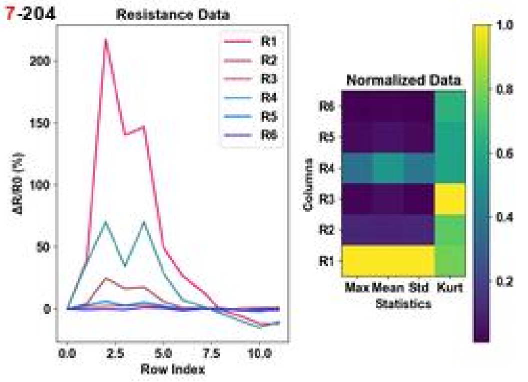

7-205
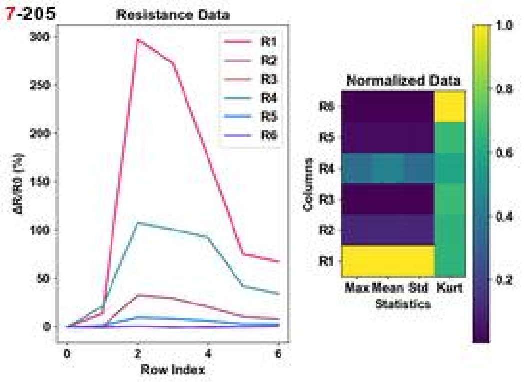

7-206
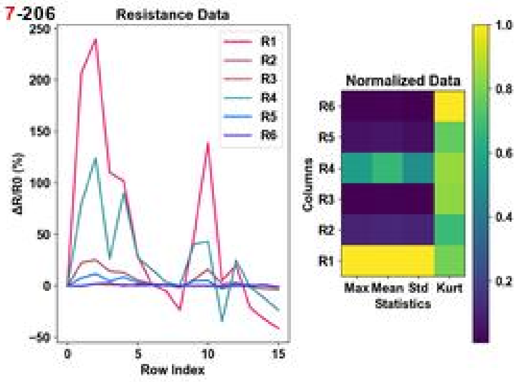

7-207
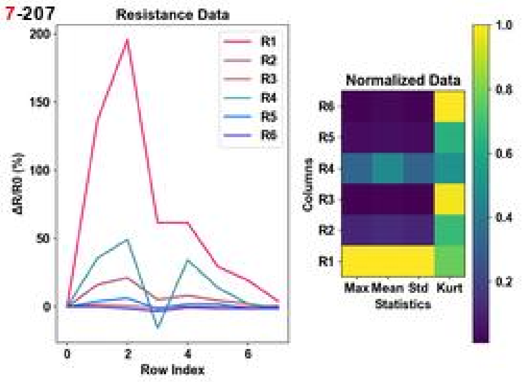

7-208
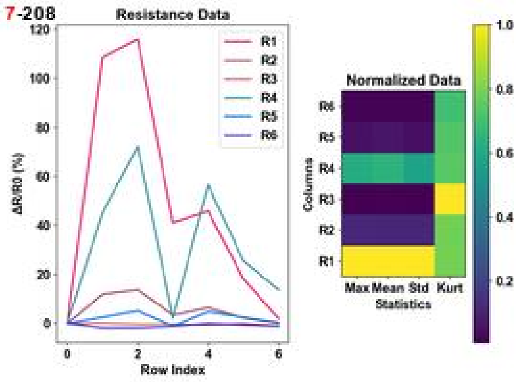

7-209
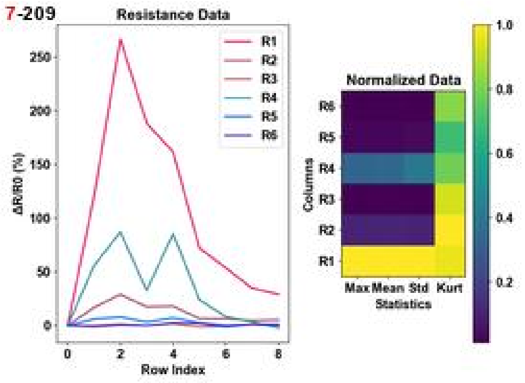

7-210
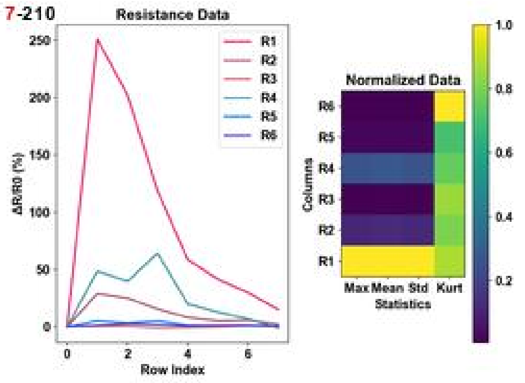

7-211
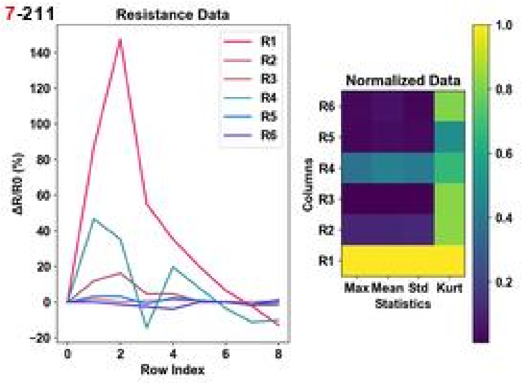

7-212
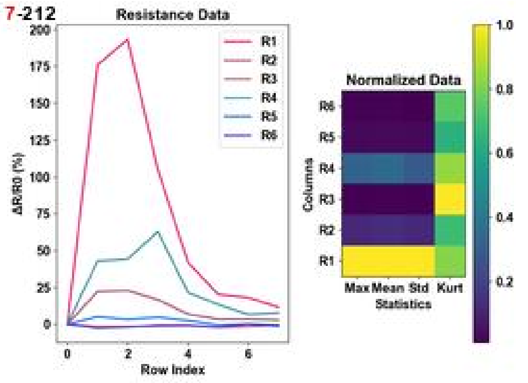

7-213
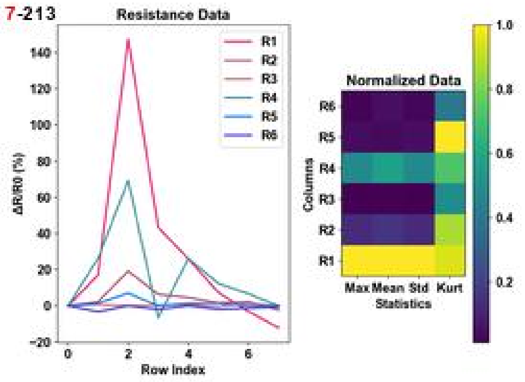

7-214
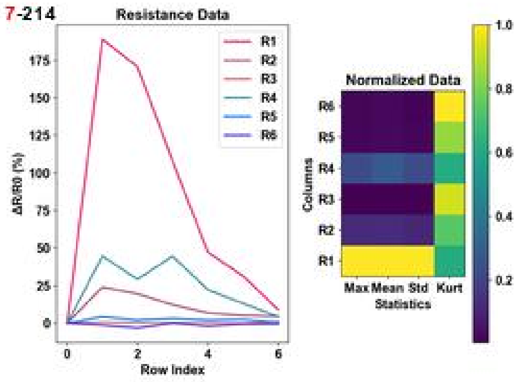

7-215
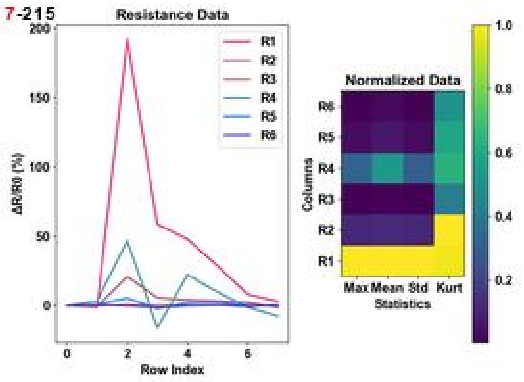

7-216
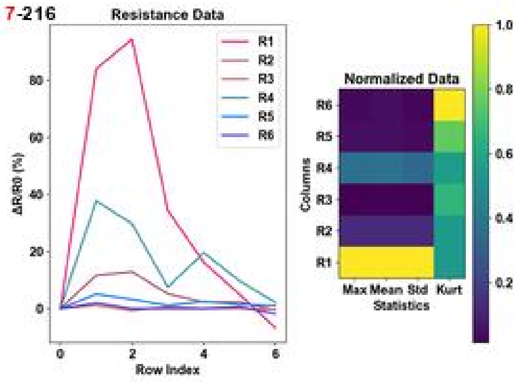

7-217
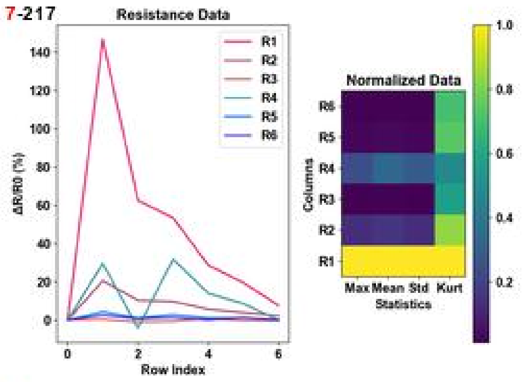

7-218
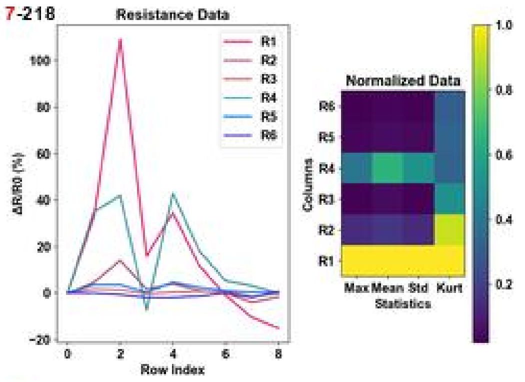

7-219
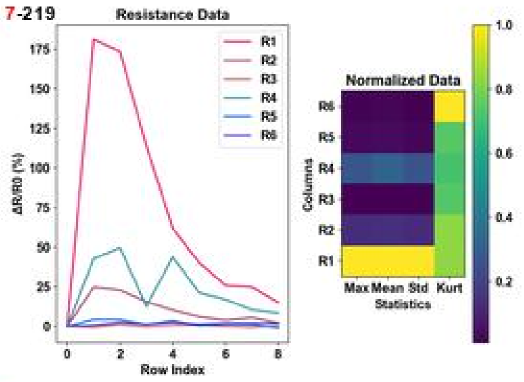

7-220
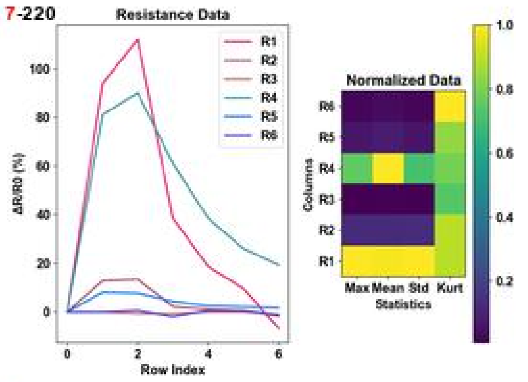

7-221
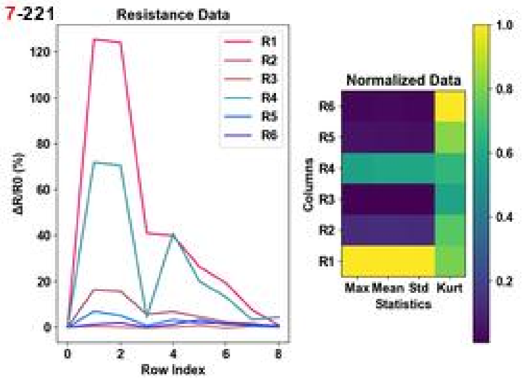

7-222
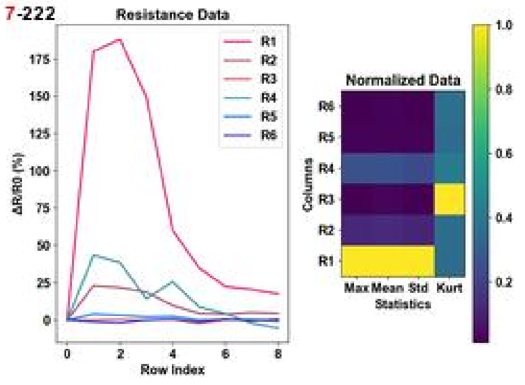

7-223
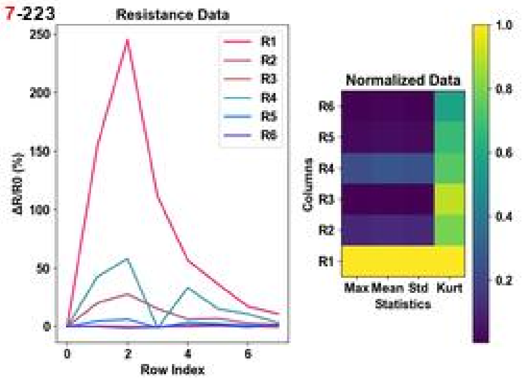

7-224
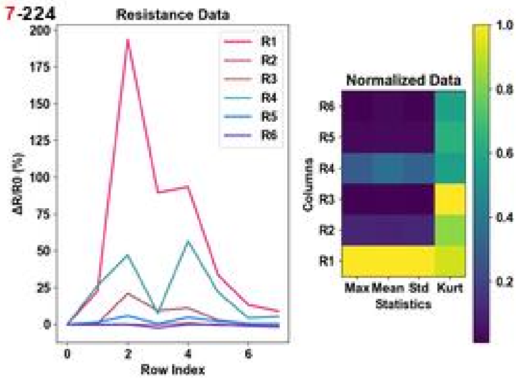

Pressure-sensitive data of type 7

7-225

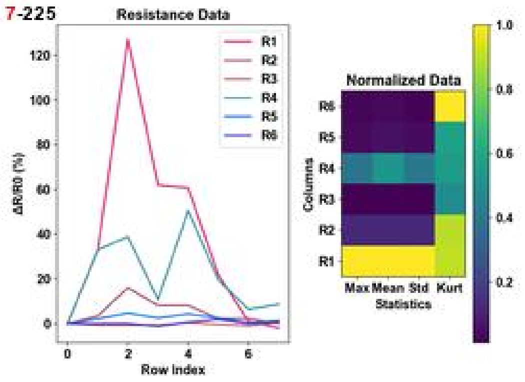

7-226

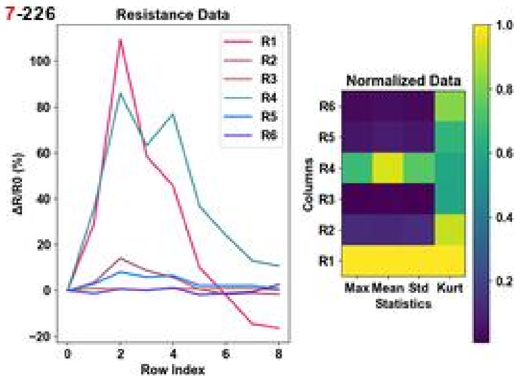

7-227

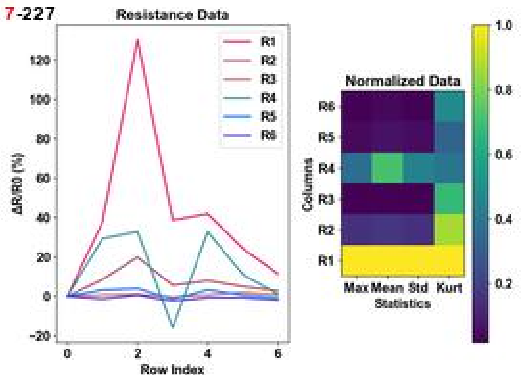

7-228

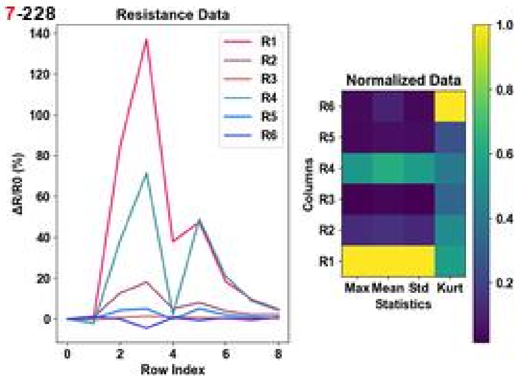

7-229

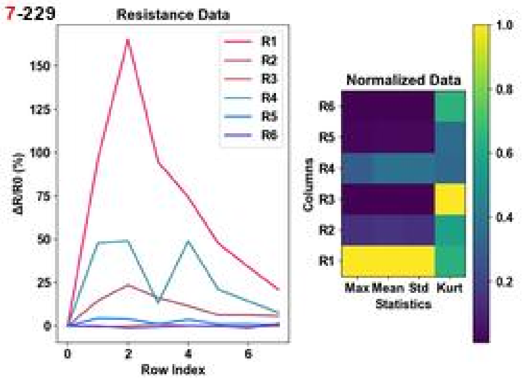

7-230

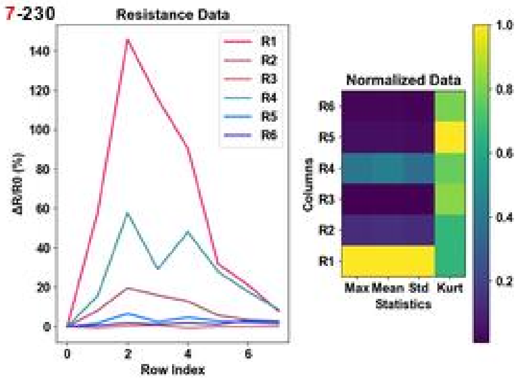

7-231

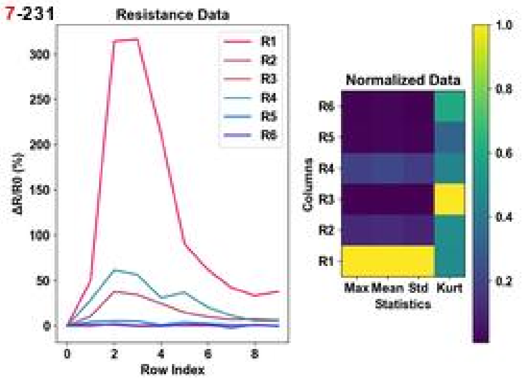

7-232

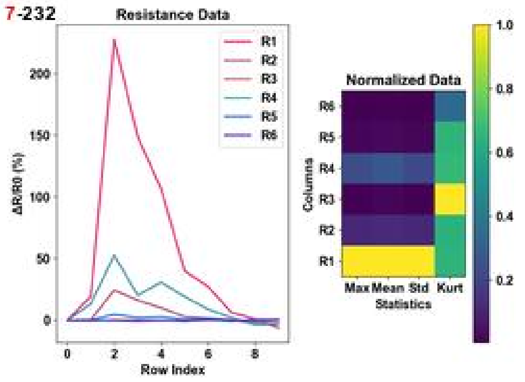

7-233

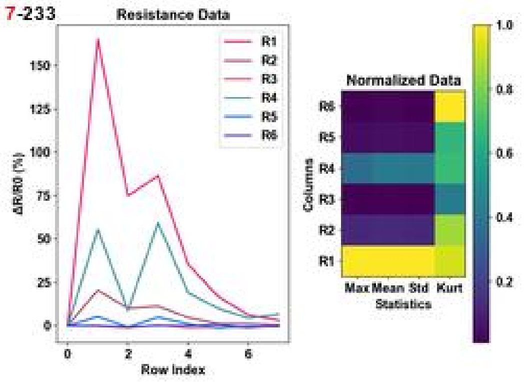

7-234

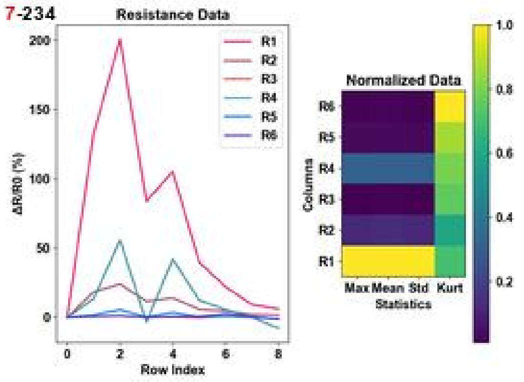

7-235

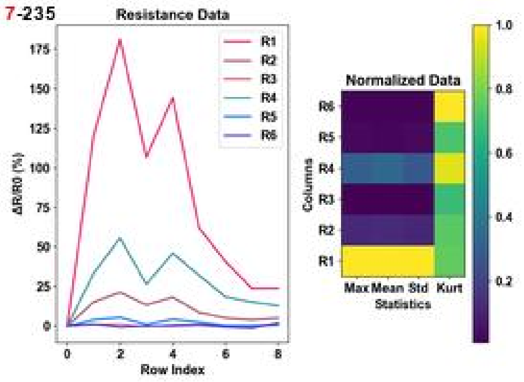

7-236

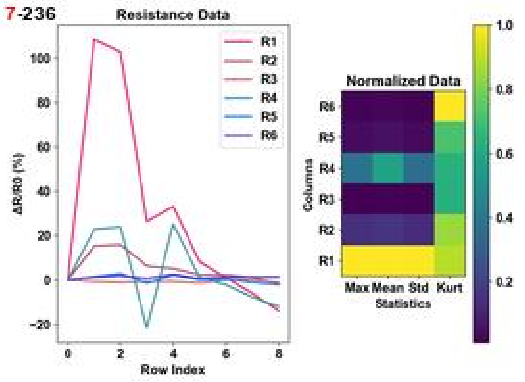

7-237

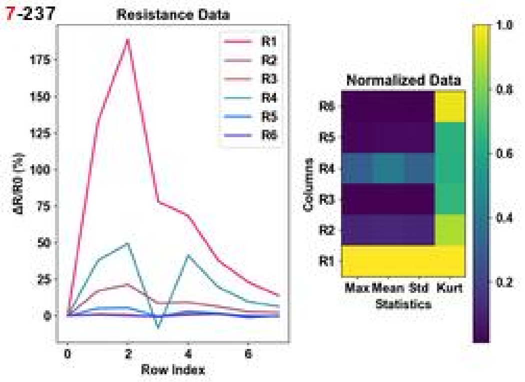

7-238

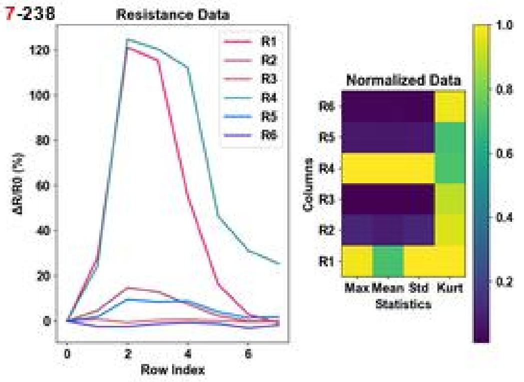

7-239

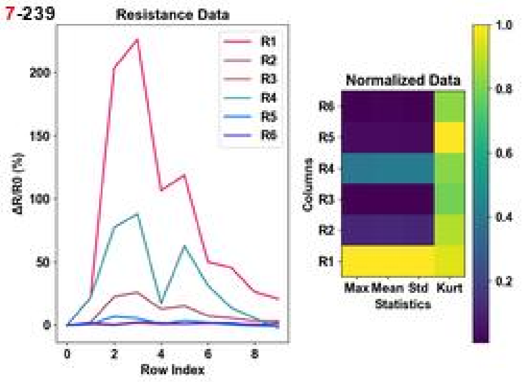

7-240

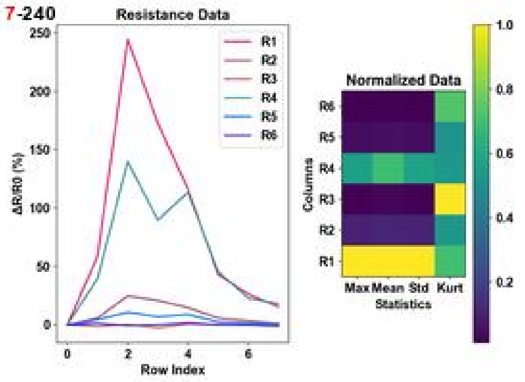

Pressure-sensitive data of type 8

8-1
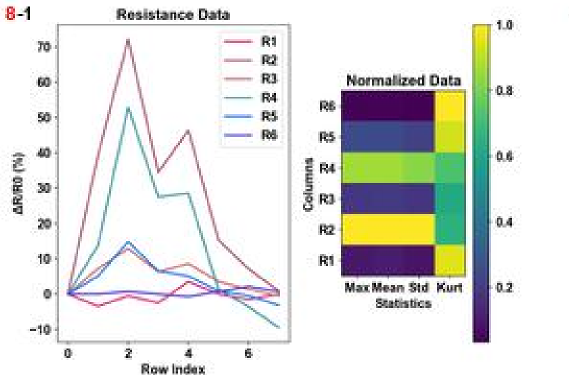

8-2
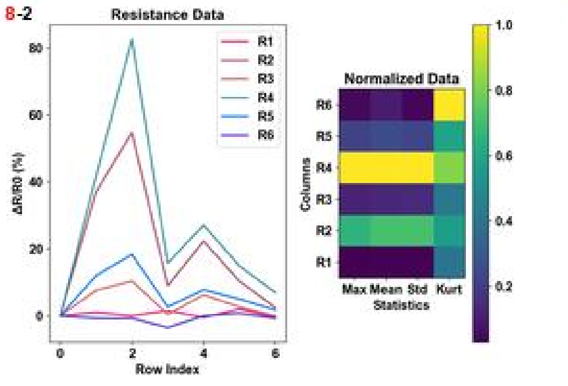

8-3
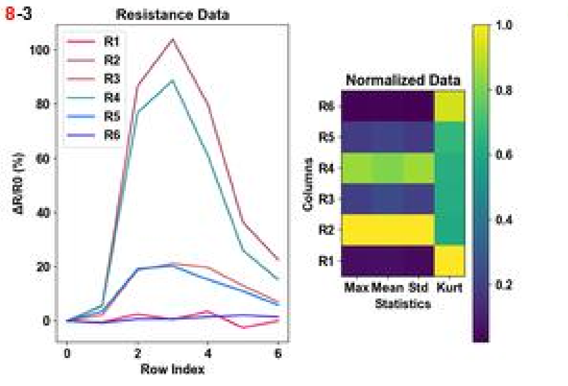

8-4
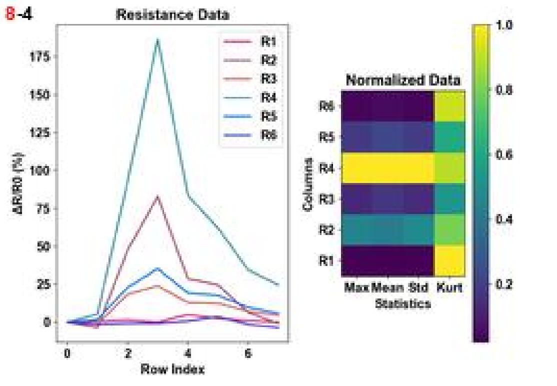

8-5
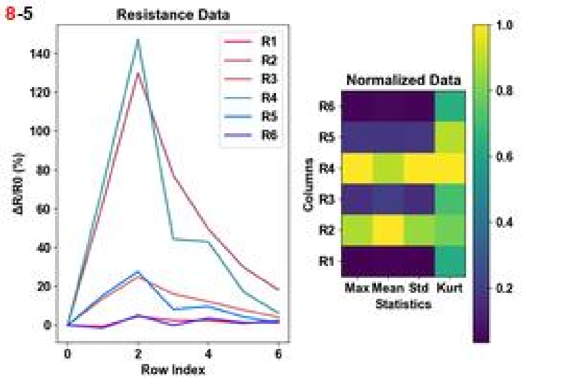

8-6
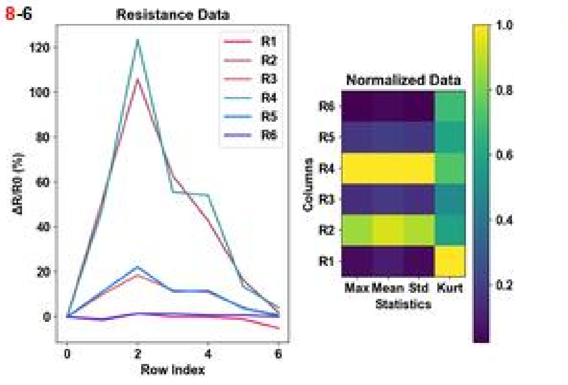

8-7
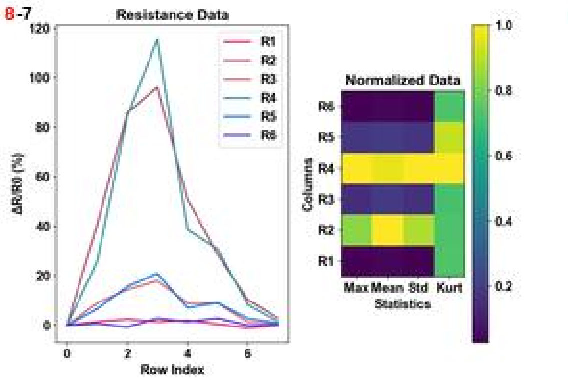

8-8
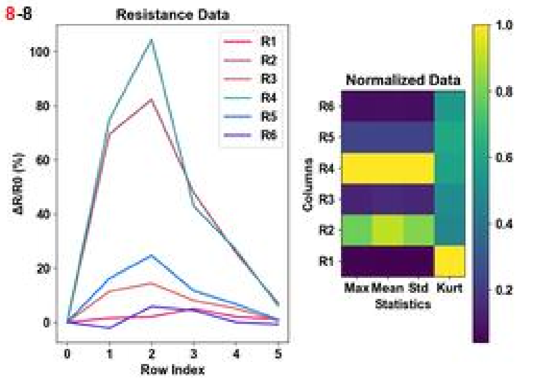

8-9
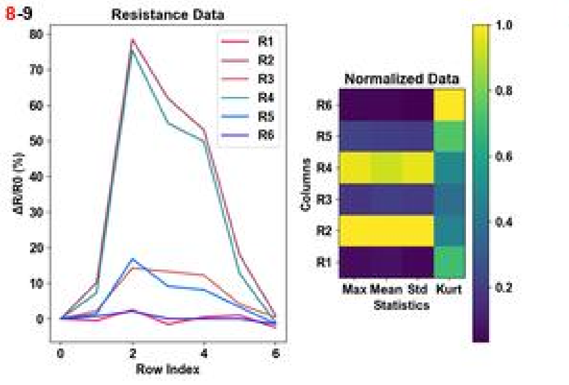

8-10
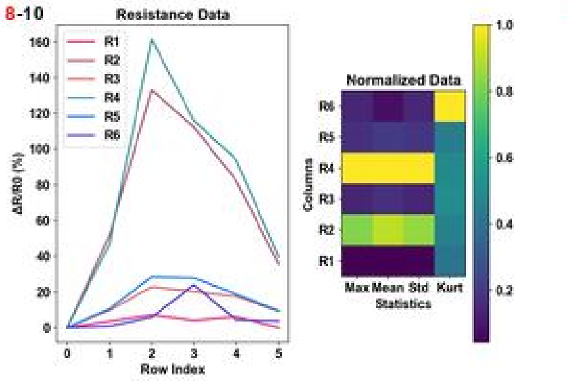

8-11
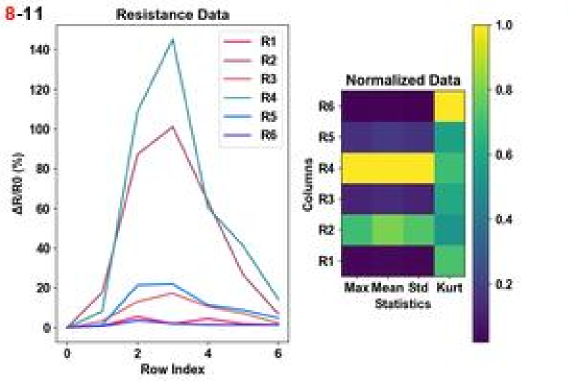

8-12
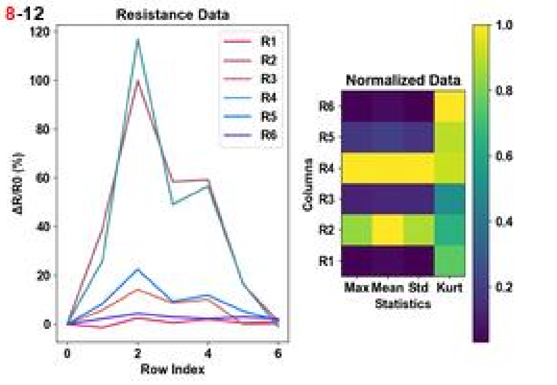

8-13
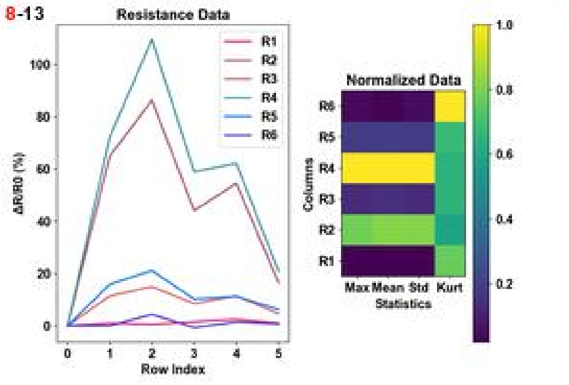

8-14
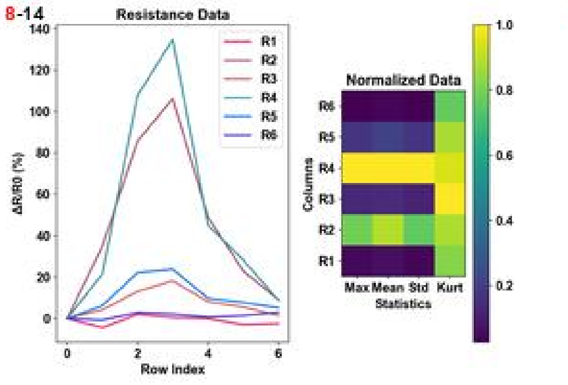

8-15
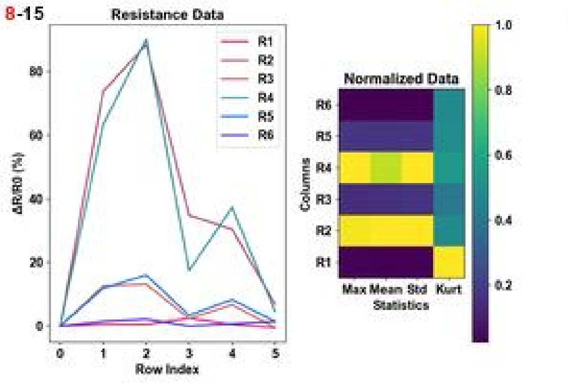

8-16
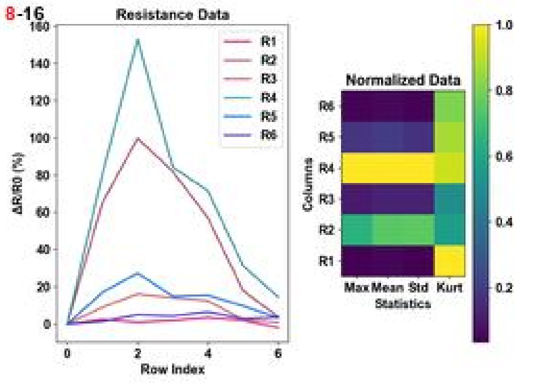

8-17
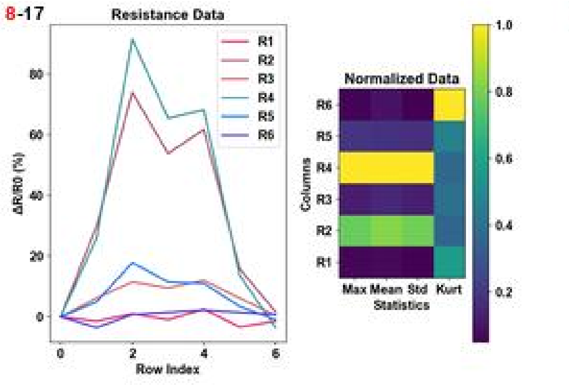

8-18
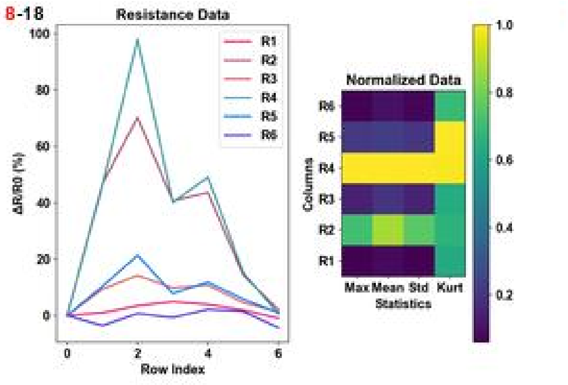

8-19
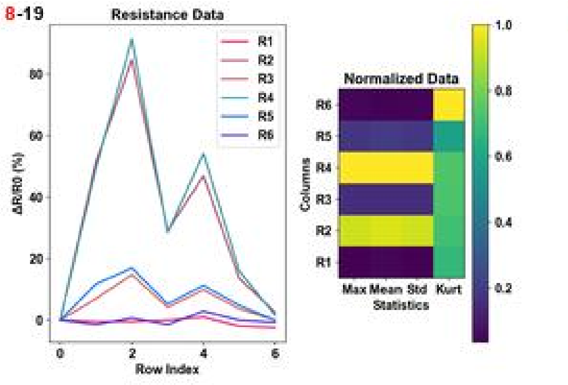

8-20
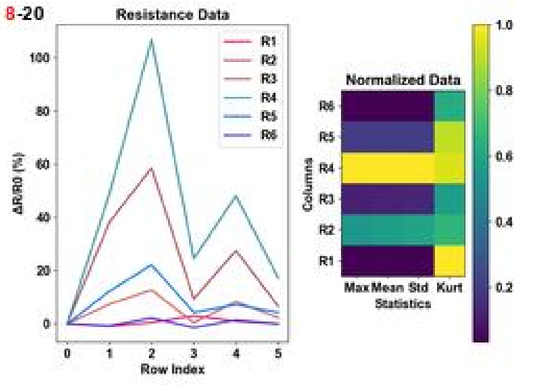

8-21
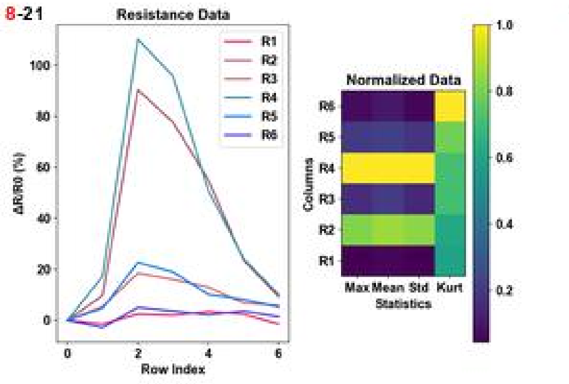

8-22
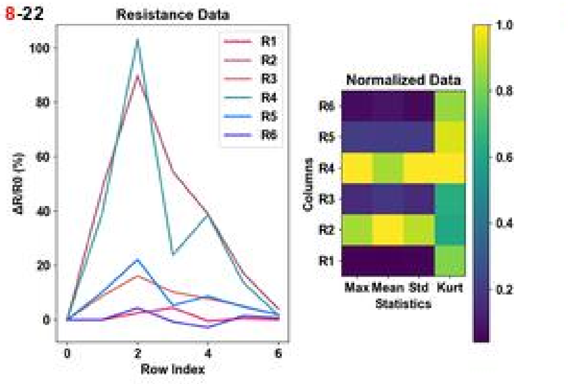

8-23
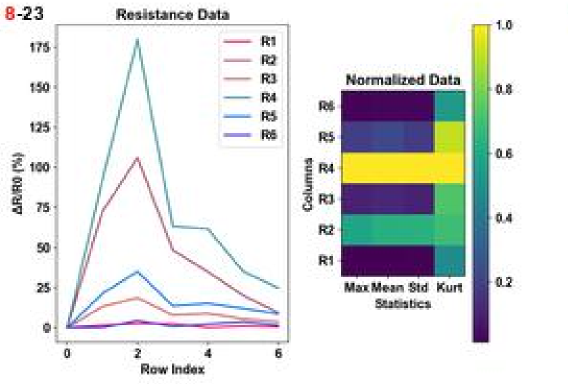

8-24
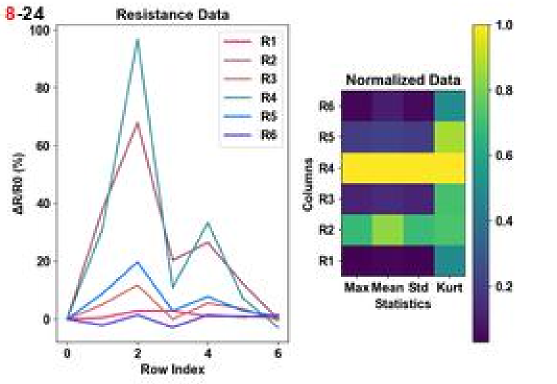

8-25
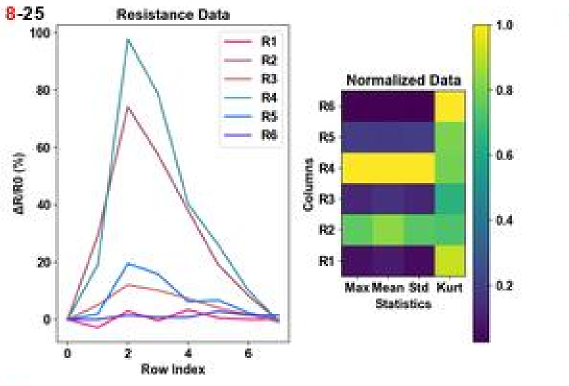

8-26
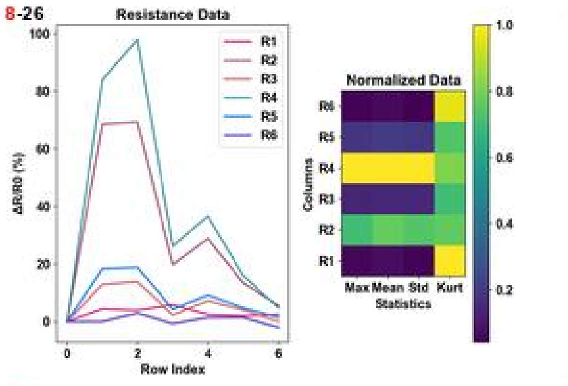

8-27
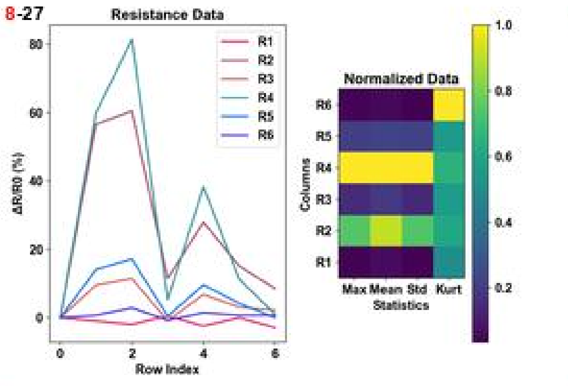

8-28
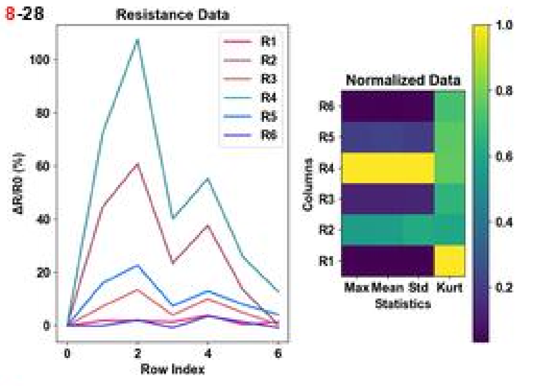

8-29
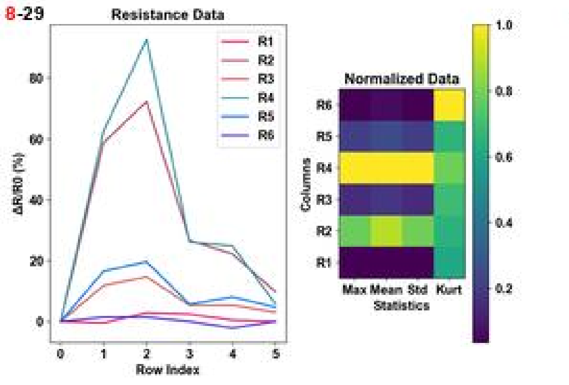

8-30
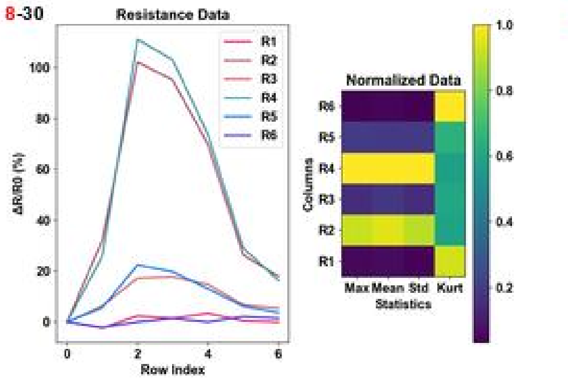

8-31
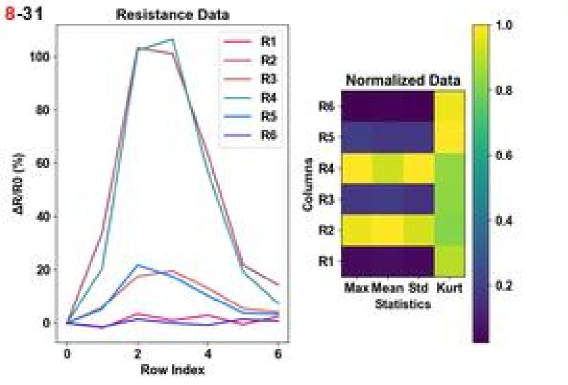

8-32
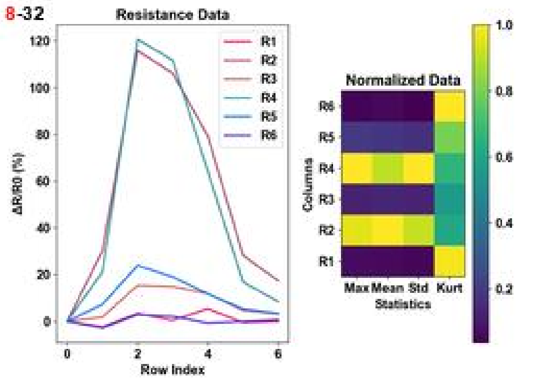

8-33
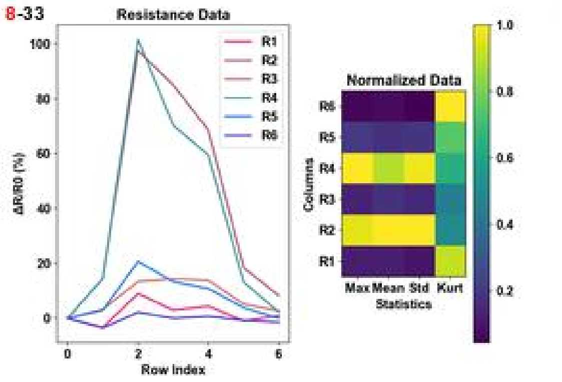

8-34
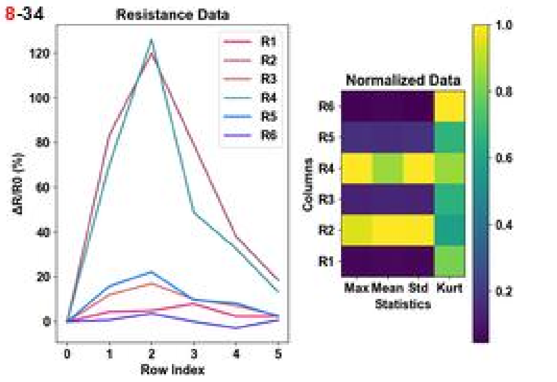

8-35
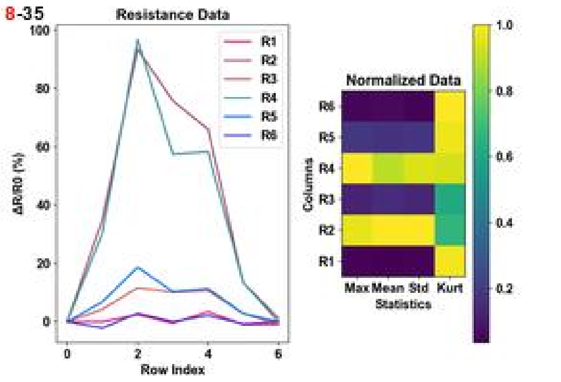

8-36
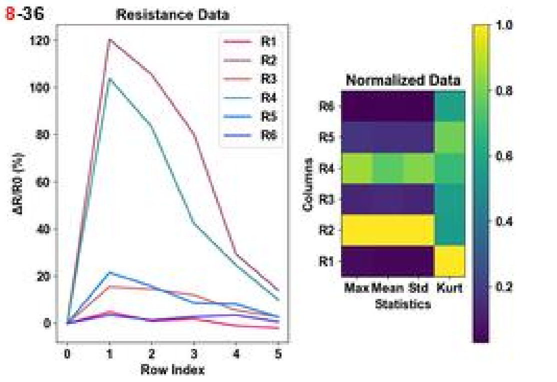

8-37
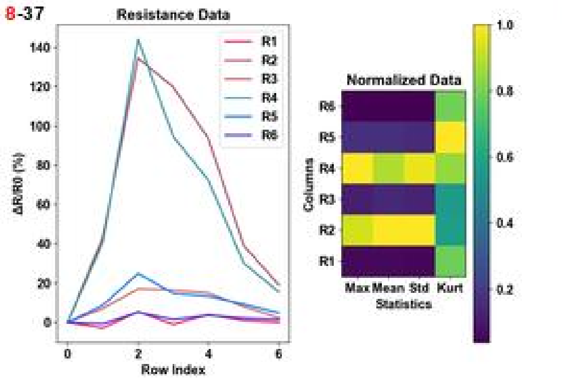

8-38
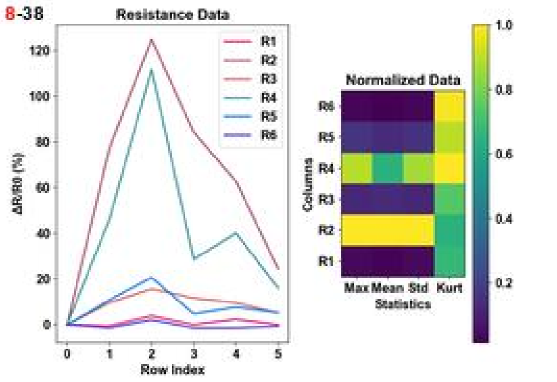

8-39
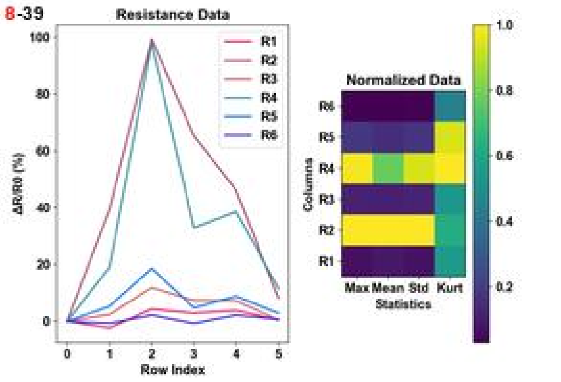

8-40
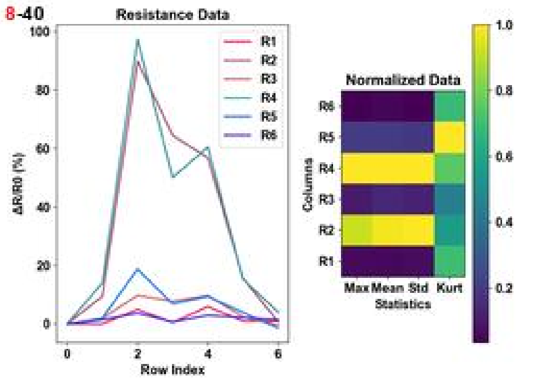

8-41
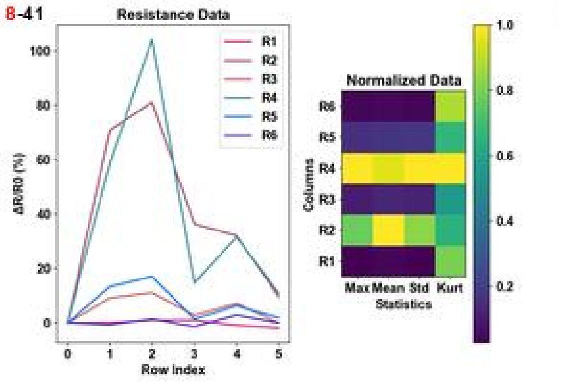

8-42
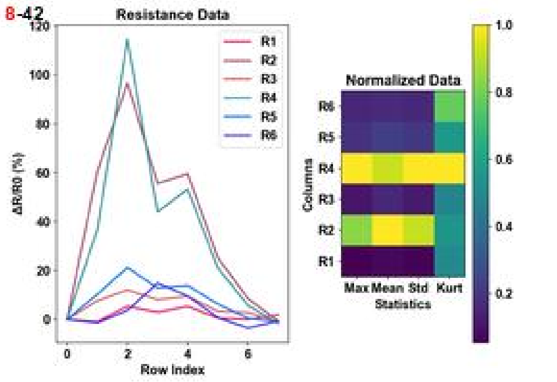

8-43
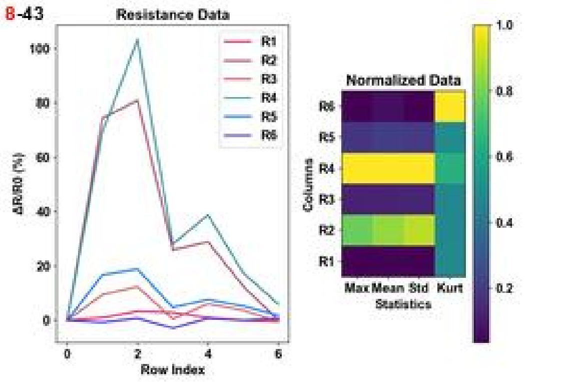

8-44
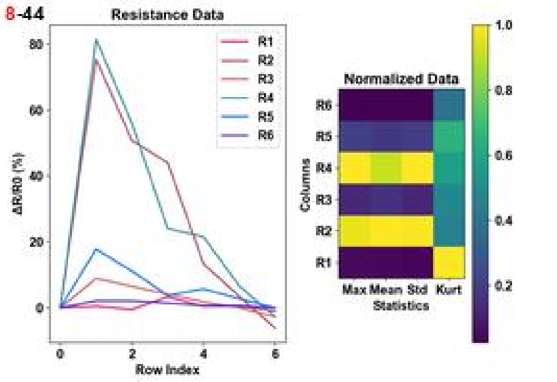

8-45
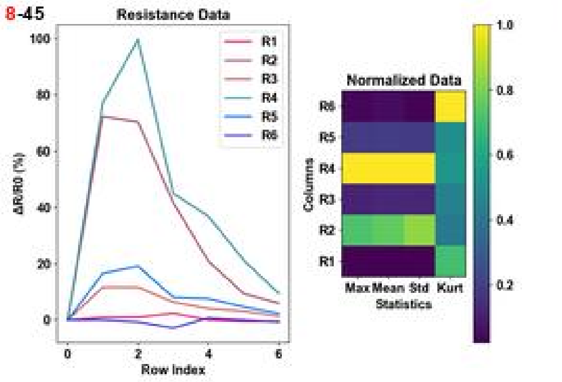

8-46
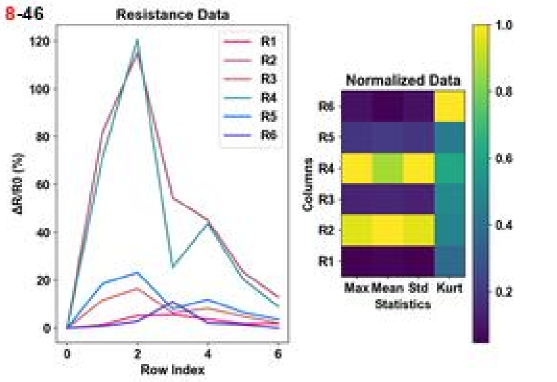

8-47
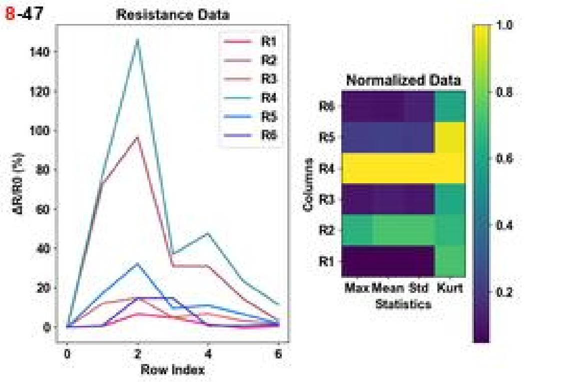

8-48
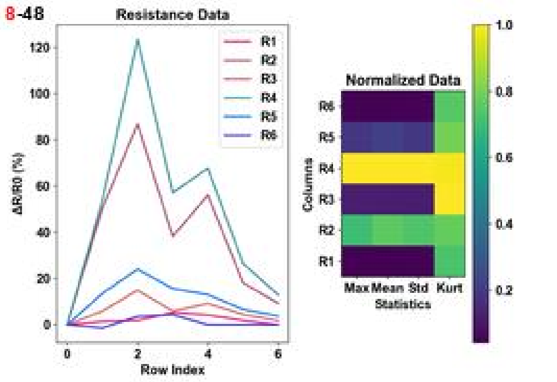

8-49
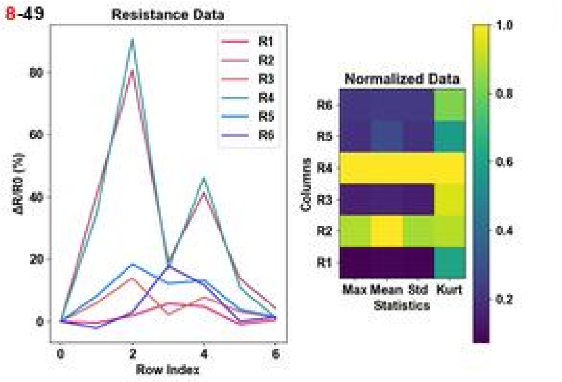

8-50
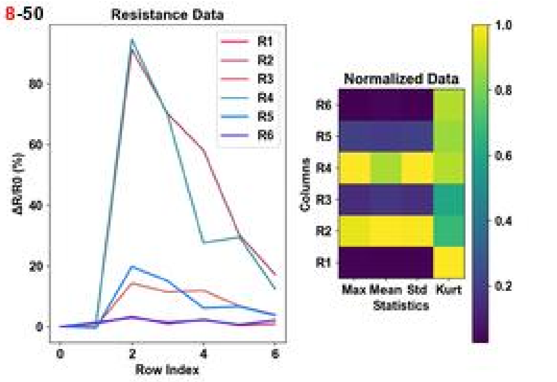

8-51
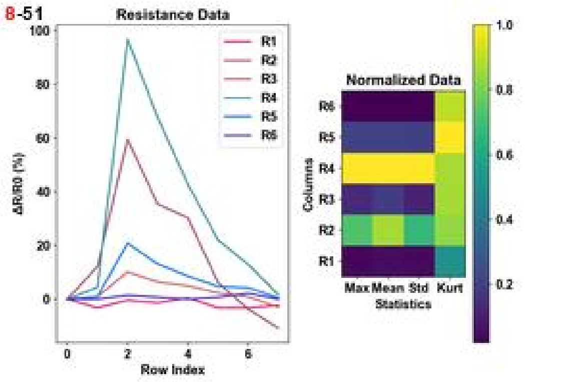

8-52
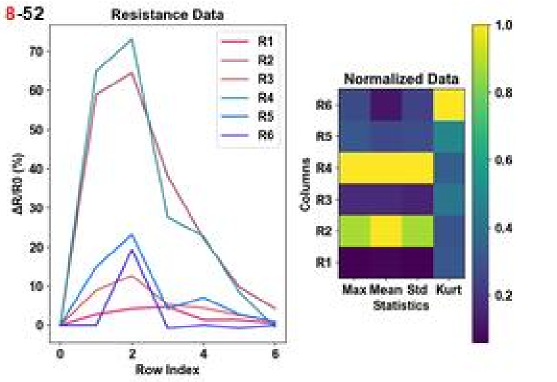

8-53
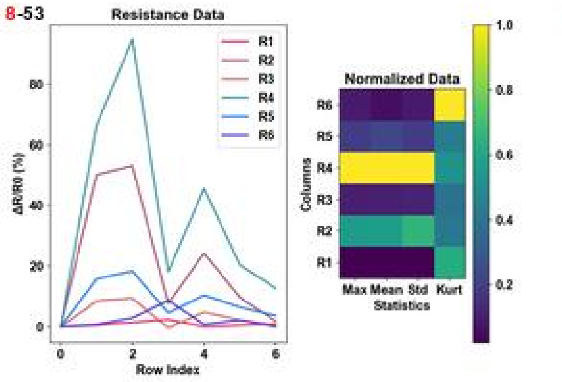

8-54
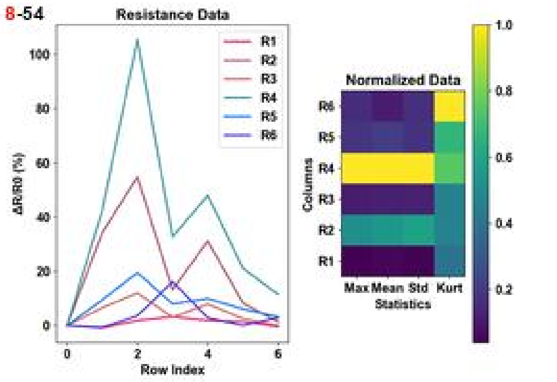

8-55
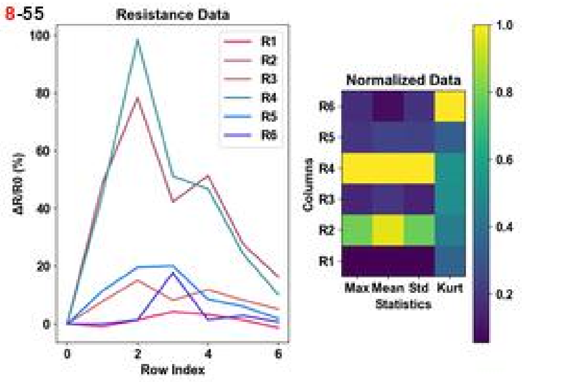

8-56
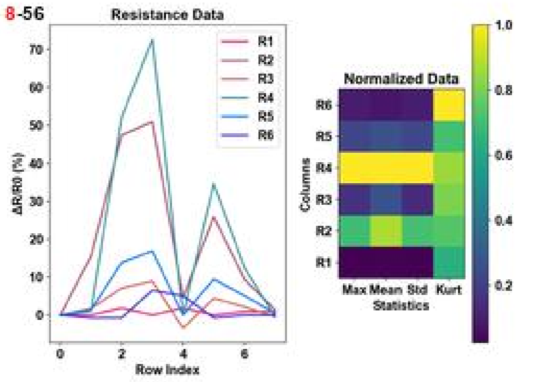

8-57
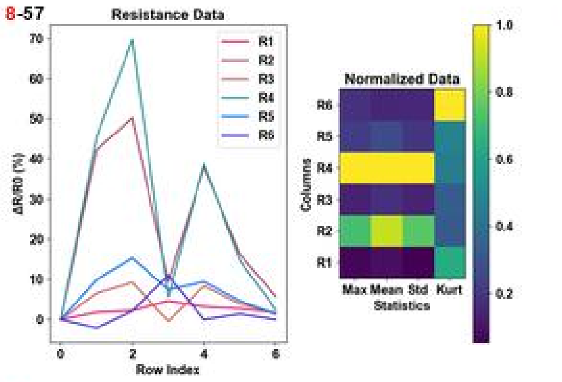

8-58
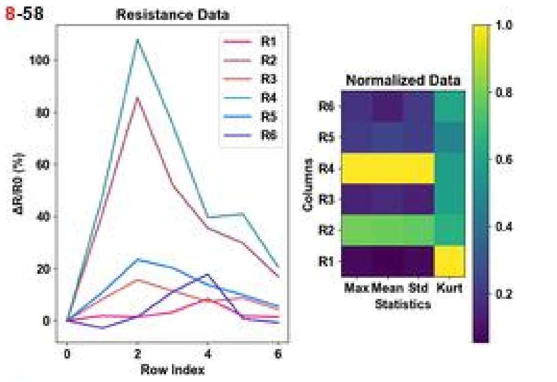

8-59
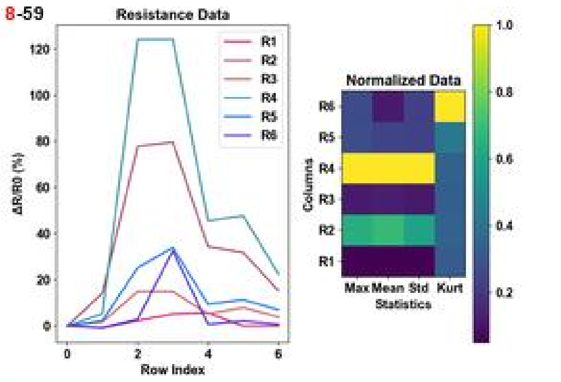

8-60
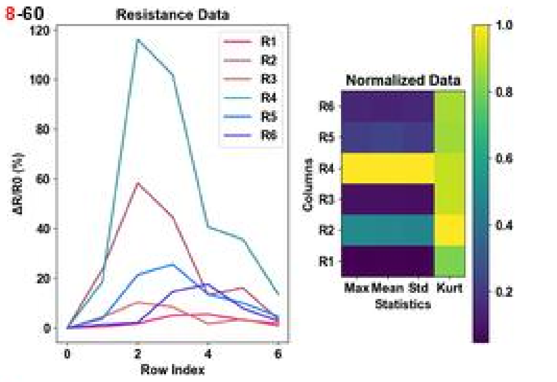

8-61
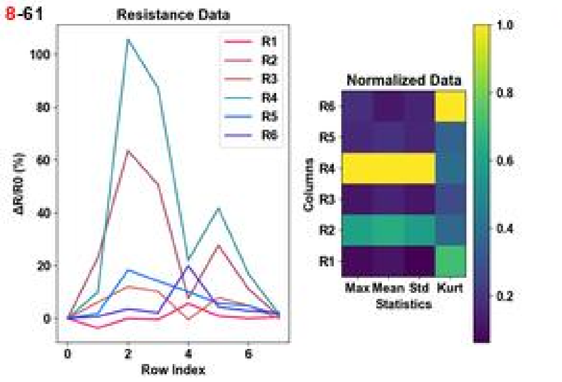

8-62
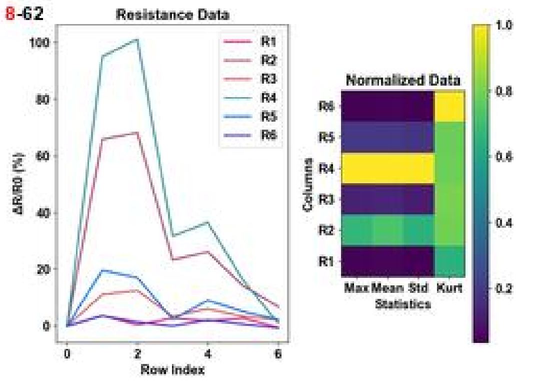

8-63
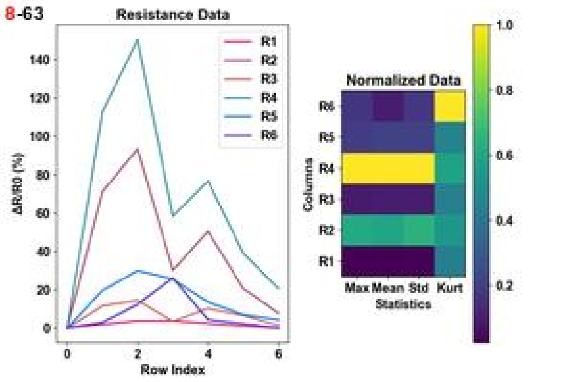

8-64
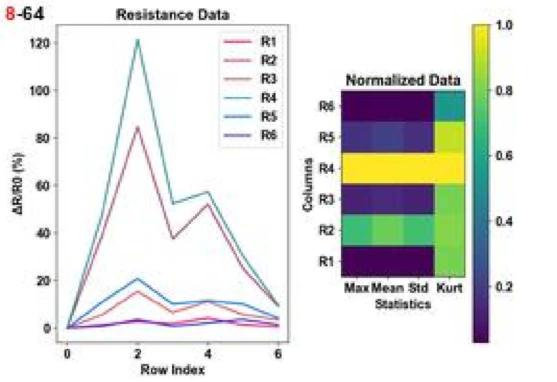

8-65
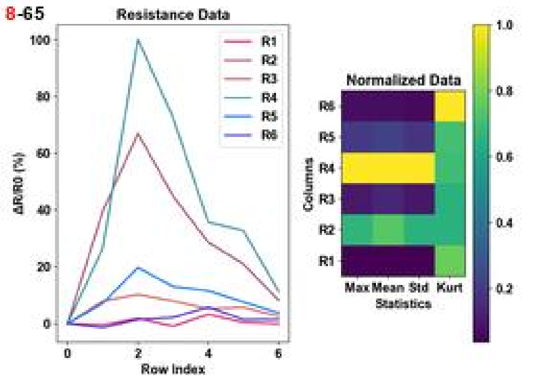

8-66
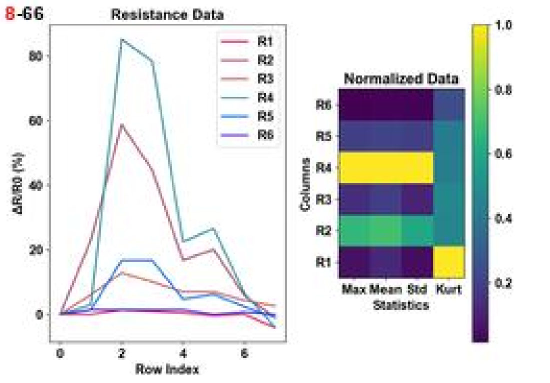

8-67
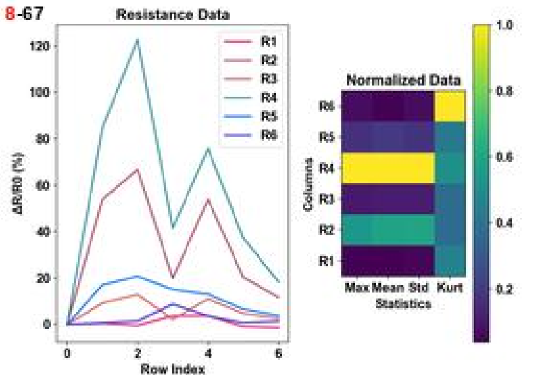

8-68
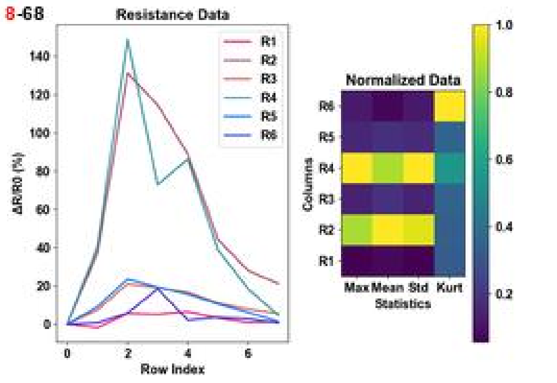

8-69
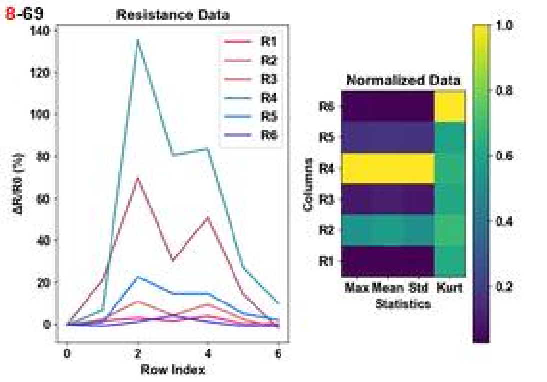

8-70
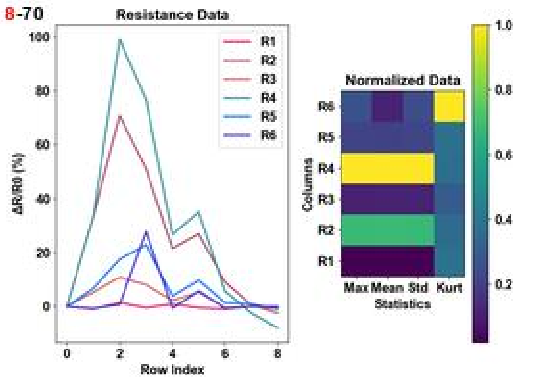

8-71
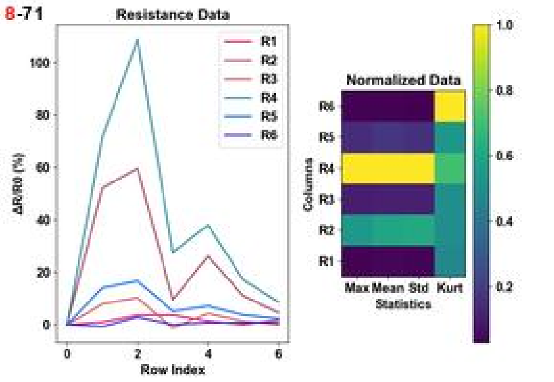

8-72
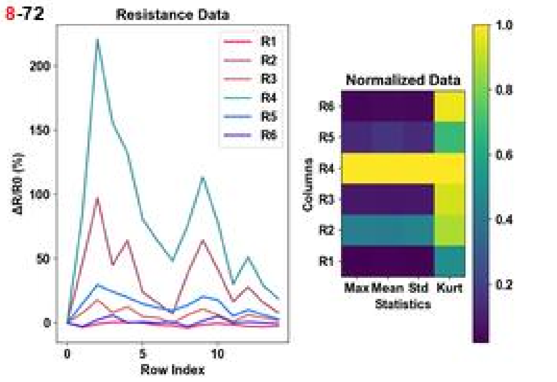

8-73
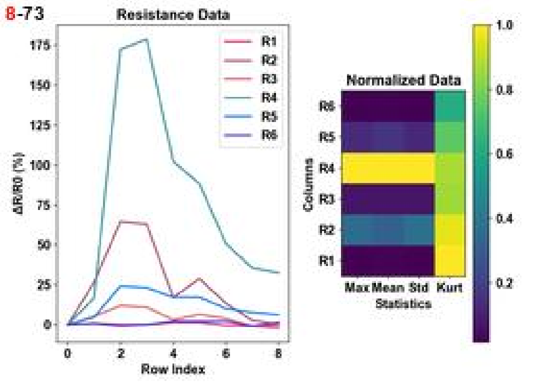

8-74
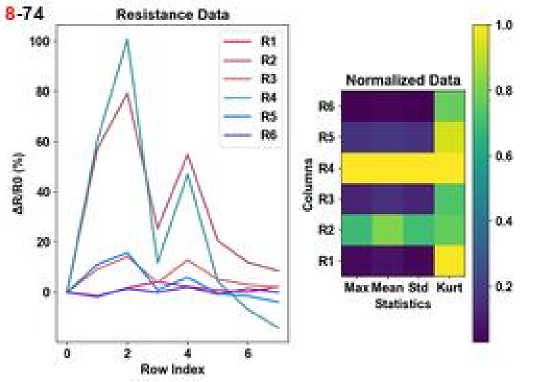

8-75
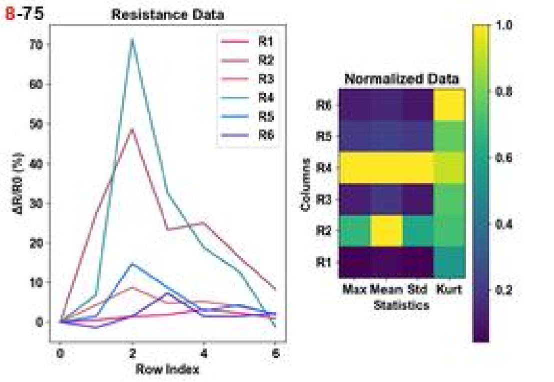

8-76
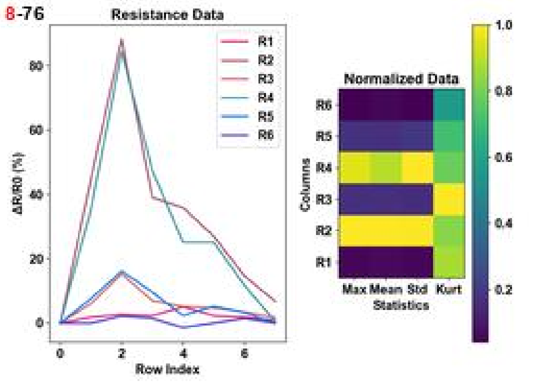

8-77
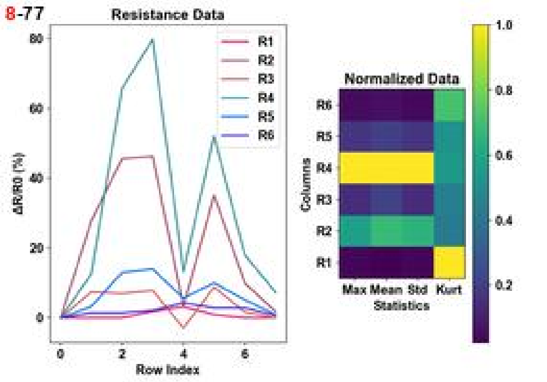

8-78
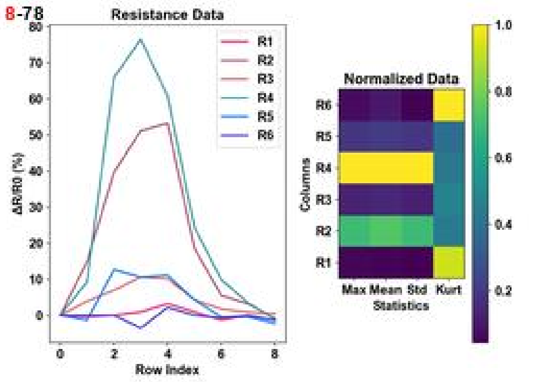

8-79
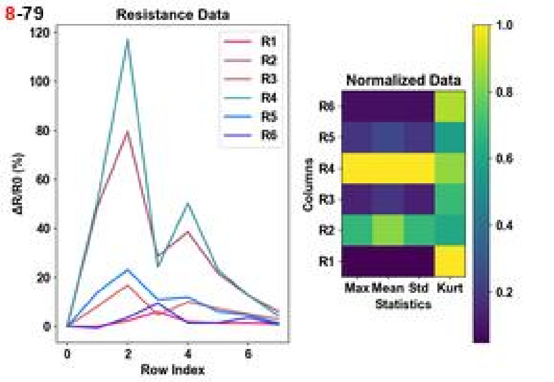

8-80
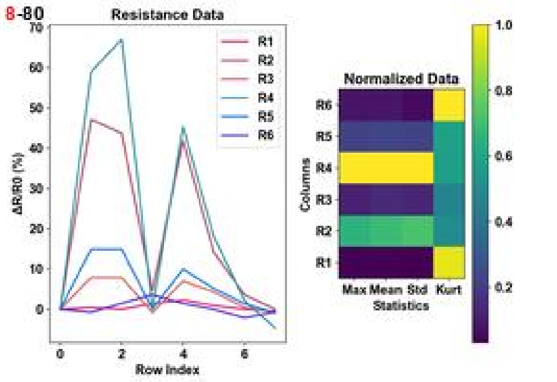

8-81
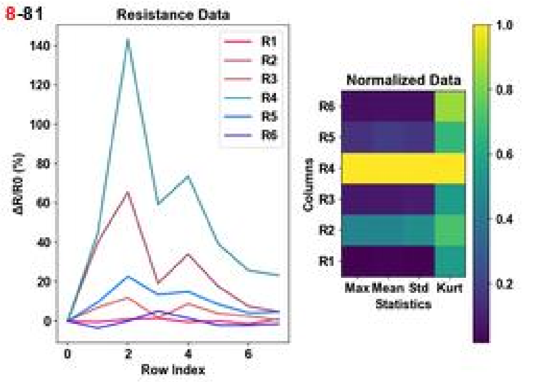

8-82
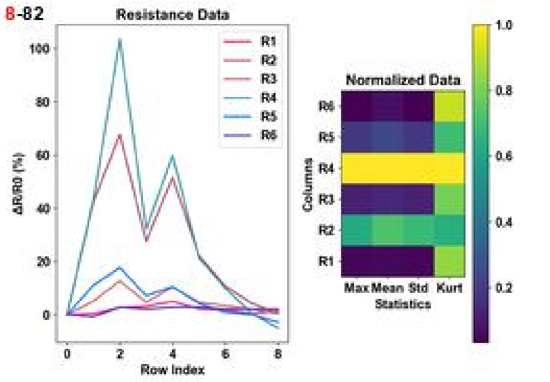

8-83
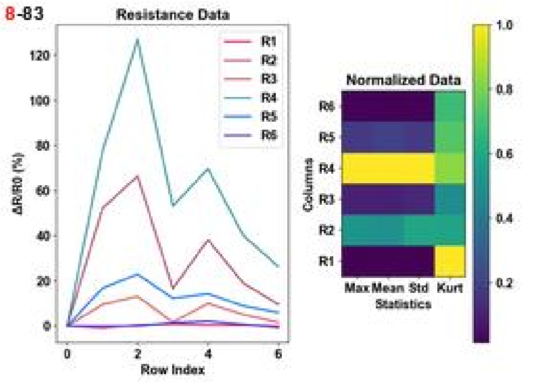

8-84
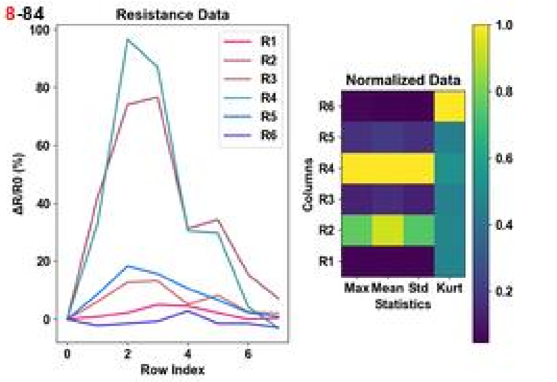

8-85
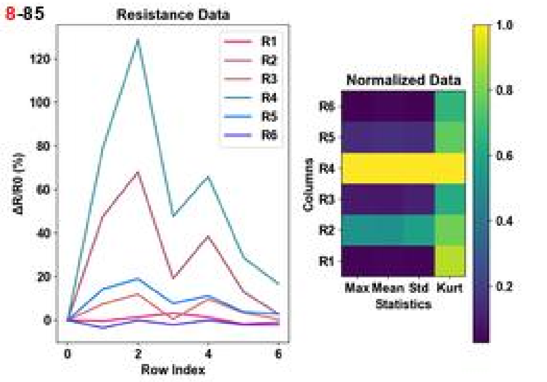

8-86
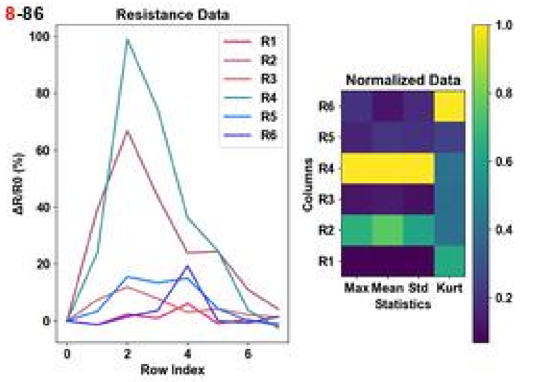

8-87
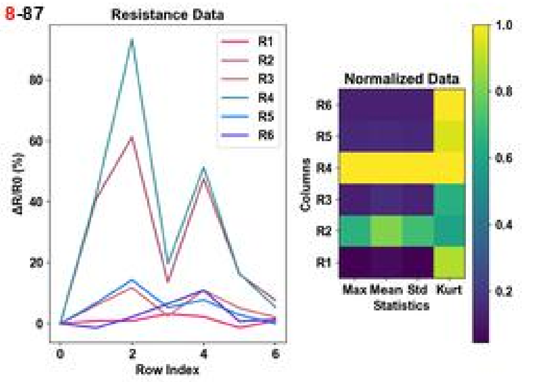

8-88
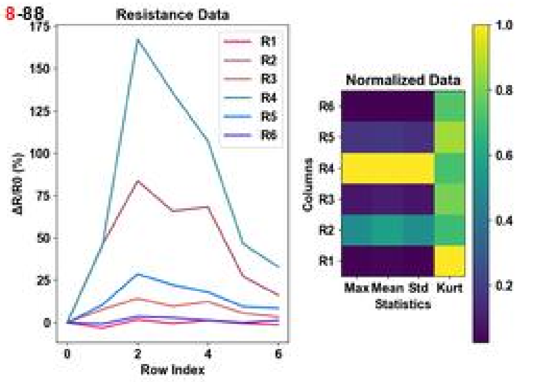

8-89
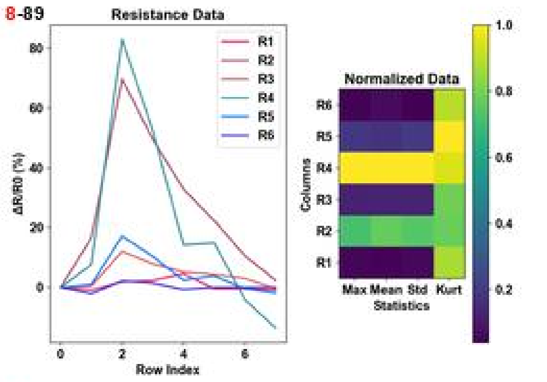

8-90
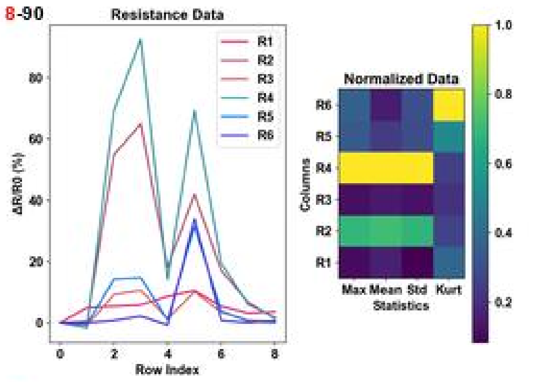

8-91
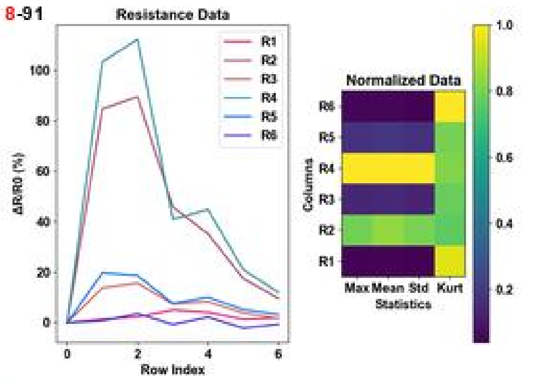

8-92
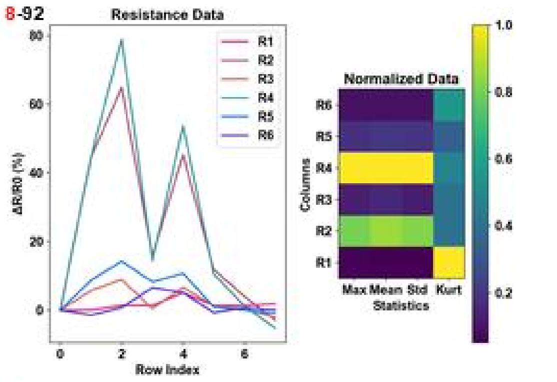

8-93
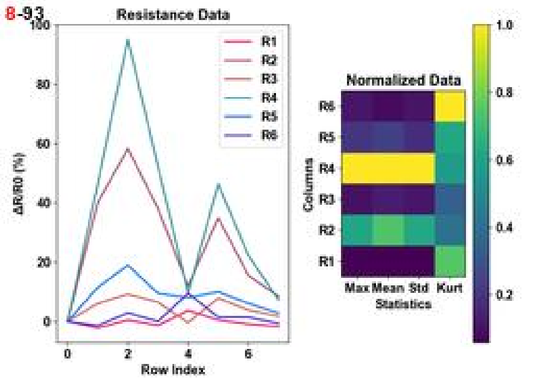

8-94
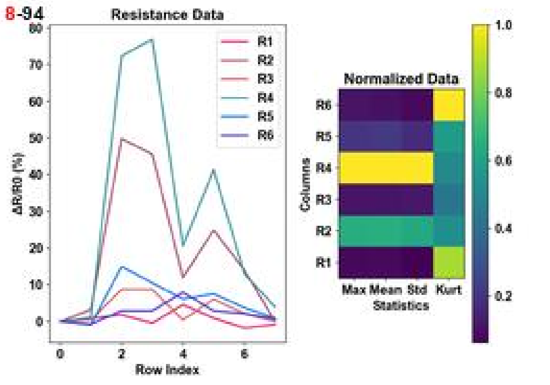

8-95
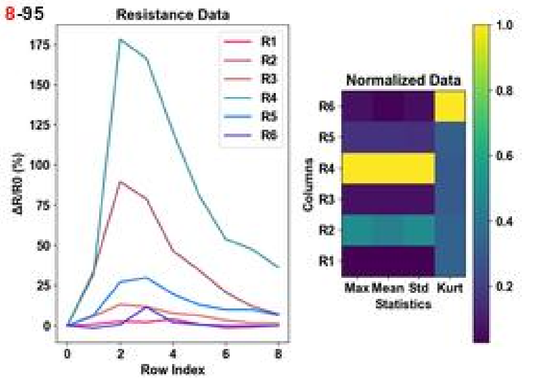

8-96
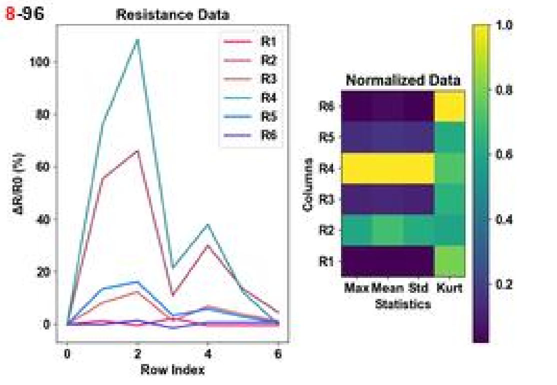

Pressure-sensitive data of type 8

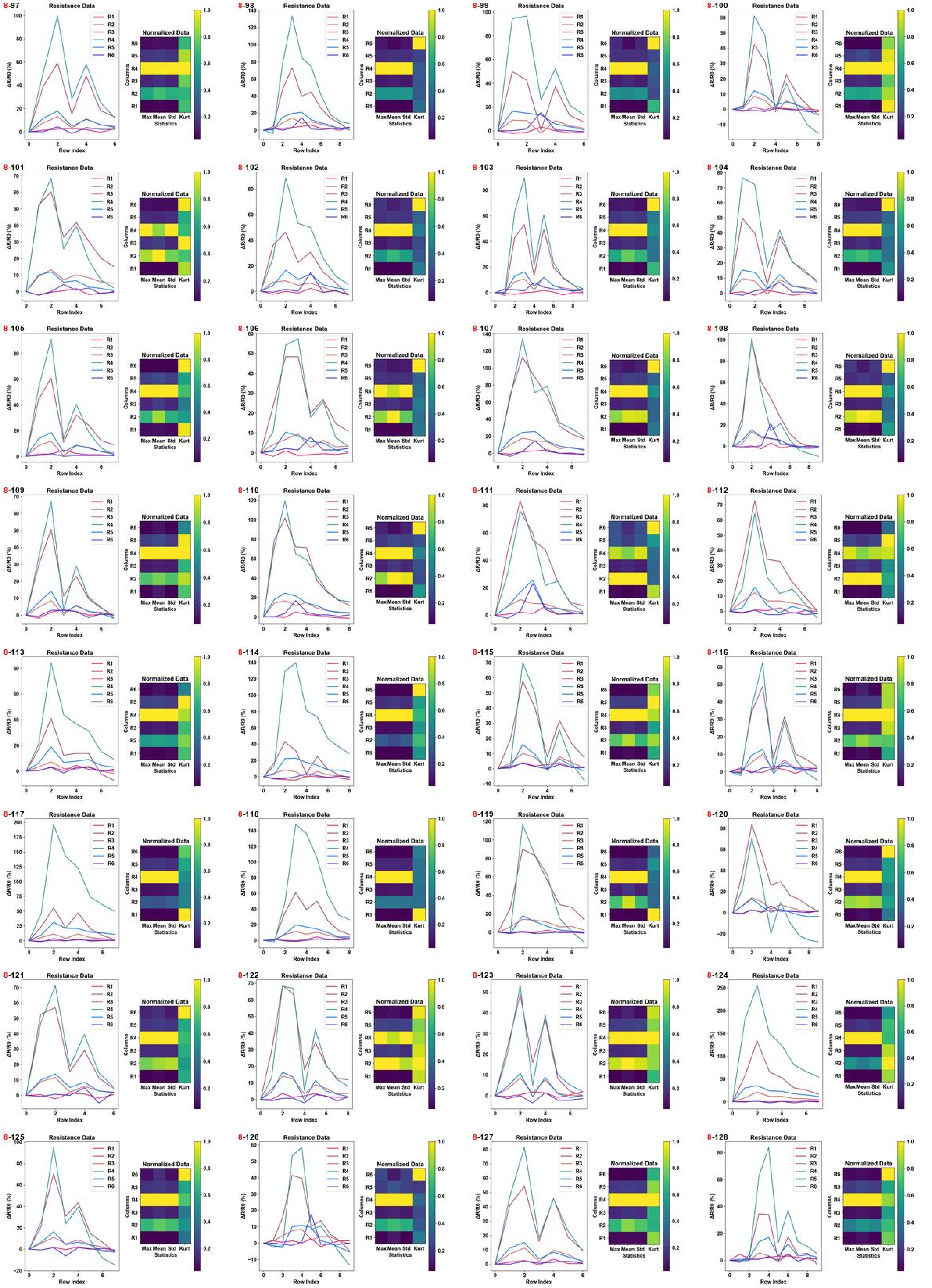

8-129
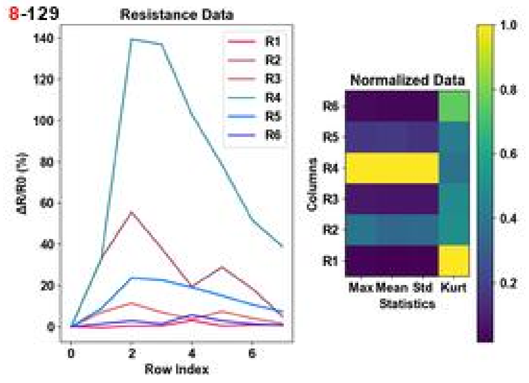

8-130
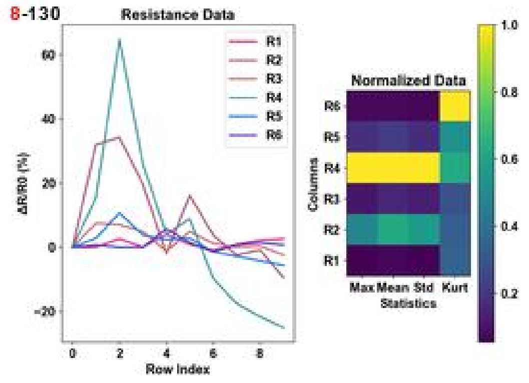

8-131
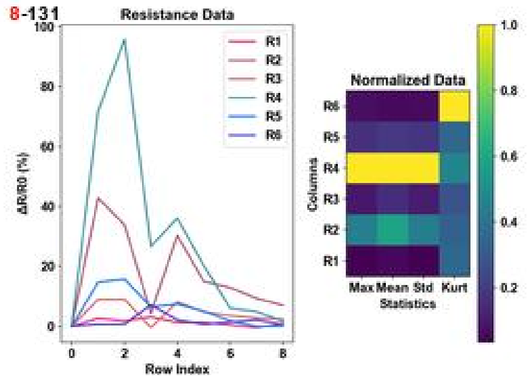

8-132
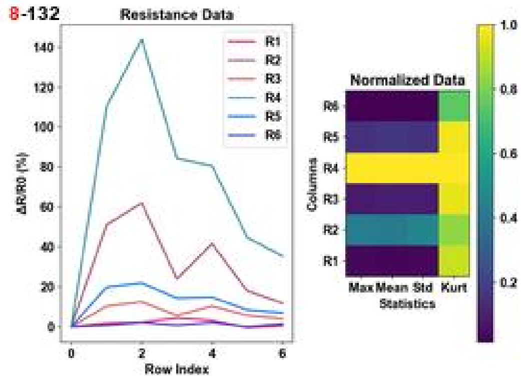

8-133
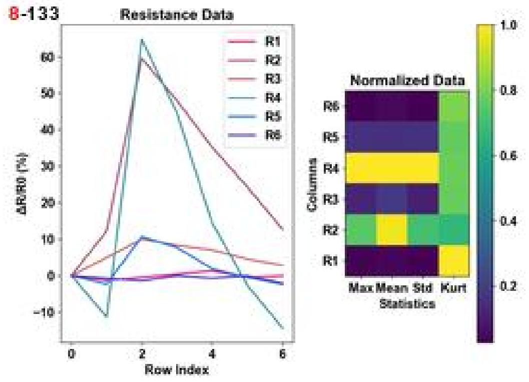

8-134
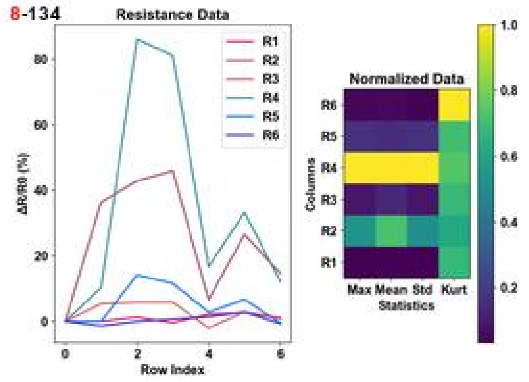

8-135
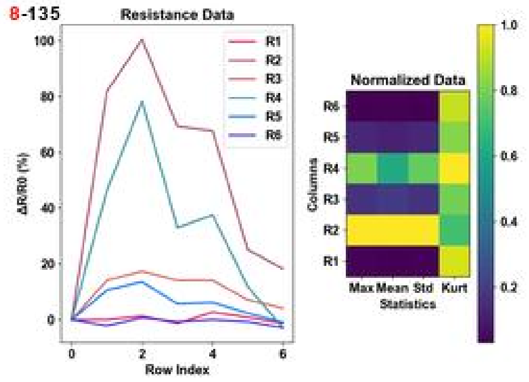

8-136
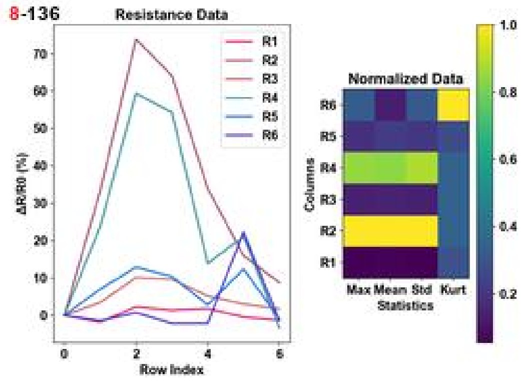

8-137
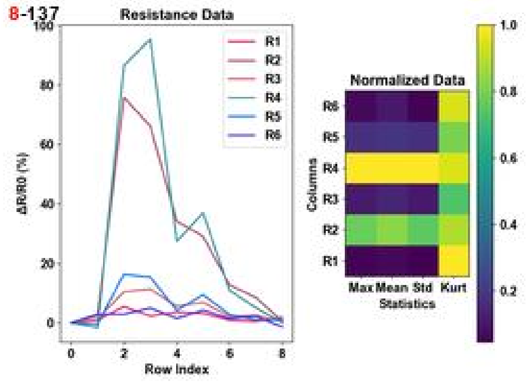

8-138
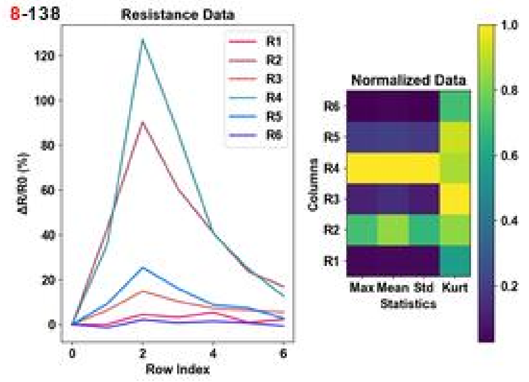

8-139
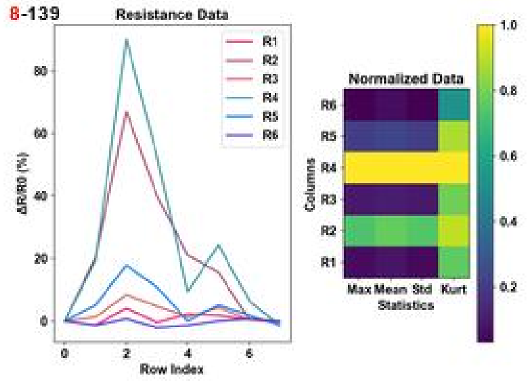

8-140
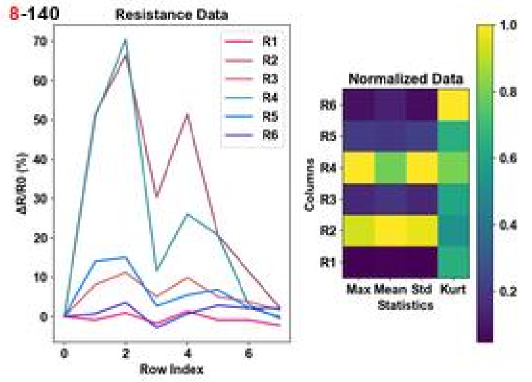

8-141
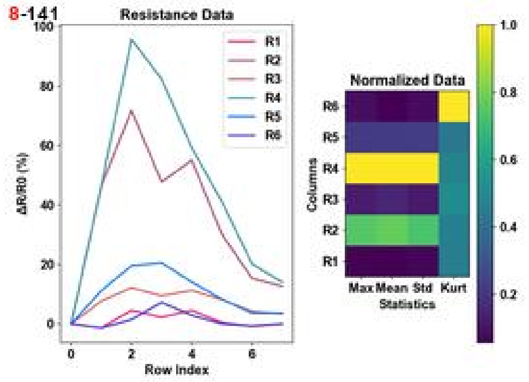

8-142
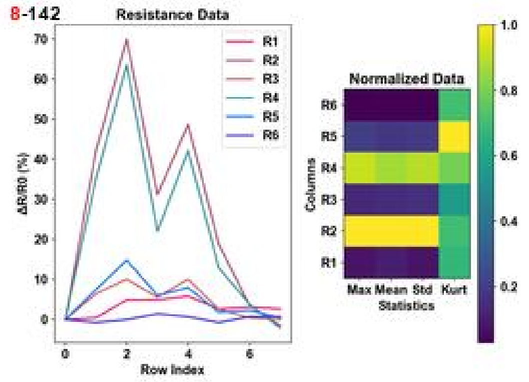

8-143
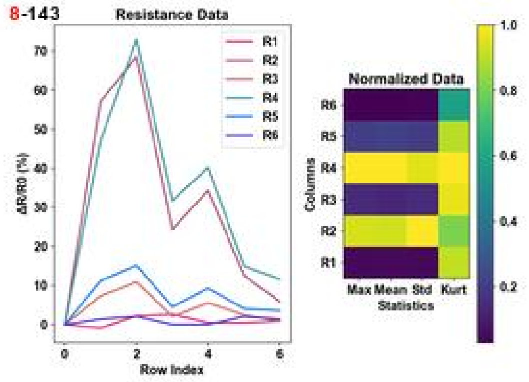

8-144
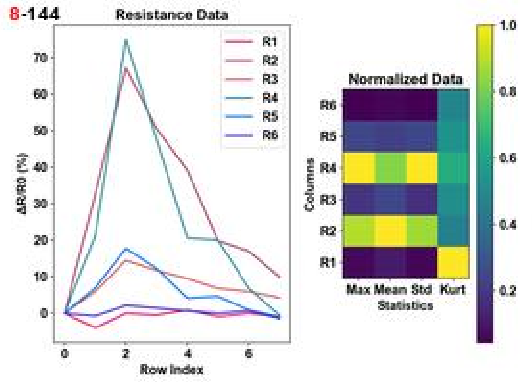

8-145
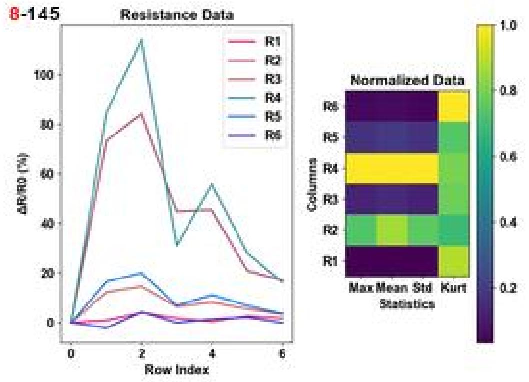

8-146
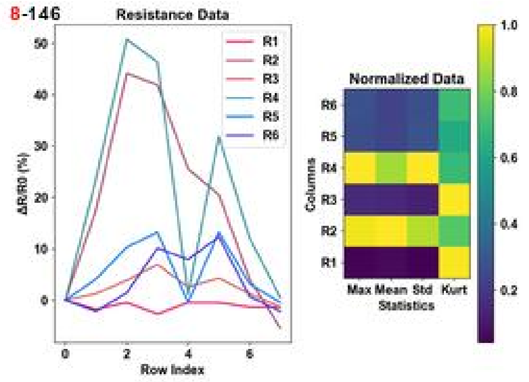

8-147
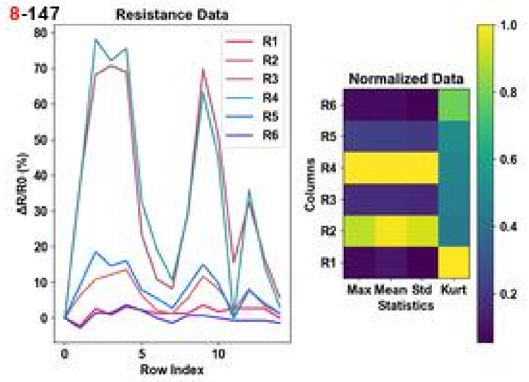

8-148
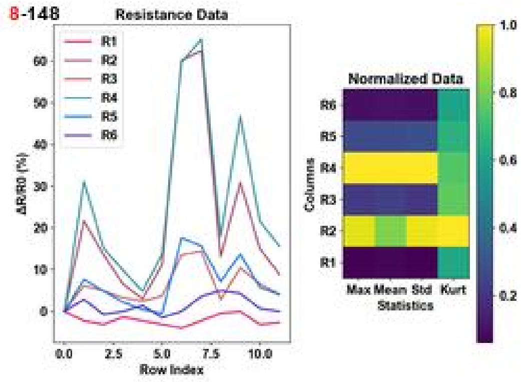

8-149
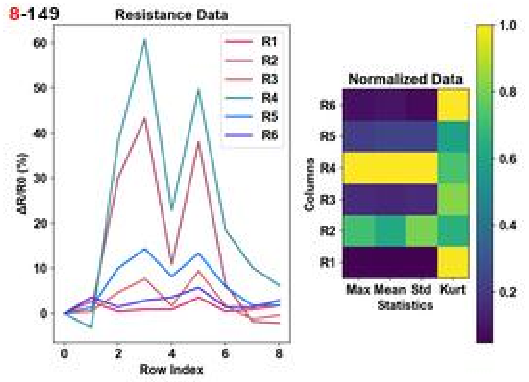

8-150
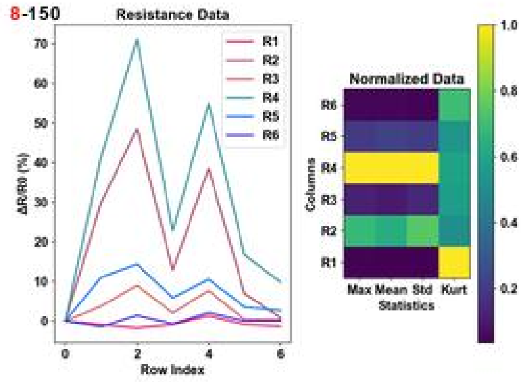

8-151
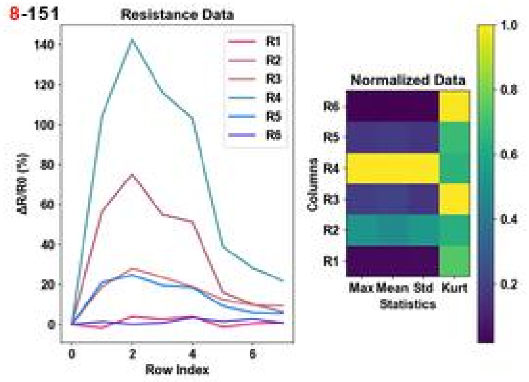

8-152
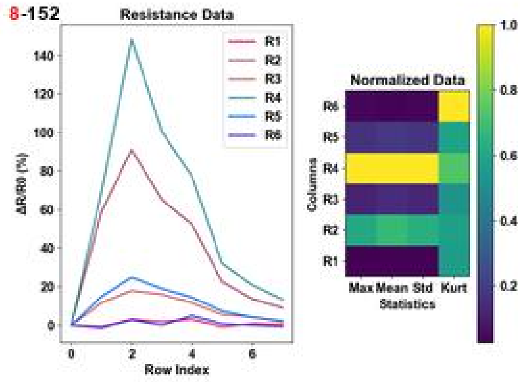

8-153
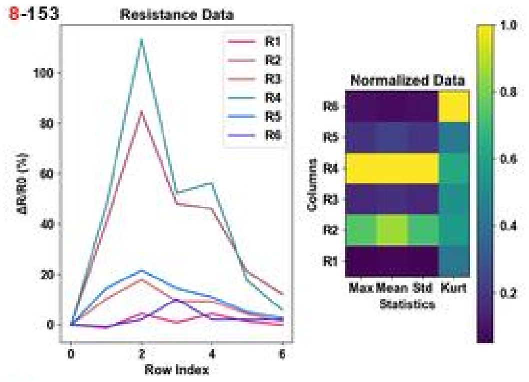

8-154
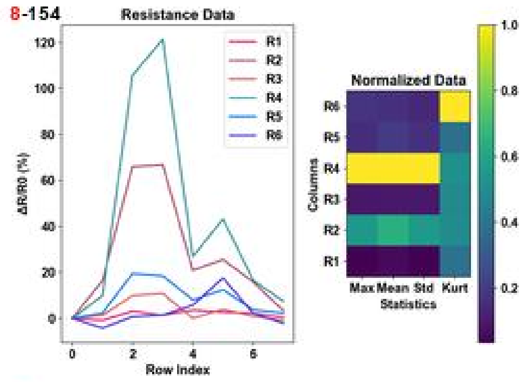

8-155
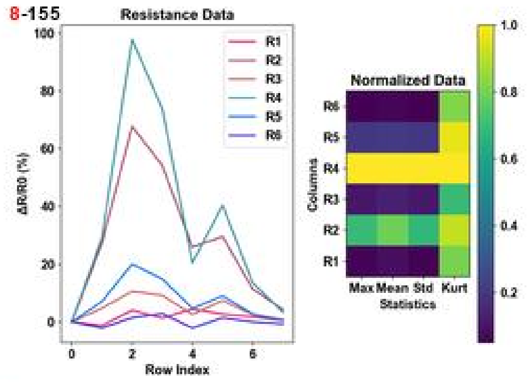

8-156
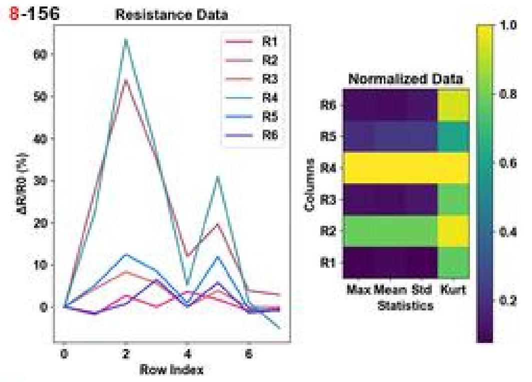

8-157
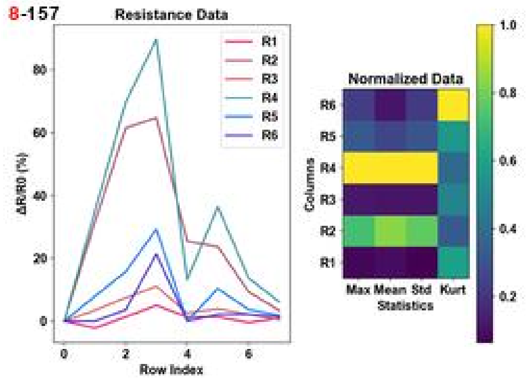

8-158
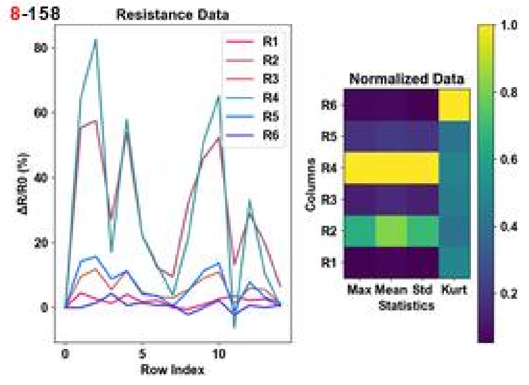

8-159
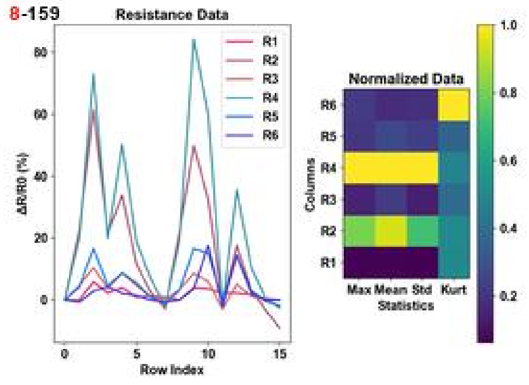

8-160
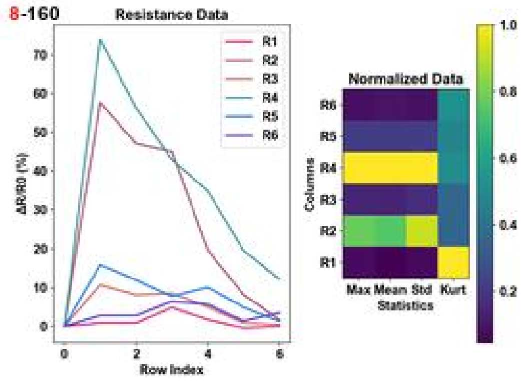

8-161
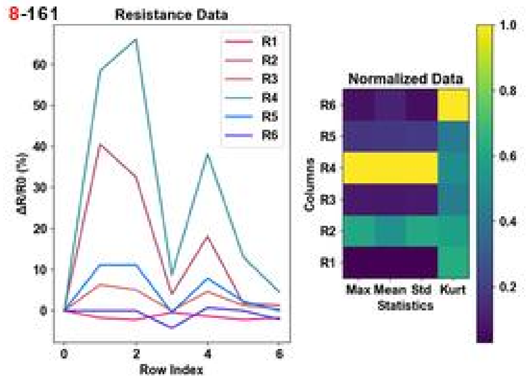

8-162
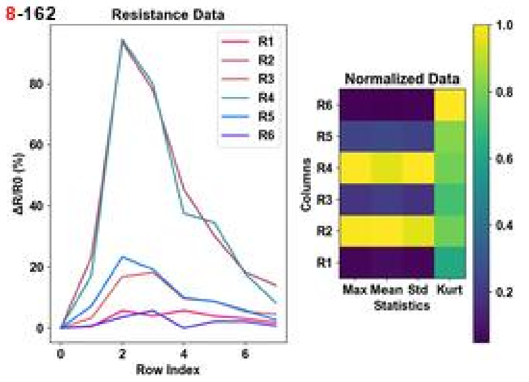

8-163
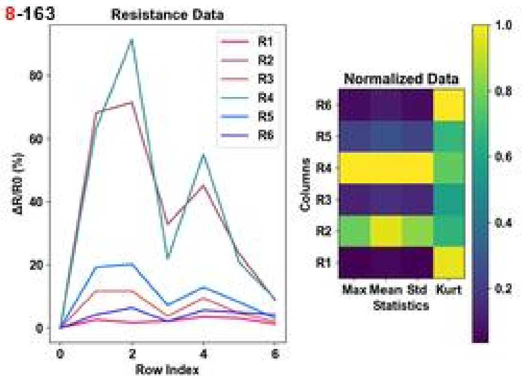

8-164
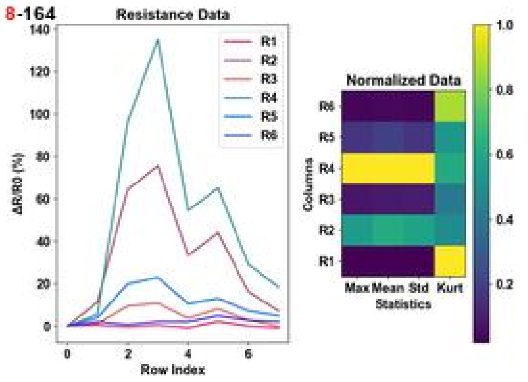

8-165
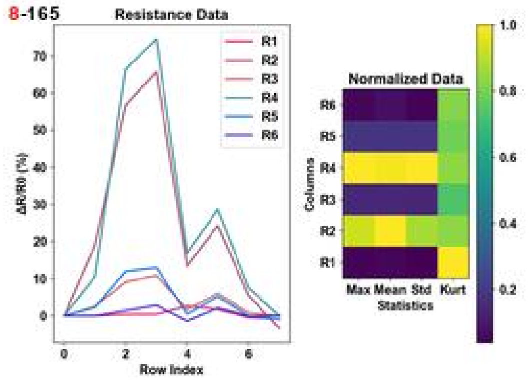

8-166
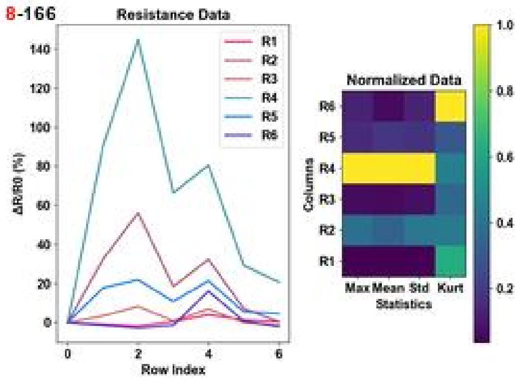

8-167
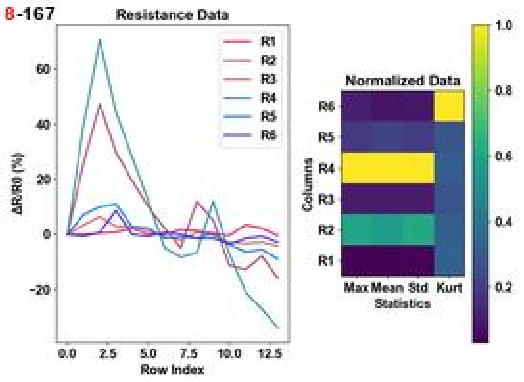

8-168
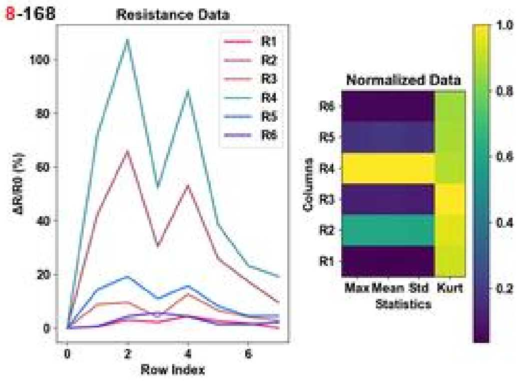

8-169
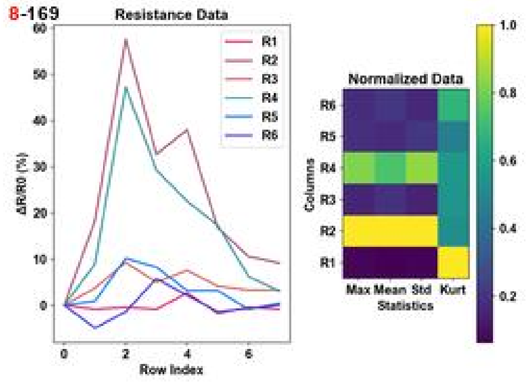

8-170
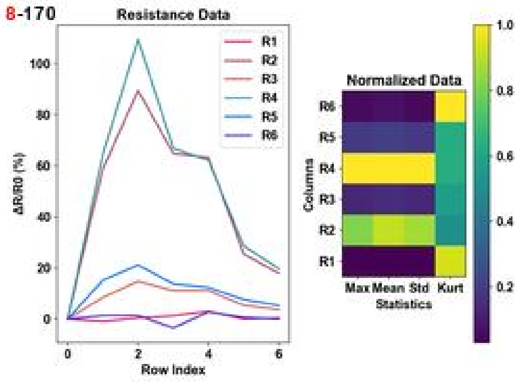

8-171
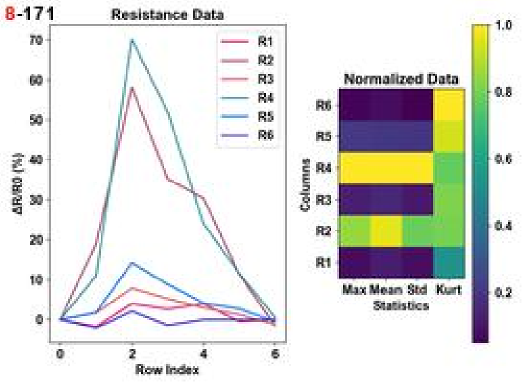

8-172
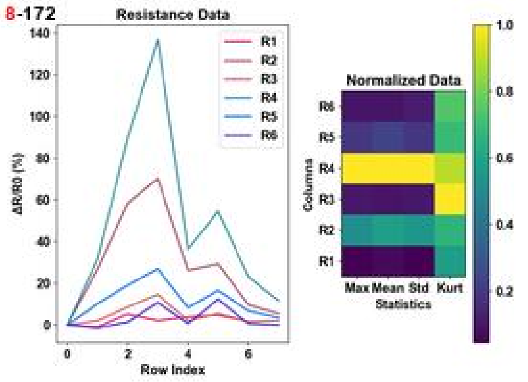

8-173
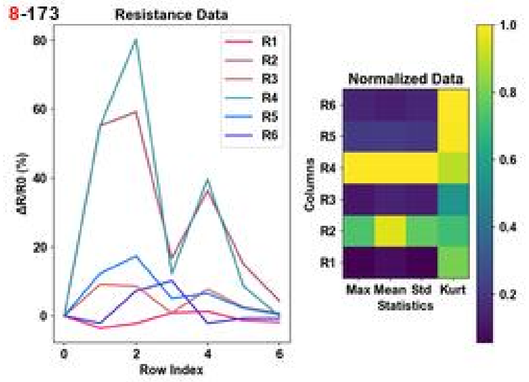

8-174
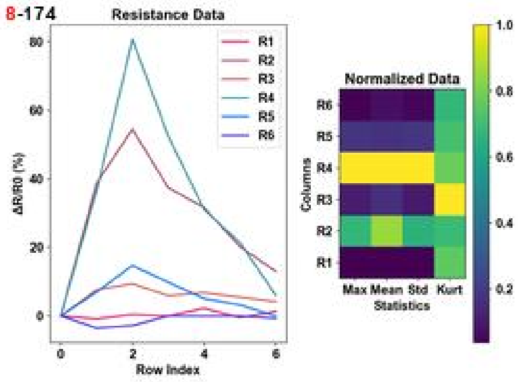

8-175
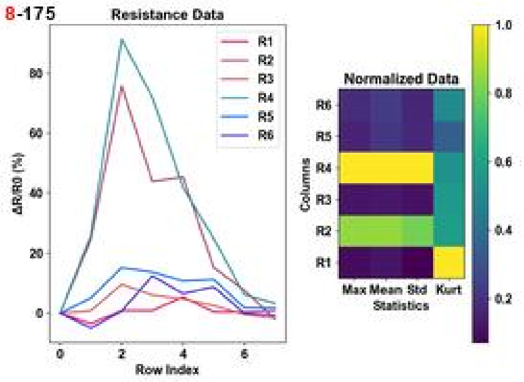

8-176
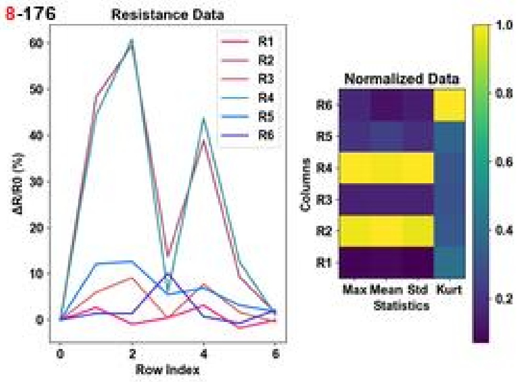

8-177
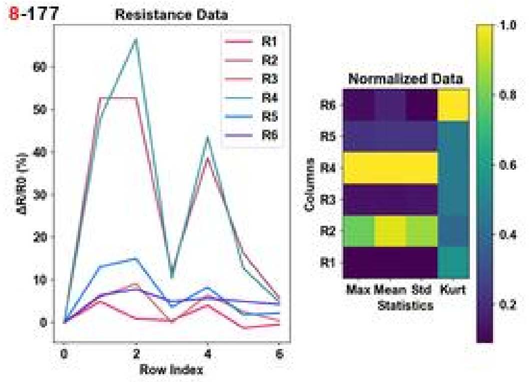

8-178
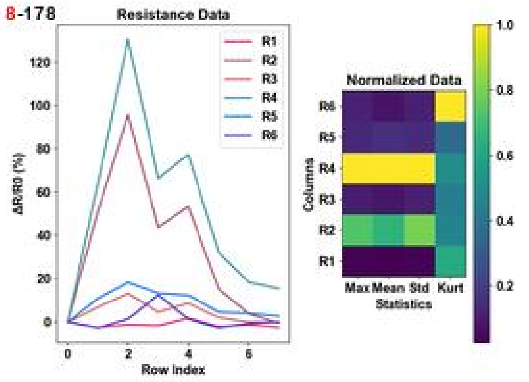

8-179
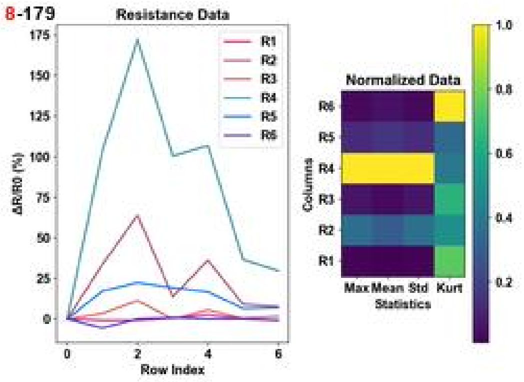

8-180
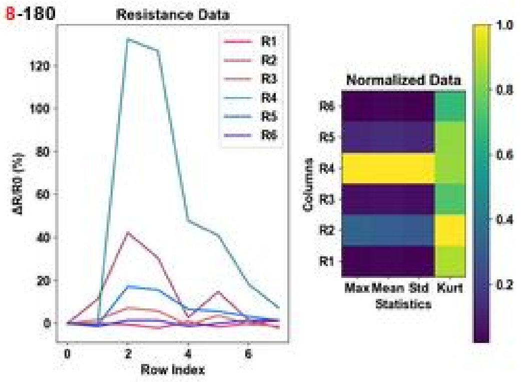

8-181
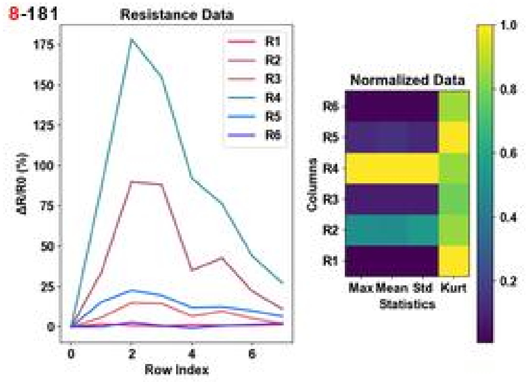

8-182
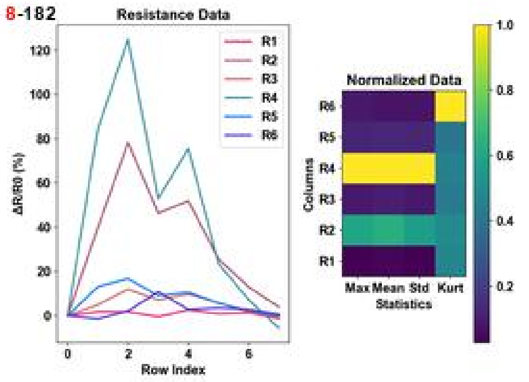

8-183
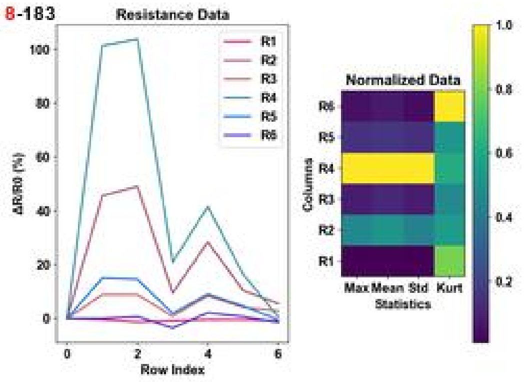

8-184
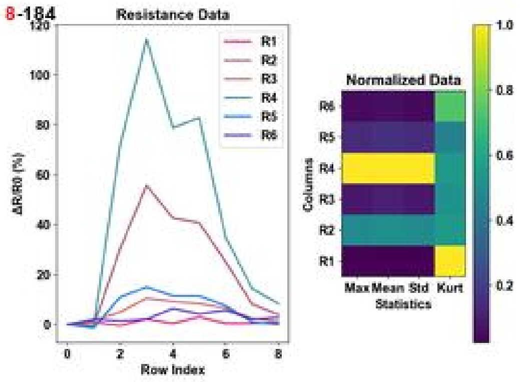

8-185
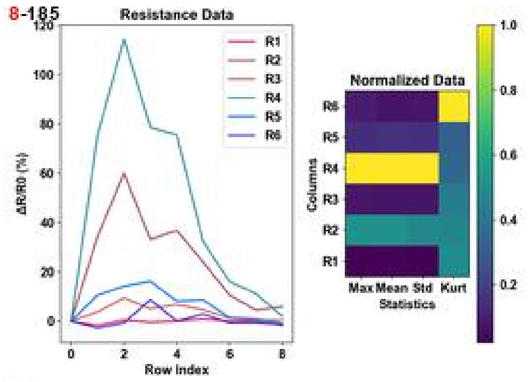

8-186
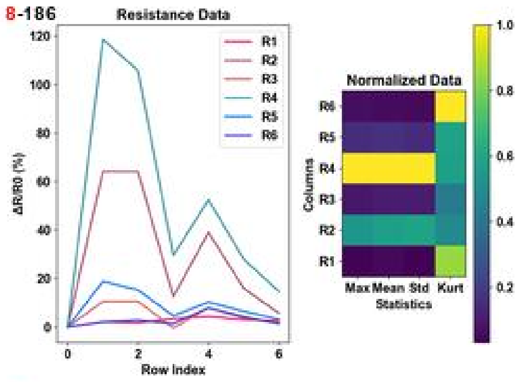

8-187
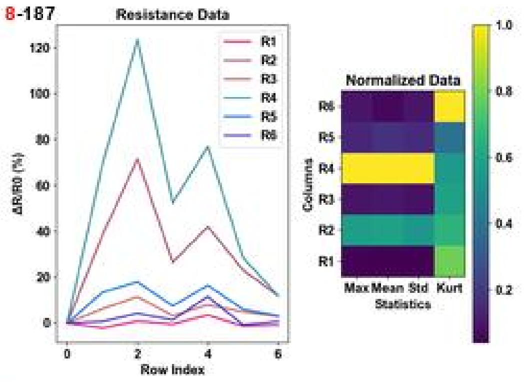

8-188
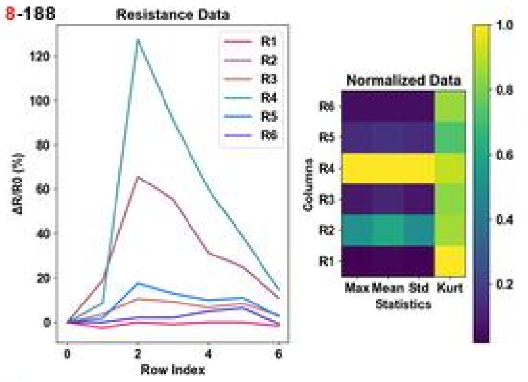

8-189
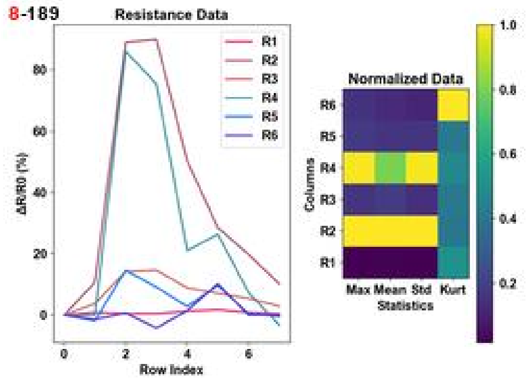

8-190
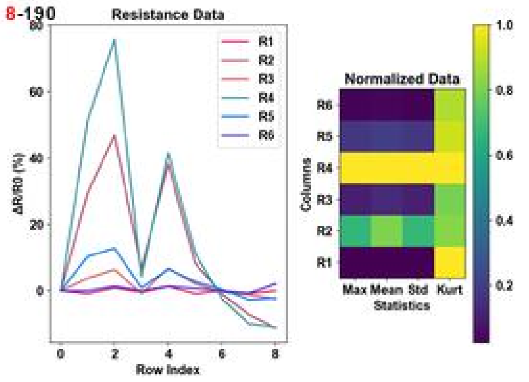

8-191
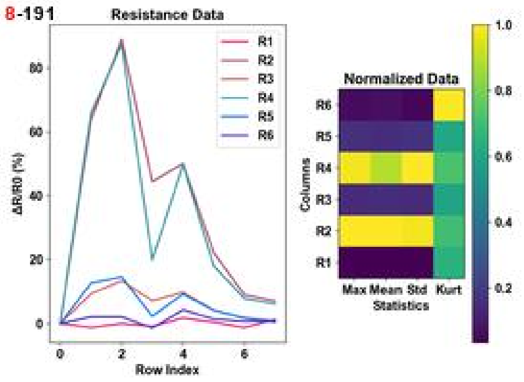

8-192
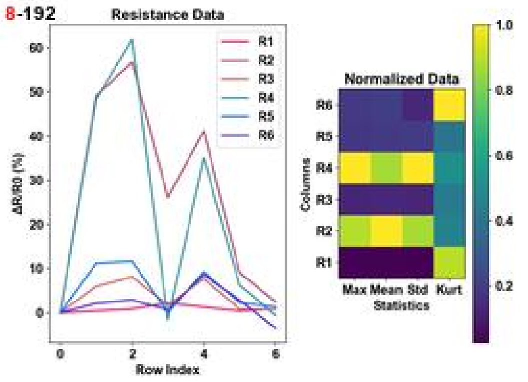

8-193

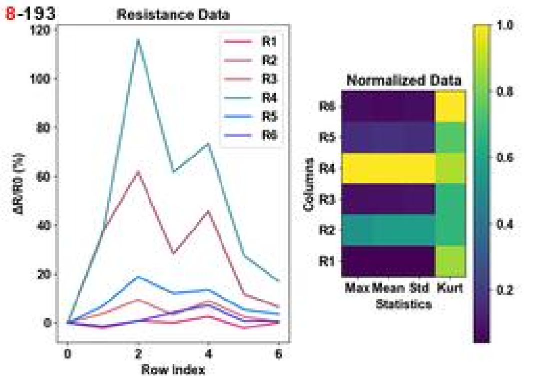

8-194

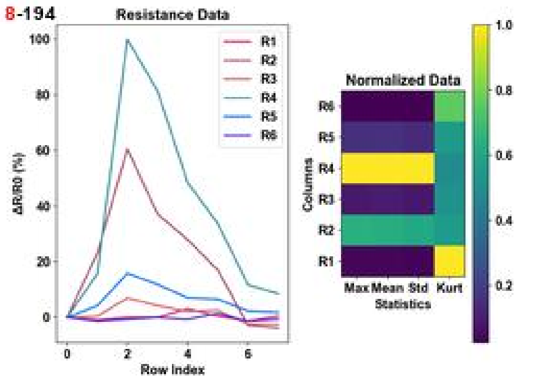

8-195

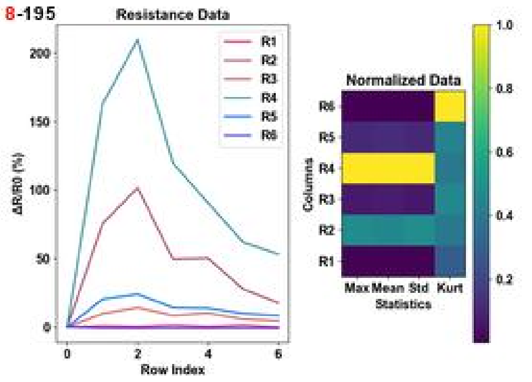

8-196

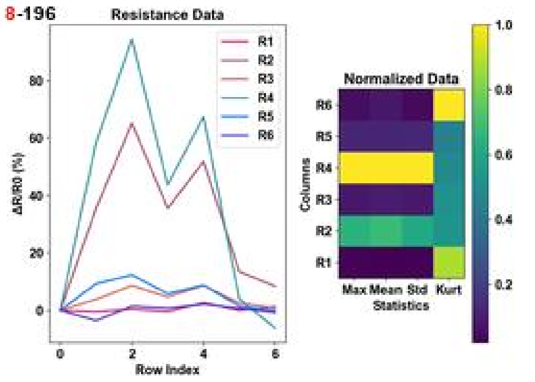

8-197

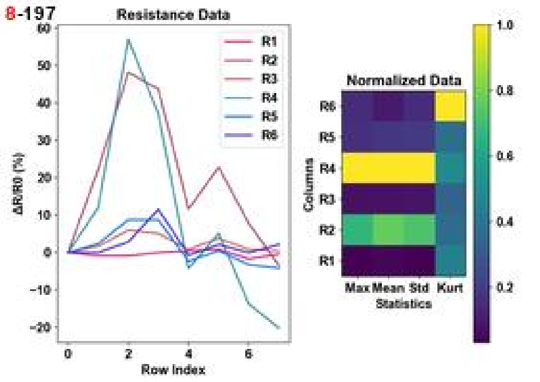

8-198

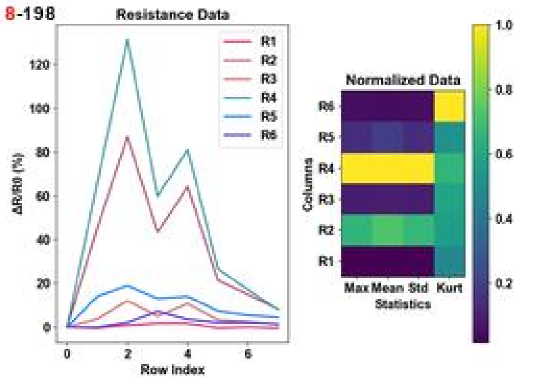

8-199

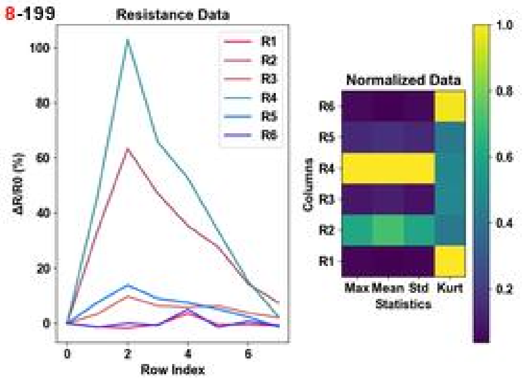

8-200

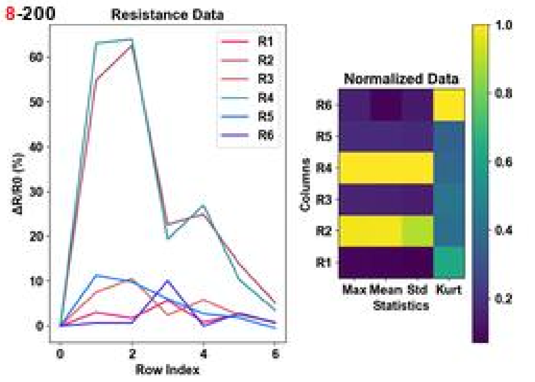

8-201

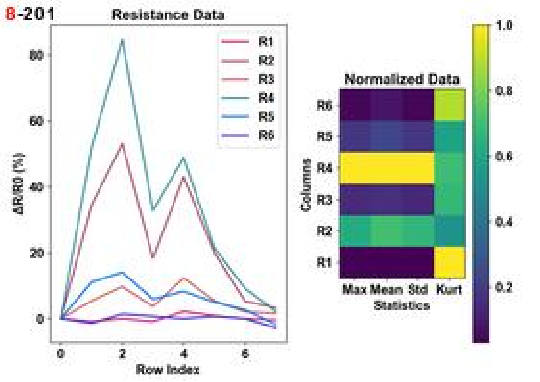

8-202

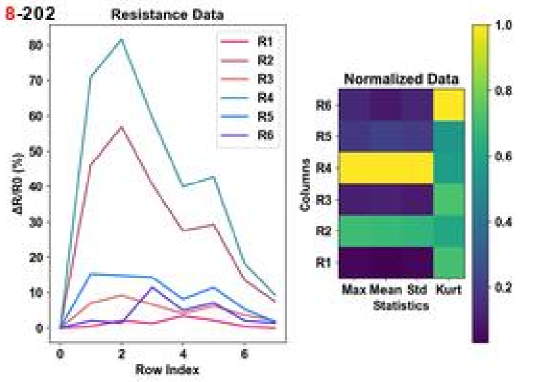

8-203

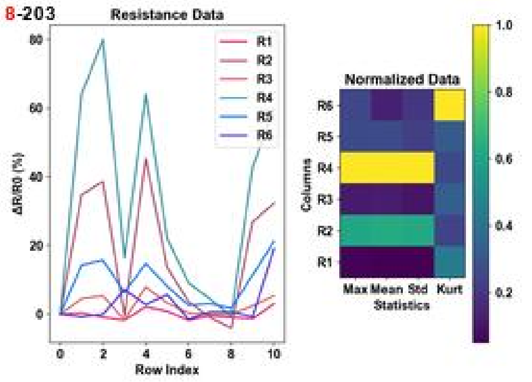

8-204

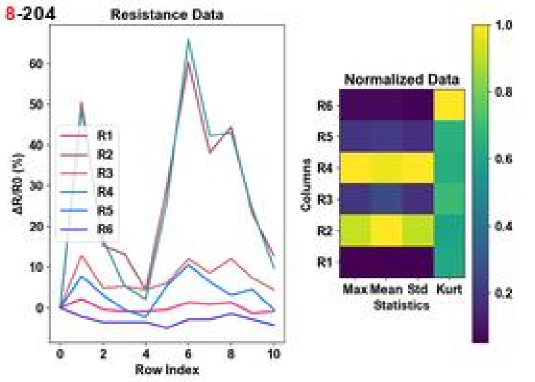

8-205

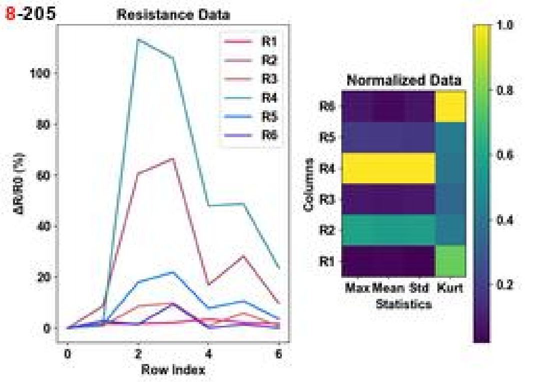

8-206

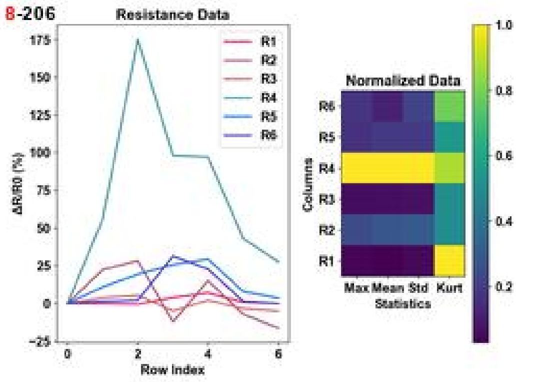

8-207

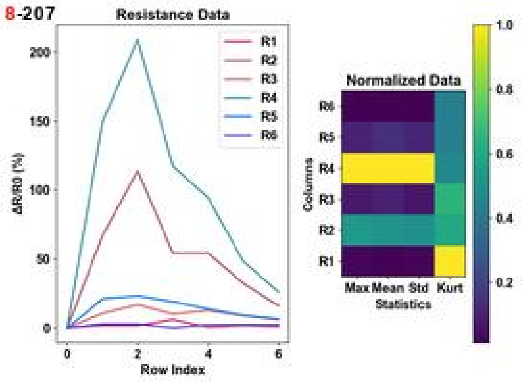

8-208

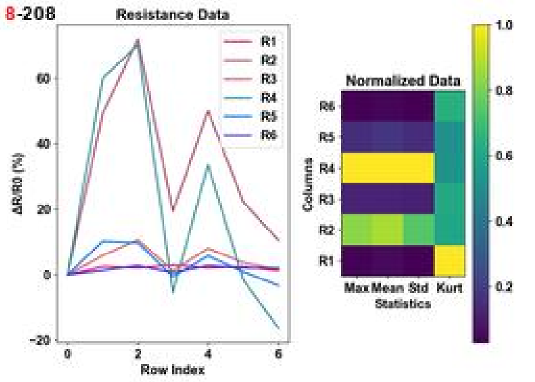

8-209

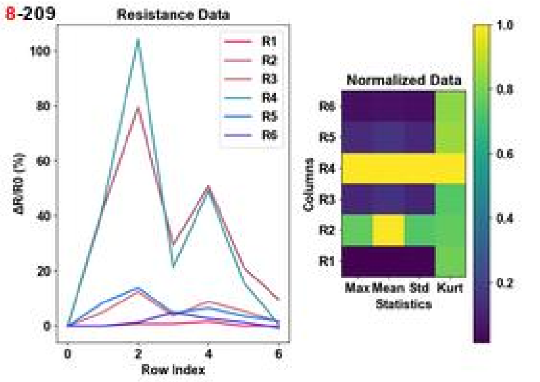

8-210

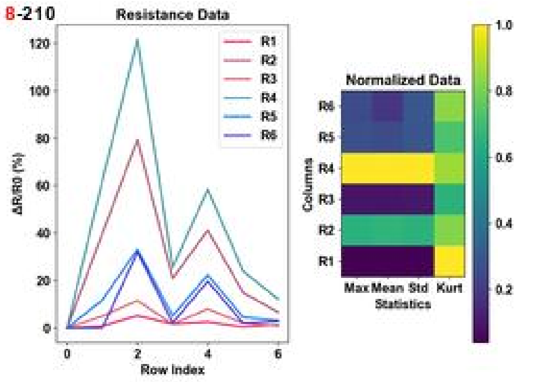

8-211

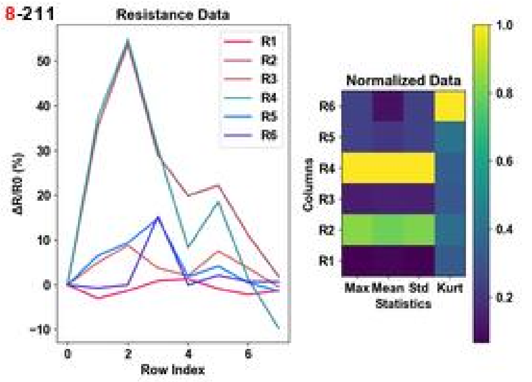

8-212

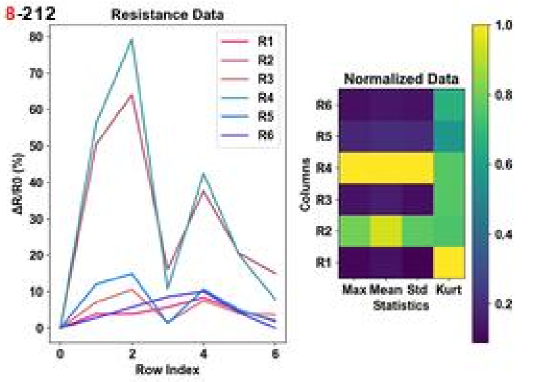

8-213

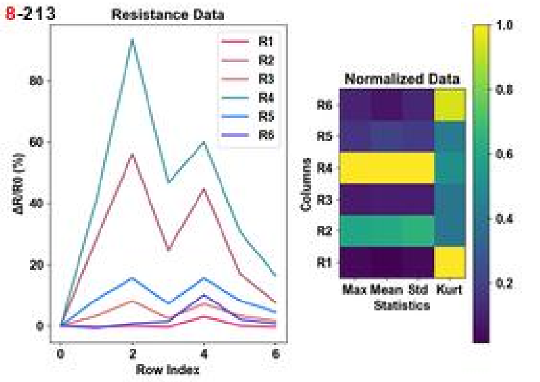

8-214

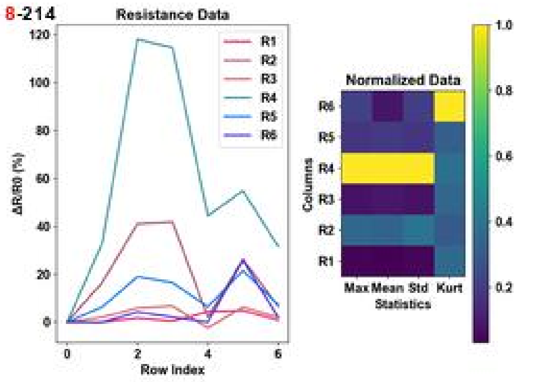

8-215

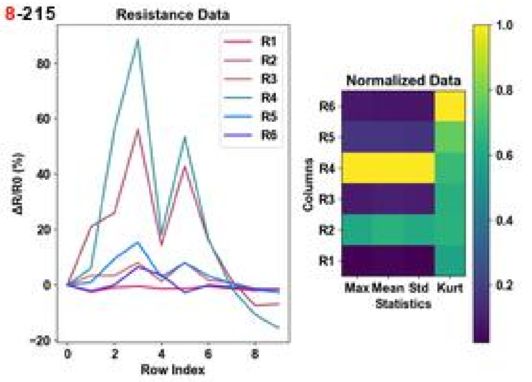

8-216

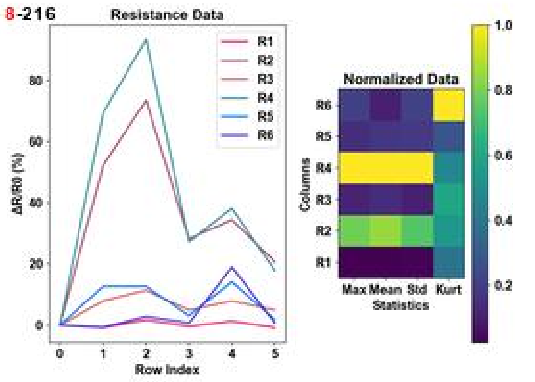

8-217

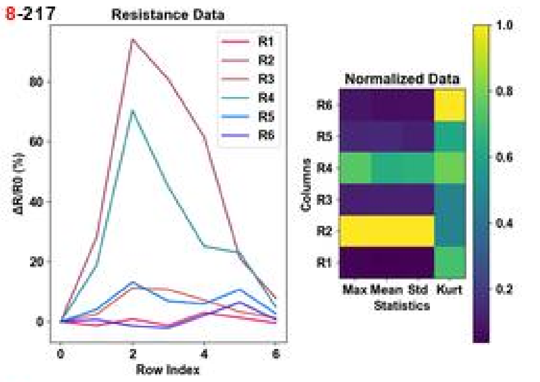

8-218

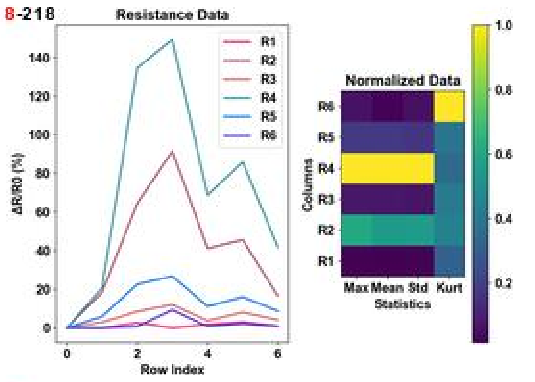

8-219

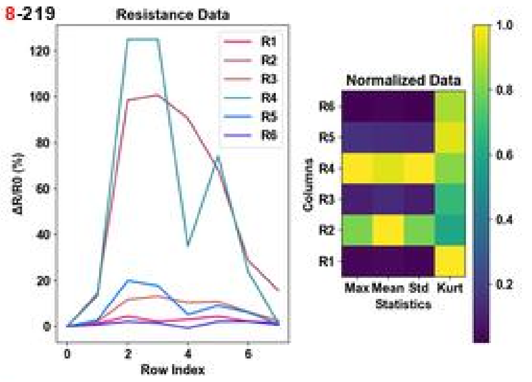

8-220

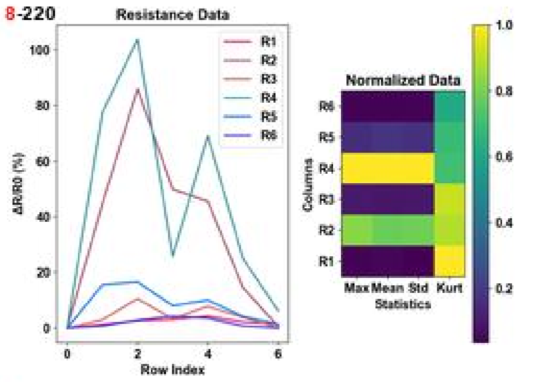

8-221

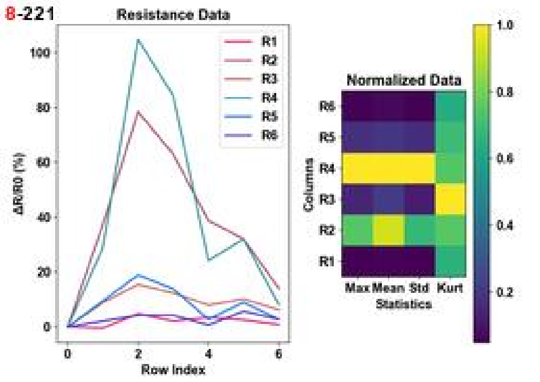

8-222

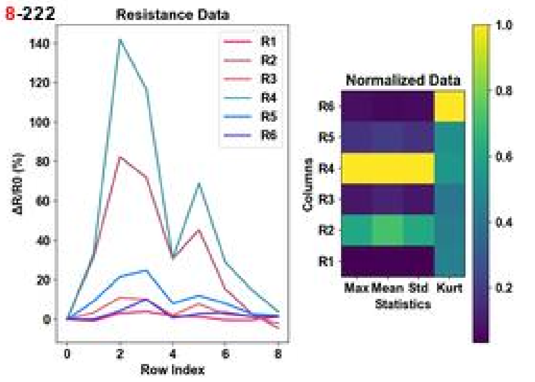

8-223

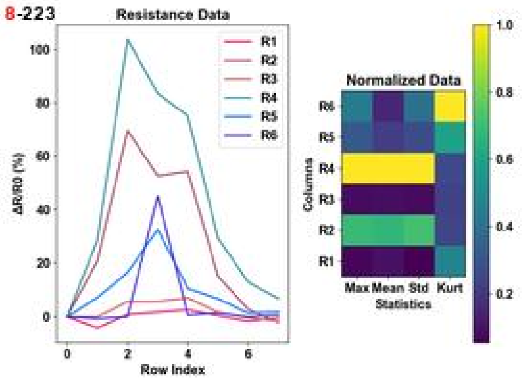

8-224

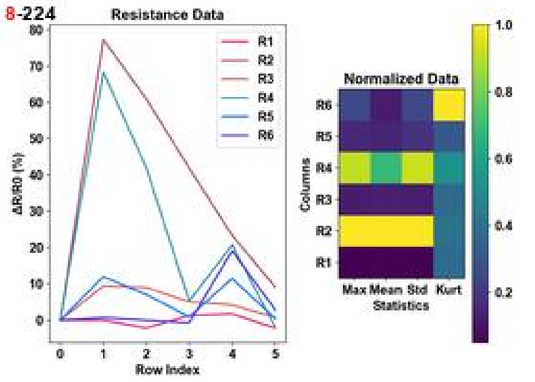

8-225
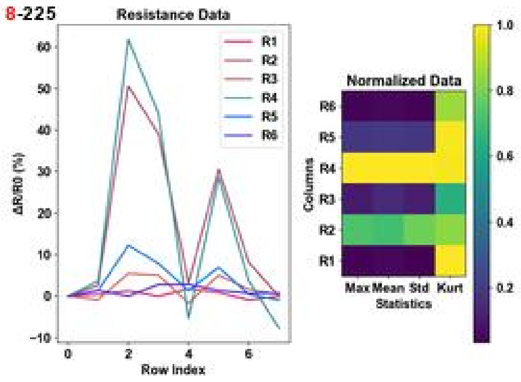

8-226
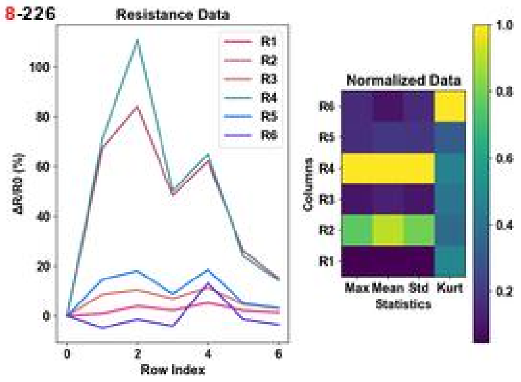

8-227
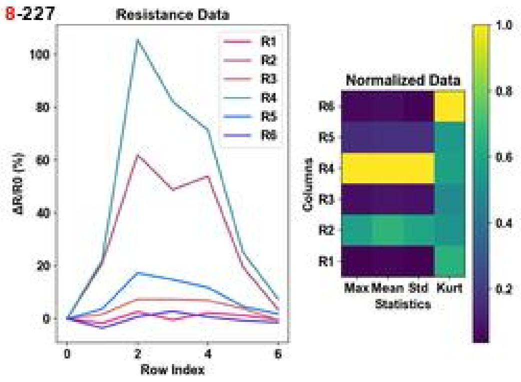

8-228
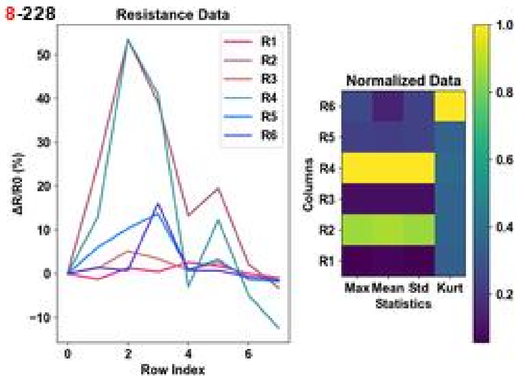

8-229
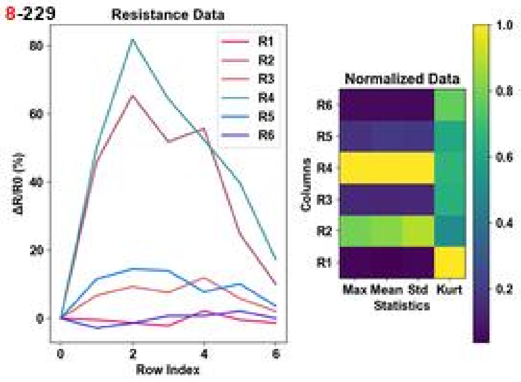

8-230
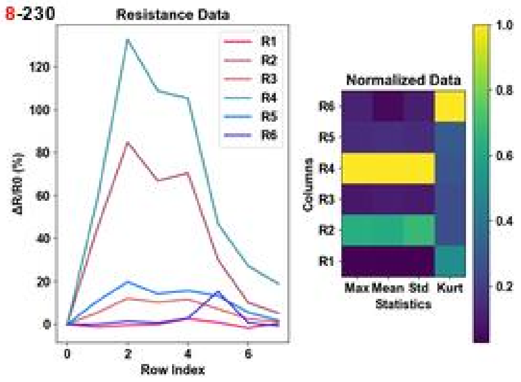

8-231
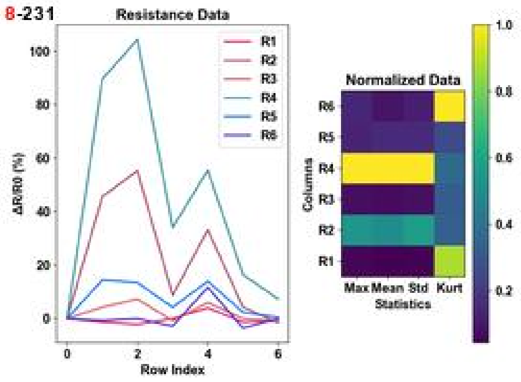

8-232
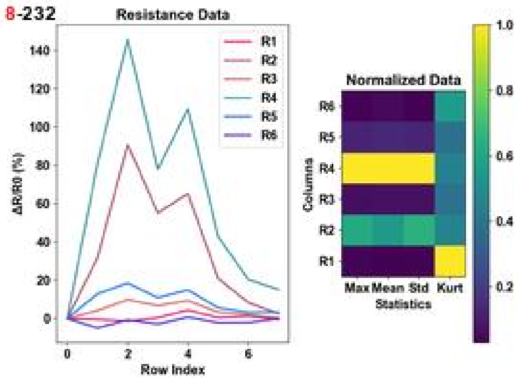

8-233
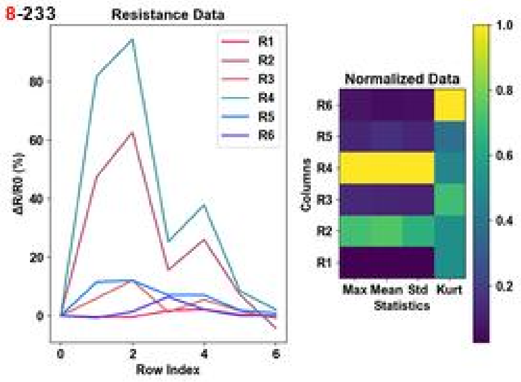

8-234
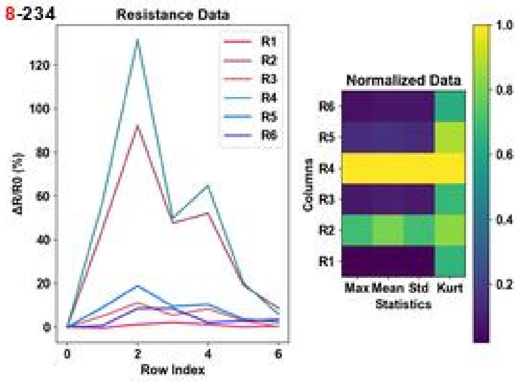

8-235
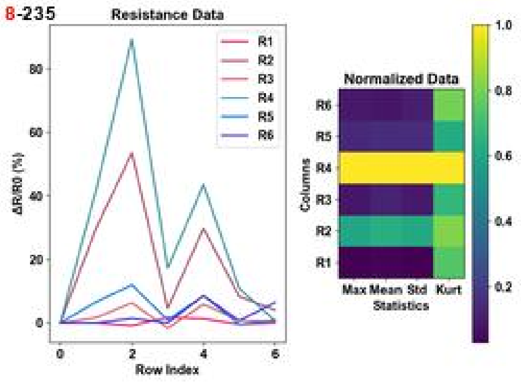

8-236
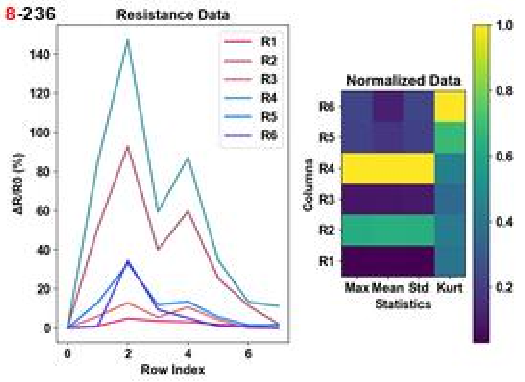

8-237
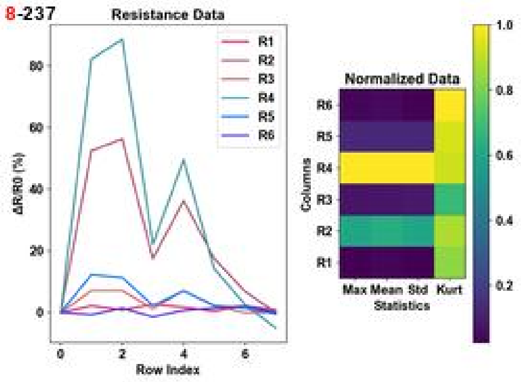

8-238
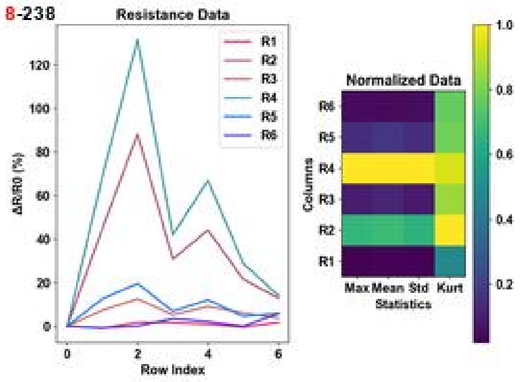

8-239
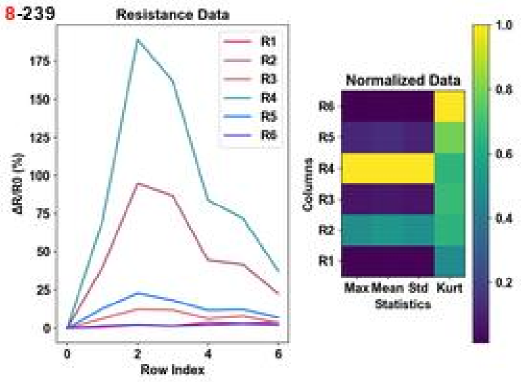

8-240
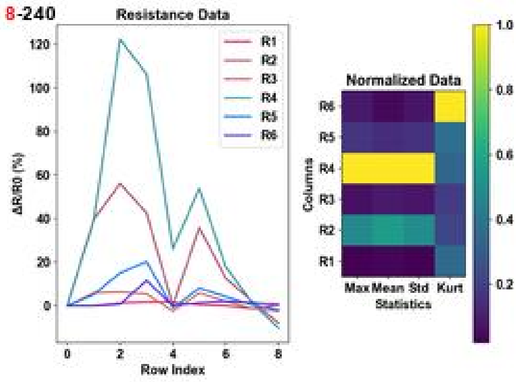

9-1
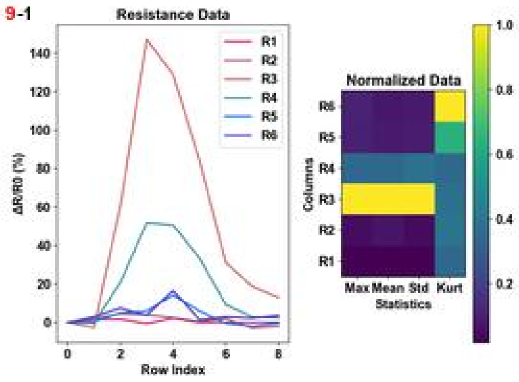

9-2
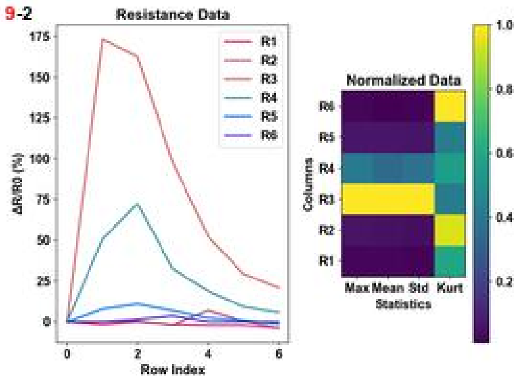

9-3
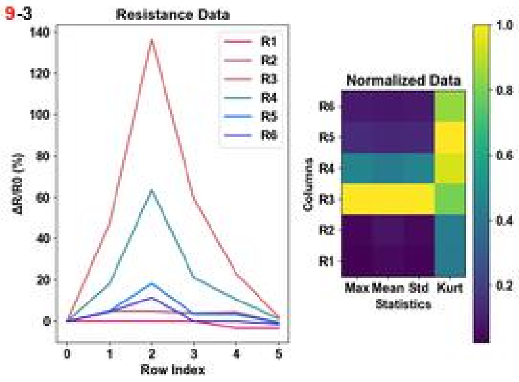

9-4
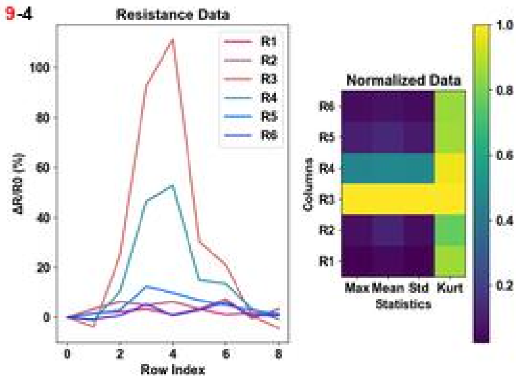

9-5
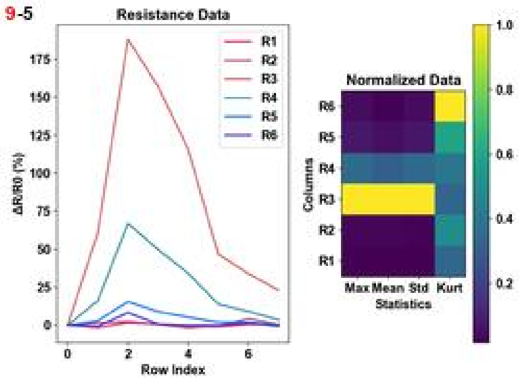

9-6
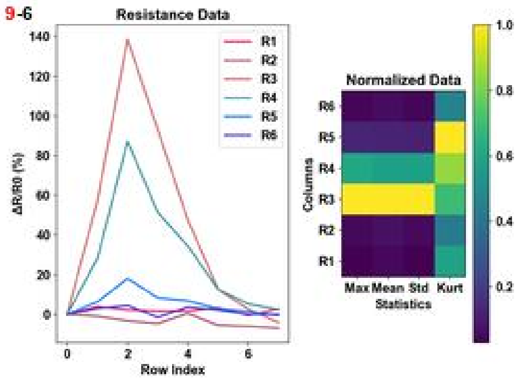

9-7
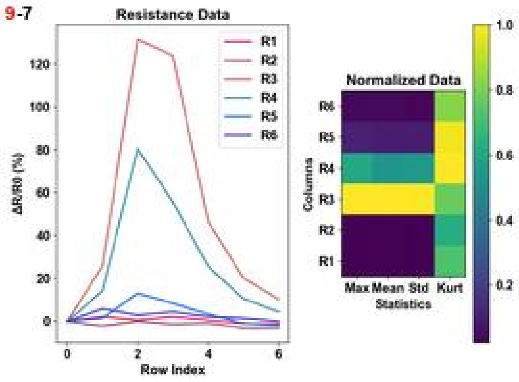

9-8
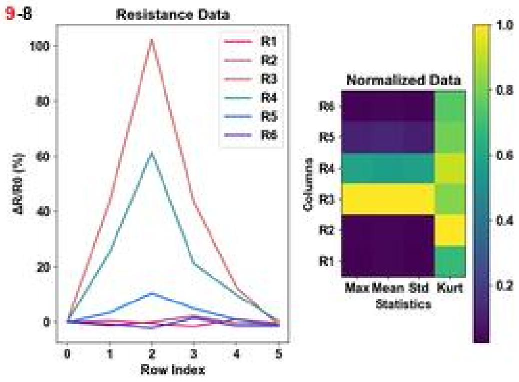

9-9
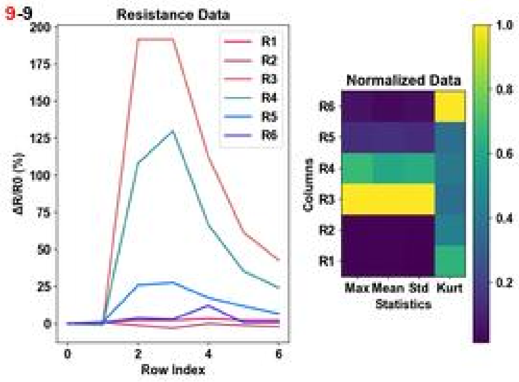

9-10
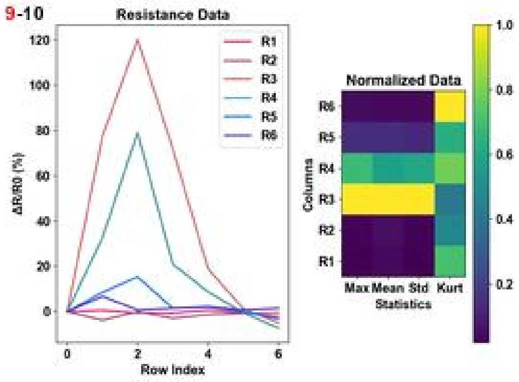

9-11
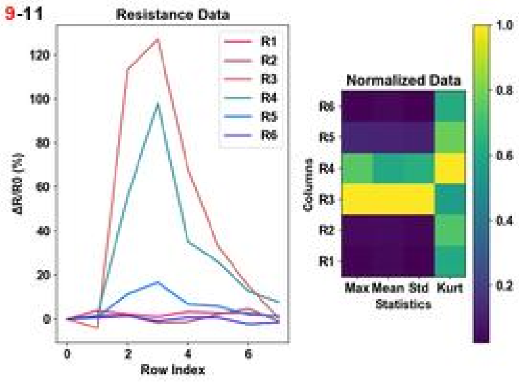

9-12
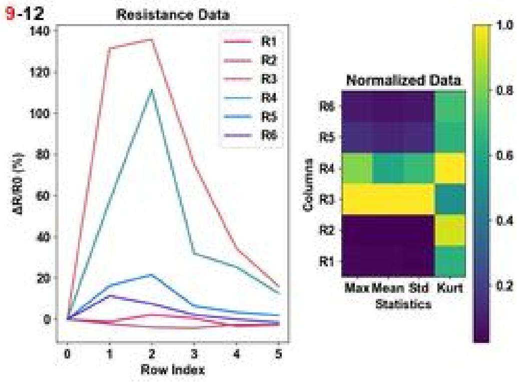

9-13
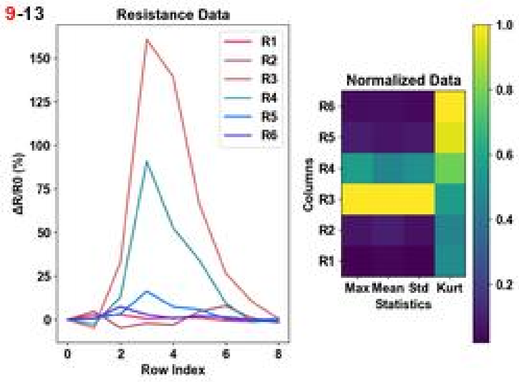

9-14
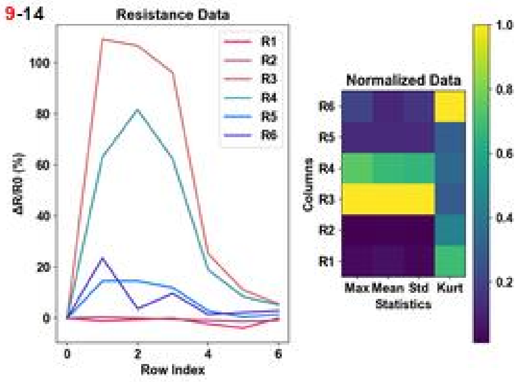

9-15
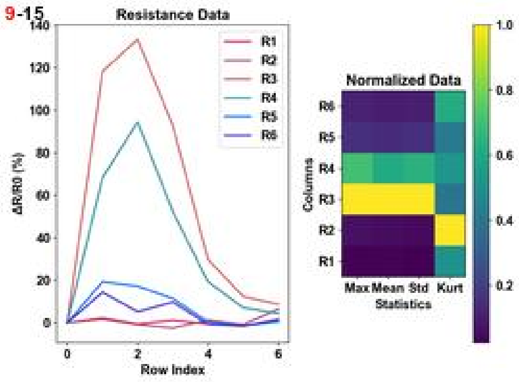

9-16
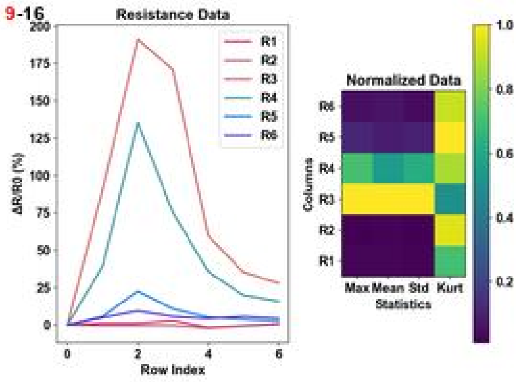

9-17
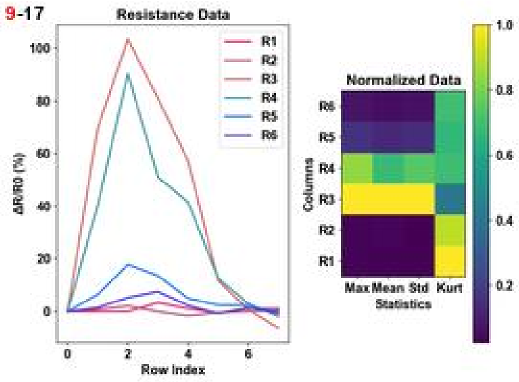

9-18
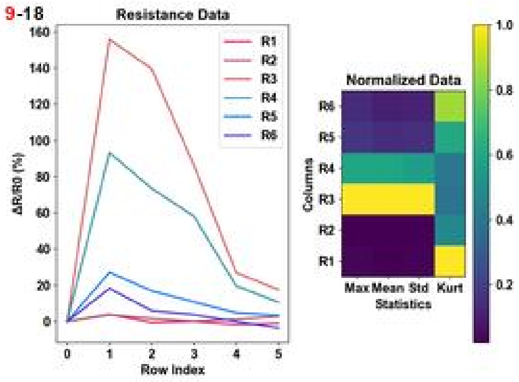

9-19
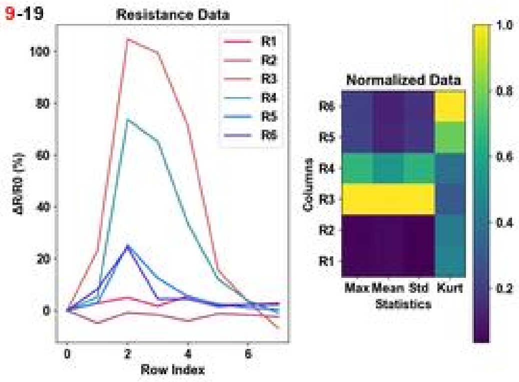

9-20
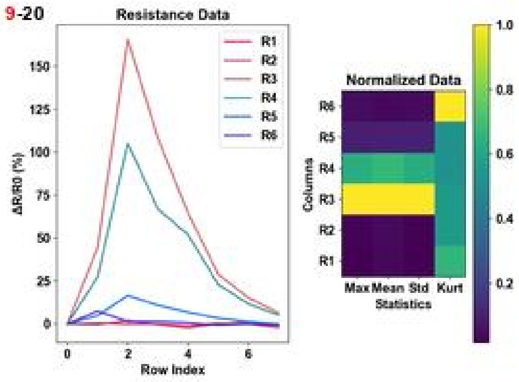

9-21
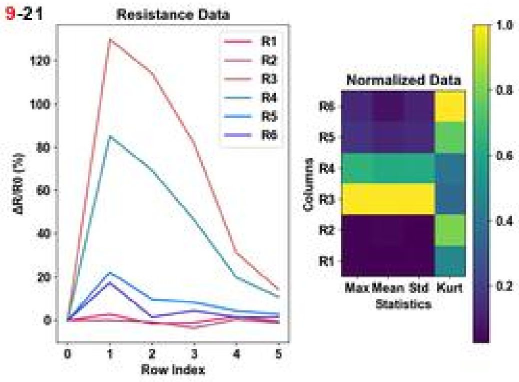

9-22
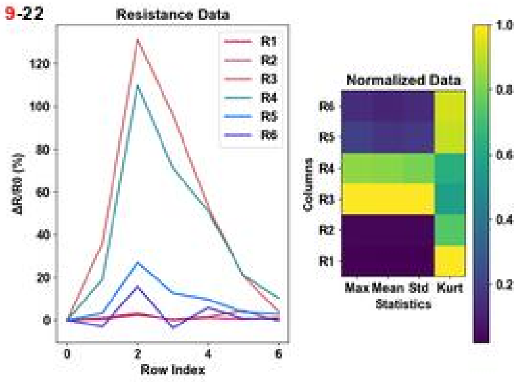

9-23
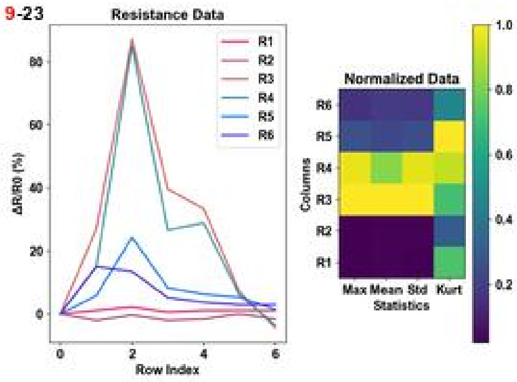

9-24
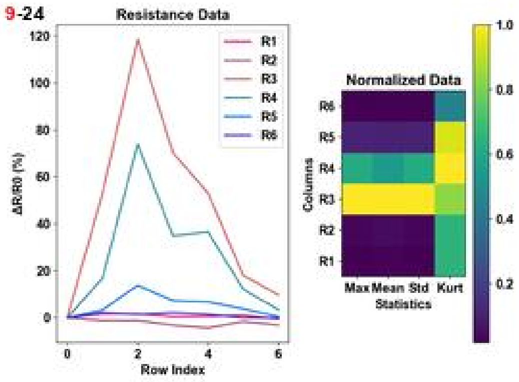

9-25
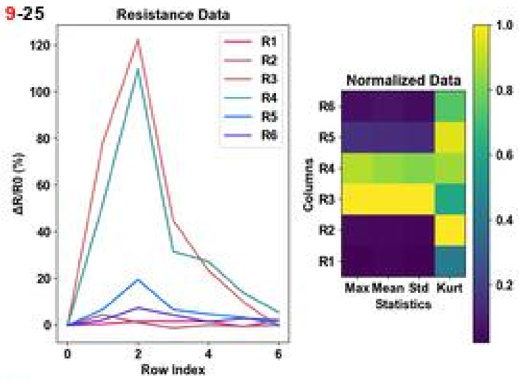

9-26
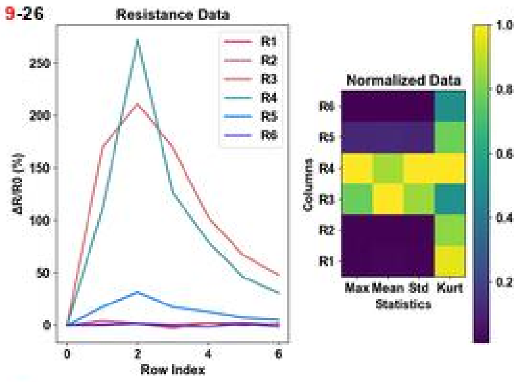

9-27
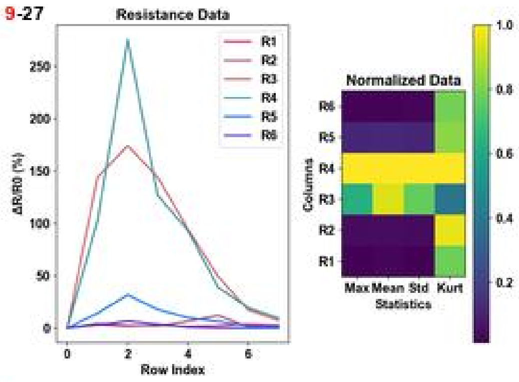

9-28
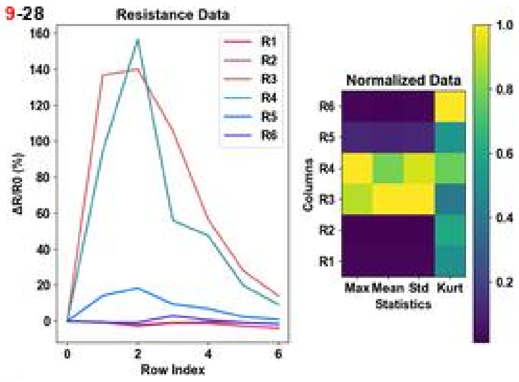

9-29
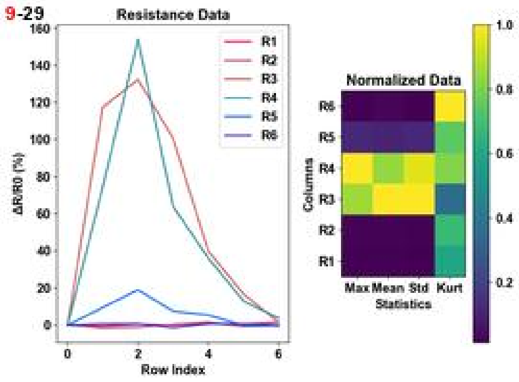

9-30
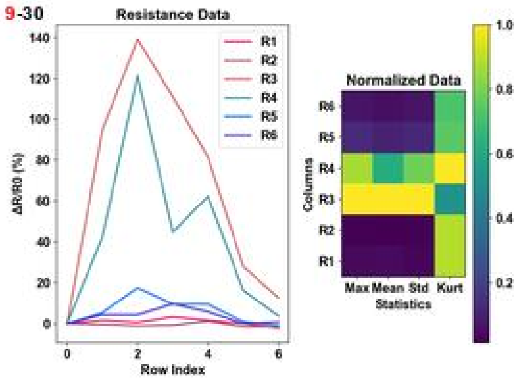

9-31
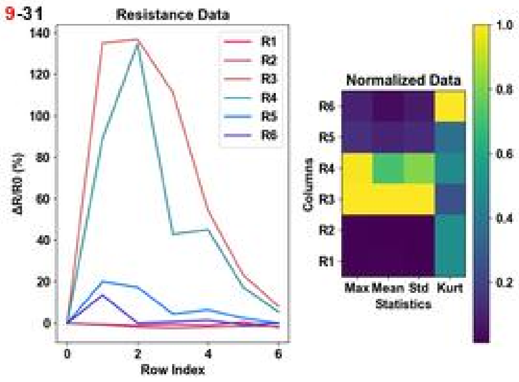

9-32
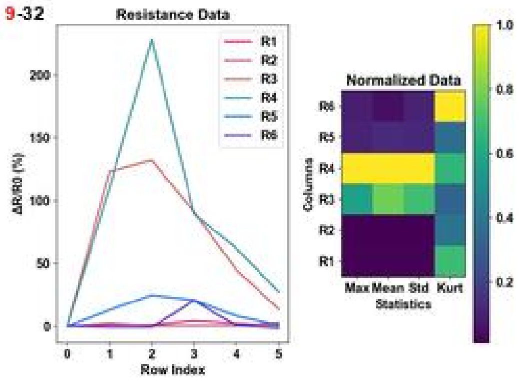

Pressure-sensitive data of type 9

Pressure-sensitive data of type 9

9-33
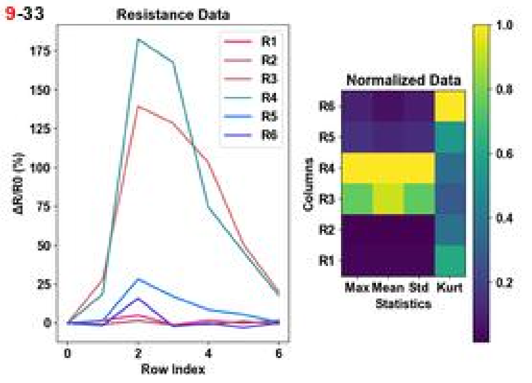

9-34
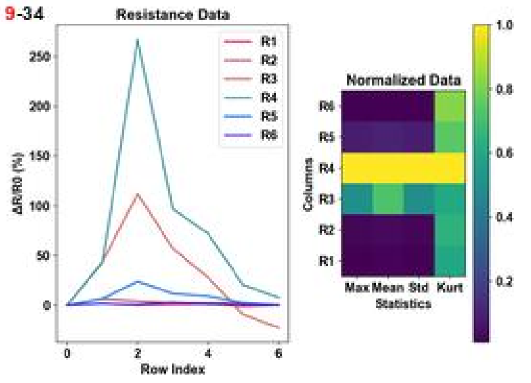

9-35
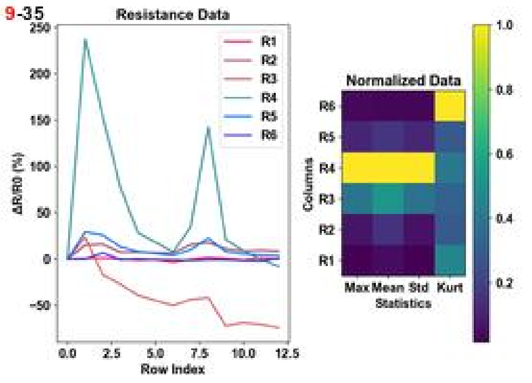

9-36
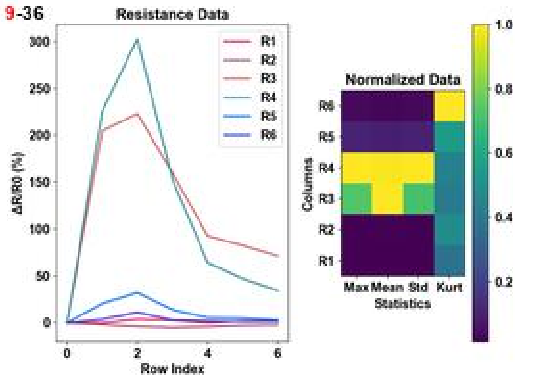

9-37
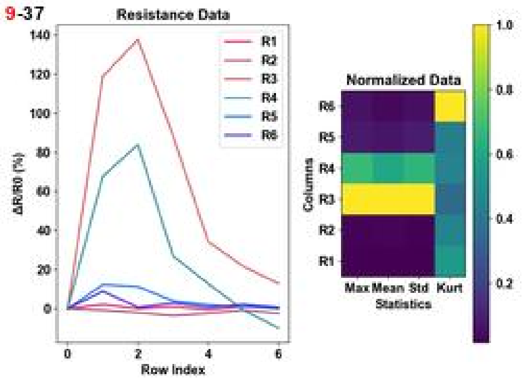

9-38
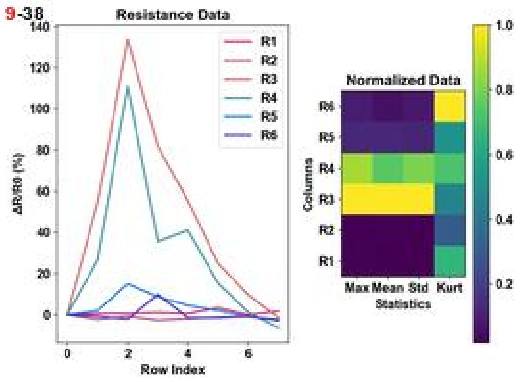

9-39
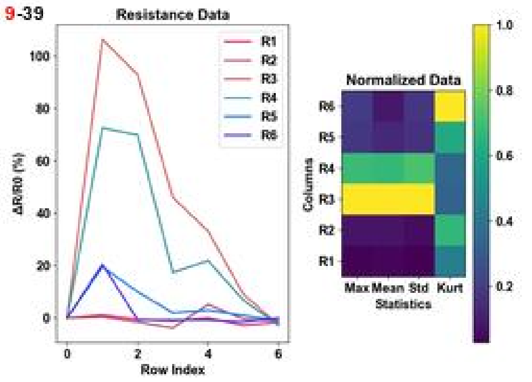

9-40
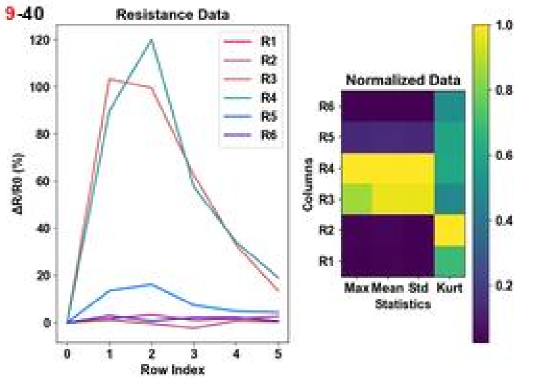

9-41
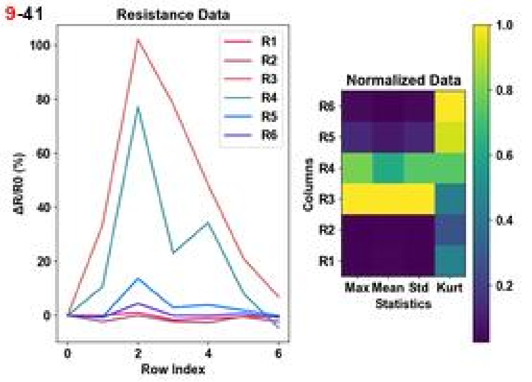

9-42
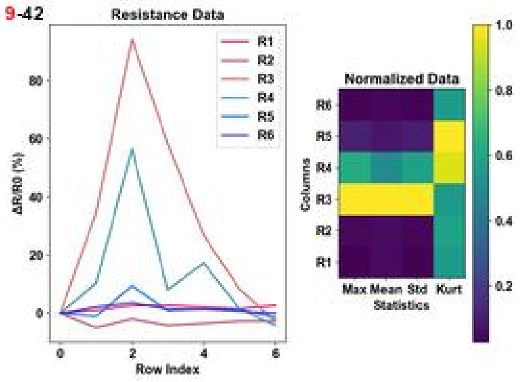

9-43
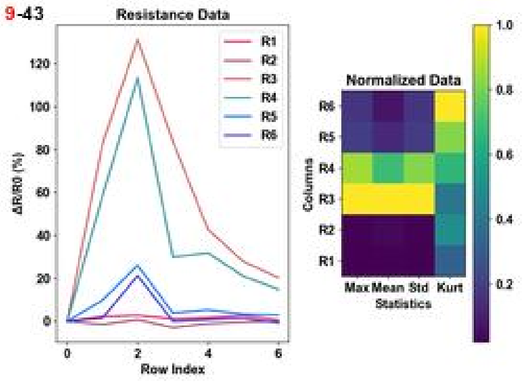

9-44
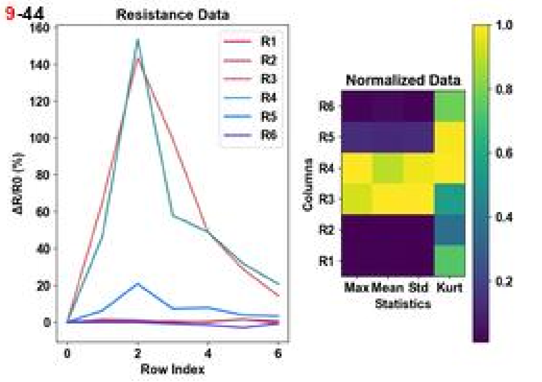

9-45
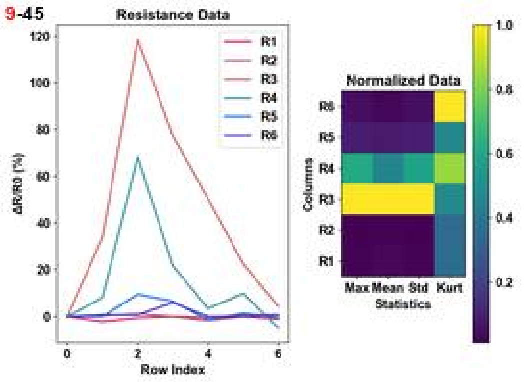

9-46
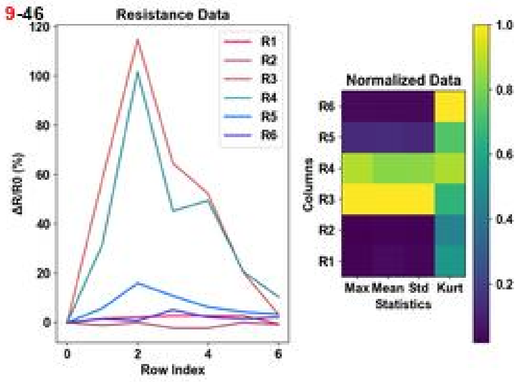

9-47
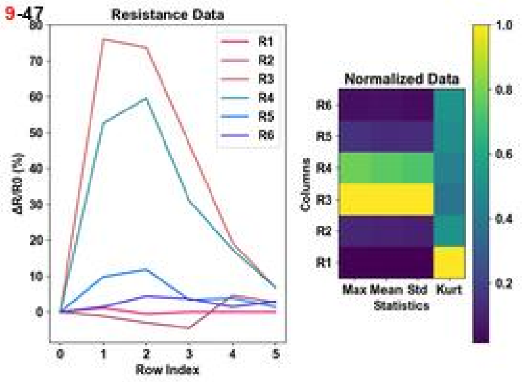

9-48
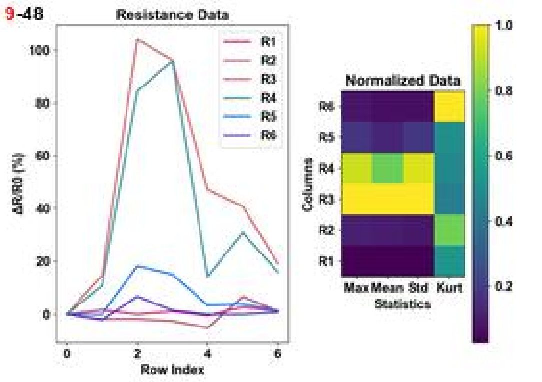

9-49
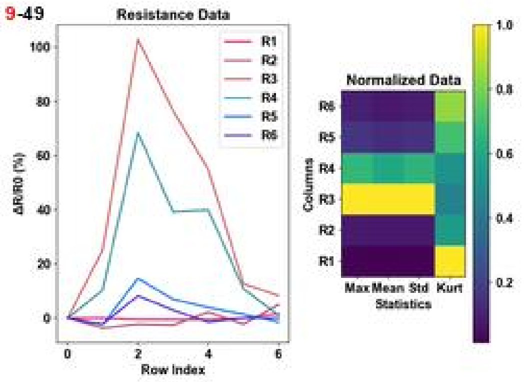

9-50
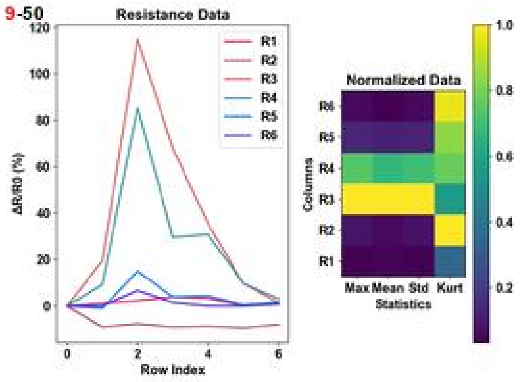

9-51
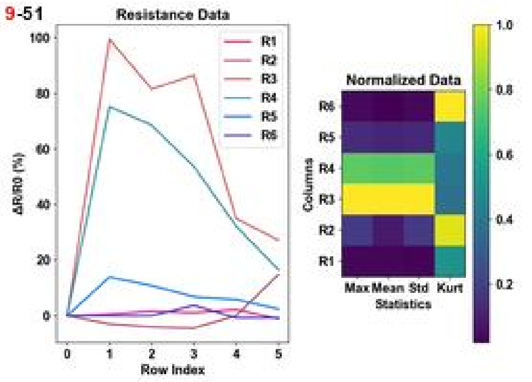

9-52
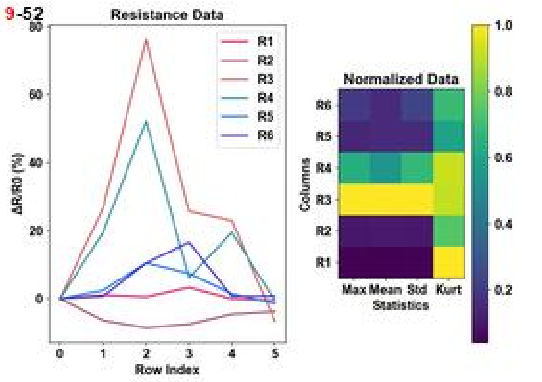

9-53
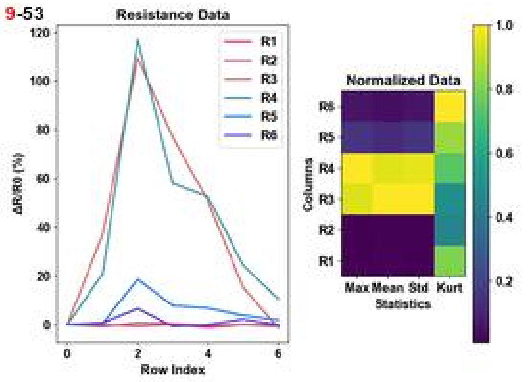

9-54
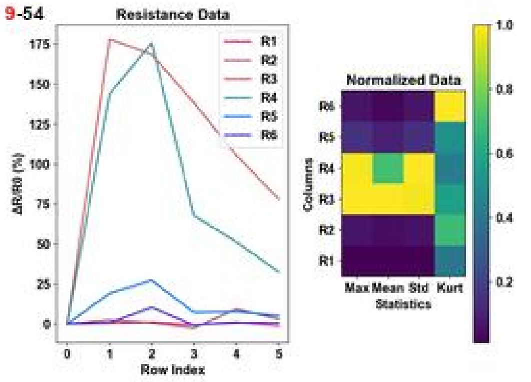

9-55
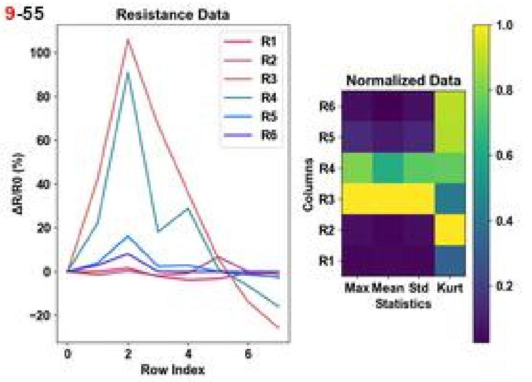

9-56
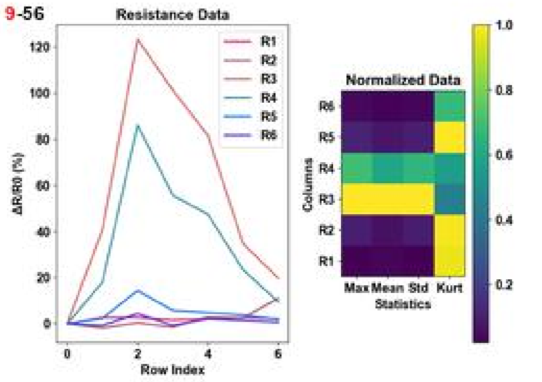

9-57
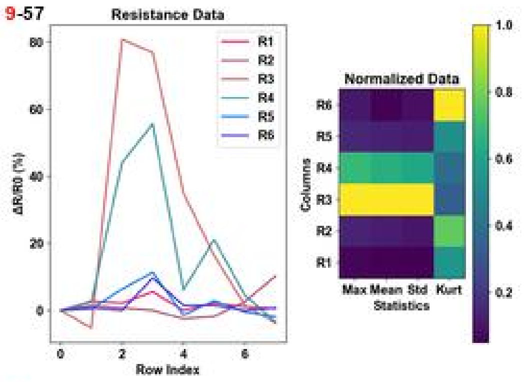

9-58
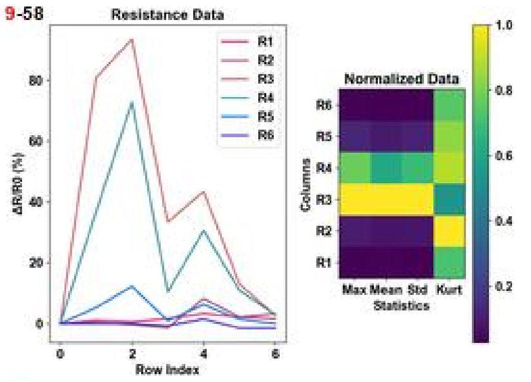

9-59
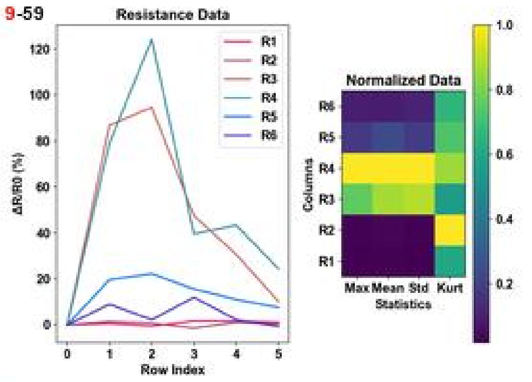

9-60
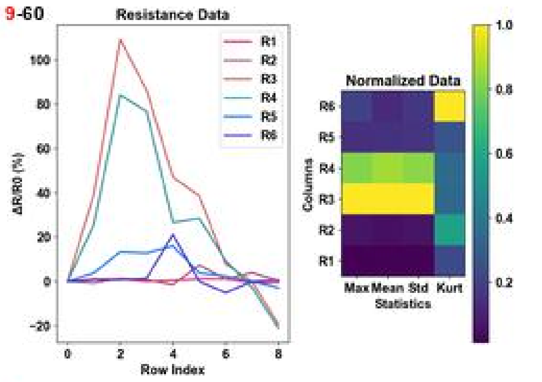

9-61
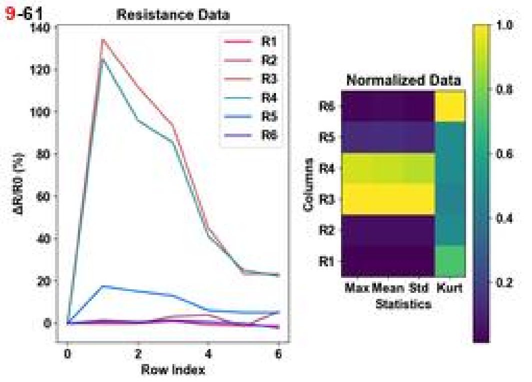

9-62
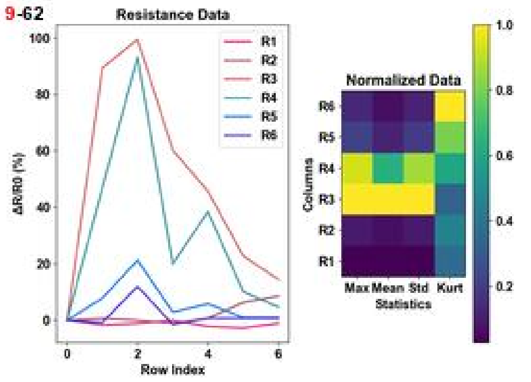

9-63
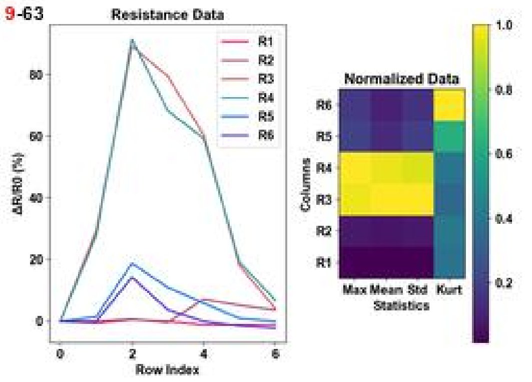

9-64
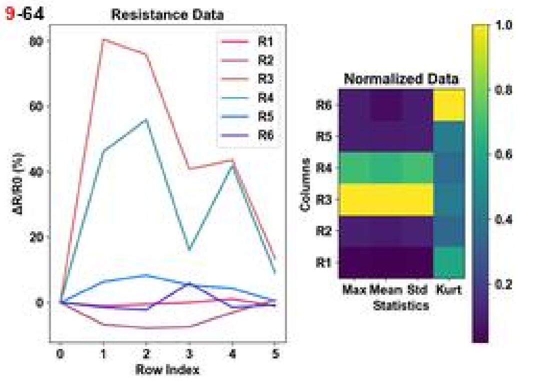

9-65
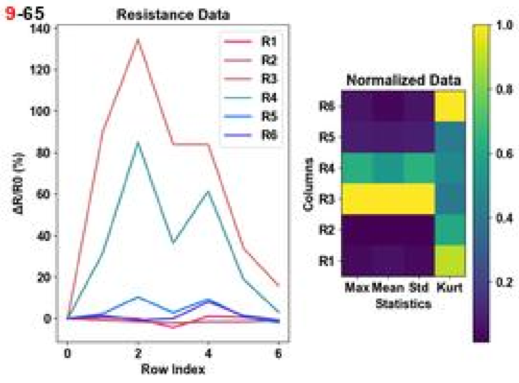

9-66
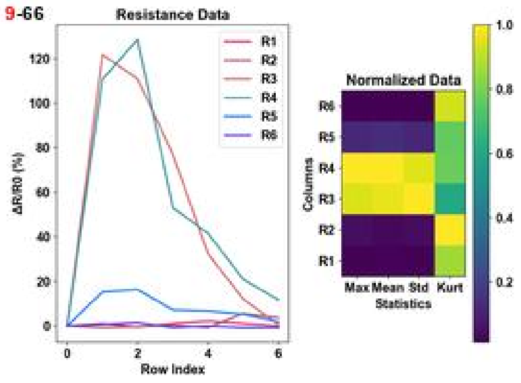

9-67
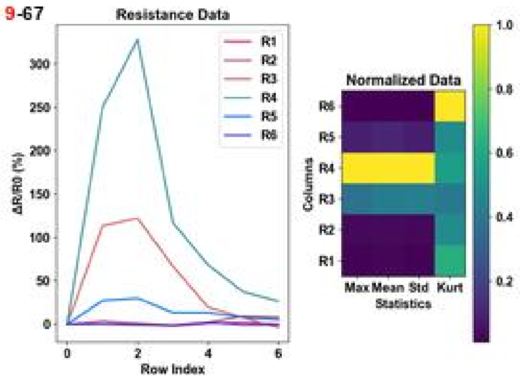

9-68
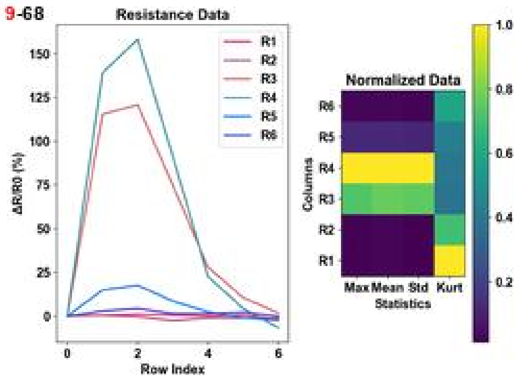

9-69
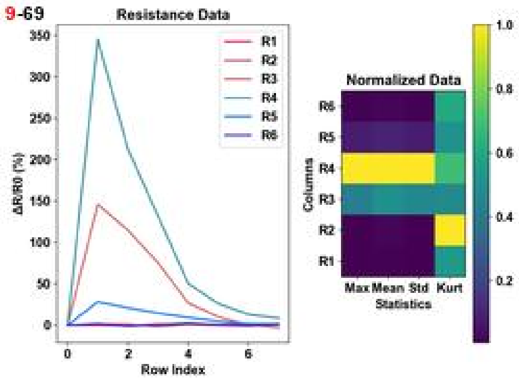

9-70
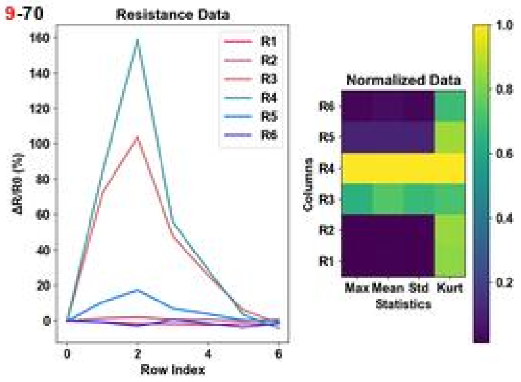

9-71
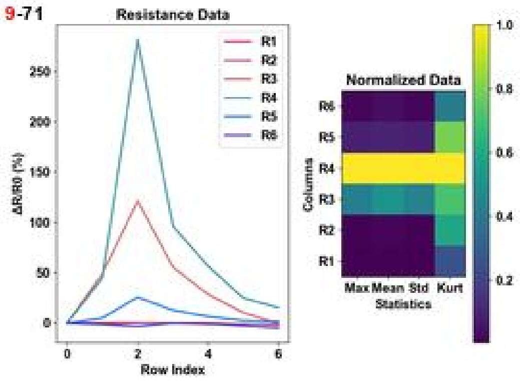

9-72
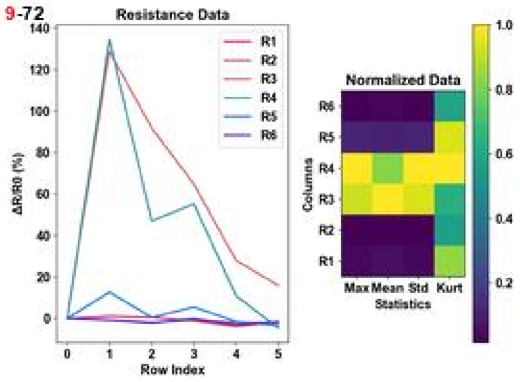

9-73
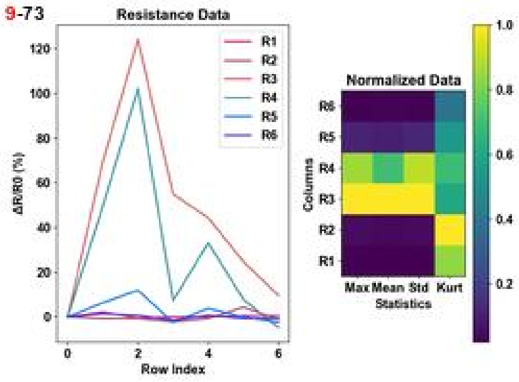

9-74
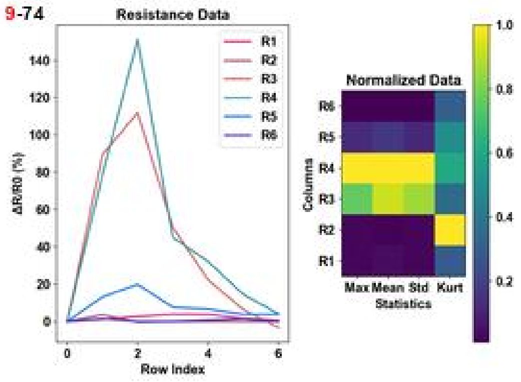

9-75
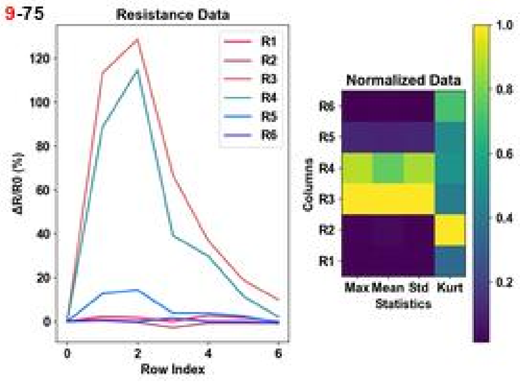

9-76
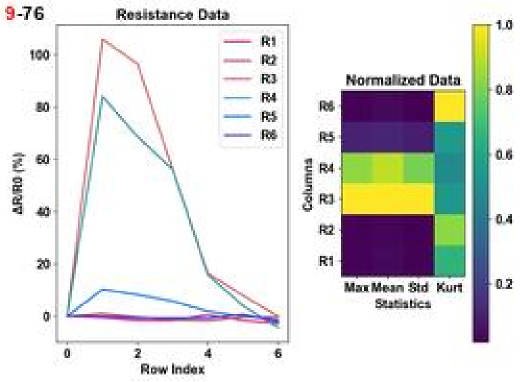

9-77
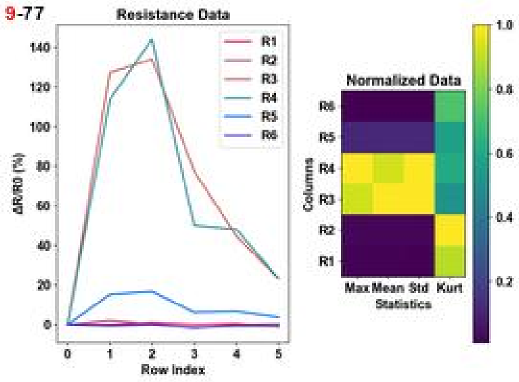

9-78
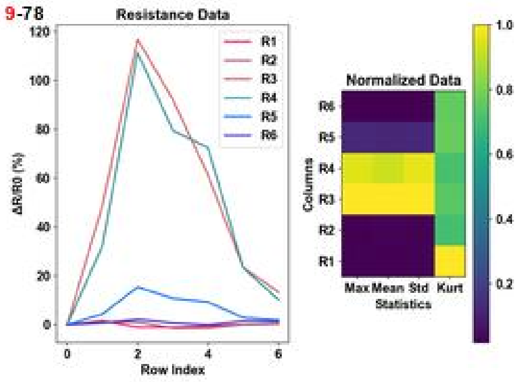

9-79
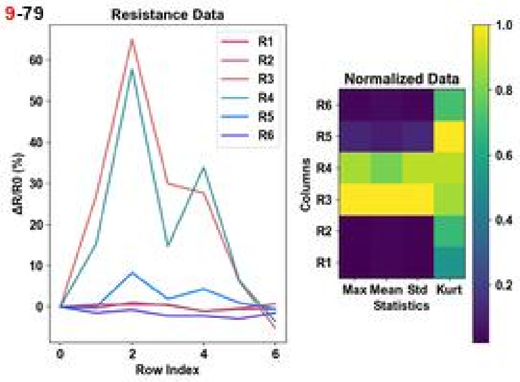

9-80
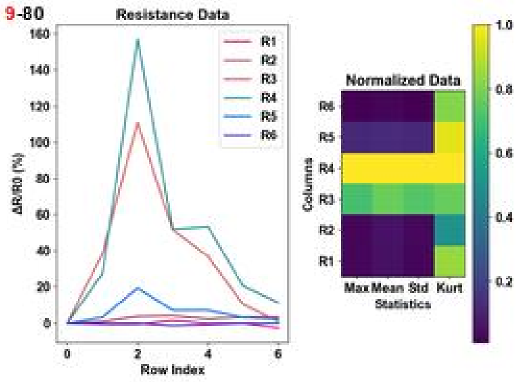

9-81
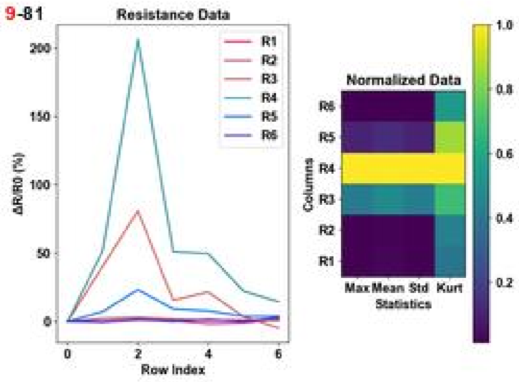

9-82
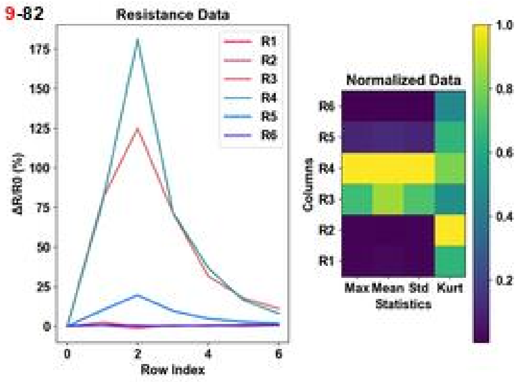

9-83
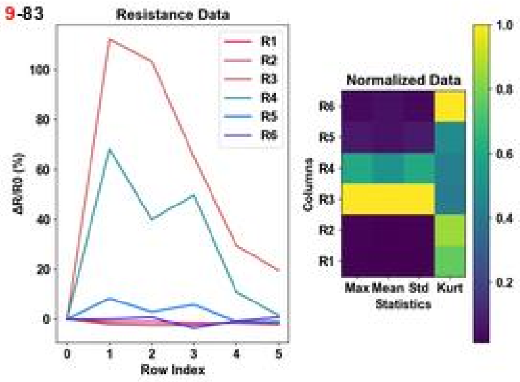

9-84
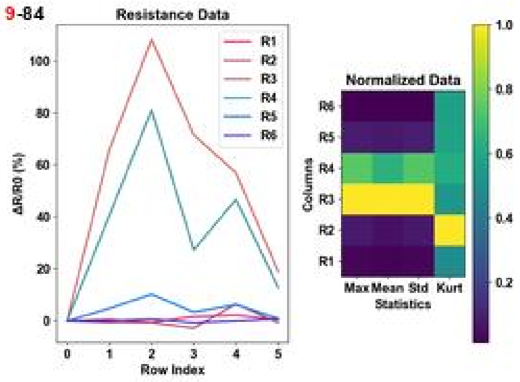

9-85
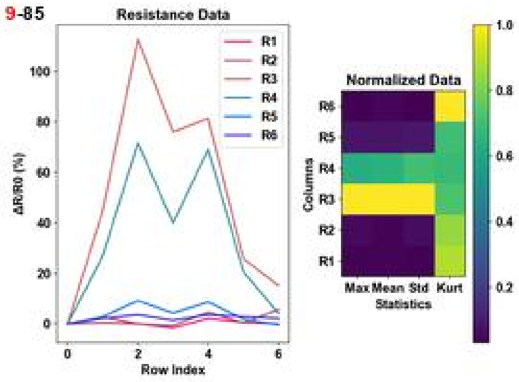

9-86
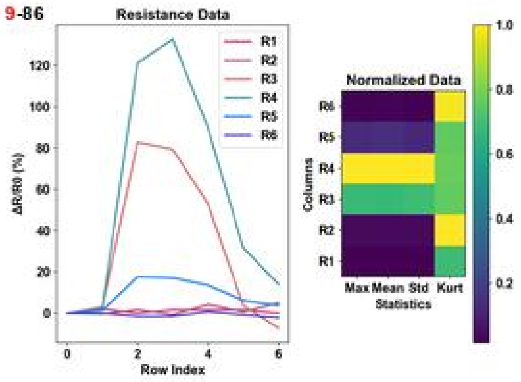

9-87
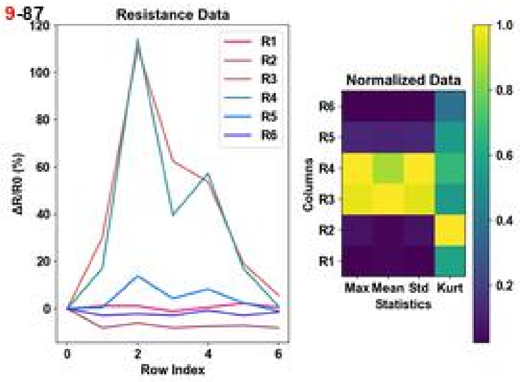

9-88
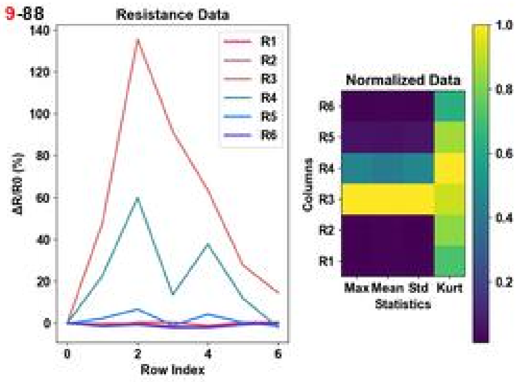

9-89
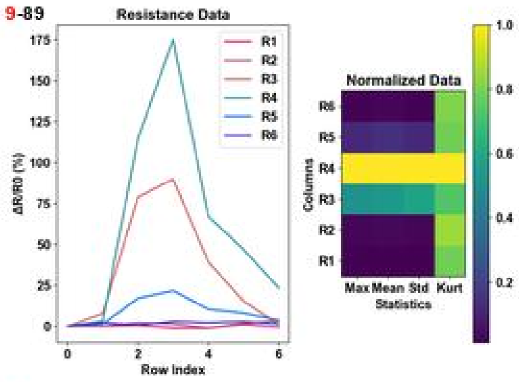

9-90
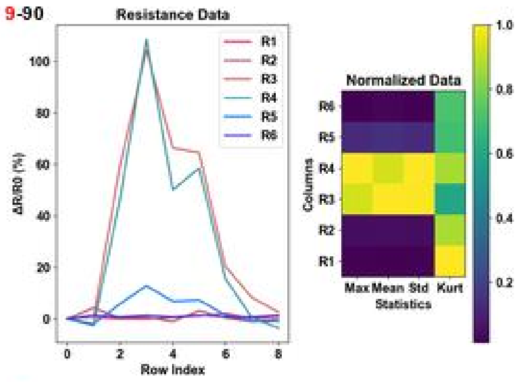

9-91
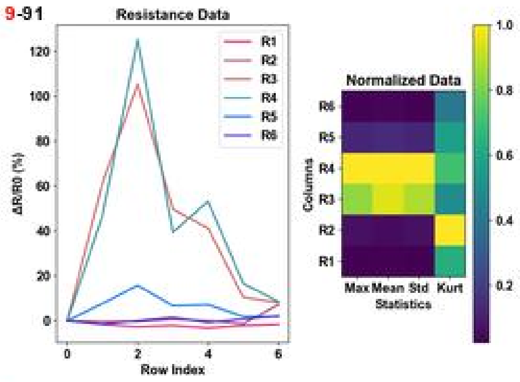

9-92
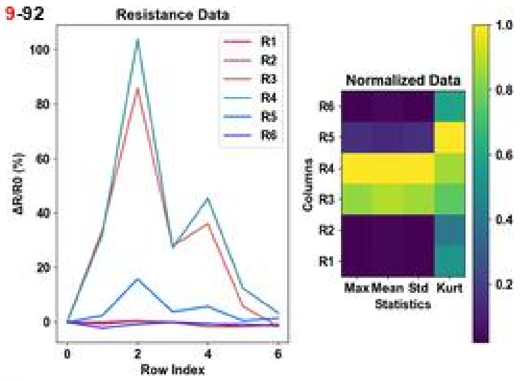

9-93
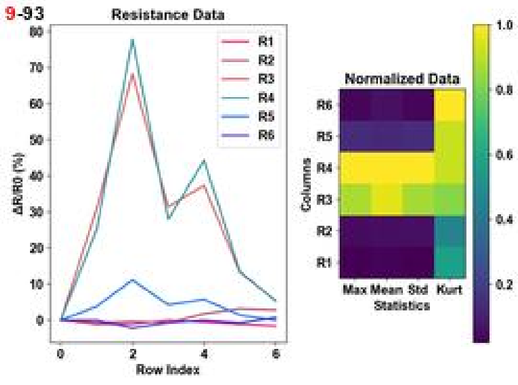

9-94
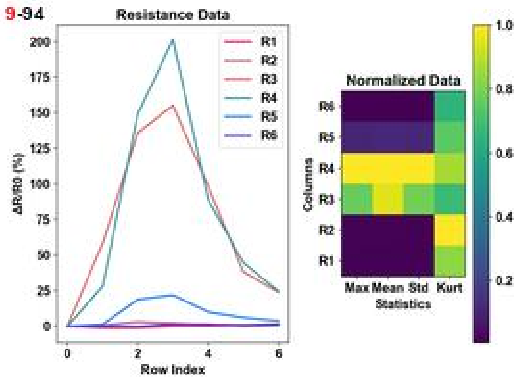

9-95
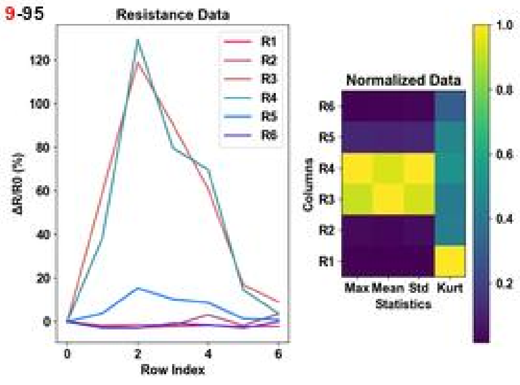

9-96
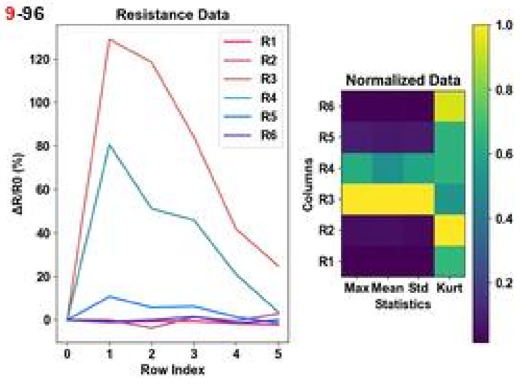

9-97
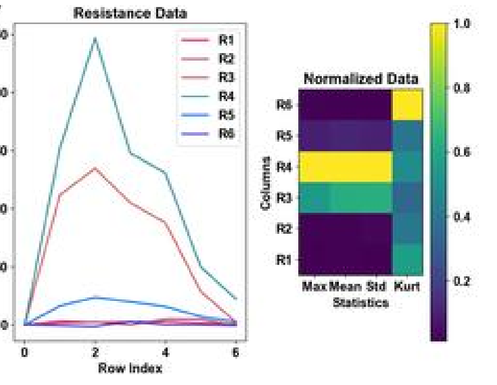

9-98
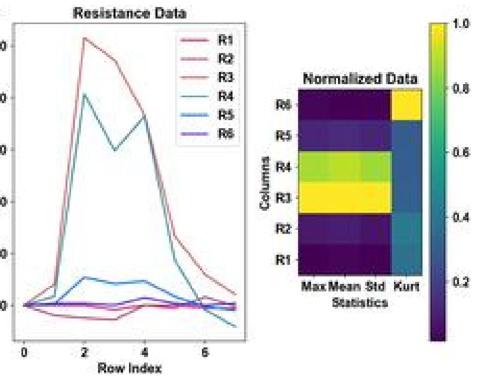

9-99
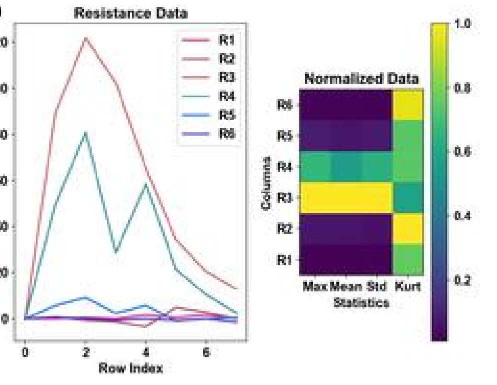

9-100
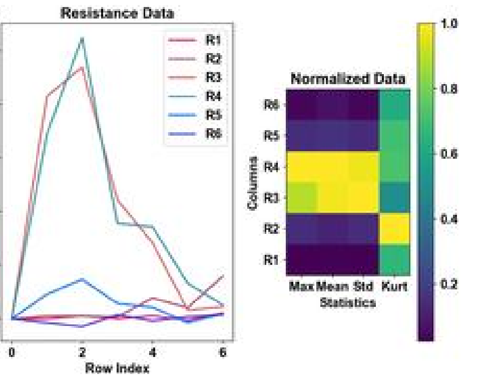

9-101
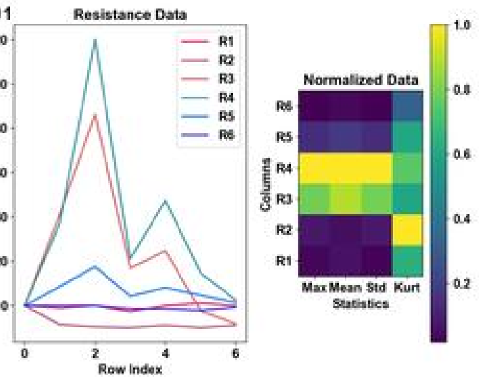

9-102
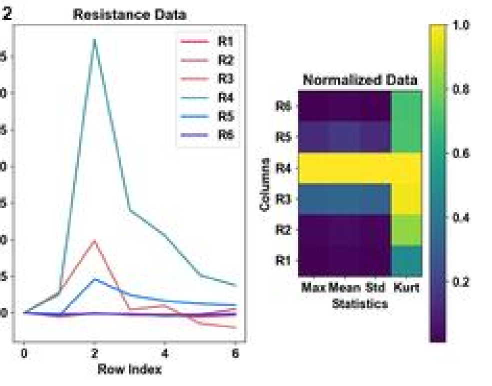

9-103
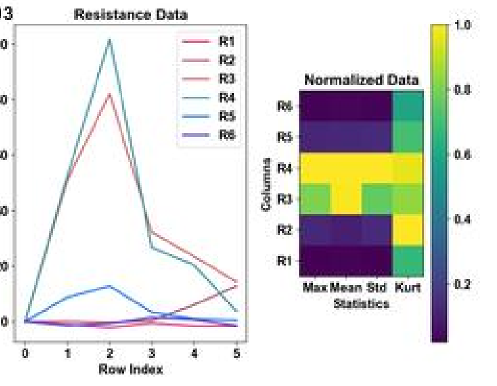

9-104
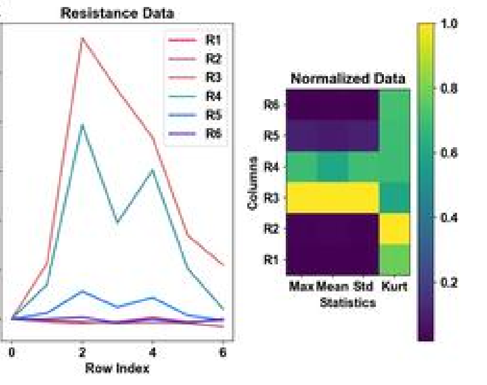

9-105
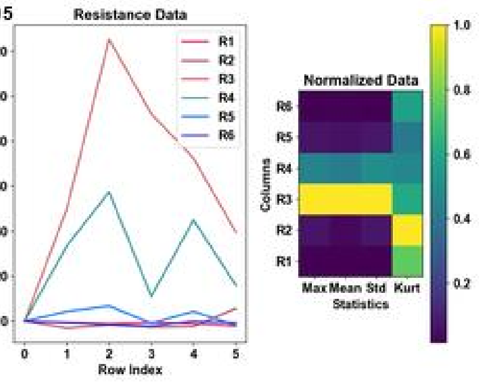

9-106
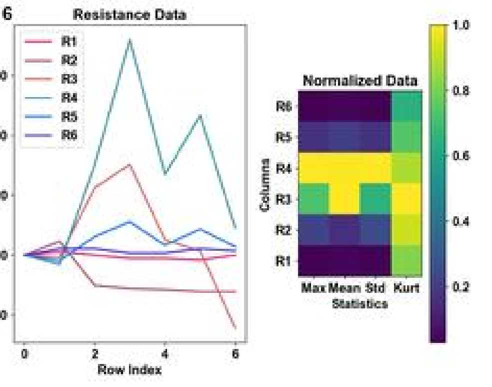

9-107
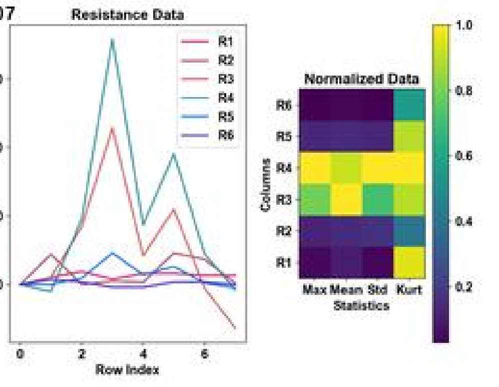

9-108
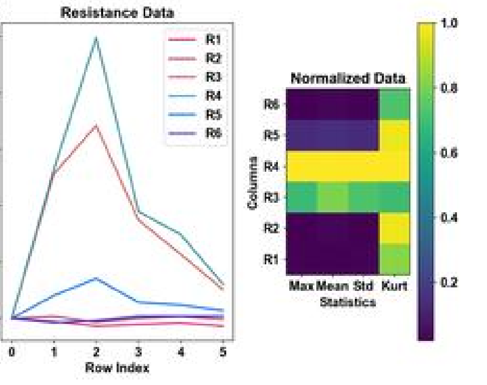

9-109
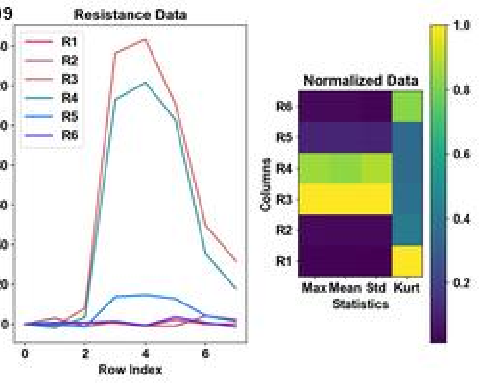

9-110
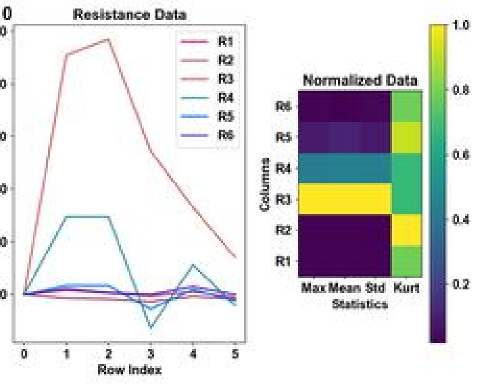

9-111
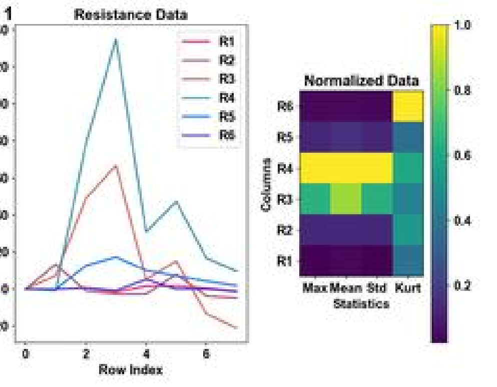

9-112
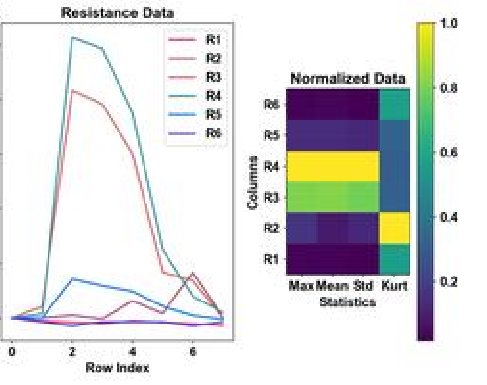

9-113
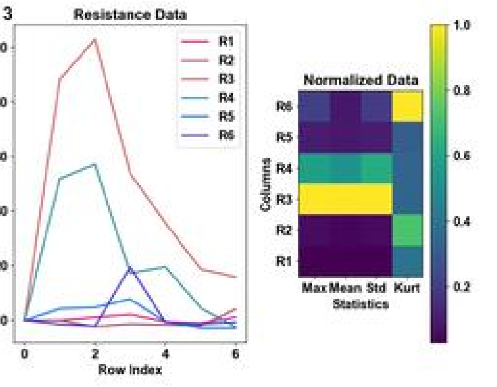

9-114
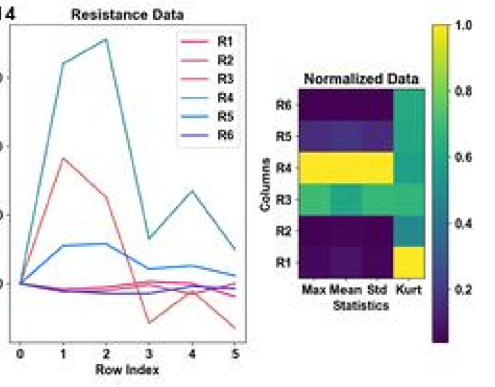

9-115
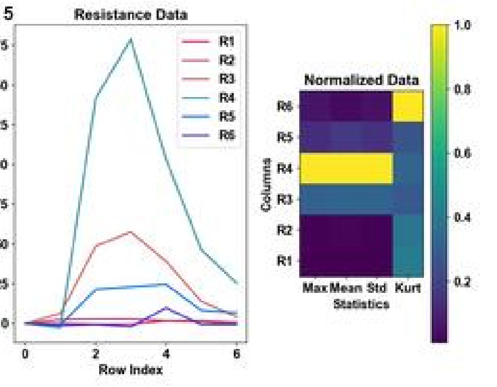

9-116
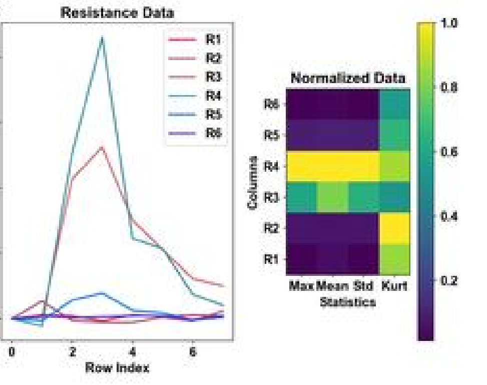

9-117
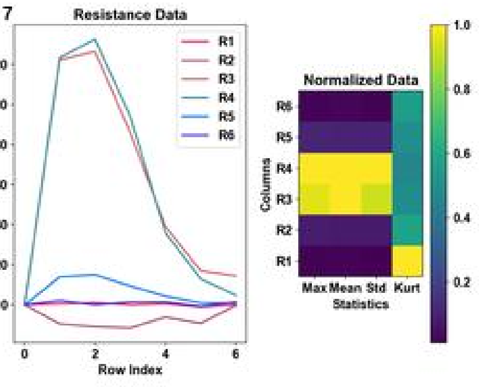

9-118
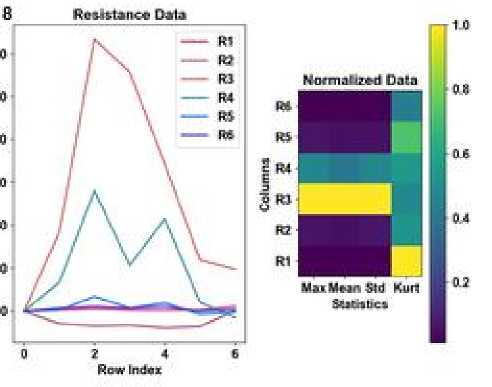

9-119
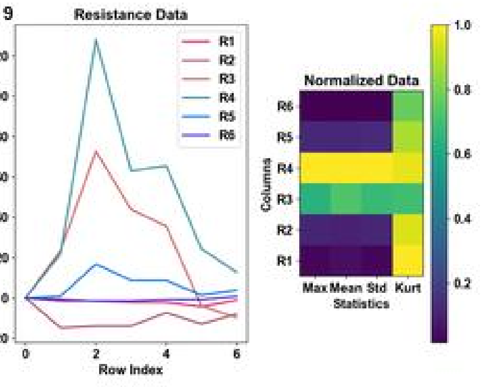

9-120
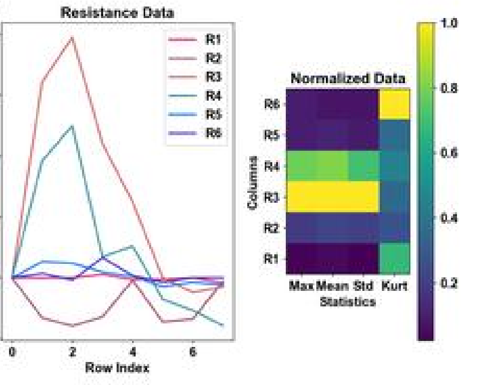

9-121
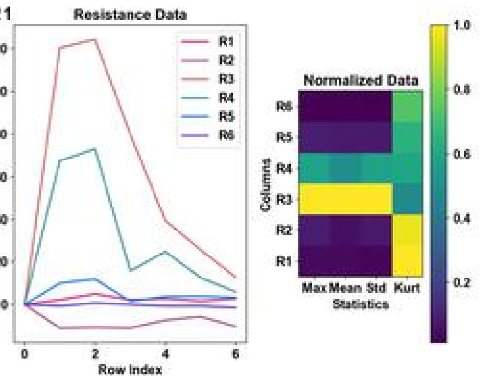

9-122
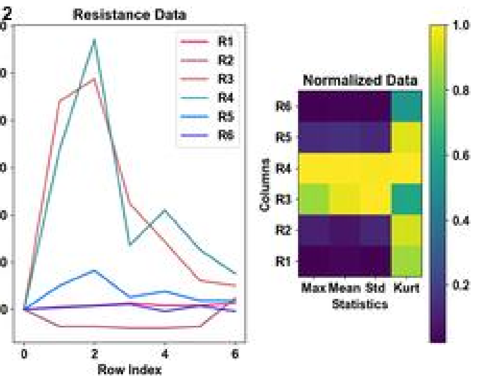

9-123
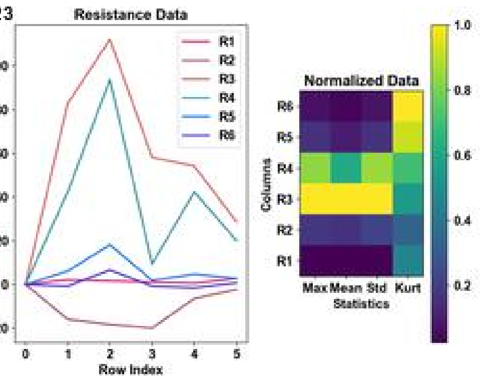

9-124
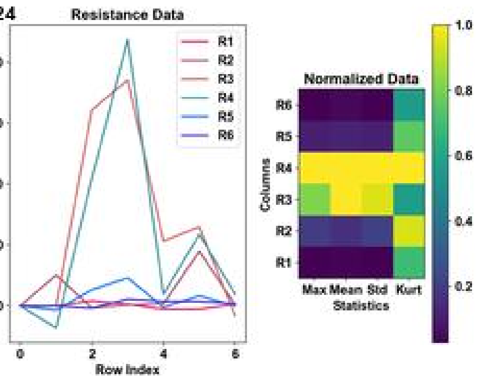

9-125
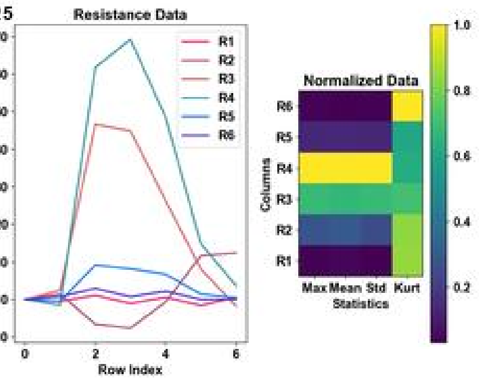

9-126
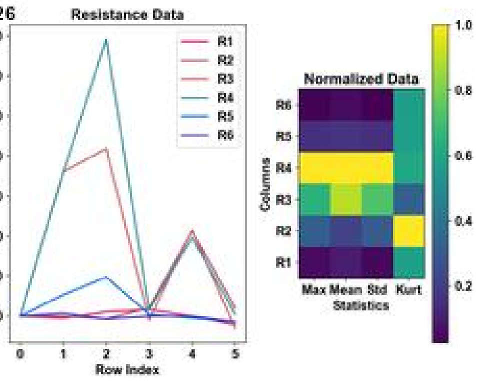

9-127
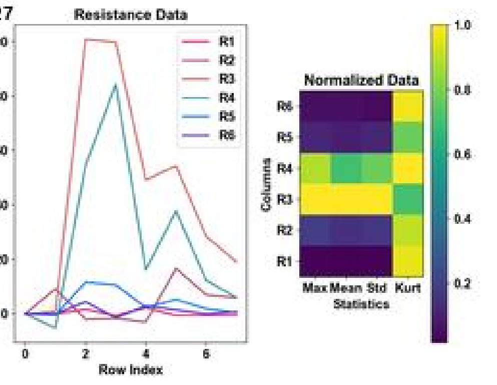

9-128
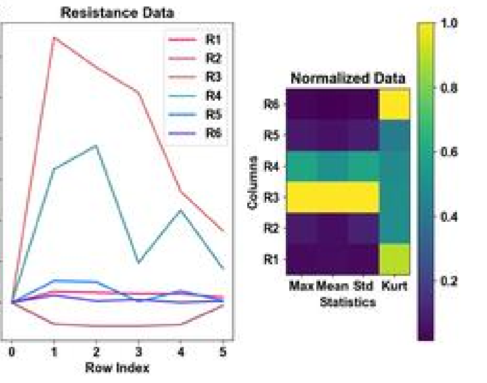

Pressure-sensitive data of type 9

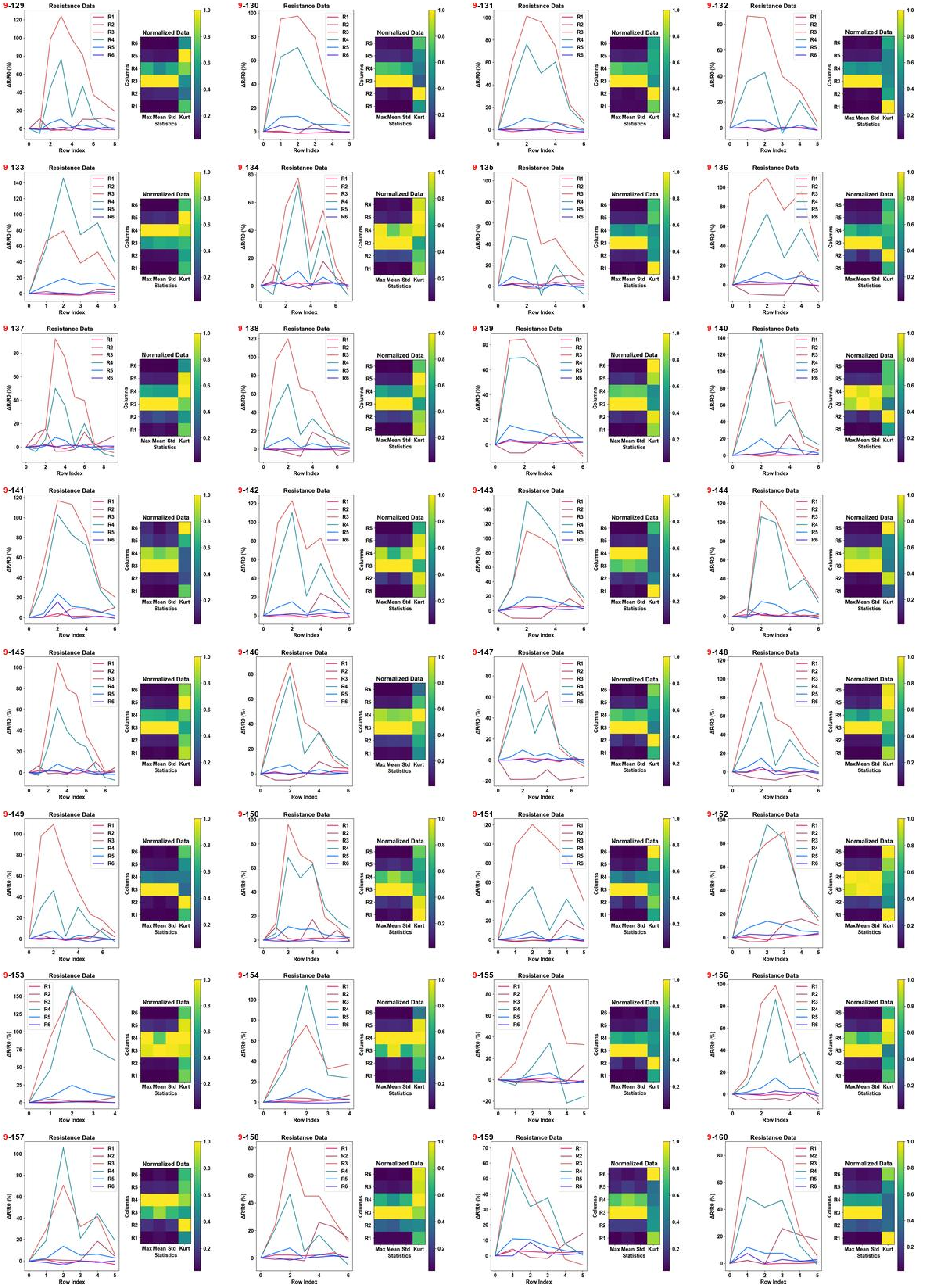

9-161
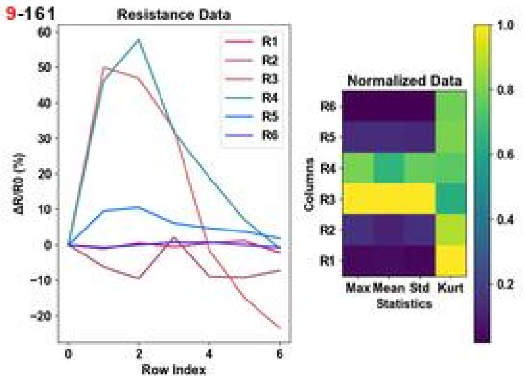

9-162
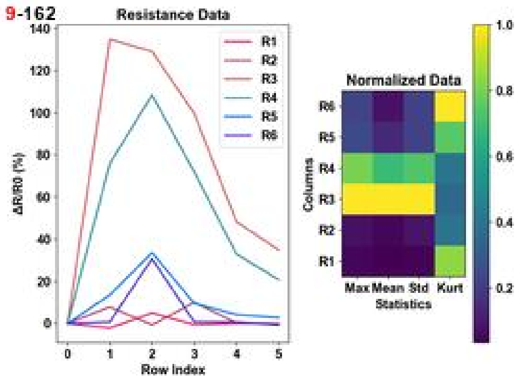

9-163
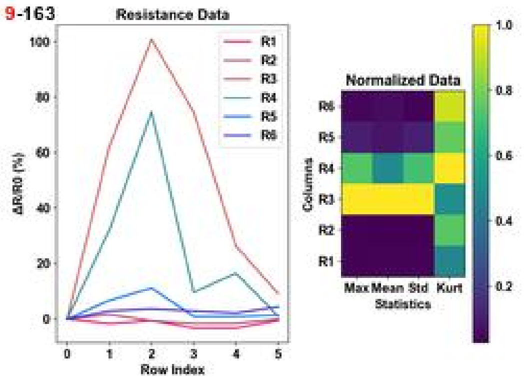

9-164
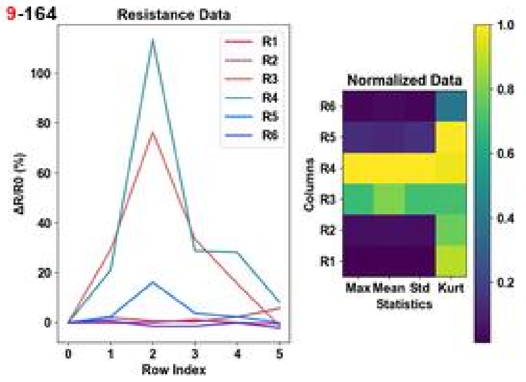

9-165
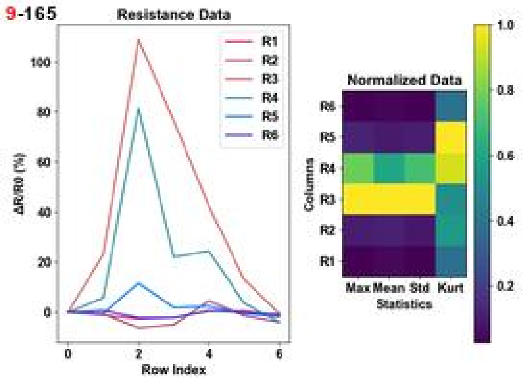

9-166
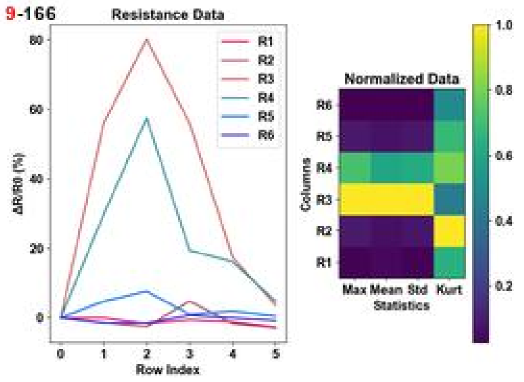

9-167
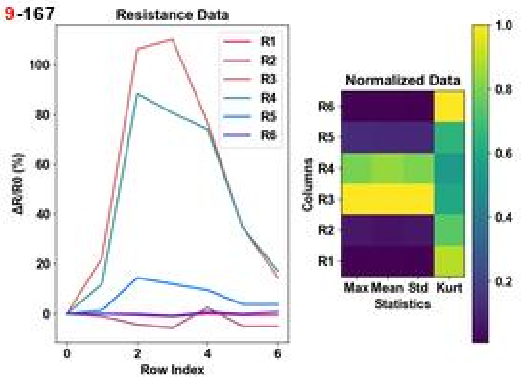

9-168
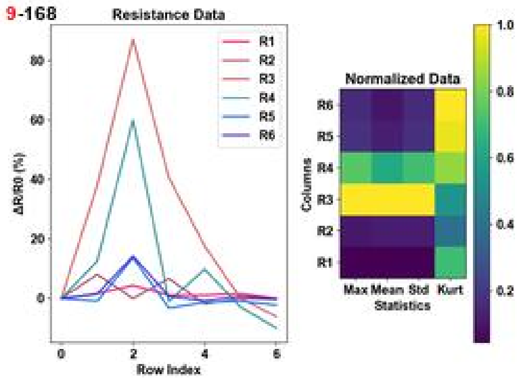

9-169
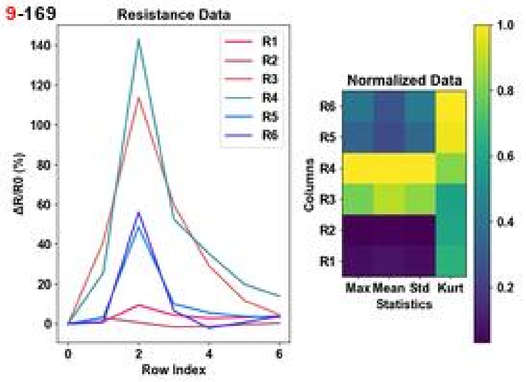

9-170
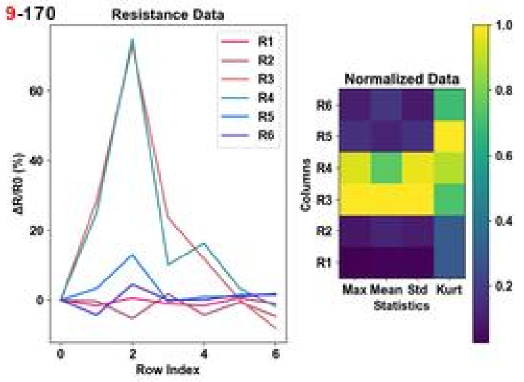

9-171
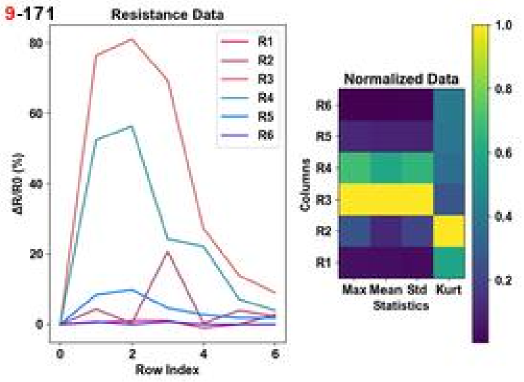

9-172
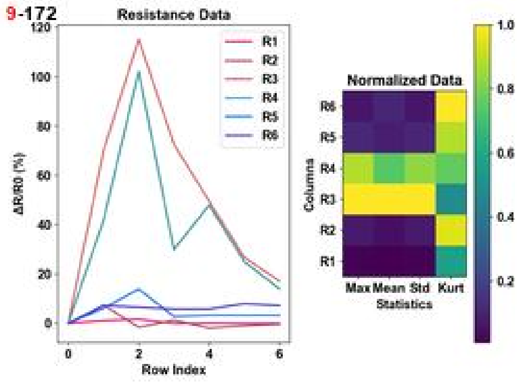

9-173
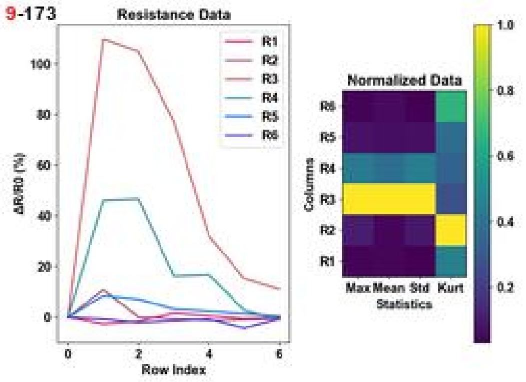

9-174
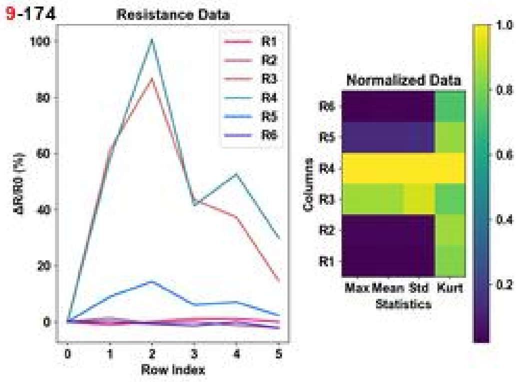

9-175
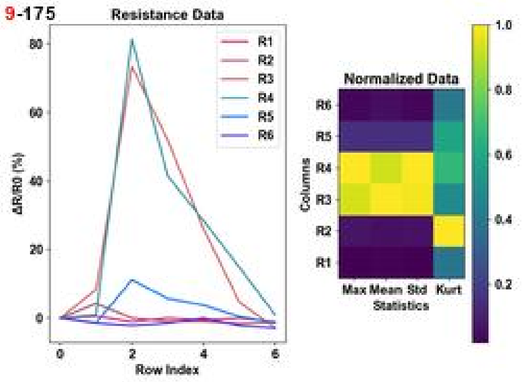

9-176
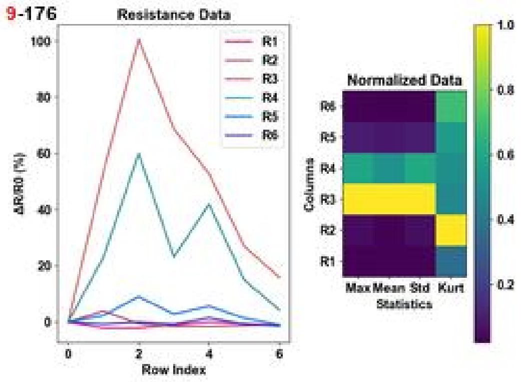

9-177
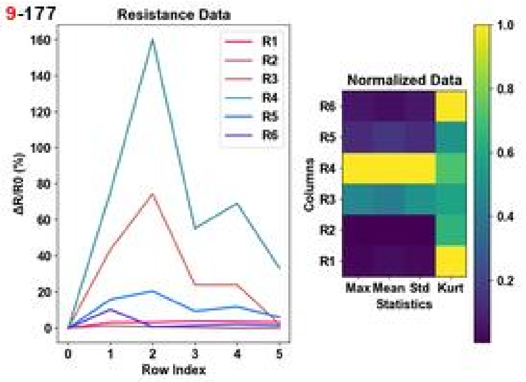

9-178
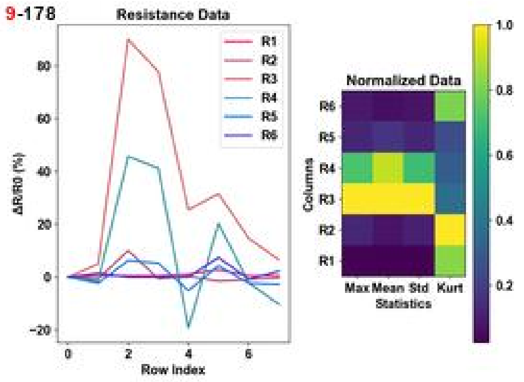

9-179
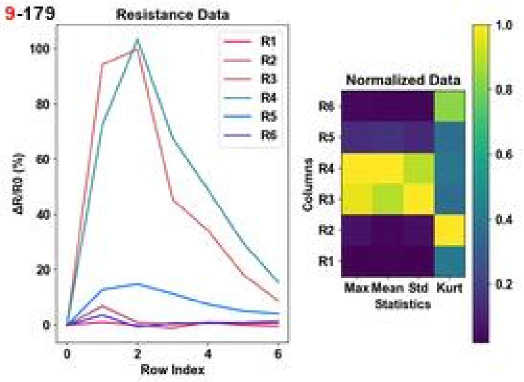

9-180
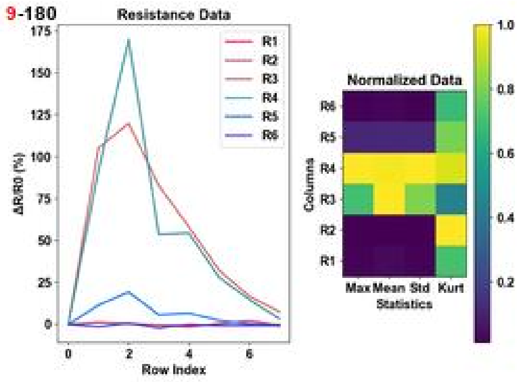

9-181
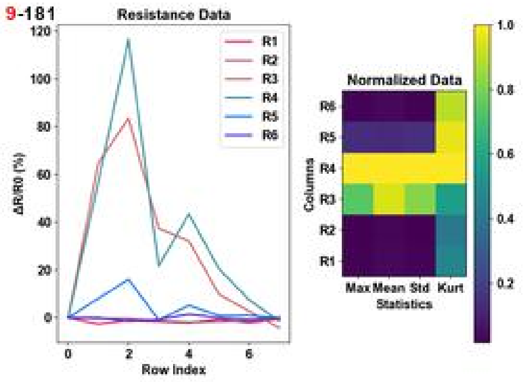

9-182
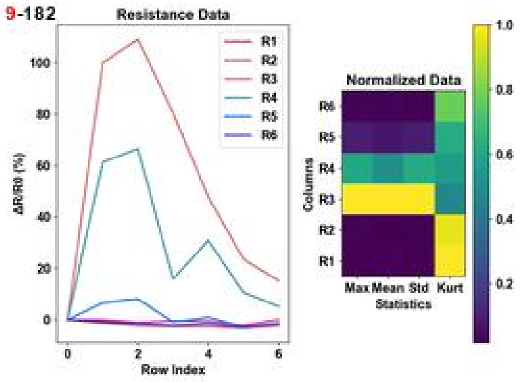

9-183
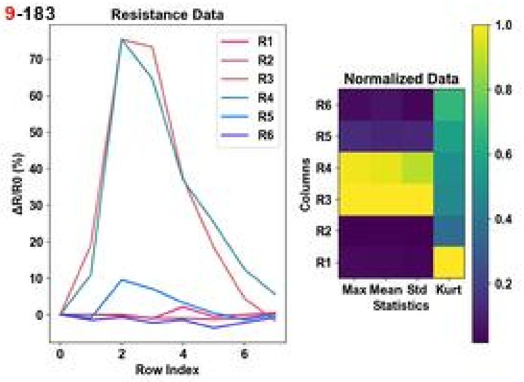

9-184
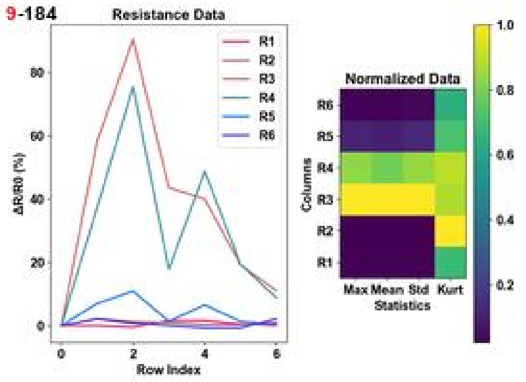

9-185
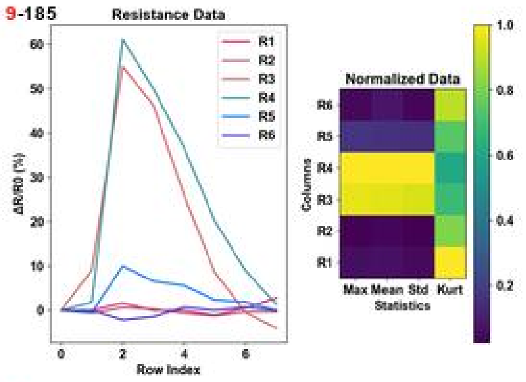

9-186
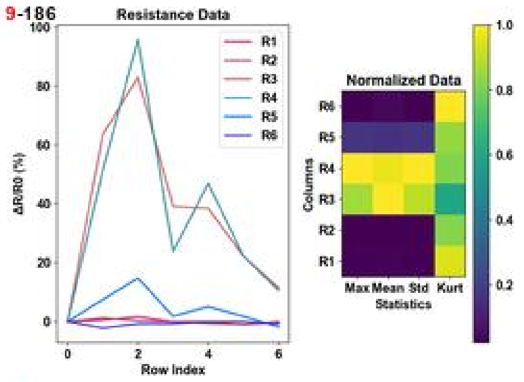

9-187
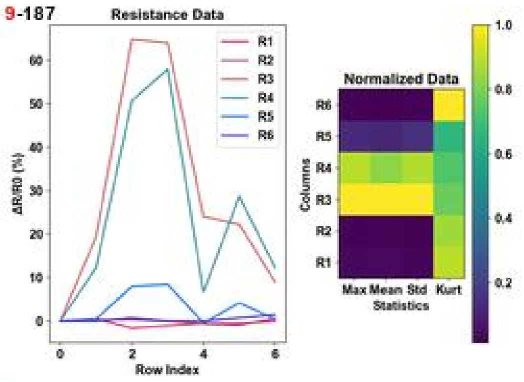

9-188
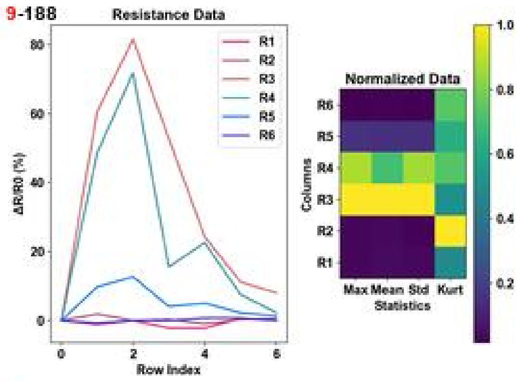

9-189
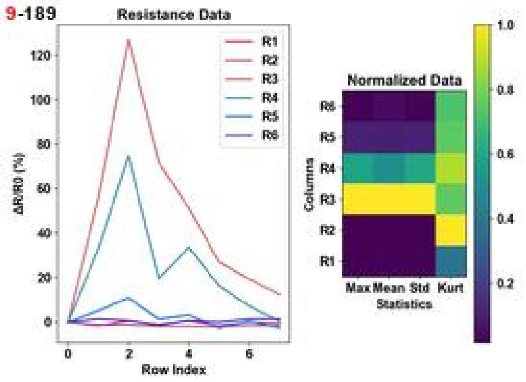

9-190
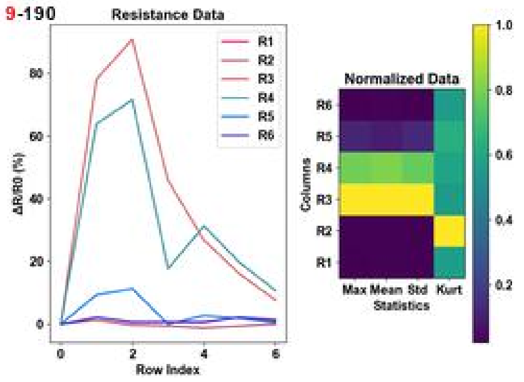

9-191
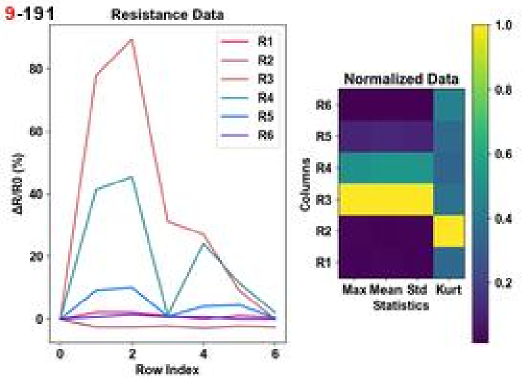

9-192
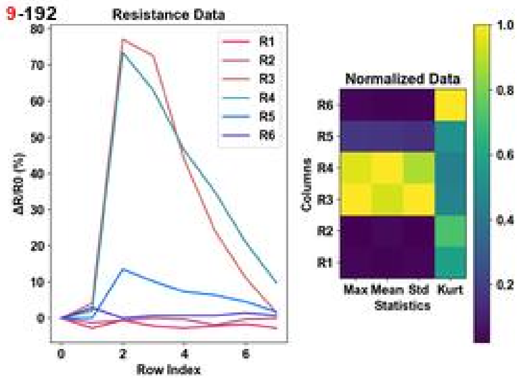

9-193
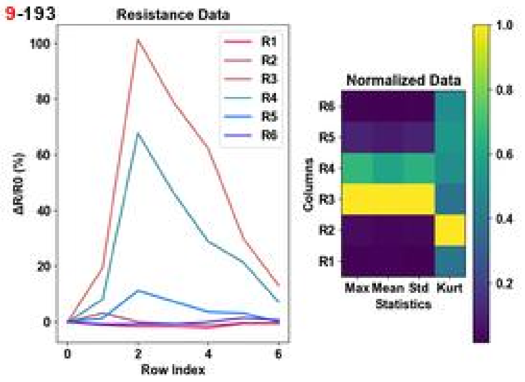

9-194
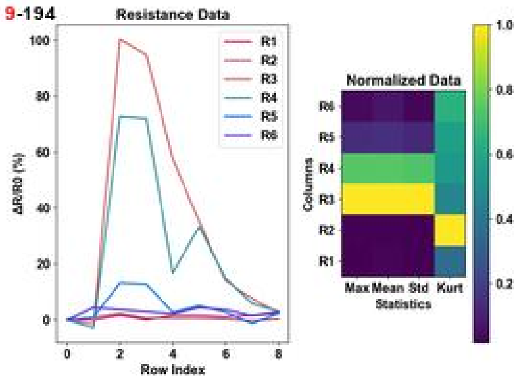

9-195
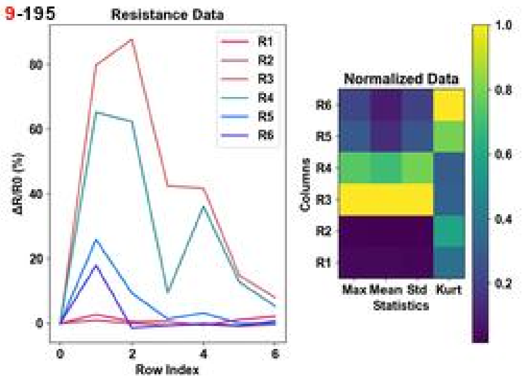

9-196
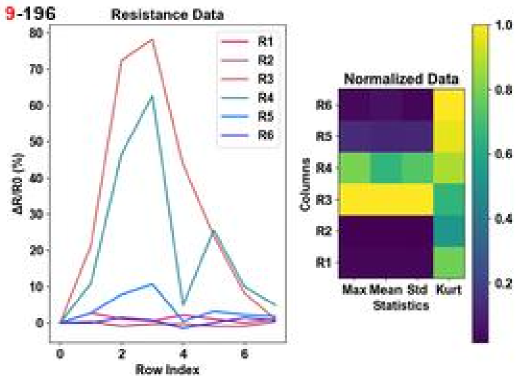

9-197
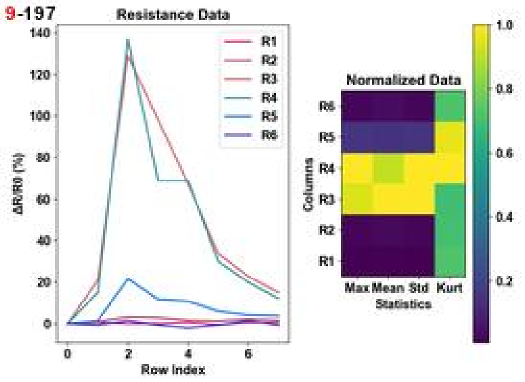

9-198
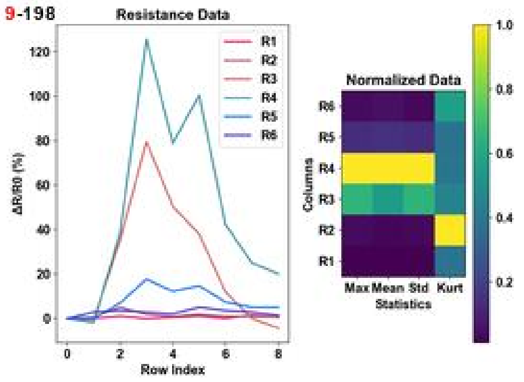

9-199
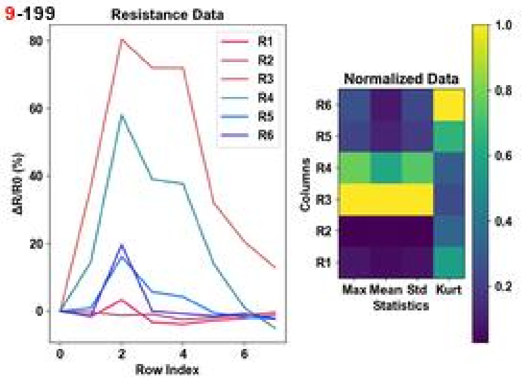

9-200
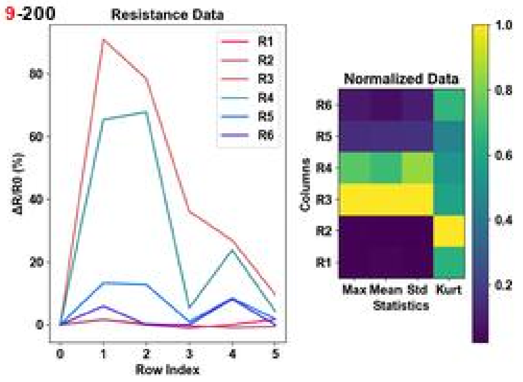

9-201
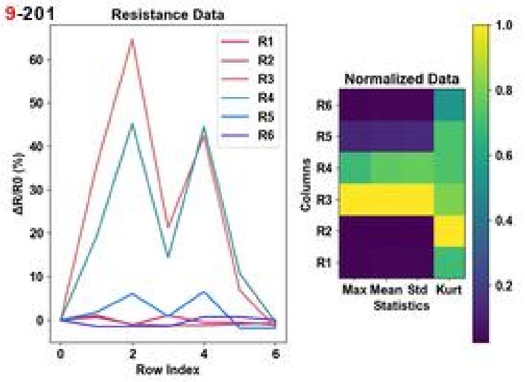

9-202
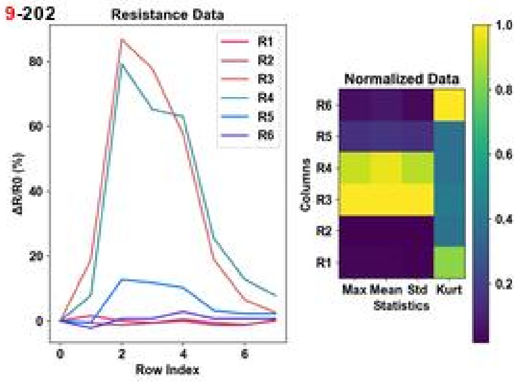

9-203
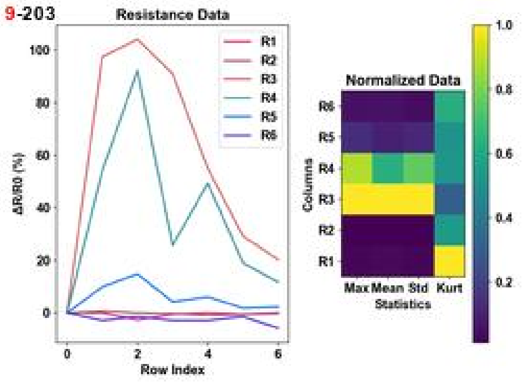

9-204
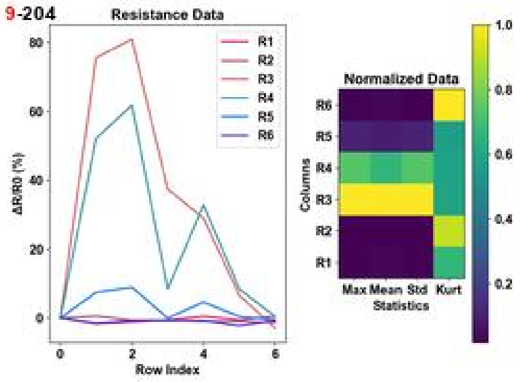

9-205
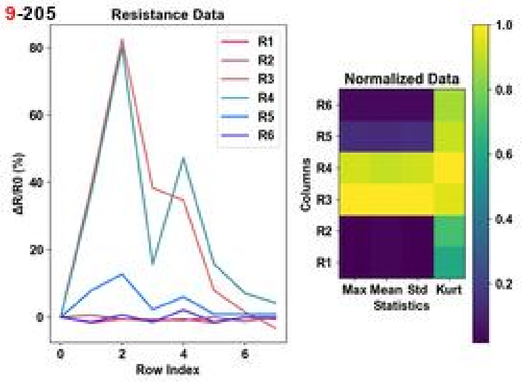

9-206
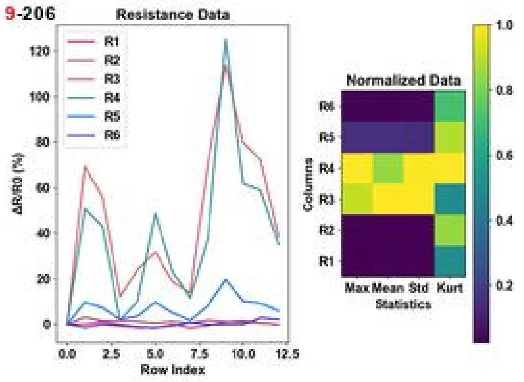

9-207
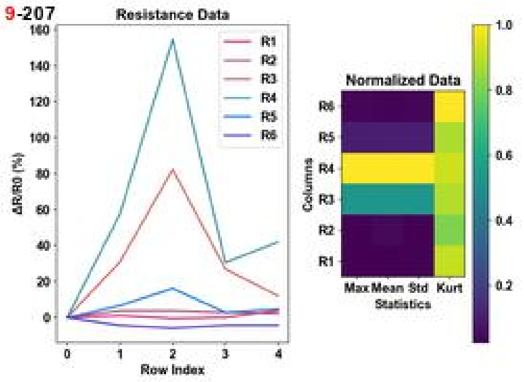

9-208
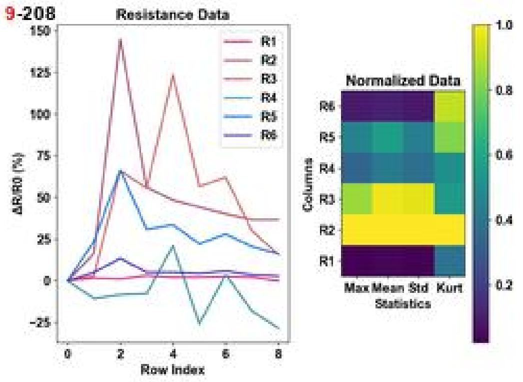

9-209
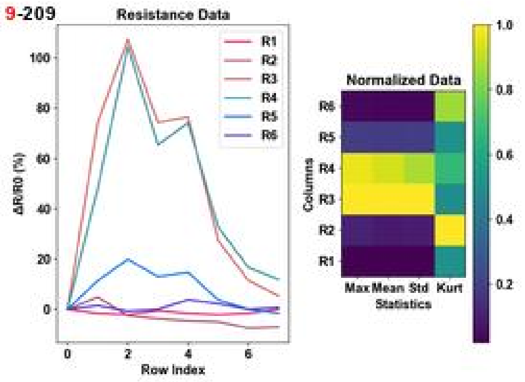

9-210
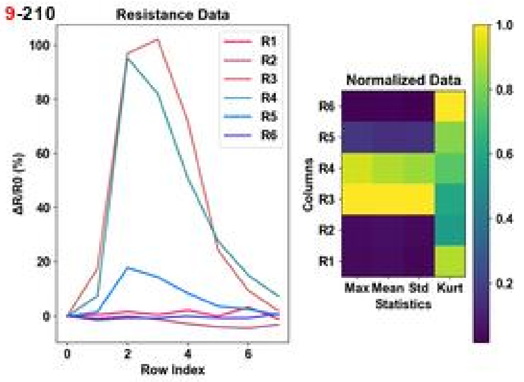

9-211
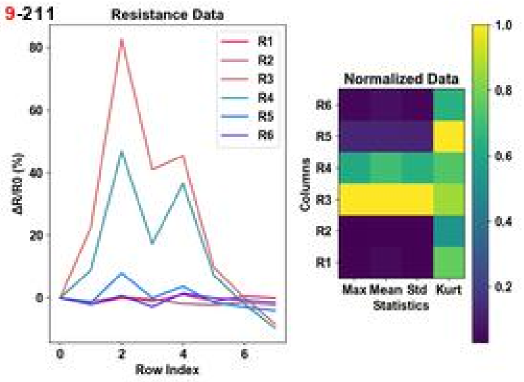

9-212
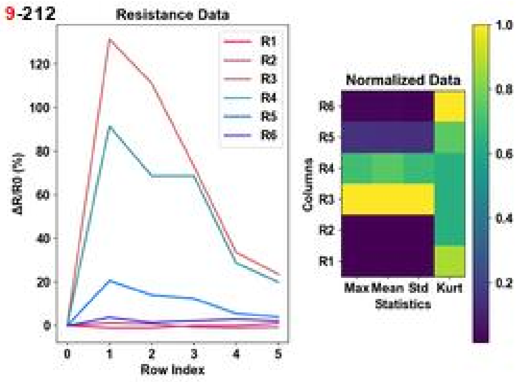

9-213
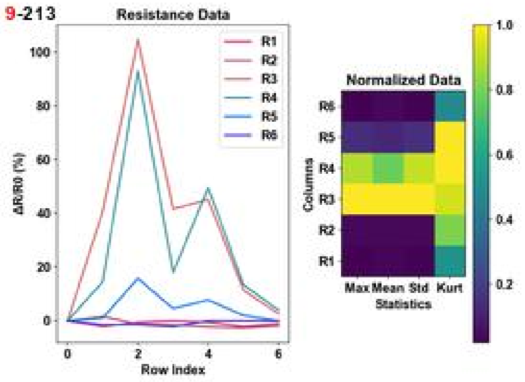

9-214
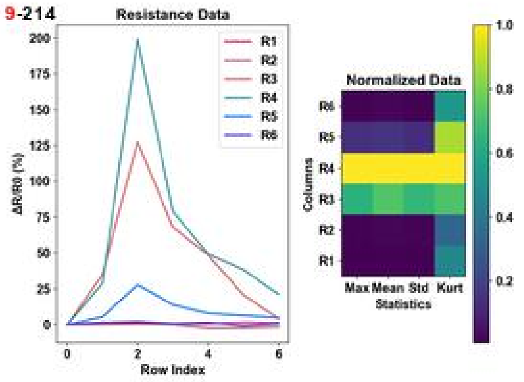

9-215
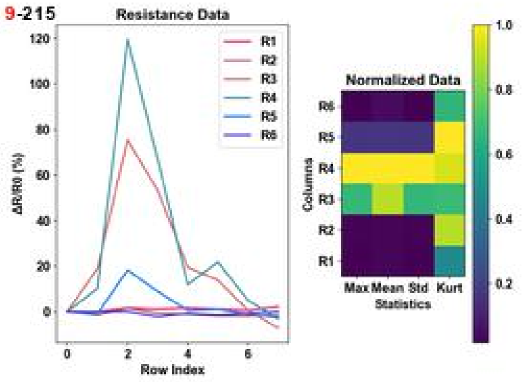

9-216
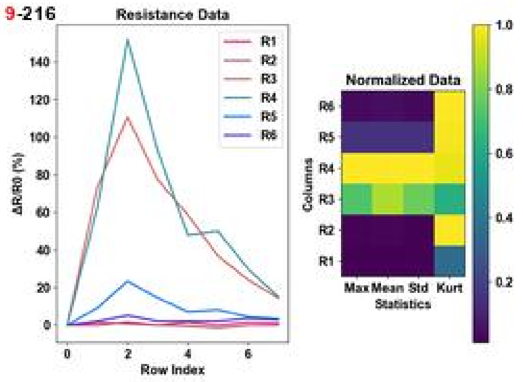

9-217
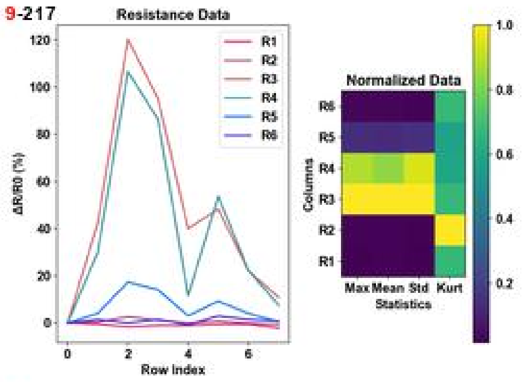

9-218
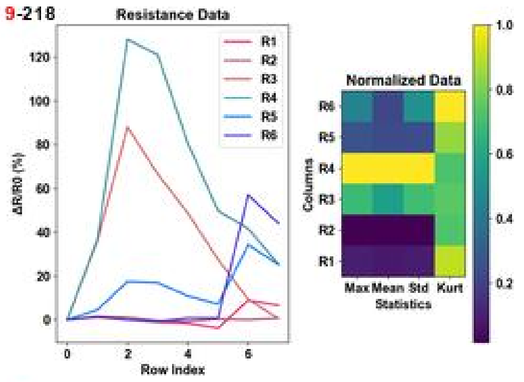

9-219
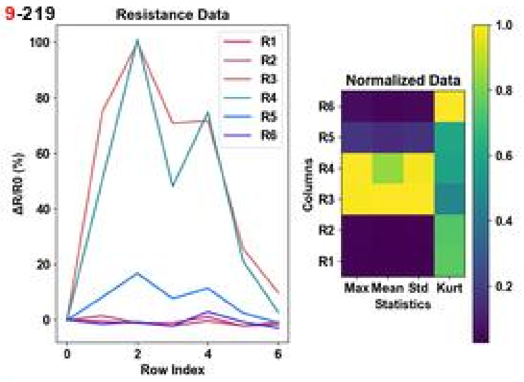

9-220
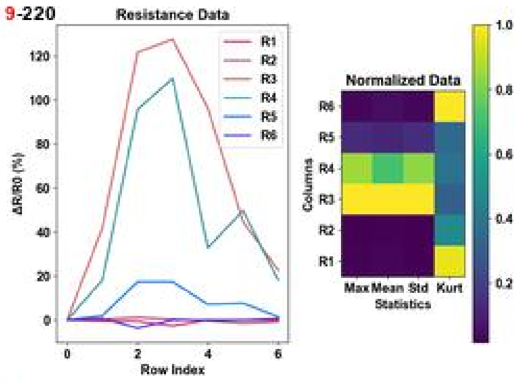

9-221
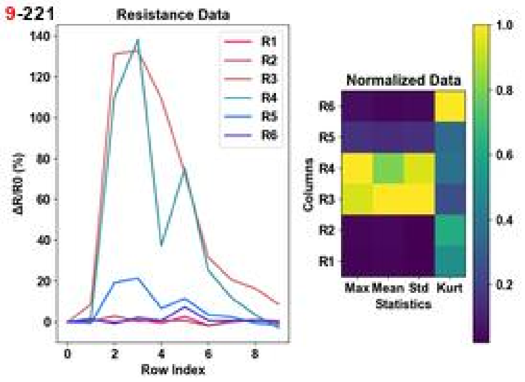

9-222
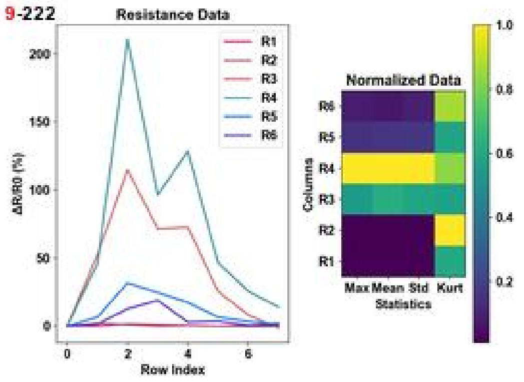

9-223
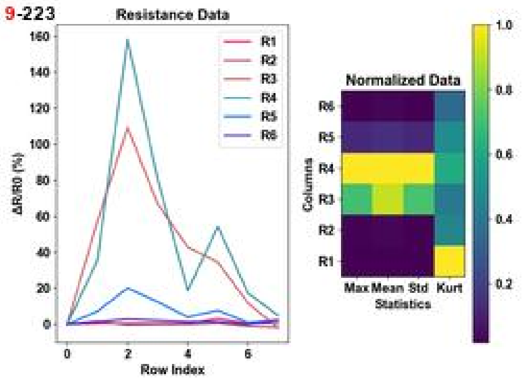

9-224
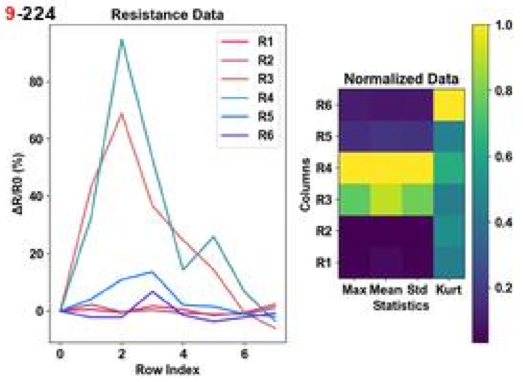

Pressure-sensitive data of type 9

9-225
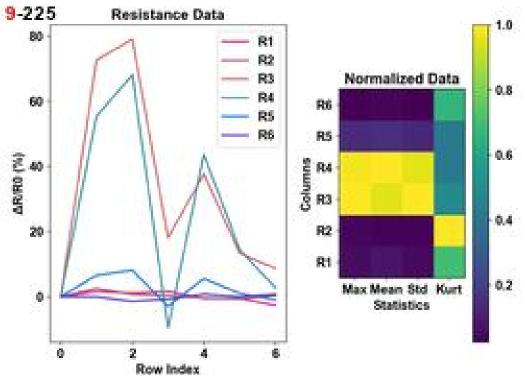

9-226
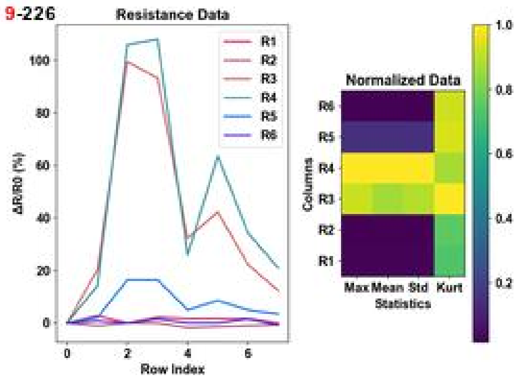

9-227
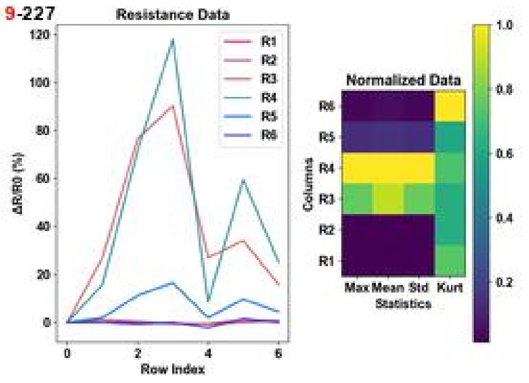

9-228
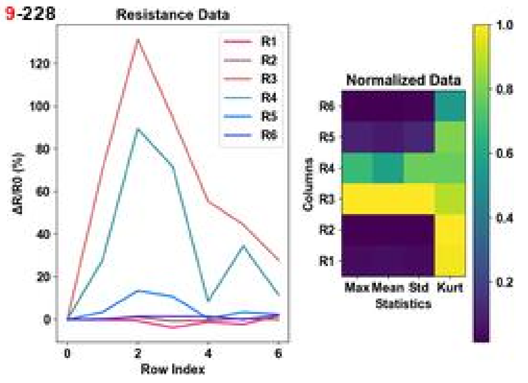

9-229
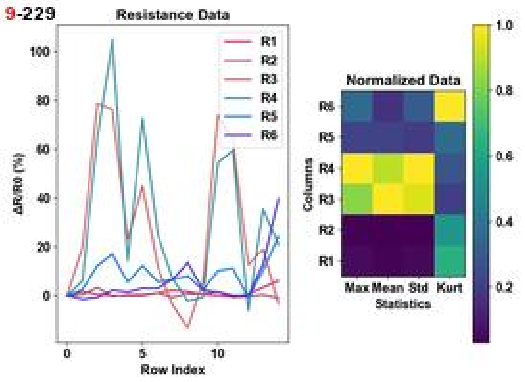

9-230
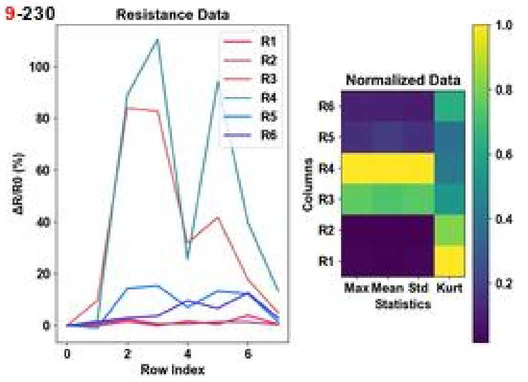

9-231
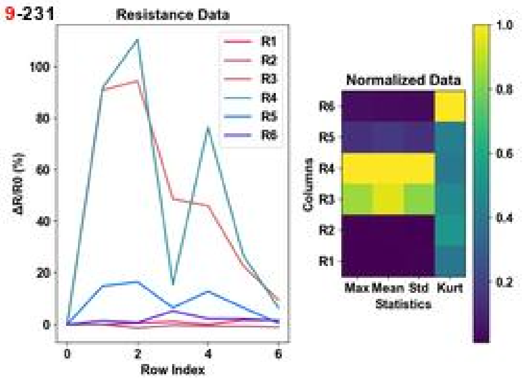

9-232
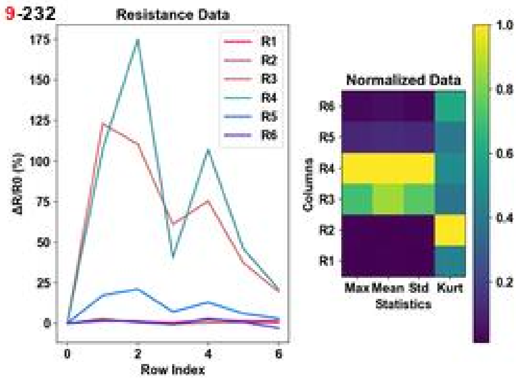

9-233
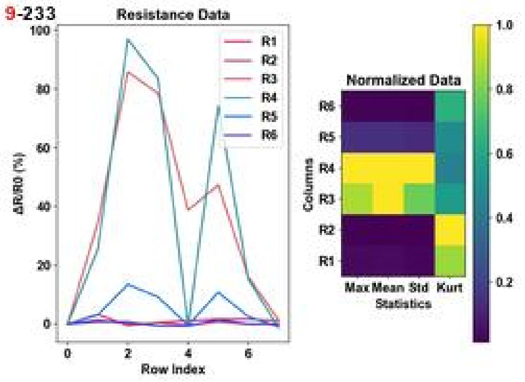

9-234
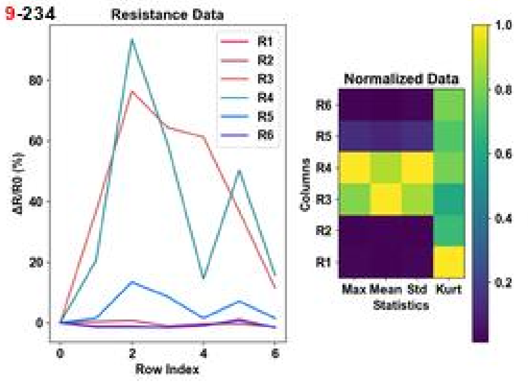

9-235
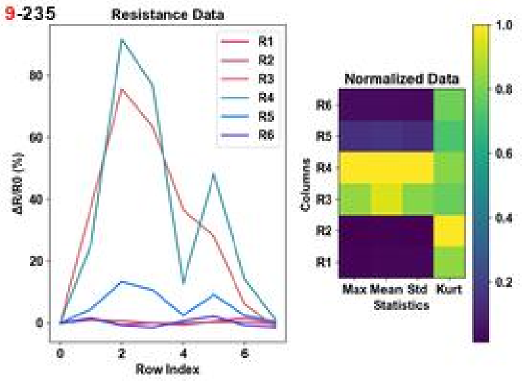

9-236
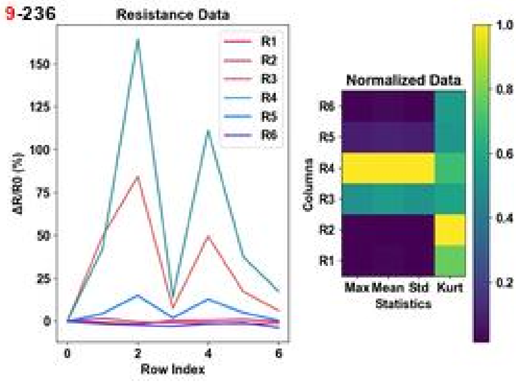

9-237
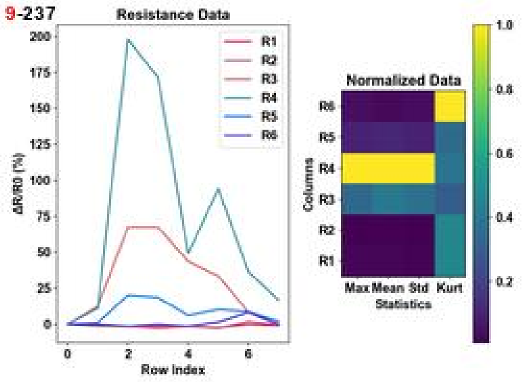

9-238
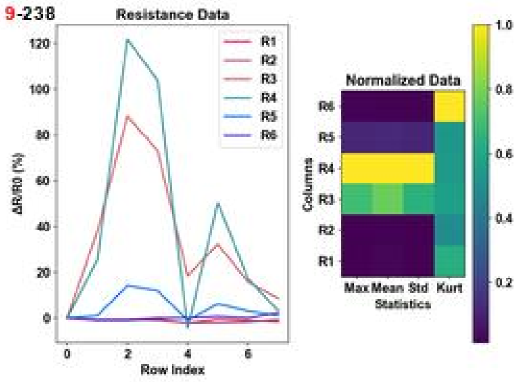

9-239
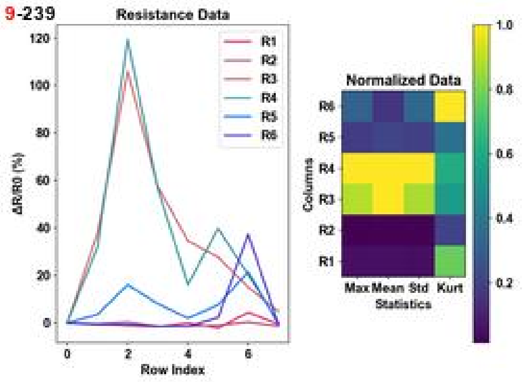

9-240
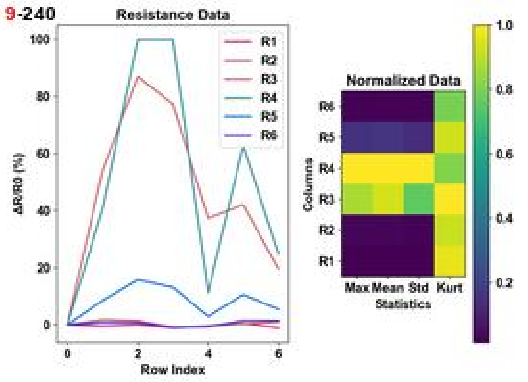

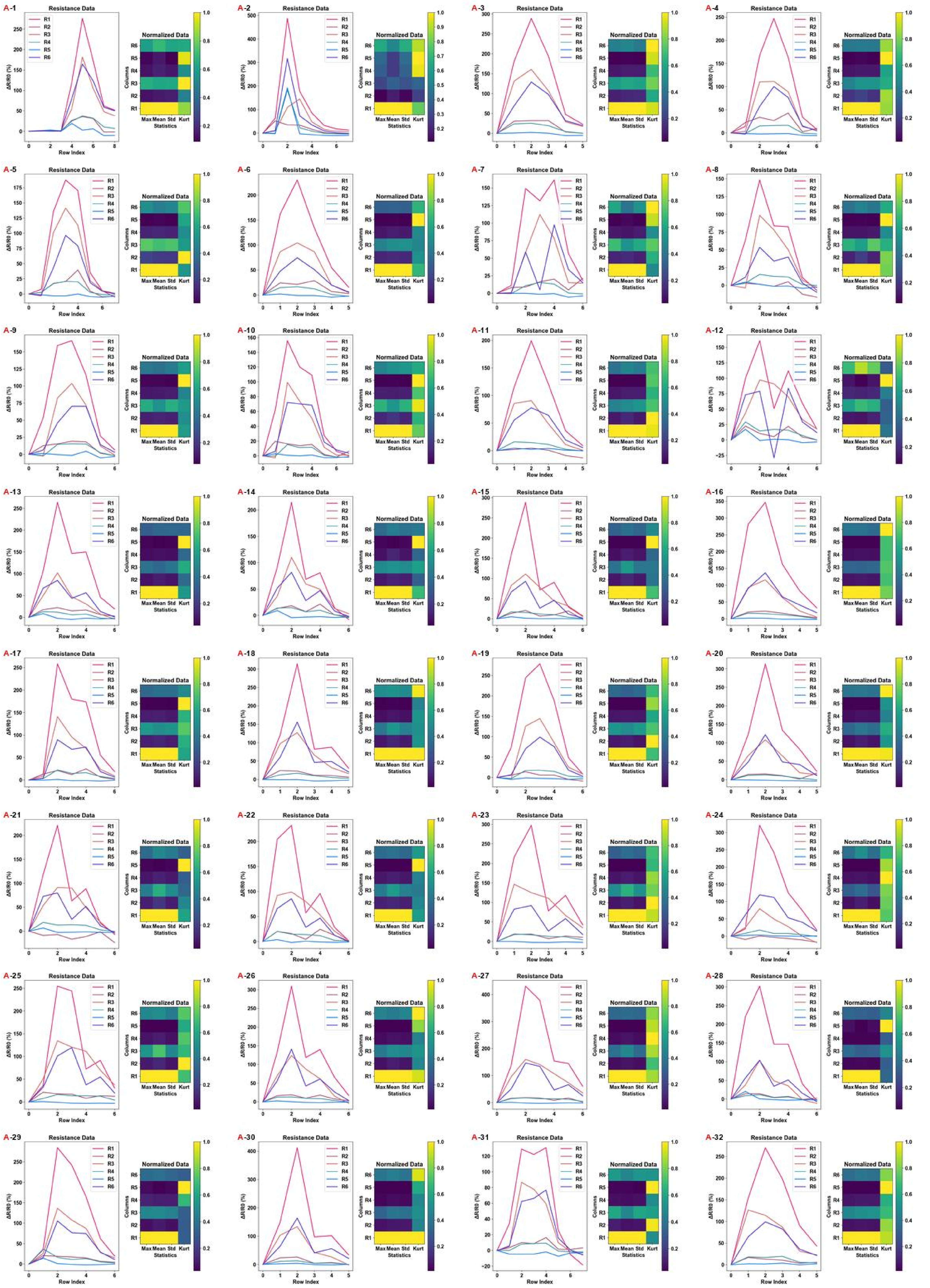
A-1
A-2
A-3
A-4
A-5
A-6
A-7
A-8
A-9
A-10
A-11
A-12
A-13
A-14
A-15
A-16
A-17
A-18
A-19
A-20
A-21
A-22
A-23
A-24
A-25
A-26
A-27
A-28
A-29
A-30
A-31
A-32
Resistance Data
ΔR/R0 (%)
Row Index
R1
R2
R3
R4
R5
R6
Normalized Data
Columns
Max Mean Std Kurt
Statistics

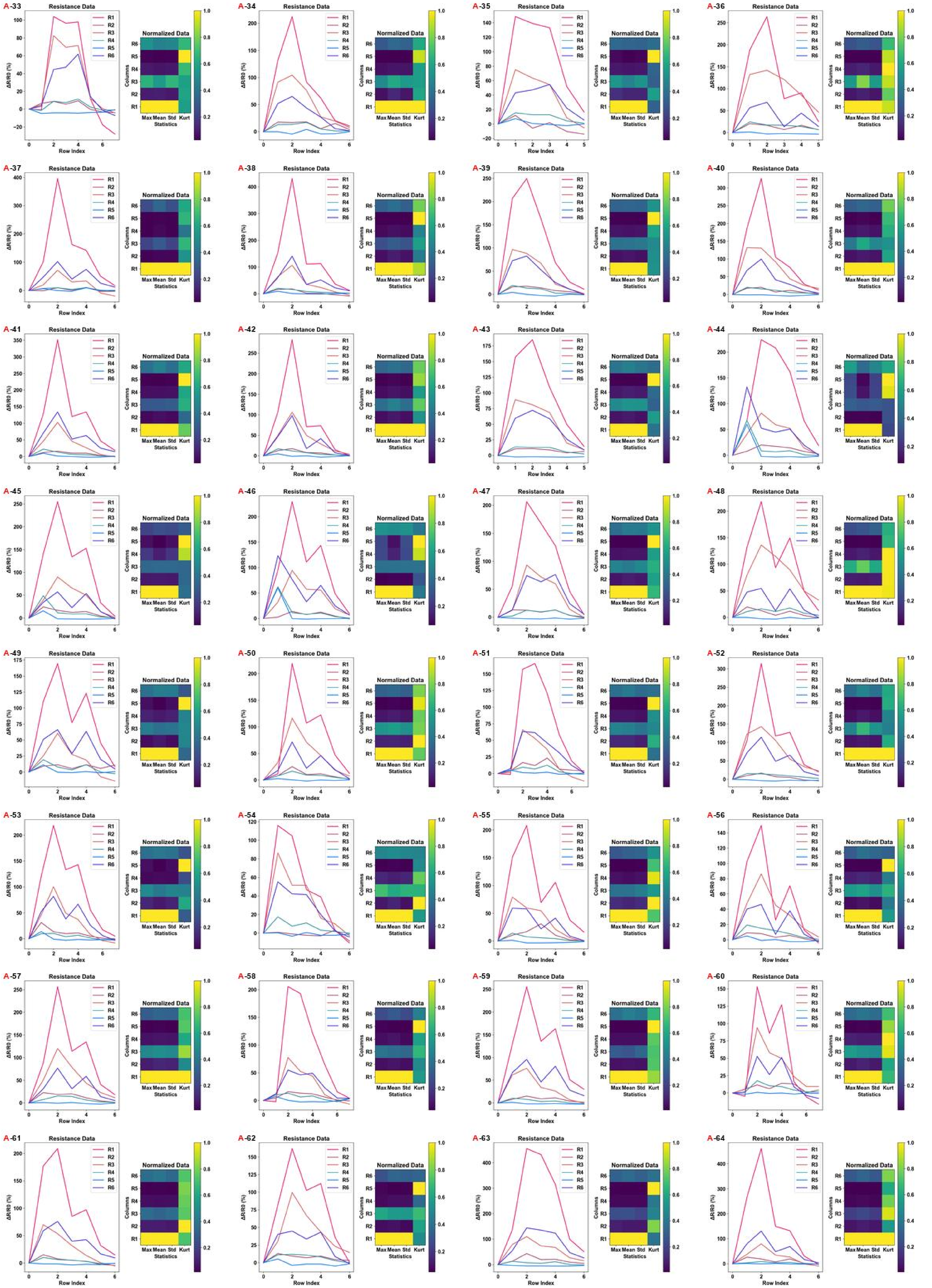
A-33
A-34
A-35
A-36
A-37
A-38
A-39
A-40
A-41
A-42
A-43
A-44
A-45
A-46
A-47
A-48
A-49
A-50
A-51
A-52
A-53
A-54
A-55
A-56
A-57
A-58
A-59
A-60
A-61
A-62
A-63
A-64
Resistance Data
Normalized Data
ΔR/R0 (%)
Row Index
Columns
Statistics
Max Mean Std Kurt
R1
R2
R3
R4
R5
R6

A-65
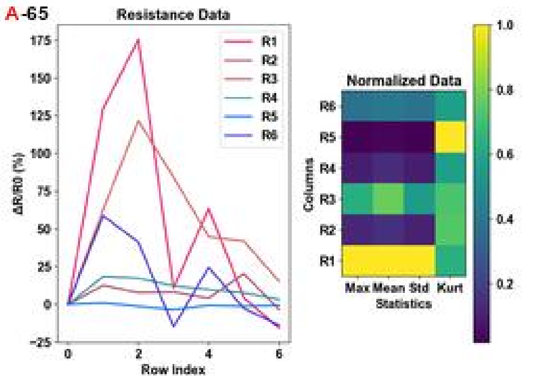
A-66
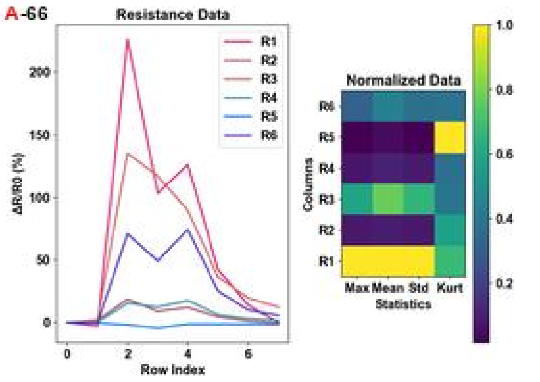
A-67
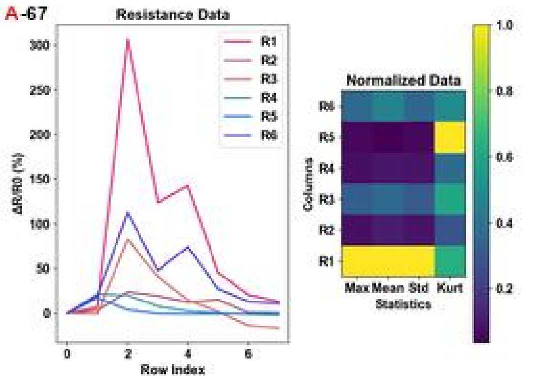
A-68
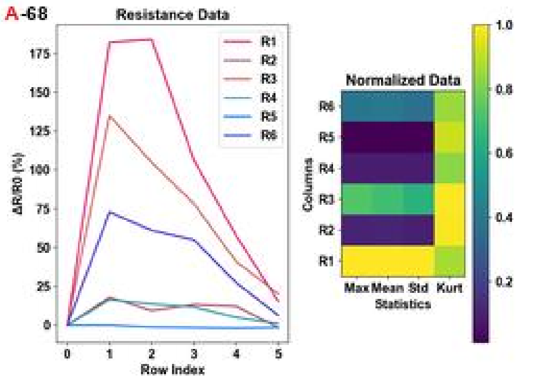
A-69
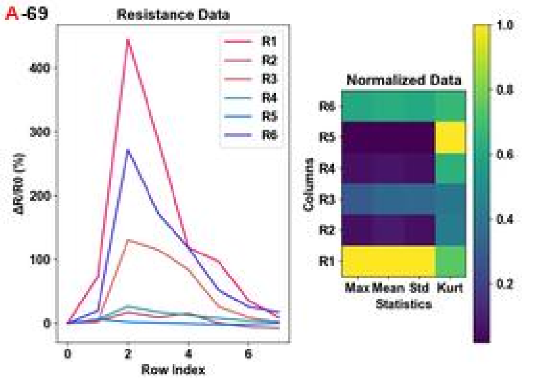
A-70
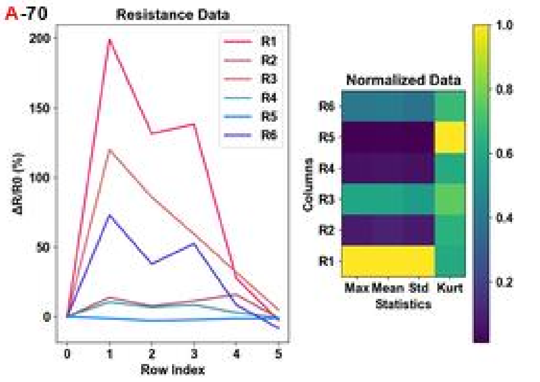
A-71
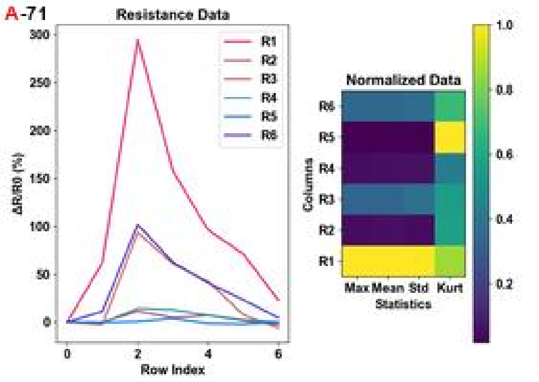
A-72
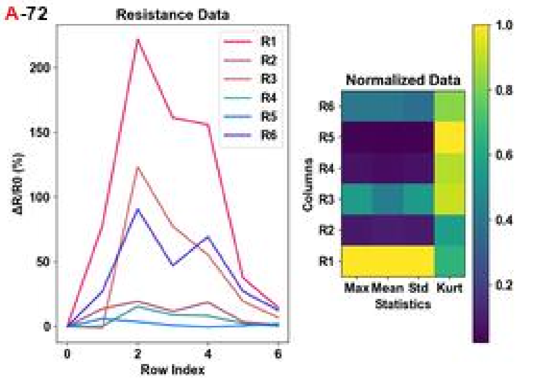
A-73
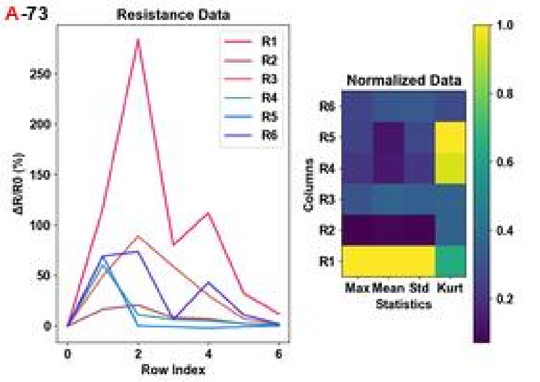
A-74
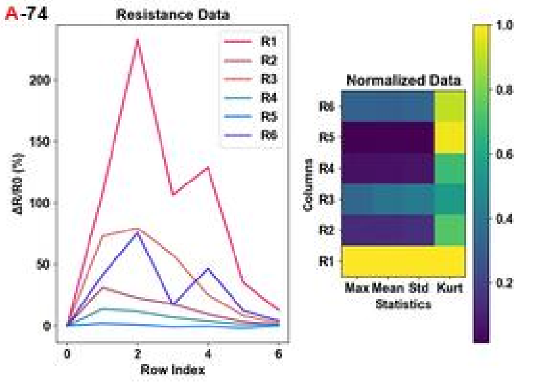
A-75
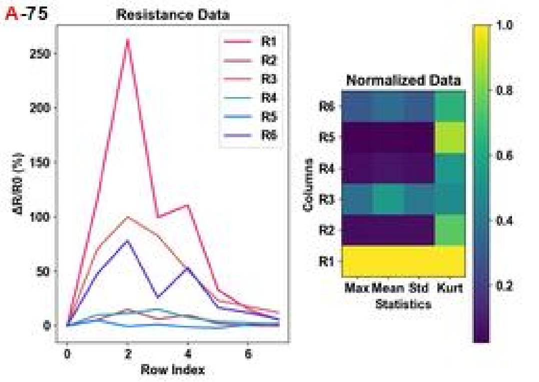
A-76
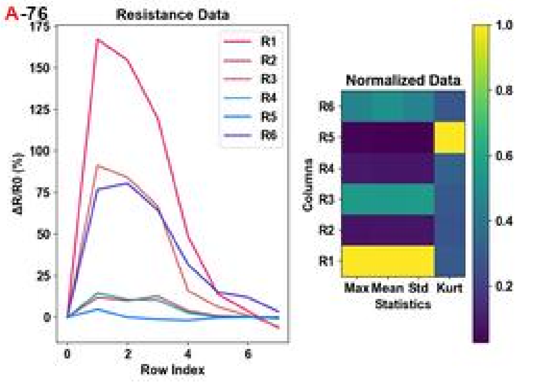
A-77
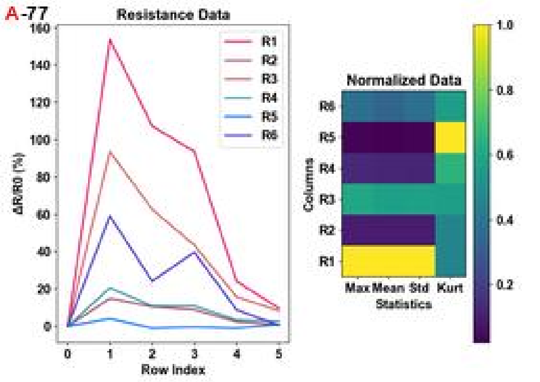
A-78
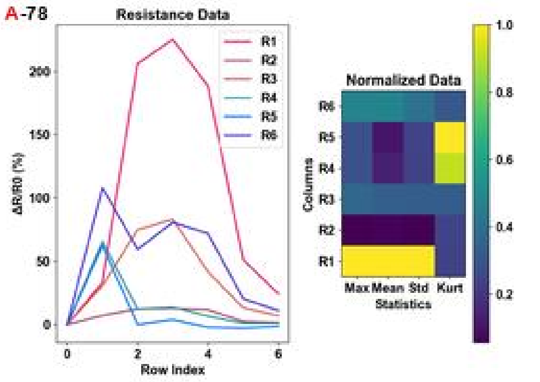
A-79
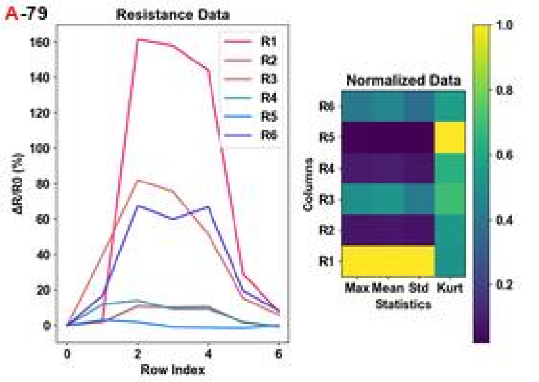
A-80
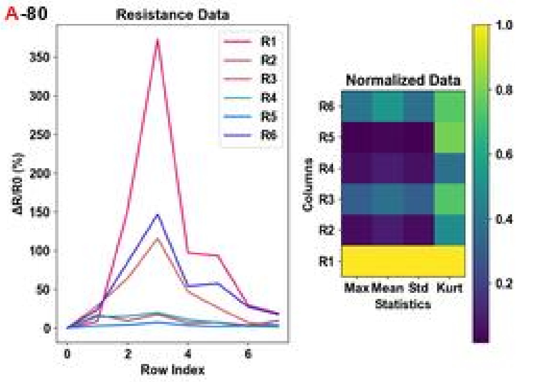
A-81
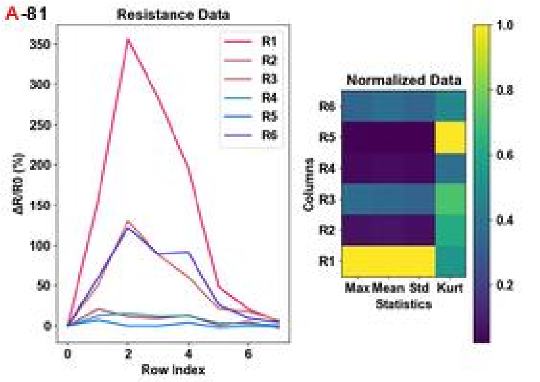
A-82
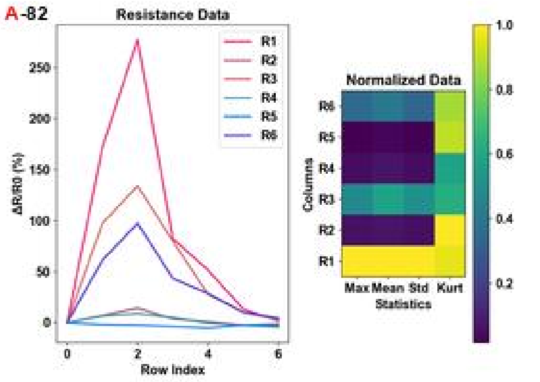
A-83
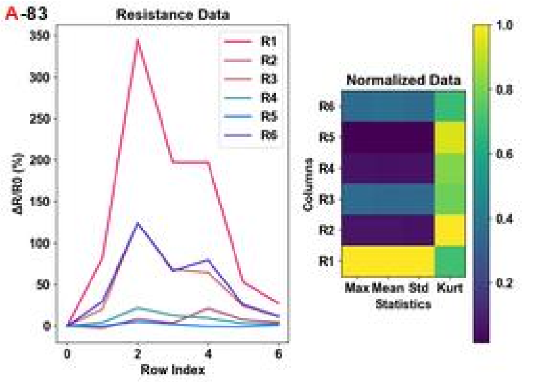
A-84
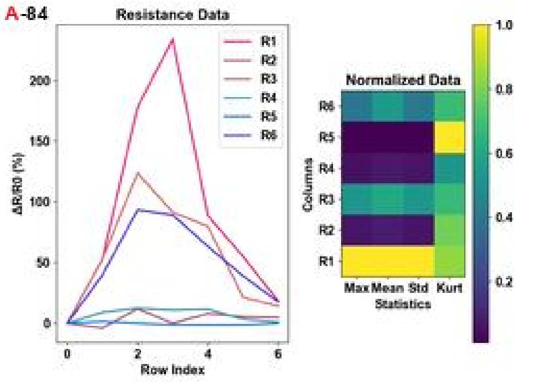
A-85
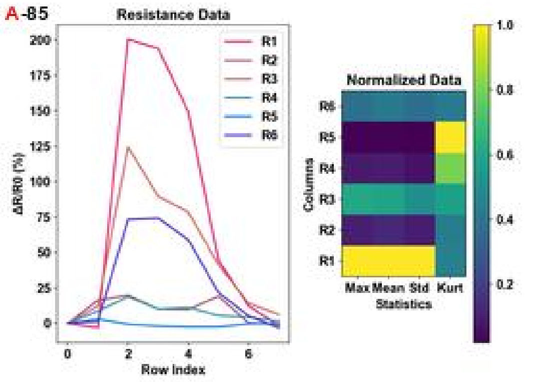
A-86
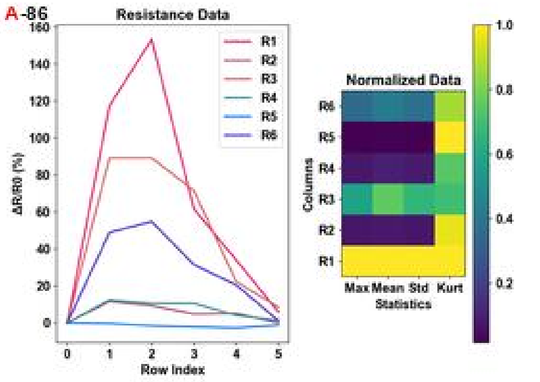
A-87
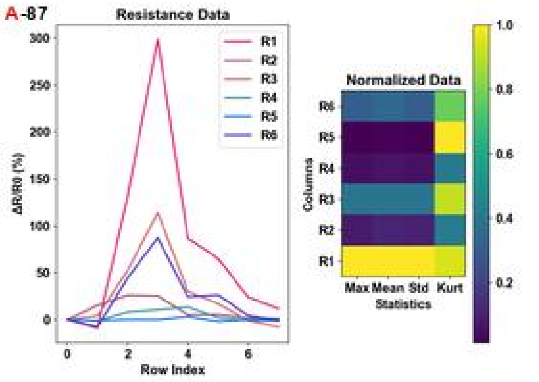
A-88
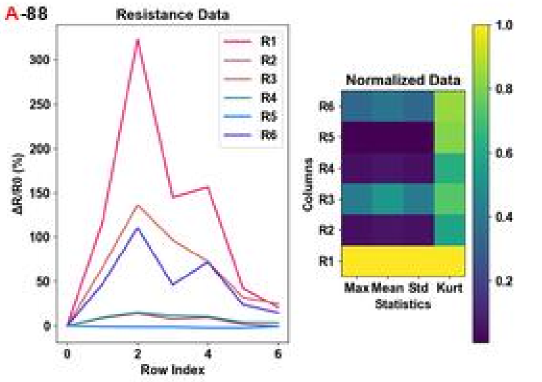
A-89
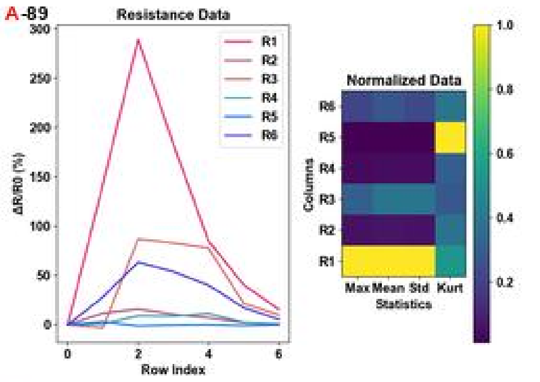
A-90
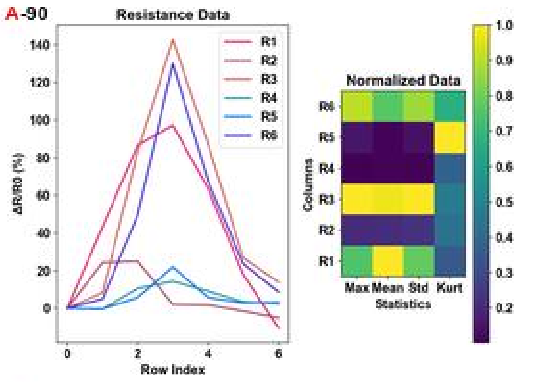
A-91
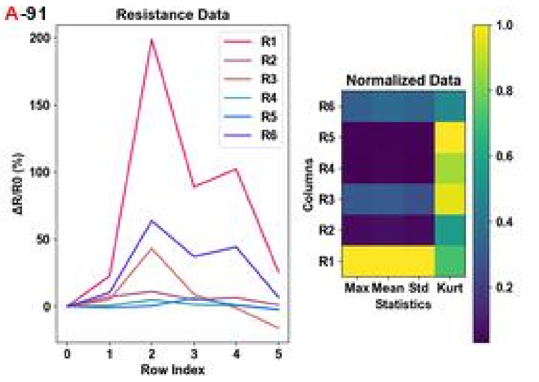
A-92
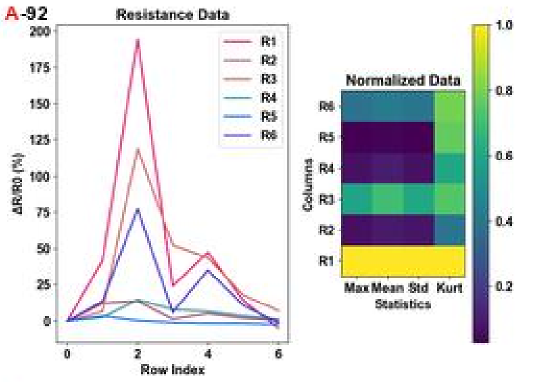
A-93
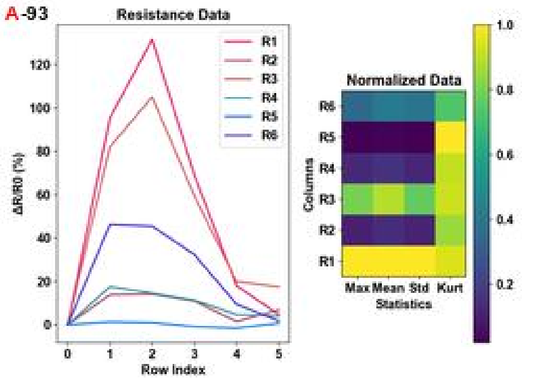
A-94
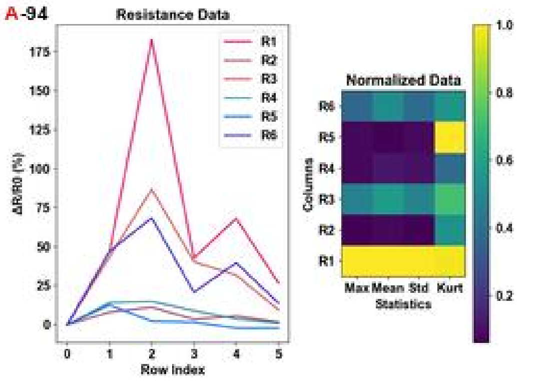
A-95
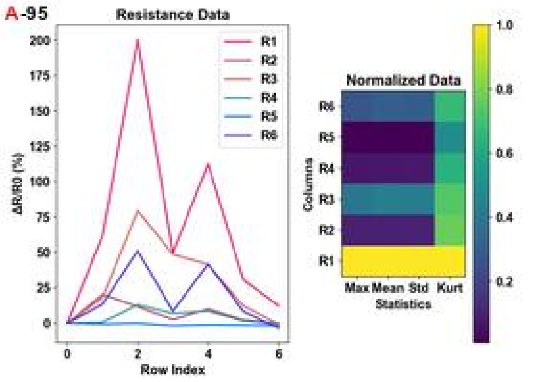
A-96
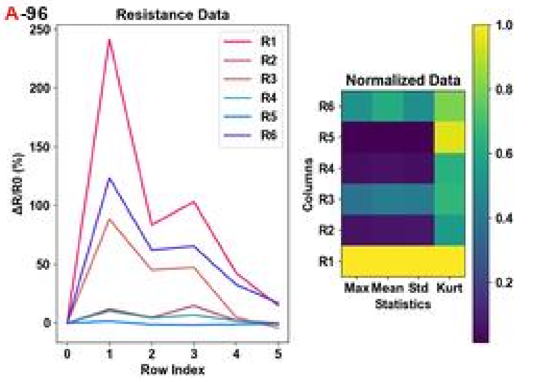

A-97
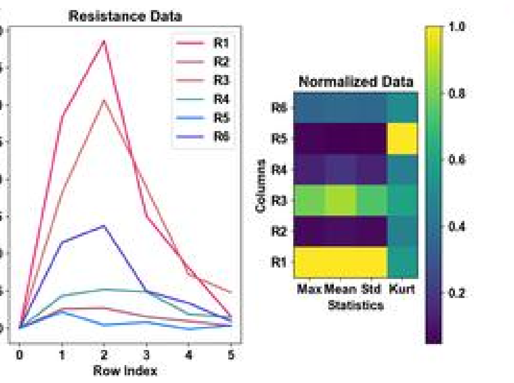
A-98
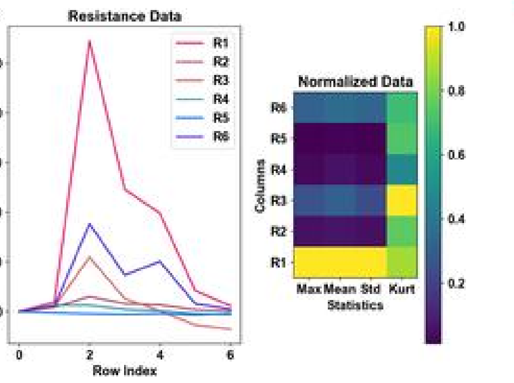
A-99
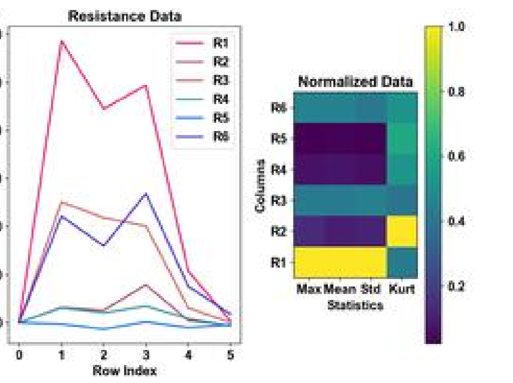
A-100
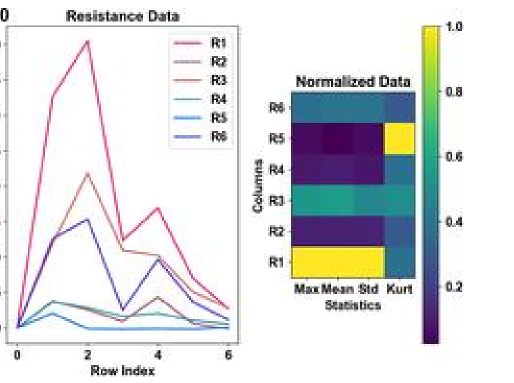
A-101
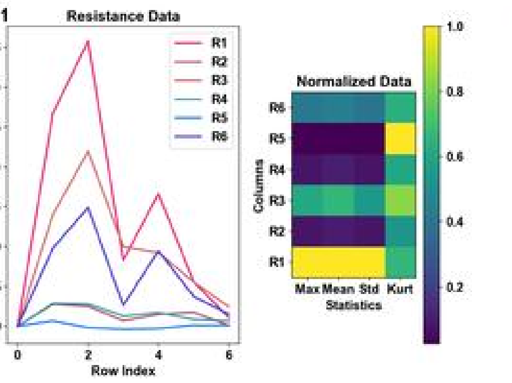
A-102
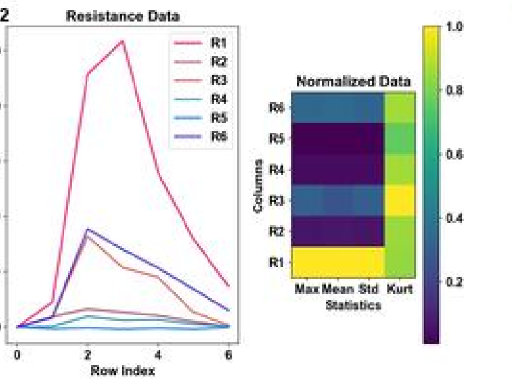
A-103
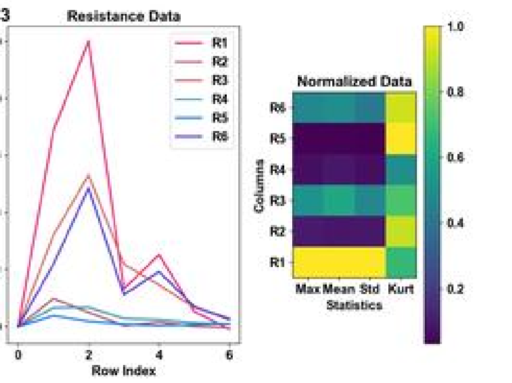
A-104
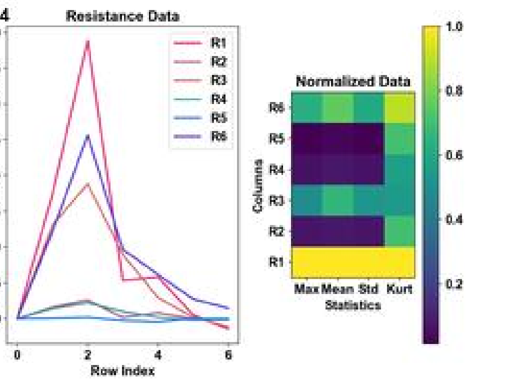
A-105
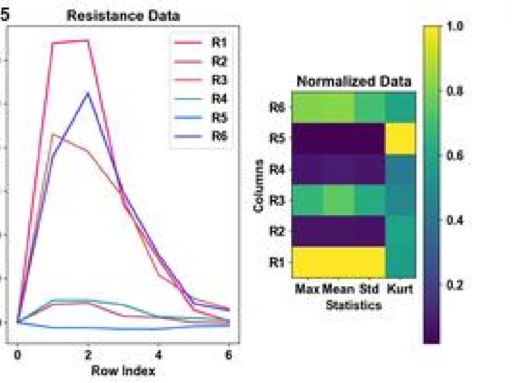
A-106
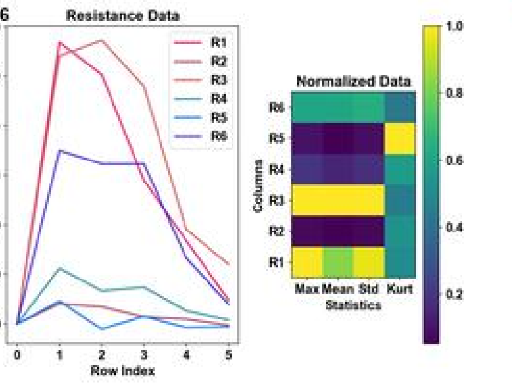
A-107
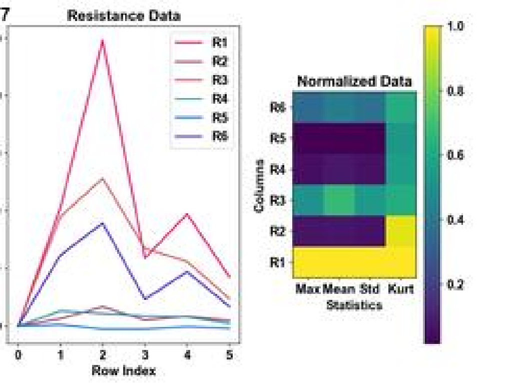
A-108
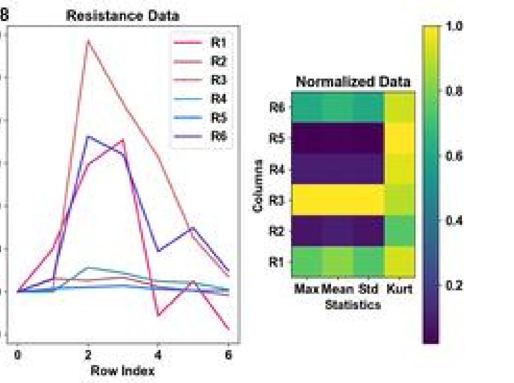
A-109
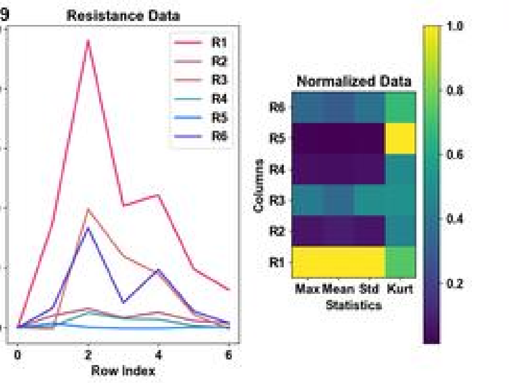
A-110
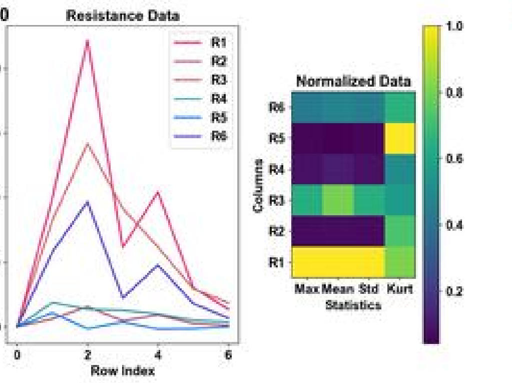
A-111
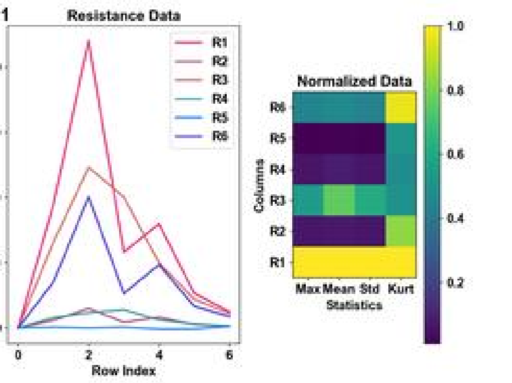
A-112
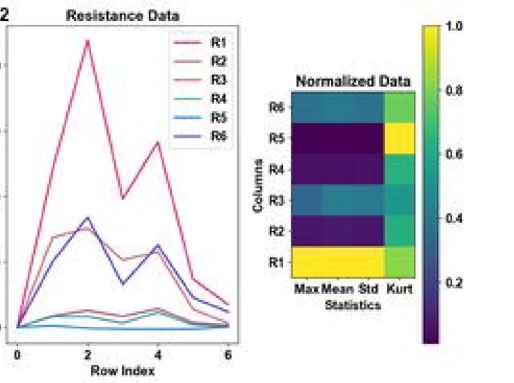
A-113
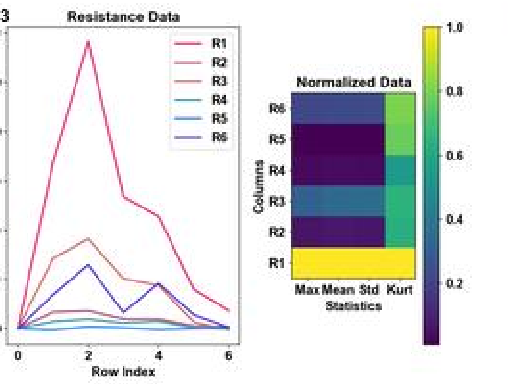
A-114
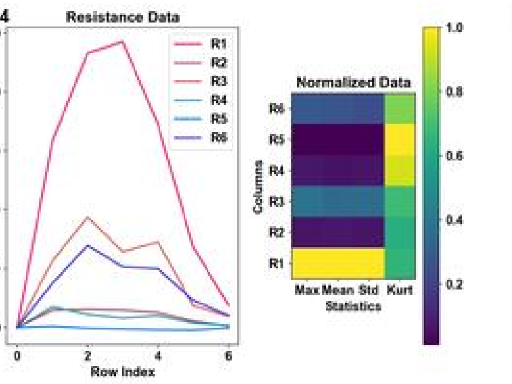
A-115
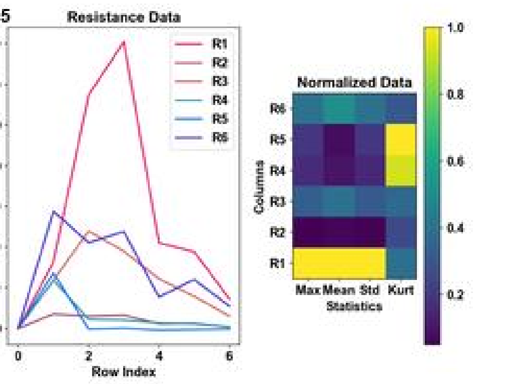
A-116
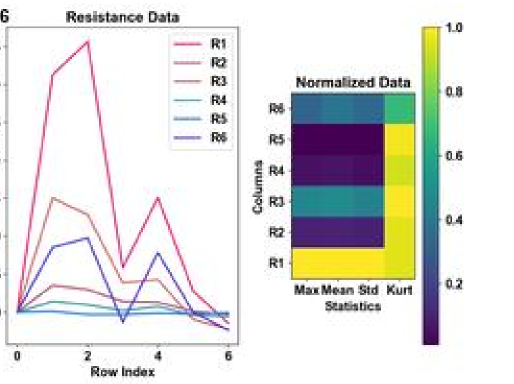
A-117
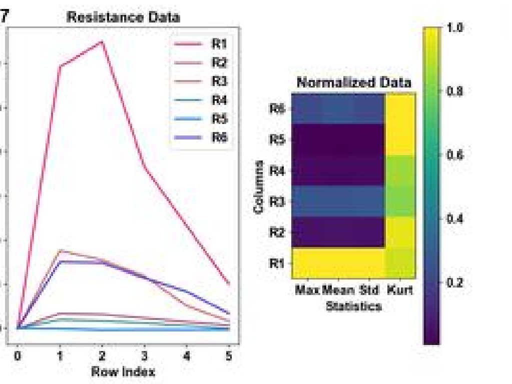
A-118
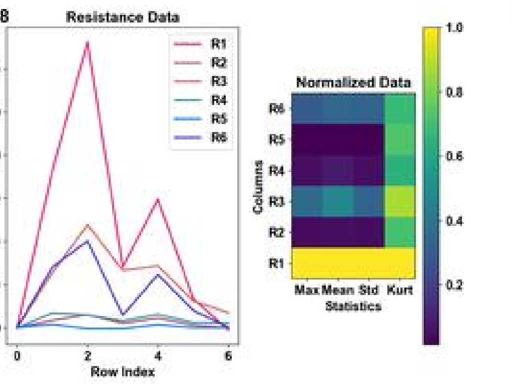
A-119
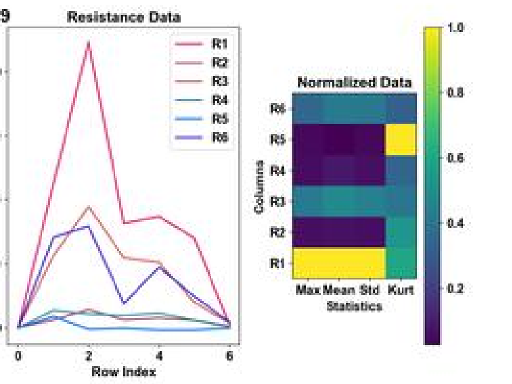
A-120
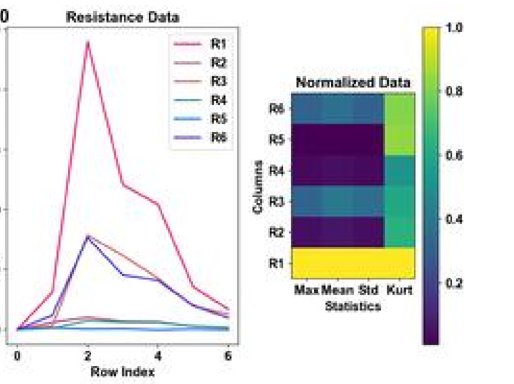
A-121
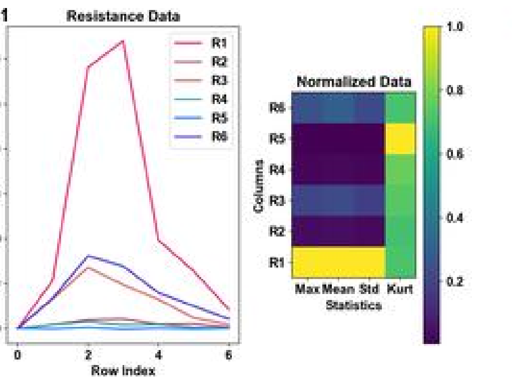
A-122
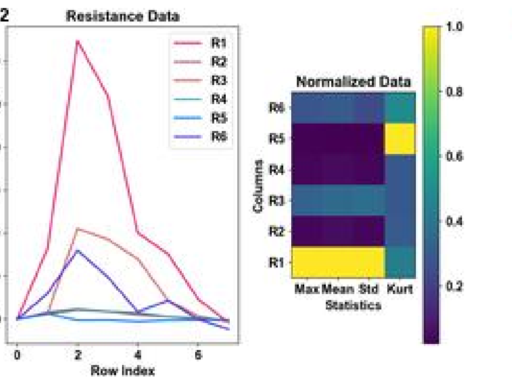
A-123
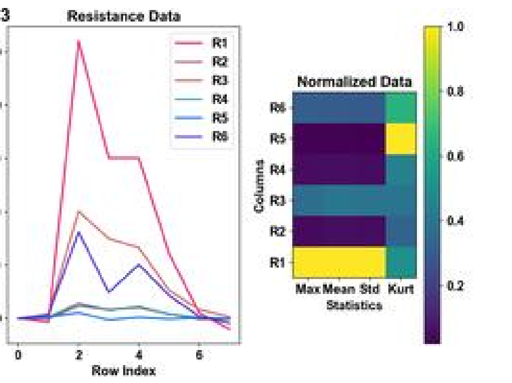
A-124
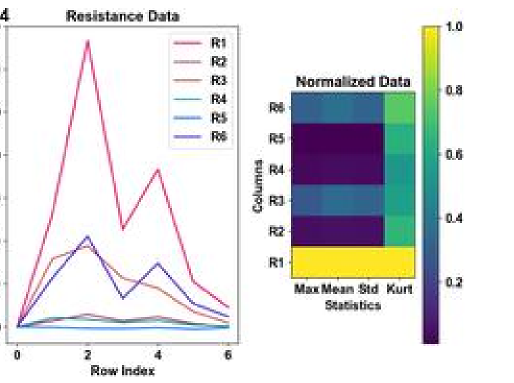
A-125
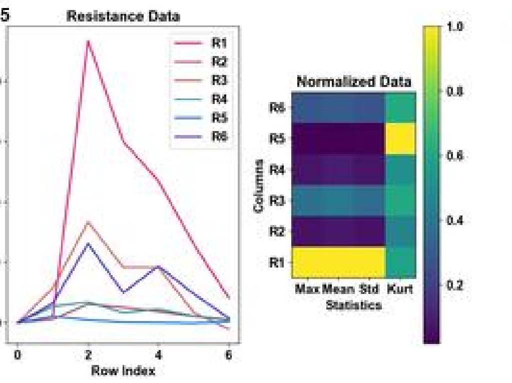
A-126
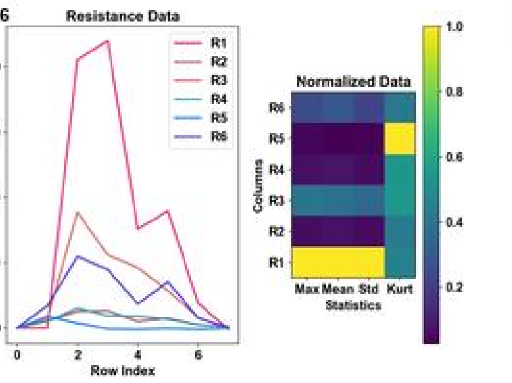
A-127
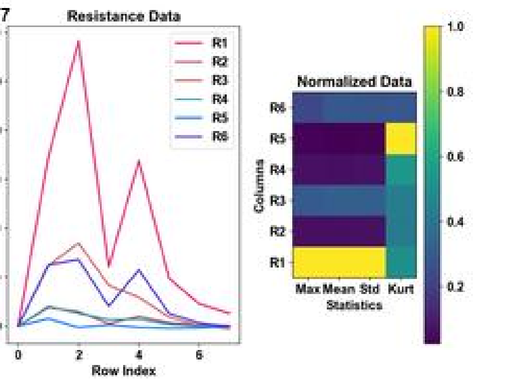
A-128
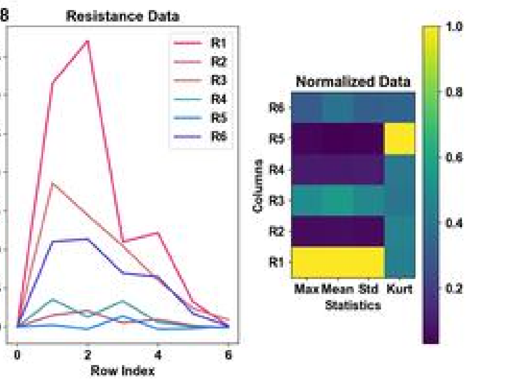

A-129
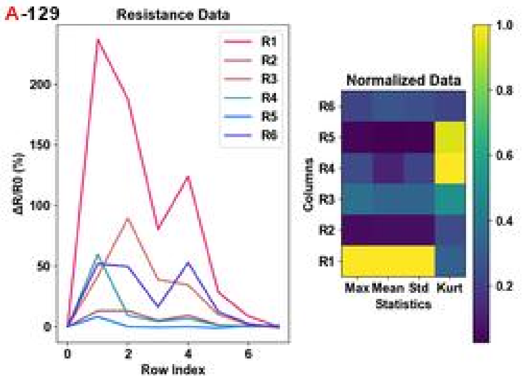

A-130
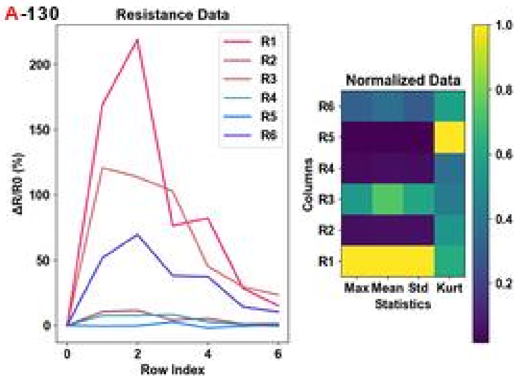

A-131
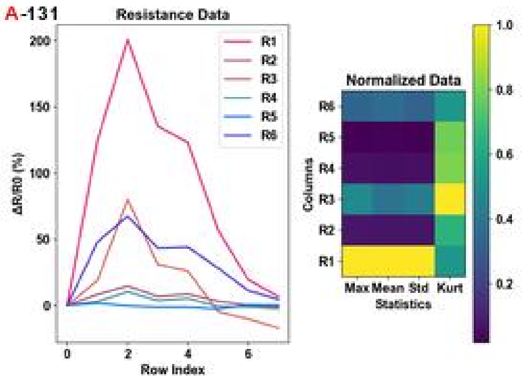

A-132
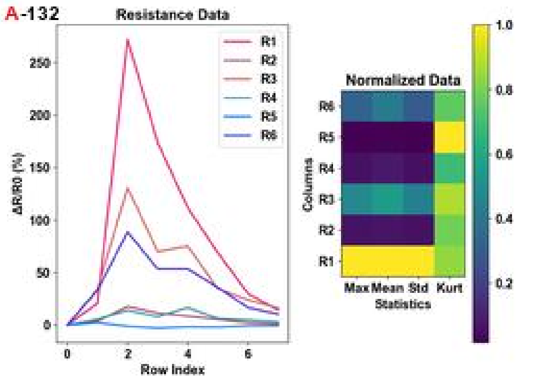

A-133
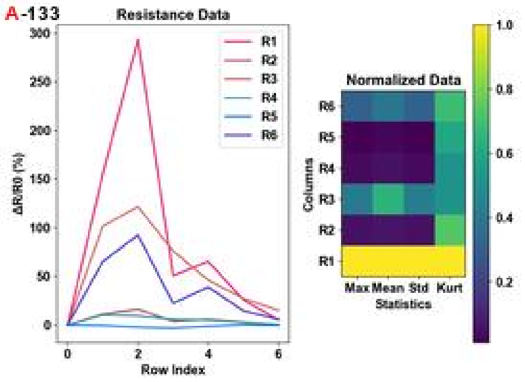

A-134
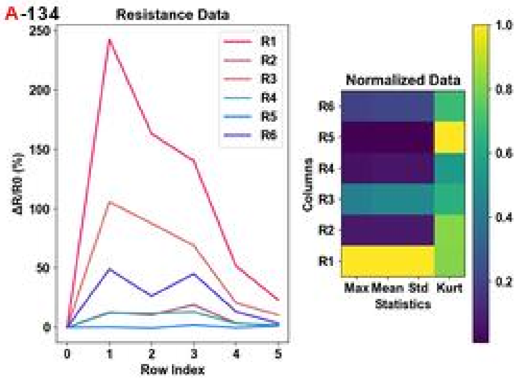

A-135
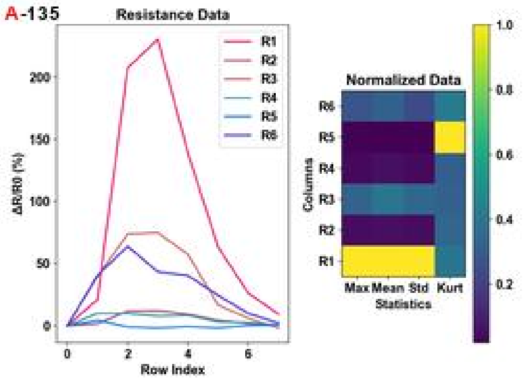

A-136
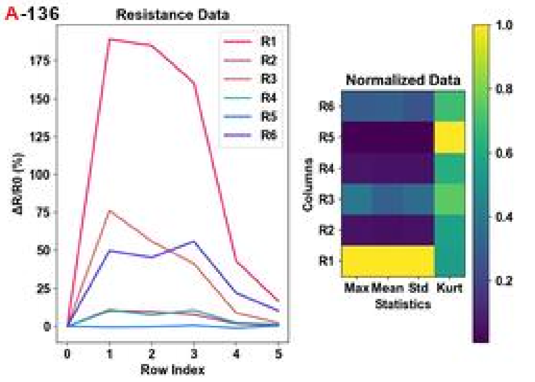

A-137
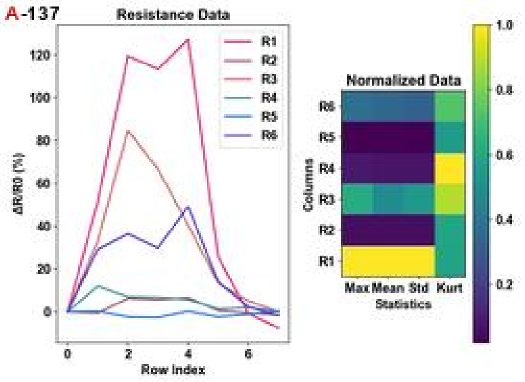

A-138
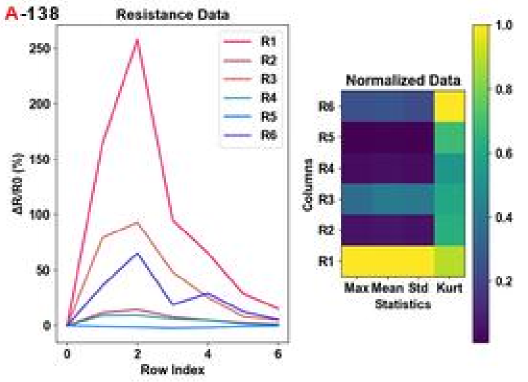

A-139
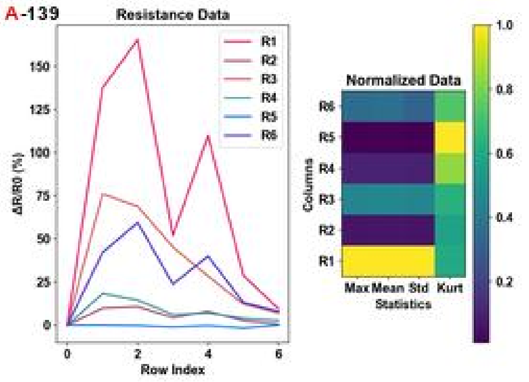

A-140
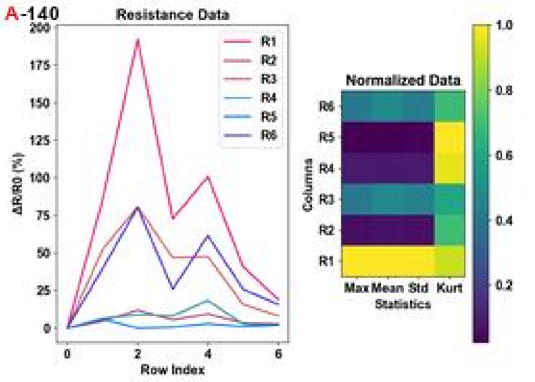

A-141
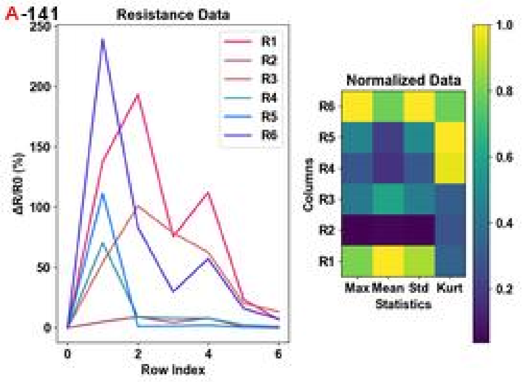

A-142
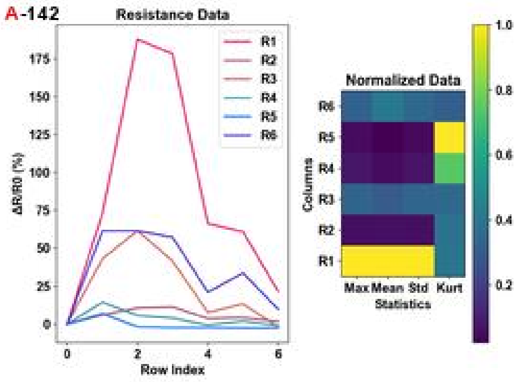

A-143
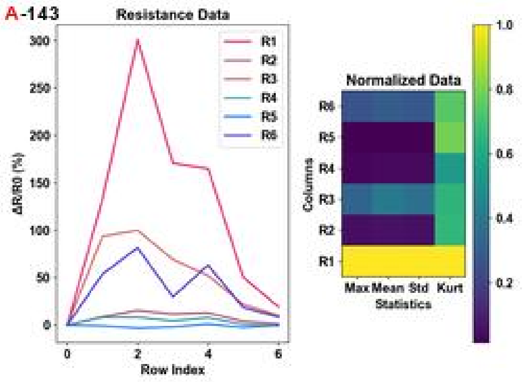

A-144
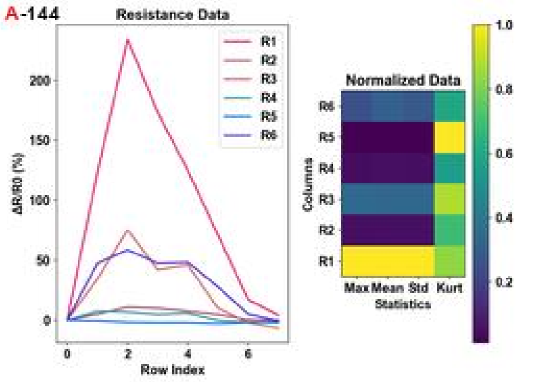

A-145
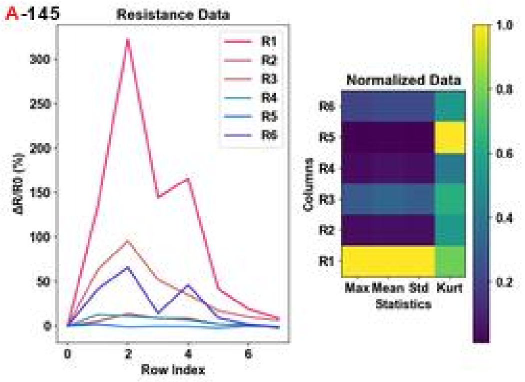

A-146
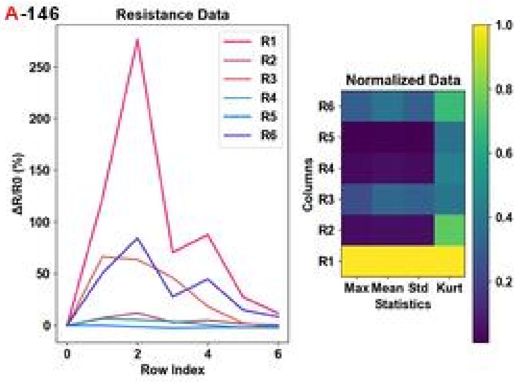

A-147
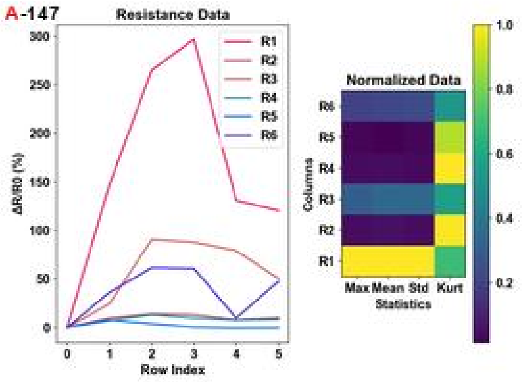

A-148
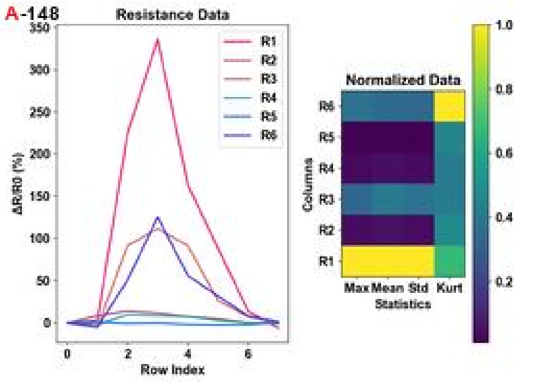

A-149
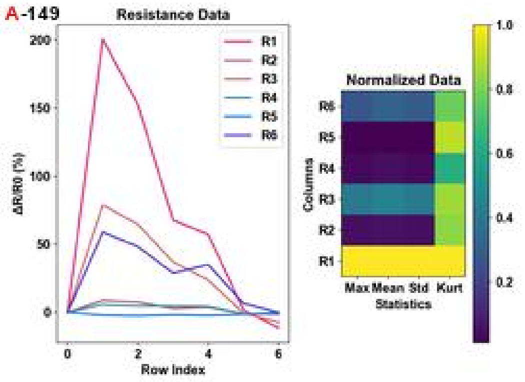

A-150
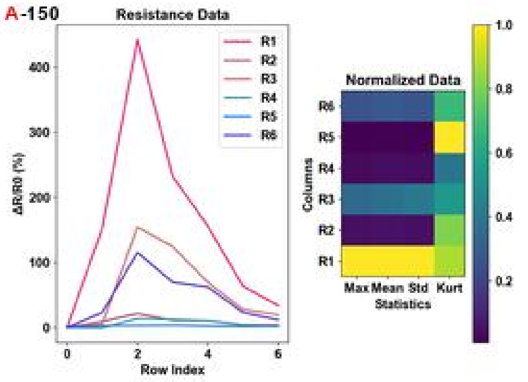

A-151
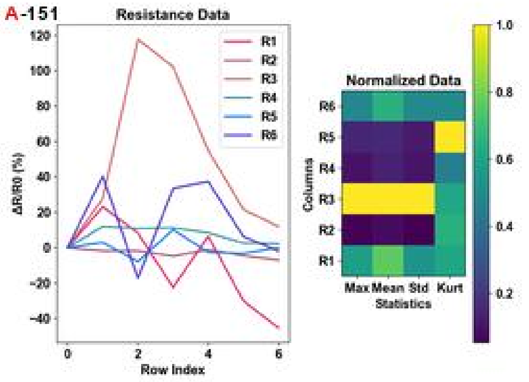

A-152
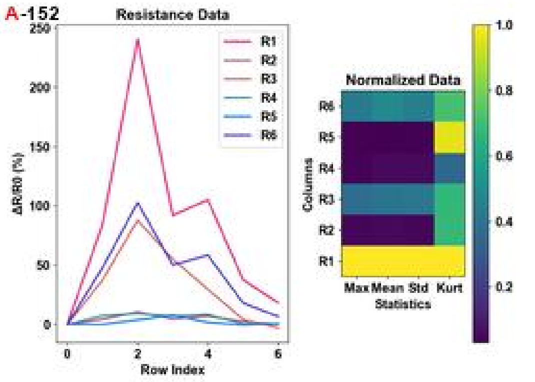

A-153
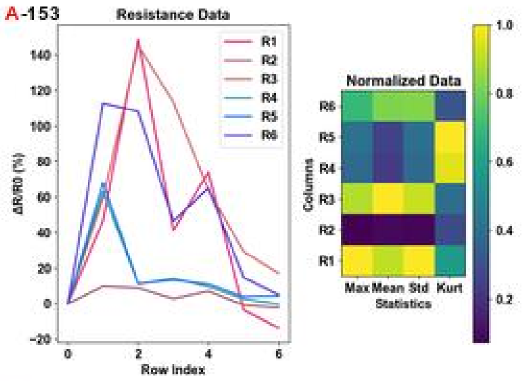

A-154
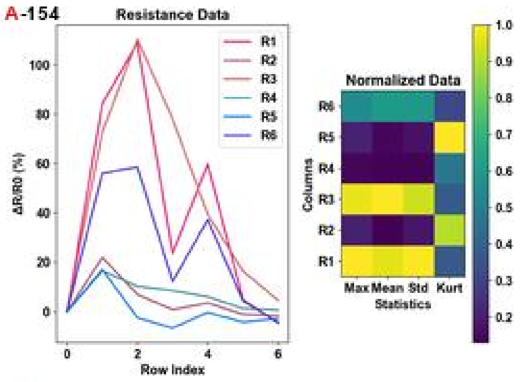

A-155
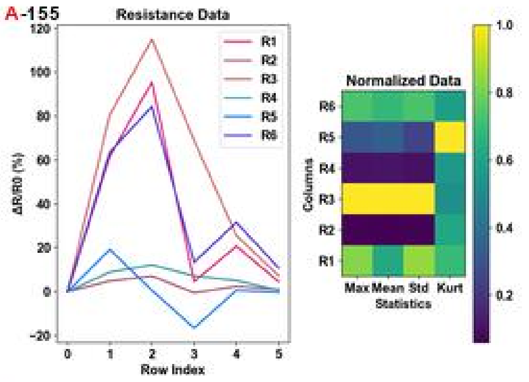

A-156
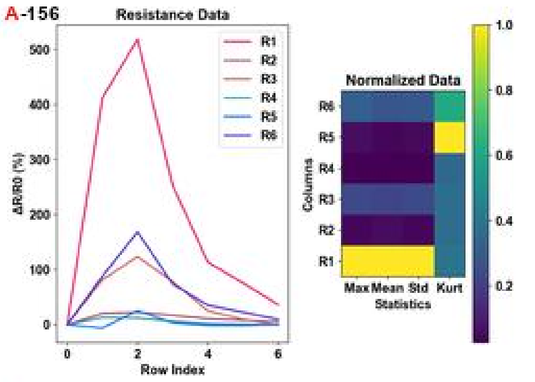

A-157
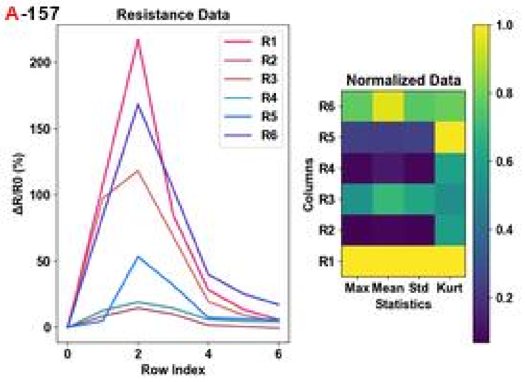

A-158
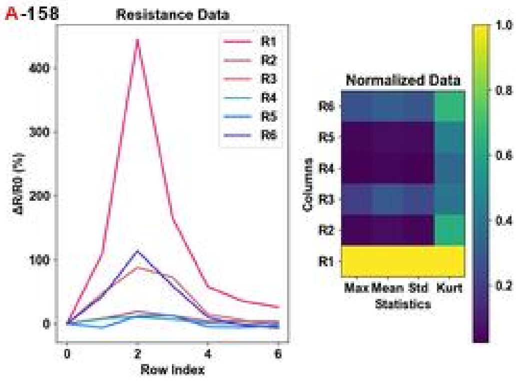

A-159
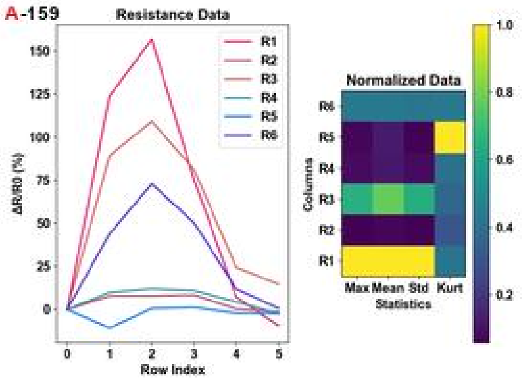

A-160
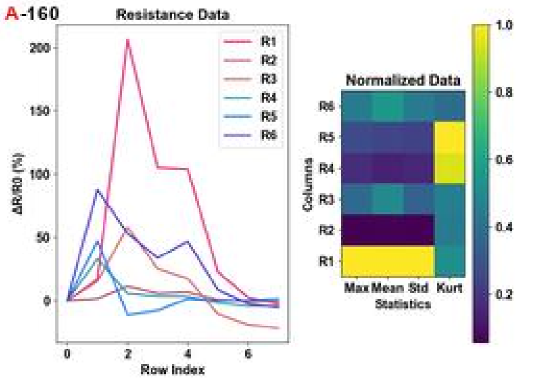

A-161
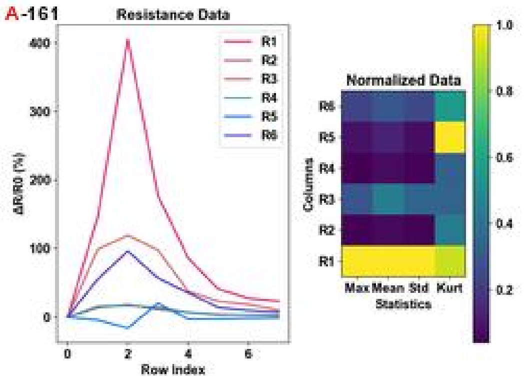

A-162
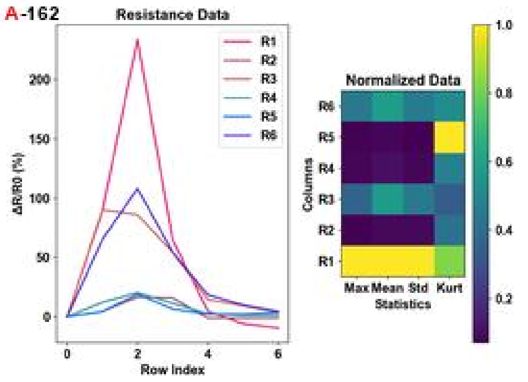

A-163
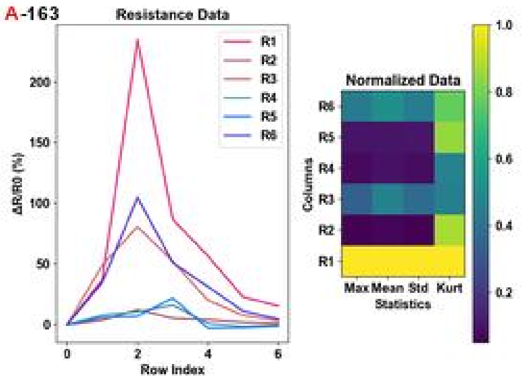

A-164
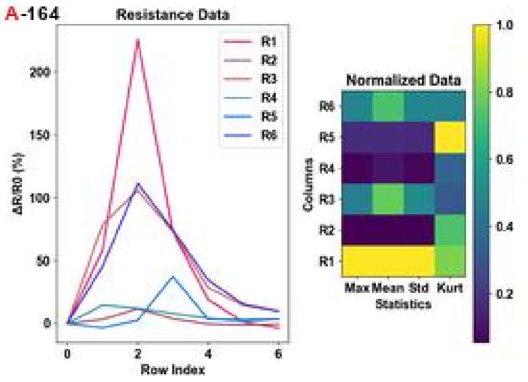

A-165
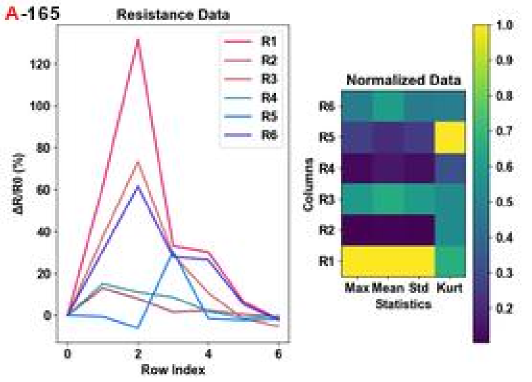

A-166
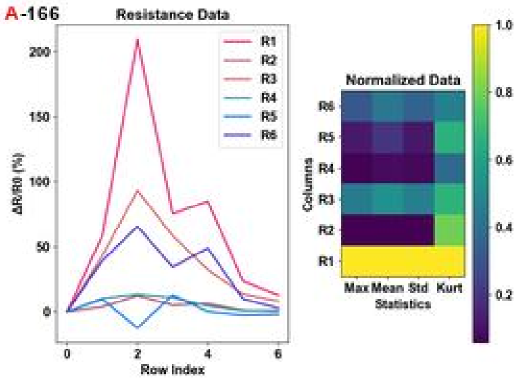

A-167
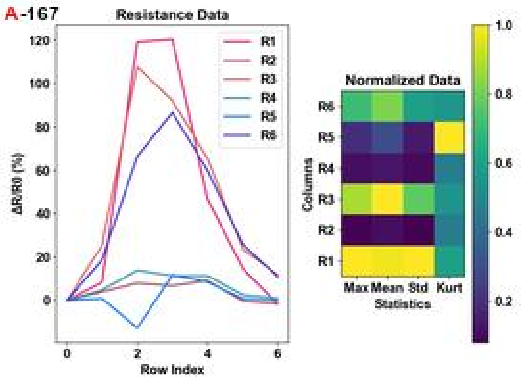

A-168
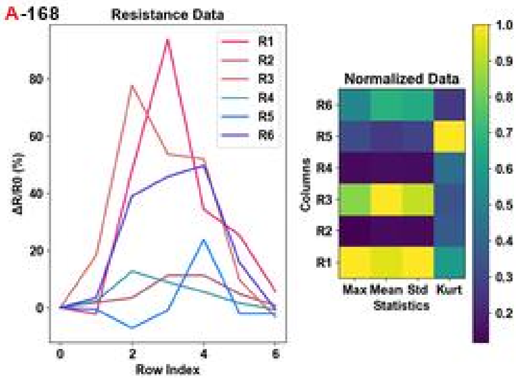

A-169
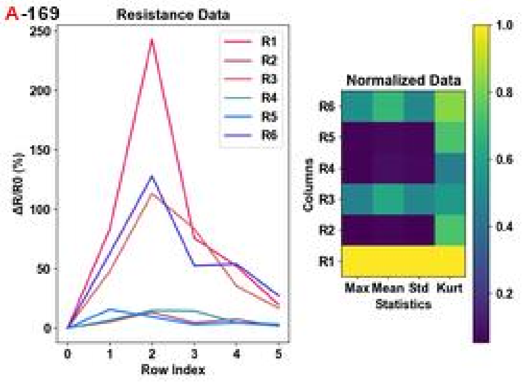

A-170
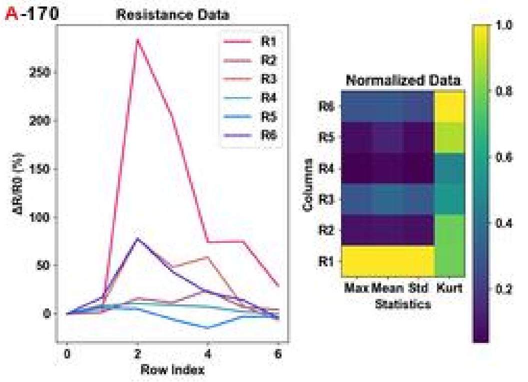

A-171
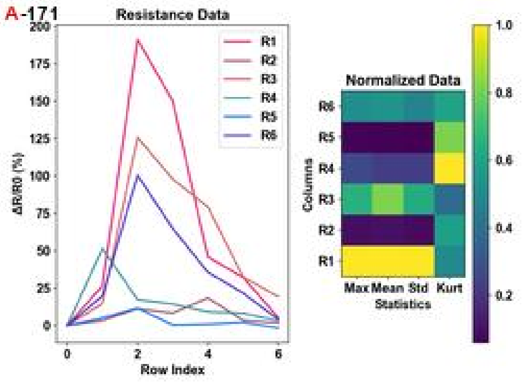

A-172
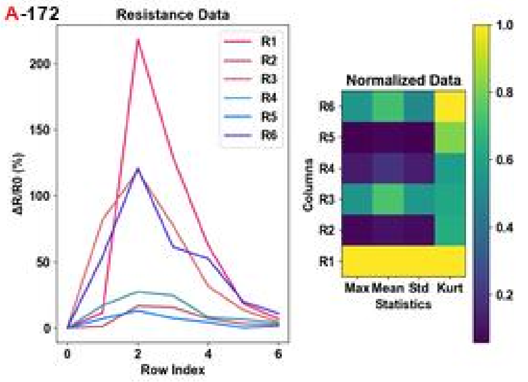

A-173
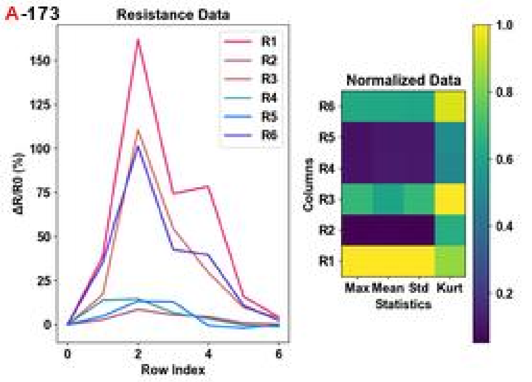

A-174
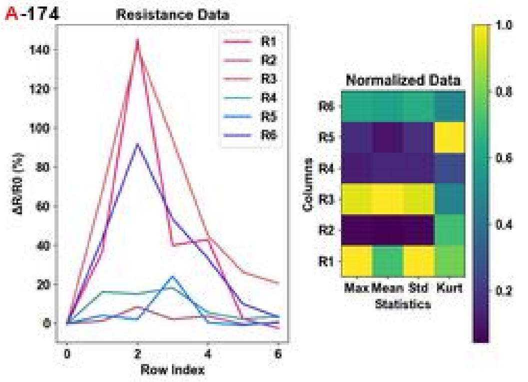

A-175
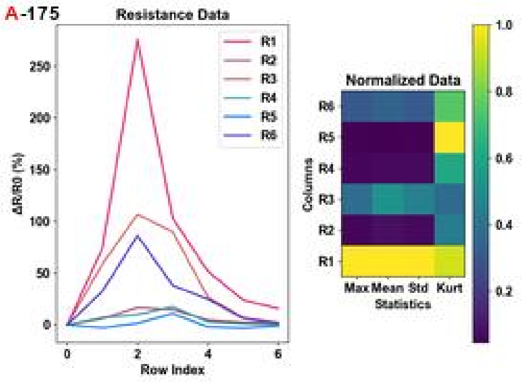

A-176
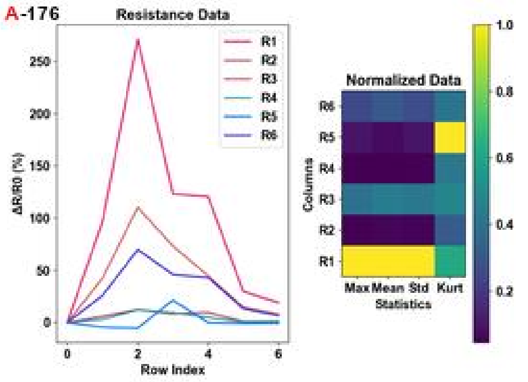

A-177
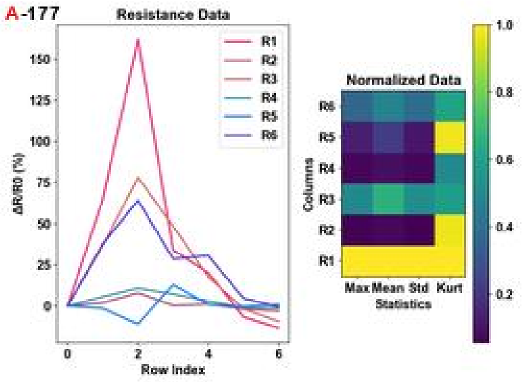

A-178
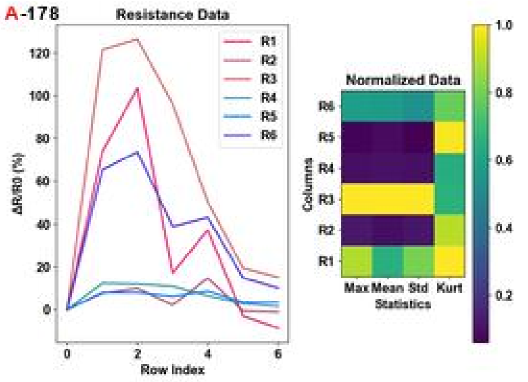

A-179
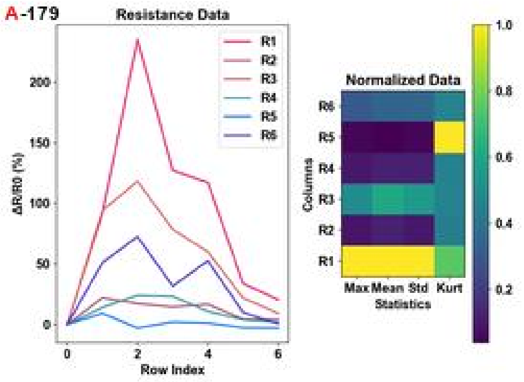

A-180
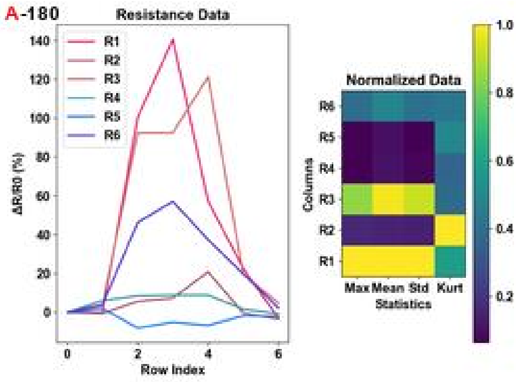

A-181
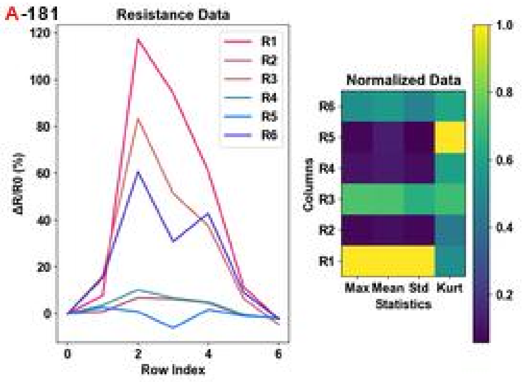

A-182
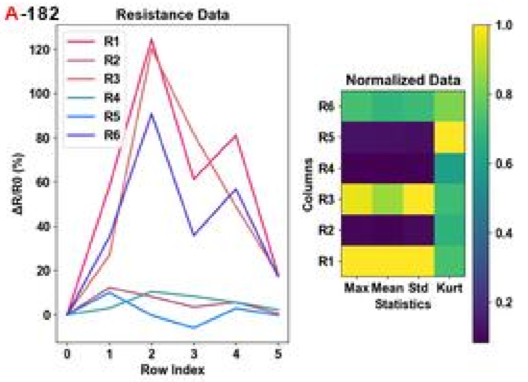

A-183
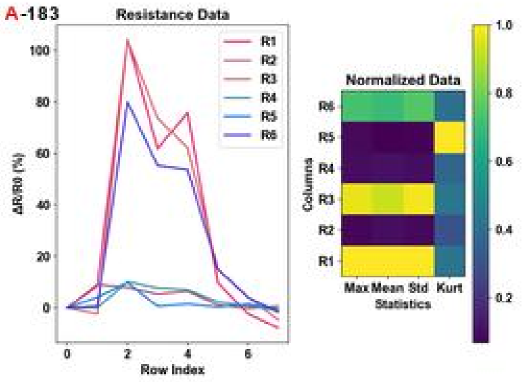

A-184
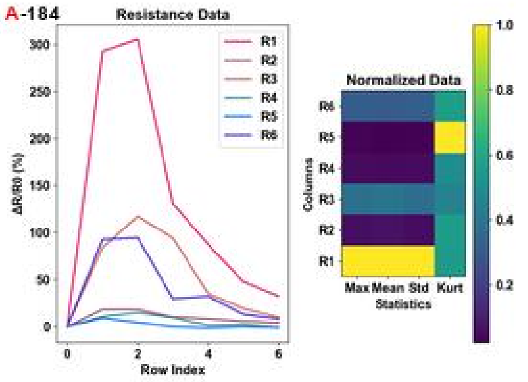

A-185
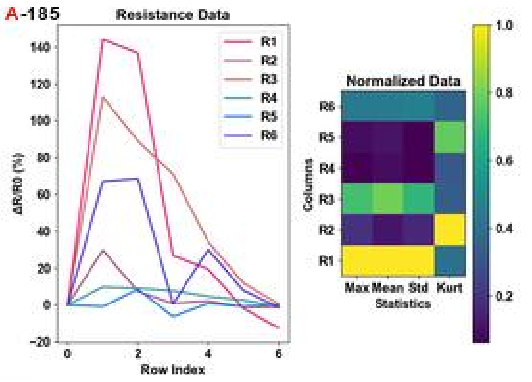

A-186
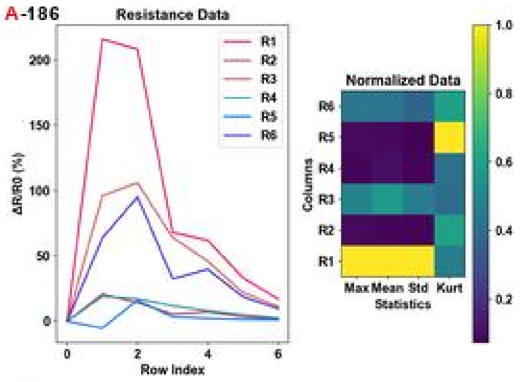

A-187
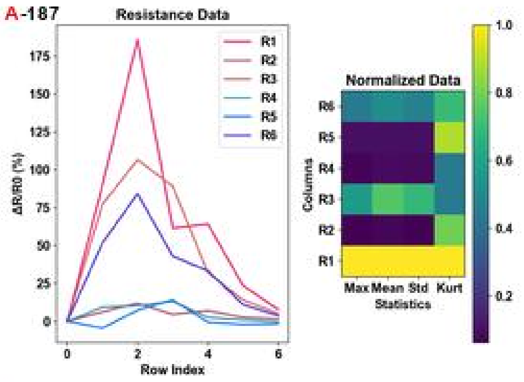

A-188
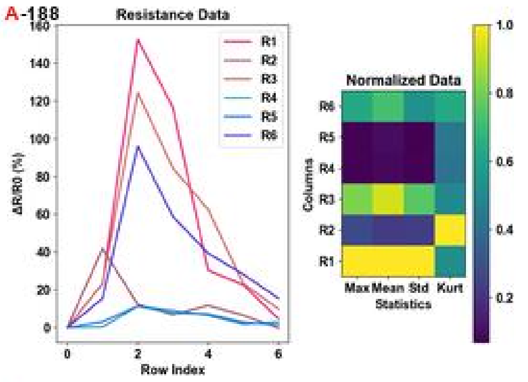

A-189
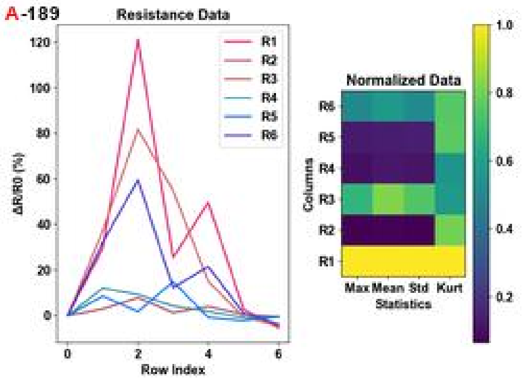

A-190
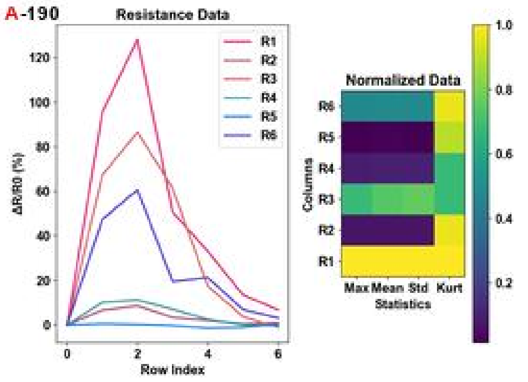

A-191
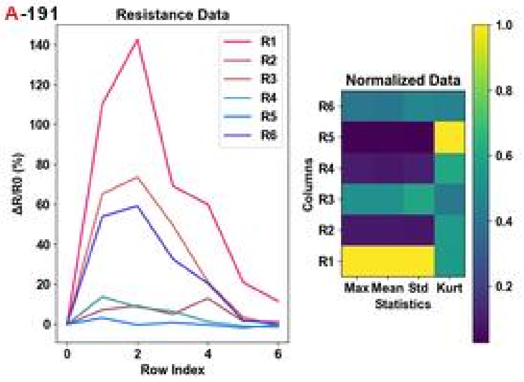

A-192
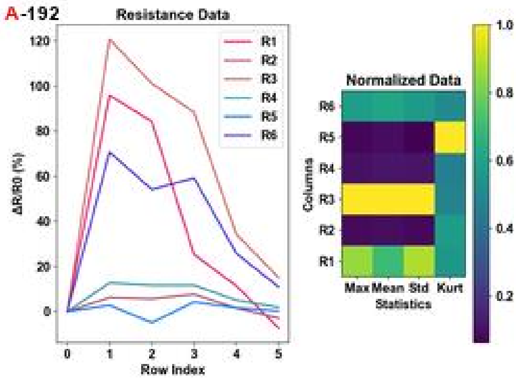

Pressure-sensitive data of type A

A-193
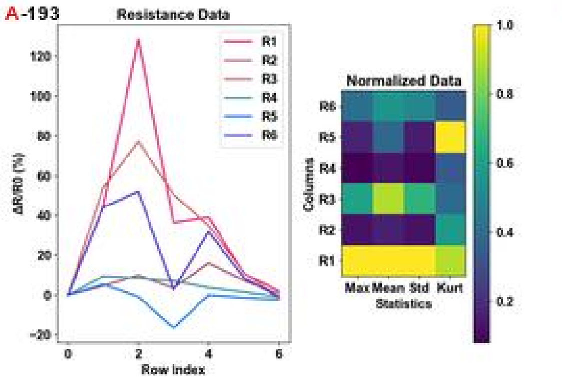

A-194
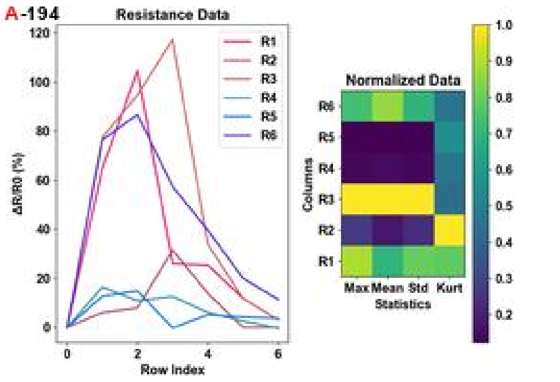

A-195
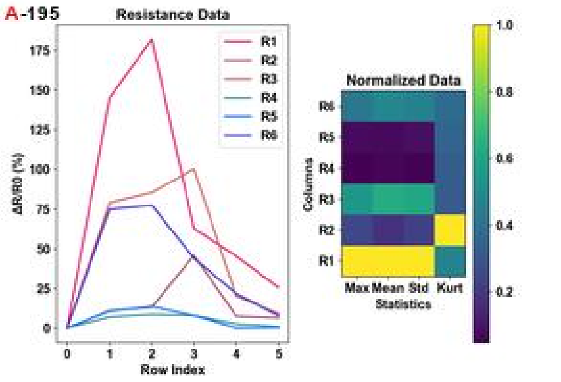

A-196
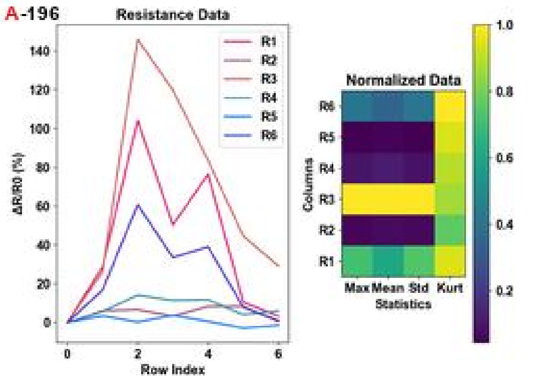

A-197
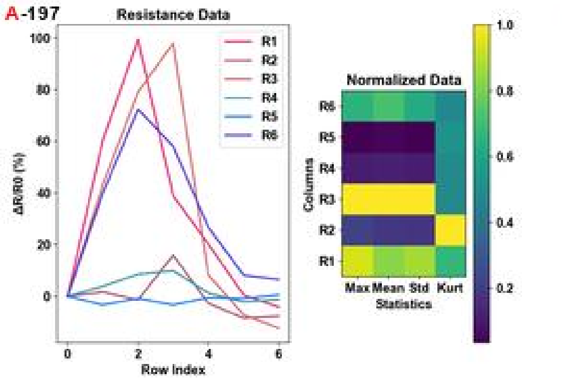

A-198
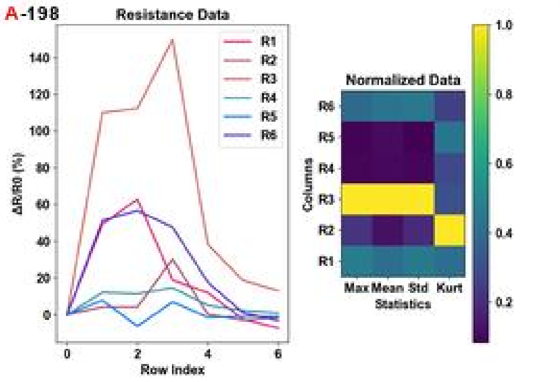

A-199
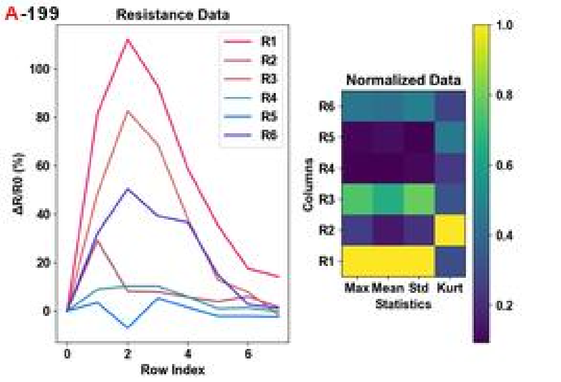

A-200
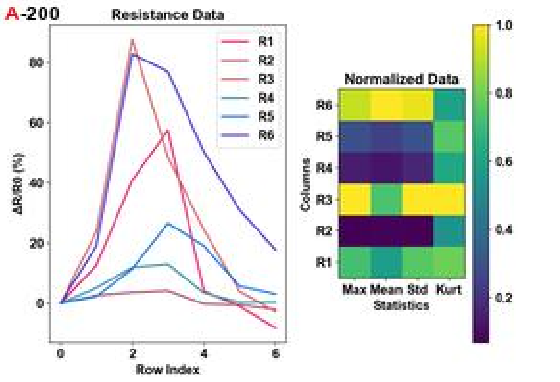

A-201
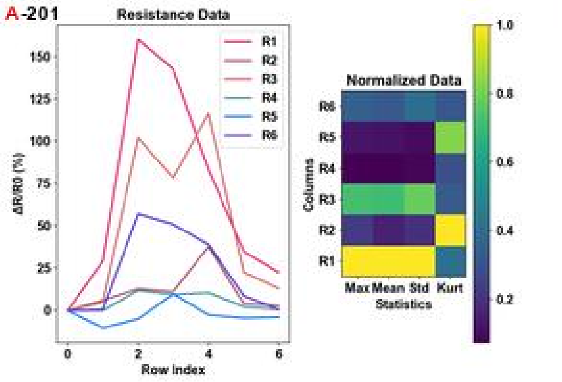

A-202
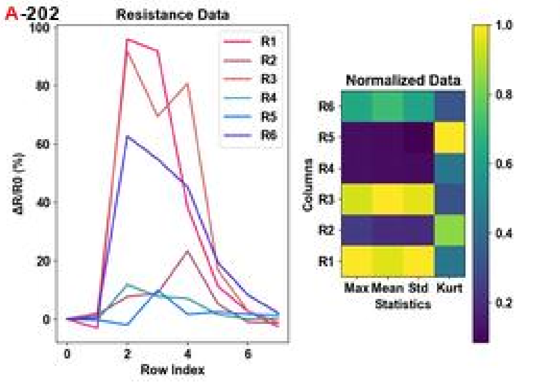

A-203
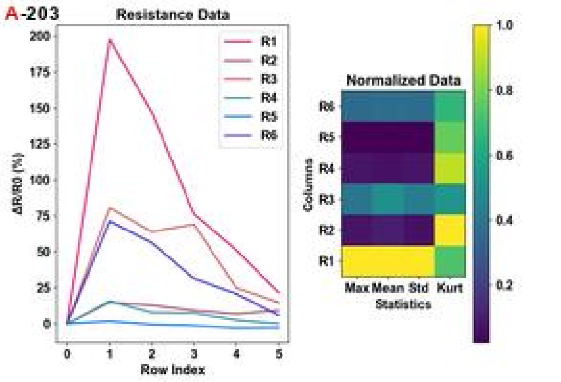

A-204
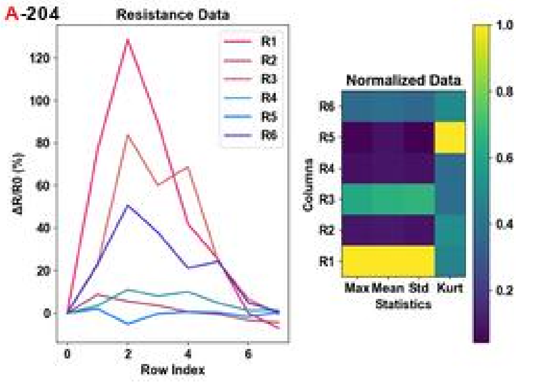

A-205
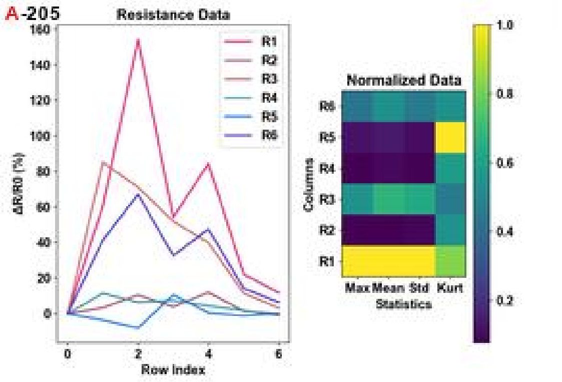

A-206
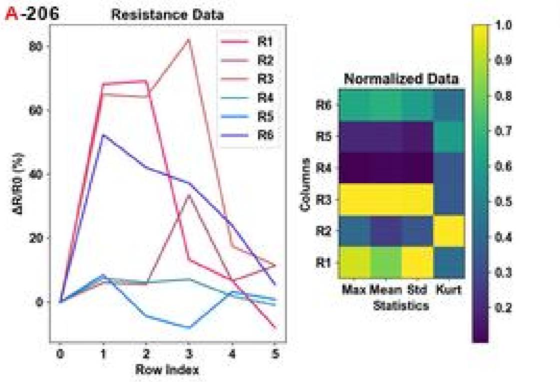

A-207
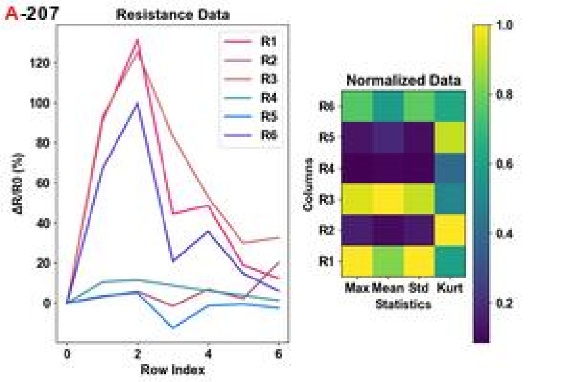

A-208
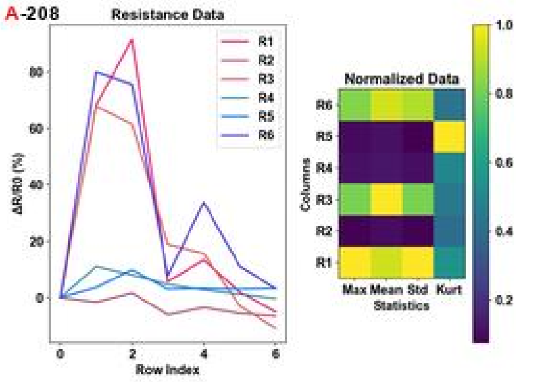

A-209
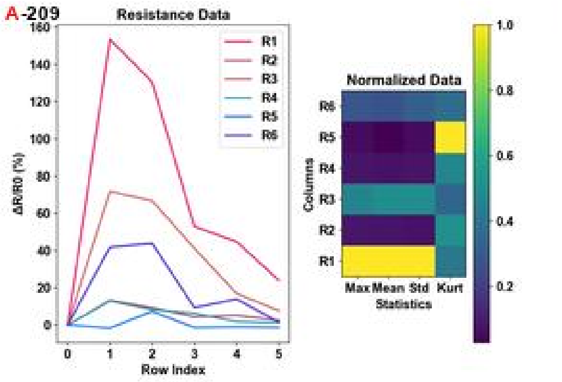

A-210
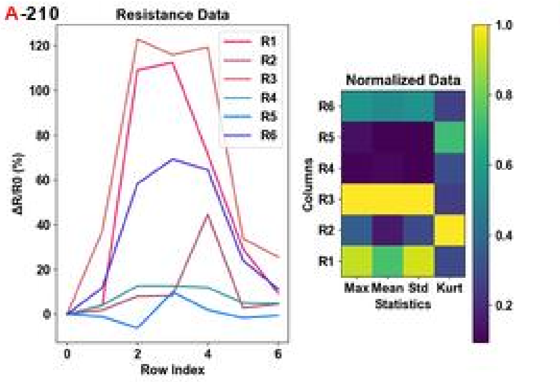

A-211
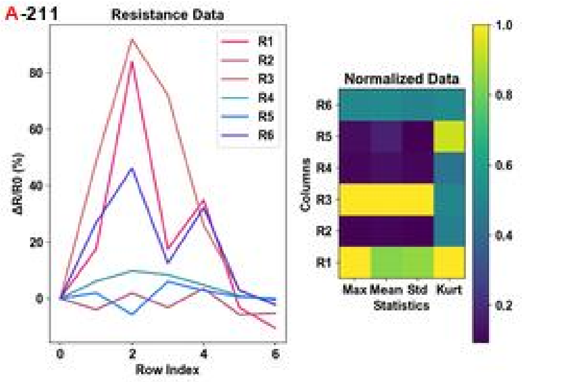

A-212
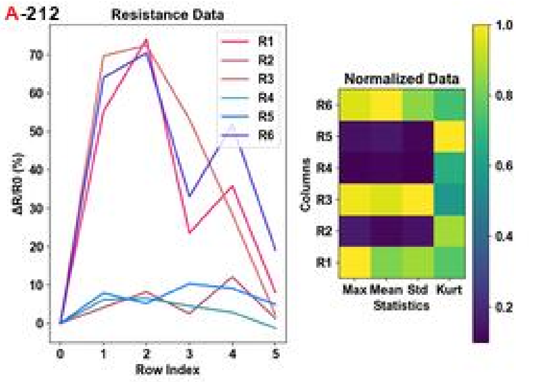

A-213
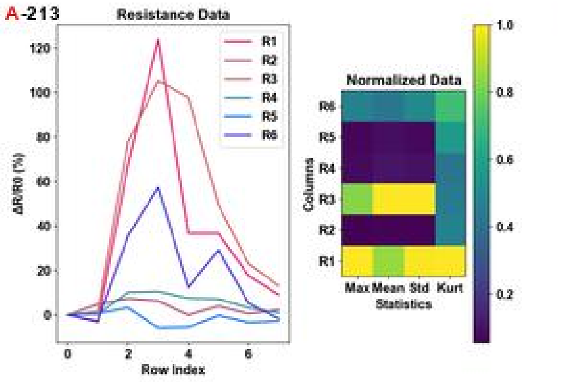

A-214
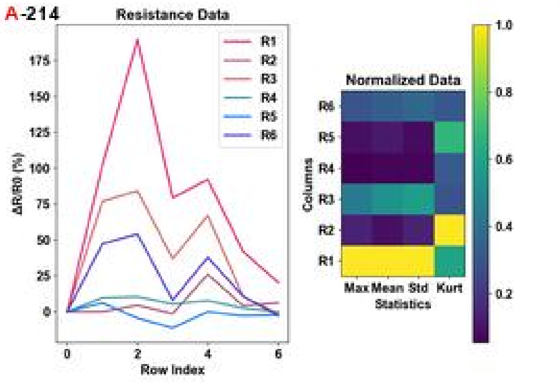

A-215
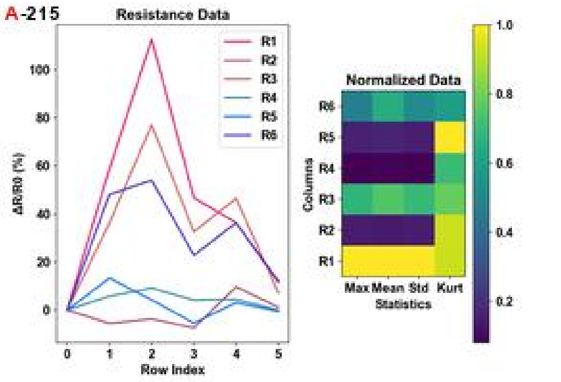

A-216
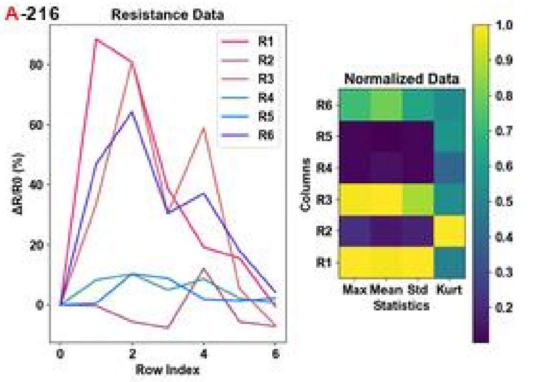

A-217
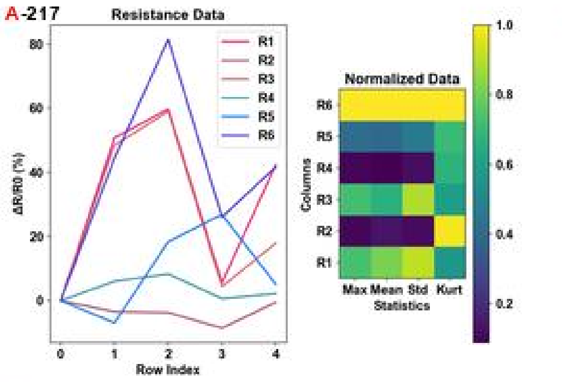

A-218
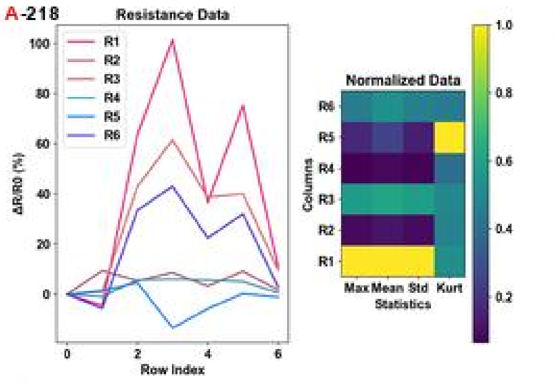

A-219
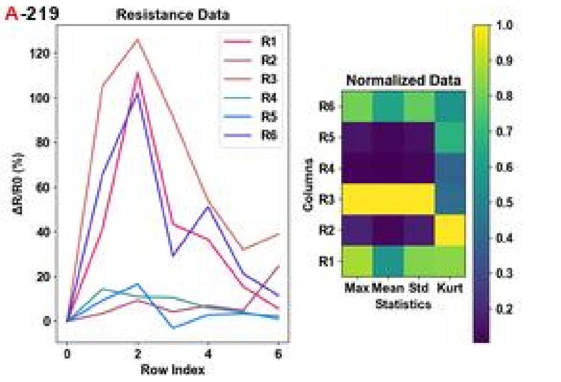

A-220
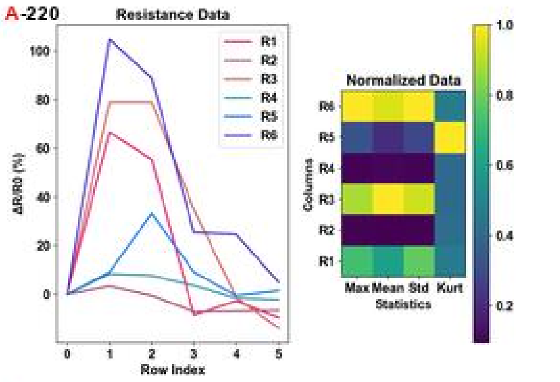

A-221
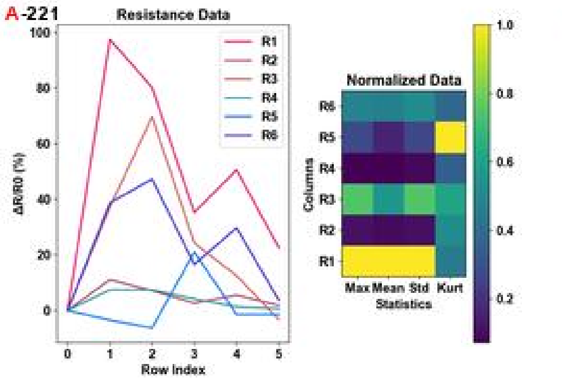

A-222
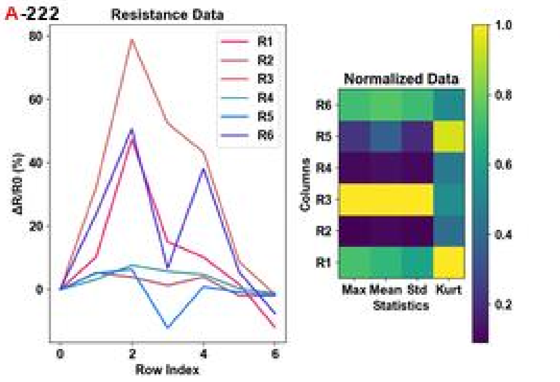

A-223
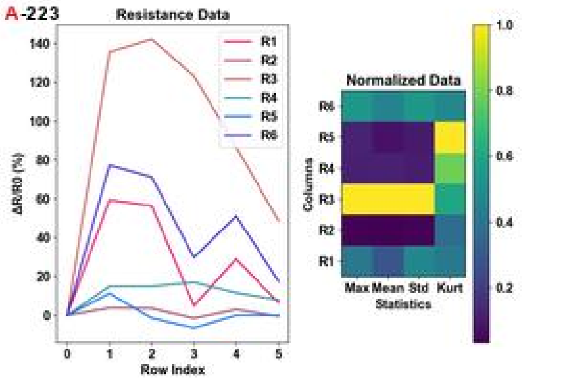

A-224
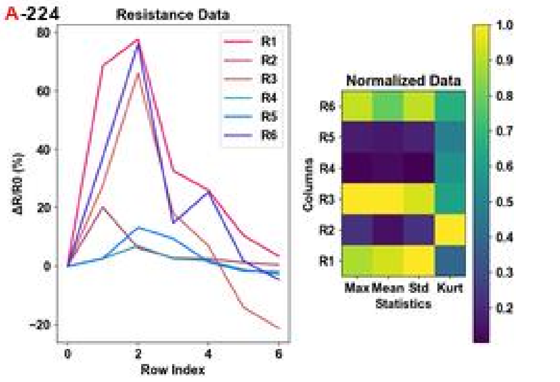

A-225

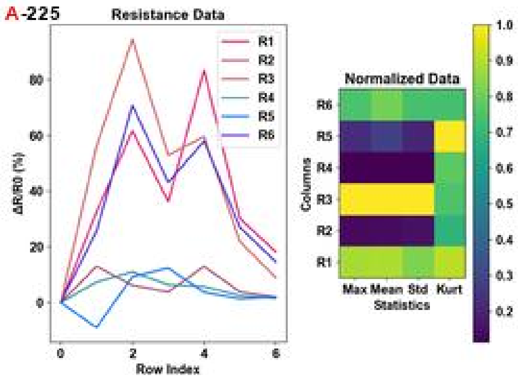

A-226

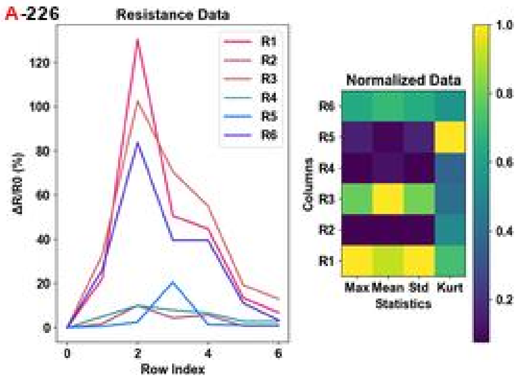

A-227

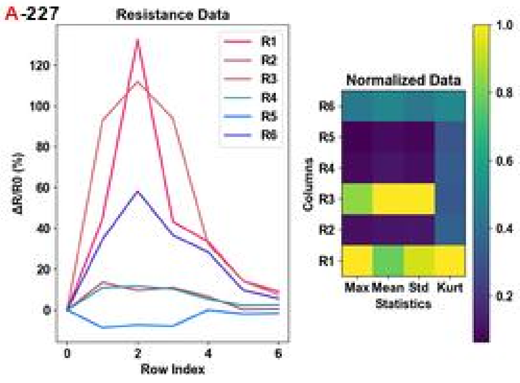

A-228

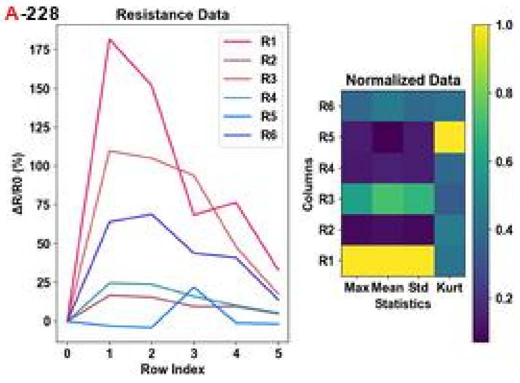

A-229

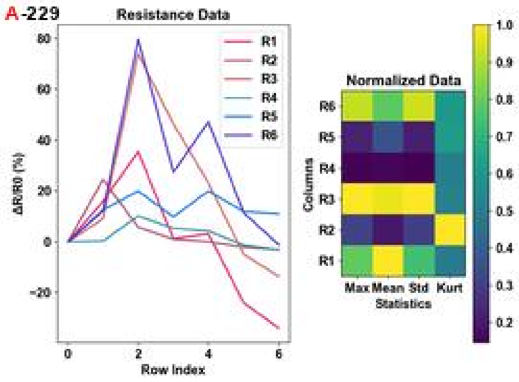

A-230

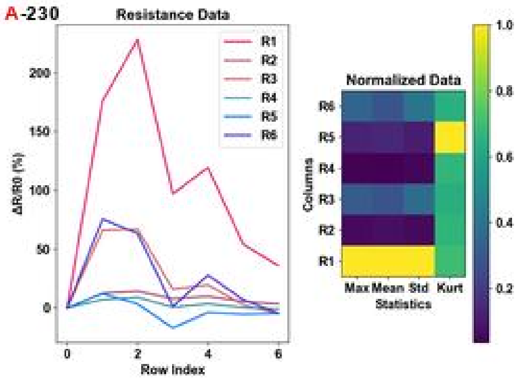

A-231

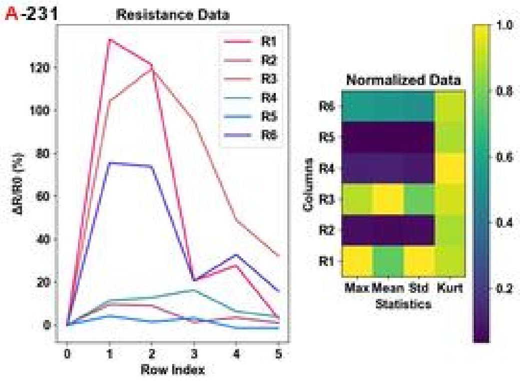

A-232

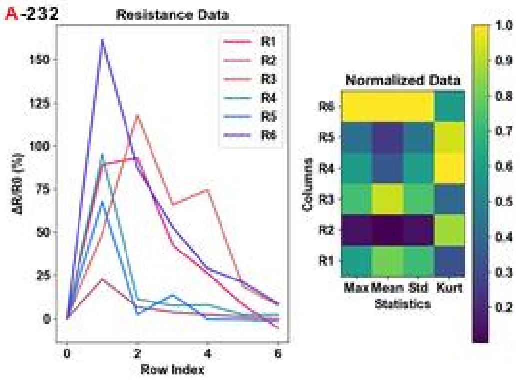

A-233

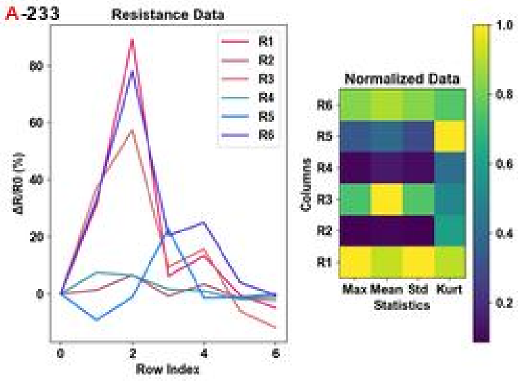

A-234

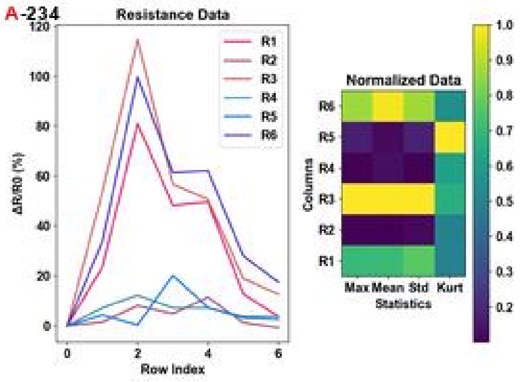

A-235

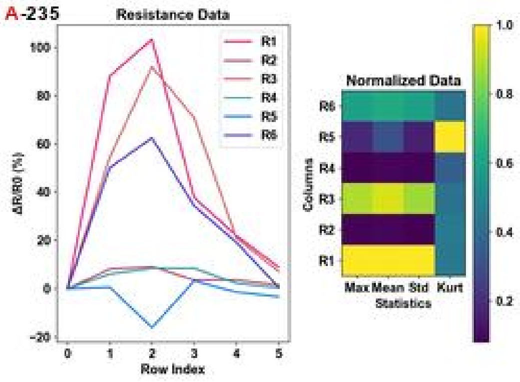

A-236

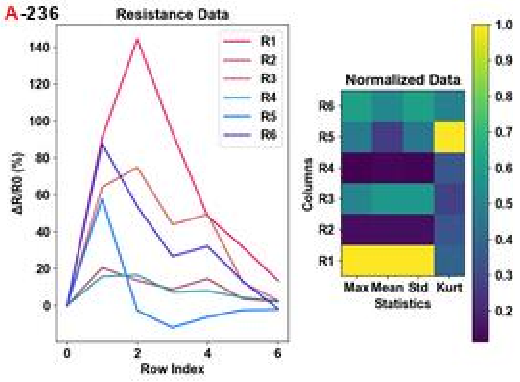

A-237

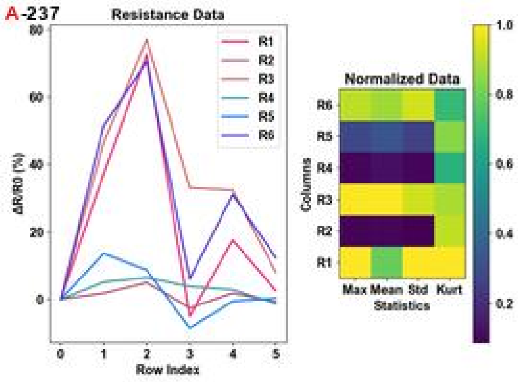

A-238

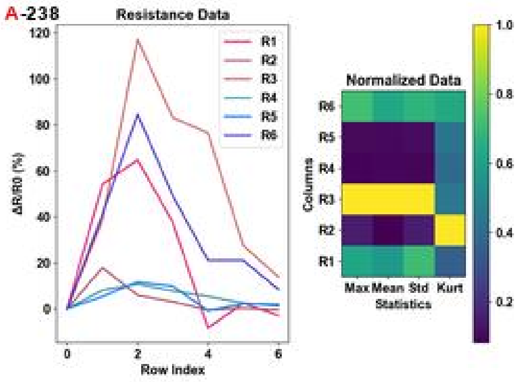

A-239

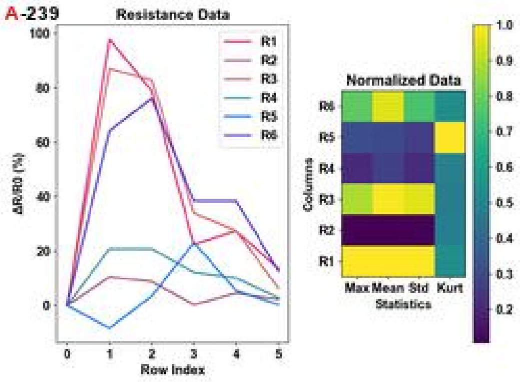

A-240

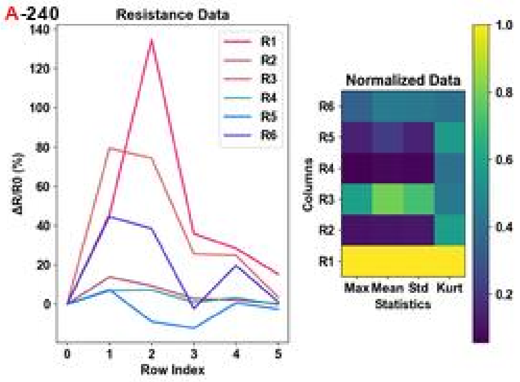

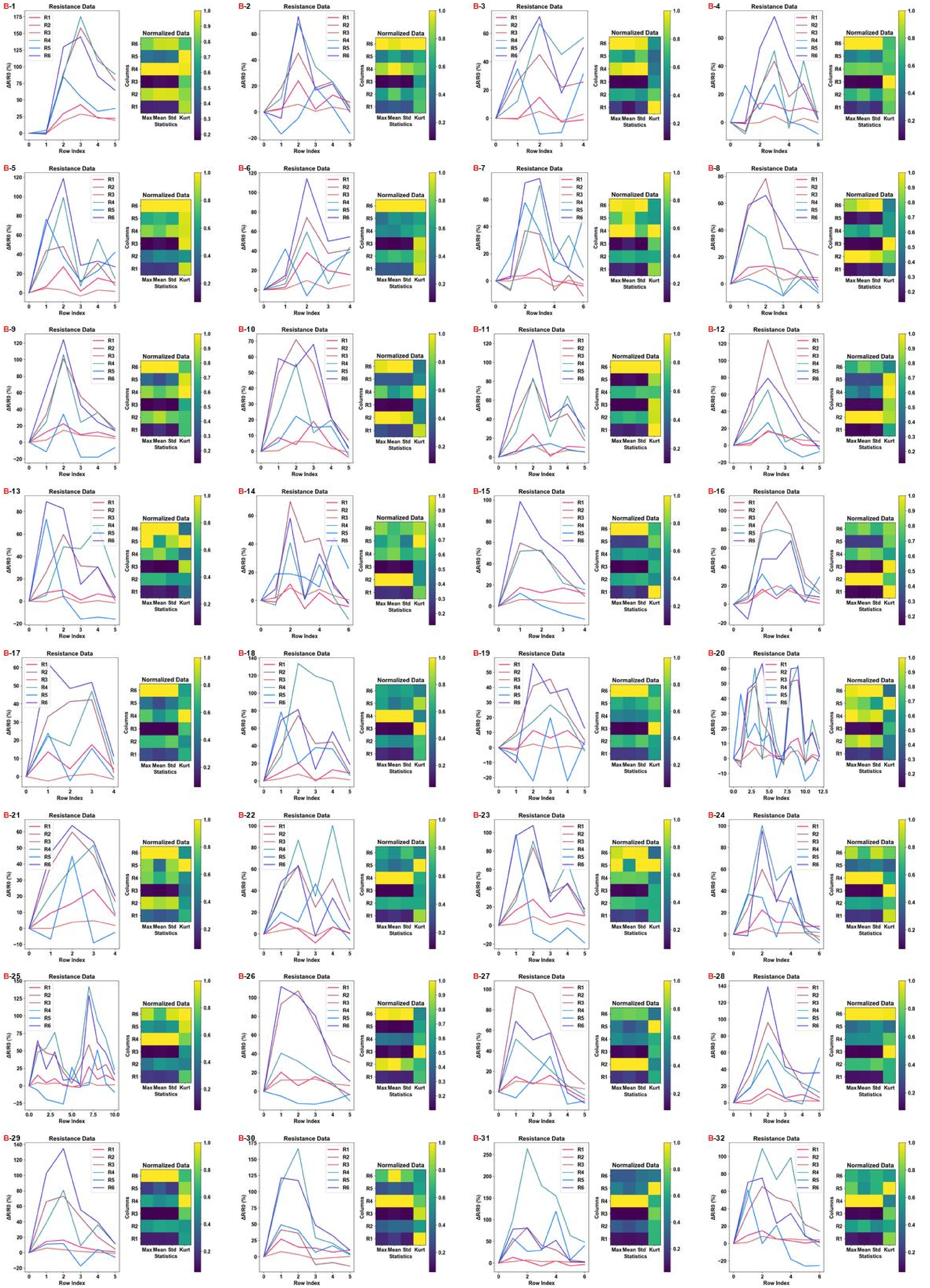
B-1
B-2
B-3
B-4
B-5
B-6
B-7
B-8
B-9
B-10
B-11
B-12
B-13
B-14
B-15
B-16
B-17
B-18
B-19
B-20
B-21
B-22
B-23
B-24
B-25
B-26
B-27
B-28
B-29
B-30
B-31
B-32
Resistance Data
ΔR/R0 (%)
Row Index
R1
R2
R3
R4
R5
R6
Normalized Data
Columns
Statistics
Max
Mean
Std
Kurt

B-33
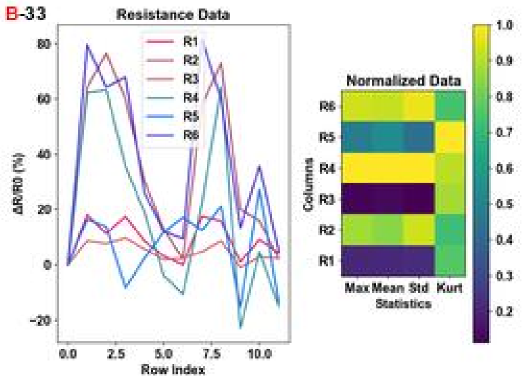

B-34
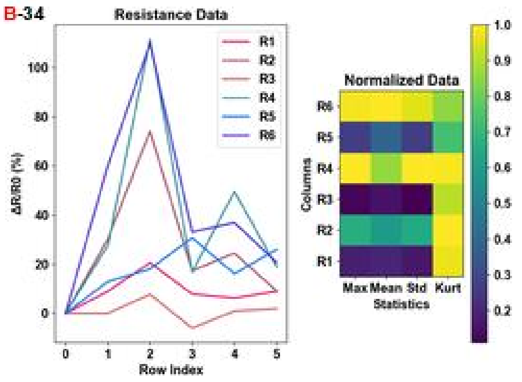

B-35
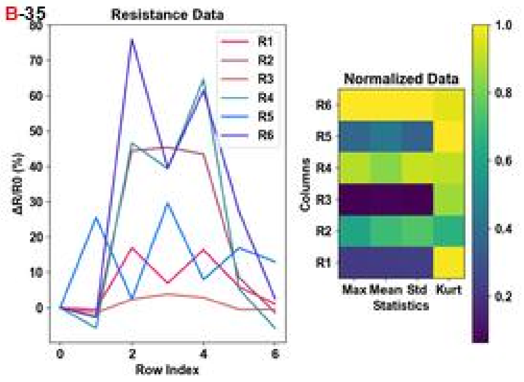

B-36
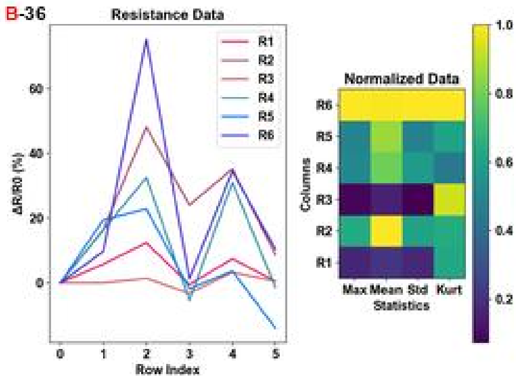

B-37
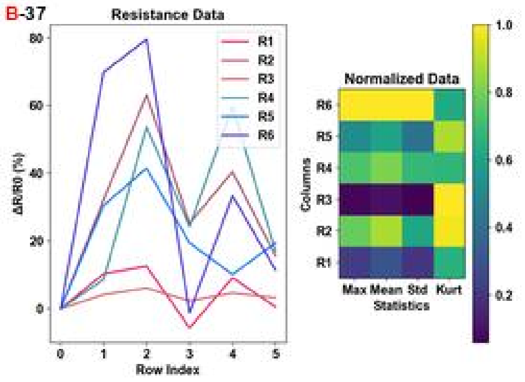

B-38
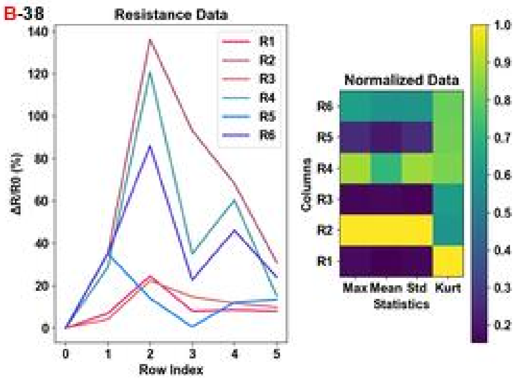

B-39
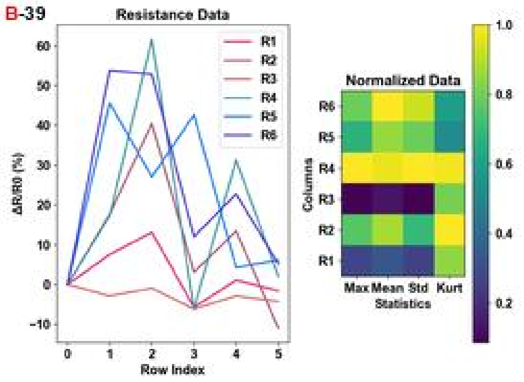

B-40
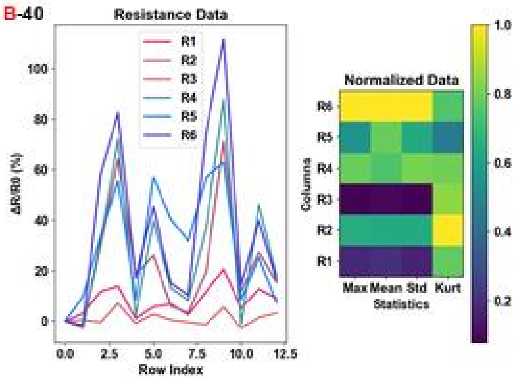

B-41
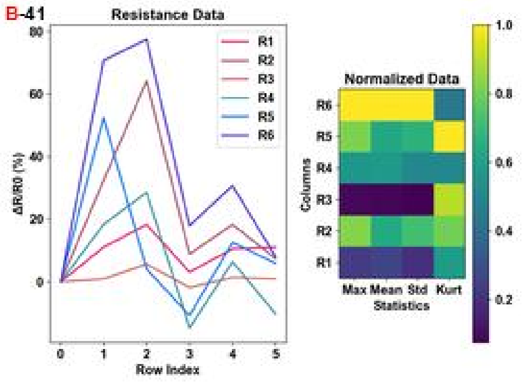

B-42
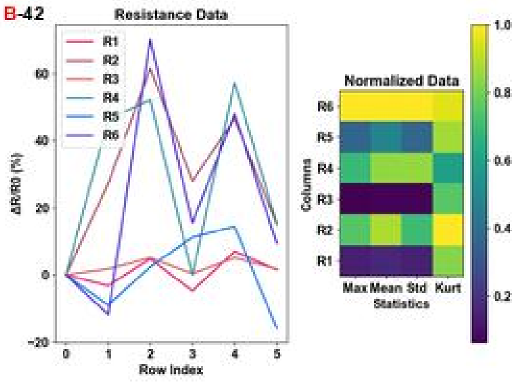

B-43
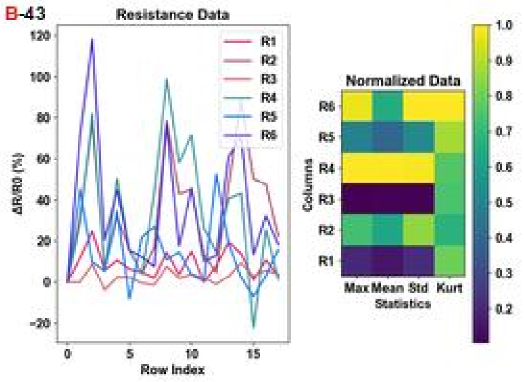

B-44
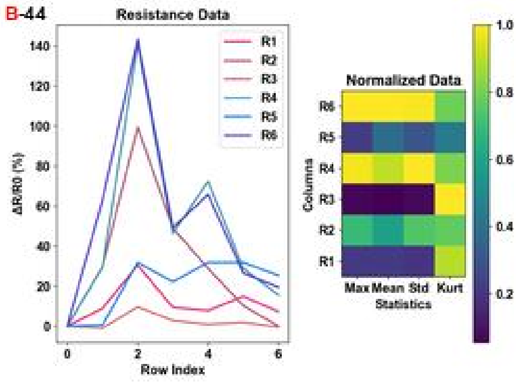

B-45
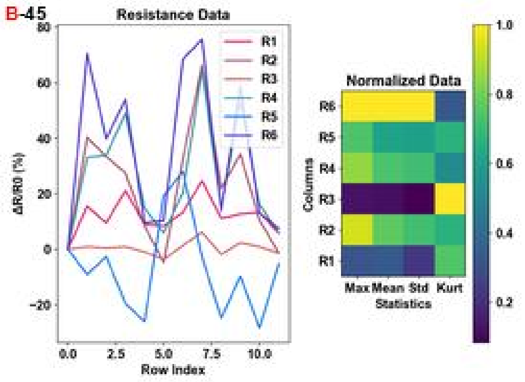

B-46
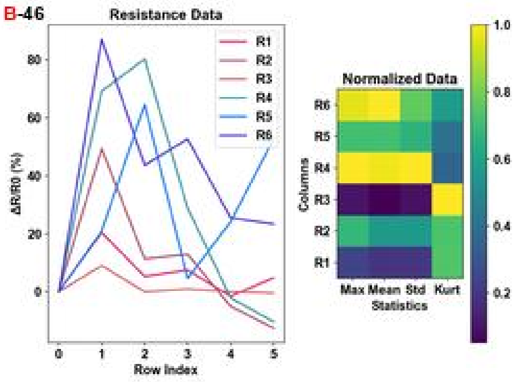

B-47
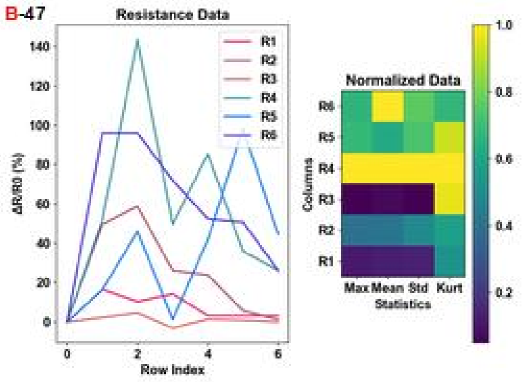

B-48
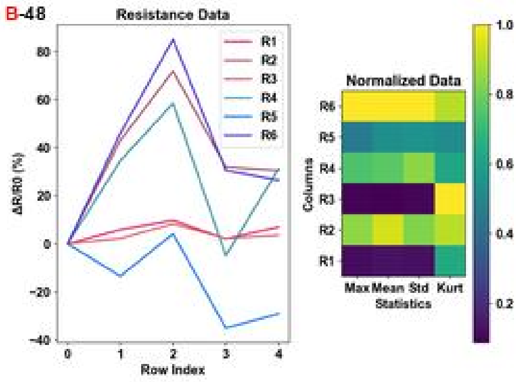

B-49
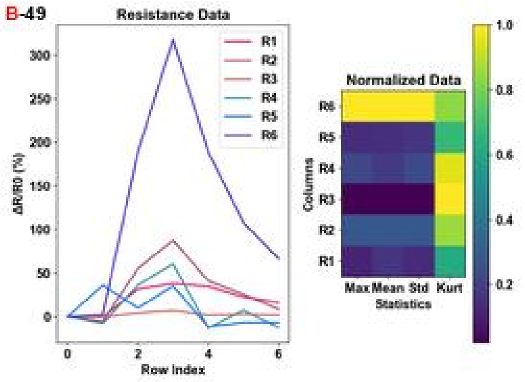

B-50
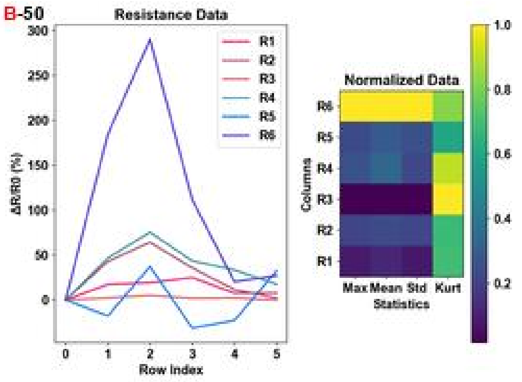

B-51
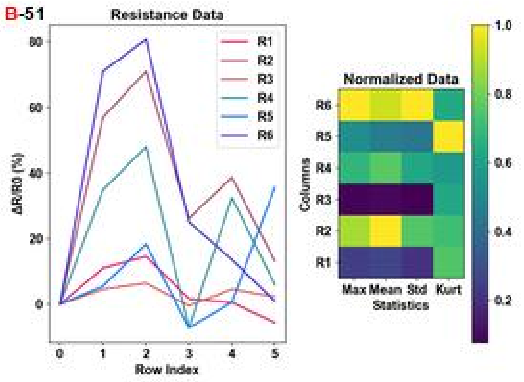

B-52
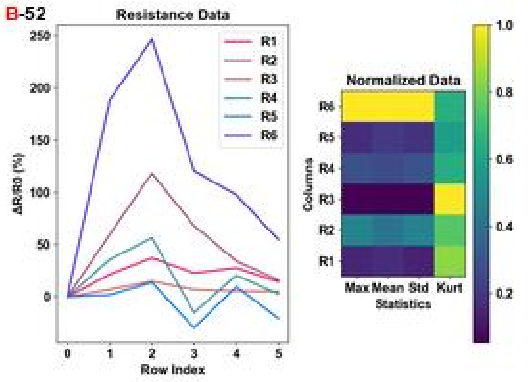

B-53
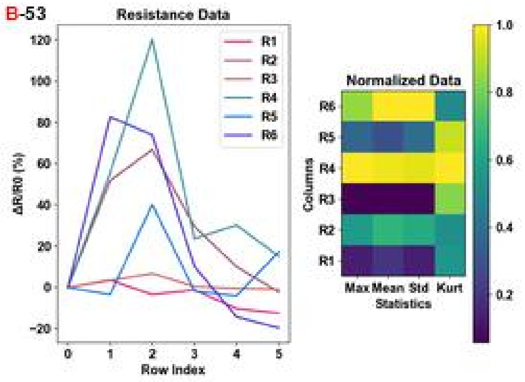

B-54
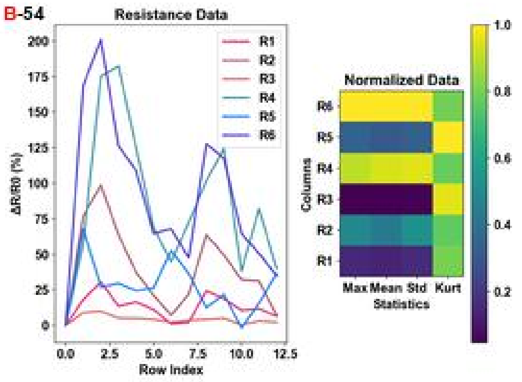

B-55
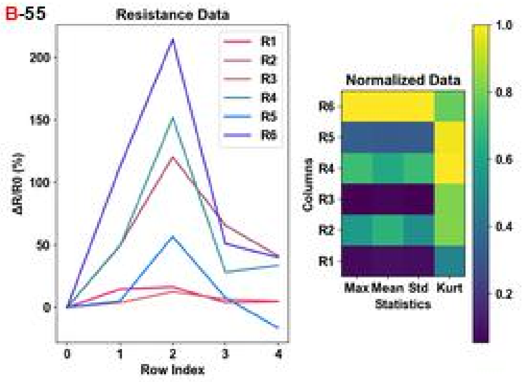

B-56
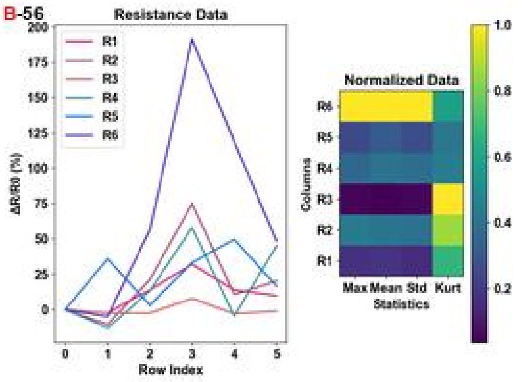

B-57
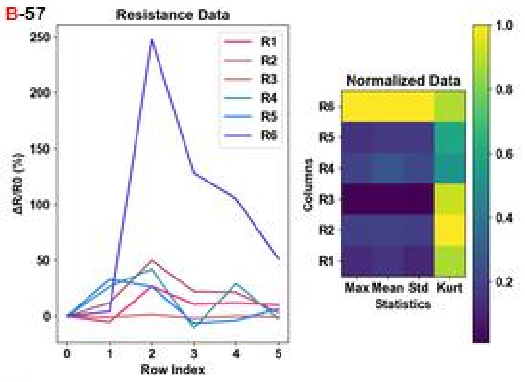

B-58
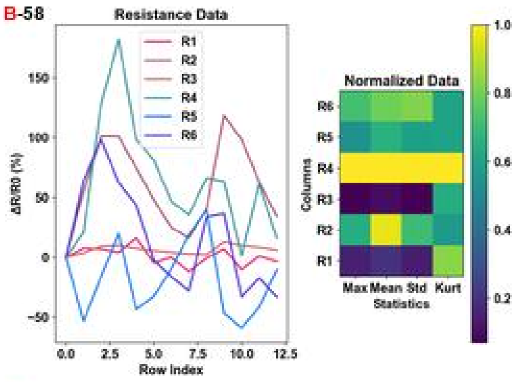

B-59
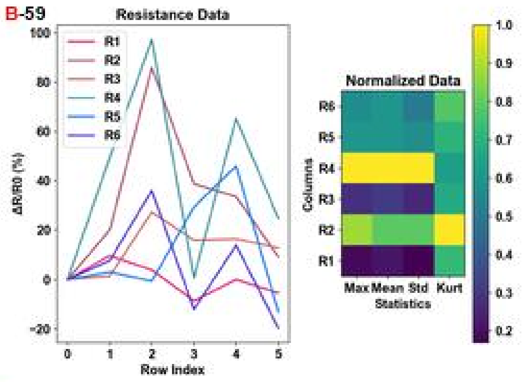

B-60
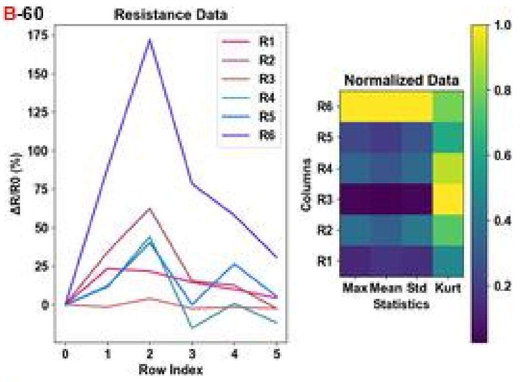

B-61
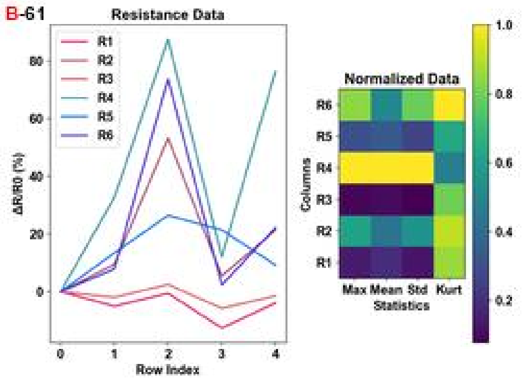

B-62
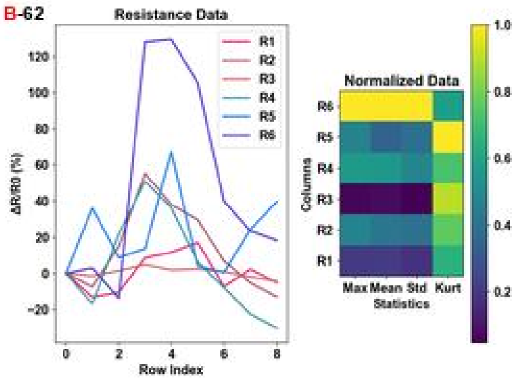

B-63
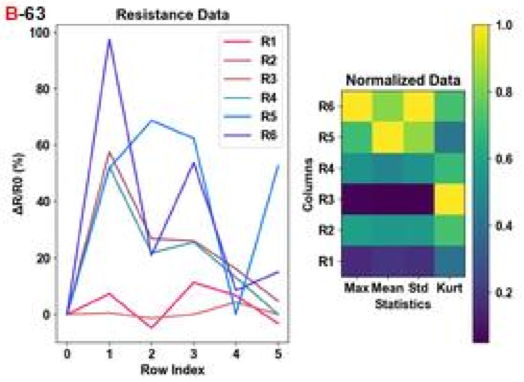

B-64
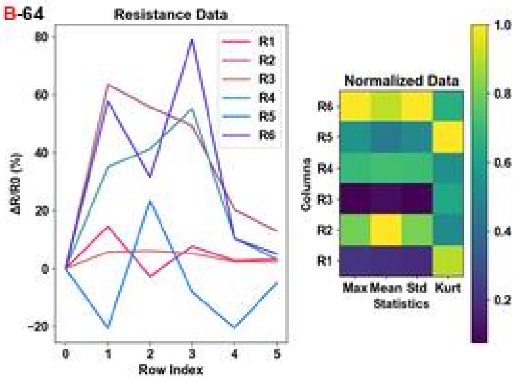

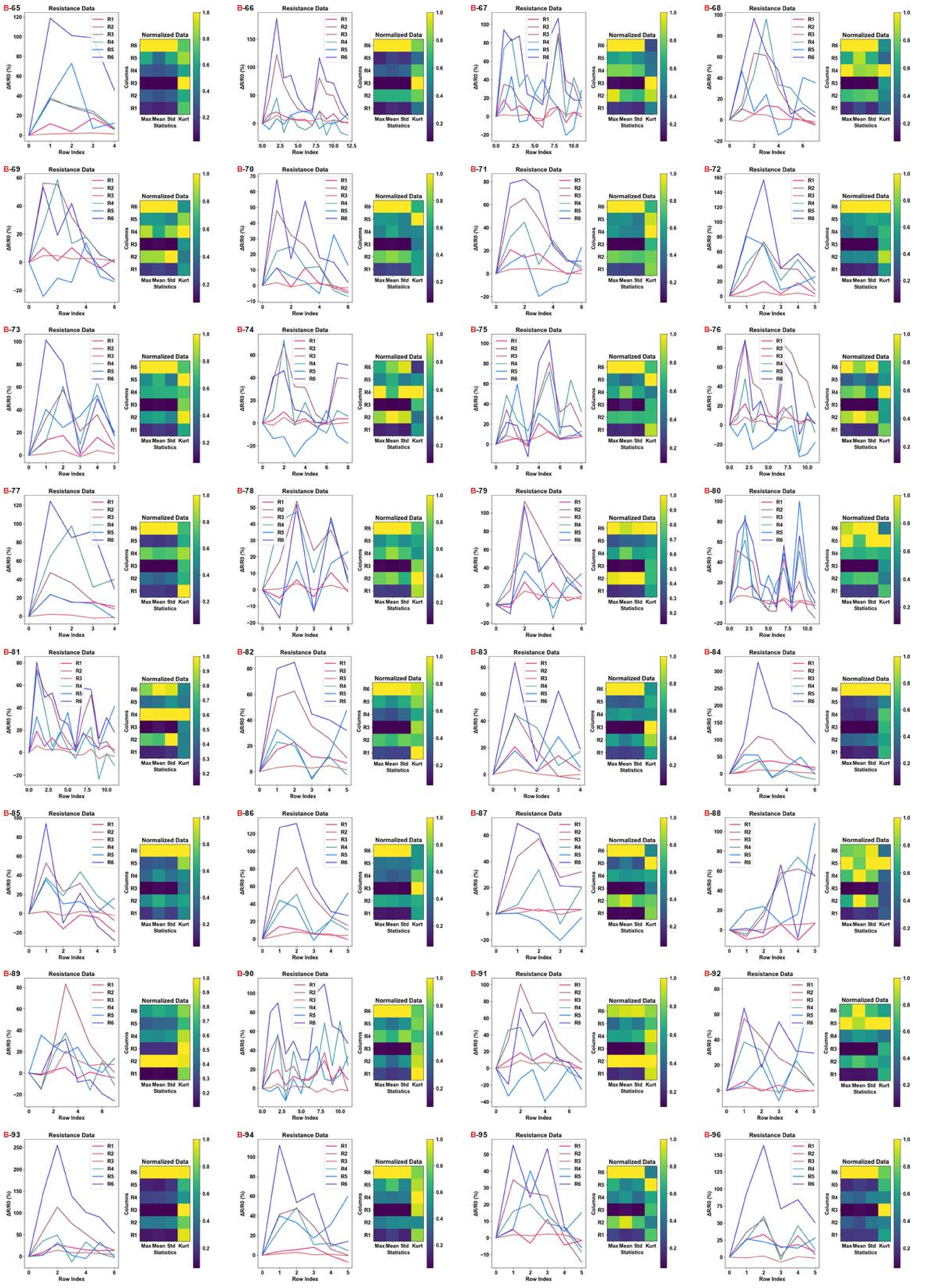
B-65
B-66
B-67
B-68
B-69
B-70
B-71
B-72
B-73
B-74
B-75
B-76
B-77
B-78
B-79
B-80
B-81
B-82
B-83
B-84
B-85
B-86
B-87
B-88
B-89
B-90
B-91
B-92
B-93
B-94
B-95
B-96
Resistance Data
Normalized Data
ΔR/R0 (%)
Row Index
Columns
Statistics
Max Mean Std Kurt
R1 R2 R3 R4 R5 R6

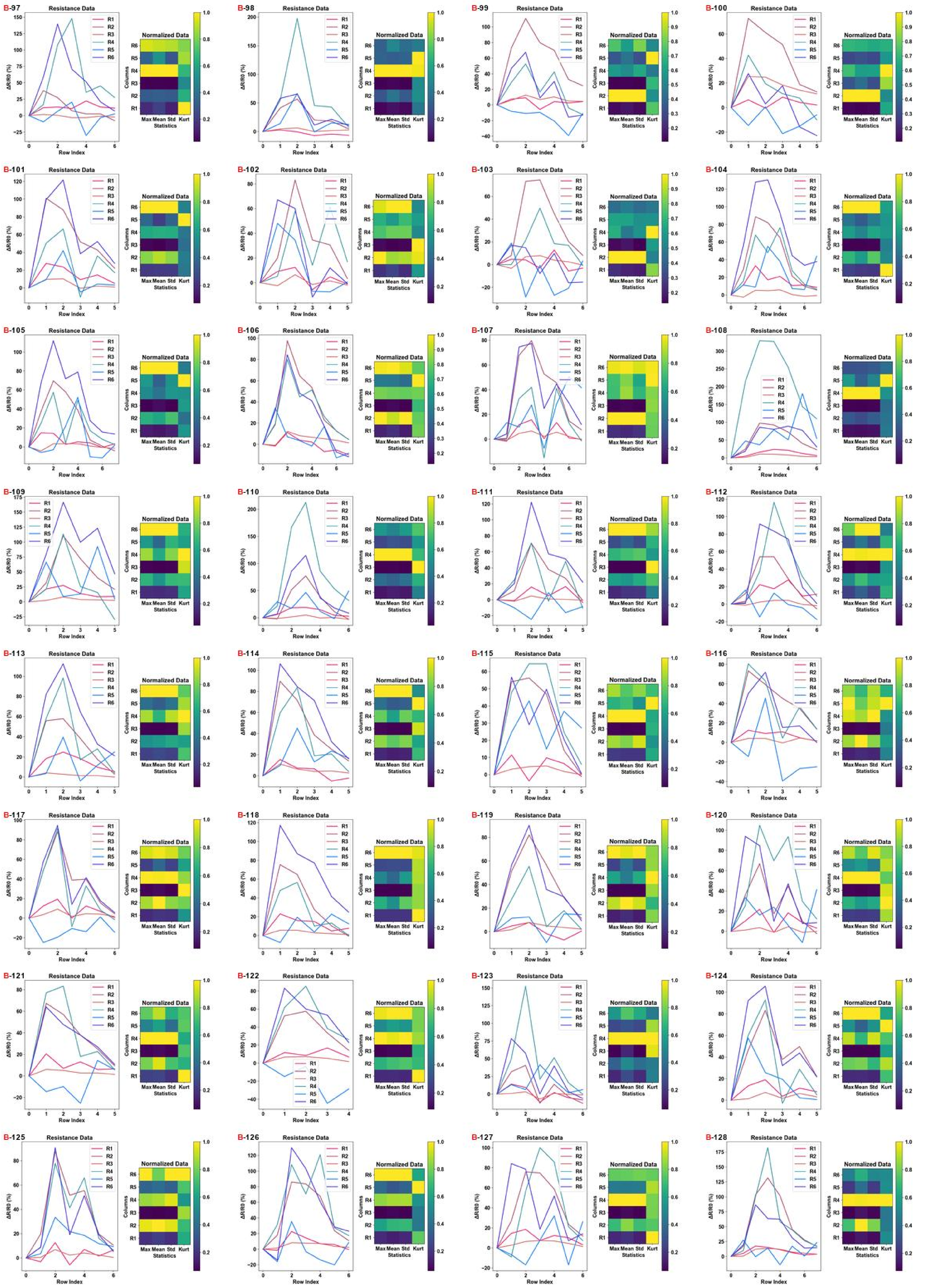

B-97
B-98
B-99
B-100
B-101
B-102
B-103
B-104
B-105
B-106
B-107
B-108
B-109
B-110
B-111
B-112
B-113
B-114
B-115
B-116
B-117
B-118
B-119
B-120
B-121
B-122
B-123
B-124
B-125
B-126
B-127
B-128
Resistance Data
Normalized Data
ΔR/R0 (%)
Row Index
Columns
Statistics
Max Mean Std Kurt
R1
R2
R3
R4
R5
R6

B-129
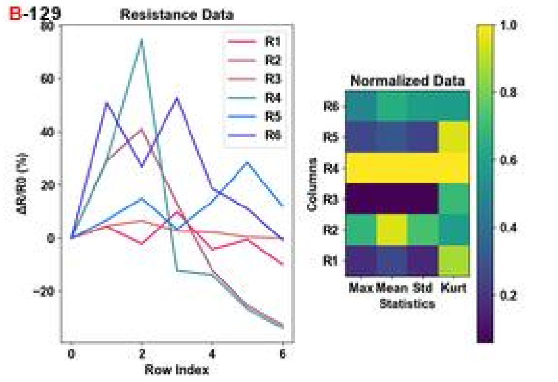

B-130
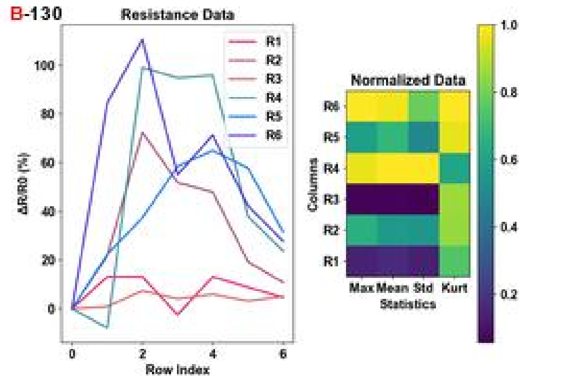

B-131
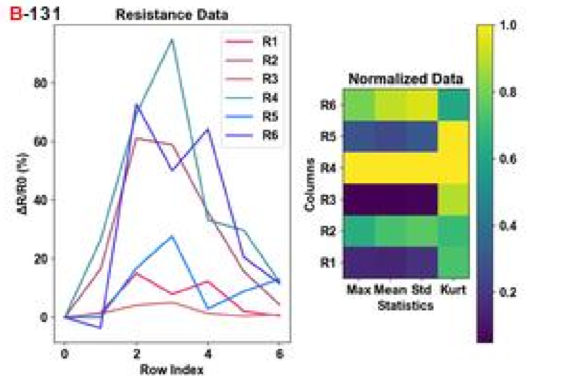

B-132
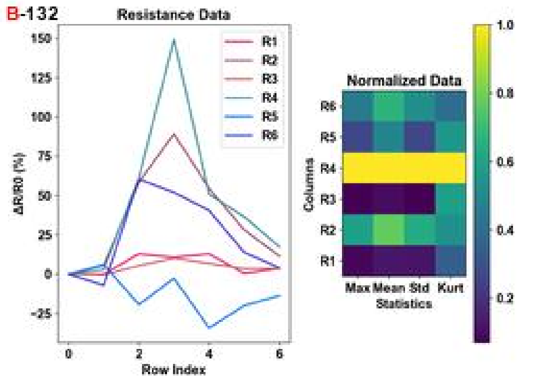

B-133
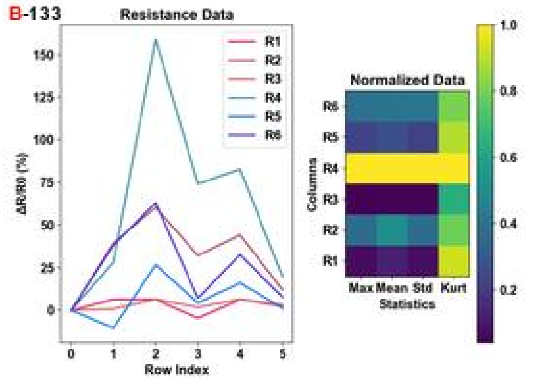

B-134
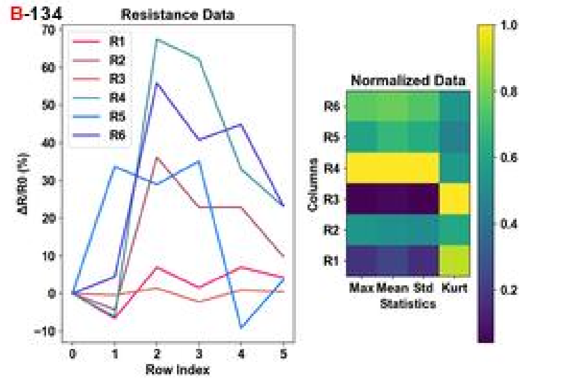

B-135
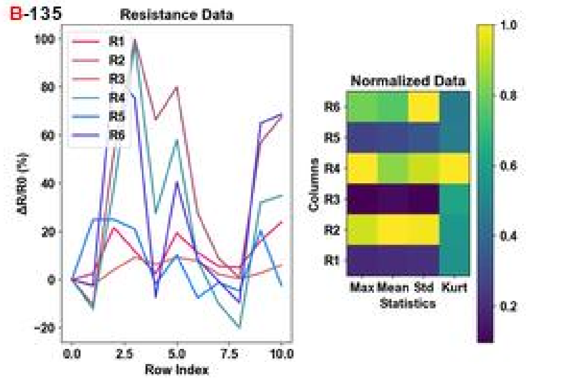

B-136
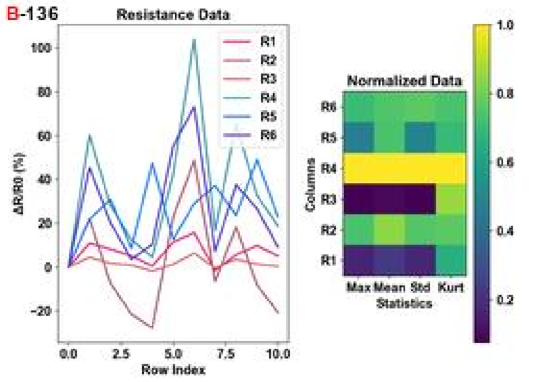

B-137
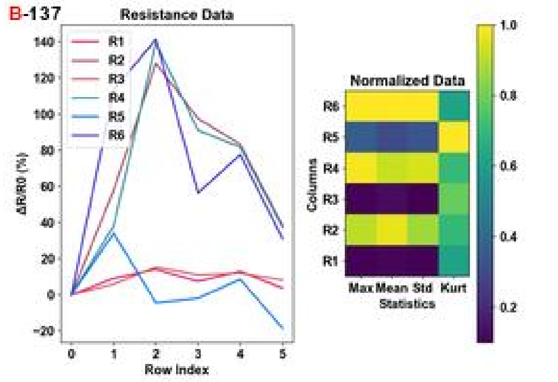

B-138
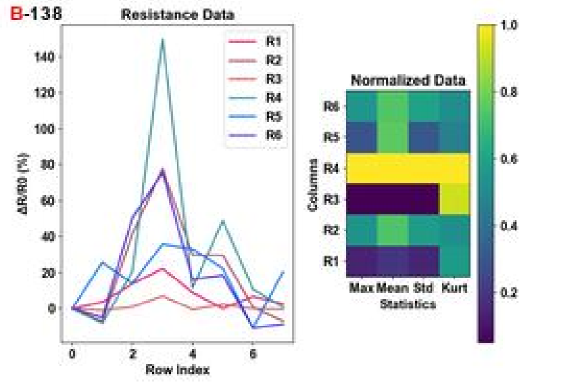

B-139
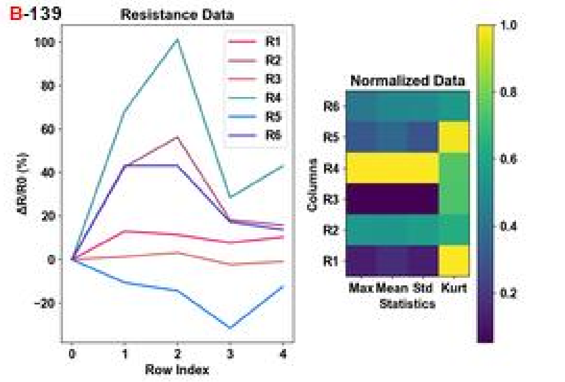

B-140
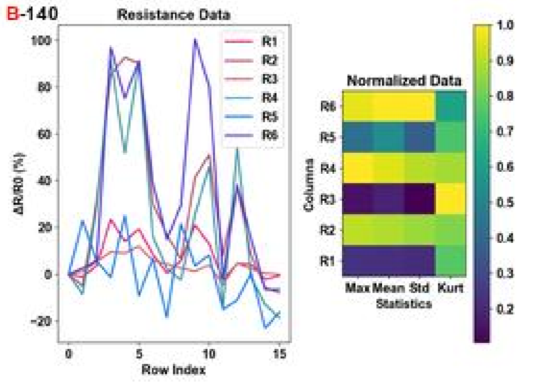

B-141
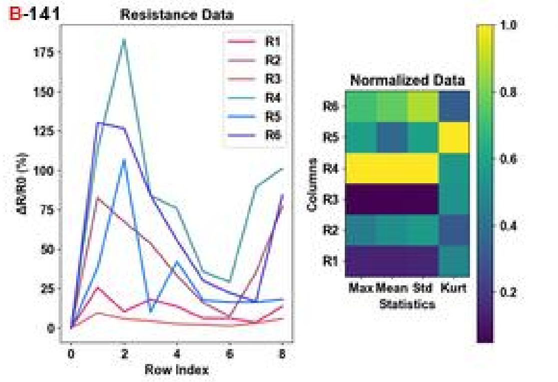

B-142
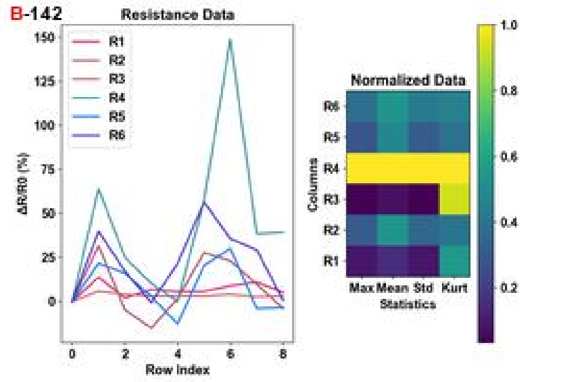

B-143
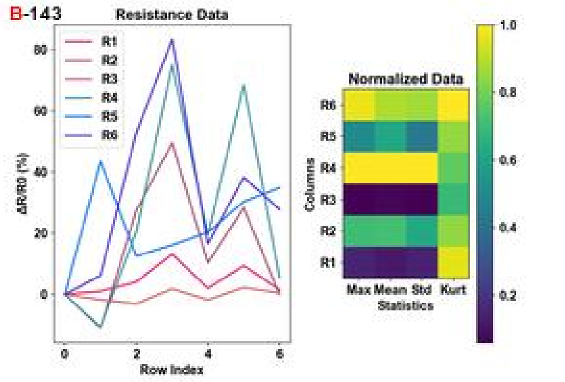

B-144
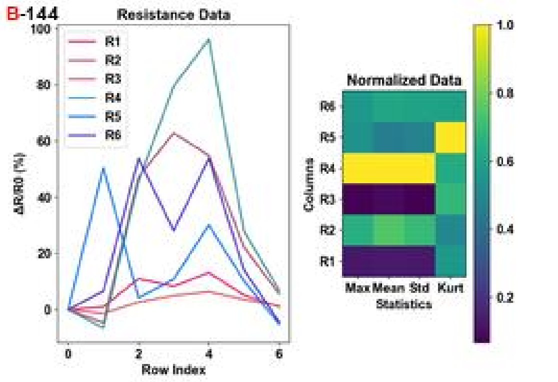

B-145
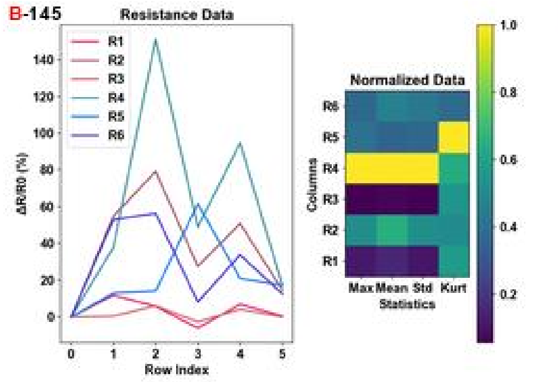

B-146
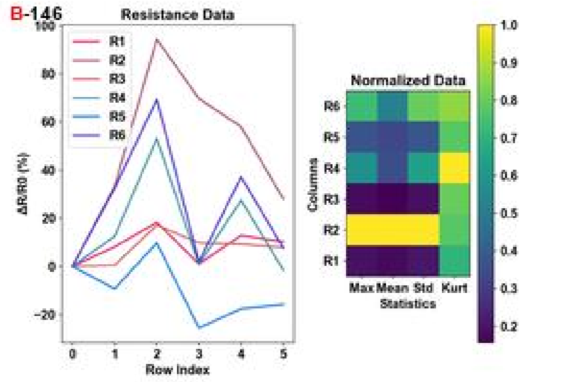

B-147
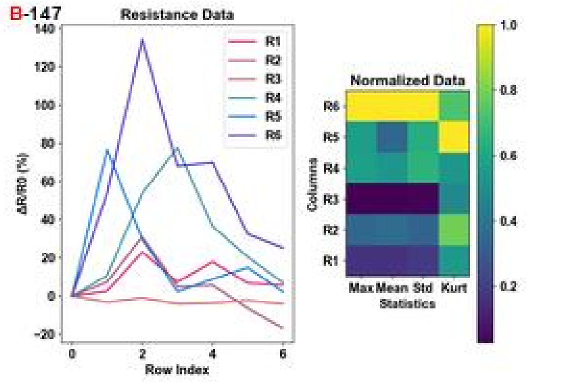

B-148
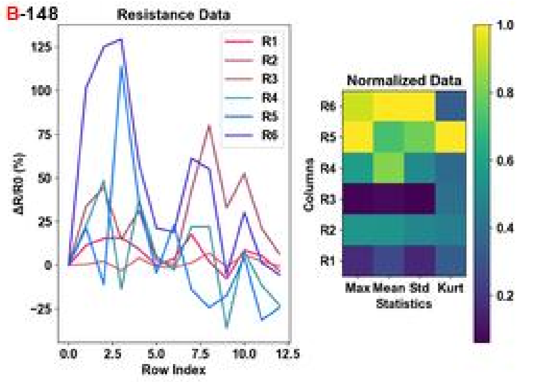

B-149
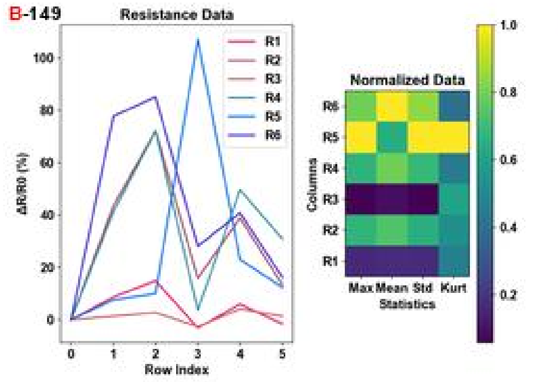

B-150
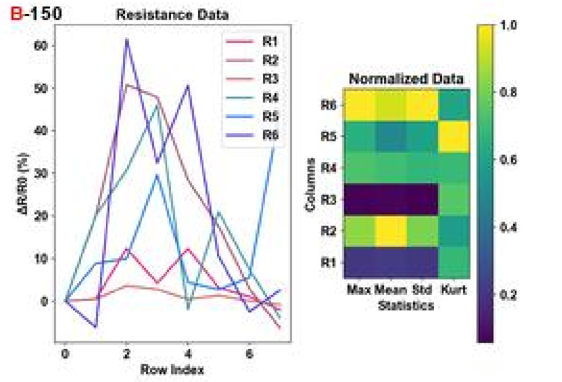

B-151
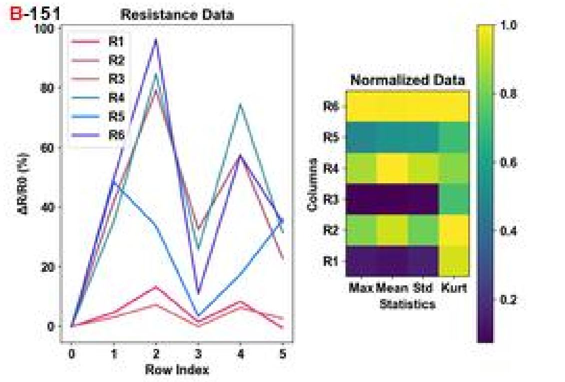

B-152
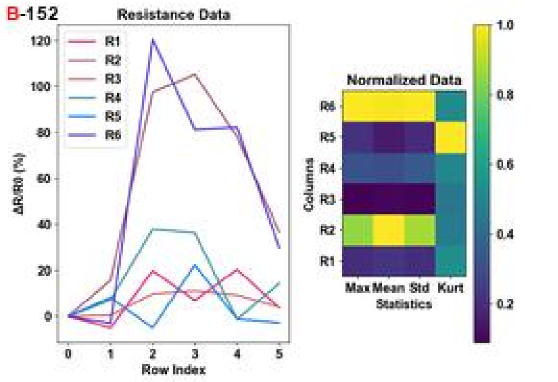

B-153
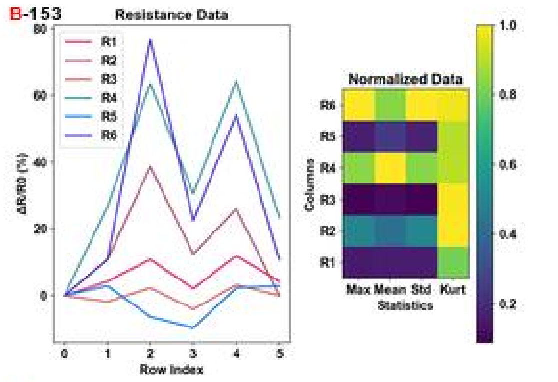

B-154
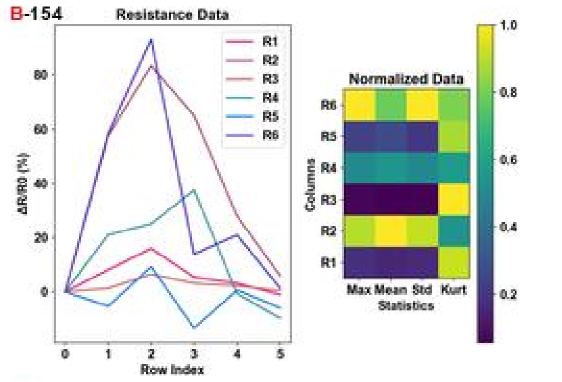

B-155
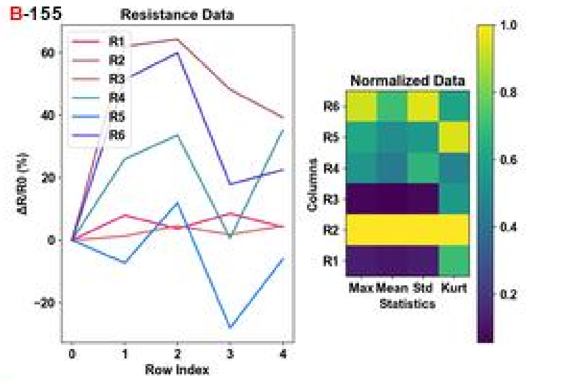

B-156
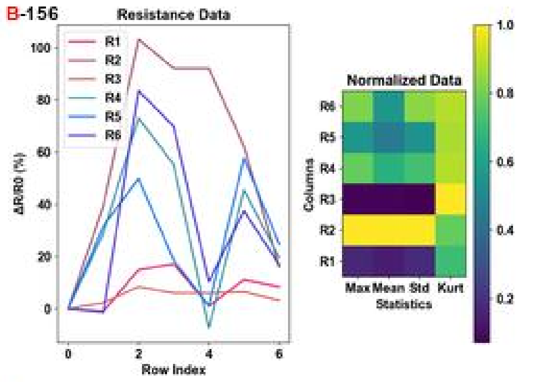

B-157
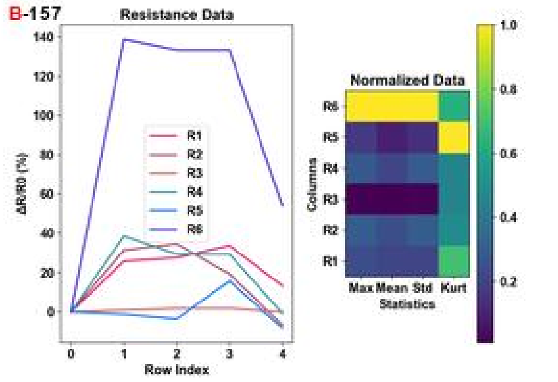

B-158
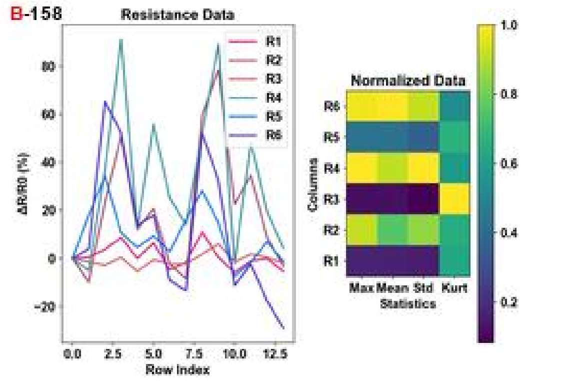

B-159
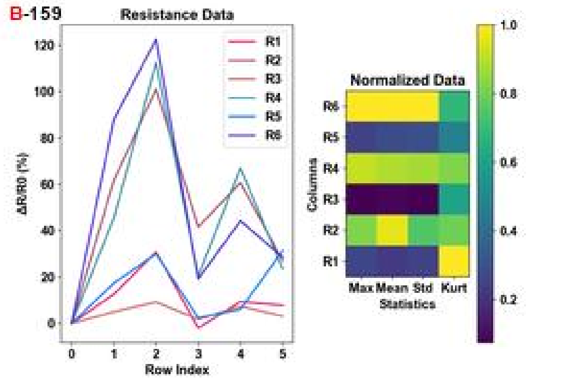

B-160
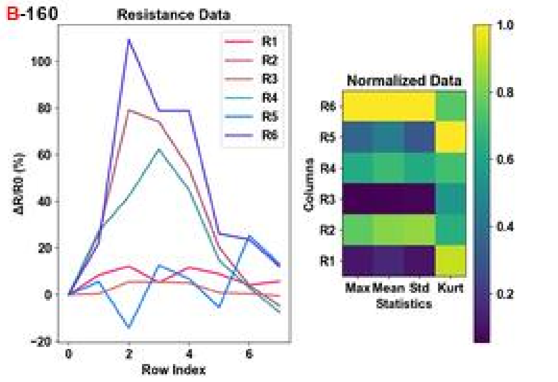

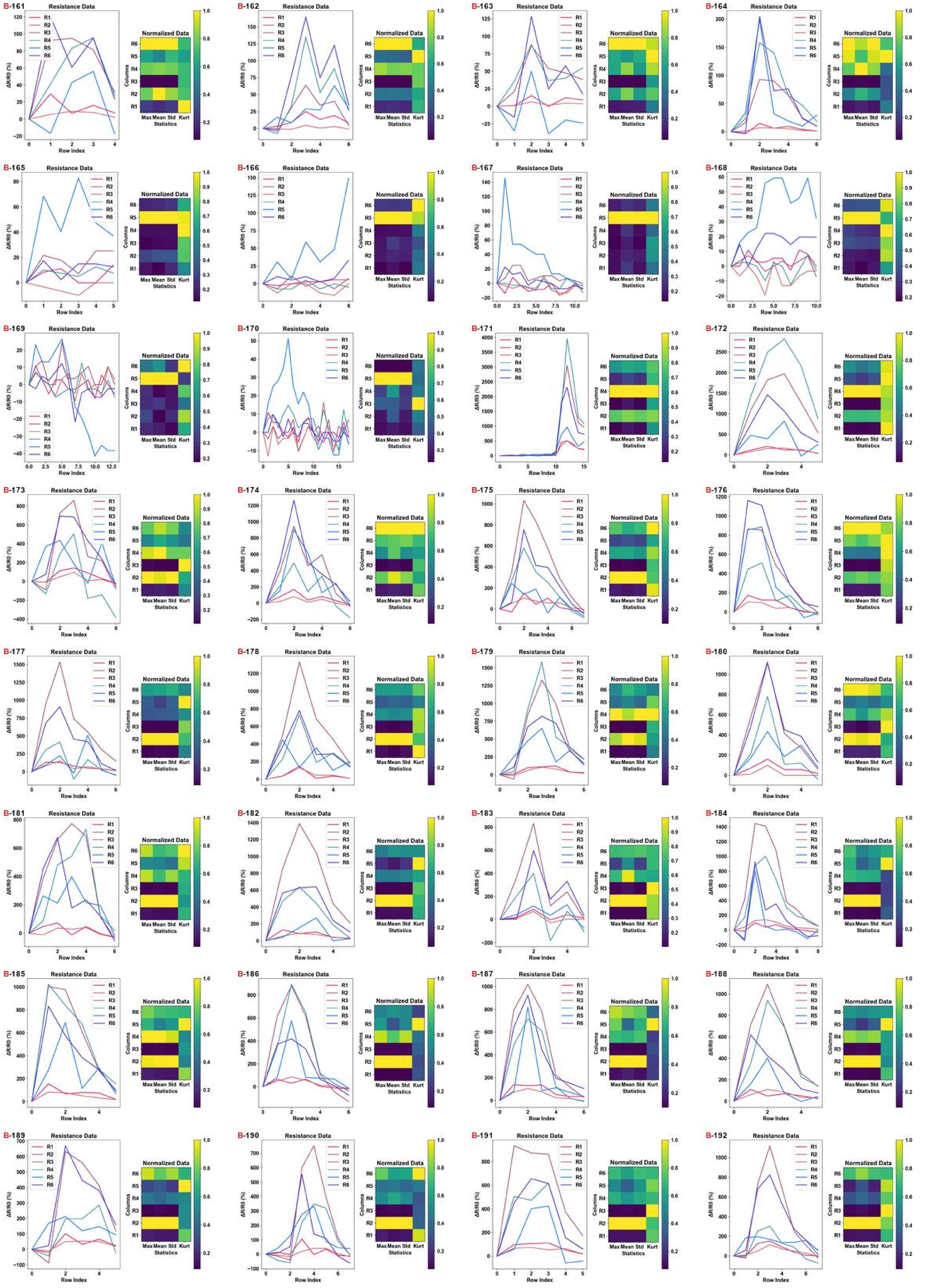
B-161
B-162
B-163
B-164
B-165
B-166
B-167
B-168
B-169
B-170
B-171
B-172
B-173
B-174
B-175
B-176
B-177
B-178
B-179
B-180
B-181
B-182
B-183
B-184
B-185
B-186
B-187
B-188
B-189
B-190
B-191
B-192
Resistance Data
Normalized Data
ΔR/R0 (%)
Row Index
Columns
Statistics
Max Mean Std Kurt
R1
R2
R3
R4
R5
R6

Pressure-sensitive data of type B

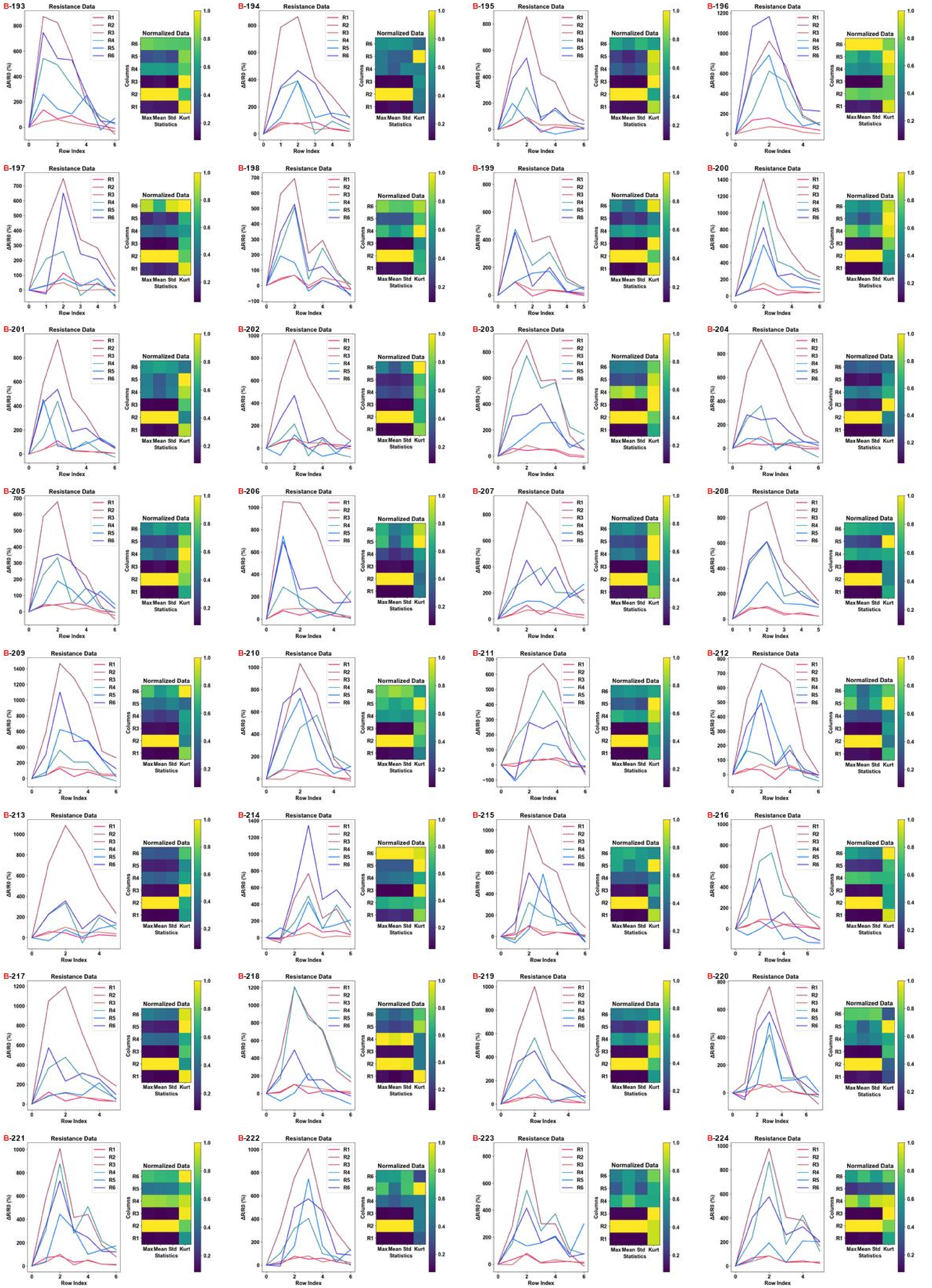

Pressure-sensitive data of type B

B-225
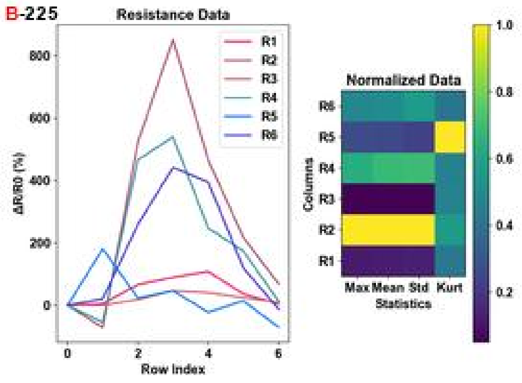

B-226
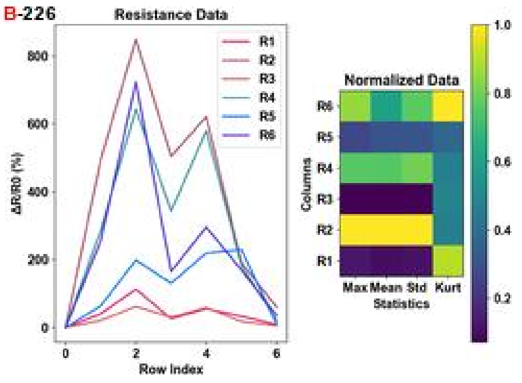

B-227
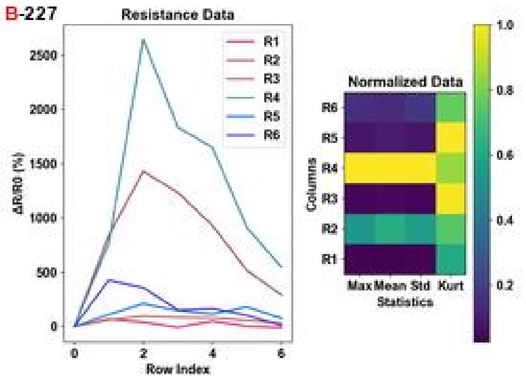

B-228
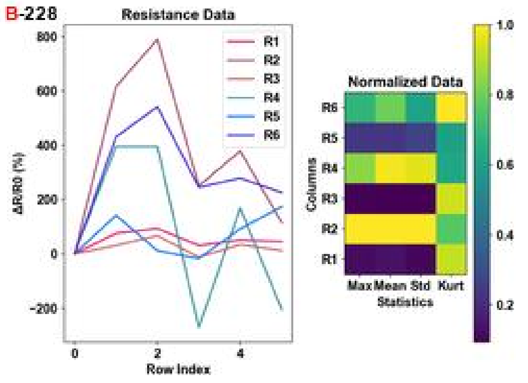

B-229
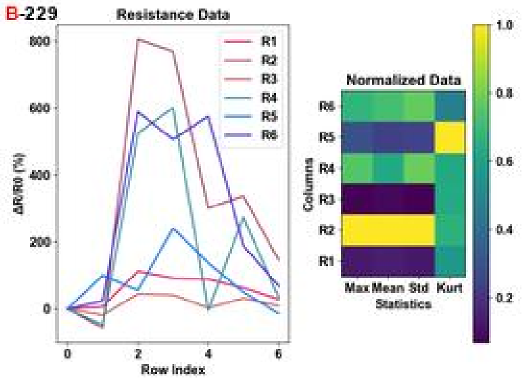

B-230
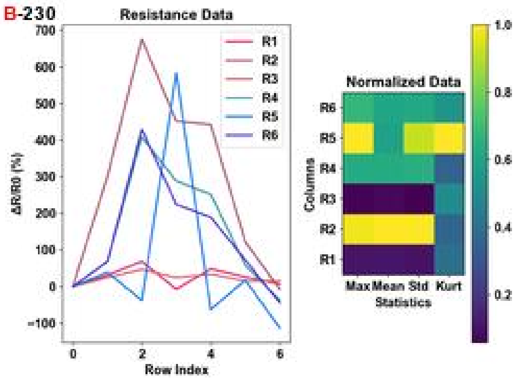

B-231
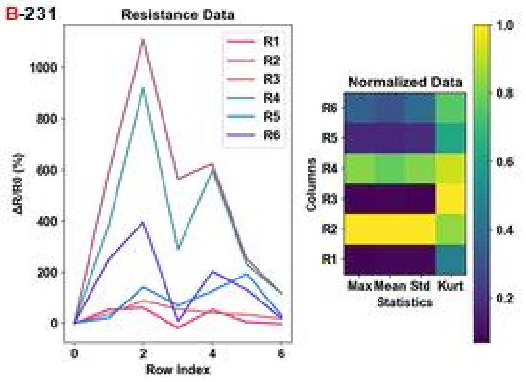

B-232
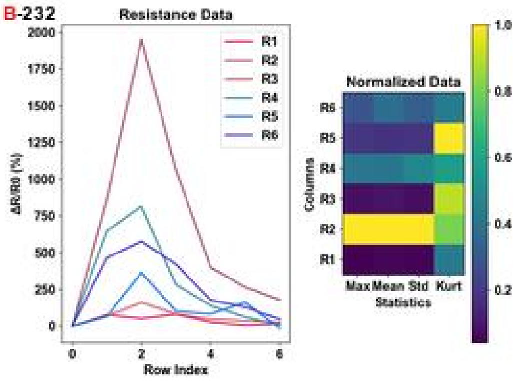

B-233
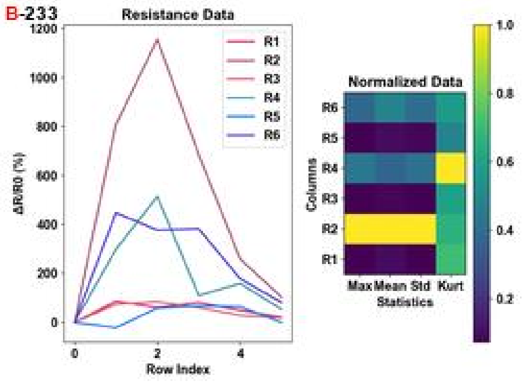

B-234
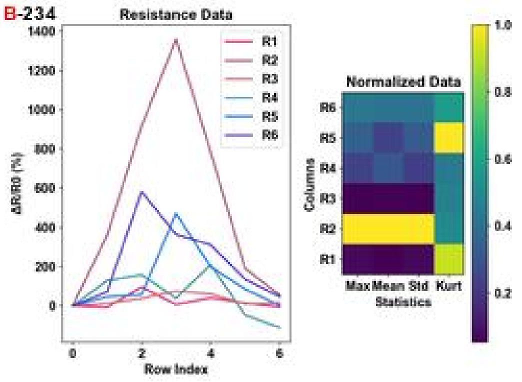

B-235
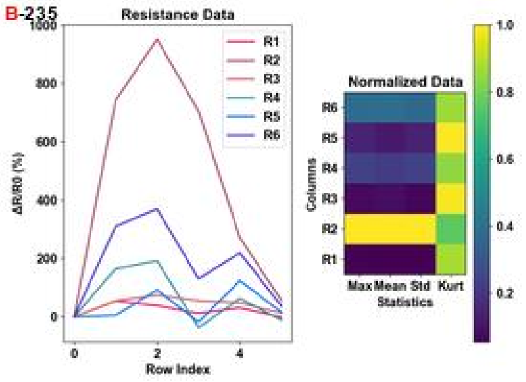

B-236
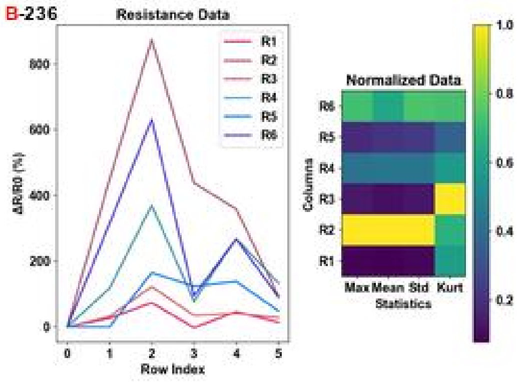

B-237
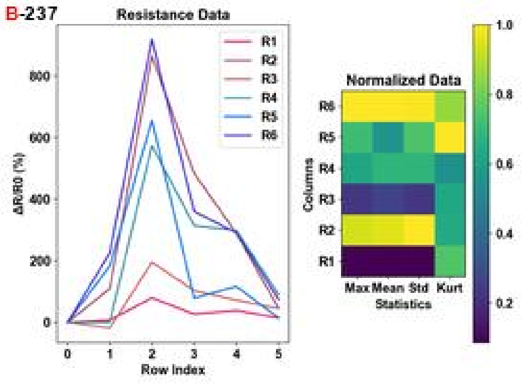

B-238
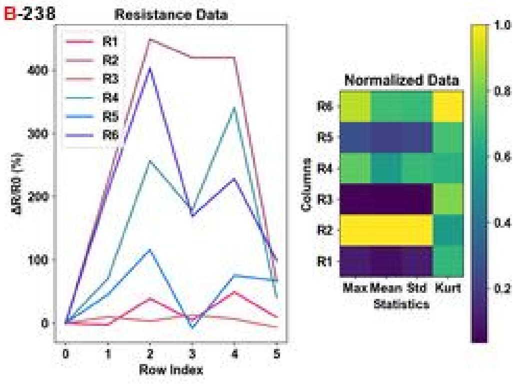

B-239
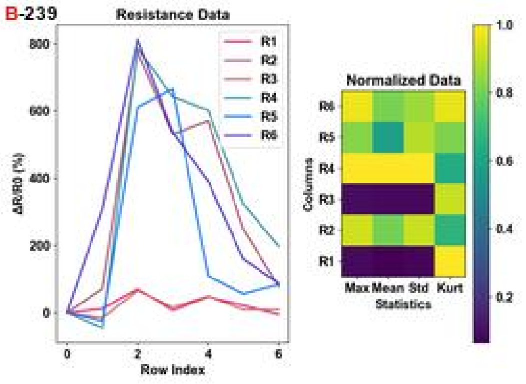

B-240
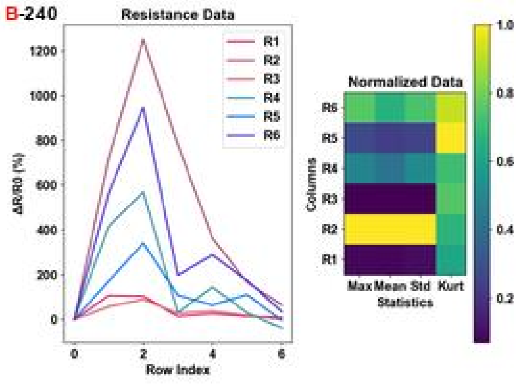

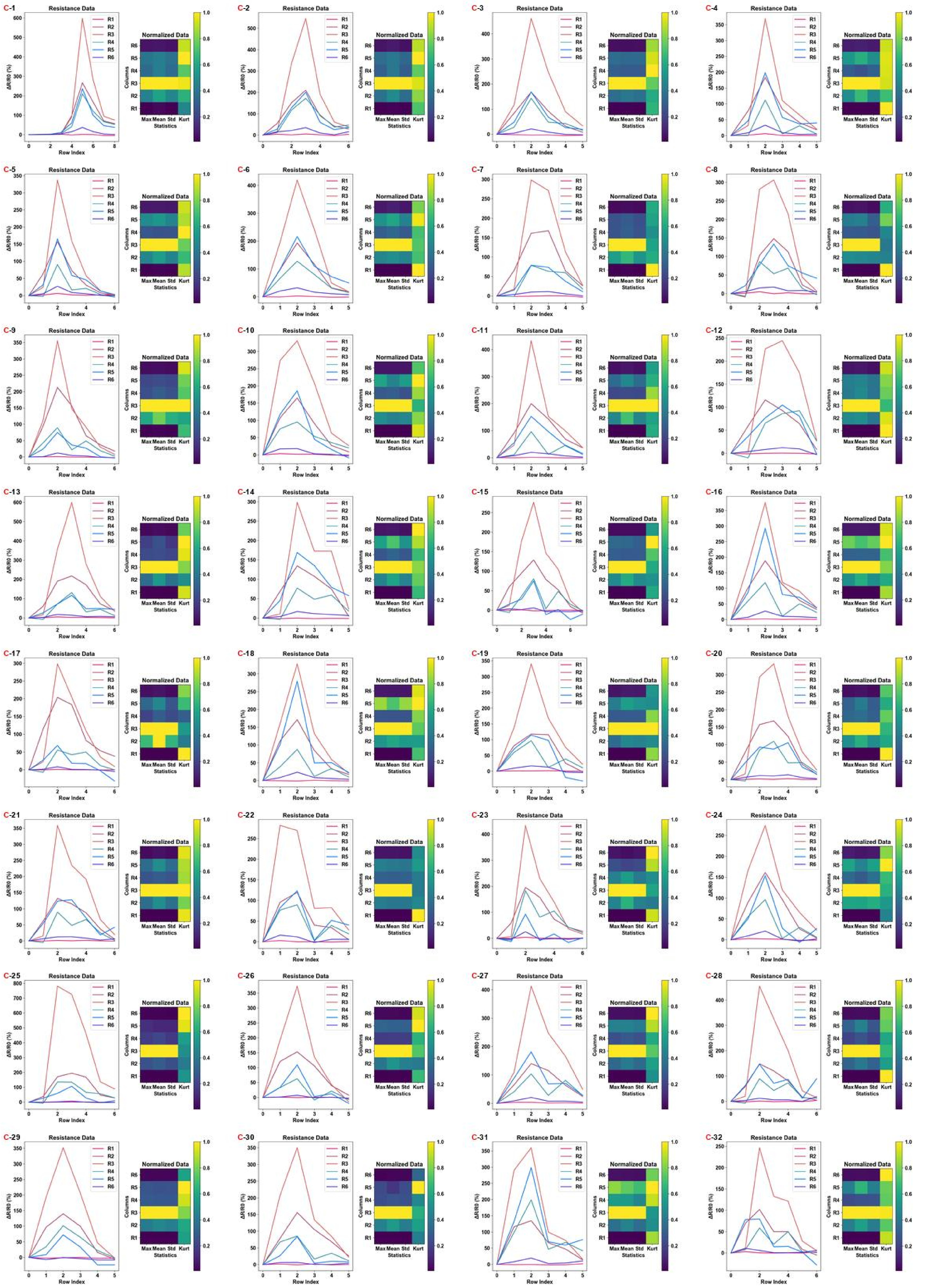
C-1 Resistance Data
C-2 Resistance Data
C-3 Resistance Data
C-4 Resistance Data
C-5 Resistance Data
C-6 Resistance Data
C-7 Resistance Data
C-8 Resistance Data
C-9 Resistance Data
C-10 Resistance Data
C-11 Resistance Data
C-12 Resistance Data
C-13 Resistance Data
C-14 Resistance Data
C-15 Resistance Data
C-16 Resistance Data
C-17 Resistance Data
C-18 Resistance Data
C-19 Resistance Data
C-20 Resistance Data
C-21 Resistance Data
C-22 Resistance Data
C-23 Resistance Data
C-24 Resistance Data
C-25 Resistance Data
C-26 Resistance Data
C-27 Resistance Data
C-28 Resistance Data
C-29 Resistance Data
C-30 Resistance Data
C-31 Resistance Data
C-32 Resistance Data
ΔR/R0 (%)
Row Index
R1 R2 R3 R4 R5 R6
Normalized Data
Columns
Max Mean Std Kurt
Statistics

Pressure-sensitive data of type C

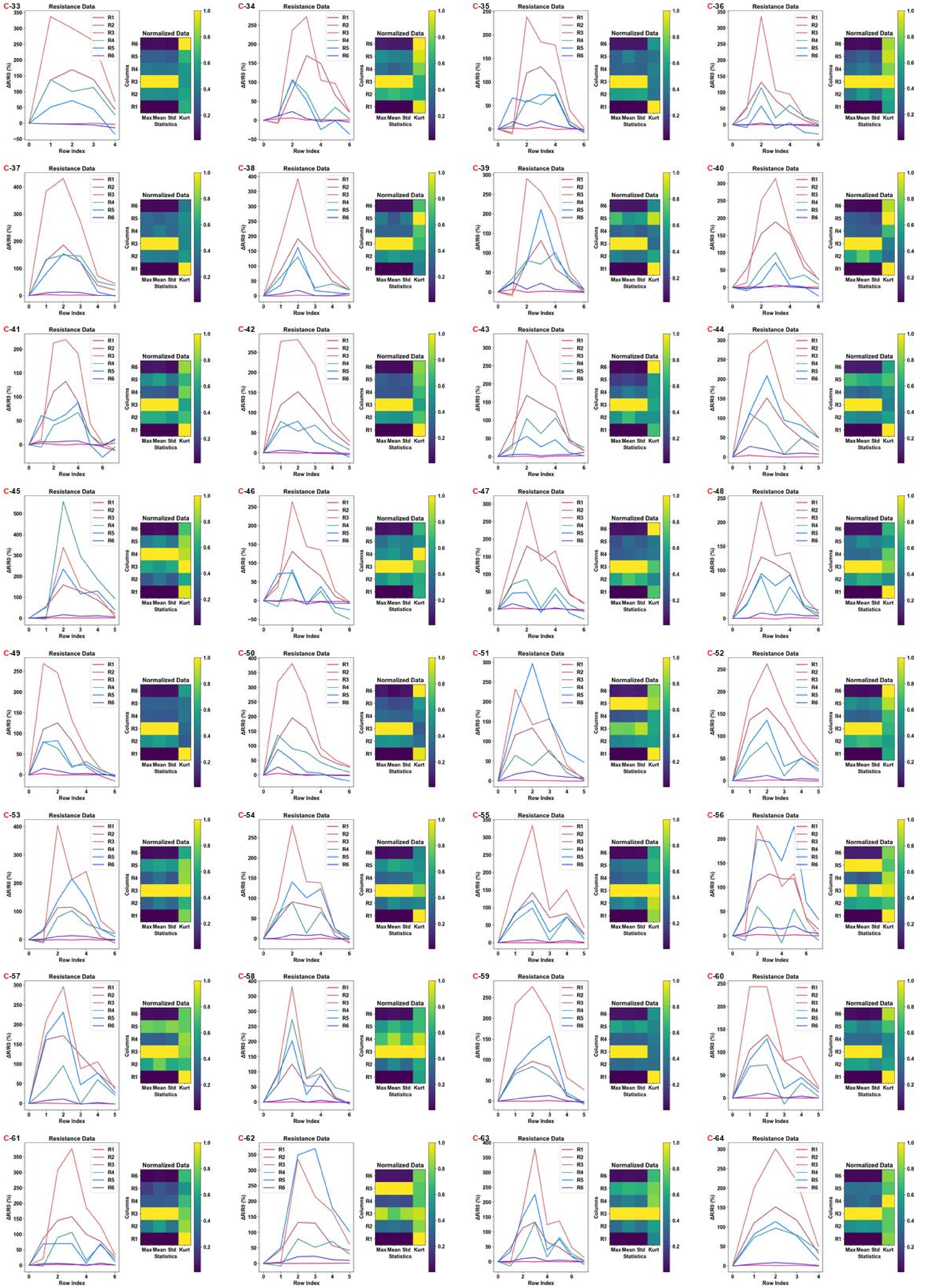

C-65
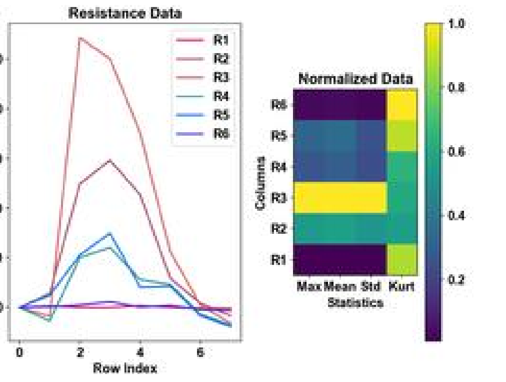

C-66
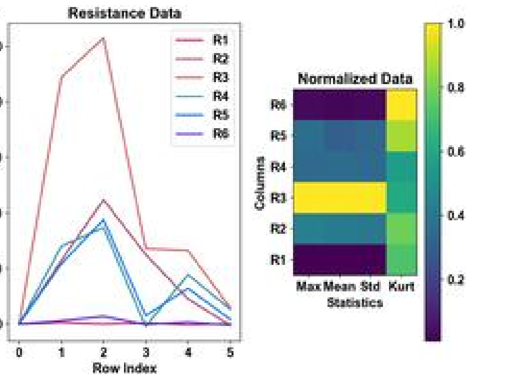

C-67
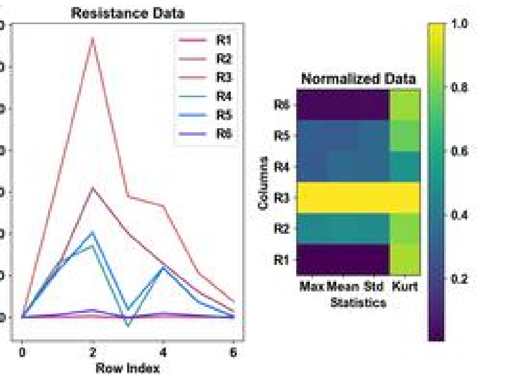

C-68
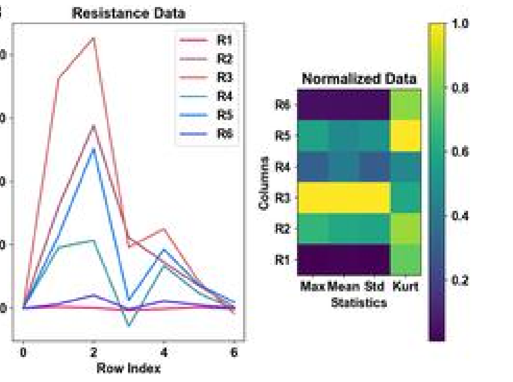

C-69
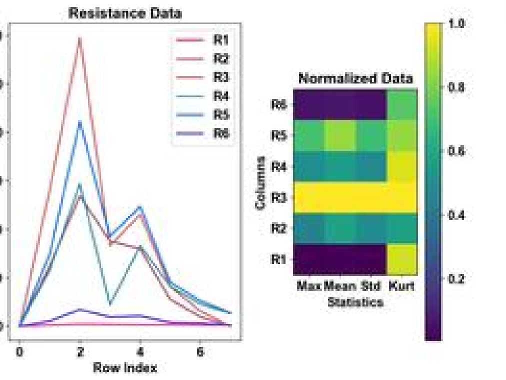

C-70
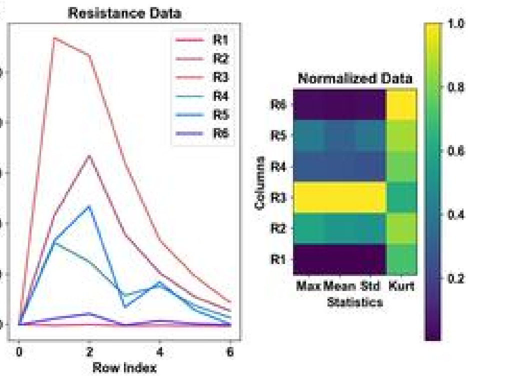

C-71
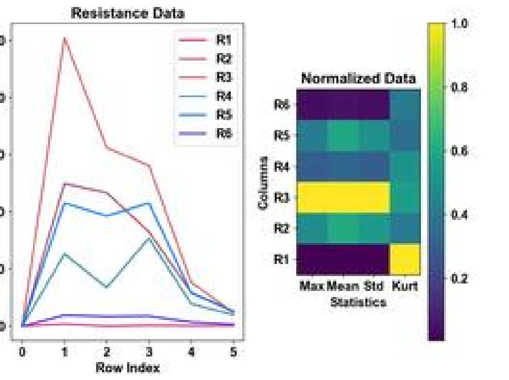

C-72
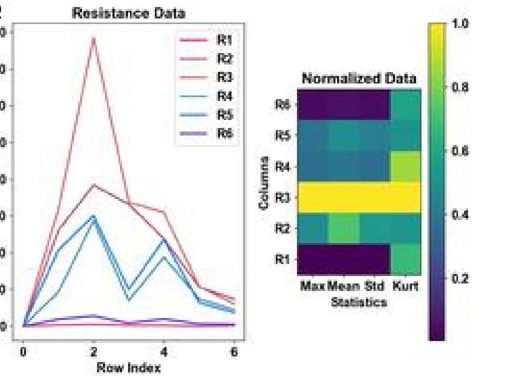

C-73
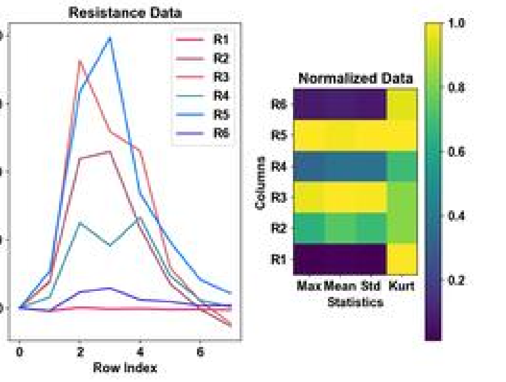

C-74
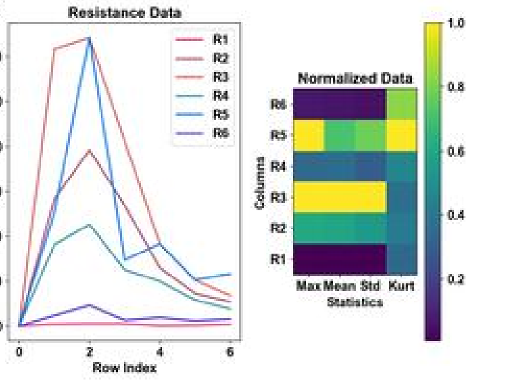

C-75
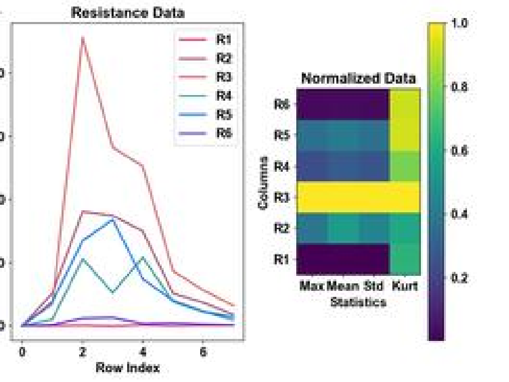

C-76
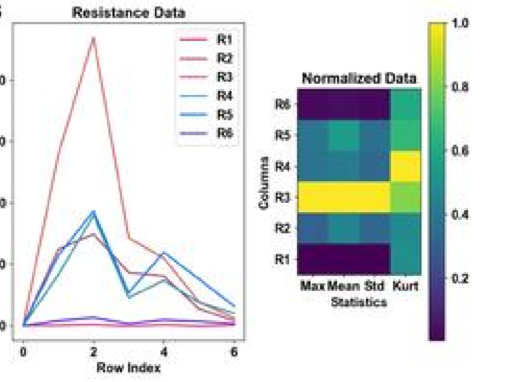

C-77
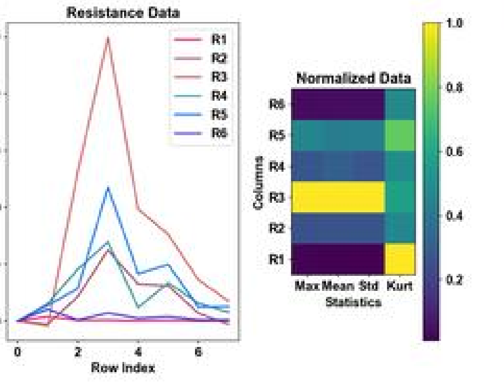

C-78
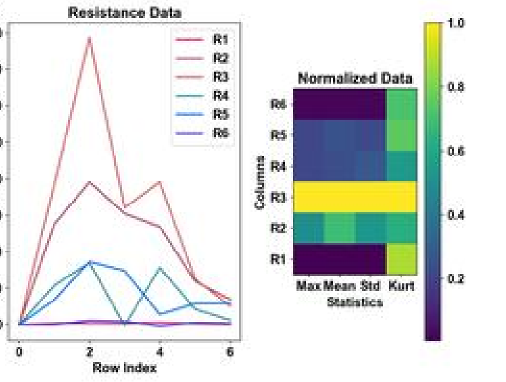

C-79
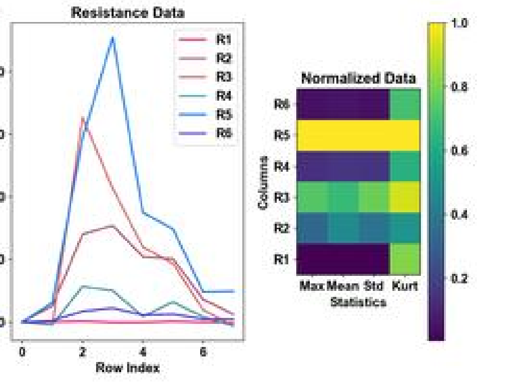

C-80
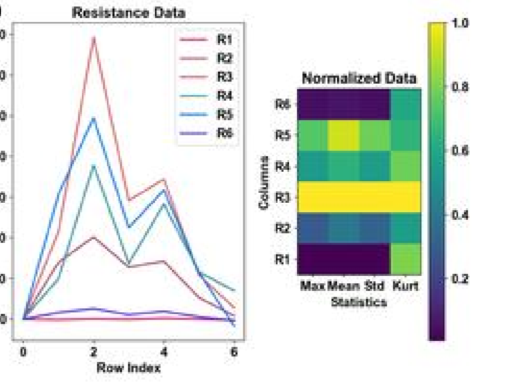

C-81
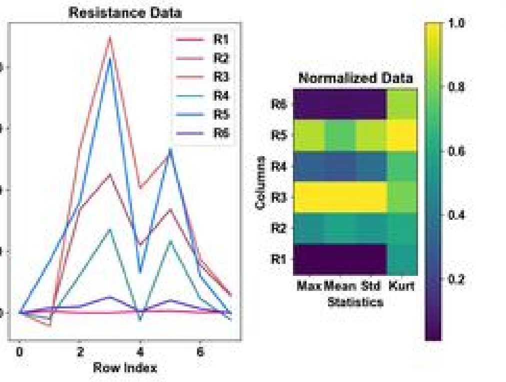

C-82
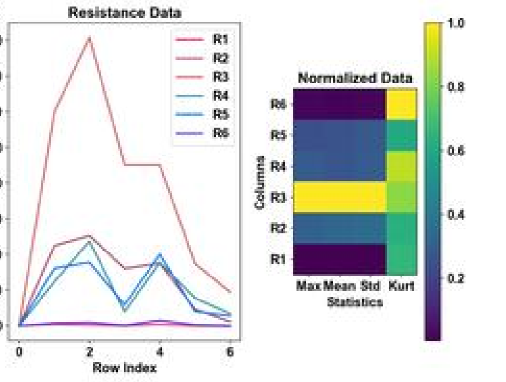

C-83
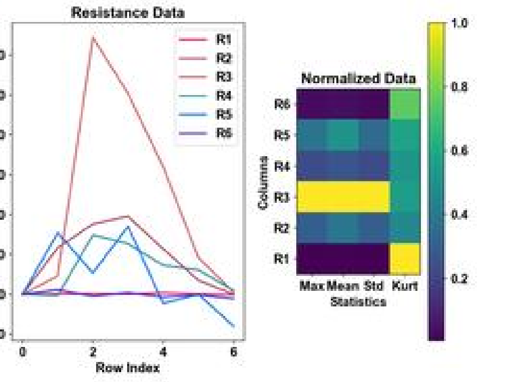

C-84
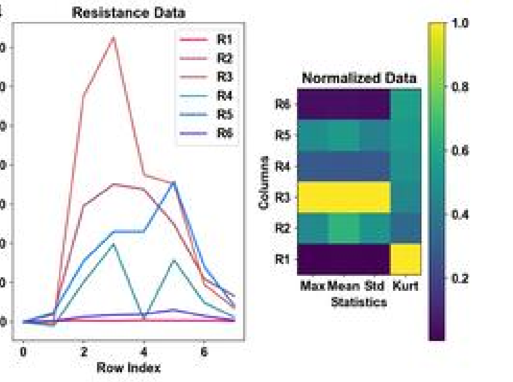

C-85
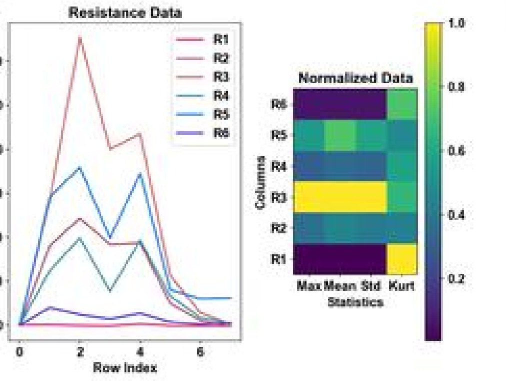

C-86
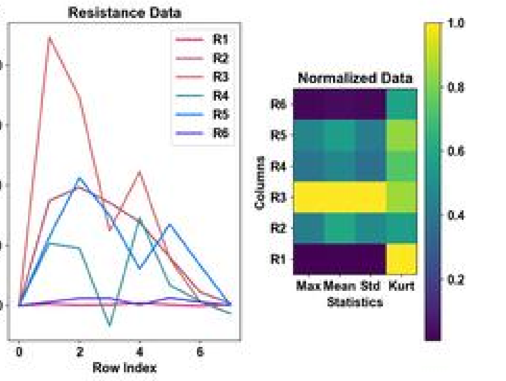

C-87
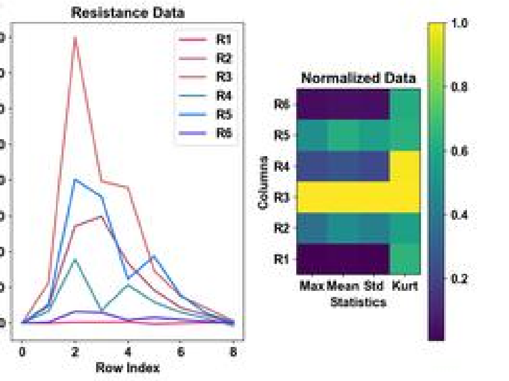

C-88
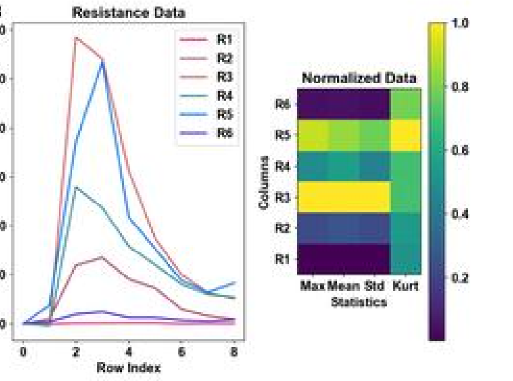

C-89
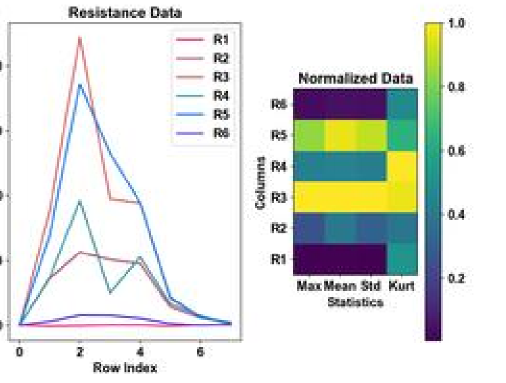

C-90
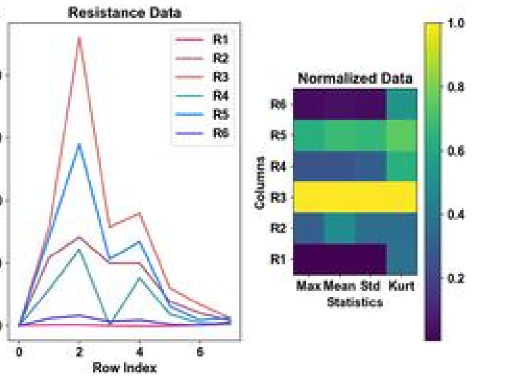

C-91
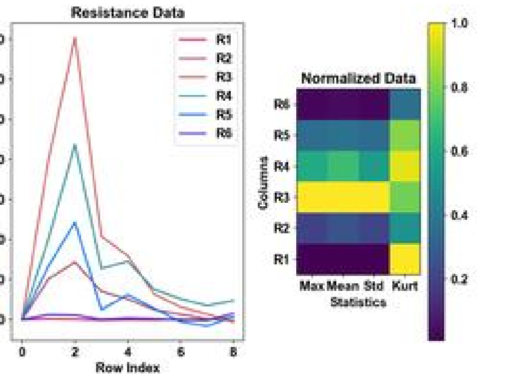

C-92
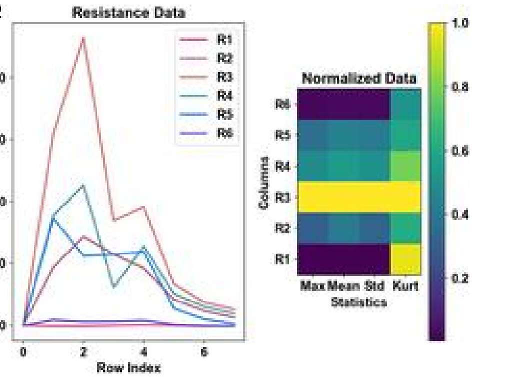

C-93
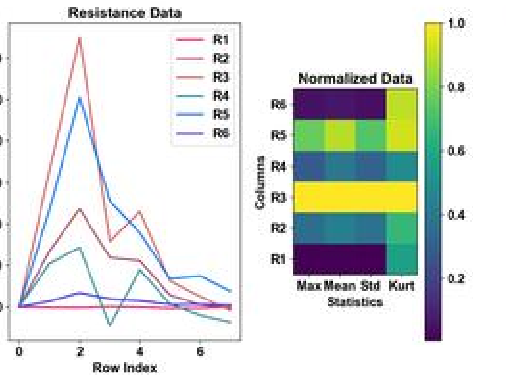

C-94
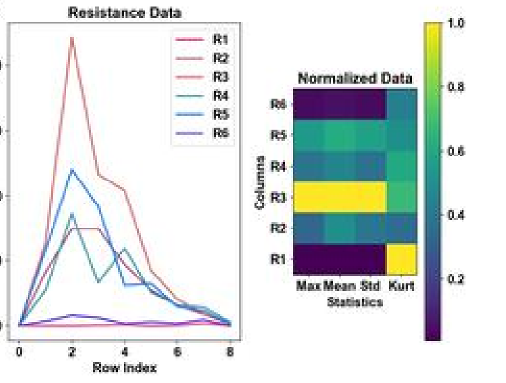

C-95
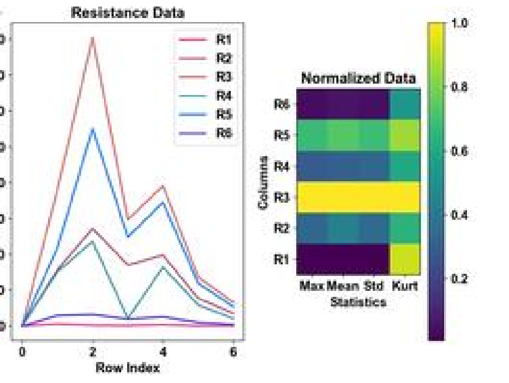

C-96
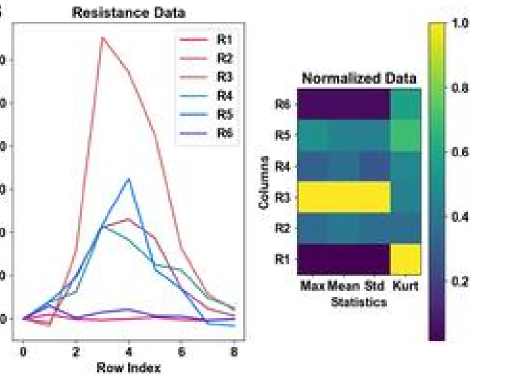

C-97
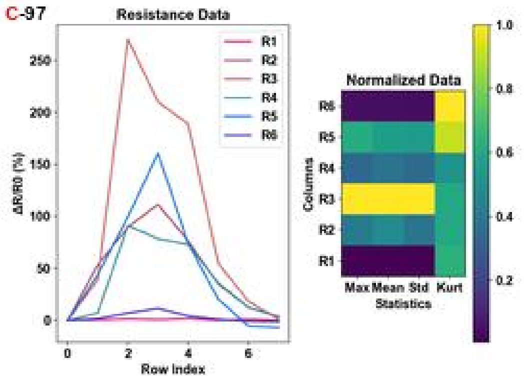

C-98
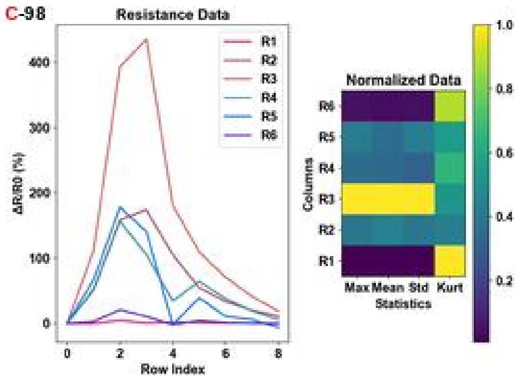

C-99
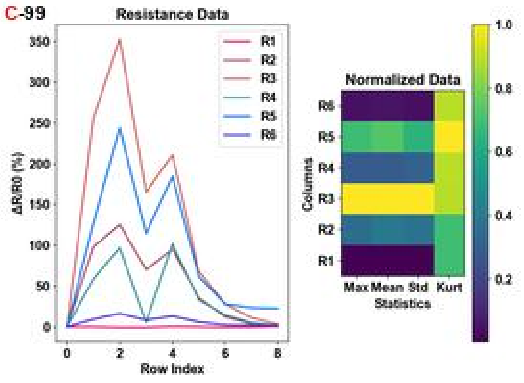

C-100
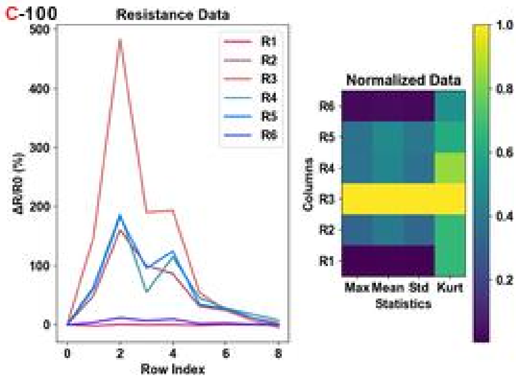

C-101
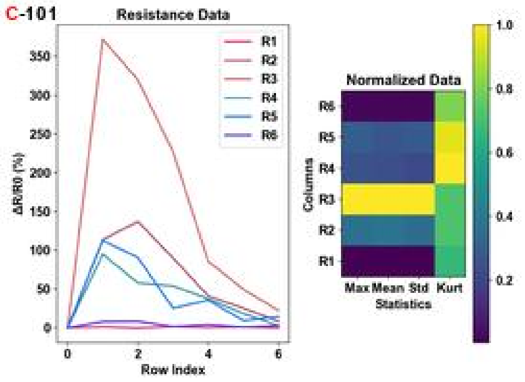

C-102
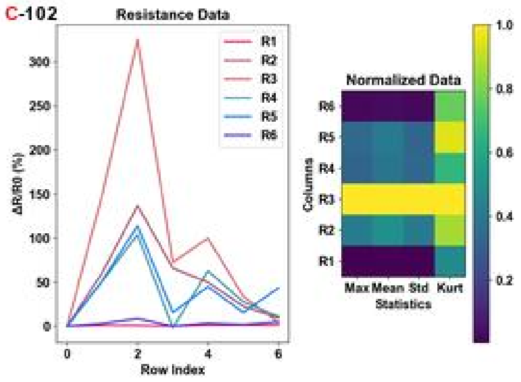

C-103
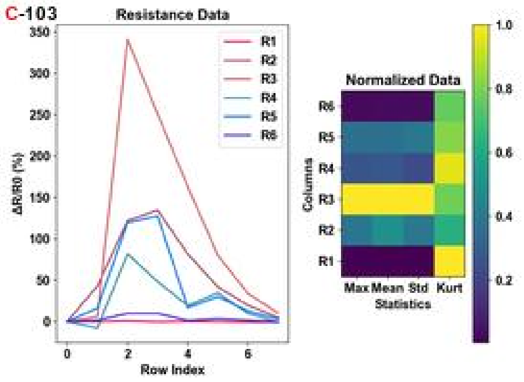

C-104
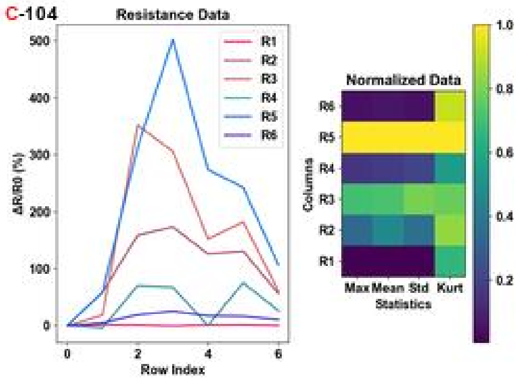

C-105
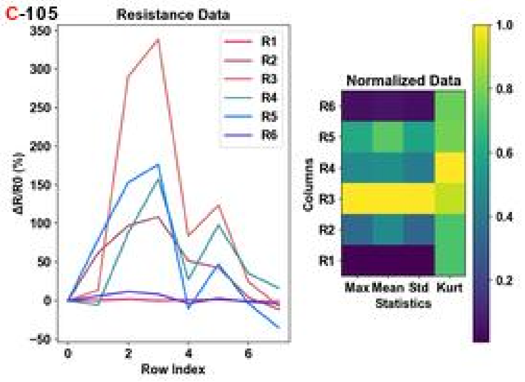

C-106
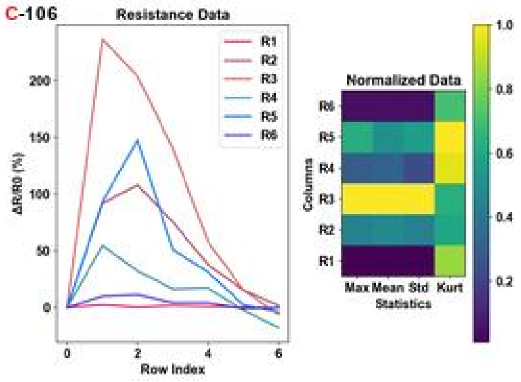

C-107
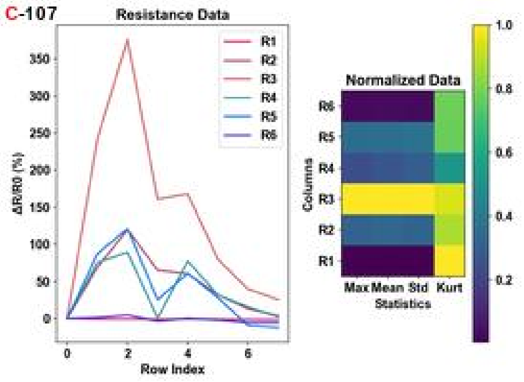

C-108
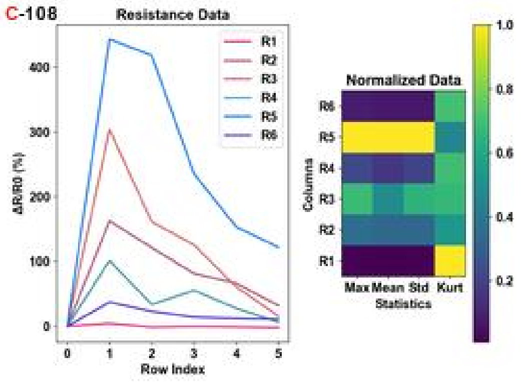

C-109
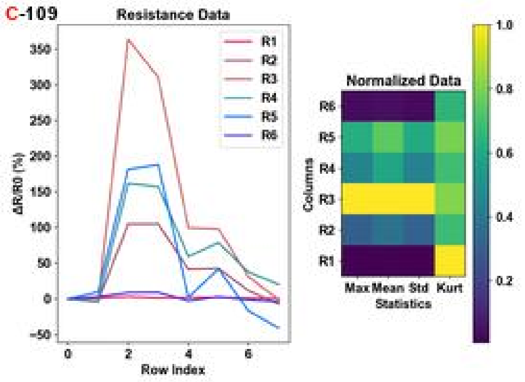

C-110
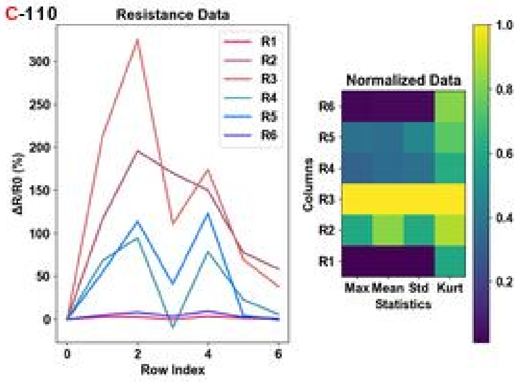

C-111
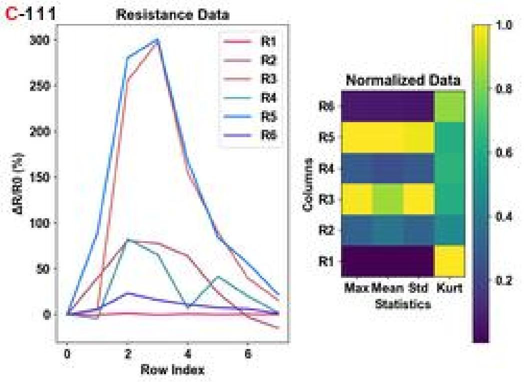

C-112
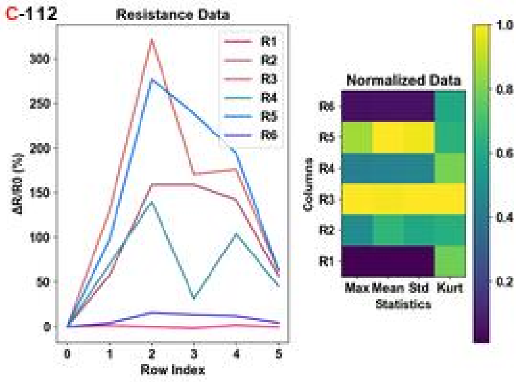

C-113
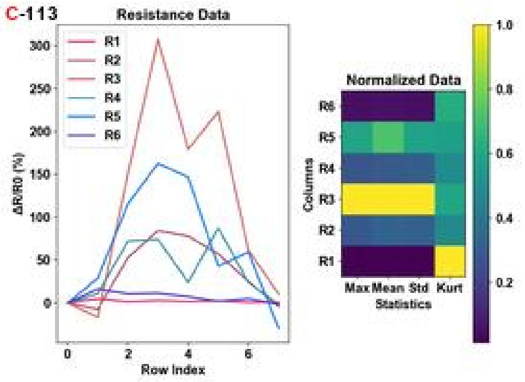

C-114
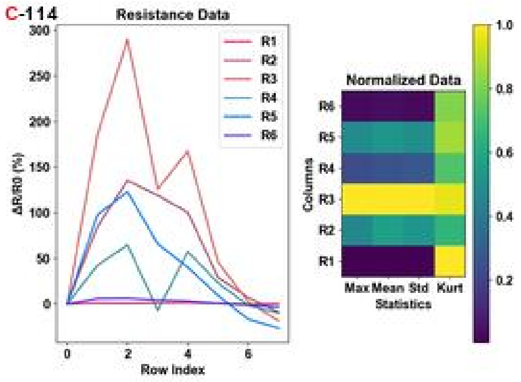

C-115
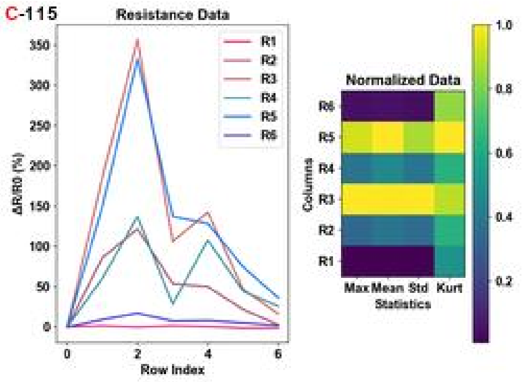

C-116
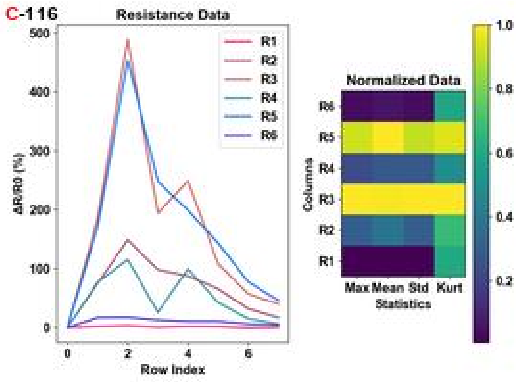

C-117
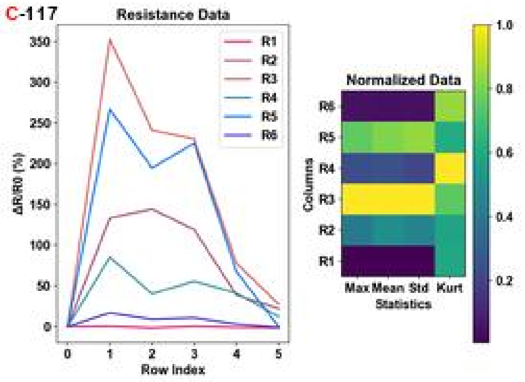

C-118
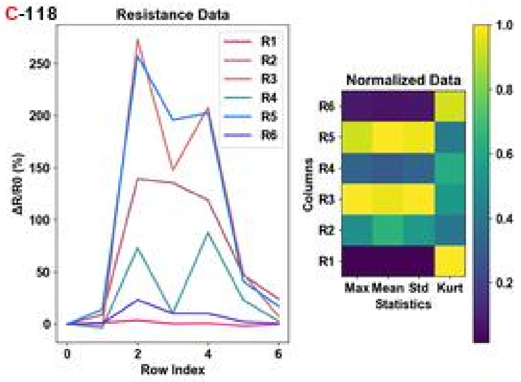

C-119
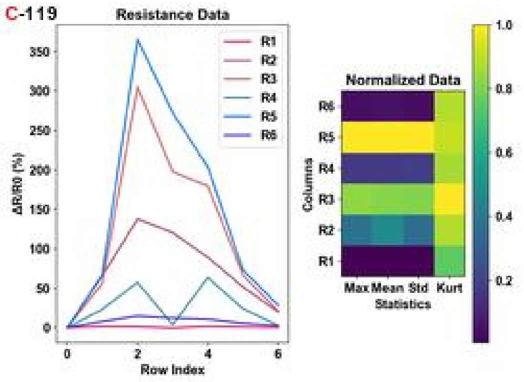

C-120
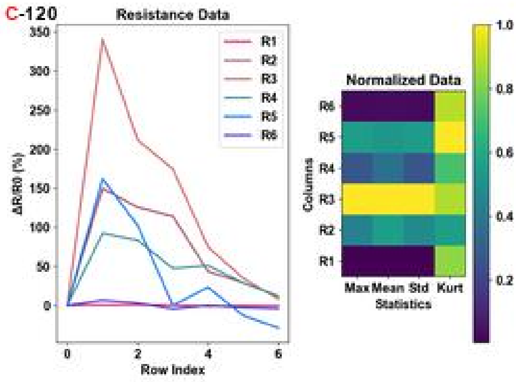

C-121
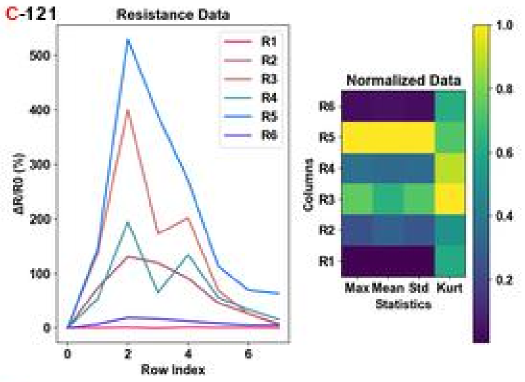

C-122
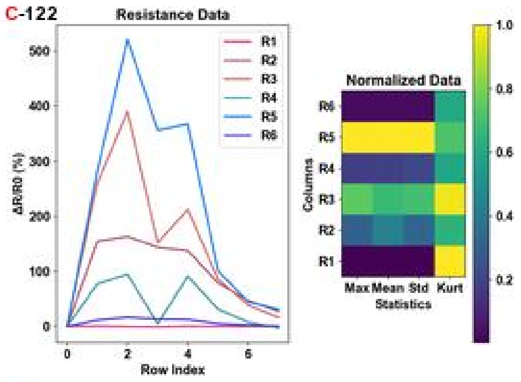

C-123
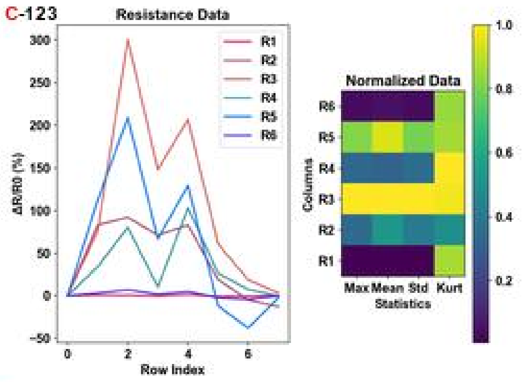

C-124
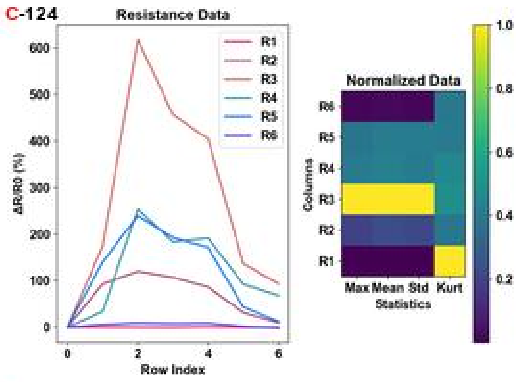

C-125
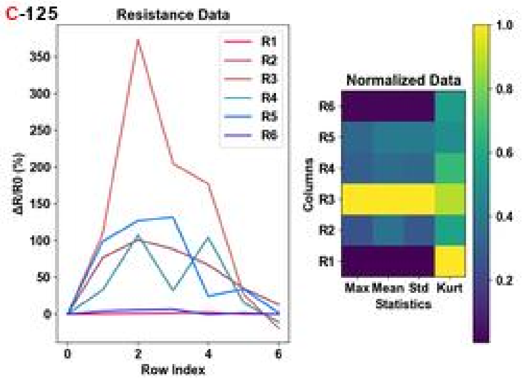

C-126
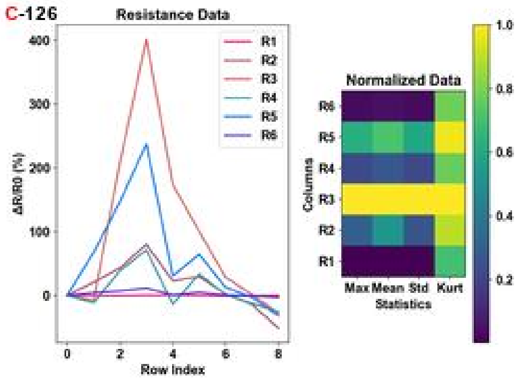

C-127
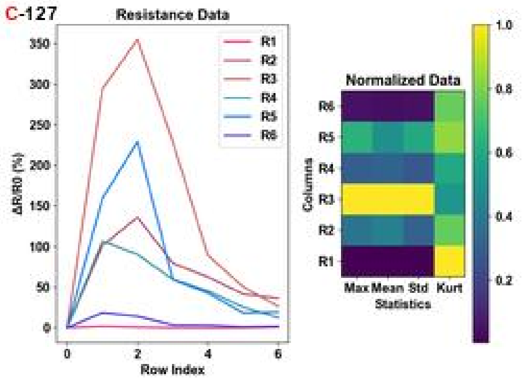

C-128
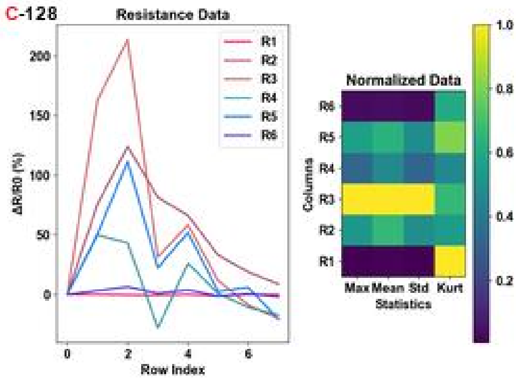

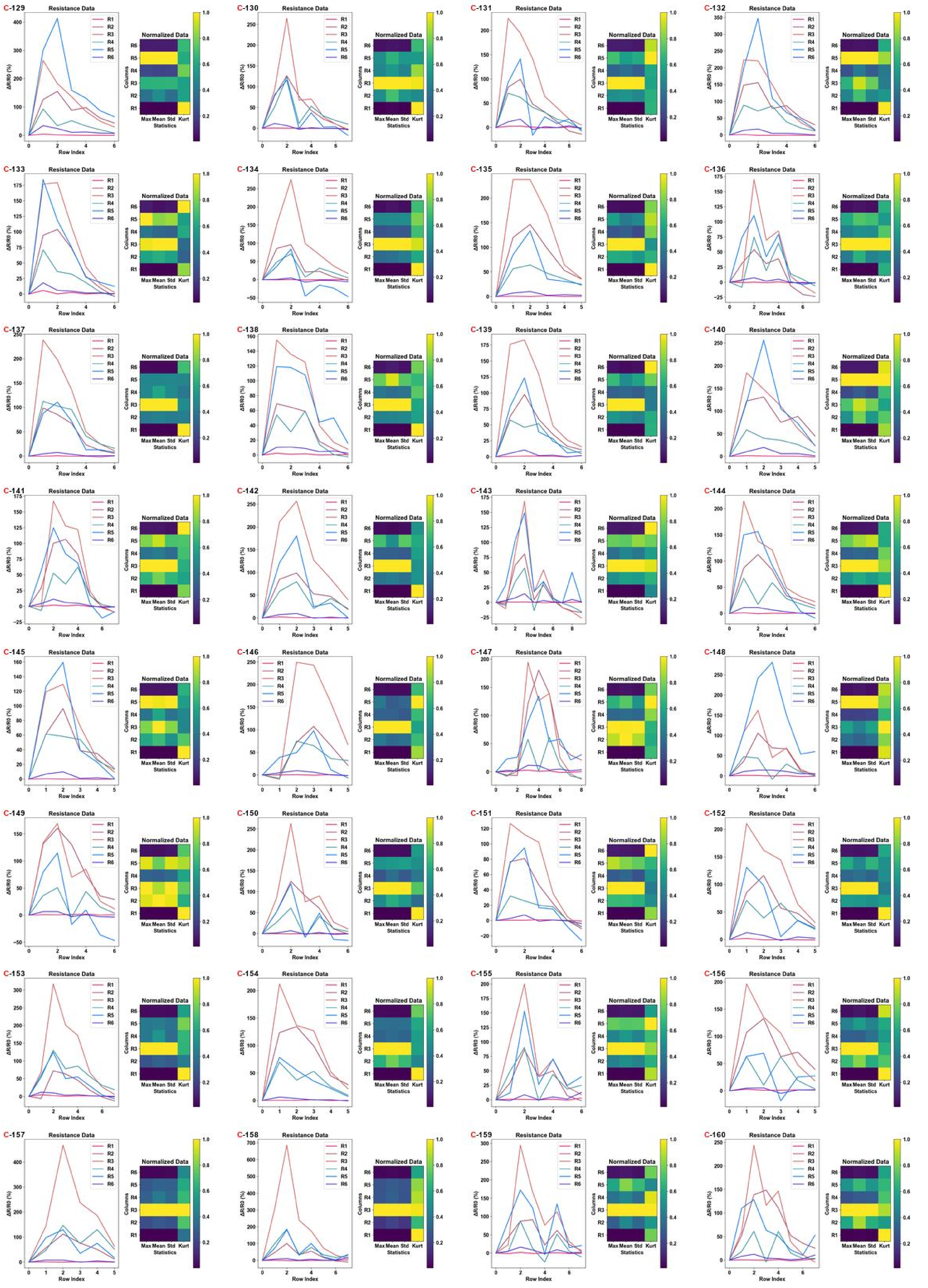
C-129
C-130
C-131
C-132
C-133
C-134
C-135
C-136
C-137
C-138
C-139
C-140
C-141
C-142
C-143
C-144
C-145
C-146
C-147
C-148
C-149
C-150
C-151
C-152
C-153
C-154
C-155
C-156
C-157
C-158
C-159
C-160
Resistance Data
ΔR/R0 (%)
Row Index
R1
R2
R3
R4
R5
R6
Normalized Data
Columns
Max Mean Std Kurt
Statistics

C-161
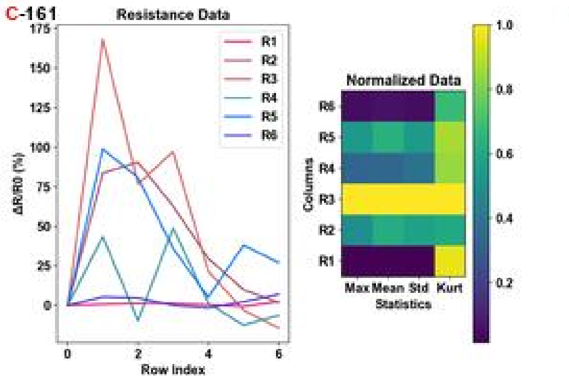

C-162
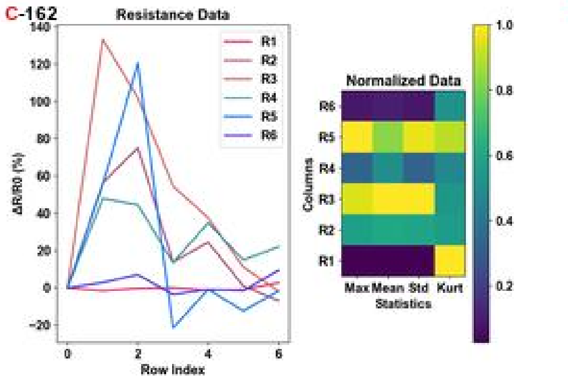

C-163
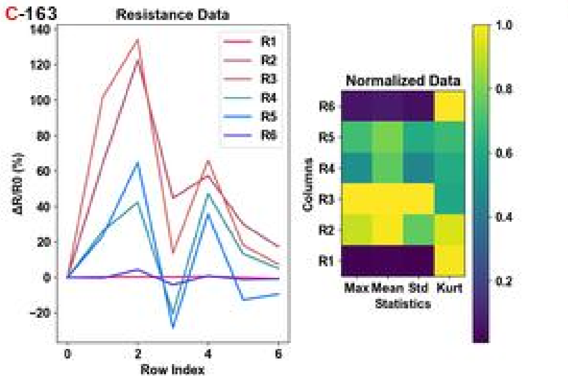

C-164
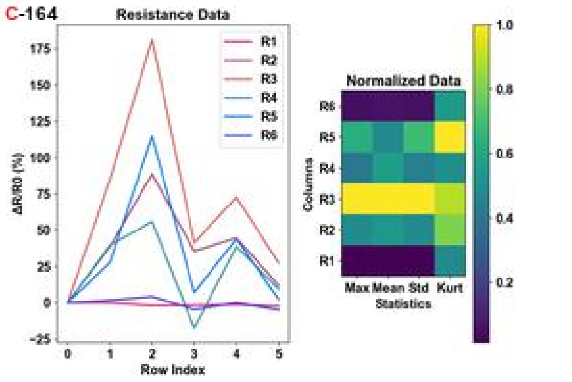

C-165
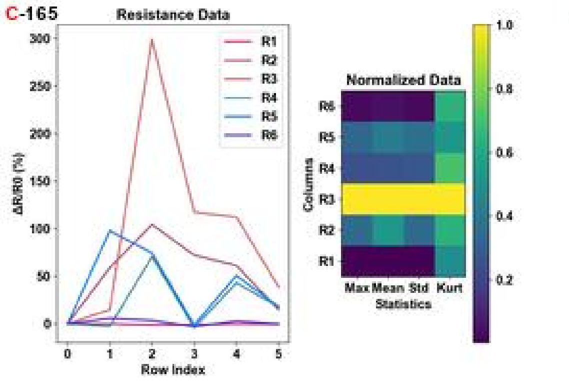

C-166
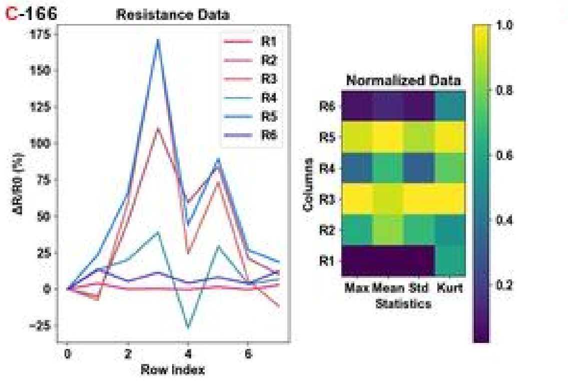

C-167
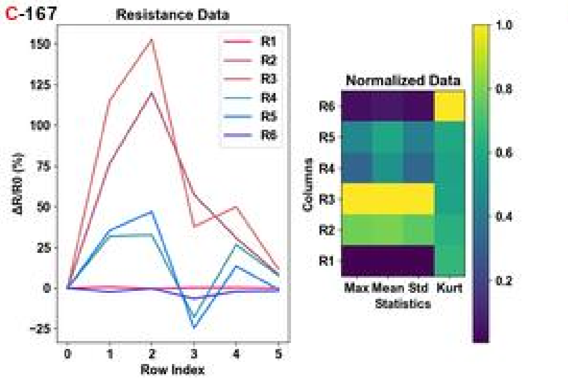

C-168
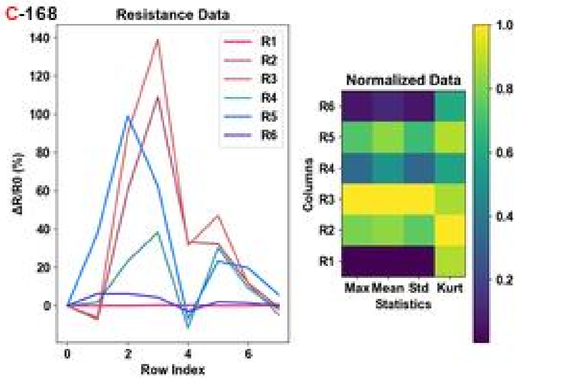

C-169
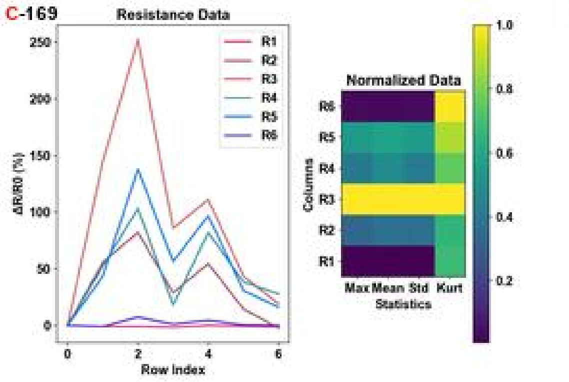

C-170
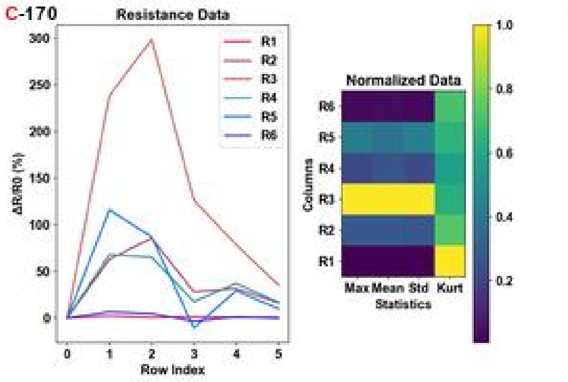

C-171
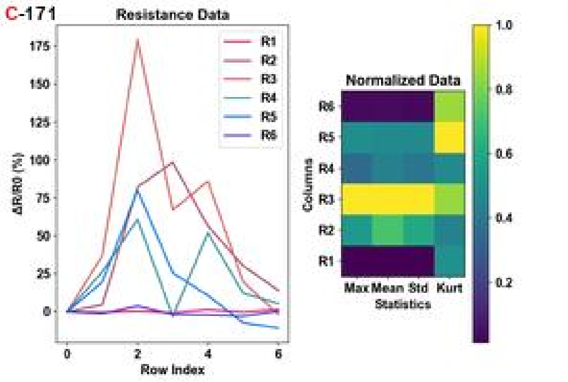

C-172
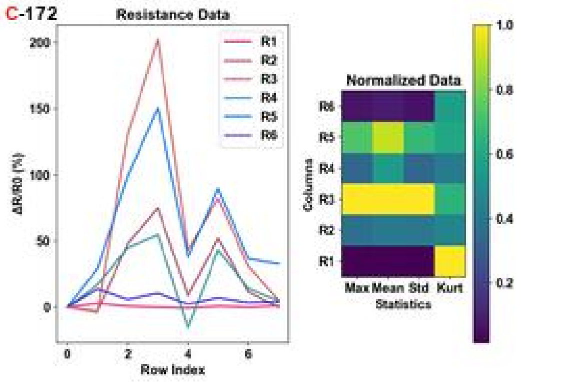

C-173
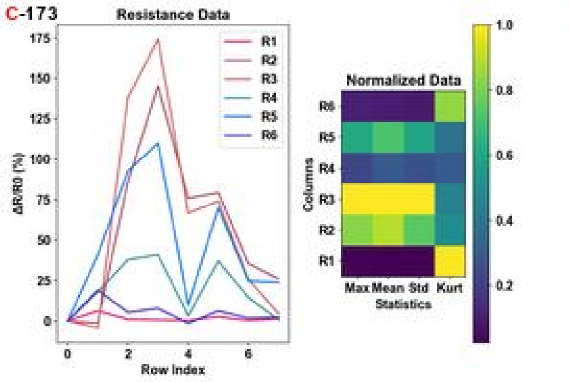

C-174
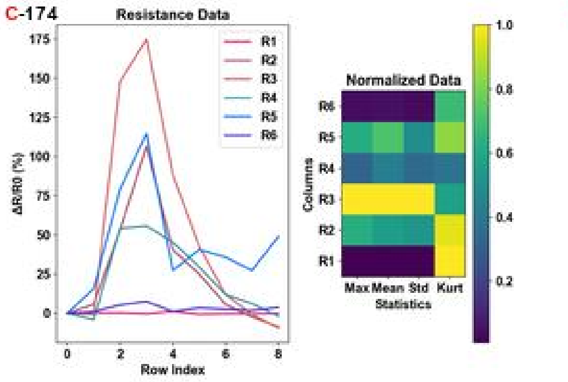

C-175
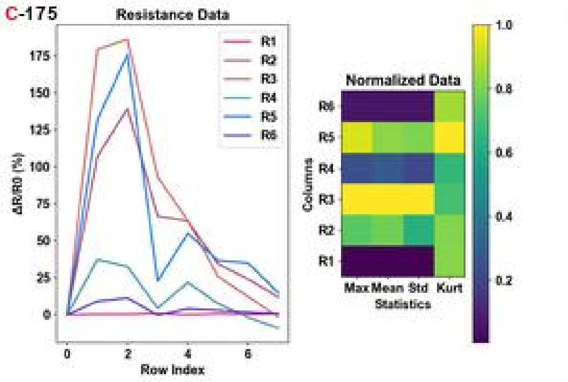

C-176
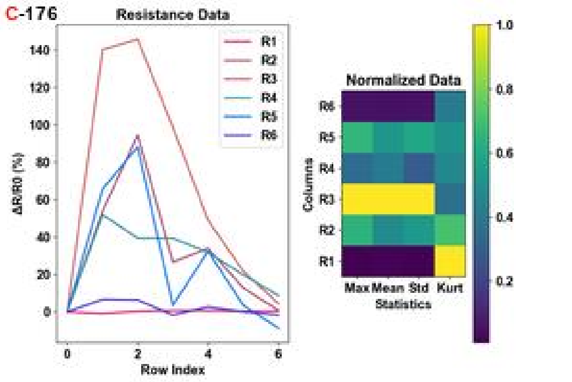

C-177
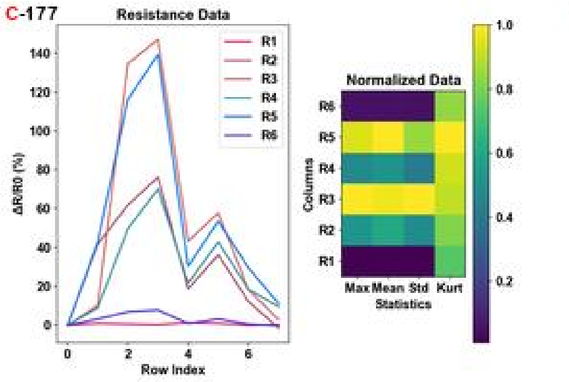

C-178
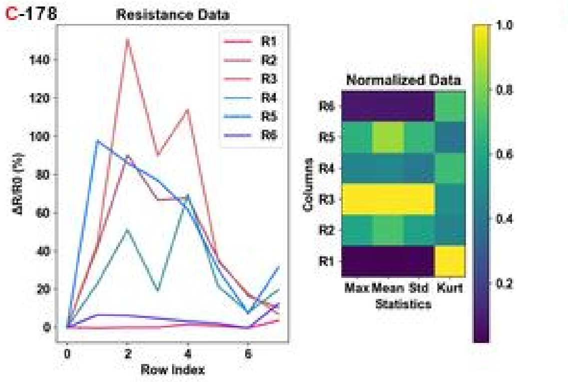

C-179
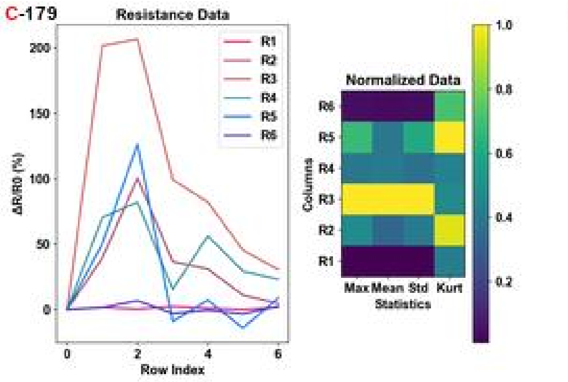

C-180
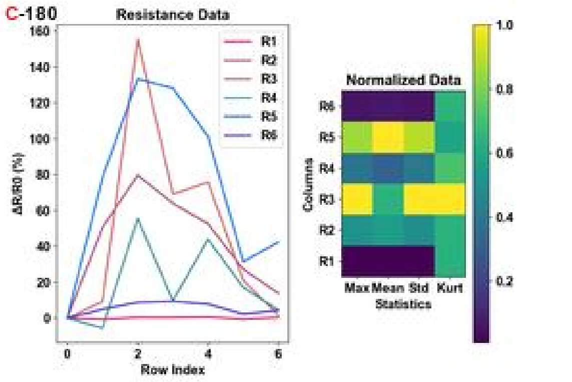

C-181
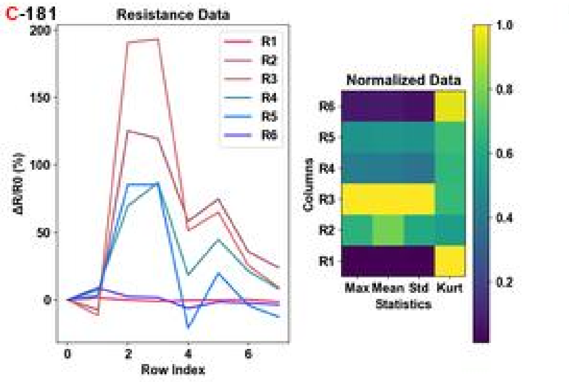

C-182
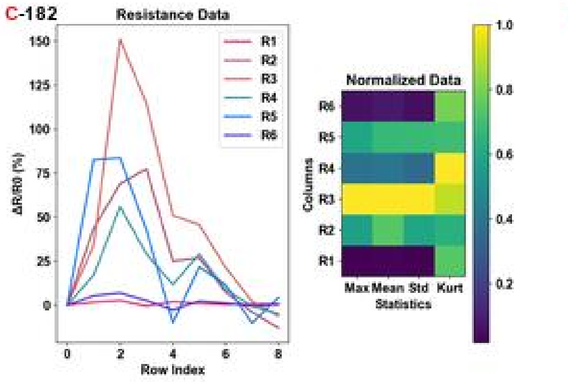

C-183
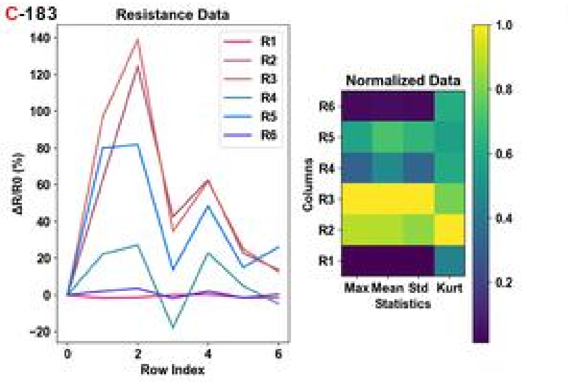

C-184
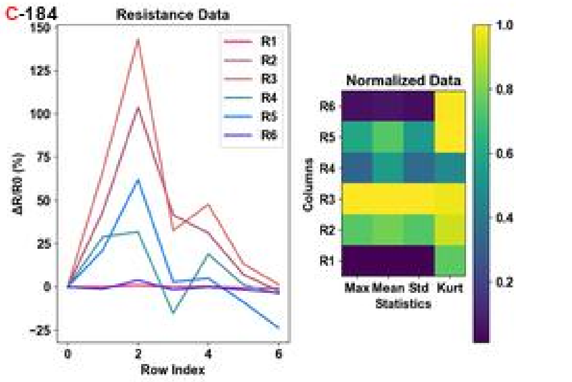

C-185
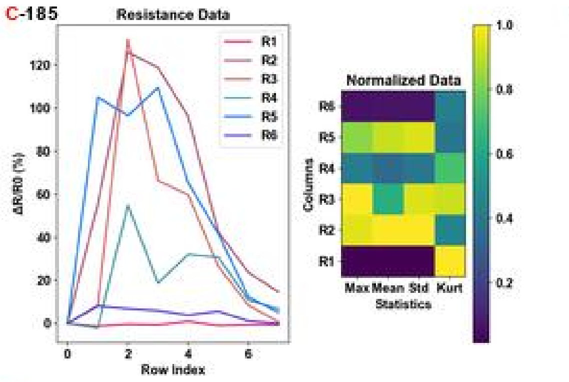

C-186
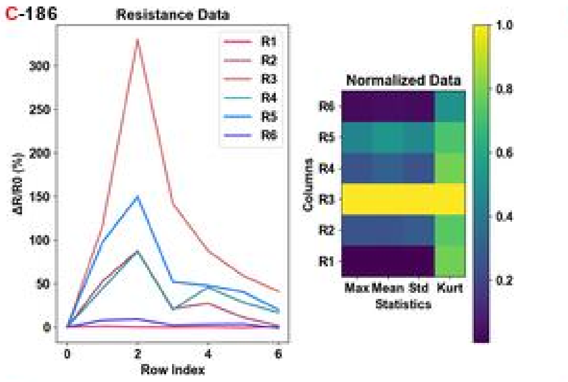

C-187
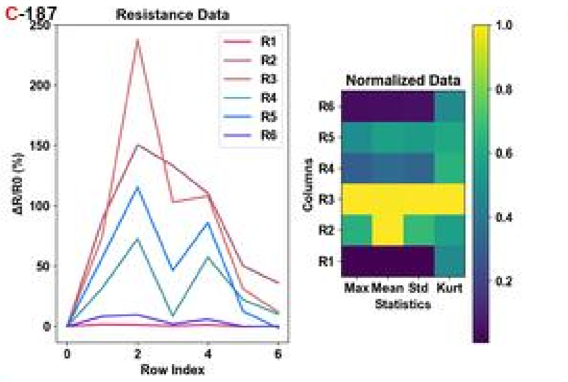

C-188
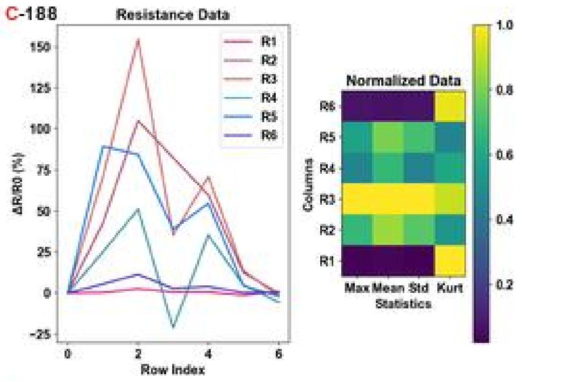

C-189
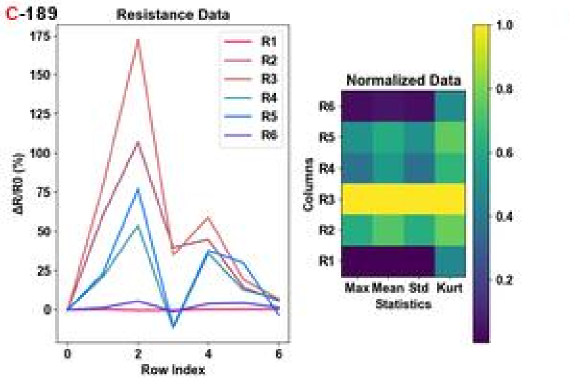

C-190
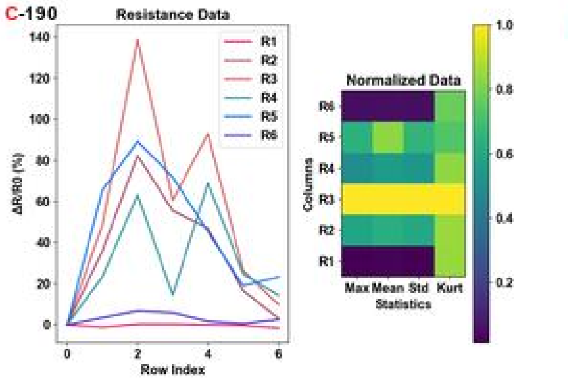

C-191
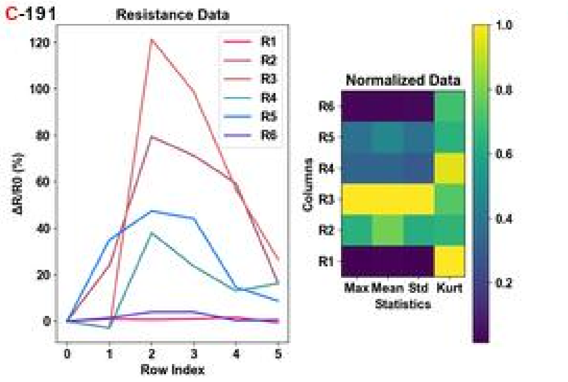

C-192
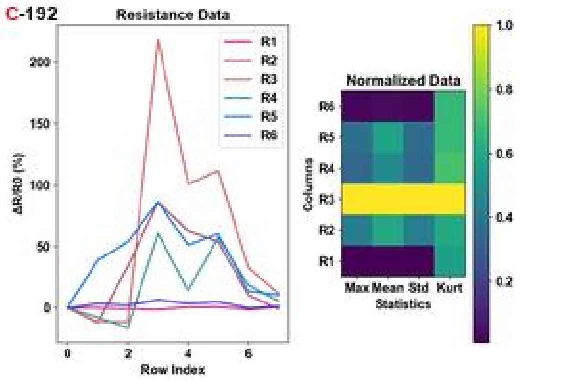

C-193
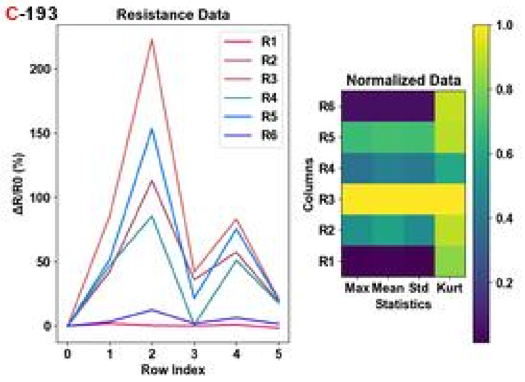

C-194
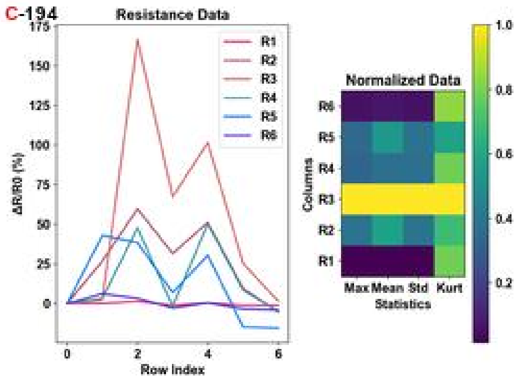

C-195
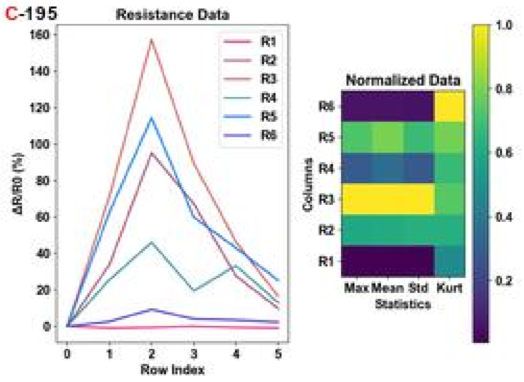

C-196
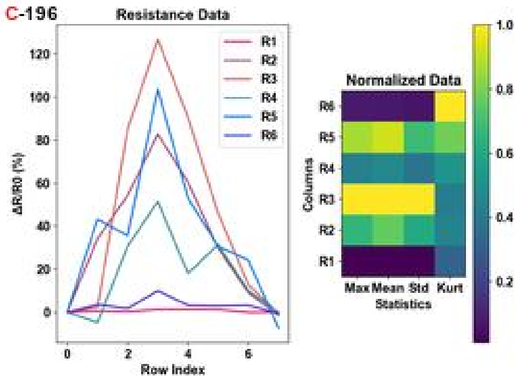

C-197
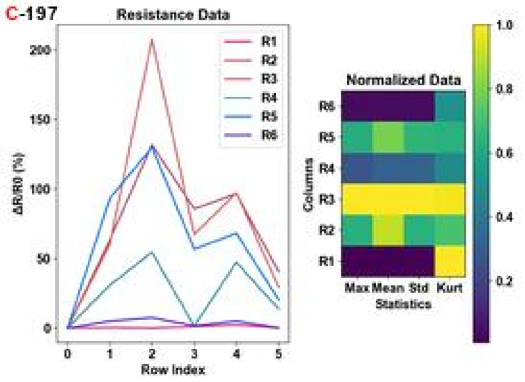

C-198
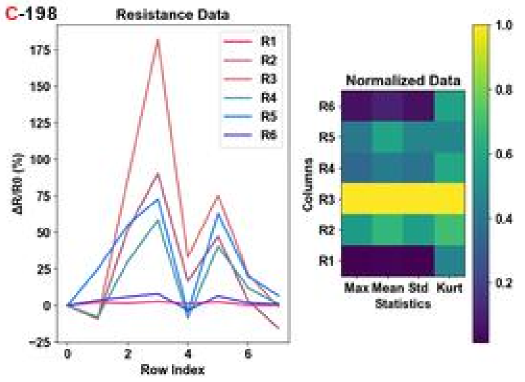

C-199
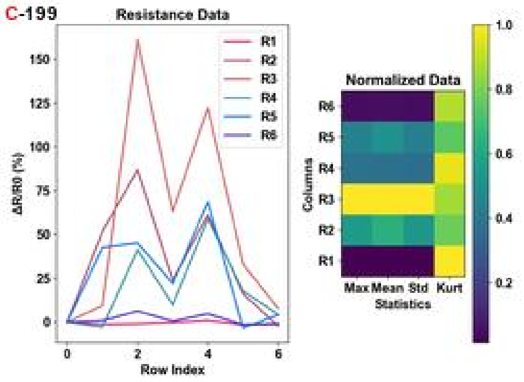

C-200
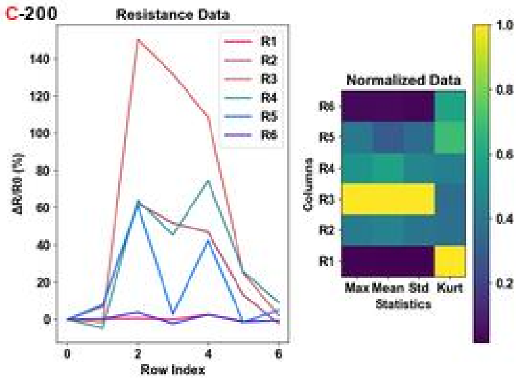

C-201
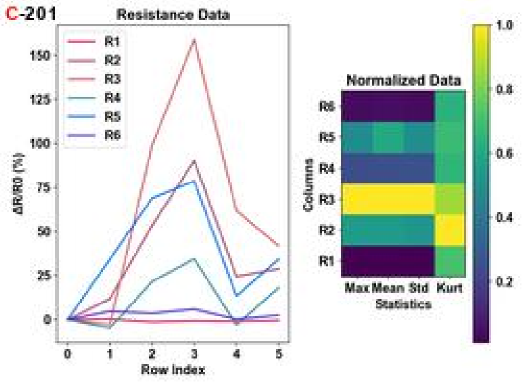

C-202
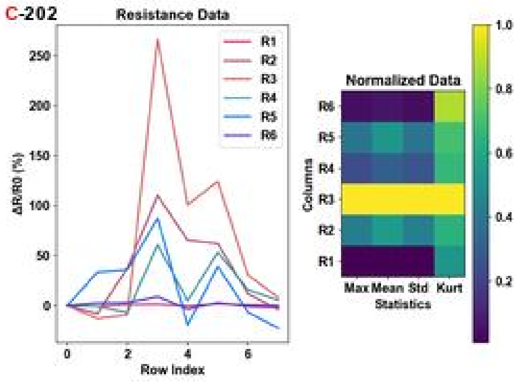

C-203
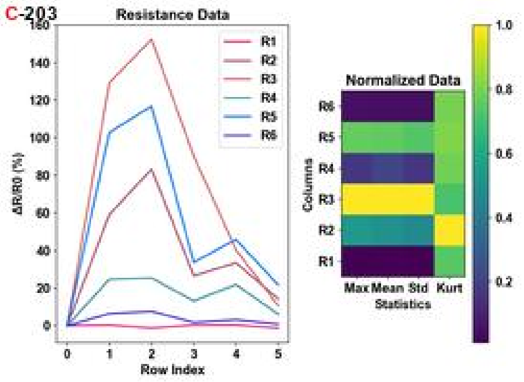

C-204
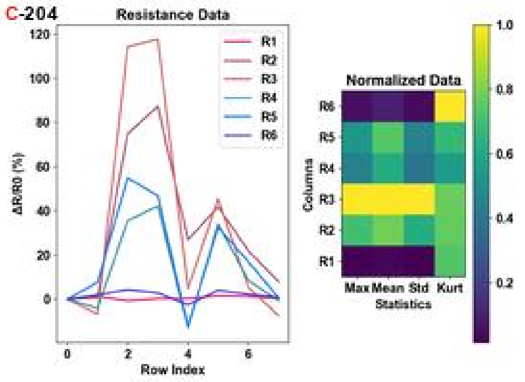

C-205
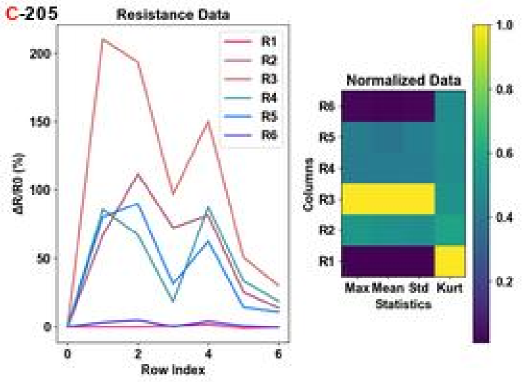

C-206
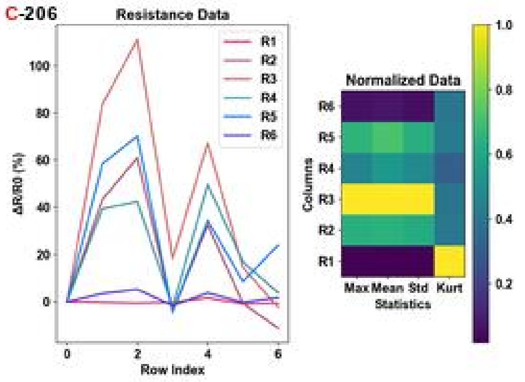

C-207
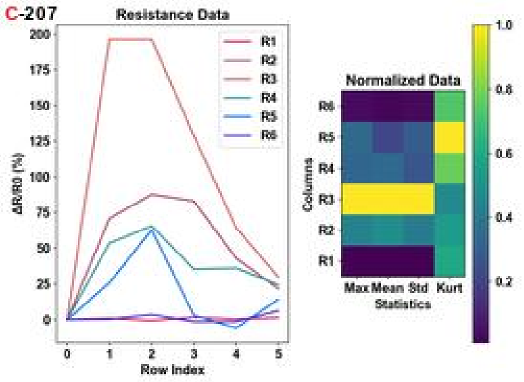

C-208
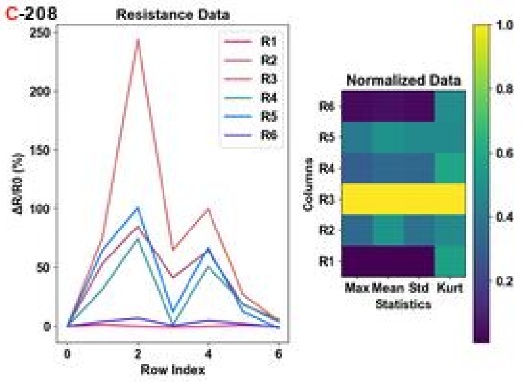

C-209
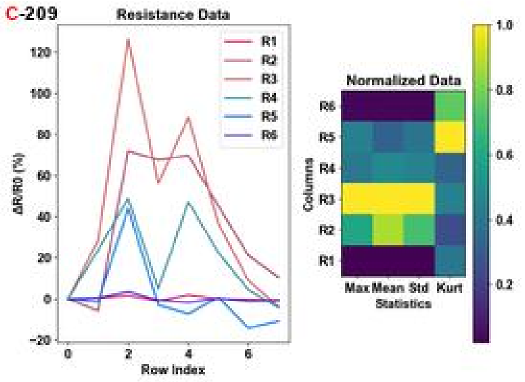

C-210
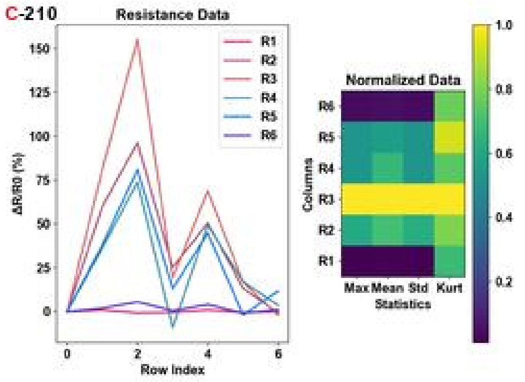

C-211
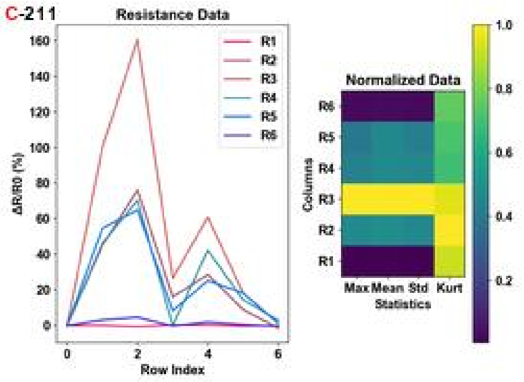

C-212
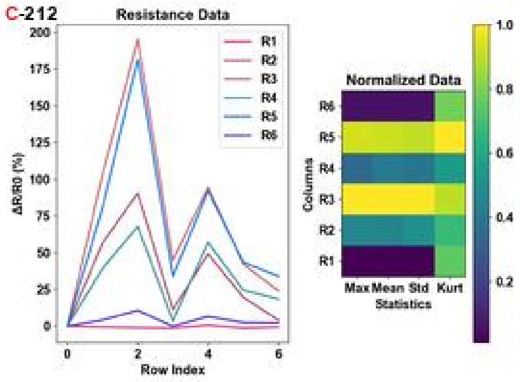

C-213
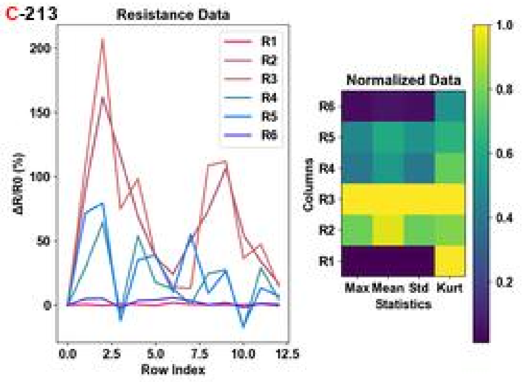

C-214
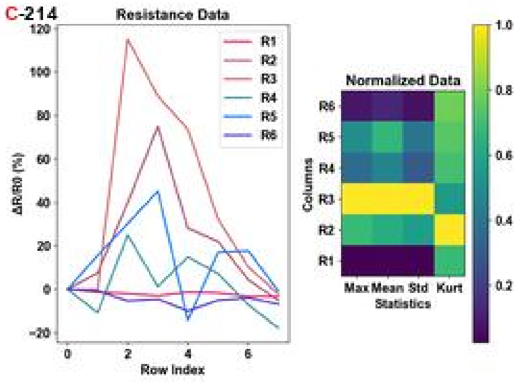

C-215
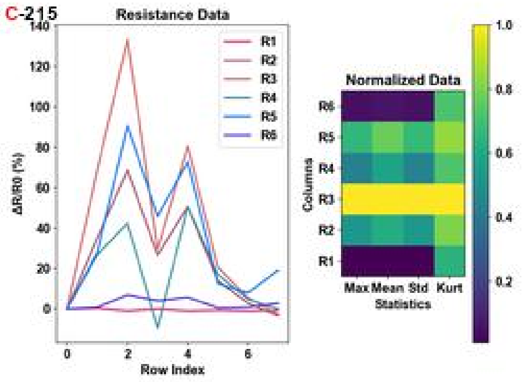

C-216
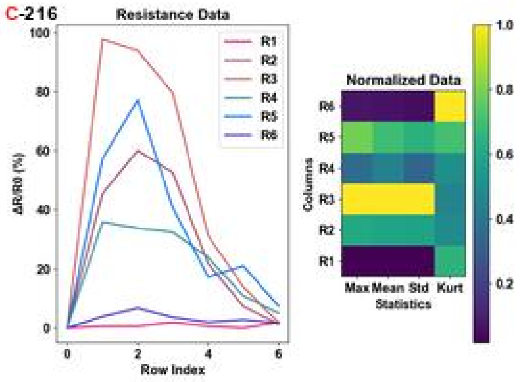

C-217
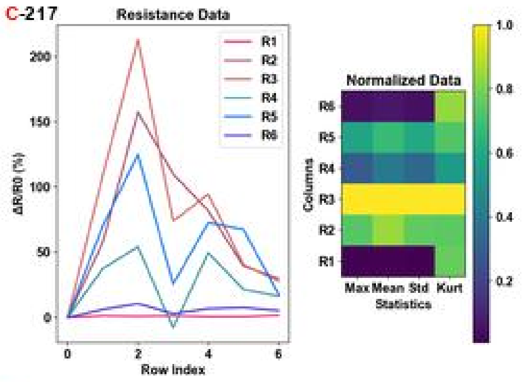

C-218
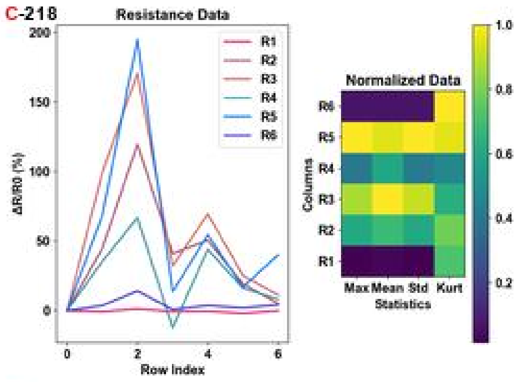

C-219
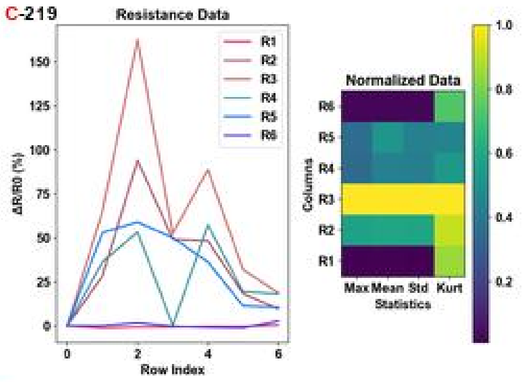

C-220
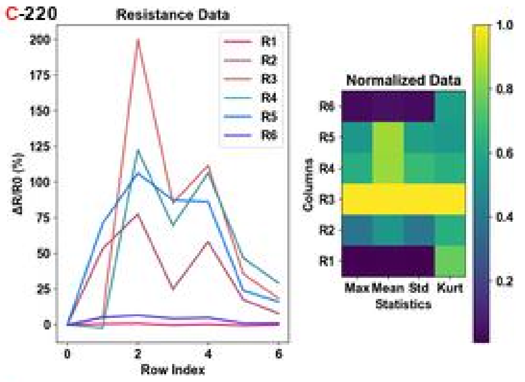

C-221
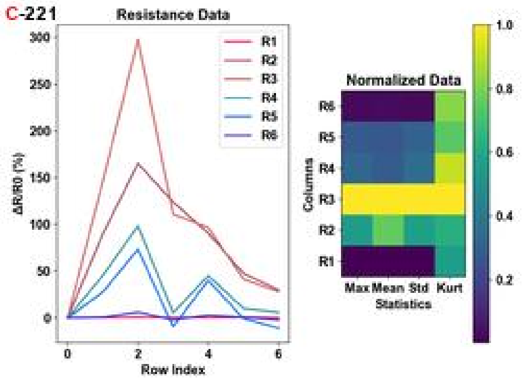

C-222
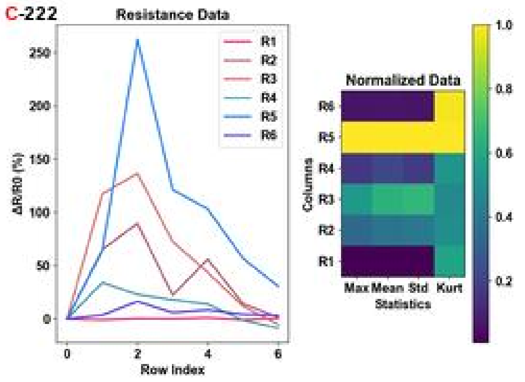

C-223
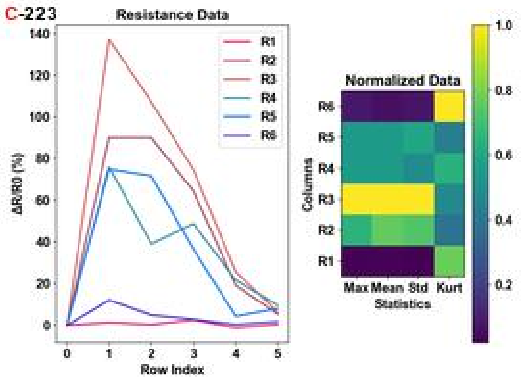

C-224
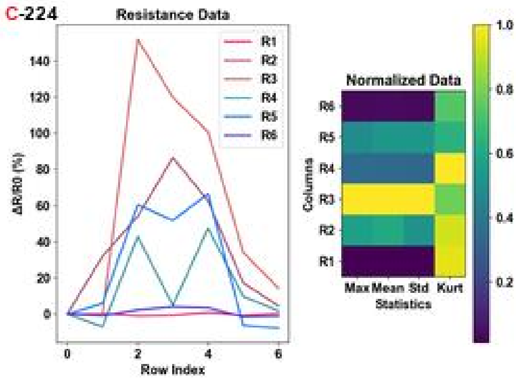

C-225

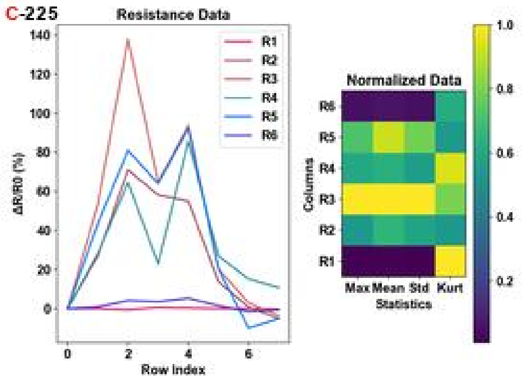

C-226

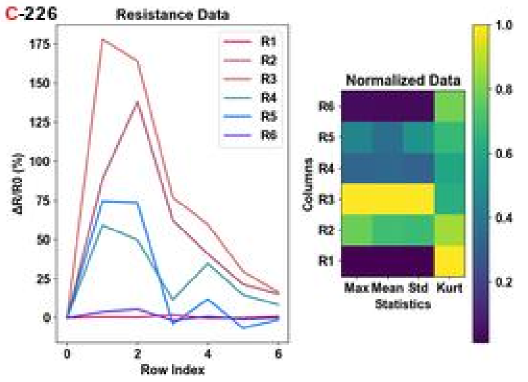

C-227

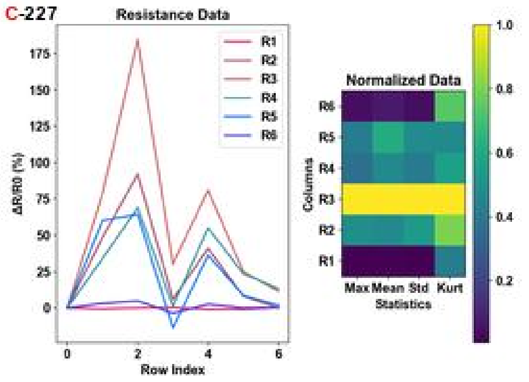

C-228

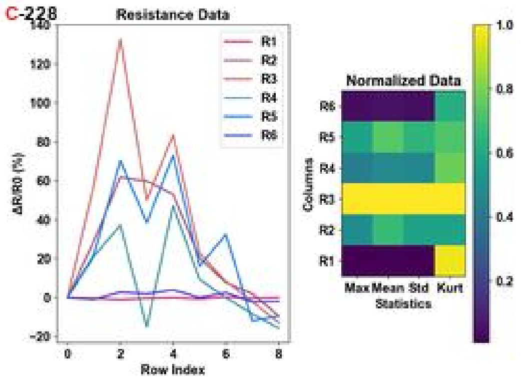

C-229

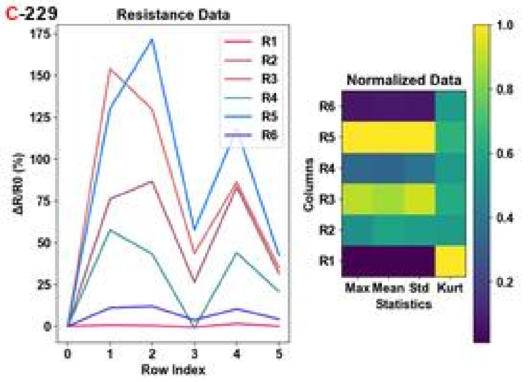

C-230

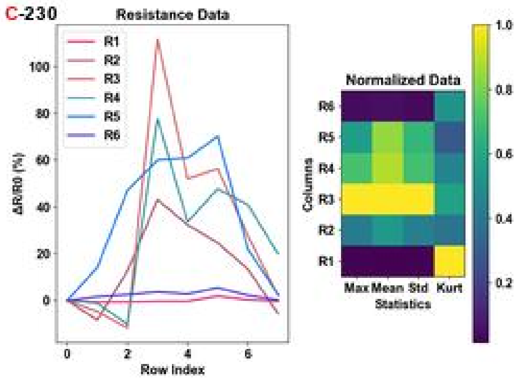

C-231

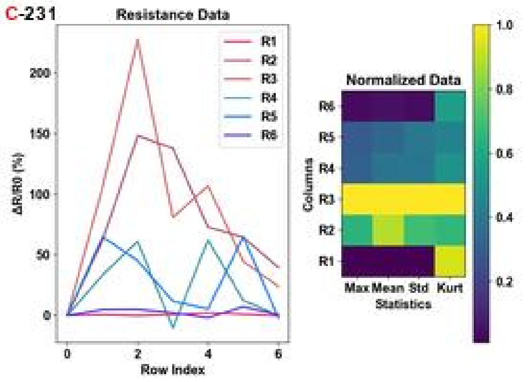

C-232

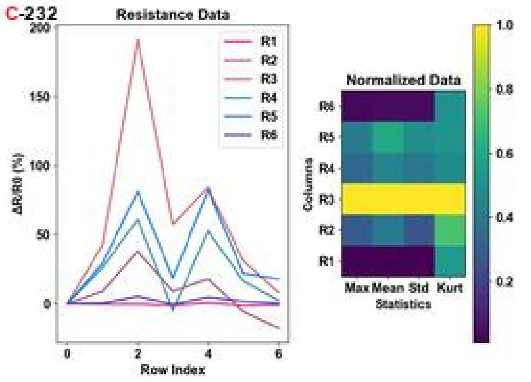

C-233

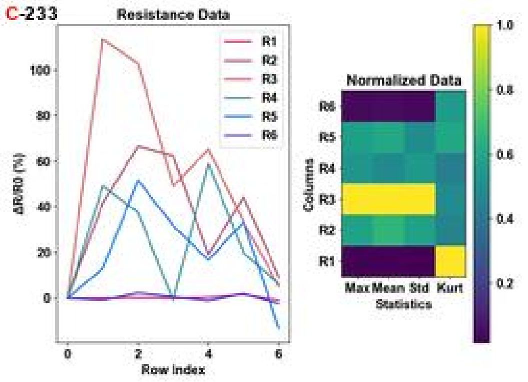

C-234

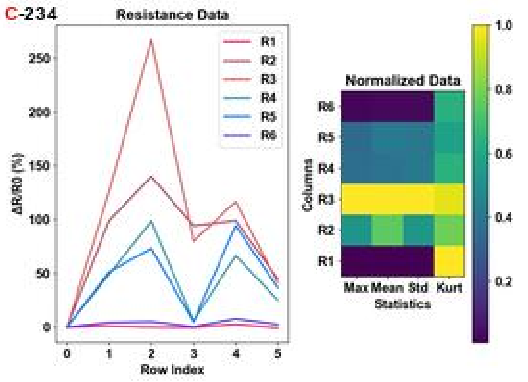

C-235

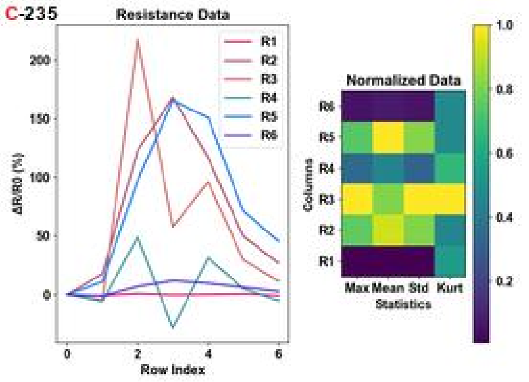

C-236

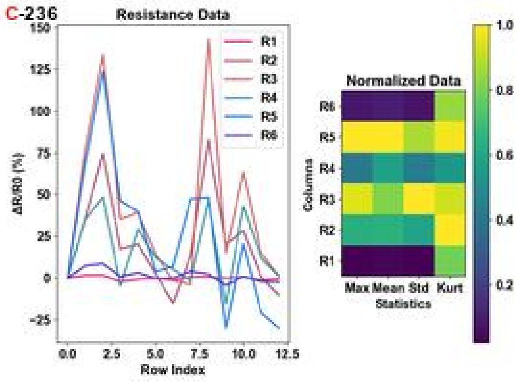

C-237

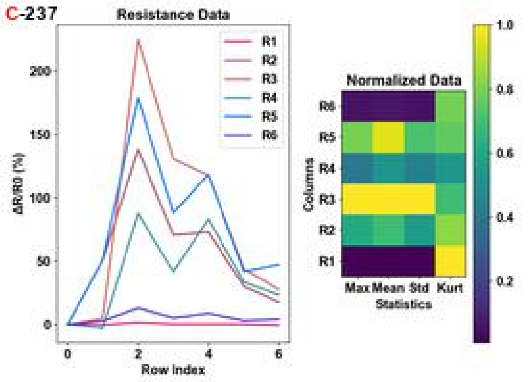

C-238

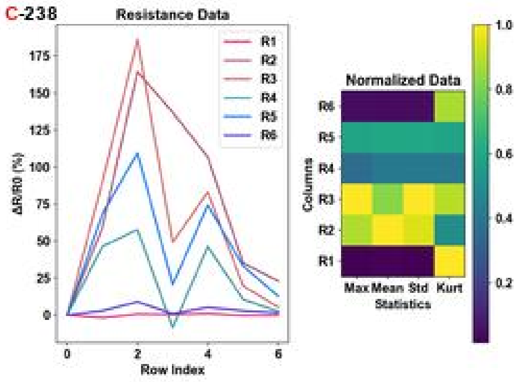

C-239

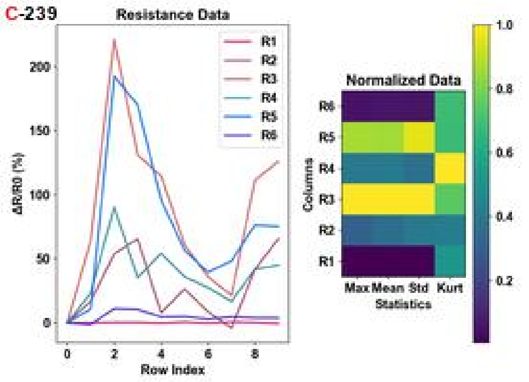

C-240

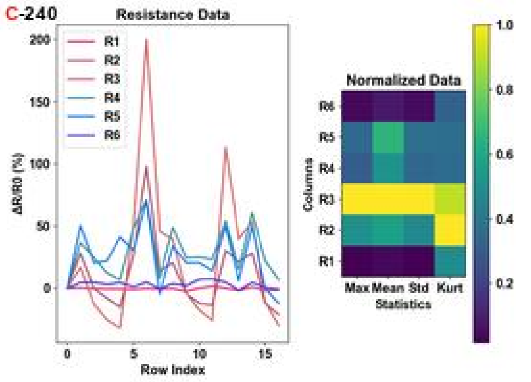

Supplement: Supplementary file 6 — Supporting Information [file ADMA-37-2509631-s003.pdf]
