# Supplementary material for: Fibrous Pressure Sensor with Unique Resistance Increase under Partial Compression: Coaxial Wet‐Spun TiO2/Graphene/Thermoplastic Polyurethane Multi‐Wall Multifunctional Fiber
Source: Adv Mater. 2025 Jul 16;37(40):2509631. doi: 10.1002/adma.202509631 (PMC12510290; doi:10.1002/adma.202509631)

ADVANCED MATERIALS

Supporting Information

for *Adv. Mater.*, DOI 10.1002/adma.202509631

Fibrous Pressure Sensor with Unique Resistance Increase under Partial Compression: Coaxial Wet-Spun TiO_2/Graphene/Thermoplastic Polyurethane Multi-Wall Multifunctional Fiber

*Ziwei Chen, Dandan Xie, Kanae Kojima, Chunxia Gao, Jian Shi, Jian Xing, Hideaki Morikawa and Chunhong Zhu**

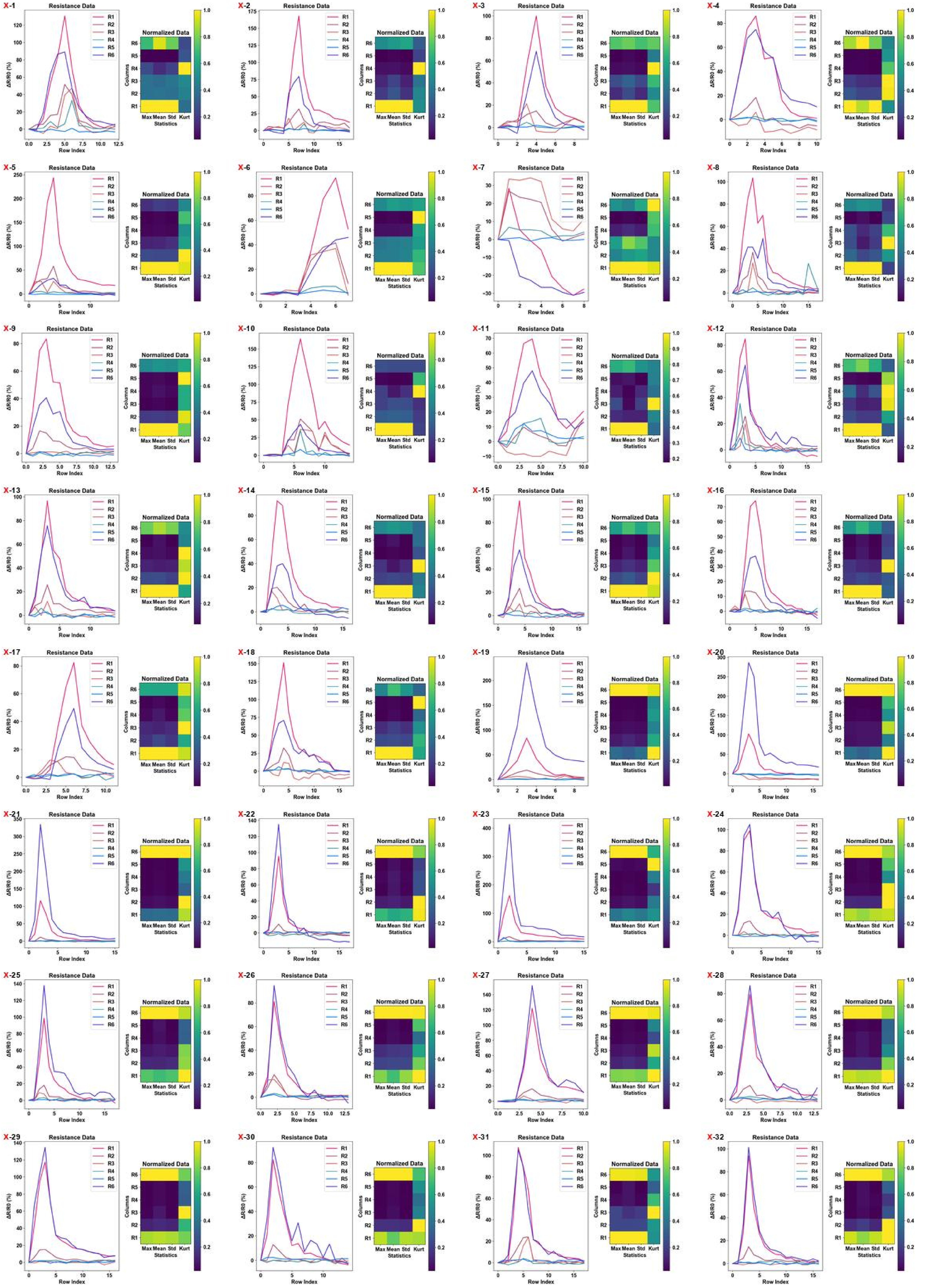
X-1
X-2
X-3
X-4
X-5
X-6
X-7
X-8
X-9
X-10
X-11
X-12
X-13
X-14
X-15
X-16
X-17
X-18
X-19
X-20
X-21
X-22
X-23
X-24
X-25
X-26
X-27
X-28
X-29
X-30
X-31
X-32
Resistance Data
ΔR/R0 (%)
Row Index
R1
R2
R3
R4
R5
R6
Normalized Data
Columns
Statistics
Max
Mean
Std
Kurt

Pressure-sensitive data of type X

X-33
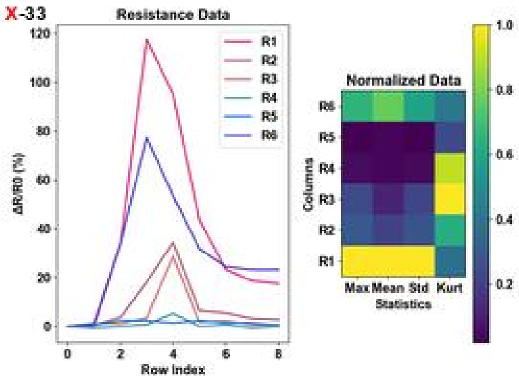

X-34
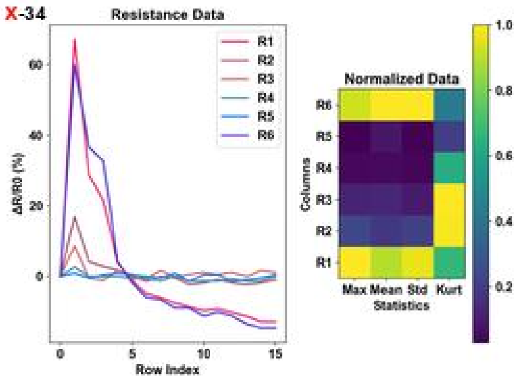

X-35
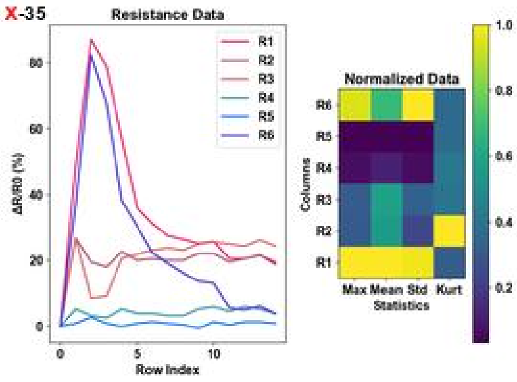

X-36
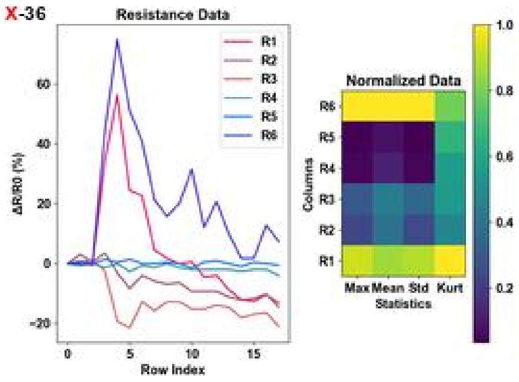

X-37
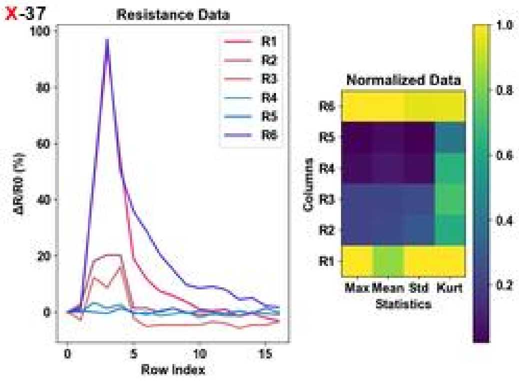

X-38
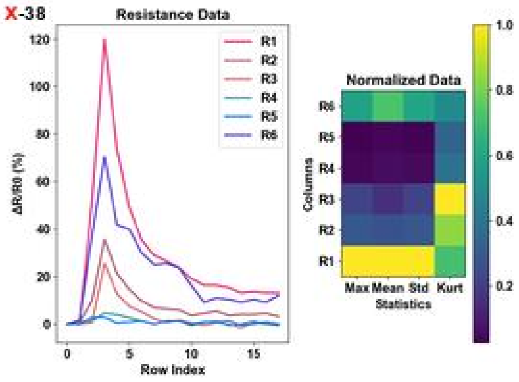

X-39
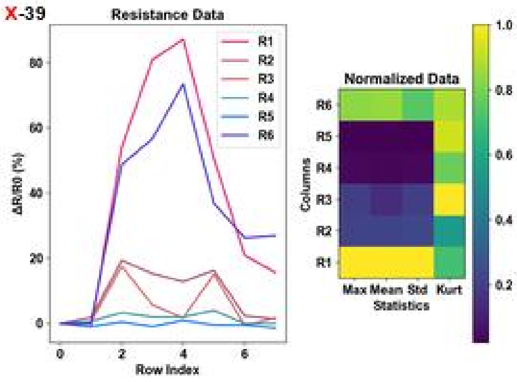

X-40
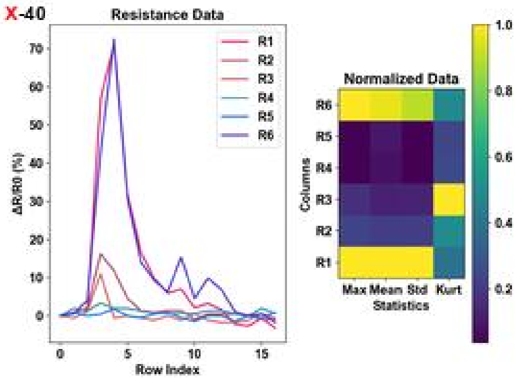

X-41
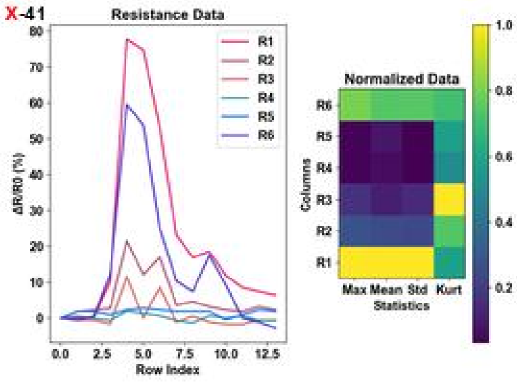

X-42
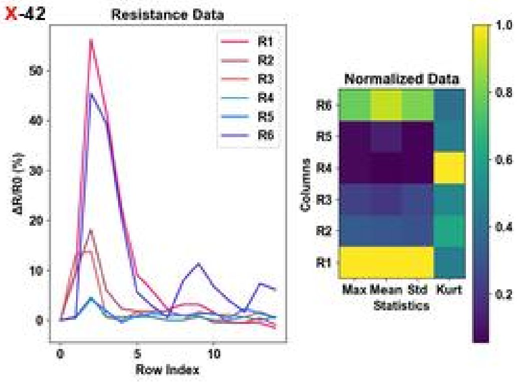

X-43
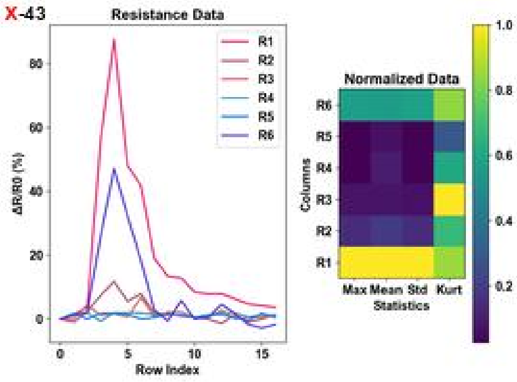

X-44
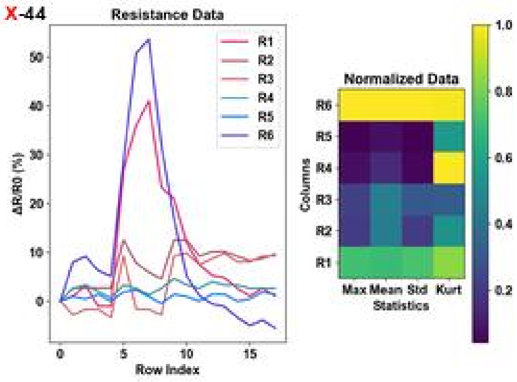

X-45
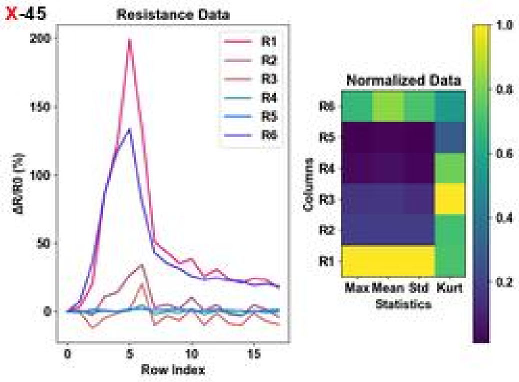

X-46
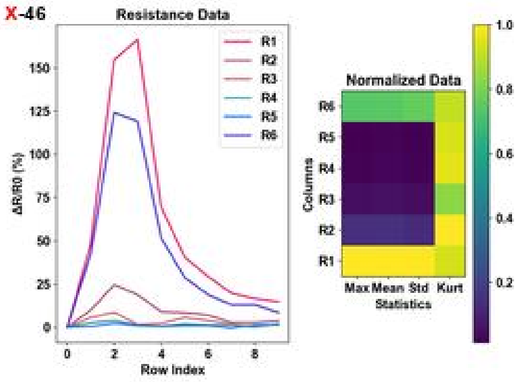

X-47
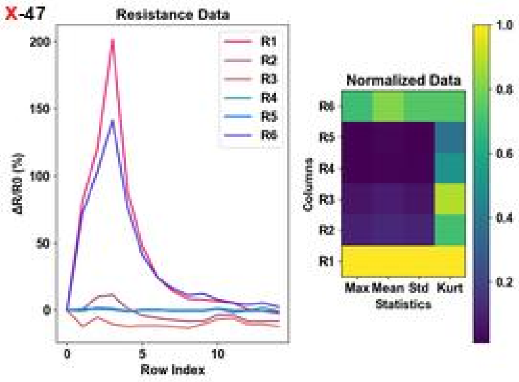

X-48
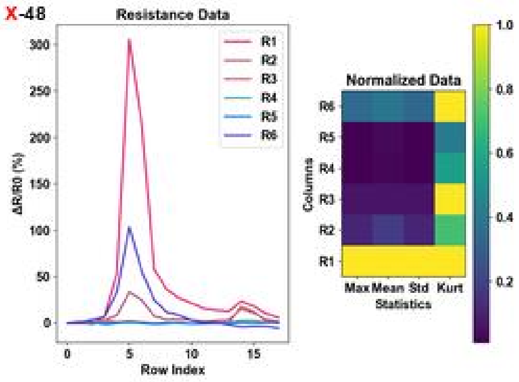

X-49
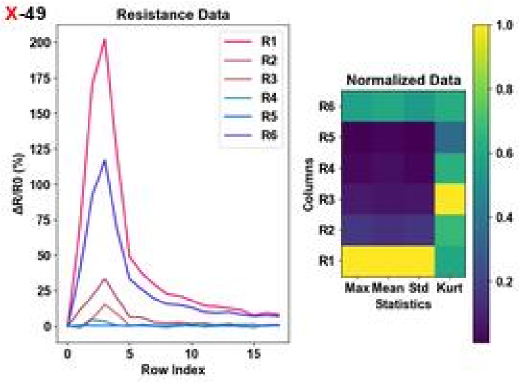

X-50
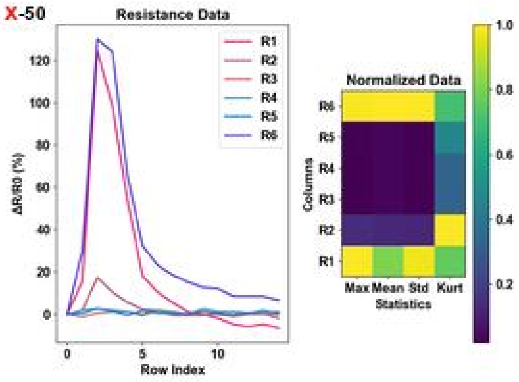

X-51
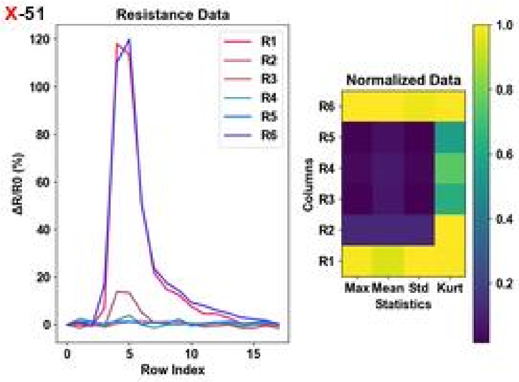

X-52
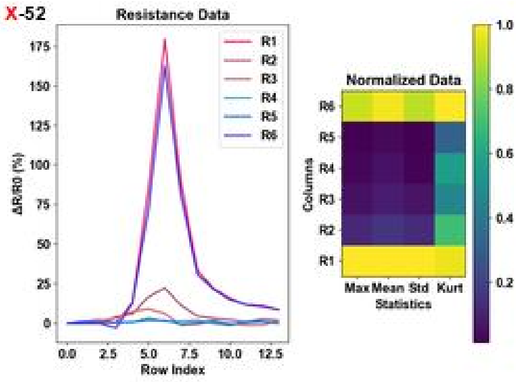

X-53
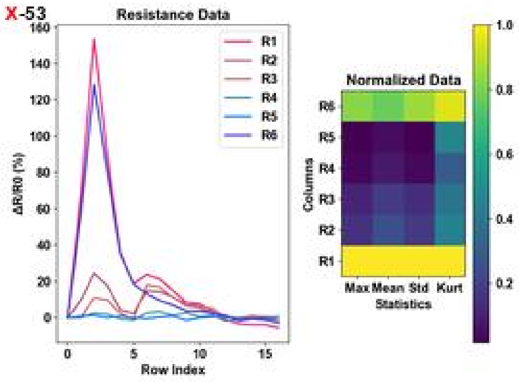

X-54
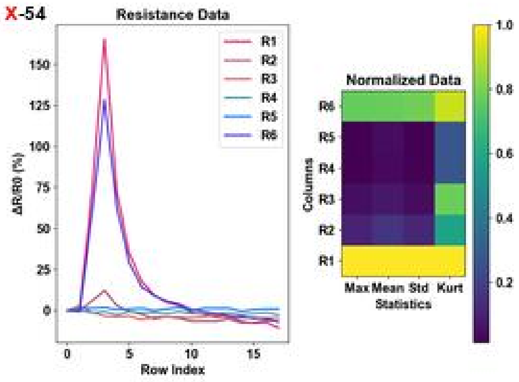

X-55
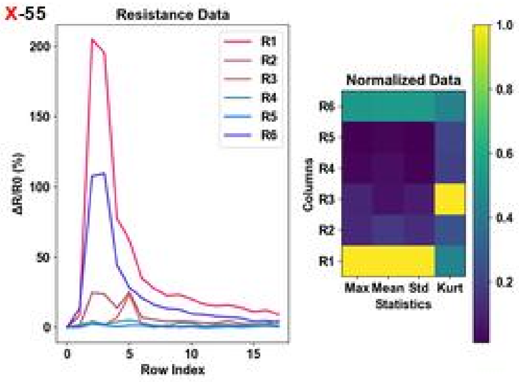

X-56
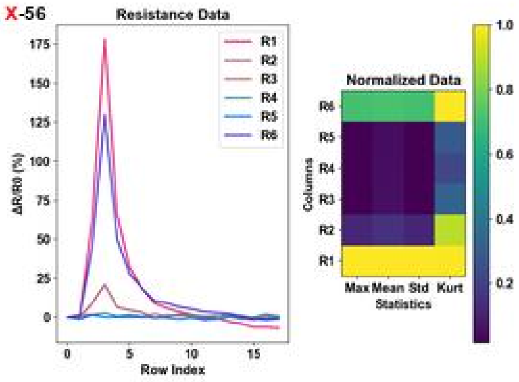

X-57
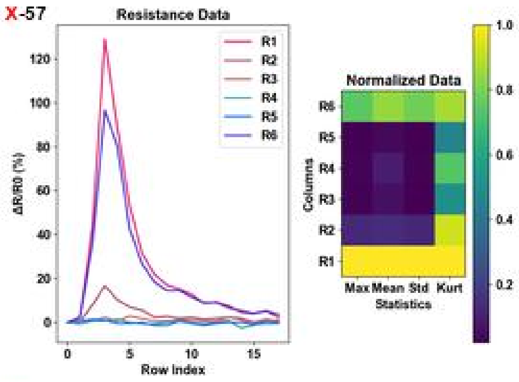

X-58
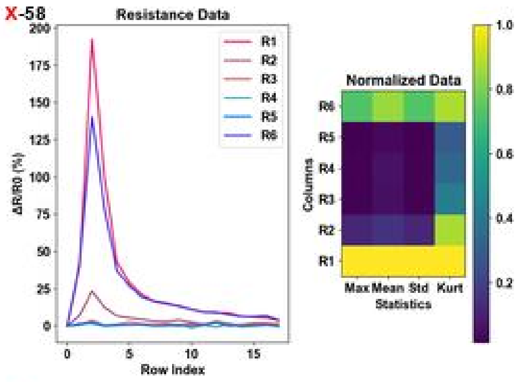

X-59
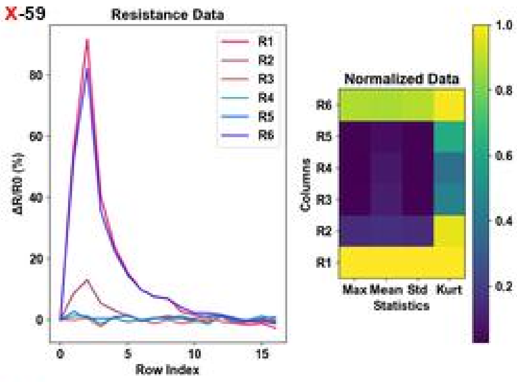

X-60
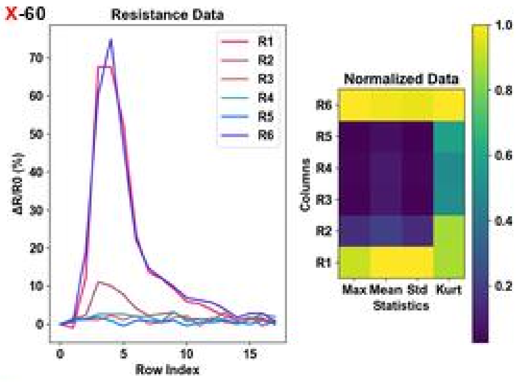

X-61
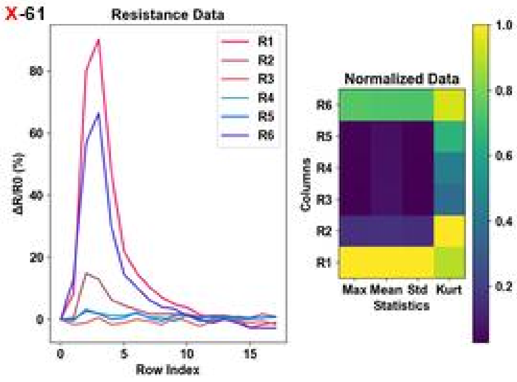

X-62
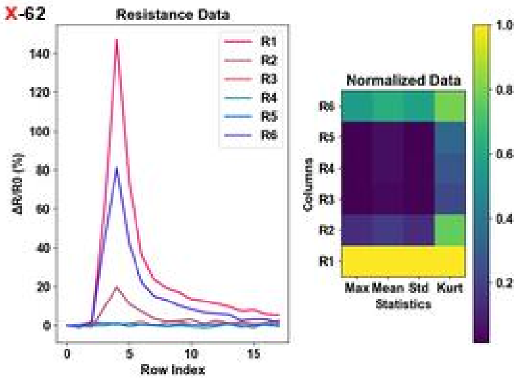

X-63
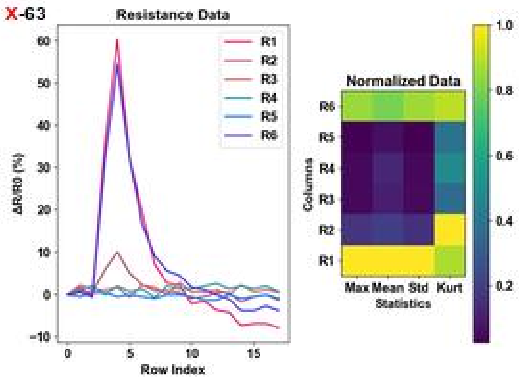

X-64
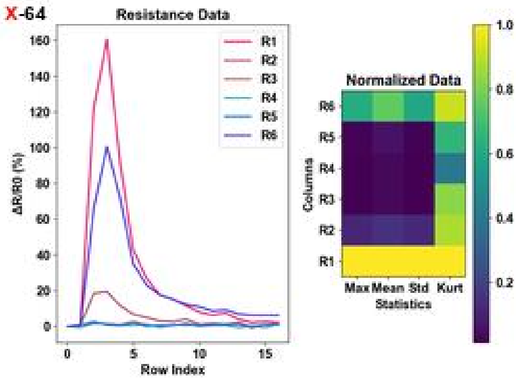

Pressure-sensitive data of type X

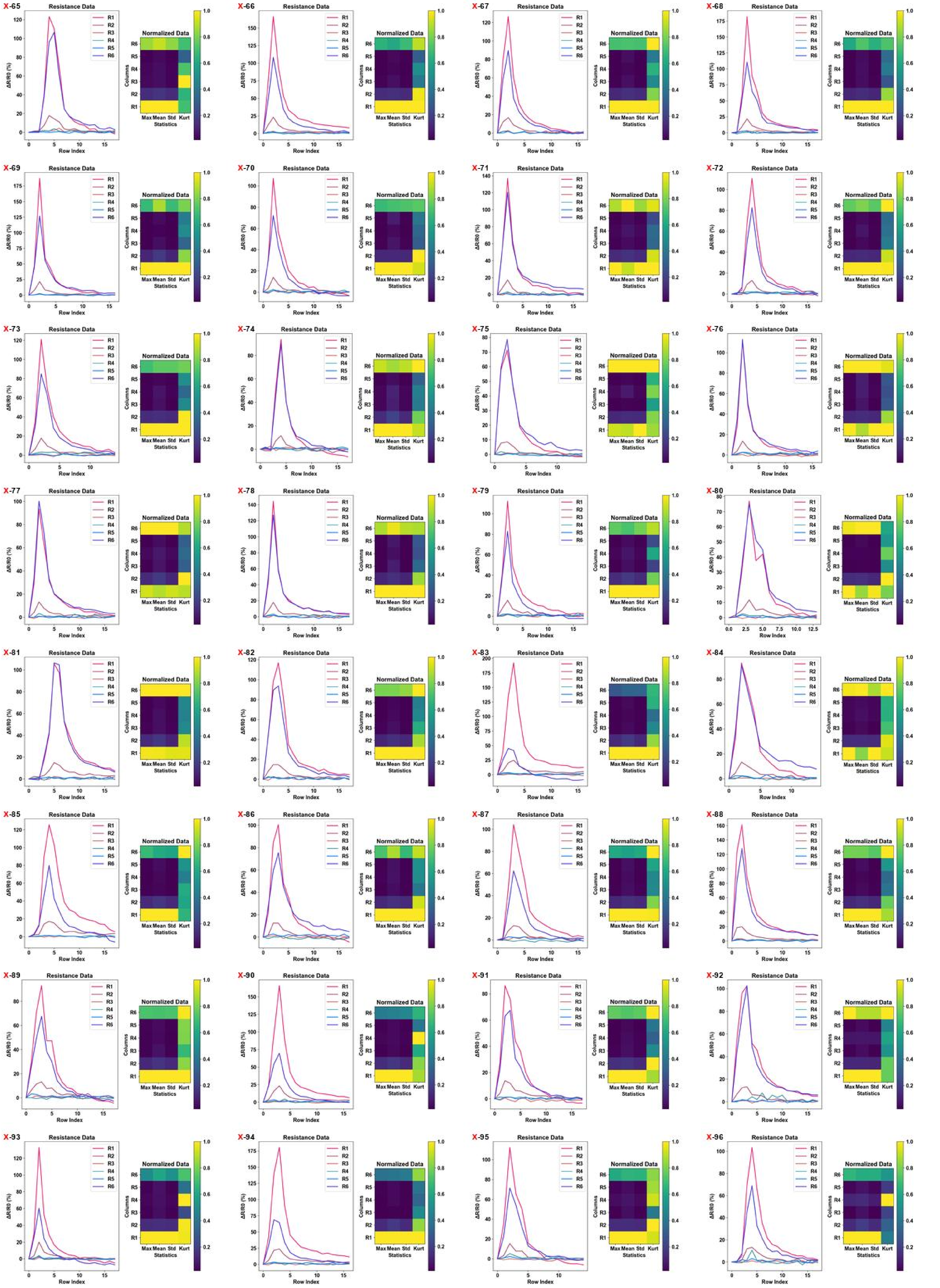

Pressure-sensitive data of type X

X-97

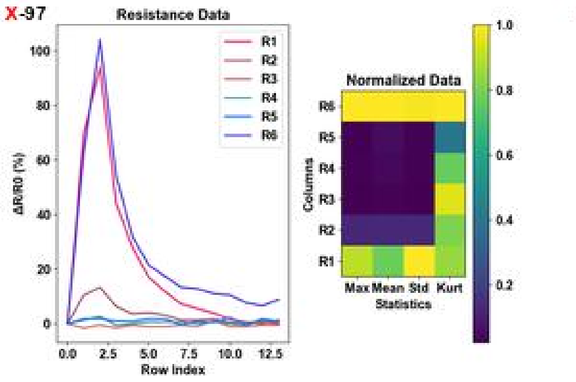

X-98

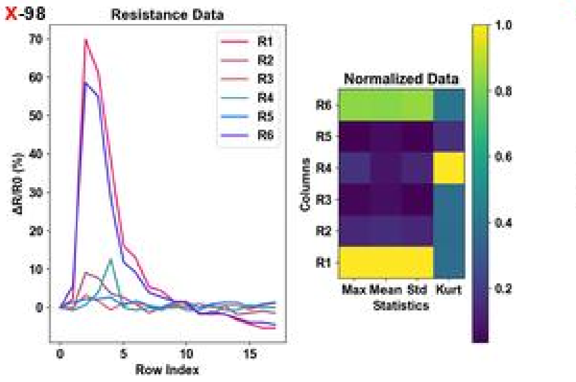

X-99

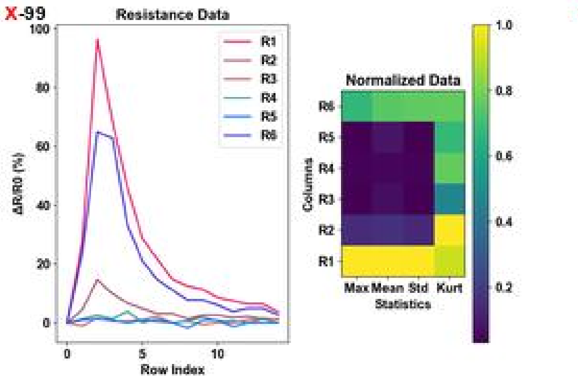

X-100

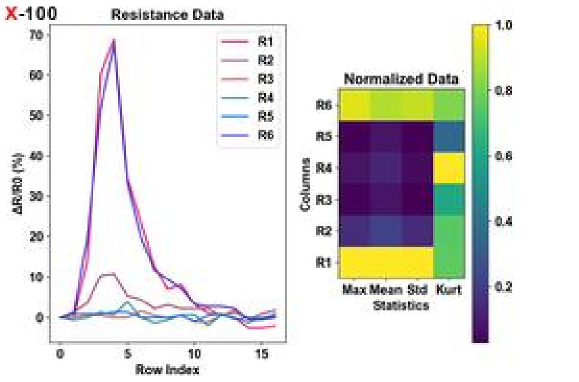

X-101

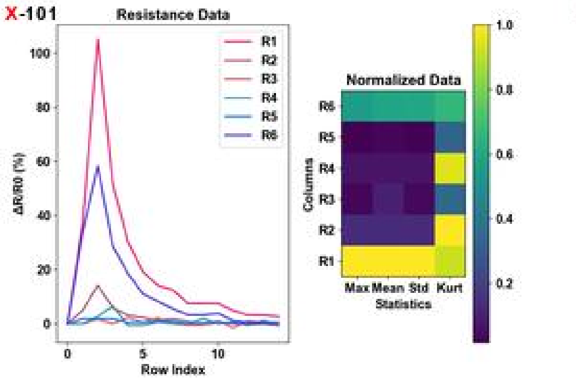

X-102

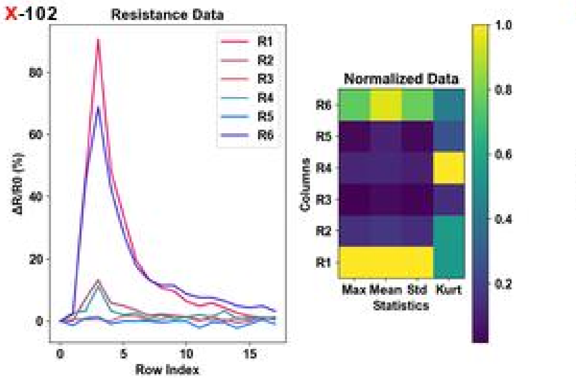

X-103

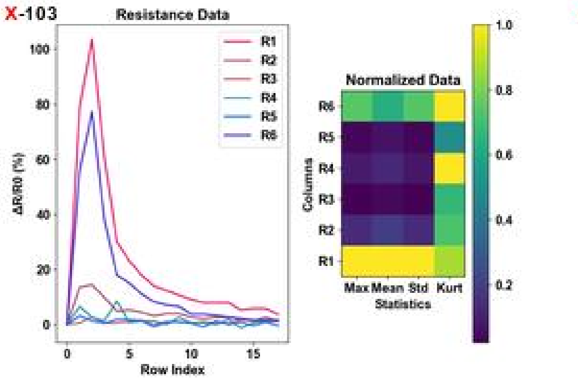

X-104

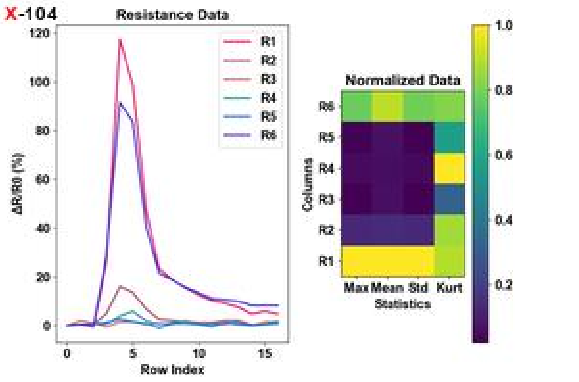

X-105

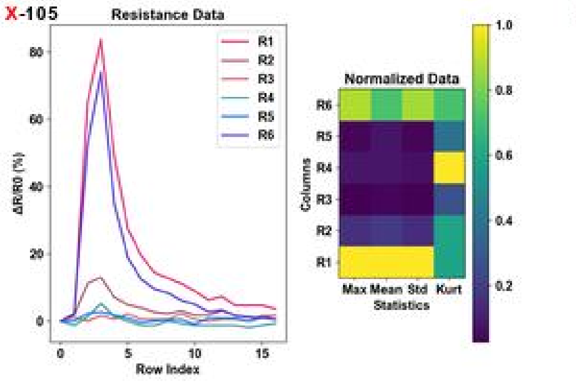

X-106

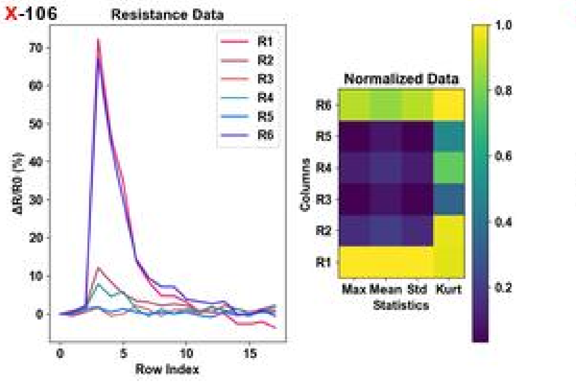

X-107

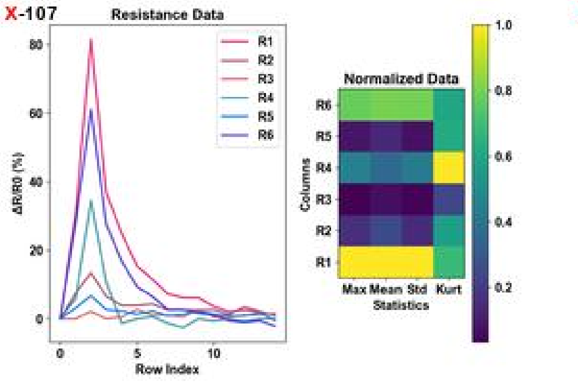

X-108

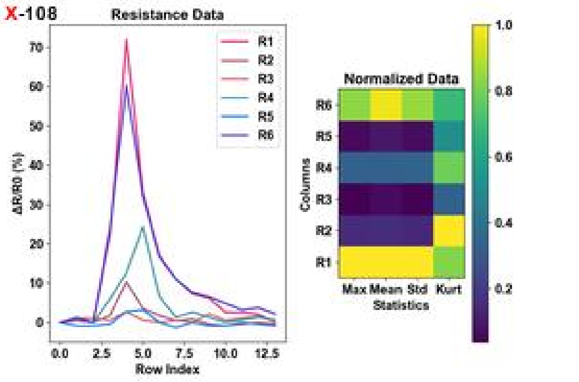

X-109

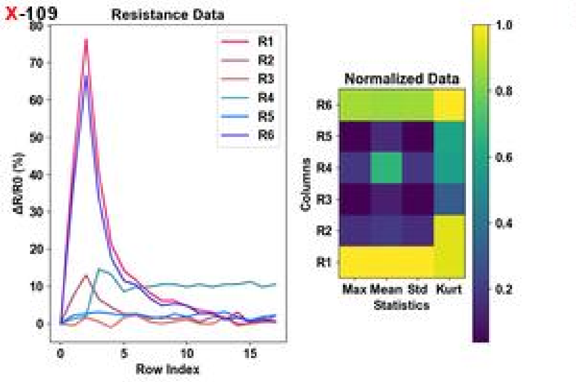

X-110

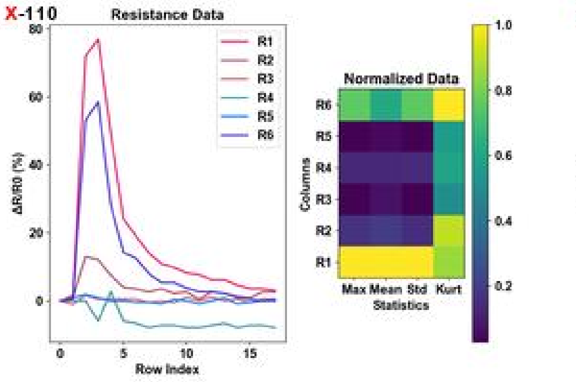

X-111

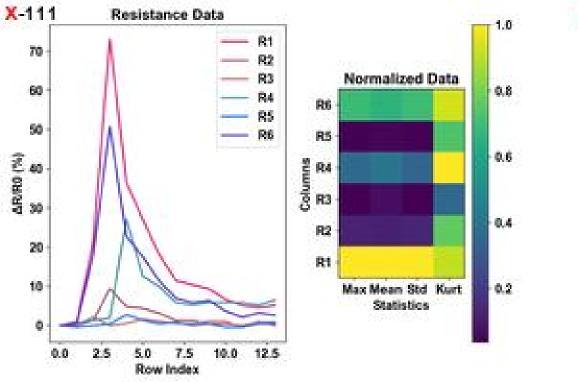

X-112

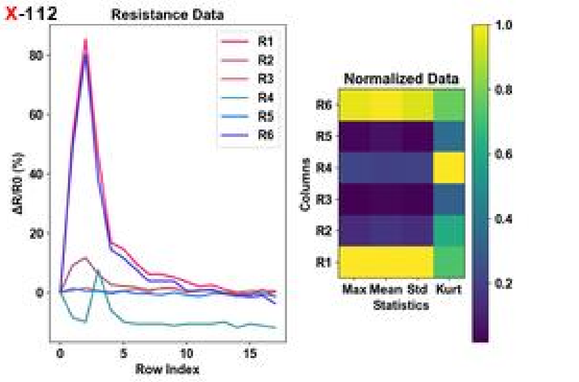

X-113

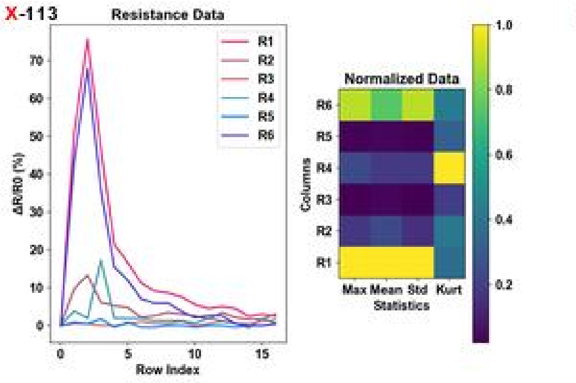

X-114

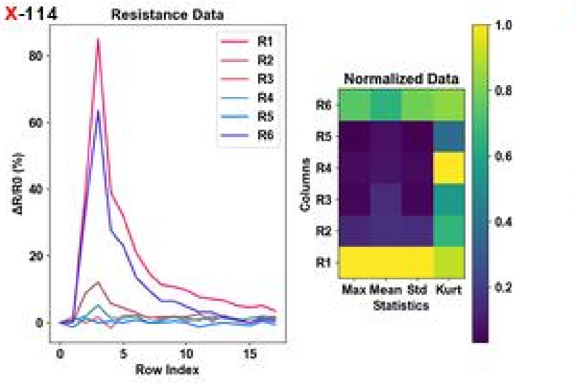

X-115

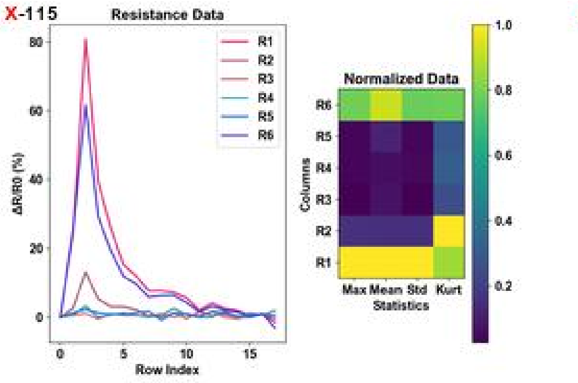

X-116

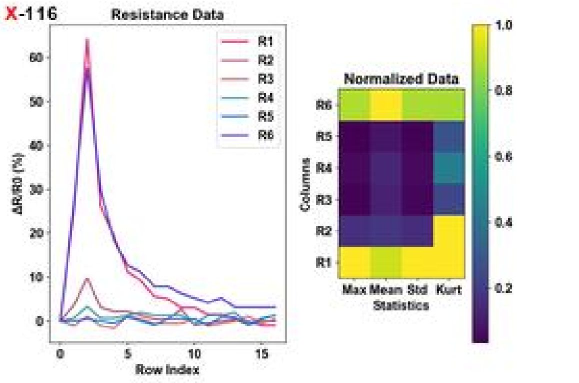

X-117

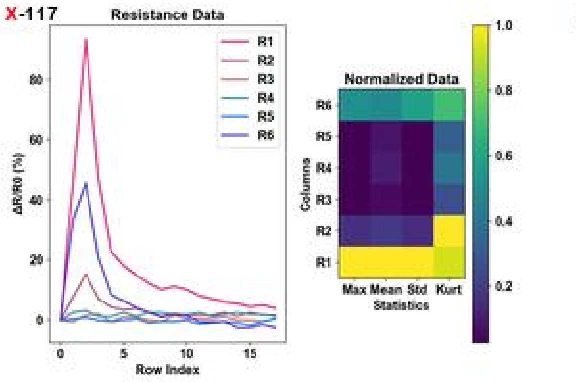

X-118

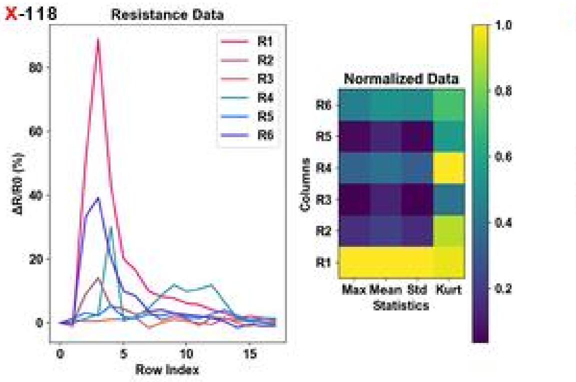

X-119

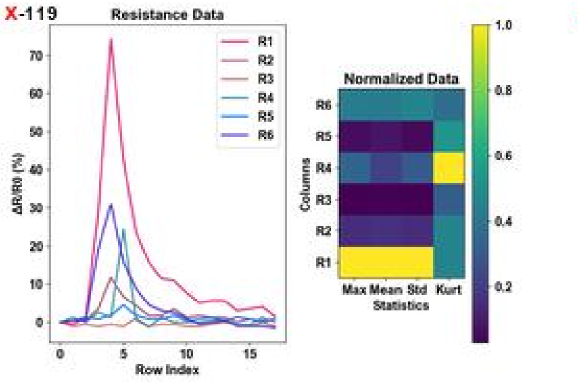

X-120

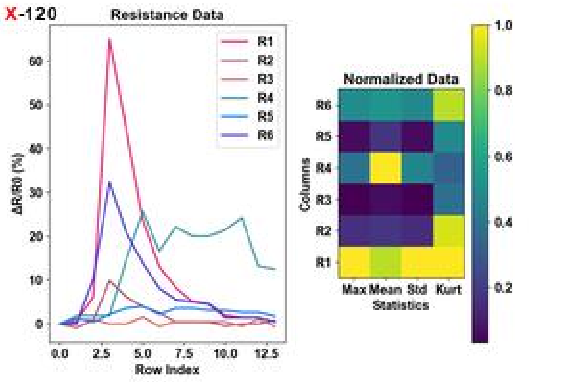

X-121

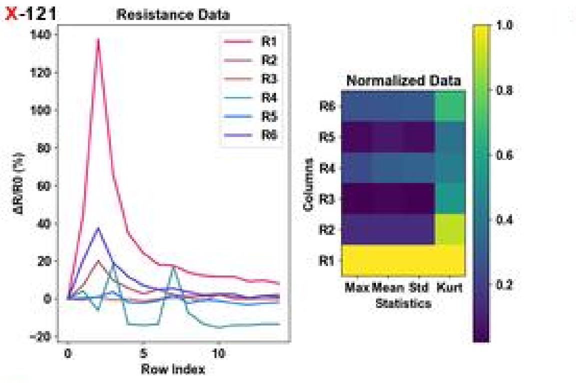

X-122

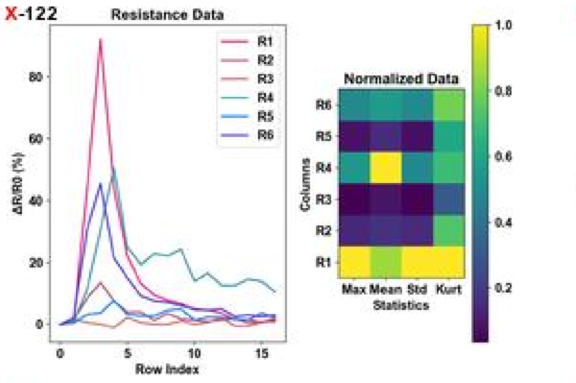

X-123

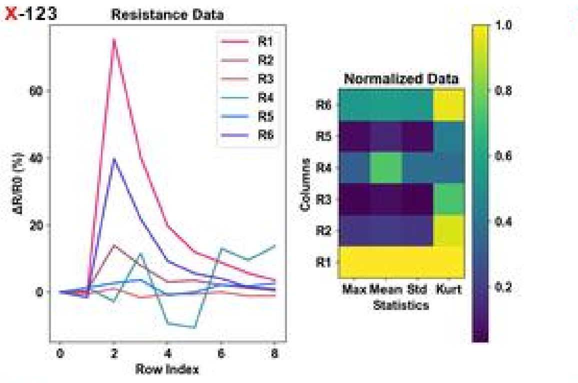

X-124

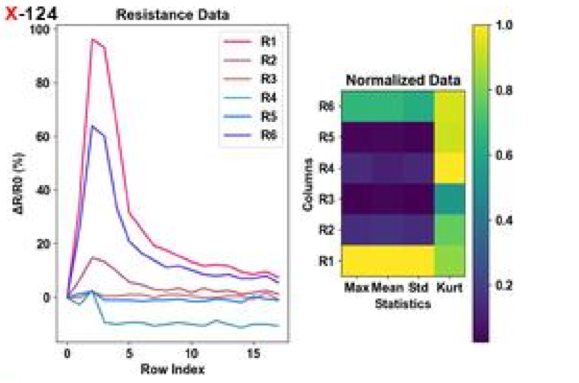

X-125

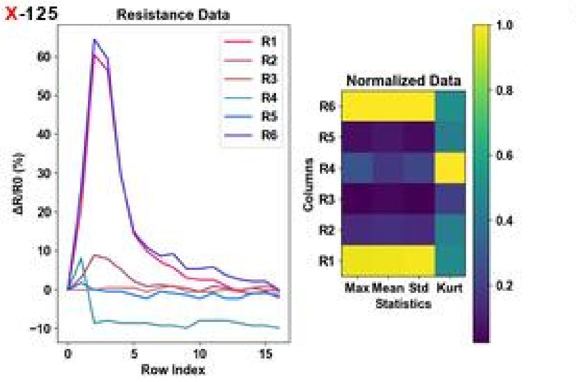

X-126

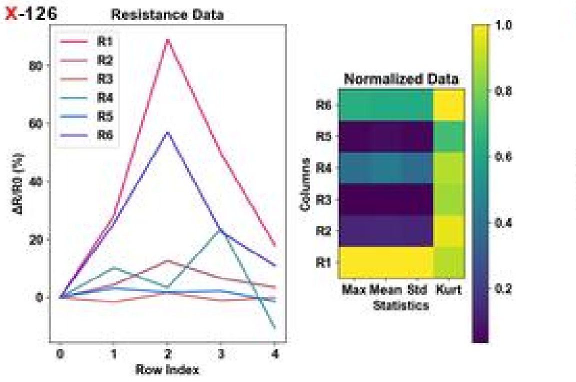

X-127

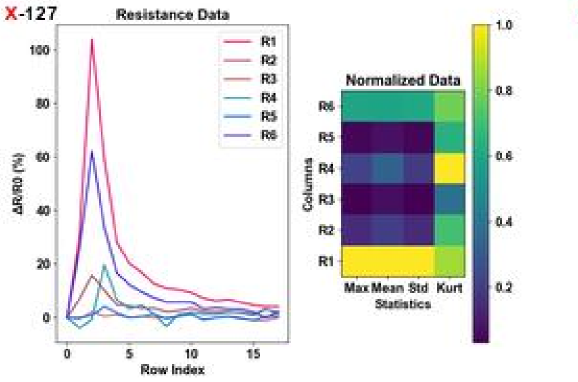

X-128

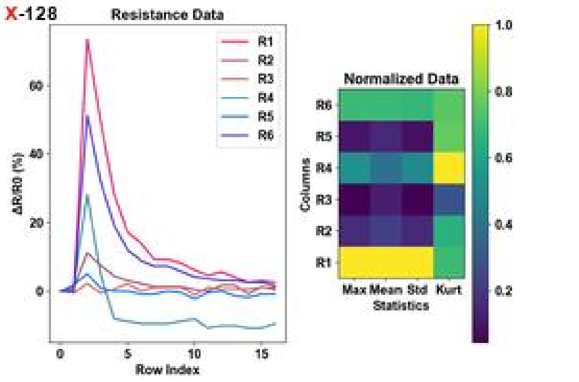

Pressure-sensitive data of type X

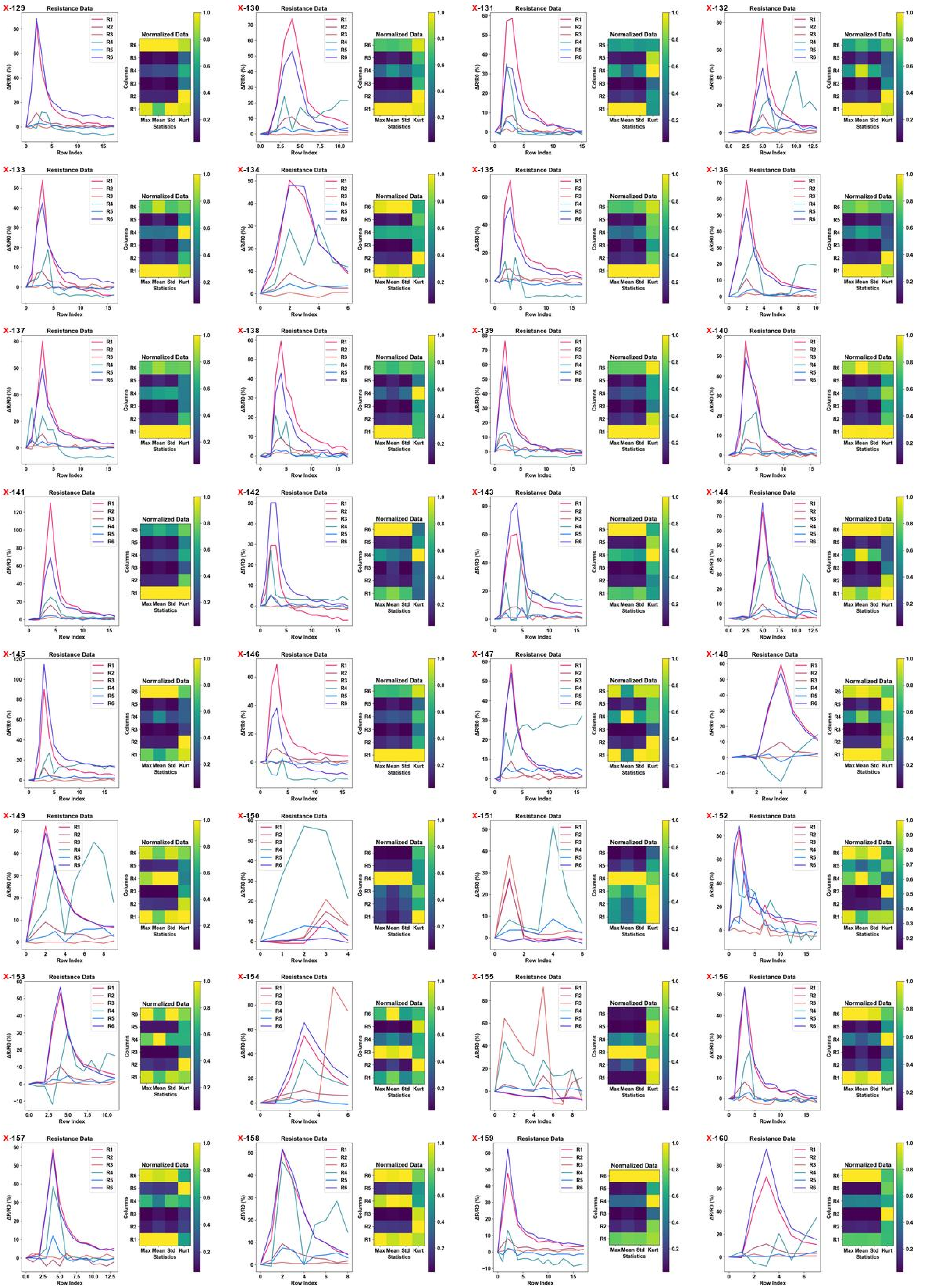

X-161
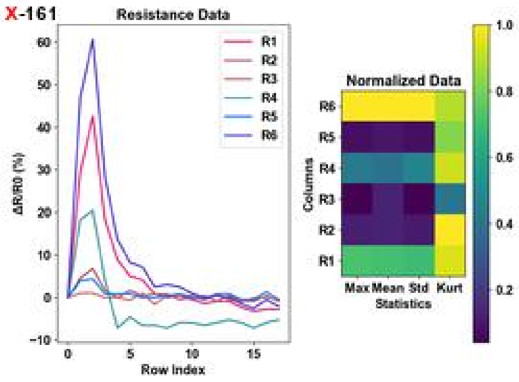

X-162
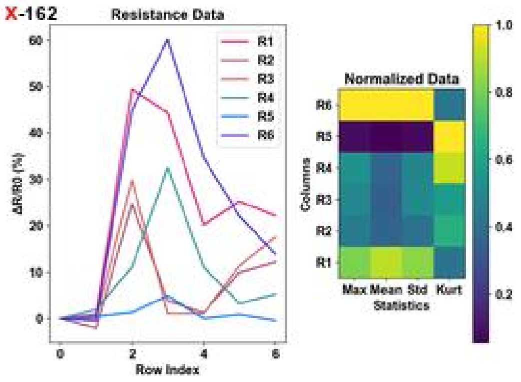

X-163
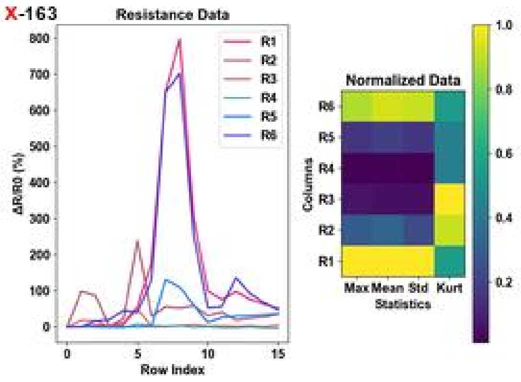

X-164
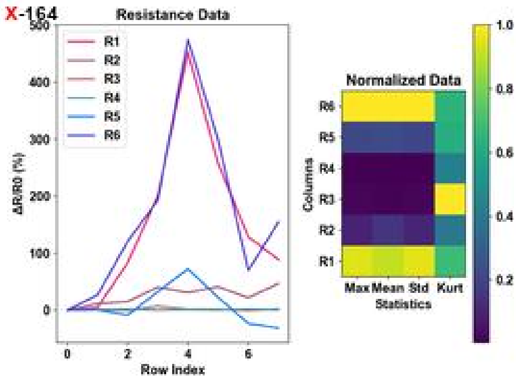

X-165
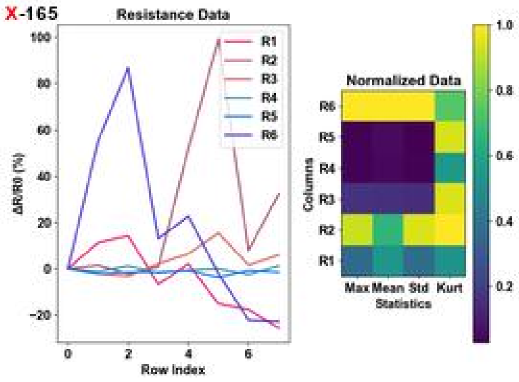

X-166
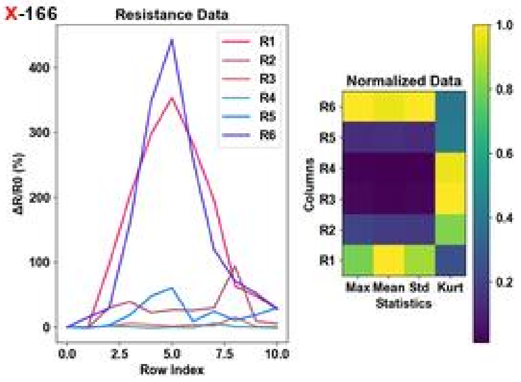

X-167
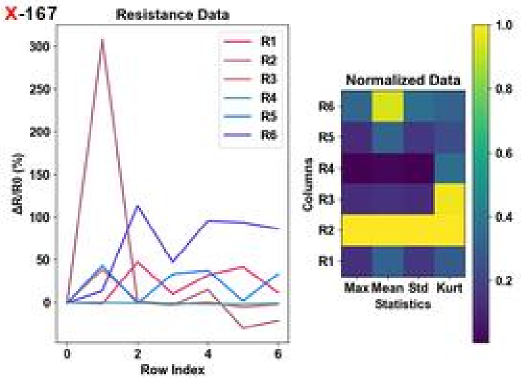

X-168
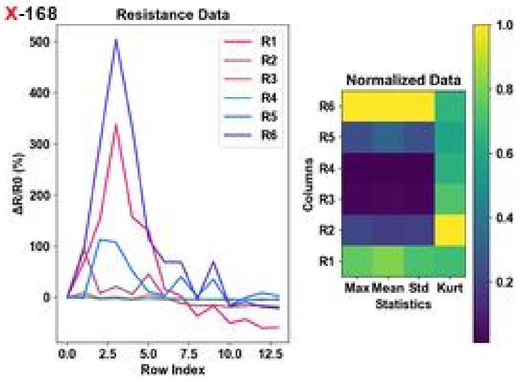

X-169
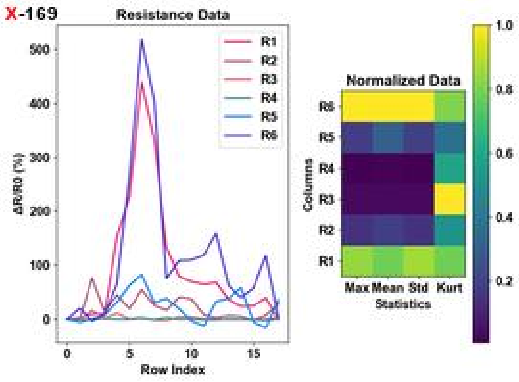

X-170
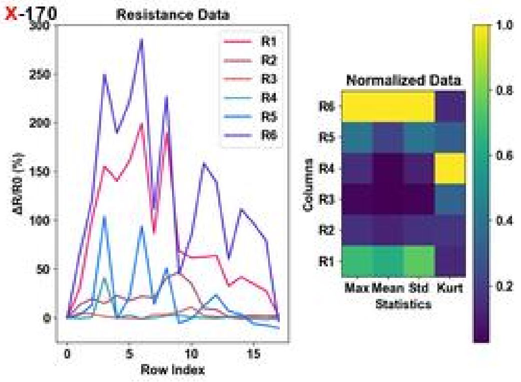

X-171
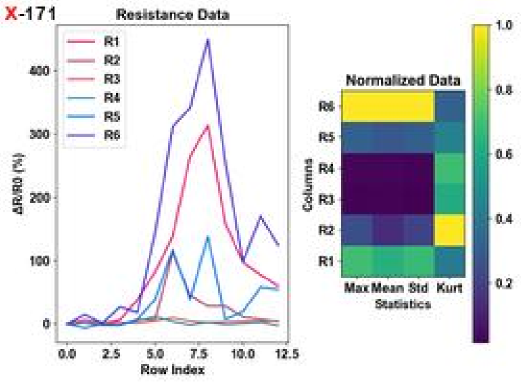

X-172
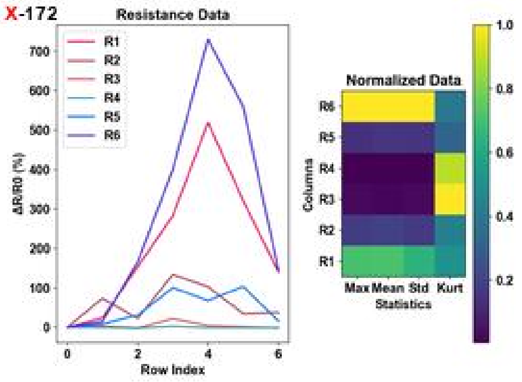

X-173
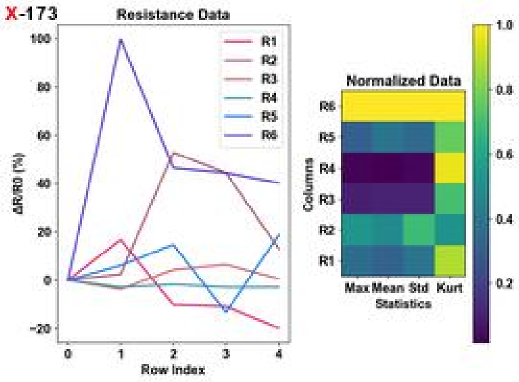

X-174
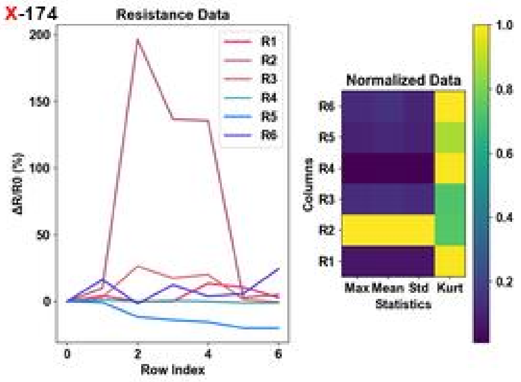

X-175
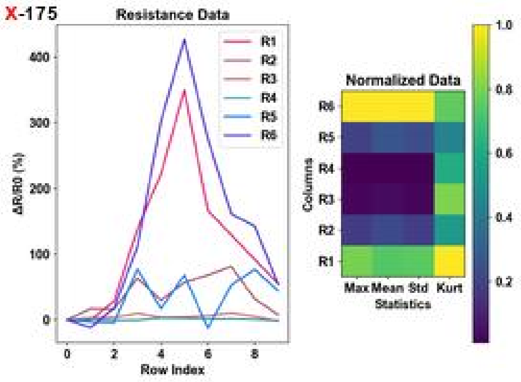

X-176
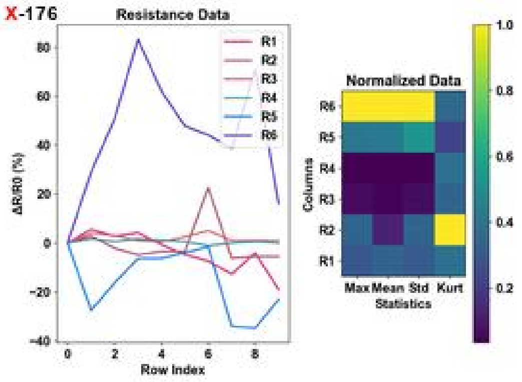

X-177
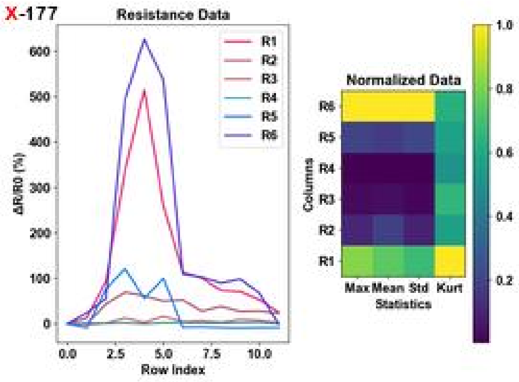

X-178
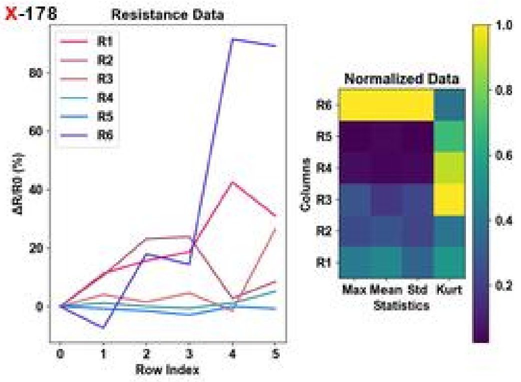

X-179
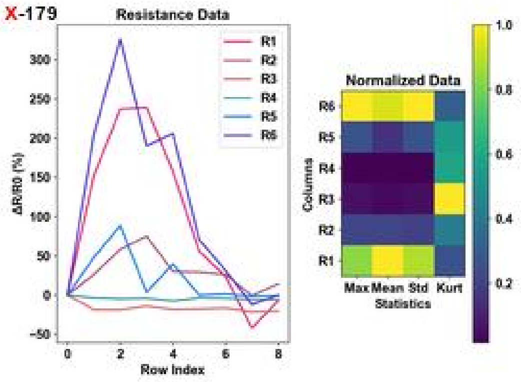

X-180
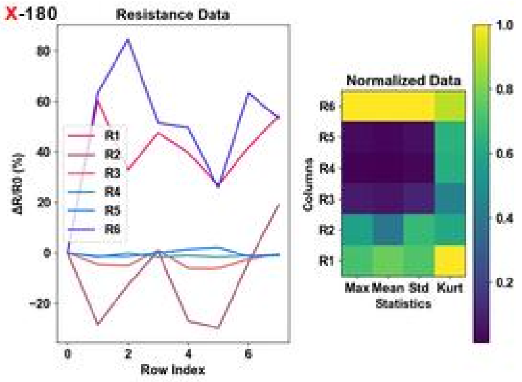

X-181
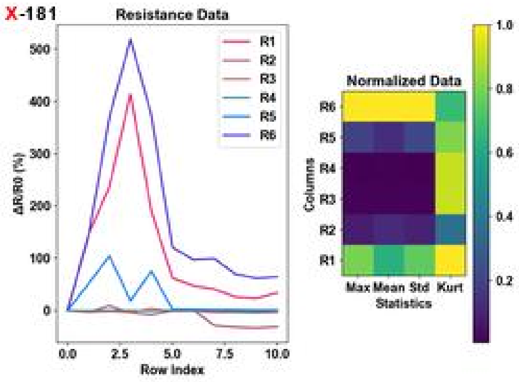

X-182
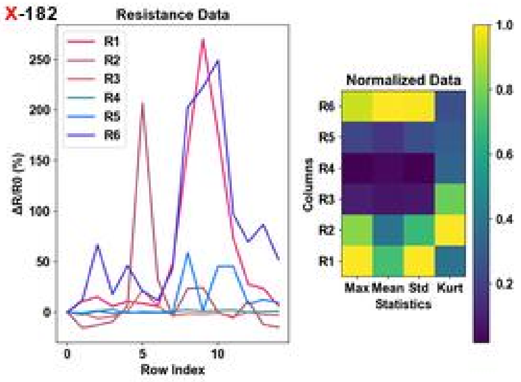

X-183
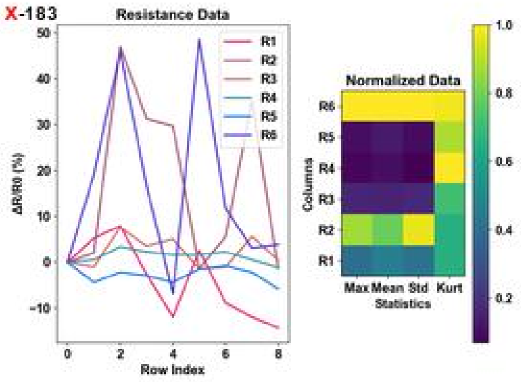

X-184
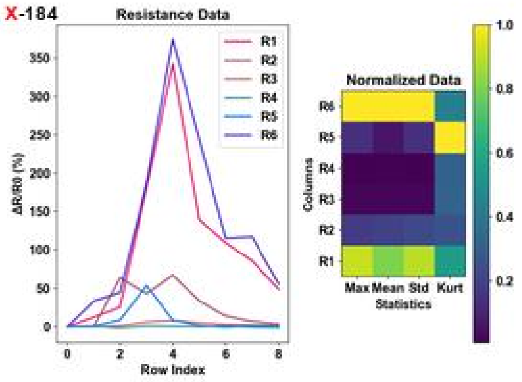

X-185
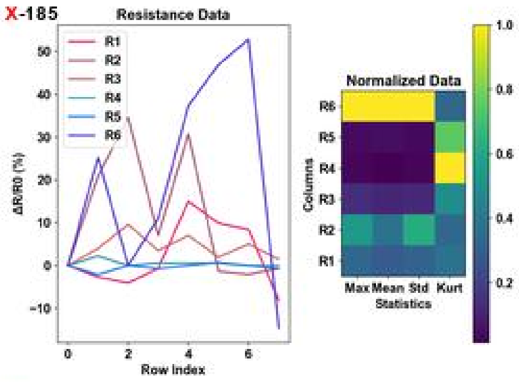

X-186
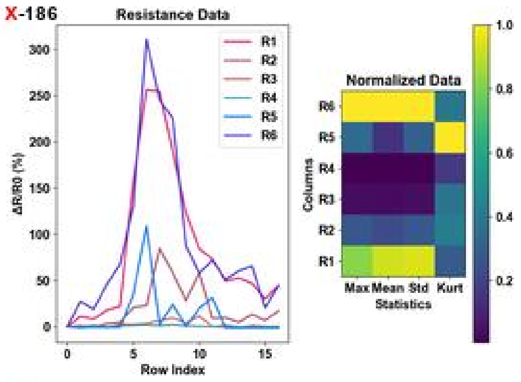

X-187
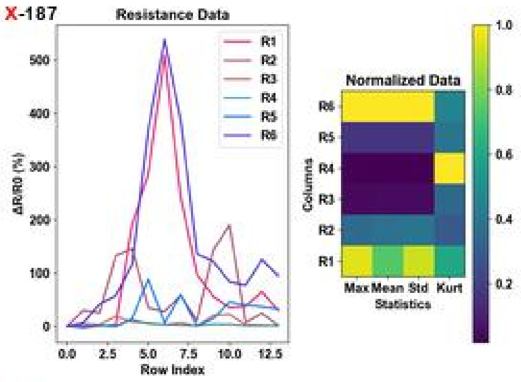

X-188
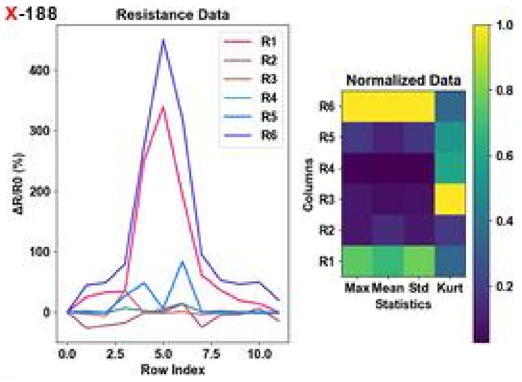

X-189
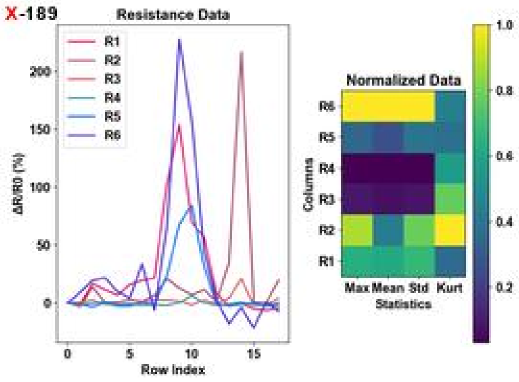

X-190
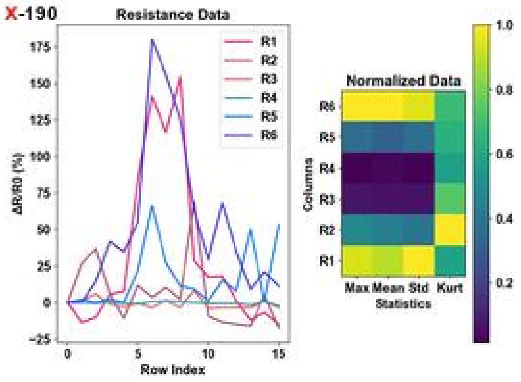

X-191
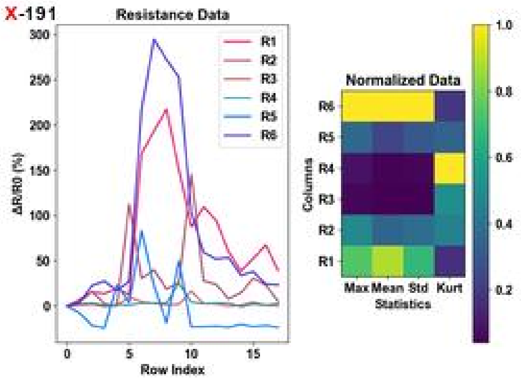

X-192
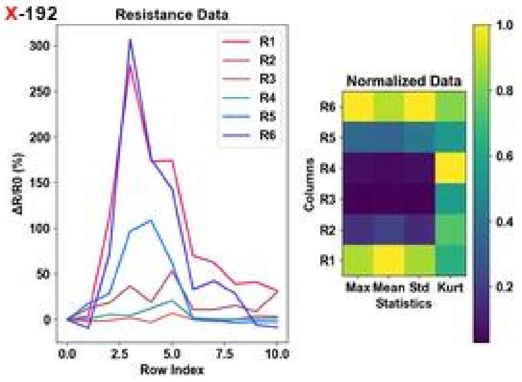

Pressure-sensitive data of type X

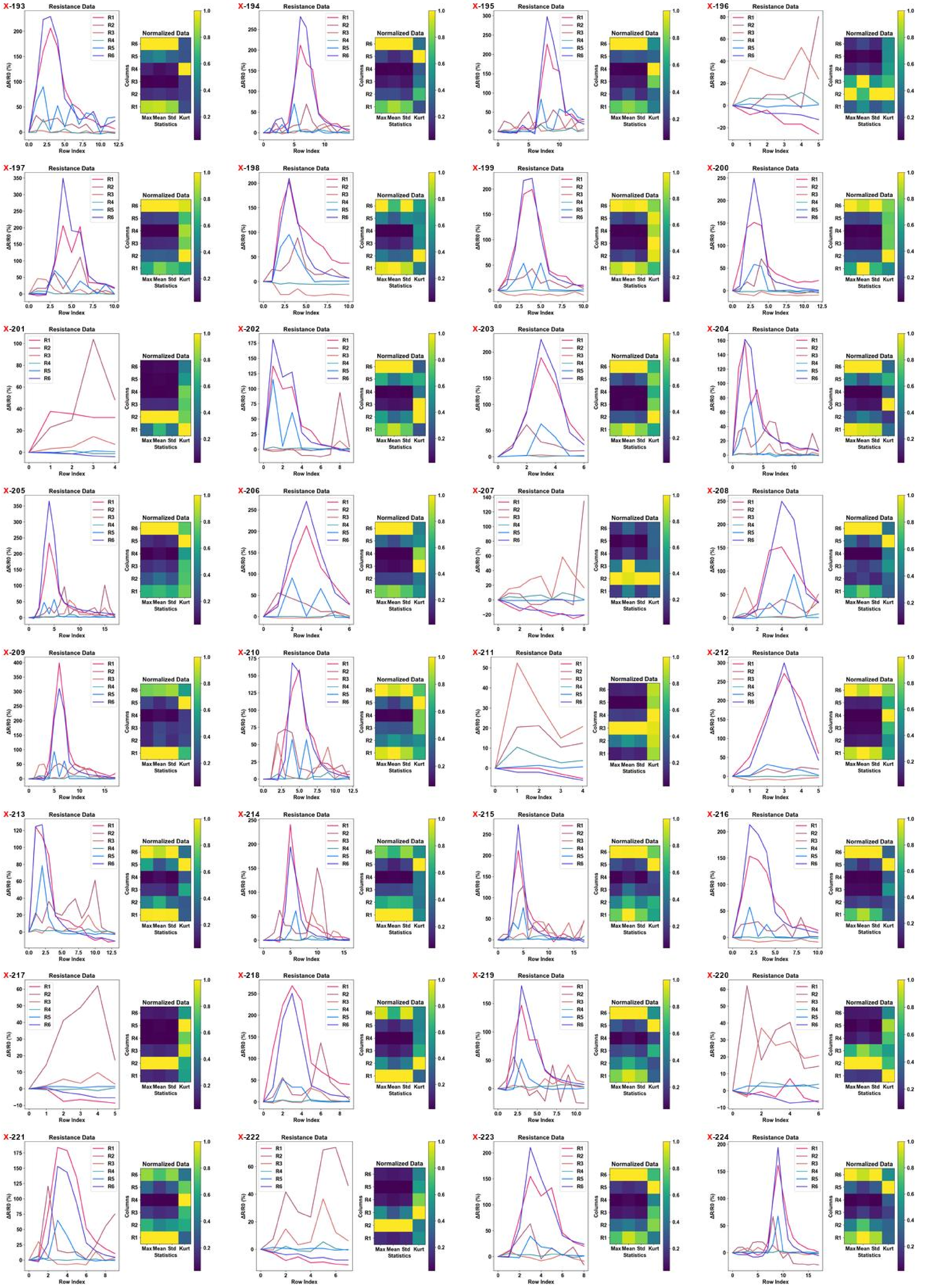

X-225

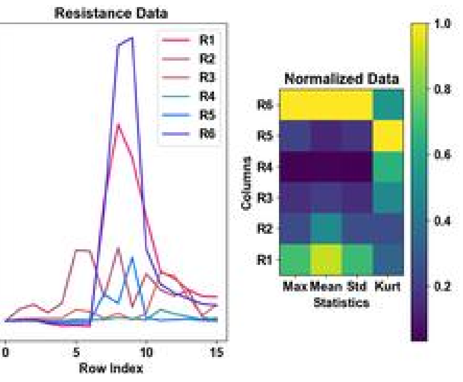

X-226

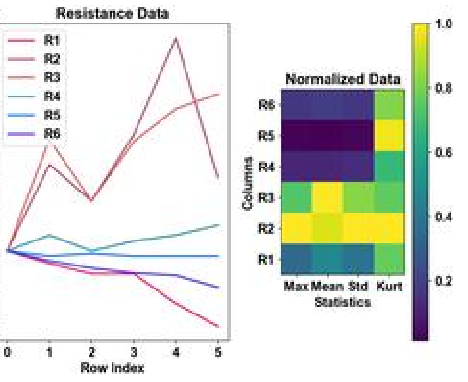

X-227

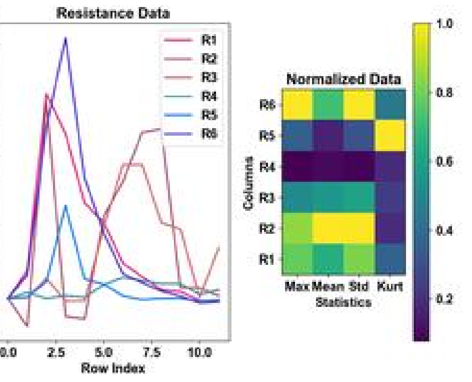

X-228

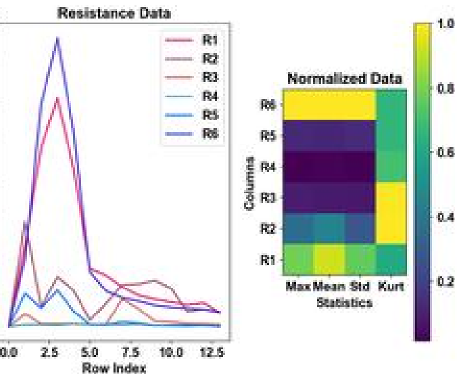

X-229

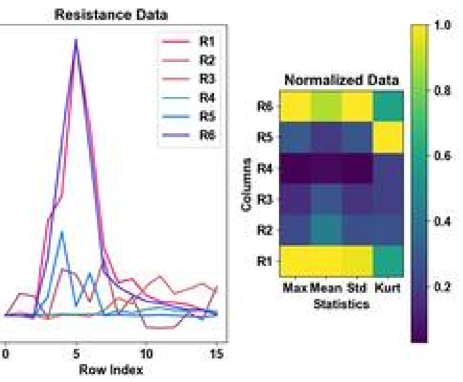

X-230

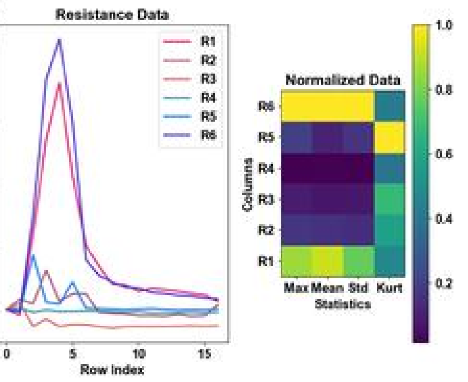

X-231

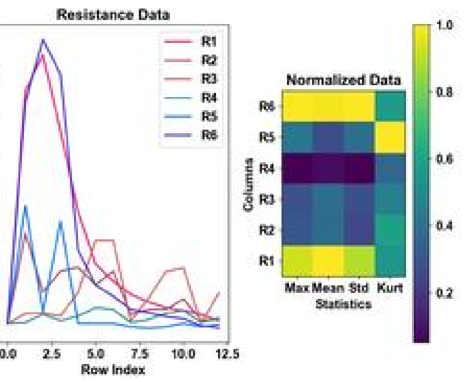

X-232

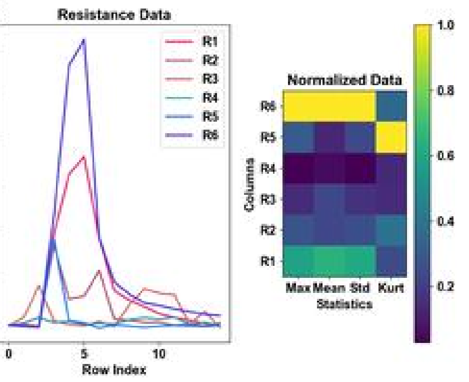

X-233

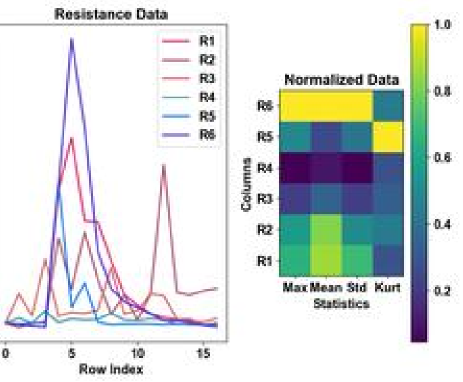

X-234

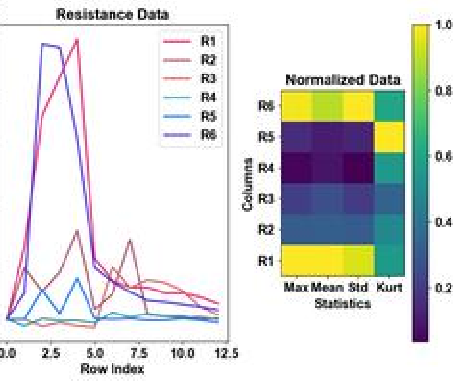

X-235

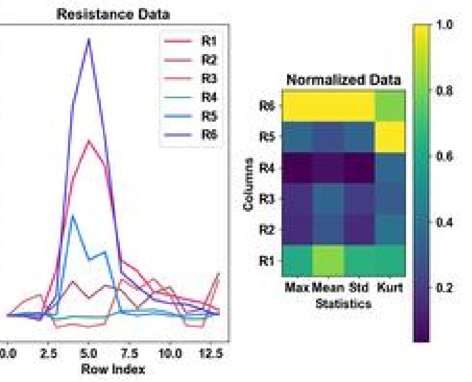

X-236

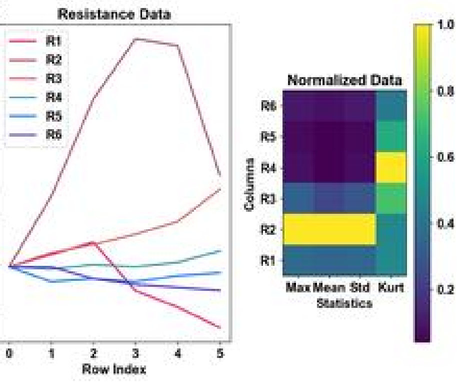

X-237

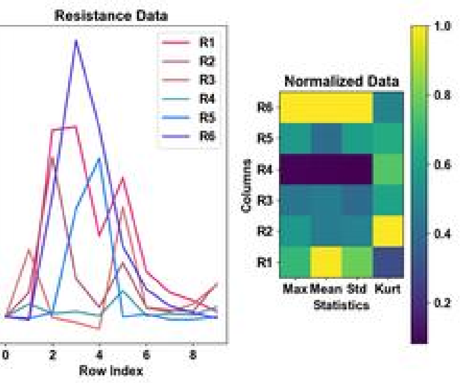

X-238

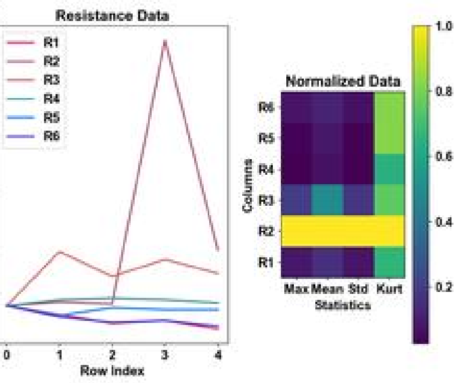

X-239

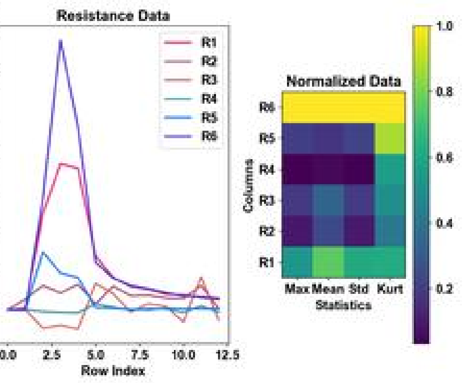

X-240

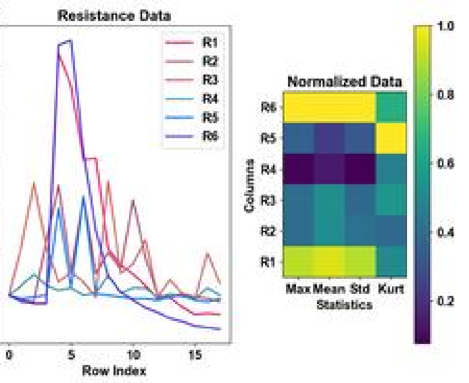

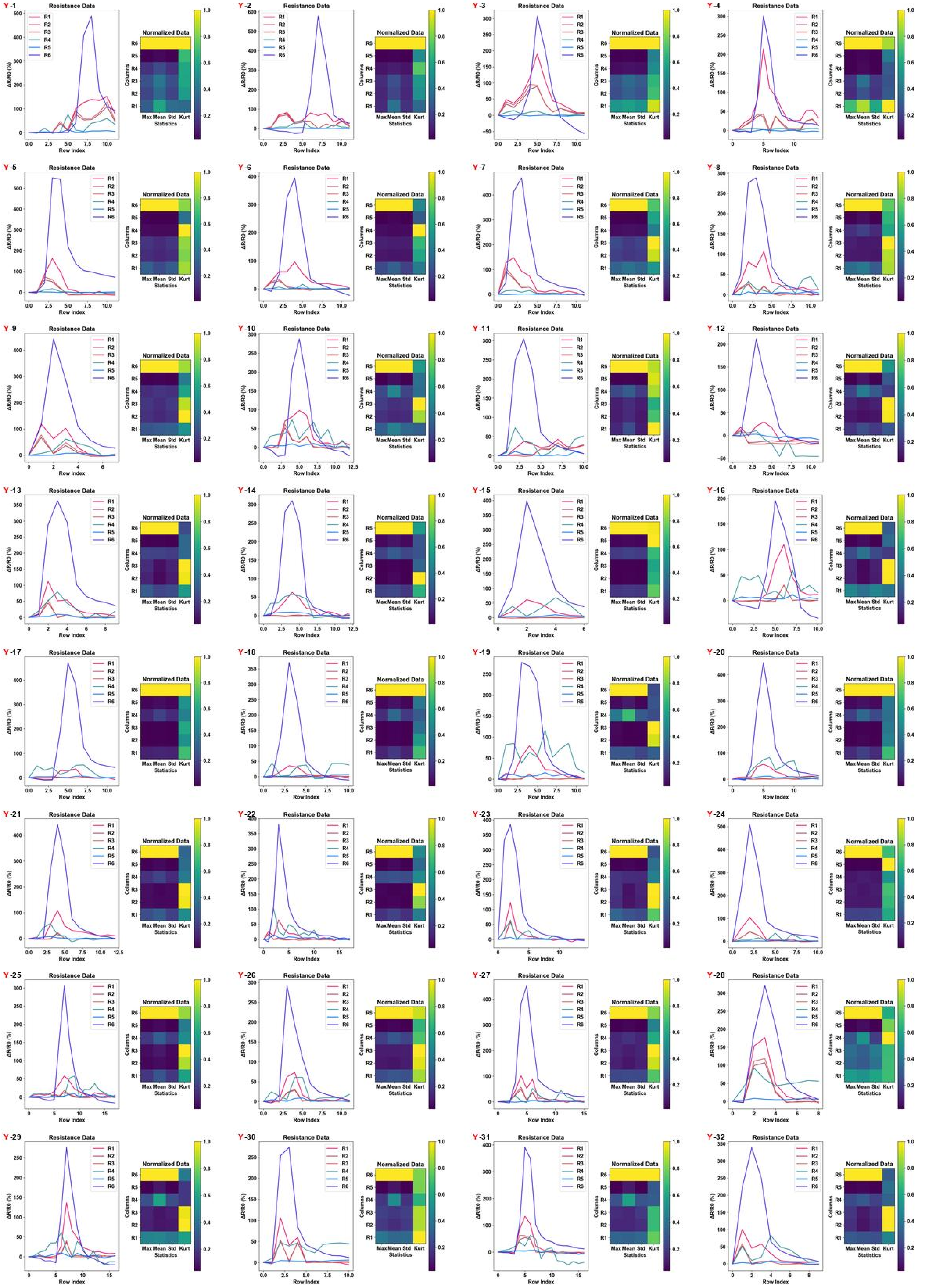
Y-1
Y-2
Y-3
Y-4
Y-5
Y-6
Y-7
Y-8
Y-9
Y-10
Y-11
Y-12
Y-13
Y-14
Y-15
Y-16
Y-17
Y-18
Y-19
Y-20
Y-21
Y-22
Y-23
Y-24
Y-25
Y-26
Y-27
Y-28
Y-29
Y-30
Y-31
Y-32
Resistance Data
Normalized Data
ΔR/R0 (%)
Row Index
Columns
Statistics
Max Mean Std Kurt
R1 R2 R3 R4 R5 R6

Pressure-sensitive data of type Y

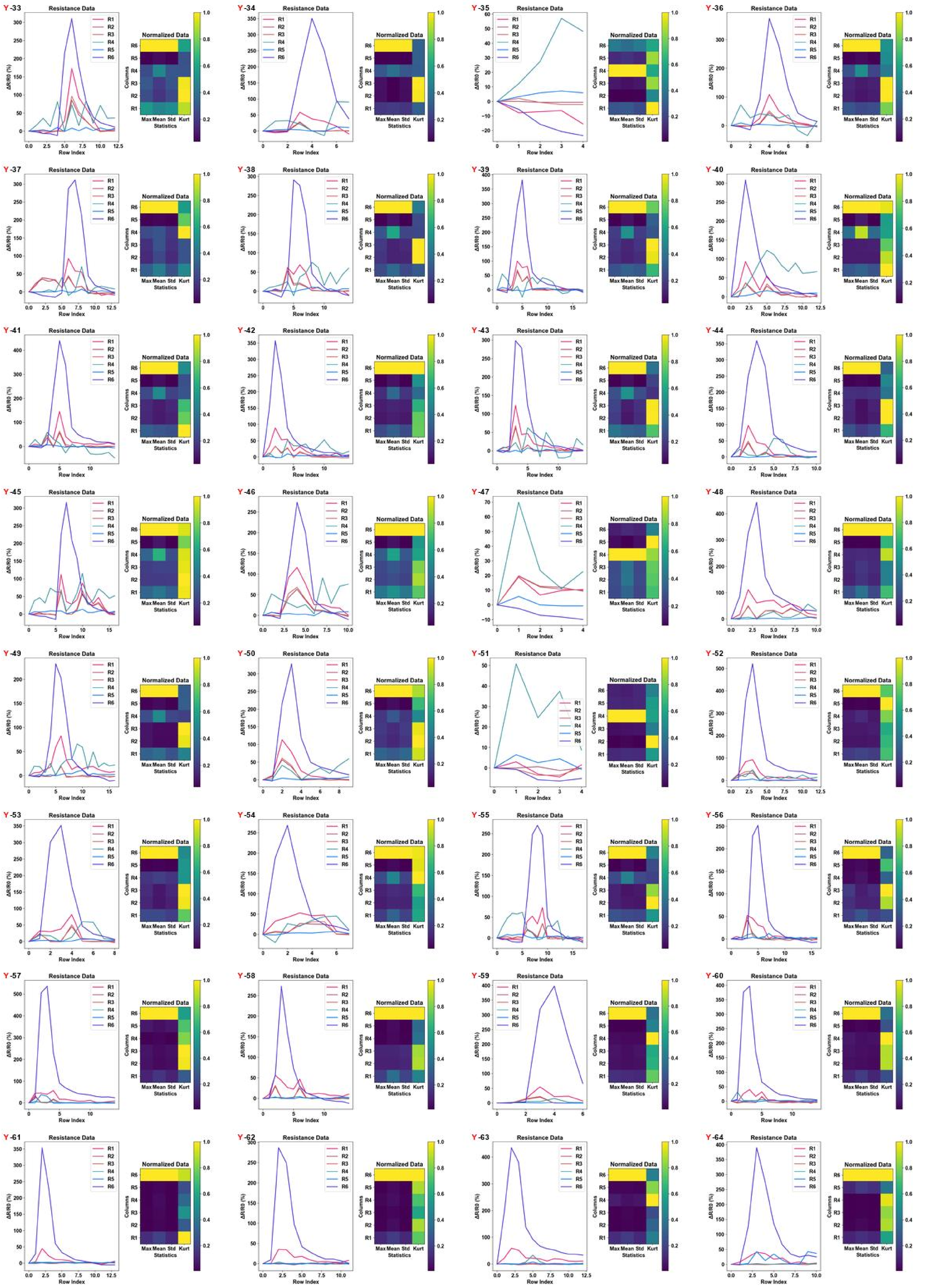

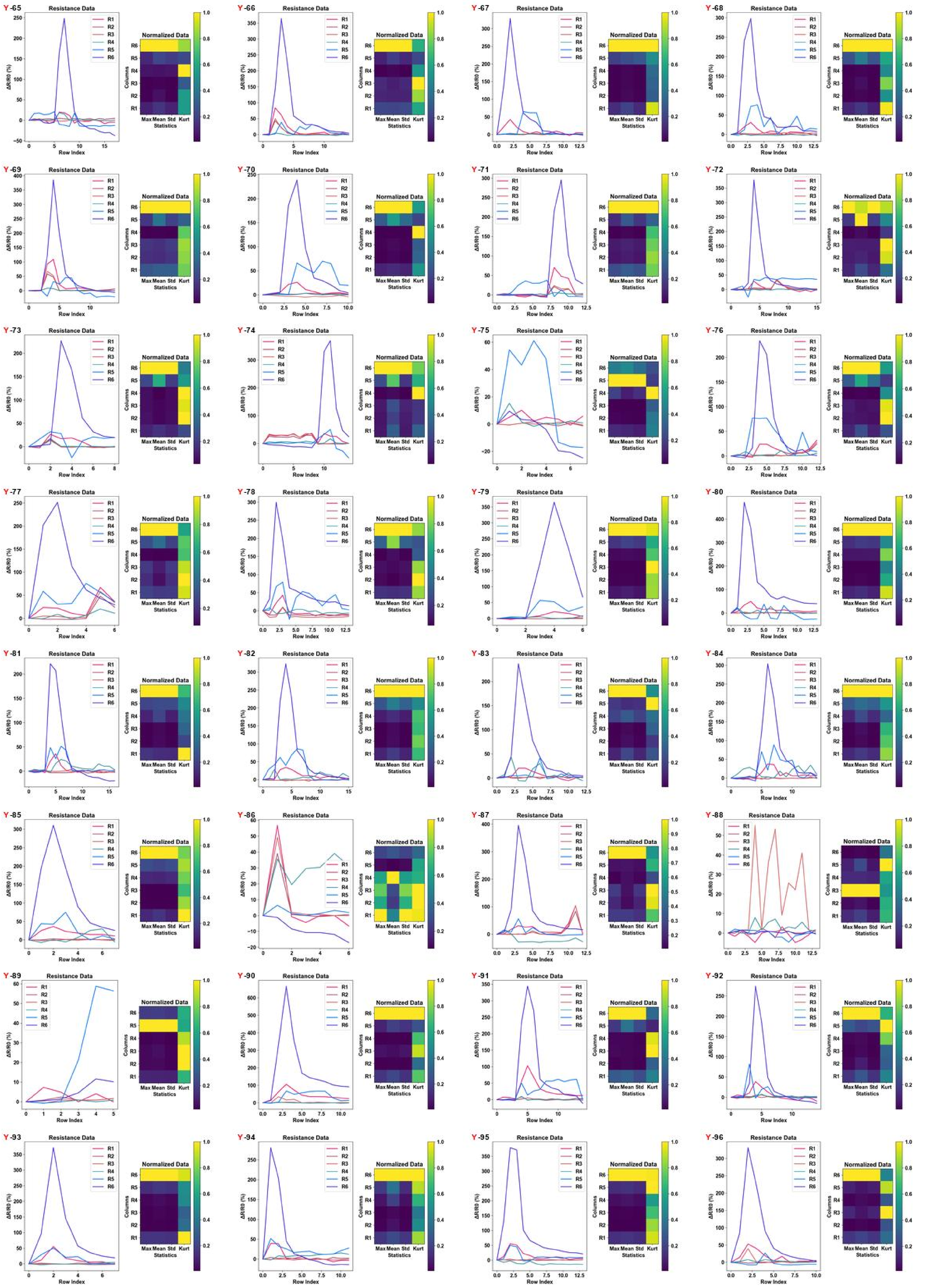
Y-65
Y-66
Y-67
Y-68
Y-69
Y-70
Y-71
Y-72
Y-73
Y-74
Y-75
Y-76
Y-77
Y-78
Y-79
Y-80
Y-81
Y-82
Y-83
Y-84
Y-85
Y-86
Y-87
Y-88
Y-89
Y-90
Y-91
Y-92
Y-93
Y-94
Y-95
Y-96
Resistance Data
Normalized Data
ΔR/R0 (%)
Row Index
Columns
Statistics
Max Mean Std Kurt
R1 R2 R3 R4 R5 R6

Pressure-sensitive data of type Y

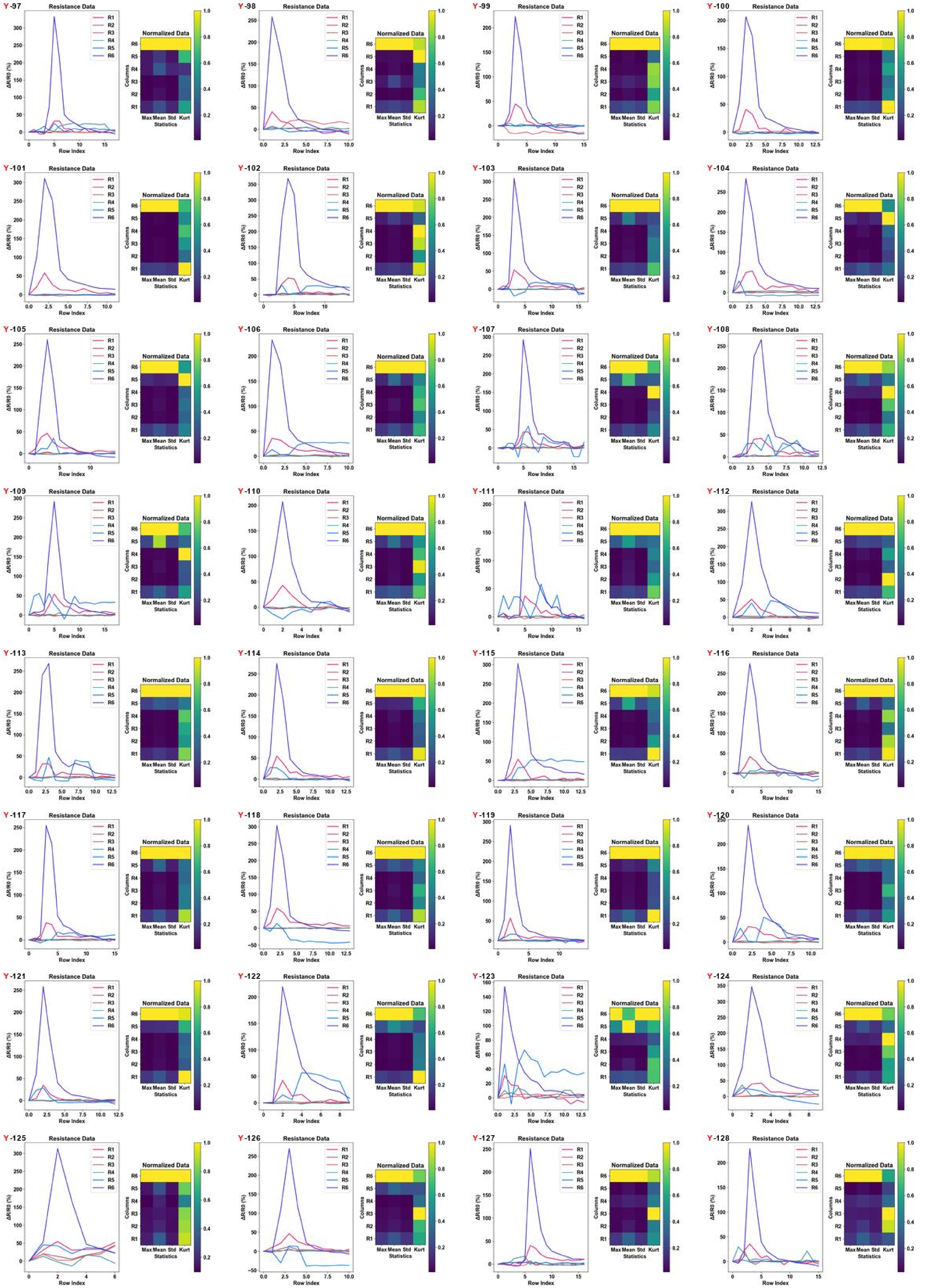

Pressure-sensitive data of type Y

Y-129 Resistance Data

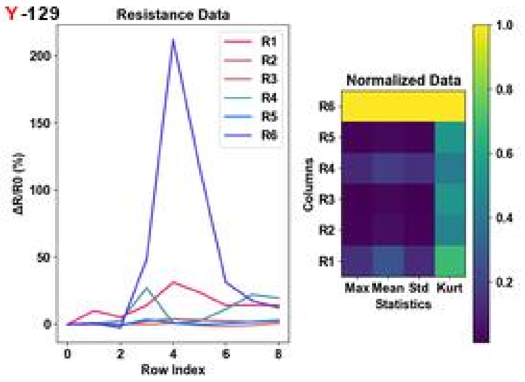

Y-130 Resistance Data

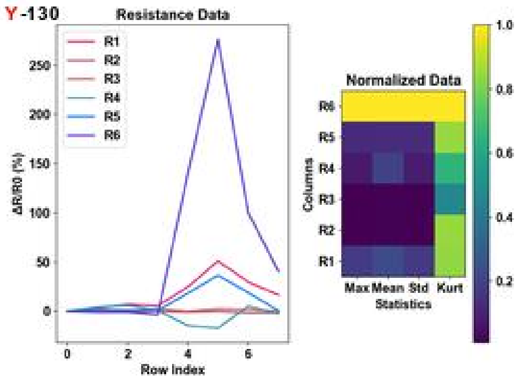

Y-131 Resistance Data

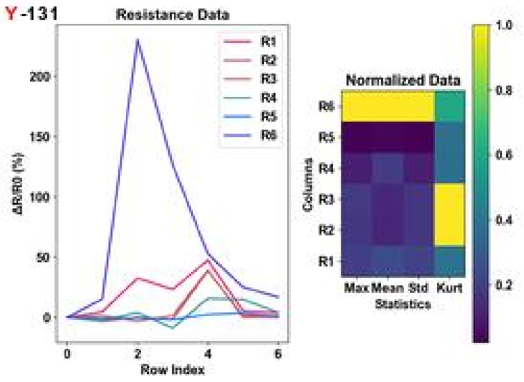

Y-132 Resistance Data

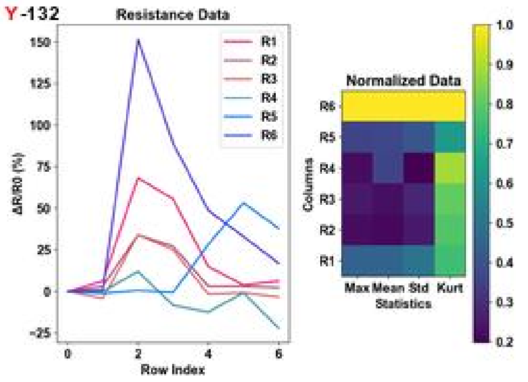

Y-133 Resistance Data

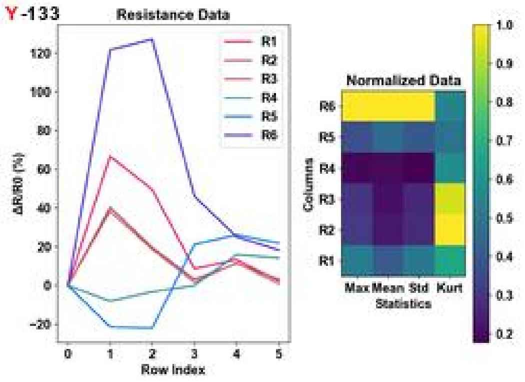

Y-134 Resistance Data

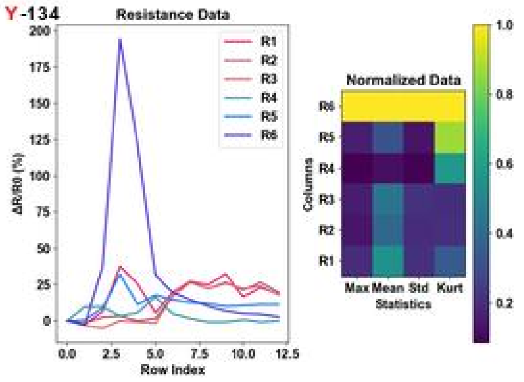

Y-135 Resistance Data

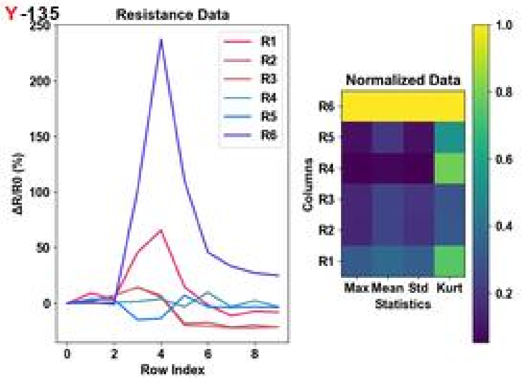

Y-136 Resistance Data

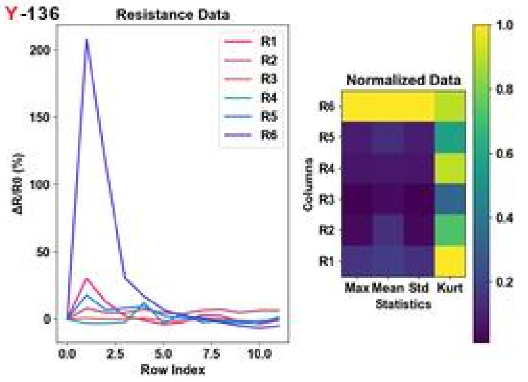

Y-137 Resistance Data

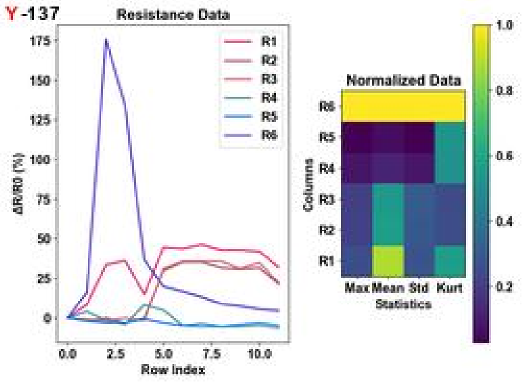

Y-138 Resistance Data

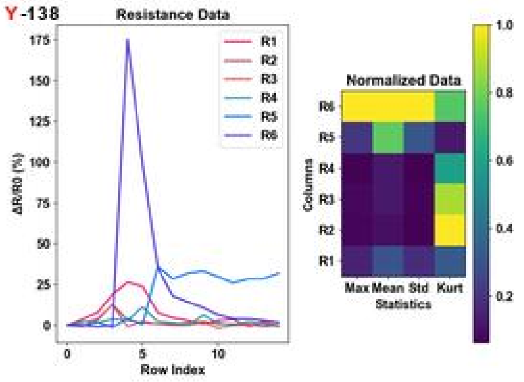

Y-139 Resistance Data

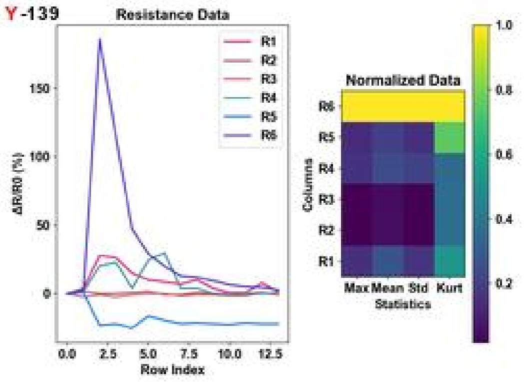

Y-140 Resistance Data

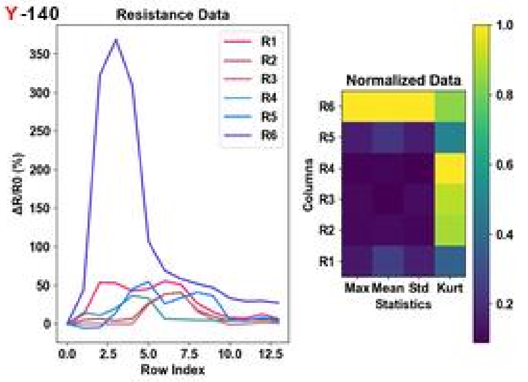

Y-141 Resistance Data

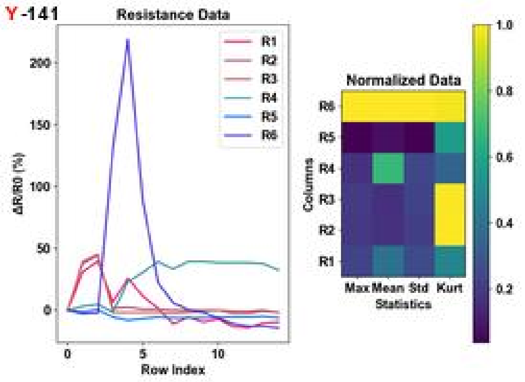

Y-142 Resistance Data

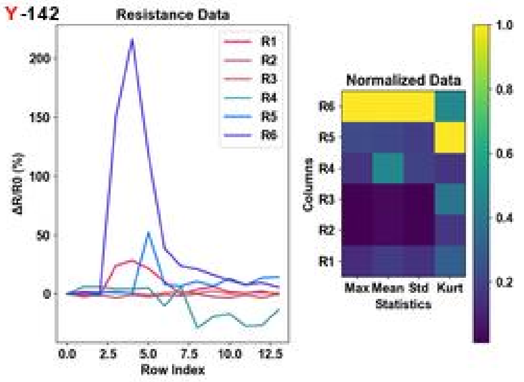

Y-143 Resistance Data

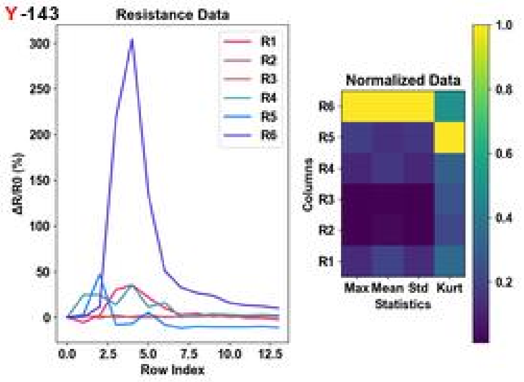

Y-144 Resistance Data

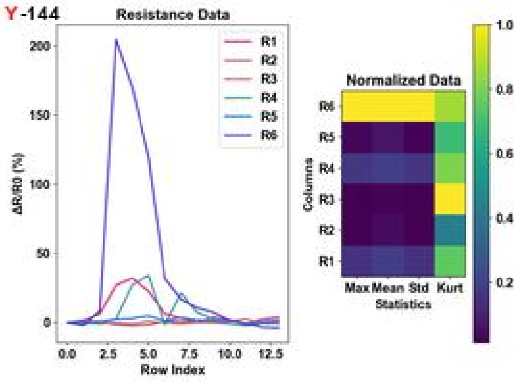

Y-145 Resistance Data

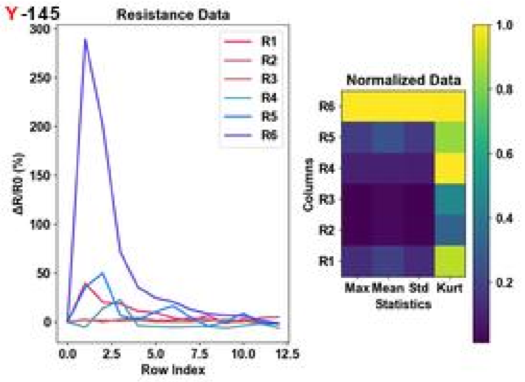

Y-146 Resistance Data

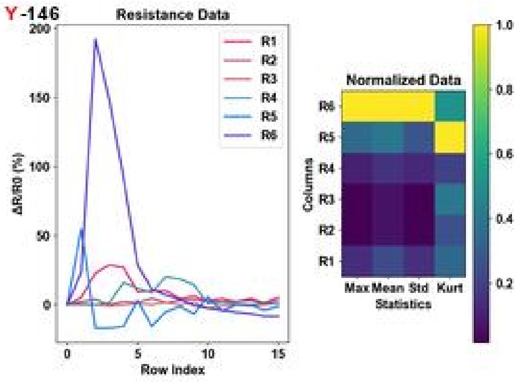

Y-147 Resistance Data

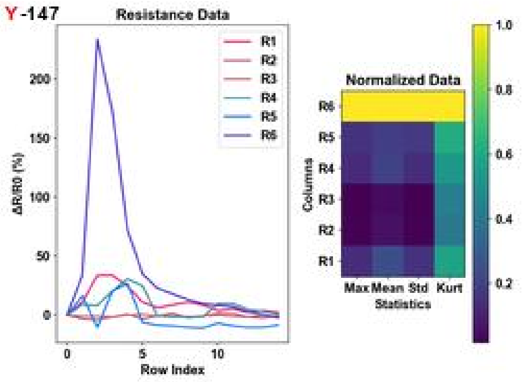

Y-148 Resistance Data

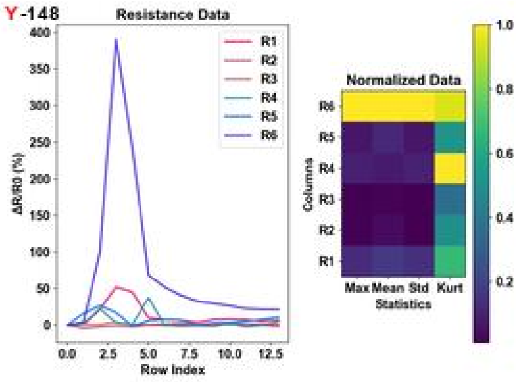

Y-149 Resistance Data

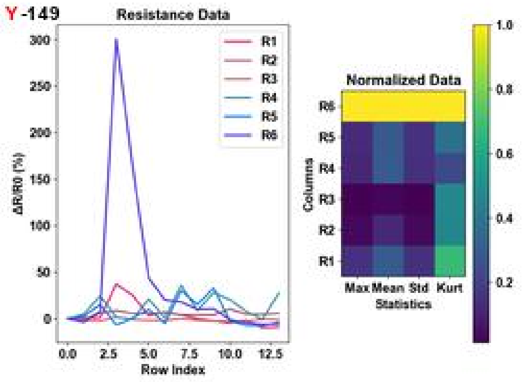

Y-150 Resistance Data

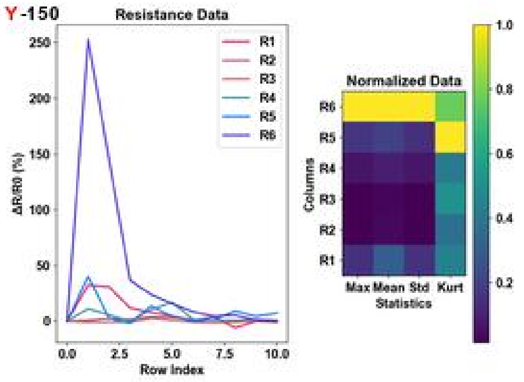

Y-151 Resistance Data

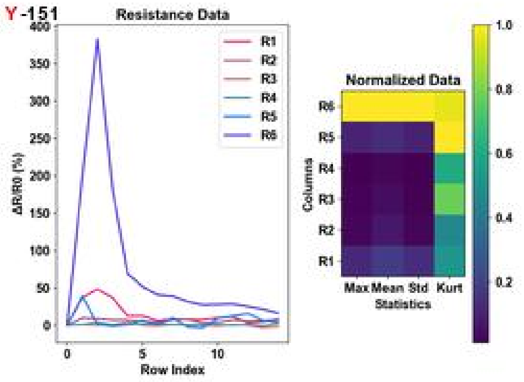

Y-152 Resistance Data

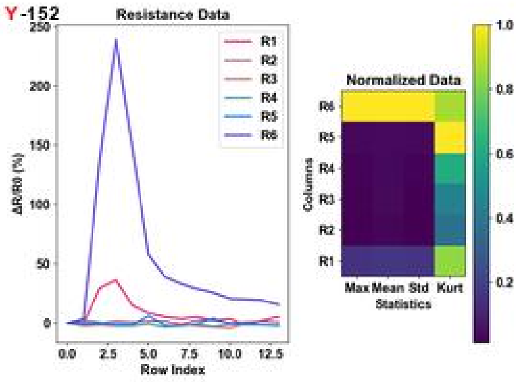

Y-153 Resistance Data

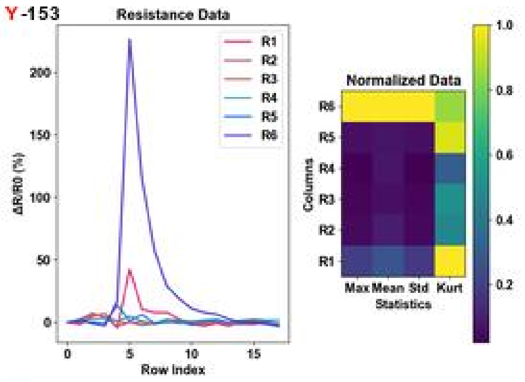

Y-154 Resistance Data

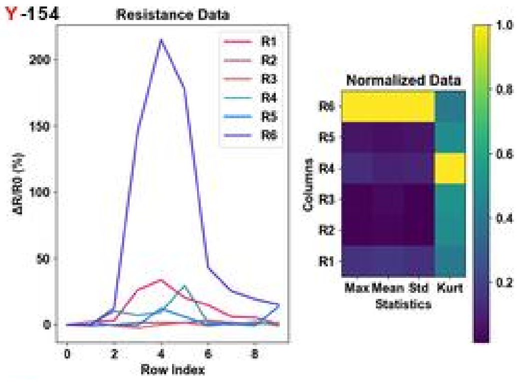

Y-155 Resistance Data

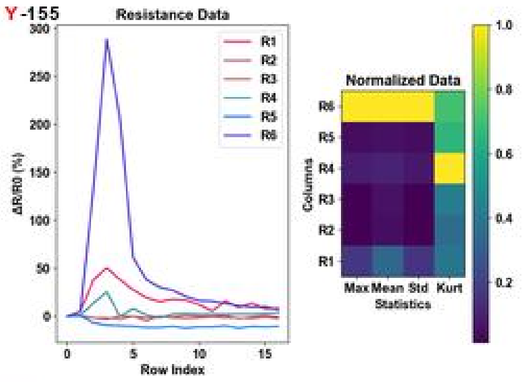

Y-156 Resistance Data

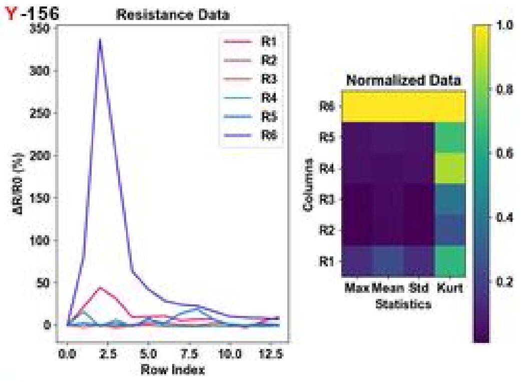

Y-157 Resistance Data

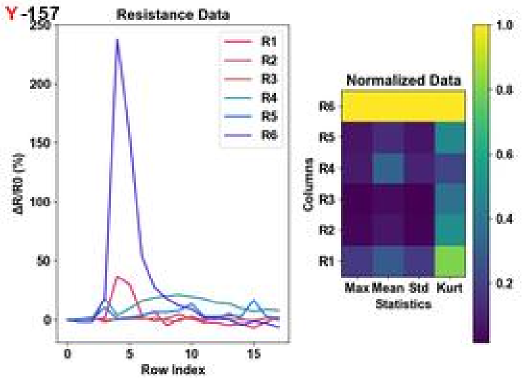

Y-158 Resistance Data

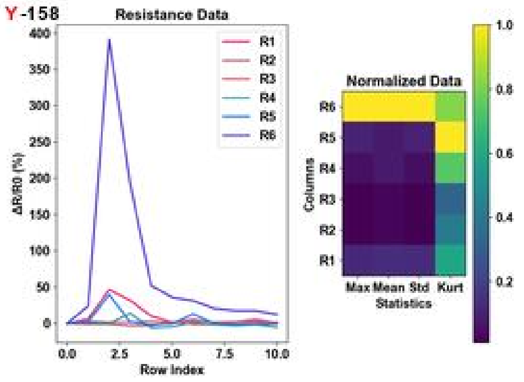

Y-159 Resistance Data

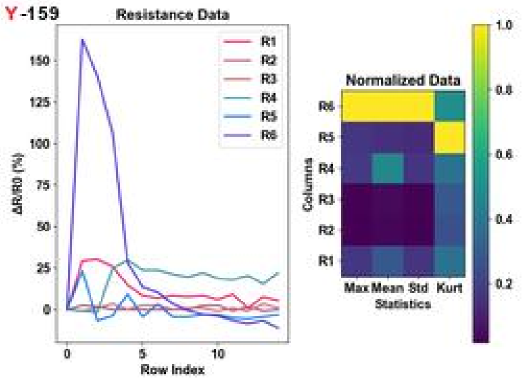

Y-160 Resistance Data

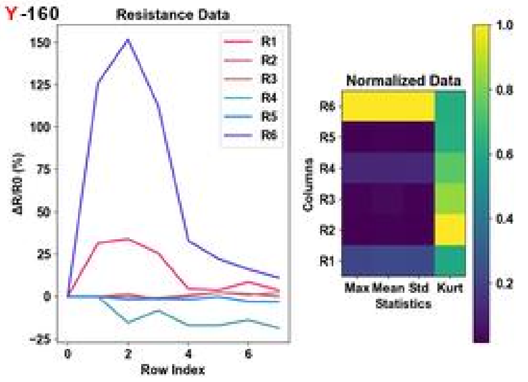

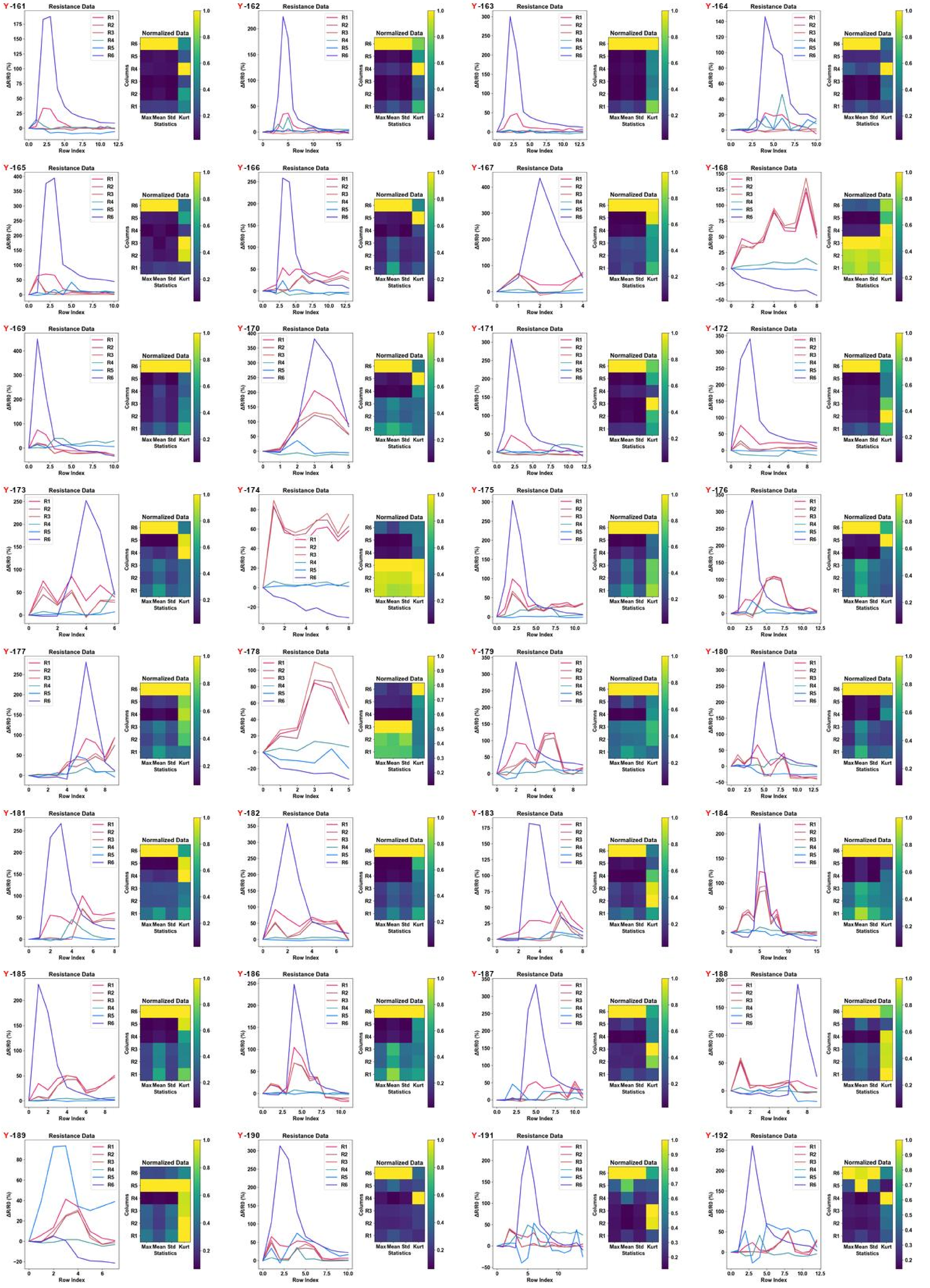
Y-161
Y-162
Y-163
Y-164
Y-165
Y-166
Y-167
Y-168
Y-169
Y-170
Y-171
Y-172
Y-173
Y-174
Y-175
Y-176
Y-177
Y-178
Y-179
Y-180
Y-181
Y-182
Y-183
Y-184
Y-185
Y-186
Y-187
Y-188
Y-189
Y-190
Y-191
Y-192
Resistance Data
Normalized Data
ΔR/R0 (%)
Row Index
Columns
Statistics
Max Mean Std Kurt
R1 R2 R3 R4 R5 R6

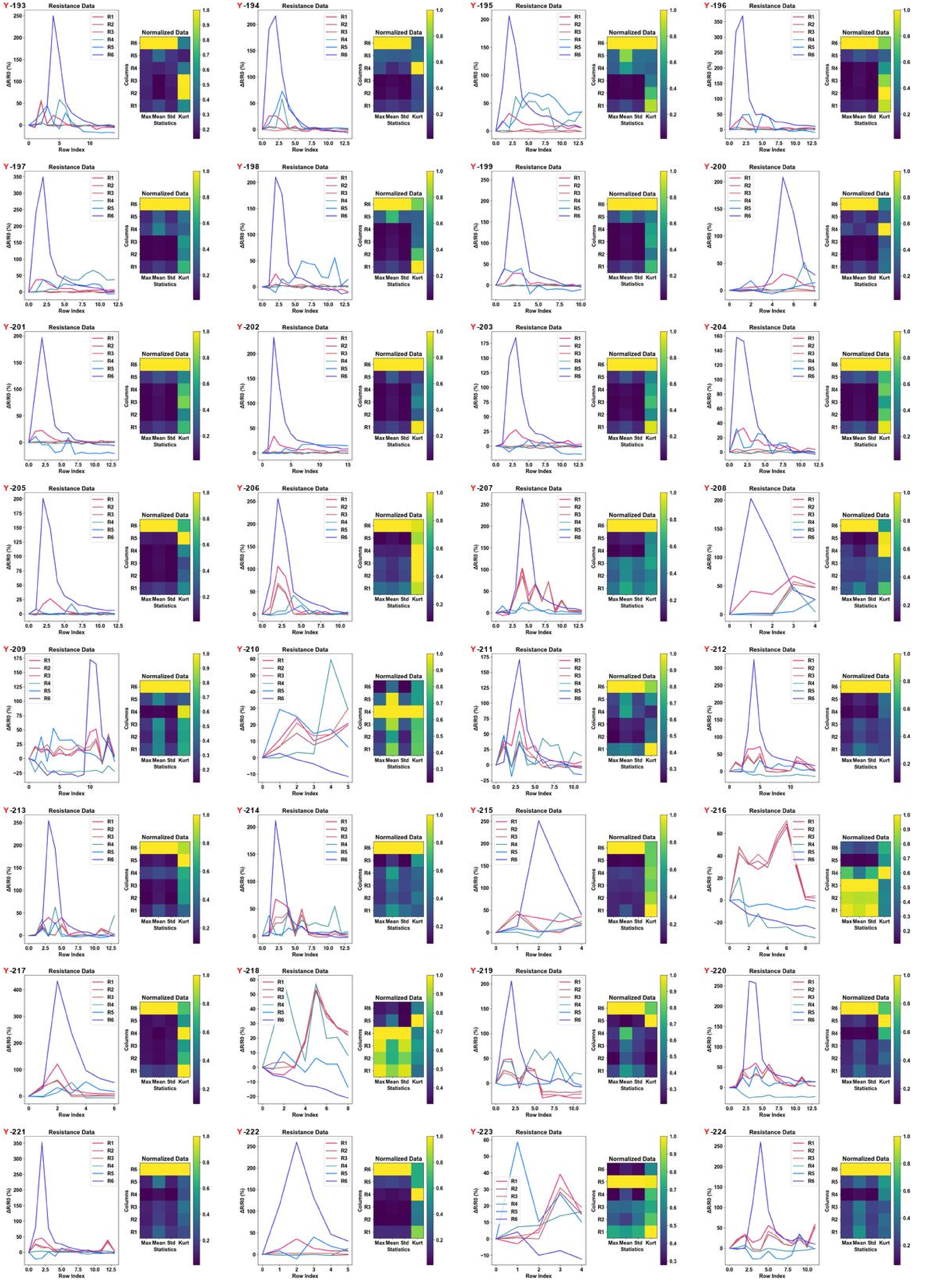
Y-193
Y-194
Y-195
Y-196
Y-197
Y-198
Y-199
Y-200
Y-201
Y-202
Y-203
Y-204
Y-205
Y-206
Y-207
Y-208
Y-209
Y-210
Y-211
Y-212
Y-213
Y-214
Y-215
Y-216
Y-217
Y-218
Y-219
Y-220
Y-221
Y-222
Y-223
Y-224
Resistance Data
Normalized Data
ΔR/R0 (%)
Row Index
Columns
Statistics
Max Mean Std Kurt
R1 R2 R3 R4 R5 R6

Y-225

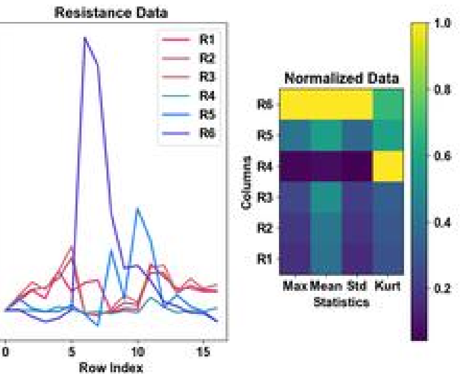

Y-226

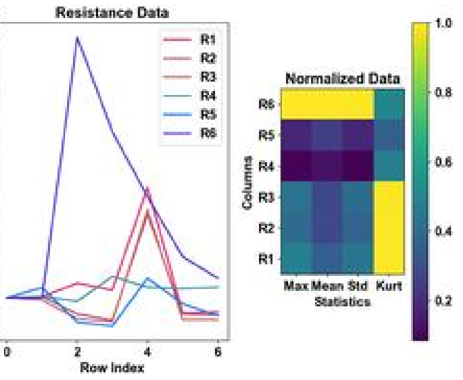

Y-227

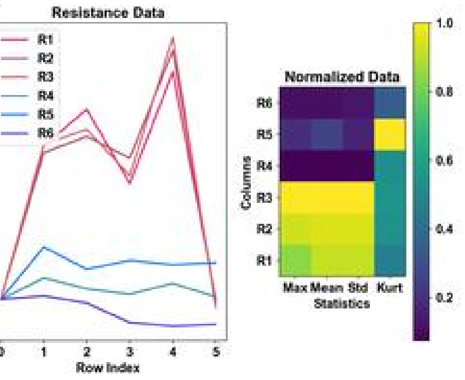

Y-228

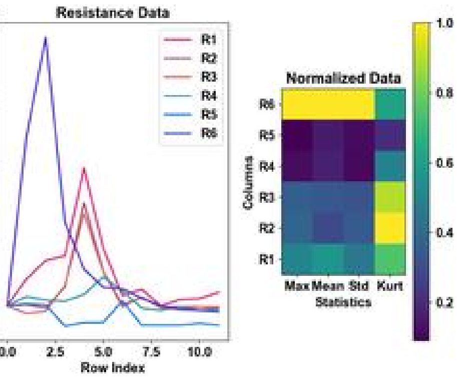

Y-229

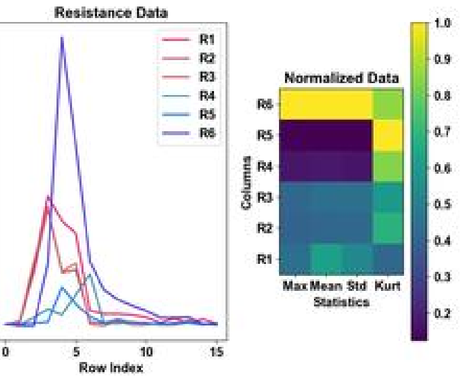

Y-230

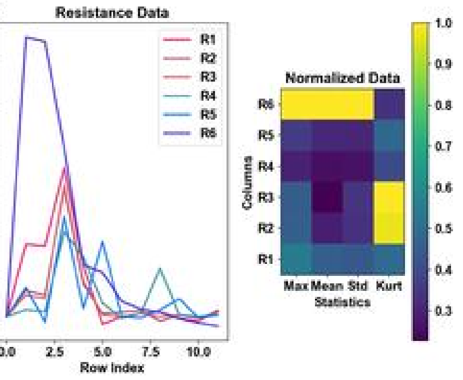

Y-231

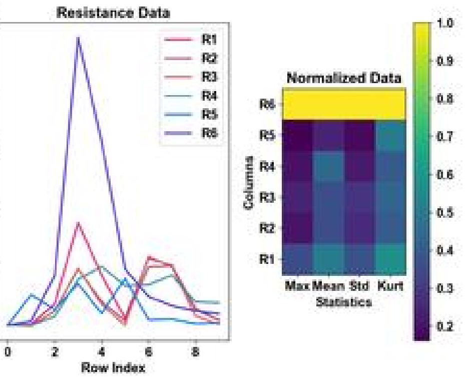

Y-232

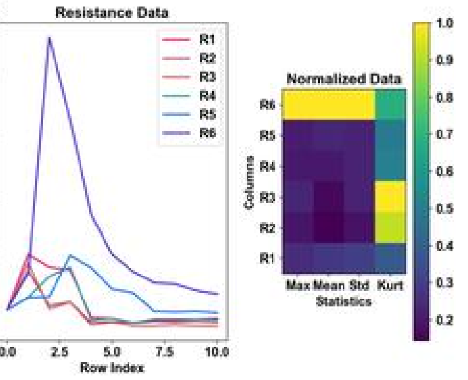

Y-233

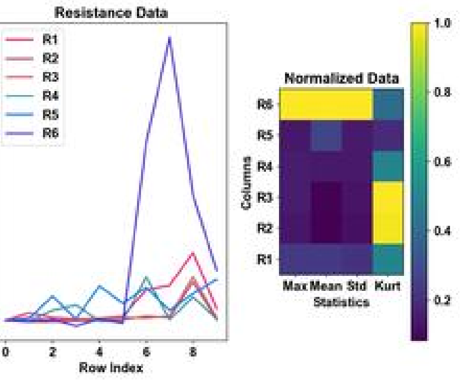

Y-234

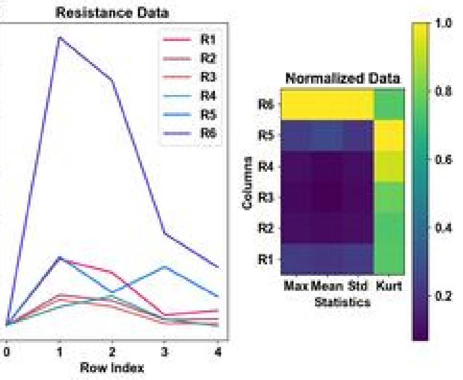

Y-235

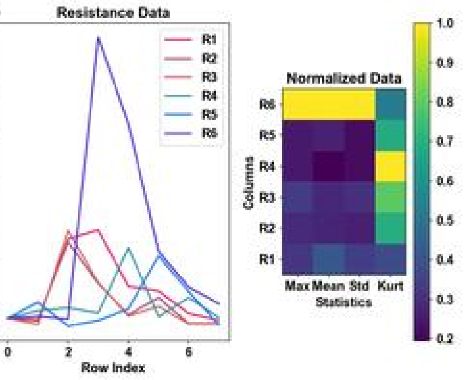

Y-236

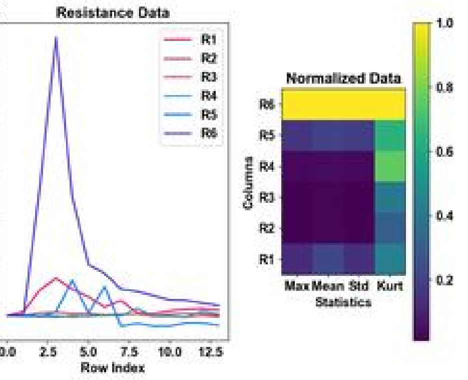

Y-237

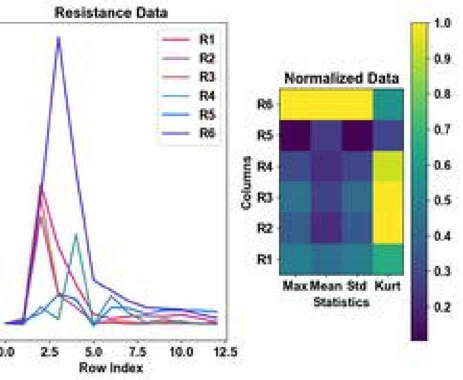

Y-238

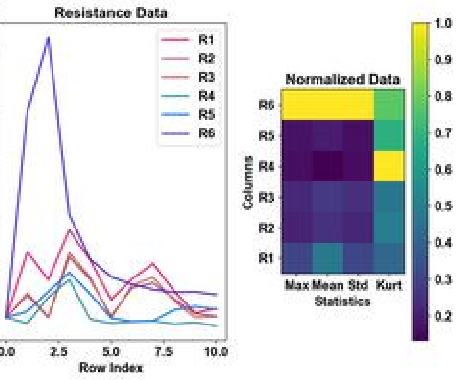

Y-239

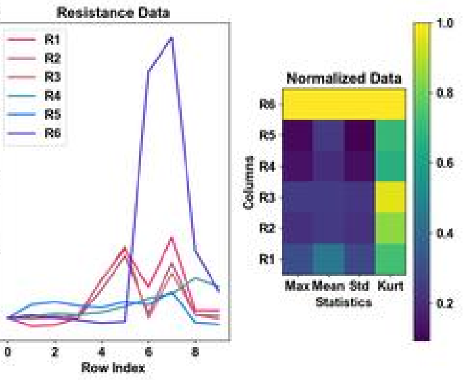

Y-240

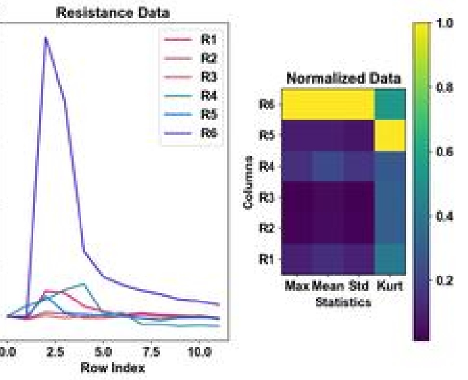

Pressure-sensitive data of type Z

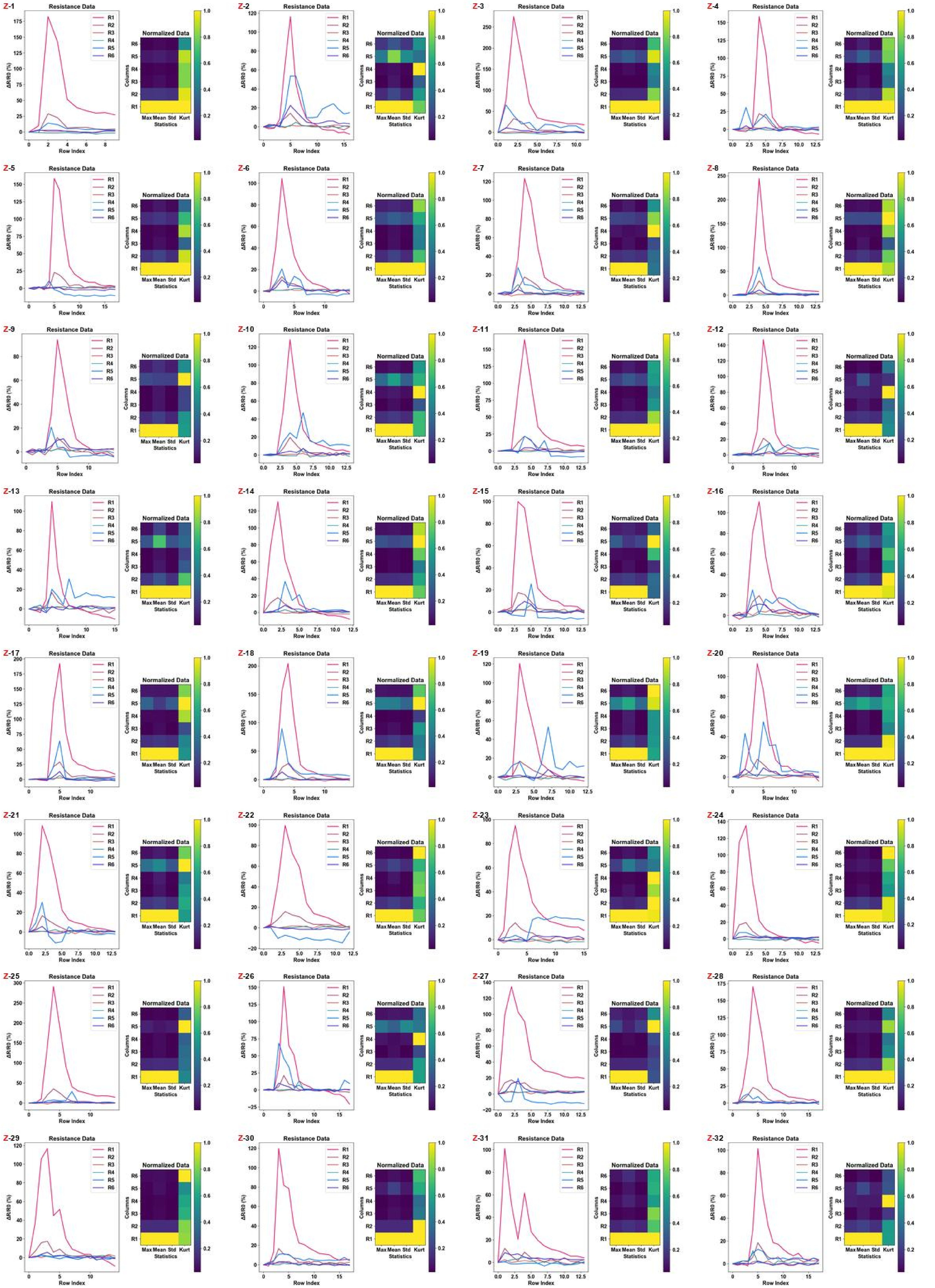

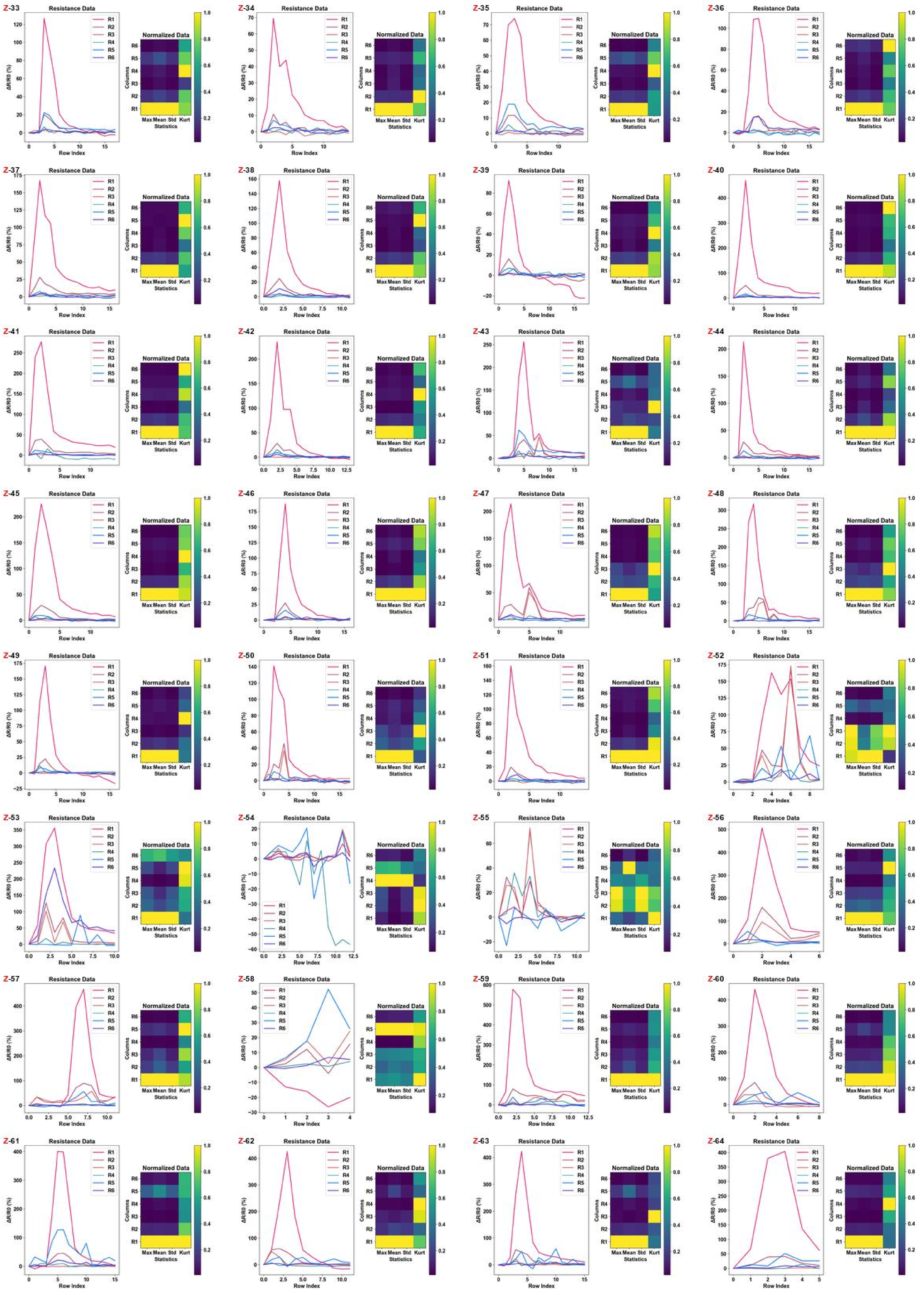
Z-33
Z-34
Z-35
Z-36
Z-37
Z-38
Z-39
Z-40
Z-41
Z-42
Z-43
Z-44
Z-45
Z-46
Z-47
Z-48
Z-49
Z-50
Z-51
Z-52
Z-53
Z-54
Z-55
Z-56
Z-57
Z-58
Z-59
Z-60
Z-61
Z-62
Z-63
Z-64
Resistance Data
Normalized Data
ΔR/R0 (%)
Row Index
Columns
Statistics
Max Mean Std Kurt
R1 R2 R3 R4 R5 R6

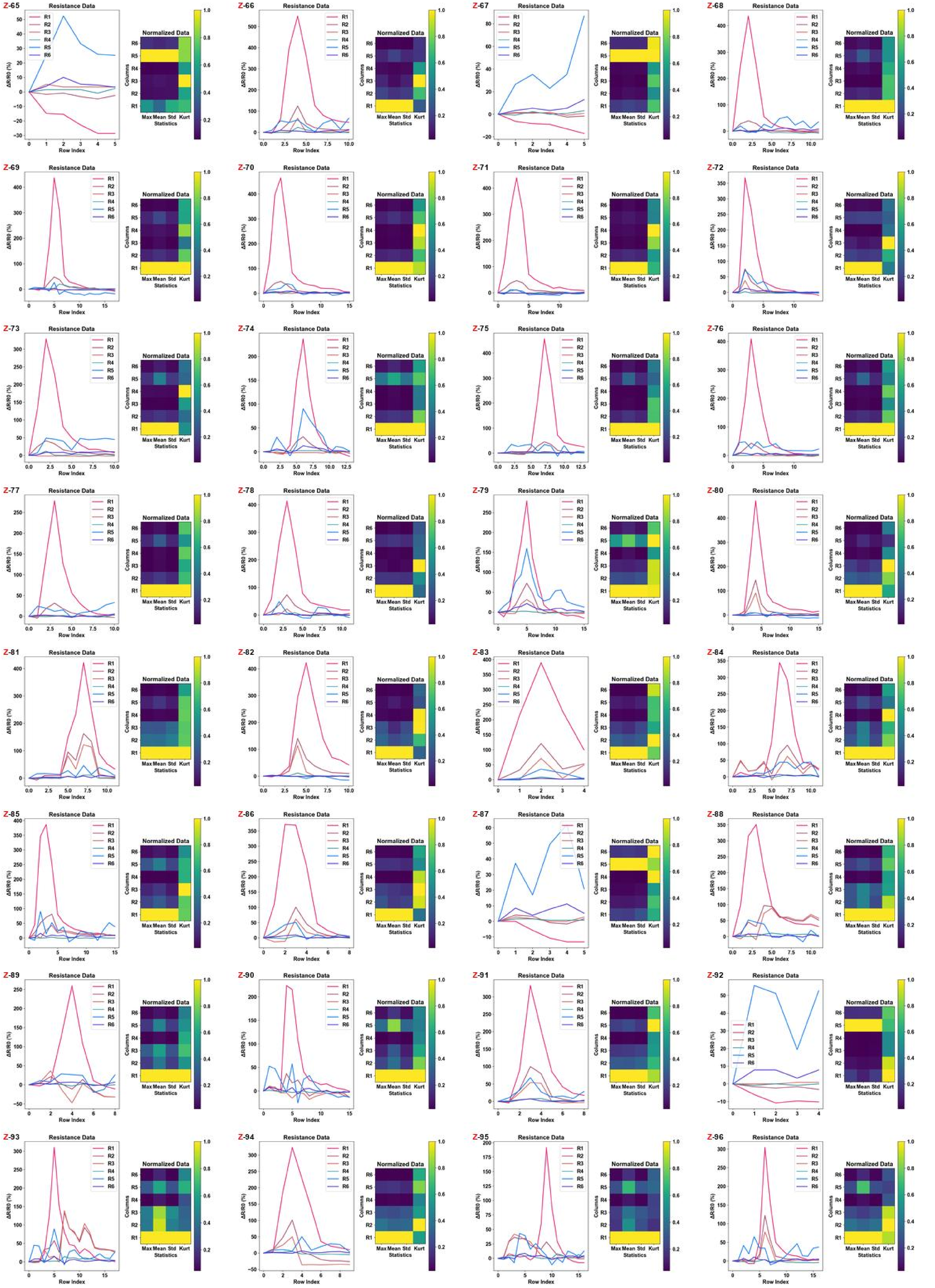
Z-65
Z-66
Z-67
Z-68
Z-69
Z-70
Z-71
Z-72
Z-73
Z-74
Z-75
Z-76
Z-77
Z-78
Z-79
Z-80
Z-81
Z-82
Z-83
Z-84
Z-85
Z-86
Z-87
Z-88
Z-89
Z-90
Z-91
Z-92
Z-93
Z-94
Z-95
Z-96
Resistance Data
ΔR/R0 (%)
Row Index
R1
R2
R3
R4
R5
R6
Normalized Data
Columns
Statistics
Max
Mean
Std
Kurt

Pressure-sensitive data of type Z

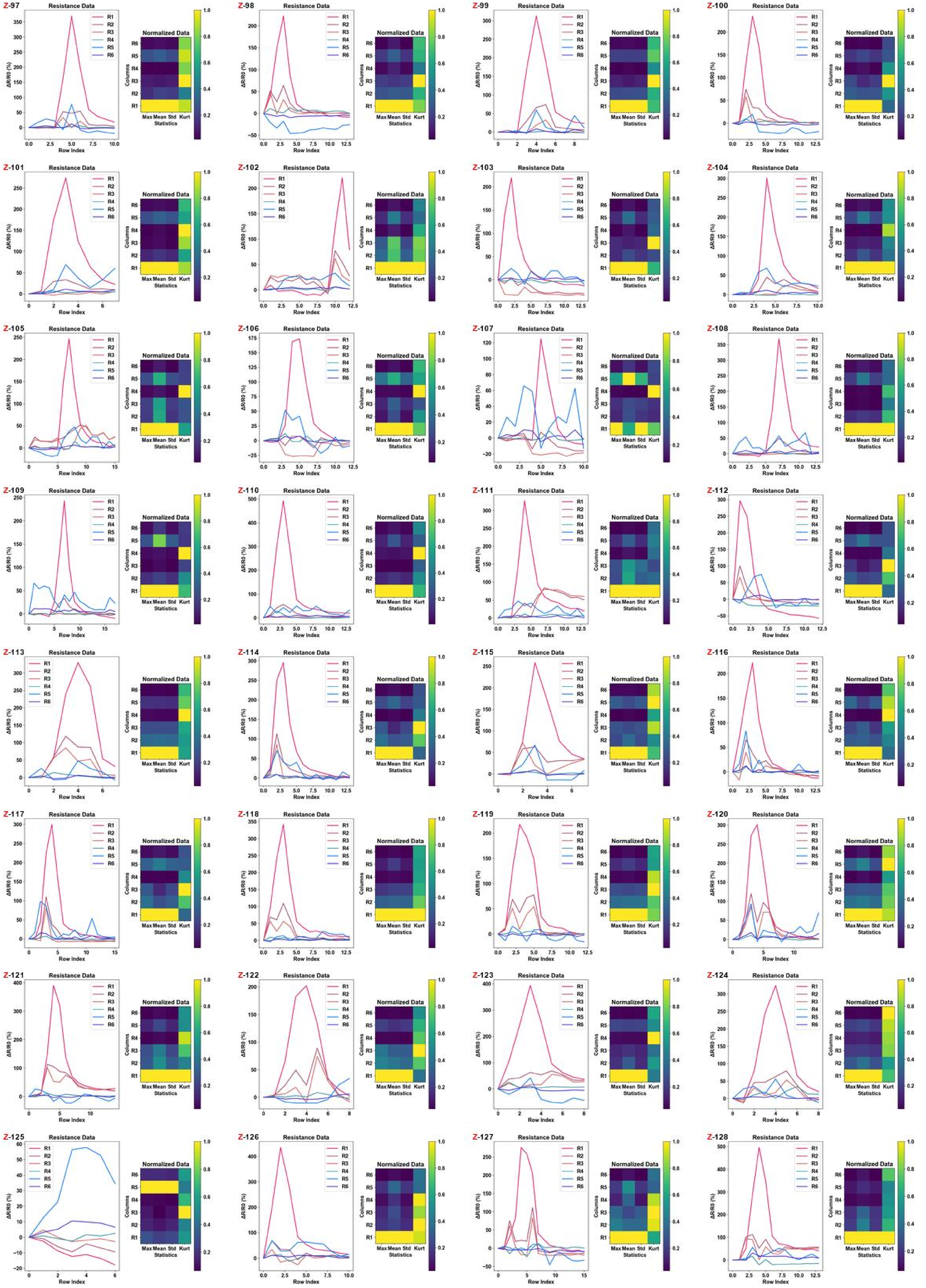

Z-129
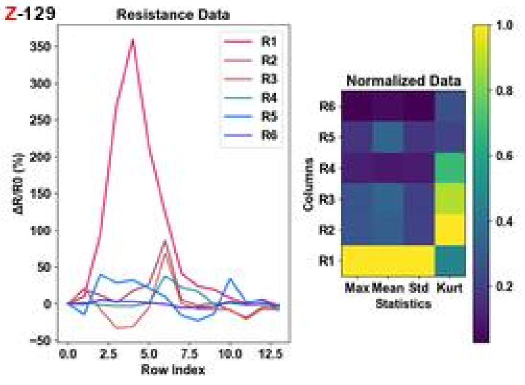

Z-130
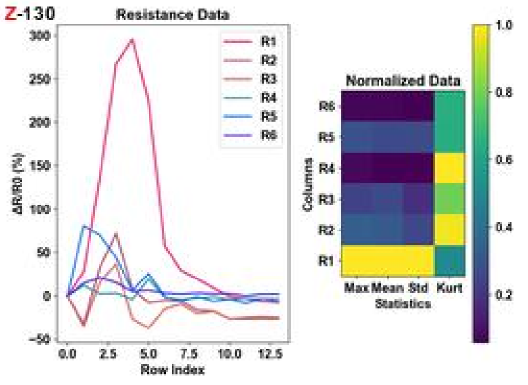

Z-131
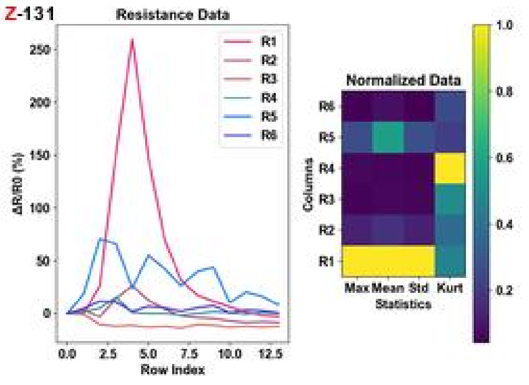

Z-132
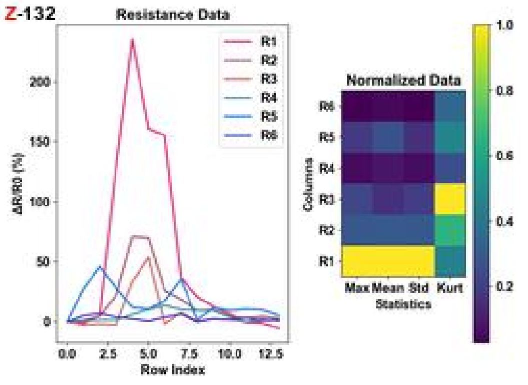

Z-133
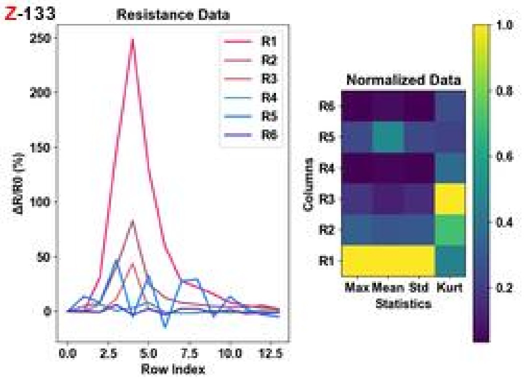

Z-134
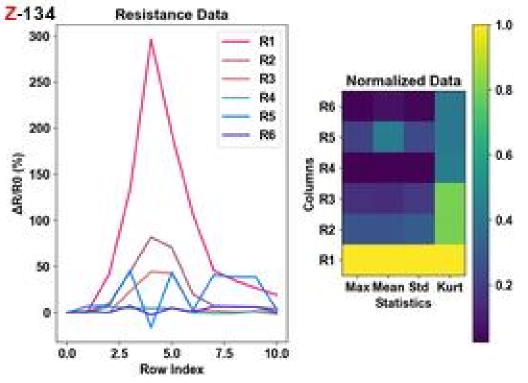

Z-135
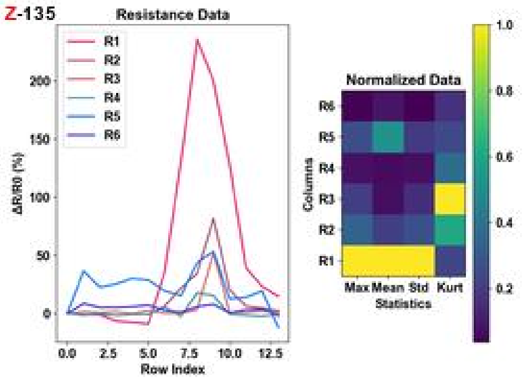

Z-136
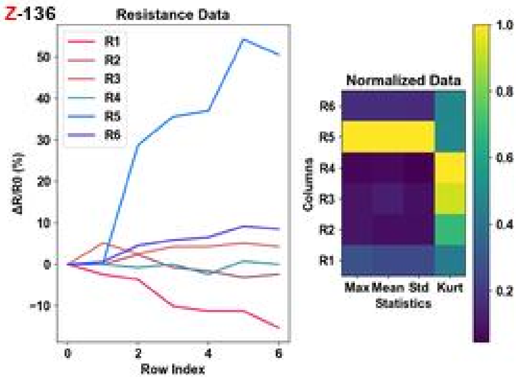

Z-137
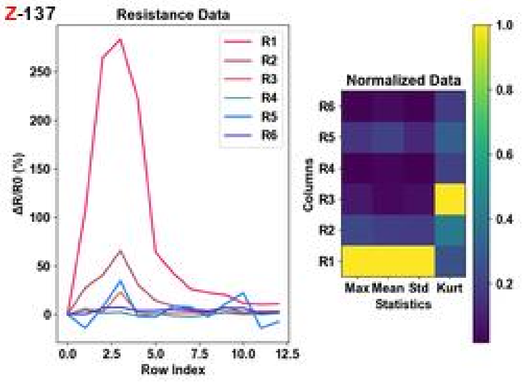

Z-138
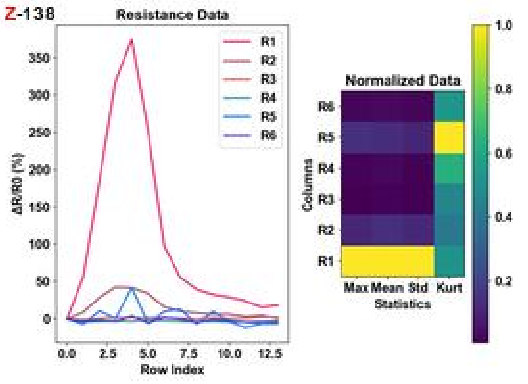

Z-139
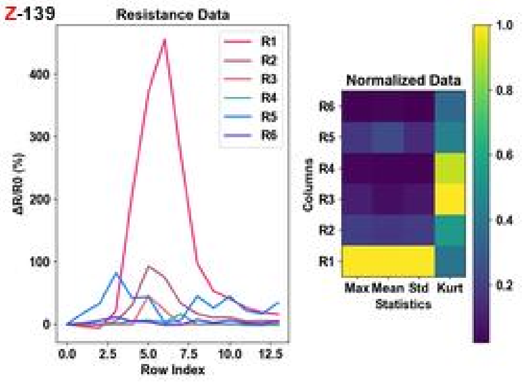

Z-140
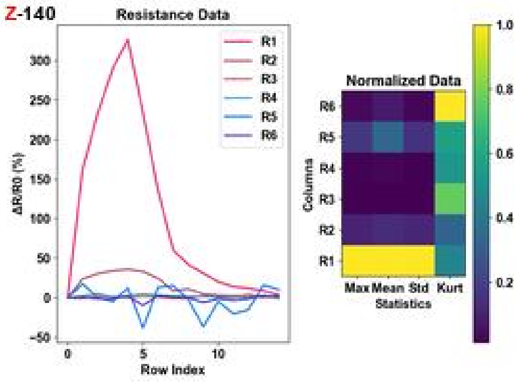

Z-141
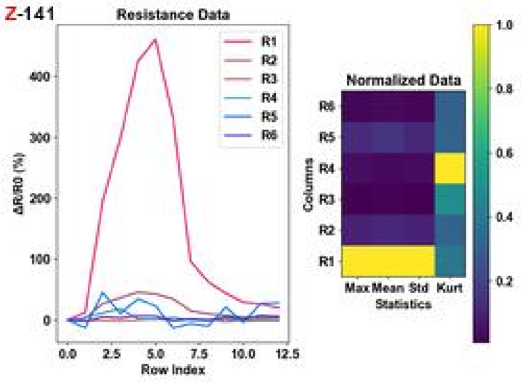

Z-142
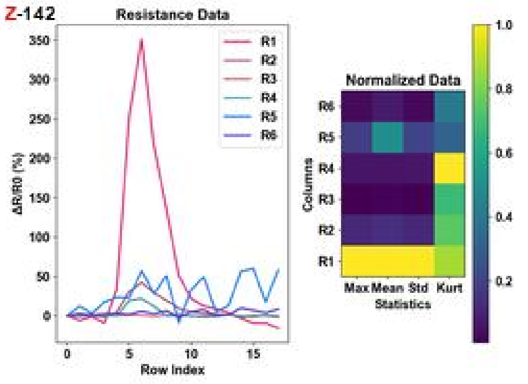

Z-143
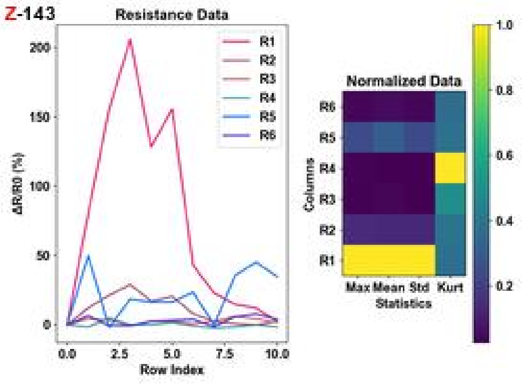

Z-144
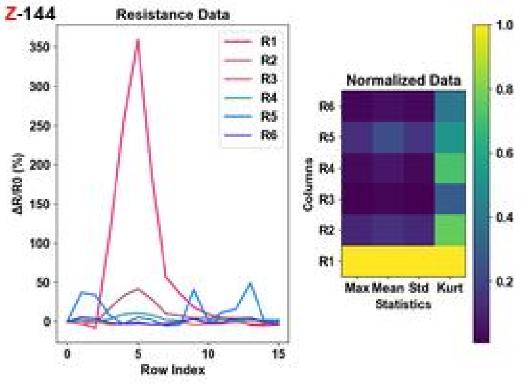

Z-145
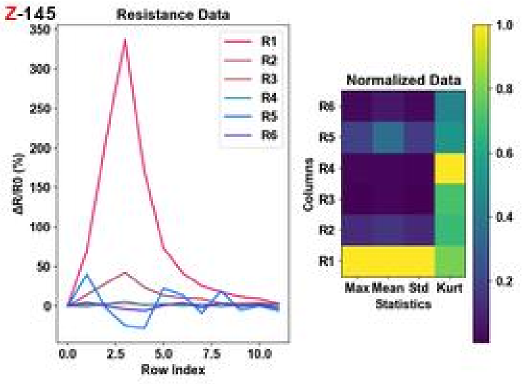

Z-146
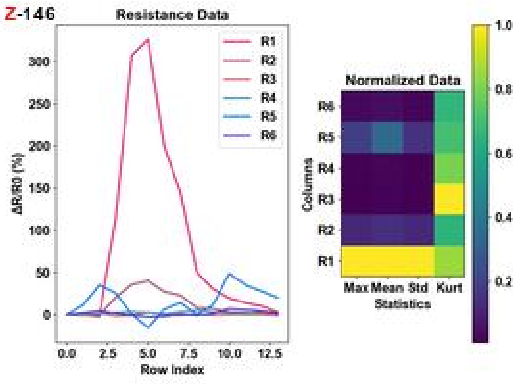

Z-147
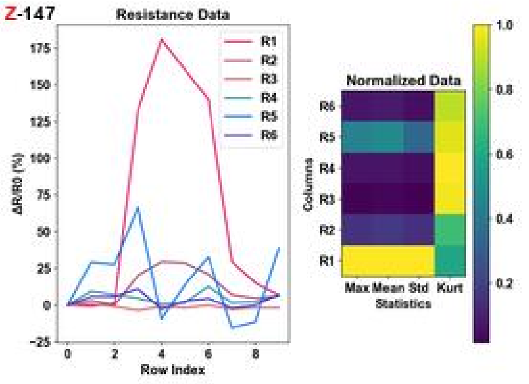

Z-148
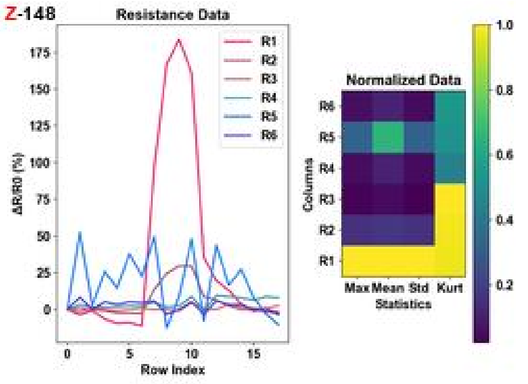

Z-149
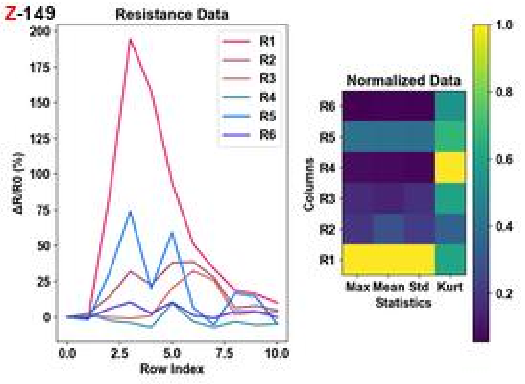

Z-150
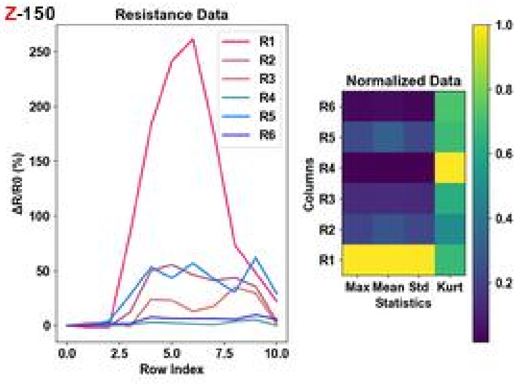

Z-151
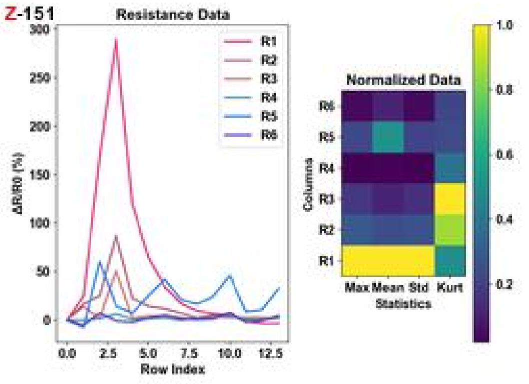

Z-152
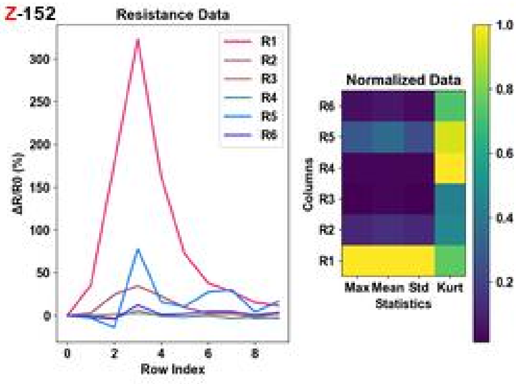

Z-153
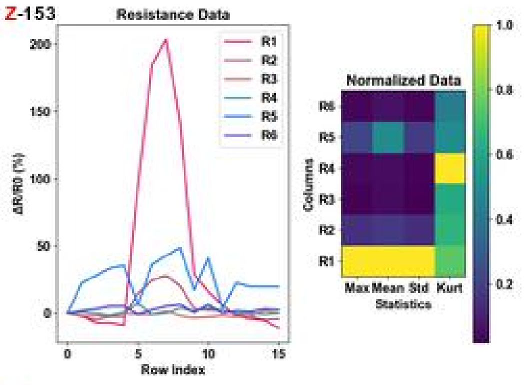

Z-154
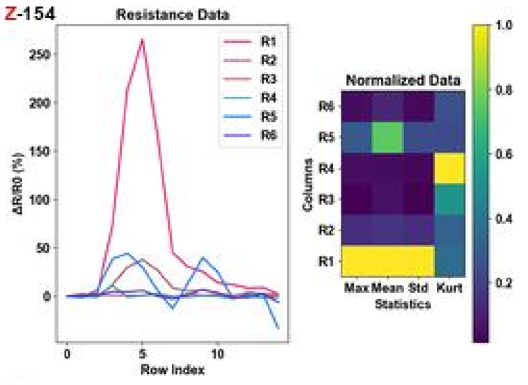

Z-155
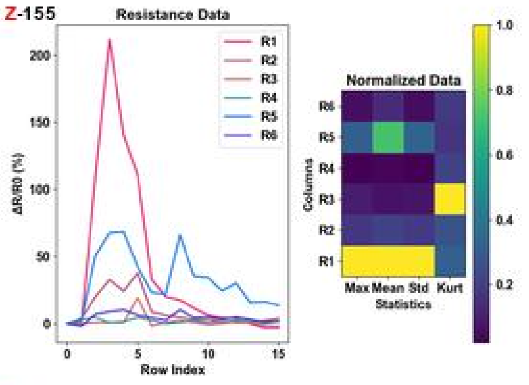

Z-156
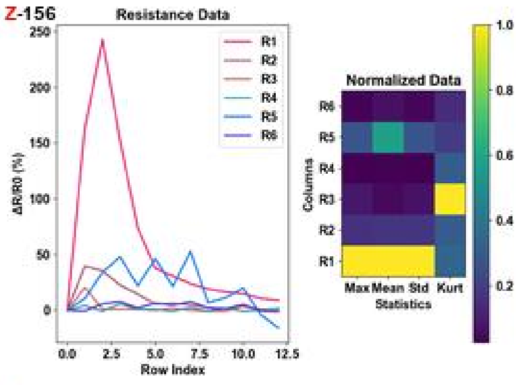

Z-157
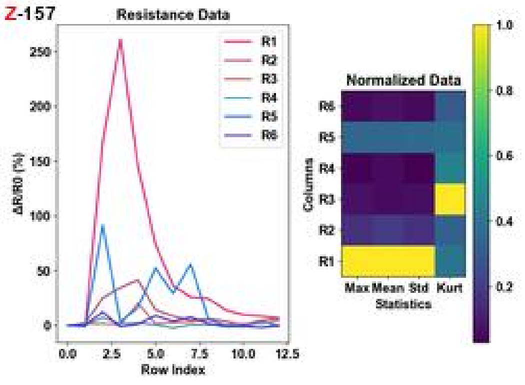

Z-158
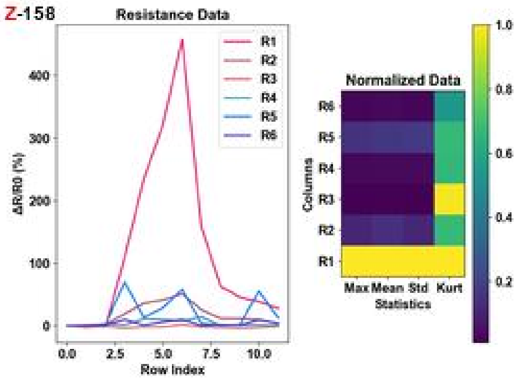

Z-159
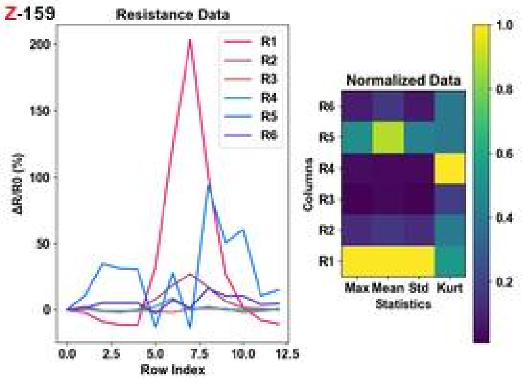

Z-160
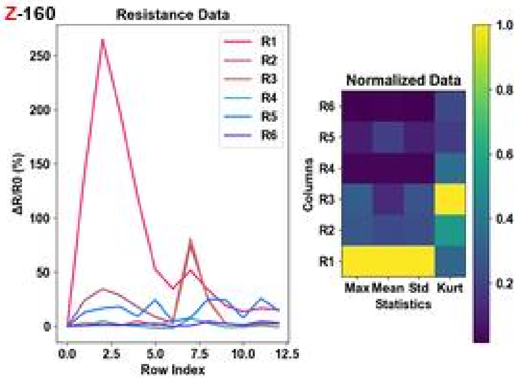

Pressure-sensitive data of type Z

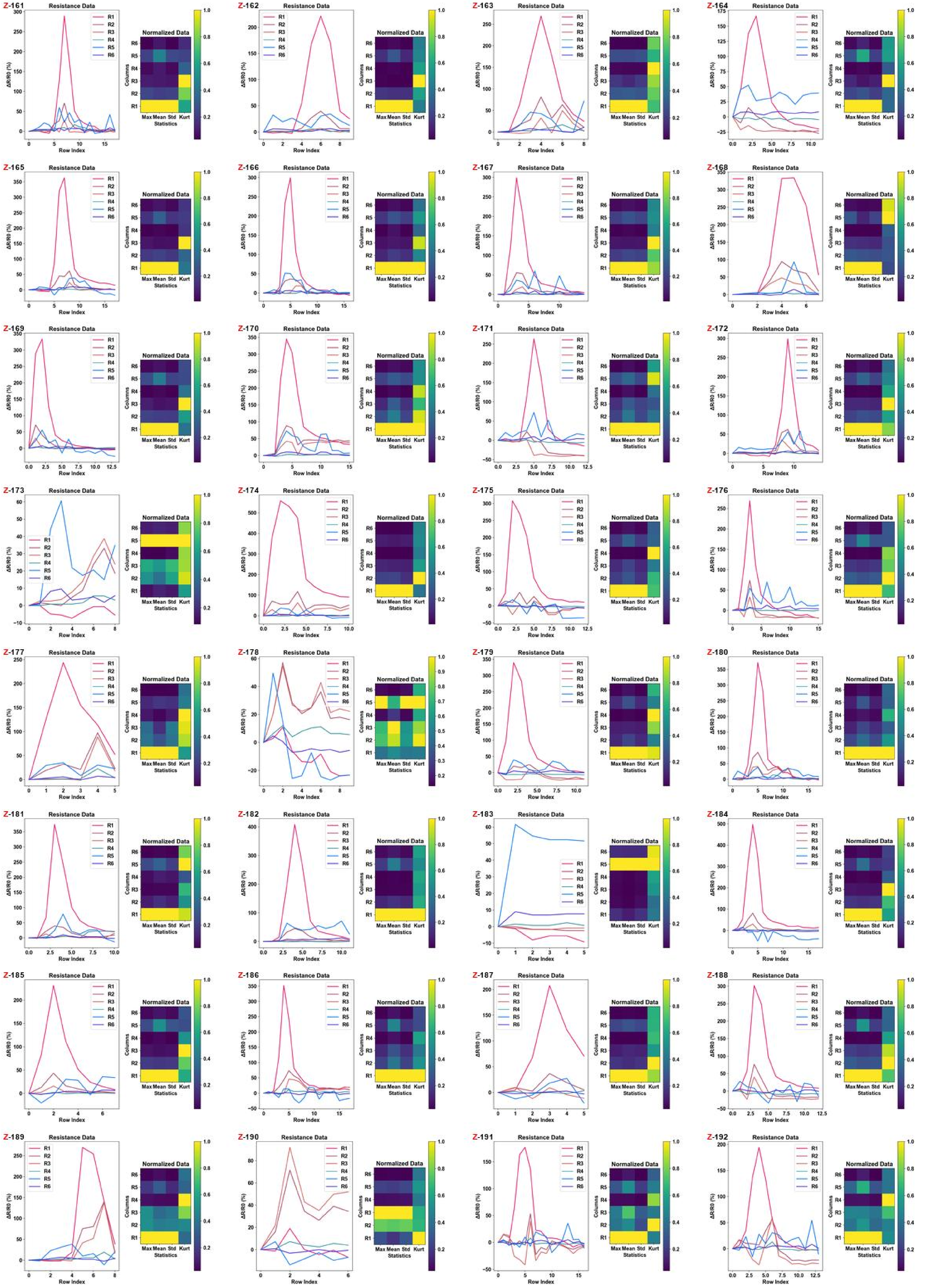

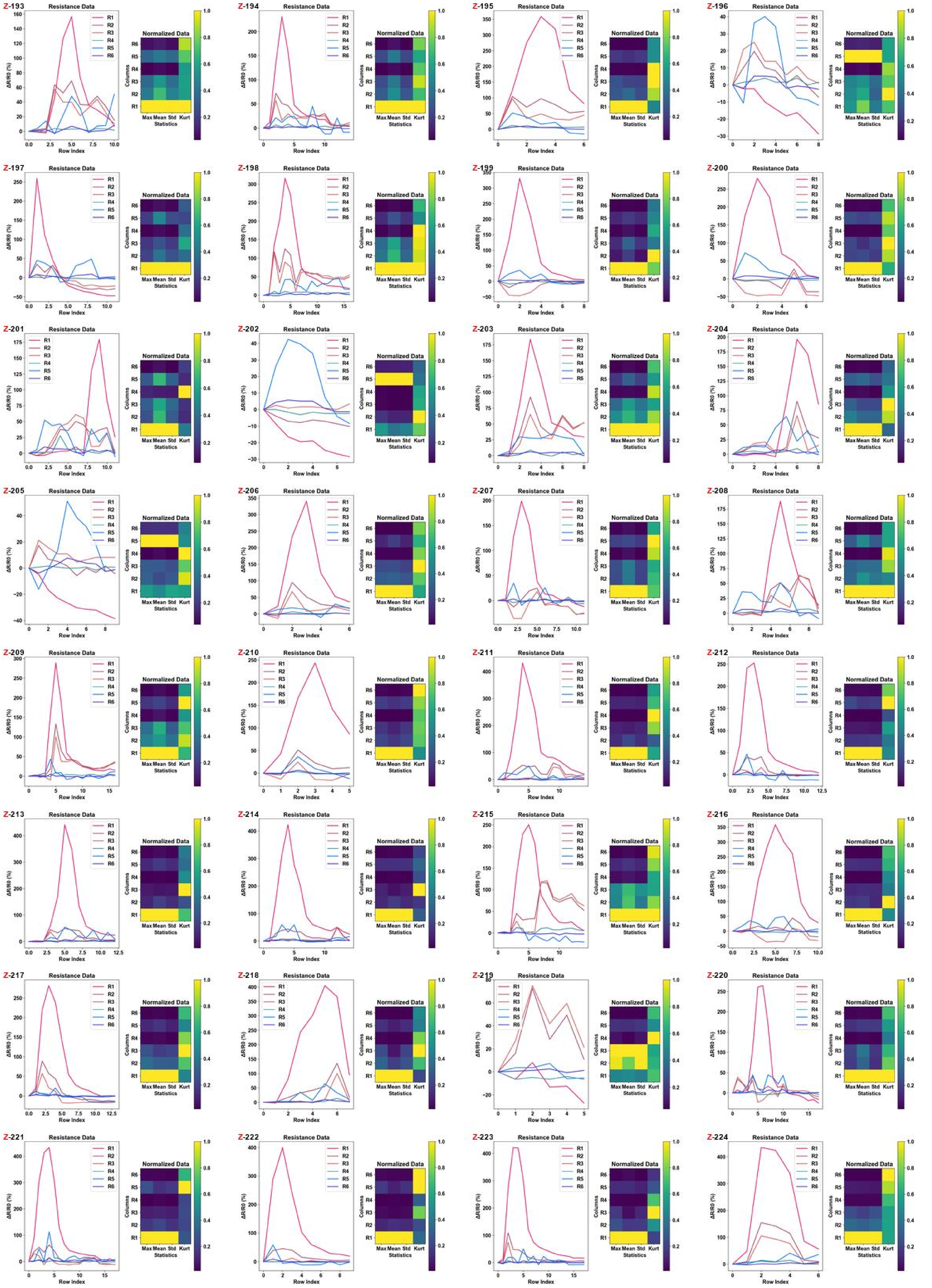
Z-193
Z-194
Z-195
Z-196
Z-197
Z-198
Z-199
Z-200
Z-201
Z-202
Z-203
Z-204
Z-205
Z-206
Z-207
Z-208
Z-209
Z-210
Z-211
Z-212
Z-213
Z-214
Z-215
Z-216
Z-217
Z-218
Z-219
Z-220
Z-221
Z-222
Z-223
Z-224
Resistance Data
ΔR/R0 (%)
Row Index
R1
R2
R3
R4
R5
R6
Normalized Data
Columns
Max Mean Std Kurt
Statistics

Z-225

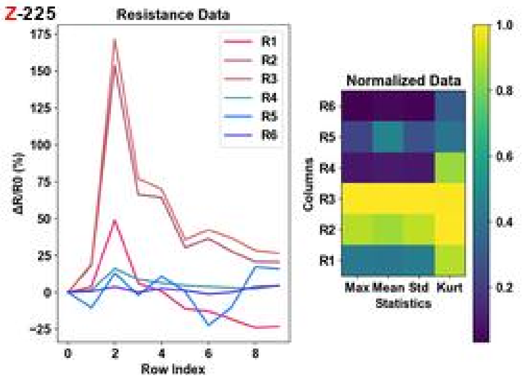

Z-226

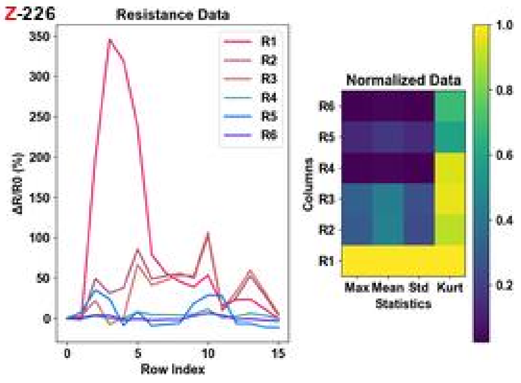

Z-227

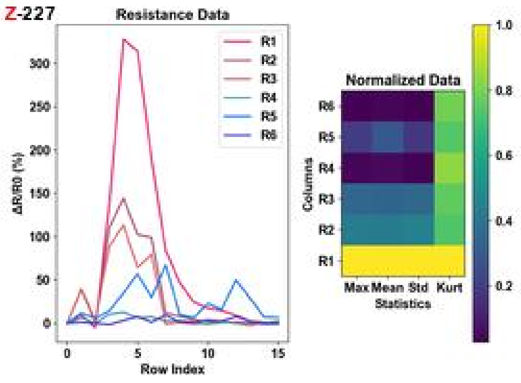

Z-228

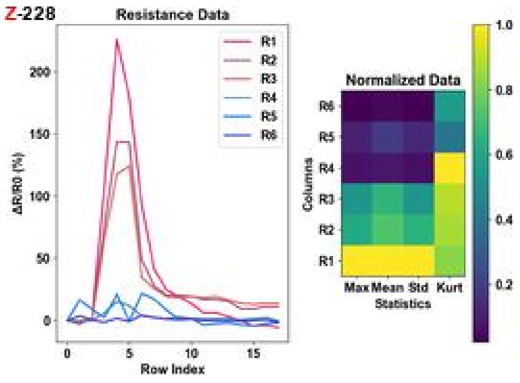

Z-229

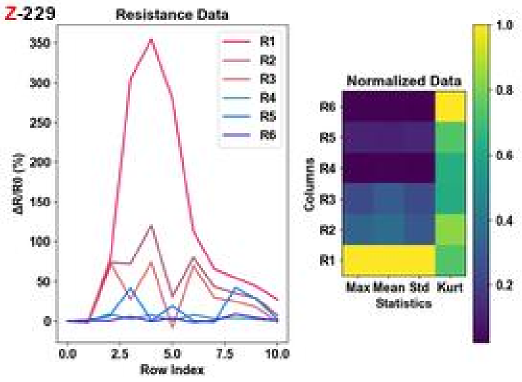

Z-230

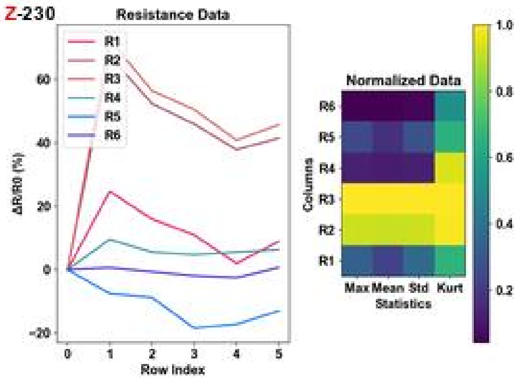

Z-231

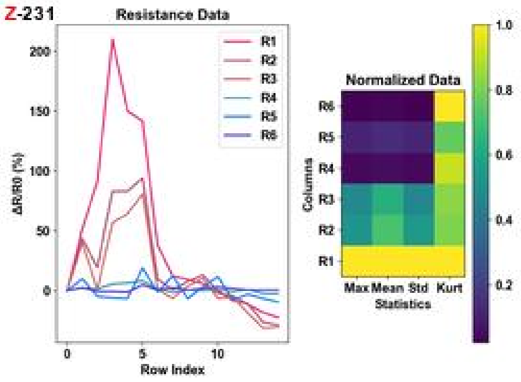

Z-232

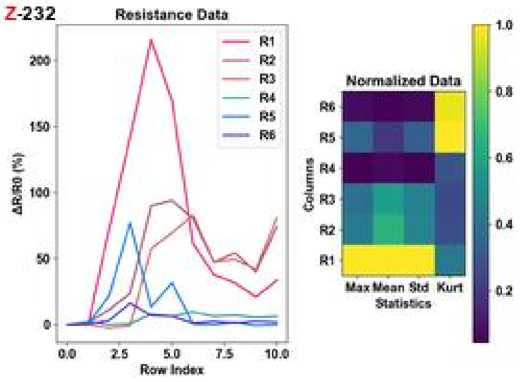

Z-233

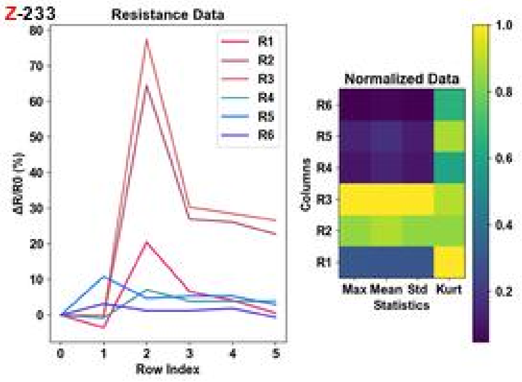

Z-234

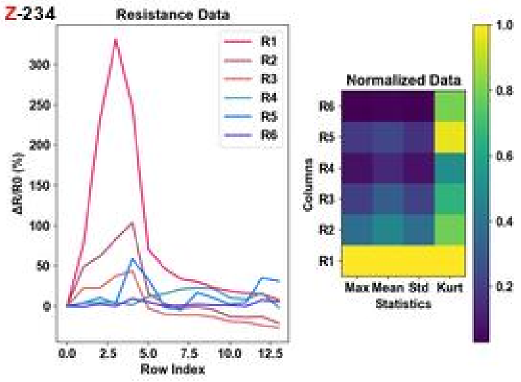

Z-235

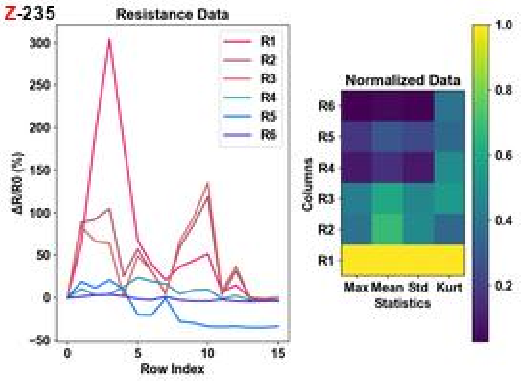

Z-236

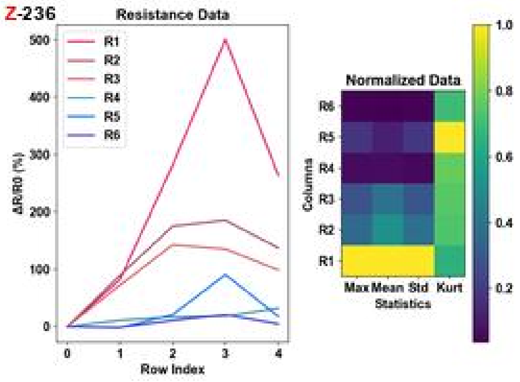

Z-237

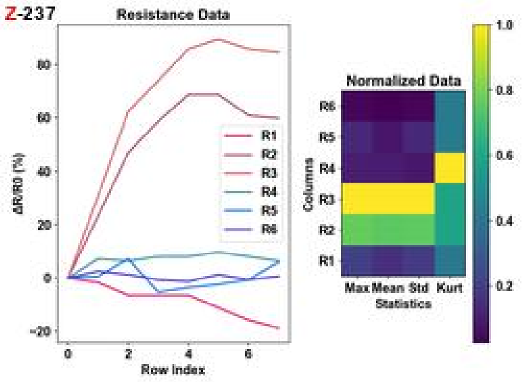

Z-238

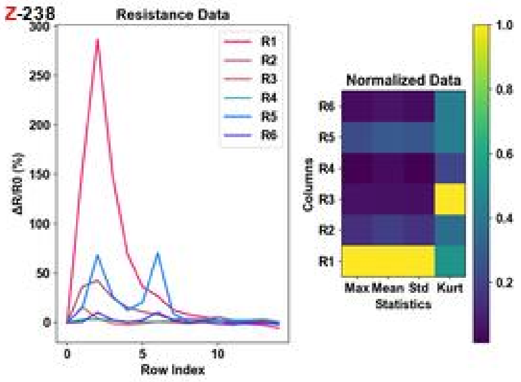

Z-239

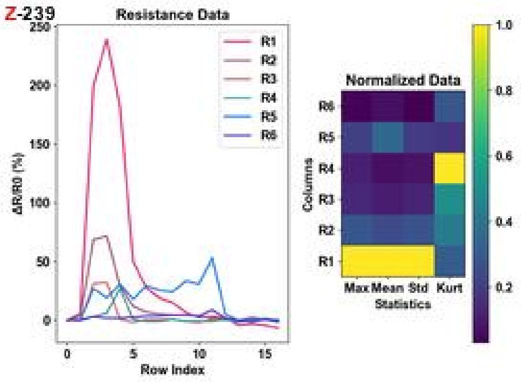

Z-240

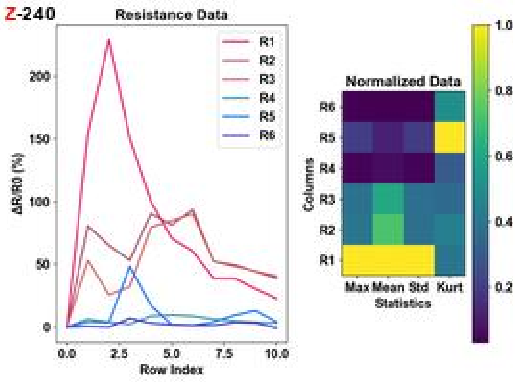

Supplement: Supplementary file 7 — Supporting Information [file ADMA-37-2509631-s002.pdf]
